# Analysis of MAPK and MAPKK gene families in wheat and related Triticeae species

**DOI:** 10.1186/s12864-018-4545-9

**Published:** 2018-03-05

**Authors:** Ravinder K. Goyal, Dan Tulpan, Nora Chomistek, Dianevys González-Peña Fundora, Connor West, Brian E. Ellis, Michele Frick, André Laroche, Nora A. Foroud

**Affiliations:** 1 Lethbridge Research and Development Centre, Agriculture and Agri-Food Canada, 5403 - 1st Avenue South, Lethbridge, Alberta T1J 4B1 Canada; 2 Information and Communication Technologies, National Research Council of Canada, 100 des Aboiteaux Street, Moncton, New Brunswick E1A 7R1 Canada; 3 Michael Smith Laboratories, University of British Columbia, #301 - 2185 East Mall, Vancouver, British Columbia V6T 1Z4 Canada

**Keywords:** Mitogen-activated protein kinase (MAPK), Wheat, Triticale, Rye, Barley, Cell signalling, Protein interactions, Gene expression, Phylogenetics

## Abstract

**Background:**

The mitogen-activated protein kinase (MAPK) family is involved in signal transduction networks that underpin many different biological processes in plants, ranging from development to biotic and abiotic stress responses. To date this class of enzymes has received little attention in Triticeae species, which include important cereal crops (wheat, barley, rye and triticale) that represent over 20% of the total protein food-source worldwide.

**Results:**

The work presented here focuses on two subfamilies of Triticeae MAPKs, the MAP kinases (MPKs), and the MAPK kinases (MKKs) whose members phosphorylate the MPKs. *In silico* analysis of multiple Triticeae sequence databases led to the identification of 152 MAPKs belonging to these two sub-families. Some previously identified MAPKs were renamed to reflect the literature consensus on MAPK nomenclature. Two novel MPKs, MPK24 and MPK25, have been identified, including the first example of a plant MPK carrying the TGY activation loop sequence common to mammalian p38 MPKs. An EF-hand calcium-binding domain was found in members of the Triticeae MPK17 clade, a feature that appears to be specific to Triticeae species. New insights into the novel MEY activation loop identified in MPK11s are offered. When the exon-intron patterns for some MPKs and MKKs of wheat, barley and ancestors of wheat were assembled based on transcript data in GenBank, they showed deviations from the same sequence predicted in Ensembl. The functional relevance of MAPKs as derived from patterns of gene expression, MPK activation and MKK-MPK interaction is discussed.

**Conclusions:**

A comprehensive resource of accurately annotated and curated Triticeae MPK and MKK sequences has been created for wheat, barley, rye, triticale, and two ancestral wheat species, goat grass and red wild einkorn. The work we present here offers a central information resource that will resolve existing confusion in the literature and sustain expansion of MAPK research in the crucial Triticeae grains.

**Electronic supplementary material:**

The online version of this article (10.1186/s12864-018-4545-9) contains supplementary material, which is available to authorized users.

## Background

Mitogen-activated protein kinases (MAPKs) are key signalling enzymes involved in the regulation of various aspects of biology in eukaryotic organisms, including cell division, development, metabolism and stress responses [1, 2]. As members of the protein kinase (PK) superfamily, they operate by catalyzing the phosphorylation of target proteins leading to conformational changes that alter their activity, associations, stability and/or cellular localization. The MAPK family is divided into three subfamilies that interact in a sequential manner [1, 3]: the MAPK kinase kinases (MKKKs) are activated by phosphorylation in response to specific environmental or developmental cues and subsequently activate one or more MAPK kinases (MKK), which in turn activate MAPKs (or MPK). MPKs have a multitude of cellular targets including metabolic and regulatory enzymes, and transcription regulators [4–6]. The acronym MAPK is used here as a general term, whereas MKKKs, MKKs and MPKs refer to members of the specific subfamilies.

The MKKKs comprise the most divergent group of MAPKs and are represented in large numbers in different plant genomes. Arabidopsis, rice and maize genomes each encode upwards of 70 MKKKs, while 155 MKKKs were recently reported in wheat [7–10]. The large proportion of MKKKs is consistent with the ability of plants to respond to a wide range of environmental and developmental cues. MKKs/MPKs are typically represented in smaller numbers in a given plant genome compared with the MKKKs. For example, in rice [3] and Brachypodium [11] there are 8 and 12 MKKs, and 15 and 16 MPKs, respectively. Thus, defined plant behavioral responses regulated through a small number of MKK/MPK cascades can be triggered from a broad set of signals transduced through members of the diverse group of MKKKs.

Plant MKKKs are serine/threonine (S/T) protein kinases and phosphorylate cognate residues in MKK (S/T)xxxxx(S/T) ‘activation loops’, also known as ‘T-loops’. The plant MKK family is composed of dual-specificity kinases that phosphorylate both threonine and tyrosine residues in the TxY activation loop of their corresponding MPK targets [12]. MPKs are S/T protein kinases and, in mammalian systems, are loosely divided into three categories: extracellular signal-related protein kinases (ERKs), c-Jun N-terminal kinases (JNKs) and p38 kinases. JNK and p38 kinases have TPY and TGY activation loop motifs, respectively, whereas ERKs have TEY motifs [13, 14]. Plant MPKs are ERK-like and typically possess TEY (MPK clades A, B, and C) or TDY (clade D) activation loop motifs [12].

Comparative analysis and interpretations of gene families across species is made more difficult when arbitrary and multiple names inconsistent with established nomenclature are assigned to a gene. To facilitate comparisons among predicted MAPK orthologues from different plant species a standardized nomenclature was earlier proposed by The MAPK Group [12], based primarily on the Arabidopsis gene families. Hamel et al. [3] subsequently employed this nomenclature to assign orthologous names and numbering for MPKs and MKKs in rice and poplar. Since then, a substantial number of plant MAPK gene identification studies have been reported [7, 8, 11, 15–22], but not only are orthologous genes in different species often named differently, in some instances multiple names have been assigned to a given MAPK accessions. For example, three different MAPK gene family nomenclatures have been proposed in rice [3, 4], which becomes particularly problematic when it results in two different family members having the same name, depending on which nomenclature is being employed. With the rapid expansion of plant genomic information, the continued arbitrary assignment of MAPK names will entangle the subject matter further.

There are only a few descriptions of wheat and barley MAPK sequences in the literature, but there is already discord in the nomenclature within each of these species [23–27] and this problem is also reflected in GenBank entries for these species. Furthermore, with few exceptions [28, 29], the published nomenclatures for wheat and barley MPKs and MKKs do not correspond to the established nomenclatures used in Brachypodium and Arabidopsis.

The information available on MAPKs in the Triticeae tribe of the Poaceae family of plants is limited. Triticeae includes important grain crops such as wheat, barley, rye and triticale used in food/feed around the world. There do not appear to be any reports on predicted MAPK sequences in rye or triticale and only a few studies have been reported in barley [26, 27, 29–32]. The first wheat MAPK publication came in 1999 where the expression of *TaMPK3*, then known as *WCK-1*, was shown to be induced by a fungal elicitor [33]. Surprisingly little progress has since been made on wheat MAPKs (Additional file 1). The best characterized wheat MAPKs are TaMPK3 and 6 [28, 34–36], whose enhanced expression and/or activation has been reported in response to biotic or abiotic stresses. There are also some reports on wheat MKKKs [9, 37–39], and one MPK phosphatase has been identified and characterized from durum wheat (*T. durum*) [40, 41].

With recent development of more complete and better annotated wheat and barley sequence databases, particularly in Ensembl Plants (http://plants.ensembl.org/index.html), many more MAPK sequences are now available. However, a comprehensive analysis is needed in order to provide a more complete and accurate assignment of the MAPK nomenclature for these species. At this early stage in cereal MAPK research it is critical that orthology-based naming is established in order to facilitate cross-species comparisons. We have therefore surveyed publicly available genomic databases of barley, wheat and the ancestral wheat species, *Aegilops tauschii* and *Triticum urartu*, for predicted MPK and MKK sequences, while in-house databases were used to identify orthologues in triticale and rye. To assign the correct nomenclature, the phylogenetic relationship of these sequences was compared with those from Brachypodium and other species. Previously reported naming is listed together with the orthology-based naming provided here in order to facilitate cross-referencing of each MAPK. Our analysis led to the identification of novel MAPK sequences and motifs that appear to be unique to Triticeae, and also offers new insights into monocot-specific signatures. In addition to the detailed sequence analyses, we provide a wheat MAPK interaction map along with some preliminary functional analysis of abiotic stress-related MPK gene expression, development-related MAPK gene expression and MPK activation. This work should serve as a foundation on which advances in MAPK research can be built in cereals, not only to enhance our knowledge of MAPK signalling pathways in plants, but also elucidate how they differ in Triticeae in controlling important agricultural traits of these crops.

## Results

### Identification and annotation of Triticeae MAPKs

MPK and MKK families encoded in the genomes of key Triticeae species (wheat (*Triticum aestivum*; ABD genomes), barley (*Hordeum vulgare*; H genome)*,* rye (*Secale cereale*; R genome), triticale (*xTriticosecale*; ABR genomes), goat grass (*Aegilops tauschii*; D genome), and red wild einkorn (*T. urartu*; A genome)) were identified by searching publicly available databases, seeking candidates homologous to the Brachypodium sequences reported earlier by Chen et al. [11]. The identified MPKs and MKKs are named according to the nomenclature earlier defined by the MAPK Group [12], and also employed for Brachypodium MAPKs [11]. The Triticeae MAPK genes and their accession numbers are listed in Additional files 2 and 3. Additional file 2 is limited to Ensembl Plants accessions, but incorporates additional details, including intron/exon number, predicted molecular weight and isoelectric point. Additional file 3 provides all identified accessions from multiple databases. A total of 152 MAPKs were identified, not including copy number or multiple accessions reported in various databases. Of the 152 MAPKs, within each Triticeae species up to 17 *MPKs* (Table 1) and up to 14 *MKKs* (Table 2) were identified. Some of the wheat and barley MAPKs have already been reported; these are listed in Table 3 (*MPKs*) and Table 4 (*MKKs*), together with previous naming. The available genomic DNA sequences are provided in Additional files 4 (*MPKs*) and 5 (*MKKs*), and the transcript sequences for triticale MAPKs in Additional file 6.

**Table 1:** Number of Triticeae genes orthologous to Arabidopsis (*At*), rice (*Os*) [3, 18], Brachypodium (*Bd*) *MPKs* [11] identified from the genomic database (Ensembl Plants) for wheat (*Ta*), barley (*Hv*), *A. tauschii* (*Aet*), *T. urartu* (*Tu*), and from the TSA database (GenBank) for rye (*Sc*) and triticale (*Ts*)

*AtMPKs*	*Os*	*Bd*	*Ta* ^a^	*Hv*	*Sc*	*Ts* ^a^	*Aet*	*Tu*
*AtMPK3*	1	1	1 (3)	1	1	1 (1)	1	1
*AtMPK4*	1	1	1 (3)	1	1	1 (1)	1	–
*AtMPK6*	1	1	1 (3)	1	1	1 (1)	1	–
*AtMPK7*	1	2	1 (4)	1	1	1 (1)	1	1
*AtMPK11*	1^b^	1	1 (3)	1	1	1 (2)	1	1
*AtMPK14*	1	1	1 (3)	1	1	1 (1)	1	1
*AtMPK16*	2	1	1 (3)	1	1	1 (2)	1	1
*AtMPK17*	2	1	1 (3)	1	1	1 (3)	1	1
*AtMPK20*	5	5	5 (15)	5	5	5 (10)	5	4
Other MPKs								
*OsMPK21*	2	2	2 (3)	2^c^	1	2 (4)	2	1
*MPK24*	–	–	1 (3)	1	1	1 (1)	1	1
*MPK25*	–	–	1 (3)	1	1	1 (1)	1	–
Total	17	16	17 (52)	17	16	17 (28)	17	12

^a^Numbers in brackets indicate number of copies for polyploid genomes

^b^*OsMPK11* was first reported as *OsMPK4–2* [18]

^c^GenBank accession identified for *HvMPK21–2*, but no corresponding sequence found in Ensembl Plants

**Table 2:** Number of Triticeae genes orthologous to Arabidopsis (*At*), rice (*Os*) [3, 18], Brachypodium (*Bd*) *MKKs* [11] identified from the genomic database (Ensembl Plants) for wheat (*Ta*), barley (*Hv*), *A. tauschii* (*Aet*), *T. urartu* (*Tu*), and from the TSA database (GenBank) for rye (*Sc*) and triticale (*Ts*)

*AtKKKs*	*Os*	*Bd*	*Ta* ^a^	*Hv*	*Sc*	*Ts* ^a^	*Aet*	*Tu*
*AtMKK1*	1	1	3 (7)	2	1	–	–	–
*AtMKK3*	1	3	3 (6)	2	2	2 (4)	2	2
*AtMKK4*	1	1	1 (3)	1	1	1 (1)	1	3
*AtMKK5*	1	1	1 (3)	1	1	1 (2)	1	1
*AtMKK6*	1	1	1 (6)	1	1	1 (1)	1	1
*AtMKK10*	3	5	5 (19)	5	1	1 (1)	2	3
Total	8	12	14 (44)	12	7	6 (9)	7	10

^a^Numbers in brackets indicate number of copies for polyploid genomes

**Table 3:** Previously assigned nomenclature for Triticeae *MPKs*

Gene Name	Previously assigned nomenclature
*TaMPK3*	*WCK-1* ([33], GenBank), *TaMAPK1* [24], ***TaMPK3*/*MPK3*** **(**[28]**, Ensembl Plants)**, *TaMPK4* [36], *TaMAPK9* [25], * TaMAPK15 * [25], *TaMAPK25* [25], *TaMAPK30* [25]
*TaMPK4*	***TaMAPK4*** [24], *TaMPK6* [23, 51, 52], TaMAPK14 [25], *TaMAPK28* [25], *TaMAPK32* [25]
*TaMPK6*	*FLRS* ([34, 35], GenBank), *MAPKflrs* ([80], GenBank), *TaMPK1* [23, 51, 52], *TaMAPK2* [24], *TaMAPK19* [25], *TaMAPK48* [25], *TaMAPK51* [25]
*TaMPK7*	*TaMPK4* [23, 51, 52], *MAPK4* ([81], GenBank), *MAPK5* [GenBank], *TaMAPK12* [25], *TaMAPK21* [25], *TaMAPK54* [25]
*TaMPK11*	*TaMAPK3* [24]
*TaMPK14*	*TaMPK3* [23, 51, 52], *TaMAPK6* [24], *TaMAPK36* [25], *TaMAPK45* [25]
*TaMPK16*	*MAPK2A* ([80], GenBank), *MAPK2B* ([80], GenBank), *MAPK2C* ([80], GenBank), *TaMAPK4* [25], *TaMAPK11* [25], ***MPK16*** **[Ensembl Plants]**, *TaMAPK27* [25]
*TaMPK17*	*MAPK1a* ([80], GenBank), *MAPK1b* ([82], GenBank), *MAPK1C* [GenBank], *MAPK1d* ([83], GenBank), *MAPK1f* ([80], GenBank), *TaMPK12* [23], *TaMAPK14* [24], *TaMAPK20* [25], *TaMAPK49* [25], *TaMAPK53* [25]
*TaMPK20–1*	*TaMAPK8* [25], *TaMAPK38* [25],
*TaMPK20–2*	*TaMAPK6* [25], *TaMPK9* [23, 51, 52], *TaMAPK9* [24], *TaMAPK34* [25], *TaMAPK43* [25]
*TaMPK20–3*	*TaMAPK3* [25], *TaMAPK7* [25], *TaMAPK52* [25]
*TaMPK20–4*	*TaMAPK7* [24]*, TaMAPK8* [24], *TaMAPK16* [25], *TaMAPK24* [25], *TaMAPK39* [25]
*TaMPK20–5*	*TaMPK7* [23, 51, 52], *TaMAPK29* [25], *TaMAPK33* [25], *TaMAPK41* [25]
*TaMPK21–1*	*TaMAPK5* [25], *TaMPK17* [23, 51, 52], *TaMAPK42* [25]
*TaMPK21–2*	*TaMPK16* [23, 51, 52], *TaMAPK23* [25]*, TaMAPK40* [25]
*TaMPK24*	*TaMAPK17* [25], *TaMPK12;1* [23, 51, 52], *TaMAPK35* [25], *TaMAPK44* [25]
*HvMPK3*	***MPK3*** **[Ensembl Plants],** *HvMPK5* [27]
*HvMPK4*	*HvMPK6* [27],
*HvMPK6*	*HvMPK1* [27]
*HvMPK7*	*HvMPK4* [26, 27]
*HvMPK11*	*HvMPK2* [27]
*HvMPK14*	*HvMPK3* [27]
*HvMPK16*	*HvMPK14* [27], ***MPK16*** **[Ensembl Plants]**
*HvMPK20–2*	*HvMPK9* [27]
*HvMPK20–3*	*HvMPK11* [27]
*HvMPK20–5*	*HvMPK7* [27]
*HvMPK21–1*	*HvMPK17* [27]
*HvMPK21–2*	*HvMPK16* [27]

Gene names that match assignment presented in the current manuscript are marked in bold. For accession numbers refer to Additional file 3

**Table 4:** Previously assigned nomenclature for Triticeae *MKKs*

Gene Name	Previously assigned nomenclature
*TaMKK3–2*	*TaMAPKK2* [25]*,* ***TaMKK3–2*** **[GenBank],** *TaMAPKK13* [25]
*TaMKK3–3*	*TaMAPKK3* [25]*, TaMAPKK4* [25], *TaMAPKK18* [25]
*TaMKK4*	*TaMAPKK1* [25]
*TaMKK6*	*TaMPKK1* [23, 51, 52]
*TaMKK10–1/3*	*TaMAPKK6* [25], *TaMAPKK7* [25], *TaMAPKK12* [25], *TaMAPKK17*
*TaMKK10–4*	*TaMAPKK5* [25], *TaMAPKK9* [25], *TaMAPKK10* [25], *TaMAPKK14* [25], *TaMAPKK15* [25]
*TaMKK10–5*	*TaMAPKK11* [25], *TaMAPKK16* [25]
*HvMKK3–2*	*HvMKK3*/*MKK3* ([29], Ensembl Plants, GenBank)
*HvMKK6*	***MKK6*** **[Ensembl Plants]**

Gene names that match assignment presented in the current manuscript are marked in bold. For accession numbers refer to Additional file 3

### Phylogenetic relationship of Triticeae MPKs and MKKs and identification of two new MPKs

**Fig. 1:**
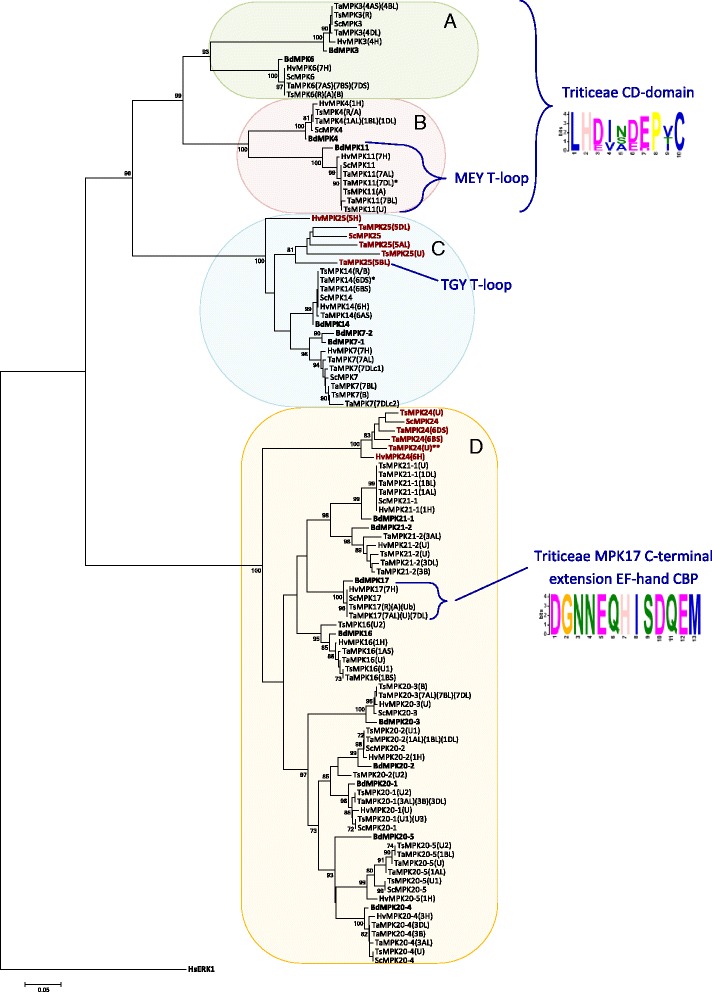
Phylogenetic distribution of Triticeae and Brachypodium MPKs. PK domains of wheat (Ta), barley (Hv), rye (Sc) and triticale (Ts) MPKs were aligned with Brachypodium (Bd) MPKs, and the tree rooted with human (Hs) ERK1. Descriptive gene names are followed by chromosome number when available. Accessions used in the analysis are marked in bold in Additional file 3. BdMPKs are bolded in tree to visually separate them from the sequences identified herein. Only accessions with complete PK domains are included in the analysis. MPKs are divided into four clades (A, B, C, and D). MEME analyses shows amino acid distribution in Triticeae of some conserved domains. The “common docking” CD domain in the C-terminal extensions of clades A and B, which in Triticeae is shown to have the consensus sequence LH(D/E)xx(D/E)(D/E)PxC, similar to that reported in Brachypodium [11]. Unique to Triticeae MPK17s, an EF-hand CBP domain is found in their C-terminal extensions. The A, B and C clades carry TEY T-loops and D clade MPKs carry TDY motifs, with the exception of (a) MPK11s which carry an MEY motif, and (b) a TGY motif found in one copy of TaMPK25. An asterix (*) is used to indicate that the sequence includes one unidentified (X) amino acid residue and two aesterix (**) indicates that a stretch of residues are unidentified; these unknowns did not affect placement in the phylogenetic tree

MPKs from four cultivated Triticeae species (wheat, barley, rye and triticale) were analyzed for their phylogenetic relationship with the corresponding Brachypodium sequences, and the results are presented in Fig. 1. Homologues for each of the 16 Brachypodium MPKs (BdMPKs), along with some gene duplications, were found in all four Triticeae crops. Seventeen MPKs are also present in *A. tauschii*, but only 12 were found in *T. urartu* (Table 1 and Additional file 3). Similar to other plant species, the Triticeae MPKs are distributed into all four clades (A, B, C and D).

**Fig. 2:**
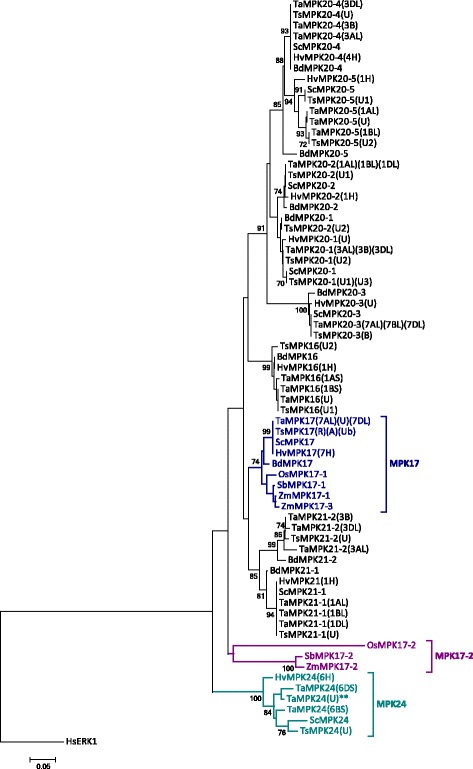
Phylogenetic analysis of clade D MPKs: a close up of monocot MPK17s. PK domains of wheat, barley, rye and triticale clade D MPKs were aligned with BdMPKs, as well as rice (Os), maize (Zm) and sorgum (Sc) MPK17s. The tree is rooted with HsMEK1. MPK17s are in blue font, MPK17–2 in purple and MPK24 in teal

Previously, phylogenetic analysis of Arabidopsis, poplar and rice suggested a gene duplication event in the clade D rice *MPK17* resulting in *OsMPK17–1* and *17–2* [3]. This duplication seems to be absent in Brachypodium. The Triticeae genomes were surveyed to identify potential orthologues of *OsMPK17–2*. However, when the phylogenetic analysis was repeated using Triticeae MPKs together with the Brachypodium sequences (Fig. 1), the clustering pattern suggested that the so-called Triticeae orthologues of OsMPK17–2 (later named MPK24) are not MPK17s. Since the Brachypodium genome does not appear to encode an orthologue of OsMPK17–2, an additional phylogenetic tree focused on clade D, and including MPK17s from several additional monocot species, was analyzed (Fig. 2). A clear separation of Triticeae MPK17–2 s (presented as MPK24s) from OsMPK17–2 and other MPK17s indicates that they are not orthologous to the Triticeae MPK gene products and that they should be designated as a novel MPK; namely, MPK24. Furthermore, there appears to be a tendency of monocot MPK17–2 s (maize, sorghum and rice) to branch out from their MPK17–1 paralogues in the phylogenetic tree.

The Brachypodium genome encodes two variants of MPK7 (BdMPK7–1 and 7–2). Only one copy was identified in Triticeae, but another novel MPK, MPK25, was identified in clade C. MPK25 was found in all of the surveyed Triticeae species, with the exception *T. urartu*; although a copy is present in the A genome of wheat (Fig. 1), which was presumably inherited from *T. urartu*. Comparatively longer branches are observed among MPK25s than for other MPKs, and HvMPK25 is further branched out from the other Triticeae members, suggesting that this group may be diverging.

**Fig. 3:**
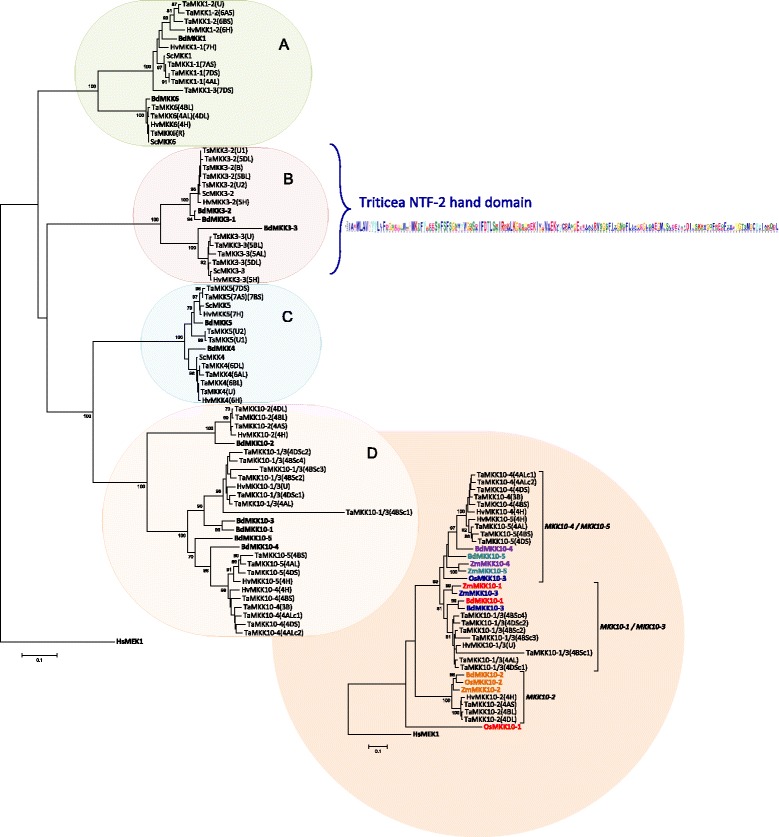
Phylogenetic distribution of Triticeae and Brachypodium MKKs. PK domains of wheat, barley, rye and triticale MKKs were aligned with BdMKKs, and the tree rooted with HsMEK1. Descriptive gene names are followed by chromosome number when available. Accessions used in the analysis are marked in bold in Additional file 3. BdMKKs are bolded in tree to visually separate them from the sequences identified herein. Only accessions with complete PK domains are included in the analysis. MKKs are divided into four clades (a, b, c, and d). MEME analysis shows amino acid distribution of the NTF-2 domain conserved in the C-terminal extensions of MKK3s. An additional tree is presented for clade D (MKK10s), where the Triticeae species were aligned with Brachypodium, rice (Os) and maize (Zm) and the tree rooted with HsMEK1. Font colours are used to distinguish MKK10 orthologues from different species: MKK10–1 (red), MKK10–2 (orange), MKK10–3 (blue), MKK10–4 (purple), MKK10–5 (teal)

Phylogenetic analysis of MKKs from wheat, barley, rye and triticale with the corresponding Brachypodium sequences was also carried out (Fig. 3). Brachypodium hosts 12 members of the MKK subfamily (BdMKKs), which are distributed in four clades (A, B, C and D). Triticeae homologues could be identified for all 12 BdMKKs, except MKK3–1 (Fig. 3). On the other hand, homologues to the other BdMKK3s (MKK3–2 and − 3) were identified in wheat, barley, rye and triticale. It should be noted that a partial *TaMKK3* sequence was identified in genome 3B, where the remaining *TaMKK3s* (two *TaMKK3–2 s* and three *TaMKK3–3*) were found on Chr. 5 (Additional file 3). Interestingly, a partial *TuMKK3* sequence was also identified in addition to the *TuMKK3–2* and *3–3* sequences. Gene duplication of *MKK1* was observed in Triticeae, where three orthologues were identified in wheat and two orthologues in barley (Fig. 3).

In Brachypodium, five MKK10 paralogues are encoded. According to the available resources, barley hosts four MKK10 paralogues, and partial sequences were identified in rye and triticale (Additional file 3). Interestingly, a total of 19 wheat *MKK10s* were identified at different locations on the genome, but as discussed in the next section, the exact number of paralogues (excluding genomic copies) is not clear. As a result, MKK10 numbering in Triticeae is loosely assigned and divided into the following categories: MKK10–1 or 3 (MKK10–1/3), MKK10–2, MKK10–4 and MKK10–5. The *A. tauschii* and *T. urartu* MKK10s identified in the Ensembl database did not offer further resolution of the MKK10 nomenclature. While putative start and stop codons are identified in the predicted sequence for the two *AetMKK10s* and three *TuMKK10s* identified to date (Additional file 3), both *AetMKK10s* and two *TuMKK10s* contain long stretches of unidentified nucleotides (N’s) in the available sequence, and as a result, 30–68% of the protein sequence is not available (Additional file 5). While complete genome sequences are available for these two species [42, 43], some refinement is perhaps necessary to provide greater sequencing depth. It is anticipated that when complete genomic sequences for the identified and unidentified *AetMKK10s* and *TuMKK10s* become available, the MKK nomenclature for wheat and related species can be better resolved.

### Chromosomal distribution and exon-intron composition of wheat and barley MPKs and MKKs

Wheat and barley MPKs and MKKs were mapped to the genome to understand the distribution and organization of MAPKs on the chromosomes of these species. It should be noted that chromosome mapping led to the identification of the several MAPKs for which no sequences were available in GenBank. Transcripts of wheat and barley *MPK25* were not identified in GenBank, but copies of these genes were found on Chr. 5 in barley and on each of the three wheat genomes. Within the MKK subfamily, only *TaMKK1–1*, *1–2*, *3–2* and *6* sequences have been reported to date for wheat in GenBank. In barley, six un-annotated nucleotide accessions corresponding to MKKs could be identified, but for some of these the predicted protein was translated in the wrong frame (Additional files 3 and 5). Chromosome mapping led to identification of the remaining Triticeae MKKs*.*

**Fig. 4:**
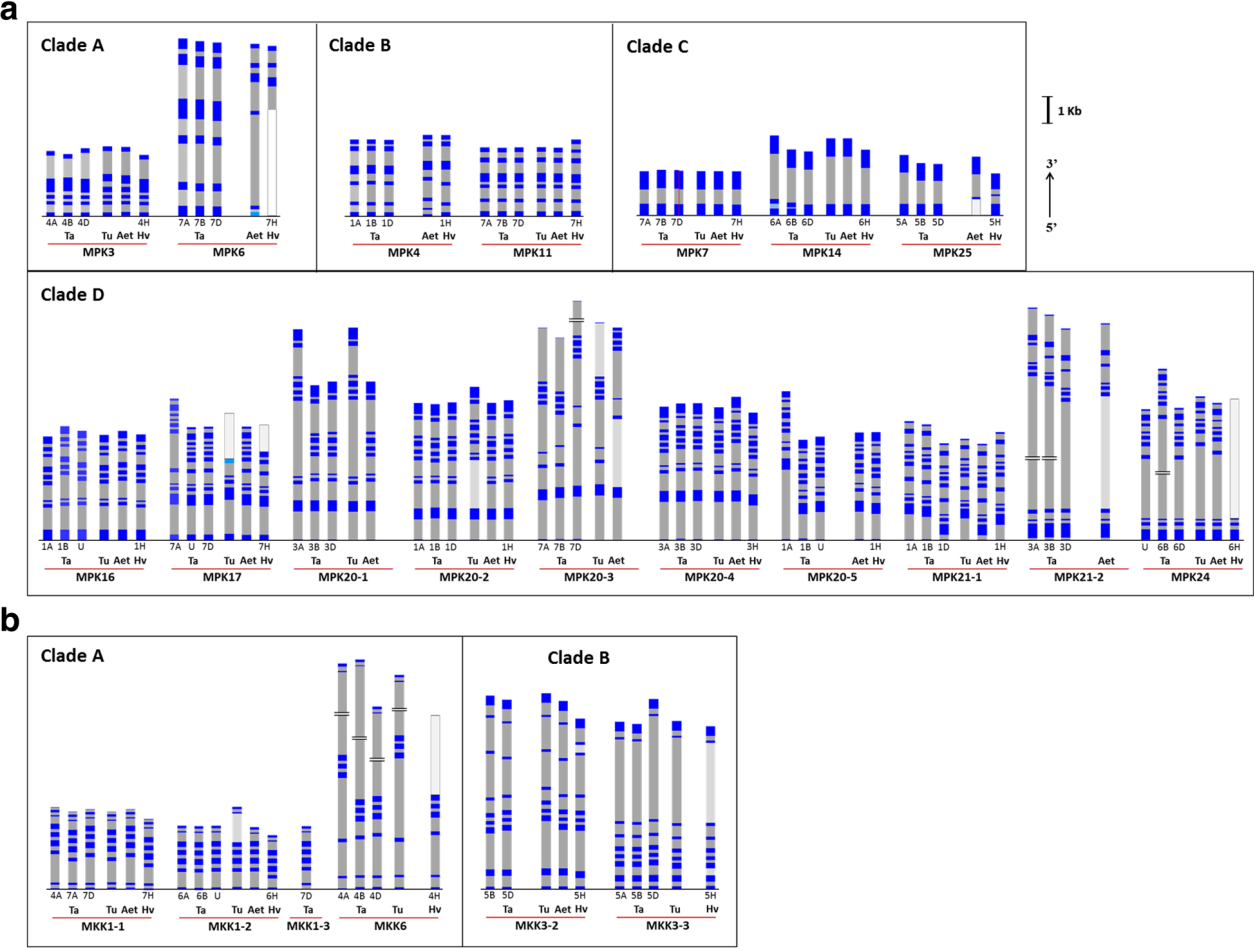
Exon-intron architecture of MAP kinases in Triticeae species. (**a**) MPKs; (**b**) MKKs; Clades C (MKK4s and 5 s) and D (MKK10s) do not have introns and are therefore not represented here. The blue (dark and light) blocks represent the exon lengths and grey (dark and light) blocks represent the intron lengths. The light blocks indicate an uncertain length that could not be verified on a given chromosome due to sequencing noise or other reasons. The blank blocks represent incomplete genomic information of the indicated portion of a gene. A split in the bar (red line) depicts two copies of *TaMPK7(D)* with different intron lengths. Cut bars indicate a size greater than that which is shown here. An arrow depicts orientation of the genes and the sizing bar refers to one kilobase (kb) length of genes. The numbers below bars indicate chromosome number in wheat and barley, where U refers to unknown chromosome number. *A. tauschii* and *T. urartu* chromosome numbers were not availabe and are therefore not indicated

Chromosomal locations for the MPKs are shown in the phylogenetic trees (Figs. 1 and 2) and are also listed in Additional file 3. Different wheat MPKs were consistently found distributed on a distinct subset of chromosomes originating from A, B and D genomes, where three copies were identified for all *TaMPKs* (Fig. 4a). For example, all copies of *TaMPK3* are localized on Chr. 4, and *TaMPK4s* on Chr. 1. It should be noted that there are four cases, *TaMPK16*, *17*, *20–5* and *24*, where a chromosome number was not assigned to one of the three copies. An exception to the “three copy” trend (one copy on each genome) was observed for *TaMPK7*, where four copies were identified, all on Chr. 7, the fourth copy arising from a presumed gene duplication event on 7D. The two *TaMPK7* copies on 7D share 97% identity in their cDNA sequence, and 92% identity (with a cumulative gap of 1% in the intronic region) in the genomic DNA sequence from the start to the stop codon. The 500 bp regions upstream and downstream of the coding region only share 59 and 90% identities, respectively.

Without exception, all *HvMPKs* identified in Ensembl are localized to the same chromosome number as their wheat orthologues (Fig. 4a). By contrast, different paralogues of a given MPK are not necessarily localized to the same chromosome number. For example, *TaMPK20–1* through to *20–5* are distributed on Chr. 1, 3 and 7.

Chromosomal locations for MKKs are shown in the phylogenetic analysis (Fig. 3) and are listed in Additional file 3. Based on the available data, a similar discipline of distribution appears to have been retained for MKKs, including matching chromosome numbers for *HvMKK* orthologues (Fig. 4b), with some notable exceptions for *TaMKK1–1* and *TaMKK10*. *TaMKK1–1* is localized on 7A, 7D and 4A. Interestingly, while only one copy of a putative *TaMKK1–3* was identified, this was also found on 7D. Of the three *TaMKK1–2* copies, two were localized on Chr. 6 while the third could not be assigned a chromosome number. Only two copies of *TaMKK3–2* were found (5B and 5D).

Chromosomal mapping revealed a rather large number of *TaMKK10s*, with a total of 19 identified accessions identified primarily on Chr. 4. It is difficult to discern how many versions of *TaMKK10* are present; therefore, no obvious assignment could be defined based on chromosomal location. Furthermore, *MKK10s* are not known to carry introns in their genomic sequences; as such there are no exon-intron patterns to offer more insights. While some clustering was observed, *MKK10* phylogenetic analyses based on either (a) the PK domain (Fig. 3), (b) the full length protein sequences, or (c) the nucleotide sequences could not clearly resolve the number of *TaMKK10* paralogues.

The abundance of non-coding sequences within a genome, such as introns, is considered as an indicator of genome complexity [44] and intron pattern analyses can reveal insights on the structure and evolution of genes [45–47]. To understand the relationship or divergence among paralogues and orthologues, the exon-intron architecture of wheat*, A. tauschii*, *T. urartu*, and barley MPKs and MKKs was studied. Paralogues of given MPK and MKK genes in wheat have the same number of introns and a similar pattern of exon-intron distribution though different speciation by intron size are noticeable in many genes (Fig. 4). There are few exceptions, however, to the similarity among orthologues. For example, *MPK3* and *4* of wheat mirror with barley rather than its ancestors *T. urartu* and/or *A. tauschii*. On the other hand *HvMPK11* stands apart in its 3′ region from all other orthologues. It should be noted that in some cases, the Ensembl-predicted exons did not correspond to the transcript sequence data available in the nucleotide databases (Triticeae full-length CDS database or GenBank), but alternative splicing showing a different exon-intron pattern often reconciled these differences. For sequences where we propose alternative splicing, the corresponding nucleotide accessions presented in Additional files 2 and 3 are marked with an ‘X’ suffix, and the alternative exon-intron structure is described in Additional files 4 and 5.

Not surprisingly, the wheat exon-intron patterns closely matched those seen in their ancestral genomes (*A. tauschii* and *T. urartu*) with few exceptions as aforementioned. Remarkably, the phylogenetically segregated groups, which are distinguished by the amino acid sequence of the protein kinase domain, share a similar number of introns and exons. For example, while a range of 6–8 introns were identified in clades A and B, no introns were observed for the MKK clades C and D members, (Fig. 4b). The clade A *TaMPKs* (*MPK4* and *MPK6*) had a relatively low number (4–5) of introns. In clade B, with the exception of barley, Triticeae *MPK4s* and *11 s* have 5 introns. The *MPK7*, *14* and *25* Triticeae members of clade C (Fig. 4a), with the notable exception of *HvMPK25*, have a single intron in their genomic sequences. Clade D contains five types of MPKs that share a higher degree of gene complexity (8–10 introns) compared to clades A, B and C MPKs. Among the five paralogues of the *MPK20s*, all have 9 introns except for *MPK20–3*, while *MPK20–1, 20–2 and 20–4* have an almost identical exon-intron distribution profile. By contrast, *TaMPK20–3* has a different exon-intron pattern and intron number compared with other *TaMPK20* paralogues. *MPK17* and *MPK24* (originally thought to be an orthologue of *OsMPK17–2*) not only have different number of introns but also do not match in their exon-intron patterns. Interestingly, the two *MPK21* paralogues (*21–1* and *21–2*) showed little similarity in their exon-intron pattern and number of introns.

### Domain analysis of Triticeae MPKs and MKKs

Conserved domains or signature sequences are highlighted in the MPK alignment file (Additional file 7). The signature sequences identified are: (i) the phosphate binding P-loop, GxGxxG which as further defined as IGxGxYGxV for MPKs [18], found within the ATP binding signature sequence for PKs, (ii) the ‘MAP kinase’ signature found within the PK domain, (iii) the catalytic C-loop, D(L/I/V)K, found within the S/T PK active site signature, and (iv) the activation- or T-loop, TxY. In addition to these sequences, two conserved domains were identified in the C-terminal extensions outside of the PK domain (represented in Fig. 1): (a) the common docking (CD) domain found in clades A and B MPKs, and (b) an EF-hand calcium binding protein (CBP), which was found in all Triticeae MPK17s (with the exception of *T. urartu* for which the sequence appears to be truncated at the C-terminal end), but not observed in Brachypodium or rice.

**Fig. 5:**
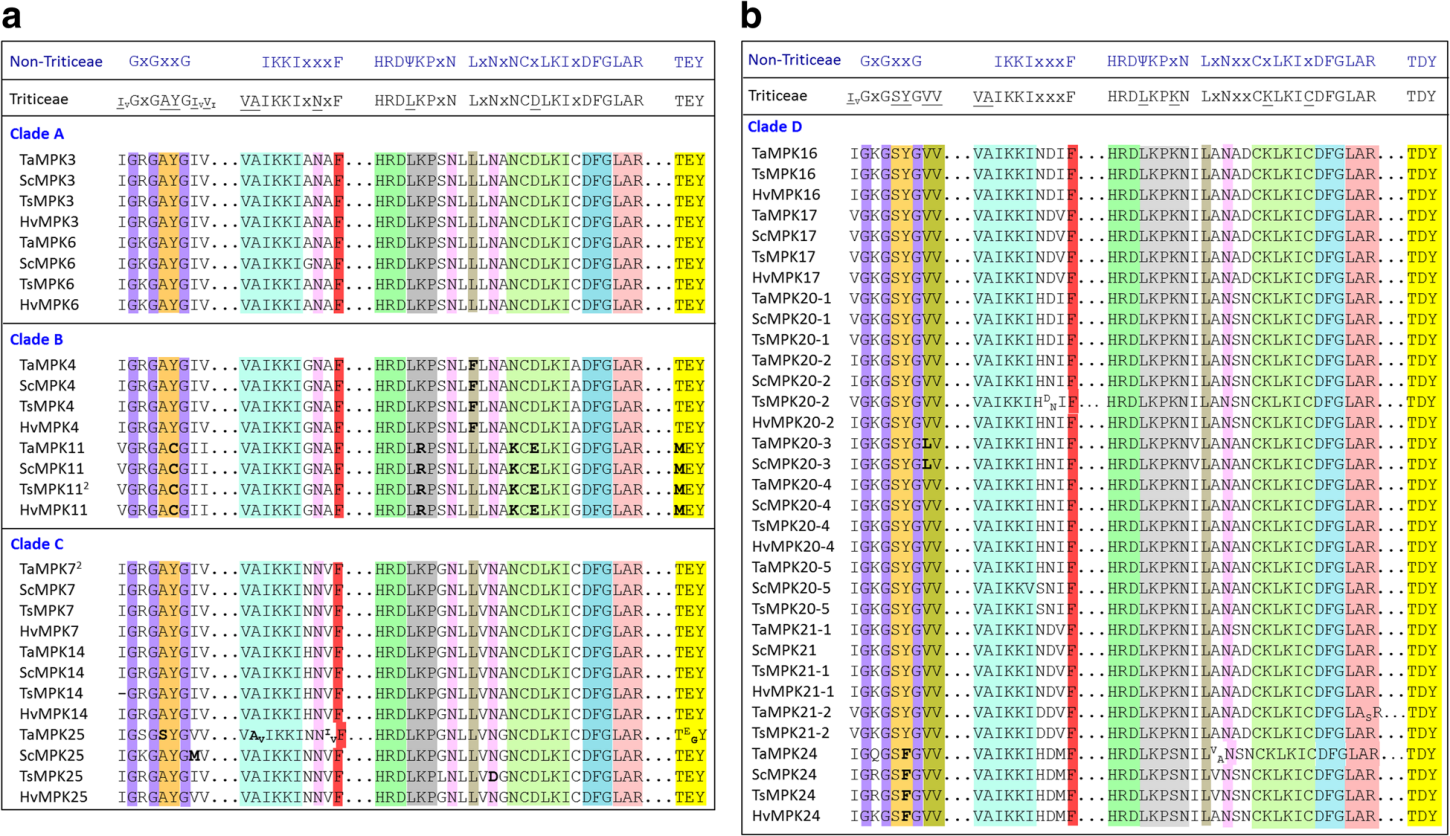
Alignment of important domains of Triticeae MPKs. (**a**) clades A, B and C, and (**b**) clade D MPKs. The amino acid sequences from multiple genomic copies marked in bold in Additional File 3 were aligned in Clustal Omega (www.ebi.ac.uk/Tools/msa/clustalo/) using default settings. The conserved domains are highlighted in different colours. Conserved domains previously identified for plant MPKs [18] are represented above the alignment. All genomic copies of a given MPK from wheat or triticale are represented together. Superscript/subscripts are used where there is sequence variation among copies. Letters in parenthesis after names indicate the source genome. U indicates unidentified chromosome number. The bold letters represent deviation from preferred amino acids at that position

Eleven additional MPK conserved domains, which have been identified in a wide range of plant species [18], are reported here for Triticeae in Additional file 7. Each of the 11 MPK conserved domains was found in all Triticeae MPKs, with some minor variations. In Triticeae MPKs, the IKKIxxxF motif is preceded by VA; the VAIKKIxxxF is highly conserved in Triticeae (Fig. 5a and b) with two exceptions. In one case, one of the three copies of *TaMPK25* (from the D genome) encoded the sequence VVIKKIxxxF (Fig. 5a; Additional file 7). The other exception is found in the CDS sequence of *TaMPK3* in GenBank (accession AF079318) which encodes VASKKIxxxF (Additional file 7). Only a single point mutation in an AUU codon (encoding I) to AGU (the incriminating codon encoding the S residue in of AF079318) would be necessary for this variant to occur. That being said, none of the three genomic copies of *TaMPK3* carry this mutation (Fig. 5a). Taken together with the highly conserved nature of this motif, the variation in AF079318 is likely either a sequencing error or a cultivar-specific SNP. This sequence is part of the ATP binding signature, and there is no physical evidence that an I to S mutation at this site would affect ATPase activity.

All Triticeae MPK11s showed five unique signatures within their conserved domains (Additional file 7). Most notably, the activation loop MEY motif replaces the TEY motif (Fig. 5a) also found in some other clade B MPKs [11, 16, 18, 48]. Interestingly, Triticeae MPK11s also encode the canonical active site signature of Tyr kinases, rather than an S/T kinase signature that is common to the MPK subfamily. Specifically, the DLK active site within this signature is replaced with a DLR motif (Fig. 5a). It should be noted that Tyr kinase signatures are present in other MPKs, including other clade B members such as AtMPK12. The presence of a Tyr kinase signature sequence will automatically eliminate the identification of a ‘MAP kinase’ signature in the PROSITE algorithm since the ‘MAP kinase’ consensus pattern includes elements of an S/T kinase signature. That being said, Triticeae MPK11s all carry other key features of a ‘MAP kinase signature’ including the F and C anchors (Fig. 5a) and the 11 MPK conserved domains (Additional file 7).

Other signatures unique to Triticeae MPK members were also observed, though in most cases these were specific to a whole group. For example, the FxDIYxxxELM motif is shown to be FxDVYxxxELM for all Triticeae MPKs except for the clade D members. In one case, a TGY activation loop reminiscent of the mammalian p38 MAPK [14] was found on one of the three genomic copies of *TaMPK25* (Fig. 5a; Additional file 7), located on 5B, presuming there are no sequencing errors in this region. Although, it should be noted that the wheat TSA database in NCBI carries a partial CDS sequence, accession GFFI01245266, that shares 100% nucleotide identity with *TaMPK25(5BL)* and translates into an 85 residue stretch that includes the TGY motif in question. The other two TaMPK25s encoded on the wheat genome (5AL and 5DL) carry the classic ERK-like TEY motif.

**Fig. 6:**
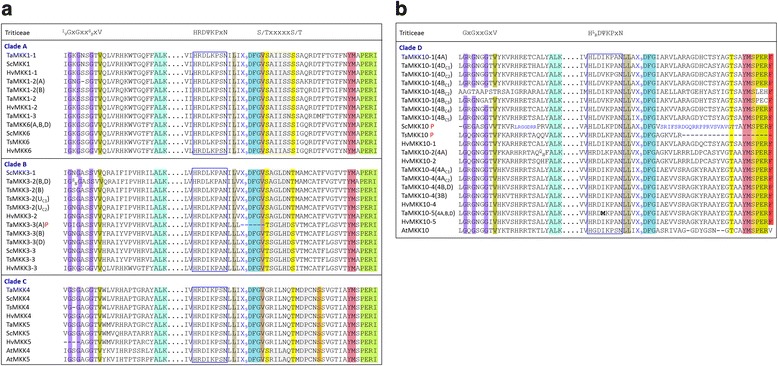
Alignment of important domains of Triticeae MKKs. (**a**) clades A, B and C, and (**b**) clade D MKKs. The amino acid sequences from multiple genomic copies marked in bold in Additional file 3 were aligned in Clustal Omega (www.ebi.ac.uk/Tools/msa/clustalo/) using default settings. The conserved domains are highlighted in different colours. The conserved P-loop anchor found in the ATP binding pocket of PKs (GxGxxG) and the C-loop (DΨK consensus; where Ψ refers to the aliphatic amino acids [79] L/I/V, but not M) are represented above the alignment. All genomic copies of a given MKK from wheat or triticale are represented together. Superscript/subscripts are used where there is sequence variation among copies. In some cases, due to variability among sequences, multiple copies of wheat and triticale are represented. Letters in parenthesis after names indicate the source genome. U indicates unidentified chromosome number. The subscripts (C1….C5) at the end of the MKK names indicate copy numbers. The bold letters represent deviation from preferred amino acids at that position. Smaller font size (blue font) was used for some sequences in clade D for alignment adjustment purposes. The letter P (red font) refers to accessions for which only a partial sequence is available

A detailed analysis of conserved MKK sequences is not available for plant MKKs, but alignments of Triticeae MKKs highlighting the P-, C- and T-loops are presented in Additional file 8. Furthermore, the conserved nuclear transport factor 2 (NTF2) domain found in all plant MKK3s is represented for Triticeae in Fig. 3 and highlighted in Additional file 8. Variations of some of the conserved MPK motifs are also present in Triticeae MKK sequences. For example, the P-loop IGxGxYGxV motif is conserved in MKKs as (I/V)GxGx(S/G)GxV (Fig. 6a). The ATP binding signature in Triticeae MPKs always terminates with the sequence VAIKK, while (F/Y/L)ALK is found at the C-terminal end of Triticeae MKK ATP binding signatures. The MAPK C-loop is defined as D(L/I/V)K, and in Triticeae the sequence HR**DLK**PxN is highly conserved, whereas HR**D(L/I/V)K**PxN is observed for the MKKs, with the exception of TaMKK10–5 where all three genomic copies (4A, 4B and 4D) encode HR**DMK**PxN.

The Triticeae MKK4, 5 and 10 variants all lack properly constructed activation loops ((S/T)xxxxx(S/T)) at the canonical position (Fig. 6a and b), as has also been observed in other species [3, 11]. While for MKK10s this observation has been made for all plant species, in MKK4 and 5 this may be a monocot-specific anomaly as it has only been reported in rice and Brachypodium [11], not in Arabidopsis or other dicots. The activation loop of Triticeae MKKs is preceded by the highly conserved DFGx sequence, and interestingly, a third S/T is present five residues downstream of the activation loop. As shown in Fig. 6a, this highly conserved sequence, DFGV(S/T)xxxxx(S/T)xxxxx(S/T), is also present in AtMKK4 and 5, which, in contrast to the monocot clade C members, do carry canonical activation loop sequences. The Triticeae MKK4 and 5 sequences differ from AtMKK4 and 5 primarily by the absence of the first S/T which is thought to be part of the activation loop. Therefore, while an (S/T)xxxxx(S/T) is present downstream of the canonical T-loop position in Triticeae clade C MKK members, this motif seems to be highly conserved in other MKKs and is not otherwise defined as the T-loop.

### Tissue-specific developmental gene expression and activation of wheat MAPKs

**Fig. 7:**
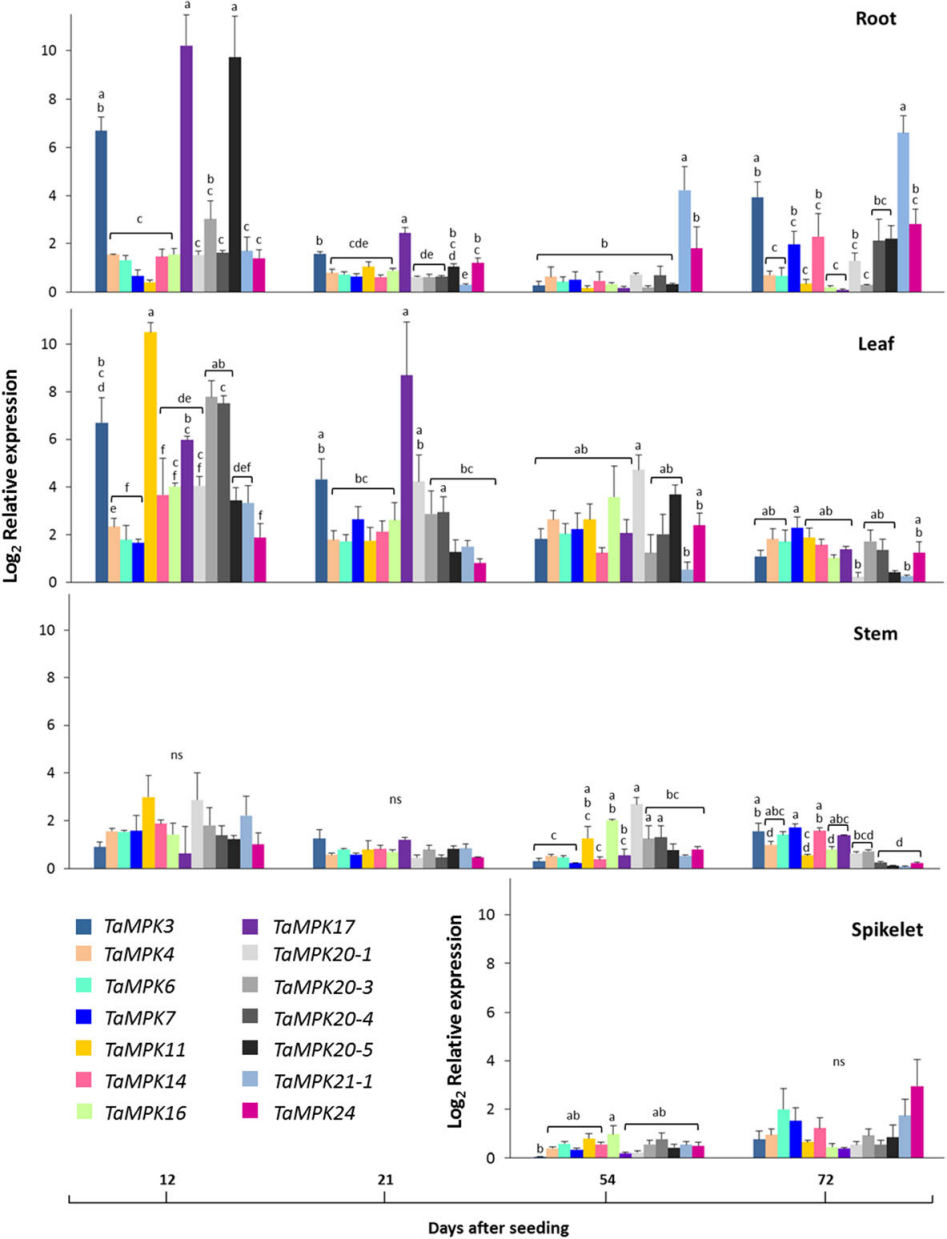
Tissue-specific relative expression of wheat *MPKs* at different developmental stages. The relative expression was measured through qRT-PCR in different tissues at indicated time points of development. Normalized relative expression was calculated from the cycle threshold values using qBASE Plus software, where the calculated expression level is relative to the lowest value in the data set. Bars represent standard error of means (n = 3). Statistical analysis was carried out in SPSS 15.0 using a mixed linear model with an REML estimation method (gene = fixed effect). Bonferroni multiple comparison adjustments were employed to analyze the main effects (*p* ≤ 0.05)

Tissue-specific (root, flag leaf, stem and spikelet) MAPK gene expression patterns at four developmental stages (12, 21, 54 and 72 days after seeding (DAS)) were evaluated by qRT-PCR in the Canadian spring wheat cultivar ‘Superb’. Since there is no untreated reference for a tissue-specific developmental gene expression experiment, expression values are relative to the lowest expression value in the qRT-PCR data set; for this reason all relative expression values are positive integers. Different expression levels were observed among the 14 *TaMPKs* studied (Fig. 7), and in a few instances transcripts were barely detectable. At 12 DAS, relative expression of some of the MPKs in clade A (*TaMPK3*) and D (*TaMPK17 & 20–5*) was higher in the roots compared with other MPKs, but as the roots developed to 72 DAS *TaMPK17* transcript abundance declined to levels near the threshold of detection. In the leaf tissue, one clade B MPK (*TaMPK11*) was present in significantly higher abundance compared with most of the other MPKs at 12 DAS. At 21 DAS there was little difference in expression levels among the MPKs surveyed, with the possible exception of *TaMPK17.* The expression levels of all MPKs was relatively low and undifferentiated in the stem and in spikelets. In general, the MPKs were more actively expressed in younger stages of all tissues, except in the spikelets.

**Fig. 8:**
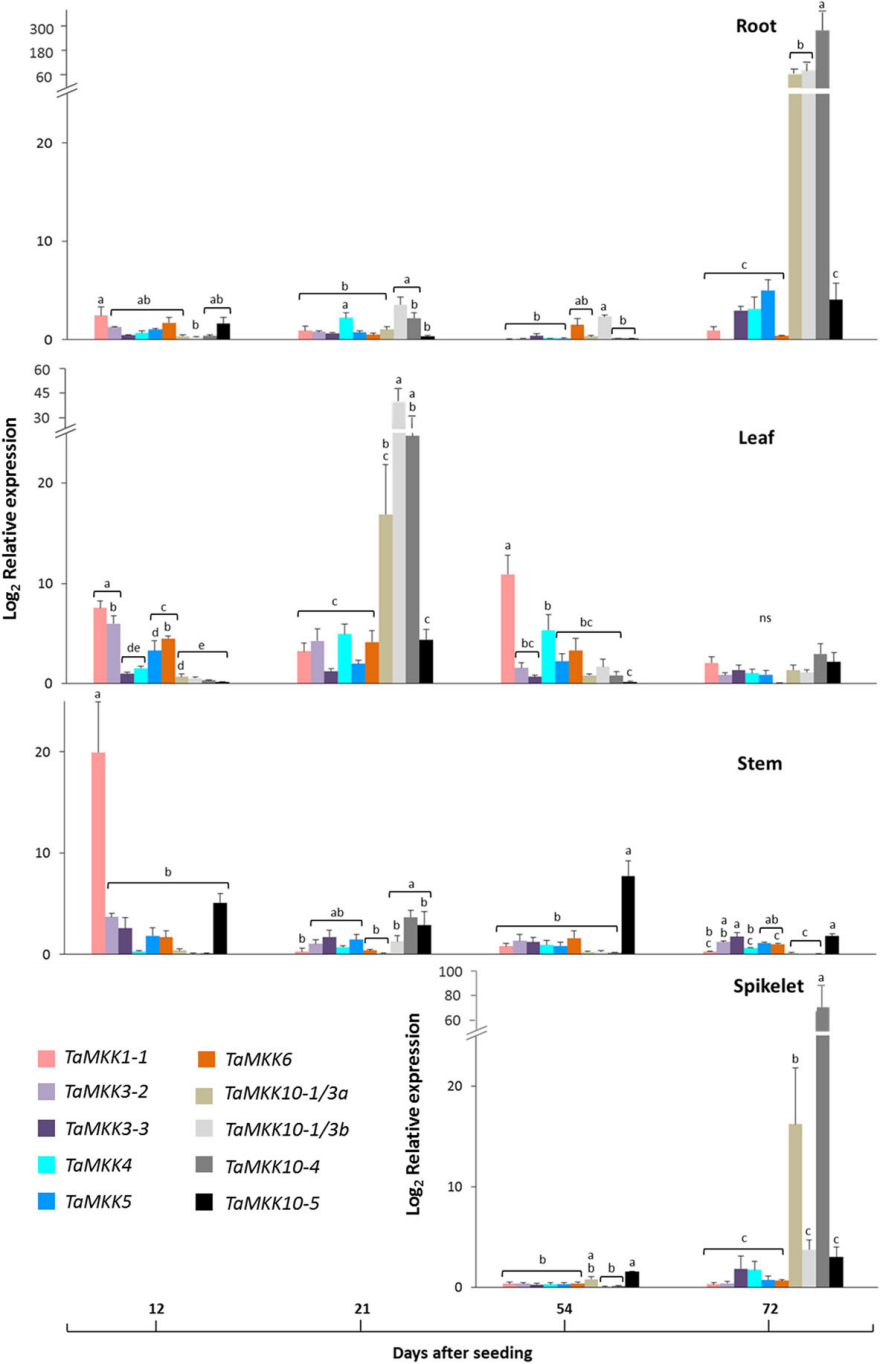
Tissue-specific relative expression of wheat *MKKs* at different developmental stages. The relative expression was measured through qRT-PCR in different tissues at indicated time points of development. Normalized relative expression was calculated from the cycle threshold values using qBASE Plus software, where the calculated expression level is relative to the lowest value in the data set. Bars represent standard error of means (n = 3). Statistical analysis was carried out in SPSS 15.0 using a mixed linear model with an REML estimation method (gene = fixed effect). Bonferroni multiple comparison adjustments were employed to analyze the main effects (*p* ≤ 0.05)

Developmental and tissue-specific expression patterns of ten *TaMKKs* (Fig. 8) were also investigated in ‘Superb’. As reported for *TaMPKs*, while different expression patterns were observed among *TaMKKs*, the levels were generally low. *TaMKK1–1* expression was generally higher at 12 DAS in any given tissue, and expression of various *TaMKK10s* increased at 21 DAS in leaf tissue. Some of the *TaMKK10s* were also expressed at higher levels in the stem and spikelet tissues at 72 DAS.

**Fig. 9:**
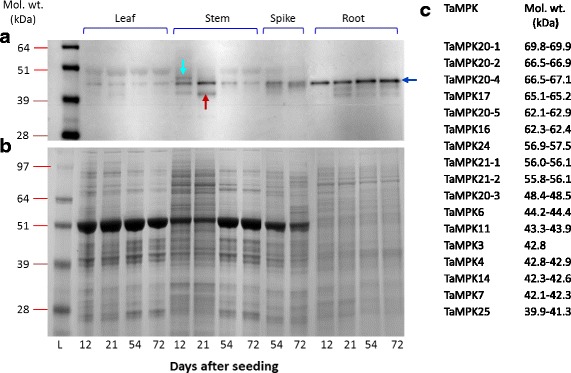
Immunodetection of ERK-like and pERK-like MAPKs in different developmental wheat tissues. (**a**) Immunoblot with anti-pERK1/2; (**b**) coomassie-blue stained gel indicating the loading comparison of proteins in different samples; (**c**) predicted molecular weight range of TaMPKs. L, ladder representing approximate proteins sizes. The arrows pointing to different bands on the blots are discussed in the text

While patterns of transcript levels can offer some insight into potential biological function, translation to the protein and, in the case of MAPKs, the level of catalytic activation are also important components of kinase functionality. To gain insight into these levels of functionality, the distribution of activated wheat MPK proteins in different developing tissues were assessed by immunoblot analysis. Anti-pERK1/2 antibodies were used to detect phosphorylated ERK1/2-like MPKs in different tissues and the results are presented in Fig. 9. Putative phosphorylated ERK-like proteins were observed in the 39 to 51 kDa range (Fig. 9a). This molecular weight range excludes the clade D MPKs with the exception of TaMPK20–3 (48.4 to 48.5 kDa among copies). Relatively higher accumulation of the phosphorylated ERK-like proteins at roughly 45 kDa range (blue arrow) was observed compared to other bands detected. Interestingly, while total protein loaded from root isolations was less than the other tissues assessed (Fig. 9b), the intensity of this band is generally higher when compared with other tissues. A differential accumulation of phosphorylated protein was noticeable in stem tissue at (red arrow), and at the same time an increase in band intensity was observed for the approximately 45 kDa protein (blue arrow) and a band of higher molecular weight observed at 12 DAS faded at 21 DAS (teal arrow).

### Cold and salt stress induced differential expression of wheat MPKs

**Fig. 10:**
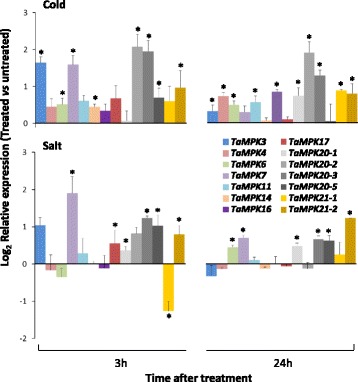
Abiotic stresses and expression of wheat *MPKs*. qRT-PCR was employed to assess differential expression of *TaMPKs* in young seedlings in response to cold and salt stress. Normalized relative expression represents changes in transcript abundance during abiotic stress compared with untreated control plants. Asterisks mark significant differences (treatment vs control) at *p* < 0.05

The effect of cold and salt stress on the expression of 14 wheat MPKs were assessed by qRT-PCR in young seedlings. Cold stress resulted in the up-regulation of eight wheat MPKs at 3 h (*p* < 0.05), and this up-regulation was sustained at 24 h for five of these genes (*TaMPK3*, *6*, *20–2*, *20–3* and *21–2*) (Fig. 10). Meanwhile, up-regulation of four additional MPKs was also observed at 24 h (*TaMPK4*, *11*, *16*, *21–2*). Salt stress resulted in the up-regulation of seven genes at 3 h including *TaMPK7*, *20–1*, *20–3*, *20–5*, *21–2*, which continued to show up-regulation at 24 h. Only one gene, *TaMPK21–1*, was significantly (*p* < 0.05) down-regulated, and this was observede at 3 h in response to salt stress. Some similarities were observed in the expression patterns of MPKs in response to both stresses. For example, *TaMPK20–3* and *21–2* were up-regulated in response to either stress at both 3 and 24 h. Similarily, *TaMPK7* and *20–5* were up-regulated in response to cold and salinity at 3 h, and again at 24 h but in this case only in response to salinity.

### Interaction map of wheat MAPKs

**Fig. 11:**
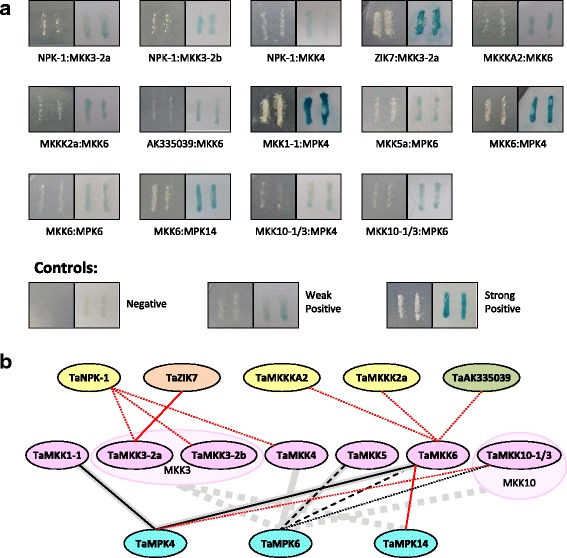
Wheat MAPK interaction map. **a** Pictures of positive Y2H interactions. Robust or weak growth on dropout medium (SC-Leu-Trp-His +3AT) (left interaction boxes) and colour intensity in an X-gal assay (right interaction boxes) indicate strong positive or weak positive interactions, as compared with the controls. **b** Schematic representation of positive interactions observed between TaMKKK-TaMKKs and between TaMKKs-TaMPKs. TaMKKKs from the Raf-like (yellow), ZIK-like (peach), and MEKK-like (green) clades were screened for interactions with TaMKKs (violet), which were screened for interactions with TaMPKs (teal). Positive TaMKKK-TaMKK and TaMKK-TaMPK Y2H interactions are indicated with solid (strong positive), dashed (moderate positive) and dotted (weak positive) black or red lines. Red lines indicate novel interactions unreported in other species for TaMKK-TaMPK interactions. Thick grey solid (strong positive) and dashed (moderate/weak positive) lines indicates MKK-MPK interactions that have previously been reported in other plant species [4, 11, 49, 50, 65]. Homologous interactions in other species were not surveyed for the TaMKKK-TaMKK interactions. It should be noted that Arabidopsis and rice have only one paralogue of MKK3, and Arabidopsis has only one paralogue of MKK10, previously reported interactions are marked as a group for these MKKs. For a list of all pairs assessed for interactions please see Additional file 9

In a yeast two-hybrid screen, seven of the ten TaMKKs tested had positive interactions with one or more TaMPKs and TaMKKKs, in total seven TaMKKK-TaMKK and seven TaMKK-TaMPK interactions were observed (Additional file 9, summarized in Fig. 11). To our knowledge, interactions corresponding to the TaMKKK-TaMKK interactions observed here have not been reported in other plant species. The highest numbers of physical interactions were observed with TaMKK6, for which three TaMKKK partners and three TaMPK partners were identified. TaMKK6 was also the only MKK to show interactions with both upstream and downstream partners.

Two of the seven TaMKK-TaMPK interactions are novel, while the remaining five have been reported in other species. In Arabidopsis*,* MKK6 interacts with MPK4, 6, 11 and also with MPK13 [49], for which there is no wheat homologue. The interactions with MPK4 and 6 were previously confirmed in rice [4]. An additional, previously unreported, interaction with MKK6 was also observed here with MPK14. Another novel interaction was observed with TaMKK10–1/3 and TaMPK4. The only interactions previously reported among the MKK10 homologues are AtMKK10 with AtMPK17 [49] and the MKK10–2 orthologues in rice and maize with Os/ZmMPK6 [4, 50].

No interactions with TaMPKs were observed for the TaMKK3s, TaMKK4, TaMKK10–2 or TaMKK10–4. In Brachypodium, it was previously shown that BdMKK3–1, but not BdMKK3–2, interacts with both BdMPK7 and BdMPK14 [11], the negative result in BdMKK3–2 was confirmed here for TaMKK3–2. Similar observations were reported between AtMKK3 and corresponding AtMPKs [49] and also between ZmMKK3–1 and ZmMPK14 [50].

## Discussion

### Nomenclature of Triticeae MAPKs and discrepancies in the literature

A comprehensive survey and analysis of Triticeae MPK and MKK sequences is presented here. All four classical clades in each of the MPK and MKK subfamilies of plant MAPKs are represented in each of the six species surveyed: hexaploid wheat, two ancestral diploid wheat genomes (*A. tauschii* and *T. urartu*), barley, rye and the wheat x rye hybrid, hexaploid triticale. The availability of genomic sequence information for Triticeae has improved substantially over the past ten years, and with on-going progress in this field additional MAPK genes will ultimately be added to this list.

As wheat and barley MAPK research is starting to grow (Additional file 1) it is critical to establish a consistent and informative nomenclature for the relevant genes. In the wheat and barley literature there are already inconsistencies in the reported MAPK nomenclature (summarized in Tables 3 and 4). For example, TaMPK3 has been named WCK-1, TaMAPK1 and TaMPK4, while TaMPK6 has been reported as FRLS, TaMPK1 and TaMAPK2. Meanwhile, the name MPK3 (or MAPK3) has been independently assigned to TaMPK11 and 14, while the name MPK6 (or MAPK6) has been assigned to TaMPK4 and 14 (refer to Additional file 3 for details). These nomenclature problems are not only evident in the primary literature, but have been imported in some cases into the GenBank databases. The wheat and barley nomenclature presented in the current study is derived from established plant MAPK gene nomenclature guidelines [3, 12], which are based on sequence homology. This MAPK nomenclature model has already been applied in a range of species, including rice and Brachypodium [3, 11, 17–22]. The resulting names for wheat and barley MAPKs therefore differ in several instances from the naming assigned for these genes in GenBank and from that reported in the literature for these species [23–25, 27, 36, 51, 52] (Tables 3 and 4; Additional file 3). On the other hand, the rye and triticale MPKs and MKKs described here have not been previously reported, and therefore have no nomenclature discrepancies.

Orthology-based naming is crucial for facilitating cross-species comparisons, since orthologous relationships provide a starting point for predictions of possible gene function [53]. Even when non-systematic gene names have become established in the literature specific MAPKs (e.g. wound-induced protein kinases (WIPK) and salicylic acid-induced protein kinase (SIPK), orthologues of MPK3 and 6, respectively; [54, 55]), it would be important when using these names to consistently couple them with the systematic MAPK name in order to facilitate comparison of results among different studies. The situation with rice provides a compelling example of the confusion that use of non-systematic nomenclature can generate. Rice orthologues of AtMPK3 and 6 were named OsMPK3 and 6, respectively in 2006 [3], and have subsequently been reported as OsMPK5 and 1, respectively in 2012 [4]. In addition, OsMPK3 and 6 were used to respectively describe the orthologues of AtMPK14 and 4 [4], as well as of AtMPK6 and 3 [56]. Thus, OsMPK3 and 6 each have had at least three different MPK names, in addition to other previously assigned nomenclature. Other examples of discrepancies in existing plant MAPK nomenclature have been compiled in Additional file 10. Additional file 3 will help to correct and then maintain accurate nomenclature in Triticeae.

### MPK24, a novel Triticeae sequence diverged from MPK17

Our survey of genomic databases revealed the presence of a novel MPK variant (MPK24) belonging to clade D, which may be Triticeae-specific. Triticeae MPK24 was initially assumed to be a variant of MPK17 or an orthologue of OsMPK17–2. However, the MPK24 sequences were clearly resolved from the rice, sorghum and maize MPK17–2 sequences in our phylogenetic analysis (Fig. 2), indicating that the MPK24s represent a novel group within clade D. Interestingly, a similar divergence tendency is seen between the rice, sorghum and maize MPK17–2 s and their MPK17–1 paralogues (Fig. 2). However, based on phylogenetic distance, this divergence appears to be more pronounced in the Triticeae. Exon-intron arrangements can also provide clues to potential gene divergence patterns [57], and the exon-intron profiles of MPK17s and MPK24s (Fig. 4), which are independent of phylogenetic metrics, provide further support for the idea that MPK24s are different from MPK17s, and have possibly diverged from the latter. A similar pattern of exon-intron dissimilarity and its reflection in phylogenetic distances was visible in MPK20–3 paralogues.

All Triticeae MPK17s have a single EF-hand CBP motif in their C-terminal extensions (except for TuMPK17, which appears to have a truncated C-terminal end) (Fig. 1; Additional file 7), a signature which is not only absent in Triticeae MPK24, but to our knowledge has not been reported in any other MPK. Typically, EF-hand domains occur in interacting pairs (or even multiples) within a protein that sometimes form hydrophobic pockets [58]. Nonetheless, there are reports of other single EF-hand domain containing proteins [59–61], and conformational changes upon calcium binding have been observed in at least one of these proteins [60]. Interestingly, while there do not appear to be other plant MPKs with EF-hand domains, examination of the TaMKKK gene sequences reported by Wang et al. [9] revealed the presence of tandem EF-hand CBP motifs in numerous TaMEKK-like proteins. Furthermore, cross-talk between calcium and MAPK signalling has been reported in plants [62, 63]. The presence of this novel signature did not affect the alignment for phylogenetic analysis with MPK17s from other species since this motif falls outside of the PK domain. Taken together, our results suggest that (a) MPK24 is a novel MPK specific to Triticeae, and while it is unlikely a variant of MPK17 it may have evolved from MPK17–2, and (b) non-Triticeae monocot MPK17–2 s as a group are more divergent from the canonical MPK17 than previously thought. This MPK24/MPK17–2 divergence would have occurred early in the evolution of Triticeae species, as the genomes of both ancestral parents of wheat, *A. tauschii* and *T. urartu*, carry the MPK24 sequence (Additional file 2). The TaMPK24 sequence shares 98 and 94% identity with the coding sequences of *TaMPK24(6DS)* and *TaMPK24(U*), respectively.

### Triticeae MPK25: ERK- and p38-like MAPKs

Another novel MPK, MPK25, was identified among the Triticeae group. Similar to other clade C members, all genomic copies of *TaMPK25* have one intron (with the exception of barley, which has two) (Fig. 4). However, the three genomic copies of *TaMPK25* share only 85–89% amino acid identity with one another, which contrasts with the much stronger paralogue relatedness (95–99% sequence identity) found for other multi-copy *TaMPK* genes. Interestingly, the Brachypodium genome encodes two paralogues of another clade C member, *BdMPK7–1* and *− 2*, but only one *MPK7* gene was identified within the Triticeae tribe. The possibility thus exists that *MPK25s* are diverged from ancestral clade C *MPK7* sequences, but this hypothesis remains to be tested.

The identification of *MPK25* transcripts in the TSA databases of rye and triticale indicates that these genes are expressed in Triticeae and likely functional. Interestingly, *TaMPK25(5BL)* carries a TGY activation loop reminiscent of the conserved motif of mammalian p38 MAPKs, rather than the ERK-like T(E/D)Y motif typically observed in plants. While other novel activation loop motifs have been described for plant MPKs, for example TSY, TQY, TVY [16, 18] and the MEY motif discussed herein, to our knowledge this is the first report of a plant MPK sequence with a p38-like activation signature motif. In this context, it is interesting that p38-like activity was earlier detected in durum wheat (*Triticum turgidum*; AB genomes) root cell extracts [64]. While the responsible gene was not identified, anti-phospho-p38 reacted with a hyperosomitically-induced protein, which incidentally was not recognized by anti-phospho-ERK. Further to this, treatment with SB203580, an inhibitor of p38-like activity, resulted in reduced immunoreactivity with anti-phospho-p38, whereas PD98059 or U0126 inhibitors of ERK-like activity had no effect.

### Evolution of some MAPK sequences—Divergence from the classical MAPK family?

Clade C MKKs in monocots appear to be missing activation loops in the canonical position, as originally noted in Brachypodium and rice [11], and also observed here in the Triticeae MKKs (Fig. 6a). It was earlier suggested that the activation loop in such proteins may be located a few residues downstream of the canonical position [11], and indeed, the S/TxxxxxS/T sequence (TMDPCNS) located five residues downstream of the canonical position is fully conserved among the Triticeae clade C MKKs (Fig. 6a). Arabidopsis orthologues are also included in this figure to show that this motif is also conserved in MKK4 and 5 sequences in another species; furthermore, TMDPCNS is also found at the same location, downstream of the canonical activation loop site, in the rice and Brachypodium orthologues as reported by Chen et al. [11]. Whether the monocot MKK4s and 5 s have functional activation loops or not, the encoding genes are expressed in wheat tissues (Fig. 8) as well as in Brachypodium [11], and both TaMKK4 and 5 were shown to have a physical interaction with another MAPK in the Y2H assays (Fig. 11).

To date all MKK10s identified in dicots or monocots, including those from the Triticeae tribe, appear to be missing properly constructed activation loops at the canonical position (Fig. 6b). Interestingly, surveys of MKK gene families in several dicot species, namely *B. napus*, cucumber and pepper revealed that no MKK10 homologue was identified in any of these species [17, 20, 22]*.* If dicot MKK10 sequences have evolved to a non-functional state, it is reasonable to assume that the corresponding gene may have been lost in some dicot species. On the other hand, extensive duplications were observed for monocot homologues of this gene, especially in wheat (Fig. 3). In addition, expression of these MKK10 genes was detected in wheat, and transcripts from multiple copies of *TaMKK10* were found in particularly high abundance in root and spikelet tissues at the 72 DAS developmental stage, and in stem tissues at 21 DAS (Fig. 8). Furthermore, it is shown herein that TaMKK10–1/3 interacts with two MPKs (Fig. 11), and this is corroborated with results in rice and Arabidopsis where OsMKK10–2 and AtMKK10 were each shown to interact with an MPK [49, 65]. Together, these data support the hypothesis put forth by Hamel et al. [3] that if MKK10 is in fact non-functional, at least in a classical MAPK cascade, MKK10 gene products may have evolved novel functions, as has been reported for some yeast and mammalian MAPKs which act as scaffolding proteins rather than active enzymes [66].

The same may be true for the Triticeae MPK11s. MPK4 is a close paralogue of MPK11, these two gene products do not appear to be functionally redundant in Arabidopsis [67]. Zeng et al. demonstrated that an *mpk11* mutant in Arabidopsis shows no obvious phenotypic differences from wild-type plants [67]. That being said, AtMPK11 is required for embryo and seed development in the *mpk4* mutant background [68]. Furthermore, AtMPK11 has been shown to be activated by fungal elicitors [68, 69].

Consistent with reports from other monocot species [11, 18, 48], the activation loop for all known Triticeae MPK11 orthologues are MEY motifs. This motif is also present in two dicot species, namely the potato and tomato orthologues of AtMPK4 or 11 [16, 18]. Other variations of the T(E/D)Y motif have also been observed in plants, where the acidic residue (E/D) and/or one of the phosphorylation targets (T or Y) has been substituted with another amino acid [18, 19]. It is not known whether a substitution of the T or Y group, including a T to M substitution such as that observed in monocot MPK11s, affects enzymatic activity. In any case, transcripts of both Brachypodium [11] and wheat (Figs. 7 and 10) *MPK11* have been detected, implying that the expressed protein may have a biological function.

Interestingly, monocot MPK11 orthologues also carry Tyr kinase signatures rather than the classic S/T kinase signatures. While this signature has not been described for MPKs in the literature, it is also present in the AtMPK12 sequence. In fact, it may be that monocot MPK11s are orthologues of AtMPK12 rather than of AtMPK11, as suggested by the clustering presented in Additional file 11. However, a more detailed analysis is needed to test this idea; in a previous phylogenetic analysis with Brachypodium, rice and Arabidopsis, the reported BdMPK11 did cluster with AtMPK11 [11].

### Analysis of gene duplication events in monocots

There seems to be a chromosomal bias for the localization of MAPKs. Interestingly, none of the MPKs/MKKs are known to reside on Chr. 2, although the genomic information on all members of Triticeae is not yet complete. Currently, the significance of this unusual observation is unknown but may be deciphered over time. It was interesting that the barley MAPKs identified in Ensembl are localized to the same chromosome number as their wheat orthologues (Fig. 4a) which is perhaps indicative of the genetic relationship between these species. It does appear as though gene duplication events have occurred since the divergence of these species, for example there are four copies *TaMPK7* where two of these are localized on the D genome. Other gene duplication events seem to have occurred earlier in the evolution of these species. Hamel et al. [3] observed fewer members of the A, B and C MPK clades in rice compared with Arabidopsis, but extensive gene duplication within the D clade, particularly for *MPK20*. Homologues of all five *OsMPK20* variants (along with some additional duplications) were also observed in the monocot species Brachypodium [11], and in five of the six Triticeae species we have surveyed (only four paralogues have been identified to date in *T. urartu*) (Additional file 3). An earlier comparison of monocot and dicot species revealed that, with rare exceptions, dicots carry one or two *MPK20* variants [18], whereas monocot species possessed upwards of four variants. It thus appears that the family of *MPK20* paralogues continued to expand after the monocot-eudicot divergence. It is interesting to note that not all variants of wheat or barley MPK20s are on the same chromosome. For example *MPK20–1* and *20–4* are on Chr. 3, *MPK20–2* and *20–5* on Chr. 1, while *MPK20–7* is on Chr. 7.

There are no known eudicot homologues of *MPK21*, for which two variants appear in both rice and Brachypodium [3, 11]. Both variants, *MPK21–1* and *MPK21–2* (found on Chr. 1 and 3, respectively, in wheat), are represented in all of the Triticeae evaluated here except in rye and *T. urartu* each of which appears to possess only one sequence.

Monocot-specific gene duplication events have also been detected in the MKK family. The rice genome encodes eight MKKs, although three of these are paralogues of MKK10 [3]. The Brachypodium genome encodes homologues of all eight rice MKKs, but additional gene duplication was observed for both *MKK10* and *MKK3* [11]. *MKK3* gene duplication was also observed here in the Triticeae tribe. This may be a monocot-specific gene-duplication pattern, since *MKK3* variants have also been reported in other monocot species, including maize [50], but there are no reported dicot *MKK3* gene duplication events.

Gene duplication was also observed in Triticeae *MKK1*. In wheat and barley, *TaMKK1–1* is located on Chr. 7 (although one of the three wheat copies was found on 4AL), and *TaMKK1–2* on Chr. 6 (Fig. 3). A third variant was also found in wheat *TaMKK1–3* (7DS), although this may be a recent gene duplication event as only a single copy was identified and no orthologue was found in *A. tauschii* (the D genome progenitor for wheat) or *T. urartu* while both of these species do carry *MKK1–1* and *1–2*.

*MKK10* gene duplication is especially interesting. With the exception of wheat, up to three *BdMKK10* paralogues were identified in Triticeae species, with only incomplete DNA sequences available in some cases. By contrast, 19 *TaMKK10s* were identified, though if genomic copies are counted there are considerably less. Unfortunately, due to previously described challenges in assigning nomenclature to these sequences, it is difficult to predict how many versions of the identified *TaMKK10s* are genomic copies and how many are true variants. The assignment of *TaMKK10–2* copies was fairly straightforward: the gene products of three *TaMKK10s* clustered together with MKK10–2 s of other monocots (Fig. 3), and one copy was identified on Chr. 4 of each of the three wheat genomes (A, B and D). By contrast, in several cases multiple copies of other *TaMKK10s* were found on a single genome. For example, the gene products of five *MKK10* sequences clustered together with *BdMKK10–1* and *− 3*, are localized on 4BS, along with one copy on 4AL and two on 4DS. It is not clear if half of these are *TaMKK10–1* and the other half *TaMKK10–3*, or if there is also an additional variant (or two) of wheat *MKK10*, such as *TaMKK10–6* (and *− 7*). These eight sequences have been tentatively named *TaMKK10–1/3* (Fig. 3). There is no obvious trend in the chromosomal distribution pattern to provide further insights and based on the complexity of wheat genome and the available sequencing depth, it is perhaps too early to systematically name the Triticeae *MKK10* genes.

### A glimpse into wheat MAPK function: Comparative analysis of plant MAPKs

The three MPKs best known for their role in plant response to biotic and abiotic stress are MPK3, 4 and 6 [70]. In wheat, up-regulation of *TaMPK3* and *6*, has been reported in response to pathogen elicitors as well as drought stress [28, 33–36]. In the work presented herein, *TaMPK3* and *6* are among the genes that are up-regulated in response to cold and salt stress, and *TaMPK4* expression was also increased in response to cold, but not salt (Fig. 10). Similarly, cold stress has been shown to up-regulate *MPK3* and expression in Brachypodium [11] and maize [71]. *BdMPK6* expression does not appear to have an affect by cold or salinity 3 h after treatment of Brachypodium seedlings; however, *BdMPK4* is up-regulated in response to cold [11]. In Arabidopsis expression of *AtMPK4* and *6*, and activation of their gene products by the upstream AtMKK2, has been reported in response to cold and salt treatments [72]. It is not known whether these enzymes are activated under abiotic stress in wheat, and there are no *MKK2* orthologues in monocot species. The Y2H interaction studies here show that TaMKK6 interacts with both TaMPK4 and 6, and so does TaMKK10–1/3. While these interactions have not been verified *in planta*, MKK6 has also been shown to interact with MPK4 and/or 6 in other species [4, 11, 49, 65]. Interestingly, overexpression of a constitutively active form of OsMKK6 in rice led to constitutive activation of OsMPK3 and enhanced chilling tolerance [73].

As with other MAPKs, MPK3, 4 and 6 are involved in numerous biological functions. For example, in addition to plant defence, AtMPK3 and 6 are involved in stomata development [74] and AtMPK4 is required for pollen development [67]. In wheat, there were no major differences in the abundance of the *TaMPK4* transcript compared with other MPKs in the spikelet during anthesis (54 DAS) or post-anthesis (72 DAS) (Fig. 7). The most notable difference in MAPK expression in the spikelets was the relatively high abundance of *TaMKK10–1/3a* and *TaMKK10–4* at 72 DAS (Fig. 8). Several *TaMKK10* transcripts were also particularly high in leaf (21 DAS) and root (72 DAS) tissues. The biological significance of these tissue-specific expression levels of MAPKs at different stages of development is not yet known.

Often MAPKs are activated within minutes of exposure to a signal and their activation can be fairly transient. For example, AtMPK4 and 6 are activated within the first 15 min of exposure to cold stress, and the activity returns to basal levels within a few hours [72]. Other MPKs may be constitutively active in a specific tissue and/or at a specific developmental stage. For example, in wheat we observed a phosphorylated ERK-like signal at roughly 45 kDa at all four developmental stages assessed from 12 to 72 DAS (Fig. 9a). Interestingly, a similar size product was observed in other tissues as well, with different band intensities. Activation of MPKs occur through a cascade of events, whereby the upstream MKKKs are the first responders followed by MKK activation and finally MPK activation. Lee et al. [49] published the Arabidopsis MKK-MPK interactome in 2008, and subsets of MKK-MPKs interactions were later reported in other species [4, 11, 50, 65]. Singh et al. [75] carried out a library screen to identify interacting partners for a number of rice MKKKs, although no MKK interactions were reported. Wankhede et al. [65] performed a comprehensive analysis of rice MPK-MKK interactions using *in silico* docking studies complemented by Y2H. Some of the interactions they observed by Y2H matched those in Arabidopsis and other rice studies, and some new interactions were also observed. In order to compare interactions between wheat MAPKs and those observed in other species, Y2H experiments were carried out using as bait each of the TaMKKs identified here, with the TaMPKs and with a subset of TaMKKKs. A total of 14 interactions were observed (Fig. 11); three of the 17 TaMPKs screened showed positive interactions with TaMKK(s), while seven of the eleven TaMKKs were shown to have interactions with TaMPK(s) and/or TaMKKK(s). Two interactions not yet reported for orthologues in other species were observed here: one between TaMKK6 and TaMPK14, and the other between TaMKK10–1/3 and TaMPK4. TaMKK10–1/3 was also shown to interact with TaMPK6; similarly, OsMKK10–2 was reported to interact with OsMPK6 in another study [4]. The biological significance of these interactions is unknown. MPK14 homologues are not well characterized and little is known about the function of the MKK10 family.

## Conclusions

The purpose of this study is to provide information that will support and stimulate research in cell signalling in important small grain crops. MAPKs are central to plant signalling networks, and while they are well studied in Arabidopsis and other crops, they have until recently received little attention in cereals. However, the rapidly expanding availability of genomic and transcriptomic sequence data among cereal crops has now made it possible to develop a much more comprehensive picture of the MAPK family in these species. It is important at this point to assign the correct nomenclature to MAPKs in Triticeae species in order to avoid confusion in the literature and to facilitate comparisons with other crops. With these goals in mind, we have surveyed public and in-house sequence databases of six Triticeae species for MAPK sequences, and also explored MAPK interactions and expression patterns. Our findings may be summarized as follows:

1. A total of 301 MAPKs were identified (including genomic copies and multiple accessions) from six Triticeae species: wheat, barley, rye, triticale, *A. tauschii* and *T. urartu*. Each diploid genome encodes up to 17 MPKs and up to 14 MKKs.
2. The MPK17 group is shown to be more divergent in the Triticeae species than other groups, where MPK17 carries an EF-hand CBP domain unique to Triticeae. Furthermore, phylogenetic analysis reported here suggests that the monocot MPK17–2 group may have diverged further from the MPK17 group than previously thought. Coincidentally, MPK24, which appears to be unique to Triticeae, may have branched off of MPK17–2, which is absent in these species.
3. A p38-like activation loop motif, TGY, is reported here for the first time in plants and was found in one of the three genomic copies of TaMPK25, a novel Triticeae MPK.
4. All monocot MPK11’s identified to date, including the Triticeae MPK11s, carry Tyr kinase signature sequences and have MEY activation loops in place of the TEY motif found in other clade B plant MPKs.
5. Fourteen MAPK interactions were observed in a Y2H screen, including two MPK-MKK interaction patterns previously unreported from orthologues in other species: TaMKK6:TaMPK14, and TaMKK10–1/3:TaMPK4.
6. Tissue-specific gene expression analysis at different developmental stages revealed high expression levels of wheat *MKK10* family members, in contrast to low expression levels of *MKK10* orthologues in other species.

Future in depth analyses are needed to understand the specific biological roles of both the novel Triticeae MPKs and those MAPK family members whose functions remain obscure even in other plant species. The correct assignment of Triticeae MAPKs presented here offers the tools to more accurately compare Triticeae MAPK signalling with that of other plant systems, including Arabidopsis and cereal models, and to understand how these cascades are involved in regulating important crop traits.

## Methods

### Sequence identification and domain analysis

Translated BLAST runs were carried out on the wheat CDS sequences in the Triticeae full-length CDS database using the Brachypodium MPK and MKK sequences identified by Chen et al. [11]. Similar searches were carried out using the TSA databases (in-house triticale database and the rye NCBI BioProject PRJDB2278). Collected sequences were accepted based on their alignment with Brachypodium orthologues or if they carried MPK or MKK consensus sequences including the conserved residues D(L/I/V)K in the active site motif, and the S/TxxxxxS/T activation loop for MKKs and TxY for MPKs. Nucleotide sequences were then used to search the Ensembl genomic database for wheat, barley, *A. tauschii* and *T. urartu* homologues. Domain analysis was carried out with ExPASy PROSITE (http://prosite.expasy.org/), which identified the protein kinase domain (PS50011) and related signature sequences: MAP kinase signature (PS01351); PK ATP binding signature (PS00107); S/T PK active site signature (PS00108). Additional conserved domains specific to plant MPKs as defined by Mohanta et al. [18] were also assessed by visual sequence analysis.

Multiple Expectation maximization for Motif Elicitation (MEME) analysis was carried out with the intent of generating sequence distribution figures, rather than identifying domains. Conserved domains of interest from alignments were used as input sequences in the MEME freeware (http://meme-suite.org/tools/meme).

### Phylogenetic analysis

Phylogenetic analysis was carried out in MEGA 7.0. The PK domain of wheat, barley, rye and triticale sequences were aligned with the Clustal W algorithms using the following pairwise and multiple alignment parameters employed by Hamel et al. [3]: (i) pairwise alignment gap opening and extensions of 35.0 and 0.75, and (ii) multiple alignment gap opening and extension of 15.0 and 0.30, respectively. Trees were assembled using the Neighbor-joining method and the Poisson model with 1000 boostraps. Partial MAPK sequences lacking a complete PK domain were not included in the phylogenetic analyses.

### Sampling for gene-expression and immunoblotting

Seeds of Canadian spring wheat cultivar ‘Superb’ or winter wheat cultivar ‘Norstar’ were sown in 1 gal pots containing Cornell Mix [76] and grown in a greenhouse at 21 °C (day) and 18 °C (night) with a 16 h photoperiod. Plants were watered daily as needed and fertilized biweekly with 20–20-20 (N-P-K). At the 5–7 leaf stage (approximately 42 days after seeding (DAS) plants were treated with Tilt™ (2.5 mL L-1 propiconazole sprayed onto wheat leaf surfaces, Syngenta Crop Protection Canada) and Intercept™ (0.24 mg of imidacloprid in 50 mL water per pot) as preventative measures against powdery mildew and aphids, respectively.

For development-related tissue-specific gene expression and immunoblotting, root, stem, flag leaf and spikelet samples were collected from ‘Superb’ plants at 12, 21, 54 and 72 DAS.

‘Norstar’ seedlings (8 DAS) were exposed to abiotic (cold or salt) stress and the leaf harvested 3 and 24 h after treatment. For cold stress, seedlings were moved to a growth cabinet at 4 °C until harvest. For salt stress, the soil was saturated in 150 mM NaCl at time zero.

Three biological replicates were carried out for each experiment, where one biological replicate consisted of five tissue samples (each collected from one plant) pooled together. All samples were flash frozen at harvest and stored at -80 °C for RNA or protein extractions.

### Quantitative reverse transcription-PCR (qRT-PCR)

Total RNA was isolated from ‘Superb’ using Qiagen’s RNeasy® Midi Kit with an on-column DNase digestion. RNA was quantified using a NanoDrop 8000 (Thermo Scientific™) and quality assessed with a 2100 Bioanalyzer (Agilent Genomics). The absence of DNA contamination was confirmed by PCR using housekeeping genes (Additional file 12) with RNA as a template. cDNA was synthesized using 1 μg of total RNA in 20 μL reactions with BioRad’s iScript™ cDNA synthesis kit according to manufacturer’s instruction.

qRT-PCR was carried in 10 μL reactions with 2.5 μL of cDNA RT mix diluted 125-fold, 5 μL of PerfeCTa SYBR® Green SuperMix (Quantabio) and 0.25 μM of each gene-specific primer. Primer sequences are presented in Additional file 12. Following polymerase activation at 95 °C (15 min), 40 quantification cycles were carried out at 95 °C for 15 s, Tm (60 °C for 30s and 72 °C for 30s), followed by a melting curve analysis. Each reaction was repeated with three biological replicates and three technical replicates along with negative controls. Amplification efficiency for each MAPK gene was assessed using a standard curve using a pooled cDNA mixture diluted in a four-fold dilution (1, 1:4, 1:16, 1:64, 1:256, and 1:1024).

For development-related tissue-specific gene expression, the data was processed with qBASE Plus software and CT-values were normalized with two housekeeping genes (elongation factor 1-alpha (*TEF1*) accession M90077 and a hexose transporter (*Contig5*) accession CK155621 [77]; Additional file 12). The results are represented as relative expression—relative to the lowest expressing data point which was set to a value of 1. In the case of *TaMPKs*, the expression values were relative to *TaMPK3* expression in the spikelet at 54 DAS. In the case of *TaMKKs*, the expression values were relative to *TaMKK10–4* expression in the stem at 72 DAS. Therefore, all expression values are positive integers. Log_2_ relative expression was calculated and statistical analysis was then carried out in SPSS 15.0. Relative expression between *TaMPKs* or between *TaMKKs* were compared within each tissue at a given time point using mixed linear model with the REstricted Maximum Likelihood (REML) estimation method, with gene as a fixed effect. Bonferroni multiple comparison adjustment was used to analyze the main effects, with significance set at *p* < 0.05.

Abiotic-stress related gene expression was analyzed using Relative Expression Software Tool (REST) [78]. The data was normalized using *TEF1* and *Contig5* genes and relative expression between untreated control and a treatment was determined. The software output for differential gene expression at *p* < 0.05 of statistical significance was used.

### Protein extraction and immunoblotting

Root, stem, flag leaf and spikelet tissues collected from ‘Superb’ were ground in liquid nitrogen with a mortar and pestle and the powder transferred to a tube with extraction buffer (75 mM NaCl, 15 mM EGTA, 15 mM glycerophosphate, 15 mM paranitrophenylphosphate, 10 mM MgCl_2_, 10 mM DTT, 10 mM NAF, 1 mM Na_3_VO_4_, 1 mM PMSF, 1% Protease Inhibitor Cocktail (Sigma, Catalogue no. P9599), 0.1% Tween-20, 25 mM Tris-HCl, pH 7.8). Extraction buffer was added at a ratio (*w*/*v*) of 1:1 (root), 1:1.5 (stem), 1:2 (flag leaf and spikelets). Samples were collected in 2 mL Eppendorf tubes, which were shaken for 10 min at 4 °C prior to centrifugation at 16,000 x *g* for 10 min at 4 °C. The supernatant was collected and spun for another 10 min at 16,000 x *g*, 4 °C to remove any debris. The supernatant was collected again and samples were quantified using a Bradford assay (BioRad) according to manufacturer’s instructions. Samples were combined with NuPAGE® LDS sample buffer (ThermoFisher Scientific), vortexed and heated at 100 °C for 5 min, and 5 μg of total protein (2 μg for root tissues) of each sample was loaded onto 8 cm 12-well Bolt™ 10% Bis-Tris Plus Gels (ThermoFisher Scientific). Proteins were resolved in NuPAGE® MOPS SDS Running Buffer (ThermoFisher Scientific) at 110 V for 80 min and the gels subsequently fixed in 50% methanol and 10% acetic acid. Uniformity of protein loading was assessed by Coomassie blue staining, keeping in mind that the concentration of Rubisco varies from different tissues and developmental stages.

For immunoblotting, protein samples were resolved on 8 cm Bolt™ 10% Bis-Tris Plus Gels (as described above) and then transferred to 60 cm^2^ PVDF membranes at 40 V for 3.5 h. Immunoblotting was carried out with Western Breeze Chemiluminescent Immunodetection Kit (ThermoFisher Scientific) according to manufacturer’s instructions. The successive washing steps were carried out in distilled water and the solutions provided in the kit. The blots were incubated in primary antibody overnight and the colour developed after 1 h of incubation with the chromogenic substrate provided with the kit. Anti-ERK (Anti-MAP Kinase (ERK-1, ERK-2); M5670 Sigma) and anti-pERK (anti-phospho MAP Kinase ERK1/2 (pThr^202^/pTyr^204^); SAB4301578 Sigma) were used at 1:5000 and 1:1000 dilutions, respectively. Both antibodies were generated using HsERK-derived peptide sequences raised in rabbits (Sigma). The results were confirmed by repeating the experiment for three biological replicates.

### Yeast-two-hybrid (Y2H) assays

Bait and prey plasmids were generated using ProQuest™ Two-Hybrid System with Gateway Technology (ThermoFisher Scientific, Catalog no. PQ10001–01) according to the manufacturer’s protocol. All of the screened MAPKs were first cloned into Gateway pDONR221 and then transferred into the appropriate destination vector according to manufacturer’s instructions. For testing interactions among MPKs-MKKs, and MKKs-MKKKs, the interacting partners of the MAPKs were either cloned into pDEST22 (prey) vector or pDEST32 (bait) vector. The prey vector fuses the protein of interest with GAL4 activation domain (AD) and bait vector with GAL4 DNA binding domain (DBD), which together activates *LACZ*, *HIS3* and *URA3* reporter genes. All the clones/plasmids were verified through PCR and sequencing for the presence of full length genes.

The constructs were transformed into a modified *S. cereviceae* strain (MaV203; Thermofisher Scientific) for the Y2H assay. The strain harbors non-reverting auxotrophic mutations: *leu2* and *trp1* to allow selection for the bait and prey fusion vectors, and *his3* for growth upon induction of the reporter gene GAL1::HIS3. Yeast competent cells were prepared using the *S.c.* EasyComp™ Transformation Kit (Thermofisher Scientific, Catalog no. K5050–01). The competent cells were then transformed with appropriate prey and bait plasmids following manufacturer’s instructions. Four different colonies of the transformed yeast cells were grown on SC-Leu-Trp plates at 30 °C for 18 h along with the following controls: pEXP™32/Krev1 and pEXP™22/RalGDS-wt (strong positive interactions), pEXP™32/Krev1 and pEXP™22/RalGDS-m1 (weak positive interaction), pEXP™32/Krev1 and pEXP™22/RalGDS-m2 (negative interaction), pDEST™32 and pDEST™22 (negative activation), bait plasmid and pDEST™22 (negative activation) and pDEST™32 and prey plasmid (negative activation). From this master plate the yeast cells were replica transferred to the following YPDA plates: (1) SC-Leu-Trp-Ura, (2) SC-Leu-Trp + 5-fluoroorotic acid (0.2%), (3) SC-Leu-Trp-His+ 3-amino-1,2,4 triazol (25, 50, 75 and 100 mM) and (4) YPDA plates overlaid with a nitrocellulose membrane for an X-gal assay. The plates were incubated at 30 °C for 24 h and replica cleaned to leave minimal layers of yeast cells. The phenotypes for plates 1, 2 and 3 were monitored following 2 days of incubation at 30 °C. For the X-gal assays (plate 4), after 24 h of incubation at 30 °C the nitrocellulose filter was transferred to a new plate containing X-gal and buffer as per manufacturer’s protocol, incubated at 37 °C and scored after 24 h. The interaction was scored negative (−) for three negative tests out of the four tests, weak interaction (+) for all positive tests but either the growth of cells or X-gal assay colour were faint, and strong interaction (+++) for all tests positive with robust growth and colour. The screen was discarded when any of the negative controls tested positive. The positive interactions were confirmed by repeating the experiment.

## Additional files

Additional file 1: Number of MAPK publications in Brachypodium and Triticeae since 1996. A quick survey of the literature shows the number of publications focused on MAPKs in Brachypodium, wheat and barley is presented. To our knowledge, there are no publications on rye or triticale MAPKs. This survey included MKKKs; however, due to the large representation of this divergent gene family in plants, some publications on MKKKs may have been overlooked. (PDF 118 kb)

Additional file 2: Triticeae MPK and MKK accessions from Ensembl Plants. The table includes scaffold locations, intron numbers, molecular weight, isoelectric point and gene descriptions. (XLSX 54 kb)

Additional file 3: Triticeae MPK and MKK accessions and nomenclature. Six tables are presented below, one for each species: *Triticeae aestivum* (pages 2–5), *Hordeum vulgare* (pages 6–7), *Secale cereale* (page 8), *xTritosecale* (page 9), *Aegilops tauschii* (page 10), *Triticum urartum* (page 11). Nucleotide accessions were collected from various databases as indicated in the footnote for each table. Translated sequences from nucleotides marked in bold were used for phylogenetic analyses. (PDF 308 kb)

Additional file 4: Genomic sequence of Triticeae *MPKs*. Genomic DNA sequences were obtained from Ensembl. Font colour of predicted genes differentiate UTRs (orange), exons (blue), introns (grey), sequences upstream/downstream of the predicted gene (green). Where Ensembl predictions for exon-intron structure did not match available transcript data, alternative exons are proposed and highlighted in yellow; corresponding accessions have an ‘X’ suffix. Black nucleotides are used for genomic sequence for which Ensembl did not predict a gene sequence or for predicted ncRNA sequences, where proposed exon sequences are highlighted in yellow. The underlined nucleotides highlighted in various colours indicate nucleotide variants as defined by Ensembl. (DOCX 452 kb)

Additional file 5: Genomic sequence of Triticeae *MKKs*. Genomic DNA sequences were obtained from Ensembl. Font colour of predicted genes differentiate UTRs (orange), exons (blue), introns (grey), sequences upstream/downstream of the predicted gene (green). Where Ensembl predictions for exon-intron structure did not match available transcript data, alternative exons are proposed and highlighted in yellow; corresponding accessions have an ‘X’ suffix. Black nucleotides are used for genomic sequence for which Ensembl did not predict a gene sequence or for predicted ncRNA sequences, where proposed exon sequences are highlighted in yellow. The underlined nucleotides highlighted in various colours indicate nucleotide variants as defined by Ensembl. Purple font is used for GenBank sequence that were used to fill in the gaps where complete gene sequence was not identified from Ensembl. (DOCX 274 kb)

Additional file 6: Triticale *MPK* and *MKK* transcript sequences. Transcripts were identified from an in-house transcript assembly library which has been submitted to GenBank by Laroche et al. *TsMPKs* and *TsMKKs* sequences are provided. Blue and red fonts are used to mark putative start and stop codons, respectively. (PDF 152 kb)

Additional file 7: Alignment and domain analysis of Triticeae MPKs. The amino acid sequences from multiple genomic copies, along with the accessions reported in Additional file 3, of all Triticeae MPKs identified were aligned in Clustal Omega (www.ebi.ac.uk/Tools/msa/clustalo/) under default settings. A lower case ‘p’ next to the gene name indicates that only a partial sequence was identified for the given gene. The PK domain is in black font, and the ‘MAP kinase’ signature (specific to MPKs) is bolded where ‘MAP kinase’ flanks (F and C) are in red font; N- and C-terminal extensions in teal font. Missing sequence information, namely ‘X’ residues, are shown in pink font. Highlighted sequences are the ATP binding signature (blue), the catalytic C-loop (red), the activation T-loop (black), CD domain (teal) and EF-hand CBP (pink). Sequence deviations from the T(E/D)Y activation loop of plant MPKs are marked in yellow font. Previously reported conserved MPK domains [18] are written in blue font above the alignment, including the conserved clade-specific docking domain found at the C-terminal end of the PK signature (highlighted grey; deviations from proposed sequence in a darker shade). Deviations from these sequences are highlighted in yellow; some of these deviations were observed in the ATP binding signature and are highlighted in a bright blue. Additional conserved sequences described as putative phosphorylation sites distinct from the T-loop [18] are marked with a box. An asterix indicates a stop codon. (PDF 230 kb)

Additional file 8: Alignment and domain analysis of Triticeae MKKs. The amino acid sequences from multiple genomic copies, along with the accessions reported in Additional file 3, of all Triticeae MKKs identified were aligned in Clustal Omega (www.ebi.ac.uk/Tools/msa/clustalo/) under default settings. A lower case ‘p’ next to the gene name indicates that only a partial sequence was identified for the given gene. The protein kinase domain is in black font, and the N- and C-terminal extensions in teal font. Missing sequence information, namely ‘X’ residues, are shown in pink font. Highlighted sequences are the ATP binding signature (blue) which carries the P-loop consensus sequence (GxGxxG), the catalytic C-loop (red) with the DΨK consensus (where Ψ refers to the aliphatic amino acids L/I/V, but not M), the activation T-loop (black), NTF2 domain (pink). Sequence deviations from the S/TxxxxxS/T activation loop of plant MKKs are marked in yellow font. Other deviations from consensus are highlighted in yellow. An asterix indicates a stop codon. (PDF 173 kb)

Additional file 9: Yeast-two-hybrid table and relevant MAPK sequences. Results from Y2H gave weak (+) and strong (+++) positive or negative (−) MPK-MKK and MKK-MKKK interactions are presented in the table below (p.2). Positive Y2H interactions for orthologous genes observed in Arabidopsis [49], rice [4, 65], Brachypodium [11], maize [50] are also shown. The MPK and MKK sequences used that do not match a nucleotide identifier from Additional file 3 are marked as clones or synthetic (syn), and the alignment for these sequences are provided at the end of this file (p.6–18). TaMKKK sequences are provided (p.4–5) along with phylogenetic analysis (p.3) of MPKKK sequences used here together with MPKKK sequences identified in Wang et al. [9]. (PDF 231 kb)

Additional file 10: Summary of MAPK identifiers and complementary information obtained from eight studies. The table includes gene identifiers and complementary annotations for 8 published sets of MAPKs that were used for comparison in this work. (XLSX 53 kb)

Additional file 11: Phylogenetic analysis of MPK11s. Phylogenetic analysis was carried out on full-length protein sequences of Arabidopsis, Brachypodium, rice and Triticeae clade B MPKs along with MPK4 and MPK11 from six other species. The monocot species (maize (Zm), sorghum (Sb), foxtail (Si), switch grass (Pv)) and eudicots (tomato (Sl) and potato (St)) were each shown to carry one or more clade B MPK with MEY activation loops [18]. SlMPK4–1 (accession JF791807) was previously reported as SlMAPK6 [16]. MPKs with MEY activation loops are shown in blue font. All of the monocot species with the MEY motifs also carried Tyr kinase signatures instead of the classic S/T kinase domain. AtMPK12 carried a Tyr kinase signature, but with a TEY activation loop typical of clade B MPKs. Three clade D members were included in the analysis, namely AtMPK16 and two OsMPK16s. It should be noted that the accession originally described for OsMPK16–2 (LOC_Os08g06060) [18], appears to have inadvertently been permuted with that for OsMPK4–2 (LOC_Os05g05160) (Tapan K. Mohanta, personal communications). (PDF 99 kb)

Additional file 12: qRT-PCR primer sequences. Forward (F) and reverse (R) primers used to amplify wheat *MPKs* and *MKKs*. Housekeeping genes, wheat elongation factor 1-alpha (*TEF1*) accession M90077 and a hexose transporter (*Contig5*) accession CK155621 are shown in blue font. (PDF 85 kb)

## Abbreviations

CBP: calcium binding protein
CD: common docking domain
DAS: days after seeding
ERK: extracellular signal-related protein kinase
JNK: c-Jun N-terminal kinase
MAPK: mitogen-activated protein kinase
MEME: Multiple Expectation maximization for Motif Elicitation
MKK: MAPK kinase
MKKK: MAPK kinase kinase
NTF2: nuclear transport factor 2
PK: protein kinase
qRT-PCR: quantitative reverse transcription-polymerase chain reaction
REML: REstricted Maximum Likelihood
TEF: *Triticum aestivum* elongation factor 1-alpha
Y2H: yeast-two-hybrid
