# Supplementary material for: Analysis of MAPK and MAPKK gene families in wheat and related Triticeae species
Source: BMC Genomics. 2018 Mar 5;19:178. doi: 10.1186/s12864-018-4545-9 (PMC5838963; doi:10.1186/s12864-018-4545-9)
Supplement: Supplementary file 1 — Number of MAPK publications in Brachypodium and Triticeae since 1996. A quick survey of the literature shows the number of publications focused on MAPKs in Brachypodium, wheat and barley is presented. To our knowledge, there are no publications on rye or triticale MAPKs. This survey included MKKKs; however, due to the large representation of this divergent gene family in plants, some publications on MKKKs may have been overlooked. (PDF 118 kb) [file 12864_2018_4545_MOESM1_ESM.pdf]

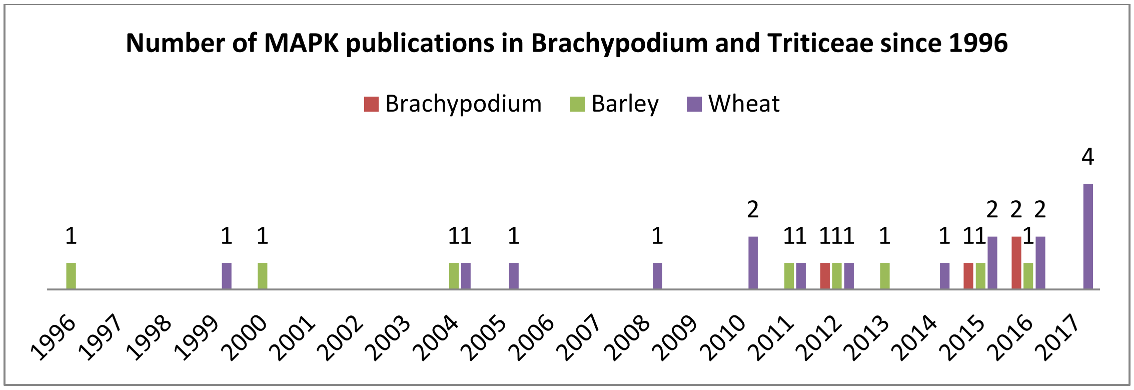

Additional File 1. Number of MAPK publications in Brachypodium and Triticeae since 1996. A quick survey of the literature shows the number of publications focused on MAPKs in Brachypodium, wheat and barley is presented. To our knowledge, there are no publications on rye or triticale MAPKs. This survey included MKKKs; however, due to the large representation of this divergent gene family in plants, some publications on MKKKs may have been overlooked.
