# Supplementary material for: Analysis of MAPK and MAPKK gene families in wheat and related Triticeae species
Source: BMC Genomics. 2018 Mar 5;19:178. doi: 10.1186/s12864-018-4545-9 (PMC5838963; doi:10.1186/s12864-018-4545-9)
Supplement: Supplementary file 3 — Triticeae MPK and MKK accessions and nomenclature. Six tables are presented below, one for each species: Triticeae aestivum (pages 2–5), Hordeum vulgare (pages 6–7), Secale cereale (page 8), xTritosecale (page 9), Aegilops tauschii (page 10), Triticum urartum (page 11). Nucleotide accessions were collected from various databases as indicated in the footnote for each table. Translated sequences from nucleotides marked in bold were used for phylogenetic analyses. (PDF 308 kb) [file 12864_2018_4545_MOESM3_ESM.pdf]

Additional File 3. Triticeae MPK and MKK accessions and nomenclature. Six tables are presented below one for each species: *Triticeae aestivum* (pages 2-5), *Hordeum vulgare* (pages 6-7), *Secale cereale* (page 8), *xTritosecale* (page 9), *Aegilops tauschii* (page 10), *Triticum urartum* (page 11). Nucleotide accessions were collected from various databases as indicated in the footnote for each table. Translated sequences from nucleotides marked in bold were used for phylogenetic analysis (Figs 1, 2 and 3, and Additional File 11).

References

1. Takezawa D. Elicitor- and A23187-induced expression of WCK-1, a gene encoding mitogen-activated protein kinase in wheat. Plant Mol Biol. 1999;40:921-33.
2. Lian W-w, Tang Y-m, Gao S-q, Zhang Z, Zhao X, Zhao C-p. Phylogenetic analysis and expression patterns of the MAPK gene family in wheat (*Triticum aestivum* L.). J Integr Agric. 2012;11:1227-35.
3. Rudd JJ, Keon J, Hammond-Kosack KE. The wheat mitogen-activated protein kinases TaMPK3 and TaMPK6 are differentially regulated at multiple levels during compatible disease interactions with *Mycosphaerella graminicola*. Plant Physiol. 2008;147:802-15.
4. Cevher-Keskin B, Yıldızhan Y, Kulen O, Onarıcı S. Quantitative expression analysis of TaMPK4 and TaTIP1 genes in drought tolerant and non-tolerant wheat (*Triticum aestivum* L.) cultivars. Plant Omics Journal. 2015;8:270-7.
5. Zhan H, Yue H, Zhao X, Wang M, Song W, Nie X. Genome-Wide Identification and Analysis of MAPK and MAPKK Gene Families in Bread Wheat (Triticum aestivum L.). Genes. 2017;8:284.
6. Wen Y, Li X, Guo C, Ma C, Duan W, Lu W, Xiao K. Characterization and expression analysis of mitogen-activated protein kinase cascade genes in wheat subjected to phosphorus and nitrogen deprivation, high salinity, and drought. J Plant Biochem Biotechnol. 2015;24:184-96.
7. Yao S, Wang Y, Yang T, Kou F, Lu W, Xiao K. Expression pattern and function of wheat mitogen-activated protein kinase (MPK) cascade genes under micronutrient-deprived conditions. Acta Physiol Plant. 2016;39:40.
8. Yao S-f, Wang Y-x, Yang T-r, Hao L, Lu W-j, Xiao K. Expression and functional analyses of the mitogen-activated protein kinase (MPK) cascade genes in response to phytohormones in wheat (*Triticum aestivum* L.). J Integr Agric. 2017;16:27-35.
9. Gao Y, Liu X, Stebbing J-A, He D, Laroche A, Gaudet D, Xing T. *TaFLRS*, a novel mitogen-activated protein kinase in wheat defence responses. Eur J Plant Pathol. 2011;131:643-51.
10. Alsaiari S. Functional analysis of FLRS and FLR in wheat during drought stress. *M.Sc. Thesis.* Ottawa, Canada: Carleton University; 2012.
11. Liu, X., Lin, Y., Liu, D., Wang, C., Zhao, Z., Cui, X., Liu, Y., and Yang, Y. (2017). MAPK-mediated auxin signal transduction pathways regulate the malic acid secretion under aluminum stress in wheat (*Triticum aestivum* L.). Scientific Reports 7:1620.
12. Hao L, Wen Y, Zhao Y, Lu W, Xiao K. Wheat mitogen-activated protein kinase gene TaMPK4 improves plant tolerance to multiple stresses through modifying root growth, ROS metabolism, and nutrient acquisitions. Plant Cell Rep. 2015;34:2081-97.
13. Singh D, Bhaganagare G, Bandopadhyay R, Prabhu K, Gupta P, Mukhopadhyay K. Targeted spatio-temporal expression based characterization of state of infection and time-point of maximum defense in wheat NILs during leaf rust infection. Molecular Biology Reports. 2012;39:9373-82.
14. Křenek P, Niks RE, Vels A, Vyplelová P, Šamaj J. Genome-wide analysis of the barley MAPK gene family and its expression patterns in relation to *Puccinia hordei* infection. Acta Physiol Plant. 2015;37:1-16.
15. Abass M, Morris PC. The *Hordeum vulgare* signalling protein MAP kinase 4 is a regulator of biotic and abiotic stress responses. J Plant Physiol. 2013;170:1353-9.
16. Nakamura S, Pourkheirandish M, Morishige H, Kubo Y, Nakamura M, Ichimura K, Seo S, Kanamori H, Wu J, Ando T, et al. Mitogen-activated protein kinase kinase 3 regulates seed dormancy in barley. Curr Biol. 2016;26:775-81.
17. Khalil HB, Ehdaeivand M-R, Xu Y, Laroche A, Gulick PJ. Identification and characterization of rye genes not expressed in allohexaploid triticale. BMC Genomics. 2015;16:281.

Wheat (*Triticum aestivum*)

Gene Name	Nucleotide	Loc	Protein	Reported Names	Comments
**Wheat MPKs**					
TaMPK3	AF079318 [GB]	-	AAC28850	*WCK-1* [GB,1]<br>*TaMAPK1* [2]<br>*TaMPK3* [3]	Genbank Gene ID 542829
	TA63689_4565 [GB]	-	-	*TaMPK4* [4]	Genbank accession provided by [4] could not be retrieved from the database; however, primer sequences show homology to TaMPK3 and its 3'-UTR
	**306486_AA1008960.2** [EP]	**4AS**	A7L5U5	*MPK3* [EP]<br>*TaMAPK30* [5]	
	**320270_AA1033300.1** [EP]	**4BL**	A7L5U5	*MPK3* [EP]<br>*TaMAPK9* [5]<br>*TaMAPK15* [5]	
	**344691_AA1148960.1** [EP]	**4DL**	W5EFF0	*MPK3* [EP]<br>*TaMAPK25* [5]	
TaMPK4	AK330924 [GB]	-	-	*TaMPK6* [6-8]<br>*TaMAPK4* [2]	
	**000118_AA0003960.1** [EP]	**1AL**	W4ZQS8	*TaMAPK14* [5]	
	**030274_AA0084790.2** [EP]	**1BL**	W5A5U1	*TaMAPK28* [5]	
	**061983_AA0206840.1** [EP]	**1DL**	-	*TaMAPK32* [5]	
TaMPK6	AY173962 [GB]	-	AAO16560	*FLRS* [GB,9,10]<br>*TaMAPK2* [3]<br>*TaMPK6* [2]	Genbank Gene ID 543088; insertion encoding a ten residue stretch (277-VFPCGISSLQ-287) within the PK domain of AA061959.1 (see Additional File 6)
	JX646783	-	AG061959	*MAPKflrs* [GB,11]	Partial sequence; 100% sequence ID with AA016560
	AK333212 [GB]	-	-	*TaMPK1* [6-8]	Single nucleotide and amino acid difference between RFL_contig3769 and AK333212
	RFL_contig3769 [TRI]	-	-	-	
	**570369_AA1834630.1** [EP]	**7AS**	-	*TaMAPK48* [5]	
	**593766_AA1954300.1** [EP]	**7BS**	-	*TaMAPK51* [5]	
	**622461_AA2040040.1** [EP]	**7DS**	-	*TaMAPK19* [5]	
TaMPK7	HQ650112 [GB]	-	AET10457	*MAPK5* [GB]	
	HQ650111 [GB]	-	AET10456	*MAPK4/TaMPK4* [GB,12]	
	Ta.7205 [GB]	-	-	*TaMAPK5* [2]	
	tplb0018e09 [TRI]	-	-	*TaMPK4* [6-8]	
	**558385_AA1792950.1.X** [EP]	**7AL**	-	*TaMAPK12* [5]	
	**576902_AA1859240.1** [EP]	**7BL**	A0A088AX68	*TaMAPK54* [5]	
	**603254_AA1979200.2** [EP]	**7DL**	-	*TaMAPK21* [5]	
	**603035_AA1974260.1** [EP]	**7DL**	-	-	
TaMPK11	HAAB01041577 [GB]	-	-	-	Proteins share 100% sequence identity
	**555982_AA1751700.1** [EP]	**7AL**	W5H9L7	-	
	JV870088 [GB]	-	-	*TaMAPK3* [2]	Proteins share 100% sequence identity
	**580661_AA1914980.1** [EP]	**7BL**	-	-	
	**603600_AA1986180.1** [EP]	**7DL**	-	-	
TaMPK14	AK332648 [GB]	-	-	*TaMPK3* [6-8]<br>*TaMAPK6* [2]	
	**487866_AA1573320.1.X** [EP]	**6AS**	-	*TaMAPK36* [5]	
	**514340_AA1658600.1.X** [EP]	**6BS**	-	-	

Gene Name	Nucleotide	Loc	Protein	Reported Names	Comments
	**544171_AA1747030.1** [EP]	**6DS**	-	*TaMAPK45* [5]	
TaMPK16	DQ322665 [GB]	-	ABC54584	*MAPK2A* [GB,11]	Genbank Gene ID 100136975
	DQ322666 [GB]	-	ABC54585	*MAPK2B* [GB,11]<br>*TaMAPK11* [2]	Genbank Gene ID 100137006
	DQ322667 [GB]	-	ABC54586	*MAPK2C* [GB]	
	**020601_AA0078640.1** [EP]	**1AS**	W4ZYA7	*TaMAPK11* [5]<br>*MPK16* [EP]	
	**049733_AA0160590.2** [EP]	**1BS**	A9RAB0	*TaMAPK27* [5]<br>*MPK16* [EP]	
	**642759_AA2122920.1** [EP]	**U**	A9RAB1	*TaMAPK4* [5]<br>*MPK16* [EP]	
TaMPK17	AK333198 [GB]<br>RFL_contig3756 [GB]	-	-	*TaMPK12* [6-8]	Stop codon in PK domain
	AY881102 [GB]	-	AAX20165	*MAPK1a* [GB,11]<br>*TaMAPK14* [2]	
	AY881103 [GB]	-	AAX20166	*MAPK1b* [GB]	
	DQ322668 [GB]	-	ABC54587	*MAPK1C* [GB]	
	DQ334402 [GB]	-	ABC65848	*MAPK1d* [GB,13]	Genbank Gene ID 100125895; premature stop?
	DQ444321 [GB]	-	ABD97883	*MAPK1f* [GB,11]	
	**556562_AA1765430.2** [EP]	**7AL**	A9RAB3	*TaMAPK49* [5]	
	**641980_AA2108810.2** [EP]	**U**	-	*TaMAPK53* [5]	
	**604680_AA2000630.1** [EP]	**7DL**	Q5BU11	*TaMAPK20* [5]	
TaMPK20-1	KT187392 [GB]	-	AKS50324	-	
	Ta.13373 [GB]	-	-	*TaMAPK10* [2]	
	**194002_AA0624350.1** [EP]	**3AL**	A0A0K0YGT2	*TaMAPK38* [5]	
	**224428_AA0796670.1.X** [EP]	**3B**	-	-	Unidentified nucleotides (presumably 27 residue stretch) in C-terminal extension
	**253300_AA0894090.1** [EP]	**3DL**	-	*TaMAPK8* [5]	
TaMPK20-2	AK331818 [GB]	-	-	*TaMPK9* [6-8]<br>*TaMAPK9* [2]	
	tplb0059e09 [TRI]	-	-	-	Predicted protein in database is translated in the wrong frame
	**002534_AA0042880.5.X** [EP]	**1AL**	-	*TaMAPK43* [5]	
	**030959_AA0104540.1** [EP]	**1BL**	-	*TaMAPK6* [5]	
	**063491_AA0227870.2** [EP]	**1DL**	-	*TaMAPK34* [5]	
TaMPK20-3	KR422425 [GB]	-	AKL80627	-	
	**557823_AA1786610.1** [EP]	**7AL**	W5HC04	*TaMAPK3* [5]	
	**577050_AA1864020.2** [EP]	**7BL**	-	*TaMAPK52* [5]	
	**604962_AA2003380.1** [EP]	**7DL**	-	*TaMAPK7* [5]	
TaMPK20-4	AK333309 [GB]<br>RFL_contig3856 [TRI]	-	see comment	*TaMAPK7* [2]<br>*TaMAPK8* [2]	NCBI Genomic Scaffold Match: HG670306; RFL predicted protein is translated in the wrong frame
	**195023_AA0643580.5** [EP]	**3AL**	-	*TaMAPK39* [5]	
	**225288_AA0806810.1** [EP]	**3B**	A0A077RTL3	*TaMAPK16* [5]	
	**250331_AA0866300.5** [EP]	**3DL**	-	*TaMAPK24* [5]	
TaMPK20-5	KR422426 [GB]	-	AKL80628	-	
	tplb0009i20 [TRI]	-	-	*TaMPK7* [6-8]	
	**001596_AA0032670.1** [EP]	**1AL**	-	*TaMAPK41* [5]	

Gene Name	Nucleotide	Loc	Protein	Reported Names	Comments
	**032512_AA0130820.2** [EP]	**1BL**	-	*TaMAPK29* [5]	
	**643598_AA2133390.1** [EP]	**U**	-	*TaMAPK33* [5]	
TaMPK21-1	KR422427 [GB]	-	AKL80629	-	
	Ta.18660 [GB]	-	-	*TaMAPK12* [2]	
	tplb0009k03 [TRI]	-	-	*TaMPK17* [6-8]	
	**002281_AA0040420.1.X** [EP]	**1AL**	-	*TaMAPK42* [5]	
	**032784_AA0134120.1** [EP]	**1BL**	-	-	
	**061695_AA0201870.1** [EP]	**1DL**	-	*TaMAPK5* [5]	
TaMPK21-2	AK330685 [GB]<br>RFL_contig1494 [TRI]	-	-	*TaMAPK13* [2]<br>*TaMPK16* [6-8]	
	**196288_AA0659130.2** [EP]	**3AL**	-	*TaMAPK40* [5]	
	**221708_AA0748020.2** [EP]	**3B**	-	-	
	**249051_AA0836180.1** [EP]	**3DL**	-	*TaMAPK23* [5]	
TaMPK24	AK334728 [GB]	-	-	*TaMPK12;1* [6-8]	
	Ta.68224 [GB]	-	-	*TaMAPK15* [2]	
	RFL_contig5132 [TRI]	-	-	-	
	**640751_AA2072490.1.X + AA2072480.1.X** [EP]	**U**	-	*TaMAPK35* [5]	Fusion of 2 accessions; Unidentified nucleotides (presumably 28 residue stretch) in PK domain
	**513631_AA1646180.1** [EP]	**6BS**	-	*TaMAPK17* [5]	
	**543835_AA1744530.1** [EP]	**6DS**	-	*TaMAPK44* [5]	
TaMPK25	**375843_AA1227850.X** [EP]	**5AL**	-	-	
	**405765_AA1334990.1** [EP]	**5BL**	-	-	
	***TGACv1_Scaffold_433177_ 5DL:99000-100916** [EP]	**5DL**	-	-	
TaMKK1-1	**290148_AA0982230.1** [EP]	**4AL**	**-**	-	
	**570427_AA1835590.1.X** [EP]	**7AS**	**-**	-	
	**HP624471** [GB]	**-**	**-**	-	Coding sequences share 100% identity
	**622118_AA2033130.2** [EP]	**7DS**	**-**	-	
TaMKK1-2	**HP621269** [GB]	**-**	**-**	-	
	**485693_AA1550190.1.X** [EP]	**6AS**	**-**	-	
**Wheat MKKs**					
TaMKK1-1	**290148_AA0982230.1** [EP]	**4AL**	**-**	-	
	**570427_AA1835590.1.X** [EP]	**7AS**	**-**	-	
	**HP624471** [GB]	**-**	**-**	-	Coding sequences share 100% identity
	**622118_AA2033130.2** [EP]	**7DS**	**-**	-	
TaMKK1-2	**HP621269** [GB]	**-**	**-**	-	
	**485693_AA1550190.1.X** [EP]	**6AS**	**-**	-	
	**513382_AA1639520.1.X** [EP]	**6BS**	**-**	-	
	**644387_AA2139180.1.X** [EP]	**U**	**-**	-	
TaMKK1-3	**623511_AA2053970.1.X** [EP]	**7DS**	**-**	-	
TaMKK3	**228257_AA0826360.1.X** [EP]	**3B**	**-**	-	Partial sequence
TaMKK3-2	**KT187393** [GB]	**-**	**AKS50325**	-	
	**405566_AA1330480.6.X** [EP]	**5BL**	**-**	*TaMAPKK2* [5]	
	**432926_AA1394740.1** [EP]	**5DL**	**-**	*TaMAPKK13* [5]	
TaMKK3-3	**373997_AA1187200.3.X** [EP]	**5AL**	**-**	*TaMAPKK4* [5]	
	**405070_AA1319320.2.X** [EP]	**5BL**	**-**	*TaMAPKK18* [5]	

Gene Name	Nucleotide	Loc	Protein	Reported Names	Comments
	**435057_AA1445420.4** [EP]	**5DL**	**-**	*TaMAPKK3* [5]	
TaMKK4	**472053_AA1517260.1.X** [EP]	**6AL**	**-**	-	Described in Ensembl as ncRNA
	**502626_AA1625820.1** [EP]	**6BL**	**-**	-	
	**528809_AA1716310.1.X** [EP]	**6DL**	**-**	*TaMAPKK1* [5]	
TaMKK5	**572257_AA1851770.1** [EP]	**7AS**	**-**	-	
	**592906_AA1946090.1** [EP]	**7BS**	**-**	-	
	**623332_AA2052160.1.X** [EP]	**7DS**	**-**	-	
TaMKK6	**AK330201** [GB]	**-**	**-**	TaMPKK1 [6-8]	
	**290485_AA0985860.1** [EP]	**4AL**	**-**	*MKK6* [EP]	
	**320624_AA1044950.2** [EP]	**4BL**	**-**	*MKK6* [EP]	
	**342786_AA1122060.5** [EP]	**4DL**	**W5EJ68**	*MKK6* [EP]	
TaMKK10-2	**307146_AA1017600.1** [EP]	**4AS**	**-**	-	
	**323005_AA1073590.1** [EP]	**4BL**	**-**	-	
	**344093_AA1143600.1** [EP]	**4DL**	**-**	-	
TaMKK10-1/3	**288275_AA0943400.1.1** [EP]	**4AL**	**-**	*TaMAPKK17* [5]	
	**327917_AA1078630.1.1.X** [EP]	**4BS**	**-**	-	
	**328797_AA1093760.1.1** [EP]	**4BS**	**-**	*TaMAPKK7* [5]	
	**328797_AA1093770.1.1.X** [EP]	**4BS**	**-**	*TaMAPKK7* [5]	
	**329516_AA1102150.1.1** [EP]	**4BS**	**-**	*TaMAPKK6* [5]	
	**329775_AA1103890.1.1** [EP]	**4BS**	**-**	-	
	**362002_AA1175570.1.1** [EP]	**4DS**	**-**	*TaMAPKK12* [5]	
	**362154_AA1177260.1.1** [EP]	**4DS**	**-**	-	
TaMKK10-4	**220919_AA0723740.1.1** [EP]	**3B**	**-**	*TaMAPKK9* [5]<br>*TaMAPKK10* [5]	
	**289532_AA0972640.1.1** [EP]	**4AL**	**-**	*TaMAPKK14* [5]	
	**291020_AA0991400.1** [EP]	**4AL**	**-**	*TaMAPKK15* [5]	
	**329491_AA1101870.1.1** [EP]	**4BS**	**-**	*TaMAPKK5* [5]	
	**361832_AA1173360.1** [EP]	**4DS**	**-**	-	
TaMKK10-5	**292438_AA0998860.1** [EP]	**4AL**	**-**	*TaMAPKK16* [5]	
	**331242_AA1109740.1** [EP]	**4BS**	**-**	-	
	**362002_AA1175550.1.1** [EP]	**4DS**	**-**	*TaMAPKK11* [5]	

Notes:

- **Nucleotide** - Accessions were collected from EP (Ensembl Plants, http://plants.ensembl.org/index.html), GB (GenBank, https://www.ncbi.nlm.nih.gov/genbank/), or TRI (Triticeae Full-length CDS database, http://trifldb.psc.riken.jp/v3/index.pl). Abbreviated nucleotide identifiers were used for EP accessions (for full gene identifiers refer to Additional File 2) which should be should be preceded with 'TRIAE_CS42_CN_TGACv1_', where CN refers to the chromosome number as defined under location. In some cases where nucleotide sequences were identified in EP, accessions were not available—in these instance, genomic locations are provided under Nucleotide instead. Sequences for which alternative splicing is proposed are include the suffix 'X' at the end of the nucleotide identifier.
- **Loc** (location) - Gene sequence were located on chromosomes 1-7 of the A, B and D genomes of *Triticum aestivum*. U refers to unknown location, where U is followed by a number if more than one orthologue was identified with unknown location. Location is not available (-) for sequences obtained from GB or TRI.
- **Protein -** GB or UniProt (Ensembl) protein accessions.
- **Reported Names -** Other names that have been assigned in GB, EP or according to references (marked with superscript numbers). EP names were assigned to the genes where MPKs and MKKs are spelled out ('mitogen activated protein kinase' and 'mitogen activated protein kinase kinase', respectively; note that in some cases UniProt sequence was assigned a different MPK/MKK number than that assigned to the gene.

Barley (*Hordeum vulgare*)

Gene Name	Nucleotide	Loc	Protein	Reported Names	Comments
**Barley MPKs**					
HvMPK3	**MLOC_17814.3** [EP]	**4**	M0V3Q0	*MPK3* [EP]	Coding sequences share 100% identity
	Locus_5782_Transcript_3/3_Confidence_0.864 [HW]	-	-	*HvMPK5* [14]	
HvMPK4	AK366765 [GB]	-	BAJ97968	*HvMPK6* [14]	Coding sequences share 100% identity
	**MLOC_5653.1** [EP]	**1**	F2DS97	-	
HvMPK6	AK355058 [GB]	-	BAJ86277	*HvMPK1* [14]	Proteins share 100% sequence identity
	AK376245 [GB]	-	BAK07440	-	
	AK359140 [GB]	-	BAJ90351	-	
	***7:33935418-33937780(-)** [EP]	**7**	-	-	Partial sequence; full length PK domain was reconstructed with BAJ86277 sequence for phylogenetic tree
HvMPK7	AK252980 [GB]	-	-	*HvMPK4* [14,15]	Coding sequences share 100% identity
	**MLOC_74277.1** [EP]	**7**	M0YY17	-	
HvMPK11	AK364586 [GB]	-	BAJ95789	*HvMPK2* [14]	
	**MLOC_71588.1** [EP]	**7**	M0YQ06	-	
HvMPK14	AK353742 [GB]	-	BAJ84961	*HvMPK3* [14]	Coding sequences share 100% identity
	AK354217 [GB]	-	BAJ85436	-	
	**MLOC_44271.1** [EP]	**6**	F2CQ40	-	
HvMPK16	AK356908 [GB]	-	BAJ88123	-	
	AK354221 [GB]	-	-	-	
	AK251500 [GB]	-	-	*HvMPK14* [14]	Coding sequences share 100% identity
	**MLOC_60926.2** [EP]	**1**	M0XKT1	*MPK16* [EP]	
HvMPK17	AK252439 [GB]	-	-	-	
	AK376500 [GB]	-	BAK07695	-	Proteins share 100% sequence identity
	**MLOC_31474.2** [EP]	**7**	F2EK23	-	
HvMPK20-1	**AK252802** [GB]	U	-	-	Partial or chimeric sequence?
HvMPK20-2	AK361158 [GB]	-	BAJ92365	*HvMPK9* [14]	
	AK361419 [GB]	-	-	-	Coding sequences share 100% identity
	**MLOC_64743.1** [EP]	**1**	F2DB94	-	
HvMPK20-3	**Locus_4929_Transcript_3/3_Confidence_0.714** [HW]	-	-	*HvMPK11* [14]	
HvMPK20-4	AK365649 [GB]	-	BAJ96852	-	Coding sequences share 100% identity
	AK365810 [GB]	-	BAJ97013	-	
	**MLOC_4609.1** [EP]	**3**	F2DP31	-	
HvMPK20-5	AK357581 [GB]	-	BAJ88795	*HvMPK7* [14]	Coding sequences share 100% identity
	**MLOC_36752.2** [EP]	**1**	F2D124	-	
HvMPK21-1	AK377157 [GB]	-	BAK08351	*HvMPK17* [14]	Chimeric sequence?
	**MLOC_11730.2.X** [EP]	**1**	M0UKB5	-	
HvMPK21-2	**AK373134** [GB]	-	BAK04331	*HvMPK16* [14]	
HvMPK24	***6:62507485-62509104(1) + 6:47704546-47706434(-)** [EP]	**6**	-	-	Fusion of 2 chromosomal regions
HvMPK25	***5:449484666-449486231(-)** [EP]	**5**	-	-	
**Barley MKKs**					
HvMKK1-1	**MLOC_5024.1** [EP]	**7**	M0WE95	-	

Gene Name	Nucleotide	Loc	Protein	Reported Names	Comments
HvMKK1-2	***6:24836464-24837555(-)** [EP]	**6**	-	-	Partial sequence; N-terminal region found in GenBank accession AC264970.1
HvMKK3-2	**AK364882** [GB]	**-**	BAJ96085	-	Partial PK domain
	**AB598128** [GB]		BAU62004	*HvMKK3* [GB, 16]	
	**MLOC_51567.4.X + *5:556255205-556260000(-)** [EP]	**5**	M0WHK8	*MKK3* [EP]	Fusion of accession with downstream sequence shows homology with other Triticeae MKK3-2 sequences. Protein accession (M0WHK8) represents translated sequence from MLOC_51567.4
HvMKK3-3	***5:282831404-282831930(-) + 5:85174242-85175936(+) + 5:111354822-111355452(-)** [EP]	**5**	-	-	Fusion of 3 chromosomal regions
HvMKK4	**AK374401** [GB]	**-**	see comment	-	Predicted protein (BAK05597) is in frame with translated sequence but is missing the N-terminal region reported herein
	***6:510407310-510408104(+)** [EP]	**6**	-	-	Partial (N-term truncation)
HvMKK5	**AK370268** [GB]	**-**	see comment	-	Predicted protein (BAK04169) is translated In the wrong frame
	***7:71862432-71862578(+) + MLOC_21954.1.X** [EP]	**7**	-	-	Fusion of accession with upstream sequence; missing sequence between accession and upstream sequence provided, but the sequences have homologies to AK370268.
HvMKK6	**AK250233** [GB]	**-**	-	-	
	**MLOC_4417.1.X + *4:519072245-519072491(-)** [EP]	**4**	-	*MKK6* [EP]	Fusion of accession with downstream sequence
HvMKK10	**MLOC_58682.1.X** [EP]	**1**	-	-	Partial sequence
HvMKK10-2	**AK363295** [GB]	**-**	BAJ94499	-	
	**MLOC_77796.1.X** [EP]	**4**	-	-	Partial sequence
HvMKK10-1/3	***morex_contig_1636696:1-1144(+)** [EP]	**U**	-	-	Partial sequence
HvMKK10-4	***4:23822500-23823800(-)** [EP]	**4**	-	-	
HvMKK10-5	***4:23881977-23883581(-)** [EP]	**4**	-	-	

Notes:

- **Nucleotide -** Accessions were collected from EP (Ensembl Plants, http://plants.ensembl.org/index.html), GB (GenBank, https://www.ncbi.nlm.nih.gov/genbank/), or HW (HarvEST:Web, http://harvest-web.org/hweb/utilmenu.wc).For nucleotide sequences identified in EP, but accessions were not available, genomic locations are provided instead, and marked with an asterix. Sequences for which alternative splicing is proposed are include the suffix 'X' at the end of the nucleotide identifier.
- **Loc** (location) - Gene sequence were located on chromosomes 1-7 of H genome (*Hordeum vulgare*). Location is not available (-) for sequences obtained from GB or HW.
- **Protein -** GB or UniProt (Ensembl) protein accessions.
- **Reported Names -** Other names that have been assigned in GB, EP or according to references (marked with superscript numbers). EP names were assigned to the genes where MPKs and MKKs are spelled out ('mitogen activated protein kinase' and 'mitogen activated protein kinase kinase', respectively; note that in some cases UniProt sequence was assigned a different MPK/MKK number than that assigned to the gene.

Rye (*Secale cereale*)

Gene Name	Nucleotide	Comments
**Rye MPKs**		
ScMPK3	**GCJW01013649**	
ScMPK4	**GCJW01021504**	
ScMPK6	**GCJW01029298**	
ScMPK7	**GCJW01012233**	
ScMPK11	**GCJW01021982**	
ScMPK14	**GCJW01021899**	
ScMPK17	**GCJW01009290**	
ScMPK20-1	**GCJW01023871**	
ScMPK20-2	**GCJW01009928**	
ScMPK20-3	**GCJW01009952**	
ScMPK20-4	**GCJW01023593**	
ScMPK20-5	**GCJW01010817**	
ScMPK21	**GCJW01028630**	
ScMPK24	**GCJW01027829**	Re-amplified sequence does not have stop codon as reported in GenBank
ScMPK25	**GCJW01029388**	
**Rye MKKs**		
ScMKK1	**GCJW01014171**	
ScMKK3-2	**GCJW01010230**	
ScMKK3-3	**GCJW01028545**	
ScMKK4	**GCJW01029276**	
ScMKK5	**GCJW01029147**	Partial sequence
ScMKK6	**GCJW01013226**	
ScMKK10	**GCJW01031072**	Partial sequence

Note: Nucleotide accessions were collected from GeneBank's Transcript Assembly (TSA) database. Bioproject PRJDB2278 [16].

Triticale (*xTritosecale*)

Gene Name	Nucleotide	Loc	Comments
**Triticale MPKs**			
TsMPK3	**Contig10429**	**R/A/B**	
TsMPK4	**Contig67966**	**R/A**	Re-amplified sequence does not have stop codon reported in contig file
TsMPK6	**Contig251617**	**R**	
TsMPK7	**Contig199810**	**B**	
TsMPK11	**Contig120398**	**U**	
	**Contig88156**	**A**	
TsMPK14	**Contig220413**	**R/B**	
TsMPK16	**Contig209684**	**U1**	
	**Contig244429**	**U2**	
TsMPK17	**Contig26009**	**R**	
	**Contig75139**	**A**	
	**Contig38651**	**U**	
TsMPK20-1	**Contig53750**	**U1**	
	**Contig64364**	**U2**	Multiple stop codons
	**Contig185846**	**U3**	
TsMPK20-2	**Contig111052**	**U1**	Deletion or sequencing error; single nucleotide (T) missing in sequence leads to disruption of full length MAPK sequence (see Additional File 5)
	**Contig113411**	**U2**	
TsMPK20-3	**Contig68811**	**U**	
	**Contig19795**	**B**	
TsMPK20-4	**Contig251629**	**U**	
TsMPK20-5	**Contig67306**	**U1**	
	**Contig67307**	**U2**	
TsMPK21-1	**Contig53625**	**U**	Partial sequence
TsMPK21-2	**Contig68757**	**U1**	
	**Contig201182**	**U2**	
	**Contig221182**	**U3**	
TsMPK24	**Contig116794**	**U**	
TsMPK25	**Contig146788**	**U**	
**Triticale MPKs**			
TsMKK3-2	**Contig143511**	**B**	
	**Contig224242**	**U1**	
	**Contig229009**	**U2**	
TsMKK3-3	**Contig90697**	**U**	
TsMKK4	**Contig104479**	**U1**	
TsMKK5	**Contig141775**	**U2**	
	**Contig154845**	**U3**	
TsMKK6	**Contig212597**	**R**	
TsMKK10	**Contig129481**	**-**	Partial sequence

Note: Nucleotide accessions were collected from a TSA project not yet available in GenBank [17]; Locations predicted based on homologies with wheat and rye sequences, where U = unknown.

Goat Grass (*Aegilops tauschii*)

Gene Name	Nucleotide	Protein	Reported Names	Comments
**Goat Grass MPKs**				
AetMPK3	EMT08915.X	-	*MPK3*	
AetMPK4	EMT33725.X	-	*MPK6*	
AetMPK6	EMT30535.X	-	*MPK1*	
AetMPK7	EMT18927	R7W9L5	-	
AetMPK11	EMT18008.X	-	-	
AetMPK14	EMT33478.X	-	*MPK3*	
AetMPK16	EMT19997.X	-	*MPK16*	
AetMPK17	EMT12771.X	-	-	
AetMPK20-1	EMT32532.X	-	*MPK10*	
AetMPK20-2	EMT15153.X	-	-	
AetMPK20-3	EMT10986.X	-	*MPK11*	
AetMPK20-4	EMT09350.X	-	*MPK8*	
AetMPK20-5	EMT10806.X	-	*MPK7*	
AetMPK21-1	EMT25710.X	-	*MPK17*	
AetMPK21-2	EMT27546.X	-	*MPK16*	
AetMPK24	EMT28412.X	-	-	
AetMPK25	EMT00145.X	-	-	
**Goat Grass MPKs**				
AetMKK1-1	*Scaffold59969:73755-76762(-)	-	-	
AetMKK1-2	EMT27166.X	-	*MKK1*	
AetMKK3	EMT30937.X + EMT30938.X	-	*MKK1*	
AetMKK3-2	EMT25805.X	-	*MKK6*	
AetMKK4	EMT05580.X	-	*MKK4*	
AetMKK5	EMT03871.X	-	*MKK4*	
AetMKK6	EMT19002.X	-	*MKK6*	
AetMKK10-5	EMT23048.X	-	*MKK4*	Partial Sequence
AetMKK10	EMT19304.X	-	-	Partial Sequence

Note: Nucleotide and protein accessions and reported names from Ensembl Plants.

Red Wild Eincorn (*Triticum urartu*)

Gene Name	Nucleotide	Protein	Reported Names	Comments
**Red Wild Einkorn MPKs**				
TuMPK3	TRIUR3_20327-T1.X	-	*MPK5*	
TuMPK7	TRIUR3_17440-T1	M8A8M9	*MPK4*	
TuMPK11	TRIUR3_30143-T1	M7ZEQ4	*MPK2*	
TuMPK14	TRIUR3_09881-T1	M7ZYV1	*MPK3*	
TuMPK16	TRIUR3_00870-T1.X	-	*MPK14*	
TuMPK17	TRIUR3_13756-T1.X	-	*MPK12*	Partial sequence
TuMPK20-1	TRIUR3_21548-T1.X	-	*MPK10*	
TuMPK20-2	TRIUR3_19922-T1.X	-	*MPK9*	
TuMPK20-3	TRIUR3_18187-T1.X	-	*MPK11*	
TuMPK20-4	TRIUR3_32310-T1.X	-	*MPK8*	
TuMPK21	TRIUR3_01866-T1	M8A5Z4	*MPK17*	
TuMPK24	TRIUR3_08179-T1.X	-	*MPK13*	
**Red Wild Einkorn MKKs**				
TuMKK1-1	TRIUR3_03040-T1.X	-	*MKK1*	
TuMKK1-2	TRIUR3_10169-T1.X	-		Described in Ensembl as nontranslating CDS
TuMKK3	TRIUR3_00549-T1.X	-	*MKK6*	Partial sequence
TuMKK3-2	TRIUR3_07377-T1.X	-	*MKK6*	
TuMKK3-3	TRIUR3_10006 + 10007	-	*MKK1*	Fusion of 2 accessions; 'Other name' associated with TRIUR_10006
TuMKK4	TRIUR3_14235-T1.X	-	*MKK4*	
TuMKK5	TRIUR3_05471-T1.X	-	*MKK5*	
TuMKK6	TRIUR3_04435-T1	M7YY42	*MKK1*	Stop codon not reported in Ensembl, but is found in 1st three nucleotides downstream of proposed coding sequence
TuMKK10-1/3	TRIUR3_19643-T1.X	-	*MKK4*	Partial sequence
TuMKK10-4	TRIUR3_30060-T1.X	-	-	
TuMKK10	TRIUR3_19642-T1.X	-	-	Partial sequence

Note: Nucleotide and protein accessions and reported names from Ensembl Plants.
