# Supplementary material for: Analysis of MAPK and MAPKK gene families in wheat and related Triticeae species
Source: BMC Genomics. 2018 Mar 5;19:178. doi: 10.1186/s12864-018-4545-9 (PMC5838963; doi:10.1186/s12864-018-4545-9)
Supplement: Supplementary file 4 — Genomic sequence of Triticeae MPKs. Genomic DNA sequences were obtained from Ensembl. Font colour of predicted genes differentiate UTRs (orange), exons (blue), introns (grey), sequences upstream/downstream of the predicted gene (green). Where Ensembl predictions for exon-intron structure did not match available transcript data, alternative exons are proposed and highlighted in yellow; corresponding accessions have an ‘X’ suffix. Black nucleotides are used for genomic sequence for which Ensembl did not predict a gene sequence or for predicted ncRNA sequences, where proposed exon sequences are highlighted in yellow. The underlined nucleotides highlighted in various colours indicate nucleotide variants as defined by Ensembl. (DOCX 452 kb) [file 12864_2018_4545_MOESM4_ESM.docx]

**Additional File 4. Genomic sequence of Triticeae MPKs.** Genomic DNA sequences were obtained from Ensembl. Font colour of predicted genes differentiate UTRs (orange), exons (blue), introns (grey), sequences upstream/downstream of the predicted gene (green). Where Ensembl predictions for intron/exon structure did not match available transcript data, alternative exons are proposed and highlighted in yellow; corresponding accessions have an ‘X’ suffix. Black nucleotides are used for genomic sequence for which Ensembl did not predict a gene sequence or for predicted ncRNA sequences, where proposed exon sequences are highlighted in yellow. The underlined nucleotides highlighted in various colours indicate nucleotide variants as defined by Ensembl.

**>TaMPK3(4AS) TRIAE_CS42_4AS_TGACv1_306486_AA1008960.2**

GGTTGGTGCGTGCAGCGCGCGTCCATACAAGAGAGCAGCAGCTGCTGCCGGTTGACCGTT

GAACCCTCCGAGCCGTCCGATGAGCGCGCTGCCACGAGGCGGCCTCCATCGGGCCGTCCG

TCCCGGCCCCTCAATTCCCGGGCGATACGCACAGAAAAGAGCCAGAGACCGCGCGCACCA

GCACAGCACAGCAGCTGTGGTGTGCTCCCGCTCGCTTTCTCGCTACCTTCCTCAGCTGGG

CCGAGAGCATTAGCGGAGCCGCCCTTTTCTATTTATAATTCTCTCCTCCTCGCCCCTACA

AGAATCGAGAGCCGGGATAAAGTCAATCATCAGCTCAGCCTCAGCCTCATCCCGTTGCGC

GTGCGTGCGTGCGTGTTGCCTCGCTCCTGTGATTGGGTTTTGAGAGTTTCTTGGCTGTGC

TTGAGAATGGACGGCGCTCCGGTGGCCGAGTTCCGGCCGACGATGACGCACGGCGGCCGC

TTCCTCCTCTACAACATATTCGGCAACCAGTTCGAGATCACGGCCAAGTACCAGCCGCCG

ATCATGCCCATCGGCCGCGGCGCCTACGGGATCGTCTG

gtacgtacgttccgcccgtcttaatttgctcctcaatcaccatcgatttcttgccaggtt

tggtcaagccgttgcggctctgatcggagaacagaggggggattggctctgggcctctgg

ccatggggaaagatgggattaggccaattcactcctttctgtgaacaacactagtttagt

cgcactatatttttatcagttgagcaaatatgttagtaatctgttagtgctctgcggttg

gtggtgcttaatcttgtcctgaaattccacttgtggttgcgtgggcgcag

CTCGGTGATGAACTTCGAGACGAGGGAGATGGTGGCAATCAAGAAGATCGCCAACGCCTT

CGACAACAACATGGACGCCAAGCGCACGCTCCGGGAGATCAAGCTCCTCAGGCACCTCGA

CCACGAGAAC

gtaatcagcatctatctcttccgcttcagatgtgtgtatgtatctgactgaattcggcaa

ttcaggcgctaacctgttactctacatggatgcag

ATAGTAGGCCTCCGAGATGTGATCCCGCCGGCGATCCCGCAGTCCTTCAACGACGTCTAC

ATCGCCACTGAGCTCATGGACACGGACCTCCACCACATCATCCGCTCCAACCAAGAACTC

TCGGAAGAACACTGCCAG

gtagtaaagagaagagaaataagccatcgattttgttcatgtttgtgacaatttgatccg

tatattcatgcaccatggtgttcttgggtggcttccag

TACTTCCTGTACCAGCTGCTGCGCGGCCTCAAGTACATCCACTCGGCGAACGTGATCCAC

CGCGACCTCAAGCCGAGCAACCTGCTGCTGAACGCCAACTGTGACCTCAAGATCTGCGAC

TTCGGCCTGGCGCGGCCGTCATCGGAGAGCGACATGATGACGGAGTACGTGGTCACGCGG

TGGTACCGGGCCCCGGAGCTGCTGCTCAACTCCACCGACTACTCCGCGGCCATCGACGTC

TGGTCCGTCGGCTGCATCTTCATGGAGCTCATCAACCGCGCGCCGCTCTTCCCGGGGAGG

GACCACATGCACCAGATGCGGCTCATCACGGAGGTGATCGGCACCCCCACCGACGACGAC

CTGGGATTCATCCGGAACGAGGACGCCAGGAGGTACATGAGGCACCTGCCGCAGTTCCCT

CGCCGGTCCTTCCCGGGCCAGTTCCCCAAGGTGCAGCCCGCCGCGCTGGACCTCATCGAG

AGGATGCTCACCTTTAACCCGCTGCAGAGGATCACAG

gtgcgttgcgtcccacgtccgcgtcactctgctttgatcttcacgtgtgaatttcgtagc

gccgcaccgcactgcacgcaatgctgctgctgttagagcgccgtgctttgttgactggct

ttgtacggccagcgttggggtcagtgtcagtgctaaaaacatctttgtgctgggtataag

taggagtagatgcaatctgcagccacttcggcccgtatatttaagtttactgcatgtgga

catgtgctccctttttagacaaaaaccatgtgtgctgcccacttgattaagaaaagatct

ttctttgcctatctttggagctagtagtatctgctgtagacgacaagggctgtgcgtgta

gtgcagaatcacacttttttttagaagaaacgtatctgtttttggcaacagatatggatg

gaagagatctgctgcagctactctcaaaggctagattcctgtatctgtttcacaacagca

ataatagaagtagctttttgagacgtcgaccagacaattctagtgaaaagaaggctagga

agcaaaccgtccgtctcataatatagaagtgtttttgtcattatatagtcccccagtctc

ataatagtgtttttgacactacaggacggagggagtacattttatttttcttcggttata

cttgatgtcttagtatgcattctcgctgaaattgctcttgtcacgtagaaagtttttttg

ttttttgaaacatggcaatttttttgccattttcattgaataaggagtatcagagctctt

gtcaggtaggaagttatgtctcatcagttaattagtgtcttactggtgtggattcttact

gaatttgctattgtgatgcag

TTGAAGAGGCGCTGGAGCACCCGTACCTAGAGCGGCTTCACGACGTCGCCGACGAGCCCA

TCTGCACGGACCCCTTCTCCTTTGACTTCGAGCAGCACCCTCTGACGGAAGACCAGATGA

AGCAACTCATATTCAACGAAGCCCTGGAGTTGAACCCCAACTTCCGATACTAGATGATTC

ATTCCTAGTTACCCAACCCCCAACAGTATGATTTCGGTATAAAACTGTCTGTAAATAGGA

GTAAGATCAAAGAACATGGTAGATTGCGCACTGCATTTGTTTGTTTATTCTGTGTAAGTT

GTTGCTGCTTCTTCAAACCTTTGTCCCTCAGCTGTGCGAATAAAAAGTTTGGAGCATGAT

TTGAGGGCAAATCATTCTCTAGTTTATGAAGAAATGGTTTTTATTAATCAGGTATCCATT

C

**>TaMPK3(4BL) TRIAE_CS42_4BL_TGACv1_320270_AA1033300.1**

TACGCACAAAAAAAGAGCGAGAGACCGCGCGCACCAGCACAGCAGCTGTGGTGTGCTCCC

GCTCGCTTTCTCGCTACCTTCCTCAGCTGAGCCGAGAGCAAAGCATTAGCGGAGCCGCCC

TTTTCTATTTATAATTCCCTCCTCCTCACCCCTACAAGAATCGAGAGCCAGGATAAAGTC

AATCATCAGCTCAGCCTCATCCCGTTGCGCGTGCGTCCGTGCGTGTTGCCTCCTGTGATT

GGGTTTCCGGTTAGTTTCTTGGCTGTTTGAGAATGGACGGCGCTCCGGTGGCCGAGTTCC

GGCCGACGATGACGCACGGCGGCCGCTTCCTCCTCTACAACATATTCGGCAACCAGTTCG

AGATCACGGCAAAGTACCAGCCGCCGATCATGCCCATCGGCCGCGGCGCCTACGGGATCG

TCTG

gtacgtaccttcgcctcatcttaatttgctcctcaatcaccatcgatttcctgccaagct

tgttcgagtcgttgcggctctgatcggaaaacagagggggaattggctcgggccatgggg

aaagatagggttaggccaattcacgcctttctgtgaacaacactagtttagtagcactat

acttttatcagttgagcaaatatgttagtaatctgtcagtgctcagtggttggtggtgtt

taatcatgtcctgaaattccacttgtggttgcgtgggcgcag

CTCGGTGATGAACTTCGAGACGAGGGAGATGGTGGCAATCAAGAAGATCGCAAACGCCTT

CGACAACAACATGGACGCCAAGCGCACGCTCCGGGAGATCAAGCTCCTCAGGCACCTCGA

CCACGAGAAC

gtaatcaacatctttctcttccgcttcagatgtttgtatctgactgaattcggcaattca

ggcgctaacctgatacgctgtatcgacggacatcacacgcag

ATAGTAGGCCTCCGAGATGTGATCCCGCCGGCGATCCCGCAGTCCTTCAACGACGTCTAC

ATCGCCACCGAGCTCATGGACACGGACCTCCACCACATCATCCGCTCCAACCAAGAACTC

TCAGAAGAACACTGCCAG

gtagtaaggagtaaggactaaggagaagagaagggcatcatattctgaatgaacagaaat

aagccatcgattttgttcatatttgtcacgatttgatccgtatgtatattcatgtgccgt

ggtgttcttgggtggctttcag

TACTTCCTGTACCAGCTGCTGCGCGGCCTCAAGTACATCCACTCGGCGAACGTGATCCAC

CGCGACCTGAAACCGAGCAACCTGCTACTGAACGCCAACTGCGACCTCAAGATCTGCGAC

TTCGGCCTCGCGCGGCCGTCGTCGGAGAGCGACATGATGACGGAGTACGTGGTCACGCGG

TGGTACCGGGCCCCGGAGCTGCTGCTCAACTCCACCGACTACTCGGCCGCCATCGACGTC

TGGTCCGTCGGCTGCATCTTCATGGAGCTCATCAACCGCGCGCCGCTCTTCCCGGGGAGG

GACCACATGCACCAGATGCGGCTCATCACGGAGGTGATCGGCACCCCCACCGACGACGAC

CTGGGCTTCATCCGGAACGAGGACGCCAGGAGGTACATGAGGCACCTGCCGCAGTTCCCT

CGCCGGTCCTTCCCGGGACAGTTCCCCAAGGTGCAGCCCGCCGCGCTGGACCTCATCGAG

AGGATGCTCACCTTCAACCCGCTGCAGAGGATCACAG

gtgcgtccgcgtcactccgctttgatcttcacgtgtgaatttcgtagtgccgcaccgcac

tgcacgcaatgctgctgctgttagagcgccgtgctttgttgactggcttcgtacggccag

cgttggggtcagtgtcagtgcttaagacatctttgtgctggttataagtagatgcaatct

gccgccacttcgacccctatttttaagtttactgcatgtggacatgtgctccctttttag

acaaaaaccatgtgttctgcccacttgattaagaaaagatctttctttgcctatctttgg

agctagtagtatgtgctgtagacgacaaggactgtgcgtgtagtgcagaatcacactttt

tttagaagatcgtactgtatctgtttttggcaacagacatgaatgggagagatctgctgc

agctactctcaaaggctagattcctgtatctgtttcacgacagcaataatagaagtagct

ttttgagacgtcggccagagaacagtagaaaagttctaccaaatcagtgacacttatttt

gggagaggaagtaggttttatttgtgtttcttcagttatacttgatgtctgagtgatgtg

cattcttactgaaattgctcttgtcacgtacgtagaaagttatgtttcatcagttaattg

gtgtgtgactgatgtggattcttactgaatttgctcttatgatgcag

TTGAAGAGGCGCTCGAGCACCCGTACCTAGAGCGGCTTCACGACGTCGCCGATGAGCCCA

TCTGCACGGACCCCTTCTCCTTCGACTTCGAGCAGCACCCTCTGACGGAAGACCAGATGA

AGCAGCTCATATTCAACGAAGCCCTCGAGTTGAACCCCAACTTCCGATACTAGACGATTC

ATTCCTAGTTGCCCAACCCCCAATAGTATGATTTCGGTGTAAAACTGTCTGTAAATAGGA

GTAAGATCAAAGAACATGGTAGGTTGCGCACTGCATTTGTTTGTTTGTTCCGTGTAAGTT

GTTGCTGCTTCTTCAGACCTTTGTCCCTTGGCTATGCGAATAAAAAATTTGGAGTATGAT

TTGAGAGCAAATCATTCTCTAGTCTACGAAGAAATAGATTTTATTAATCAGGTGGATGCT

CACCTGATATCAGTTTATGTCTCTATATATGAAAGACAACAGTATTTAGGATATGAACAA

GAGCCTGTTTGTCATATCCCTTAACGTCTCCTCTAAGTGCTACATAATTTTACTATTGTC

AATTAAATGAAAAATGGCTATGCATAAGCAATGAAAAATACTCCCACCCTC

**>TaMPK3(4DL) TRIAE_CS42_4DL_TGACv1_344691_AA1148960.1**

GCGATACGCACAAAAAAGAGCGAGAGACCGCGCGCACCAGCACAGCACAGCAGCGGCGTG

CGCGCTCTCGCTCGCTCGCTTTCTCGCTACCTTCCTCAGCTGGGCCGAGGGCATTAGCGG

AGCCGCCCTTTTCTATTTATAATTCTCTCCTCCTCAACCCTACAAGAATCGAGAGCCGGG

ATAAAGTCAATCATCAGCTCAGCCTCATCCCGTTGCGCGTGCGTGCGTGCGTGTTGCCTC

CCTCCTGTGATTGGGTTTTGAGAGTTTCTTGGCTGTGTTTGAGAATGGACGGCGCTCCGG

TGGCCGAGTTCCGGCCGACGATGACGCACGGCGGCCGCTTCCTCCTCTACAACATATTCG

GCAACCAGTTCGAGATCACGGCCAAGTACCAGCCGCCGATCATGCCCATCGGCCGCGGCG

CCTACGGGATCGTCTG

gtacgttccgcccgtcttaatttgctcctcaatcaccatcgatttcttgccaagattgtt

catgtcgttgcggctccgatcggagaacagaggggggattggctcgggccatggggaaag

ataggattaggccaattcactcctttctgtgaacgacactagtttagtactagcactata

actttatcagttgagcaaatatgtgaatctgtcagtgctctgtggttggtggtgtttaat

cttgtcctgaaattccacttgtggttgcgtgggcgcag

CTCGGTGATGAACTTCGAGACGAGGGAGATGGTGGCAATCAAGAAGATCGCAAACGCTTT

CGACAACAACATGGACGCCAAGCGCACGCTCCGGGAGATCAAGCTCCTGAGGCACCTCGA

CCACGAGAAC

gtaatcaacaactatcttttccgcttcagatgtgcgtatctcattgaattcggcaattca

ggcgctaagctgttccgctacatggatgcag

ATAGTAGGCCTCCGAGATGTGATCCCGCCGGCGACCCCGCAGTCCTTCAACGACGTCTAC

ATCGCCACCGAGCTCATGGACACGGACCTCCACCACATCATCCGCTCCAACCAAGAACTC

TCGGAAGAACACTGCCAG

gtactagcaaggaggggaaataagccattcattttgttcatgtttgtgacaatttgatcc

gtatgtatattcatgcgacgtggtgttcttgggtggctttcag

TACTTCCTGTACCAGCTGCTGCGCGGCCTCAAGTACATCCACTCGGCGAACGTGATCCAC

CGCGACCTCAAGCCGAGCAACCTGCTGCTGAACGCCAACTGCGACCTCAAGATCTGCGAC

TTCGGCCTGGCGCGGCCGTCGTCCGAGAGCGACATGATGACGGAGTACGTGGTCACGCGG

TGGTACCGGGCCCCGGAGCTGCTGCTCAACTCCACCGACTACTCCGCGGCCATCGACGTC

TGGTCCGTCGGCTGCATCTTCATGGAGCTCATCAACCGCGCGCCGCTCTTCCCGGGGAGG

GACCACATGCACCAGATGCGGCTCATCACGGAGGTGATCGGCACCCCCACCGACGACGAC

CTGGGCTTCATCCGGAACGAGGACGCCAGGAGGTACATGAGGCACCTGCCGCAGTTCCCT

CGCCGGTCCTTCCCGGGACAGTTCCCCAAGGTGCAGCCCGCCGCGCTGGACCTCATCGAG

AGGATGCTCACCTTCAACCCGCTGCAGAGGATCACAG

gtgcgttgcgctccacgtccgcgtcactctgctttgatcttcacgtgtgaatttcgtagc

gccgcactgcactgcacgcaatgctgctgctgttagagcgccgtgctttgttgactggct

tcgtacggccagtgttggggtcagtgtcagtgctaaaaacatctttgtgctggttataag

taggaatagatgcaatctgcagccacttcggcccctatttttaagtttttactgcatgtg

gacatgtgctccctttttagacaaaaaccatgtgtgctgcccacttgattaagaaaagat

ctttgcctatctttggagctagtagtatgtgctgtagacgacaaggactgtgcgtgtagt

gcagaatcacactttttttagaagaacgtatctgtttttggcaacagatatgaatggaag

agatctgctgcagctactctcaaaggctagattcctgcatctgtttcacaacagcaataa

tagaagtagctttttgagacgtcggccagagaatagtagggaaggctaggaagtacagta

gtttctattttgttgttatacgtacttgatgtctgagtgacgcgcattctcacttaaatt

gctcttgtcacgtagaaagttactccctctgtaaagaatagtaatctaaacactcttgta

ctcaatggcggagctatgttggggccgggggccgctgcccccccccccccccccagcctt

tgatccattcctaaagaaaaattatttaaggaggtttagattagacttttgttagctttt

ggcccccccaaacactgaggatttttgtttggccccccaatgatcttcggctagctccgc

cactgcttgtactccagttaattagtgtctgactgatgtggattcttaatgaatttcttt

atggaggaagtatgtttcatcagttaattagtgtctgactgatgtggattcttactgaaa

ttgctcttgtgatgcag

TTGAAGAGGCGCTGGAGCACCCATACCTAGAGCGGCTTCACGACGTCGCCGACGAGCCCA

TCTGCACGGACCCCTTCTCCTTCGACTTCGAACAGCACCCACTGACGGAAGACCAGATGA

AGCAGCTCATATTCAACGAAGCCCTGGAGTTGAACCCCAACTTCCGATACTAGACGATTC

ATTCCTAGTTACCCCCAATAGTATGATTTCGATGTAAACTGTTTGTAAATAGGAGTAAGG

TCAAAGAACATGGTAGATTGCGCACTGCATTTGTTTGTTTGTTCTGTGTAAGTTGTTGCT

GCTTCTTCAGACCTTTGTCCCTCGGCTGTGCGAATAAAAAGTTTGGAGCATGATTTGAGA

GCAAATCATTCTCTAGTTTATGAAGAAATGGATTTTATTAATCAGGTGGATGCTCGCCTG

ATATTGAGAATGCAGTTTATGTCTATATATGAAAGGCAGCAGTATTTAGGATTCA

**>TaMPK4(1AL) TRIAE_CS42_1AL_TGACv1_000118_AA0003960.1**

CCTCGCGTCCGCTCGCTCCCATCCGCATTCCCCAGTCGCCGCCCAAGTCAAAAAGAGGGG

GAAGCTTCTCCACCAGAGTCCCCCAAGCCCATGAACGACTAGTCGCTGCGAAGCACCAAC

CCTAGAACCCAACCCTGCTCTCCCCCTCCTCGGCTCCTCCCCCTCGCGAGCGGCGACGAA

GAGGCCATGGATACCTCCGGCGGCGGCGGCGGCGCCGCGGGCGGGGCCGCGCAGATCCAG

GGGATGGCGACGCACGGCGGCCGCTACGTGCTCTACAACGTCTACGGCAACCTCTTCGAG

GTCGCCTCCAAGTACGCCCCGCCCATCCGCCCCATCGGCCGAGGCGCCTACGGCATTGTC

TG

gtgcgttctcccctcccgaatcacggattttatgcgtgccgctgcagttaatagcgaatc

cgagctgctatctatctatgccatgtgtagaatattccgcgccgtgcggcggtgtctgcc

cagatttggcacacatgcgggtatgacgtttttaggcataaagttatgatgaggaagtga

gtcacggcgacgccccaccctaagggttgcagtaggagtgcttatatttgcctactcttg

ttccctatttgtag

CGCGGCGGTTAGTTCGGATACAGGAGAGGAGGTTGCGATCAAGAAGATTGGAAATGCGTT

TGACAACCACATCGACGCCAAGCGGACGCTGAGAGAAATAAAGCTTCTTCGCCACATGGA

CCATGAGAAT

gtaagacttgtctgttttaatttgtttggacatgtatagtgattgctcgatgtacttgcc

ataccgctataagtgtttgctgtgtgctttacatttctatacataagatagcttaccact

gtgcttgagcaacacatgttcaactgtgaacgctgctcttattagtttctttttgaatac

caaattttggcagaggcaagtggtcatgtacttctggttatagagaattactatgcataa

ggaaagaactactttaggtgtaatatgttgaatgtaaatcgcagagtatggccctaccca

ggaactgacccttattgcttgattatgtacatgatgaagttctgtggtaatacttaagct

tcttactaaagaagcatctagaagcatagggatcagctggcatcacagtcatagggtcga

gtgtgcgctgacaattttctctgaagtttgcttctactagtgtttttttttcctttgtcc

aagtttctagaagcggtgtgcctgagtggcttctgttttttgtcattacaattttactgt

taatgggctttgttgtccactcatactcacactgtcagtcattcctatgatgggtttatt

cttatcatggtttcaaaattggatcacag

ATTCTTGCCATAAAGGATTTAATACGCCCCCCAAGAAGAGATGATTTTAAGGATGTGTAC

ATTGTTACTGAGTTGATGGACACAGATCTCCACCAGATCATTCGCTCAAATCAATCATTG

ACTGATGACCATTGCCAG

gtttgttgcctcctgcttctgccaagattcttatgtatttgctgcaattgcagttgaaac

aaatcttctggtgactaatgtagatatcgattgacatgtactcacaattgtgttcttcac

cttctttagttcttagcttaggtttatcagctttgtcctccagaaatcataagttacatg

ttgtgttccgtaccttctttagttcttagtttaggtttatgagctctattttccagcaat

cataagttacatgttttgcattgcag

TACTTCTTGTATCAGTTGCTTCGAGGGCTAAAATATGTGCACTCAGCAAATGTCTTGCAC

CGTGATCTGAAGCCAAGCAATTTGTTCCTAAACGCAAATTGCGATCTCAAGATTGCAGAC

TTTGGGCTTGCAAGGACCACTTCAGAAACTGATCTCATGACAGAGTATGTGGTCACTCGT

TGGTACCGAGCACCAGAGTTGCTGTTGAACTGTTCACAATATACTGCTGCAATTGATGTC

TGGTCAGTTGGGTGCATACTTGGTGAAATTATTACTCGTCAACCCCTGTTTCCTGGAAGA

GATTACATCCAACAGTTAAAATTGATCACTGAG

gtaggtttgttcaactcgtagcttgccttttttggtatccacattgacaagtcatgttgt

tgtctatttggaagctgtcaatcaatcaagatgagtctgtttctgagcttctttaataga

atttgacaattgttatatttggaacaataggataactggatctttcttggcacatctgac

tgtattgttagacctgtttcacatcttgtgtttgaatagaagttgtttacttttacgcca

acactgaattagtaaatgtattttgcatctggtagcatgttggtgctgcaacattttact

gtgtcatcagatctggaatccatatttggattattaacttcgtgcaagtttctgtggatg

tctcttctcttcaaagtatacattttactatatatactggttggtttctgtgcaataaat

tcttagcttcaaatcgaccattcttagtaccacggaatgcatttttgcttctttttttaa

tgatgtctcttgaacctgttgtgtcaaatattaatcagtcctttccggctttctgcttca

aaag

TTGATAGGATCACCAGATGATTCGAGCCTAGGATTTCTTCGGAGTGATAATGCACGAAGA

TACATGAAGCAATTACCACAGTACCCAAGGCAGGACTTCCGCCTGCGCTTCCGCAACATG

TCTGATGGTGCAGTTGATCTGTTAGAGAGAATGCTGGTGTTTGACCCAAGCAGACGCATC

ACTG

gtacatgggcttgaactaactacttatgttagttttgtttgctttactatggctcaaaat

catttttcatgcag

TTGATGAGGCTCTGCATCACCCATACTTGGCTTCTCTTCATGACATCAACGAAGAACCCA

CTTGCCCAGCACCTTTCAGCTTTGATTTTGAGCAACCATCTTTTACAGAAGAACATATGA

AAGAGCTCATCTGGAGGGAAACTTTAGCATTTAACCCTGATCCTCCCTACTAAGAGCCAA

G

gtttgtatcgtatacgtctggttgaaaatatttcgtttcttaaaaccaattatctagaag

aacacaatgtgatcagacgactaatctagatcttggacgtgaggaggttcatacaggctt

gttattgatatttatctgcaatatttcttaggttgagtaatttagttcttgaaaattgtg

ccccgtttggaaaccaaatatctatctggatcagtaagactttagtttgagaacataccc

tgcttaaaaattctatttgtaagtcaagtcttgtttttccatgtttcttgtctgtgccga

ttctccgttgtctcagttcattttgaatttcatttcatttggttcaagattgtttccagg

gattcaagctaattcattttgttttccttgggagcacag

ACCAAATTACCAGCTGAGGGCATTGAAGATCTACCTCTAGCTCTAGTGAAGCCGATATTT

CCATGTCTTGTGCATTTATTTATTTTATGCTCGCTTATTGGGCGAATGGGCATGGATTAT

TTGTTGCTGTAACTATTCCTTTTGGGGCCTTTCTGAAGAAACGACATTTGTATGAGATGG

CTTGCGCCTGGGCTTGTGTTATACCGGTTGATTGAATAAACTCTTCTTGTGTATCTAAGG

CTTGTAATTTGTCTACACTTATTTAAACATCCCATGTATATCTGAGTTAACATATTGCCT

TTTATTATCTCAAGCATGTAATTTTGCATCTTGTGCCAGTCGTATTGAAGTTGCTGATAC

AATTTGCTCTGAAATTCCATTCACTGGTAGGTGTATCCCACAGTAAAAGTTCCTACATGC

TATTCTGCGCACTAGTACTTGGTTATTAGTATATCCTTAAGCTTAAAAGACAGCATGAAC

GCATGGTCGAGGAGTAGAAAGTTTTAGCCTCACGGGAATTTGGCAAAAGCTCTTCAAGCT

CAAGCCATTCACCGTCCACGGCGCAGTGAGCCCGCCGACATTGTTGCACATGACTGACAT

GATGACGACCATGTCAGCGACGTCAAGGAGCCGAAGTCGATGGAGTCGACGTCTGAGAAG

ACGAGCACGGCATCCTCCTGCATTTCCTCCTCGCCGTTGAGGTACTGGCAAACCTGCCTC

ATGCTAGGCCGTGCCTCCGGCCGCCACTGGCTGCAAGCGAGACCCAGCCACAGCACCAGC

CTCACCTCCTCCTTGTCATACCACCCGTCGAGCCTCTCGTCCACGGCTCGCACCAGGTCG

CCCTTGACGCCGTGGTCTCGGACCCAGCGCACCAGGTTCACGCCGGTGATGGGGTCGATG

GGCCGCCGCCCGCTCGCCACCTCGAGAAGCAGGCCGCCGAAGCCGAACACGTCGCTGGCC

GTGGTGGCCTTGCCAGTCACGGTGAGCTCCGGCGCCATGTAGCCCAGCGTCCCGACGACG

CGAGTCGTGGCAGGTTCCGCGCCGTGCTCGTAGAGGCGCGCGAGGCCGAAGTCGCCGAGC

CGCGCGCTCATGTCCGCGCCCAGCAGCACGTTGCTGGCCTTGACGTCGCGGTGCACCACC

ACCTGCTCCCACCCCTCGTGCAGGTACACCAGCCCGGACGCGACGCCCCTGAGGATCCTC

AAACGCTGCTCCCACGTGAGCAACGCCGGCGACGGGGACACGCCGACGCCGGCGCCGGCG

CCGGCACTGCCGAACAGGTGCGCTTCGAGGCTGCCGTTGGGCATGAAGTCGTAGACCAGA

AGCAGGTCTTGCCCGTGCTTGCACCACCCGCGCAGCTCGACCAGGTTCCGGTGCCGCATG

CGCCCGAGGCTCGCGACCTCGGCGACGAACTCGCGCATCCCCTGCGACCCGTTGCTTGAG

ATCCTCCTGATGGCCACCGTGTTGCCAGAGCGCCGGAGCACGCCTCTGTACACCTGGCCG

AAGCCACCGGCGCCCAGGAGCTCACTGTTCTTGAACCCCTTCGTCGCCTTGTACAGCTCC

TTGTATGGCAACCTGTGCGGGTGATCCAGCTCCCACTCCTCAAGCGTCTCGGCCAGCGCC

GCCCTTCTCCGCAGCCAAAACGCGGCACCGATGGCGGCGACGACCATGACCAGCGTGGCG

GCGCAAGACAGAGCGACGATCTTGATGATGGTGGACGTGGAAGGTGGAGGAGCCGGCGCC

TTTGGGACTTTCGGCAGCCGGGAGAGATCGATGGCTTGCGCGACTCCGTCGGTACGGAAG

CTCCAGGCAAGAACGTAATGCGAGCTCGCCAACTTCCCTGTCGCCGCCGAGAAACCGACG

TACATGTCTTCCTTAAAGATCGGCGAGAGGTCAATGACCTGCGATATAAGCGGCCGGCGA

GGCCGCGTTTGCAAGGACGCGGGAGCGATGGTCACGTTGAGGACCTTGGCGCTGCCGTCG

TAGTCCACCCACGCCTGGATCGGCTGCGCGCTCCCGAGCGGCACGGAGACGCTCCTGCCG

TCGTCATCGGTGAAGTAGGCGGCCGGCTCCGACACGTTGGACACGAGGCTGTTCAAGTCG

ACGCCGACGTGGTTGGCGTTCGTCTCGTTCAGGAGCCCGTTCGCCTGCACCGTGTCCAAC

TCGACGGCGAAAACGTGGTTCGAGGAGTTGCCCAGATTTCCTTTGCCGAGGAGGCCAAGG

TACTGCTCGTCACTTGCCCCGGGGAGCACCTTGGAAGCCGCGACCACGAAAGCGAGTCCA

TGGCCGCCGCTTCTGCCGACAGTAACGATGTCGAAGACGAAGGCCGTGCTGAAGGAGATG

ACCGCCTTGTTCACGAGCATTGGCACTGGCGCCGCGAAGAAGGCGTGGCCCATGAGGTTG

TTGCTGTCGTTGGTGAGCTGAAGCGCGCCGCCGCGCAAGATGGACGCCGAGCCGTCTAGG

CTCAGGTCGGCGGCGTGCTGGAAGCCGTTGTAGATGAAGCTAAGTGCGGCCGAAGAAGGG

ACGAGAGCAACGAGGGTGAGCACGGTGAGGTGGCGAAGGAGAGCGATGGACGACTCCAGA

GACATGGTCGAAACAATGTGCTGTGTATGCACGTAGTTCTGGACAGAGCACAGTTGTTGT

TGTTGCGCAAAGTTGCCTATGGACAGGTATCAACTATCAATCATCCATGGAGTGAAGAGA

ATCTCAGATGGGCGAGATGAATGACCAATGAATGGAGCTTCTGTAACCAATGAATGGTAG

TACTAGTAGTACTGCACTACAGGTGTAGCTCTACAGTTAATACTCCACAACTGTGGATAA

GACGGAAT

**>TaMPK4(1BL) TRIAE_CS42_1BL_TGACv1_030274_AA0084790.2**

CGGTCTCCGTCCTCGCCTCCGCTCGCTCCCATCCTCATTCCCCAGTCGCCGCCCAAGTCA

AAAAGAGGGGGAAGCTTCTCCACCAGAGTCCCCAAGCCCATGAACGACTAGTCGCTGCGA

AGCACCAACCCTAGAACCCAACCCTACTCTCTCCCCTCCTCGGCTCCTCCCCTCGCGAGC

GGCGACGAAGAGGCCATGGATACCTCCGGCGGCGGCACCGCCGCGGGCGGGGCCGCGCAG

ATCCAGGGGATGGCGACACACGGCGGCCGCTACGTGCTCTACAACGTCTACGGCAACCTC

TTCGAGGTCGCCTCCAAGTACGCCCCTCCCATCCGCCCCATCGGCCGAGGCGCCTACGGC

ATTGTCTG

gtgcgttcccccctcccgaatcgcggattatatgcgtgctgctgcagttaatagcgaatc

cgagctgctatctacctatgccatgtgtagaatattccgtgccgtgcggcggtgtctgcc

cagatttggcacacatgcgtgtgtgacgttttaggcataaagttatgatgaggaagtgag

tcacggcgacgccccacttgcagtaggagtgcttatatttgcctactcttgttccctatt

tgtag

CGCGGCGGTTAGTTCGGATACAGGAGAGGAGGTTGCGATCAAGAAGATTGGAAATGCGTT

TGACAACCACATCGATGCCAAGCGGACGCTGAGAGAAATAAAGCTTCTTCGCCACATGGA

CCATGAGAAT

gtaagagttgtttgtttttagtttgttgacatgtatagtgattgctgtgtgtacttgctg

taccactataagtgcttgctgtgggttttacatttctatacataagatagcctaccactg

tgcttgagcaacacatgttggattgtgaacgctgctcttattagttccttttcaacacca

aatttaaaatctagcggtgatttaatatgtactccctctgtaaactaatataagagcatt

tagatcactaacttagtaatctaaacgctcttatattagtttacggagggagtacttaga

atgtaaatcttagagtacggccctacccaggaactgacccttattgattgattatgtaca

tgatgaagttttgtggtaatacttaagcttcttactgaagaagcatctagaagcataggg

atcagctggcatcacagtcatagggtcgagtgtgcactgacaactttctctgaagttggc

ttctactagtggttttatttccgttgtctaagtttctagaagtggtgtgcctgagtgggt

tctgtttcttgtcattacaattttactgttgatgggctttgttgtccactcaatactcac

actgtcagtcattcctatgatgggtttattctgatcatggtttcaaaattggatcacag

ATTCTTGCCATAAAGGATTTAATACGCCCCCCAAGAAGAGATGATTTTAAGGATGTGTAC

ATTGTTACTGAGTTGATGGACACCGACCTCCATCAGATCATTCGCTCAAATCAATCATTG

ACTGATGACCATTGCCAG

gtttgttgcctcctgcttgtgccaaggttcttatgtatttgctggaattgtagttgaaac

aagtcttgttgtgactaatgtagatatcgattgacatgtactcacagttttgttctgtgc

tttctttagttcttagcttaggtttatcagctttgtcctccagaaatcataagtcacatg

ttgtgttctgtgccttctttagttcttagcttaggtttatgagctttatcttccagcaat

cataagttacatgttttccattgcag

TACTTCTTGTATCAGTTGCTTCGAGGGCTAAAATATGTGCACTCAGCAAATGTCTTGCAC

CGTGATCTGAAGCCAAGCAATTTGTTCCTAAACGCAAATTGCGATCTCAAGATTGCAGAC

TTTGGGCTTGCAAGGACCACTTCAGAAACCGATCTCATGACAGAGTATGTGGTCACTCGT

TGGTACCGAGCACCAGAGTTGCTGTTGAACTGTTCACAATATACTGCTGCTATTGATGTC

TGGTCAGTTGGGTGTATACTTGGTGAAATTATTACTCGTCAACCCCTATTTCCTGGAAGA

GATTACATCCAACAGTTAAAATTGATCACTGAG

gtaggtttgttcaactcgtcgcgtgatttttttggtatccactttgacaagtcatgttgt

tgtctatttggaagctgtcaatcaatgaagatgcgtctgtttctgagcttctttagtaga

atttgacaattgttctatttgaaacaatagcatctgactgtattattagacctgtttcgc

atcttgtgtttgaatagaagttggttacttttacgcaaacactgaattagtaaatgtatt

ttgcatctggtagcatgtaggtgcggcaacattttactgtatcatcagatctggaatcca

tatttggattgttaacttcgtgcaagtttctgtggatgtctcctctcttcaaagtataca

ttttactatatatactggttgttttctgtgcaatacattctgagcttcaaatagaccatt

tttagtacccactgaatgcatttttgcttccttttttaatgatgtctcttgaacctgttg

tatcaaatattaatcagtcctttccgcctttctgcttcaaaag

CTGATAGGATCACCAGATGATTCGAGCCTGGGATTTCTTCGGAGTGACAATGCACGAAGA

TACATGAAGCAATTACCACAGTACCCAAGGCAGGACTTCCGCCTGCGCTTCCGCAACATG

TCTGATGGTGCAGTTGATCTGTTAGAAAGAATGCTGGTGTTTGACCCAAGCAGACGCATC

ACTG

gtacatgggcttgaactaactacttatgttagttttgttcgctttactatggctcaaaat

catttttcatgcag

TTGACGAGGCTCTGCACCACCCATACTTGGCTTCTCTTCATGACATCAATGAAGAACCCA

CTTGCCCGGCACCTTTCAGCTTTGATTTTGAGCAACCATCTTTTACAGAAGAACATATGA

AAGAGCTCATCTGGAGGGAAACTTTAGCATTTAACCCTGATCCTCCCTACTAAGAGCCAA

G

gtttgtatcatatacgtctggttgaaaatatttcgtttcttaaaaccaattatctaaaag

aatgcaatgtgtcagacgactaatctagatcctggaatcctgagaatgttcatacagact

tgttattgatatttatctgcaatatttctgaggttgagtaatttagatcttgaaaattgc

acccttttggaaccaaatatctacctggaccagtaagactttaatttgagaacataccct

gcttaatattctatttgtaagtcaagtcttgtttttccacgtttcttgtgtgtgtgccag

ttctctgttgtctcagttcattttgaatttcatttcatttggttcaagattgtttccagg

gattcaagttaattcattttgttttccttgggagcacag

ACCAAATTACCAGCTGAGGGCATTGAAGATCTACCTCTAGCTCTAGTGAAGCCGATATTT

CCATGTCTTGTGCATCTATTTATTTTATGCTCGCTTATTGGGCGAATGGGCATGGATTAT

TTGTTGCTGTAACTATTTCTTTTGGGGCCTTTCTGAAGAAACGACATTTGTATGAGATGG

CTTGCGCCTGGGCTTGTATTATACCGGTTGATTGAATAAATTCTTCTTGTGTATCTAAGG

CTTGTAATTTTGTGTACACTTATTTAAACATCCCATGTATATCTGAGTTGACATATTGCC

TTTTATTATCTCAAGCATGTAATTTTGCATCTTGTGCCAGTCGTATTGAAGTTGTTGATA

CAAATTGCTCTGAAATTCCATTCACTGGTAGGTGTATCCCATAGTAAAAGTTCGTACATG

CT

**>TaMPK4(1DL) TRIAE_CS42_1DL_TGACv1_061983_AA0206840.1**

ACAGCAATAAATTAAGCGAAAGGGATATTTACCCCTATCTACCTTTTTTTCCTTCTGTTT

TCCCCTTAACGTCGCCCCACGCGCGCGGTCTCCGTCCTCGCGTCCGCTTGCTCCCATCCT

CATTCCCCAGTCGCCGCCCAAGTCAAAAAGAGGGGGAAGCTTCTCCACCAGAGTCCCCAA

GCCCATGAACGACTAGTCGCTGCGAAGCACCAACCCTAGAACCCAACCCTGCTCTCCCCC

CCTCCTCGGCTCCTCCCCCTCGCGAGCGGCGACGAAGAGGCCATGGATACCTCCGGCGGC

GGCGCCGCCGCGGGCGGGGCCGCGCAGATCCAGGGGATGGCGACACACGGCGGCCGCTAC

GTGCTCTACAACGTCTACGGCAACCTCTTCGAGGTCGCCTCCAAGTACGCCCCTCCCATC

CGCCCCATCGGCCGAGGCGCCTACGGCATTGTCTG

gtgcgttccccccctcccgaatcgcggattatatgcgtgccgctgcagttaatagcgaat

ccgagctgctatctatctatgccatgtgtagaatattccgtgccgtgcggcggtgtctgc

ccagatttggcacacatgcgggtgtgacgttttaggcataaagttatgatgaggaagtga

gtcacggcgacgccccaccctaagggttgcagtaggagtgcttatatttgcctactcttg

ttccctatttgtag

CGCGGCGGTTAGTTCGGATACAGGAGAGGAGGTTGCGATCAAGAAGATTGGAAATGCGTT

TGACAACCACATCGACGCCAAGCGGACGCTGAGAGAAATAAAGCTTCTTCGCCACATGGA

CCATGAGAAT

gtaagacttgtctgttttaatttgtttgggcatgtatagtgattgctcggtgtacttgcc

gtaccgctacaagtgtttgctgtgtgttttacatttctatacataagatagcttaccact

gtgcttgagcaacacatgttcaattgtgaacgccgctcttattagtttcctttttgaata

ccaaattttggcagaggcaagcggtcatgtacttctggttatagagaattactatgcata

aggaaagaactactttaggtgtaatatgtagagtgtaaatctcagagtacggccctaccc

aggaaaggacacttattggttgattatgtacatgatgaggttttgtggtaatacttaagc

ttcttactaaagaagcatctagaagcatagggatcagctgacatcacagccacagggtcg

agcatgcgctgacaattttctctgaagttggcttctactagtggttttatttcctttgtc

taagtttctagaagtggtgtgcctgagtgggttctgtttcttgtcattacaattttactg

ctgatgggctttgttgtccactcatactcatactgtcagtcattcctatgatgggtttat

tctgatcatggtttcaacattggatcacag

ATTCTTGCCATAAAGGATTTAATACGCCCCCCAAGAAGAGATGATTTTAAGGATGTGTAC

ATTGTTACTGAGTTGATGGACACGGATCTCCACCAGATCATTCGCTCAAATCAATCATTG

ACTGACGACCATTGCCAG

gtttgttgccgcctgcttgtgccaagattcttatgtgtttgctggatttgtagttgaaac

aaatcttgttgtgactaatgtagatatcgattgacatgtactcacagatgttttctgtgc

cttctttagttcttagcttaggtttatcagctttgtcctccagaaatcataagttacatg

ttgtgttctgtaccttctttaattcttagtttaggtttatgagctctgttttccagcaat

cataagttacatgttttgcattgcag

TACTTCTTGTATCAGTTGCTTCGAGGGCTAAAATATGTGCACTCAGCAAATGTCTTGCAC

CGTGATCTGAAGCCAAGCAATTTGTTCCTAAACGCAAATTGCGACCTCAAGATTGCAGAC

TTTGGGCTTGCAAGGACCACTTCAGAAACTGATCTCATGACAGAGTATGTGGTCACTCGT

TGGTACCGAGCACCAGAGTTGCTGTTGAACTGTTCACAATATACTGCTGCAATTGATGTC

TGGTCAGTTGGGTGCATACTTGGTGAAATTATTACTCGTCAACCCCTGTTTCCTGGAAGA

GATTACATCCAACAATTAAAATTGATCACTGAG

gtaggtttgttcaactcgtcgcttgccttttttggtatccacattgacaagtcatgttgt

tgtctatttggaagctgtcaatcaattaagatgagtctgtttctgagcttctttaataga

atttgacaattgttatatttggaacaataggataactggatctttcttggcacatctgac

tgtattgttagacctgtttcgcatcttgtgtttgaatagaagttgtttacttttacgccg

acactgaattagtaaatgtattttgcatctggtagcatgttggtgctgcaacattttact

gtatcatcagatctggaatcctggattattaacttcgtgcaagtttctgtggatgtctct

tctcttcaaattatacattttactatgtatactggttggtttctgtgcaatacattctta

gcttcaaatcaaccatttttagtaccaccgaatgcatttttgcttctttttttaatgatg

tctcttgaacctgttgtatcaaatattaatcagtcctttccggctttctgcttcaaaag

CTGATAGGATCACCAGATGATTCGAGCCTAGGATTTCTTCGGAGTGATAATGCACGAAGA

TACATGAAGCAATTACCACAGTACCCAAGGCAGGACTTCCGCCTGCGCTTCCGCAACATG

TCTGATGGTGCAGTTGATCTATTAGAGAGAATGCTGGTGTTTGACCCAAGCAGACGCATC

ACTG

gtacatgagcttgaaccaaccacttacgttagttttgtttgctttactatggctcaaaat

catttttcgtgcag

TTGATGAGGCTCTGCATCACCCATACTTGGCTTCTCTTCATGACATCAACGAAGAACCCA

CTTGCCCGGCACCTTTCAGCTTTGATTTTGAGCAACCATCTTTTACAGAAGAACATATGA

AAGAGCTCATCTGGAGGGAAACTTTAGCATTTAACCCTGATCCTCCCTACTAAGAGCCAA

GGTTTGTATCGAATACGTCTGGTTGAAAATATTTCGTTTCTTAAAACCAATTATCTAAAA

GAATACAATGTGTCAGACGACTAATCTTGATCCTGGAATCCTGAGGAGGTTCATACAGAC

TTGTTATTGTTATTTATCTGCAATATTTCTGAGGTTGAGTAATTTAGATCTTGAAAATTG

TGCCCGTTTGGAAACCCATTATCTATCTGGATCAGTAAGACTTTAGTTTGAGAACATGCC

CTGCTTAAAATTCTATTTGTAAGTCAAGCCTTGTTTTTCCACGTTTCTTGTGTGTGTGCC

AATTCTCTGTTGTCTCAGTCCATTTTGAATTTCATTTCATTTGGTTAAGACTTTTTCCAG

GGATTCAAGCTAATTCATTTTGTTTTCCTTGGGAGCACAGACCAAATTACCAGCTGAGGG

CATTGAAGATCTACCTCTAGCTCTAGTGAAGCCGATATTTCCATGTCTTGTGCATCTATT

TATTTTATGCTCGCTTATTGGGCGAATGGGCATGGATTATTTGTTGCTGTAACTATTTCT

TTTGGGGCCTTTCTGAAGAAACGACATTTGTATGAGATGGCTTGTGCCTGGGCTTGTATA

CCGGTTGATTGAATAAACTGTTCTTGTGTATCTAAAAACTTGTGATTTTGTGTACACTTA

TTTAAACATCCCATGTATATCTGAGTTAACAAATTTGCCTTTTATTATCTCAAGCATGTA

ATTTTGC

**>TaMPK6(7AS) TRIAE_CS42_7AS_TGACv1_570369_AA1834630.1**

GCCGACAACACCTGACAATGCCTACACGGCCACGCACGGACACAAGGTGGCAGCACACCA

GCCAGCGCCGGCACCGTCGAAAAGGCGCCCTCGACAGCACCACACCCATAATCACACCGG

TGGCGATGCGCAGCCCCCAACCGCCGCCGCCACCACAACCACATCCATCTCGTGGATTTA

TACATATCATAACGCACTTAGGGATGGGCCCTAGCTTTCTTTTCTAGGGTTAGGGATGCA

CAGTGTGTATGTAGCTTATGGCCATAGGTAGGCATGATCCTCCATTTTCCCACTTGGAGT

AAAAAAGGCACCTTCCCATAGCAGCCGGCCACTCCCACTCGCCACTGGCACGCGCGCTGC

CAGCCATCTCCCGTCTCCACGCAGGCCGGCACGGCTGCCCCCATCTCATCCCATCCCCTC

CCCAGCCCAGAAACTGAAGGAGAGAGAGGAGAAGGGGAGCTACCACACCACCTAAACAAA

CAAGAGGCGGAAAGCGACCAAATCTCGGGAGCGAATTCCCCTCCCCTCCCCGCACCCCCG

CGCGCGCGATCCGGGCGGAGATGGACGCCGGCGGGGCGCAGCCGCCGGACTCGGAGATGG

CGGAGGCCGGGGCGGCCGCGGCCGCCGCGGCGGCCGCGGCGGGGGCGGCGCCGGGCGGCG

CGATGGACAACATCCAGGCCACGCTCACCCACGGCGGGAGGTTCATCCAGTACAACATCT

TCGGCAACGTCTTCGAGGTCACCGCCAAGTACAAGCCCCCCATCCTCCCCATCGGCAAGG

GCGCCTACGGCATCGTCTG

gtacgccccctccccctttctttcccctccatccccatctccatctccggctggctgacc

gactcactgacccccggcgcgtggcag

CTCCGCGCTCAACTCCGAGACGGGGGAGCAGGTGGCCATCAAGAAGATCGCCAACGCCTT

CGACAACAAGATCGACGCCAAGCGCACGCTGCGGGAGATCAAGCTGCTCCGCCACATGGA

CCACGAGAAT

gtcagtccccgatccgcccctccctccatttttttcctctctcggagaatctccttgaat

gctccttgtggttgtgctggttggttgcattgaatggaacggcaggtggatgggttcggt

tagattttagccgccgccaaatgattggcttcgccgacagctccgtggccggcttttcct

tcctcgtttgcgcgagaccttcaggactagtaatcctaggcgcgcctttaaagaacccaa

caacctcttgggatgcgattcattgtgttcttcttcctcggcacacctggagcagcacca

cttgatgatgctctcgctcctactgctagattctgctgatgtcaaaagctgcctcaggac

caatctatctgcctgtgtcccttttgctgcctagctcctccgaatccagtgggtgtgtgg

tggaagtgattgcgcagttcctaggctagggggctactactgtttgaaagttcttattag

gatgttggacttggatctttccatttcatagtcctgtgataactaaactgggtaagagaa

atctgagttaggcatcttgggtcttttcatgtgccttctttaaaaggctttggcgttctt

cttgtaatatgaaatgactcgtgttttggcgcgtatttgagggggaaaaatgtttttttg

gggaagatagccgtgctcgcagtacactagaggtggattgtatcttgacagccttccttg

gacacctcagcatatgctctgagatgtgtggatttcaagtttaggtgatgtcaagtaatc

tgctagcagtacgagcatgaagagaatggcaccctttggctttgtattctttgtttcatc

aagcatcgtcatttgcagattgcgtattctggtggaaggttttcagagtgacactggttt

tggctagtatcctacgtaagctagggtttcagatttaatgctacacctgaatagacaaac

ttctttgggagtttgaccgtcagacaattatgaccgttggcactgaaatatcggattagg

caaggtctgattaataacggaaatgcttgttgacacgtgctttgtacactgtcagccgta

aacgtatcacctgctgtatcttctagctactataaaggcagaatttttccgttacgagat

cctgtactttgctttttttttttcttttgatggtgcaaacgagcattcctgacaagaggt

gtattaggcggtggttggtggctggcacaaaagctgaaatgggcagcgtggcggcaccag

tgggcggctcaatagctaactcttgtgaacggacagagcaacggctgcattacctcatct

tgggttcgcaggaagcaaattggacttttcagtgggcttaaggcctggtaatggaaccac

agcacaggagaaaacatcctgaccataaaggaaaataatgcacaggaccacttacgccac

atatttgcctttatagtagttatggcaggtgatacggttccctgtcagctatttatgtcc

aatacattccataccataactgtatgatgctgtcttattcattattattctccatgtcaa

cccccaaaaaggatgagctacataataaagccataaagatttggagaccctgtattccaa

tagctatattagaagccatagtttatgtaagggaaaagcctctttgtttgctgacacgaa

tttagttccccctccaggtcccaaaagcacagagaattacatttgcaaattttcggaaaa

agaaacagtcttgtagatctacctgtagtcctgtacaaaagtatgtgtacaaaaatgcat

ctatgcactagtttttgttagtacacatattatcgcacaatgcacatcaaaatttcccct

ccattctggaaaattgcaaatcagttctttttttcacaagggaacatgtgcagctagaaa

ttgcatgttgtttacctcctaaaccaagtaacattcggcttaatgcagctacttgggtga

tgcacttgtttcatctatgctagctacctacttgtatcttgtatcttgtttgttgtattt

tatacatacataccctgactcttggttgtactcggcatatccatgctcgaagcagcaatg

actggtcatatccactctctaagcagtaagaagtggtcagaacttcctatactgctcatg

tgtcatgtgggaacatatgcagctggtatagtgctgcattgtgttcaagttagttacaag

ggttttgaagatggttcacaatgcaacataaaaacagaggctaacaacaaataaaaattg

gaaagctccagaattgctctattctggctcccatgccaagtatttatgccttctactgct

ttaaattttgaatgcgtaaaatttgtggtatttgcactgctacaaaaggcagccagaaat

gtgcaaggttacacccttcactcttcaaacaagcaaaataaataaataaattaggttgtc

atcatcggcaccggcatctttgaattatatttcatatgatgctgtgagaagtactactag

ctgcagattcttaataatgactattcatttcactcattttctggacaccataattctgtt

atctttttggtgaaaaataatagaatattgctatgattgtgttaataatatgatgccaat

ttacttctgtggtgctgttgttgtagaataatatatcttcaaaaaacatgtttaacctaa

aaaagaagccaacaactcatcaaaatgtcactgggtgcatctcagctctaacaaatgttg

ggtttgtagtatccagaaacgagtttgaaatggaaatgttaacttggtagaattttagaa

gtttccacgcaacttagttcagctgtaactgtagccaacatagttaagtgaagtgtatgt

gttcatcagctttccacggcttgttgattatttcaatgcattcaagatgaaatataaagt

tgactcaagccatcgaaagaacgtacagattgtcagctgtaaccatagaaaacataagtt

atatgaagtgtatgtgttcttaagctttccatgacctgttgactatttcaattcattcaa

gatgcaagatatgtcaaatataaagttgatatgcattcaagatgaaatataaagttgact

caagccatcgaaagaaagtacagatatgtcaaattgtattgtcttggtgtatgcgttttt

tgtgcaagatgtaaaaactagacattatgtaaggcatgtggccctgttgctgctaaccat

agttattgcctttttggaagattttttttaagtatgaattaatgctacgaaggggagcct

tggtgcagtaataaagctgctgccttgtgaccatgaggtcacgggttcaagtcctggaaa

cagcctcttacagaaatgtagggaaaggctgcgcaccatagacccaaagtggtcggaccc

tgcgcaagcgggagttacatgcaccgggctgcccttttttttatgaattaatgctacaaa

atatgtttcttacaagaggacatttcttatgcag

ATTGTTGCAATAAGGGATATTATACCTCCTGCACAAAGGACTGCATTTAATGATGTCTAT

ATTGCATATGAATTGATGGACACCGACCTGCATCAAATTATTCGCTCAAATCAAGCTTTA

TCGGAGGAGCACTGCCAG

gtacttattatttatttcgatatagaagtaaaaccttctcataccccctttgtaggataa

gtttgcttatctaattatattattgtttgtctatagaaattcttttctctagtatctcat

ttcatctgattccccgagtacattatactgtcaattatctgtttcattattattttctgc

ctcagctcgtacatacttctgtaagatattagtattctgggagcagggagctaatgagta

ataactaaagcaagtcaattctttttctgctcagcctgaatcatcttgctactttgctag

tctaggactacgtgccctaaataagtttgcctggtgtatagtataaaaattgacctttgt

ggtggaattatagggatatatttgatgactgtagatatattggattagcactctgacata

ggtgaactgtaatatactgacattgaataagtaccttactgtattacacctcttagaaag

gatacgacatattagaaacattcaatgcacccctctgaacattcggtcgaacaaagggag

cagttgaacaagttcatcacatatagtactctctccgttccaaaataagtgactcaaatt

tctactaactttgtactaaagctagtacaatgttgagtcacttattttgggatggaggga

gtacatgcttagcgctgacacatcagtatactacttaatgctagcaattcttttttctat

cctttgtcaaacacagccatgcatcattgctaagggatgtttcctaaatagaagtgcttt

ctatatatcagagtggtctcttttgctaacaataccatgcttgctgatataaaatcatgt

tctactttcgcaaataccatcggataatccttggtggaaaagttccatggaaaaactgtt

tcctataaataatatgcctgtactgtcagtgcatacttgggggtttctagcataaagaat

tgacgctaaatcacacgtgagaaatcagtggttttctggagtggtcacgtttctcttccg

ttgatcttgttctttctagtttcttttcaatccccaaacttctgtcttgttctcagaaat

tgtacttaatctgatgtggatgttttgtgtaatttttccttgtgcag

TATTTCCTTTATCAGATCCTTCGTGGCTTGAAGTATATACATTCAGCAAATGTTCTCCAC

CGAGACTTGAAGCCTAGCAATCTTCTTTTGAATGCAAACTGTGACCTAAAAATTTGCGAT

TTTGGGCTTGCTCGTACCACCTCAGAAACTGATTTTATGACCGAGTATGTTGTGACAAGA

TGGTACAGGGCACCAGAGCTTTTGTTGAACTCCTCCGAATACACTGCAGCAATTGATGTG

TGGTCTGTGGGCTGTATATTTATGGAACTGATGGACCGGAAACCTTTGTTTCCAGGAAGA

GACCATGTCCATCAGCTACGTCTACTAATGGAG

gtttagatagtcccctatgaacttcttttatatcgaatttaaatcatgttgtgtggtcct

gttttagcatatctagtatgatcatagtttttttctcgaagtagaggtatttaagcactt

gactggtatatataataagtacttttctggtgcacattaattcattttcaattcccttgt

ggtatttccttgttgcag

CTCATTGGAACACCAAATGAGGCTGATTTGGATTTTGTAAATGAAAATGCAAGAAGATAT

ATCCGCCAACTTCCCCGTCATGCAAGGCAATCATTATCTGAGAAGTTTCCACATGTTCAC

CCTTCAGCAATTGACTTGGTTGAAAAGATGCTGACTTTCGATCCTAGACAGAGAATAACA

G

gcaagttctggcatgtgttctagtgatttaaccatccgcgtccatctgctgggatttatt

gttgcttgactgacgatcttatgttccaaacagcaagataaaaagagccgtctttttttt

ttcggtatcatgaatagtttaatttagaacttgctccatttgttaaatattagtacctgg

aatctcatctgggtatgccatcctagtgtttcgttttatatatcattagctaacatcatt

tcctccattcaaatggaaaaaagtaattaaattgggaagatcacacagttaaatatatgg

actatccgggctggatttacagcccaagtgggtaggaatgtgtccatactctctgtaggt

atgcaaagtgcattagtttgaagtgggaatgctaaagtaatcattgttgcctgttgggtg

gtgttgctatagttgacaatccatagttttgactgcaaaatatccgtctccaagattcat

cctttactttctttggtcagttattgatcgatagttactgattgttcctttattttttgg

tcag

TTGAAGGCGCACTTGCGCATCCTTACTTGGCATCGCTGCATGACATAAGTGATGAGCCAG

TCTGCACGATGCCCTTCAGCTTTGACTTCGAGCAGCATGCATTGTCAGAAGAACAAATGA

AAGATCTAATCCACCAAGAGGGCATCGCGTTCAACCCTGATTACCAGTAGGTGGAGTTCC

TTTGATCGGCTTCATTCATATGGAAAGTTTTCGGTCCTCCTGCCGCCATAAAATGTCGCT

AGCTGTAAATAATTGCCTCACCCAGAGAATCAAAAGGAGATGGCGTGTTAAGGGTAGATG

ACAAGAGCTGTGGTGGTCAAATTTCCGTGTAGCCTATGGATTCTTGTGCTTGTGTATGTT

GTTTTATGTGGATTTTTTCCCCTTCTGCTTGAAGATGTTTCAGCATTTTCCGTAAGATGC

GATAGTCCGTGAACGATGGCTGCCTAATTTCTGTGGCCGTCATGAGATTTTTACATTGTG

GTGGATTATGGATTATGTAATGTTGTCCTGGTTAATGAACTCCGACTGACTGATTGGAAT

ATTGTGTTTGAATGCGGACTGACCGTGATTTAGTGGTTGCTGTTTGAAGTTGTAGCCCTA

TTTAAGAGTTGTCACCCTATTCTCTGGGTTTACAAGGCTCTTGTGCATTTCTAGATCACC

AGTTTGATTGAAATATGTATTACGTCACAAAAAATAAGTATCTGGGATGTGC

**>TaMPK6(7BS) TRIAE_CS42_7BS_TGACv1_593766_AA1954300.1**

TTCTTTTCTCCGGAGAAGGTTGATGCCCCATCCCATTTCCCATCCCACTTGGAGTAAAAA

AGCCGTCTTCCCATAGTAGCCGGCCACTCCCACTCGCAACTCGCACGCGCGCTGCCAGCC

ATCTCCTCCCTCCCCCACCCGTAGGCCGGCACGGCAGCCCCATCCCATCCCGAGCCCAGA

AACTGAAGGAGAGAGAGAGGGGAGCTACCACACCACCTAAACAAACAAGAGGCGGAAAGC

GACCAAATCTCGCGAGCGAAATCCACCGCACGGGCTCTCCCCTCCCCTCCCCGCACCCCC

CACGCGCGCGATCCGGGCGGAGATGGACGCCGGCGGGGCGCAGCCGCCGGACGCGGAGAT

GGCGGAGGCCGGCGCCGCGGCGGCGGCCGCGGCGGGGTCGGCGCCGGGCGGCGCGATGGA

CAACATCCAGGCCACGCTCACCCACGGCGGGAGGTTCATCCAGTACAACATCTTCGGCAA

CATCTTCGAGGTCACGGCCAAGTACAAGCCCCCCATCCTCCCCATCGGCAAGGGCGCCTA

CGGCATCGTCTG

gtacgcccccttcccctttctttcccctccatccccatctccggccggctgaccgactca

ctgacccccggcgcgtggcag

CTCCGCGCTCAACTCCGAGACCGGGGAGCAGGTGGCCATCAAGAAGATCGCCAACGCCTT

CGACAACAAGATCGACGCCAAGCGCACGCTGCGGGAGATCAAGCTGCTCCGCCACATGGA

CCACGAGAAT

gtaagtccccgatccgcccctccctccattttttttctctctcggagaatctccttgaat

gctccttgtgctggttggttgcattgaatggaacggcaggtggatgggttcggttaggtt

ttagccgctgaatgattggattcgccgacagacagctccgtggccggctgcctactccgg

ggccggcttttccttcctcatttgcgcgagagcttcaggaatagcaatcgtaggcgcgcc

tttaaagaacccaccaaccttttgggatgcgattcattgtgttcttcctcggcacacctg

gagcagcagcacttggtgatgccctcactcctactgctagattctgctgatgtcaaaagc

tgcctcaggaccaatctatctgcctgtgtcccttttgctgcctagctcctccgaatccag

tgggtgtgtgtgtggtggaagtgaatgcgcagttcctaggctaggggggctactactgtt

tgaaagttcttattaggatgttggacttggatctttctaatgtcctgtgataactaaact

aagtaagagaaggaacctacatctgagttagccatcttgggtcttttcattcatgtgcct

tctgtaaaaagctttggtgttcttcttgtaatataaatgactcatgttttggcgcgtatt

tgagaagaaaaaaagtattttgaaatgaccgctgtgctaggagtagactagagatggata

gtatcttgacagcccgccttgggcaccacagcatatgctctgagatctgcggagttgaag

tttagatgacgataagtactctgctagcaataagtgcatgaagagatttctcatgaggta

ttgaggtctatgcacaaatgggaccccttccgctttctattctttgtttcatcaagcatt

gttgtttgcagattgtgtattctcgtggaaggttttcagaatgaaactggttttgggcta

gtatcctacgtaagctaagggtttcacattttaatgctccactgaaatagtcaaacttct

ttgggagtttgaccgtcgaacaattttgactgttagtactgaaacgtcggatttggcaag

gtctgattagtaacagaaatgcttgtcgacacgtgctttatacactgtcagctgtaaacg

tatcacctgctgtatctaccagccactataaaggcataatttttctgttatgagatcctg

tactttgtttttttcttttggtggtgcgaacgagcattcctgacaagaggtatctgagac

agtggttggtggctggcagaaaagctgaaatgggcagcgtggcagcaccagtgcgcggct

caatagctaacttttatgaacggacagagcaacagccacattaccttgccaggaagcaaa

ttggactttccagtgggcttaaggcctggtgatagaaccatagcacaggagaaaacatcc

tgaccatagaggaaaacaatgcacaggaccactaatggcacacatttgcctttatagtag

ctatatggcaggtgatgcggctccctgtcacctatttatgtccaaaactttccataactg

tgatgctgtcttattcattcttattctccatgtgaaccccccaaaagggtgagacataat

aataaagccataaagatttggagatcctgtattcctgtagctatattagaacccttagat

tatgcaagggaaaagcctctttttttgctgacacgaattttgttctccctccaggtccca

aaggcacagaaaattacatttgcatttcttcaaaaaagggaacaattttgtagatatacc

tttagtcctgtacaaaagtatgtgtacaaaaatgcatacctttgcacaagtttttgcttg

tacacataatattgcacaatgtacatcaaaatttcccctctcttttggaaaattgcaaat

gcattttttcaccagggaacatatgcagctagaaattgcctgttgtttacttccgaagcc

aagtaatattcagcttaccgcaactaatttggttgatgcacttgttttatctatgctagc

tacctacttatatcttgtatcttgtttgttgtattctacacatacgtactttaactctta

ggcctcctttggttagtaggaatattgtaggaattctataggataggattttcaaagcac

aaaattccttcggagcccttccgtttgtaggaatggattcctcctattcgtatgtaggat

aggaaccaatccttaacatttcaaaggaaaagactcaatggaaaaattcctatcctatgc

atcaaatgacatctctttccctataggaattgagatacatttcatctcacttcctatgat

tttcctattcctatgatattcctatagagcctattcctatagttgtgctctcatatccat

gctctaagcagcaacaactggtcatatccattctttaagcagtaagaactggtcagaact

tcctataatgctcatgtgctatgtggaacatatgcagctggtatagtgctgccttgtgtt

caagttagtcacaagggttttgaagatggtttacaatggaacataagaacatagggcaca

aacaaataaaaaatggaaatctccagaattgttttgttctgactcccataccaagtattt

atgccttcggctgctttgaaattttgaatgtgtaatatttgtggtatttgcactgctacg

aaaggcagttggaaatgtgcaaaggttacacccttcactcttcaaacaagcaaaataaat

aaataaattaggttgtcatcaccaccacttgcatctttgaatcatatttcatatggtgct

gtcagaagtactaccagctgcagattctcaataatcattattcatttgcctcattttctg

gacatcgtaattctattatctttttggtgaaaattaataaaatattgctatgattgtgct

aataatatgatgtcaatttacttatgttgtgctgttgttgtagagtactgtatcttcaaa

aacatgtttaacctaaaacagaagtcaacacctcatcaaagtgtcattgggttcatctca

gctctaacaaatgttgggtttgtagtgtccaaaaacaagtttgaaatggaaattccactt

ggtagaagtttccacacaacttggttcagctgtaaccgtagccaacataagttaagtgaa

gtgtatgtgttcatcagctttccatgacttgttgattatttcaatgcattcaagatgaaa

gataaagttgaatcgagccatcgaaagaacttacagatatgccagctgcaaccatagcaa

acataagttaaatgacagtgtatgcgttcttcagctttccatgacctgttgactatttca

attcattcaagatgaaagatagagctgactcaatccatcgaaagaaagtacagatatgtc

aaatgcaagatgtaaaaactaaacattatctcaggcatgtggaaaaaactgaaccataat

tattgcctttttggaaagaaaaataaatcaagtatcgattaatgctacaaaatatgttgc

ttacctgagtccgtttctatgcag

ATTGTTGCAATAAGGGATATTATACCTCCTGCACAGAGGACTGCATTCAATGATGTCTAT

ATTGCATATGAATTGATGGACACCGATCTGCATCAAATTATTCGCTCAAATCAAGCTTTA

TCGGAGGAGCACTGCCAG

gtacttataagttatcttggtataaaagcaaaaccttctcattcccgctttgtaggacaa

gttagcttatctaattatgttattgtttggctatagaaattcttgtctcctgtatctcac

ttcatctgattcctcgagtacattatactattaattctctgtttcattttattttttgcc

tcagcctcatacatacctctgccagatattagtattctgggagttaatgagtaataacta

taagtcttgcgacctttcttatttttctggtcggtctgaatcatcttgctactttgctag

tctaggtctacgtgctctaaataagtttgcctgatgtatgttataaacattgatgttagt

ggtggaattattggggtatgtttgatgactgtctgttatgttttgcttctttgtaataca

atggaaattggatccgtgcttcgacataggtgaactgtaatattgagaagtaccttactg

tatcacacctcttagaaatgatacaatatatttagaagcttccatatatcaatgcacctc

tgaacatttggtcgagcaaagggagcagttgaacaagttcatcatgtatagcacatgaga

gctgacatattagcatacagcttaatgctagtaaatcctttttctattctttgtcaaaca

cacccatgcatcgtcattgctaatggatgattccctaaatagaagtgctttctcgatatc

agagttgtctcttttgttaacaataccatgtttagctgatactaaatcatgttctacttc

cccaaataccattggataatccttggtggaaaagttccatggaaaactgtttgctatagt

atgcccatgttgtcagcgcctactcgggtttctagcataaaaaaggttgacgctaagtca

tacatgagaaatcagtggatttctggaatggtcacctatttctcttctgttgatcttgtt

ctttccagtttcttttcaatctgcaaatttctgtcttgttctgagaaattgtccttaatc

tgatatggatgttttttgtaattgttccttttttgtgcag

TATTTCCTTTATCAGATCCTTCGTGGCTTGAAGTATATACATTCAGCAAATGTTCTCCAC

CGAGACTTGAAGCCTAGCAATCTTCTATTGAATGCAAACTGTGACCTAAAAATTTGTGAT

TTTGGACTTGCTCGTACCACCTCAGAAACTGATTTTATGACTGAGTATGTTGTGACAAGA

TGGTACAGGGCACCAGAGCTTCTGTTGAACTCCTCTGAATATACTGCAGCAATTGATGTG

TGGTCTGTGGGCTGTATATTTATGGAACTGATGGATCGGAAACCTTTGTTTCCGGGAAGG

GACCATGTCCATCAGCTACGCCTACTAATGGAG

gttagaaagtaccctatgaacttcttttacatcgaatttaaatcatgttgtgtgatcctg

ttttagcataatagaggtatttaagcactttctggtatatataataagtacttttctggt

gcacattaattcattttcaattcccttgtggtatttcctcgctgcag

CTCATTGGAACACCAAATGAGGCCGATTTGGATTTTGTAAATGAAAACGCAAGAAGATAT

ATCCGCCAACTTCCCCGTCATGCAAGGCAATCATTATCTGAGAAGTTTCCACATGTTCAC

CCTTCAGCAATTGACTTGGTTGAAAAGATGCTGACTTTCGATCCTAGACAGAGAATAACA

G

gcaagttttggcatgtgttctagtgatttaaccgtccgcatccatctgctgggatttact

gttgctacaagattgacgatcttatgttccaaacagcaagataaaaatagccgtcctttt

tttgtatcatgaatagtttaatttagaacttgctccatttgttaaatattagtaactgga

atctcatctgggtatgccatcctagtgtttcgttctatatatcattagctaacatcagtt

cctccattcaaatgaaaaaaaagtaattaaattgggctggatttacagcccaagtgggta

ggaacgtgtccatactctctgtaggtatgcaaaggtcattagtttgaagtgggaatgcta

aagtaatcattgttgcctgttgggtggtgtttctattgttgacaatccatagttttgact

gcaaaatatctgtctccaagattcatcctttatttttttggtcagttattgatcgatagt

tactgattgttcctttattttttggtcag

TTGAAGGTGCACTTGCGCATCCTTACTTGGCATCGCTGCATGACATAAGTGATGAGCCAG

TCTGCACGATGCCCTTCAGCTTTGACTTCGAGCAGCATGCGTTGTCGGAAGAACAAATGA

AGGATCTAATCCACCAAGAGGGCATCGCGTTCAACCCTGATTACCAGTAGCTGGAGTTAC

TTTGATCGGCTTCATTCATATGGAAAGTTTTCGGTCCTCCTGCCGCCATAAAATGTTGCT

AGCTGTAAATAATTGCCTCACCTGGAGAATCAAAAGGAGATGGGGTGTTAAGGGTAGATG

ACGAGCTGTAGTGGTCAAATTTCCGCGTAGCCTATGGATTCTTGTGCTTGTGTATGTTGT

TTTATGTGGAATTTTTTTCCTTCTGCTTAAAGATGTTTCAGCGTTTTCCGTAAGATGCGA

TAGTCCGTGAACGATGGCTGCCTAATTTCTGTGGCCGTCATGAGATTTTTACATTGTGGT

CGATTATGGATTATGTAATGTTGTCCTGGTTAATGAACTCTGATTGACTGTGATTGGACT

ATTGTGTTTGAATGTGGACTGACTGGTTTAGTGTTTGAT

**>TaMPK6(7DS) TRIAE_CS42_7DS_TGACv1_622461_AA2040040.1**

CACCATCACCACAACCATATCCATTTCGTGGATCTATACATACCACAACGCACTTAGGGA

TGGACTGTAGCTTTCTTTTCTAGGGTTAGAGATGCAGATAGGTAGGCATGATGATCTTCT

TTTCTCTACCAGAGAAGGTTGATGCCCCATCCCATTTCCCACTTGGAGTAAAAAAGTCAT

CTTCCCGTAGCAGCCGGCCACTCCCACTCGCCACTCGCACGCGCGCTGCCAGCCATCTCC

CCTCCCATCTGCATCCTCCCTCCCCCACCCGTAGGCCGGCACGGCAGCCCCATCCCATCC

CATCCCGACTTCCCGAGCCCAGAAACTGAAGGGGGGAGAGAGAGAAGGGGAGCTGCCACA

CCACCTAAACAAACAAGAGGCGGAAAGCGACCAAATCTCGCGAGCCAAATCCACCGCACG

GGCTTTCCCCTCCCCTCCCCACGCGCGCGATCCGGGCGGAGATGGACGCCGGCGGGGCGC

AGCCGCCGGACTCGGAGATGGCGGAGGCCGGTGCGGGCGCTGCGGCGGCGGCGGGGACGG

CGCCGGGCGGCGCGATGGATAACATCCAGGCCACGCTCACCCACGGCGGGAGGTTCATCC

AGTACAACATCTTCGGCAACGTCTTCGAGGTCACCGCCAAGTACAAGCCCCCCATCCTCC

CCATCGGCAAGGGCGCCTACGGCATCGTCTG

gtaccccccctttccccttctcctttctttcccctccatctccggtccggccgcctgact

gaccgactgactgcgcgcag

CTCCGCGCTCAACTCCGAGACGGGGGAGCAGGTGGCCATCAAGAAGATCGCCAACGCCTT

CGACAACAAGATCGACGCCAAGCGCACGCTGCGGGAGATCAAGCTGCTCCGCCACATGGA

CCACGAGAAT

gtcagtccccgatccgcccctccctccatttcccctttctcgttgaatgctccttgaatg

ctccttgtggttgtgctggttggttgcattgaatggaacggcaggtggatgggctcggtt

aggttttagccgccgaatgattggggttcgccgacagacagctccgtggccggctaccta

atccggggccggcttttccttcctcatctgcgcgagagcttcaggagtagcaatcgtagg

cgagcctttaaagaacccaccaaccttttgggatgcgattcattgtgttcttcctcggtg

cacctggagcagcagcacataagatgctctcgctcctactgctagattctgctgatgtca

aaagctgcctcaggaccaatctatctgcttgtgtcccttttgctgactagctcctccgaa

tccagtgggtgtgtggtggaagtgaatgcgcagttcctaggctagggggctactactgtt

ggagagttcttattaggatgttggacttggatctttctaatgtcctgtgataactaaact

aagtaataggaggaacctacatctgagttaggcatcttgggtcttttcatgtgccttctt

taaaaggctttggtgttattcttgtaatatgaaatgactcgtgttttggcgcgtatttga

gggggaaaaatgttttttggggaagatagccgtgctagcagtacactagaggtggattgt

atcttgacagccttccttggacacctcagcatatgctctgagatgtgtggatttgaagtt

taggtgatgtcaagtaatctgctagcagtaagagcatgaagagaatgggaccctttggct

ttctattctttgtctcatcaagcattgtcgtttgcagattgcgtattctggtggaaggtt

ttcagagtgaaattggtttttggctagtatcctacttaagctagggtttcagatttagtg

ctacactagaatagacaaactttgggagtttgaccgtcagacaattatgaccgttggtag

tgaaacgtcggattaggcaaggtctgattaataacggaaatgcttgtcgacacgtgcttt

atacactgtcagctgtaaacgtatcacctgctgtagctactataaaggcggaagttttca

tttacaagatcctgtacttcgcttttctctctctttccttctgatggcgcgaacgagcat

tcctgacaagcggtgtctgagacggtgcttggtgactaggacaaaagctgaaatgggtag

tttggtggcaccagtgggcgattcaatagctaacccttacgaacggacagagcaacggcc

acattgcattgtcatgcgttcccaggaagcaaattggacttttcagtgcgcttaaggcct

ggtaataaaaccatagcacaggagaaaacatcctgaccataaaggaaaacaatgcaacga

cgcacatttgcctttatagtagctatggctggtgatatggttccctgtcagctatttatg

tccaaaactttctgtaactgtatgctgtcttattcactcttattctccatgtcaaccccc

aaaaaggatgagctatgtaataaagccataaagatttggagaccctgtattccaatagct

atattagaagccttagtttatgtaagggaaaagcctctttgtttgctgacacgaattgtg

ttccccctccaggtcccaaaagcacagagaattacatttgcaaatttttggaaaaagaaa

cagttttgtagatgtacctgtagtcctgtacaaaagtatgtgtacaaaaatgcatgccta

tgcactagtttttgttagtacacatattattgtacgatgcacatcaaaatttcccctctc

atctggaaaattgcaaatgcatttttttcaccagggaacatgtgcagctagaaattgcct

gttgtttacctcctaaaccaagtaatattcggcttagtgcagctacttgggtgatgcact

tgtttcatctatgctagctacctacttgtatcttgtatcttgtttgttgtattttataca

tacataccctgaccttggttgtactcggcatatccatgctcgaagcagcaacgtcaggtc

atatccattctaaagcagtaagaagtggtaacttcctatactgctcatgtgtcatgtggg

aacatatgcagctggtatagtgctgccttgtgttcaagttagttacaagggttttgaaga

tggttcacaatgcaacataaaaacagaggctaccaacaaatagaaaatggaaagctccag

aattgctctgttctgactcccatgccaagtattatgccttctactgctttaaattttgaa

tgcgtaaaatttgtggtatttgcactgctacaaaaggcagccggaaatgtgcaaggttac

accctaaaggctacacccttcactcttcaagcaagcaaaataattaaataaattaggttg

tcatcaccagcaccggcatctttgaattatatttcatatgatgctgtcagaagtactacc

agctgcagattcttaataatgattattcatttgcctcattttctggacatcataactgtt

ttctttttggtgaaaaataataaaatattgctatgattgtgttaataatatgatgccaat

ttacttctgttgtgctgttgttgtagaatattatatcttcaaaaacatgtttaacctaaa

aaagaagccaacaactcatcaaaatgtcattgggtgcatctcagctctaacaaatgttgg

gtttgtactatccaaaaacaagtttgaaatggaaatgttaacttggtagaagtttactcc

ctccgttccaaaatagatgactcaactttgtataactttagtacaaagttgggtcatcta

ttttggaacggagggagtagaagtttccacgcaacttagttcagctgtaactgtagccaa

catagttaagtgaagtgtatgtgttcatcagctttccacgacttgttgatcatttcaatg

caatcaagatgaaatatataagttgactcaagccatcgaaagaacgtacagattgtcagc

tgtaaccatggcaaacataagttatatgaagtgtatgtgtaatgtgttcttcagctttcc

atgacctattgactatttcaattcattcaagatgcaagatatgtcaaatataaagttgat

atgcattcaagatgaaatataaagttgactcaagccatcgaaagaaagtacagatatgtt

aaattgtattgtcttggtgtatgagttttttgtgcaagatgtaaaaactagacattatct

aaggcatgtggccctgttgctgctaaccatagttattgcctttttggaagatttttttct

aagtatgaattaatgctacaaaatatgtttcttacaagagggcatttcttatgcag

ATTGTTGCAATAAGGGATATTATACCTCCTGCACAAAGGACTGCATTCAATGATGTCTAT

ATTGCATATGAATTGATGGACACCGATCTGCATCAAATTATTCGCTCAAATCAAGCTTTA

TCGGAGGAGCACTGCCAG

gtacttattatttatttcgatatagaagtaaaaccttctcataccccctttgtaggataa

gttcgcttatctaattatattattgtttggctatagaaattcttgtctctagtatctcat

ttcatctgattccttgagtacattatactgtcaattatctgtttcattattttttttttg

cctcagctcgtacatacttctgtaagatattagtattctgggagcagggagctaatgagt

aataactaaagcctctcggcctttcttctttttctggtcgtcctgaatcatcttgctact

ttgctagtctaggactacgtgctctaaataagtttgcctggtgtatagtataaacattga

ccttagtggtggaattatggggatatatttgatgactgttggttgatgtttcgcttctct

gtaatacaatggaaattggattagcacaccgacataggtgaactgtaatatactatcatt

gaataagtaccttactgtattacacctcttagaaaggatacaacatattagaaactttca

atgcacccctctgaacatttggtcgaacaaagggagcagtcgaacaagttcatcacatat

agtacatgcttgagagctgacacatcagtatactacttaatgctagcaattcttttttct

atcctttgtcaaacacacccatgcatcgtcattgctaagggatggttcctaaatagaaga

gctttctatatatcagagtggtctcttttgttaacaataccatgtcttgctgatattaaa

tcatgttctacttccgcaaataccatcggataatccttggtggaaaagttccatggaaaa

actgtttgctataatatgcctgtactgtcagtgcatacttgggtttctagcataaagaat

tgacgctagatctcacatgagaaatcagtggttttctggagtggtcacctgtttctcttc

cattgatcttgttctttctagtttcttttcaatccgcaaacttctgtcttgttctgagaa

attgtacttaatctgatgtggatgttttgtgtaattgttccttgtgcag

TATTTCCTTTATCAGATCCTTCGTGGCTTGAAGTACATACATTCAGCAAATGTTCTCCAC

CGAGACTTGAAGCCTAGCAATCTTCTTTTGAATGCAAACTGTGACCTAAAAATTTGCGAT

TTTGGGCTTGCTCGTACCACCTCAGAAACTGATTTTATGACCGAGTATGTTGTGACAAGA

TGGTACAGGGCACCAGAGCTTTTGTTGAACTCCTCCGAATACACTGCAGCAATTGATGTG

TGGTCTGTGGGCTGTATATTTATGGAACTGATGGACCGGAAACCGTTGTTTCCAGGAAGA

GACCATGTCCATCAGCTACGTCTACTAATGGAG

gtttagatagtgctctatgaacttcttttatatctaatttaaatcatgttgtgtggtcct

gttttagcataatacggagtactatctagtatgatcatagttttttctcgaagtagaggt

atttaagcacttgactggtatatataataagtacttttctggtgcacattaattcatttt

taattcccttgtggtatttcctcgttgcag

CTCATTGGAACACCAAATGAGGCCGATTTGGATTTTGTAAATGAAAATGCAAGAAGATAT

ATCCGCCAACTTCCCCGTCATGCAAGGCAATCATTATCTGAGAAGTTTCCACATGTTCAC

CCTTCAGCAATTGACTTGGTTGAAAAGATGCTGACTTTCGATCCTAGACAGAGAATAACA

G

gcaagttttggcatgtgttgtagtgatttaaccatccgcatccatctgctgggatttatt

gttgcttcaagactgacgatcttatgttccaaacagcaagatagaaagagctgtcttatt

ttttggtatcatgagtagtttagtttaaaacttgctccatctgttattagtaactggaat

ctcatctgggtatgccatcctagtgtttcgtgttatatatcatgagctaacatcagttcc

tccattcaaatgaaaaaaaagtaattaaattggaaaggtcacccaggtaaatatatggat

catccgggctggatttacagcccaagtgggtaggaatgtgtccatactctctgtaggtat

gcaaaggtcattagtttgaagtgggaatgctaaagtaatcattgttacctgttgggtggt

gtttctattgttgacaatccatatttttgactggaaaatatccatctccaagattcatcc

tttactttttcggtcagttattgatcgatagttactgattgttcctttattttttggtca

g

TTGAAGGCGCACTTGCGCATCCTTACCTGGCATCGCTGCATGATATAAGTGATGAGCCAG

TCTGCACGATGCCCTTCAGCTTTGACTTCGAGCAGCATGCGTTGTCGGAAGAACAAATGA

AGGATCTAATCCACCAGGAGGGCATCACGTTCAACCCTGATTACCAGTAGCTGGAGTTCC

TTTGATCGGCTTCATTCATATGGAAAGTTTTCGGTCCTCCTGCCGCCATAAAATGTCGCT

AGCTGTAAATAATTGCCTCACCCGGAGAATCAAAAGGAGATGGCGTGTTAAGGGTAGATG

ACAAGAGCTGTGGTGGTCAAATTTCCGCGTAGCCTATGGATTCTTGTGCTTGTGTATGTT

GTTTTATGTGGAATTTTTTTCCTTCTGCTTAAAGATGTTTCAGCATTTTCTGTAAGATGC

GATAGTCCGTGAACGATGGCTGCCTAATTTCTGTGGCCGTCATGAGATTTTTACATTGTG

GTCGATTATGGATTATGTAATGTTGTCCTGGTTAATGAACTCTGACTGACTGTGATTGGA

CTATTGTGTTTGAACGCGGACTGACCATGATTTAGTGGTTAATGTTTGAAGCTGT

**>TaMPK7(7AL)** **TRIAE_CS42_7AL_TGACv1_558385_AA1792950.1.X (based on AA1792950.1)**

AGCGCCAGCGGCACCCATCGCCATCTTCCTCATCGCAGCCCCACCACCACTCCCCCGAGT

CCAAGTCCGTGGAGCTACGCGCGTGCGTGCGTGCGTACGTACGTTCCTTCCTTCCTCCCT

CCCTCCCTCCCTTCCCCGAAATAAAAAATAGACAGAGAGCCTCATGCTCGGTCGCTCCCT

GCCTTTGCCTCCGCCTCCGTCTCCGCGTGCGCTCCCCATCCCCATCCCCTGCCTCCCCCG

CCCCCGGACGTGCTCTGCTTCTCCAGATCCGCCGCCGACGCTGCTGCTGCCGCCGCGTCC

TGAG

gtgagtggacaatccgcccgcgcacgggacggggagcttctccccgcccccattttcttt

cctttcctttcctttcctcgtactgctttcgtgaggcgcagcggagccgaattgggtcgc

ttccggtgccggccgccgctaggatgctcgtgtcaggggcagccgggacgagttgcgtcg

aaatgcggccgattcggtccgattcggcggcggattgcagcgcccagccatggattgtgc

tgcgctagctggggttgtggggggagagagggtggctctgattcaaaacacaccactggg

actgggtcagtcaaacaaacagtgttagacaggcactggtgttaggttgaggataaaaat

tgtgcgggggtgcaaatacatgctgctggtaaaggtcaagctcataagacttggcaaaat

taaaatggatgaaggcttaagcgcatcgctcgcgccatggttatgatgaattgtgtgtgg

ggtggaaacagagactgttactaccacggggctaaaaagtcaagctcataagacttggca

aaattaaattggcaccctaaactagtaaacttcgtccttgttattatccttccattgttc

tcaattaatagacagctaactctatgtttcctttttccccgtag

AAAATGGCGATGATGGTGGATCCACCGAACGGCATGGGAAACCACGGGAAGCACTACTAC

ACCATGTGGCAGACCATGTTCGAGATTGACACCAAGTACGTGCCGATCAAGCCCATCGGA

AGAGGAGCCTACGGGATAGTTTGCTCCTCGATCAACCAGGAGACCAACGAGAAGGTCGCC

ATCAAGAAGATAAACAACGTCTTTGACAACCGCGTGGATGCGCTGAGGACGCTGCGCGAG

CTGAAGCTCCTTCGGCACCTGCGCCATGAGAATGTCATTGCCTTGAAGGATATAATGATG

CCGATACATAGGAGGAGCTTCAAGGATGTCTACTTGGTCTCCGAGCTCATGGACACGGAT

CTGCATCAGATTGTCAAGTCGTCTCAGCCGCTGTCCAATGACCACTGTCAGTATTTCCTT

TTTCAG

gtatgcgccggctctgtttttcattcttacctttgaattccatgtagtccttcatttgtt

ctcaccattctaccataataatctgttcacacctcaagtgtatggcaagataatgttctt

tttatcgttcatactaatttgcattaagtgcatgatctatctaaagttgttgagtttcaa

ctagttgagttgccaaagaatgatatgctaatttatggaagaagcgcatgccagtttgaa

tatttctagcaggagcttctatagctgctccccttggaagtgcatttcttaaaagcaacc

taatacacagcagatctgcacatggtatttgcatgaacctgtaacttaagcctctgtgaa

ttatttcaggatagctatatacaaattgaacttgagcatcttgtattggactgattctta

gatgcttgtcttatgaattagtcttgtttgctatcatgtcactttctcacattcttctga

tcctttttctcccatatttgtcaataacaccttttttttcttgactgtag

CTGCTCCGAGGACTGAAGTACCTTCATTCAGCAGGGATACTCCATAGAGACCTGAAGCCT

GGGAACCTTCTGGTCAATGCAAACTGTGACCTGAAGATCTGTGACTTTGGTCTGGCTCGC

ACAAATAACACTAAAGGTCAGTTTATGACTGAATACGTTGTCACCCGCTGGTACAGAGCT

CCCGAGCTGCTGCTCTGCTGCGACAACTATGGCACCTCCATAGATGTCTGGTCTGTTGGC

TGCATCTTTGCTGAGCTACTTGGCCGCAAGCCGATCTTTCCAGGAACCGAGTGCCTTAAT

CAGCTTAAGCTCATAGTCAATGTTCTTGGCACCATGAGCGAGGCTGACCTCGCGTTCATT

GACAACCCGAAAGCGCGCAACTACATTAAATCCCTTCCATACACCCCAGGGATTCCCCTC

AGTAGCATGTACCCGCAAGCGCACCCTCTTGCCATTGACCTGTTGCAGAAGATGCTTGTC

TTTGACCCTTCCAAAAGGATCAGTGTCACCCAGGCTCTGGAGCACCCCTACATGTCCCCG

CTGTATGATCCCAGCGCAAACCCTCCTGCGCAGGTGCCCATCGATCTCGACATAGATGAA

AACATTGGCACAGATATGATCCGAGAAATGTTGTGGCAGGAGATGCTCCAGTACCACCCC

GAGGCCGCCAGGATGGTGAATATGTGACAAGCAGGAATGAACATGTGAGAGCAGTGTGCC

ACACCAGGGTCTTCACATGTTCGTTCTTGGTATAAAGCTTTAATGCAATTATCGCAATGC

CGTCGAGTGACCTGTTTATGTAAATATGTGCACAATAAACGGCGTATGGATTTCTCTAGC

TGTGGGTCAGTACTTGGTAGTATATATGGACTGCTGTGTTGTAGAAGCGGAATTCGGTTA

TGAAGAAGCTTGTGTAAGTTGTAATCTTTTTTTTTCCTTGATTGTAAGTTTGTAATCTTA

GACACTGCTCTATGTATATGGACTACTGTGTTGTAGAAGCGGAATTCAGTTTATTACGAA

TTGGGTTATGAAGAAGCTCGTGTAAGTTGTAATCTTCTCTTCTCCCCATGGTTGTAAGTT

GTAATTTTAGAAGCTGCTCTATGTATGGAGGTCTAAACTTTCAAGTGACCTTGTTTTGCT

TCTACCTTCAAAATTGTCTTGTTTCTTCAAGAGCTAGGAAATTGCAGTTTGTATGGTATT

TTCTGTTTGGCTTAAAATGTATAAACTCTGAAGCCCCCT

**>TaMPK7(7BL)** [**TRIAE_CS42_7BL_TGACv1_576902_AA1859240.1**](http://plants.ensembl.org/Triticum_aestivum/Transcript/Exons?db=core;g=TRIAE_CS42_7BL_TGACv1_576902_AA1859240;r=TGACv1_scaffold_576902_7BL:49137-52305;t=TRIAE_CS42_7BL_TGACv1_576902_AA1859240.1;tl=BRUtAdpyg0y79eF1-9487995-175733733)

CCTGCCGCGTGTTCCATCGCTAGCTGCCCGCCCCTTCGCTGTACCTGAGCGGCACCCATC

CATCGCCATCTCCTCGTCACCACTCCCACAAGTCCAGTGGAGCTACGTGCGTGCGTACGT

TCGTTCCTTCCTTCCTTCCTTCCCCGAAATAAAAAATAGACAGAGAGCCCTCCTGCTCGG

TCGCCCTCTGCCCCTGCCTCCGTCTCCGCGTGCGCTCCCCACCCCCTTCCTCCCCCGCTC

CCGGACGTGCTCTGCTTCTCCAGATCCGCCGCCGACGCTGCTGCTGCCGCGTCCTGAG

gtgagtgatcgatcccgcccgcacacggggcggggggctcgccccctcctcctccatttt

cgttccctttcttgaggcgaagcaaagcaagagcttatgcatgcgggtggcgaattgggt

ttgcttccggctccggtcgccggtaggatgctcgtgttaggggcagccgggaggagttgc

gtcgaaatgcggccgattcggtccgattcggcggcggattgcagcgcccagctttggatt

gtgctgcgctggccgctgggcttggggtcagagggtggctctgattcgtttcaaaacaca

ccactgggactgggtcagtcaaacaaacagtgttaggcagacactggtgttaggttgagg

ataaaattgtgggggtggaaacacatgctgctagtagttgtgccatggaaaaggtcaagc

tcataagacttggcaaaattaaattggccaaggcggatgagcgtcgctcaccgtgttagc

ctgttagacacttgtgttagggtgatgatgacttgtgtgtggggtggaaacagagactgt

tagtaccatgggtcaagctgataagacttggcaaaattaaattggcaccctaaactagta

aactttttccttgttatgcttcctttgttctcaattaatagactggtaactctatgtttc

cttcttctccgcag

AAAATGGCGATGATGGTGGATCCCCCGAATGGCATGGGAAACCACGGGAAGCACTACTAC

ACCATGTGGCAGACCATGTTCGAGATCGACACCAAGTACGTGCCGATCAAGCCCATCGGG

AGAGGAGCCTACGGGATAGTTTGCTCCTCGATCAACCAGGAGACCAACGAAAAGGTCGCC

ATCAAGAAGATAAACAACGTCTTTGACAACCGCGTGGATGCGCTGAGGACGCTGCGCGAG

CTGAAGCTCCTTCGGCACCTGCGCCATGAGAATGTCATTGCCTTGAAGGATATAATGATG

CCGATACATAGGAGGAGCTTCAAGGATGTCTACTTGGTCTCCGAGCTCATGGACACGGAT

CTGCATCAGATAGTCAAGTCGTCTCAGCCGCTGTCCAATGACCACTGCCAGTATTTCCTT

TTTCAG

gtatgcgtcggctctgttttgcattcttacctttgcataacgcttatccatgtagtcttt

catctgttcaccattctgacataagctagtttgttcatgcttcaggtgtacgccagtata

atgttgtttttgcagttcgtactaatttgcattaagtttatgatctatctaaagctgttg

ggttttaactagttaagttgccaaagaacaatctgctaatttatggaagaagcacatgtc

aatttgaatatgtctagtagtagcttagcttctatagctgctccctaatggtgcttgtgc

cctggaagtgcatttcttaaaagcaacctaatacacagcagacatgcatatggtatttgc

atgaacctataacttgagcctctgtaaattatttgaggaaaactatgtagaaactgaact

tgagcatcttgcattggaccgataattaaatagttgtcctacagattagtcttgtttgtt

accatgccatcttctcctttttctgctgatcctttttctctcatatttgtcgatcaatca

ataatctaagaccccactattttccttgactgtag

CTGCTCCGAGGACTGAAGTACCTTCATTCAGCGGGGATACTCCATAGAGACCTGAAGCCT

GGGAACCTTCTGGTCAATGCGAACTGTGACCTGAAGATCTGTGACTTTGGTCTGGCTCGC

ACAAATAACACTAAAGGTCAGTTTATGACCGAATACGTTGTCACCCGCTGGTACAGAGCT

CCCGAGCTGCTCCTCTGCTGCGACAACTATGGCACCTCCATAGATGTCTGGTCTGTTGGC

TGCATCTTTGCTGAGCTACTTGGCCGCAAGCCGATCTTTCCAGGAACCGAGTGCCTTAAT

CAGCTTAAGCTTATAGTCAATGTTCTTGGCACCATGAGCGAAACTGACCTTGCGTTCATT

GACAACCCAAAAGCTCGCAACTACATTAAATCCCTTCCATACACCCCGGGGATGCCCCTC

AGTATCATGTACCCGCACGCCCATCCTCTTGCCATTGATCTGTTGCAGAAGATGCTTGTC

TTTGACCCTTCCAAGAGGATCAGTGTCACCCAGGCTCTGGAGCACCCCTACATGTCCCCG

CTGTATGATCCCAGCGCAAACCCTCCTGCTCAGGTGCCCATCGATCTCGACATAGATGAA

AACATTGGCACAGATATGATCCGAGAAATGTTGTGGCAGGAGATGCTCCAGTATCACCCT

GAGGCCGCCAGGATGGTGAATATGTGACAAGCAGGAATGAACATGTGAGAGCAGTGTGCC

ACACCAGGGTCTTCACATGTTCGTTCTTCATATAAAGCTTTAATGCAATTATCGCAATGC

CGTCGAGTGACCTGTTTATGTAAATATGTGCACAATAAACGGCGTATGGATTTCTCTAGC

TGTGGATCAGTACTTGCTAGTATATATAGACTACTGTGTTGTAGAAACGGAATTCGGTTA

TCAAGAATCTGGTTATGAAGAAGCTTGTGTAAGTTGTAATCTTCTCTCTTTTTTCCCCTG

ATTGTAAGTTTGTAATCTTAGACACTGCTCTATGTATATGGACTACTGTGTTGTAGAAGC

GGAATTCGGTTTATTAAGAATTGGGTTATGAAGAAGCTTGTGTAAGTTGTAATATTCCCT

TTTCCCCATGATTGTAAGTTGTAATTTTAGAAGCTGCTCTATGTAAGGAGGTCTAAACTT

TCAAGTGACCTTGTTTTGCTTCTACCTTCAAATTTGTCTTGTTTCTT

**>TaMPK7(7DLc1) TRIAE_CS42_7DL_TGACv1_603035_AA1974260.1**

GCCTTCCCCATCATCTTCCTCAGCCTGCCGCGTGTGCCATCGCTAGCTGCCCGCCCCTTC

GCTGTACCTGAGCGGCACCCATCGCCATCTTCCTCATCCCACTCCCCCAAGTCCAGTGGA

GCTACGTGCGTGCGTACGTTCGTTCGTTCCTTCCTTCCTTCCCCGAAATAAAAAATAGAC

AGAGTGCCCTCCTGCTCGGTCGCTCTCTCTCTCTACCTTTGGCCTCCGCCTTCGTCTCCG

CGTGCGCTCCCCATCCCCTTCCTCCCCCGCCCGCTCCCGAACGAGCTCGGACGTGCTCTG

CTTCTCCAGATCCGCCGCCGACGCTGCTGCCGCCGCGTCCTGAAG

gtgagtgatcgatcccgcccgcgcacggggcggggggcttgccccctcctcctccatttt

cgttccctttcgtgaggcgaagcaaagcaagagcttatgcatgcgggtggcgaattgggt

ttgcttccggttccggtcgccgctgggatgctcgtgttaggggcagccgggaggagctgc

gtcgaaatgcggccgattcggtccgattcggcggcggattgcagcgcccagctatggatt

gtgctgcgctggccgctgggcttcggggacagatggtggctctgattcagtttcaaaaca

caccgctgggactgggtcggtcaaacaaacagtgttagacagacactggtgttaggttga

cgataaaattgtgggggttgaaacacatgctgctagtagtggtgccatggaaaaggtcaa

gctcataagacttggcaaaattaaattggccaaggcgtatgagcgtcgctcaccgcgttg

ttctgttagacacttgtgttagggtgatcatgaattgtgtgtagggtggaaacagagact

gttagtaccatggggaaaaaagtcaagctcataagacttggcaaaattaaattggcgccc

taacctagtaaacgttttcattgctatgcttccattattatcaagtatagaatgttaacc

cgtgtttccttcttcttggtag

AATATGGCGATGATGGTGGATCCTCCGAACGGCATGGGAAACCACGGGAAGCACTACTAC

ACCATGTGGCAGACCATGTTCGAGATCGACACCAAGTACGTGCCGATCAAGCCCATAGGG

AGAGGAGCCTACGGGATAGTTTGCTCCTCGATCAACCAGGAGACCAACGAGAAGGTCGCC

ATCAAGAAGATAAACAACGTCTTTGACAACCGCGTGGATGCGCTGAGGACGCTGCGCGAA

CTGAAGCTCCTTCGGCACCTGCGCCATGAGAATGTCATTGCCTTGAAGGATATAATGATG

CCGATACATAGGAGGAGCTTCAAGGATGTCTACTTGGTCTCCGAGCTCATGGACACAGAT

CTGCATCAGATTGTCAAGTCGTCTCAGCCGCTGTCCAATGACCACTGCCAGTATTTCCTT

TTTCAG

gtatgcgtcggctctgtttttcattcttacctttgaattccatgtagtccttcatttgtt

caccaatctaccataataagttaatctgttcacacctcaggtgtatggcaagataatgtt

gtttttgtcgttcatactagtttgcattaagtgcatgatctatctaaagttgttgagttt

caactagttgagttgccaaagaatgatctgctaatttatggaagaagcgcatgccagttt

gaatatttctagcaggagcttctatagctgctccccctggaagtgcatttcttaaaagca

acctaatacacagcagatctgcacatggtatttgcatgaacctataacttaaacctctgt

gaattatttcaggatagctatatacaaattgaacttgagcatcttgtattggactgatac

ttagatgcttgtcttacgaattactcttgtttgttaccatgtcactttctcatattcttc

tgaaccttcttctcccatgtttgtcaatcaatcattaatctaagatgccaccatttttct

tgactgtag

CTGCTCCGAGGACTGAAGTACCTTCATTCAGCAGGGATACTCCATAGAGACCTGAAGCCT

GGGAACCTTCTGGTCAATGCAAACTGTGACCTAAAGATCTGTGACTTTGGCCTGGCTCGC

ACAAATAACACTAAAGGTCAGTTTATGACTGAATACGTTGTCACCCGTTGGTACAGAGCT

CCCGAGCTGCTGCTCTGCTGCGACAACTATGGCACCTCCATAGATGTCTGGTCTGTTGGC

TGCATCTTTGCTGAGCTACTTGGCCGCAAGCCGATCTTTCCAGGAACCGAGTGCCTTAAT

CAGCTTAAGCTTATAGTCAATGTTCTTGGCACCATGAGCGAGGCTGACCTCGCGTTCATT

GACAACCCGAAAGCGCGCAACTACATTAAATCCCTTCCATACACCCCGGGGATGCCCCTC

AGTAGCATGTACCCACGCGCGCACCCTCTTGCCATTGATCTGTTGCAGAAGATGCTTGTC

TTCGACCCTTCCAAGAGGATCAGTGTCACCCAGGCTCTGGAGCACCCCTACATGTCCCCG

CTGTATGATCCCAGCGCAAACCCTCCTGCTCAGGTGCCCATCGATCTCGACATAGATGAA

AACATTGGCACAGATATGATCCGAGAAATGTTGTGGCAGGAGATGCTCCAGTACCACCCT

GAGGCCGCCAGGATGGTGAATATGTGACAAGCAGGAATGAACATGTGAGAGCAGTGTGCC

ACACCAGGGTCTTCACATGTTCGTTCTTGGTATAAAGCTTTAATACAATCATCGCAATGC

CGTCGAGTGACCTGTTTATGTAAATATGTGCACAATAAACGGCGTATGGATTTCTCTAGC

TGTGGGTCAGTACTTGGTAGTATATATGGACTGCTGTGTTGTAGAAGCAGAATTCGGTTA

TTAAGAATCTGGTTATGAAGAAGCTTGTGTAAGTTGTAATCTTCTATTTTTTTTCCCTGA

TTGTAAGTTTGTAAGCTTAGCCACTGCTCTATGTATATGGACTACTGTGTTGTAGAAGCG

GAATTCGGTTTATTAAGAATTGGGTTATGAAGAAGCTTGTGTAAGTTGTAATCTTCTCTT

CTCCCCATGATTGTAAGTTGTAATTTTAGAAGCTGCTCTATGTATGGAGGTCTAAACTTT

CAAGTGACCTTGTTTTGCTTCTACCTTCAAATTTGTCTTGTTTCTT

**>TaMPK7(7DLc2) TRIAE_CS42_7DL_TGACv1_603254_AA1979200.2**

TTCTCATCGTCTTCCTCAGCCTGCCGCGTGTTCCATCTCGCTATCTGCCCGCCCCTTCGC

TGTACCTGAGCGGCACCCATCGCCATCTTCCTCATCACCACTCCCCCAAGTCCAGTGGAG

CTACGTGCGTGCGTACGCTCGTTCGTTCCTTCCTTCCTTCCTTCCCCGAAACAAAAAAAA

TAGACAGAGAGCCCTCCTGCTCGGTCGCTCTCTGCCTTTGCCTCCGCCTCCGTGATGATG

AATTGTGTGTGGGGTGGAAACAGAGACTGTCAGTACCATGGGTCAAGCTGATAAGACTTG

GCAAAATTAAATTGGCACCCTAAACTA

gtaaactttttccttgttatgcttccattgttctcaattaatagataactctatgtttcc

ctcttctccgcag

AAAATGGCAATGATGGTGGATCCCCCGAACGGCATGGGAAACCACGGGAAGCACTACTAC

ACCATGTGGCAGACCATGTTCGAGATCGACACCAAGTACGTGCCGATCAAGCCCATCGGG

AGAGGAGCCTACGGGATAGTTTGCTCCTCGATCAACCAGGAGACCAACGAGAAGGTCGCC

ATCAAGAAGATAAACAACGTCTTTGACAACCGCCTGGATGCGCTGAGGACGCTGCGCGAG

CTGAAGCTCCTTCGGCACCTGCGCCATGAGAATGTCATTGCCTTGAAGGATATAATGATG

CCGATACATAGGAGGAGCTTCAAGGATGTCTACTTGGTCTCCGAGCTCATGGACATGGAT

CTGCATCAGATAGTCAAGTCGTCTCAGCCGCTGTCCAATGACCACTGCCAGTATTTCCTT

TTTCAG

gtatgcgtcggctctgtttttcatttttacctttgcataacgcttatccatgtagtcttt

catctgttcaccattctgacataagctagtttgttcatgcttcaggtgtacgctagtata

atgttgtttttgcagttcatactaatttgcattaagttcatgatctatctaaagctgttg

ggttttaactagttaagttgccaaagaacaatctgctaatttatggaagaagcacatgtc

aatttgaatatgtctagtagtagcttagcttctatagctgctccctaatggtgcttgtgc

cctggaagtgcatttcttaaaagcaacctaatacacagcagacatgaatatggtatttgc

atgaacctataacttgagcctctgtaaattatttgaggaaaactatgtagaaactgaact

tgagcatcttgcatcggaccgataattaaatagttgtcctacagattagtcttgtttgtt

accatgtcatcttctcctttttctgctgatcctttttctctcatatttgtcgatcaatca

ataatctaagacccccaccattttccttgactgtag

GTGCTCCGAGGACTGAAGTACCTTCATTCAGCGGGGATACTCCATAGAGACCTGAAGCCT

GGGAACCTTCTGGTCAATGCGAACTGTGACCTGAAGATCTGTGACTTTGGTCTGGCCCGC

ACAAATAACACTAAAGGTCAGTTTATGACAGAATACGTTGTCACCCGCTGGTACAGAGCT

CCCGAGCTGCTACTCTGCTGCGACAACTATGGCACTTCCATAGATGTCTGGTCTGTTGGC

TGCATCTTTGCTGAGCTACTTGGCCGCAAGCCGATCTTTCCAGGAACTGAGTGCCTTAAT

CAGCTTAAGCTTATAGTCAATGTTCTTGGCACCATGAGCAAGGCTGATCTCGCGTTCATT

GACAACCCAAAAGCTGGCAACTACATTAAATCCCTTCCATACACCCCGGGGATGCCCCTC

AGTATCATGTACCCGCACGCGCACCCTCTTGCCATTGATCTGTTGCAGAAGATGCTTGTC

TTCGACCCTTCCAAGAGGATCAGTGTCACCCAGGCTCTGGAGCACCCCTACATGTCCCCG

CTGTATGATCCCAGCGCAAACCCTCCTGCTCAGGTGCCCATCGATCTCGACATAGATGAA

AACATTGGCACAGATATGATCCGAGAAATGTTGTGGCAGGAGATGCTCCAGTACCACCCT

GGGGCCGCCAGGATGGTGAATATGTGACAAGCAGGAATGAACATGTGAGAGCAGTGTGCC

ACACCAGGGCCTTCACATGTTCATTCTTCGTATAAAGCTTTAATGCAATTATCACAATGC

CGTCGAGTGACCTGTTTATGTAAATATGTGTACAATAAACGGCGTATGGATTTCTCTAGC

TGTGGGTCAGTACTTGGTAGTATATACATAGACTACTGTGTTGTAGAAGCGGAATTCGGT

TATGAAGAAGCTTGTGTAAGTTGTAATCTTCTTCTTTTTTTCCCTGATTGTAAGTTTGTA

ATCTTAGACACTACTCTATGTATATGGACTACTGTATTGTAGAAGCGGAATTCGGTTAAT

TAAGAATTGGGTTATGAAGAAGCTTGTGTAAGTTGTAATCTTCTCTTCTCCCCATGATTG

TAAGTTGTAATTTTAGAAGCTGCTCTATGTATGGAAGGCCTAAACTTTCAAGTGACCTTG

TTTGCTTCTACCTTCAAATTTGTCTTGTTTCTTCAAGAGCTAGAAAACTGCAGTTTGTAT

GATGTTTTCTGTTTGGCTTAAAATGTATAAACTCTGAAGCCCCCT

**>TaMPK11(7AL) TRIAE_CS42_7AL_TGACv1_555982_AA1751700.1**

ACGGGCATTTTCCCTCCAAAAAGCTTCCTCTCCTCCTTTTTCCCCTTTTCCCCAACGCTC

ATATCTTCTCCCTCGTCCATCCAACCAAGCTAGCAATCTCTCTCTCTCTCTCATCCTCCG

CGCGGCGTGCGTTTCTTTCTTTCTTGGCTAGCTCCTCGATCAAGGTGTTCGCGCGCAGGC

GTGCGGGTCGATGCGCATGGAGGGTGGAGGCGCCGGGGCAGGAGGAGGAGGTCACGGCGG

CGGCCATGGCCTCGGCGGCGAGGCGCAGATCAAGGGCACGCTCACCCACGGCGGCAGGTA

CGTGCAGTACAACGTCTACGGCAACCTCTTTGAGGTCTCTGCTAAGTACGTCCCACCCAT

CCGACCTGTCGGCCGCGGCGCCTGCGGCATCATCTG

gtacgtacgtcatgcgctgcatctgctcgcgtatattcctcagttacctcagaaaatgga

ttgctttgctcgatcagctgactgcgatcctgttgcgagttcgctacagcatatacgact

gaaagtgggtcttgattaaagctacaaattaccatagcctagcgtatatatatattatag

tattaaaccataaagctttctataacggaccagtactagttaattgttatggtatggcta

tatttaacagcaaaaatagcttctcaatactaaaaggaaacaagagctgtttctagttga

ttttgagcctgtggtgacaaattcttttctgtgatcttcttctccag

CGCTGCTGTAAATGCACAGACTCGTGAGGAGGTCGCTATCAAAAAGATAGGTAATGCGTT

TGACAACCAGATCGATGCCAAACGCACTTTGCGAGAAGTAAAGCTGCTTCGCCACATGAA

TCATGAGAAT

gtgagccatctttcctcctctctcatgtgccttcttgtaataaacctctgtggttcacat

gctcatatctgaaatgttttgtgatttctatttgaccatggttaactctgtagtttatag

caaaggtaagccgtcctgaggagatctgccggaaattctggtgattttctttcaaagcca

gaatcgcctgtcattatttagttactatacagacgacatgtgtgacaaaagttgaagttc

cctacctgtgctaccgtgtttaaactttgaactatcttgcaacataagtccacatgatgt

gtgattatctccacttatgatatcgaaaatatatatggtgtgaccctgaatttggtggta

tgatcatgacaaaatctgacttttggcag

GTGATTTCAATAAAGGACATCATACGCCCACCAAGGCGGGAGAACTTCAACGATGTTTAC

ATCGTCTATGAACTGATGGACACTGATCTTCACCACCTTCTAAGATCAAACCAGCCACTC

ACGGATGATCACTGTCAG

gtatgtacattgttctttccatcatcccttcagcatcagtccaaacattctacaattggt

cagctttcctttcaccttaacattttcatgatgcag

TATTTTCTCTACCAAGTGCTCCGAGGATTGAAGTATGTGCATTCAGCAAAGGTCTTGCAC

CGGGACCTCAGACCGAGCAACCTGCTGCTCAATGCTAAGTGTGAACTCAAGATTGGAGAT

TTTGGCTTGGCTAGGACCACGACTGAGACTGACTTCATGATGGAGTATGTTGTTACTCGG

TGGTACAGGGCACCGGAGCTCCTGCTCAACTGCTCGGAGTACACTGCAGCAATTGATATC

TGGTCAGTGGGTTGCATCCTCGGTGAGATTGCTATGAGGGAGCCACTGTTTCCTGGAAAA

GATTATGTTCATCAGCTGAGGCTAATTACTGAG

gtatggccactcactactacacgagtcaagattttattttttcccttctgcttctttttt

cttctttttgtatattctcctgctctctttttgcctcggagtctgacactatgattgtca

gttctgatttagcataggttccaactgctatgctttttccatgcttatatctcttggatt

accaactcaattagcacaagtacaccacataggttgaagcttacactggtatctggtgta

gcatgatacttcatgcatatgccttcattcagactagtgttttggtccaagcatgtgatt

attctagatgcactaacatcttctcttgttctactaaatggaaatgtctaataaatcttc

tcgatgctgtttgtaatcctgattcattcttgatccaaag

CTGATAGGCTCACCAGATGACACGAGCCTTGGGTTTCTTCGAAGTGATAATGCCCGCAGA

TACGTGAG

gtctcttcnnnnnnnnnnnnnnnnnnnnnnnnnnnnnnnnnnnnnnnnnnnnnnnnnnnn

nnnnnnnnnnnnnnnnnnnnnnnnnnnnnnnnnnnnnnnnnnnnnnnnnnnnnnnnnnnn

nnnnnnnnnnnnnnnnnnnnnnnnnnnnnnnnnnnnnnnnnnnnnnnnnnnnnnnnnnnn

nnnnnnnnnnnnnnnnnnnnnnnnnnnnnnnnnnnnnnnnnnnnnnnnnnnnnnnnnnnn

nnnnnnnnnnnnnnnntgag

GTCTCTTCCTCAATACCCGAAACAGCATTTTGGTTCACGGTTCCCCAGTATGTCCACTGG

CGCCATGGATTTGCTTGAGAGGATGCTCGTATTTGATCCGAGCAAGAGGATTACTG

gtaatcttacagcatagcatgatcttgcttgattgcaaaatgacaaatttaataatttga

accatcatttcagaatcgtgtgcgaattattaaaaccaagttgaaatgacacttcttacg

ttaattaaaccattaatgtaagaaatattacgaggaactttctcttggaagtttaacctt

tttttttcttggtggcttcgcag

TTGATGAGGCTCTATGCCATCCTTATTTAGCATCCCTTCATGAGATAAATGATGAACCTG

TCTGCCCAGCGCCTTTCAGCTTCGACTTCGAACAGCCATCATTTACTGAGGAAGATATCA

AAGAACTCATTTGGAGGGAGGCTCTCAAGTTCAACCCTGAACCAATTCACTGAAAAGTTT

CCAATGTAGAAAAAGAAGCAATTTCAAATGGCAAATCTATCAGCTCCGTGGGAAAATACA

TGAGCTGACAGCACGATGTACAGAGTAATCAGTGATCACGTCTCAGCATCTTGTCATACG

GCTTGTTGATGACACCATGACGTCGAAAACCTGGAAATTTAGGAATAATGAACGCTTGAT

CAATAATTTGTTAGCCAAAAAAAGTGGGGCCCTTATC

**>TaMPK11(7BL): TRIAE_CS42_7BL_TGACv1_580661_AA1914980.1**

ATCCAACCAAGCTAGCAATCTCTCTCTCTCTCATCCTCCACGCGGCGCGCGGTTCTTTCT

TTCTTGGCTAGCTCCTCGATCAAGGTGTTCGCGCTCAGGCGTGCGGGTCGATGCGCATGG

AGGGTGGAGGCGCCGGGGCAGGAGGAGGAGGCCTCGGCGGCGAGGCGCAGATCAAGGGCA

CGCTCACCCACGGCGGCAGGTACGTGCAGTACAACGTCTACGGGAACCTCTTTGAGGTCT

CTGCTAAGTACGTCCCGCCCATCCGACCTGTCGGCCGCGGCGCCTGCGGCATCATCTG

gtacgtacgtcatgcgctgcatctgctcgcgtatattcctcagttacctcataatatgga

ttgctttgctcgatcagctgactgcgatcctgttgcaagttcgctatagcatatacgact

gaaagtgggtcttgattaaagctacaaattaccatagcctagcgtatatatagtattaaa

ccgtaaaagctttctctaatggaccagtactagttacagtaattgttatggtatatggct

atatttaacagaaaaaatagcttctcaatactaaaaggaaacaagagctgtttctagttg

attttgagcctctggtgacaaattcttttctgtgatcttcttcttcag

TGCTGCTGTAAATGCACAGACTCGTGAGGAGGTCGCTATCAAGAAGATTGGTAATGCGTT

TGACAACCAGATCGATGCCAAACGCACTTTGCGAGAAGTAAAGCTGCTTCGCCACATGAA

TCATGAGAAT

gtgagccatctttccttctctctcatgtgccttcttgtgataaacctctgtgggtcacat

gctcatatctgaaatgttttgtgatttctatttgaccatgattaactctgtagtttatag

caaaggtaagccgtcctgaggagatctgccggaaattctggtgattctctttcaaagcca

gaatcgcctgtcattatttagttactatacagatgacatgtgtgacaaaagttgaagttc

cctacctgtgctactgtgtttaaacttttaactatcttgcaacataagtccgcgtgatgt

tgattatctccacttatgatgtcgaaaatatatatggtgtgaccctgaatttggtggtat

gatcatgacaaaatctgacttttggcag

GTGATTTCAATAAAGGACATCATACGCCCAACAAGGCGGGAGAACTTCAACGATGTTTAC

ATTGTCTACGAACTGATGGACACTGATCTTCACCACCTTCTAAGATCAAACCAGCCACTC

ACAGATGATCACTGTCAG

gtatgtacattgttctttccatcatcccatcagcatcagcccaaacattccacaattggt

cagctttcctttcaccttaacattttcatgatgcag

TATTTTCTCTACCAAGTGCTCCGAGGATTGAAGTATGTGCATTCAGCAAAGGTCTTGCAC

CGGGACCTCAGGCCGAGCAACCTGCTGCTCAATGCCAAGTGTGAACTCAAGATTGGAGAT

TTTGGCTTGGCTAGGACCACCACTGAGACTGACTTCATGATGGAGTATGTTGTTACTCGG

TGGTACAGGGCGCCGGAGCTCCTGCTCAACTGCTCGGAGTATACTGCAGCAATTGATATC

TGGTCAGTGGGTTGCATCCTCGGTGAGATTGCTATGAGGGAGCCACTGTTTCCTGGAAAA

GATTATGTTCATCAGCTGAGGCTAATTACTGAG

gtatggccactcactactacacgagtcaagattttgttttttcccttctgtttctttttt

cttctttttgtatattctcctgctctctttttgcctcggagtctgacactatgattgtca

gttctgatttagcataggttccaactgctatgatttttccatgcttatatctcttggatt

actaactcaattagcacaagtacaccacataggttgaagcttacactggtatctggtgta

gcatgatacttcatgcatatgccttcattcagactaatgttttggtccaagcatgtgatt

attctagatgcactaacatctcttgttctactaaatggaaatgtctaatgaatcttcttg

acgctgtttgtaatcctgattcattcttgatccaaag

CTGATAGGCTCACCAGATGACACGAGCCTTGGGTTTCTTCGAAGTGATAATGCCCGCAGA

TACGTGAG

gtctcttcctcaatacccgaaacagnataggctcaccagatgacacgagccttgggtttc

ttcgaagtgataatgcccgcagatacgtgag

GTCTCTTCCTCAATACCCGAAACAGCATTTTGGTTCACGGTTCCCCAGTATGTCCACTGG

CGCCATGGATTTGCTTGAGAGGATGCTCGTATTTGATCCGAGCAAGAGGATTACTG

gtaatcttgcagcatagcgtgatcttgcttaattgcaaaatgacaaatttaataatgtga

accatcatttcagaatcgtgtgtgaatcattaaaaccaagttgaaatgacacttcttacg

ataattaaaccgttaatgtaagaaatattacgaggaactttctcttggaagtataacctt

tacttattttcttggtggcttcgcag

TTGATGAGGCTCTATGCCATCCTTATTTAGCATCCCTTCATGAGATAAATGATGAACCTG

TCTGCCCAGCGCCTTTCAGCTTCGACTTCGAGCAGCCATCATTTACTGAGGAAGATATCA

AAGAACTCATTTGGAGGGAGGCTCTCAAGTTCAACCCGGAACCAATTCACTGAAAAGTTC

CCAATGTAGAAAAAGAAGCAATTTCAAATGGCAAATCTATCAGCTCCGTGGGAAAATACA

TGAGCTGACAGCACGATGTACAGAGTAATGAGTGATCACGTCTCAGCATCTTGTCATACG

GCTTTGTTGATGACACCATGACTTCGAAAACCTGGAAATTTAGGAAACATGAACGCTTGA

TCAATAATTTGTTAGCCAAAAAAAGTGGGGCCATTATCTGTATCATGTCAGGTTGTATCT

ACCCCCTTTTTGTTGTATGCTTAACAAGACCTGAGTTATGGACTATGGTATTACAGCTTC

CGGCCACTCTCCGGTCGTCAGTGTTGTTCACCATCCAACAGATGTTACTTATTATTATTG

TGTTCTAAACCCTGCAATCAATATACATGTAGTCTATTGTGTCCCGGATACACCTAAGAA

AGTTGTCGATTGTGC

**>TaMPK11(7DL): TRIAE_CS42_7DL_TGACv1_603600_AA1986180.1**

CCAACCAACTCCTCGCCGGATCACGGGCATTTTCCCTCCAAAAAGCTTCCTCTCCTCCTT

TTTCCCCTTTTCCCCAACGCTCTTATCTCCTCCCTCGTCCATCCAACCAAGCTAGCAATC

TCTCTCTCTCTCTCTCTCTCATCCTCCCCGCGGCGTGCGTTTCTTTCTTTCTTGGCTAGC

TCCTCGATCAAGGTATTCGCGCGCAGGCGTGCGGGTCGATGCGCATGGAGGGTGGAGGTG

CCGGGGCAGGAGGAGGAGGCCACGGCGGCGGCCATGGCCTCGGCGGCGAGGCGCAGATCA

AGGGCACGCTCACCCACGGCGGCAGGTACGTGCAGTACAACGTCTACGGCAACCTCTTTG

AGGTCTCTGCTAAGTACGTCCCGCCCATCCGACCTGTCGGCCGCGGCGCCTGCGGCATCA

TCTG

gtacgtacgtcatgcgctgcatctgctcgcctatattcctcagttacctcagaatatgga

ttgctttgctcgatcagctaattgcgatcctgttgcgagttcactgtagcatatacgact

gaaagtgggtcttgattaaagctacagattaccatagcctagcgtatatagtattaaacc

ataaagctttctataatggaccagtactagttaattgttatggtatggctatatttaaca

gcaaaaatagcttctcaatactaaaaggaaacaagagctgtttcttgttgattttgagcc

tgtggtgacaaattattttctgtgatcttcttcttcag

TGCTGCTGTAAATGCACAGACTCGTGAGGAGGTCGCTATCAAGAAGATTGGTAATGCGTT

TGACAACCAGATCGATGCCAAACGCACTTTGCGAGAAGTAAAGCTGCTTCGCCACATGAA

TCATGAGAAT

gtgagccatctttccttctctcccatgtgccttcttgcgataaacctctgtggttcacat

gcttataattgaaatgttttgtgatttctatttgaccatggttaactctgtagtttatag

caaaggtaagccgtcctgaggagatctgccggaaattctggtgattttctttcaaagcca

gaatcgcctgtcattatttagttactatgcagacgacatgtgtgacaaaatttgaagttc

cctacctgtgctactgtgtttaaactttgaactatcttgcaacataagtccacatgatgt

gtgattatctccacgtatgatatcgaaaatatatatggtgtgaccctgaatttggtggta

tgatcatgacaaaatatgacttttggcag

GTGATTTCAATAAAGGACATCATACGCCCACCAAGGCGGGAGAACTTCAATGATGTTTAC

ATCGTCTACGAACTGATGGACACTGATCTTCACCACCTTCTAAGATCAAACCAGCCACTC

ACAGATGATCACTGTCAG

gtatgtacattgttctttccatcatcccatcagcatcagtccaaacattccacaattggt

cagctttcctttcaccttaacattttcatgatgcag

TATTTTCTCTACCAAGTGCTCCGAGGATTGAAGTATGTGCATTCAGCAAAGGTCTTGCAC

CGGGACCTCAGGCCGAGCAACCTGCTGCTCAATGCCAAGTGTGAACTCAAGATTGGAGAT

TTTGGCTTGGCTAGGACCACCACTGAGACTGACTTCATGATGGAGTATGTTGTTACTCGG

TGGTACAGGGCACCGGAGCTCCTGCTCAACTGCTCGGAGTACACTGCAGCAATTGATATC

TGGTCAGTGGGTTGCATCCTCGGTGAGATTGCTACGAGGGAGCCACTGTTTCCTGGAAAA

GATTATGTTCATCAGCTGAGGCTAATTACTGAG

gtatggccactcactactacatgagtcaagattttgttttttcccttctgtttctttttt

cttctttttgtatattctcctgctctctttttgcctcagagtctgacactgattgtcagt

tctgatttagcataggttccaactgctatgctttttccatgcttatatctcttggattac

caattcaattagcacaagtacaccacataggttgaagcttacactggtatctggtgtagc

atgatacttcatgcatatgccttcattcagactaatgttttggtccaagcatgtgattat

tctagatgcactaacatcttctcttgttctactaaatggaaatgtctaataaatcttctc

gatgctgtttgtaatcctgattcattcttgatccaaag

CTGATNATAGGCTCACCAGATGACACGAGCCTTGGGTTTCTTCGAAGTGATAATGCCCGC

AGATACGTGAGGTCTCTTCCTCAATACCCGAAACAGCATTTTGGTTCACGGTTCCCCAGT

ATGTCCACTGGCGCCATGGATTTGCTTGAGAGGATGCTCGTATTTGATCCGAGCAAGAGG

ATTACTG

gtaatcttgcagcatagcattatcttgcttgattgcaaaatgacaaatttaataatgtga

accatcatttcagaatcgtgtgcgaattattaaaaccaagttgaaatgacacttcttacg

ttaattaaaccattaatgtaagaaatattacgaggaactttctcttggaagtttaacctt

tatttttctttcttggtggcttcgcag

TTGATGAGGCTCTATGCCATCCTTATTTAGCATCCCTTCATGAGATAAATGATGAACCTG

TCTGCCCAGCGCCTTTCAGCTTCGACTTTGAGCAGCCATCATTTACTGAGGAAGATATTA

AAGAACTCATTTGGAGGGAGGCTCTGAAGTTCAACCCTGAATCAATTCACTGAAAAGTTC

CCAATGTAGAAAAGAAGCAATTTCAAATGGCAAATCTATCAGCTCCGTGGGAAAATACAT

GAGCTGACAGCACGATGTACAGAGTAATCAGTGATCACGTCTCAGCATCTTGTCATACGG

CTTGTTGATGACACCATGACGTCGAAAACCTGGAAATTTAAGAACCATGAATGCTTGATC

AATAATTTGTTAGCCAAAAAAAGTGGGGCCATTATCTGTATCATGTTAGGTTGTATCTAC

CCCCTTTTTGTTGTATGCTTAACAAGACCTGAGTTATGGACTATGGTATTACAGTTTCCG

GCCACTCTTCCGTTGTCAGTGTTGTTCACCATCCAACGGATGTTACTTATTATTATTGTG

TTCTAAACCCTGCAATCAATATACATGTAGTCTATTG

**>TaMPK14(6AS): TRIAE_CS42_6AS_TGACv1_487866_AA1573320.1.X (based on AA1573320.1)**

GCTCCCACGCGCCTAGCCGGGGACGGAGGAATATNNNNNNNNNNNNNNNNNNNNNNNNNN

NNNNNNNNTGCTCTCCCTTCCCCTCTCGT

gtaagtgagccttcctcccctcctcctcgcccttctctccgcaatccttgtgctgggcct

gcacggcccgagatcggcgggttcttgtccggacggactgcgccgctctccgtccgtcgg

agatcggtcgcccctgatccgagcagccccagctggcgagctcggattacgccgcctggt

gcgtgcgtgcgcgcgtgcctactcgagtgtcccgcccaaataattcgattccccgtacct

gacgcccgcccgtgctccggtgatgcatcatgcatcatgccatggtgctcgccagggtac

gcgatgactggccgggggcatttctgtgacccgctggtagggtggtaggctgcccggcga

atcatctgggcagacgatctggcgcgaccgggagctatggtttcttgcaggccatccgtc

cgccggtcgccgggactgcggattcggtttgtgcgtttagcactgaggctgatgagaatg

cgcgtgagaggtggtagtgagtggcattgcatggtggtgatgcacaaattggcagcgcta

gcgatcgaagttaccatttataagcttgtataaatggacattgaagctcatcaattcaag

agctgaattggtcagcgtcagtgtccatttataagcttagataagtacaactgcacttag

atgacatgccaacatgtactgcttatatatatatacttcattcactgttgccgactctga

tcagaatttatgagccttgtagctcgtggtgcatggtacagtttaggttcagttcagaat

tcttctacttggagttccacataaacacttaccaaaaaaagaaaaagtgttctattggca

tccgaggtcatgtaaatttgttaccaaagcctggtttagttggagactggtatactgtca

acctctcctcatcagcttaagcttttgggtgaactggttatggttaggtgcattcacttc

aacacggtatcagagtcaagtcaagggatcttgagtttaagacccaaacaacttaaatat

agattcagcctatcgatccttcgtctatggtccggaggttaggagagtgttcaaagatgg

gtggttagaaccacattgttacaacttgtattttataatgaagctgactttttcacattc

aaagtttggtgattgtgtaagctgaaagaaattaattttgcttctcgtactgttatttat

ggtatactaagttatgttttgttttgttag

TTGAAAATGGCAATGCTGGTGGATCCTCCGAATGGCATGGGAAACCAAGGGAAGCACTAT

TACTCAATGTGGCAAACCTTGTTTGAGATTGACACCAA

gtatgtgcctatcaaacccataggccgaggagcttatggaatagtttgctcatccataaa

ccgtgagacaaacgagaaagtagcgataaagaagatacataatgtattcgacaaccgtgt

ggatgcactaaggaccttgcgggagctgaannnnnnnnnnnnnnnnnnnnnnnnnnnnnn

nnnnnnnnnnnnnnnnnnnnnnnnnnnnnnnnnnnnnnnnnnnnnnnnnnnnnnnnnnnn

nnnnnnnnnnnnnnnnnnnnnnnnnnnnnnnnnnnnnnnnnnnnnnnnnnnnnnnnnnnn

nnnnnnnnnnnnnnnnnnnnnnnnnnnnctgaaactcctccggcatctccgccatgagaa

tgttatttctttgaaggatataatgatgcctgtacaaaggaggagctttaaggatgtgta

cttggtttatgagctcatggatactgacctgcatcag

ATAATCAAATCGCCTCAGGGGCTTTCCAACGACCACTGCCAATATTTTCTTTTTCAG

gtaatgctcggattacaccttttccgtcactcttccacatttaattatttgatagtagtg

agtatgtgtttctcatactccttattactttcactacctgcaccatttatgacaatacat

cttatatctgcacatgtacaatgttcgtaaggtcactcatattcatcaagcatgatacct

tagtctgcaccagattaattggtcgctgcaatacacaatgttaggtgtctttgtcgattc

gaacttgaaatacacactgttttgtaaacacttaaatgttagtcatcagtgtttatagac

gactggtgtgagtttgatgtcgtttcttaaccatcaagttgctgatcctatccatttatc

cctttagcccttcaaaatcatttgaaccatgaacttcttttcacatttttacactattct

tgaagtttgtgcccgctcggatgaaaactgtgaaacctaggtaaaatcgggaataattgt

gcaccttggcatatcgttgtcatcctgtgtgattctggtcagtttcacatgttccttcac

tcactgggttttgaaagaactggcaataactgaccacttatcttcgtttcaaagcccagg

ttgcttcctttagattgacagctggacagaaggtatcttctactagtaactttagaatgg

aaggcagatgcactctgtagctatatagacaacctcaaactgggtgacatagaatatatt

gttgttgttgtttttttaccttccctacttcgtttcggtacaatagcataattcatatgt

gatatgtgaatttttccttttctgcaagtcacatcaacctgtattgctctgacagtcaca

ttgtgaggccttgtcttgctgcctcatgaaataaataaataaataagaccaacacaggct

gatgtccagtgcaacctaaaactagcaccttctgtttagatgtttctgaattacagccag

acctaccaatccagctgatatattgaatatgactagttggacttcactttatcacttgtc

agatgtccgcatccaaataaagataacttcgagtgaattggggctttctggtaaaaagct

atacaaatgacattctttttacaatttcagcttttatgtacatatattaaactaaagaag

tatctgtatgactacatgtagtacaagctgttaaacttcatgcactagccaacgcaacca

aaagtctgaactgatggaaagggctaggcaatctatacacttcaacaccccctctcacgt

gtgtcgtgaaaagtccaacatgtggatagactcagaggtatggctcaagaggcctatacg

tagacaaaggggggcagctgcaatttttagatgaactgtgaaagccaggacttgaactca

agaccttagcaccgataccatgttaaacttcatgcactagccaacgcaaccaaaagtccg

aactgatggaaagggctaggcaatctacatatacacttcaacacaagcatccattctaaa

agaaccatctctaattataagcattcattctgaagaagtataagcatgctgatgaattta

cagttcatgttcccttgcacttcatttaacctgttgtcttaagatcctttgaattcattt

attcttttctatttttcctatttacattttttgttgataatttgagatgatgttgttttc

ttcattgcag

TTGCTTCGAGGACTGAAATACCTCCATTCAGCAGAGATACTCCACAGAGACCTAAAACCT

GGGAACCTACTGGTGAATGCAAACTGTGATCTGAAGATATGCGATTTTGGTCTTGCACGT

ACAAACAGTAGTAAAGGCCAGTTTATGACTGAATATGTCGTCACCCGCTGGTATAGGGCT

CCTGAGTTGCTGCTTTGCTGCGACAACTACGGCACTTCCATTGATGTTTGGTCTGTTGGC

TGCATCTTTGCTGAGCTACTTGGCCGCAAGCCTATTTTCCCCGGGACAGAGTGCCTAAAT

CAGCTAAAACTGATAGTCAATGTTCTTGGCACGATGAGCGAGTCTGACCTGGAGTTCATC

GACAACCCAAAGGCCCGCAGATATATCAAGACCCTCCCCTACACTCCTGGTGTTCCACTT

GCAAGTATGTACCCACATGCACACCCTCTGGCCATCGATCTATTACAGAAGATGCTCATC

TTCGACCCTACCAAAAGGGTCAGTGTTACCCAGGCCCTTGAGCACCCTTACATGTCTCCT

CTGTATGACCCAAGTGCAAACCCTCCCGCGCAAGTGCCCATCGATCTCGACATAGATGAG

AACATCAGCTCAGAGATGATCAGGGAAATGATGTGGCAGGAGATGCTTCACTACCACCCT

GAAGCCGCCACGGCAGTAAACATGTGACGACGATCTTGCAGTGCCCTGGGAAGAACCCGG

CAGGCTCACTCCTTTTTTCCTCGAAAAGACTACTGCGATTATCGCACCTATTAAGTAACC

ACGACGTGCAGTGTGGAGAGGTATCTCCGTGTAAATATGCAGTGCGATAAGAACCGCATA

TGGATAGTTCTTGTTATGGACCACTTTGGTGTATGTATACTGTTGTGTTGTTGTATAAGC

TCATGAAAGAACTGTTGAAGCGAATTCAGTAAGTTGTTGATTTGTGAT

**>TaMPK14(6BS): TRIAE_CS42_6BS_TGACv1_514340_AA1658600.1.X (based on AA1658600.1)**

NNNNNNNNNNNNNNNNNNNNNNNNNNNNNNNNNNNNNNNNNNNNNNNNNNNNNNNNNNNN

NNNNNNNNNNNNNNNNNNNAGCTGCAATTCACCTCCGAATTCGGTCGCCCATGGCGCCGC

GGCTGCTCTAATCCTACCCACCTACCCCTCCCCCCTCCTCTCCTCTACCAGCTGCTCTCC

CCTCCCCTCTCGT

gtaagtgagcatctctcccttctctccgcaatccttgtgcttggcctccgcggcccgaga

tcggcgggttcttgtccggacggactgcgccggtctccgtcccgcggagatcgggcgccc

ctgatccaagcagccccagctggcgagctcggattgcgccgccgggtgcgtgcctacgtg

cgtgcctagtcgagtgtcccgcccaaataattcgattcccggtaccgcgcgcacgcctgt

gctccggctgaccggtgatgcatcgggccatgctgttcacgggggtgtgcgatgattgtc

tgggggcatttcttgccctgataatcatctgggcgacgatctggcgaccggagctatggg

tttcttgcacgccatccgtctgcccgtcgctgggactgcggattcgatttgtgcgtttag

cactgaggctgatgagaatgcgcgtgagaggtggcagtgagtagcattgcatggtggtga

tgcgcggattggcagcgctagcgatcaaagttaccatttataagcttatataaatggaca

ttgaagctgatcatttcaagagctgaattggtcagcgtccatgcccatttataagcttag

ataagtacaactgcacttagatgacatgcgaacatgtactgcttatatatagttcattca

ctgttgcctattctgatcagaatttatgagccttgtagctcgtggtgcatggtacagttt

aggttcagttctgaattcttcttctacttggagttccacataaacacttagtaccaaaaa

aaaagaagattgtgtgttctattggcatctgaggtcatgtaaatttgttaccaaagcctg

gtttagttggagaattgtatactgtcaacctctcctcatcagcttaagcttttgggtgaa

ctggttatggttaggtgcatgcacttcaatacggtatcagagtcaagtcaagggatcttg

agtttaagacccaaacaacgtaaatatagattcagctattgatcctaaggtccggaggtt

aggagagtgttcaaagatgggtggttagaaccacattgttacaactagtattttataatg

aagctgacttaatttttcagattcaaaatttggtgattgtgtaagctcaaagaaattaaa

tttgcttattgtactgttatttatggtatactaagttatgttttgttttgttag

TTGAAAATGGCAATGCTGGTGGATCCTCCGAATGGCATGGGAAACCAAGGGAAGCACTAC

TACTCAATGTGGCAAACCTTGTTTGAGATTGACACCAAGTATGTGCCTATCAAGCCCATC

GGCCGAGGAGCTTATGGAATAGTTTGCTCATCCATAAACCGTGAGACAAACGAGAAAGTA

GCGATAAAGAAGATACATAATGTATTCGACAACCGTGTGGATGCACTAAGGACCTTGCGG

GAGCTGAANTATTCGACAACCGTGTGGATGCACTAAGGACCTTGCGGGAGCTGAAACTCC

TCCGGCATCTCCGCCATGAGAATGTTATTTCTTTGAAGGATATAATGATGCCTGTACAAA

GGAGGAGCTTTAAGGATGTGTACTTGGTTTATGAGCTCATGGATACTGACCTGCATCAGA

TAATCAAATCGCCTCAGGGGCTTTCCAATGACCACTGCCAATATTTTCTTTTTCAG

gtaatgctcggatcacaccttttccatcgctctttgacatttaattatttaattgtagtg

agtatgtatcttctccttattactttcactacctgcaccatttgtgataatacatcttat

atctgcatgtgtacattacccattatattcgcaagttcactctagtattcatcaaggatg

gtactttagtctgtaccagattaattggtcgctgcaatacacactgttaggtgtctttgt

cgattcgaacttgaaatacacacactgttttgtaaacacttaaatgttactcaatccaac

catgaacttcttttcacatttttacacaatattcttgaagtttgtgccagctcgaatgaa

aacctaggtaaaattgggaacagaagtgcaacttggcatgtcattgtcatcctgtgtgat

tctggtcagtttcacatggtccttcactcaatgggttttgaaagaactggcaataactgg

ccacttatctttgtttcgaagcccaggttgcagccctttagattgacagctggacagaag

gtatcttctagtaactttagagtggaaggcagcttcactctgtagctatatagacaactt

cgaattggatggcatggaatatattgttgggttcttcaccttttctattacgctttggta

tttatttttaaacgttagacacaatagcatagttcatacgcgatatgtgaattttccttt

tctgcatcaacctgtattactctcagtcacattgcgaggcattgtcttgctgcctcatga

aataaataaaaaagaccaacacaggctcatgtccagatgcaacctaaaattagcaccttc

tgtttggatgtttctaattacaacttattttgttaacatggtaacagccagacttaccaa

tccagctgatatattgaatatgactacttggacttcactttgtcacttgtcagacctccg

catccaatgaattggggctttctaatattttttttataaaatgacattctttttagagtt

taagcttttatgtacatacattaaactaaaaaagtatctctaccactacatgaagtataa

gcatgcattctaacagaactatgtctctacgtaaaagcattcattctaaagaagtaaaag

cattcattctacacaagtataagcatgctgatgaatttttaatttatgtgacccttgtgc

ttcatttaacctgttattgtaagatcttttgaattcttttttattcttcctatctacatt

ttctgttgatgatttaagatgttgttattttgattgcag

TTGCTTCGAGGACTGAAATACCTCCATTCAGCAGAGATACTCCACAGAGACCTAAAACCT

GGGAACCTACTGGTGAATGCAAACTGTGATCTGAAGATATGTGATTTTGGTCTTGCACGT

ACAAACAGTAGTAAAGGCCAGTTTATGACTGAATACGTCGTCACCCGCTGGTACAGGGCT

CCTGAGTTGCTGCTTTGCTGTGACAACTACGGCACTTCCATCGATGTTTGGTCTGTTGGC

TGCATCTTTGCTGAGCTACTTGGCCGCAAGCCTATTTTTCCTGGGACAGAGTGCCTCAAT

CAGCTAAAACTGATAGTCAATGTTCTTGGCACCATGAGCGAGTCTGACCTGGAGTTCATC

GACAACCCAAAGGCTCGCAGATATATCAAGACCCTTCCCTACACTCCCGGTGTTCCACTC

GCAAGTATGTACCCACATGCACATCCTCTGGCCATCGATCTATTACAGAAGATGCTCATC

TTCGATCCTACTAAAAGGATCAGTGTTACCCAGGCCCTTGAACACCCTTACATGTCTCCT

CTTTATGACCCAAGTGCAAACCCTCCCGCGCAAGTGCCCATCGATCTTGACATAGATGAG

AACATCAGCTCAGAGATGATCAGGGAAATGATGTGGCAGGAGATGCTTCACTACCACCCT

GAAGCCGCCACGGCAGTAAACATGTGATGACGATCTTGCAGTGCCCTGGGAAGAACCCGG

CAGGCTCATCTCTTTTTTCCTCGAAAAGACTACTGTGATTATCGCACCTATTAAGTAACC

ATGACGTGCAGTGTGGGGAGTTATCTCCGTGTAAATACGCAGTGCGATGAGAACCGCATA

TGGATAGTTCTTGTTATGGACCACTATTTGGTGTATGTATACTGTTGTGTTGTTGTATGA

GCTCAAGAAAGAACTGTTGAAGCGAATTCAGTAAGTGTTGGTTTGTGATGGCCCTCCTGT

GTTTGTTGTTGCAGTTGTTGGTATTTGAAGCAGACCTGCTGTTATTCGCTTGTGTGTTTT

ATTTAGTACTATTAATCGGCAAATATTGCACTAGTAAAAATGCATGTTAACCTTG

**>TaMPK14(6DS): TRIAE_CS42_6DS_TGACv1_544171_AA1747030.1**

GCATCCTCCTCCCTCCCCCAGCTGCAATTCACCNNNNNNNNNNNNNNNNNNNNNNNNNNN

NNNNNNNNNNNNNNNNNNNNNNNNNNNNNNNNNNNNNNNNNNNNNNNNNNNNNNNNNNNN

NNNNNNNNNNNNNNNNNNNNNNNNNNNNNNNNNNNNNNNNNNNNNNNNNNNNNNNNNNNN

NNNNNNNNNNNNNNNNNTCCCTCCCCCTCTCAT

gtaagtgagccttcctcccctcctcctcgcccttctctccgcaatccttgtgctgggcct

gcaccgcccgagatcggcgggttcttgtccggacggactgcgccggtctccgtcccgcgg

agatcggccgcccctgatcccagcagccccagctggcgagctcggattgcgccgccgagc

gcctgcgtgcctagtcgagtgtcccgcccaaataattcgattccccgtaccgcccgcccg

ctcgtgctccggtgatgcatcgtgccatggtgctcgccggggtgcgcgatgactggacgg

gggcatttctgcgacccgctggtagggtggtaggctgcccagcgaatcatctgggcagac

gatctggcgcgaccgggagctatggtttcttgcaggccatccgtccgccggtcgccggga

ctgcgggttcggtttgtgcgtttagcattgaggctgatgagaatgcgcgtgagaggtggt

agtgagtagcattgcatggtggtgatgcacagattggcagcgctagcgatcaaagttacc

gtttataagcttatataaatgggcattgaagctgatcaattcaagagctggattggtcag

tgtcaatgtccatttataagcaagtacaactgcacttagatgacatgcgaacatgtactg

cttatatatatagttcattcactgttgccgattctgatcagaatttatgagccttgcagc

tcgtggtgcatggtacagtttaggttcagttcagaattcttctacttggagttccacata

aacacttaccaaaaaagaagagtgggtgttctattggcatctgaggtcatgtaaatttat

taccaaagcctcttgtagctggagatttgtttattgtccatgtctcctcatcagcttaag

cttttgggtgaactggttaggcgcatgcaattcaatatggtattagagtcaaggggtctc

gagttcaatacttggccaacatactaacaataaatatacagcagcctagatcgatcccag

gtctaaggtctggaggctaggaaagtgttcaaagatggggacattgttacaacttgtatt

ttataatgagtctgtcttatttttcacatttgaaatttggtgattgtgtaagctaagaga

aactaaatttgcttctaatactgttatttatggtatactaagttatgttttgttttgtta

g

TCGAAAATGGCAATGCTGGTGGATCCTCCGAATGGCATGGGAAACCAAGGGAAGCACTAC

TACTCAATGTGGCAAACCTTGTTTGAGATTGACACCAAGTATGTGCCTATCAAGCCCATT

GGCCGAGGAGCTTATGGAATAGTTTGCTCATCCATAAACCGTGAGACAAACGAGAAAGTA

GCGATAAAGAAGATACATAATGTATTCGACAACCGTGTGGATGCACTAAGGACCTTGCGG

GAGCTGAANCTGAAACTCCTCCGGCATCTCCGCCATGAGAATGTTATTTCTTTGAAGGAT

ATAATGATGCCTGTACAAAGGAGGAGCTTTAAGGATGTGTACTTGGTTTATGAGCTCATG

GATACTGACCTGCATCAGATAATCAAATCGCCTCAGGGGCTTTCCAACGACCACTGCCAA

TATTTTCTTTTTCAG

gtaatgctcggatcacaccttttccatcgctcttcgacatttaattatttaatagtagtg

agtatgtatcttctccttattactttcactacctgcaccatttgtgataatacatcttat

atctgcatgtgtagtacactacccattcttatattcgcaaggtcactctaattttcatca

aggatggtaccttagtctgtacaaaattaattggtcgctgcaatacacactgttgggtgt

ctttgtcgattcgaacttgaaatacacacacttttgtaaacacttaaatgttagttaatc

cgaccatgaacttcttttcacatttttacacaagattcttgaagtttgtgccagctcgaa

tgaaaactgtgaaacctaggtaaaatcgggaacagtagtgcaacttggcatatcggtgtc

atcctgtgtgatctggtcagtttcacatgttccttcactcaatgggttttgaaagaactg

gcaataactggccacttatcttcgtttcaaagcccaggttgcagccctttagattgacag

ctggacagaaggtatcttctagtaactttagagtggaaggcagcttcactctgtagctat

atagacaacttcaaattggatggcatggaatatattgttgggtttttcaccttttctact

tccctttggtatttttttaatcgttagacacaatagcatagttcatatgcgatatgtgaa

ttttcctttcctgaaagtcacatcaacctgtactctcacattcacattgcgaggcattgt

cttgctgcttcatgaaataaataaaaaagaccaacacaggctcatgtccagtgcaaccta

aaactagcaccttctgtttagatgtttctaattacaaattattttgttaacatagtaaca

gccagacctaccaatccagctgatatattgaatatgactacttggacttcactttctcgc

ttgtcagaccaccgcatccaatgaattggggctttctgattttttttaataaaatgacat

tctttttagagtttcagcttttatgtacatacattaaactaaaaaagtatctctaccact

acatgaagtataagcatgcattctaacagaactacatgtctctacgtaaaggcattcatt

ctaaagaagtataagcattcattctacacaagtatactgatgaattttcaatttatgtta

cccttgtgcttcatttaacctgttattgtaagctcttttgaattcttttttattcttcct

atctacattttctgttgatgatttaagatgttgttattttgattgcag

TTGCTTCGAGGACTGAAATACCTCCATTCAGCAGAGATACTCCACAGAGACCTAAAACCT

GGGAACCTACTGGTGAATGCAAACTGTGATCTGAAGATATGTGATTTTGGTCTTGCACGT

ACAAACAGTAGTAAAGGCCAGTTTATGACTGAATACGTCGTCACCCGCTGGTATAGGGCT

CCTGAATTGCTGCTTTGCTGTGACAACTACGGCACTTCCATCGATGTTTGGTCTGTTGGC

TGCATCTTTGCTGAGCTACTTGGCCGCAAGCCTATTTTTCCTGGGACAGAGTGCCTGAAT

CAGCTAAAACTGATAGTCAATGTTCTTGGCACCATGAGCGAGTCTGACCTGGAGTTCATC

GACAACCCGAAGGCTCGCAGATATATCAAGACCCTTCCCTACACTCCCGGTGTTCCACTC

GCAAGCATGTACCCACATGCACATCCTCTAGCCATCGATCTATTACAGAAGATGCTCATC

TTCGATCCTACCAAAAGGATCAGTGTTACCCAGGCCCTTGAGCACCCTTACATGTCTCCT

CTGTATGACCCAAGTGCAAACCCTCCCGCACAAGTGCCCATCGATCTCGACATAGATGAG

AACATCAGCTCAGAGATGATCAGGGAAATGATGTGGCAGGAGATGCTTCACTACCATCCT

GAAGCCGCAGCTGCAGTAAACATGTGATGATGTTCTTGCAGTGCCCCGGGAAGAACCCGG

CAGGCTCACTACTTTTTTCCTCGAAAAGACTACTGCGATTATCGCACCTATTAAGTAACC

ACGACGTGCAGTGTGGAGAGCTATCTCCGTGTAAATATGCAGTGCGATAAGAACCGCATG

GATAGTTCTTGTTATGGACCACTATTCGGTGTATGTATACTGTTGTGTTGTTGTATGAGC

TCATGAAAGAACTGTTGAAGCGAATTCAGTAAGTTGTTGATTTGTGATGGCCCTCCTGTG

TTTGTTGTTGCAGTTGTTGGTATTTGAGGCAGACCTGCTGTTATTCTCTTGTGTTTATTA

CCAGTATTAGTACGACCTATGTAATATTATTCTTACCTTTTAACCGCCACCAAGATAATA

TCTGCAAATATTGCACTGGTAAAAATACATGCTGTTATCCTTCGTGATA

**>TaMPK16(1AS): TRIAE_CS42_1AS_TGACv1_020601_AA0078640.1**

GGCTTCTCTCTCCTGACTGCCCAGCTACCACCGCCCCCCTCCCCCCTCTGTCCTCGAGCT

TCGCCTTGGATCGCAGCGCAGCTGGACTCCTCCCCTCGGCCGCGTCCCCCTCGTCGTGTG

CGCGCGTGAGCGA

gtgagctccctccccgccctgcttctcccccccctcccccctctctcttgctggatcggg

ggtgctcaaagatctcgcctttctcgcgcaaaagatcccctctcctccggggagagatct

gccgtgaggaggcggcaggggcgggcgaagaaggccgcgcccctctctctgttccgctga

gcgttcctgccgcccccaccctctagtgccgtgcggggagttagtagcagccttctttct

tttcttggagtggattggtagtgttcttggttcgggatcttggtgcgtttggagcttttg

ctccccgaatcgcgaagccccgcggtcctggtggtaggcttgttccccgccgtaaaacct

ggcggctagacggatctactcgatttggagttgagtcgctttacagattcgtgggcgtaa

gctgctcgtacgttttggtggaaagaattgacgtcggctgcttggtttcgtaggtctcgt

cagttcctcttgtaaattcatgcccggcagtaatggcgctactgttcatttattcacaac

cctctcagttcgagctatggacttgacttgagtagttgtgctgtcgtgttcatatttcta

cccacctctctgtgcggcaatatgcatgcttcatgccatcccaacaattccatcgtttgg

gcaaaaagtttcaacttgttttggagaacgaaagtaatgccccctagacatcgttgcatc

acgtggttctagtgcttttcttttgcttccaagcagcttttttcagataaaatttgttac

ttgacgattgagaccgtgctagtttgcgttgtatgaattctccatggaacttaatttcct

atgtggtcattttcgcgtaatacttggctagtgtctttttgtacatagtcctcgtcgctt

gccaaattaggactctaatatctattttgagtgtcaccatctcaccatgtcatactaaag

aacgcaattcgtcagtgctcaaggtgactcgtattctgttttgtgtgacacag

ACATCTCAAATGGACTTTTTTACCGAGTATGGTGAGGGAAACAGATACAAGATTGAAGAG

GTTATAGGGAAAGGGAGTTATGGCGTGGTCTGCTCTGCTTTGGATACTCACACGGGTGAG

AAAGTTGCTATCAAGAAGATAAACGACATCTTTGAACATGTGTCTGATGCCACACGGATA

CTCCGCGAGATCAAGTTGCTTAGGCTCCTAAGACATCCCGATATCGTGGAAATTAAACAT

ATTCTTCTTCCTCCGTCGAGGAGAGAGTTCAAGGATATATACGTTGTTTTCGAACTCATG

GAGTCTGATTTGCACCAAGTTATAAAGGCGAATGATGACTTGACTCCAGAGCATTATCAG

TTTTTCTTGTATCAGTTGCTCCGAGGATTGAAATACATACATACAG

gtaaccgatttattttcgcatcaaagatggctcttttgcatattacaacagctggatatc

aggcatctcaatttactaataaatggtcaatgtgtttaaaaggcatacacatgtcagtag

ataacaatgccatgtcaaattcgaaacaatatttctagtatctggcgacgatgtattggt

tgtgttggtatttgtgaaaacctttaaaacccatggtatgttccacaaactttgtagtca

ggagagcaatatggacctgtatagtactttgatacaggtcacacagttcgctatagtgtc

acagcctgagttgtgtgttctagtctaatcacctagtaactgatcaattctgtgatggaa

agttactgttaatcatttacgtagctacaatctggacgtgctcccaaagcttcttgtgat

gctacaacagaactgtggtcctagtgaggagctctttgtgccaaaagtcagcaactagat

aggcatactaatcttgtgaagcactgtttgaggctagagaacttctgtttaatctttttt

ataaatataaataacttatttgtgatcaagtaggtattttcagttctcttttttggcatg

ctaagaaataatattttgctggtataataacaatgaaaaacacatgctcatctctccatt

catagagcaatactgtagaaatccctattgttcagattatttaaacaaatgagtattaat

acttttgctatagatacaaatgtggcattgtgagttgattggcaataatataaaacaaga

ctcatagaaaaatcattttggctgatgcatcttccaatagcttcttgcag

CAAATGTATTTCATCGAGATCTCAAACCAAAGAATATCTTGGCTAATGCTGATTGTAAGC

TCAAAATATGTGATTTCGGTCTTGCAAGGGTAGCTATAAGTGATACTCCAACCGCAATAT

TTTGGACG

gtattgctatttccccctaccatgctgaccatttcttttggctactcaatatcaataaca

gcagctaatttcttatcctgtttttag

GATTATATCGCAACAAGGTGGTACCGAGCACCTGAACTATGTGGATCTTTTTTCTCCAAG

gtttgtatatgttaaatttcatatgaatcatggtatcaacagtatttttcattatttttc

cctcatatgaatctgatttacttcgtttaatgtttaaggctttaagggtgctgcaacttt

gtgacagcaaagcaaagtaacctccccctggcatttccttagtagttcttttcctatttc

atatcaggttatgttctaatatatattagctatgggaaacaatgccaatcagccagcatg

gttaaaaaataaatgtttaagaacacattccaacatctggcacagtttatgttttttttt

tgtcttccaccctcaacggacaaatattttttaatgtgcatgttttgatgttcagggtaa

tcatattgcttcaatgtcattcaggaacgaccttgttttctgtatttctttttgcaaacc

gaatgattaagctgtcaaaggaataagggcacctctagttgtaactcaagtactctatgt

ctaggtttgattaatccagctatacatattctggaatatctggaacgctgtataacatta

tgttcttcaatttcttgcag

TACACACCAGCAATAGATATATGGAGTATTGGATGTATATTTGCGGAACTTCTAACTGGC

AAACCTCTTTTTCCTGGGAAAAATGTGGTGCATCAACTTGATATAATCACAGATCTCCTG

GGAACGCCTGCTCCAGAAACAATTGCTAGG

gtcagtatggttggcataaaacagtgtgttgtttccaatataacctctgatgtttatagc

tatcctacag

ATTCGAAATGAGAAGGCCAGGCGCTACTTGAGCAGTATGAGGCGGAAAAAGCCTGTACCG

TTCACGCATAAGTTTCCAAATGCAGATCCACTTGCATTAAATTTGTTAGAGAGAATGCTA

GCATTTGATCCAAAAGACAGGCCAAGTGCTGAAGAG

gtagtttgttttagtaccatgcctgccagcattttttcctgtgtttcttctttccttgta

accttgaataccattagctaatgtattaggcatgttgacag

GCTCTTGCTGATCTTTATTTCAAGAACATTGCGAGTGTGGATAGGGAGCCTTCTGCACAG

CCCATTACTAAGCTTGAGTTCGAGTTTGAGAGACGAAGAATTACGAAGGACGACATAAGG

GAACTCATATACAGAGAAATTCTGGAATATCATCCAAACATGCTGAGGGAATTCCTTGAG

GGGGCTGAGCCAACTAATTTCATGTACCCAAG

gtgcgaagttctctttcaatactttaacatcgtggagtgccttgtttaattccagagaca

atgcagtgacattattcttggtcagtctagttctacagattttttttggtatcattaatg

tctgtgcaggttcatgaataaatatcaagtgtttatacacacactattatggttattttt

ctactcttttggtgatggtgatattgataaataaagcatgcggaaattgtttttgtttat

tgttgattaggtagtgcatacctattctgtgttcttatttgttgcttggagtcagtcgcg

cactgcataagatactgaccgattcatcgatctaacattgtttgctctattcggatttca

g

TGCAGTAGATCATTTCAAAAAGCAATTCACATTCCTTGAAGAGCATTATGCAAAGGGATC

AACAGCAGCACCGCCTGAGAGGCAACATAATTCATTACCAAG

gtatgatattgactttgctttaacattgtatgatggactgatgtcactttagccacaaat

tggaagtgtggttcttgactatctagttttctgttaacag

GCCGAGTGTTATCTATTCGGATAACCGGCCACAGTGTTCAGATAGCCGACCACAGGGTGC

TGCCAACATTACGGATGATCTTTCCAGGTGTATAATCAGAGATAATGCACAAAAGCCACG

CAGAGATCCTGCTTCAGTTGGTGCAAACAGAGTTCCTCAAG

gtaagcacctgggtcgtccaatacttttgaatctgcaaacaagcctgcatgactgtatcc

ttaactgtatctatcgggccttgtaactcgagggtaacttgatacaccacttcatcatac

ag

GTGCTGCCGTTGCAAGGCCTGGTAAAGTGGTTGGTTCGGCACTTCGTTATGGTAACTGTT

CAACATCTGGTACTGAGCAATATGAACAGCGAAGGGTCGTCACAAGCCAAGGAATTGTTC

CAAACGGTGTTCCTTCGGGCAGCTCATACCCTAGAAGAAATAACACCTGCAAGAGCGAAA

CAGGTGAAGCTGAAAGGATTGACGTGAGCCAAGCTGGGCCACCAAAGCCATATACCGGAA

ATAAACTAGCTGCAACCGTGGATGGCCGCAACGGGCACTGGTAGGCTGCTGTGGCAATGC

CAGAAAATCCTTGCCATTGGATCAGGAAGGGTTACTCATTCCAAGGACACACCTTCAAAC

ATGCCATTCTGTACAGAGTTATATTATCTGTCATGCTTACTTCTCCCGGAGACGATAAAA

CTGTATGTGAAGTTGTTGCGGCAGGCTAGTCTCCTGTTAAGCTCAACTCTGGTCTGGTTA

CCTGCAATTGGGTAGATCCAGTTCAACTGTTCAACAAGTATAACCAAAAAACTGGAAACT

TTGAGTGGCATTCGCGACAAAGGACTGTTACTGACCTGACTGATGGTGTTCCGCAATTCG

TTTCTCCAAGCTGGTGACCCTGAGGGAGGTTCAGAGAGCCCACTGTACACTATCTAATGT

TGTAACACGGAGTTTAATTAATTGACTTTAAAGTATGTTCTTGTTGCTTGTAGTCTGTCA

TCCAGCTGTAGTGTCGAACTATTGAACACTGCTATTTCTGATAACACATTATTTGGACCC

TGCTCGCTATTGAAACCCCAGGTTTTTTGTGCGGAATTTGTAATGTGATATGCAGTAAGT

TAGTAGCCCGAGCAAAACGGAGTTGCGACGTATGTTTCCTGAATTGCGCAACATGTTTAC

TGTCCTGTTTTTCAAGAAATTACATAGTGCCTG

**>TaMPK16(1BS): TRIAE_CS42_1BS_TGACv1_049733_AA0160590.2**

CCGGAAATCTAGTTAAACAAGGAGAGAAATAAATCTCGGGTATAAATTCCGAAAGCGGAG

GCCACCGGTGGCCTTCTCTCTCCTGACCTCCCAGCTACCACCGCCCCCCTCCCCTCTGTC

CTCGAGCTTCGCCTTGGATCGCAGCGCAGCGCAGCTGGACTCCTCCCCCTCGTCGTCGTG

TGCGCGCGTGAGCGA

gtgagctcccttccctcccctccccgccctgcttctcccccgtcccccctctctcttgct

ggatcggggctgctcaaagatctcgcctttctcgcgcaaagatcccctctcctctcccct

cctcctgggagagatctgccgcgaggagtgcggcaggggcgggcgaacaaggccgcgccc

ctctctctgttccgcggagactttccgccgtccccgtgctgggacttggcggcgttcttt

cttttcttggagtagattggtagtgtgttcttcgctcgagatcttggcgagtttggagct

tctgtttgcccaaccgcgaagctgcggtgttctggtattaggtctgttttgtcccacgct

gtgaaacttggcggccaaacggatccactcgattcggagtagctttgctttacagattcg

cgagcgtaataagatgccagcagtacgttttggtgcaaagaattggcgccggctgttttg

ctcctgtaggccttgtcggctcctcttttctaaatcgatgacgctcggtagtaatggcgc

tactgttcattcactgatgatgccctgctacagggtttcttttgagtcacctcagttcga

gccatgaaatgaacccaacttggttacttgagtcgttgctgttgtgtttacatttatttt

ttttacctgcctctcagtgcggcaatatgcatgcttcatgtcaatcccaattcggcaaaa

gtttcatcgtgtattggagaacgaaagtaatgaatgccccctagtccatcattgcaccat

gtggttctagtgctgttcttttgcttccaagcaacttttcagataaaatttattacttga

tgtttagatcatgctagtttgccttgtataaattctccatggaacttaattttccatgtg

gtcattttcgcatattatttggctagtgccttttgtacatagtcctcgtcgcttgccaaa

ttatgactctaatatctattttgagtgtcaccatctcaccatgtcatactaaagaacaca

attcgtcagtgctcaagctgactcgtattctgtttcttgtgacacag

ACATCTCAAATGGACTTTTTTACCGAGTATGGCGAGGGAAACAGATACAAGATAGAAGAG

GTTATAGGAAAAGGGAGTTATGGCGTGGTCTGCTCTGCTTTGGATACTCACACGGGTGAG

AAAGTTGCTATCAAGAAGATAAACGACATCTTTGAACATGTGTCTGATGCGACACGGATA

CTCCGCGAGATCAAGTTGCTTAGGCTCCTAAGACACCCTGATATTGTGGAAATAAAACAT

ATTCTACTTCCTCCGTCGAGGAGAGAGTTCAAGGATATATACGTTGTTTTCGAACTCATG

GAGTCTGATTTGCACCAAGTTATAAAGGCGAATGATGACTTGACTCCAGAACATTATCAG

TTTTTCTTGTATCAGTTGCTCCGAGGATTGAAATACATACATACAG

gtaaccgatttattttcgcatcaaagatggctcttctgcatattccaacagctggatgtc

aggcatttcaatttactaataattgtagcagccataaatggtcaatgtgtttaaaagtga

tacacatgtcagtagataacaatgccatgtcaaattcgaaactatatttctagtatctgg

tgacgatgtattggtcgtgttggtatttgtgaaaacctttaaaacccatggtatgtttca

caaactttgtagtcaagagagcaatacgggtcacacagttcgctatagtgctcagagaac

tctggaaatccaggtcttatttgtgatttgttgaatttgtgctagcttgtcacagcctga

gttgtgtgttctagtctaatcacctagtaactgaccaatgctgtgatgaaaagttactgt

taatcatttacgtagctacaatctggacgtgctcccaaagcttcttgtgatgctacaaca

gaactgtggtcctagtgaggatgctattggtgctgctctttgtgccaaaagtcagtgact

agataggcatactaatcttgtgaagcactgtttgaggctagagaacttctgtttaaactt

ttttataaatataaataacttatttgtgatcaagtagttattttcagttctctttttatg

gcatgctaagaaataatattttgctggtataataacaatgaaaaacgcatgcgtcatctc

tccattcaaagagcaatactgtagaaatccctatttttcagattatttaaacagatgaga

attaatacttttgctatagatacaaatgtggcattatgagttgattggcaataatataaa

acaagactcatagaaaactcattttggctgatgcatcttccaatagcttcttgcag

CAAATGTATTTCATCGAGATCTCAAACCAAAGAATATCTTGGCTAATGCTGATTGTAAGC

TCAAAATATGTGACTTCGGTCTTGCAAGGGTAGCTATAAGTGATACTCCAACCGCCATAT

TTTGGACG

gtattgcaatttccccctaccatgctgaccatttcttttggctactcaatatcaataaca

gcagctaatttcttatcctgtttctag

GATTATATCGCAACAAGGTGGTACCGAGCACCTGAGCTATGTGGATCTTTTTTCTCCAAG

gtttgtatatgttaaatttcatatgaatcatggtatcgacagtatttttcattatttttc

cctcatgaatctgatttacttcgtttactgtttaaggctttaagggtgctgcaactttgt

gacagcaaagcaaagtaacctccccctgtcatttccttagtagttcttttcctatttcgt

aacaggttatgttctaagggcctctttgattcaaaggattttcataggattttttaagga

ttcgaacccttaggaatttttcccgcattgatcgtttgattcacaggattgaatcccata

ggattttttccaatggattcatttgtactacatttcataggaattccagcatccactcca

acctcttggaagaaatcctttgtttttcctgtgatacaatcaaacaaactcaaatcctat

agaaatccaatgtgcatgtcatttcaatcctacatttttcctattcatgtgtttttgcaa

tcctgcgaatcaaagaggccctaatatgtattagctatgggaaacaatgccaatcagcca

gcatggttaaaaaataaatgtttaagaacacattccaacatctggcacagttcatgtttc

tttttgtctttccaccctcaacggatcaaatatttcttaatgtgcatattgtttttatgt

tcagggtaatcatattgcttcaatgtcattcaggaacgaccttgttttctgtatttcttt

tcgcaaaccgaatgcttgagctgtcaaaggaataagggcacctctagttgtaactcaagt

actctatgtctaggtttgattagtccagctaaacatattctggaatatcttgagtgctgt

ataacattatgttcttcaatatcttgcag

TACACACCAGCAATAGATATATGGAGTATTGGATGTATATTTGCGGAACTTCTAACTGGC

AAACCTCTTTTTCCTGGGAAAAATGTGGTGCATCAACTTGATATAATCACAGATCTACTG

GGAACGCCTGCTCCAGAAACAATTGCTAGG

gtcagtatggttggcataaataagcgtgttgtttccaatacaatcctctgatgtttgtag

ctatcctacag

ATTCGAAATGAGAAGGCCAGACGCTACTTGAGCAGTATGAGGCGGAAAAAGCCTGTACCG

TTTACACAGAAGTTTCCGAATGCAGATCCACTTGCATTAAATTTGTTAGAGAGAATGCTA

GCATTTGATCCAAAATACCGGCCAAGTGCTGAAGAG

gtagcttgttctagtaccatgtctcccagcaatttttcctgtgtttcttctttccttata

accttgagtaccattagctcatgtattaggcatgttgacag

GCTCTTGCTGATCTTTATTTCAAGAACATAGCTAGTGTGGATAGGGAGCCTTCTGCACAG

CCCATTACTAAGCTCGAGTTTGAGTTTGAGAGACGAAGAATTACGAAGGACGACATAAGG

GAACTCATATACAGAGAAATTCTGGAATATCATCCAAACATGCTGAGGGAATTCCTTGAG

GGGGCAGAGCCAACTAATTTCATGTACCCAAG

gtgcgaagttctctttcaatactttaacatccttaactggagtggagtgccttgtttaat

tccagagacaatgtagtggcattattcttgtgggtcagtctagttctacattttttttgt

atcaataatgtctgtgcaggttcatgaataaatatcaagtgttatacccacactattatg

gtttttttctgctgttttggtgatattgctgaataaagcatgcgaaaattgtttttgttt

attgttgattaggtagtgcatacctattctctgtgttcttatttgttgcttggagtcagt

cgcgcactgcataagatactgaccgattcatcgatctaacatcgtttgctctatttggat

ttcag

TGCAGTAGATCATTTCAAAAAGCAATTCACATTCCTTGAAGAGCATTATGCAAAGGGATC

AACAGCAGCGCCGCCTGAGAGGCAACATAATTCATTACCAAG

gtatgatattgactttgctttaacattgtatgatggagtgatgtcacttctagacacaaa

ttggaagtgtggttcttgactatctattttctgttaacag

GCCGAGTGTTATCTATTCGGATAATCGGCCACAGGGTTCAGATAGCCGACCACAGGGTGC

TGCCAACATTACGGATGATCTTTCCAGGTGTATAATCAGAGATAATGCACAAAAGCCACG

CAGAGATCCTGCTTCAGTTGGTGCAAACAGAGTTCCTAAGG

gtaagcacctgggccgtccaatacttttgaatctgcaaacaagcctgcatgactgtatcc

ttaactgtatctatcgggcctcgtaactcgagactaacttgatacgccacttcatcatac

ag

GTGCTGCTGTCGCAAGGCCTGGTAAAGTGGTTGGTTCGGCACTTCGTTATGGTAACTGTT

CAACATCTGGTACTGAGCAATATGAACAGCGAAGGGTCATCACAAGCCAAGGAATTGTTC

CAAACGGCGTTCCTTCAGGCAGCTCATACCCTAGAAGAAATAACACCTGCAAGAGTGAAA

CAGGTGAAGCTGAAAGGATCGACGTGAGCCAAGCCGTGCCACCAAAGCCATATACCGGAA

ATAAACTACCTGCAACTGTGGATGGCCGCAATGGGCACTGGTAGACTGCTGTGACAATGC

CAGAAAATCCTTGTCACCTCATTCCAAGGACACACCTTCAAACATGCCATTCTGTACAGA

GTTATATTATCTGTCAAGCTTACATCTCCCGGAGACGATAGAACTGTATGAGAGGCCTTT

GCGACAGGCTAGTCTCCTGTTAAGCTCAACTCTGGTCTGGTTACCTGCAATTGGGTAGAT

CCAGTTCAACTGTTCAACAAGTATAACAAAAAAACTGGAAACTTTGAGCGGCATTCGCGA

CAAAGGACTGTTACTGACCTGACTGATGGTGTTCCTCGATTTGTTTCTCCAAGCTGGTGG

CCTTGAGGGAGGTTCAGAGAGCCCACTGTACACTATCTAATGTTGTAACACGGAGTTAAT

TAATTGACTTTAATGTTCTTGTTGCTTGTAGTCTGTCATCAAGCTGTAGTATTGAACTAT

TAAACACGGCTATTTCTGATAACACATTATTTGGACCCTGCTCGCTATTGAGTGCGGAAT

TTATAATGATGTATGCAGTAATCAAGTAGCCCGAGTAAAAATGGAGTTGCGACGTATGTT

TCTTGTATTGCATAACATGTTTACTGTCCTGTTTTTCAAGAAATTAACCTTTCTAGCAAT

TTAAGAAATTAACTGTTTTTCT

**>TaMPK16(U): TRIAE_CS42_U_TGACv1_642759_AA2122920.1**

GGAAATCTAGTTAAACAAAGAGAGAAATAAATCTCGGGTATAAATTCCGAAAGCGGAGGC

CACCGGTGGCCTTCTCTCTCCTGACTGCCCAGCTACCGCCGCCCCCCTCCCTCCCCTCTG

TCCTCGAGCTTCGCCTTGGATCGCAGCGCAGCGCAGCGCAGCTGGACTCCTCCCCTCGGC

CGCGTCCCCCCTCGTCCTGCGCGCGCGTGAGCGA

gtgagctccctccctcccttcccccatccccatgcccccgctctcctcctcctcccctac

tttcttgctggatcgggggcgcttgaagatctcgcctttctcgcgcaaaagatcccctct

cctcctgggagagatctgccgtgaggaggcggcaggggcggatgaaggaggccgtgcccc

tctctgttccgctgagcgctcctgccgcccccacgctctagcgccgcgctgggagttagc

agccttctttcttttcttggagtggattggtagtgttcttggttcgggatcttggtgagt

ctggagcttttgctcgccgaatcgcgaagctgcgctgttctcgtggtagacttgttcccc

gctgtaaaacctggcggctagacggatctactcgatttggagttgggttgctttacagat

ttggagtactttttggtggaaaagaattggctcggccgtttggttttgtataggtctagt

cagttcctcttgtaaattgattgatgctcggcagtaatggtgctactgttcatttattca

caaccctctcagttcgagctatgaacccaactttgttaaagccttgacttgagtagttgt

gctgtcgtgttcatatttttacccacctctctgtgcggcaatatgcatgcttcatgccat

ccgaacaattccatcgtttgggcaaaaagtttcaacttgttttggagaacgaaagtaatg

ccccggacaacgttgtatcacgtggttctagtgccgttcttttgcttccaagcaactttt

ttcagataaaatttgttacttgacgattgagaccatgctagtttgcgtcgtatgaattct

ccatggaacttaatttcctatgtggtcattttcgcataatatttggcaagtgtctttttg

tacatagtcctcgtcgcttgccaaattatgactctaatatctattttgagtgtcaccatc

tcaccatgtcatactaaagaacgcaattcatcagtgctcaagctgactcatattctgttt

tttgtgacacag

ACATCTCAAATGGACTTTTTTACTGAGTATGGTGAGGGAAACAGATACAAGATAGAAGAG

GTTATAGGAAAAGGGAGTTACGGCGTGGTCTGCTCTGCTTTGGATACTCACACCGGTGAG

AAAGTTGCTATCAAGAAGATAAACGACATCTTTGAACATGTGTCTGATGCGACACGGATA

CTCCGCGAGATCAAGTTGCTTAGGCTCCTAAGACATCCCGATATCGTGGAAATAAAACAT

ATTCTACTTCCTCCATCGAGGAGAGAGTTCAAGGACATATACGTTGTTTTCGAACTCATG

GAGTCTGATTTGCACCAAGTTATAAAGGCGAATGATGACTTGACTCCAGAACATTATCAG

TTTTTCTTGTATCAGTTGCTCCGAGGATTGAAATACATACATACAG

gtaaccgatttattttcgcatcaaagatgcctcttttgcatattccaacagctggatgtc

aggtgtctcaatttactaataattgtagcagccataaatggtcaatgtgtttaaaagtga

tacacatgtaagtagataacaatgccatgtcaaattcgaaactatatttctagtatccgg

cgacgatgtattggtcgtgttggtatttgtgaaaacctttaaaaccaatggtatgttcca

caaactttgtagtcacgagagcaatatggacctgtgtagtgctttgatacaggtcacaca

gttcgctatagtgcccggagaactctggaaatccagatcttatttgtgatttgttgaatt

tgtgctagcttgtcacagcctgagttgtgtgttctagtctaatcacctagtaactgatca

atgctgtgatgaaaagttactgttaatcatttgagtagctacaatctggacgtgctccca

aagcttcttgtgatgctacaacagaactgtggtcctagtgaggatgctattggtgctgct

ctttgtgccaaaagtcagcgactagataggcatactaatcttgtgaagcactgtttgagg

ctagagaacttctgtttaaactttttttataaatataaataacttatttgtgatcaagta

gttattttcagttctcttttttggcatgctaagaaataatattttgctggtataataaca

atgaaaaacgcatgcgtcatctctccattcaaagagcaatactgtagaaatccctttttt

tcagattatttaaacagatgagtattaatacttttgctatagatacaaatgtggcattat

gagttgattggcaataatataaaacaagactcatagaaaaatcattttggctgatgcatc

ttccaatagcttcttgcag

CAAATGTATTTCATCGAGATCTCAAACCAAAGAATATCTTGGCTAATGCTGATTGTAAGC

TCAAAATATGTGATTTCGGTCTCGCAAGGGTAGCTATAAGTGATACTCCAACCGCAATAT

TTTGGACG

gtattgcaatttccccctaccatgctgaccatttcttttggctactcaatatcaataaca

gcagctaatttcttatcctgttttag

GATTATATCGCAACAAGGTGGTACCGAGCACCTGAGCTATGTGGATCTTTTTTCTCCAAG

gtctgtatatgttaaatttcatatgagtcatggtatcgacagtattttttttatttttcc

ctcatatgaatctgatttacttcgtttactgtttaaggctttaagggttctgcaactttg

tgacagcaaagcaaagtaacatccccctggcatttccttagtagttcttttcctatttca

taacaggttgtgttctaatatatactccctccgttccaaaatagatgactcaacttagta

caaagttagtacaaagttgagtcatctattttggaacggagggagtattagctatgggaa

acaatgccaatcagccagcatggttaaaaaataaatggttaagaacacattccaacatct

ggcacagttcatgtttttttgtcttccaccctaaacggatcaaatattttttaatttgca

tattgtttttatgttcagggtaatcatactgcttcaatgtcattcaggaacgaccttgtt

ttctgtatttcttttcgcaaaccgaatgattaagctgtcaaaggaataagggcacctcta

gttgtaactcaagtactctatgtctaggtttgattaatccagctaaacatattctggaat

atcttgagcgatgtataacattatgttcttcaaaatgttgcag

TATACACCAGCAATAGATATATGGAGTATTGGATGTATATTTGCGGAACTTCTAACTGGC

AAACCTCTTTTTCCTGGGAAAAATGTGGTGCATCAACTTGATATAATCACAGACCTCCTC

GGAACGCCTGCTCCAGAAACAATTGCTAGG

gtcagtattgttggcataaaacagcgtgttgtttccaatacaatcctctgatgtttgtag

ctatcctacag

ATTCGAAATGAGAAGGCCAGGCGCTACTTGAGCAGTATGAGGCGGAAAAAGCCTGTACCG

TTTACGCAGAAGTTTCCGAATGCAGATCCACTTGCATTAAATTTGTTAGAGAGAATGCTA

GCATTTGATCCAAAAGACCGGCCAAGTGCTGAAGAG

gtagtttgttttagtaccatgcctgccagcaatttttcctgtgtttcttctttccttata

accttgaataccattagctaatgtattaggcatgttgacag

GCTCTTGCTGATCTTTATTTCAAGAACATAGCTAGTGTGGATAGGGAGCCTTCTGCACAG

CCCATTACTAAGCTTGAATTCGAGTTTGAGAGACGAAGAATTACGAAGGACGACATAAGG

GAACTCATATACAGAGAAATTCTGGAATATCATCCAAACATGCTGAGGGAATTCCTTGAG

GGGGCAGAGCCAACTAATTTCATGTACCCAAG

gtgcgaagttctctttcatcaatactttagcattcttcattgaaatggagtgcctagttt

aattccagagacaatgtagtgacattattcttgtcgaccactaatgtctctgaatttcag

agacaatgtagttctatctgaatttctacaatttttttgtgccactaatgtctgtgcagg

ttcatgaataaatattaagtgtttatacacacacgattatggttatttttctactctttt

ggtgatggtgatattgataaataaagcatccggaaattgtttttgtttattgttgattag

gtagtgcatacctattctatgtgttcttatttgttgcttggagtcagtcaggcactgcat

aagatactgaccagttcatcgaacgaacattgtttcctctatttggatttcag

TGCAGTAGATCATTTCAAAAAACAATTCACATTCCTTGAAGAGCATTATGCAAAGGGATC

AACAGCAGCACCGCCTGAGAGGCAACATAATTCATTACCAAG

gtaagatattgactttgctttaacatggtatatggatggactgatgtcacttctagccac

aaattggacgtgtggttcttgactatctatttttctgttgacag

GCCGAGTGTTATCTATTCGGATAACCGACCACAGGGTTCAGATAGCCGGCCACAGGGTGC

AGCCAACATTACGGATGATCTTTCCAGGTGTATAATCAGAGATAATACACAAAAGCCACG

CAGAGATCCTGCTTCAGTTGGTGCAAACAGAGTTCCTCAAG

gtaagcacctgggttgtccaatacttctgaatctgcaaacaagcctgcatgacaattgta

tctgtcggccctcgtaactcaagactaacttgatacatcacttcatcatacag

GTGCTGCTGTCGCAAGGCCTGGTAAAGTGGTTGGCTCGGCACTTCGTTATGGTAATTGTT

CAACACCTGGTACTGAGCAATATGAACAGCGCAGGGTCATCACAAGCCAAGGAGTCGTTC

CAAACGGTGTTCCTTCAGGCAGCTCATACCCTAGAAGAAATAACACCTGCAAGAGCGAAA

CAGGTGAAGCTGAAAGGATAGACGTGAGCCAAGCTGGGCCACCAAAGCCATATACAGGAA

ATAAACTACCTGCAACCGTGGATGGCCGCAACGGGCACTGGTAGACTGCTGTGGCAATGC

CAGAAAATCCTTGTCACTGGATCAGGAAGGGTTTACTCATTCCAAGGACACACCTTCAAA

CATGCCATTCTGTATATTATCTGTCACGCTTACATCTCCCGGAGACGATAAAACTGTATG

CGAAGTCATTGTGGCAGGCTAGTCTCCTGTTAAGCTCAACTCTGGTCTGGTTACCTGCAA

TTGGGTAGATCCAGTTCAGCTGTTCAACAAGTGTAACCAAAAAACTGGAAACTTTGAGCG

GCATTCGCGACCAAGGACTGTTACTGACCTGACTGATGGTGTTCCTCAATTCGTTTCTCC

AAGCTGGTGGCCCTGAGGGAGGTTCAGAGAGCCCATTGTACACTATCTAATGTTGTAACA

CGGAGTTAATTAATTGACTTTAAAGTATGTTCTTGTTGCTTGTAGTCTTGTCATCCAGCT

GTAGTATTGAACTATTGAACACTGCTATTTCTGATAACACATTATTTGGACCCTGCTCGC

TATTGAAACCCCAGTTTTTTTGTGCGGAATTTGGTATTGTATGCAGTGATTTAGTAGCCT

GAGTAAACACGGAGTTGCGACGTATGTTTCTT

**>TaMPK17(7AL): TRIAE_CS42_7AL_TGACv1_556562_AA1765430.2**

GCCCGCGATCCTTCCCGGCAGCGGCAAGCCGGTCAATCCTCTTTCTCTCCCCCGGGTACG

CGCCGCGCGGTTTTTTTTTTGAAAGTCGCGCCGCGCGGTTTGTTTGCCTTCGTCAACACA

CACCGTGTGTGAGCGAGCGTGTGCTGTGACAGCTTGTATCTACGTATCACTCACCCGCAC

GGCAAAAAAACGCAGAGGCGAGGGCCGGAAAAGGAGAGAAAATTCCTTCCGCCCCGCCCC

CAAATCGCGCCGGGCAGTATAAGAGCACACCCCGCCGTTTGCGCCCCGCATCCAATCCGT

CCGTCCTCAGCGCCGACGACACCGCCGGAGATAGCGGCCGAGCACTCCACCTCGAGCCAG

TGAGTGGCGGCGGGAGGGGGAGGCGAGAAGAGTTGGTGGAGGAGGAGGAGGAGGAGCTGT

GGGCTGTGGCTGGAGGAGGCGGCTAGCAGCTAGCCAATGCCGCAGGGGTAGGGAGGGGAT

ACATGGGGGGAGGGAACGGCATCGTCGACGGCTTCCGCCGCTTGTTCCACCGCCGCACGC

CCTCCGGCTCCGTGCTCGGCAGCTCCAACCAGTCCTCCGCCGGCGAGGACTCCTCCGACG

CCGAGGCCGCCGAGGACCTGGATCTCGTGGGCCTCCGCCCCATCCGCGTCCCCAAGCGCA

AGATGCCGCTCCCCGTCGAGAGCCACAAGAAG

gtgagaggagtactctgatgctctctgctgcttattctcgatgcttccagattgggaccc

ggagcttctggcagatgctatttccatttctcgtgaccctcgattcgtcgtcacgacgag

ttcttagccaagatcggatctttgcggcacctgcttagctcagatacgccactaccgtac

aatactagcttgccttttctttttcttttcttttcttttcttttactattactagtagtt

gacacggcagctcggcaggcgagcttgattcttcgatccaatttcatatctccggatcga

tacgacgtaggcggcactgcttgtgatcgagttaccgatcggcctgctgcttgactgata

tgggaaaatgttatgcaaaaccgaatgttgtttcttcagagatgcgtagttggagcatct

gccgcataaagcccggtgccctttggggcttctcttcacacttttttttgttgattttca

actactacggttgctttcagcatccgcattgaacttatccgaaggggctcctcatgtcca

atttgtccacttggttctacagcgactgaatcggctactgatcagttaggctgacttggt

ctggacctcgtgctcaactgctcatgtccacctcactcttgcaccatggtggctgcttcc

ttagtttctcggtaaacctgaccatttccctgattgctcattgctgagaatggtcctcca

cctaccgggctagactttttccgttctgggggttcgggctgtgtgttgtggtggtggtac

tctaagatctctttaggagcccaacgacctggggactgggaccggtctaatgggattgag

gatccacatcgttaaaagaatggtgtttggtcatgataaattataggtgaagatattgta

ttttataaccaaatgtatttataagcttgtgcgtttgcgtatgacattcgctttcccgtc

gaattgcttttacttatcaactattgaagtaaccttaggttattttactactaaatatag

tactttaacacaattctgaattcattcttcagtttcttcaatccaaaatgttccatacat

gagtaaccggttttaacttgctcttgtgaattctggtcttgtag

AACATAATGGAGAAAGAATTCTTCACAGAGTATGGAGAGGCAAGCCAGTACCAAATCCAG

GAAGTTGTTGGCAAGGGGAGTTATGGAGTAGTTGCTGCTGCAATAGATACCCGCACCGGC

GAGCGGGTTGCGATTAAGAAGATCAACGATGTGTTTGAGCACGTCTCGGATGCCACACGC

ATCCTCCGCGAGGTCAAGCTCCTTCGGCTGCTACGTCATCCAGACGTGGTGGAGATCAAG

CACATAATGCTCCCCCCTTCTCGGAGGGAGTTCCAAGATATATATGTTGTTTTCGAGCTC

ATGGAGTCGGATCTCCATCAGGTCATCAGAGCTAATGATGACCTCACGGCGGAGCATTAC

CAGTTTTTCCTTTACCAGCTTCTCCGTGCTCTCAAGTACATCCATGGCG

gtaagtcaatgaatgctaatctccatggatctgctagcccatttattagcacaatctttg

agttgatttaccaaattgtgattggacttctgttcatattctgacatattttgctgttta

tcttggcacag

CTAATGTATTTCATCGCGATCTGAAGCCCAAGAATATACTGGCCAACGCAGACTGCAAAC

TGAAAATTTGTGACTTTGGACTTGCGCGTGTATCATTTAATGATGCTCCTTCAGCTATAT

TTTGGACG

gtaaagagcttgtcattgtatgccataattcacattttactataattcttactttttgca

ttgtttttctttgtgaag

GATTATGTAGCAACAAGGTGGTACAGAGCCCCTGAATTATGTGGCTCCTTTTTCTCGAAA

gtgagttcttcactcctgaactcataaagtctcatccaagcaagattaatgcttttggag

cactatatgtgcagatgctgtctttggatttgtaatatatttattgtgttacatttatat

atccctattgctatttttatttttcttaaaaaactctgtaatctacagcttgtactaagt

aaagtacacaaaattaatgattaaacagtacaacaagtttttgttgttatttctcattgt

aattgtgcatacacccagttcttctgtcaaaacaagctgcaaacgaactaaatcagttgc

aaattagactagtaaaattcatgtagagacttagtatactctctgtgcaagtgtcagatt

cgtcccatcgttctgataagcgcccatttttgtgcag

TACACTCCTGCTATTGATATTTGGAGTATTGGATGCATATTTGCCGAGCTTCTCACTGGA

CGGCCACTCTTTCCTGGGAAGAATGTTGTACACCAGTTAGATATAATAACAGATCTTCTT

GGAACTCCATCATCAGAAACCTTATCTCGG

gtatgtttgttccctgtcccttatttttgcttcattcactcacaaggtacttaaacatgt

tgcatttcactaacaaggtgaaacaatgcattccattcctgctgcacccttaagggtgtg

tttggttcgggaaggaagtagaatggaatgtcatggttcattccattagaatgggtcggt

tccgtctttgtgtttggtagtgacaatttgaaggaatggaatggttacattttgatgttt

ggtttgtcagatggaatgaaatgggtttattcatctcaccataaactgaattggattcca

gatcaggaactacccaaataccaaagtctgcatctgatctgattgctacaagcaaaaaat

gcagcttgctagtccagtcttgaccgcagccttgctcctccaatccagaagatcaacagc

ggcggccttcctgtcaccacgccacacgcacacttgtgtctcctcacgaacggcggcgtt

tggatcgcctactccgtcaggggcagcgcttggagcttctgctccgtcaagagcggcagc

gcggtgtttcccagtgtgacgcgcgagtgcggcggcagcgtggcctcccagcctcgttcc

gctgattatgcgcaaaaaaaaaagaagcctcgttccgctgatggatctcccgccatggag

cagagtgctcgcctccttcttccgccagatccttggtcgaggtgcggggtgggggaggac

gaggggcgggagacgccggcgaggctgcacttccggccgccgccatggccatcgagggag

agaggagggcacaggtcgtggttggctggggcgccaggtgggtgagtcggggagaagggg

agagaggcgcgagtgaggcgttccgcgtggtccggccgattcggaggaacgcccaggttc

ggcataaacagggaatatgccgttctctggaattagtgggttctggttccttcgccaatc

taaacacaggaacgggccctcgggatggaaccgacccgtgacattccgcttcatgataga

aaccaaacacaccctaagtaaaatgcattgtagactgctagagcaattccccaaatccca

tctaattactccctccgtccgacaatacttgtcaccaaaatgaataaaaagagatgtatc

tagaactaaaatacgtctagatacatcccctttcatccattttgatgacaagtatttccg

gacggagggagtatatctcttgtgatatatgctggccttcatctgcaaattttgcaatgg

tacattaggttacaggagtaataacttacagctgtctatgaccattgcag

ATTCGAAATGAGAAGGCCAGGAGGTACTTAAGTTGCATGCGGAAAAAACACCCTGTACCC

TTGACTCAGAAATTTCCTAATGCCGATCCATTGGCGGTTCGCCTACTGGGTCGTTTACTT

GCATTTGATCCTAAAGACCGGCCTTCAGCTGAAGAG

gtaaactcatctacatttgtttaacagtttcacaaacacatatatgtgtcttggtatgct

ataattttgtcgtgtctgtaattcttcag

GCTTTAGCAGACCCATATTTTGCATCTCTTGCCAATGTGGAGCGTGAGCCATCAAGGCAT

CCAATTTCGAAACTCGAGTTTGAGTTTGAGAGACGAAAGGTTACAAAAGATGATGTTAGG

GAATTGATCTATCGAGAG

gtaaggaggcatagcttgggtttcctattttgtgttaataaatatatttatgtctcttga

taattgattatttgttatgagaacaaacag

ATTTTGGAGTACCATCCACAGATGCTGGAGGAATACATGAAAGGTGGAGACCAGATTAGC

TTCCTCTATCCAAG

gtgtgcaacgcggacttgctttaattttgatgcaagttatcagacttaataaaatacaca

gctaacatatctttatatgggctattatgacgctgcag

TGGTGTTGACCGCTTTAAACGGCAGTTTGCACACCTGGAGGAGCATTACAGCAAAGGAGA

ACGAGGTTCTCCGCTGCAAAGAAAGCATGCCTCTTTACCAAG

gtacatataacaccgcagtcatcagaaattagaagtgccgagcttgttactctgatctcg

gttattcagttatttgtgctgagttctgcattgacttgtgtgcaacag

GCAGAGGGTAGGTGCATCGAACGACGGTAATAATGAGCAGCATATTAGTGATCAGGAGAT

GAGTGCAGAGCCTGATGCCCATGGTGCAGTGAGCCCTCAAAAGTCACAGGATGCACCCGG

TGTTGGCCAGAATGGTCTGAGCCCCACCAGCTTGAGCTCGCGGACCTACCTCAAGAGCGC

GAGCATTAGCGCTTCCAAGTGCGTCGTTGTCAACCCGAATAAACAGCCAGAG

gtactagtgctgtactacctgaattcttcattgattattgcaaacgactaggcatggcat

agctctgcactatcaacaaataagaaaggatggtggtgcaagttgaactcaagcactgac

tctttgtttctattattgttcag

TATGACGACGCGATCTCTGAGGAAACAGAAGGAGCCGTCGATGGACTATCCGAGAAGGTC

TCAAAGATGCATGCCTAGTGCAGCAACGACGACGGAAGTCGGCACCGCATTTCTCATTTC

TGTCAGGTACACCGGATGCTGATAGCTGATAGCAAAGCACTGCAAGTTGGAGATGGTTTA

GCGGAGTGCATGTTCCTGTCAAGCGTCGTTGCTGCACACAGGAGCTGGCCCTGCATGGAT

TGATGTATTTTTACACTCAATGTAACACTGGGATTAGTTCCTACACCTATCATCTATGCA

TGATTGTAACAACGAACAGAGCAAGACATCTTGACGCTGTCATGTTTCGCTGTCGAAAAT

TCTTAATATATCAAGAAGCGTTACTGCTGTGCATCTACTGAGTTTGGCGTTGAGTTTTAC

TTATTGCCGTGTTTGGATAAAAATTGCGGTAAGCC

**>TaMPK17(U): TRIAE_CS42_U_TGACv1_641980_AA2108810.2**

CATGTGGTAACATAGTTGGTTACTGTAACATTACACATATCAAGACAAGATGAGTCTACA

ACCTAATAAATGAAGTGTTGCATTACACCACACATATGTTACTCTCACTATAGAGATAGT

AACATAGACTAGTAACATGCAGACAAGCCCCTAATAAATGAGGAAATAGGGGCATATGTT

ACTAGTCTAAGTTACTACCCATTGTGACTAGTATGAGACTGAGAGTGCATGGAATTCCTC

AAAAGAAAAATAATGTGAATAAATCGCCGGCTAAAAATTCTAGTCTTGAACTTGATGACA

ACTTTTTTTTTACTAGATTAACTTGTGAAAACCGTGCGACCCAAAGATGCCCGAGACGGC

CAGTGCTCGCCGGCGGCCGAACAAAGACGGTACATTTCAGGCTCCCCCACGCCACATTTT

CCATGGCCAGCAGACAAGCCCCTACGCCCTAGACGACAGTGGACTCCGGGGCTGCGTGTC

TGGCTCGCCCAGAAAGACCACAGCTGTACCGGAATTCCGGCACCACCACCCATACGGCGC

CCGCGATCCTTCCCGGCAACGGCAAGCCGGTCAATCCTCTTTCCCTCCTCCGAGTACGCG

CCGCGCGGTTTGTTTGCCTTCGTCAACACACACCGTGTGTGAGCGAGCGTGTGCTGTGAC

AGCTTGTATCTGTCACCCGCACGGCAAAAAAACGCAGAGCCGAGGGCCGAAAAAGGAGAG

AAAATTCCTTCCGCCCCGCCCCCAAATCACGCCGGGCAGTATAAGAGCAGACCCCGCCGT

TTGCGCCCCGCATCCAATCCATCCGTCCATCCATCCTCACCGCCGACGACACCGCCGGAG

ATAGCGGCCGAGCACTCCATCTCGAGCCAGTGAGTGGCGGCGGAAGGGGGAGGCGAGAAG

AGTAGTAGTAGTAGTAGGAGGAGGAGCTGTGGGCTGTGGCTGGAGGAGGCGGCTAGCAGC

TAGCCAATGCCGCAGGGGTAGGGAGGGGATACATGGGAGGAGGGAACGGCATCGTCGACG

GCTTCCGCCGCTTGTTCCACCGCCGCACGCCCTCCGGCTCCGTGCTCGGCAGCTCCAACC

AGTCTTCCGCCGGCGAGGACTCCTCCGACGTCGAGGCCGTCGAGGACCTGGATCTCGTGG

GCCTCCGCCCCATCCGCGTCCCCAAGCGCAAGATGCCGCTCCCCGTCGAGAGCCACAAGA

AG

gtgagggcagtattctgatgctctctgctgcttatcgatgcttcagtctggtgggcggga

gcttctggtagatgctatctccatttttgcgtgaccctcgattcgtcgtcacgacgagtt

cttagccaagatcggatctttgcggcacctgcttagctcagatacgccactaccgtacaa

tactagcttgcctgttctttttcttttcttttcttttactattactagtagttgacacgg

cagctcggcaggtgagcttgattcgtcgatccaatttctcggttcgatacggcgtaggcg

ccactgcttgcgatcgagttaccgatcgatctgccgcttgactgatcttggaaaatgtta

tgcaaaaccgaatcctgtttcttcagagatgcgtagcttgagcatctgctggataaagct

ggtgcccttttgggcttctcttcacacttttttttgttgattttcaactactacggttgc

tttcagcatccgtatagcacttatccgaaggggctcctaatgtccaatttgtccacttgg

ttctacagcgactaaatcggctactgatcagttaggttgacatggtctggacctcgtgct

caactgctcatgtccacctcactcttgcaccatggtggctgcttccttagtttctcagta

aacctgaccatttccctgattgctgattgctgagaatagtccttcacctaccgggctaga

ctttttccctttcggggttcgagctttgtgttataggagcccaacgacctggggactggg

accggtctaatgggattgaggatccacattgttaaaaaaaagatatttggtcatgataaa

ttctaggtgaagataatgtattttataaccaaatgtatttataagcttctgcgtttacgt

atggcattcgctttcccgtcgaattgcttttactgatcaactattgaaggaaccttaggt

tatttcactactaaatatagtactttaacacaattctgaattcattcttcagtttcttca

atccaaaatgttccatacatgagtaactggctttaacttgctattgtggattctggtctt

gtag

AACATAATGGAGAAAGAATTCTTCACAGAGTATGGAGAGGCGAGCCAGTACCAAATCCAA

GAAGTTGTTGGCAAGGGGAGTTATGGAGTAGTTGCTGCTGCAATAGATACCCGCACCGGC

GAGCGGGTTGCGATTAAGAAGATCAATGACGTGTTTGAGCACGTCTCGGATGCCACACGC

ATCCTCCGCGAGGTCAAGCTCCTTCGGCTGCTACGTCATCCAGACGTGGTAGAGATCAAG

CACATAATGCTCCCCCCTTCTCGGAGGGAGTTCCAAGATATATATGTTGTTTTCGAGCTG

ATGGAGTCAGATCTCCATCAGGTCATCAGAGCGAATGATGACCTCACAGCTGAGCATTAC

CAGTTTTTCCTTTACCAGCTTCTCCGCGCTCTCAAGTACATCCATGGGG

gtaagtcaatgaatgctaatctccatggatctgctagcccatttattagcacaatctttg

agttgatttaccaaattgtgattggacttctgttcatatttctgacatattttgctgttt

atcttggcacag

CTAATGTATTTCATCGCGATCTGAAGCCCAAGAATATACTGGCCAACGCAGACTGCAAAC

TGAAAATTTGTGACTTTGGACTTGCGCGTGTATCATTTAATGATGCTCCTTCAGCTATAT

TTTGGACG

gtaaggagcttgtcattgtatgtcataattcacattttactataatttcttactttttgc

attgtttttctttgtgaag

GATTATGTAGCAACAAGGTGGTACAGGGCCCCTGAATTATGTGGCTCCTTTTTCTCGAAA

gtgagttcttcactcctgaactcatgaagtctcatccaagcaagattgcttttggagcac

aatatgtgcagatgctgtctttatatttgtgatatatgtattgtgttacatttatagatc

cctattgctatttttatttttcttaaaaaactctgtaatctacagcctgtactaagtaaa

gtacacaaaattgaggattaaacagtacaacaagtttttgttgttatttctcatcgtaat

tgtgcatacacccagttctcctgtcaaaacaagctgcaaactaactaaatcagtttaaaa

ttagactagtaaaattcatgtagagacttagtatactctctttgcaagtgtcagattcgt

cccatcattctgataagagcccatttttgtgcag

TACACTCCTGCTATTGATATTTGGAGTATTGGATGCATATTTGCCGAGCTTCTCACTGGA

CGGCCACTCTTTCCTGGGAAGAATGTCGTACACCAGTTAGATATAATAACAGATCTTCTT

GGAACTCCATCATCAGAAACCTTATCTCGG

gtatgtttgttccctgtcccttatttttgcttcattcactcacaaggtacttcaacgtga

ttcatttcactaacaagatgaaacaatgcatctcattcgtgctgtgcccttaagtaaaat

gcattgtaggctgctagagcaattccccagattccatctagttatattttttgtgatata

tgctggccttttagctgcaaattttgcaatggtgcattaggttacaggattaataactta

gagctgtctatgaccattgcag

ATTCGAAATGAGAAGGCCAGGAGGTACTTAAGTTGCATGCGAAAAAAACACCCTGTACCC

TTGACTCAGAAATTTCCTAATGCTGATCCATTGGCGGTTCGCCTACTGGGTCGTTTACTT

GCATTTGATCCTAAAGACCGTCCTTCAGCTGAAGAG

gtaaactcttctacatttgtttaacagtttcacaaacacatatatgtatctttgatatgc

tataattttgctgtgtccgtaattcttcag

GCTTTAGCAGACCCATATTTTGCATCTCTTGCCAATGTGGAGCGCGAGCCTTCAAGGCAT

CCAATTTCGAAACTTGAGTTTGAGTTTGAGAGACGAAAGGTGACAAAAGATGATGTTAGG

GAATTGATTTATCGAGAG

gtaaggaggcatagtttgggttccctattttgtgttaataatatattttatgtctctcga

taattgattatttgttatgagaacaaacag

ATTTTGGAGTACCATCCACAGATGCTGGAGGAATACATGAAAGGTGGAGACCAGATTAGC

TTCCTCTATCCAAG

gtgtgcaacgcggacttgctttaactatgatgcaagttatcggactaatacgcagctaac

atatatttatatgggctgttatgatgctgcag

TGGTGTTGACCGCTTTAAACGGCAGTTTGCACACCTGGAGGAGCATTACAGCAAAGGAGA

ACGAGGTTCCCCGCTGCAAAGAAAGCATGCCTCTTTACCAAG

gtacatataacaccgcagtcatcagaaattagaagtaccgagtttcttactctgatctcg

gttaatcagtgctgagttctgcattgacttgtgtgcaacag

GCAGAGAGTAGGTGCGTCGAACGACGGTAATAATGAACAGCATATTAGTGATCAGGAGAT

GAGTGCAGAGCCTGATGCCCATGGCGCAGTGAGCCCTCAAAAGTCACAGGATGCACCCAG

TGTTGGCCCGAATGGTCTGAGCCCAACTAGCTTAAGCTCGCGGACCTACCTCAAGAGCGC

GAGCATTAGTGCTTCCAAGTGTGTCGTTGTCAACCCGAATAAACAGCCAGAG

gtactagtgctgtactacctgaattcttcattggttattacaactaggcatggcatagct

ttgcactatcaaacgctatcaacaaataagaaaggatggtggtgcaagttgaactcaagc

actgactccatttccactctttgtttctattattgttcag

TATGACGATGCAATCTCTGAGGAAACGGAAGGGGCCGTCGATGGACTATCCGAGAAGGTC

TCCAAGATGCACGCCTAGTGCAGGGACGCCGATGAAAGTCGAGCTCGGCGCCGCATTTCT

GTCAGGTACGCCGGATGCTGATAGCATAGCAGTGCACTCCTAAGGAAGCAGAGCGCTGCA

AGTTGGAGATTGGTTTAGCCGAGTGCATGTTCCTGCCAAGCATCGTTGCTGTACCCAGGA

GCTGGCCCTGTCTGTCTTTGATGTATTTTTACATTCAAGCTCACAACGTAACACTGCGAT

TTGCTCTCTGTCATCTATGCCGGATTGTAACGAACAGAGCAATGCATCTAAACACTGTCC

TGTTTGTGTCGAACATTCTTAATCTTAATATATCAAGAAGCGTTACTGCTCCGCGTCTAC

TCAGCTTGGCGTTGAGTTTTGTTTCCTTCCGTGTTTGGATAAAAAAGTACTACCTCTGTT

CCAAAATACTTGAAATTTTTTGCGATAATTAAGGGCATCAAGGAGCGAGGGACTAGCATA

T

gtgagtttcttccttcttccgcgtcgtcgacctccaattccggagctatggttaaattgg

caagaaatcaccggtgagcgtcgtgtgtggctaggtggaataccggtcactagatgctat

tcccataatttcctatgcccactccttcgatccccgaaaatgagaggccatttcttcttg

cccatacatggtaagctagccatggtcggagatggtggcaaaaacatcttctacgaggtc

accgacacccctaggacttgatcgattttcccttaacttcttcatggtcatgtgtgcaaa

agtgaaggacatgtggaaactgggcttgctctctaaggtttaacaaactcccggggcttg

gtagtattggaagggattatcataccggtacacagagtacctttccttgtgcccctcttg

gtagtatggttgtcctgcactcgatgcgaatcttggattcatcaaccaaaaatctaggaa

ttacgttctaaaatcttgaggtagacttcactttgaaaagttaaggtaggggtcaccatt

catgttccactcatagcacgaggacacacttcatgaaataacctagataaacacaatagt

cctctcaattgttttttcattaatcatcaaaactcaataagagtatctaacaaatgcact

ttttgtactgtctatataagatgatcccaacatattttatgatttgtcgtgaatatttga

ttgggaaatacacatatataaacttagattttacttatcgttgttgctttgagaggtgct

actagtgaacctatgaactctggtccaattttaccaaatcacttctatatgcacctcact

agaacaattggaagcacaagtgggtcttaatttgtttccaatgtttgttgattgatgcta

ggggtgttatcactacggattgttaccttagttctcaaccgaggtgggagttaccacttg

ttgcgaatccttgcacttgggggctgaccacttggattctaggatataggtggaaacaag

cagccaacactcatcactgtaccatgagatagtatcaaccggggtaccaacctgaggcat

gtgttttcttgggtaccag

TGGTGCTGAATAGCTTTGCCCATAAGAGAGCAAT

gtatgtatattttctctcggtggtcggcgcctacattattcatgttctatcaaagataga

ggtctatataggattctccattttggtcgataatgcatacctctatttatcaaatctag

GAAATGGTAAATGAGCACAGAGATCGGCCAACATCCACTCTAAGCTTCGAGCGACAGGGA

CCTTGGAAGCGAAGCATACACGGTCGCCTGTGAATGAGCTAGGGA

**>TaMPK17(7DL): TRIAE_CS42_7DL_TGACv1_604680_AA2000630.1**

CTAGACGACAGTGGACTCCGGGGCTGCGTGTCCGGCTCGCCCAGAAAGATCGATGCTTTG

CGCACCTGACCACAGCTGTACCGGAATTCCGGCACCACCACCCATCCATTGATCCATACC

CCTGGCGCCCGCGATCCTTCCCGGCAACGGCAAGCCGGTCAATCCTCTTTCCCTCCTCCG

AGTCCGGGTACGCGCCGCGCGGTTCGTTTGCCTTCGTCAACACACACCGTGTGTGAGCGA

GCGTGTGCTGTGACAGCTTGTATCTGTCACCCGCACGGCAAAAAAACGCAGAGGCGAGGG

CCGAAAAAGGAGAGAAAATTCCTTCCGCCCCGCCCCCAAATCGCGCCGGGCAGTATAAGA

GCAGAGCAGACCCCGCCGTTTGCGCCCCGCATCCAATCCGTCCGTCCTCAGCGCCGACGA

CACCGCCGGGGACAGCGCGGCGGAGAGAGTGAGTGAGCTGCCTTGAGTGGCGGCGGGAGG

GGGAGAGGCAAGAGGACGAGGAGGAGGAGGAGGAGCTGTGGGCTGTGGCTGGAGGAGGCG

GCTAGCAGCTAGCCAATGCCGCAGGGGTAGGGAGGGGATACATGGGGGGAGGGAACGGCA

TCGTCGACGGCTTCCGCCGCTTGTTCCACCGCCGCACGCCCTCCGGCTCCGTGCTCGGCA

GTTCCAACCAGTCCTCCGCCGGCGAGGACTCCTCCGACGTCGAGGCCGCCGAGGGCCTGG

ATCTCGTGGGCCTCCGCTCCATCCGCGTCCCCAAGCGCAAGATGCCGCTCCCCGTCGAGA

GCCACAAGAAG

gtgagaacagtcagtattctgatgctctctgctgcttattctcgatgcttcagtttgggg

gtcgggagcttctggcagatgctatctccatttctcgtgaccctcgattcgtcgtcacgg

cgagttcttagccaagatcggatctttgcggcacctgcttagcttcgatacgccactacc

gtacaatactagcttgcctttcctttttattttcttttcctttcttttactagtagttga

cacggcaggtgagcttgattcttcgatccaatttcatatcttcggatcgatacgacatag

gcgctactgcttgtgatcgagttaccgatcgacctgccgcttgactgatctgggaaaatg

taatgcaaaacggaatgttgtttcttcagagatgcgtagcttgagcatctgctgcataaa

gccggtgttcttttgggcttctcttcacacttttttgttgattttcaactactaaggttg

ctttcagcatccgtattgcacttatccggaggggctcctcatgtccaatttgtccacttg

gttctacagcgactgaatcggccactgatcagttaggttgacatggtctggacctcgtgc

tcaactgctcatgtccacctcactcttgcaccatggtggctgcttccttagtttctcagt

aaacctgaccatttccctgattgctcattactgagaatagtccttcacctaccggcctag

acttttttccgttttggggattcgggctatgtgttctggtggtggtagtgtaggatttct

tataggagcccaacgacctggggactggaaccggtctaatgcattgaggatccacattgt

taaaaaaaaattatgtttggtcaaaataaattctaggtgaagataataagccataaccaa

atgtatttataaccttataggtgtacgcattacattttctttcctgtcgaattgctttta

ctgatcaactattaaagtaaccttaggttatttcactactaagtacttcaacataattct

ggattccttcttcagtttcttcaatccaaaatgttccatacatgagtaactggctttaac

ttgctcttgtgaattctggtcttgtag

AACATAATGGAGAAAGAATTCTTCACAGAGTATGGAGAGGCAAGCCAGTACCAAATCCAA

GAAGTTGTTGGCAAGGGGAGTTATGGAGTAGTTGCTGCTGCAATAGATACCCGCACCGGC

GAGCGGGTTGCGATTAAGAAGATCAACGATGTGTTTGAGCACGTCTCGGATGCCACGCGC

ATCCTCCGCGAGGTCAAGCTCCTTCGGCTGCTACGTCATCCAGACGTGGTGGAGATCAAG

CACATAATGCTCCCTCCTTCTCGGAGGGAGTTCCAAGATATATATGTTGTTTTCGAGCTC

ATGGAGTCGGATCTCCATCAGGTCATCAGAGCTAATGATGACCTCACGGCGGAGCATTAC

CAGTTTTTCCTTTACCAGCTTCTCCGCGCTCTCAAGTACATCCATGGAG

gtaagtcaatgaatgctaatctccatggatctgctagcccatttattagcacaatctttg

agttgatttaccaaattgtgattggacttctgttcatatttctgacatattttgctgttt

atcttggacag

CTAATGTATTTCATCGCGATCTGAAGCCCAAGAATATACTGGCCAACGCAGACTGCAAAC

TGAAAATTTGTGACTTTGGACTTGCGCGTGTATCATTTAATGATGCTCCTTCAGCTATAT

TTTGGACG

gtaaagagcttgtcattgtatgtcataattcacattttactataatttcttactttttgc

attgtttttctttgtgaag

GACTATGTAGCAACAAGGTGGTATAGAGCCCCTGAATTATGTGGCTCCTTTTTCTCGAAA

gtgagttcttcactcctgaactcatgaagtctcatccaagcaagattaatgcttttggag

cactatatgtgcagatgctgtctttatatttatgatatatttattgtgttacatttatag

atccctattgctatttttatttttcttaaaaaactctgtaatctacagcctgtactaagt

aaagtatacaaaattaaggattaaacagtacaacaagtttttgttgttatttctcattgt

aattgtgcatacgcccagttcttctgtcaaaacaagctgcaaactaactaaatcagttga

aaattagactagtaaaattcatgtagagacttagtatactctctttgcaagtgtcagatt

cgtcccgtcattctgataagagcccatttttgtgcag

TACACTCCTGCTATTGATATTTGGAGTATTGGATGCATATTTGCTGAGCTTCTCACTGGA

CGGCCACTCTTTCCTGGGAAGAATGTCGTACACCAGTTAGATATAATAACAGATCTTCTT

GGAACTCCATCATCAGAAACCTTATCTCGG

gtatgtttgttccctgtcccttatttttgcttcattcactcacaaggtacttcaacgtga

ttcgtttcactaacaagatgaaacaatgcatctcattcctgctgcgcccttaagtaaaat

gcattgtaggctgctagagcaattccccagattccatctagttatatatttttgatatat

gctggcctttcagctgcaaattttgcaatggtacattaggttacaggattaataatttac

agctgtctatgcccattgcag

ATTCGAAATGAGAAGGCCAGGAGGTACTTAAGTTGCATGCGAAAAAAACACCCTGTACCC

TTGACTCAGAAATTTCCTAATGCTGATCCATTGGCGGTTCGCCTACTGGGTCGTTTACTT

GCATTTGACCCTAAAGACCGGCCTTCAGCTGAAGAG

gtaaactcttctacatttgtgtaacagtttcacaaacacatatgtatcttgatatgctat

aattttgtcgtgtccgtaattcttcag

GCTTTAGCAGACCCATATTTTGCATCTCTTGCCAATGTGGAGCGTGAGCCTTCAAGGCAT

CCAATTTCGAAACTTGAGTTTGAGTTTGAGAGACGAAAGGTGACAAAAGATGATGTTAGG

GAATTGATCTATCGAGAG

gtaaggaggcatagtttgggttccctattttgtgttaataatatattttatgtctctcga

taattgattatttgttatgagaacaaacag

ATTTTGGAGTACCATCCACAGATGCTGGAGGAATACATGAAAGGTGGAGACCAGATTAGC

TTCCTCTATCCAAG

gtgtgcaacgcggacttgctttaattttgatacaagttatcggacttaataaaatacgga

gctaacatatctttatatgggctattatgatgctccag

TGGTGTTGACCGCTTTAAACGGCAGTTTGCACACCTGGAGGAGCATTACAGCAAAGGAGA

ACGAGGTTCCCCGCTGCAAAGAAAGCATGCTTCTCTACCAAG

gtacatataacaccgcagccatcagaaatccgaagtgccgagcttcttaccctgatcacg

gttaatcggttatttgtgctgagttctgcattgacttgtgtgtaacag

GCAGAGAGTAGGTGCATCAAACGACGGTAATAATGAACAGCATATTAGTGATCAGGAGAT

GAGTGCAGAGCCTGATGCCCATGGCACAGTGAGCCCTCAAAAGTCACAGGATGCAGCCGG

TGTTGGCCAGAATGGTCTGAGCCCTACCAGCTTGAGCTCGCGGACCTACCTCAAGAGCGC

GAGCATTAGTGCTTCCAAGTGCGTCGTTGTCAACCCGAATAAACAGCCAGAG

gtactagtgctgtccaacctgaattcttcgttgattattgcaaacgactaggcatggcat

agctctgcactatcgacgaataagaaaggatggtggtgcaagttgaactcaagcactgac

tctttgtttctattattgttcag

TATGACGACGCGATCTCTGAGGAAACGGAAGGGGCCGTCGACGGACTATCCGAGAAGGTC

TCCAAGATGCATGCCTAGTGCAGCAACAACGACGGAAGTCGGCACCACATTTCTCATTTC

TGTCAGGTACACCGGATGCTGATAGCAAAGCAGTGCACTGCAAGTTGGAGATGGTTTAGC

GAGTGCATGTTCCTGTCAAGCGTCGTTGCTGCACGCAGGGGCTGGCCCTGCATGGATTGA

TGTATTTTTACACTCAAGCTCGCAATGTAACACCGAGATTTGTTGTTCTCTAGCATCTAC

TACTATACGTGATTGCAACGAACGGACCAAGACATCTGATGAACACTTGTCATGTTTCCC

TGTCGAACATCCTTAATATATCAAGAAGCGTTGCTGCTCTGCATCTATATCAACAAGAAG

TGCTGAGTTTCATTTCCTTTCTTTCGTGTTTGGATAAATGTGGCAATCAAGCTGAACCCT

GAGTACAGATTTAGAAACATCTAGTAGTGTATTTCTCA

**>TaMPK20-1(3AL): TRIAE_CS42_3AL_TGACv1_194002_AA0624350.1**

GAGGGGCAATCTCGTCCGCCCGCGCACGGAAATCAAAGCAGGATTCCTCTCATTTCATTT

CATGGCAACAAAAACAAGCCAGACACACTGCCTGCCTCCTCTCCTCTCCTCTCCACCCGT

CCTCCTCCTCTCTCTCTCTTATTCCCCTCCCGGAGCGCGAGCGCAGACCAGGAGAGGAGG

AACAGGAGAGAGAGAGAGAGGGAGCCCTAGCCCTGCCCCGGCGCCCCGTCGCTAGGGTTA

GGGTCTCCCCCCCAGTTTCCCGCCCTCCCTTCGTCGCCTCGTCCCGTCTCACGATCCGCC

CGCCGGCGCCCTGCGGCGTGTGCGCTTTTCGGCCTCGCCGGCCCGGCCCGGCGCCAGCAA

ATGCCGTGTGTCTGACCGTGGTGATTCGGAGGTGGGCTTGGTGTTGTTGTTGTCGTTGCC

GCCCGTACGGCACAGGGGAACCGAGGAGATTCCGGGCGAGATCCGGTGAGCGGCGAGATG

CAGCAGGATCAGCGCAAGAAG

gtgggctccttcccagagatccctagcatttttgcgtgcgtgaattccgctccagtcgag

ctggttgatgcagctcagctgctcctttgccttgtttttttttttattatttcgaggcaa

tgctttgccttgtttggggtctctgtccgttcaagccttcgttttccctttctgttcggc

actgtttcttgtttctgttctgggcatcactcccatagatttgacgttccgccgccgtat

cactattctcatttcagtgccgtgtcaatccggatgtttgaatttatttttattcccaca

agggtaggtattttttggcagtattttcgttagccgtccacgatcttttccctctgtaac

ctgaatctctaatatgatcatgtcggtccttctcgctttcatcttcttgtcccatgcatt

ttccgcactcccgtcgcatcgcattgagttatgcggcagtcttgcttctatatttgggaa

aaaaggtgtaatttggcgctcctttactataacagttcgcaattcacatgcaaaagttga

ccgcgttctgtgacctcgagaggctgcccgcgcaaacacattgtggccgttgcttttcct

tgtcggtgggcaaagctgttggcttttcttgtggggttctcttctccccgatctgtgcac

gagcacttgactcacaagtggtgcgtgtcacgatttcagtttcgcaaccaacgcttcaag

atctttctcctcaagaaaatttctttgatctacctgcacaacagttgaatttggcatcta

ttccttaatactggttgtagcttcccaatgattagttattttacatgtcgtccattaaaa

gtatctggagatatgccatcatttttgcgacccaccacctaccgtgccatttctgctttt

gcttgacagcgtgacatcaattgtttgtatatacgtatatgattgcgagaactgatggtc

cattgatcactcaagcttcttgtcgccgggagcttctgacgagcttccagtgctagattt

gtgaaatgtatttgacatgatatctgcaattcttttccag

AGTTCCGCAGAGGCAGAGTTTTTTACGGAGTACGGCGACGCAAGTCGATACAAGATTCAG

GAAATCGTTGGAAAGGGCAGCTACGGTGTCGTGTGCTCCGCAATAGATGTCCACACCGGA

GAGAAAGTGGCGATAAAAAAGATACATGATATCTTTGAGCACATATCTGATGCTGCACGG

ATTCTCCGTGAGATCAAGCTTCTGAGGCTCTTAAGACATCCTGACATTGTTGAGATCAAG

CATATTATGTTACCTCCATCGAGACGAGACTTCAAAGATATTTATGTCGTTTTTGAGCTC

ATGGAGTCTGATCTCCACCAGGTTATAAAGGCTAATGATGACTTGACAAAGGAGCATTAC

CAATTCTTTCTTTACCAATTACTCCGGGCTCTGAAGTACATTCACACTG

gtaggtaacataccgatttatctttctcgaagatcatatgtcgctggaccatacagtaaa

tttgctctgtggtgagcttgtatcaggtcaccaccttctagttagatatactagctaaat

aaacccgaacgcttatagttggtgcagtatgtacggtattgttagcatatacaaataatt

tgcatatggcatatattagattagctgaagttcttgagttcaaagcaaaaaaaatctgta

ccaactagtgccatactggttgggtagggaagataggtgataaacatatggccatcacct

gaattccaaatatgctcagaaacagcaaattatctcaatcgtaggcattagggggtgaac

aacaggatagattttctttcatagagtgcgggttgtttcctcatgatttaaagattttat

tacgcaatttcctgataacttacatatttgctggttcaaatggtatacctgtgtggtggt

agtcttttcaacaaccaaaaaggagtatctccacagttctttccatgaacaaaatctgta

taaataacatgtccagtcgtgctccttttgctgcctgcacccattgctaatcctatgacc

atatgtaactgcag

CTAATGTTTATCACCGGGACCTAAAGCCAAAGAATATATTGGCAAATTCCAACTGCAAAC

TGAAGATTTGTGATTTTGGATTGGCACGAGTTGCATTCAACGATACCCCTACAACAATCT

TTTGGACG

gtatgttcaaggaatacctatattctgccatccttacatttccgacttgtttgatttgag

ctaatcatcatgatgtctatttgttcaattgcactacag

GATTATGTTGCGACAAGATGGTATAGAGCTCCAGAGCTCTGTGGTTCCTTCTTTTCAAAG

gtaagcttccctccgttattagcattctcttggttgaattttggttaaagcactctgctg

acagcttgacataacgaaagactaatggtgctgcgttgacataccatgcatctttcgtta

caattaatcaacctattaatacaaatcgttgttcttcggtgattggtccattagttttgt

tccaagcttatttatgctgtggcatgaaacctcatttccatacagatcttagggcatctc

caacggcaacccgcaaatttcctcccgcatccgtccgcggactggtaaacatgacggatt

tcatacaaacacgacggattttcatcacatttctaacatttagaacaaaaggaaaaagag

ccgaccctaaacctatcctacggcggcggcgtccggcgtccaagcccttgtcatcctcca

tccgcgtcttcatgagcaccggcgataagagcgatgaatttagttgcggtctctgacgag

gaagagtagaacacgggagacggagtggcccacatgggacacaaggccctgccgcctcca

gtgccctactgatcctcggaggagtctgcgccttgtccgttgccggtggacgtgaggtct

acgatggaaatggtgatgcccgcgctatcgtcgtcccaccgggtcgtcggatagatcatc

ggcggagcagctggcgttgttctcctccgcgatcacctggagctgtgcctcggcggcggc

cgcttcctggagagcggcggcttgagcgcggacggcatcgtagatgacacgctgctccgc

gacgaagttggggttcgtcgcagccatggccgccatggcctcctcgctcgcacgctcgcc

gtagatctggccctccgccagccggtgctgctccagaaagaacatgttgtaccactgata

gaggcaaaggtgtcccatcttttgatgagatggtgaatatcgtttcggaggagtagactt

tcacgatccgactacgaacgtgcgaggacgtcgtgccttagcaatcgctaaatcaactcc

gagaggttattgaccacgctggagcatgatcaacgtgaccacaaaggtctatttcctgca

agcaaacgaagaacaagcaagaaactaagattgcaaatctggatattgcgaatataagat

gaaagctttattgatcaaggtggggttctgtgacgccttggtctggtcgttgaacacaaa

cgaagtacgcgaagttgcagctatggcgaatttttaatctaaacaaaacccaaagtctaa

acgacgccctaagggctgtatatatggatgaagaggggggaatttcgtggcccttgtagg

aggggtccgaaaccaaccctatctcttgtttccccacacatacggactctaaaacagcct

atacttatgtgtttcgaaattacatgggcctggcccaataataaggtgacgcagcaccta

taatagcctctggacgaaatttgtgaagtggcatcttgtatatttcgtccaagacttcat

gcactcattacggtggcttcaaagtcctgaaatcatcacttgaaactccgttcttgtttc

cttgcgcatgccatcatctccatgcttgttcttgctccaatgttcatccttctccaagct

aggcccttcatttgtaagcaaaacaaatgtatccaatttaggcagcatcatattctcatg

aacattagaatcattaccaagaaacggaagtacctggtaatttaaccggcgtgcacgagc

tctagtaattggtccagtatgtatagtagcaggggctgtgggtgtaacaatggtattgat

gtcctcatcaaccactgttcctcctgcacctgctggaacgccgacaaaggcgtcggtgca

ggctgtacctttgggtgtggagttagccgggtgtggccgcctttatatagcggattccgg

tgaggcaaggtgtccgggtgcgtcaatgcgcgtcgccggagtctcggctggcgagcctgc

ttaatggcggcatacggatggacagcggcattgaacgggtgtggtagatgtccgtcccgt

ccagtcacgcatccccggcattgaacaggtgcggacatcggagacgcatcgcgggcggcg

ccctcggccagcacggccgcttcaatgacggaggcagtgagaggtcgcgtccgccctgag

ccgtcttcaatgttaagcggagcgctctacagcggcatgaatgcgggcagctggcgccgg

gcgggaacgtgcgcgggcgagggaggaggggttttggtgggccaaggcggtcagaagagg

gcgtgggatcggtctggactcccgcaaagtccccccacgtttgtctccggtttgctggag

agaaaatcgcgtccggaccgctccgcggaccaatacaggttgcgggaggtttgagggttg

ccgttggagatgcccttatagggatattttatgggagcacgacaccttctattgaaacaa

gctctgcttctttgtgaaaagctaatttaattcataaaccatcactttgactttccccac

cgtcataatcatatgtttgttgtttccttatcacattgttgatcatgcattaagtgctca

aacatgaagttgctccatttttatgtaaatttatgctacatagaattttgacttcgtatc

gttctcggtaggagctgttaaaaatcatatgagttacatttgtgttgagacttgtgaaag

catctcagacattgttatatttgattgttctcaatgaacattatcatgatgcggtactga

taaacaaataacttgttactcatgcatgtttttttctaacataaatgctatgttaacaaa

attgcag

TATACACCCGCCATTGATGTCTGGAGCATCGGGTGCATCTTTGCTGAGGTCTTGACAGGA

AAACCTTTGTTTCCTGGTAAAAATGTTGTACATCAGTTAGATTTGATGACTGATCTTTTG

GGCACACCATCTATGGATACAATCTCTCGG

gtatagttcttgttttatttgggaatccttctatagtctatttggtcggttatccaattg

acattaataggttcttcctttccag

GTCCGGAATGAGAAAGCAAGGAGGTATCTGAGCAGCATGAGAAAGAAAGAGCTGATTTCA

TTTTCGCATAAATTTCCCAATGCAGATCCTTTGGCCTTAGACCTGTTGCAAAGGCTTTTA

GCGTTTGATCCAAAGGACCGCCCAACTGCTGAAGAG

gtacattgctagtgtttttacagccctgcttatgttggtagcagtgtcttgtactctcga

gttattgaggtgcataaagtgacattatttggaaacttgtgatttctgtcag

GCATTGTCTCATCCGTACTTTAAAGGGCTTGCCAAGGTTGAGAGAGAACCATCCTGCCAA

CCAATCACAAAAATGGAGTTTGAGTTTGAGCGTAGAAGAGTGACAAAAGAAGACATAAGG

GAGCTTATATTTCGTGAGATATTGGAATACCATCCACAATTGCTCAAAGACTACACCAAT

GGCACTGAAAGGACAACCTTTCTATACCCAAG

gtctgagcaataatatctttggaattagatggtgcttttgcacatgctttcacctttttt

tcattgtatctttgcagtctactgactgggacacttctctacctatgtttatgcag

TGCTGTTGATCAATTTAGGAAGCAGTTTGCTCATCTTGAAGAAAATAGTGAGAATGGCCC

TGTAGTTCCAATGGATAGAAAGCACACCTCTCTTCCTAG

gtaacctttcacgttccatgatatttctttctctacctttattatattagcggcaggagt

tcagaacagctaaaaggcacacatcagagcttaatacattggtcagggtcacagtttgga

acttaggcacaccgtcatttgtcacacttcaccttgtctagtgataatgttgtcttcata

tggtttgacaagaaggtgaaacgagcctatgttaatatgatacaatgaatacattggcca

cactttaactccgatgacaatgttatcttaatatgctttcacaagaaggtgaaatgagcc

aatgttgacatgacataatgaataacattttggatttcaatgtctgtagctagagctggg

caaaaattaaactgaattattgggtttttcaattggcagctgaaagttcggtttgtagaa

ttgactaaccgaactagtgcagtcaaattcagttttaagtcgcggttaactgaaaatcca

aagaaaaccaccgtgtagggcttaggccaagtaatagtcatggcccaaggcctcctgtga

gatgcattttggcatctcgctgagactagggcacatgcagccacacacatcatgcctgca

tgccatgaacagccgaaccaaacactgccatctccctcacgccgctagcgctaggaccta

ggaccacaccgccacgctcgcatcccgcctctgagcaaaggctgatggagggatccctgg

cacaccgtgatgccccactgctgaccggccgctgttgtaaaatccatattgctaaaacag

tgataaaaatatggcaaaatatgttaataaagtgaaatcgtaacgaccactatgttaatg

gaacagttcctaaccaaatagcgagcataaaatttgggccatgtcagagttggcttcatg

caactgaatgtggattgtagaacattgaagtcaactttcctatgtactgttaggctggtc

ggccaaattgacatgccaggagatgatacacacttcaaattgcccactacctggctggac

atttagttcttgcgaactgattcatgaaacatttgttaatgtttttacag

GTCTACTATTGTTCACTCAGCTCCAATTCATGGAAAGGAACAACCCCGTATTGGCCCATC

AAGGGATAAGCCTTCGCCTGACGAGTCTTATAAAAATCATCGAGATTCAGAAAAATATTC

TGGCAATGTCCAGAGAACCTCACAGGCTCCACAAAGAGTGCCAACAG

gtgctttctagtccatgcttgatatacgtgcttagctggtactagttttaatctaatcat

ataatgcttgtcaattatacag

CAAGACCAGGAAGGGTTGTTGGTCCAGTTCTGCCTTACGAAAATGGTGGCATGAAGGATA

CATATGACCCACGAAGAGTGGCAGCAATGAGCTCAGGTTATCCTCCCCAACAGCAAATCC

CGCAAATATACGGTTATTATCAGACACCGGCAAAGTCAGCTTGCTCTGAGCCATCGCAGG

CCGAAATGTATACGCTGCACCAGCAGGCCTATGCTTGCGCAAACAGTTCAACTGTGCCTG

ATGTTGCTCTGGACATGAGAGCACCTCCGTTCCATCAATCAGCAGGGCCGAAAAGGGGTT

CTTCTGATAGGCTATCAGCGGAATCAAACCTATACTCGAGATCCCTTAATGGCATGGCTC

CAACTACAACAGGAGTGGCGGCAAGTGCTCACAGGAAGGTCGGCGTTGTTCCCTACGGTA

TGTCGCAGATGTATTAGTGGCCAGTCAGGCGCATCCGACATGCCATCCTGGAGTGCGGCT

GGTGGCTAACGAAACAGCGAGGAACGAAGTGGCGGATTAAGCTTGGGTGGGGATGCCGCA

AGGTTTACAGCGCAGGGCAGCCCATGGTTTGATGCTTATGTGTGCTGTCTGATCTGGAAG

CGTGTGAAGCTCTTCGTTCTGTTCCGGGCCATCAGCTTGTTGTATCTGCCTGTGTGCACA

CTGGATGGGGGTGGGTAGTCAGTCCAGTGGCTTGTAATCTGAGCTATTTAGGCGCCGGTT

ACTTTTTCTAGGGCGAGGAGACAGAAGGGGAAGGAAGGAATGCTAACCCGTTTTCTGTTG

TAGAGATGTTTGTCATCAATGTGCAACTTCAGAAAGATATATATAAACTTATAAAGGACA

CTTTTGCTGCAGGTGATAATTGCCACCACGTAGTCGAATCAGTGGCTTCTGGTTTTAAAC

ACGGCAAACGCAGATGATCATTTCCTGT

**>TaMPK20-1(3B): TRIAE_CS42_3B_TGACv1_224428_AA0796670.1.X (based on AA0796670.1)**

GGGGAGGGGCAATCTCGTCCGTCCGCGCACGGAAATCAAAGCAGGATTCCTTTCATTTCA

TTTCATGGCAACAAAAACAAGCCAAACACACTGCCTGCCTCCTCTCCACCCGTCCTCCTC

CTCCTCTCTCTCTTTCTTATTCCCCTCGCGGAGCGCGAGCGCAGACCAGCAGAGGAGGAG

GAGGAACAGGGAGGAGAGAGGGAGCCCTAGCCCTGGCCCGGCGCCCCGTCGCTAGGGTTA

GGGTCTCCCCCCCAGTTTCCCGCCCTCCCTTCGTCGCCTCGTCTCGTCTCGTCTCACGAT

CCGTCCGCCGGCGCCCTGCGGCGTGTGCGCTTTTCGTCCTCGCCGGCCCGGCCCGGCGCC

GGCAAATGCCGTGTGTCTGACCGTGTCCATTCGGAGGCGGGCTTGGTGTTGTCGTTGCCG

CCCTTACCTTACGGTACGGGGGAGCTGAGGAGATTCTGGGCGAGATCCGGTGAGCGGCGA

GATGCAGCAGGATCAGCGCAAGAAG

gtcggctccttcccagagatccctagcatttttgtgcgcgtgaattccgccccagtccag

ctgattcatgcagctgagctgctcctgcgcttcctttttactattattattatttcgagg

gaatgctttgccttgtttggggtctctgtccgttcaagccttccttttccctttctgttc

ggcacagtttcttgtttctcttctgggcatcactcccatagatttgacgttccgccgccg

tatcactattctcatttcagtgccgtgtcaatccggatgtttgagtttatttttattccc

acaagggtaggtattttttggcagtattttcgttagccgtccccgatcttttccctccct

ctgtaacatgaatctctaatatgatcatgtcggtccttctcgctttcaccttcttgtccc

atgcattttccgcacccccgtcgcatcgcgttgagttatgcggcagtcttccttctatat

ttgggaataaaggtgtaatttggcgctcctttattactatagctcgcaattcacatgcaa

aagttgaccgcgttctgtgacctcgagaggctgcccgcgcaaagacattgtggccgttgc

ttttccttgtcggtgggcaaagctgttggcttttcttgcggggttctcttctccccgatc

tgtgcacgagcacttgactcacaagtggcgcgtgtcacgatttcagttgcgcagcgaatg

cttcaagatcttgctcctcaagaaaatttcttcatctacctgcacaacagttgaatttag

catctattccttaatactggttgtagcttcccaatgattagttattttacatgtcgtcca

ttaaaagtatctggagatctgtcatcatttttgcgacccaccacctaccgtgccatttct

gctttgcttgacagcgtgacatcaattgtttgtatatacgtatatgattgcgagaactga

tggtccattgatcactcaagcttcttgtcgtcgggagcttctgacgagcttccagtgaca

gatttgtgaaatgtatttgacgtgatatctgcaattcttttccag

AGTTCCGCAGAGGCCGAGTTTTTTACGGAGTACGGCGACGCAAGTCGATACAAGATTCAG

GAAATCGTTGGAAAGGGCAGCTATGGTGTCGTGTGCTCCGCAATAGACGTCCACACCGGA

GAGAAAGTGGCGATAAAAAAGATACATGATATCTTTGAGCACATATCTGATGCTGCACGG

ATTCTCC

gtgagatcaagcttctgaggctcttaagacatcctgacattgttgagatcaagcatatta

tgttgcctccatcgagaagagacttcaaagatatttatgtcgtttttgagctcatggagt

ctgatctccaccag

GTTATAAAGGCTAATGATGACTTGACAAAGGAGCATTACCAGTTCTTTCTTTACCAATTA

CTCCGGGCTCTGAAATACATTCACACTG

gtaggtaacataccgatttcttatctttctggaaatcatatgtcgcaggaccatgcagta

tatttgctctagtgaacttgcatcaggtcaccaccttccagttagatatactagctaaat

aaacccgaacgcttatggttggtgccgtatgtacggtatcgctagcatattcaaataatt

tccatatgacatatactccctctgatccaaaacaagtgttgcagctttgaactaaagttg

aactaaccttagttcaaagctgcgacactttttttggattggagggagtattagattagc

tgcggttactgagttcatagcaataaaatctgtaccaactagtgccatgctggttgggta

gagaagagaggtgataaatatatgcccatcacttgaattcaaaatatgctcaaaaacagc

taattatcttaatctttgtaggcattaggtggtgaacaccaggatagatattctttcata

gagtgcgtgttgttacctcatgatttagagattttatattacgcaatttcctgataatac

atatttgctggttcaaatggtatacccgcgtggtggtagtcttttcaacaaccaaaaagg

agcatctccacagttctttccatgaacaaaatctgtataaataacatgtccagtcgtgct

acttttgctgcctgcacccattgctgatactatggccatatgtaactgcag

CTAATGTTTATCACCGGGACCTAAAGCCAAAGAATATATTGGCAAATTCCAACTGCAAAC

TGAAGATTTGTGATTTTGGATTGGCACGAGTTGCATTCAACGATACCCCCACAACAATCT

TTTGGACG

gtatatttcaaggaaaacctatatcctgccatccttacatttctgacttgtttgatttga

gctaatcatcatgatgtctatttgttcaattgcactacag

GATTATGTTGCGACAAGATGGTATAGAGCTCCAGAGCTCTGTGGTTCCTTCTTTTCAAAG

gtaagcttccctccattattagcattctcttggttgaattttggttaaagcattgagctg

acagactgatataacggtagactaatggtgctgcgttgccataccatgcatctttcgtta

ccattaatcgacctattaatacaaaccattcttcttcggtgattggtccattagttttgt

tccaagcttatttatgctgtggcatgaaacctcattttccccacagatcttatagaatat

tttatgggagcacagcaccttttattggaacaagctctgcttctgtgtgaaaagctaatt

taatctgcaaaccatcactttgactttccccactgtcataaccatacgtttgttgtttcc

ttatcgcattgttgatcatgcattgagtgctcaagcatgaagttactccatttctatgtc

aatctatgctacatagaatcttgacttcgtatcgttctcggtaggagctgttaaaaatca

tatgagttatatttgtgttaagacttgtgaaagcatctcaggcattgttatatttgatta

ttctcaatgaacattatcatgatgcgatactgataaacagtaacttgttactcatgcatg

tttttctctaacataaatgctctgttaacaaaattgcag

TATACACCCGCCATTGATGTCTGGAGCATCGGATGCATCTTTGCTGAGGTCTTGACAGGA

AAACCTTTGTTTCCTGGTAAAAATGTTGTACATCAGTTAGATCTGATGACTGATCTTCTG

GGCACACCATCTATGGATACAATCTCTCGG

gtatagtgctggttttatttgggaatccttctatagtctatttggtcggttatccaattg

acattaataggttcttcctttccag

GTCCGGAATGAGAAAGCAAGGAGGTATCTGAGCAGCATGAGAAAGAAAGAGCTGATTTCA

TTTTCGCATAAATTTCCCAATGCAGATCCTTTGGCCTTAGACCTGTTGCAAAGGCTTTTA

GCGTTTGATCCAAAGGACCGCCCAACTGCTGAAGAG

gtacattggtagtgtttttacagccctgcttatgctggcacagtgtcttgtacttttgag

ttattgaggtgcataaagtgacattatttggaaacttgtgatttctgtcag

GCATTGTCTCATCCGTACTTTAAAGGGCTTGCCAAG

gttgagagagaaccatcctgccaaccaatcacaaaaatggagtttgagtttgagcgtaga

agagtgacaaaagaagacataagggagcttatannnnnnnnnnnnnnnnnnnnnnnnnnn

nnnnnnnnnnnnnnnnnnnnnnnnnnnnnnnnnnnnnnnnnnnnnnnnnnnnnnnnnnnn

nnnnnnnnnnnnngactaggacacttctctacccttgtttatgcag

TGCTGTTGATCAATTTAGGAAGCAGTTTGCTCATCTTGAAGAAAATAGTGAGAACGGCCC

TGTAGTTCCAATGGATAGAAAGCACACCTCTCTTCCTAG

gtaaccttcattgttccatgatatttctttctctaccttttattatattcgcggcaggag

ttcaaaacagctaaaaggcacacatcagagcttaatagattggtcagggtcacagtttgg

aacttaggcacaccgtcatttgtcacacttcacctcgtctaataataatgttgtcttcat

atggttagacaagaaggtgaaacaaacctatgctaatatgacacaatgaatggcattttt

ggcttcagtttggaactttggaacaccgacattggccacactttaactccgatgacaatg

ttgtcttaatatgctttcacaagaaggtgaaatgagccaatgttgacgtggtggcataat

gaataacattttggacttcaatgtatgtagctagagctgggcaaaaattaaaatgaatta

ttgggtttttcagttggcagctgaaaattcggtttgtagaattgactaaccgaactggtg

cagtcaaattcagttttgagtcccggttaactgaaaatccgaagaaaaccaccttgtagg

gtttaggccaagtaatagccaaggcctgctgtgagatgcattttggcatctcgctgagac

tagggcgcatgcagccacacacatcacgcctgcatgccatgaacagtcaaaccgaacgct

gccatctccctcacgccgctaggacctaggactacaccgccacgctcgcatcccgcctct

gagcaacggccgatggagggatccctggcacaccgcaacgccccacacggccttactagc

gttctgccgtcgctgctgaccggccgctgttgtaaaatccatatttattgctaaaagaat

gataaaaatatgacaaaatatgttaataaagtgaaatcgtaatgaccactaaatgttaac

taaacagttcctaacaaaatagcaagcataaaatttgggccatgttgacatgccaggaga

tgatacacacttcatattcggaatctcagctctcacaacctgatagttgattagtgcact

ttgatgcaggatgtattgattctcttgcagtctacctggctggacatttaattcttgcaa

attgattcatgaaacatctgttaacgtttttacag

GTCTACTATTGTTCACTCAGCTCCAATTCATGGCAAGGAACAACCCCGTATTGGCCCATC

AAGGGATAAGCCTTCGCCTGACGAGTCTTATAAAAATCATCGAGATTCAGAAAAATATTC

TGGCAATGTCCAGAGAACCTCACAGGCTCCACAAAGAGTGCCAACAG

gtgctttctagtccatgcttgatatacgtgcttagctggtactagttttaatctaatcat

ataatggttgtcaattatacag

CAAGACCAGGAAGGGTTGTTGGTCCAGTTCTGCCTTACGAAAATGGTGGCATGAAGGATA

CATATGACCCATGAAGAGTGGCAGCAATGAACTCCGGTTATCCTCCCCAACAACAAATCC

CGCAAATATACGGTTATTATCAGACACCGGCAAAGTCAGCTTGCTCCGAGCCATCGCAGG

CCGAAATGTATACGCTGCACCAGCAGGCCTATGCTTGCGCAAACAGTTCAACTGTGCCTG

ATGTTGCTCTGGACATGAGAGCACCTCCCTTCCATCAGTCAGCAGGGCCGAAAAGGGGTT

CTTCTGATAGGCTATCAGCGGAATCAAACCTATACTCGAGATCCCTTAATGGCATAGCTC

CAACTACAACAGGAGTGGCGGCAAGTGCTCACAGGAAGGTCGGCGTTGTTCCCTACGGTA

TGTCGCAGATGTATTAGTGGCCAGTCAGGCGCATCCGACATGCCTTCCTGGAGTGCGGCT

GGTGGCTTAACGAAACAGCGAGAAACGAAGTGGCGGATCAAGCTTGGGTGGGGATGCCAC

AAGGTTTACAGCGCAGGGCAGCCCGGGGTTTGATGCTTATGTTTGCTGTCTGATCTGGAA

GCGTGTGAAGCTCTTCGTTCTGTTTCGGGCCATCAGCTTGTTGTATCTGCCTGTGTGCAC

ACTGGATGGGGGTGGGTAGTCAGTCCAGTGGCTTGTAATCTGAGCTATTTAGGCGCCGGT

TACTTTTTCTAGGGCGAGGAGACAGAAGGGAAAGGAAGGAATGCTAACCCGTTTCCTGTT

GTATAGAGATGTTTGTCATCAATGTGCAACTTCAGAAAGATATATATAAACTTATAAAGC

ACACTTCTGCTGCAGGTGATAATTGCCACCAGGTAGTTGAATCAGTGGTCACCGGTTTTA

AACATGGCAAACGCAGATGATCATTTCCTGT

**>TaMPK20-1(3DL): TRIAE_CS42_3DL_TGACv1_253300_AA0894090.1**

GGGAGGGGCAATCTCGTCCGCCCGCGCACGGAAATCAAAGCAGGATTCCTTTCATTTCAT

TTCATGGCAACAAAAACAAGCCAAACACACTGCCTGCCTCCTCTCCTCTCCACCCGTCGT

CCTCTCTCTCTCTCCCTCTCTTATTCCCTCCCGGAGCGCGAGCGCAGACCAGGAGAGGAG

GAGGAGGAACAGGAGAGAGAGAGAGAGAGAGAGCCCTAGCCCTGCCCCGGCGCCCCGTCG

CTAGGGTTAGGGTCTCCCCCGAGTTTTCCCGCCCTCCCTTCGTCGCCTCGCCTCGTCTCA

CGATCCGCCCGCCGGCGCCCTGCGGCGTGTGCGCTTTTCGGCCTCGCCGGCCCGGCCCGG

CGCCAGCAAATGCCGTGTGTCTGACCGTGTCTGTTCGGAGGCGGGCTTGGTGTTGGTGTT

GGTGTTGCCGCCCTTACGGTACGGGGGAGCCGAGGAGATTCCGGGTGAGATCCGGTGAGC

GGCGAGATGCAGCAGGATCAGCGCAAGAAG

gtgggctccttcccagagatccctagcatttttgcgtgcgtgaattccgctccagtcgag

ctgattgatgcagctcagctgctcctttgccttgcttttattattattattatttcgagg

caatgctttgccttgtttggggtctctgtccgttcaagccttcgttttccctttctgttc

ggcacagtttcttgtttctgttctgggcatcactcccatagatttgacgttccgccgccg

tatcactattctcatttcagtgccgtgtcaatccggatgtttgaatttatttttattccc

acaagggtaggtattttttggcagtattttcgttagccgtccacgatcttttccctctat

ataatctgatgaatctctatatgatcatgtcgctccttctcgctttcatcttcttgtccc

atgcattttccgcactcccgtcgcatcgcattgagttatgcggcagtcttccttctatat

ttgggaaaaaaggtgtaatttggcgctcctttactataccagctcgcaattcacatgcaa

aagttgaccgcattctgtgacctcgagagtctgcccgcgcaaacacattgtggccgttgc

ttttccttgtcggtgggcaaagctgttggcttttcttgtggggttctcccgatctgtgca

cgagcacttgactcacaagtggtgcgtgtcacgatttcagtttcgcaaccaacgcttcaa

gatctttctcctcaagaaaatttctttgatctacctgcacaacagttgaatttggcatct

attccttaatactggttgtagcttcccattgattagttattttacatgtcgtccattaaa

agtatctggagatctgccatcatttttgcgacccaccacctaccgtgccatttctgcttt

gcttgacagcgtgacatcaattgtttgtatatacgtatatgattgcgaaaactgatggtc

cattgatcactcaagcttcttgtcgccgggagcttctgacgagcttccagtgatagattt

gtgaaatgtatttgacatgatatctgcaattcttttccag

AGTTCCGCAGAGGCAGAGTTTTTTACGGAGTACGGCGACGCAAGTCGATACAAGATTCAG

GAAATCGTTGGAAAGGGCAGCTACGGTGTCGTGTGCTCCGCAATAGATGTCCACACCGGA

GAGAAAGTGGCGATAAAAAAGATACATGATATCTTTGAGCACATATCTGATGCTGCACGG

ATTCTCCGTGAGATCAAGCTTCTGAGGCTCTTAAGACATCCTGACATTGTTGAGATCAAG

CATATCATGTTACCTCCATCGAGAAGAGACTTCAAAGATATTTATGTCGTTTTTGAGCTC

ATGGAGTCTGATCTCCACCAGGTTATAAAGGCCAATGATGACTTGACAAAGGAGCATTAC

CAGTTCTTTCTTTACCAATTACTCCGGGCTCTGAAATACATTCACACTG

gtaggtaacatacagatttcatatctttctggaaatcatatgtcgcaggaccatacagta

tatttgctctagtgaacttgcatcaggtcaccaccttccagttagatatactagctaaat

aaacccgaacgcttatggttggtgccgtatctacggtatcgctagcatattcaaataatt

tccatatgacatatactccctctgatccaaaataagtgttgcagctttgaactaaagttg

aactaaccttagttcaaagctgcgacagtttttttggatcggagggagtattagattagc

tgaagttattgagttcatagcaaaacaatctgtaccaactagtgccatgctggttgggta

gagaagaggggtgataaatatatgcccatcacttgaattcaaaatatgctcaaaaacagc

taattatcttaatctttgtaggcattaggtggtgaccaccaggatagattttctttcata

gagtgcgtgttgttccctcatgatttagagattttatattacgcaatttcctgataacta

catatttgctggttcaaatggtattcccttgtggtggtagtcttttcgtcaaccaaaaag

gagcatctccacagttctttccatgaacaaaatctgtataaataaaatgtccagtcgtgc

tacttttgctgcctgcacccagtgctgatactatggccatatgtaactgcag

CTAATGTTTATCACCGGGACCTAAAGCCAAAGAATATATTGGCAAATTCCAACTGCAAAC

TGAAGATTTGTGATTTTGGATTGGCACGAGTTGCATTCAACGATACCCCCACAACAATCT

TTTGGACG

gtatgttcaaggaatacctatatccttccatccttacatttctgacttgtttgatttgag

ctaatcatcatgatgtctatttgttcaattgcactacag

GATTATGTTGCGACAAGATGGTATAGAGCTCCAGAGCTCTGTGGTTCCTTCTTTTCAAAG

gtaagcttccctccattattagcattctcttggttgaattttggtgaaagcattgagctg

acagactgatataacggtagactaatggtgctgcgttgccataccatgcatctttcgtta

ccattaatcgacctattaatacaaaccattcttcttcggtgattggtccattagttttgt

tccaagcttatttatgctgtggcatgaaacctcattttccccacagatcttatagaacat

tttatgggagcacagcaccttctattggaacaagctctgcttctgtgtgaaaagctaatg

taatctgcaaaccatcactttgactttccccactgtcataatcatacgtttgttgtttcc

ttattgcattgttgatcatgcattgagtgctcaaacatgaagttactccatttctatgta

aatctatgctacatagaatcttgacctcgtatcgttctcggtaggagctgttaaaaatca

tatgagttatatttgtgttaagacttgcgaaagcatctcaggcattgttatatttgatta

ttctcaatgaacattatcatgatgcgatactgataaacagtgacttgttactcatgcatg

tttttctctaacataaatgctctgttaacaaaattgcag

TATACACCCGCCATTGATGTCTGGAGCATCGGATGCATCTTTGCTGAGGTCTTGACAGGA

AAACCTTTGTTTCCTGGTAAAAATGTTGTACATCAGTTAGATCTGATGACTGATCTTCTG

GGCACACCATCTATGGATACAATCTCTCGG

gtatagtgctggttttatttgggaatccttctatagtctatttggtcggttatccaattg

acattaataggttcttcctttccag

GTCCGGAATGAGAAAGCAAGGAGGTATCTGAGCAGCATGAGAAAGAAAGAGCTGATTTCG

TTTTCGCATAAATTTCCCAATGCAGATCCTTTGGCCTTAGACCTGTTGCAAAGGCTTTTA

GCGTTCGATCCAAAGGACCGCCCAACTGCTGAAGAG

gtacattggtagtgtttttacagccctgcttatgctggcagcagagtgtcttgtactttt

gagttattgaggtgcataaagtgacattatttggaaacttgtgatttctgtcag

GCATTGTCTCATCCGTACTTTAAAGGGCTTGCCAAGGTTGAGAGAGAACCATCCTGCCAA

CCAATCACAAAAATGGAGTTTGAGTTTGAGCGTAGAAGAGTGACAAAAGAAGACATAAGG

GAGCTTATATTTCGTGAGATATTGGAATACCATCCACAGTTGCTCAAAGACTACACCAAT

GGAACTGAAAGGACAACCTTTCTATACCCAAG

gtctgagcaataatatctttggaattagatggtgcttttgctcatgctttgacctttgtt

ttcattgtatctcttcacagtctactgactaggacactctctacctgtgtttatgcag

TGCTGTTGATCAATTTAGGAAGCAGTTTGCTCATCTTGAAGAAAATAGTGAGAACGGCCC

TGTAGTTCCAATGGATAGAAAGCACACCTCTCTTCCTAG

gtaacctttcattgttccatgatatttctttctctaccttttattatattagcggcagga

gttcaaaacagctaaaaggcacacatcagagcttaatagattggtcagggtcacagtttg

gaacttaggcacaccgtcatttgtcacacttcacctcgtctaataataatgttgtcttca

tatggcttgacaagaaggtgaaacaaacctatgctaatatgacacaatgaatggcatttt

tggcttcagtttggaactttggaacaccgacattggccacactttaactccgatgacaat

gttgtcttaatatgctttcacaagaaggtgaaatgagccaatgttgacgtgacataatga

ataacattttggacttcaatgtatgtagctagagctgggcaaaaattaaactgaattatc

gggtttttcagttggcagctgaaaattcagtttgtagaattgactgaccgaactggtgca

gtcaaattcagttttaagtcccggttaactgaaaatccgaagaaaagcaccttgtagggt

ttaggccaagtaatagtcatggcccaaggcctgctgtgagatgcattttggcatctcgtt

gagactagggcgcatgcagccacacacatcacgcctgcatgccatgaacagtcgaaccga

acgctgccatctccctcacgccgctaggacctaggactacaccgccatgctcgcatcccg

cctctgagcaacggccgatggagggatccctggcacaccgcgacgccccacacggcctta

ctagcgttcagccgtcgctgctgaccggccgctgttgtaaaatccatattattgctaaaa

gaatgataaaaatatggcaaaatatgttaataaagtgaaatcgtaatgaccactaaatgt

taactaaacagttcctgaccaaatagcgagcataaaatttgggccatgttggagttgggt

tcatgcaactgaatgtggattgtagaacattgaagtcaactttcctgcgtactgttaggc

tggttggccaaattgacatgccaggagatgatacacacttcatattcggaatctcagctc

tcacaacctgatagttgattagtgcacattgatgcaggatttactgattctcttgcagtc

tacctggctggacatttagttcttgcgaattgattcatgaaacatctgttaacgttttta

cag

GTCTACTATTGTTCACTCAGCTCCAATTCATGGCAAGGAACAACCCCGTATTGGCCCATC

AAGGGATAAGCCTTCGCCTGACGAGTCTTATAAAAATCATCGAGATTCAGAAAAATATTC

TGGCAATGTCCAGAGAACCTCACAGGCTCCACAAAGAGTGCCAACAG

gtgctttctagtccatgtttgatatacgtgcttagctggtactagttttaatctaatcat

ataatgcttgtcaattatacag

CAAGACCAGGAAGGGTTGTTGGTCCAGTTCTGCCTTACGAAAATGTTGGCATGAAGGATA

CATATGACCCACGAAGAGTGGCAGCAATGAGCTCAGGTTATCCTCCCCAACAGCAAATCC

CGCAAATATACGGTTATTATCAGACACCGGCAAAGTCAGCTTGCTCCGAGCCATCGCAGG

CCGAAATGTATACGCTGCACCAGCAGGCTTATGCTTGCGCAAACAGTTCAACTGTGCCTG

ATGTTGCTCTGGACATGAGAGCACCTCCCTTCCATCAGTCAGCAGGGCCGAAAAGGGGTT

CTTCTGATAGGCTATCAGCGGAATCAAACCTATACTCGAGATCCCTTAATGGCATGGCTC

CAACTACAACAGGAGTGGCGGCAAGTGCTCACAGGAAGGTCGGCGTTGTTCCCTACGGTA

TGTCGCAGATGTATTAGTGGCCAGTCAGGCGCATCCGACATGCCTTCCTGGAGTGCGGCT

GGTGGCCAACGAAACAGCGAGAAACAAAGTGGCGGATCAAGCTTGGGTGGGGTTCCCGCA

AGGTTTACAGCGCAGGGCAGGGCAGCCCAGGGTTTGATGCTTATGTGTGCTGTCTGATCT

GGAAGCGTGTGAAGCTCTTCGTTCTGTTTCGGGCCATCAGCTTGTTGTATCTGCCTGTGT

GCACACTGGATGGGGGTGGGTAGTCAGTCCAGTGGCTTGTAATCTGAGCTATTTAGGCGC

CGGTTACTTTTTCTAGGGCGAGGAGACACAAGGGAAAGGAAGGAATGCTAACCCGTTTTC

TGTTGTATAGAGATGTTTGTCATCAATGTGCAACTTCAGAAAGATATATATAAACTTATA

AAGCACACTTTTGGTGCAGGTGATAATTGCCACCACGTAGTCGAATCAGTGGCTTCTGGT

TT

**>TaMPK20-2(1AL): TRIAE_CS42_1AL_TGACv1_002534_AA0042880.5.X (based on AA0042880.5)**

CGAGCATCGCACCAGCAGCCGCAGGCCGCAGGTCGCAGCCCATGGCATGTGGCCCAGAGG

CAACGCGCACGCATCTCTCTCTCTCTCTCTCTCTCTCTCTCCCGATTAAAACCAACAACA

ACAACGGCAAGTCGGCAGACATCACAAAGAAGAGAAAGAAATCTACCTCCCCTCCCCTCC

GGCATCCCGCTCCCCTTCCCTTCTCCGGCGCGCCGCCAGATCGGGCCAGAAGAGTCCGTC

CGGCACCTTGGGCCCGGCATCGGCCGCCGCGGCATACGTGTGAGTTCGGCGGGCGGCTCG

GCCGACCGACCGGGAGGCGGGGGGTCCGGCGCCGCGGGAGGGCAGGGGTCCGGCTTCCGG

AGCGGCCTTGGAGCGCAGATCGGCCCGCCCGGCAAGCGGCAATGCAGCAGGCGCAGCGGA

CCAAG

gtgggttccgactcccgtgccgtacctccccctcctcctgcgtctgtcttgtagacatga

tttgatttgtgcctgttcttgcggcgtcgatgcctgctgctcgtctcggttccaggacga

cgcaggcgagccggcgtatttttgaatcctcgctgccgtcttttcaaattcaaacctctt

tgacagattcttcgctacttttgttgtgatttcggtgctgccaacccttcctttagccga

aatctttgatctgtgtttctgtcagtagaaaaatagtcaaatgccgtcggttccattcca

gtacggcgacaccgcgtgatgggttacagacagctcacgcctcgcatgggaaagttgacc

gcctccttgccgcttcctgaatccagatgccttctgtgccaaacgctagacgaaatccgc

aagatgccgcttttcccctgccgcctgtgggtggcgcgcagagcttctccattttaatcc

ttgttggagtcctcgcagatgcccaccagcaagcaacctgcgttcagtggcagaaaaagt

tttaaactgtttgattgcttgctggtgctttggcttttcttaatgcctggttctgttgca

atgtggccactccaaatctctgtcggtctccaatgattttctccaacccccaccacacca

ctcattgtgccgtctcagttccccacatcaaccgtttaaagttctctctgttttaacagt

tgggttttgacagtttgctgattcggtgttttccag

AGTTCGGCGGAGGTGGACTTCTTCACGGAGTACGGCGACGCCAACCGGTACAAGATCCAG

GAGGTCATTGGCAAGGGGAGCTATGGGGTTGTGTGCTCTGCCCTTGATCTGCAGACCAGG

CAGAAAGTGGCCATCAAGAAGATACACAATATTTTCGAGCATACCTCCGACGCCGCGCGG

ATTCTCCGTGAGATCAAGCTTCTGAGGCTCTTGCGGCATCCCGACGTCGTCGAGATCAAG

CACATTATGTTGCCTCCCTCCAGAAAGGACTTCAAAGACATTTATGTTGTTTTTGAGCTC

ATGGAGTCTGATCTCCACCAAGTTATTAAGGCCAACGATGACTTGACAAAAGAGCATTAC

CAGTTCTTTCTCTACCAGCTACTCCGAGCCCTCAAATACATTCATACTG

gtaaaaaatcgttgccttcaagtctactgttgttgcttggaaaacaatgttttaccatga

aggtggtgtgtgttttagtttacaatcatgtgtagtaggtcagatcctcttttcatctct

ttttcacttgcctctcaaacactccaccgccccccccacccccccccccccccccccccc

cccccccccccccnnnnnnnnnnnnnnnnnnnnnnnnnnnnnnnnnnnnnnnnnnnnnnn

nnnnnnnnnnnnnnnnnnnnnnnnnnnnnnnnnnnnnnnnnnnnnnnnnnnnnaagtacc

aaaatgtgacttgcttcgagtgtagtcaaccaagttaacaacagggtttatcatttgata

ttaaatcctgtgtatcatcatctctcaatctacagttgcatctttctccgtttgctttgt

gctacagagacatctttaagatacgtatcatcccttatcaacaattcttaaaatatgacc

atattacatctattcagatattagcctacattctctgaattcctcaaagtgttttttttc

ctttgaggccatagatcatcccgtagctaaaataacgacaataattattaaacggaggga

gtagcagtcaagataagtaccagtccatatctgattcaacctttgttcacctcttgaaat

gaaatattttggtgttttgttcattatttatccctttatatcgtaggcactctagccaag

actctgtaatttgattgctcgttggaaatcttttccacttaattgccatcacaaagcttg

tcatggtattgaattgatactaccagaaattgcacccccaatgtttctctttagtgatat

actgtagaaaacgttacttgattgcgtattctatttttatcaccactttattttttttgc

tgataaggatttcatatgactgcag

CTAGTGTTTACCATCGTGACCTGAAGCCCAAGAATATTTTGGCAAATTCTAACTGCAAAT

TGAAGATATGTGATTTTGGACTAGCACGAGTTGCATTCAACGACACCCCAACAACTGTCT

TCTGGACG

gtatgttagtacaaactcaatttatgttttttatagcagattttttatgaagtatcataa

tttatagatattatgcataatccttgcag

GATTATGTCGCAACAAGATGGTATAGAGCTCCAGAGCTCTGCGGATCCTTCTTCACAAAG

gtgaggtttagagttcatggctttgtgcttattaaaaaatgaagaaaagaactaggttct

taatctgttgacaggcttatagtgtaagaaagaacatgcttacaattgttctagcatgag

ttgattgaactgtgagctgtaacatgcaaatggtttatgatttatggttctgtatcattg

tctttgctttcatcttctcttatgataggttttctggtgaaaattaaaatgcactatgta

cctgttcatcctatgttttattgttttgccttttttgtcaaagtcgtaaatagcctatgc

tttattgttatggtttctccatctatcatagattagatctgttcattgtcatttctatcc

ttttgtatcttgtgacctaatttcattcatgaattgatcttaacacttgttcatcctatg

ttctacacgaatactcgatcactttcctcttgaatagtaactttgacttacacgtgaact

tctcttacattcgagaacagtaaaagcatctttagtagttttcaggattttgacagatgc

atgttcttaatctatttttggcttttgtttaccaaaatgcag

TATACACCTGCCATTGACATCTGGAGCATTGGGTGTATTTTTGCTGAGGTGCTGACAGGA

AAGCCTTTGTTTCCTGGTAAAAATGTTGTCCATCAGTTAGACTTGATGACTGATCTTCTA

GGCACACCTTCAATGGATACAATTTCTCGG

gtacgacactaaattcaggaggagactatatatttagtttgtctggtaaatgtattcgtt

gagccaaatatttgactattgtttttgttccttcccag

GTCCGAAACGAGAAAGCAAGAAGGTACTTGAGCAGTATGAGAAAGAAAGACCCGGTCCCA

TTTTCTCAGAAGTTTCCTAATGCAGATCCTTTGGGAGTAAAACTCTTGGAGAAGCTATTA

GCCTTTGATCCAAAAGACCGTCCAACTGCGGAAGAG

gtagtcttatggtgttattttgtctttctgatgccttggtttcttctctctgtttgcagc

attagtttgttatctttgtatggactaaggtgtaatgtttccttcaatgtttgtattttg

tcag

GCATTGACTGATCCATACTTTAGAGGCCTTTCCAAGCCAGAGAGAGAACCATCCTGTCAG

CCAATCAGAAAAATGGAGTTTGACTTTGAGCACAGCAGAGTGTCAAAGGATGATATAAGG

GAGCTGATATTCCAGGAGATATTGGAATATCATCCGCAATTACTGAAGAGCTACGTAGAT

GGCACAGAGAGGACAACCTTTCTCTACCCAAG

gttttagtttttctatgtttctattccctcatttctgtttgctgtgtcaactcttactga

cagcaatatttcttggttatgtttgtcag

TGCTGTCGATCATTTTAAGAAGCAGTTCTCTCATCTCGAAGAAAGCGATGGTAGTGGCCC

TGTAGTCCCAGCAGATAGAAAACACGCATCTCTTCCTAG

gtaatgttgcatcgtactcttgtcttgtgttcatttcttttccttccatatcactggcac

gtaatcaaaccacttgctaatggcgcactttgaagtgcactgctcctttgcttcatatta

agatgggctgagtttcaaataatgggataattatcatgttatgaacttggtatgagttga

ttatctggatagtgaaccgggctatataaacttgagagtgaagtgaagtgacaggagttc

aaagatctactactgtaatctattttaaacttgagagtgaagttagtgggaaattaatac

ccggttttctcaacggagttggggttacaattgatatatgtaggttatcagatctgattg

ttaggtcctgtgcgatactttatcatcactttgttagtcgaggttctgttcgaaatgtaa

gtgagggcttaatcagtatgtgttcagaatgttgatactcgagacccatttcataatttg

gttgcttttttgccactcagttagcacacctgtatgcctttcatcattatatgttttata

ttagtacggtaagcctgcgttattgctgataagcatctttgtttttgcag

GTCCACTATTGTTCACTCGGCTCCAATTTCCGCGAAGGACACTCGGCCGCTCTTTGGCAA

GCCTTGCAGCAAAACTTCCTCGGAGACAGGGAGGTATGCTGGGAACGGTCGCGGTGCTTC

TCAGGCTTCACATGCGGCACAAGCAG

gtgcgtctgcgttcattctgctatacatattttgtcggcattcactcggaacttaatgtc

agacttgcttattttgcag

TGGTTTCACGGAGAGCTGCTGGTTCAGCGTTACCTTATGACGCGGCAAGCAGGCCGACGA

TTAGCCCAGGATGCCCTCCTCAGCAGCAGATCCCACAAATGTATGGGCAATATCAGCATC

AAGCACCTGCTGGTGCTGGGATACCACAGGCCATGGGGGGCTATGCCTGTGGTGGGTACA

CCAAAGGCACAGCGCCGAATGCTGCTGCTGCTCCAGCCATGAGAGCACCCCCCTACCGCC

ACCTTCCGGCGGGGCAGAAGAACGGTCCGCTGGACAGACTGGCGGTGGAGACCACCGACA

TATACACGCGGTCGCTCAACGGCATCGTTGCCGCCGCCGCAGCATCGGCTGGCACTGCCA

GCGCCCACAGGAAGGTTGGCGCCGTCCCGTTTGGCATGCCGACGACTTACTAGCGTCTGA

GAAGATGGCAGCGGCGCGTCTCTAGACACTTCGGCCGGAACCGTAGCAACCGGAGGGAGG

CATTGGCCTGAAATAAGTTGGCGGATGAGGATCGTCTGACAACGACGAAGGCATACAGAG

ATAGTAGGTCTGAAATCCTGTTCCATGTCGTCTCTAGGCTACACACAAAAAGACGGAGGG

TGGACCGTGGACGGGTGTGGCCAGACGAGCTGTTTATTTAGGTGGAAATGAAAATTAATT

AGCCGGTTAGCGGCCGGCTGGAGACAGGCCAACTCAACACAGAAATGAAGTCTGTACTGT

GCTACTGCTACTATCCAACAGACTGATTAATTAATTAAAGGGATGATGGTCCATTGATCA

TCATCATATGATTCATTTGCTCGAAAAAATAGTGCCCTTAAACTGAAACTG

**>TaMPK20-2(1BL): TRIAE_CS42_1BL_TGACv1_030959_AA0104540.1**

AAACCAACAAACAACAACGGCAAGTCGGCAGACATTCACAAAGAAGAGAAAGAAATCTAC

CTCCCCCCTCCCCTCCCCGACTCCGGCATCCCGCTCCCCTCCCTTCCCGTCTCCGGCGCG

CCGCCAGATCGGGCCAGAAGAGTCCGTCCGGCACCCATCCCTCGCTCCCCGCCGGCACCT

TGGGCCCGGCATCGGCCGCCGCGGCGCGTCAGTTTGGCGGGCGGCTCGGCCGACCGGGAG

GCGGGCGGTCCGGCGCCGCGGGAGGGCAGGGGTCCGGTTTCCGGTCTCCGGAGCGGCCTT

GGAGCGCAGATCGGCCCGCCCGGCAAGCGGCAATGCAGCAGGCGCAGCGGAGCAAG

gtgggttccgacttccgagccgtccctccccctcccccctcctcctcatgcgtctagtat

gagcaagatgatttgcgcctcttcttgcggcgtcgatgcccgctgcccgtctcggttcca

tccaggacgaagccggcgacccagcgttttctgaatcctcgctgccctgccctgttttca

aattcattcaaacctctttgacagattcttggctacttttgttgctttttcggcgccggc

aacccttcctgccgccgaaatctctgatctgctgtttccgtcagtaaaagttagcagtca

aatgccgtcggttccaatccaatacggcagcaccgcgtgatgagctgcagacagcccgcg

cctcacatggcaaagttgaccgcctcctcgccgcttcctgaatccagatgccaaacgcta

gacgaagtccgcaagatgccccttttcccctgcctgcccgtcggtggcgcgcaaagcttg

tactacattttaatccttgttggagtcctcgcagatgcccgccagcaacagcaagcaacc

ggcgttcagtggcacaaaaagttttaaactgtttgattgcttgccggtgctgtggctttt

ctcaatggctggttccgttgcaatgtggccactccaaatccctgtcggtctccaatgatt

cctccaacccccaccacagcaatcattgtgccgtctcagctccccggatcaaccgtttaa

agttctctctgttttagcagctgggtgttgagcgcttgctgattcattcagtgtgtgcaa

tccatgttttgcag

GGTTCGGCGGAGGCGGACTTCTTCACTGAGTACGGCGACGCCAACCGGTACAAGATCCAG

GAGGTCATCGGCAAGGGGAGCTACGGGGTTGTGTGCTCCGCCCTTGATCTGCAGACCAGG

CAGAAAGTGGCCATCAAGAAGATACACAATATCTTCGAGCATACCTCCGACGCCGCGCGG

ATCCTCCGTGAGATCAAGCTTCTGAGGCTCCTACGGCATCCCGACGTCGTCGAGATCAAG

CACATTATGCTGCCTCCCTCCAGAAAGGACTTCAAGGACATCTACGTTGTTTTTGAGCTC

ATGGAGTCTGATCTCCACCAAGTTATTAAGGCCAACGATGACTTGACGAAAGAGCACTAC

CAGTTCTTTCTCTACCAGCTACTCCGAGCCCTCAAATACATTCATACTG

gtgagaaatcgttaccttcaagtctactgttgttgcttagaaaacaatgttttaccctga

aggtggtgtgtgttttagtttacaatcatgtgtagtaggtcagatcctcttttcatctct

ttttcacttgcctatcaaacactcccccccagaggcttgctcttcaatattaaaaagtcc

aatgatgggtcagttatggtttcctcagtgtgcagaactagataatatacactgcggttt

agtcaacagatcctcagtaacagttgattctttaagtgccaaagtgtgacttgcttcgcg

tgtagtcaaccaagttaacaacagggtttatcatttgatattaaatcctgtgtattatca

tctctcaaccttcagttgcatctttctccgtttgctttgtgctacacattactacttcac

tattattaagatatgtatcatcctttatcagcatttttaaaatatgaccatattaaatcc

attcatatattagcctgcattctctgaatttcttgaattgttttttttccctttgaggcc

gtagatcatcccgtagctaaaacaatgacaataattatgaaacggagggagtagcagtca

agataagtaccagaccattatctgatgcaacctttgttcacctcttcaaatgaattattt

ttggtgttttgttcattatttatcccttttatattgtaggcgctctagccaagactctgt

aatttgattgctcgttggaaatcttttccacttacttgccatcaaaaagcttgtcatggt

gttgaattgacactaccagaaattgcaccccaatgtttttcagtgatacactgtagaaaa

cgttacttgattgcgtattctatttttattaccactttttttttgctgataaggagttca

tatgactgcag

CTAGTGTTTACCATCGTGACCTGAAGCCCAAGAATATTTTGGCAAATTCTAACTGCAAAC

TGAAGATATGTGATTTTGGACTAGCACGAGTTGCATTCAACGATACCCCAACAACTGTCT

TCTGGACG

gtatgttagtacaaactcaatttatgttttttatagcagatttttgatgaagtatcataa

tttatagatattatgcataatccctgcag

GATTATGTCGCGACAAGATGGTATAGAGCTCCAGAGCTCTGCGGATCCTTCTTCACAAAG

gtgaggtttagagttcatggctttgtgcttattaaaaaatgaagaaaagaactaggttct

taatctgttgacaggcttatagtgtaacaaagaagatgcttacaattgttctagcatgag

ttgattgaactgtgagctgtaacaggcaaatgtttatggtgaaaattaaaatgcactatg

tacctgttcatcctatgttttatagttatggtttctccatctatagtatagattagatct

gttcactgtcatttctatccttttgtattttatggcctaatttcattcatgaattgatct

taacatttgttcatcctatgttctacacgaatacttgatcactttcctcttgaatagtaa

ctttgactacatgtgaatttctcttacttccaagaacagtaaaagcatcttccgtaattt

ccaggattttgatcacatgcatgttcttaatctattttggatttttgtttcaccccaaaa

tgcag

TATACACCTGCCATTGACATTTGGAGCATTGGGTGCATTTTCGCTGAGGTGCTGACAGGA

AAGCCTTTGTTTCCTGGTAAAAATGTTGTCCATCAGTTAGACTTAATGACTGATCTCCTA

GGGACACCTTCAATGGATACAATTTCTCGG

gtacgctactaaattcaggggaagaatatatatttagttgatctggtaagcgtaatcagc

gagcccaacgtttgactctattatttttgttttttcctag

GTCCGAAATGAGAAAGCAAGAAGGTACTTGAGCAGTATGAGAAAGAAAGACCCGGTCCCA

TTTTCTCAGAAGTTTCCTAATGCAGATCCTTTGGGAGTTAAACTCTTGGAGAAGCTATTA

GCCTTTGATCCAAAAGACCGTCCAACTGCGGAAGAG

gtagtcttatggtgctattttgtctttctgatgccttggtttcttctctctgtttgcagc

attagtttgttatatatttatatggagtaagttgtaatgtttccttcaatgtttgtattt

tgttag

GCATTGACTGATCCATACTTTAGAGGCCTTTCCAAGCCAGAGAGAGAACCATCCTGTCAG

CCAATCAGAAAAATGGAGTTTGACTTTGAGCACAGCAGAGTGTCAAAGGATGATATAAGG

GAGCTGATATTCCAGGAGATATTGGAATATCATCCGCAATTGCTGAAGAGCTACACTGAT

GGCACAGAGAGGACAACCTTTCTCTACCCAAG

gttttagtttttctatgtttctattccgtcatttctgttcattgtgtcaactcttactga

cagcaatattccgcggttatgtttgtcag

TGCTGTCGATCATTTCAAGAAGCAGTTCTCTCATCTTGAAGAAAGCGATGGCAGCGGCCC

TGTAGTCCCAGCAGATAGAAAACATGCATCTCTTCCTAG

gtaatgttgcatcgtactcttttcttttgttcatttcttttccttccatatcactggcac

ataatgctaatggctcactttgaagtgcactgctcctttgcttcatattaagatggattg

agtttcaaataatgggataattatcatgttatgaatttggttagatttgattttatctgg

atagtgaactgggctatataaacttgagagtgaagtgactgggatctacgtgatcacagg

agttcaaagatctactactgtaatctattttaaacttgagagtgaagtgactgggaactt

aatacccggttttctcatcagagttggggttacaattgatatatgtaggttatcagatca

gattgttaggtcctgtgcgatactttatcatcactttgttagtcgaggttctgtttgaaa

tctaagtgatggcttaatcagtatgtgttcagaatgttgatactcgagacccacttaata

atttggttgcttttttgccactcagttagcacacctgtatgcctttcatcactatatgtt

ttaatattagtaaggtaagcctgtgttattgctgataagcatctttgtttttgcag

GTCCACTATTGTTCACTCGACTCCAATTCCCGCGAAGGACACTCGGCCGCTCTTTGGCAA

GCCTTGCAACAAAACTTCCTCGGAGACAGGGAGGTATGCTGGGAATGGTCACGGTGCTTC

TCAGGCTTCACACGCGGCACAAGCAG

gtacgccctcgtccatttttttatacatgttttgttgtcatttacttgaagcataatgtc

tcccatttctaagactatttcttattatgcag

TGATTTCGCGGAGAGCTGCCGGTTCAGCGTTACCTTATGACGCGGCAAGCAGGCCGACGA

TTAGCCCAGGATGCCCTCCTCAGCAGCAGATCCCACAAATGTATGGGCAATATCAGCATC

AAGCACCTCCTGGTGCTGGTGCTGGTGCTGGGATACCACAGGCCATGGGGGGCTATGCCT

GTGGTGGGTACACCAAAGGCACGGCGCCTAATGCTGCTGCTGCTGCTCCGGCCATGAGAG

CACCCCCCTATCGACACCTACCGACGGGGCAGAAGAACGGCCCGCTGGACAGACTGGCAG

TGGAGACCACCGACATATACACGCGGTCGCTCAACGGCATCGTCGCCGCCGCCGCGGCAT

CGGCCGGTGCCACGGGCGCCCACAGGAAGGTGGGCGCCGTGCCGTTTGGCATGCCGACGA

CTTACTAGCGGCTAAGAAGGCGGCAGCGGCGCGTACCTAGACCCTTCGGCCGGAACAGTA

GCAACCGGAGGGAGGCATTGGCCTGAAATAAGTTGGCGGATGAGGATCGTCCGACGACGA

CGACGGCGGCGAAGGCATACAGAGATAGTAGGTGCGAAATCCTCTTCCAAGTCGTCTCTA

GGCTACACACAAAAGACGGAGGGACGGATGTGGCCAGACGAGCTGTTTATTTAGGTGCAA

ATGAAAATTAATTAGCTGGTTAGCGGCCGGCTGGAGACAGGTCAACTCAACACAGAAATG

AAGTCTGTACTGTGCTACTGCTTACTATCCAACAGACTGATTAATTGATTAAAGGGATGA

TGGTCCATTGATCATCATCATATGATTCATTTGCTCGGAAAAATAGTTGCCCTTAGACTG

AAACTGCAATG

**>TaMPK20-2(1DL): TRIAE_CS42_1DL_TGACv1_063491_AA0227870.2**

AACGGCAAGTCGGCAGACATCACAAAGAAGAGAAAGAAATCTACCTCCCCCCCTCCCCTC

CGGCATCCCGCTCCCCTCCCCCTTCCCTTCTCCGGCCCGCCGCCAGATTAGATCGGGCCA

GAAGAGTCCGTCCGGCACCTATCCCTCGCTCCCCCGGCGGCACCTTGGGCCCGGCATCGG

CCGCCGCGGCGTACGTGTGAGTTTGTTGGCGGCTCGGCCGACCGGGAGGCGGGCGGTCCG

GCGCCGCGGGAGGGCAGGGTGCGGTCTCCGGAGCGGCCTTGGAGCGCAGATCGGCCCGCC

CGGCAAGCGGCAATGCAGCAGGCGCAGCGGAGCAAG

gtgggttccgacttccgagccgtccctccccctcctcctcctcctcctgcgtctgcctag

tagagacgatttgcgcctgttcttgcggcgtcgatgcccgctgcccatcttggttccagg

acgacgccggcgacccagcgttttttgaatcctcgctgccctgttttcaaattcattcaa

acttctttgacagattcttcgctacttttatcgggatttcggtgctgccaacccttcctt

tagccgaaatctttgatctgtatttctgtcagtagaaaattagtactcaaatgccgtcgg

ttccaatccaatacggcaacaccgcgtgatgagttgcagacagcccacgcctcgcatggc

aaagttgaccgcctccttgccgcttcctgaatccagatgccaaacactagacgaagtccg

caagacgccgcttttcccctgcctgcccgtcggtggtgtgcgaagctcgtacattttaat

tcttgttggagtcctcgcagatgcccaccagcaagcaaccggcgttcagtggcacaaaaa

gttttaaactgtttgattgcttgctggtgctttggcttttcttaatgcccggctctgttg

caatgtggccactccaaatctctgtcggtctccaatgattttctccaacccccaccacac

caatcattctgctacttcagttccccaaaccaaccgtttaaagttctctctattttaaca

gctgggctttgagagtttgctgattcggtgtgtgcaattcatgttttgcag

AGTTCGGCGGAGGTGGACTTCTTCACGGAGTACGGCGACGCCAACCGGTACAAGATCCAG

GAGGTCATTGGCAAGGGGAGCTATGGGGTTGTGTGCTCTGCCCTTGATCTGCAGACCAGG

CAGAAAGTGGCCATCAAGAAGATACACAATATTTTCGAGCATACCTCCGACGCAGCGCGG

ATCCTCCGCGAGATCAAGCTCCTGAGGCTCCTACGGCATCCTGACGTCGTCGAGATCAAG

CACATTATGTTGCCTCCCTCCAGAAAGGACTTCAAAGACATCTACGTTGTTTTCGAGCTC

ATGGAGTCTGATCTCCACCAAGTTATTAAGGCCAACGATGACTTGACAAAAGAGCATTAC

CAGTTCTTTCTCTACCAGCTACTCCGAGCCCTCAAGTACATTCATACTG

gtaagaaatcgttaccttcaagtctactgttgttgcttggaaaacaatgttttaccctca

aggtggtgtgtgttttagtttacaatcatgtgtagtaggttagatcctcttttcatctct

ttttcacttgcctctgaaacactccccccccagaggcttgctcttcaatattaaaaagtc

taatgatgggtcagttatggtttccacagtgtgcagaactagataatatacactgcgtgc

ggtttagtcaacagatcctgagtaacagttgatactttaagtaccaaaatttgacttgct

tcgagggtagtcaaccaagtttggttaacaacagggtttatcatttggtattaaaacctg

tgtatcatcatctctcaacctacagttgcatctttctccgtttgctttgtgctacgcgtt

gctagttcactatctttaagatacgtatcatcccttatcaacaattcttaaaatatgacc

atattacatctattcagatattagcctacattctctgaattcctcaaagtgtcttttttc

ctttgaggccatagatcatcccgtagctaaaacaacgacaataattattaaacggaggga

gtagcagtcaagataagtaccagtccatatctgattcaacctttgttcacctcttgaaat

taaatattttggtgttttgttcattatttatccctttatatgtaggcactgtagccaaga

ctctgtaatctgattgctcgttggaaattttttgcacttacttgccatcacaaagcttgt

catttgtttgaattgacactaccagatattgcacccgaatgtttttctttagtgatacac

tgtagaaaatgttacttgattgtgtattatatttttatcaccactttttttgctgataag

gagttcatatggctgcag

CTAGTGTTTACCATCGTGACCTGAAGCCCAAGAATATTTTGGCGAATTCTAACTGCAAAT

TGAAGATATGTGATTTTGGACTAGCACGAGTTGCATTCAACGATACCCCAACAACTGTCT

TCTGGACG

gtatgttagcacaaactcaatttatgttttttatagcagatttttgatgaagtatcataa

tttatagatattatgcataatccctgcag

GATTATGTCGCGACAAGATGGTATAGAGCTCCAGAGCTCTGCGGATCCTTCTTCACAAAG

gtgaggtttagagttcatggctttgtgcttattaaaaaatgaagaaaagaactaggttct

taatctgttgacaggcttatggtgtaacaaagaacatgcttacaattgttctagcatgag

ttgattgaactgtgagctgtaacaggcaaatggtttatggttctgtatcattgtcttcgc

tttcatcttctcttatgataggttttctggtgaaaattaaaatgcactatgtacctgttc

atcctatgctttattgttatgctctttttttttgtcgaagttataaatgacctatgcttt

attgttatggtttctccatcttttgtatagattagatctgttcgctgtcatttctatcct

tttgtattttgtgaccaatttcattcatgagttgatcttaacatttgttcatcctatgtt

ctacatgaatacttgatcgctttcctcttgaatagtaactttgactacgatcacgtgaac

ttctattactcccaaaaacagcaagtgcatctttagtaatttccaggatttttatcagat

gcatgttcttaatctgttttggatttttgttccccccaaaatgcag

TATACACCTGCCATTGACATTTGGAGCATTGGGTGCATTTTTGCCGAGGTGCTGACAGGA

AAGCCTTTGTTTCCTGGTAAAAATGTTGTCCATCAGTTAGACTTGATGACTGATCTTCTA

GGCACACCTTCAATGGATACAATTTCTCGG

gtacggtactgaattcagggaaagaatatatatttagtttctctggtaaatgtattcagt

aagcccaaactttgactttactgtatttgttccttcccag

GTCCGAAACGAGAAAGCAAGAAGGTACTTGAGCAGTATGAGAAAGAAAGACCCGGTCCCA

TTTTCTCAGAAGTTTCCTAATGCAGATCCTTTGGGAGTAAAACTCTTGGAGAAGCTATTA

GCCTTTGATCCAAAGGACCGTCCAACTGCGGAAGAG

gtagtcttatggtgctattttgtctgtctgataccttggtttcttctctctgtttgcagc

attagtttgttatatttgtatggagtaagttgtaatgtttccttcaatatttgtattttg

tcag

GCATTGACTGATCCGTACTTTAGAGGCCTTTCCAAGCCAGAGAGGGAACCATCCTGTCAG

CCAATCAGAAAAATGGAGTTTGACTTTGAGCACAGCAGAGTGTCAAAGGATGATATAAGG

GAGCTGATATTCCAGGAGATATTGGAATATCATCCGCAATTGCTGAAGAGCTACATTGAT

GGCACAGAGAGGACAACCTTTCTCTACCCAAG

gttttagtttttctatgtttctattccctcatctctgtttgctgtgtcaactcttactga

cagcaatatcttggttatgtttgtcag

TGCTGTCGATCATTTTAAGAAGCAGTTCTCTCATCTTGAAGAAAGCGATGGCAGCGGCCC

TGTAGTCCCAGCAGATAGAAAACATGCATCTCTTCCTAG

gtaatgttgcatcgtactcttgtcttgtgttcatttcttttccttccatatcactggcac

gtaatcaaacgacttgctaatggcgtactttgaagtgcactgctcctttgcttcatatta

atatgggttgagtttcaaataatgggataattatcatgttatgaatttggtatgagttga

ttatctggatagtgaaccgggctatataaacttgagagtgaagtgactgggatctatgtg

atcacaggagttcaaagatctactactgtaatctattttaaacttgagagtgaagtgagt

gggaaattaatacccggttttctcaacggagttggggttacaattgatatatgtaggtta

tcagatcagattgttaggtcctgtgcgatactttatcatcactttgttagtcgaggttct

gttctgaaatctaagtgagggcttaatcagtatggtgttcagaatgttgatactccagac

ccacttcataatttggtcgcttttttgccactcagttagcgcacctgtatgcctttcatc

attatatgttttaatattagtacagtaagcctgcgttattgctgataagcatcttcattt

ttgcag

GTCCACTATTGTTCACTCGACTCCAATTCCTGCGAAGGACACTCGGCCGCTCTTTGGCAA

GCCTTGCAACAAAACTTCCTCGGAGACAGGGAGGTATGCTGGGAATGGTCACAGTGCTTC

TCAGGCTTCACATGCGGCACAAGCAG

gtgcgtctgcgttcattctgctatacatgttttgtcagcattttttactcgaaacttaat

gtctcattaataagaccatttcttattttgcag

TCGTTTCACGGAGAGCTGCTGGTTCAGCGTTACCTTATGACGCGGCAAGCAGGCCGACGA

TTAGCCCAGGATGCCCTCAGCAGCAGATCCCACAAATGTATGGGCAATATCAGCATCAAG

CAGCTCCTGGTGCTGGTGCTGGGATACCACAGACCATGGGGGGCTATGCCTGTGGTGGGT

ACACCAAAGGCACGGCGCCTAATGCTGCTGCTGCTCCAGCCATGAGAGCACCCCCCTACC

GCCACCTTCCGGCGGGGCAGAAGAACGGTCCGCTGGACAGACTGGCGGTGGAGACCACCG

ACATATACACGCGGTCGCTCAACGGCATCGTCGCCGCCGCGGCGGCATCTGCCGGCGCCA

CGAGCGCCCACAGGAAGGTGGGCGCCGTGCCGTTTGGCATGCCGACGACTTACTAGCGGC

TAAGAAGACGGCAGTGGCGCGTCTCTAGACTCTTCGGTCGGAACCGTAGCAACCGGAGGC

ATTGGCCTGAAATAAGTTGGCGGATGAGGATCGTCTGACGACGACGAAGGCATACAGAGA

TAGTAGGTCTGAAATCCTCTTATAGGCTACACACAGAAGACGGAGGGTGGACCGTGGATG

GGTGTGGCCAGACGAGCTGTTTATTTAGGTGGAAAATGAAAATTAATTAGCCGGTTAGCG

GCCGGCTGGAGACAGGCCAACTCAACACAGAAATGAAGTCTGTACTGTGTTACTGCTACT

ATCCAACAGACTAATTAATTAATTAAAGGGATGATGGTCCATTGATCATCATCATATGAT

TCATTCATTCGTAGATCTACCATCCATGCTTTTTTTTGCTCGAGAAAATAGTTGCCATGG

GAGGAGTCCGTTAAGAGAGACCTGAAGGACTGGAGTATCACCAAAGAGCTAGCTATGGAC

AGGGATGCGTGGAAACTTGCTATCCATATGCCAGAGCCATGAGTTGGTTGCGAGATCTTA

TGGGTTTCACCTCTAGCCTACCATAACTTGTTTGGGACTAAAGACTTTGTTGTTGTTGTT

GTTGCCCTTAGACTGAAACTGCAATGCCCTTGACTTTCTGCATCTACAATATTCATG

**>TaMPK20-3(7AL): TRIAE_CS42_7AL_TGACv1_557823_AA1786610.1**

ATTACAGTACATTTTTGGTCTAAAGAGAGCCCGAGCCTTGTGATCTACCTTTGGAAACGG

CGAAACGCCACCGAGAACCCACGAGATATAGCTGCCACGCACGCGGAGCTCACCTCACCA

CGCGGAGAAAACAGAGGAGAAAGAGAAGAGAAGAGAAAATATCCCTCCCACCTCGTCACC

CCAGCTTCCTCCGCTGCCTCACCACAGCACGCCGACGAGCTCTCTCCAGTCACCACGCAC

GACGCCTCCTCTCCCCTTCCCCCAGATCCGAAGCGACTGCCGCCGGCTCGCGATCTTCCC

GAACCGTACAAGAACCCGGCCGTGGCGGCGGCCGCGGCAGCGACGTACGGTGGCGGCGAG

GGGTGTATGAGGAGCAGGAACGACTGCGGGACCGCGATTCGGAGCAGGAGCCGGTGGGGC

AAGTGCGGCTGACCCGCGCCTGAGCGAGAGGCCGGTCTGTGTGTCTGGTGGGATCGTGGG

AGGAGGGAGAAGAAAGAAAGAAAGGTCGATCTTGGGAAGCTTCCACTACGGGCTCCGAGA

TGCAGAACGGCGATTTGCGCAAGAAG

gttcgtgctcttggattctttggctttgcttgtttagacgggagtagtgaaaatgttggg

aatgatggcaattcgagtaagaggtccagatggttttaatattagtttaagcgaataaaa

ctaggtgacatgctgtgactttaggacatttttgtttggtaatgttgttattctacaaag

tagctactggttataaggacacctccttcagtctttggtaataatttatttttcccacat

gctacaaagagagtgaattgtaaatttgtaatgtcagaggtgtgcgcagttcttggctct

ggagtatgtgattacatcccaaaggaatatctttatacgtacctggtagtaacagcggaa

aacctttgctccagtccactgtttcgttggaagctatatgtcatactgacactagatgat

taagttgtaggtggagagggacttgctcaggcagttctttctctggttttgtcattaata

ccactgaacgtctgtgattaacttgcagcagtgtttttgatttggaaatgtatctttgac

cccttaaactattggccgggtgtagatctggtcccacagtcagacatttttgcccttgaa

ctgccatttcgtgtacttttggtccctcaaggcggtttgaccatttcaaagcggttttta

ctgatgcggcgccagcaccatgtgtctgccgcatcactaatctcattctaaaaatccaca

aacctatttaaaaagcagagaaacaaaacaaaaaaagaaaaatataattaaaaatgatat

ttcatattctgtatttacagggaatttgagagaaaaaaggagcaatccgacattttttgg

tttattagaagaaaagcaatttaataaaagaatcataatctccttctatttgcaatttct

tgatttttcttttgactatttttttgttttcttgatttttgaaaatctgaggtttttgta

aataaaaagactttctcgagtttttttaccccattatgctgtatatgattttactctttc

cccctttctgatagttttctccatttcttcaaaactgatgtctttttactagattcgttg

atatcttcgtttgaagtatatatatattatttgcctttttctgcttcttacttaaatttt

aggaatttccgggaggaaattggtaagactgcagatatgtgatttggcctacatgtagat

gccacatcgatcaaaaccattttggaacagtcaaaaccacctcgatggagcaaaaatata

cagtattgagagttgaagggtgaaagtatccgttttaggagttgacgaccagatctacac

ccaaaataatagtttaggggtcaaaagtacacattttccttttgatcatagagtaaccaa

gtggtagtaccatcatcgatggtcattgtcttctgtgtttcgtaggtttaatgtgcaaca

gtattagttatctgactattttctctaattcag

AGTGCAGCGGAAATTGATTTCTTTACTGAATATGGCGATACTAATCGATACAAAGTTCTG

GAGGTCATAGGCAAAGGTAGTTATGGACTTGTATGTTCTGCAAATGATACACAAACAGGA

GAGAAGGTTGCAATAAAGAAGATACACAACATTTTTGAGCATATATCGGATGCTGCACGC

ATACTCCGTGAAATCAAGCTTCTCAGGCTTCTTAGGCATCCTGATGTAGTGGAAATAAAG

CATATCTTGCTCCCCCCATCCAAAAAGGATTTCAAAGATATATATGTTGTCTTTGAACTT

ATGGAGTCAGATCTTCATCAAGTAATAAAGGCTAATGATGATTTGACGAGGGAGCATTAT

CAGTTTTTCTTGTATCAGATGCTTCGAGCTTTGAAATATATGCACACAG

gtatgcacttcctcacagtctttttatgaagataaatgttacgtgcgcttctattcttat

ggaacgaccatgtagctatttgactattgtttctttcctgcacttgcattgttgtatgct

aatttgcatgcaaatttcacttgtaaccatttgatgatttgattcatcccttttccccct

tcctgttctttcttttattgagatggcactggaacaattacatgagttctaattaacatc

tattgaacgggtcgactgatttaaagtttggtacagtttggtcgagatgatagtcgttct

gaatgcacgccatggcctgcatctaaggcctggtttgaaatgaagcaatgctgttgtagc

atgtaggcttgttattgcaatatgctcctgtgccagtgtatacgcgatggaccccgacca

ctgatgtttatggccccaacctatttgctgtatcattgtgtatgacaatcacctgtgttt

atggtgtcttgttctgcatttgcccactaaggcatgctgagtatctgggtattgattact

gatgcagcattgcacatgacaactcagatatttagttgcacaaataaaagtaaaaggttg

ctcctttattttttgaacccgtgtcaactttggctagtttggtatttaccgaaaccatag

catatttggttccatactctatttgtatttgcatggttgatttttaatgttggaaattat

ataatatcatttcagttgctacatatgttcagaattggctaaatattgtttgtgctttga

tgcag

CAAATGTCTATCACCGAGATTTGAAGCCCAAGAATGTGCTTGCCAACGCAAATTGCAAAC

TCAAAATTTGTGACTTTGGCTTGGCGAGAGTTGCATTCAATGATGCACCTACGACAGTCT

TCTGGACA

gtaagcaatatcttttgttattgtatctcaatagcatatgttattggttgatcttgcagt

actaacaattggagatttactagcaccgaatcatgctttgttccccgttgtcatgttgaa

cttaaggttgtcattcgagacgcaatggttctaacaaagggaaagtgaatatctaatgat

cagtgcactgtcacatacaagttgaaataagatgtcatctagttttacttttaagtgggt

cttcacgaagccatttcctcattttggttttagggtgggattggggtcgacggttctgaa

gctaaccattaatttagggccttggggctgctctggagtcttccattgctcatccaatgt

tttctattcctgtcaaatgaacttctaaattagaataaacttcgcttttcag

GATTATGTGGCAACGAGATGGTATAGAGCACCTGAACTTTGTGGGTCCTTCTATTCTAAG

gtaagcaatgttacaaacttatgatttatcagaagttagagaggttgacatgtatacata

tagatgatgccgacctaataggcattatttttgtgtgatggtactggtactggtgctggt

aaatgacattgttgggatgcgggtctggaacttgatgtccggtgcctgttgatcacctca

ccttgtagtgagtatgagggaattggccatgaggtgagtatgagggaaattacagcagtg

acggcagcagcaagataaacagtacattgtaattgagtttaaatggtaaacagtaaagat

actcttttgcccgcgcccatctggactgtccattgcttctcgtcttgatcaatctggatc

attggttaactgacgaagtgccaataggaatacaacactagtgggttcagtcagggtcct

gacagtcggagtcacctcacattgtagtgagtattgagggaattgccatgaggcggcttt

tgacttctttttaatatttggatttgtctgtcttatatgcctatagcaccaaaaaaaaag

tgtaatgcatcgtttacacctgtatgcgcaaatttgagcaaaaaagttgatgccatgaac

tcatcatatacttgtaaatcagcaaatgtcccatggcaaattggcaataaagaagtaaca

ctaatgattcataagcccatattgtatgctgttcaaacttgtaactgtgctgtcatatgg

ctgcaaacaatagaatgatggatagcactgcaaggttggaactccatgtctttgcgtgta

tcactaaatgtttgtatacatattgtacaccaaggaccactgcagttttagaccacatca

ttaccgatactctaatatagttgtacacttgtactagtagctcctcatggttcctccact

agcacctctggacaatcacaagttgcgattgtccttacctgacagcctcgcaacaacacc

tctcaaggtatccacacgtataaaagcgggaacgctgtgtgctagcctagtagcctgcta

gggtgctagcactgacaagcacggccaggctttcatgggtgggagtggtggctaggcttt

ctgaagtcccacgaatttgtgcccctaatgccaacatgcctccctttgcatagattcccg

tggtaggtctccacattggaggctcactgagcccagtaggtaccagaccactttccgaaa

cctgttccagcctgttagtgtttgtcgcacataaataacttgaatgggaactaaaccatg

tggcaactatcttgccataccatgttcatattccatattggggaaacatgaatagagttt

gaattataattttaataggaatacggttttgcttactaactaccattcagatgtggctac

cgtcgagtttgaattattttctgcacaatttacctgtttatacaccctcttaatttacag

tagagaaggtagatagcaactatcactaacaaccttccgtaagtaaatggatcccttcag

cacaatttcttttgctcatgctgcttattgcaaagttggaaacatatccgttacttgagc

aactgaacagtgcatagcattagttgggttaaagaaatattcaaatggtcccgattggaa

gttgactgtcccaattacaaatcgtcttatttggtattgaccacagttgtggcattattt

ttctgttatatttcttttgtaaacaagatcttcccccggtcctgatttgagttggtatgg

ccaattgttcgtgtcactgaaatttgccatcttttgggttgcag

TATACACCAGCTATTGATATATGGAGTATAGGGTGCATTTTTGCCGAGGTGTTGATTGGA

AAGCCTCTATTTCCTGGTAAAAATGTTGTTCACCAGCTGGATTTGATAACTGATGTTCTG

GGGACACCTTCATTAGATGCTATTTCTCAG

gtatggatctttggaagtttgctgtcaaagttatatgtagttctgtctccttaaaatttt

ctactgtcctacctctgctaag

GTGCGGAATGACAAGGCAAGAAAATATCTGACATGCATGCGGAAGAAACAGCCTGCTTCG

TTTTCGCAGAAATTTCCAAAGGCTGACCCATTAGCATTACGATTGCTTAGGAGGCTTCTA

GCTTTTGATCCAAAGGATCGTCCCTCTGCTGAAGAG

gtatgccatatatatgaatcttaaccggtcccatgttctgcatctgccagtacaagatat

tgtgatcttgatatatctgcaaattgtag

GCATTGGCAGATCCATACTTTAATGGACTAGCAAAGGTAGAGCGAGAACCATCTTGTCAA

CCGATACCAAAAATTGAATTTGAATTTGAGGGTCGTAGAGTAACAAAGGAGGACATCAAG

GAACTGATCTTTGAAGAAATTTTGGAGTATCATCCTCAATTACTGAAGGAGCATATCAGT

GGAACAGACAGACGAAACTTTGTTCATTTAAG

gttagatatgttattgcacacttgtttatcttctcttagtcatcatccatgtctgatacg

cttgcatcttctggtattgcag

TGCTGTCGACCAATTTAAGAAACGCTTCGCTGAACTCGAGGAAAATGGTGGCGAAAATGG

ATCAGCTGTTTCATCACAGAGGAAACATTCTTCTTTGCCAAG

gtaaatgtcttatttccaactcaaatctcttttactgggatgggatctagacagctaatc

atttatacaagggatgatccagtcataatatgtattgatatcaccaatgatgcaacatga

agaataaaacatccattttcaaactgcttgcaccctttcttttggcaaaagtctcagtcc

cattcaagcatctagtcctaactaacagactttgttctttcttcattactttcttatatt

gctggttctaattttgtaattgttgtaaaatgaaagctttattacccccggcctctgcat

cgctgatacatgcagccctgattattacattatttattacaagccatcaaaaagcaacat

aaccacacgaaacatttaggcctaatgaaaaagacaggccacacatagcatcactagaga

gtacagactgagaagcctatcactacttcactagcacaccatccaaaccaggacataaac

tccctggtaagccactctagcttcttggtgccaagctctgttgtgctgcaccttctgtaa

caatgcccatgattgcagccaactgatacacgatcatagcacctgcctgcaagattggac

cttattgtaaaaaataatactccctccaatccaaattaatcgaggccacgcttggattcg

ttgtaaataaatacagaggtgtttattttacacctgtatcttaccgtccgctcagcaatt

ctcggccggtaacaaagcgatgagccgtgacctatattctttacaggtgtatttgaaaag

aacaggacaatccaaacagggcctgacgcagcctctatacaacattgtacagaggttgta

tagagaccgtgtcaattaatttggatcagagggagtactgtttctacaaagccaaataga

ccaaaaaactgctccagccccgatcataataaagtgcttgtagcgatttatctacccccc

ctgcagccacgtcccaaatatatgtgaaatactacaggatgcagtaatgttaaatgctaa

ataaacagcctgccatgcaatgcatgctaacacgcaataaaagaatgctgaatggtctcc

tgttcatagcaaaagccgcgttgcgcgtcccgctgtcatttacatttaagtatttaacca

aattatcttgtgtgaggataacccccggcaaagaaacggaaggaacaccttgattgctgg

ttctaatggtcagtagaaagtagaaattcgcggcactgttttcctgaagtataaaatgtt

aaggttgatcttgacaaagaatcattgaaggtttcatctaaatcaaaccatcgtgcttgt

gaacctcgattaatgagaagtagttgaaaaaggtcaaatctttctccaaattttccgttt

ctagtagctataatgcacattttccctagcaatgattgacatgactaaggtatgtgcgtc

tttttctcctttgaagctagtattaaataaaattaacatgcagagtgactgttaagatta

agatgatcctagtgagacccgggaagatttagtttcttgtgatgtttttctgtcaatgtg

tcttgcttgaaattctgacaattgaagacctatgacaggtagtctagtttggccatccaa

ctgttggagtattcggtgccatcttttgatgcaatactctgatatataacatcctgcctc

tccgacatgtgtttcctcttatagtccagtttggccatccaactgttggagtattcggtg

ccatcttttgatgcaatactctgacatataacatcctgcctctccgacatgtgtttcctc

ttatagtaccccccccccccnnnnnnnnnnnnnnnnnnnnnnnnnnnnnnnnnnnnnnnn

nnnnnnnnnnnnnnnnnnnnnnnnnnnnnnnnnnnnnnnnnnnnnnnnnnnnnnnnnnnn

tttcactaccatcaaagagagttaaaaatcttctggatgttgttgtggggttactaatgc

ctttcttttgcag

GCAATCCTTCAACCCCCATTAGACGTGTAATGACCTGTGACAGTGAAAACTCACAGGATA

TGAAGATCCAAGAAGCTTACCATCTGTACTCCCTCGGCAACCAAAATATGAAGCACCGCA

CTCTCAGAACCAAGTATCTAGTGGGTCAGTGCTTGATGCGACAGGTCCAACTCCCAACAC

AATATGTACATGGAGGAGTATATGTAGACTCCAGAACAGGGAACTTACTTCTATCTGCAT

CATGGTATAAGGGTTGATATACCAGGTGAGGAGTATATGTAGACTCCAGATCAGGGAACT

TACTTCTATCTGCATCATGGTATAAGGGTTGATATACCAGGTGACAGTATCGCACAGGCA

GTCTCTGTTCCTCATGGCTCAGTTCTGGCTGTATGTTGCAGCATGGCGTTTCCAGGGATG

TTTCGAGCCTAGGGTTATTTGGCATTGAATCATGGAGGTCTGGCTTTTTTATTTGTTAGT

CCCCCAAATCATGGAGGTCGAACCTAGGATTCCAGCTTCATCTTGTACATGGGATGTTTC

ACAGCTTCTTTGCAACCAAGCCCCCCAAATGTACCACACAGTTGGATCATAATGTGCCTC

CTAGGGATTGAGTTGCAAAGCTCGTTTGTTCTGAATGCTTAAACTTTTTCCATGGGAAGC

TGTGAGGTGGCTAGGTCAAAGAATAGGATTGTCGTGATTTACTTCAACCGCTAATATCAG

TTGTTTAGGAAATACTCCTGCATTTTAACGCGAGGGACCTTGCTGTGTTTAGCAGCGTGT

TAACCAAAGGAACTTGAGCATATAATCTGAATCATTTCCAATAGTTACTGTAAAGGAATG

TGCGAAGGTCGGATGCCATAGACCAAATTAACTGTTCCGGTGAGCTGGGTAAATAAACTG

TTCTTTTCCATC

**>TaMPK20-3(7BL): TRIAE_CS42_7BL_TGACv1_577050_AA1864020.2**

ATCCCTGTCATCAATGTATTTTCAGTCTAAAGAAATTATATTTTTGGTCTAAAGAGAGCC

CGAGTCTTGTGATCTACCTTTGGAAACGGCGAAACGCCACCGAGAATCCACGAGGTATAG

CTGCCACGCACGCGGAGAAAACAGAGGAGAAAGAGAAGAGAAAATATCCCTCCCACATCG

TCACCCCAGCTTCCTCCGCTGCCTCACCACAGCACGCCGACGAGCTCTCTCCACGCACGA

CGCCCCCTCCCCCTCCCCCCAGATCCGAAGCGACTGCCGCCGGCTCGCGATCTTCCCGAA

CCGTACAAAAACCCGGCCGTGGCGGCGGCCACGGCAGCCACGTACGGTGGCGGCGAGGGG

TGTATGAGGAGCAGGAACGACTGCGGGAACGCGATTCGGAGCAGGAGCCGGTGGGGCAAG

TGCGGCTGACCCGGCCTGAGCAAGGACATCCACGGCCGGTCTGTCTGTCTGGCGGGATCG

TGGCAGGAGGGAAAAGAAAGAAAGAAAGGTCGATCTTGTGAAGCTTCCACCACGGGCTCC

GAGATGCAGAACAGCGATTTGCGCAAGAAG

gttcgagctcttgtattctttggctttgcctgtttagatggatgggtgtagtgaaaatgt

ttgggatgatggcaattcgagtaagaggtccaaatgggtttaatattagtttaagcgaat

aaaactaggggacatgctatgactttgggacatttttgtttgctaatgttgttattctac

aaagtagctgctgtttctagggacacctccttcactctttggtagtacttcctctgtaaa

ctaatataagagcgtttagatcactactctcttatattagtttacagagggagtaataat

ttatttttccacatgctacaaagtaagtgaattgtaaatttgcagtgagaggtgtgcgta

gttcttggctctggagtatgtgattacatatcaaaggaatatctttatatctacctggta

gtaacagcggaaaacctttgctccattccactgtttgcttgcaagctagtatgtcatact

gacgctggatgattaatttgtaggcggagagggacttgctctggttttgacatcaatacc

actgaacatctgtgattaatttgcagcagtgttttgattcggaaatgtatctttggcccc

ttaaactattggctgggtgtagatctggtcccacgaccagatatttttgcccttgaactg

tcatttcgtgtactttttgtcactcaagacgggttgaccgtttcaaagcggtttttactg

atgcagcgccagcaccatgtgtctgctgcatcactaatctcgttccaaaaatccacaaac

ctatttaaaaagcagagaaagaaaacaaaaaaggaaaatataataaaaaatgatatttca

tattctgcatttacaaggaatttgagagagaaaaaaggagcaaatccgacattttttggt

ttattagaagaaaagcaatttaataaacgagtcataatctccttttatttgcaatttctt

gattttccttttgactattttttgttttcttgatttttgaaaatatgagtttttgtaaat

aaaaagagtttctcgagtttttttaccccattatgctgtgtatgattttactcttttttc

ccctttctgataattttctccgtttcttcaaaactgatgtctttttgctagatgcattga

tatcttcgtttgaagtatatatatattttgcctttttatgcttcttaattaaattttagg

aatttctgggaggaaattggtaagactgcagatatgtgatttggcctacatgtagatgcc

gcaacgatcaaaaccattttggaacagtcaaaaccacctcgattgagcaaaggtatacag

tactgagagttgaagggtgaaagtaccgttttaggagttgacgaccagatctacacccaa

aacaatagtttaggggtcaaaagtacacattttccttttgatcatagagtaaccaagtgg

tagtaccatcatggatggtcattgtcttctgtctttggtaggtttaatgtgtaacggtat

tagttatctgactattttctctaattcag

AGTGCAGCGGAAATTGATTTCTTTACTGAATATGGCGATACTAATCGATACAAAGTTCTG

GAGGTCATAGGCAAAGGTAGTTATGGACTTGTATGTTCTGCAAATGATACACAAACAGGA

GAGAAGGTTGCAATAAAGAAGATACACAACATTTTTGAGCATATATCGGATGCTGCACGC

ATACTCCGTGAAATCAAGCTTCTCAGGCTTCTTAGGCACCCTGATGTAGTGGAAATAAAG

CATATCTTGCTCCCCCCATCCAAAAAGGATTTCAAAGATATATATGTTGTTTTCGAACTT

ATGGAGTCAGATCTTCATCAAGTAATAAAGGCTAATGATGATTTGACGAGGGAGCATTAT

CAGTTTTTCTTGTATCAGATGCTTCGAGCTTTGAAATATATGCACACAG

gtatgcacttcctcagtcttcagtttatgaagatcaattccatgcgcttctattcttatg

gaaccaccatgtagctatttgactattgtttctttcttgcacctgtattgttgtatgcta

atttgcatgcaaatttcacttgtaaccatttgatgatttgattcatcccttttccccctt

cctgttgtttcgaacaattacatgagttctaattaacaatctattgaacgggtctactga

tttaaagtttggtacaatttggtcgagatgatagtcgttctgaatgcgcgccatggcctg

catctaaggcctggtttgaaatgcagcaatgctgttgtagcatgtaggcttgttattgca

atatgctcctgtgtctgtgtatacgcgatggaccccgaccactgatgtttatggccccaa

cctatttgctgtatcattgtgtatgacaatcacctatgtttatggcgtcatgttctgcat

ttgcccactaatgcatgctgatctgagtatctgggtattgattactgatgcagcattgca

catgacaactcaaatatttagttgcacaaataaaagtaaaaggttgctcctttatttttt

gaatccgtgtcaactttgactagtttggtatttaccgaaaccatagcatatttggttcca

tactctatttatatttgcatgtttgatttttaatgttggaaattatataatatcatttca

gttgctacatatgatcagaattggctaactattgtttgtgctttgatacag

CAAATGTCTATCACCGAGATTTGAAGCCCAAGAATGTGCTTGCCAATGCAAATTGCAAAC

TCAAAATTTGTGACTTTGGTTTGGCGAGAGTTGCATTCAATGATGCACCTACGACAGTCT

TCTGGACA

gtaagtaatatcttttgttattgtatctcaatagcatatgttattggttgaccttgcatt

actcaaagtttctgtcttgattgttttaagttccgactgccccttcgatcagctatttat

cacagttcgtaattgattgtattgtcatgtgacaacacattacatggtgcagctaacaaa

tagccggtggtgtatttactcccaacattattcttgtgacacccactgttagtcatatta

ttaactaacaattggagatttactagcaccgaattatgctttgttccctgttgtcatgtt

gaacttcaggttgcctcattcgagacgcaatggttctaacaaagggaaagtgaatatcta

atgatcagtgtactgtcagtctgtcacatacaagttgaaataagttgtcatctagtttta

cttttaagtgggtcttcacgaagccatttcctcattctttggttttagggtgggattggg

gttgacggttgtgcagctaaccattaatttagggctttggggctgctctggagcctttca

ttgctcatccaatgttttctattcctgtcaaatgaacttctaaattaaaataaacttcgc

ttttcag

GATTATGTGGCAACGAGATGGTATAGAGCACCTGAACTTTGTGGGTCCTTCTATTCCAAG

gtaagcaatgttacgaacttatgattaatcagaagttagagagctcgacatgtatacata

tagatgatgccaacctaataggcattatttttgtgtgatggtactggtgctggtaaatga

cattgttgggatgtgggtctggaacttgatatcccgtgcctgttgagtcacctcaccttg

tagtgagcatgagggagttggccatggggtgagtatgagggaaattacagcagtgaagca

gcaagataaacagtacactgaaattgagtttaaatggtaaacagtaaagatactcttttg

ctcgcgccatctggactgtccattgcttcttgtcttgatcagtctggatcattggttaac

tgaagtgccaataggaatacaacactagtgggttcagtcagggtcctgacagtcgagtca

cctcacattgtagtgagtattgagggaattgccatgaggcgcttttgacttctttttaat

atttggatttgtctgtcttatatgcctatagcacccaaataaaattgtaatgcatcgttt

acacctgtatgcgcaaatttgagcagaaaagttgatgccatcaactcatcatatacttgt

aaatcagcaaatgtcccatggcaaattggcaataaagaagtaacactcatgattcataag

cccatattgtatgctgtttaaacttgtaactgtgctgtcatatggctgcaaacaatagaa

tgatggatagcactgcaaggttggaactccatgtctttgcgtatatcactaaatgtttgt

atacatattgtataccaaggaccactgcagttttagaccacatcattaccaatttacaga

gagaaggtagatagcaactatcactaacaaccttccgcaagtaaatggatcccttcagca

caatttcttttgctcatgctgcttattgcaaagttggaaaacatatccgttacttgagca

accgaacagtgcatagtattagttgggttaaagaaatattcagctaattacaaatcgtct

tatttggtattgaccacagttgtggcattgtttttctgttatatttcttttgtaaacaag

atcttcccccggtcctgattttagttggtatggccaattgttcgtggcactgaaatttac

catcttttgggttgcag

TATACACCAGCTATTGATATATGGAGTATAGGGTGCATTTTTGCCGAGGTGTTGATTGGA

AAGCCTCTATTTCCTGGTAAAAATGTTGTTCACCAGCTGGATTTGATAACTGATGTTCTG

GGGACACCTTCATTAGATGCTATTTCTCAG

gtatggatatttggaagtttgctgtcaaagttatagttctgtctgcttataaaattttct

gctgtcctacctctgctaag

GTGCGGAATGACAAGGCAAGAAAATATCTGACATGCATGCGGAAGAAACAGCCTGCTTCC

TTTTCGCAGAAATTTCCGAAGGCTGACCCATTAGCATTACGATTGCTTAGGAGGCTTCTA

GCTTTTGATCCAAAGGATCGTCCCTCTGCTGAAGAG

gtatgcgatatatatgaacttaactggtcccatgttctgcatctggcagtacaagatatt

gtgatcttgatatatctgctaattgtag

GCATTGGCAGATCCATACTTTAATGGACTAGCAAAGGTAGAGCGAGAACCATCTTGTCAA

CCGATACCAAAAATTGAATTCGAATTTGAGGGTCGTAGAGTAACAAAGGAGGACATCAAG

GAACTGATCTTTGAAGAAATTTTGGAGTATCATCCTCAATTACTGAAGGAGCATATCAGT

GGAACAGACAGACGAAACTTTGTTCATTTAAG

gttagatatgttattgcacacttgtttatcttctcttagtcatcatcatccatgtctgat

atgcttgcatcttctggtattgcag

TGCTGTTGACCAATTTAAGAAACGCTTCGCTGAACTCGAGGAAAATGGTGGCGAAAATGG

ATCAGCTGTTTCGTCACAGAGGAAACATTCTTCTTTGCCAAG

gtaaatgtcttatttccaactcaaatctcttttactgggaggggatctcgacagcgaatc

atttatacaagggatgatccattatgtattgatatcaccaatgatgcaacaggaagaata

aaacatccattttcaaactgcttgcaccctttcttttggcaaaagtctcatcccattcaa

gcatctagtcctaactaacagactttgttctttcttcattactttcttatattgctggtt

ctaatattgtaatttttgtaaaatgaaagctttattacccccggcctcagcatcgttgat

acatgcatccctgattattacattatttattacaagccatcaaaaagcaacataaccaca

ctaaacattataggcctaatgaaaaagacaggccacacatatcatcactagagagtagag

actgagaagcctcgcactacttcactagcacaccatccaaaccaggacataaactccctg

gtaagccactctaacttcttggtgccaagctctgttgagctacaccttctgtaacaacgc

ctatgattgcagccaactgatacacgatcgtagcacctgcctgcaagattggacattgtt

gtaaaaaaataatactccctccaatccaaattaattgaggccgcatttagattccttgta

gataaatatagaggtgtttattttgaacctgtatcttaccatctgctcagcaattctcag

ccggaaacataacgatgatctgtgacctatattctttacaggtgtgtttgaaaagaacac

gacaatccaaacagggcctgacgcagcctctatacaacattgtatagaggttgtatagag

accgtgtcaatttggatcggagggagtactgtttctacaaagccaaatagaccaaaaaac

tgctccagtcccaaccataataaagcgattatagtgatttacctacccccctgcagccac

gtcccaaatatgtgtgaaatactacaagatgcagtaatgttaaatgctaaataaacagcc

tgccatgcaatgcatgctaacacgcaataaaagaatgctgaatggtctcctgttcatagc

aaaagctgcattgcgtgtcctgctgtcatttacatttaagtatttaaccaaattatcttt

tgtgaggataaccccctggcaaagaaacggaaggaacacctttgattgctggttctaata

gtcagtagaaagtagacatttgcggcactgttttcctgaattataaaatgttaggttgat

cttgacgaagaatcgttgaaggtttcatccaaatcaaaccatcgtgcttgtgaacctcga

ttaatgaaaagtagttggaaaaggtcaaatctttctccaaattttctgttactagtagct

ataatgcacattttccctagcaatgattgacatgactgaggtatgtgcgtctttttctcc

tttgaagctattaaataaaattaacatgcagagcgactgttaagattaagatgatcctag

tgagaccctggaagatttagtttcttgtgatgcctttctgtcaatgtgtcttgcttgaaa

ttctgacaattgatgacctatggtccccccgccccggcgcccctgacacaagtatagccc

aacgggatgcagcttcagtgacgtaaaggcacctgcctttgggtccctgacatgtgggcc

agccacctgtcaggcccacattcataggcacaaaggcaggtgccttaaggcaccgaagcg

tagtccagcccaacggtagtgcaccctgctaaaataccttcaaaggatcatatgcagacg

gcatcatctagtacaaagcatgcagttgatgcatcttggaaagtacaaattcagagagta

tacattgctcaaagagagttaaacatcacgttgtctacatttgttaagttaacatttttc

gtttcactaccatcaaagagagttaaaaatcttctggatgatgttgtggggttactaatg

ccttccttcttttgcag

GCAATCCTTCAACCCTCGTTAGACGTGTAATGTCCTGTACAGTCAAAACTCACAGGAAAG

ATCCAAGAAGCTTACCATCTGTACTCCCTTGGCAACCAAATATCAAGCACCGCACTCTCA

GAACCCAGTATCTAGTGGGCCAGTGCTTGATGCAACAGGTCCAACTCCCAACACAATATG

TGCCTGGAGGAATGTGTGTAGACTCCAGATCAGGGAACTTGTACTTCTATCTGCATCATG

GTATAAGGGTTGATATACCAGGTCACAGTATCGCACAGGCAGTCTGTTCCTAATGGCTCA

GTTCCGGCTGTACGTTGCAGCATGGCGTTGCCAGGGATGTTTCGAGCTGGGTTATCGGCA

TTGAATCATGGAGGACTGACTTTCTTATTTGTCAGTCCCCCAAATCATGGAGGTCGAACC

TAGGATTCCAGCTTCATCTTGTACATGGGATGTTTCACAGCTTCTTTGCAACCGAGCCCC

CCAAATGTACCAGACAGTTGGATCATAATGTGCCTCCTAGGGATTGCGTTGCAAAGCTCG

TTTGTTCTGAATGATGAAACTTTTTCCATGGGAAGCTGTGAGGTGGCTAGGTCAAAGAAC

ATGATCGTTGCGATTTACATCAACCAGTTGTTTAAGAAATACTCCTACATTTTAACGCGA

GGGACCTTGCTGTGTTTAGCAGTGTGTTAACCAAAGAAACTTGAGCATATAGTCTGAATC

ATTTCCAATAGTTGCTGTAAAGAAATGTGCGAAAGGTCGAATGCCAGAGACCAAATTAAC

TGTTCTGATGAGCTGGGTAAATAAATTGTTTGTCTCCGTCGATATTGATGTTGTTGAGAT

TCTGTTCATACATCTTTTCTCAGATGTATGCCAATTGCTTTTAT

**>TaMPK20-3(7DL): TRIAE_CS42_7DL_TGACv1_604962_AA2003380.1**

ATTTTATAACAAATTTTCTACAAAAATTATAAGTGACATTACACTTTCTCTGACATGAAC

AAATTACATTTTTGGTCTAAAGAAAGCCCGAGTCTTGTGATCTACCTTTGGAAACGGCGA

AACACCACCGAGAATCCACGAGGTATAGCTGCCACGCACGCGGAGCTCACCTCACCACGC

GGAGAAAACAGAGGAGAAAGAGAAGAGAAAATATCCCTCCCACATCGTCACCCCAGCTTC

CTCCGCTGCCTCACCACAGCACGCCGACGAGCTCTCTCCACGCACGACGCCCGCTCCCCC

TCCCCCCAGATCCGAAGCGACTGCCGCCGGCTCGCGATCTTCCCGAACCGTACAAAAACC

CGGCCGTGGCGGCGGCCGCGGCAGCCACGTACGGTGGCGGCGAGGGGTGTATGAGGAGCA

GGAACGACTGCGGGACCGCGATTCGGAGCAGGAGCCGGTGGGGCAAGTGCGGCTGACCCA

CGCCTGAGCGAGAGGCCGGTCTGTGTGTCTGGTGGGATCGTGGGGGGGAAGGAGAAGAAA

GAAAGAAAGAAAGGTCGATCTTGGGAAGCTTCCACTACGGGCTCCGAGATGCAGAACAAC

GATTTACGCAAGAAG

gttcgtgctcttggattctttggctttgcttgtttagatgggagcagtgaaaaatgttgg

gaatgatggcaattcgagtaagaggtccaaaagtgggttcaatattagtttaagcgaata

aaactaggtgacatgctgtgactttgggacatttttgtttgctaatgttgttattctaca

aagtagctgctggttctagggacacctccttcactctttggtagcaatagtttatttttc

cacatgctataagtgaattgtaaatttgcaatgtcagaggtgtgcgtagttcttggctct

ggagtatgtgattacaccctaaaggaatatctttatatgtacctggtagtaacagcggaa

aacctttgctccagtccactgtttgcttgcaagctatgccgtactgacactagattatta

atttgtaggcggagagggacttgctctggttttgacattaatactccctccgtcccaaaa

ttcttgtcttagatttgtctagatacggatgtatctaatactaaaacgtgacttgataca

tccgtatttagacaaatctaagacaagaatttcagtacggagggagtaccactgaacatc

tgtgattaatttgagcagtgttttgattcggaaatgtatctttgcccccttaaactattg

gctgggtgtagatctggtcccacggccagatatttttgccctggaactgtcatttcgtgt

actttttgtcactcaaggcgggttgactgattcaaagaggtttttactgatgcagcgcca

gcaccatgtgtctgccgcatcactaatcttgtttcagaaatccacaaacctatttaaaaa

gcagagaaagaaaacaaaaaagaaaaatataattaaaatgatattccatattctgtattt

acaaggaatttgagagaaaaaaggagcaaatccgacatttttttggtttattagaagaaa

atcagtttaataaaagaatcataatctccttttatttgcaatttcttgattttccttttg

actatttttttgttttcttgatttttgaaaatcttagatttttgtaaataaaatgatttt

ctcaagtttttttaccccattatgctgtgtatgcttttactcctttttcccctttctgat

agttttctccatttcttcaaaactgatgtctttttactagattcattgatatcttcgttt

gaagtacatatatattttttgcctttttctgcttcttaattaaattttaggaatttctgg

gaggaaattggtaagattgcagatatgtgatttggcctacatgtagatgccacatcgatc

aaaatcatttttgaacagtcaaaaccacctcgatggagcaaaagtatacagtaccgagag

ttgaagggtgaaagtgtccgttttaggagttgacgaccagatctacacccaaaacaatag

tttaggggtcaaaagtacacattttccttttgatcatagagtaaccaagtggtagtacaa

tcatggatggtcattgtcttctgtgtttcgtaggtttaatctgtaacagtattagttatc

tgattattttctctaattcag

AGTGCAGCGGAAATTGATTTCTTTACTGAATATGGCGATACTAATCGATACAAAGTTCTG

GAGGTCATAGGCAAAGGTAGTTATGGACTTGTATGTTCTGCAAATGATACACAAACAGGA

GAGAAGGTTGCAATAAAGAAGATACACAACATATTTGAGCATATATCGGATGCTGCACGC

ATACTCCGTGAAATCAAGCTTCTCAGGCTTCTTAGGCACCCTGATGTAGTGGAAATAAAG

CATATCTTGCTCCCCCCATCCAAAAAGGATTTCAAAGATATATATGTTGTCTTCGAACTT

ATGGAGTCAGATCTTCATCAAGTAATAAAGGCTAATGATGATTTGACGAGGGAGCATTAT

CAGTTTTTCTTGTATCAGATGCTTCGAGCTTTGAAATATATGCACACAG

gtatgcacttcctcacagtctttagtttatgaagatcaatgttacgtgcgcttctattct

tatggaacgaccatgtagctgtttgactattgtttctttcttgcacttgcattgttgtat

gctaatttgcatgcaaatttcacttgtaaccatttgatgatttgattcatcccttttccc

ccttcatgttctttcttttattgagatggcactggaacaattacatgagttctaattaac

aatctattgaatgggtcgactgatttaaagtttggtacaatttggtcgagatgatagtcg

ttctgaatgcgcgccatggcctgcatctaaggcctggtttgaaatgcagcaatgctgttg

tagcatgtagacttgttattgcaatatgctcctgtgtctgtatacgcgatggaccccgac

cctgatgtttatggccccaacctctttgtatcattgtgtatggcaatcaccttttttttt

ggaaaggggaatatattaatatcgcgaagataccaattacacccagcctctgcacctaca

agatgtcacaagacatccaggatgcacacagccaaaggagaaaaacaaaaaagcaaggaa

aaacaaaggtcccgccacagcgatcaactcctctcagcaatcaactcctctcagcagtag

cacaccaaccaccaccaaatacaacacccagaaaacgtaagaggcctccaaaagcaacac

cttcaagaagggaccagtgcacaagcggcgtcgttgcccgatccaagatcttaggttttc

accctgaaggaagtccgagctctcgaaacaattccttcagcaaggcaattgccaggcaca

accaatgaaggtcagaccttaggctttcaccctgaaagtcacgactcggcacccaaagag

caccataaaaaaatgaaatcctgcaatgctgctgcccccacttatcagtgctgcttctaa

aagttatcgaacaccaggctgactccatcaccaccaaggccccgtccgactaactgatcc

tcctatctcagccaccatgaccttctccatcgccgtctattccatggaaatcgagaggcc

aacatgtcccatggtatgaacaaactccagagctttgtgctgcgccctcagaacctccta

ggctgatcttgacgcgcatggtccgaaccagatctcccgagcaagatcaccatcagcagt

tccggaacccgtcgatgaccagatcaaggagggccgatcatgccgaacgttgtcgtgatt

cgagcagagcacgaggaccgccacctttattcacggcaacaggccctcacgccaccccga

actagcctgccgccgtctggccggccaccatcggcaccaccacggtgcctacccggcgct

gtcaggcccccaaccgcgctaccgtagccgatattagatctggacgataactgagatcca

cggccaccacctccgcccgcatctggcagagagcagcgggcacgccggatgcccgcgcga

tacgccggagccaagcccggccaccgcatcatcgcaacgcggtgcctgcgcacggatcaa

agcacgccagccagatccgccgtgctggagagccgcccgtgcccatcgcgtccaggagag

cacccgcgcgagactggccgccgggccgaagcgctcgaggagccgccagcgacgtcgctc

gggacgaaggtatggcggtgtgaggggcaaaaaacgccccgccgccatcatcccggagcg

cggcgcggcttcgccggccggctctctggcggcggcgtggcgggaggcagaggaggcggg

gacgggcggaggcggctggtgttttcccccgagtcaccaaatgcgggcgacatgggggcg

gcggctgggtcttccgcctcgtacatagagttaggcttatggcgttctgcatttgcccac

taatgcatgctgatctgagtatctgggtattgattactgatgcagcattgcacatgacaa

ctcagatatttagttgcacaaataaaagtaaaaggttgctcctttattttttgaatccgt

gtcaactttggctagtctggtatttaccgaaaccatagcatatttggttccatactctat

ttgtatttgcatgtttgttttttaacgttggaaattatataatatcatttcagttgctac

atatgatcagaattggctaagtattgtttgtgctttgatgcag

CAAATGTCTATCACCGAGATTTGAAGCCCAAGAATGTGCTTGCCAATGCAAATTGCAAAC

TCAAAATTTGTGACTTTGGCTTGGCGAGAGTTGCATTCAATGATGCACCTACGACAGTCT

TCTGGACA

gtaagcaatatcttttgttattgtatctcaatagcatatgttattggttgatcttgcagt

actcgaagtttctatcttgattgttttaagttccaactggcccttcgatcagctatttac

cacagttcgtaattgattgtattgtcatgtgacaacacattacatgatgcagctaacaaa

tagccggtggtgtatttactcccaacattattcttgtgacacccactgttagtcatatta

ttaactaacaattggagatttactaccaccgaatcatgctttgttccctgttgtcatgtt

aaacttcaggttgcctcattcgagacacaatggttctaacaaagggaaagtgaatatcta

atgatcagtgcactgtcacatacaagttgaaataagatgtcatctagttttacttttaac

tgggtcttcatgaagccatttccccattctttggttttagggtgggattggggtcgacgg

ttgtgaagctaaccattaatttagggcgttggggctgctctggagtctttcattgctcat

ccaatgttttctattcctgtcaaatgaacttctaaattaacataaacttcgcttttcag

GATTATGTGGCAACGAGATGGTATAGAGCACCTGAACTTTGTGGGTCCTTCTATTCTAAG

gtaagcaatattacgaacttatgattaatcagaagttagagaggttgacatgtatacata

tcgatgatgccaacctaataggcattatttttgtgtgatggtactggtgctggtaaatga

cattgttgggatgcgggtctggaacttgatgtcccgtgcctgttgagtcacctcaccttg

tagtgagtatgagggaattggccatgaggtgagtatgagggaaattacagcagtgacggc

agcagcaagataaacagtacactgtaattgagtttaaatggtaaacagtaaagatactct

tttgcccgcgcccatctggaccgtccattgcttctcgtcttgatcaatctggatcattgg

ttaactgacgaagtgccaataggaatataacactagtgggttcagtcagggtcctgacag

tcgagtcacttcacattgtagtgagtattgagggaattgccatgaggcgcttttgacttt

ttttttaatatttggatttgtctgtcttatatgcctataacaccaaaaaaatgtgcaatg

cattgtttacacctgtatgcacaaatttgagcagaaaagttgatgccatcaactcatcat

atacttgtaaatcagcaaatgtcccatggcaaattggcaataaagaagtaacactcatga

ttcataagcccatattgtatgctgttcaaacttgtaactgtgctgtcatatggctgcaaa

caatagaatgatggatagcactgcaaggttggaaccccatgtctttgcgtgtatcactaa

atgtttgtatacatattgtataccaaggaccactgcagttttagaccacatcattaccaa

tactctaatagtcatagttgtacacttgtactagtagctcctcatggttcctccactagc

acctctggaccgtcacaagttgcgattgtccttgcctgacagcctcgcaacaacacctct

caaggtatccacacgtataaaagcgggaacgctgtgtgctagcctgctagggtgctagca

ctgacaagcacggccaggctttcatgggtgggagtggtggctaggctttctgaagtccca

cgaatttgtgcccccaatgccaacatgcctccctttgcatagattcccgtggtaggtctc

cacattggaggctcactgagcccagtaggtaccagaccactttccgaaacctgttccagc

ctgttagtgtttgtcgcacataaataacttgaatgggaactaaaccatgtggcaactatc

ttgccataccatgttcatattccatattggggaaacatgaatagagttcgaattataatt

taataagaatgcggttttgcttactaactaccattcagatgtggctaccgtcgagtttga

attattttctgcacaatttatctgtttctacaccctcttaatttacaatagagaagatag

atagcaactatcactaacaaccttccacaagtaaatggatcccttccgcacaatttcttt

tgctcatgctgcttattgcaaagttggaaacatatccgttacttgagcaactgaacagtg

catagcattagttgggttaaagaaatattcaaatggtcccagttggaagttgactgtccc

aattacaaatcatcttatttggtattgaccacagttgtggcattgtttttctgttatatt

tcttttgtaaacaagatctccccccggtcctgatttgagttggtatggccaattgttcgt

gtcactgaaatttaccatcttttgggttgcag

TATACACCAGCTATTGATATATGGAGTATAGGGTGCATTTTTGCCGAGGTGTTGATTGGA

AAGCCTCTATTTCCTGGTAAAAATGTTGTTCACCAGCTGGATTTGATAACTGATGTTCTG

GGGACACCTTCGTTAGATGCTATTTCTCAG

gtatggatctttggaagtttgctgtcaaagttatagttctgtctgcttataaaattttct

actgtcctacctctgctaag

GTGCGGAATGACAAGGCAAGAAAATATCTGACATGCATGCGGAAGAAACAGCCTGCTTCG

TTTTCGCAGAAATTTCCAAAGGCTGACCCATTAGCATTACGATTGCTTAGGAGGCTTCTA

GCTTTTGATCCAAAGGATCGTCCCTCTGCTGAAGAG

gtatgccatatatatgaatcttaactggtcccatgttctgcatctggcagtacaagatat

tgtgatcttgatatatctgcaaattgtag

GCATTGGCAGATCCATACTTTAATGGACTAGCAAAGGTAGAGCGAGAACCATCTTGTCAA

CCGATACCAAAACTTGAATTCGAATTTGAGGGTCGTAGAGTAACAAAGGAGGACATCAAG

GAACTGATCTTTGAAGAAATTTTGGAGTATCATCCTCAATTACTGAAGGAGCATATCAGT

GGAACAGACAGACGAAACTTTGTTCATTTAAG

gttagatatgttattgcacacttgtttatcttctcttagtcatcatccatgtctgatacg

cttgcatcttctggtattgcag

TGCTGTTGACCAATTTAAGAAACGCTTCGCTGAACTCGAGGAAAATGGTGGCGAAAATGG

ATCAGCTGTTTCGACACAGAGGAAACATTCTTCTTTGCCAAG

gtaaatgtcttgtctccaactcaaatctcttttactgggaggggatctagacagctaatc

atttatacaaggtatgatccagtcataatatgtattgatatcaccaatgatgcaacatga

agaataaaacatccattttcaaactgcttgcaccctttcttttggcaagagtctcagtcc

cattcaatcatctagtcctaactaaaagactttgttctttcttcattactttcttatatt

gctggttctaattttgtaatttttgtaaaatgaaagctttattgccctggcctctgcatc

attgatacatgcagccctgattattacattatttattacaagccatcaaaaaggagaacc

acactaaacattataggcctaatgaaaaagacaggccacagatatcatcactagagagta

gagactgagaagcctatcactacatcactagcacgccatccaaaccaggacataaactcc

ctggtaagccactctagcttcttggtgccaagctctgttgtgctacaccttctgtaacaa

cgcccatgattgcagccaactgatacacgatcgtagcacctgcctgcaaggttagacctt

gttgtaaaaaataatactccctccaatccaaattaattgaggccacgcttggattgttgt

aaataaatacggaggtgtttattttacacctgtaccttaccgtccgctcagcaattctcg

gccagaaacaaaacgatgagccgtgacctgtattgtttataggtgtatttgaaaaagaac

atgacaatccaaacagggcctgacgcagcctctatacaacattgtatagaggttgtatag

agaccgggtcaattaatttggatcggagggagtactgtttctacaaacccaaatagacca

aaaaactgctccagccctgaccataataaagcgattgtagcgatttatctacccccctgc

agccacgtcccaaatatatgtgaaatactataggatgcagtaatgttaaatactaaataa

acagcctgccatgcaatgcatgctaatatgtaataaaagaatgctgaatggtctcctgtt

catagcaaaagctgcgttatgtgtccctctgtcatttacatttaaccaaattatcttttg

tgaggataaccccctggcaaagaaacagaaggaacaccttgattgctggttctaatggtc

agtagaaagtagaaatttgcggcactattttcctgaatcataaaatgtcccaaatgataa

gtgccacacgtgtggcatgaagtaacaccgccctgacttttttacaactaaaattgtcat

ccgaaaaaacccccctaaaaatactgaaacttgccatccaaacagaaagttgctatgcta

ttgccatcctcgggtaactaaacttgccgtcaaaaaagtcgggacggaattgcttcgtgc

cacacgtgtggcacttatcagggtcctaaaatgttaggttgatcttgacaaaaaatcgtt

gaaggtttcatccaaattaaaccatcgtgcttgtgaacctcgattaatgaaaagtagttg

gaaaaggtcaaatctttctccaaattttccgttactagtagatataatgcacattttccc

tagcaatgattgacagtatgtgcgactctttctcctttgaagctactccttccggtccta

aatgtaagtctttttagagatttcaatatggactacatacggagcaaaatgagtgaacct

acactctaaaatacgtctatatacatccgtatatagtccgcattgaaatctctaaaagga

ctcatatttaggaacggaggtagtattaaataaaattaacatgcagagcgactgttaata

ttaagatgatcctagtgtgtcgtgtggggccagacttataggcgcaagggccctgggcca

ccctagttctgtctcggtttaattatagagtccttctagatttaggttaaagagtctggg

ttttagttggaaaggtttagtcccgaagaaggttaccctgccaacacccaattagagttg

gagtctgtcaggtcattgtaataggctacttatagccaggagtctgtcacgtcattgtaa

taggctacttatagccacccctatgcgatcaaatgagtacaaccagaaaataatctatct

tctcaccttgtgcgcctgcgcctcggtgccccctcctcacctctttctccaaccataggc

cgatgagagtttcgccctcgtcctccgaccacccacggctgcaacctccggtccaatcca

accccagcccgtgacaacctggtatcagattcaccttcgatcccggtggctgccatggcc

cctccgccgccccttctgcaaaccctagaagaactcgccgccaaacagcaggcggaccat

gaagccgtggtcgcgactctcaccagcctctccgatgaaaatcacaccgcgagggctgac

cgcaccaccctcaagtccatcgccgagacgctccagaagctccagggccagctcgctgat

acgtcacagcaacaacgcgcgtagaatccggccctcctccgtcttgaaggcaagggcatg

cctcaacttggtggcctcgggctcatgcagccacttgcggctacgtccaacgccggggac

agggccgccctgtcggatccatccggtcgtgctccgcgactctacaattgacttccccct

ctttgatggggcgagtgacccacggccgtggttgacacgctgcaacctgttcttcctcgg

ccagcggacgcaggattccgataagacgtggctcgcctcctaccacctcaccgacgtcgc

cgccttatggtacggtcatctcgaggcaaagttgggccagcggccgtgctggggagagtt

tcaaacactcatctctaaccactttggaccgccgacacacgccaacccctttggcgagct

gatcttcactcgccgctccggcaccgtggcggagtactccaagcgtttccttgagaatct

ctctcgtgtgcacccaattgccgacgccgaggagcgtgatatcttcaccaacaattgggc

gagcccatgaaaactcaggtcgagatgcttaaacctgcgactctagacgtggctatggac

ttggcaattttgtttgagcacctcaacactgtcaccggagccacggccgccgctgctcgg

ccgagccgccaaattcgccccatggccaaccctggtgcggtattgtctgaatcctccagc

tccacgccgccgctcgtattcaaaaagctcaccctggctgagatggacgaccggcgcgcc

aaaggcctttgcttcaactgcgacgagaagttcattcggggccatcgctgcaaacgtctt

ttctacatccagtcagcagacgaagaagaatagccattagcggatttccaggaggccaca

atctcgcttctggctgtcacagggattcccactagcgacaccacgcaggtcgctcttcgc

gtcggggatcgtgacctcgtcgccttgcttgattccggtagcactcataacttcatccac

gaggagctggccaatgtcgtggggctgcccttctcctccggccgctgcctcggggtcacg

gtcgctaatggtgataaggttacctgccatggactcctcaagcatgctgctatcatgatt

ggcaagcaacgcttcattgttgacctgcacgccatacagttgggcggatttgacgtcatc

ctcggcacgcgcttcctcaaaaccttggggcccattctatgggatttcaatacccagtgg

atatcgttttggcacagggatcaccgcgtggagtggtcaagactaggctcaacaggacgg

cctgcccacttacatgtgtgcaacagcaaggacctcctcgacagcctcctggtcgcattc

acagacgtcttcgccgagccccagggactgccgccgccgcgcgcccatgaccatcatatt

caccttattccagggacgcagcctgttgccgtacggcctttttaccgctaccgggctatt

caaaaggatgaactggagagacaatgtgctgagatgctggcttgtggccttatccgccgt

agttcatcggccttttcatcacccgtcctacacgtcagaaagcatgatggtacttggcgt

ttctgtatcgactaccgtggactcaaccttgtcaccatcaaggacaagtttcttattact

ggtgcttgatgatgctcatgtagccttcggcctgctggacaataggagaagaagacgacg

cctgcagtaatagatcaatttagttcaactaaagaattgggatgagaggtgtgactccaa

ttcaaattgtggtaatgtatcaagccacatgaacacatctaattagcaattagaccttga

atagacaaaatagttgaaccagtgggatgtggctttaaaatcgaagactaatgtgtgccg

agatcagtacaagagggagcgagggccgagggatggatgccatgccgttggaatctggaa

tctgggatctggatggatgcgttggcgcgccgtccaccagtgcctgccgttgtacgcgca

tgtgatcaggcggtcatggccgatggcaattagccctggctgcctggttcgtctgctact

ggatggattgatagatttttctttcgatggaggtaactgtatcagcgatggatgcctggc

tgatgcgtgcgcacagtgcagcctgaagccaatttggcctatgggtcatgtcgtgggtgg

tgagggagagtaggaggagctggtctagcttctgtttttgggctgaaatacttggttaga

acattttattacatcagttttgttttatcttgtatatgcatatggcatacactatataca

tctagtttttttgacaaaaataatgggaatacccttgaattgaggtgggcccgccactgg

gtagtctagtttggccatccaactgttggagtattcggtgnnnnnnnnnnnnnnnnnnnn

nnnnnnnnnnnnnnnnnnnnnnnnnnnnnnnnnnnnnnnnnnnnnnnnnnnnnnnnnnnn

nnnnnnnnnnnnnnnnnnnnnnnnnnnnnnnnnnnnnnnnnnnnnnnnnnnnnnnnnnnn

nnnnnnnnnnnnnnnnnnnnnnnnnnnnnnnnnnnnnnnnnnnnnnnnnnnnnnnnnnnn

nnnnnnnnnnnnnnnnnnnnnnttaacatttttcgtttcactaccatcaaagagagttaa

aaatcttctggatgatgttgtggggttactgatgccttccttcttttgcag

GCAATCCTTCAACCCTCGTTAGACGTGTAATGTCCTGTGACAGTCAAAACTCACAGGATA

TGAAGATCCAAAAAGCTTACCATCTGTACTCCCACTCCCTTGCAACCAAATATCAAGCAC

CGCACTCTCAGAACCCAGTATCTAAGTGGGCCAGTGCTTGATGCAACAGGTCCGACTCCC

AACACAATATGTGCCTGGAGGAGTATGTGTAGACTCCAGATCAGGGAACTTGTACTTCTA

TCTGCATCATGGTATAAGGGTTGATATACCAGGTGACAGTATCGCACAGGCAGTCTGTTC

CTAATGGCTCAGTTCCGGCTGTACGTTGCAGCATGGCGTTGCAGGGTTGTTTTGAGCCTA

GGGTTATCGGCATTGAATCATGGAGGTCTGGCTTTCTTATTTGTCAGTCCCCCAAATCAT

GGAGGTCGAACCTAGGATTCCAGCTTCATCTTGTACATGGGATGTTTCACAGCTTCTTTG

CAACCGAGCCCCCCAAATGTGCCAGACAGTTGGATCATAATGTGCCTCCTAGGGATTGAG

TTGCAAAGCTCGTTTATTCTGAATGATGAAACTTCTTCCATGGGAAGCTGTGAGGTGGCT

AGGTCAAAGAACATGATCGCCGCGATTTACTTCAACCAGTTGTTTAAGAAATACTCCTAC

ATTTTAACGCGAGGGACCTTGCTGTGTTTAGCAGTGTGTTAACCAAAGAAACTTGCATAT

AATCTGAATCATTTCCAATAGTTGCTGTAAAGAAATGCGCGAAAGGTGAATGCCAGAGAC

CAAATTAACTGTTCTGATGAGCTGGGTAAATAAATTGTTTGTCTCCGTCGA

**>TaMPK20-4(3AL): TRIAE_CS42_3AL_TGACv1_195023_AA0643580.5**

GTCGCCCATCACATGGTGACCCACCGCCGCGCGGCTGTCAAAAGAAAGAAAGAAAGCAAG

CACTTCTTTTTAACGTCAGTGGTTCCCCGAACGAACCCGATCGCTCGCACCAGACGAAGG

AGGCGAGGAGCTCCTTGTGTTTGGGTGGATTTGCCCTTTGCGTTGCCCCCACCCTCACCG

GGCCACAGGACGAAACCAACGCCACAACAACTCACACCCCCTTCGCCCGCGACTTGCTCA

GTCGCAGCGAGCCGGGCCACAAGACGCGAGCGTTCCGGTTCAGGTTGGGGTTGGGGTTGC

GCTCCCTCGTTTCATCGTCGATCCGCCCGCCCCGCCACACCGGCCCGGGGGCAGACAGAG

ATCTCTCCCTCGCGCGCGCGTGCCGGCGGTGACAAATGCGGCTTGATTCGCCGGCGGACA

AGCGGCGGTGAGCGATGCAGCCGGACCAGAACCAGCAGCGGAGGAAG

gtgagaccgtgcgccaggggttgctgtttgttgcggggtggttggctgcctttcggtgcc

cgtgtgggttgttggcgcttcgagatcggtcggttcgtccggcgggttggatgaacctac

ctcgcctcgcgggaggattctttttctttttgatgggatccggggggttccggcgcgcac

gctcgtcagaattatctcttgccattggtatcctgcaacttctcttccccttccgtcgac

aattccttccttccgtatagttgaggttgttgattccagctacggagtccaactgaattc

ttctcttccatttcttattgcgaaaaagggatccttttcactcctgtttctggatcacaa

ttcatctcaattattaacacaaggttctccgttggatttccctagtgggtgctttcctga

aatcttcttctgcggttgggcttgggcaccgttttttcttgctttgtccctctataaagg

aaatgatattcttttctaacttttccgttccatgctagtagttgaatactcattgcttcg

taaaggattgctgaggccttttcaactcctgtaatcaacaccaggggaaagaaaaaaaag

gtgattcagatgcaacctaagaggtgccacaaacgcaaatgcggtattagattcgctgag

tagatatgtttatctcaactcaacccaattctgtggtcttcttaccgtcctttttcttgc

aaaaagttatagccaatcatcttaattgtctcagtggattaggttaggttgtcaatatat

tctatgcccacttgggaggcatattgatattgatatagatggttttatgcccaagaaaac

atccttaaatagtcattcttgggccctttgtgctccataagcgctgttttcctcatagct

ggacgccaaggagttttgcatcggacagggagttgtagctaccttctttgtgcgcgagcg

atccagaagctcacggaatccaactttttgtgctgttcacaactctgtgagtcagttgga

tgcagattacaagtctgctgtttcagtggtctgttcggtatgaagtgacctttacacaaa

atttggctgttgtattgaggaattttttttgggggcggaaaacctgggtttaacttcttt

ttgtcatctaagttgtttcattgaaagcctatgtggctagtatgtgttatcaacctgggg

tttatccaaaggatgagctgaaccttttctggtcctttactaagtttatttgctggcagg

ctgggccatctgacattgagctcaatgcttgaggcagatattactcagtaaattgggcct

tcttttctgtatgtgtgtcttttgtttcagttgcggaaaggttctccattgcgcagtatt

aactctgtttttttccacttctctcag

GGTTCATCGGAGATGGACTTCTTCAGTGAATATGGCGATGCTAATAGATACAAAATTCAG

GAAGTCATCGGTAAAGGGAGTTACGGTGTCGTTTGTTCAGCTATTGACCAACATACTGGC

GACAAGGTGGCAATCAAGAAAATACACAATATCTTTGAGCATTTATCTGATGCTGCTCGG

ATCCTCCGTGAGATCAAATTACTCTGCCTATTGAGACATCCTGATATAGTTGAGATCAGG

CATATAATGTTGCCTCCGTCAAGGAGGGATTTCAAGGATATTTATGTCGTCTTTGAGCTG

ATGGATACAGACCTCCACCAAGTCATCAAGGCCAACGATGACTTAACCAAAGAGCACCAC

CAGTTCTTTCTCTATCAGATGCTTCGTGCACTGAAATATATTCATACCG

gtatgctgcttttttcaaattgagatctgaattagcatacagttctctattgtcgtgtta

taatctttcttgaatatctaggagtaggtattttatccataccctatctgtagatcgctg

ctgatgctttgctccgctccatgtggagcatacacaatttggagaaatgcactcacataa

aattcactctagtgccaaagttaaaaaggcactatacgcacattaaccaaaatttagcgc

taggcattttgctatcgcctagagcctaggccctaggcgtgcttaagcacccgccttagg

cgccccttttttaaccaagtgctaacccaataaccatggccgccatattttagagaaact

tgtcctttgtcccagattagttatgttcttgttgatcatttgtcttctggtgattttgat

atttggcctttcattgacctgaaaaatatctcccactttgtttcatgctttttgttgtga

aatgtacacttctttgtatttacctttaatgtcatcatgattattttccactatccattg

ttgagctactaacattatcagcttcaaaaatcatccatttccaatttaaaatgttcattt

accattgtctgtgatctgaccaatatttatttgattgcag

CTAATGTTTATCATCGTGATTTGAAGCCAAAGAATATATTGGCAAATGCTAACTGTAAAC

TCAAAATATGTGATTTTGGGCTAGCACGAGTTGCATTCAATGACACTCCCACGACTGTAT

TTTGGACG

gtatgtaaattttggtagactagaatcacatcctgtgttatttgccaaattagatttgta

ttcaaaggtagtcttttggcacttaatttttatcttctattcatcattgcttgcag

GATTATGTTGCTACTAGATGGTATAGGGCTCCTGAGCTTTGTGGATCTTTCTTTACTAAG

gtaactagtctgcacatctctgttgtgtttgctaggatatcttgtccgctactttccaca

taaacatgcctcatgcaattgctgggcaggctatgctgtttaataccacattattcttgc

gcacatagtaataaaagaagataaatcagtgtcacaactcacaacctatacaaggtttca

ctatggtgttccatgtagtttgcagctgtgctgtacagtggatatatatatatatatata

catgacatgagaccgtattcaaggttcatacgactactagatcctacgtagtcagctggt

cttgctaataaaatgacaatgcaaacccttctagagcctactgtttgagtgttttcacaa

ctacagagtatatgctactagttatcattaatacctgatctttcactccttaaaaggctc

cgatgttgaattcaggatgctaaattggttgttctgtgcag

TATTCACCAGCTATTGACATATGGAGTATTGGTTGCATTTTTGCGGAGATTTTAACTGGG

AAACCTTTGTTTCCTGGTAAAAATGTAGTTCACCAGTTGGATTTAATGACTGATCTCTTG

GGTACGCCATCACTGGATACTGTTTCCAGG

gtacgcttttccattcatgatccatgatccaaagtaaggtatccataaatgtcaagcatc

ctaatccctttgcaactgcag

ATCCGGAATGAGAAGGCAAGGAGGTACTTGAGTAGTATGAGGAAAAAACAACCGGTATGT

TTTTCTGAGAGGTTCCCCAAAGCAGATCCTGCTGCACTCAAACTTATGCAGCGGCTTTTA

GCATTTGACCCCAAGGATAGACCAACGGCAGAAGAG

gcaagtgtttctagataaaacttcagattcaaaaacaggtgttaaaaactttgtcattct

aacaatatttatgtgcacatcataatcag

GCGTTAGCTGATCCATATTTTAAAGGCCTTGGGAAGGTAGAGAGAGAACCATCCTGCCAG

CCAATATCGAAATTTGAGTTTGAGTTTGAACGGAAAAAGGTGACAAAAGAGGACGTAAAG

GAACTTATATTCCGCGAGATATTGGAGTATCATCCTCAACTTCTCAAGGATTACATGAAT

GGAACTGAAAAAACGAACTTCCTATATCCTAG

gtttttaccataagatctcctcgactttttttccattaaatgccaattttcataactgat

ccgttcttggttcctatgttattatgaag

TGCTGTAGACAATTTCCGAAGGCAATTTGCTAATTTGGAGGAAAATGGAGGGAAGGGAGG

GGCAATCGTTCCATCGGACAGGAAGCATGTTTCGCTCCCCAG

gtacccactgcaagattttaccaatagtatatgctgttatgttctgctttttgttaaaaa

aagatgccattaaattaaataaatagctcatgtgtgcatggttaaaatatagttctttgg

tgatctcatggtactatggaatcgctttacatgacaaaagatggtcaggtagtcaggaat

ttcagttcaagtttgatgatttggcatgataatttcagagactgccactcaactttgctt

gctgacatttagtttgcatttgacag

GACTACTACAGTTCATTCTACACCAATTCCTCCAAAAGATCAGAAGTCTTCCCAAGTTCC

CCAAAGGATTCCAACAG

gtagtagtttctcctaagtagaagtctagtgttctattttcttcagatagcaccaatatg

acatgtatatatcctttgctgcaaaatttgcag

GTAGACCAGGAAGAGTGGTTGGCCCGGTAATACCATTTGAGAATTCATGTGCTATGGATC

CTTACAGTCAACGAAGGGTGGCGAGGAATCCAGTACTTCCTGCAGCTGCTACCAATGTAT

CAGCATACGCATACCACCGAAAGTCAGACAGTTCAGAGAGAGAGTTACAGCAGGAGCTTG

AAAAAGACCGCATGCAGTACCAGCCGATGCAGCGTTTCATGGATGCCAAGATGGTCTCCC

CTGACTTGAGGTCTACCTCCTATTACATGCCAAAGGGTGCCCCAAAAGCCGATGTAGCAG

AAAGGACTGGTTTGCAGCCAAACATGATGCAGGGAATTGCCCCGTTTAATGGCATTGCTG

CAGTTGGAGGTAGCTACAATAAGGCCAGTGCTGTTCAGTATGGAGTTTCAAGGATGTACT

AAGTGATCTAGTGCAATGACCACTTCCAAAATGCTTGGCTTAGCTGAAGTAATAACATGG

AAATCGAAGTACTTCCTGAGTTGCCTTATGGGCTGAAGGTTCAGACAGTGGATCGATGAT

CTTCGATCGAGGAGGAAGTTGATGTCTCATGAAGAACAAAAAAAATAAGATTATGCAAAG

ACAACATCCTGCCTCTTGATCTAGCTGCCAAGGAAAGAATGACATCTGGTAATTTGCTGC

TAAGGGGAAAAGCCGAAGTTGGGGGTACAAGATGATTGAGGTCTATGTACTTTGAAAGAA

CGGAAACATATCGGAATAATGGAAATGGTGAACTTCTGTACATAAATAACTTGATTTTTC

TTTGTATTACTGTCGGCGAGAGCTGTCAGTTTTGCTCCATATTCATGTGGCAATGTGATT

TATTTTTCTGGGTTTGAAGCACTTATGCAGTTATGCTTCTGTAGGAGTTCAGCGTTGTAA

TACTTTGGAGTATGTTTAGATCTGTAATAGAATAACAAGACCAAACTTCAGTAAGATAGA

ATGGTAATGAACTGCTAGTTTTGGGGGTTGTTGGCTCAACCGTGGTACATATTAAACCAG

AGTTGATATAGAGGAACAGTAGAAACATAAAATAGCAAGGTACTGATCATTT

**>TaMPK20-4(3B): TRIAE_CS42_3B_TGACv1_225288_AA0806810.1**

AGGACCAGTCAGTCGCCGAACGAACCCGATCGCTCGCACCAGACGAACGAGACGAGACGG

AGCTCCTTGTGTTTGCCCTTCACGTTGCCCCACCCCCACCGGGCCACAGGACGAAACCAA

CGCCACAACAACTCACACCCCCTTCGCCCGCGAGTTGCTCAGTCGCAGCGAGCCGGGCCA

CAAGACACGAGCGTTCCGGTTCAGGCTGGGGTTGGGGTTGCGCTCGTTTCATCGTCGATC

CGCCCACCCCGCCATACCGGCCCGGGGACAGAGATCTCTCCCTCGCGCGCGCGTGCCGGC

GGTGACAAATGCGGATTGATTCGCCGGTGGACAAGCGGCGGTGAGCGATGCAGCCGGACC

AGCAACAACACCAGCACCAGCAGCGGAGGAAG

gtgagaccgtgcgccaggggttgctgtttgttgcggggtggttggctgcctttcggtgcc

cgtgtgggttgttggcgcttcgagatcggttcgttcgtccggcgggttggatgaacctac

ctcgccccgggggaggattcttttttctttttgattgatgacgagggggcactggactcg

tgggatccggggggttccgccgcgcacgcccgtcagaattatctcttgccattgctatcc

tgcaacttctcttctccttccgtcgacaattccttccttccttccgtttagttgaggttg

ttgatttcagcttcggagtccaactgaattcttttcttccatttcttattgcgaaaaggg

gatccttttcactcctgtttctggatcacaattcatctcaattatcaacacaaggttctc

tgttggatttccctagtgggtgctttcctgaaatcttcttctgcggtcgggcttgggcac

cgtcttttcttgccttgtccctctataaaggaaatgatattcttttctaacttttccatt

ccatgctagtagttgaatactcattgcttcgtaaaggattgttgaggcctttccaactcc

tgtaatcaacaccaggggaaagaaaaaaaaagagggtgattcagatgcaacctaagaggt

gccacaaacgcaaatgcggtattagattcgctgagtagatatgtttatctcaactcaacc

caattctgtggtcatcttaccgtcctttttcttgcaaaaagttatagccaatcatcttaa

ttgtctcagtggattaggttaggttgtcaatatattctatgcccacttgggaggcatatt

gatattgatatagatggttatatatgcccaagaacacatccttaaatagtcattcttggg

ccctttgtgctcgataagcgctgttttcctcatagctggaggccaaggaataggaatttt

gcatcggaaagggagttgnnnnnnnnnnnnnnnnnnnnnnnnnnnnnnnnnnnnnnnnnn

nnnnnnnnnnnnnnnnnnnnnnnnnnnnnnnnnnnnnnnnnnnnnnnnnnnnnnnnnncc

tgggttcaacttctttttgtcatctaagttgtttcactgaaagcctatgtggctagtatg

tattatcacctggggtttatccaaaggatgagctgaaccttttctggtcctttgctaagt

ttatttgctggcaggctgggccatctgacattgagctcaaagcttgaggcagatattact

cagtaaattcggccttcttttctgtatgtgtgtcttttgtttcagttgtggaaaggttct

ccactgcacagtattaactctgattttttccacttctctcag

GGTTCATCGGAGATGGACTTCTTCAGTGAATATGGCGATGCTAATAGATACAAAATTCAG

GAAGTCATCGGTAAAGGGAGTTACGGTGTCGTCTGTTCAGCTATTGACCAACATACTGGC

GACAAGGTGGCAATCAAGAAAATACACAATATCTTTGAGCATTTATCTGATGCTGCTAGG

ATCCTCCGTGAGATCAAATTACTCCGCCTATTGAGACATCCTGATATAGTTGAGATCAGG

CATATAATGTTGCCTCCGTCAAGGAGGGATTTCAAGGATATTTATGTCGTCTTTGAGCTG

ATGGATACAGACCTCCACCAAGTCATCAAGGCCAACGATGACTTGACCAAAGAGCACCAC

CAGTTCTTTCTCTATCAGATGCTTCGTGCACTGAAATATATTCATACCG

gtatgctgcttttttcaaattgagatcggagttagcatacagttctttgttgtcgtgcta

taatctttcttgaatatccagtaggtatttatatccataccctatctgtagttcgctgct

gatgctttgctccgctccatgtggagcatacacaatttggagaaatgcactcacacataa

aattcagtctagtgccaaagttaaaaaggcactaggtgttaattgtgcgttttgccaccg

ccttgtgcttttctgagcaaagcctacacttaagcgcaactatacgcacattaaccacaa

ttaagcgctaggcattttgctatcgcctagagcctaggcgtgcttaagcacccgccttag

gcgcgccttttttaaccaagtgctaacccaataaccatgtccgccatattttagagaaaa

ttgtcctttgtcccacattagttatgttcttgttgatcatttgtcttctgatgattttga

tatttggcctttcattgacctgaaaaatatctcccactttgtttcatgctttttgttgtg

aaatgtacacttctttgtatttacctttaatgtcatcatgattattttccactatccatt

gttgagctactaacattatcagcttcaaaaatcatccatttccaatttaaaatgcatgtt

catttaccattgtctgtgatctgaccaatatttatatgattgcag

CTAATGTTTATCATCGTGATTTGAAGCCAAAGAATATATTGGCAAATGCAAACTGTAAAC

TCAAAATATGTGATTTTGGGCTAGCACGAGTGGCATTCAATGACACTCCCACGACTGTAT

TTTGGACG

gtatgtaaattttggtacactagaatcacatcctgtgttatttgccaaattagatttgta

ttcaaaggtagttttttggcacttaatttttatcttctattcatcattgtttgcag

GATTATGTTGCTACTAGATGGTATAGGGCTCCTGAGCTTTGTGGATCTTTCTTTACTAAG

gtaactagtctgcacatctctgttgtgtttgctaggatatcttgtgcgctactttccata

taaacatgcctcatgcaattgctgggcaggctatgctgtttaatatcacattattcttgc

acacatagtaataaaagaagataaatcagtgtcacaactcacaacctatacaaggtttca

ctatggtgttccacgtagtctacagctgtgctgtacagtggatatatatatacatgacat

gagaccgaattcaaggttcatacgactactagatcctacgtagtcagctggtcttgctaa

taaaatgacaatgcaaacccttctagagcctactgttttcacaactacggagtatatgct

actcccctcgtcccataatataagagcgtttttgacactagtgtagtgtcaaaaacgctc

ttatattatgggatgaagggagtagttatcattaatacctgatctttcactccttaaagg

ctccgatgttgaattcaggatgctaaattggttgttctgtgcag

TATTCACCAGCTATTGACATATGGAGTATTGGTTGCATTTTTGCGGAGATTTTAACTGGG

AAACCTTTGTTTCCTGGTAAAAATGTAGTTCACCAGTTGGATTTAATGACTGATCTCTTG

GGTACGCCATCACTGGATACTGTTTCCAGG

gtacgcttttccattcatgattcatgatccaaagtaaggtatccataaatgtcaagcatc

ctaatccctttcaactgcag

ATCCGGAATGAGAAGGCAAGGAGGTACTTGAGTAGTATGAGGAAAAAACAACCGGTATGT

TTTTCTGAGAGGTTCCCCAAAGCAGATCCTGCTGCATTCAAACTTATGCAGCGGCTTTTA

GCATTTGACCCCAAGGATAGACCAACAGCAGAAGAG

gcaagtgtttctagataaaactaaagattcaaaaacaggtgttaaaaactttgtcattct

aacaatgtttatgtgcacatcataatcag

GCATTAGCTGATCCATATTTTAAAGGCCTTGGGAAGGTAGAGAGAGAACCATCCTGCCAG

CCAATATCGAAATTTGAGTTTGAGTTTGAACGGAAAAAGGTGACAAAAGAGGACGTAAAG

GAACTTATATTCCGCGAGATATTGGAGTATCATCCTCAACTTCTCAAGGATTACATGAAT

GGAACTGAAAAAACGAACTTCCTATATCCTAG

gtttttaccataagatctcctcgattttttttccattaaataccaattttcataactgat

ccgttcttggttcctatgttattatgaag

TGCTGTAGACAATTTCCGGAGGCAATTTGCTAACTTGGAGGAAAATGGAGGGAAGGGAGG

GGCAATCGTTCCATCGGACAGGAAGCATGTTTCGCTCCCCAG

gtacccactgcaagattttaccaatagtatatgctgttatgttctgctttttgttaaaaa

aaagatgccattaaattaaataaatagctcatgtgtgcatggttaaaatatagttcattg

gtgatctcatggtactatggaatcgctttccatgacaaaagatggtcaggtagtcaggaa

tttcagttcaagtttgatgatttggcacgataatttcagagactgtcactcaactttgct

tgctgacatttagtttgcatttgacag

GACTACTACAGTTCATTCTACACCAATTCCTCCAAAAGATCAGAAGTCTTCCCAGGTTCC

CCAAAGGATTCCAACAG

gtagtagtttctcctaagtagaagtctagtgttctattttcttcagatggcaccaatatg

atatgtatattatcctttgctgcaaaatttgcag

GTAGACCAGGAAGAGTGGTTGGCCCGGTAATACCATTTGAGAATTCATGTGCTATGGATC

CTTACAGTCAACGAAGGGTGGCAAGGAATCCAGTACTTCCTGCAGCTGCTACCAATGTAT

CAGCATACGCATACCACCGAAAGTCAGACAGTTCAGAGAGAGAGTTACAGCAGGAGCTTG

AAAAAGACCGCATGCAGTACCAACCGATGCAGCGTTTCATGGATGCCAAGATGGTCTCCC

CTGACTTGAGGTCTACCTCCTATTACATGCCAAAGGGTGTCCCAAAAGCAGATGTAGCAG

AAAGGACTGGTTTGCAGCCAAACATGATGCAGGGAATTGCCCCGTTTAATGGCATTGCTG

CAGTTGGAGGTAGCTACAATAAGGCCAGTGCTGTTCAGTATGGAGTTTCAAGGATGTACT

AAATGATCTAGTGCAATGGCTACTTCCAAAATGCTTGGCTTAGCTGAAGAAATAACATGG

AAGAATCGAAGTACTTCCTGAGTTGCCTTATAGGCTGAAGGTTCAGACAGTGGATCGATG

ATCTTTGATCGAGGAGGAAGTTGATGTCTCGTGAAGAACAAAAAAAAATAAGATTATGCA

AAGACAACATCCTACCTGTTGATCTAGCTGCCAAGGAAAGAATGACATCTGGTAACTTGC

TGCTAAGGGGAAAAGCCGAAGTTGGGGGTACAAGATGATTGAGGTCTATGTACTTTGAAA

GAACGGAAACATTTCGGAATAATGGAAATGGTGAACTTCTGTACATAAATAACTTGATTT

TTCTTTGTATTACTGTCGGCTAGAGCTGTCAGTTTTGCTCCATATTCATGTGGCAATGTG

ATTTATTTTTCTGGGTTTGAAGCACTTATGCAGTTATGCTCTGTAGGAGTTCAGCGTTGT

AATACTTTGGAGTATGTTTAGATCTGTAATAGAATAACAAGACCAAACTTCAGTAAGATA

GAATGGCAATGAACAGCTAGTTTTGGGGGTTGTTGGCTCAACCGTGGTACTTATTAAACC

AGGGTTGGTATAGAAGAACAGTAGAAACATAAAAGAGCAAGGTACTGATCATTTCC

**>TaMPK20-4(3DL): TRIAE_CS42_3DL_TGACv1_250331_AA0866300.5**

GGAACGGATCTCCATAAAATCATCCACATCGTCAATCACATGGTGACCCACCGCCGCGCG

GCTGTCAAAAGAAAGAAAACAAGCAAGCACTTCTTTTTAACGCCCGCCCGCCCGTCAGTG

GTTCCCCGAACGAACCCGATCGCTCGCACCAGACGAACGAGACGAGACGAGACGAGGAGG

AGGAGGAGGCACGGAGCTCCTTGTGTTTGGGTTTGCCCTTTGCGTTGCCCCACCCCCACC

GGGCCACAGGACGAAACCAACGCCACAACAACTCACACCCCCTTCGCCCGCGAGTTGCTC

AGTCGCAGCGAGCCGGGCCACAAGACACGAGCGTTCCGGTTCAGGTTGGGGTTGGGGTTG

CGCTCCCTCGTTTCATCGTCGATCCGCCCGACCCGCCACACCGGCCCGGGGACAGACAGA

GATCTCTCCCTCGCGCGCGCGTGCCGGCGGTGACAAATGCGGCTTGATTCGCCGGCGGAC

CAGCGGCGGTGAGCGATGCAGCCGGACCAGCACCAGCAGCAGCAGCGGAGGAAG

gtgagaccgtgcgccacgccaggggttgctgtttgttgcggggtggttggctgcctttcg

gtgcccgtgtgggttgttggcgcttcgagatcggtcggttcgtccggcgggttggatgaa

cctacctcgcctcgggggaggattcttttttctttttgatttgatgaggagggggcactg

ggctcgtgggatccggggggttccgccgcgcacgcccgtcagaattatctcttgccattg

gtgtcctgcaacttctcttctccttccgtcgacaattccttccttccgtataagttgagg

ttgttgatttcagcttcgcagtccagctgaattattttcttccatttcttatcgcgaaaa

agggatccttttcactcctgtctctggatcacaattcacctcaattattaacacaaggtt

ctctgttggatttccctagtgggtgctttcctgaaatcttcttctgcggttgggctgcgt

cttttcttgcattgtccctctataaaggaaatggtattcttttctaacttttccattcca

tgctagtagttgagtactcgttgcttcgtaaaggattactgaggccttttcaactcctgt

aatctacaccaggggaaagaaaaaaaaagggtgattcagatgcaacctaagaggtgccac

aaacgcaaatgcggtattagattcgctgagtagatatgtttatctcaactcaacccaaat

ctgtggtcttcttaccgtcttttttcttgcaaaaagttatagccaatcatcttaattgtc

tcagtggattaggttaggttgtcatattctgtgcccacttggaaggcatattgatattga

tatagatggttttatgcccaagaacacatccttaaatagtcattcttgggccctttgtgc

tcgataagcgctgttttcctcattgctggaggccaaggagttttgcatcggacagggagt

tgtagctaccgtctttgcgcaccgtgatccagaagcttacggaatccaactttttgtgct

gtatacaactctgtgagtcagttggatgcagattacaagtctgctgtttcagtggtctgt

tcggtatgaagtggcctttatacaaaatttgactgttgtattgaggaatttttttgggcg

gtggaaaacctgggtttaacttctttttgtcctttaagttgtttcattgagagcctatgt

ggctagtatgtattatcacctggggtttatccaaaggatgagccgaaccttttctggtcc

tttactaagtttatttgctggcaggctgggccatctgacattgagctcaatgcttgaggc

agatattactcagtaaattgggccttcttttctgtatgcgtgtcttttgtttcagttgtg

gaaaggttctccattgcgcagtattaactctgtttttttccacttctctcag

GGTTCATCGGAGATGGACTTCTTCAGTGAATATGGCGATGCTAATAGATACAAAATTCAG

GAAGTCATCGGTAAAGGGAGTTACGGTGTCGTTTGTTCAGCTATTGACCAACATACTGGC

GACAAGGTGGCAATCAAGAAAATACACAATATCTTTGAGCATTTATCTGATGCTGCTCGG

ATCCTCCGTGAGATCAAATTACTCCGCCTATTGAGACATCCTGATATAGTTGAGATCAGG

CATATAATGTTGCCTCCGTCAAGGAGGGATTTCAAGGATATTTATGTCGTCTTTGAGCTG

ATGGATACAGACCTCCACCAAGTCATCAAGGCCAATGATGACTTAACCAAAGAACACCAT

CAGTTCTTTCTCTATCAGATGCTTCGTGCACTGAAATATATTCATACCG

gtatgctacttttttcaaattgagatctgagttagtatgcagttctctattgtcgtccta

taatcttttttgaatatccaggagtaggtattttatccataccctatctgtagttcgctg

ctgatgctttgctccgctccatgtggagcatacacaatttggagaaatgcactcacacat

aaaattcactctagtgccaaagttaaaaaggcactaggcgttaattgtgcgttttgcaac

cgccttgtgcttttctgagcaaaccatacacttaagcgcaactatacgcacattaaccac

aattaagcgctaggcattttgctatcgcctagagcctaggcgtgcttaagcacccgcctt

aggcgcgccttttttaaccaagtgctaacccaataaccatggccgccatattttagagaa

aattgtcctttgtcccacattagttatgttcttgttgatcatttgtcttctgatgatttt

gatatttggcctttcattgacctgaaaaatatctcccactttgtttcatgctttttgttg

cgaaatgtacacttctttgtatttacctttaatgtcatcatgattattttccactatcca

ttgttgagctactaacattatcagcttcaaaaatcatccatttccaatttaaaatgcatg

ttcatttaccattgtctgtgacctgaccaatatttatatgattgcag

CTAATGTTTATCATCGTGATTTGAAGCCAAAGAATATATTGGCAAATGCTAACTGTAAAC

TCAAAATATGTGATTTTGGGCTAGCACGAGTGGCATTCAATGACACTCCCACGACTGTAT

TTTGGACG

gtatgtaaattttggtagactagaatcacatcctgtgttatttgccaaattagatttgta

ttcaaaggtagtcttttggcacttaatttttatcttctattcatcattgtttgcag

GATTATGTTGCTACTAGATGGTATAGGGCTCCTGAGCTTTGTGGATCTTTCTTTACTAAG

gtaactagtctgcacatctctgttgtgtttgctaggatatcttgtgcgctactgtccaca

taaacatgcctcatgcaattgctgggcaggctacgctgtttaataccacattattcttgc

gcacatagtaataaaagaagataaatcagtgtcacaactcacaacctatacaaggtttca

ctatggtgttccatgtagtctacagctgtgctgtacatatatatatatatatatatagat

gaaatgagaccaaattcaaggttcatatgactactagattctacgtagtcagctggtctt

gctaataaaatgacaatgcaaacccttctagagcctactgttttcacaactacggagtat

atgctactccctccgtcccagtgtagtgtcaaaaacgctcttatattatgggatggaggg

agtagttatcattaatacctgatctttcactccttaaaaggctctgatgttgaattcagg

atgctaaattggttgttctatgcag

TATTCACCAGCTATTGACATATGGAGTATTGGTTGCATTTTTGCGGAGATTTTAACTGGG

AAACCTTTGTTTCCTGGTAAAAATGTAGTTCACCAGTTGGATTTAATGACTGATCTCTTG

GGTACGCCGTCACTGGATACTGTTTCCAGG

gtacgcttttccattcatgattcatgatccaaagtaaggtatccataaatgtcaagcatc

ctaatccctttgcaactacag

ATCCGGAATGAGAAGGCAAGGAGGTACTTGAGTAGTATGAGGAAAAAACAACCGGTATGT

TTTTCTGAGAGGTTCCCCAAAGCAGATCCTGCTGCACTCAAACTTATGCAGCGGCTTTTA

GCATTTGACCCCAAGGATAGACCAACGGCAGAAGAG

gcaagtgtttctagataaaacttaagattcaaaaacaggtgttaaaaactctgttatcta

acaatgtttatgtgcacatcataatcag

GCGTTAGCTGATCCATATTTTAAAGGCCTTGGGAAGGTAGAGAGAGAACCATCCTGCCAG

CCAATATCGAAATTTGAGTTTGAGTTTGAACGGAAAAAGGTGACAAAAGAGGACGTAAAG

GAACTTATATTCCGTGAGATATTGGAGTATCATCCTCAACTTCTCAAGGATTACATGAAT

GGAACTGAAAAAACGAACTTCCTATATCCTAG

gttttttaccataagatctcgattttttccccattaaatgccaattttcataactgatcc

gttcttggttcctatgttattatgaag

TGCTGTAGACAATTTCCGAAGGCAATTTGCTAACTTGGAGGAAAATGGAGGGAAGGGAGG

GGCAATCGTTCCATCGGACAGGAAGCATGTTTCACTCCCCAG

gtacccactgcaagattttaccaatagtatatgcttttatgttctgctttttgttaaaac

taagatgccattaaattaaataaatagctcatgtgtgcatggttaaaatatagttctttg

gtgatctcatggtactatggaatcgctttacatgacaaaagatggtcaggtagtcaggaa

tttcagttcaagtttgatgatttggcatgataatttcagagactgtcactcaactttgct

tgctgacatttagtttgcatttgacag

GACTACTACAGTTCATTCTACACCAATTCCTCCAAAAGATCAGAAGTCTTCCCAAGTTCC

CCAAAGGATTCCAACAG

gtagtagtttctcctaagtagaagtctagtgttctattttcttcagatggcaccaatatg

acatgtatatatcctttgctgcaaaatttgcag

GTAGACCAGGAAGAGTGGTTGGCCCGGTAATACCATTTGAGAATTCATGTGCTATGGATC

CTTACAGTCAACGAAGGGTGGCGAGGAATCCAGTACTTCCTGCAGCTGCTACCAATGTAT

CAGCATACGCATACCACCGAAAGTCAGACAGTTCAGAGAGAGAGTTACAGCAGGAGCTTG

AAAAAGACCGCATGCAGTACCAGCCGATGCAGCGTTTCATGGATGCCAAGATGGTCTCCC

CTGACTTGAGGTCTACCTCCTATTACATGCCAAAGGGTGTCCCAAAAGCCGATGTAGCAG

AAAGGACTGGTTTGCAGCCAAACATGATGCAGGGAATTGCCCCGTTTAATGGCATTGCTG

CAGTTGGAGGTAGCTACAATAAGGCCAGTGCTGTTCAGTATGGAGTTTCAAGGATGTACT

AAGTGATCTAGTGCAATGACCACTTCCAAAATGCTTGGCTTAGCTGAAGTAATAACATGG

AAGAATCGAAGTACTTACTGAGTTGCCTTATGGGCTGAAGGTTCAGACAGTGGATCGATG

ATCTTCGATCGAGGAGGAAGTTGATGTCTCATGAAGAACAAAAAAAATAAGATTATGCAA

AGACAACATCCTGCCTGTTGATCTAGCTGCCAAGGAAAGAATGACATCTGGTAATTTGCT

GCTAAGGGGAAAAGCCGAAGTTGGGGGTACAAGATGATTGAGGTCTATGTACTTTGAAAG

AACGGAAACATATCGGAATAATGGAAATGGTGAACTTCTGTACATAAATAACTTGATTTT

TCTTTGTATTACTGTCGGCGAGAGCTGTCAGTTTTGCTCCATATTCATGTGGCAATGTGA

TTTATTTTTCTGGGTTTGAAGCACTTCTGCAGTTATGCTTCTGTAGGAGTTCAGCGTTGT

AATAGTTCGGAGTATGTTTAGATCTGTAATAGAATAACAAGACCAAACTTCAGTAAGATA

GAATGGTAATGAACTGCTAGTTTTGGGGGCTGTTGGCTCAACCGTGGTACTTATTAAACC

AGGGTTGATGTAGAGGAACAGTAGAAACATAAAATAGCAAGGTACTGATCATTTCCAAAT

TAACTGAATGTCTAGCCTTGTCATAAAAGTTTGACCAAGTCAATATATCAACGTTTGCAA

G

**>TaMPK20-5(1AL): TRIAE_CS42_1AL_TGACv1_001596_AA0032670.1**

ACCTGCCTGCTTAGACTTGTTTAACACCCAGTACCGCACCACCGCTTCCTCCTCCCTTCT

CCGGGTGACATCGCCGCAGACCGGACCGGCGGCGAAGCGCGGCCTTCCGTGCTCCTCAGG

ATGCTCGAGGAAAATGCGGGTGCCCACATCGGCGGCGAGCAGCGTGACAAG

gttcgagtcattttgttgtagtgtgcgctatttacggtctgcacacacctaccacgcaag

gggcagtcccattcaaatcttttcacttcctttttatagtttcagttatacttttcttct

ccccatcctcgccggtgacaagaaatggtcgattttctctctgaaacttggtatagaaag

ttcattaccaataccaatttatgttgtagatacacctctagctcatacattgtcgagatt

ggagttcatgcatatcgtgtacgcacgcctctaggaagcatctgtgggcggcacgtggta

tctcgtcacgacgcatcgcttctttccaaaaacagttctacctcacagactctagtcctc

actagatgcgatcaagagcggcgtgtaggtggtatcggtggcgactctagaatgtaaacg

gaggacacacttgaacaagtgaggaggtgcttggatgggcctagaagtagccgatttgct

gggctgggccgttatcatcactagtagtaaaaacaaaacccatctggttggagggcaggc

tcaagcctgttaaagcctgtacagtagattcgcctctactcctcggcgggtgtgcggcat

ccgcgtgaattacaaccaccaatgttttttttgcacgacgcatatataagcgggttaacg

ggtcacaccgaagagcacccgtgctgtcctcactcacacgcaaagggacagtagcccaag

cccaccaacccacacccgatagggaaataccgaaaacaccctcgctgttgcccgtgaact

acagcagaaccagacctcacttcccccttccactggagggacaagactgtccaccccagc

ccaaatatttttattaatagcacctcgcgtcgcccttgtgctgctcagctcacctccccg

cgcctgcctgcttggacttgtttaacacccaccaccgccccctcctccctcctccgggtg

acgtcgccgcggaccgcaccggaccggcagcgaggcgaggcgcggccttctacccccccc

cccccccccccccccccccccccccccccccnnnnnnnnnnnnnnnnnnnnnnnnnnnnn

nnnnnnnnnnnnnnnnnnnnnnnnnnnnnnnnnnnnnnnnnnnnnnnnnnnnnnnnnnnn

nnnnnnnnnnnnnnnnnnnnnnnnnnnnnnnnnnnnnnnnnnnnnnnnnnnnnnnnnnnn

nnnnnnnnnnnnnnnnnnnnnnnnnnnnnnnnnnnnnnnnnnnnnnnnnnnnnnnnnnnn

nnnnnnnnnnnnnnnnnnnnnnnnnnnnnnnnnnnnnnnnnnnnnnnnnnnnnnnnnnnn

nnnnnnnnnnnnnnnnnnnnnnnnnnnnnnnnnnnnnnnnnnnnnnnnnnnnnnnnnnnn

nnnnnnnnnnnnnnnnnnnnnnnnnnnnnnnnnnnnnnnnnnnnnnnnnnnnnnnnnnnn

nnnnnnnnnnnnnnnnnnnnnnnnnnnnnnggggaacggacggacagagtcgctttctgc

gcagaaagacttcttcttcctcctcttctttttatttcctgccttgtcggcgccggccat

gggttccttcgcggtgctctgcttctgcgggctgggtcgtgctgggatagaggggcgctc

tccctcctggatttggggcggataaagatgtggatttcggggaggattgggtttccccaa

acaagaggagtctgcttcctgcccgcgaggtggacggattggcttgctcgcgccgcgatt

tcttgccttgttagctaccaggaggtccccgggtccaaatccccgggcaggcggcggcat

atccgccctctgtttcctattcaagtcctgggaaatctctcgcttcagcgccgcggagac

ccctcttttggcagatgttgatgaggggtcggtcggtcggtcgccgtggttggtaaatgg

ggtagtattcgattcggtgaccgggcaagtttatccaaatctgcagttactttcctttaa

gtcagagattggataacgctggcgctgcgcatatattcagtccagtccgtggaatccatg

cctacccaggagttgcttagaagtttcccgatattttttcagccaaagggatatatatat

atatatatatatatatatatatatatatatatatatatatatatatatatatatatatat

atatatatatcaaaacacaagtggtggtaactaccactgcttgtagaaaacatttgtcct

atatatatatatatatagcctttttcaggaggggtcgttcatcagcctcttgacatttgt

tctctgaattggttgctgtcagttttctgattttgggtgggtgagtacaggccgagaaag

ttccgattatggcgcatcagtagtagttgttacacgtattattatgattccaagttgtaa

atatatgtttttgtttatctgaactattataagctaggttgaatttgtggctgttttttc

aagccaacgcctgatctggatgtctttttcatttgcaaaagagaatttgccatgtgcact

gtgctaactttctccacgaattttcag

GTTCAGAGTTCAGACGTGATGAGTTTCTTCAGTGAATATGGAGATGCCAGCAGATACAAG

ATCGAGGAAATCATCGGCAAAGGGAGTTATGGAGTTGTGTGTTCAGCCATCGACCGGCAG

ACCGGCGACAAGGTTGCCATAAAGAAGGTGTCCAACATCTTCGAGCATATAACCGATGCC

GCCCGGATCCTCCGCGAGATCAAGCTTCTCCGGCTTCTCAGGCACCCCGACATCGTCCAG

ATCAAGCACATAATGCTGCCTCCCTCCAGGAGGGACTACAAGGACATATTTGTTGTCTTT

GAGCTCATGGACACAGACCTCCACCAGGTTATCAAGGCCAATGATGACTTGACAAAGGAG

CACTACCAGTTCTTTCTCTATCAGATGCTCCGTGCGCTCAAATATATCCATACTG

gtaattatataaggatcttctttcgagattgattctattctcccctcttacgccccccct

cccccaatgaaactgaaagaagaaagctcttctagttctatttatagtatagtatcatca

ctgtggtgcttcaatgtttatttagagttacagtagccaatttcacttcatttaaccaat

gatgatgatatcctattttcatctgcattgctcaaaattaaatgtttatcatctcttgtt

atgtattgcctgcttgctctgtgctgatgactaatctttgtggctgcag

CTAATGTCTATCATCGTGATTTGAAGCCAAAAAACATATTGGCAAATGCAAACTGCAAGC

TCAAGATATGTGATTTTGGACTAGCAAGAGTTGCATTCAATGACACCCCTACAACTGTTT

TCTGGACG

gtatgttacttctacagtttactagatttgatccacaatactctggttttgtcaattctg

cttgacagattcgatagaatgctacattctgtacaaaagagccctacttgaaacttaaag

ctcactgtcttccccaacatggtttgcag

GACTATGTTGCCACTAGGTGGTACAGAGCTCCGGAGCTCTGTGGTTCTTTCTTTACCAAG

gtaattaataatttcctcttcctgtgcttttgttcattttagtactgtctgtggcattcc

gctacttcctcattctatagggttgtatagcctaagattctaggtgattagtagtagctt

ttgatccttattctgtaatctatggatacatcactgtgttttagaggagtacagcctcgt

agttataacaaggtgtttgttcaagcactaaagttgtgtacaaattgatatacattacgt

ggtgttacatttgccatcatttgtttatcctcttcagtttctatattaacttgatgacac

taagttgactccctcaatgcag

TATTCACCGGCTATTGATACATGGAGCATTGGTTGCATTTTTGCGGAGATCTTGACAGGA

AAGCCTTTGTTCCCTGGTAAAAATGTGGTTCAGCAGTTGGATATGATGACTGATTTCTTA

GGCTCACCGTCGCCTGAGATTATTTCTCGG

gtatgatttactgtttgcagaaatgtcgtcaatcaactactcaagcataaggtccaattc

tctaatattttttatcaccgcag

ATTCGAAATGAGAAGGCAAGGAGGTATCTTAGCAGCATGAGGAAGAAACTGCCAGTACCT

TTTTCAGAGAAGTTCCCCAAAGCAGATCCTGCAGCAGTCAAGCTCTTGCAAAAGCTTCTA

GCATTTGACCCAAAGGACCGACCCACTGCTGAAGAG

gtaggcgtgtcttagtgaaacaagatgattttcatttttctagtattttgtgctgtcatt

tgttttactacttaaaacgaatcacatattcaaggtcgtctgtatgttattctaactagt

gttctatgcataatcag

GCGTTGGCTGATCCCTATTTCAATGGCCTCGCGAAAGTGGAGAGAGAACCATCATGCCAA

CCGATTTCGAAAATGGAGTTTGAGTTTGAACGGAGAAAGTTTACCAGAGAGGACGTCAAG

GAACTTATATTCAGGGAGATATTGGAGTACCACCCTCAGCTTCTCAAGGATTACAGCAAC

GGCTCAGAGAAAACGAACTTTCTATATCCTAG

gtttctaccttgacatagtatctttaagctcttttctctttgttattctgtctatctatg

tagcaatactcatcatggctcttgaattttctgttatgcag

TGCTGTCGACAACTTCCGGAGGCAATTTGCTAACTTAGAGGAAGATGGAGGAAAGGGCGG

GGCAGCCGAGAGGAAGCATGTTTCTCTCCCGAG

gtaacttacactttaaatacagttgttctgaatgtgatcaattatatctggttctcaatt

atttgtgccgccatacatagcttgtgttgaaactgattaaaactaccaaaatgcattagt

taatctagaagtgtggagccctagaatacagaacttttgtttgagtttcactattaaccc

ttgattggctgcttgcag

GACTACAACAGTTCACTCTACCCCAGTTCCTACAACAAATTGTCCGGCCTCCCAAGCTCC

TCAAAGGATTCCAGCAG

gtaactggtcctgtttgcttttgtctggtacctcttcatttgaacttcagagagagcacc

agtctaacacatttcatcttcggcactgttgcag

CCCGACCGGGCAGAGTGATTGCCTCAGCGACACCAACCGAGAACGCGGCCTTCACCGATC

GACAAATGGGTCGAAGAATGGCGAGGGACCCCGCGGGGGCTCCAGCAGCGGCTGCCGGTC

ACCACCTGAGGCCGGACTGCTCCGACAGGCAACATGAGGTGGAGAAGGACCGCGCGCACT

ACAGGCCGTCGCACCATTTCAGGGACGCCAGGGTGGCGCCCGAGGCCGAGGCGCGGCCCT

CGGCCTACTACATTCCCCCGTTCAATGGCATCGCAGCGGTTGGCGCGGCAGGAAGGATGT

ACTAGTGCAATTGCTCAGCTGAGTTTGCCGTGAGCTGGAGGACGGCCGCGATCGATCGCC

GTTGCGGGGAATGCAGGGGGGAGGGAGGGAGGGTGAAGTTGTCTGAAATTTAGGAGGTAG

GAGACAGCCGAACAGGTGGTGCTCTTGTGTATATATTGCCGGCAGCCAAATTGGTTGGTT

CTGTGGTATGTTTCTCTGTGTCGATAGATAGGTTCAGAGTCCAAACAGTATGCTCTGCTC

TGAGAGGTGAAAGGGCCTCTGTTGAGGAGGGGAGGGAGGGGGTTGGGGGCTGTAGAGTTT

TTGTTTTTGTTTCCGGATGGTGTTAATAGGATGGATGAATAAATGGATGATGGAAGCTCT

GATCCGTG

gtgtgtcaactcaactcttgtgtctgctagctgctggaatgtggtgtctgtttttcattt

tttttttgtttgctccgttagaccggcctggttgcctctgccgttggcatcacccggtcg

ccggcgattagccggagccattaattagcggacaagaatgatttgtgaatgcaatgcgaa

gtgtggtctgcttcttgcttgtgccactcgatccaggcagcaggggagaggagaccaccc

aagctgagtgatgaagagcagaaatatctcatccgacgatatgggtctacacacacaggc

acagccactctttttttttttttttttttttttgagaaaacaggcacagccactcgagcg

cagcggcgcgggccacccgcaggcagagcccgcgatcgacgagacagcatataggaacga

gcagtattctagaatttgcggttgcagcggagcacgggccccgatcgactaaccagtggc

gacggcggagcatagcagcgtgggatgacacgtttcgagccaggacagggcagcccccgc

atgccgcgctgtgtaaacatattcggcccaacatgacccacgccgggcccgacggcaccg

agtatactacttgttggcatttctaaaaaaaaaaaaaacttgttggcgcgacggcggcgt

gcggccggccttggcgacaatcaacccgacggcgcacctgccgctcgctggtgactcatc

gacgatggcgaaagggaggcggatctcttcttcttcttgctgcagtgagtgatgccgctg

atgagtccatgtctggtggtacagtgcagaggatccttccttgggtctccatcggcttcg

gccgtcgtcgccttcgtcatcgtgcgtgcggtcgctgctgctggttgagcactcgatgga

ctgggacgtaggtacgactacttagcccctttggagcccacaaacggcccatgtctgatg

tctctctcaatacggataccaatttgttcggcccagtagcccaccacaccacaccacacc

acaccacaccaaagaagacaaagcgccaggtctctcctcgtatatatatatatatataac

ggaggctgaccag

GAGCATCGATCGATAATTCGATATCTAATCCATCGTTTCCGCCTTTCGGCGCCGACTAGC

CCGCCTAGGAAAGTGATCGCCTGAAATTCGCCG

gtgagtcttctttagttcgtactatgtactagtagtaatttatacggcgaactgatacgt

acttactacggtagtaagctgcagaaattcttgggttagacggaattaattagggcaata

atttctcgtttatctcacaaatatatgtagtactgcacacacggattcattctcttgttt

gagttagtctgtccgttgtcaccgatctgtatttttgtgtttgcgcagtcctgtttgatc

ttcttggattatagcgtcaaagtttagttctcttctgacacactggattctagcgtcaaa

tatgcactgtaaattaggcatggcgctagattctagcgccaaagtttagttctgatgaaa

aattatagacatcttcgatcttcttggtaatcgcaaggccaatgtacactcaagtccgtg

atgggagttaattcatactgaatgtttacacatttaacttcagtctcgatgtaagttggt

ccctaattctgcaattcctgtaaccgtgatggacgggacttgaagaaaaatcttcctgtg

aaatctttctggagttgaagaaaaattctagattttggtgacccaagctgtgtgtgtgta

catagttctagtttttattgctatgagttcaagtgatagctcaacttattgttgtcactt

cttactgccgtcactactgataactcaacttattattgtcacttctagaacagaggtact

gactgttcacacatttgacttcag

TGTCGATGATCCTCGGAAATGGTGTTCCTGATGCCCCAGCCCCTCCACCACCCCTCGACT

CAAGGCCTGTGCTAGATCCTAGGGATTTCCAGAATAAATGGAGCCAACTAGCATTATCTG

TTTCTCAG

gtgcgttgcttttaagtttttgtccctgctacatttcatgattatgggcagcgcacgcag

catactcttctgtctgtctttttatcattgcctggtcaagtttcttatgattttagcaaa

gtgatcgcgtgaaatcctatgttccgccgctgtctaatttgtcttctcatgaagctcatc

tcgcctcagctgagtttgaaattagccgttctgatgtagtgctgttctatgttcctctaa

atggtttcacttggtttgagattattatgattttactttggactttggtagtatatcaca

agaggattccatgtgatgctctgctgctgtttgtcgtgttcgtgtatgggtccgcccttg

aaattcatcacattgatgagctcgacatctgtaattaattaacaaactttcttctcttgt

gtggctttgttctgttgtag

GAATGCTCTTTAACTCCACAAGGGGCAGCTTCTTTGATGAATCCCCAATCGCTCATTCGT

CATATGCAAAGCAACTACATTCAATGCGTTGCTTCAGGCAGTCAGCCTCCAAACTGCAAG

TTCTTCTTTTATGGTCAGAAACCCGGGGCTGCTGATTTCTACCTTGTAGAGTGCACTGTG

AACACAGCATCACCGAAGGCCAAGCTCAAAATTAAGGCCGACGATGGGGCTACCGCTGAG

GCCTTTTGTACTTTATTTCAGTCAGTATTGTCTGAATTTGGGCTTTCTTGATCATGAGAG

ACTAATTTTCTTCAAGGTTATGGTGGTTTAGATGGTGTGTAACCTTGTTGCTGGAGTGGA

CATGAATATCATCTCCGTTGATGTGGAGAGAATAGATGTAAATCTGTAGTAATTTATTTG

CGGGGAGCAATAAAGCAAGAGTATAGTCGACTTGTTC

**>TaMPK20-5(1BL): TRIAE_CS42_1BL_TGACv1_032512_AA0130820.2**

GCCACTAGAGGGGCAAAACCGTCCGCCGAAGCCCAAACATTTTTATTAATACCACCTCGC

GTCGGCCTTGTGCTGCTCAGCTCATCTCACCTCCCCGCGCCTGCCTGCTTGGACTTGTTT

AACACCCACCACCGCTCCCTCCTCCCTCCTCCGGGTGACGTCGCCGCGGACCGCACCGGC

GGCGGGGCGAGGCGCGGCGCGGCGCGGCCTTCTATCTCCCGTGCTCCCCAGGATGCCGGA

GGCAAATGCGGGTGCCCGCGGCGGCGGCGAGCAGCGCAGCAAG

gtgggggaacggacggacagagtcgctttccgcgcagaaagactccttcttcctcctcct

cttctttttatttcctgccatgtcggtgccgccgcgccggccatgggttcctaagcggtg

agccgcggcggcgctctgcttctgcgggctggctcgtgctgggacagaggggcgctctcc

cccctggatttggggcggataaagatgtggatttcggggaggattcggacggattcggtt

tccccagacaagaggagtctgcctcccgcccgcgaggacggacggattggcttcgctccc

gccgcgatttcttgccttgttagctgctgtgtccaaatccccggggaggcggcagcatat

ccgctcgctgtttcctattcaattcctgggaaatccatggcttcagcgccggggagaccc

ctcttttggcagatgttgatgaggggtcggtcggtcggtcggcgtggttggtaaatgggg

cagtattcgattcggcgaccgggcaagtttatccaaatctgcagttactttccttttagc

cagagatggataacgccagcgccgcgcatatattcagtccagtccgtggatggaatccat

gcctacccaggagttgcttagaagtttcccgatattcttttcagccaaagggatatatat

atacatccttttccaggaggggttgttcatcagcctcctgacactgttgttctttgaatt

ggttgctgtcacttttctgattttgggtgggtgagtacaggccaaggaagttctgattat

gggtcatcagtagtagttgaattcgtggctgtttttcaagccaacgtctgatctggatgt

ctttttctttttcttttgcaaaagagaatcaatttgccatgtgcaccgtgctaactttct

ccacgaattttcag

GTTCAGAGTTCGGACGTGATGAGTTTCTTCAGTGAATATGGAGATGCAAGCAGATACAAG

ATTGAGGAAATCATCGGCAAAGGGAGTTATGGGGTGGTATGTTCAGCCATCGACCGGCAG

ACCGGCGACAGGGTGGCCATAAAGAAGGTGTCCAACATCTTCGAGCACATAACCGACGCC

GCTCGGATGCTCCGCGAGATCAAGCTTCTCCGGCTTCTCAGGCACCCCGACATCGTCCAG

ATCAAGCACATAATGCTGCCTCCCTCCAGGAGGGACTACAAGGACATATTTGTTGTCTTT

GAGCTCATGGACACGGACCTCCACCAGGTTATCAAGGCCAACGATGACTTGACAAAGGAG

CACTACCAGTTCTTTCTCTACCAGATGCTCCGCGCGCTCAAATACATCCATACTG

gtaattacataaggatcttcattcgaagattgattccccccctcttttttccccccatga

aactggaagaagaaaggtcttctagttctatttatcatctcttgttatgttttgcctgct

tgctctgttctgacgacgaatccttgtggctgcag

CTAACGTTTATCATCGTGATTTGAAGCCAAAAAACATATTGGCAAATGCAAACTGCAAGC

TCAAGATATGTGATTTTGGACTAGCAAGAGTTGCATTCAATGACACCCCTACAACTGTTT

TCTGGACG

gtatgttacttcttgtacatcttactagatttgatccaaaatactctagttttgtcaatt

ctgcttgacgaattcgatagaatgctatgttgtgtgccaaagaaccctacttgaaactta

aagctcaccgtcttctccaacatggtttgcag

GACTATGTTGCCACTAGGTGGTACAGAGCTCCGGAGCTCTGTGGTTCTTTCTTTACCAAG

gtaattcatagtttcctcttcctgtggttttgttcattttactaccgtttgtggcattcc

gccacttgctcagtctataggtgtttgttcagccactaaatttctttgtctacaaattga

tatgcattacttagtgttacatttgctatcatttgtttataccttttcagtttctatatt

aacttgatgacactaagtcgactccttcaatgcag

TATTCACCGGCTATTGATACCTGGAGCATTGGTTGCATTTTTGCGGAGATCTTGACAGGA

AAGCCTTTGTTCCCTGGTAAAAATGTGGTTCAGCAGTTGGATATGATGACTGATTTCTTA

GGCTCGCCGTCGCCTGAGATTATTTCTCGG

gtatgatttactgtttgcagaaatgtcgtcaatcaactactcaagcataaggtccaattc

tctaatattttttatcaccgcag

ATTCGAAATGAAAAGGCAAGGAGGTACCTGAGCAGCATGAGGAAGAAACTGCCAGTACCT

TTTGCAGAGAAGTTCCCCAAGGCAGATCCTGCAGCAGTCAAGCTCTTGCAAAAGCTTCTA

GCATTTGATCCAAAGGACCGACCGACTGCTGAAGAG

gtaggcgtgtcttagcgaaacgacatgattttcattttactagtattttgtgctgtcatt

tgttttacttttttttaaaaatcacatattcaaggtcgtctatatgttattctaacgagt

gttctatgcataatcag

GCGTTGGCTGATCCCTATTTCAACGGCCTCGCGAAAGTGGAGAGAGAACCATCATGCCAA

CCGATTTCGAAAATGGAGTTTGAGTTTGAACGCAGAAAGTTTACCAGAGAGGACATCAAG

GAACTTATATTCAGGGAGATATTGGAGTACCACCCTCAGCTTCTCAAGGACTACACCAAC

GGCTCTGAAAAAACAAACTTTCTATATCCTAG

gttcttaccttgacatagtatctttaagctcttttctctttgttattattctgtctatat

ctatatgtagcaatacgcatcatggctcttgaattttctgttatgcag

TGCCGTGGACAACTTCCGGAGGCAATTTGCGAACTTGGAGGAAGATGGAGGAAAAGGCGG

GGCACCCGAGAGGAAGCATGTTTCTCTGCCGAG

gtaacttacatgataactgatcttctgaatggtgatcattatgtctggttctcaattatt

tgtgccgccataaagctgattaaaactaccaaatgcattagttcatcaataggcttctga

aagatctatcattgcataatatggtagaacacatcgcggtactttacggattttgagttt

aactattaacccttgacgggctgcttgcag

GACTACAACAGTTCACTCTACCCCAGTTCCTACAACAAATGGTCCGGCCTACCAAGCTCC

TCAAAGGATTCCAACAG

gtaactggttctgtttgctttaatctggtacctcttcatttgaacttcagagagagcagc

agtcttaaaacacatttcatctctgacactcttgcag

CTAGACCAGGCCGAGTGATTGCCTCAGCGACACCGACCAAGAACGCGGCCTTCGCCGACC

GACAAACGGGCCGAAGGATGGCGAGGGACCCCGCGGCTCCCCCAGCAGCAGCGGCTGCCG

GCTACCACCAGAGGCCGGACTGCTCCGACAGGCAACAGGAGGTGGAGAAGGACCGCGCGC

ACTACAGGCCGGCGCACCATTTCAGGGACGCCAGGGTGGCGCCCGAGGCCGAGGCGCGGC

CCTCGGCGTACCACATTCCCCCGTTCAACGGCATCGCCGCGGTCGCCGGCGGGTACAGCA

AGGTCGGCGTGGCAGGAAGGATGTACTAGCGCAATTGCTGGGCGAGTTTGCCGGGAGCTG

GAGGACGGCCGCGATCGATGGCCGTTGTGGGGAATGCAGGGGCTGGGAGGGTCTCAAATT

TAGGACGTAGGAGACAGCCGGACAAGGTGGTGCTCTTGTGTACATAGATATTACCGGCAG

CCAAATTGGTTGGTCTGTTTTTCTGGGTTGATAGATATGTTGAAAGTTGGAACAGTGAGC

TCTGCTCTGAGAGGTGAAAGGGCCTCAGGGAGGGGGGAGGGGGTTGGGGGCTGTAGAGTT

TTTCTTTCTTGTTTTGTTTCCGGATGGTGTTAATAGGATGGATGGATGAATAAATGGATG

ATGATGGAGGTTCTGATGGCTTCTCCGTGGATGAACTCAACTCT

**>TaMPK20-5(U): TRIAE_CS42_U_TGACv1_643598_AA2133390.1**

ACCCTCGCTCTTGCCGGAGAACTACGGCTGAACCAGACCTCACTCCCCCTGCCACTCAAG

GGGCAAAACCGTCCACCCCAGCCCAAACACTTTTATTAATACCACCTCGCGTCGGCCTTG

TGCTGCTCAGCTCACCTCCCCGCCCCTGCCTGCTTGGACTTGTTTAACACCCACCACCGC

TCCCTCCTCCCTCCTCCGGGTGACGTCGCCGCGGACCGCACCGGCGGCGAGGCGAGGCGC

GGCGCGGCCTTCTATCTCCCCCGGCGACCGGCCGTGCTCCCAAGGATGCCGGAGGCAAAT

GCGGGTGCCCGCGGCGGCGGCGAGCAGCGCAGCAAG

gtgggggaacggacggacagagtcgctttccgcgcagaaagacttcttcttcctcctctt

ctttttatttcctgccttgtcggcgccggcgcggtgctctgcttccgcgggctggctcgt

gctgggatggaggggcgctctcccctggatttggggcggataaagatgtggatttcgggg

aggattcggtttccccaaacaagaggagtctgcttccagcccgcgaggtggacggattgg

cttgctctcgccgcgatttcttgccttgttagctgccgggaggtccccgtgtccaaatcc

ccggggaggcggcagcatatctgcggcatatccggtcgctctttcctattcaattcctgg

aaaatctctcgcttgagcgccgcggagcccctcttttggcagatgttgatgaggggtcgg

tcggtcggcgtggttggtaaatggggtagtattcgattcggtgaccgggcaagtttatcc

aaatctgcagttactttccttttagccagagatggataacgccggcgctgcgcatatatt

cagtccagcccggcgaatccatgcctgcccaggagttgcttagaagtttcctcgtatttt

cagccaaagggatatagcctttttcaggaggggtcgttcatcagcctcctgacatttgtt

ctttgatttgaattggttgctatcacttttctgatttcgggtgggtgagtacaggccaag

gaagttctgattatggcgcatcagtagtagttacacctattattaggatttccgattgcg

aatatatgttgcatcactccgtaaagaaatataaaagcgtttagatggctactttagtaa

tctaaacgctcttatatttctttacggagggagtactatttgtttatctcaagtattaga

agctcggttgaatttgtggctgtttttttcaagccaacgcgatctggatgtccttttctt

ttgcaaaagagaatttgccatgtgcactgtgctaactttctccacgaattttcag

GTTCAGAGTTCAGACGTGATGAGTTTCTTCAGTGAATATGGAGATGCAAGCAGATACAAG

ATTGAAGAAATAATTGGCAAAGGGAGTTATGGAGTGGTATGTTCAGCAATCGACCGGCAG

ACCGGCGACAGGGTGGCCATAAAGAAGGTATCCAACATCTTCGAGCATATAACCGACGCC

GCTCGGATGCTCCGCGAGATCAAGCTTCTCCGGCTTCTCAGGCACCCCGACATCGTCCAG

ATCAAGCACATAATGCTGCCTCCCTCCAGGAGGGACTACAAGGACATATTTGTTGTCTTT

GAGCTCATGGACACGGACCTCCACCAGGTTATCAAGGCCAATGATGACTTGACAAAGGAG

CACTACCAGTTCTTTCTCTACCAGATGCTCCGCGCGCTCAAATATATCCATACTG

gtaattaattatataaggatcttctttcgaacagtgangcttcagtgtttagaattacat

acagtaccctatttcacttcatttaaccaatgatgatgacgatagtcctattttcatctg

cattgctcaaaattaaatgtttatcatctcttgttatgtattgcctacttgctctgttct

gatgactaatctttgtggctgcag

CTAACGTTTATCATCGTGATTTGAAGCCAAAAAACATATTGGCAAACGCGAACTGTAAGC

TCAAGATATGTGATTTTGGACTAGCAAGAGTTGCATTCAATGACACCCCTACAACTGTTT

TCTGGACG

gtatgttacttctacagcttactagatttgatccacaatactttcaattcctctcgacaa

actgggtagcatgctacgctgtgtagcaaagaaacctacttgaaactcaaatctcacaat

cttctccaacatgatttgcag

GACTATGTTGCCACTAGGTGGTACAGAGCTCCGGAGCTCTGTGGTTCTTTCTTTACCAAG

gtaattaaaagtttcctcttcctgtggttttgttcattttattagtactgtttgtggcat

tccgctacttcctcattctacagggttgtatagcctaagattctaggtgattagtagtag

ctttcgatccttattttacaatctttggatgcatcattgcttttagggaagtttagctcg

tagttataataggatgcttgttcaagcactaaatttctttgtgtacaaattgatatacat

tacgtagtgtagtgttacatttgccatcatttgtttataccttttcagtttctatattaa

cttgatgacactaatttagttgactccttcaatgcag

TATTCACCGGCTATTGATACATGGAGCATTGGTTGCATTTTTGCGGAGATCTTGACAGGA

AAGCCTTTGTTCCCTGGTAAAAATGTGGTTCAGCAGTTGGATATGATGACTGATTTCTTA

GGCTCACCGTCGCCTGAGATTATTTCTCGG

gtatgatttactgtttgcagaaatgtcgtcaatcaactactcaagcataaggtccaattc

tctaatatttttatcaccgcag

ATTCGAAATGAGAAGGCAAGGAGGTATCTGAGCAGCATGAGGAAGAAACTGCCAGTACCT

TTTTCAGAAAAGTTCCCCAAGGCAGATCCTGCAGCAGTCAAGCTCTTGCAAAAGCTTCTA

GCATTTGATCCAAAGGACCGACCGACTGCTGAAGAG

gtaggcgtgtcttagtgaaacgacatgattttcattttactagtattttgtgctgtcatt

tgttttacttcttaaataaaatcacatatccaaggtcgtctatatgttattctaacgagt

gttctatgcataatcag

GCGTTGGCTGATCCCTATTTCAATGGCCTCGCGAAAGTGGAGAGAGAACCATCATGCCAA

CCGATTTCAAAAATGGAGTTTGAGTTTGAACGCAGAAAGTTTACCAGAGAGGACATCAAG

GAACTTATATTTAGGGAGATATTGGAGTACCACCCTCAGCTTCTCAAGGACTACACCAAC

GGCTCTGAAAAAACAAACTTTCTATATCCTAG

gttcttaccttgacatatagtatctttaagctcttttctctttgttattctgtctatcta

tgtagcaatactcatcattgctcttgaattttctgttatgcag

TGCCGTCGACAACTTCCGGAGGCAATTTGCAAACTTGGAGGAAGATGGTGGAAAAGGCGG

GGCACCCGAGAGGAAGCATGTTTCTCTCCCGAG

gtaacttgcatgattacagttcttccgagtgtgattaattataccaggttttctgttatt

aacgtttgattggctgcttgcag

GACTACAACAGTTCACTCTACCCCAGTTCCTACAACAAATGGTCCGGCCTCCCAAGCTCC

TCAAAGGATTCCAACAG

gtaactggttctgtttgctttggtctggtacctctcttcatttgaacttcagagagagca

ccagtctaacacatttcatctctggcactcttgcag

CCCGACCGGGCAGAGTGATTGCCTCAGCGACACCGACCGAGAACGCTGCGTTCACCGATC

GACAAATGGGCCGAAGGATGGCGAGGGACCCCGCGGCGCCTCCAGCAGCGGCTGCCGGCT

ACCACCAGAGGTCGGGCTGCTCCGACAGGCAACAGGAGCTGGAGAAGGACCGCACGCACT

ACAGGCCAGCGCACCATTTCAGGGACGCCAGGGTGGCGCCCGAGGCGGAGGCGCGGCCTT

CGGCCTACTACATTCCCCCGTTCAACGGCATCGCCGCGGCCGCCGGTGGGTACAGCAAGG

TTGGCGCGGCAGGAAGGATGTACTAGTGCAATTGCTCGGCGAGTTTGCCGGGAGCTGGAG

GCCGTTGTGGGGAATGCAGGGGCTGGGAGGGAGGGTCTCAAATTTAGGAGGTAGGAGACA

GCCGGACAAGGTGGTGCTCTTTGTGTACATATTACCGGCAGCCAAATTGGCTGGTTCTGG

TCTGTTTTTTCTGTGTGGATAGATAGGTTCAAAGTTGGAACAGTGTGCTCTGCTCTGAGG

AGGTGAAGCGCCTCTGTTGAGGAGGGGGGAGGGGGTTGAGGGCTGTAGAGTTTTTTTTCC

TTTTTGTTTCCGGATGGTGTTAATACTAGGATGGATGAATAAATGGATGATGGAAGCTCT

GATGGGCTTGTTCGTCGGTCTACTCAACTGTGCCTGCTGC

**>TaMPK21-1(1AL): TRIAE_CS42_1AL_TGACv1_002281_AA0040420.1.X (Based on AA0040420.1)**

cgcaccaacccgccgccacgcatggatcccggcaagaaggtgcgtccttgctcgccgtgc

gccctcttctccttaattagtccccttcccccgcggcccccttccgccgttctcgttcaa

tgtcggttgccgtcgatctctcccggatgaaatatgttgccgtgctgccgggattgctct

gttcgcttcgtggattggggaagatccacccttgggattgctctgttcttgctcccttgc

cagttctttgcggatctgttttcctggaaccaccgattttgatgtggtgacggtcaatgc

cagttcttggatagttgttacacttattaatgtcatatacaataaactcataatattagc

ttgtgcaaagtgcgccacacatgaatgccgtccgtgacagcctaataaagcgcggcatta

tttaggagcaagcagtgtcacgggagccctgttttcgctgttcatgtctcatcagagaca

aacatgatgcatggtggaagaggttatgcttttgcttttcaattattggtccttgttgac

gattccacgtttgccacttttggtagcagcaggtagatatattcagaaaaatacactgtt

taaagtaccaaatgttcaaccacttcgtgattcattcagtccaaagtgcaaacccagcac

AAGCAAGAAGCTGTCTCGAGTGACTTTCCAGTTGTGTGCATGTCTTCTGATCCTTTTTGC

CCACCACTATCATTGTTTGTAAGGCCATCAGTATTTTTTTCGGAAAGGGGATTACCCCGG

CCTCTGCATCACAGGGATGCACACAGCCATGTAAGGCCGTCAATATTGTTTCGTATCTTT

GTGAGGCCATCGGTATCTTCAGTTGAGATGTTCTATCTTTCTTTTTCTCGGCGCGGTTTT

ACCACCACTGGTAAATCAGTATGTTAGCACCATCTAGCTCTCTGAACTAATGGTGCCAAT

ATGCGCTTGCTCCTTCCTTTTCAGACCTCGGAATCCGAGTTCTTCACGGAGTATGGTGAG

TTAAACCGGTATCAGGTCAGCGAGGTCATTGGCAAAGGGAGTTATGGAGTTGTGGCTGCT

GCTATCGACACCCAGACCGGCGAGCGTGTGGCCATCAAGAAGATCAATGACGTCTTTGAT

CATGTCTCCGATGCCACCCGCATCCTTAGGGAGATCAAGTTGCTCCGGCTGCTGCGCCAC

CCGGACATAGTTCAGATCAAGCACATTATGCTCCCCCCTTCAAGGAGGGAATTCAGGGAC

ATATATGTGGTCTTTGAGCTGATGGAGTCCGATCTCCATCAGGTAATAAAAGCGAACGAT

GATCTCACACCAGAGCATCACCAGTTCTTCTTGTATCAGCTGCTCCGGGGAATGAAGTAC

ATCCATGCAG

gtgattagactacacaaaggaaatcgataagtgttcattcaacctttttcatctcatgtt

gctgtactgattgtttgtttacatgatggttttgcag

CGAGTGTTTTCCATCGGGATCTTAAGCCCAAGAATATTCTAGCGAATGCTGACTGCAAGC

TGAAGATTTGTGATTTTGGGCTTGCCCGTGTATCATTTAATGACGGGGCTCCATCAGCCA

TATTCTGGACG

gtagccattttagtaatgttgagcagataatgtttatcctgagcagccaaatcttggttt

ctgatgattttgcatgccttgtctag

GACTATGTTGCAACTAGATGGTATCGTGCTCCAGAATTGTGTGGCTCTTTTTTCTCAAAG

gtgatgtaattttcatcctaacttcatattaccaacaacaatacccaggctattaaaaaa

gcacaatgaccatactatagattttcttccaaaattgttgttttaatatgatatgtttcg

atgcaacatagcaatactatttgttgatttcactagtaacacatgttgattaatttccat

ttttgcaactaaagttcccttcccttttagagttgctactacagttctcaaatgtcggtt

caacctgtcatgatctgtatttcatatgttgcactaagttctctcccagtaaaaatgcca

aaaaattgttacttgtggtgttttgccctcttctttcatataatcatagctgaatgtagc

gacctggcttgggcgctaattcaaacttgatgatgtactttgtttcttaattaatgtctg

tgcatgtttagcaaatgctaaaagcagctgaactttgaattttgcag

TACACACCTGCAATTGATATTTGGAGCGTAGGGTGTATCTTTGCAGAAATGCTCACAGGG

AGGCCACTCTTTCCAGGGAAGAATGTTGTCCATCAATTGGATCTCATGACTGATGTACTT

GGCACTCCTTCAGCAGAATCTCTCGCTAAG

gtatgcttactccacacttgtcaccagcaagttagaacaatagttggagttatatatttt

gccacttcctatttttcatattttcataatatgcgaatttcctgcaaaattgaaccttgc

attgataacttgtgtacatggttatgaatttattattggcttgttccatttctctcagta

tcttttttgcatccacgcccaatacttacaaactctgattctccttaatatggtttgcgt

gcttggttactgagctacatggaatacggatgtcggcacgaattggcatcaatatcagca

caatctctcgccatgttatatatttttcatatagaatgaaatgtaaattgtgggatgatg

tttaaaccatatgtcttttacacccttttaccgaatggttgcag

ATACGGAACGAGAAAGCTCGGCGATACTTGAGCAATATGAGGAAGAAGCCTAAAGTTCCC

CTTACCAAAAAATTTCCAGGCATCGATCCTATGGCTCTCCATTTGCTTGAGCGTCTTCTT

GCTTTTGATCCTAAGGATAGGCCAACTGCTGACGAG

gtaggaatggctgagaccaacttctatttactataaagatatattacatctttgaaaatg

ttgggtgaatcactgtcctcagtactgagatatgaaaatgttcttcaag

GCCCTGACAGACCCATACTTTAACGGATTAGCAAATTCAGAACGTGAACCCATAGCACAG

CCCATCTCAAAACTTGAGTTTGAGTTCGAGAAGAGAAAGTTGGGCAAAGATGATGTCCGA

GAATTAATTTACAGAGAG

gtacaattgctcagctattttgtggagatacagtagagctgctgcccgactctgtagtta

gctaacaacttctacatctttcag

ATTTTAGAGTACCATCCTCAGATGTTGCAAGAATACCTACGTGGAGGAGACCAGATGAGC

TTCATGTACCCTAG

gtgagaccattattttcccttagaaacaagatttacttgtgttgagaattttatgatgac

tatgctttactctcttctgcatacactgaattatgccagttatatgtatatgactggttg

atggtatattgcaaactctgatgtttgtgcatgtatgttgacag

TGGGGTGGATCGCTTTAAGCAGCAATTCGCTCATTTGGAAAAAGGTGGTGCAAAGGGTGA

AAAATCCAGTCCACAGTTGCGACAAAATGCTTCCTTACCAAG

gtaatatgctctgaccgagacttaaatgtatacttccaaggcaataaatatctttatgta

gaatgggcatgtcgatacttatggacgaaatatttgttttctaaccaaaatttctttcct

ttgacaaccttgtacaaatttcccaaagatgatctgtttggacttggcaagaatattcaa

caaaataaatactaaatcataagtatcatatttgttttgctttttgcaagttgccagaga

ttttaaatcaaatattttttaatgttaaatttgatagagaataatgtttcacatgatcag

atgcttatatatacatataaaatgagtcatcctacaaatagacaaccttttaagtttttg

tatctaacaattctcgcatcttgtctcag

GGAAAGAGCAATTGGCAATAAGCACGGAGATAGTGACTACCAAGTAAAGCTGAATGCAGG

TGAAAAGCCAGTACATGCATCAGTGACAGATGGAATAAGCAAACCCCTCATGAGTGCTCG

GAGCTTACTGAAGAGTGAAACCATGAGTGCTTCCAAGTGTATAGGTGAAATAAAAAATAA

AGAT

gtgagttcagctcgtcctttgttactacagtaaatatatttgccccctctcttcttacga

aattgctggtatatttgagttccatacttgcaggctgtctttcacctttttttttccttt

ggagtgtttaattgtttatatctctaagatatctatatctaatttgagcaatttcttagt

taatgctgcaatgtccattcaacattttccag

GATGAATACGAGAGCGTGGATGCAGCTGACGGCGTCTCTCAGAAGATCGCTCAACTGAAA

ACCTGATTTTCGAAGGTTACAGAAGCAATCGGTAGTATATACTATGTACAACAGCATGTC

TATAGGCGCTCAAGATCAGAATGATCTTTAACCCAAGCCAAGAATTCTATACAACAAATA

AGACCACAAAATCACCTGTTATGGTAACCTTGTTGTGCTGTAATGTAAAATTTTCTACTC

GTTTTTTGTACTTTGTCAAAGAAGAACCATGTCTAACCGACAAGCTAGTTCCTCGTCCTT

TCCTCGGAGGAGCTGCCATGATCCTGTACAATTTTGTCAATATGAAGTATCACGCCCCTA

TATTTCTGTTCATATGGTGCTATTCTGATCTTGTAAACCTTCTGCAATCAGAGATGCTCT

TTGCCTCTGTTAATTTATGCAAATGTTTGTC

**>TaMPK21-1(1BL): TRIAE_CS42_1BL_TGACv1_032784_AA0134120.1**

CTCCCTCATCCGCTGGCTGCGCCACCACCGCTCCCGCCGCGTCGCCTCCGCCTCATCCTC

GTCCTCCTCCCACCTGACCGCGCGCACCAATACATCCTCCGCGACCACCGCCACCAGCGA

CCCCCGCGCCCGCTCGCTCCCGCAGCCGCAGGACGACGAGGCCGACTGGGAGCAGGAGGA

GCAGGAGCAGGAGGTCGCCGACGGCCCCGAGCCCGGCCCCGAGGGATACATTGTGCTCGA

GCGGGAGGGGGAGGCCGGGAGCCTGCGCGTCGCCCTACCCCGCGCGCCCGCGCGCACCAA

CCCGCCGCCACGCATGGATCCCGGCAAGAAG

gtgcgtccttgcttgcctcgccgccgtgcccccctcttctccttaattattagtcccctt

cccccgctacctccttccgccgtccccgctcaatgtcggttgccatcgatctctctcggc

tgaaatatgtcgccgtgctgccgggattgctctgctcgcttcatggatcggggaagatcc

aatccacccttgggattgctctgttcttgctccctcgtcagttcttgcggatctgtttgc

ctggaatctccgatttttcagggtcaatgcggcatccacatgccaagatagttgttaact

aatgtcataataaactcgtaatagtattagcttgcgccacacatgaatgccgtccgtgac

agcctaataaagcagggcaaacagcgtcacgggagccctgttttcgctgttcatgtctca

tcagaggcaaacatgatgcatggtggaagaggttacgcttttgcttttcaattattggtc

ctttgtggacgattccgcgtttgccacttttggtagcagcaggtagatatattcagaaaa

atacactgtttaaagtaccaaatgttcaaccacttcctgattcattcagtccaaagtcca

aacccagcacaagcaagaagctgtctcgagtgactttccagttgtgtgcatgtcttccga

tccttttcgcccaccactatcattctttgtaaggccatcagtgttttttacggaaagggg

attaccccggcctctgcatcacaaggatgcacacaggacacagccgtgtaaggccatcaa

tattgtttcgtatctttgtgaggccgtcggtatcttcaattgagatgttgtatctttctt

ttcctcggcgcgtttttaccaccactaataaatcagtatgttagcaccatctagcgctct

gaactaatggtatgaatgtgagcttgctccttccttttcag

ACCTTGGAATCCGAGTTCTTCACGGAGTATGGTGAACTAAACCGGTATCAGGTCAGCGAG

GTCATTGGCAAAGGGAGTTATGGAGTTGTGGCTGCTGCTATCGACACCCAGACCGGCGAG

CGCGTGGCCATCAAGAAGATCAATGACGTCTTTGATCATGTCTCCGACGCCACCCGCATC

CTTAGGGAGATCAAGTTGCTCCGGTTGCTGCGCCACCCGGACATAGTTCAGATCAAGCAC

ATTATGCTCCCCCCTTCAAGGAGGGAATTCAGGGACATATATGTGGTCTTTGAGCTGATG

GAGTCCGATCTCCATCAGGTAATAAAAGCGAACGATGATCTCACACCAGAGCATCACCAG

TTCTTCTTGTATCAGCTGCTCCGGGGAATGAAGTACATCCATGCAG

gtgactagactccacaaaggaaatcgataatggttcgttcgaccttttttcatctcatgt

tgttgtattgattgtttgtttacatgatggctttgcag

CGAGTGTTTTCCATCGGGATCTTAAGCCCAAGAATATTCTAGCGAATGCTGACTGCAAGC

TCAAGATTTGTGATTTTGGGCTTGCCCGTGTATCATTTAATGACGGGGCTCCATCAGCCA

TATTCTGGACG

gtagccattttagtaatattgagcagatagtgtttatcctgagcggccaaatcttggttt

ctgatgattttgcatgccttgtctag

GACTATGTTGCAACTAGATGGTATCGTGCTCCAGAATTGTGTGGCTCTTTTTTCTCAAAG

gtatgtaattttcatcttaacttcatattaccaacaacagtatccaggctattaaaaaaa

gcacaatgaccatactacagattttcttccaaaattgttgttttaatatgatatgtttcg

atgcaacatagcaatactatttgttgattattttccatgtttgcaactaaaattcccttc

ccttttagagagagttgctactacagttctcaaatgttggttcaacctgtcatgatctgt

atttcatatgttgcactaagttctctcccagtaaaagtgccaaaaaattgttacttatgt

tgttttgccctcttctttcatatagttgtagctgaatgtagcgacgtggcctgggcgcta

attcgaacttgatgatgtacttggttcttaattaatgtctgtgcatgtttagcaaatgct

aaaagcagctgaactttgaattttgcag

TACACTCCTGCAATTGATATTTGGAGCGTAGGGTGTATCTTTGCAGAAATGCTCACAGGG

AAGCCACTCTTTCCAGGGAAGAATGTTGTCCATCAATTGGATCTCATGACTGATGTACTT

GGCACTCCTTCAGCAGAATCTCTCGCTAAG

gtatgcttactccacactagtcaccagcaagttagaacagcagttggagttatatatttt

gccacttccttttttcatattttcataatatgcgaatttcctgcaacattgaaccttgca

ttgataacttgtgtacatggttctgaatttattattggcttattccatttctctcagtat

cttttttgcatccacgcccaatatttacgaactctgattttccttaatatggttgcgtgc

ttggttactgagctacatggactacggatgtcggcacgaattggcatcaatatcggcaca

atctcttgccatgttatatatttttcatatagaatgaaatgtaaattgtgggatgatgtt

taaaccatgtgtctttcacacccttttaccgaatggttgcag

ATACGGAATGAGAAAGCTCGGCGATACTTGAGCAATATGAGGAAGAAGCCTAAAGTTCCC

CTTACCAAAAAATTTCCAGGCATCGATCCTATGGCTCTCCATTTGCTTGAGCGTCTTCTT

GCTTTTGATCCTAAGGATAGGCCAACTGCTGACGAG

gtacgaatggctgataccaacttctatttactataaagatatattgcatcattgaaaatg

ttgggtaaatcactgtcctcaatgctgagatgtgaaaatgttcttcaag

GCCCTGACAGACCCATACTTTACCGGATTAGCAAATTCAGAACGTGAACCCATAGCACAG

CCCATCTCAAAACTTGAGTTTGAGTTCGAGAAGAGAAAGTTGGGCAAAGATGATGTCCGA

GAATTAATTTACAGAGAG

gtatttgctcagctattttgtggagatacagtagagctgctgctcgactctgtagttagc

taacaacttctacttctttcag

ATTTTAGAGTACCATCCTCAGATGTTGCAAGAATACCTACGTGGAGGAGACCAGATGAGC

TTCATGTACCCTAG

gtgggaccattatttttccttagacacaagatttacttgtgttgagaattttatgatgac

tatgctttactctcttctgcatacactgaattatgctagttatatgtatgattggttgat

ggtatattgcaaactctgatgtttgtgcatgtatgttgacag

TGGGGTTGATCGCTTTAAGCAGCAATTCGCTCATTTGGAAAAAGGTGGTGCAAAGGGTGA

AAAAGCCAGTCCACAGTTGCGACAAAATGCCTCCTTACCAAG

gtaatatgctgtgaccaagacttaaatctatacttctaaggcaatacatatctttatgta

gaatgggcatgtggagacttatggacgaaattatttgttttctaaccaaaatttctttcc

tttgacaaccttgtacaattttcctaaagatgatctgtttggacttggcaagagtattaa

acaaataaagacttcatcataagtaccatatttgttttgctttttgcaagttgccagaga

ttttaaatcaaatatttttaaatgttaaatttgatagagaataatgtttcacatgatcag

atgtttatatatatatatatatatatatatatatatatatatatatatatatatatatat

atatataatgagtcatcctacaaatagacaaccttttaagtttttgtatctaacaattct

cgcatcttgtctcag

GGAAAGAGCAATTGGTAATAAGCACGGAGATAGTGAGTACCAAGTAAAGCTGAATGCAGG

TGAGAAGCCAGTACATGCATCAGTGACAGATGGAATAAGCAAACCCCTCATGAGCGCTCG

GAGCTTACTGAAGAGTGAAACCATGAGTGCTTCCAAGTGTATAGGTGAAGTAAAAAATAA

AGAT

gtgagttcagctcatcctttgttactacagtaaatatatttgcccctcttttcttatgaa

aattgctcttttttttcctttggagtgtttaattgtttatatctctaagacatctctacc

taatttgagcaatttcttagttaatgctgcaatgtccattcaacattttccag

GATGAATACGAGAGCGTGGATGCAGCTGACGGCGTCTCTCAGAAGGTCGCTCAACTGAAA

ACCTGATTTTCGAAGGTTACAGAAGCGATCGGTAGTATATACTATGTACAACAGCATGTC

TATAGGCGCTCAAGATCAGAATGATCTTTAACCGAAGCCAAGAATTCTATACAACAAATA

AGACCACAAAATCATCTGTTAT

**>TaMPK21-1(1DL): TRIAE_CS42_1DL_TGACv1_061695_AA0201870.1**

ACCAATGTTCAACCACTTCGTGATTCATTCAGTCCAAACCCAGCACAAGCAAGAAGCTGT

CCTGAGTGACTTTCCAGTTGTGTGCATGTCTTCTGATCCTTTTCGCCCACCACTATCATT

GTTTGTAAGGCCATCGGTATTTTTTTCGGAAAGGGGAATACCCGGGCCTCTGCATCACAA

GGATGCACACGCACAGCCAT

gtaaggccatcaatattgtttcatatctttgtgaggccatcggtatcttcaattgagatg

ttctatctttctttttctcggcgcgtttttaccgccactcgtaaatcagtatgctagcac

catctagcactctgaactaatggtatgaatgtgcgcttgctccttccttttcag

ACCTCGGAATCCGAGTTCTTCACGGAGTATGGTGAATTAAACCGGTATCAGGTCAGCGAG

GTCATTGGCAAAGGGAGTTATGGTGTTGTGGCTGCTGCTATCGACACCCAGACCGGCGAG

CGTGTGGCCATCAAGAAGATCAATGACGTCTTTGATCATGTCTCCGATGCCACCCGCATC

CTTAGGGAGATCAAGTTGCTCCGGTTGCTGCGTCACCCGGACATAGTTCAGATCAAGCAC

ATTATGCTCCCCCCTTCAAGGAGGGAATTCAGGGACATATATGTGGTCTTTGAGCTGATG

GAGTCCGATCTCCATCAGGTAATAAAAGCGAACGATGATCTCACACCAGAGCATCACCAG

TTCTTCTTGTATCAGCTGCTCCGGGGAATGAAGTACATCCATGCAG

gtgattagactccacaaaaggaaatcgataatggttcattcgacctttttcatctcatgt

tgctgtactgattgtttgtttacatgatggttttgcag

CGAGTGTTTTCCATCGGGATCTTAAGCCCAAGAATATTCTAGCGAATGCTGACTGCAAGC

TGAAGATTTGTGATTTTGGGCTTGCCCGTGTATCATTTAATGACGGGGCTCCATCAGCCA

TATTCTGGACG

gtagccattttagtaatattgagcagatactgtttatcctgagcagccaaatcttggttt

ctgatgattttgcatgccttgtctag

GACTATGTTGCAACTAGATGGTATCGTGCTCCAGAATTGTGTGGCTCTTTTTTCTCAAAG

gtgatgtaattttcatcctaacttcatattaccaacaacaatacccaggctattgaaaaa

gcacaatgaccatactatagattttttcttccataattgttgttttaataatgatatgtt

tctatgcaacatagcaatactatctgttgattaatttccatgttctcaactaaaattccc

ttcccttttagagagagttgctactacagttctcaaatgtcggttcaacctgtcatgatc

tgtatttcatatgttgcactaagttctcttccagtaaaaatgccaaaaaattgttactta

tggtgttttgccctcttctttcatataatcgtagctgaatgtagcgacgcggcttgggcg

ctaattcaaacttgatgatgtacttggttcttaattaatgtctgtgcatgtttagcaaat

gctaaaagcagctgaactttgaattttgcag

TACACTCCTGCAATTGATATTTGGAGCGTAGGGTGTATCTTTGCAGAAATGCTCATAGGG

AAGCCACTCTTTCCAGGGAAGAATGTTGTCCATCAATTGGATCTCATGACTGATGTACTT

GGCACTCCTTCAGCAGAATCTCTCGCTAAG

gtatgcttactccacactagtcaccagcaagttagaacagcagttggagttatatgtttt

gccacttcctatttttcatattttcataatatgtgaatttcctgcaaaattgaaccttgc

attgatagcttgtgtacatggttatgaatttattattggcttattccatttctctcagta

tcttttttgcatccacgcccaatatttacaaactctgattttccttaatatggttgcgtg

cttggttactgagctacatggactacggatgtcggcacaaattggcatcaatatcagcac

aatctcttgcatgttatatatttttcatatagaatgaaatgtgaattgtgggatgatgtt

taaaccatatgtcttttacactcttttaccgaatggttgcag

ATACGGAATGAGAAAGCTCGGCGATACTTGAGCAATATGAGGAAGAAGCCTAAAGTTCCC

CTTACCAAAAAATTTCCAGGCATCGATCCTATGGCTCTCCATTTGCTTGAGCGTCTTCTT

GCTTTTGATCCTAAGGATAGGCCAACTGCTGACGAG

gtacgaatggctgagaccaacttctatttactataaagatatattgcatcattgaaaatg

ttgggtaaatcactgtcctcaatactgagatatgaaaattttcttcaag

GCCCTGACAGACCCATACTTTACCGGATTAGCAAATTCAGAACGTGAACCCATAGCACAG

CCCATCTCAAAACTTGAGTTTGAGTTCGAGAAGAGAAAGTTGGGCAAAGATGATGTCCGA

GAATTAATTTACAGAGAG

gtattattgctcagctattttgtggagatacagtagagctgctgctcgactctgtagtta

gctaacaacttctacttctttcag

ATTTTGGAGTACCATCCTCAGATGTTGCAAGAATACCTACGTGGAGGAGACCAGATGAGC

TTCATGTACCCTAG

gtgagaccattatttttccttagaaacaagatttacttgtgttgagaattttatgatgac

tatgctttactctcttctgcatacactgaattatgtatgattggttgatggtatattgca

aactctgatgtttgtgcatgtatggtgacag

TGGGGTGGATCGCTTCAAGCAGCAATTCGCTCATTTGGAAAAAGGTGGTGCGAAGGGTGA

AAAATCCAGTCCACAGTTGCGACAAAATGCTTCCTTACCAAG

gtaatatgctgtgaccgagacttaaatgtatacttccaaggcattacatttctttatgta

gaatgggcatgtggatacttatggacgaaattgtttgttttctaaccaaaatttctttcc

tttgacaaccttgtacaattttcctaaagacgatctgtttggacttggcaagagtattca

acaaattaaagacttcatcataagtatcatatttgttttgctttttgcaagttgccagag

attttaaatcaaatatttttgaatgttaaatttgatagagaataatgtttcacatgatga

gatgtttatatatataaaaaaagacaaccttttaagtttttgtatctaacaattctcgca

tcttgtctcag

GGAAAGAGCAATTGGCAATAAGCACGGAGATAGTGACTACCAAGTAAAGCTGAATACAGG

TGAAAAGCCAGTACATGCATCAGTGACAGATGGAATAAGCAAACCCCTCATGAGTGCTCG

GAGCTTACTGAAGAGTGAAACCATGAGTGCTTCCAAGTGTATAGGTGAAATAAAAAATAA

AGAT

gtgagttcagctcatcctttgttactacagtaaatatatttgctcctcttttcttacgaa

attgccggtatatttgagttccatacttgcaggctgtctttcacctttttttttttcctt

tggagtgtttaattgtttatatctctaagacatctctatctaatttgagcaatttcttag

ttaatgctgcaatgtccattcaacattttccag

GATGAATACGAGAGCGTGGATGCAGCTGACGGCGTCTCTCAGAAGGTCGCTCAACTGAAA

ACCTGATTTTCGAAGGTTACAGAAGCGATCGGTAGTATATACTATGTACAACAGCATGTC

TATAGGCGCTCAAGATCAGAATGATCTTTAACCGAAGCCAAGAATTCTATACAACAAATA

AGACCACAAAATCATCTGTTATGGTAACCTTGTTGTGCTGTAATGTAAAATTTTCTACTC

GTTTTTTGTACTTTGACAAAGAAGAACCATGTCTAACCGACAAGCTAGTTCCCCGTCCTT

TCCTCGGAGGAGCTGCCACGACCCTGTACAATTTTGTCAATATGAAGTACCATGCCCCTA

TATTTCTG

**>TaMPK21-2(3AL): TRIAE_CS42_3AL_TGACv1_196288_AA0659130.2**

ATATCCCTGGCCGCGCTCCGCGCTACCGCGGCGATCGGGCGTCCACTGCCCAGTCGCGCA

CCTTCCCTCGCCGTACGAATATGGCCCCTGTTCCCGCTCCTATCGCGCGCCTCCCCCTCC

TCCCTCGCCGTGTCCACACCCCCCTCCCTCATCCCCGACCGTCAGCACCGGCGGAGGCAC

GACACACCGCGCGCACGAGGGCGCCGCAGCGGATCGGAGGAGTGGATCCGGGGCGAAGGG

AGACGGCGCGGGGTAGNCGTGTCCACACCCCCCTCCCTCATCCCCGACCGTCAGCACCGG

CGGAGGCACGACACACCGCGCGCACGAGGGCGCCGCAGCGGATCGGAGGAGTGGATCCGG

GGCGAAGGGAGACGGCGCGGGGTAGCAGTACATCACTCCGGAGCACGGGGCGACTGGCGC

GGCGGGCGCCATGGCTAAAAAG

gcgcgcatgtgaaccaccctagttgacattttgtgcctcgcctgatgcgtatccgattgc

gggattgactcatacgcttccag

GGCTCGGATAAGGCGGGGTTCTTCACGGAGTACGGCGAGGCGACCCGGTACGAGATCGGC

GAAGTGGTCGGCAAAGGCAGCTACGGCGTCGTGGCGGCTGCCGTCGACACCCACACCGGC

GAGCGCGTCGCCATCAAAAAGATCGACGACGTCTTCGAGCATGTCGCCGACGCCACCCGC

ATTCTCCGCGAGATCAAGCTTCTCCGCCTTCTGCGCCACCCAGACATCGTCGAGATCAAG

CATATCATGCTCCCGCCGTCGCGCCGCGAGTTTCGTGACATCTACATTATCTTCGAGCTC

ATGGAGTCCGACCTCCACCAGGTCATCAAGGCCAACGACGACCTCAGCCCGGAGCACTTT

CAGTTCTTTTTCTACCAGCTGCTTCGCGGGATGAAGTACATGCACGCTGCCAATGTCTTC

CACCGGGACCTCAAGCCCAAGAACATTCTAGCCAATGCCGATTGCAAGCTCAAGATTTGC

GACTTCGGCCTTTCTCGGGTTTCCTTCAATGACACCCCGTCCGCGATATTCTGGACG

gtaggtagtttatactctaataaaagcttaccacagattatgtgtattatgtaagcagcc

aaccaaaatttagacttttatgctcgcgtggccatctgtcccag

GATTATGTAGCAACAAGGTGGTATCGTGCTCCAGAATTATGTGGCTCCTTCTTTTCAAAG

gtgagaaaacttcatctcgacaaaatttgaccttaaccaaattagagaccgtgaatccgt

gataattcatatgtgagcatcaatatcctcgttgatgttgtcgagtttgtttataaatcc

gagttttcttgtacacttgattattggcagtcaatgcacatgttaccacattgtataatt

cgggtttcactatactgaagttagtatttccttttacag

TATACTCCTGCAATTGATATTTGGAGCATAGGATGTATATTTGCCGAAATGCTTACAGGG

AGGCCGCTCTTTCCTGGCAAAAACGTGGTACATCAATTGGATCTCATGACTGATCTACTT

GGCACTCCTTCAGAAGAATCTATTTCCAGG

gtgagttcacttccctgcttctaaaaatgataaaaacagttaagagacctacctccaatg

tgattgccttaccaactctttgtgatggtggttatcatctccgttatcacttggatctcc

gttattacagcatggaactatctatctgaagttcaaactactgaaatattactgctataa

ctggccctgctgttatatacatactgctaagactaattcttgcattatgtcctaaaagta

agggactgcctagaacgcaaacgcctgattattttcataggggcagttgattttgctgaa

ccactggatttagatccaacagctcgctctacttcttcaacctccggacacctgtcttct

ttctcacctttatcctcaccctaaaccctctgccaaggtatcccctgcgtgtcgacaact

tccctgcctcgcggtccaccacctgtattctcttcaccttcggcttcggtttccaacgtc

ttccatgacacgcacaaccctaaccccctaaccactttggcaacgtctcctgcctcggct

ccacagctgtcgccactcctgtgtggccggtgttgaataaataggcaatttttcgattaa

tttaatccaagaataaatcactagcatggcattgacagaaataaccacataccgatattc

aatcatgcaagcacatactacgctaattgcagacagatctatgtataacgctagcatggc

taaaacagaaaacaataggagcgagataaacgagttataccctccaataggctaggaagc

tgcggcggcagcggtggcagcattgacgtcctcggcggccttcttgtcggcttcgatctt

ctcagcggcggcacgtttagcgtcggtggacatggtgatgacgagggcgacgcgaacata

gaggaagtagacgaacagaggcgagcagtcgcgtagtcgcttcccaaaaacctaatcgcc

cctctcccgtacaggatccggagaggcggggtttcagagacctgctctcccgtcaaccat

gtacgcggtggacgggatggagtcgccggcggcagcagcagcaaaggaacgacagtgggc

atgaaggcagagatgcgatatgtttcacgtgacggctggttgggagacgcctcacacata

tataggcgcaaccgcgtggagagacgtgggcttgacccatgtccgagtccgtgacagccc

acgattcgacgtctcagatcgtggcccagctgtcaaaaactctccgttactgacaggcaa

aaataagggcataggtgtgagctcggctcggctcattcccgcaacccgcggcacgtcgtg

acaaggcgtggcgtggcgaggcgagcggcagaggaggagtgcgcgagggcctcttctatt

ctcaagctccaatagcatgtggaagagaatcccttataaagaggtccaactccttctcca

ctagcagggtgggactaaacttcccactacacctagtgccatataacccacatggaccct

tagagattttttgaaattgttagatgagcctaaagcccactccatatttcaacaattccc

caccagatctcgagggcccattgtgtcctctgttccaattgctgtttcgatataccagtg

tttcagtgaaggcacgttaaggttgaacttcacctagagcaagcagctacactccttcac

aactaaacaatggactatgccttgaattgtcagtttggtgtaaagaagtttcccacatgt

cttactagtactaggctgccgaaggctgacccctcagggtggagcatataagtcacactc

ctagcctattcataagtttactagagatcaccccaatctcatagactgtgaccaacagtc

aggctcatataggtgtgttcctccaaagatcgctttgtaggatagcatcttgcttacata

agctttggaacacattaacacagtagccatcctaccatacagtatccgaaagtattgcat

ctccaatggagtgggttagtaaagttactctcctcagtttaccattggcttgttttctca

ggtcctacttcacgggatctccgatcacataggttgggttaccaccatggcaactcacgt

gggtctcatacccatctccctcgatgcattatctatcacaacacgtgatagtccttttgt

aaagggatctgccagattcttagccgtttggatataatccaacgctatcactctggagtt

tcttaattttttgacagcttttaatctcatttttatatgtttgttggacttcatgttgtc

ctttgaactgttcaccttggtgataacagtttgattgtcacagttcataaggatagccgg

aaccggcttctcaaccaatggcaagtccatcaaaagatctcgaagcgatcctgcttcgac

accagacgtgtctaacgctgttaattctgcttccattgtcgatcttgttaaaatcgtttg

cttgcaagacttccaggaaacaacgccacctccaagagtaaacatatacccggttgcggc

cttcatctcatcagcatcagagatccaattcacatcactatacccttcaagtaccgacgg

gtatccggtatagtgaagtccatagttcatagtacctttcagatagcgcataactctttc

aacagcatgccaatgtacatcacctgttttggaaacaaatcggctcagtttgctcacagc

aaatgcgatgtcaggcctcgttgcgctcgctaggtacatgagtgaaccaatgatttaaga

gtatctcaattgacctttagccgtgccttcgaactttcgaatcaacacactaggatcata

tggtgtttgagatggtttgcagtccgaatatccaaaacggctcaacaccttctcaacaca

atgggattgcagaagtgtaatcccaccctcattatatctctgagtagcttgatgttcaag

ataacatcagccacaccaaggtctttcatctcaaagttctgagatagaaacgacttgacc

tcctcaatgactttgaggttggttccgaatatcagtatgtcatcaacatacaagcacagt

ataactccttcgcccccaccatggctatagtatacacatttgtcagcttcattcacaata

aagccaacagatgtcagtattgtattaaacttgtcatgccactgcttaggtgcttgtttc

aggccatataaagatttcagcaacctacacacttttccttcctgaccatctatcacaaag

ccatttggctgttgcatgtagatttcctcgtctagctctccattcaggaaagtcgtctta

acatccatctggtggacgagaagaccatgcgaggccgccaacgagagtagtactcgaatg

gtggtcagtctggccacaggtgaataagtatcaaagaaatcttcctcttctttctagtca

tagcccttggccacaagcctagccttgtacttttcaattgtatcatcgggcctaagcttc

tttttgaacacccacttacatcccaatggtttgcaaccatagggacgttcagtaatctcc

catgtcccgttagccatgatggaatccatctcgctacggaccgcattcttctagtagtcc

gcttctagagaggcatacgcttctgaaatagaagtgggagtatcatccacgaggtacaca

aagaaatcatcaccaaaggtctttgcagtcctttgtctcttatccctaccaagggtttcc

tcgtcatcctcatgattatcatcatgtgtttgttcataatattccataggaatggcaggc

tcaggagtctcctcagattcctgtctagaagtgctttgcatatctcataggaaaaatatc

ctcgaagaatgtagcaccttagactccataattgtactgaccttttggtcacgtacctca

gatttcactactagaaatctatagccagcgctattcttagtgtagcccaaattaacgcaa

tccatagcctttgttccaagcttatgctttttggggatcggcacattgactttcgccaaa

cagccccaagtacgcaagtacaagagtgttgtccttctcttcgcccatttctcataggga

gtgatctcattatcctttgttggaactttattcaggacatgacatgccgtcaatatagcc

tccccccaccatgccttggataaacccgatgtatctaacatggcgttaaccaaatcagtt

atagtacggtttttccgctcggcaacccccgtttgactgggtaaatagggaggcgtcctc

tcatgaataatgtcatgttccgcacaaaaagaatcaaacttactcgagaagtactctcca

ccaggatctgaccggactcgtttaattttcttttcaagttgattctcaacttctgcctta

tagattttataggagtgtagagcctcatctttagtatttaacagatacacatagcaatat

ctagtggaatcatctatcaatgtcatgaagtatttctttccacctttagtcaacacacca

ttcatctcacaaagatcagaatgtatgagttctaatggtgccaagtgtctctccttcgca

gctttgtgaggcttgcaaggttgcttagcttgcacacatgaaaggcacttagaacctttg

gctaaaatgaaacttgggattaaattcaacttagctagccgcgtcataacaccgaaacta

atgtgacaaagacgtgaatgccaaactttagattcattaacactcaatgaatatggttca

tgactttattacaaaaatctgcgaggaaaagacggaacatccctctgctctcataacctt

ttccaataaagagtccatattttgtaacaactaatttattagactcgaagaccaatttaa

acccttctctacatagaagggagccactaacgaggttcttcttgatggcggggacatgtt

gcacgttcttcagctgcacgatccttcctgaagtaaacttcagatcgaccgtgccaacac

catgaacagaagcactcgcgccattccccatcaatacagacccgtggcctgtgacccggt

aagaagaaaataatgaaatgtcagcacacacatgaacacctgcacctgtatccacccacc

aatcgatggactgaacactgaaaaaacagtaaataaattaccatacccagatgcaccatt

ctcattgttgcccacaatcatgttgacagacttggagttctgccctggcttcttatactt

gtttgggcacttgttggcccaatgttcaaccgaaccacaagtgaaacaaccctcatcctt

cttgttcttcttgaaggtcgtcttacccttcttcttaaagtcggtattctgttggacacc

gttctttcccttgggcttgtgggaattgaagttcctctggtgcaccatgttggcgacaga

agttccttcggtcccttttccgtgcgagtcctttgcccttgaattttgctcaacattcag

atggccgatgacatcctctaccgagaattcacgcctctgatgtttcagagtggtagcaaa

gtttctccacgaattagggagcttggcgattatacatcccgcgacaaatttgcccggtaa

atcgcacttaagaagctcaagctccttaacgatgcatattatctcatgagcctgctccaa

tacaggacagttctcaacgatcttgtaatcatggaactgctgtataatatacatctcgct

cccaacatcggtggccccgaatttagatttgagcgcctcccacaagtccttggcaacatg

cacatgcaaatatgcgtcaaccaacttatcaccgatcacgctaagaactgctccgagaaa

tacgacggtggcctccctaaacgccttctcctgttcaggagcaatcgttcctgtggaaga

ggcaccggtgacccagaacacgttcttagccgtgagccataaagtggtcttagtctgcca

acgcttaaagtacgtgccggtaaacttatccggtttcagtgcagcggcaaagccacttgc

cgaaaaattcctacacacattaggtttttggattgttcaataaataggcaattttccgat

taatttaatccaaaaatgaatcactagcatggcattgacagaaataaccacatactattc

aatcgtgcaagctcatactacgctaattgcagacagatctatatataatgctagcatggc

taaaacagaaaacaataggagcgggataaacgagttataccctccagtaggccaggaagc

cgcgacggcggcggtgtggcagcagcggcgtcctcggcggccttcttgtcggcttcgatc

ttcttggcggcggcacgttcagcgtcggtggacatggtgatgacgagggcgacacgaacg

tagaggaagtagacgaacagaggcgagcagtcgcttcccaaaaacctaattgcccctctc

ccgtacaggatccagagaggcggagtttcggagacctgctctcccgtcgaccgtgtacgc

ggcagacggggtggagtcgccggcgggagcagccaaggaacgacagtgggcgtggaggca

gagatgcgatctgtttcacgtgacggctggttgggagacgcctcacacatatataggcgc

agccgcgtggcgtccgcgtggagagttgaggcgaggcgggcggcggcgaaggagtgcgcg

agggcctcttctattctcaagctccaatagcatgtggaagagaatcccttataaagaggt

ccaactccttctccactagcggggtgggactaaacttcccactacacctagtgccatata

atccacttgggcccttagagattttctgaaattgttagatgggcctaaagcccaccccat

atttcaacagccggtggctctgcatctgcattttaaataactacccgacagcaaaagggt

aaaaattcatgctttgtttaatttgtgcttatcataagaaacttcagagtgatgttttgg

ttgaataattcactacctggacaggcatctggtcttaagacagtcccacactcccagtga

tcatatatttgcttgatttgtataccttgtaatccctaattctggaaagggcaaacgagg

tatcgaaaaaaatatggacgataattgatatctaacaatgatgctttacaaacaactaaa

cttgccccatctatggctgagggagttatcttttgaacattattcttacagaaaataact

gtggttgtaaaccttttttttgcatggaaggcattcagagtgatgttttggtcaaataat

tcactacctgaataggcatgttgtctcagaaagccccgcactcacattgaccatatattt

gcttgatctgtgtagtttgtaatccaatttctggaaagggccaacacgatatctggtcac

cattgtgctaaaaaaatatgaacgccaattgatatttaacaatgattcatgacaagcaat

gagcttgctctatctaaggataagggagttatcttttgaacattattcttatggaaatag

ctgtggatgtaaacattttttctggacaaaaggctttcaccctcctttatagatacagcc

acaaatggctatgagtacagaagttccacaaatactcatgattccttaaagaagttgcat

agcacaattacaacacaaacacaaacactcgcacacaaaacaccccagccactcccgaga

tagcaaaaagggcgacacgtgaagtttcctccatgtcctcctttgatgatggcatcacga

gctgcaaacaaaaaatgatcattttgaacaaacaattagatggatgtttgggtgcagcta

tggcctttgggggcaaccattgggagatgataacacgggattcattcaagcagttttgca

tggcggtacagtaataggttatgtgtctgagtgatgtaatcctgttattggtcagttgtg

attgggccttgctttcacttgttaaacttttttggatgttggtcacggtactttaattaa

gatggttgtgcatcataggttgcagattccggggttttaaccttcttttcgaagggaaag

atggatgtttggggataacccactccatagtgagtttgttgcgtgtgtcctagagtgcac

aattctgagctgcaaaggcgacccaaatgtccctagaaatgcaacttgaaaccccctaga

tatgattgagaagcttgcacgaagcacctaaggttccagtttgcgtcttcaaattcccta

gagtgcacaggtcccattggacaggccgtggcgcttgagaacctggtcactcaaaggcaa

gcagccatgggtcaactgccaaaggaagatttgtggcctaggttttggttaatgcttcag

tcttttggttattgtactatgatttttgtgatcatctttactttgatattttttccaaat

atattaaaacaaagtaacaacacagatagtaaattatatttgttcgaatatccatttctg

aatctctccttttcaatgaaaagaaacgcaaagcttttgcgttttctcgaaaaatatcca

tttctgagcccgggctcatctgcacccggttaagagaaaattcaaaacaaatactaaaat

attttttgcatggaagatgatttggtgcgtgagctccactccaaatttcaggtcatttgg

acatatgagtagctctcagcaaaaaagacaaattgggtcaaaacagtacatgaacagtac

tctgtttcacagaccccaaatttgtttttttttttgccaagagatactcagatgtccaaa

tgagttgaactttggagtgaacctcacgcacctatgcatcttccattccaaaaaaatcgg

attttttttgaaaattttctagtattttttttaatttactgttcaccatgggagtagatg

agcccgggagccaaatagccgcactcttgtttgttctgctaagtatttttttaggtgata

aatcacgttcagaaatgattcttaaactctgcatctgttgcctatttgtactgcatatag

GTTCGAAACGAAAAAGCTCGACGATATTTGGGAAACATGAAAAAAAAGCATCCAATACCT

TTTTCTCAGAAGTTTCCTGGTGTAGACCCCATGGCACTCCATTTGCTTGAGCGTCTTCTT

GCTTTTGACCCCGCAGATCGGCCAACTGCTGCAGAG

gtaggaaatataaacaacaagaatcttagctcaaaaagcttgtttcttttgttattctcg

atactgatatgatatcaatgtaattcaag

GCCTTGGCAGACCCATACTTTACTGGATTGGCAAATTCTGAACTCGAACCCACAGCGCAA

CCCATCTCGAAACTTGAGTTTGAGTTTGAGAGAAGGAAGCTGGCCAGAGAGGATTTACGT

GAATTAATTTACAGAGAG

gtaatgttagtcaattgtacggtgggcattgtggtagtagtctttactaatgcatgaagt

atctctag

ATTTTAGAGTACCATCCTCAGATGTTGCATGACTATCTTCGTGGTGGAGATCAGGCAAAC

TTTCTTTACCCAAG

gtgagtttggaaaaaatgctttaatccaccatttcttgttacaagaagaataatatactc

cctccgttctaaaatagatgacccaattttatagtaactttgtactaaagttagtacaaa

gttgagtcatctattttggaacggagggagtacatgtgttatctatgtaatgttttgttc

agttagtaaagtttgcttttgacaatgataggcatatgtttcagtaggtgttattcgttg

aacttttgcatggtacaacttgagtggaggtgagacagaaaagcataccagtgtacatta

tgatcctatgaatgtaacgagcaatgtgtagcttaaaatattatttttataatcattgaa

tatcattatcaagcaactgcatatgtggaagctgtttaacataatcagttgcgatgcgtt

aatgacgtttttcttgtgttaaacaattgtccctttggattttgatgtacaatcttttca

ttgtaaaaatatgcttttaatttccctaaataagaattcatgctattgtaaacctcattc

aaatatggcatactaaagaaacaccatttctttaaccacagttatgattggttaagacat

tgccagtatactaactgatatattattgccag

TGGGGTGGATCGTTTCAAGAGCCAATTTGTTCATCTTGAAGAAATCGGTGCTAAGGGTGA

AAAGACTAGCCCGCAGCTGCGGCAGCATGCTTCCTTACCAAG

gcaataccttcagcttagtaaactataatgcttctcttcagtaaaaatatgtgcatgtct

cttgaactaatatatcctccatcttatctcag

GGAAAGAGTTATCGGCAGCAGTGATGAGCCTGAAAAGCCAAATGCAGACTACTGTATAAA

ATTGCATGTAGATGAGCTACCAGGTCACACATCGGTGACGGATGGCCTTAACAAGCCACT

GTTGAATACTCGAAACTTCTTGAAGAGCGAAAGCATCGGTGCCTCCAAGTGCATCGTTGT

CAAAGAAAAGCAAGAAAAAGAT

gtaagtgccgcgacagcattacctccctaaaactattaacaaaaccaaatatgttgagtt

gacctttttttatattaataaccaaactggatcaatgttgaaagttggccaaaaattata

ggaaatgttaaaagcccgacaccagatttttagcgctaaaaatctgacgatcagatttgc

catgcgaaaacagataaaattgccatgctttagtataaggagatttgccattgttctaca

gtgcaaattgccatgtgtagcataaaaagccgcagaaaaattgccatggtgcaacgattg

attgccatgtgttcgatcgggatccgacgtacttgaggacgtcagatttttagtactata

aatctaacgtcagagttatattgagaaaattcttgtctagagttaaagactcgattgtct

aatgcaaatgcgatatagaacacgatgcttttaggtgaagagttaaaggctcgattgtct

accactccctcgaactaaaaccacgacaaggattatggaacggggggagtagttcctaaa

taacatgggtatggtaaaagctgtgtgatgtgcgattcctatttctgaaatcaggcaaca

ttgagtcaccgagtacggaatagttccagtgtcaatgtaggtggttgagaaactgatata

aaaaatgcttgagtctattaaagcttaaataaagctcattatgtatagataagctgaact

tctgtgcatgttggttggatttctttttctcagaccaaactgtcgtggagggcagacagt

atacctctctacaaggggtccaattgtaccctagccgctttcagaacatgccttataagt

ccttggaatccagtgattcattgagtgaacacattgaagttccacgctacgagttgattc

tctgatgtaatggcccctctaacttggataacttgctatcaatcaaattatgtgtgctcc

tttgtttttgcag

GAGGAATCTATGTCTGAGTATATGCATGAAGCATCTGATTAGGAACCGAAAAGGATTGCT

CAACTCAAAAGTTTGTGATGGCACAGGGAAGACAGAAGCACGTCCGATCCATGGGAGCTT

CAATTCAGAATCCCGAGCGCACGAATCAGATCGATGAACCACACTGGTGCCGGTGCCGGT

GCCGATACCGGATAGTCATCTCATTGGTTAGCAGAGCATAGGATTTATTTCAATTTTTTG

GGCGCAACTTTGATCGATGCTCCCATTATGTAACTAGTAGAGTTTAGGATGTAACCAACA

GAGTGTAGAATGTAACAACATATACTGGAGTACATGACTCGACTTCTTATTACTCGTGCA

ATGCTTTCTTACTAG

**>TaMPK21-2(3B): TRIAE_CS42_3B_TGACv1_221708_AA0748020.2**

GAGAATCTGGTTTTGACTCTCCCTCGCCGGCGGCCGGCGCCGTGCTCCCGCAGCGGCGCA

GCCCCCGTGTCCAACCCCAACATCTACGCCCCGTTGAGTCACGTGCCGTGCCCGCACGAA

CACCTGCTCCCATATCCCTGGCCGCGCTCCGCGCTACCGCGGCGATCGGGCGTCCACTGC

CCAGTCGAGCACCTTCCCTCGCCGTACGAATATGGCCCCTGTTCCCGCTCCTATCGCGCG

CCTCCCCCTCCTCCCTCGCCGTGTCCACACCCCCCTCCCTCATCCCCGACCGTCAGCACC

GGCGGAGGCACGACACACCGCGCGCACGAGGGCGCCGCAGCGGATCGGAGGAGTGGATCC

GGGGNCGCGCCTCCCCCTCCTCCCTCGCCGTGTCCACACCCCCCTCCCTCATCCCCGACC

GTCAGCACCGGCGGAGGCACGACACACCGCGCGCACGAGGGCGCCGCAGCGGATCGGAGG

AGTGGATCCGGGGGGAAGGGAGACGGCGCGGGGTAGCGGCACATCACTCCGGAGCACGGG

GCGACTGGCGCGGCGGGCGCCATGGCTAAAAAG

gcgcggcatgtgaaccaccctagttgacattttgcttcgcctgatgcgtatccggttgct

ggattgactcgtacgcttccag

GGCTCGGATAAGGGGGGGTTCTTCACGGAGTACGGCGAGGCGACCCGATACCAGGTCGGC

GAAGTGGTCGGCAAAGGCAGCTACGGCGTCGTGGCGTCTGCCGTCGATACCCACACCGGC

GAGAGCGTCGCCATCAAAAAGATCGACGACGTCTTCGAGCATGTCGCCGACGCCACCCGC

ATTCTCCGCGAGATCAAGCTGCTCCGCCTTCTGCGCCATCCAGACATCGTCCAGATCAAG

CACATCATGCTCCCGCCGTCGCGCCGCGAGTTCCGTGACATCTACATAATCTTCGAGCTC

ATGGAGTCCGACCTCCACCAGGTCATCAAGGCCAACGACGACCTCAGCCCGGAGCACCAT

CAGTTCTTTTTCTACCAGCTGCTTCGTGGGATGAAGTACATACACGCTGCCAATGTCTTC

CACCGGGACCTCAAGCCCAAGAACATTCTAGCCAATGCCGATTGCAAGCTCAAGATTTGC

GACTTCGGCCTTGCTCGGGTTTCCTTCAATGACACCCCGTCCGCGATATTCTGGACG

gtatgtacttcgctctaatgaaagcttagaatgattatgtctattatgtaagcagccagc

caaaatttagacttttatgctcgactggccatttttctcccag

GATTATGTAGCAACAAGGTGGTATCGTGCTCCAGAATTATGTGGCTCCTTCTTTTCAAAG

gtgagaaactccatctcgacaaaatttgaccttgaccaaattagagactgaatccgtgat

aattcatctgtgagcatcagtatcctcttgatgctgtcgagtttgttcataaatctgagt

tttcttgtacacttgattgttgacagttgacaccatgtgaatgcacaatattaccacatt

ggacaattcggggttcactgtactgaacttagtatttgcttttgcag

TATACTCCTGCGATTGATATTTGGAGCATAGGATGTATATTTGCAGAAATGCTTACAGGG

AGGCCGCTCTTTCCTGGCAAAAATGTGGTACATCAATTGGATCTCATGACTGATCTACTC

GGCACTCCTTCGGCAGAATCTATTTCTAGG

gtgagttcaatccccgcttctaaaataaataaatcagttagagacttatctcgtgcgtga

tttatttaccaactctttgtgcttgtggttaccatttcctttatcaagatgaaatattcc

tgctataattggatctgccgtttaatacatacttacattatgtctgaaaagtaaaggact

gcctagaactcaaaaggcctgattatttttataggggcagtcgattttgttgaaccactg

gatttagatccaatagctcgctgtacttctgcaacctccagactctgtcttccttctttc

acctttatccccgctgtaaaccctctgccaaggtatcccctgcgcgttggcagctaccct

acctcgcggtccactgtattctcttaacctttggcttggggtttccagcggcttccacga

aaccaacaatcctacccccctaaccacttcggcaaggtctcctgcctcggccagacggcc

acatagccttaaccactcttgtgtcgccggtggctctgcatcaaaatccaccggtgcgtg

aggttgacatgtcacatgactgcatttcaaagaactacccgacagcaaaaggataaaatt

catgcttcgtttaatttgtgtttatcataagaaacttccaagtgatgttttggttgaata

attcactatctgaaaaggcatctggtcttagacagtcccacactcccaatgatcatatat

ttgcttgatttgtatactatgtaatccttaatttctggaaagggcacacaaggtttttgg

tccgttgtgctaaaaaaatatggacgataattgatctaacaatgatgctttacaaacaac

taagcttgctccatctatggctgagggagttatcttttgaatattattcttacagaaaat

aactgtggtttgtaaaccttttttctggatgcaaggcattcagagtgatggtttggtcaa

ataattcactacctgaacaggcatgttgtctcagacagtcctgcactcccagtgaccata

tatttgcttgatctgtgtatagtttgcactccttaatttctggaaagggcaaacacgata

tctggtccattgtgctaaaaaaaaatgggcggcaattgatatttaacaatgattgatgac

aagcaaataagcttgctccatctaaggctgagggatttatctttcgacatatgtaatttc

cgaacattcggattttgtctttagatccgaacgaccacttctcgcgtgcgcattactgca

cgatctgcttatgacgcgcgcgaagatttcgcggagaaaaaaaagtgcgcccacatgaat

ttgaactccgacctgctggttgaggagcagctacgcaaccacccagactgagcctacttt

tgtgttttcgtttcaggttagctgagttttatactctgttagtctgtttgatagcgagaa

aaaacgtgacgagggggcagacccatggaccaagttatttcaaggatcgatgcattgtgt

ttgtacctctcgatgagtaattatagagaaaagaatgtttctcttcgtgtagaattcgaa

ctccaatcattgttttgtgtgtaaacccgaggaaaaaaattgttttgaaatatacgttgg

atagtctgataatttacacaccacaaacctggtaatttatatacaaacaccgcaacaatt

ttttttgacccagattttttgttgttaaaatataagatccgataacttacggggcaacac

cgcggtaacctcttttttatctgatagcatagtagtttaggtctttaggaatcacatacc

tgataacttacatgccaacaccgcgataatcttttattcatcaggaaaaaagttgatgaa

atataccccggtaacttctgtgtgaatagcatgataatttacacaccgcaaatctgataa

cttatgtacctcgggtctggtaactttgacgggggaggggactttgttgaaacatacaac

ggtaacttctgtgcaattatcatggtattttcacacgctgactgataacttacataccct

tagcatggtaactttgacccaaggaaaaaagtatgaaacatagcccgatcatttcttttt

aaagagcatggtaatttatgcacaacattcctcataacttacctagaaacaccgcggtaa

cttttgacccaggagtttttttgtttgaaaacatatcaccggtaatttctatgtacatga

tggtaatatacgcactgcggacctggtagcttacgtacaaacattgtgtttaactttgac

cccaagagaaaactgttgaagcatactcccgtactccgacaatttctatgtaagtagcat

ggtaacttccgtaccatggaccttctaacctaggtacaatcaccatggtaactttgaccc

gagggaaaaaattattgaaaaacatacccccgataacttgggtgtaaataacatgccaat

atcctgttaagtacaaaacaatgcgggaacttccctcaaaaaaaaaaaaaacaatgcgtg

aactacaaaggcatctgtggtgacttttttcaatctcaagatgaagtgtcggcctagtct

ctcggatgctcataggggtagtgtgtccgcgtgtgtgtgtttataggggttaggatatgt

gtgtgtatgagcgcccgcgtttgtattgtgtttaaaataagaactttggccgaaggtgag

gaggggacagaattgctgaaaaagtatatccgatacttatgtgtaaataaggtatgtatg

aaccatagacccgatgataacttcggcgcatggataaagtagtcgaaacatccttgtgat

aacttcagtgcaaacactatgataagttacgtgctgcagtggtgatagcttacgtagttt

aggcgtggtaactttggactggaaaaaaatatcgccgaaacataccaacatgggatctcg

tttcgaagatctcgtcgcgatgatttttttatgtgaaaaccgattttcaatcggagcgac

ggtttgagctacaaaacattttgaagtttgaaaaaaaaagaatctaggatgacatcagct

tttcgaccttttgtgcatatatgcatgtaattagagggaggagtgcacgtgaaaggtaaa

ttagtcattctaatacatgcatgtatgtaattagagtgaagtaagaatcattcttgccta

ttgctaaaatccgaacgtttggtacttatagaagtcctttatcttttgaacattgttctt

atggaaataactgtggctgtaaccttttttttttggacaaaaggcctttcgccctccttt

atagataaagccacaaatggctatttgcacaaaagttccacaaatactcatgatccataa

agaagttgcatagcacgcttacagcacaaacactcgcgcagcaaaacaccccagccacct

ctgagatagcaaaaaagggcgacacctgaattttcctcaatgtcctcctttgatgatggc

atcacgagctgcaaacaaaatgatcgttttgaaaaaacaattagagggatggttgggtgt

gtggaggatcggtgcctatgtcctttgggtgcaaccattgggagatgatagaacaggata

cattcaaccgattttggatggtagtccagtaataggttatgtgtctgagtcatgtaatca

tgttattggccaggcgacacctgaagtttcctcgatgtcctcctgtgatgatggcatcac

gagctgcaaacgaaaatgatcattttgaacaaacaattagatgaatgtttgggtgtgtgg

aggatcggcgcctatggcctttggatggaaccattgggagatgatagaaagggatacatt

caaccgattttggatggtggtccagtaataggttatgtgtgagtcatgtaatccctgtta

ttgggcagttgtggttgggccttgttttcacttgttaaacttttttagatgttggtcata

gtactttaattaagatggttgtgtgcatggtatgatgcaaaatgcgcgcggggggggggg

ggggggggggggggggggggggggggggggataacccctgcaaagaaaaagatggatgtt

tggggataacccactccgtggtgagtttgttgcacgtgtcctagagcgcacagctctgag

ctgcaaagccgacccacatgtccctacaaatgcgaccctaagcctaaaccccctggatat

gattgtgaaactcgcacgaagcacctaagattcccagtttgcgtcttcgaattcctcaga

gtgcacaggtcccattggacaggccgtggcgcttgagaatctggtcactcggaggcaagc

agccatggatcaactgccaacggaagatattgatcttgggcaggagagacaccccaaagg

atgattttgaccttgagcaggagagccacccgccaaacatcccgaatttgaatcaccgcg

gccccctggcacagcgcagtttacaatggagcgatttgaccgaaaaaaatcctgacatta

agacccaggaaatttcatcggcgccctcagacaggttgaaagaattcacttcttgcctca

gatattacaattctactaattctgcctacccaaatgaccaaagaatgtagagccccctcg

gattgctgcacgcgtggtcgatggtgatcaaaggatccttacaagaggcagaaaagatag

agggnnnnnnnnnnnnnnnnnnnnnnnnnnnnnnnnnnnnnnnnnnnnnnnnnnnnnnnn

nnnnnnnnnnnnnnnnnnnnnnnnnnnnnnnnnnnnnnnnnnnntcatcaacttcatatg

caaatttcccagacccccaagtccttgggtttacacacgtcagcccatttgaccatgtgt

tatttacgcttgctctcagttccttcccaaaagaacctggaccgttgtgaatccagagcg

atgtggattctgccaggagcaggtacatcccaatgatagataggcagatggagggcaact

ttccaggtagatagttgcgctgaggggatacttgataaccagtgcagttcggtagcatat

gtaattgggagaatcactgacgaggctcgctgctggaaggaggcagggctatccctccta

aagactatttgagccaccctgatctggttcaatggtgtgtagtaatgtaatctagctggc

tttgttgttactcccgggcaccagcaccgacccccatctctaatttgtaacaatactgtt

atgtttcttctccttcattaatgaaatatgcagcatccagctgtctcttcgtcaaatgat

agataggtaggagttggtccggatcagttttctcccaaggacataaacttccctttccaa

ggctccaccctgttgcaacttgtactacggatgcacaacagtttcagaaatcaaatatgt

cgatcaataatctgaacatctctttttcctgaaaaactagcctactttgccattcaatta

agaatagcaataatacacaagaggcagtttatcaaacgaatctggcagaaaacgctgacc

cttcatttgtactgtactatttttctgtcagagacctttagataaatttactgctatatt

ttatagcatgttaatcaacctgcttagctaacaatatgactatttatttgagctttcatc

ttataaatccctctccccttcagcttattttggaagatctaatttgtggcctaggtttta

acaaagtatctagccgcaaagaattaaatgtgtttggcctatttttaaatttcacaaacc

gctaagatgaacctgaagcttggccaaacagatccaaagtttggactaattcatggatct

acagataattcgttctgctgttccaagtgtatattgcatttgcattctgaaacttttgga

taatgcttcagtctttttgattaattgtactagtatgatttttgtgattatcttcacctt

gaaataatatttcctaataaaaaatatttgaacaaaggaacaatacagatagtgagtaat

atttgttccgctaagtacttttctaagtgataaatcatgttcagaaatgtttcttaaact

ctatctgttgcctatttgtactgcatgcag

ATTCGAAACGAAAAAGCTCGGCGATATTTGGGAAACATGAAAAAAAAGCATCCAATACCT

TTTTCTCAGAAGTTTCCCGGTGTGGACCCCATGGCACTCCATTTGCTTGAGCGTCTTCTT

GCTTTTGATCCCGCAGATCGGCCAACAGCTGCAGAG

gtaggaaatataatcaacaagaatgttagctcaaaatgctcgattcttttgttatatatt

cgatactgatatgatatcaatgtaattcaag

GCCTTGGCAGACCCATACTTTACTGGATTGGCAAATTCTGAACTCGAACCCACAACGCAA

CCCATCTCGAGACTTGAGTTTGAGTTTGAGAGAAGGAAGCTGGGCAGAGAGGACGTACGT

GAATTAATTTACAGGGAG

gtaatgtcagtcaactgtacgctggggattatggtagtagtctttactaatgcatgaggt

atctctag

ATTTTAGAGTACCATCCTCAGATGTTGCATGAGTATCTTCGTGGTGGAGATCAGGCAAAC

TTTCTTTACCCAAG

gtgagtttggacaaaatgctttgatccacccttgttacaagaagaaagatatacatgtgt

tatctatgtaatgttttgttccgttagtaaagtttgcttttgacaatgataggcatatgt

ttcagtaggtgttattcgttgaactttttgcatgctacaacttgagtggaggtgagaaag

aaaagcataccggtgcacattatgatcctacgaatataacgagcaatgtgtagcttaaaa

tattattttgataatcgttgaaatatcattatcaagcaactgcatatgttgaggctgttt

aacgtaatcagttgcgatgtggtaatggcgtttttcttgtgttaaacaatggtccctttg

gattttgatgtacaagcttttcatttccctaaataaaaatttatgctattgtaaacctta

ttcgaatatgccaaacaggaaacaccatttctttaaccacagttatgattggttaagata

ttgccagtatactaactgatatattattgccag

TGGGGTGGATCGTTTCAAGAGGCAATTTGTTCATCTCGAAGAAATCGGTGCTAAGGGTGA

AAAGACTAGCCCGCAGCTGCGGCAGCATGCTTCCTTACCAAG

gcaataccttttttttactgaagaaaagcattatagtttactcagctggaggtgtttttt

tcatgtcattagagtgaaataggataattcattgatttaaattctgctgcatgaagcaga

gttctgtatgcctgccatatgaggtggatcacgcaaaactcatttgagtttctataatca

ttttacaaataataagcaatatgaacaacacatagggtgtggcgtcgttcctcttaggtc

tctataataataataataataattcattgcctgcatgcctgtgatctctcttgaactaat

atatcctctatcttatctcag

GGAAAGAGTTATCGGCAGCAGTGATGAGCCCGAAAAGCCAAATGCAGATTACTGTATAAA

ATTGCATGTAGGTGAGGTACCAGGTTACACATCGGTGACGGATGGCCTTAACAAGCCACT

GTTGAATACTCGAAACTTCTTGAAGAGCGAAAGCATCGGTGCCTCCAAGTGCATCGTCGT

CAAAGAAAAGCGAGAAAAAGAT

gtaagtgccgcgacaacattacctccctaatacttaataaaaccaaatattttgagttga

ccttttttttctagtaataaccaaaatggatcaatgtcgaaagttgacctaaaaattata

tgaaatgttaaaaacccgaccccagatttttagcgctaaaaatctgacaatcagatttgc

catgcgaaaacagatacaattgcgtataaggatttgccattgttctacagtgcaaattgc

catgtagcataaaaaaaacacagaaaaattgccatggtgcagcgattaattgccatgcgt

tcaatcaggatccgacatacctgaggacgtcagatttttaatactataagtctaacgtca

gatttatattgagaaaattattgtctagatttaaaggctcgattgtctatgcaaatgcga

tatagaacctgatatgatgttgtactgcttttaagtcaagagttaaaggctcgattgtct

accactcccttcattccataattcttgtcgtggttttacttcaaatttgaaattaaacca

tggttagaattatgggacggagggagtaattcataaataacatggtatggtaaaaagctg

tgtgatatgtgattcgtatttccgaaatgagccaacattgagtcacagagtacggaatag

ttccaatgtcaatgtaggttgagaaactgaaaaaaatgcttgagtctattaaagctgaaa

gctcattatgtatagataagttaaacttttgtgcatgttggttggatttctgtttctcag

aacaaactgtcgtggagggtaggcagtatacctctctacaaggggtccagttgtacccta

gcaaagtccttggaatccagtaattcgcattgagtgaacacattgaagttccacgctatg

agttgattctctgatgtaatgtcccctataacttggataacgttgctatcaatcaaatta

tgcgtgctcctttgtttttgcag

GAGGAATCTATGTCTGAGTATATGCATGAAGCATCTGATTAGGAAGCGAAAAGGATTGTT

CAACTCAAAAGTTTGTGATGGTACGGGGAAAACAGAAGCACGTCCGATCCATGGGGGCTT

CACTTCAGAATCCCGAGCGCACGAATCAGATCGATGAACCACACTGGTGCCGGTGCCTGT

GCCGGTGCCGGATAGTCATCTCATTGGTTAGCAGAGCCTAGGATTTATTTCAATTTTTTG

GGCACAACTTTGATCGATGCTCCCATTCATGTAACTAGTAGAGTTTAGAATGTAACCAAC

AGAGTGTACAATTGTCCTTTTCTAAAACAGAGTGTACAATTGTAACCAACATATACTGGA

GTACATGGCTCGACTTCTTATCACT

**>TaMPK21-2(3DL): TRIAE_CS42_3DL_TGACv1_249051_AA0836180.1**

AAGTCGGAGAATCTGGTTTGACTCTCCCTCGCCGGCGGCCGGCGCCGTGCTCCCGCAGCG

GCGCAGCCCCCGTGTCCAACTCCAACATCTACGCCCCGTCGAGTCACGTGCCGTGCCCGC

ACGAGCACCTGCCCCTTATCCCTGGCCGCGCTCCGCGCTACCGCGGCGATCGGGCGTCCA

CTGCCCAGTCGCGCACCTTCCCTCGCCGTACGAATATGGCCCCTATTCCCGCTCCTATCG

CGCGCCTCCCCCTCTTCCCTCGCCGTGTCCACACCCCCCCTCCCTCATCCCCGACCATCA

GTACCGGCGGAGGCACGACACACCGCGCGCACGAGGACGCCGCAGCGGATCGGAGGAGTG

GATCTGGGGGGAAGGGAGACGGCGCGGGGTAGCAGCACATCACTCCGGAGCACGGGGCGA

CTGGCGCGGCGGGCGCCATGGCTAAAAAG

gcgcggcatgtgaaccaccctacattttgtgcttcgcttgatgcgtatccgtttgcggga

ttgactcatacgcttccag

GGCACGGATAAGGGGGCGTTCTTCACGGAGTACGGCGAGGCGACCCGGTACCAGGTCGGC

GAAGTGGTCGGCAAAGGCAGCTACGGCGTTGTGGCGTCTGCCGTCGACACCCACACCGGC

GAGCCCGTCGCCATCAAAAAGATCGACGACGTCTTCGAGCATGTCGCCGACGCCACCCGC

ATTCTCCGCGAGATCAAGCTGCTCCGCCTTCTGCGCCATCCAGACATCGTCCAGATCAAG

CACATCATGCTCCCGCCGTCGCGCCGCGAGTTCCGTGACATCTACATTATCTTCGAGCTC

ATGGAGTCCGACCTCCACCAGGTCATCAAGGCCAACGACGACCTCAGCCCGGAGAACCAT

CAGTTCTTTTTCTACCAGCTGCTTCGTGGGATGAAGTACATACACGCTGCCAATGTCTTC

CACCGGGACCTCAAGCCCAAGAACATTCTAGCCAATGCAGATTGCAAGCTCAAGATTTGC

GACTTCGGCCTTGCTCGGGTTTCCTTCAACGACACCCCTTCTGCGATATTCTGGACG

gtaggtacttcgctctaatgaaagcttagcacagattatgtgtagtagagtattatgtaa

gcagacagccaaaatttagacttttatgctcgcctggtcatttttatcccag

GATTATGTAGCAACAAGGTGGTATCGTGCTCCAGAATTATGTGGCTCCTTCTTTTCAAAG

gtgagaaactttatctcgataaaatttgaccttaaccaaatcagagaccgtgaatccgtg

ataattcatctgtgagcatcagtatcctcgtcgatgttgtctagtttgtttataaatccg

agttttcttgtacacttgattgttggcatttgacaccatgttgatgcacatgttaccaca

ttggataattcgggtttcactgtactgaacttagtatttccttttgcag

TATACTCCTGCAATTGATATTTGGAGCATAGGATGTATATTTGCCGAAATGCTTACAGGG

AGGCCGCTCTTTCCTGGCAAAAATGTGGTACATCAATTGGATCTCATGACTGATCTACTC

GGCACTCCTTCGGCAGAATCTATTTCTAGG

gtgagttcacttccctgcttctaaaataaataaatcagttagagacttatctcgtgtgtg

atttatttaccaactctttgtgcttgtggttaccatttcctttatcaagatgaaatattc

ctgctataattggacctgccgtttaatacatacttgtaagactaattcttacattatgtc

tgaaaagtaaggaactgcctagaacgcaaaaggcctgattattttcataggggcagtcga

ttttgttgaaccactggatttagatccaatagttcgctgtacttctgcaacctccagact

ctgtcttccttctttctcctttatccccgccctaaaccctctgccaaggtatcccctgca

cgtcggcagcttccctacctcgcggtccactgtattctcttaacctttggcttgggtttc

cagcggcttccacgaaaccaacaacccgaaccccctaaccacttcggcaaggtctcctgc

ctcggccacacagccttaaccactcctgtgtcgccggtggctctgcatcaaaatccaccg

gtgcgcgaggctgacatgtcacatgactgcatttcaaagaactacccgacagcaaaagga

taaaattcatgcttcatttaatttgtgtttatcataagaaacttccaagtgatgttttgg

ttgaataattcactacctgaaaaggcatctggtcttagacagtcccacactcccagtgat

catatatttgcttgatttgtatactatgtaatccttaatttctggaaagggcacacgagg

tatctggtccgttgtgctaaaaaatatggacgataattgatctaacaatgattctttaca

aacaactaagctgagggagttatcttttgaatattattcttacagaaaataactgtggtt

gtaaaccttttttttggacgcaaggcattcagagtaatattttggtcaaataattcacta

cctgaacaggcatgttgtctcagacagttctgcactcccagtgaccatatatttgcttga

tctgtgtagtttgtattccttaatttctggaaagggcaaacacgatatctggtccattgt

gctaaaaaaatatggacggcaattgatatttaacaatgattcatgacaagcaaataagct

tgctccatctaaggctgagggatttatcttttgaacattgttcttatggaaataactgtg

gcggtaacccttttttctggacaaaaggcctttcgccctcctttatagataaagccacaa

atggctataagcacaaaagttccacaaatactcatgattccataaagaagttgcatacac

gcttacagcacaaacactcgcgcaacaaaacaccccagccacctctgagatagcaaaaaa

gggcgacacctgaagtttcctcaatgtcctcctttgatgatggcatcatgagctgcaaac

aaaatgatcattttgaaaaaacaattagatggatggttgggtgtgtggaggatcggcgcc

tatggcctttgggtgcagccattgggagatgatagaacaggatacattcaaccgattttt

tatggcagtccagtaataggttatgtctgagtcatgtaatcgtgttattggccaggcgac

acttaaagtttcctcgatgtcctcctgtgatgatggcatcacgggctgcagacaaaaatg

atcattttgaacaaacaattagatgaatgtttgggtgtgtggaggatcggcgcctgtggc

ctttgggtacaaccattgggagaatgatagaacgggatacatttcaaccgattttggatg

gcggtccagtaataggttatgtgtgagtcattaatccctgttattggccagttgtggttg

ggccttgttttcacttgttaaactttttagatgttggtcacggtactttaattaagatgg

ttgtgtgcatggtatgatgcaaatctacatatgcgaccttaaatcccctggatatgattg

agaaactcgcacgaagcaccttgcgtctttgaattcctcagagtgcacaggtcccattgg

acaggccgtggcgcttgagaatctggtcactcggaggcaagcagccatggatcaactgcc

aaaggaagattttgatcttgggcaggagagacaccccaaaggatgattttgaccttgagc

aggagagccacccgccaaacatcccgaatttgaatcaccgcggccccctggcacaacaca

gtttacattgaagcgatttgaccgaaaaacaccctgatattaagacccaggaaatttcat

gggcgccctcggacaggttgaaagaattcacttcttgcctcagatattacgattctacta

attctgcctacccaaatgaccaaagaatggagagccccctcggagcatgcgtggtcaatg

gtgatcgaaggatcctcacaagaggcaaaaaaaaaaaaaacaagggggggggggtgtccg

tatttgaggagccccctgctccagccaccaatccgaccagaacatagtggcttctccggt

acccacacgatgtttggagcccagcggaaaggaccctttaatctgttgaaggctgatcta

gagctgggatcccttgcgtgtgagtcccaagagcagtgccgcaaatcagctcaggcctcg

gagcccctgctcattttggccaagtttcctaagctgtttcatcattaggccgatgttcat

caacttcatatgcaaatttcccagacccccaagtccttgggtttacacacgtcagcccat

ttgaccatgtggtatttacgcttgctctcagttccttcccaaaagaacctgtaccgttgt

gaatccatagcaatgtggatgctgccgggagcaggtacatcccaatgatagataggcaga

tggagggcaactttccaggtagatagttgcgctgaggggatactcgataaccagtgcggt

tcggtagcatatgtaattgggagaatcaccgacaaggctcgctgctggaaggaggcaggg

ctatccctcctaaagactatttgagccaccctgatctggttcaatggtatgtagtaatgt

aatctagctggctttgttattactcccgggcaccagcaccgacccaatttgtaacaatac

tgttatgtttcttgtccttcattaatgaaatatgcagcatcctgctgtctcttcgtaaaa

aatgatagataggtaggagttggtccggatcagttttctccgaaggacataaacttccct

ttccaaggctccaccctgttgcaacttgtactaagaatgcattacagtttcagaagccaa

atatatcgatcaataatctgaacatctctttttcctgaaaaactagcatactttgccatt

caattaagaatagcaataatacacaagaggcagtttatcaaaggaacctagcagaaaatg

ctgacccttcatttgtactatttttctgtcagagacctttagataaatttactgctatat

tttatagcatgttaatcaacctgcttagctaacaatatgactgtttatttgagcttccat

cttataaatccctctccccttcagcttgttttggaacatctaatttgtggcctaggtttt

accaaagtatctagccgcaaagaaatttaaatgtgtttggcctatttttaaatttcacaa

accgctaagatgaacctgaagcttggccaaacagatccaaagtttggactaattcatgga

tctacagataattcgttctgctgttccaagtgtatattgcatttgcattctgaaactttt

ggataatgcttcagtctttggttaattgtactagtatgatttttgtgattatcttcacct

tgagataatctttcataataaaaatattgaaacaaaggaacaatacggatagtgattaat

atttgttcgctaagttcttttctaagtgataaatcatgttcagaaatgtttcttaaactc

tatctgctgcctatttgtactcatgtag

ATTCGAAACGAAAAAGCTCGGCGATATTTGGGAAACATGAAAAAAAAGCATCCAATACCT

TTTTCTCAGAAGTTTCCCGGTGTAGACCCCATGGCACTCCATTTGCTTGAGCGTCTTCTT

GCTTTTGATCCCACAGATCGGCCAACCGCTGCAGAG

gtaggaaatatgatcgacaagaatgttagctcaaaaaggccgattcttttgttatatatt

caatactgatatgatatcaatgtaattcaag

GCCTTGGCAGACCCATACTTTACTGGATTGGCAAATTCTGAGCTCGAACCCACAACGCAA

CCCATCTCGAGACTTGAGTTTGAGTTTGAGAGAAGGAAGCTGGCCAGAGAGGATGTACGT

GAATTAATTTACAGAGAG

gtaatgtcagtcaattgtacgctggggattatggtagtagtctttactaatgcatgaagt

atctctag

ATTTTAGAGTACCATCCTCAGATGTTGCATGAGTATCTTCGTGGTGGAGATCAGGCAAAC

TTTCTATACCCAAG

gtgagtttggaaaaaaatgctttgatccaccatttctttttacaagaagaaatatataca

tgtgttatctatgtaatgttttgttccgttagcaaagtttgcttttgacaatgataggca

tatgtttcagtaggtgttattcgttgaactttttgcatgctacaacttgagtggaggtga

gaaagaaaagcataccggtgcacattatgatcctacgagcaatgtgtagcttaaaatatt

attttggtaatcattgaatatcattatcaagcaacatatgttgaagctgtttaacgtaat

cagttgcgatgtgctaatgacgtttatcatatgttaaacgatggtccctttggattttga

tgtactccctccgtcccataatataagagcgtttttcacactagtgtagtataaaaaaag

ctcttatattatgggacggagggagtacaatcctttcattgtgaaaataagcttttcatt

tccctaaaaaaaaattatgctattgtaaaccttattcgaatatgccaaacaagaaacacc

atttctttaaccacagttatgattggttaagatattgccagtatactgactgatatatta

ttgccag

TGGGGTGGATCGTTTCAAGAGGCAATTTGTTCATCTCGAAGAAATCGGTGCTAAGGGTGA

AAAGACTAGCCCGCAGCTGCGGCAGCATGCTTCCTTACCAAG

gcaataccttttttttactgaagaaaagcattatagtttactaagctggaggtgtttttt

ttttcatgtcattagagtgaaataggataattcattgatttaaattctgctgcatgaagc

agagttctgtatctctgccatatgaggtggatcatgcaaaactcatttgagtttctataa

tcattttacaaagaatgagcaatataaacaacacaaagggtgtggcgtcgttcctcttag

gtctctataataataactcattgcctgcatgcctgtgatctctcttgaactaatatatcc

tctatcttatctcag

GGAAAGAGTTATCGGCAGCAGTGATGAGCCTGAAAAGCCAAATGCAGATTACTGTATAAA

ATTGCATGTAGGTGAGGTACCAGGTCACACATCGGTGACAGATGGCCTTAACAAGCCACT

GTTGAACACTCGAAACTTCTTGAAGAGCGAAAGCATCGGTGCCTCCAAGTGCATCGTCGT

CAAAGAAAAGCGAGAAAAAGAT

gtaagtgctgcgacaacattacctccctaatactattaataaaaccaaatatgttgagtt

gaccttttttctgttaataaccaaaatggatcaatgttgaaagttgacgccagattttta

gcgacgatcagatttgccatgcgaaaacagatacaattgcgtacaaggatttgccattgt

tctacagtgcaaattgccatgtagcgtaagaaaatcacagaaaaattgccttggtgcagt

gattaattgccatgtgttcgatcgggatccgacatacctgaggacgtcagatttttaata

ctataaatctaacgtcagatttatattgagaaagttcttgtctagatttaaaggctcggt

tgtctatgcaaatgcgatatagaacccgatatgatgttgtgctgcttttaagtcaagagt

taaaggctcagattgtctaccactcccttcattccataattcttgtcgtggttttagttc

aaatttgaacttaaaccacgattagaattatgggacggagggagtaattcctaaataaca

tggtatggtaaaaaagctgtgtgatatgtgattcgtatttccgaaatgagccaacattga

gtcacagagtacggaatagttccagtgtcaatgtaggttgagaaactgataaaaaatgct

tgagtctattaaagctgaaagctcattatgtattaataagttaaacttttgtgcatgttg

gttggatttctgtttcccagaacaaactgacgtggagggtaggcagtatacctctctaca

aggggtccagttgtaccctagcagctttcagaacatgccttataagtccttggaatccat

taattcacattgagtgaacacattgaagttccacgctacgagttgattctctgatgtaat

ggcccctctaacttggataacgttgctatcaatcaaattgtgtgtgctcgtttgtttttg

cag

GAGGAATCTATGTCTGACTATATGCATGAAGCATGAAGAACATCTGATGAGGAACCGAAA

AGGACTGCTCAACTCAAAAGTTTGTGATGATACAGGGGAGACAGAAGCACGTCCGATCCA

TGGGGGCTTCACTTCAGAATCCCGAGCACACGAATCAGATCGATGAACCACACTGATGCC

GGTGCCGGATAGTCATCTCATTGGTTAGCAGAGCATAGGATTTATTTCAATTTTTTTGGG

CGCAACTTTGATCGATGCTCCCATTCATGTAACTGGTAGAGTTTAGAATGTAACCAACAG

AGTGTAGAATGTAACCAGCATATACTGGAGTAGAGTACATGACTCGACTTCTTATTACTC

GTGCAATGCTTTCTTAC

**>TaMPK24(U): TRIAE_CS42_U_TGACv1_640751_AA2072490.1-AA2072480.1**

GCCCTTCCCCCCTTGCGGCAGTGACCGCAGCGACCTCCGGGCTCTGCTACTCGTCGGAGA

GAAAGGCGAATCGGAATCCCCCGCAGAAGGGGAGCGCCGCCTCGGAGAGCACCCGCGGCA

GCAGCGAGCAGCGTGCTGCCCAATGCCGCTCAGCGCCGCCGGAGGGGCACCACACATGGC

AGGGGAAGGGGGAAGCACCCGCGGCGGCGGCTCCGCCCCGGCGACGACGCGCGCTCGTGG

CCCTCCTCCTCACCGGCCTCGCCGCCTCCACCCGCGTCCCCAACGCCAGGCCGCCGCAGC

CGCTCGACGCGGCCCACCCCATGAACGCTG

gtgagtccctccgaacatctgtcggcgccgtcgatctcagtagaaacggctggatttggc

ttgtctagaaacgtccccgtcgtgctcgttgattcttccgtgcgacaaccccgatgcctc

ctactggttgaacacagtgtgtaagctcgctcgctcgatttgacttgtcctgcgtgctcg

gggtgttcacccggtccaaacctcccgccggcttttcaccggaaactttggtagaaattt

tccatacgaactaaggtggaaatgacggctgaatcgcttgatttcctcctacccattttg

acttgcatgaagcagtggaataaataaagccttttcctgtgccgtctttctttggtttgt

ctaggaggtctcgcatcaactaatttattccggggtaaatcattgcatcttgattgtata

ctagttggcatgtccatggcgtaccactgagacgagcaacagaggcttctccttctgcaa

agctcgcccaattgatagatggatgcggtagtggtgttgtgtgattcaaggggttcagat

tcatgttccaagaacctgcaacctgtgcgcttctaacccaaactcatcttttatttctcc

aaccgggatcatgcaggtcttctcttatctacagtttcctgttccctttttcatatctgg

agtagtggagtacaagttgatttgctgacgctttattctaatctggctacgtggcggtgc

tattccttaggtggcttctggtgttggtaatgcgaatcatacggcttaggttcctgctct

gcccatgtgctggaatctattttcgccgtagtcatctgtaccatcacgcttattctatgg

aagcaacaattgttagagctgttcacatctgactatgcaaccccaaacgaattagtcctt

ttgcagttgcttgctccaccaaggcgcatttcttttctccatttttcgtctatattatac

atctcatggggtcaaacacttatttggtgccgggtgattctcctccgcactgttgtgatt

tagattaatttcttggatagtcttgcggaagctcccgtgtccttgtccttttcatgttgt

cggcaaggtctaattctgccgtcaacatgtcttggttgcaccatggaaccagattctttt

caagtctgaccttcataaggagatcacattgttattagtctggatagagatgctagttca

ttggtcatgatatttacagggaaattactttagctggctgctggtgttggtataccataa

ggctttatttcctgctctgctctcctggccatgagcatgaaacactattcctgctgtagt

catgtgtcccatgaggattattctgtagagtgacaattattatagcttttcatatctgac

tacgcaaattcaagcggattagttcttggcagctgcttgcttcaggaaggtgcagttctc

ccatttttttttgtagagcttgtggtcttgtttttccctgcacgaggtcaaatttgtttg

atagtgccttaacttcccccagtagtgcatactagttagatttaccagttttattcgaca

ttcccgcaaaaactctggtgtccttatcactttgatgttgtctgcag

GGACTAGTTTCACCGATAAGAACTCGCAGTTCCACCATGTTCCGTGGAGCCAGATCCTTT

TAAATTGATTAAGTTTGACATTCAGAAAGGGACATCATTTTTGTCTGGAAGCGGGTGTTG

GTTCCTGGCCACGATGGTTGCAGGGAAAAGAGCATAG

gtgtgtagctattatctctctgttattttactcgcttccttgttgatttctctggtgact

ccatgagcgtttagtaggctctctgttaaacaaggtactatgcttgagcatctttctgca

aactttgtgtggttgtgtggcttttctgttgcaacctatcaagttcccttgtttgatggc

aagattttgctatgtgtcagtacccgattgatgcagttcgaaactctaaatacttatttt

ctttctctattagtcttgtacatcggactcatttgttattgaacaggttttatgatgagt

cattcttgattattcttgcatccattctgttttgacaggtttcgacaattgggtttccat

tcctttaaattctgtgtgtaactttagtacctctgaactattttttcaaatgtctttaga

tgctcaaaatcttttcttttgagctgtggactcaaatcctttgcttccagtggaagagga

atgcttcacctttcacttcatcttagctatccattttaaacgaatgagtgcaatcgaatc

ttgatctattgcagctcaggtgacagtttagggaggacactgaacctcacgagaaacagt

ctacgaatttgggcagctactgtactcttactacatggaagtgcaaactaccatgacaag

agcatcttggtgcgcaggtgctattgcagcagatcacatgttgcatggcagcaaagacaa

agagttggtaactctatgctgtaactttaatgggtgactgttgctctgttagtgccaacg

atgccgtcatctctggtcatctgcatttgccgcgctgtcgtcgcataatgtgaaggtgtt

gggatgatgaaagggagggttgctaaggtagctgacatatgtacagatgataatggcatg

cattcacagagttggatgtgaaagaggaaaaatgggatcttgagcgaaacatcagattgt

ctcttgtttggtgagacgggaacacattgcctatattgctttggaccattcttacttaag

ttgtggcaaactttggtatccgaccaaacacccctcatatcttggccctttgttcgttat

ggatatcttagataaatggaaaggcataatatgcctattttatctagagacagttgctaa

gcttactagataataaaatgccgcacttgcctaaatgaactatgtggattttattatgga

taaatttatatctatatctactagtaagttgcacatgctttgcatgtgaggagaaatttg

ttgcataggagtaatttatatggaacaaatacttgcttttttcttctcaatgcttggttg

gattaaatcacaaggtatggattgtgacacacagatcattaatctaagattttttttata

tacgaacttcaatttcaccgttcctttttctttttttctttttaccactcccacagttaa

cccgtggtctttctagacattcccaccatcttcctctcttgctctcttccagccattttt

ttctgctcactgccaccttgttccacgatgctgggtgttgtcacctattttccttgtgcc

acacactcgtttcctgtccagctccggcatgcaagccaccccaccacagcagagtggagg

tgcacacaagaaggcaccggagtgcagctggacgcctggtgtgagatttcaccaggcagc

aaggaatcgactgagccatcaaggagcgaagaggcgcgagcagctttcctatactctaat

ttaggactcaggaaatgacatatactacctctgtaccaaaatataagacgtttttgtagg

ctatattttgatacagagtgagtatcttttaggacttgaacctcacctgcaagtagataa

cgttcctctccccaataaacgcctgacaaatcttgctattgaaattctagccacaaaaga

tctgtttccatctgtggtgtactaattgcgttttccggcaaaaaggttaaaaagatcagt

tattggtgctgcaccagatgtagcatccgcgtggagaagatgaactctccactagagcca

aacttggcgttgcaaatgtgagcccatgaatttgactactttagccctcctttccctttc

ccctacctctatcccaccctccacagcacatctttgttcttcttcactcctcccgtcctc

cattgctcaccatcttgcctccgccttgtaccgaatcagcacaaatgcccaattgcttcc

aatctgtttaatttgcttcttgtggctgcagacaccactttttggccttggcgtgcagac

tttggatctaatcccgattagcttatcacatcctcgctaattggtaacttggtgtgaact

cattaatttccaagcaatcatgtgctgtggagtatttttcttattcagttcagcagatat

ttactgaataattctccattctagcacacctcatacttgtatgtctagcttaaacttaac

agtgcttcattcggatctagttatttggattgatattttctaggtgcctttgaaagttga

cacggagctcatttcctcccattctgaacattgattatgatggccatttgcttcaaatta

tgttgccatattgttgtgtgtgatggtagtaatacaaactaatccattcaattattccat

ttctgttacttgaactttattggtttcgctgtgctgcttgctcaaagtttgttggccaat

ctaatgcttttctatcaatcttctgttagacttgtccattatcatcagaagagaataaca

atcttccgctttactgtggtttagctcttgggaattgcttaagttcttctgtgacaattc

ttcttgaccttttcttctccagtagtttgcggaagctattctgttgtaaatttgcttctt

cacagtaacatatactggcttaattatttctgaacttgctttctgattgatttgttttta

ctagattaatcatttggtaatccctcttaggtatatctgccctccaacttttgcattgct

tttataatgcgcctggttttaacaaatttggcgatacatgaattagtgttatttctcttg

gcttatccttttttttctgtgtaattgactacctagaattttcccttacacatgctctat

atattgtttttgcttcagatgtttatagaattcaagattctaccaacattgtcgttcgtc

attcttctgttcatgcattctgaaattgtgatcttttag

GTAACATTGGGCATGGAGTTCTTCACAGAATATGGGGAGGCAAGCCAGTATCAGATCGAA

GAGATCATTGGCAAGGGAAGCTTCGGAGTAGTTGCTGCTGCAGTAGATACCCAAACTGGG

GAGCGGGTTGCGATCAAGAAGATACATGATATGTTTGAGCATGCCTCAAATGGCACCCGC

ATTCTTCGGGAAATCAAGCTTCTTCGGCTTCTCCGCCACCCAAACATAGTTGAGACCAAA

CACATCCTGCTTCCCCCTGCCCGAAGGGAGTTCAGAGATATTTATGTTGTTTTTGAGCTC

ATGGAGTCGGACCTACAGAAAGTGATCCAAGCAAATGAGAACCTCACTGCAGGGCATCAC

CGTTTTTTCTTGTATCAACTTCTTCATGCCCTCAAGTACATCCATGCAG

gttagttcagaaaccgtcggcatatcattcaccttatcatcaacaatcacgtcttatcaa

gtgtgaatcctttgtacagtcttattttgaactctcatgaaaccctgtcttacttttatg

catatgatttatcttgtcgcag

CTAATGTATTTCATCGTGACTTAAAACCGAGCAACATACTTGTCAATTCAAACTGCAAAC

TAAAGATCTGTGACTTTGGGCTTGCACGCGCATCGCGTGATGATCCTCCCTTGGCTATAT

TTTGGACT

gtaaggcgtttgtcgctgtattaaataatcattttctatttctctcctcaagttacttac

ctagtgcactttgcaatttgaag

GACTATGTGGCTACAAGGTGGTACCGTGCTCCTGAATTATGTGGCTCATTTTTCTCCAAA

gtaagttggtgtctgtttgtttgaggctaagtttgcctatggttgacacacatcacacac

ctaaggttgggcaagtttgattgacgtaggtaggtctttggttcaagccacaccttcggc

aggattctttttccaccaataaggtcccacatgccatacacttacaaagtgtggcaagat

tcccttaggctccaatttcgttgaactaaccttaggcaagtttggcaaaaataattgtgc

aaagtgtgacattgttagtcctaaaaccaaacagccccttagtcttcaattctcagcgca

ctatagtcaaatagtacagagaggaagcctcttgcagtactaaatctgtatgccggtgtt

gcttgttattgcag

ACTTGTTTCCAATGCATGAATCATTCTAATGCTTTAAATGTGTATATGATGGTTTTTTTC

TTTCCGAACGGGCACATAAGTGCTGGTCATTGTAGTGGAAGAAAAATGCCGAGAAGGCAG

ATGTCTCACACTGCCAAGAAACGCGAAAAAGAAAACAAAAGAACTGTAAATGTCCTAGAC

TATGGGCTATGGCTCAAGGCAAAGGATCAGGAGGGTGTCCTTGTTGTGGCAAGGCCCTCA

GCACCTTGGCACCTTGGCACCAGCGTTGTGCAGCCTCCATAGCTGCCACATGGTGAGGAT

GATGATTGAGCGCGCTCCATTGGATCTCTGACCACCGGCCCTCTCCACAGCAACTTCAAA

CCAGGTGGGAAAATTATCACCGGGCTGTGGGCAGAGATGCTGGAGCGAGAAGTGGCTTAG

GACCGAGTGCCAGACCTGATGCGCGAAGACACACCCTGTTAACAGATGCGAGTTTGTCCT

GCCCTCCTGGTCCCACGATGGGCATTGATTGGGGTCGGGTAGGCCTCGCCTTTGGAGACG

GTCAGCCGCCAACATCAGTTGGCCGCTGCTAGCCATGCAGAGACTTTGCATTTCAGAGGA

GCCCAGCTACCCCAGATCGGTGCTGAGAGTGGTTTTTAGACACCGCCGGCGAAAAGAGCC

ATGTAAGCAGATCGAGGGTTGTATGGGCGGGAGGCGGAGATAGTCCAGACGTACTGGTCG

TTGCGTGTCGGGGGAGGGTAGGGCTGTCGACGATCTCCACAAGGTGGAGTAACTCCATAA

TTCCCTGCACAATGAGGGTGCTTGAGATATCATGGATCTATTGGCCGTCAGTGCATGCTT

CGCTCACCGTGCAGCAATTGATAACACACATGCTGACTGCAGCAGGTACCATAGGGGCCA

GATTGGCCACTGACATCCTGTTATTCACCTGTCCAACCAAAATAGGGCACGGCGGCCGTC

ACTCAACATGACCTTGCTCGCTGCACGAAGGAAACTGTCGACGTGCCTTCTATCTTAATA

GGGAGTGAGCCCATGGCTTGGATGGGTCAATGCTTGGAGCCAAGGCCACCTAGTCCTCAG

AGCCCATCCCATCTTCCTCGTATCTGCAATGCCCAGACCGCCGAGCTTGACCAGGCGGCA

CACGTTTCGCCAAGCTACGGAGCAATTGCCACCTTTCGCCTCACGATGAACTCGCCAAAA

GAAATTGCATGTTTTCTTGTCGAATCGCTCGCGCACCCACTTCTGCAATCCCCCAGCCGT

CATAGTGTGTGTATGATGTTATCTTACCATATTATTAAAACAAGTTAACAGCCACCACAT

ATCGCTCTTAAGGAGGTTAGAAATTCCCACATTAATCAGAGAGGAAAGCGACATTTCAAC

ACCATTTATTTTGGTGGTCTCAAATTATAACGGTGCAGATTAATCTGGTAAATATTAAAG

CTATTACAGTTGTATAAGGATTACAACTGCATTTGTACGTACTAACAAGTGTTAAGAGTT

ATGCAGCAAATACAGCTGTGTTTTCAAA

tgtgaacatgcgctttagtatctaaccactcaggaaatatctttgaaaaa

tcacctttagaaagtacccaaatgcaccagattatggcgaaaactgaagtagt

aaagttgtaagcagtacagttggcaccaattttcctttctaaacatggaaattttgctga

acttctcttcttgcagtggacacttgtagaatcattatttgcaaagcnnnnnnnnnnnnn

nnnnnnnnnnnnnnnnnnnnnnnnnnnnnnnnnnnnnnnnnnnnnnnnnnnnnnnnnnnn

NNNNNNNNNNNNNNNNNNNNNNNNNNNCGTCAAGCACCAACTAGATCTGATAACAGATGT

CCTTGGAACTCCATCATATGAAACCCTATCCCAG

gtatgtgtacattatcacccattcactagtggacaaagatttcactgttttcctgttttt

tgttagtaccatactggtagtttctttaaaatgaagcatatatagacttggccacagata

gaattcttgctttctgtaaaatgcaatacaatgtgacagaactacttccccaacctgact

cacaaacgcacgacttctgtgtggttggttctctaagtaaattgtttcctagataataac

ctgtttgagatttttgctacttctagcatatgccattgcacagtagtgctcaagcaatga

atttcttaacatggctataacaactgcag

ATTCGTAATGAGAAGGCCAGGATATATTTGACTGGCATGAAGAGGAAACATCCTATCCCC

TTTCCTAGTATGTTTTGTAACGCTGATCCTCAGGCTGTCCGTCTCCTAGAACGCTTACTC

GCATTTGATCCTAAAGATCGACCTACTGCTGAAGAG

gtatatttttgacaaaaaagagttcttgcttttatgatgaccgattccgaaattgatgag

tacataaatgtaatattttatatatgtattctag

GCTTTAGCTGATCCATATTTTGAAGGATTTCCTAATTTGGAACATGAGCCTTCACCACAC

CCCTTTTCAAAACTTGAGTTTGAATTTGAGAGATGGAAGCTAACAAAGGATGGTATAAGA

GATCTGATATATCGAGAG

gtagaaaaccatcacttcagtgtatgttttaggctgactatatcagtctaacttaaaatt

ggaattttccttttcttgaaagcaaacag

ATTTTGGAGTACCATCCACAGATGCTTCAGGATTATATCGGAGCTGGAGGACAGACTAGT

TTCGTCTATCCAAG

gttaggttgttttgccatctgctgccagcacaccagcaccagcgtagatgcttagcatgc

acatgaaattgtatgctttgtatgcccatgtcacattttgttttcacacagaaatcgttg

ggacttaggaggtctaacctagtttaataccccttatcagcggtataatcttgctcataa

tctaatactaccacgttgcag

TGGGGTTGATGGTATGAGACTGCAGTTTGTACATCTTGAGGAGAACCACCTCAGAGGAGA

AAGAGGTACTCCACTGCGGAGGCGACATGCATCTTTGCCAAG

gtacatgagtcattattaactttcagcagtttcaacttattgtaactgcacaaattctgg

tatggttctgattccgcttgcttctgcactcttcttcatgtgtcag

GGAAAGAGTCTGTGCACCAAAAGGTAGCGATAATCAAGACTGTAACAATGAGAGAAGGAG

GACAGCATCTTCTGCTGCCCAAACTACCATAAGATCACAACAAGAGGGGCTGACACATGC

ATATGTTTATCAAAATGGCACAAGCATTCCGAACTTCTGCTCTGGGTATTACTTGCAGAA

TGGTAGTACCAGTGCTTCCAGTTGTGTCATCGATGAGCATGAAGGCCTGGAG

gtactcccggcaccaattacgatgacactgcctaaattcctcccaggatcttataattat

cagtgcacagtcactcttctcgtgacgactcgtgcttatgcgagaaactgaccaatagcc

aatctgttttctgtacag

GAGAACGGCGTCTCCGAGGAGGAGAAGGTGGCCTATGAACTGTCACAATGTTTTGCCAGG

ATTTAG

CCAGGATGCACTCCTAAGCAACCATGATCCCGACGCAGCTCCAAATTTCAGCAC

AATGGTTCCACCATGTAGTCTTATGGGTCCCGGCAGCAGAATAAACCCACCAGGGCGGAG

GTGCTGCGAACTTCACAACAACCATTTGGATTATCCGAAAACATGTAATAAATCTGAGTT

GAAAAATGTTGCGTTGATCTGCATCGGTATACATCCGGTGTAAATTGTGTGGGTACCGTA

CCGTAAGAGCCTAGACCCGAAGAACATCGTGTGTAATGTCGTAACAAAAATTGCAGACAT

TCAGTTTGTGAATTGGTCAACTGCTGGTGGGTTCAGTGGGAGCAGGTGAATGAATCTGTT

GCACGCAGGGCAAGTTCCCGGGCCAGTGACGGACGAACAAGGGGCCCATGCTGAACCCAG

TTAATCCACAGCATTTCTGCATATGTGTAGCTCTATCAAGTGCAGGATATGTAGAATCAT

CAGCACAGCAACAGCATAACTGCAAGAGAGGGACAGGCCCAGTCAGTCAAGCCGCAGCTT

CCTAACTGAACAACCACTCCATCAAGTACTTTTTTTTGAACATCAA

gtactattctctccgtcccaaaataagttttgttgatttagtataaagttagtacaaagt

ttatactaaatcaatgcttattttgggacggagggagtacataacagaaacatgcaaaca

atgcattttgtttcag

ATATAAGTTGCCTGAAATCGAATGACCTGGAGACGGGGACAAGTGGACTGGGCGTCGCCA

CAAGGGTGATGGAGGTAGGTCGCCGGCTGCCGGTGACGGGGAAGGCAGGGCAACTTACGT

ATAGCCAGTCCCATATTAATGCAGCTAGATGCAGATAACTACATACATATGACGAAAATG

GCTCAAGTCTGGTTGGGTGTGTGCGGATATGGTCTTAGTTTTAGTTCGTAGTAGATCACT

GATCTGATCTGATC

**>TaMPK24(6BS): TRIAE_CS42_6BS_TGACv1_513631_AA1646180.1**

GCGGCAGTGACCGCAGCGACCTCCAAGCTCTGCGACCCGTCGGAGAGAAAGGCGAATCGG

ATCCCCGCAGAAGGGGAGCGCCGCCGCGGAGAGCACCCGCGGTAGCAGCGAGCGGCAGCA

GGGAGAAGCACGCTGCCCAATGCCGCTCGGCGTCGCCGGAGGGGCACCACACATGGCAGG

GGAAGGGGGAAGCACCCGCGGCGGCGGCTCCGCCCCGGCGACGGCGCGAGCTCGTCGCCT

TCCTCCTCACTGGCCTGGCCGCCTCCACCCGCGTCCCCAACGCCAGGCCGCCGCTCGACG

CGGGCCACACCTCATGGACGCTG

gtgagtccctcggaacatctctctcgtcgccgtcgatctagtaagctcgcatgttccttg

tagaaacggctgggtttggcttgcctagaaacgtccccgtgatgctcgttgattcttccg

tacgacaaccccgatgcctcctactggttgcacacagtgcataagctcgctcgctcgctc

gatttgacttgtcctgcgtgctcggggagttcaccttcacccggtccaacctcccgccgg

cttttcaccggaaacttcggcagaaattttccgtacgaaatttggtagagatgacggctg

cattgcttgatatttcctcctacccattttgacttgcgtgaacccgtggaataaataaag

ctttttcctgtgccgtctttctttggtttgtctaggaggtctcgcatcaagcaatttatt

ccggagtaaagcattgcatcttgattgtatactagttggcacctccatgtccatggcgta

ccactgagacgagcaacagaggcttttccttctgtaaagctcgtgcagttgatagatatg

gatgcggtagtggcgttgtgtgattcaaggggttcaggttcatgttccaagaccctgcag

cctgtgctcttctaacccaaactcatcttttatttctccaaccaggatcatgcaggtctt

ctcttatccacagttttctgttcccttttccatatctggagtacaagttgattttctgac

gctttattccaatctggctacgtggtggtgctattcctcaggtgtcttctggtgttggta

atgcgaatcatacggcttaggttgccgctctgcccatgtgccggaatctattttcgccgt

agtcatctgtgccatcacgattattctatggaagcaacaatttttagagctgttcacatc

tgactattgcaagtttgcaaccccaaaagaattagtcttttttcagttgcttgctccact

aaggcacatttttttctccagtttccgtctatattatacgtctcatggggtcaaacactt

atttagtgctgggtgatttccctccgtactgttgtgatttagattaatttcttggatagt

cttgtgtaagctcctgtgtccttgtccttttcatgttgtcggcaaggcctaattctgctg

ccaacatgtcctggttgcaccatggaaccagattcttttcaagtgtgaccttcataagga

gatcacattgttattagtctggaatagagatgctagctcattggttattctgtagctggc

tgctgggaaattactttagctggctgctggtgttagtatacgataaggctttatttcctg

ctctgctctgctggccatgagcattgaaactattcctgctgtagtcatgtgtcccatgag

gattattctgtagagtgacaattaattatagcttttcattcatatctgactatgcaaatt

caagcggattagttcttggcagctgcttgcttcaggaaggtgcggttctcccattttttc

tgtagagtttgtggtcttgtttttccctgcacgaggtcaaatttgtttgatagtgcccaa

ctttccccagtagttcatactagttaggtttaccagtttattcgacatcccgcaaaaact

ctggtgtccttatcactttgatgttctctgcag

GGACTAGTTCTACCGATAAGAACTCTCGGTTCCACAATGGTCCATGGAGCCAGATCCTTT

TAAAATTGATTAAGTTTGACATTCAGAAAGGGACAGCATTTTTGTCTGGAAGCGGGTGTT

GGTTCCTGGCCACGATGGTTGCAGGGAAAAGAGCATAG

gtgtgtagcaattatccctctgttattttactctcttccttgttgatttcatttagtagg

ctctctcttaaacaaggtactacgcttgagcatctttctgcaaactttgtgtggttgtgt

ggcttttctgttgcaacctatcgagttcccttatttgatggcaagattttgctatttgtc

agtacccgattgatgcagtttaaaactctatacttattttttttctctattagtctttta

tatcggactcatttgttattgaacaggttttatgatgagtcattcttgattattcttgca

tccattctattttgacaggtttcgacaattgggtttccattcctttaaattctgtgtgta

actttagtatctctgaacttttttttttcaaatgtctttagatgctcaaaatcttttctt

ttgagctgtggactcaaatcccttgcttccaatgaaagaggaatgcttcacctttcactt

catcttagctatccattttaaacgaatgagtgcaatcgaatcttgatccattgcagctca

ggtgacagtttagggaggacattgaacctcacgagaaatagtctacgaatatgggcagct

actgtactcttactacacggaagtgcaaactaccatgacaagagcatcttggtgcgcagg

tgctattgcagcagatcacatgttgcatggcagcaaagacaaagagttggtaactctatg

ctgtaactttaatgggtgactgttgctctgttagtgccaaggatatcgtgatctctggtc

atctacatttgccgcgctgtcaccgcataatgtgaaggtgttaggatgatgaaagggagg

gttgctgaggtagctgacatatgtacagatgataatggcatacattcacagagttggatg

tgaaagaggaaaaatgggatcttgagtgaaacatcagattgtcttttgtttgatgagaca

ggaacacattgcctatattgctctggaccattcttactcaagttgtagcaaactttggta

tccgaccaaacacccctcatatcttggccctttgttcgttatggatatcttagataaatg

gaaacgcataacatgcctattttatctagagatagttgctaagcttactagataataaaa

tgccgcacttgcctaaatgaaccctgtggattttattgtggataagtttatatctatatc

tactagtattagtaagttgcacatgctttgcatgtgaggagaaatttgttccccccctca

aaatgagaagaggggagaaatttgtttcataggagtaatttatatgggacaaatactcac

tttttcttctcaatgcttggttggattaaatcacagggtatggattgtgacacgcaaatc

attaattaagcatttatatatacaaactcccttcaatttcaccgttccttttttttactt

tttaccactctcacaattaacccatattaacgtggtctttctagacatttccaccgtctt

tctctcttgctctcttccagccattttttttctcgtcatttctagtcttctctcttcaca

aaatttctcttccttgctatattacacaagtaagcctcgtcggctcactaccaccttgtt

catcgatgctgggtcttgtcacctattttccttgtgccaccccaccacagcagagtggag

gtgcacacaagaaggcaccggagtgcagctggatgcccagcgtgagatttcactaggcag

caaggaatcgactgaggagcgaagaggcgcgagcagcttccctatactctaattttagga

ctcaggaaatgacatatatcttttaggacttgaaatctcacctgcaagtaggtagcgctc

atctccccaataaacgcctggcagatcttgctattgaaattcttgccacaaaagatctgt

ttccatatgtggtgtacaaattgcgttttccacgaaaaaggttaaaaagatcagttattc

gtgctgcaccagatgtagcatccgcgtggagaggatgaactctccactagagccacactt

ggcgttgcaaacgtgagcccatgaatttgactactttagccctcctttccctttccccta

cctctatcccaccctcccagtccctagcatatcttctttgttcttcttcactcctccgtc

ctccattgctcaccatcttgcctccatcttgtaccgaatcagcacaaacgcccgattgct

tcctatctgtttaatttgcttcttgtggctgcagacaccactttttggccttggcgtgca

gactttggatctaatcccgattagcttatcacatcctcgccaattggtaacttcgtgtga

actcattaatttccaagcaatcatgtgctgtggagtattcttcttattcagttcagcaga

aatttactgaataattctccaatctagcacgtctcatacttgtgtgtctagcttgaactt

aacagtgcttcatccggatctagttatttggattgatattttctaggtggctttgaaagt

tgacacggagctcatttcctcccattctgaacattgattatgatggtcattcgcttcaag

ttatgttgctatattgttgtgtgtgatggtagtactacaaactattccattcaattattc

catttctgttacttgaactttattggtttcgctgtgctgcttgctcaaagtttgtttgcc

gatctaatgcttttctatcaatcttctgttagactcgtccattatcatcagaagagaata

acaatcttccgctttactgtggtttagctcttgggaattgcttaagttcttctgtgacaa

ttctttttgaccttttcttctccagtagtttgcggaagctattccgttgtatttgcttct

tcacggtaacatatatggcttaattatttctgaacttgctttctgattgatttgttttta

ctaggttaatcatttggtaatccctcttaggtacatctgcccggcaacttttgcattttt

ttaaaatgtgcctggttaacaaatttggcgatacatgaattagtgctatttctcttggct

tatcctttttttctgtgtaataattgactacctagaattttctctcacacatgctctatg

tattatttttgcttcagatgtttatagaattcaagattctaccaacattgtcgttcgtca

ttcttctggacatgcattctgaaattgtgatcttttag

GTAACATTGGACATGGAGTTCTTCACAGAATATGGTGAGGCAAGCCAGTATCAGATCGAA

GAGATCATTGGCCAGGGAAGCTTTGGAGTAGTTGCTGCTGCAGTAGATACCCAAACTGGG

GAGCGGGTTGCGATCAAGAAGATACATGATATGTTTGAGCATGCCCCAGATGGCACCCGC

ATTCTTCGGGAAATCAAGCTTCTTCGGCTTCTCCGCCACCCAAACATAGTTGGGGTCAAA

CACATCTTGCTTCCCCCTGCCAGAAGGGAGTTTAGAGATATTTATGTTGTTTTTGAGCTC

ATGGAGTCAGACCTACAAAAAGTGATCCAAGTAAATGAGAACCTCACCGCAGGGCATCAC

CGGTATTTCTTGTATCAGCTTCTTCATGCCCTCAAGTACATCCATGCAG

gttagttcagaaaccgtcggcgtatcattcaccttgtcatcaacaatcacgtcttatcaa

gtgtgaatcgtttgtactgtcttattttgaactctcatgaaaccctgtcttacttttctg

cgtatgattatcttgtcgcag

CTAATGTATTTCATCGTGACTTAAAACCGAGCAACATACTTGCCAATTCGAATTGCAAAC

TAAAGATCTGTGACTTTGGGCTTGCACGCGCATTGCGTGATGATGCTCCCTTGGCTATAT

TTTGGACT

gtaaggcgtttgttgctgtattaaataattattttctatttctctcctcaagttacttac

ctagtgcactttgccatttgaag

GATTATGTGGCTACAAGGTGGTACCGTGCTCCTGAATTATGTGGCTCATTTTTCTCCAAA

gtaagtcagtgtctgtttgtttggggctaagtttgcctatggttgacacacatcacacac

ctaaggttgggcaatttgattgacgtaggtaggtctttggttcaagccacaccttcggca

ggattctttttccaccaataaggtcccacatgccatacacttacaaagtgtggcaagatt

cccttaggctccaatttgaactaaccttaggcaagtttggcaaaacaattgtgcaaagtg

tgacattgttagtcctagaaccaaacaaccccttagtcttcaattccgagctcactattg

tcaatagtacagaaaggaagcctcttgcagtactaaatctgtacgccggtgttgcttgtt

attgcagacttgtttccaatgcgtgaatcgttctaatgttttaaatgtgtatatgatgtt

tttttctttccgaactggcacataagtgctgatcattgtagtagaagaaaaattccaagg

cggatgtcttacactgccaagaaaaacaaaaaaaaacaaaagaactgtacatgccctagg

ctatgggctatggctcaaggcaaaggctcaggagggtgtccttgttgaggcaaggccctc

agcaccttggcaccaggcatcatccagagatgggcttcggtgagaatggcgtccaccaac

tccggtgcgtctagtgaggcatcatcaaatacaaagcgttgtggagcctccatagctgcc

acgtggcgaggatgatgattgagcgcactccattggatctctgaccaccggccctctcca

caacaacttcaaaccaggtgggaaaattatcaccaggctgtgggcagatgctggagcgag

aagtagctttggaccgagtgccagacctgatgtgtgaaggcgaccctgttaacagatgcg

agtttgtcctgtcgtcctggtcgcacaacgggcattgattacggctgggtaggcctcgcc

tttggagatggtcaacatcagttggctgctgctagccatgcaaagattttgcatttcaga

ggagcccagctaccccagatcgacgctgagaggggttttaagacaccgccggcgaaaaga

gccatgtaagcagatcgagggttgtatgggcaggaggcggagagagtccagacgtactgg

tcgtcggtgtcggcggagagggtagggctgttgacaatctccacaaggtagagtagctcc

ataatgtcctgcacaatgagggcgcttgagatatcatggatccagtgtccgtcagtgcat

gcttcgctcaccgtgcagcgattgatgacacgcatgctggctgcagcaggtaccatgggg

gccagatcggccactgacatcccgctattcacctgtttgaccaaaatagggcatggcggc

cgtcgctcaacatgaccttgctcgctgcacgaaggagactgtcaacatgccttccatctt

aatagggagcgaggccatggcttggatgggtcaatgcttggagccaaggccacctagtcc

tcagagcccgacccatcttcctcgtatctgcaatgcccagaccgccgagcttgaccatgg

cggcacacgtttcaccaagccacgaagcagttgccacctttcgcctcacaatgaactcgc

caaaagaaattgcatgttttcttgtcaaatcgctcgcgcacccacttctgcatcccccag

ccgtcattgtgtgtgtatgatgttatctttgttggaaatatgagcaatttaccatatgat

tttattaatggaaatactagataaagcatgactaaaatagcagagataaagcaagtcatg

caatctgacagagagaaggtaaataacatctgcaaatatgaactagaaccgaacatatct

agagctaagagtagaacaagaaattgtgacaggacctctaacagaaaaaggcagaacacg

tacgggacaacaacaacaacagtagcactggacttggggtcggtgtcctcggtagccatg

tcgtcgaggaggttgtcggcgtcggggaagaagtcgttgttggggaagtagtcgtcggag

tccggggcgtctgtgacgaagaagttagtagtcgcgctgagcgctcctcaaaaaccttat

cacccttctcccgcacaggactcaaagaggtggagtttcggaggcctactgtcccgacct

gtggtgcacgccgcaagccgggatggggaagatcgtagcagcagcgcagtgcttaggaac

tttgtggcgagaggaagaggatcttctggtatatctctctgagaggagcgacctcccttt

tataggtgcaactgaaggaggcgagaggctgcgccgggagctgaagggatcgagggagac

gaaacaaacaggcagcagccgaagggtgcagcgttcgcattcagtatccactacagcaaa

aactttccagctcccgagtgacctttcgtatacccgtagtgcgtggcaaatatttagacc

tcggctcggctcattcccgcaacccgcggcgcgttgtgacgaggcgtggcgaggcgggcg

gcggaggaggagcgcgcgtggatgtccctcttgttctcatgctcatacaagtggggaaaa

aaccttccttataaagaggtccaactccctctaaactagcaatgtgggactaaactttag

ttccacctcttgccttgcacgaatgggctgcgtgggcctctaggatttattaggaatttc

tgaaactgggattgggcttggcccataatagacaaaattccagcaatctcctaccagatc

ccagaggcacacaaattttgcctttggttccaaaacactgttttatataccggtactgta

gtggagactgttaagttgaacttccacctagaactctatgttacactagtaaggaacttg

aacagtggactgggctttgaactgcaagttttctgcgaatctagcttcacacaaagcctt

gaccgatactgggctaccgtgggtcttccccgcgggtggagcttatgcgtcatgctccga

gacctttcatgagtttactagagagaaccctactctcatagattgcgacgtttaacaatc

agactcatataggtgtgttcttcaaaagatgttctgcaggacaacatctctgcttaaaag

agccacttagaacacattaagatatacatcatcctgccatgcagattatgagagtattgc

atcttcatggagtggtattgttaacagtaaggatactctcctctcagttgaccaccagct

tgtctttcacatctaattcacgggatctccgatcacaaagaataggttaccactgtgaac

aactcatattgtgggtctcatacccatctccctcgatgcattatctatcacattacgtga

tatacccatagtaaaaggatctgccagatttttagacgtttggatataatccaatgcaat

aactccagagtttctcatttttctgacagactttaaccttctctgaacgtgtcttggtga

cttcatgttatcctttgagttgctcactttcgtgatcacagtttgattgtcgcagttcat

aaggatacccggtacaggtttctcaacaacaggcaagtcattaaagagtcgacgaagcca

atctgcttcgactgtagctgtatctagtgctgtgagttctgcttccattgttgaccttgt

taagatggtctgcttgcaagacttccaagaaacagcgccacctccatgagtgaatacata

ttcgctcgtggcctttatctcatcagcatgtgagatccagtttgagtcactatacccttc

aagcacctttgggtgcccagtgtagtgaattccataattcgcagtgcctttcaaataaca

taaaactctctctagagctttccaatgcacatctcctggttttgagacaaaccggctcag

tttgctaacagcaaaagagatgtaaggtcttgtagcactggttaaatacataagcgagcc

aataatctgagaatatttcaattggtctctagcaattcttcgattccttcgaagcaacac

actagcatcatatggtgttggagagggcttgcagtcattgtagccaaagcgactcaagat

cttttccacatagtgagattgaagcaatggaattccatcatcatcatctctcaacaattt

gatgtttagaatgacatcagccactcctaaatccttcatctcaaagcaacgagataggaa

atccttgacctccttaataaccttcagatttgttccaaaaatcagaatgtcatcaacata

caagcaaaggataaatccctcacccccaccatggcgatagtacacacatttatcaacttc

gtttacaacaaagcctgcggctgttaaagttctttcaaacttctcatgccactgcttggg

tgcttgcttaagtccatacaaagacttcagcaacttgcacactttcccttcctgaccatc

tactacaaacccatctggttgttccatataaatttcctcttctaactctccatttaggaa

agcagtcttaacatccatttgatgaacgagaagaccatgtgaggcagcaagtgaaagtag

tactcgaatagtggtcagtcgagccacaggtgagtaagtatcaaagaagtcttcaccttc

cttttgggtataacccttagccacgagccgagccttgtacttctcaatagtaccatcagg

cctaagctttttcttgaatacccatttgcatcctataggtttgcacccataaggacaatc

agttatctcccaaatttcatttgccaagatggagtccatctcactacggaccgcttcctt

ccagtagtcagcatcttcagatgcataggcctctgaaatagaactgtgagtgtcatctat

gagatacagtagtcagcatcttcagatgcataggcctctgaaatagaactgggagtgtca

tctatgagatacacaagaaaatcatgaccaaaggactttgcagtcctctgtctcttcctc

ctagtaggaacttcattgttctcctccacaagactttcaaagtgttccattgaaatggca

ggttcagtaattgtaactggttcctgattcgatgaactaggcatctcctgattagatgag

gtagccatatccttcatgggaaagatatcttcaaagaaagtcgcatcattcgactccatg

atcgtaccgacatgcatgtcaagtacctcagattttacaaccaagaatctataaccaatg

atatgaaaagcatatcccatgaaaacacaatccatagtttttggtcccaacttccgcttc

tttggaattggcattgactttcgctaaacaaccccagattcgtagataagagagctttaa

gcttttcttttcccattcctcgaatggagttatctctttgttctttgtgggaactcagtt

taggacatgacatgcagtcaatatcgcctccccccaccatgccttggagagacccgatgt

gtctaacatggcgttaaccaaatcagttagagtacggttctttcttttggccaccccatt

tgactgaggtgaatagggaggcgtcctctcatggattataccatgttctgcacaaaagga

atcaaattcatttgaaaaatactctccaccacgatcggacctaagcctcttgatctttcg

atcaagttggttttccgctttagctttatagatcttgaaatagttcaaagcctcatcctt

agatttcagaagattcacatgacaatatctagtggagtcatcaattaacgtcataaaata

cttctttccaccttttgtcaaaacaccattcatttcatatagatctgaatgtatgagctc

tagtggtgcaagatttctcgtttccgcagtcacatgagacttacgaggttgcttagcttg

cacacacacacttgacacttagatccctagacagtggtggaactagggattaagtttaac

ttcgctagtcgcgacatgcaaccaaagttaacatgacagagacgtgaatgccacacattt

gattcactattgttgcaaacatgattaacaactttattgcaaacttctgataaggataaa

cgaaacaggcctcctgactcatagcctttaccaacaaaggttccatacttggatattaca

aatttattcgactcaaagacaagattgtagccatctctacacagaagagatccgctaaca

agatttttattgacggaggggacataatgcacgttcttcagccgcacgatcttccccgaa

gtaaacttcagatcgaccatgccaacaccacgaacagaagcacttgaaccgttgcccatc

agcacggtggaagcccctgcggtctgataagacgaaaacatggaaatatcactgcataca

tgcacattagcacccgtgtcaattaaccaatcaggagaatgacatactgaaagaatagtg

cccaacatccttcatgtcagtgtctccaatgacaacattagtggtcttgccacctttccc

aagatgacgcttgtcatagtgattagggcaactaggagtccaatggtcaggatctccaca

cacatgacaaacacctttcttgtcattcttcttcttgaagttcgtgtgctgtacatcctt

gttcttcccatcaaactttgctttaccatcaaacttgcccttgttcttgaacttgtgggt

ctggaagtttttcttctgcaccagattggcactagaacctccctcaatacctcgagcacg

gttgtcctttgctctcgccttttcttccacatcaagagtatcaatgagatccgggacgga

aaactcctgcctcttatgctttagtaaggtagcaaagttcctccacgtgggaggaagctt

agtgatgatacctccggcaataaacttgtccggtaggatacaattgaagtgctcaagttc

tctagcaaatgactgtatctcatgagcttgctcaaccacggagcgctcttcagtcatcct

gtaatcatagaattgctccatgatgtacaactcagtgccagcattcgagaccccaaactt

ggcctcgagtgcgtcccacatatcttttccattatcaattgacgcataagcatcaactat

gttctcatcaagaacactcaaaagagcagccttaaacagagtatccattttctgaaaagc

ttgtgcctgttgagcatcatactcttcttcaggtttgccaagaatggcgtcatagcagct

catggtttgaaactatagaacggctctcacgcgccacctcttatagtggataccctcaaa

cataggaggtctcatgaaagcagcaaaaccacttggggtaaattgcctataataaggttt

ttggattgttggaaatatggcaatttaccatatgattttattaatggaaatactagataa

agcatgactaaaacaacagagataaatcaagtcatgcgatctgacaaagagaaggtaaat

aacatctgcaaatatgaactagaaccgaacatatctagagctgagagtagaacaagaaat

tgtgacaggacctctaacagaaaaagccagaacatgtacgggacaacaacagcaacagta

gcactggacttggggtcggtgtcctcggtagccatgtcgtcgagaaggttgtcgacgtcg

gagaagtagtcgtcggagtccggggcgtccgggacgaagaagtcagtagtcgcgctgagc

gcttcccaaaaaccttatcacccttctctcgtacaggactcaaagaggtggagtttcgga

ggcctattgtcccgacctgcggtacacgccgcaagccaggatggggaagatcgtagcagc

agcgcagtgctcaggaactttgtggcgagaggaagaggatcttctggtgtatctctctga

gaggagcgaccccttttataggcgcaattgaaggaggcgaaaggctgcgccgggagctga

agtgaacgagggagacgaaacgaacaggcagcagccgaagggtgcagcgttcgcattcag

tatccactacagcaaaaactttccagctcctgagtgacctttcgtatacccgtagtgtgt

ggcaaaaatttagacatcggctcggctcattcccgcaacccgtggcatgacgcgtcgtga

cgaggcgggcggcggaggaggagggcgcgtggatgtccctcttgttctcatgctcataca

agtggggaaagaaccttccttataaagaggtccaactccctctaaactagcaatgtggga

ctaaaccacctcttgccttgcacgaatgggctgtgtgggcctctaggatttattaggaat

ttctgaaactggattgggcttggcccataatagacaaaattccagcaatcttaccatatt

attaaaacaagttaacagccaccacatatcgctcttaagggggttagaaattcccacatt

aatcggagaggaaagcgacatttcaacgccatttattttggtggtctcaaattataatgg

cgcagattaatctggtaaatattaaagctattacagttgtataaggattacaagtacatt

tgtactaacaagtgttaagagttatgcagcaaatatatacagctgtgctttcaaatgtga

acatgcgcttcagtatctaaccactcaggaaatatctttgaaaaaacacctttagaaagt

atccaaatgcagccgattatggcaaaaactgaagtagtaaagttttaagcagtacagtgg

caccaattttcctttctaaacactgctgattttctctgtacag

TACACCCCTGCAATTGATATTTGGAGCATAGGGTGCATATTTGCTGAAGTTCTCACTGGA

AAACCATTATTTCCTGGGATGAATGTCACACACCAACTAGATCTGATAACAAATGTCCTT

GGAACTCCATCATATGAAACCCTATCCCAG

gtatgtctacatattacccattcaccagtggacaaagatttcactcttttcctgtctttt

gttagtaccatactggtggtttctttaaagtgaagcatatatagacttggccacagatag

aaatccggctttctgtaaaatgcaatacaatgtgacagaactacttccccaacctggttc

acaaacgcacgacttctgtgtggttggttctataagtaaatcatttcctagataataacc

tgtttgagatttttgctacttctagcacatgccattgcacagtagtgctcagacaatgaa

tttcttaacatggctataacgactgctataacaactgcag

ATTCGTAATGAGAAGGCCAGGAGATATTTGACTGGCATGAAGAGGAAACATCCTATCCCC

TTTCCTAGTATTTTTTGTAACGCTGATCCTCAGGCTGTCCGTCTCCTGGAACGCTTACTC

GCATTTGATCCTAAAGATCGACCTACTGCTGAAGAG

gtattacttttcacaaaaaagagttcttgcttttatgatgactgattccgaaattgatga

gtacataagtgtaatattttatatatgtattctag

GCTTTAGCTGATCCATATTTTGAAGGATTTCCTAACTTGGAACATGAGCCTTCACCACAC

CCCTTTTCAAAACTTGAGTTTGAATTTGAGAGATGGAAGCTAACAAAGGATGGTGTAAGA

GATCTGATATATCGAGAG

gtagagaaccatcacttcagtgtatgttttaggctgactatatccgtctaacttaaaatt

ggattttttttccttttcttgaaagcaaacag

ATTTTGGAGTACCATCCACAGATGCTTCAGGATTATATCAGAGCTGGAGGACAGACTAGT

TTCGTCTATCCAAG

gttaggttgctttgccatctgctgccagcacaccagcactagcatagatgcttagcatat

acatgaaattgtatgctttgtatgcccatgtcacattttgttttcacacataaatcattg

ggacttgggaggtctaacctagtttaataccccttatcagcggtataatcttgctcataa

tttaatactaccacgttgcag

TGGGGTTGATGGTATGAGGCTGCAGTTTGTACATCTTGAGGAGAACCACCTGAGAGGAGA

AAGAGGCACTCCACTGCGGAGGCGACATGCATCTTTGCCAAG

gtacatgagtcattattaactttcagcagtttcaacttattgtaactgcacaaattctgg

tatggttctgattgcgcttgcttctgcactcctcttcatgtgacag

GGAAAGAGTCTGTGCACCAATGGGTATTGATAATCAAGACTGTAACAATGAGAGGAGGAC

AGCATCTTCTGCTGCCCAAATTACCATAAGATCACAACAAGAGGGGCTGACACATGCATA

TGTTTATCAAAATGGCACAGGCATTCCGGACTTCTGCTCAGGGTATTACTTGCAGAATGG

TAGTACCAGTGCTTCCAGTTGTGTTATCGAAGAGAATGAAGGCCCAAAG

gtacccccggcaccaatacaatggcactgcctaaattcctcccaggacctcataattacc

actgccaacttgtgtaaatgtgagagactgaccaatggcctatctgttttactcttgctg

ttttccactcttcag

GAGAATGGCGTCTCCGCGGAGAAGGTGGCCTATGAACTGTCAGAACGGCTTGCCAGGATC

TAGCCAGGACGCACCTTCTAAGGCAGCCATGATCCCGACGCAGCTCTAAATTTCAGCGCA

GTGGTTCCACCATGTAGTCTGATGGATCCCGGCAGCACAGAATAAACTCCACCAGGGCGG

AGGTGCTGCGAACTTCACACCAACCATTCACATTATCTGAAAACATGTAATAAATCTGAG

TTGAAAAATGTCGCGCCGATCTGCATCGGTATACATCCGGTGTAAATTATGTGGGTACCG

TAGTGTGATGTCGTAACAGAAATTGCAG

**>TaMPK24(6DS): TRIAE_CS42_6DS_TGACv1_543835_AA1744530.1**

AGTGACCGCAGCGACCTCCGGGCTCCGCGACTCGTCGGAGAGAAAGGCGAATCGGAATCC

CCCGCAGAGGGGGAGCGCCGCCCTGGAGAGCACCCGCGGTAGCAGCGAGCGACAGCAGCG

AGCGACAGCAGCGAGCGACAGCAGCGAGCAGCGCGCTGCCCAATGCTGCTCGGCGTCGCC

GGAGGGGCACCACACATGGCAGGGGAAGGGGGAAGCACCCGCGGCGGCGGCTCCGCCCCG

GCGACGGCGCGAGCTCGTCTCCCTCCTCCTCACTGGCCTCGCCGCCTCCACCCGCGTCTC

CAACGCCAGGCCTCCGCAGCCGCTCGACACGGCCCACACCTCATGGACGCCG

gtgagtccctcggaacatcagtcgccgccgtcgatctcagtaagcccgcatgtttcttgt

agaaacggctggatttggcttgcctagaaacgtccccgtcgtgctcgttgattcttccgt

acgacaaccccgatgccgcctactggttgaacacagtgtgtaagctcgctcgctcgaatt

tgacttgtcctgcgtgctcggggagttcacccggtccaaacctcccgccggcttttcacc

ggaaacttcggcagaaatttttcatacgaacttcagtggaaatgacggctgcatttcttg

atttcctcctgcccatttgacttgcgtgaacccgtggaataaataaagctttttcctgtg

ccgtctttctttggtttgtctaggaggtctcgcagcaagcaatttattcgggtaaaacat

cgcctcttattctactagctagcacatccatgtccatggcgtaccactgagacgagcaac

agaggcttttccttctgcaaagctcgcccaattgatagatggatgcggcagtggcgctgt

gcgattcaaggctttcaggttcatgttccaagaacctgcaacctgtgcgcttctaaccca

aactcatcttttatttctccaaccaggatcatgcaggtcttctcttatccacagtttcct

gttccctttttcatatctggagtagtggagtacaagttgatttgctgacgctttattcta

atctggctacgtggtggtgctattccttaggtggcttctggtgttggtaatgcgaatcat

acggcttaggttcctgctctgcccatgtgctggaatctatcattctacagaagcaacaat

tgttatagctgttcacatctgactatgcaaccccaaatgaactagtcttttttgcagttg

cttgctccaccaaggcgcatttttttttcattttccgtctatattatacgtctcatgggg

tcaaacacttgtttggtgccgggtgatttccctccgtactggtgtgattctctttagatt

aatttcttggatagtcttgcggaagctcccgtgtccttgtccttttcatgttgtcggcag

ggcctaattctgccgtcaacatgtcttggttgcaccatggaaccagattcttttcaagtc

tgaccttcataaggagatcacatcgttattactctggatagagatgctagttcattggtc

atgatatttacagggaaattactttagctggctgctgttggtataccataaggctttatt

tcctgctctgctcttctggccatgtgcatgaaactattcctgctgtagtcatgtgtccca

tgaggattattctgtagagtgacaattattacagcttttcatatctgactatgcaaattt

aagcggattagttcttggcagctgcttgcttcaggaaggtgcagttctcctattttttct

gtagagcctgtggtcttgtttttccctgcacgaggtcaaatttgtttgatagtgcctaac

ttcctccagtagttcatactagttagatttaccatttttattcgacattcccgcaaaaac

tctggtgtccttatcactttaatgttgtctgcag

GGACTAGCTCCACCAATAAGAACTCTCGGTTCCACCTTGGTCCATGGAGCCAGATCCTTC

TAAAATTGATTAAGTTTGACATTCAGAAAGGGACAGCATTTTTGTCTTGAAGCGGGTGTT

GGTTCCTGGCCACGATGGTTGCAGGGAAAAGAGCATAG

gtgtgtagcaattatctctcagttattttactcgcttccttgttgatttctctggtgact

ccatgagcatttagtaggctctctcttaaacaaggtacgacgcttgagcatctttctgca

aactttgtgtggttgtgtggcttttctgttgcaacctatcgagttcccttgtttcatggc

aagattttgctatttgtcagtacctgattgatgcagttcaaaaatctaaatacttatttt

ctttctctattagtcttttacatcggactcatttgttattgaacaggttttatgacgagt

cattcttgattattcttgcatccattctattttgacaggttttgacaattgggtttccat

tcctttaaattctgtgtgtaactttagtatctctgaactttttttcaaatgtctttagat

gctcaaaatcttttcttttgagctgtggactcgaatcccttgcttccagtgaaagaggaa

tgcttcacctttcacttcatcttagctatccattttaaacgaatgagtcaatcgaatctt

gatccattgcagcttaggtgacagtttagggaggacactgaacttcatgagaaatagtct

acgaatttgggcagctactgcactcttactacatggaagtgtgaactaccatgacaaggc

gcaggtgctattgcagcagatcacatgttgcatggcagcaaaggcaaagagttggtaact

ctatgctgtaactttaatgggtgactgttcctctgttagtgcgaaggatgtcgtgatctc

tggtcatctacatttgccgcgctgtcaccgcataatgtgaaggtgttaggatgatgaaag

ggagggttgctgaggtagctgacatatgtacagatgataatgtcatacattcagagagtt

ggatgtgaaagaggaaaaatgggatcttgagcgaaacatctgattgtcttttgtttgatg

agacaggaacacattgcctatattgctttggaccattcttactcaagttgtggcaaactt

tggtatccgaccaaacacccctcatatcttggccctttgttcgttatggatatcttagaa

aaatggaaaggcataacatgcctattttatctagagatagttggtaagcttactagataa

taaaatgccgcacttgcctaaatgaaccatgtggattttattatggataaatttatatct

atatctactagtattagtaagtgaggagaaatttgttctccccctcaaaatgagagaggg

gagaaatttatttcataggagtaatttatatggaacaaatactcactttttcttctcaat

gcttggttggattgaatcacaaggtatggattgtgacacgcagatcattaattaataatt

tttctttttttatatacgaacacccttcaatttcaccattcctttttttttttacttttt

accactcccacagttaacccgtattaacgtggtctttctagacatttccaccgtctttct

ctcttgctcttttccagccatttttttctcgtcatttctagtcctctctcttcccaaaat

ttctcttccttgctgtattacacaagtaagcctcgtcggctcactaccaccttgttcctc

gatgctgggtgttgtcacctattttccttgtgccacacactcgtttcctgtccggctccg

gtgtgcaagccaccccaccacagcagagtggaggtgcacacaagaaggcaccggagtgca

gctggacgcccagcgtgagatttcactaggcagcaaggaatcgactgagccatcaaggag

cgaagaggcgcgagcagcttccctatactctaattttaggactcacgaaatgacatatat

cttttaggacttgaaatctcacctgcaagtagatagcgttcatctccccaataaacgcat

gacaaatcttgctattgaaattcttgccacaaaagatctgtttccatctgtggtgtacaa

attgcgttttccgccaaaaaggttaaaaagatcagttattggtgcttctccagatgtagc

atccgcgtggagaagatgaactctccacgagagccacacttggcgttgcaaacgtgagcc

catgaatttgactactttagccctcctttccctttcccctacctctatcccaccctccca

gtccctagcacatcttctttgttcttcttcactcctcccatcctccattgctcaccatct

tgactccgtcttgtacccaatcagcacaaacgcccgattgcttcctatctgtttaatttg

cttcttgtggctgcaggcaccaccttttggccttggcgtgcagactttggatataatccc

gattagcttatcgcatcctcgctaattggtaacttggtgtgaactcattaatttccaagc

aatcatgtgctgtggagttttcttcttattcagttcagcagatatttactgaataattct

ccaatctagcacgtcatatacttgtatgtctagcttgaacttaacactgcctcattcgta

tctagttatttggattgatattttctaggtggctttgaaagttgacacggagctcatttc

ctcccattccgaacattgattatgatggtcatttgcttcaaattatgttgccatattgac

tattgttttgtgtgatggaaacagcctcttaccgaaatgtagggaaaggctgcgtacaat

agacccaaagtgatccccggaccctgcgcaagcgggagctacatgcaccgggctgccctt

tattgttgtgtgtgatggtagtaatacaaactaatccgttcaattattccatttctgtta

cttgaactttattggtttcgctgtgctgcttgctcaaagtttgtttgccgatctaatgct

tttctatcaatcttctgttagacttgtccattatcatcagaagagaataacaatcttccg

ctttactgtggtttagctcttgggaattgcttaagttcttctgtgacaattcttcttgat

cttttcttctccagtagtttgcggaagctattccgttgtaaatttgcttcttcatggtaa

catatatggcttatttatttctgaacttgctttctgattgatttatttttactagattaa

tcatttgataatccctcttaggtacatctgccctccaacttttgcattgcttttataatg

cgcctggttttaacaaatttggcgatacatgaattagtgttatttctcttggcttatcct

tttttttctgtgtaattgactacctagaattttccctcacacatgctctatatattgttt

ttgcttcagatgtttatagaattcaagattctaccaacattgtcgttcgtcattcttctg

ttcatgcattctgaaattgtgatcttttag

GTAACATTGGGCATGGAGTTCTTCACAGAATATGGGGAGGCAAGCCAGTATCAGATCGAA

GAGATCATTGGCAGGGGAAGCTTCGGAGTAGTTGCTGCTGCAGTAGATACCCAAACTGGG

GAGCGGGTTGCGATCAAGAAGATACATGATATGTTTGAGCATGCCTCAGATGGCACCCGC

ATTCTTTGGGAAATCAAGCTTCTTCGGCTTCTCCGCCACCCAAACATAGTTGAGATCAAA

CACATCCTGCTCCCCCCTGCCCGAAGGGAGTTTAGAGATATTTATGTTGTTTTTGAGCTC

ATGGAGTCGGACCTACAAAAAGTGATGCAAGTAAATGAAAACCTCACTGCAGGGCATCAC

CGGTATTTCTTGTATCAACTTCTTCATGCCCTCAAGTACATCCATGCAG

gttagttcagaaaccgtcggcgtatcattcaccttatcatcagcaatcacgtcttatcaa

gtgtgaatcctttgtacagtcttattttgaactctcatgaaaccctgtcttacttttctg

cgtatgatttatcttgtcgcag

CTAATGTATTTCATCGTGACTTAAAACCGAGCAACATACTTGTCAATTCGAACTGCAAAC

TAAAGATCTGTGACTTTGGGCTTGCACGAGCATCGCGTGATGATCCTCCCTTGGCTATAT

TTTGGACT

gtaaggcgtttgtcgctgtattaagtagttattttctatttctctcctcaagttacttac

ctagtgcactttgcaatttgaag

GACTATGTGGCTACAAGGTGGTACCGTGCTCCTGAATTATGTGGCTCATTTTTCTCCAAA

gtaagttggtgtctgtttgtttgaggccaagtttgcctatggttgacacacatcacacac

ctaaggttgggcaatttattgacgtaggtaggtctttggttcaagccacaccttcggcag

gattctttttccaccaataaggtcccacatgccatacacttacaaagtgtggcaagattc

ccttaggctccaatttcgttgaactaaccttaggcaagtttggcaaaaaaaattgtgcaa

agtgtgacattgttaattctagaacgaaacagccccttagtcttcaattctgagcgcact

atagtcaataatacagaaaggaagcctcttgcagtactaaatctgtacgccgttgttgct

tgttattgcagacttgtttccaatgcatgaatcattctaatgctttaaatgtgtatataa

tgttttttctttccgaagggcacataagtgctggtcattgtagtagaagaaaaatgccaa

gaaggcagatgtctcacactgccaagaaacgcaaaatagaaaacaaaagaactgtaaatg

tcctagactatgggctatggctcaaggcaaaggctcaggagggtgtccgtgttgtggcaa

ggccctcagcaccttggcaccaggcatcatccagagatgggcttcggtgagactggcgtc

caccaactccagtgcatcatcaaatacgaagtgttgtgcagcctccatagccgccacatt

gtgaggatgatgatcgagtgctctccgttggatctctgagcaccagctctctccacagca

acttcaaaccaggtggggaaattatcaccgggctgtgggcagagatgctggagcgagaag

tagcttaggaccgagtgccagatctgatgtgcggaggcgcaccctgttaacagatgcgag

tttgtcccgtcgtcctggtcgcacaacgggcattgattagggctgggtaggcctcgcctt

tggagacggtcaacacagttggctgctgctagccctgcaaagactttgcatttcagagga

gcccagctaccccagattggtgctgagagtggtttttagacaccgccggcgaaaagagcc

atgtaagcagatcgagggttgtactggcgggaggcggagatagtccagacgtactggtcg

tcgcgtgtcggcggagaggatagggctgccgacgatctccacaaggtggagtaactccat

aatgccctccacaatgagggcgcttgagatatcatggatccagtgtccttcagtgcatgc

ttcactcaccgtgcggcgattgatgacacgcatgctgactgcagcaggtaccatggggga

cagatcggccactgacatcctgttattcacctgtccaaccaaaatagggtacggcggccg

tcgcacaacatgaccttgctcgctgcacgaaggagactgtcgacatgccttccatcttaa

tagggagtgagcccatggcttggatgggccaatgcttggagccaaggccacctactcctc

agagcccagcccatcttcctcgtatctgcaatgcccagaccaccgagcttgaccaggcgg

cacacgtttcgccaagccacggagcagttgccacctttcgcctcacaatgaactcgccaa

aagaaattgcatgttttcttgtcgaatcgctcgcacactcccccagccgtcatagtgtgt

gtatgatgttatcttaccatattattaaaacaagttaacggccaccacatatcgctctta

aggggggttagaaattctcacattaatcggagtggaaagcgacatttcaacgccatttat

tttggtggtctcaaattgtaacggtgcagattaatctgataaatattaacgctattacag

ttgtataaggattacaactacatttgtactaacaagtgttgagagttatgcagcaaatac

agctgtgctttcaaatgtgaacatgcgctttagtatctaaccactcaggaaatatctttg

aaaaaacacctttagaaagtatccaaatgcagccgattatggcgaaaactgaagcagtaa

agtggcaccaattttcctttctaaacatgcaaattttgctgaacttatcttcttgcagtg

gacacttgtagaatcattatttgcaaagctcttgagaccccctccccccaaatttcctac

atcaccatgaacgctgctgattttctctgtacag

TACACCCCTGCAATTGATATTTGGAGCATAGGGTGCATATTTGCTGAAGTTCTCACTCGA

AAACCATTATTTCCTGGGACGAATGTCAAGCACCAACTAGATCTGATAACAGATGTCCTT

GGAACTCCATCACATGAAACCCTGTCCCAG

gtatgtctacatatcatccattcacgagtggacacagatttcactgttttcctgtttttt

gttagtaccatactggttgatttttactggtggtttctttaaaataagcatatatagact

tggccacagatagaattctggctttctgtaaaatgcaatacaatgtgacagaactacttc

cccaacctggctcacaaacgcacgacttctatgtggttggttctctaagtaaatcgtttc

ctagatagtaacctgtttgagatttttgctacttctagcacatgccattgcacagtagtg

ctcaggcaatgaattttaacatggctataacaactgcag

ATTCGTAATGAGAAGGCCAGGAGATATTTGACTGGCATGAAGAGGAAACATCCTATCCCC

TTTCCTAGTATGTTTTGTAACGCTGATCCTCAGGCTGTCCGTCTCCTAGAACGCTTACTC

GCATTTGATCCTAAAGATCGACCTACTGCTGAAGAG

gtatatttttgacaaaaaagagttcttgcttttatgatgactgattccgaaatcgatgag

tacataaatttaatattttatatatgtattctag

GCTTTAGCTGATCCATATTTTGAAGGATTTCCTAACTTGGAACATGAGCCTTCACCACAC

CCCTTTTCAAAACTTGAGTTCGAATTTGAGAGATGGAAGCTAACAAAGGATGGTATAAGA

GATCTGATATACCGAGAG

gtagagaaccatcacttcagtgtatgttttaggctgactatatccgtctaacttaaaatt

ggatttttccttttcttgaaagcaaacag

ATTTTGGAGTACCACCCACAGATGCTTCAGGATTATATCAGAGCTGGAGGACAGACTAGT

TTCGTCTATCCAAG

gttaggttgttttgccatctgctgccagcacaccagcactagtgtagatgcttagcatac

acatgaaattgtataatttgtatgcccatgtcacattttgttttcacacagaaatcttta

ggacttaggatgtctaacctagtttaataccccttatcagcggtataatcttgctcataa

tctaatactaccacgttgcag

TGGGGTTGATGATATGAGACTGCAGTTTGTACATCTTGAGGAGAACCACCTCAGAGGAGA

AAGAGGTACTCCACTGCGGAGGCGACATGCATCTTTACCAAG

gtacatgagtcattatatttttttttgaaaactggtacatgagtcattattaactttcag

cagtttcaacttattgtaactgcacaaattcttgtatggttctgattccgcttgcttctg

cactcttcttcatgtgacag

GGAAAGAGTCTGTGCACCAAAGGGCAGTGATAATCAAGACTGTAACAATGAGAGGAGGAG

GACAGCATCTTCTGCTGCCCAAACTACCATAAGATCACAACAAGAGGGGCTGACACATGC

ATATGTTTATCAAAATGGCACAAGCATTCCGAACTTCTGCTCAGGGTATTACTTGCAGAA

TGGTAGTACCAGTGCTTCCAGTTGTGTCATCGATGACCATGAAGGCCTGAAG

gtactccctgtaccaattacaatgacactgcctaaattcctcccaggatcttataattat

cagtgcacaatcactcttctcgtgacgacccgtgtttatgcgagagactgaccaatagcc

aatctgttttctgttgttttccactcttcag

GAGAACGGCGTCTCTGAGGAGGAGAAGGTGGCCTATGAACTGTCACAATGTTTTGCCAGG

ATTTAGCCAAGATGCACTCCTAAGCAACCATGATCCCGACGCAGCTCCAAATTTCAGCAG

TGGTTCCACCATGTAGTCTTATGGGTGGAGGTGCTGCGAATTTCACAGCAACCATTTACA

TTATCTGAAAACATGTAATAAATCTGAGTTGAAAAATGTTGTGTTGATCTGCATCGGTAT

ACATCCAGTGTAAATTGTGTGGGCACCGTACTGTAAGAGTCTAGAATCGAAGAACATCGT

GTGTGTGTGTGTGTGTGTGTGTGTGTGTGTGTGTGTGTGCGTGACAGAAATAGCAGACAT

TCAGTTTGTGAATTGGTCAACTGCTGGTGGGTTCAGTGGGAGCAGGTGAATGAATCTGTT

GCACGCAGGGCAAGCTCCCTGGCAGTGACGGACCAACAAGGGGCCCATGCTGAATCCAGT

**>TaMPK25(5AL) TRIAE_CS42_5AL_TGACv1_375843_AA1227850.X (based on AA1227850.1; )**

AACGCGGTGGTGCATGATGGGAAAGCAACCCAACCGCTTCCCAGATCGGGCAAATGACCC

ACGTTTTCGTCTACCGCCAGGAGCAGTGCGCCCTCGTGGCCTGTACGCATGCATGCCCGA

TCGACCCACGAGCCGCGGTGCATGACAGTGGTGCGGCGCGAGAGGCGTTGCCGGAAAGCC

TCTTCAAGCCGTAACCTTCCTCCCAATTCCCGTTCCTCCGGCTCAAGTACGGCCGATTCA

GATATGCTGCTTCGTCAG

gtgagcgggagtttctctccacctcctcccttttttcttctacttttgtgaagcatggca

agagcctctccacctcctccattttccctatttcccctcgtaataaaattttgtagtagg

agtctctcctggtgtctctgctaaagaaaattcgagtcagattcgaggtgggttcgcagc

gcccaaactttggattctggtgcattggggttggggagggtagctcccctaattcaaaaa

gaaaaaaaaaaccagcattagattagtttgtccaacaaattcagtatcacacacttatgt

taggcttgaggataaaatcctgggatggaaacaaagactagggccatggaaaaagtcaag

ctcacacaagaacctcgcagcattggagaaggcttaatgataaactcgtaaacatttttt

gctctgttttcttatgcttccattatccccaattatggaatgttaactctatgtttcctc

ctttcacag

TCGAAAAAGGAACAAAAAATGGCGAAGATGGTAGCTCCTCTGAATGGCAAGCAAAACCAT

GGGAAGCATTACTACACGATGGACCAGACGATGTTTGAGATCGACACCAAGTACGTGCCG

ATCAAGCCCATCGGAAGTGGATCTTATGGGGTAGTTTTCTCATTCATGAACAAGGATACG

AACGAGAAGGTCGCGATAAAAAAGATAAACAACGTATTTGATAGCCGTGAGGATGCACTG

AGGACGCTGCGCGAGATGAAGCTCCTCCGGCACTTGACTCATGAGAATGTCATTGCTTTG

AAGGATGTAATGATGCCAATACGGAGGAGGAGCTTCAAGGATGTCTACTTGGTGTCTGAA

CTCATGGACACCGATCTACAACATATCATCAACTCGTCTCAAACGCTTTCCAATGACCAC

TGCCAATATTTCCTTTTTCAG

gtatgtggcagtcacctttgttttcattccttctttttgcttgacacttgtccagtaata

tttcatatgttccccattctgtcataagttagtttattcacatagtccataccatgagtt

atttggatgtaatgcctatggctatgccttcatctaaccgctaataagctatgcctaatt

atttaattggacatatatcccattccagatgcctaggtggcggtgctgtcgttggcgtga

tcttttttagtcaagctatgatgtttgattgttacagaaaagattcttgtataacaatat

aatgtttttatacttctatatatactaatttgtagttagttcatggtgcatgtaaagttg

tcaagttccaacttgttaggttatgagagaatgatcaaataatttctagaagaagcaatt

gtcaatttcaatatctgtaagtgctattgtgagctcgagctcacaggcaccctttgctac

agtacaaacgaaaaaaatatttaaaaagtttaaaaaaatctgactttttttggcaacaaa

cattgatatatttttcacatgcgtgcaaattttcatgacaaaatgacattcatgcaggtc

tcggcaaaaaaaacaaaatcgatgctccaaaatgcttccaaaaacagtttttttggagca

tgattttgttttttttgctgacacctccacaaatgtcatttcatcacgaaaattggcacg

catgtagaaaaaacatcaaagtttcttgacaacaaatgtcagatttttttgaattttttt

actattttttcggattttactgttcatccgggatcactggtgcccgggatcaattcctcc

gcgtccgtactatagtagcttctatagctgctcgcggatagtggttgtgcgcttaggaat

gatgtgtcttaaaaacaaaacgacttaatacaaatcttatttgctcatgggtatttgcat

gaacatatatggtgtaagcctctgtaaataatttaaggaaagctaaatttatacacctag

acatgagcattttaatcgatacctacctgctcgacttgtcttacaaattacattactttg

taattattatgatatctttagtttattgccgaccattttacgatttctctaatatttgtc

aatcaattaatagtctaagatgccaccttatattgactgtag

CTGCTTCGAGGGCTGAAGTATCTTCATTCAGCGAGAATACTGCATAGAGACCTGAAACCA

GGAAACCTTCTGGTTAATGGAAACTGTGACTTGAAGATCTGTGACTTTGGTCTTGCTCGC

ACAAATAACACAAAAGGCCAGCTTATGACTGAATATGTTGGCACGCGCTGGTATAGAGCT

CCTGAGCTGCTGCTCAGCTGCAACAACTATGGCAACTCCATAGATGTCTGGTCTGTTGGC

TGCATCTTTGCTGAGCTACTTGGCCGCAAGTCGATCTTTCCAGGAACCGATTGCCTAAAT

CAGCTTAAGCTTATAGTCAATGTTCTTGGCACCATGAGCGGTGCTGACCTTAAGTTCATT

GAGAACAAGAAAACTCGCAAGTACATCAAATCCCTTCCATACACCATCGGCATTCCCCTC

ACTGGAATGTACCCACAAGCACACCCTCTTGCCACGATCTATTGCAGAAGATGTTAGTCT

TCGATCCTTCCAAAAGGATCAGTGTCACTGAGGCTCTGGAGCACCCCTATATGTCTACGC

TATATGATCACAATGCAAACCATCCTGCTCAGGTGCCCATCGATATCGATATAGATGAAA

AACTTAGCGTGGAGATGATCCGAGAAATGTTGTGGCGGGAGATGCTCCAGTACCCCTGAG

GCTGCCAGGATGGTGAATATGTGTCAAGCTGGAATGAACACATGCCCACACCAGGATCTT

CACATGTTCTTTTCTATATAAAGCTTACTGCGATTA

ttgcacctatcaagttatcacaatgaacttggtaaactactatgtaaata..........

**>TaMPK25(5BL) TRIAE_CS42_5BL_TGACv1_405765_AA1334990.1**

ACCGCCGTCTTCCTCCCAATTCCCACTCCTCCGGCTCAAGCCAAGTGCAGCGGTACCGCG

TACGGCCTATTCAGACCTGCTGCTTCGTCAG

gtgagtcaccgaccgagcacaccgagcgggagattctctccacctcctccgttttttcct

tctattttcgtgaagcgtagctctgctaattcggaaaagagcattggacaaattcagtat

cacacacttaagttagggcgaggataaaaacctgggatggaaacagagactatattacca

tggaaaaggtcaagctcacactagacctggcaacaccgttgagggcttaaaaaaagttgt

gaacattttttgggttatgcttccatcattctcaacaaaaaatctcaaaaaacgaggata

taccccggcctctgcatcttgacgatgcatacatccattttatcaattattcacaaagac

ctaacaaagtaatacatcagtcagcctgaagccaccatcttggcaacacttgtcgctact

cctatccacttgatgaaggggtgccgaatgtctgagccgaataccaaacagacatcgcat

caaagcctaacatctaaagccggatgccccaaccaagccacaatgtcgggactagggtca

cacaccgatcaggcgcactctcagaggccgtcgctgccgtcttccaacgatccatctcca

gagcaggtactggcgcaccgaccttgcctggctgtcgtcgatgccaccacagcgtcagag

aacgccaccatgcatcctgcatttagcaccgctcttgctcttgtcccctccagccagcac

ttgctccaaaacgacgcccccaggagggagaacgacaccgaagacgtcgtcatcgttcga

tccggtagacctagatatacggtttcccccggaacatcccgatcgagttgacgtgacctg

caacgacaatgcctcgagaagtaaacgatgtcagagacaccgccaccatccgtcaagacc

gcagtcaggcgcgggtttcaccagaaaccgccccgtcccaatctcgcggctggatagaac

cacgcggagctttgccataaatacgtatgccgccaatggtactgcggcggagatcctcca

tgccttcacgggagatctcatcgtgtcgccgactatccacgtgaagaatcgatgatggat

ctgggtgggatccagatccgcgatcaccgccatgccaaccatcaccaacaccaccagcca

tgggggcgtcagttgccgccgcacggctagggatccaccctagccacggcgcaaagcccc

cagtccgaggtgccccagccgcctcgaggggttgcggccgcctcctcctgccttagcgcc

ttggcaccgggcgccgccgccaccgcggccaacgccgacagcagcggcgatagggtcttt

cgctgtgggttgtggcgccagcggcgccgggatctgatccccctggcatcgctctagggt

ggcggcgggagggggcggcagggtgagggcgaggggtgattctacaatttcctatcaaaa

tcaattgatagcacgaaagactctacattccatcactctcagttatggaattgtaactct

atgtttccttgtcttggtag

AAACAAAAACATAAAATGGCGAAGATGATAGATCCTCCGAAGGGCAAGGGAAACAGTGGG

AAGCATTACTACATAATGTGCGGGACGATGTTTGAGATCGACACCAAGTACGTGCCGATC

AAGCCCATCGGAAGAGGATCTTACGGGGTAGTTTGCTCGTCCATGAACAAGGATACGAAC

GAGAAGGTTGCGATAAAAAAGATTAACAATATCTTTGACAACCGTGAGGATGCACTCAGG

ACGCTGCGCGAGCTGAAGCTCCTTCGGCACTTGAGTCACGAGAATGTCATTGCTTTGAAG

GATGTAATGATGCCAATACAGAGGAGGAACTTCAAGGATGTCTACTTGGTGTCCGAACTA

ATGGACACGGATCTGCAGAAGATCATCAGCTCGTGTATAACGCTTTCCAATGACCACTGC

CAATATTTCCTATTTCAG

gtatgcgatagtcacctcttctttttcattctttttttttttgcttgacacttgtccagt

aatatttcatatgttctccattatgtcataacttagttcattcacacctcatgttattat

ccggaagtaatgcctttgggcttgcttagctagacatagtcaatcccatgagttatttgg

atgtaatgcccatggctattcttcctcccgacatgaatcaaaagatctggtaatgttaca

accttcatctaagctatgcctgcctatttaactggacatatattctattctagacgccta

ggtgctgcgattggtgagatcttgtttaatcgagtcatgatgtttggttgttccaggaaa

ggttcttgtatgataatataatgtttttatagttgtataccagtttgcagttagtccatg

gtacatgttaagttgccaagttccaacttgttaagttgccagagaatgatcagctaattt

ctggaagaagcaccggtcaatttcagtatctgtaactgtaatggcactatatatagtaga

ttctatagctgcacccggatagtagttgtgtccttaggaatgataggtcttaaaacaaaa

caacttgatacaaaacttattttgcacatgggtatttgcatgaacatatatggcgtaagc

ctgtgtgtaaataataagaaaagttacatgtatatatatacacctggactcgagcatttt

aaccgatacttacctgctcgacttgtcttacggattactttactttgtaattactgtgat

atctgtagtttttctgccgaccattttatgatttctctaatatttgtgaatcaatcaata

gtctaagctgccgccttttattgactgtag

CTGCTCCGAGGGCTGAAGTATCTTCATTCAGCAGGGATACTGCATAGAGACCTGAAACCA

GAAAACCTCTTGGTTAATGCAAACTGTGACCTGAAGATCTGTGACTTTGGTCTTGCTCGC

ACAATTAACGCTAAAGTTCAGTCTATGACTGGATACGTTGTCACCCGCTGGTATAGAGCT

CCCGAGCTGCTGCTCGGCTGCGACAACTATGGCACCTCCATAGATGACTGGTCAGTTGGC

TGCATCTTTGCTGAGCTACTTGGCCGCAAGTCGATCTTCCCAGGAACCGATTGCCTAAAT

CAGCTTAAGCTTATAGTCAATGTTCTTGGCACCATGAGCGATGATGACCTTGAGTTCATT

GACAACATGAAAGCTCGCAAGTACATCAAATCCCTTTCGTACGCCGACGGGATTCCCCTC

ACTAGCATGTACCCACAAGCGGACCCTCTCGCCATTGATCTGTTGCAGAAGATGCTGGTC

TTCGATCCTTCCAATAGGATCAGTGTCACTGAGGCTCTGGAGCACCCCTATATGTCTACG

CTGTATGATCCCGACGCAAACCCTCCTGCTCAGGCGCCCATCGATCTCGATATAGATGAA

AAACTCGGCGTGGACATGATCCGAGAAATGTTGTGGCAGGAGATGCTCCAGTACCCCTGA

GGCTGCCATGGTGGTGAATATGTGACGAGCAGGAATGAACACGTGAAGAGCTATTTGCCA

CACACCCACAACATGATCTTCACATGTCGTTTCAGTGTAAAGCTTACTGCGATTATCGCA

CCTATCAAGTTATCACAATGAACGTCTGGTGACCTGTTATGTAAATAGGTGCGCAATAAG

ACCGCCTATGGACTACTTGTAGTATATAAATATGTTGTAGAACTAGAAGTAGAGTTCAGT

TAATAAGGATTCAGTTATGAAGACGCTTGTGTAAATTGCAATCCTCTTTTTTTTTACCTG

ATTTCAAATTGAAATCTTAGAGGCTGCTCTATGTATGGAGATCTCGACTTTCAAGTGGCC

TTGGTTTTCGTCTGTTTGCTTCAGAGTGGTTACCTTGAAATCTGTCTTGTTTCTTCAAGA

GCTAGAAACTGCAGTGTGTATGGTGATTTCTTTTTGGCTGAAAGTGTATAAACTCTGAAG

CTCTCTGTAATTTGCAAATTCAGTACCATCACGTTGATGTAATGGTGGTCAAAGTTATTA

GTCCTATTCCCTGGGCAAACTACTTCCGGGAAGAATTCCGATCTCCCACTGGAGTAATCA

ATATGCGGTCACAACTTGTCGGAATAAATAATTGAGCAATTAGCAAGCAATGGTATGAGG

AAATATTACCGACACTACTATCCCATAAGCCCATAATAACTCTAGCAGATGCGTCGTATA

TGTCTGATCGCCATAATAACTACTAGTATGGCGA

**>TaMPK25(5DL) :TGACv1:TGACv1_scaffold_433177_5DL:99000:100916:1**

ATGGCGAAGATGGTAGATCCTCCGAAGGGCAAGGGAAACCATGGGAAGCATTACTAC

ATAATGTGCGAGACGATGTTTGAGATCGACACCAAGTACGTGCCGATCAAGCCCATCGGA

AAGAGGAGCTTACGGTGTAGTTTGCTCATCCATGAACAAGGATACGAACGAGAAGGTCGT

GATAAAAAAGATTAACAATGTCTTTGACACAACCACGAGGATGCACTGAGGACGCTGCGC

GAGCTGAAGCTCCTTCGGCACTTGAGTCACGAGAATGTCATTGCTTTGAAGGATGTAATG

ATGCCAATACAAAGGAGGAACTTCAAGGATGTCTACCTGGTGGCCGGACTCATGGACACG

GATCTGCAGAAGATCATCAACTCGTCTATAACGCTTTCCAATGACCACTGCCAATATTTG

CTTTTTCAGGTATGCGACAGTCACCTCTTCTTTTTCACTCTTTCTTTTTGCTTGACACTT

GCCCAGTAATATTTCATATGTTCTTCATTATGTCACAAGTTAGTTCATTCACACCTCATG

TTATATCTGTAAGTAATGTCTTTGGGCTTGCTTCGCTAGACATAGTCAATCACATTAGTT

ATTTGGATGTAATGCCCATGGCCATTCTTCCTCCCGACACGAATCAAAAGGTCTGGTAAT

GTTACAGCCTTCATCTAAGCTATGCCTAACTATTTAACTGGACATATATTCTATTCGAGA

TGCCTAGGTGTTGCCATTGGTGAGATCTTGTTTAATCGAGTTATGATGTTTGGTTGTTCC

AGGAAAGGTTTTTGTGTGATAAAATAATGTTTTTATAGTTGTATACCAATTTGCAGTTAG

TCCATGGTACATGTTAAGTTGTCAAGTTCCAAGTTGTTAAGTTGCCAGAGAATGATCAGC

TAATTTCTGGAAGAAGGACTGTTTCAATATCTGCGATGGCACTATATATAGTAGATTATA

TAGCTGCACCCGGATAGTAGTTGTGTGCTTAGGAATGATAGGTCTTAAAACCAAAACAAC

TTGATACAAAACTTATTTTGCACATGGGTATTTGCATGAACATATATGGCGTAAGCCTGT

GTGTAAATAATAAGAAAAGTTACATGTATATATACACCTGGACTTGAGCATTTTAACCGA

TACTTCCTGCTCGACTTGTCTTACGTATTACTATACTTTGTAATTACTATGATATCTATA

GTTTTTCTGCCGACCATTTTATGTTTTCTCTAATATTTGTCAATCTATCAATAGTCTAAG

ACGCCGCCTTTTATTGACTGTAGCTGCTCCGAGGGCTGAAGTATCTTCATTCAGCAGGGA

TACTGCATAGAGACCTGAAACCAGGAAACCTTCTGGTTAATGGAAACTTGGTCTTGCTCG

CACAAATAACGCCAAAGGTCAGTTTATGACTGAATATGTTGTCACCCGCTGGTATAGAGC

TCCGGAGCTCCTTCTCAGCTGCGACAACTATGGCACCTCCATAGATGTCTGGTCTGTTGG

CTGCATCCTTGCTGAGCTACTTGGCCGCAAGTCGATCTTTCCAGGAACTGATTGCCTAAA

TCAGCTTAAGCTTATAGTCAATGTTCTTGGCACCATGAGCGATGATGACCTGGAGTTCAT

TGGCAACATGAAAGCTCGCAAGTACATCAAATCCCTTTCATACACCGCCGGGATTCCCCT

CACTAGAATGTACCCACAAGCGCACCCTCTCGCCATTGATCTATTGCAGAAGATGCTGGT

CTTCGATCCTTCCAAAAGGATCAGTGTCACTGAGGCTCTGGAGCACCCCTATATGTCTGT

GTTGTATGATCCCAGCGCAAACCCTCCTGCTCAGGCACCCATCGATCTCGATATAGATGA

AAACTCGGCGTGGAGATGATCCGAGAAATGTTGTGGCAGGAGATGCTCCAGTACCCTTGA

**>HvMPK3 : MLOC_17814.3**

CTGCGCGCACCAGCACAGCACAGCAGCGGCGTGCGTGCGCGCGCGCCCGCTCTCGCTCGC

TTTCTCGCTACCTTCCTTCCTCGGCTGGGCCGAGAGCATTAGCGGAGCCGCCCTTCTCTA

TTTATAATTCTCCCCTCTTCCACCCCTACAAGAATCCAGAGCCAGGACAAAGTCAATCAT

CAGCTCGGCCTCATCCCCGTTGCGCTTGGCCTGTGATTGGGTTTTGGGAGGAGAGGAGAG

GAGAGGAGAGGAGGGGAGGGGAGGGGATTCTTGGCTGATCTCGGTTTGGTTCTTGGCTGT

TTGAGAATGGACGGCGCTCCGGTGGCCGAGTTCCGGCCGACGATGACGCACGGCGGCCGG

TTCCTCCTCTACAACATATTCGGCAACCAGTTCGAGATCACGGCCAAGTACCAGCCGCCG

ATCATGCCCATCGGCCGCGGCGCCTACGGGATCGTCTG

gtacgttcgccccatcttttcctcctcaatcaccaccggtttcttgccaggtttgttctt

caagccgttgctgccctgatcggaaaacagagggggattagttcgggccatggggaaaga

taggattaggccaattcactcctttctgtaaacaacaacactagtttaatactgtatgtg

ttgatctgtcggtgctctgtggttggtggttgtttaaccaatctggtcctgaaattgcac

ttgtggttgcgtgggcacag

CTCGGTGATGAACTTCGAGACGAGGGAGATGGTGGCAATCAAGAAGATCGCAAACGCCTT

CGACAACAACATGGACGCCAAGCGCACGCTCCGGGAAATCAAACTCCTCAAGCACCTCGA

CCACGAGAAC

gtaagcaacatctacctcttccgcttcagatgtgcgtatgcatctcactgaattcgacaa

ttcagggctaacctgttacgctgtattgatggacaccacacgcag

ATAGTAGGCCTCCGAGACGTGATCCCGCCGGCGATCCCGCAGTCCTTCAACGACGTCTAC

ATCGCCACTGAGCTCATGGACACGGACCTTCACCACATCATCCGTTCCAACCAAGAACTC

TCAGAAGAACACTGCCAG

gtactactccatactctgaatgaagagcgatttttttcatatttcgtgacgatttgatcc

gtatattcatgcgccgtggtgttcttgggtggcttccag

TACTTCCTGTACCAGCTGCTGCGCGGTCTCAAGTACATCCACTCGGCGAACGTGATCCAT

CGCGACCTGAAGCCGAGCAACCTGCTGCTGAACGCCAACTGCGACCTCAAGATCTGCGAC

TTCGGCCTGGCGCGGCCGTCGTCGGAGAGCGACATGATGACGGAGTACGTGGTCACGCGG

TGGTACCGGGCCCCGGAGCTGCTGCTCAACTCCACCGACTACTCCGCCGCCATCGACGTC

TGGTCGGTCGGCTGCATCTTCATGGAGCTCATCAACCGCGCACCGCTCTTCCCGGGGAGG

GACCACATGCACCAGATGCGGCTCATCACGGAGGTGATCGGCACCCCCACCGACGACGAC

CTGGGCTTCATCCGGAACGAGGACGCCAGGAGATACATGAGGCACCTGCCGCAGTTCCCT

CGCCGGCCGTTCCCGGGCCAGTTCCCCAAGGTGCAGCCAGCCGCGCTCGACCTCATAGAG

CGGATGCTCACCTTCAACCCGCTTCAGAGGATCACAG

gtgcgtgcgtcccacgtccgcgtcactctgctttgatcttcacgtgtcaatttcgtagcg

ccgtgtttttttgactggctttgtacggccagtgttggggtcagtgtcagtgcttaaacc

atccttgtgctggttatgtctacaatagcgtgagtagatgcaatctgccgccacttcgac

ccctatttttaagtttactgcatgtggacatgtgctccctttttaggcaaaaaccatgtg

tcccgcccacttgattaagaaaagatctttgcctatctttggagctatagtatctgctgt

tgacggcaaggactatgcgtgtagtgcagaatcactctctttttagaagaaaacgtatct

gtttttggcaaaagatgtgaatgggagagatctgctgcagctactgtcaaaggctagatt

cctgcacctgtttcacaacagcaataataggagtagctttttgagacgtcggccagggaa

tagtagggaaggctaggaagtagtagtagattttatttttctacagttatacttgatgtc

tgagtgatgtgcattctcactgaaactgctcttgtcatgtagtagaaagttatgtttcat

cagttaattggtggctgactgatgtggattctcactgaaactgctcttgtcacgtagaaa

gttatgtttcatcagttaattggtggctgactgatgtggattcttactgaaattgctctt

gtgatgcag

TTGAAGAGGCGCTGGAGCACCCGTACCTAGAGCGGCTTCACGACGTTGCCGACGAGCCCA

TATGCACGGACCCCTTCTCCTTCGACTTCGAGCAGCACCCTCTGACGGAAGACCAGATGA

AGCAGCTCATATTCAACGAAGCTCTGGAGTTGAACCCCAACTTCCGATACTAG

AAGATTC

ATTCCTAGTTACCCTCAATAGTACGCTTTCGGTGTAAAACTGTCTGTAAATAGGAGTAAG

ATCGAAGAACGGGGTTAAAAAAAACAGGGTAGATTGTGCACTGCATTTGTTTGTTTGTTC

CGTGTAAGTTGTTGCTGCTGCTTCAGACCTCTGTCCCTCGGCTGTGCGAATAAAATGTTT

GGAGTATGATTTGAGAGCAAATCATTCTCTAGTTTATGAAGAAATGGATTTTACT

Acactcaggtggatgctcacctgatattgagaatgcagttt....................

**>HvMPK4 : MLOC_5653.1**

TTCTTCTTCCTCTTTTCCGCAAACGTCGCCCCACGCGCGCCGTCTCCGTCCTCGCGTCCG

CTCGCTCCCATCCTCATTCCCCAGTCGCCGCCCAAGTCAAAAAGAGGGGGAAGCATCTCC

ACCAGAGTCCCCAGCCCATCAACGACTAGTCGCTGCGGGGCACCAACCCTAGAGCCCAAC

CCTACTCTCCCCCCCTCTCTGGCTCCTCCCCCTCGCGAGCGGCGACGAAGAAGCCATGGA

CACCTCCGGCGGCGGCGGCGGCGCCGCGGGCGGGGCCGCGCAGATCCAGGGGATGGCGAC

GCACGGCGGCCGCTACGTGCTCTACAACGTCTACGGCAACCTCTTCGAGGTCGCCTCCAA

GTACGCCCCTCCCATCCGCCCCATCGGCCGAGGCGCCTACGGCATTGTCTG

gtgcgttcccccctcccgaatcgccgattacatgcgtgtcgctgcagttaatagcgaatc

cgagctgctatctatgccatgtgtggaatgttccgtgccgccgtgcggcggtgtctgtcc

agatttggcacacatgcgggtgtgacgttttaggcataaagttatgtgaggaagtgagtc

accccacaaggagggttgcagtagaagtgcttatatttgcctattctgttccctactttt

gcag

CGCGGCTGTTAGCTCGGATACAGGAGAGGAGGTTGCGATCAAGAAGATTGGAAATGCGTT

TGACAACCACATCGACGCCAAACGGACGCTTAGAGAAATAAAGCTTCTTCGCCACATGGA

CCATGAGAAT

gtaagagttgtctgttttaatttgtaatggacatgtgtggtgattgccgagtgtactttc

cttaccactatagctgtttactgtgtcctttgcagttctatacataagatagcttaccac

tgtgcttgagcaacacatgttcaattgtgaacatcgttcttattagttcccttttttaat

accgaattttggcagaggtaagcggtcttgtacttctggttatagagaattgctatgcat

aaggagagaactactttaggtgcaatatgtagaatgtaaatctcagctacccaggaactg

acccttgttggttgattatgcacatgatgaggttttgtggtaacactcaagcttcttact

aaagaagcatatagaagtatagggatcagctgacatcacagccatagggtcgagtgtgca

ctgataattttctctgaagttggcttctactagtggttttatttcctttgtctacgtttc

tagaagtggtatgcctgagtgggttcttattcttatcattacagttttactgttgacggg

ctttgttgtccactcgtgctcacactgtcaggcattcctatgatgggtttattctgatag

tggtttcaaaattggatcacag

ATTCTTGCCATGAAGGATTTAATACGCCCCCCAAGAAGAGATGATTTTAAGGATGTGTAC

ATTGTTACTGAGTTGATGGACACTGATCTCCATCAGATCATTCGCTCAAATCAATCATTG

ACTGATGACCATTGCCAG

gtttgttgcctcctgcttgtgccagattcttatgtatttgctgcaattgtgtagttgaaa

caaatcttgttgtgactagagtagatatcaattgacatgtactcaaagttgtgttttgtg

ccttctttagttgtgtttggttcaactttttttaccaacttctgctttgaaaagctaaaa

gctaaccaaagggttaaatgttcaaagcagcttttgagaatctgtagcttcttcctagtg

taaatttcaaagctggggtaccccaacttttcagctcccagcttttccacagcagctttt

ttagaatctgcagctcaaccaaacagtcttagcttaggtttatcagctttgtcctccaga

aatcattagttacatgttgtgttctgtaccttgtttagttcttagcttaggtttatgagc

tttgtcttcaagcaatcataagttacatgttttgcattgcag

TACTTCTTGTATCAATTGCTTCGAGGGCTAAAATATGTGCACTCAGCAAATGTCTTGCAC

CGTGATCTGAAGCCAAGCAATTTGTTCCTAAACGCAAATTGCGATCTCAAGATTGCTGAC

TTTGGGCTTGCAAGGACCACTTCAGAAACTGATCTCATGACAGAGTATGTGGTCACTCGT

TGGTACCGAGCGCCAGAGTTGCTGTTGAACTGTTCACAATATACTGCTGCTATTGATGTC

TGGTCAGTTGGGTGCATACTTGGTGAAATTATTACTCGTCAACCCCTGTTTCCTGGAAGA

GATTACATCCAACAGTTAAAGTTAATCACCGAG

gtaggtttgttcaactcgtcgcctgccttttttggtatccacattgacaagtcatgtgtt

gtctatttggaagctgtcaatcaatgaaaatgggtctgtttatgaacttctttaatagaa

tttgacaattgttctatttggaacaataggataatgggatctttcttggcacatctaact

gtattgttagacctgtttcgcatcttgtgtttgaatagaagtttttacttttacacaaac

actggattagtgaatgcattttgcatctggtagcatgtaggtacggcaacattttactgt

atcatcagatatgggatccacatttggattattaacttcgtgcaagtttctgtggatgtc

tcttctcttcaaagtatacattttactatatatactggttggtttctgcgcaatacattc

ttagcttcaaatcaaccatttttagtaccatcgaaggcatttttgcttcttttttaatga

tgtctcttgaacctgttgtatcaaatattaaccagtcctttccggcattctgcttcaaaa

g

CTGATAGGATCACCAGATGATTCAAGCCTGGGATTTCTTCGGAGTGATAATGCACGAAGA

TACATGAAGCAATTACCACAGTACCCAAGGCAGGACTTCCGCCTGCGCTTCCGCAACATG

TCTGATGGTGCAGTTGATCTGTTAGAGAGGATGCTGGTGTTTGACCCAAGCAGACGCATT

ACTG

gtacatgggcttgaactaactacttaagttagttttgttcgcattactattgctcaaaat

catttttcatgcag

TTGATGAGGCTCTGCATCACCCATACTTGGCTTCTCTTCATGACATCAATGAAGAACCCA

CTTGCCCGGCACCTTTCAGCTTTGATTTTGAGCAACCATCTTTTACAGAAGAACATATGA

AAGAGCTCATCTGGAGGGAAACTTTAGCATTTAACCCTGATCCGCCCTACTAAGAGCCAA

G

gtttgtattgtatacgtctggttgaaaatattttgtttcttaaaagcaattatctaaaag

aatacaatgtgtcagacgactaatctagatcctggaatcctgaggaggttcatgcagaca

gacttgctattgatatttatctgcaatatttccgaggttgaataatttagatcttgaaaa

gtgtgaccttttggaaaccaaatatctatctggatcagtgagactttaatttgagaacat

accctgtttaaaattctactccctccgtcccataatataagacgttattacatccaatat

actcacatattggatgtaataacgtatattattataggacggagggagtatttgtaagtc

aagtcttgtttttccacgtttcttatgtgtgtgccaattctctgttatcttggttcgttc

tgaatttcatttcatttggttcaagattgtttccaggcattcaagttaattcatgttgtt

ttccttgggagcacag

ACCAAATTACCAGCTGAGGGCATTGAAGATCTACCTCTAGCTCTAGTGAAGCCGATATTT

CCATGTCTTGTGCATCTATTTATTTTATGCTCGCTTATTGGGCGAATGGGCATGGATTAT

TTGTTGCTGTAACTATTTCTTTTGTGGCCTTTCTGAAGAAACGACATTTGTATGAGATGG

CTTGTATTATACCGGTTGATTGAATAAACTGGTCTTGTGTATCTAAAGCTTGTAATTTTG

TGTACACTTATCTAAACATCCCATGTATATCTGAGTTAACATATTCCCTTTTATTATCTC

AAGCATGTAATTTGGCATTTTGTGATCGGTCGTATCGAAGTTGCCG

>HvMPK6partial : ASM32608v1:7:33935418:33937780:-1

NNNNNNNNNNNNNNNNNNNNNNNNNNNNNNNNNNNNNNNNNNNNNNNNNNNNNNNNNNNN

NNNNNNNNNNNNNNNNNNNNNNNNNNNNNNATTTTGGATGGGCTCCTAATGAGCTTATTT

TCACTATTTTTGTTTGTAGTTTTCCAGTAATTCTAGATACATGTTTGAAATTTTGGGTCT

TTTTTTGTTTAAACCGTCTATCTCCTTCGCTTGTAGTCATTTATCATTCAATTAGTGAAG

CATAAATTCATTCGATCTCCCAATATTAATTATTAATATTAATCAAATTAGCATCTTTCA

CAGACCAACACTAGTCTTTCCTGTGCCCTTTGTCCTCACTCATGTGCACCCGTGGTCAAC

TTCCTGGTCGGTCACCCATCCTAGAATTACTCCAAGCCAAGCACACTTAACTTTGGAGTT

CTTTTCGAATGGGCTCCTGGAAAAAAAGGAATTCCTTGTTGATATGGGTAGTCTTATCAT

TCATATTAAGTCAGGCTCTCACAATGATTCCCTAAATAGAAGTACTTTCTTGATATCAGA

GTGGTCTCTTTTGTTACTAGCTAATATTAAATCATGTTCTAGTTTCCCAAATACCACCGT

ATAATCCTTCGTGGAAAAGTTCCATGGAAAAACTGTTTACCATAATATGCCTGTATTGTC

AGGGCCTACTCGTGTTTCTAGCATAAAAAAGTTGATGCTAGATCACACATGAGAAATTAG

TGGATTTCTGGAATGGTCTCACCTATTTCTCTTATGTCGATCTTGTTCTTTCTAGTTTCT

TTTCAATCTGCAAACTTCTGTCTTGTTCTGAGAAATGGTCCTTCATCTGATATGGATGTT

TTGTGTAATTGTTCCTTTTTTGTGCAGTATTTCCTTTATCAGATCCTTCGTGGCTTGAAG

TATATACATTCAGCAAATGTTCTCCACCGAGACTTGAAGCCTAGCAATCTTCTTTTGAAT

GCAAACTGTGACCTAAAAATTTGTGATTTTGGGCTTGCTCGTACCACCTCAGAAACGGAT

TTTATGACTGAGTATGTTGTGACAAGATGGTACAGGGCACCAGAGCTTTTGTTGAACTCC

TCCGAGTACACTGCAGCAATTGATGTGTGGTCTGTGGGCTGTATATTTATGGAACTAATG

GATCGGAAACCTTTGTTTCCGGGAAGAGACCATGTCCATCAGCTACGTCTACTAATGGAG

GTTAGACAGTACCCTATGCAACTTCTATATCGAATTGAAATCATGTTGTGCAGTCCTGTT

TTAGCATAATAGTAGTTTTAGGGCTGTCATGTTGAAGTAGCTAGTTTGATCATAGTTTGT

TCTCGAAGTATTAAGCACTTAATAAGTACTCCGTCTGTTCCTAAATATAGTCTTTTTAGA

GATTCCACTACGGACTACATTCAGATGTATATAGACATACTTTAGANNNNNNNNNNACTT

ATTTTGCTCCGTATGTAGTCCGTAGTGGAATCGCTAAAAAGACTTATATTTAGGAACGGA

GGGAGTACTTTTCTGGTGCACATTAATTCATTTTCAATTTCCTCATGGTATTTCCTCGTT

GCAGCTCATTGGAACACCAAATGAGGCTGATTTGGATTTTGTAAATGAAAACGCAAGAAG

ATATATCCGCCAACTTCCCCGTCATGCAAGGCAATCATTATCTGAGAAGTTTCCACATGT

TCACCCTTCAGCAATTGACTTGGTTGAAAAGATGCTGACTTTCGATCCTAGACAGAGAAT

AACAGGCAAGTTTTGGCACGTGTTTAGTGATTTAACCATCCGCACCCATTTGCTGATTTA

TTGTTGCTTCAAGACAGACGATCTTATCTTCCAAACAGCAAGTTAAAAAGAGCCATCCTA

GTGTTTCGTTCTATATATCATTAGCTAACATCACTTCATCCATTCAAATGAAAAAAAGAG

TAAGTAACTAAATTGGGAAGATCACACAGTTAAATACATGGAGTATCTGGGCTGGATTTA

CAGCCCAAGTGGGTAGGAATGTGTCCATACTCTCTGTAGGTATGCAAACTGCATTAGCTT

GAAGTGGGAATGCTAAAGTAATCATTGTTGCCTGTTGGGTGGTGTTTCTATAGTTGATAA

TCCATAGTTTCAACTGCAAAATATCCGTCTCCAATATTCATCCTTTATTTTTTTTGGTCA

GTTACTGATTGTTCCTTTATTTTTTTTGGTCAGTTGAAGGCGCACTTGCGCATCCTTACT

TGGCATCGCTGCATGACATAAGTGATGAGCCAGTCTGCACGATGCCCTTTAGCTTCGACT

TTGAGCAGCATGCATTGTCGGAAGAACAAATGAAGGATCTAATCCACCAAGAGGGCATCG

CGTTCAACCCTGATTACCAGTAG

**>HvMPK7 : MLOC_74277.1**

CAGCTGAAAACTAAACCAATCAATAGAAGTGTGTATTATTTTTACAAGTGTATTTGGCAA

AAAAAAAGACATCCGAAGCAGGGCCTAAACCCGCCCAACTCGCATCACGCGGGTTCTCAC

AAAACTTTGCTACCACACGCAGCCTAAACGTAAGCACTAACAACTGACCTGGCGTAAGCG

TAACCACTAACTAATCACCATGCATGCTAAACCCGCCCAACTCGCATCACGCGGCGAAAC

CATCAGCCATGGCAGCCCCCCGCGTCGCTCGTCCGTACCGTACGCCCCGCCTGCCTGACG

GGTCAACGGGGGGAGGAAAGGCGCGCGCGCCGGCGAAGCAACCCAACCGCTGCACCGATC

GGGCAAACGGCCCACGTTCCCCTCTCCCGCCCGAACCCCGGCGCGCCCTCGTGGCCCCGC

CCGACGACGTCCACGCGCCCCGGATGTTTCACGAGCCGTCGCTGCAGCGCCGCACGCTAC

GCGGGGGGCGTTCACGTGCGAGCCGTTGCCGGAAAGCCGGCGGGCGGCTTACCCGGACCG

GAAGGATGGACGATCGGTCTCGATCCCGGTCCCGAGGAGCGGCAATGGCCATGTGTCCCG

AAGGGGCCGGCCATGTGGGCGGGCCGCCGCGGGCATATATAGGGCGGCCACAGGATCTCC

TTCTCATCATCTTCCTCGGGCTGCCGCGTGTTCCATCGCTAGCTGCCTGCCAGTGCCAGT

GCCCCTTCGCTGTCCCTGAGCGGCACCGGTCGCCATCTTCCTCCTCATCACAATCCCACC

ACCACCACTCCCCCAAGTCCAAGCCCCAGTGGAGCTACGTACGTACGTACGTTCCTTCCC

CCGGAATAAAAAAGCCTTCCTGCTCGGTCGCTCTCCGCCTTTGCCTCTGCCTTCGTATCC

CTCATCCCCTCCCTCCCCCGCCGCTCCCGGACGTGCTCTGCTTCTCCAGATCCGCCGCCG

ACGACGCCGCCGCTGCTGCGTCCCGAG

gtgagcgatccatcccgcccgcgcacggggcggatgagcttctctcctccctcattttct

tttcttttccttttcctcctgcggccttcgtgaggcgtagcggcggcgaattgggccgcc

tccggttccggtcgccgcgggcgggaggagctgcgttgaaatgcggccaattcggcgccg

gattgcagcgcccagctacggtttgtgctgcgctggccgctgggcttggggacggagggt

gtctctgattcagtttcagaagacaccactggggacttggtcagtcagacaaacagtgtt

acactggcactggtggtaggttgaggataaaatcgtgggggtggaaactcaggctgctag

tagtagtgccatggaaaaggtcaagctcataagacatggcaaagtttgaaatggatgaag

gcttaagagcattgctcgcgccgtgttagacgcttgtgttatggggggatgatgaattgt

gtgtgggatggaaacatatactgttattaccatgggggaaaaaaaagtcaagctcaccaa

aattaaattggcaccctaacctagtaaacttttccattgccatgcttccattattctcaa

ttataagtacaacttaatctgtgtttccttcttctccctag

AAAATGGCGATGATGGTGGATCCTCCGAACGGCATCGGAAACCATGGCAAGCACTACTAC

ACCATGTGGCAGACCATGTTCGAGATCGACACCAAGTACGTGCCCATCAAGCCCATCGGG

AGGGGAGCCTACGGGATAGTTTGCTCCTCCATCAACCAGGAGACCAATGAGAAGGTCGCC

ATCAAAAAGATAAACAACGTCTTTGACAACCGTGTGGATGCGTTAAGGACGCTGCGCGAG

CTGAAGCTCCTCCGGCACCTGCGCCACGAGAATGTCATTGCTTTGAAGGATATAATGATG

CCGATACATAGGAGGAGCTTCAAGGATGTCTACTTGGTCTCCGAGCTCATGGACACGGAT

CTGCATCAGATTGTCAAGTCGTCTCAGCCGCTGTCCAATGACCACTGCCAGTATTTCCTT

TTTCAG

gtatgtgtcggctctgtttttcattcttacctttgaattccatctagtccttcatctgtt

caccattctgccataataagttaatctgttcacacctcaggtgtatgccaagataatgtt

atttttattgttcttactaatttgcattaagtgcatgatctatctaaagttgttgagttt

caactagttgagttgccaaagaatggtctgctaatttatggaagaagcacatgtcagttt

gaatatgtctgctagtagcttctatagctgctcctcattcctcaatggtgcttgtgccct

ggaagtgtatttcttaaaagcaacctaatacaaagcagatatgcacatggtatttgcatg

aacctataacttgaacctctgtaaattatttgagcaaggctatgtacaaactgaacttga

gcatcttgtattggaccgatacttaaatacttgtcttacaaattactcttgtttgttacc

atgtcatcttgccattttctgctgatctttattctctcatatttgtcaatcaattgattg

tctaagatgccaccattattcttgactgtag

CTGCTCCGAGGACTGAAGTACCTTCATTCAGCAGGGATACTCCATAGAGACCTGAAGCCT

GGGAACCTTCTGGTCAATGCAAACTGTGACCTGAAGATCTGTGACTTTGGTCTGGCTCGC

ACAAATAACACTAAAGGTCAGTTTATGACTGAATATGTTGTCACCCGCTGGTATAGAGCT

CCCGAGTTGCTGCTCTGCTGCGACAACTATGGCACATCCATAGATGTCTGGTCTGTTGGC

TGCATCTTTGCTGAGCTACTTGGCCGCAAGCCGATCTTTCCAGGAACCGAGTGCCTTAAT

CAGCTTAAGCTTATAGTCAATGTTCTTGGCACCATGAGCGAAGCTGACCTCGCATTCATT

GACAACTCAAAAGCACGCAAGTACATTAAATCCCTTCCATACACCCCAGGGATTCCCCTC

AGTAGCATGTACCCGCAAGCGCATCCTCTTGCCATTGATCTGTTGCAGAAGATGCTTGTC

TTCGACCCTTCCAAAAGGATCAGTGTCACCCAGGCTCTGGAGCACCCCTACATGTCCCCA

CTGTATGATCCCAGCGCAAACCCTCCTGCTCAGGTGCCCATCGATCTTGACATAGATGAA

AACATTGGCACAGATATGATCCGGGAAATGTTGTGGCAGGAGATGCTCCAGTATCACCCC

GAGGCCGCCAGGATGGTGAATATGTGACAAGCAGGAATGAACATGTGACAGCAGTGTGCC

ACACCAGGGTCTTCACATGTTCGTTCTTGGTTTAAATCTTTAATGCAATTATCGTAATGC

CGTCGAGCGACCTGATTAAGTAAATATGTGCACAATAAACGGCGTATGGATTTCTCTAGC

TGTGGGTCAGTACTTGGTAGTATATATGGACTACTGTATTGTAGAAGCGGAGTTCAGTTT

ATTAAGACTTCGGTAATGAAGAAGCTTGTGTAAGTTGTAATCTTCTTTTTTCCCCTGATT

GTAAGTTGTAATTCTAGACGCTGCTCTATGTATATGAACTACTGTGTTGTAGAAGAGGAA

TCCGGTTTATTAAGAATTGGGTTATGAAGAAGCCTGTGTAAGTTGTAATCTTCTCTTTTC

CCTCTGTAAGTTGTAATTTTAGAATCCGCCCTATATATGGGGGTCTAAACTATTAAGTGA

CCATGTTTACTTCTACCTTCAAATTTGCCTTGTTTCTTCAAG

**>HvMPK11 : MLOC_71588.1**

CTGGAAACTCGCTCGCCCCATTACGAAAATAACACCGTTGTTAAAAACACTATCGTGGAA

GTACATTGAGAGCCAAACAGCTGGAGAGATGCTCTTACAAGTTGCAACGACCACTATAGC

CGAGAAAAAAGAAAGCCAAGCATTACTGTATGCAAGAAAGAAGCAAAGCAGGTTGCTGCC

ATGCACAAGGGAAGAGAAGTGAAGGCTCATCAAGGGGAGGGGGAGGAGAAAAAGGAGGCT

GGAATGGTCTGGAGCAGGAGCAGCAACCAACCAACTCCTCGCCGGATCACGGGCATTTTC

CCTCCAAAAAGCTTCCTCTCCTCCTTTTCCCCTTTTCCCCAACGCTCTCCCCGCCTTATC

TCCTCCCTCATCCATCCAACCAAGCTAGCGATCTCTCTCTCTCCCTTTCTCTTCCTCTGC

GCGGCGTGCGTTTCTTTCTTTCGTTAGCTCCTCGATCAAGGTGTTCGTGCGCAGACGTGC

GGGTCGATGCGCATGGAGGGTGGAGGCGTCGGGGCTGGAGGAGGAGGAGGAGGAGGCCAC

GGCGGCGGCCATGGCCTCGGCGGGGAGGCGCAGATCAAGGGCACGCTCACCCACGGCGGC

AGGTACGTGCAGTACAACGTCTACGGCAACCTCTTTGAGGTCTCTGCCAAGTACGTCCCA

CCCATCCGACCTGTCGGCCGCGGCGCTTGCGGCATCATCTG

gtacgtacatcatgcatgcgctgcatctgcttgcgtttattcctcaatatacctcagaat

atggattgctttgctcgattagctacttgcgatcctgttgcgagttcaccatagtatata

cgactgaaagtgagtcttgattaaagttacggagtatatagtattgaaccataaagcatt

ttctaataaaccagtaatagttaaccgttaagcaacaatacctaatactaaaaggaggca

agagctgtttgtagttgattttgagcctgtggtgacaaattcttctctgtgctcttcttc

ag

TGCTGCGGTAAATGCACATACTCGTGAGGAGGTCGCTATCAAGAAGATTGGTAATGCGTT

TGACAACCAGATCGATGCCAAACGCACTTTGCGCGAAGTAAAGCTGCTTCGCCACATGAA

TCATGAGAAT

gtgagccatctttccttttctctcttgtgccgccttgtgataaacctctgtggttcacat

gctcatatctgaaatgttttgtggtttctatttgaccatggttaactctgtagtttatag

caaaggtaagccgtcctgagaagacctgccggaaattctggtgattttctttcaaagcca

gaattgcctattattatttatttactatacagacgatatatgtgacaaaacttgaagttg

ccttccctacctgtgctactgtgtttaaacttcaaactatcttgcaacataagtccatct

gatgttggttaactccacttatgatgtcgaaaatatatatggtgtgaacctgaatttgat

ggtatgaccatgacaaagtctgacttttgacag

GTGATTTCAATAAAGGACATCATACGCCCACCAAGGCGGGAGAACTTCAACGATGTTTAC

ATCGTCTACGAACTGATGGACACTGATCTTCACCACCTTCTAAGATCAAACCAGCCACTC

ACAGATGATCACTGTCAG

gtatgtacattgttctttccatcgtttcatcaacatcagtccaaacattccacaattagt

cagctttcctttcgccttaacgttttcatgatgcag

TATTTTCTCTACCAAGTGCTCCGAGGACTAAAGTACGTGCATTCAGCAAAGGTCTTGCAC

CGGGACCTCAGGCCGAGCAACCTGCTGCTCAATGCCAAGTGTGAACTCAAGATTGGAGAT

TTTGGCTTGGCAAGGACCACCACTGAGACTGATTTCATGATGGAGTATGTTGTTACTCGG

TGGTACAGGGCGCCGGAGCTCCTCCTCAATTGCTCGGAGTACACTGCAGCAATTGATATT

TGGTCAGTCGGTTGCATCCTAGGTGAGATTGCTATGAGGGAGCCACTGTTTCCTGGAAAA

GATTATGTTCATCAGCTGAGGCTAATTACTGAG

gtatggccactcactactagtctactacacgactcaagatttgttttttcccttttgttt

cgtttttcttatttttgtatattctcctgctctctttttgcctcagtgtttgaccctatg

attgtcagttctgatttagcataggttccaactgctatgcttttgccatgcttatatctc

tcggattactaactcaactagcacaactacaccacataggttgaagcttacactggtatc

tggtgtagcatgatacttcatgcatatgccttcattccgactaatgttttggtccaagca

tgtgataattctagatgcactaacatcttctcttgttctactaaatggaaatgtctaata

attcttctcgatgctgtttgtaatcctgattcattcttcatccaaag

CTGATAGGCTCACCAGATGATACGAGCCTTGGGTTTCTTCGAAGTGATAATGCCCGCAGA

TACGTGAGGTCTCTTCCTCAATACCCGAAACAACATTTTGGTTCACGGTTCCCCAATATG

TCCACTGGTGCCATGGATTTGCTTGAGAGGATGCTCGTGTTTGATCCGAGCAAGAGGATT

ACTG

gtaatcttgcaacatagcatgatcttgcttgattgcaaaatgacaaatttaataatgtga

accatcattccagaatcgtgtgtgaattattaaaaccaagttgaaatgacacttcttatg

ataattaagccattaaaccgttaatgtaagaaatattatgaggaacttcctattggaaga

ttaacctttttttttctcttggtggcattacag

TTGATGAGGCTCTGTGCCATCCGTACTTAGCATCCCTTCATGAGATAAATGATGAACCTG

TCTGCCCAGCGCCTTTCAGCTTCGACTTTGAGCAGCCATCATTTACTGAGGAAGATATCA

AAGAACTTATTTGGAGGGAGGCTCTCAAGTTCAACCCTGAACCAATTCACTGAAAAGGTC

CCAATGTAGAAAAAGAAGCAATTTGAAATGGCAAATCTATCAGCTCCGCGGGGAAATACA

TGAGCTGACAGCACCATGTACAGAGTAATCAGTGATCAGGTCTAAGCATCTTGTCCTACG

GCTTGTTGATGACACCATGATGTCAAAAACCTGGAAATTTAGGAAACATGAACGCTTGAT

CAATAATTTGTTAGCCAAAAAGTGGGGCCTTTATCTTTATCACGTTAGGTTGTAACTACC

CCCTTTGGTTGTATGCTTAACAAGACCTGAGCTATAGACTATGGTATTACAGTTTCCGGC

CACTCTTCGGTGTTAGTGTTGTTCACCATCCAACGACTGTTACTTATTATTATTGTGTTC

TAAACCCTGCAATCAAT

**>HvMPK14 : MLOC_44271.1**

TCCTACCCACCCACCCCTCCCCCATCCTCTCCCAGGCCCAGCTGCCCTCCCTCCCCCTCC

CAT

gtaagcgagccttcctccccgtcctccccccgcaatcctcgtgctcgctctgcatcgccc

gagatcggccggctcttgtccggacggcccgcgccggtctctgtcccgcggagatcggtc

gcccctgatccgagcgagcagccccagctggcgagctccgattgtgccgccgggcgcgtg

cgtgcgtgcctgcctagtcgagtgtcccgcccgaattattcgattccccgtcgtgctccg

gcgatgcatcgtgccggggtgcgcgatgattgtctcggtacatttccgcgatccgctgct

aggatggcaggctgccctgttaatcatctgggcgacgatctgcccaccgggagcttatgg

gttcttgcaggctgcggattcggcttgtgcgtttagcaccgagtgaggctgatgagaatg

cgggtgagaggtggtagcgaggagtggcattgcatggtgcttagatgcgcggattggctg

cgctaccgatcaaagttaccacttataagcttatgtaaatggggacattggcgctcatca

attcaagtgctgaattggtcagcgccagtgtccatttataagcttagataagtacaactg

cacttagatgccatgccaacatgtactgcttatatatatatatatagttcattcattgtt

gccgattctgatcagaatttatgagccttgtagctcctggtgcatggtacagtttaggtt

cagttcagaatccttctaccacttggagtttcacataaacacttaccagaaaaacaagag

tgtgtgtgttctattggcatctgaggtcatgtaaactattaccaaagcctcttttagctg

gagatttgtttattgtccacgtccccttgtcagcttaagcatttgggtgccatggttagg

cgcatgcaattcaatataatatcagagttgaggggtcttaggttcaatacttggccaacg

tactatcaataagtatacatcggcctacgtcgatcccaggtctaaggtctggagggtagg

aaagtgttcaaagatggggacattgttacacttgtattttgtaatgagtctgtcttattt

ttcacatttgaaatttggtgattgtgtaagctaaaagaaactaaattttcttccagtact

gttatttatggtatactaagttatatgttttgttttgttag

TTGAAAATGGCAATGCTGGTGGATCCTCCGAATGGCATGGGAAACCAAGGGAAGCATTAC

TACTCCATGTGGCAAACCTTGTTTGAGATTGACACCAAGTATGTGCCTATCAAGCCTATT

GGTCGGGGAGCTTATGGAATAGTCTGCTCATCCATAAACCGTGAGACAAACGAGAAAGTA

GCTATAAAGAAGATACATAATGTATTCGACAACCGCGTGGATGCACTAAGGACCTTGCGG

GAGCTGAAACTCCTCCGGCATCTCCGCCATGAGAATGTTATTTCTTTGAAGGATATAATG

ATGCCTGTACAAAGGAGGAGCTTTAAGGATGTGTACTTGGTTTATGAGCTCATGGATACC

GACCTGCATCAGATAATCAAATCACCTCAGGGGCTTTCCAATGACCACTGCCAATATTTT

CTTTTTCAG

gtaatgctaggatcacaccttttccatcagtctttcacatttaagtatttaatattagta

acaatgcgtcttctcatacctgttattactttcactgccctcaccagttgtgataataca

tttatatctgcaggtgctagtgtagagcaccttccttagtctgcaccagattaattggtg

gccaatacgcactgtcaggtgtctttgtcgattcaaacttgaaatacacaaactcttctg

taaacacttcagcgttaatcatcagtgtttacagacgaccaattggagtttgatgtcatt

tctaaaccatcaaatggctgatccagtccatatatcccttgagccctaacctcaaaatca

attcgaccatgaagttcttttcatgtttttacacattattcttgtagtttgtgctcgttt

gaatgaaaactgtgaaacctaggacaatcgaaaagagtagtgcaacttggcatatatctt

tgtcatcccgtgtgattctggtcggtttcacatgttccttcactcactaggttttgaaag

aactggcaataactggccacttatctttgtttccaagtccatgttgcagccttttagttt

gacagctggacagaaggtatcttctagtaactttagaatggaaggcagctgcactctgta

gctatataggcaacctcaaactggatgtcatagaatatattgttgttttttcaccttttc

tgcttcactttagacttttagtacttttttaatcgttcgacacaatagcatagttcatat

gcgatatgtgaattttccttttctgcaagtgacatcaacctgtattactctcgcagtcac

attgtgaggcattgtcttgctgcctcatgaattaaataaacaagaccaacacaggctcat

gtccagtgaccagtgcaacctaaaactagcacattctgtttagatgtttctgagttacaa

cttatcttgttaacatagtaacagccagacctaccaatggagctgatatattgaatatgg

ctacttggacttcactttgtcatttgtcagacctccgcatccaaatagagacagcttgga

gtgaattgtggctttctggtaaaaagctataaaatggcattcttcttacatttcagcttt

tgtgtacatacattaaactaaataagtatctctacgactacatgaagtataagcatgcat

tccaaaagaactatctctaagtataagcatgctggtgaattttcagttcatgttaccctg

gtgcttcatttaacctgttgtcttaacatcctttgattttttttcctatctggattttct

gttgataatttaagatgtttttttcttgattgcag

TTGCTTCGAGGACTGAAATACCTCCATTCAGCAGAGATACTCCACAGAGACCTAAAACCT

GGGAACCTACTGGTGAATGCAAACTGTGATCTGAAGATATGTGATTTTGGTCTTGCACGT

ACAAACAGTAGTAAAGGCCAGTTTATGACTGAATACGTCGTGACCCGCTGGTACAGAGCT

CCCGAGTTGCTGCTTTGCTGTGACAACTACGGCACTTCCATCGATGTTTGGTCTGTTGGC

TGCATCTTTGCTGAGCTACTTGGTCGCAAGCCTATTTTTCCAGGGACCGAGTGCCTAAAT

CAGCTAAAATTGATAGTCAATGTTCTTGGCACCATGAGCGAGTCTGACCTGGAGTTCATC

GACAACCCAAAAGCTCGCAGATATATCAAGACCCTCCCCTACACTCCTGGTGTTCCACTC

GCAAGTATGTACCCACATGCGCACCCTCTTGCCATCGATCTGTTACAGAAGATGCTCATC

TTCGATCCTACCAAAAGGATAAGTGTTACCCAGGCCCTTGAGCACCCTTACATGTCTCCT

CTGTATGACCCAAGTGCAAACCCTCCCGCGCAAGTGCCCATCGATCTCGACATAGATGAG

AACATCAGTTCAGAGATGATCAGGGAAATGATGTGGCAGGAGATGCTTCACTACCATCCT

GAAGCCGCCGCAGCAGTAAACATGTGACCAACATCTTGCAGTGCCCCGGGAAGAACCCGG

CAGGCTCACTACTTTTTCCCCCGAAAAGACTACGGCGATTATCGCACCTATTAAGTAACC

ACGACGTGCAGTGTGGAGAGTTATCTCCGTGTAAATACGCAGTGCGATAAGAACCGCATA

TGGATAGTTCTTGTTATGGACCACTATTTGGTGTATGTATACTGTTGTGTTGTTGTATGA

GCTCCTGAAAGAACTGTTGAAGCGAATTCAGTAAGTGTTGATTTGTGATGGCCCTGTTGC

AGTTGTTGGTATTTGAAGCAGACCTGTTGTTATTTTCTTGCGTG

**>HvMPK16 : MLOC_60926.2**

CCCCGGGATATAAATTCTGAAAGCGGAGGCCTTCTCTCTCCTGACTGCCCAGCTACCACC

GCCCCCCTCCCCTCCCCTCTGTCCTCGAGCTTCGCCTTGGATCGCAGCGCAGCGCAGCTG

GACGAGGACTCCTTCCCTCGGCCGCGGCCCCCTCCCCCTCGTCGCGCGCGCGAGTGAGCG

A

gtgagctcccttcccccatccccacgcccccgctctcctcctcctcctcccccctttctt

gctggatcgggcgtgctcgaagatctcgcctttctcgcgcgaaagatcccctctcctccc

gggagagatctgccgggagggcgcggcaggcgcggaggaagcacgccgtgcccccctctc

cgttccgccgagtgcccctgccgccggcccccacgctccagcgccgtgccgggagttagt

agcagcgttctttcttttcttggagtggattggtggtcttcttggctcgggatcttggtc

agtccggagcttttgctcgccgaaccgcgaagccgcgctgtcctggccgcaggcttgttc

cccgctgtagaacccggcggctagacggatctactcgatttggagtttcggttgctttat

tacgagagatttggagtgctttttggtggaaagaactggcgccggctgcttggttttgca

ggcctcgccggttcctcttgtaaactgatgcccggcagtaatggtgctactgttcaaccc

tctcagttcgagctatatgagccaaactttgtttaaggccttgacttgagtagttatgct

gtcgtcttcatatctttacccacctctctgtgcggcagtacgcatgcttcatgccatccc

aacaattccatcggttgggcaaaaagtttcatcttgttttcgggaacgaaaggaatgccc

cctagacgtcctcgctgcatcatgtggttctagtgctgttaatttgcttccaagcaactt

ttcagataaaatttgttactttatgtttcgatcatgctatagtttgcgatgtatgatctc

tccatggaacttgatttcccatgtggtcattttggcttactatttggctagtgtcctttt

gtacatagtcctcgtcgcttgccaaattatgactctaatatctattttgagtgtcaccat

ctcaccatatcatactaaagaacacaaatcatcagtgctcaagctgacccatattctgtt

ttttgtgacacag

ACATCTCAAATGGACTTTTTTACCGAGTATGGTGAGGGGAACAGATACAAGATAGAAGAG

GTTATAGGAAAGGGGAGTTATGGCGTGGTCTGCTCTGCTTTGGATACTCACACGGGTGAG

AAAGTTGCTATCAAGAAGATCAATGACATCTTTGAGCATGTGTCTGATGCCACACGGATA

CTCCGCGAGATCAAGTTGCTTAGGCTCCTAAGACATCCCGATATTGTGGAAATTAAACAT

ATTCTACTTCCTCCGTCGAGGAGAGAGTTCAAGGATATATACGTTGTTTTTGAACTCATG

GAATCTGATTTGCACCAAGTTATAAAGGCAAATGATGACTTGACTCCGGAACATTATCAG

TTTTTCTTGTATCAGTTGCTCCGGGGATTGAAATACATACATACAG

gtaactgatttattttcacatcaaagatggttcttttgcatgtttcaacagctggatgtc

aggctctcaatttttactaataactgcagcagccatgaatggtcaatgtgtttacaagtg

atacacatgtcagtggataacaataccatgtcaaattcgaaactatacacatgtcagtag

ctaacgacgatgtattggtcatgttggcatttgtgaaaagctttaaaaccttatagtatg

ttccacaaactttatagtcaagagaacaaatatggacctgtgtagtactttgatacaggt

cacacagtttgctatagtgctcagagaattctttggaaatccagatctttcatgtgaatt

tcttgaacttgtgcaaacttgcctgagttgtgtgttctatacttctatttttaattttta

atcacctagtaactggtgaatgctgtgatgaaaagttactgctaatcatttacagtagct

cccaagcttcttgtgatgctacaacagaactgtggtcctagtgaggatgctattggtgct

gttctttgtgccaaaagacagcaactagataggcatacttaacttgtgaagcatgtttga

ggctagagaaacttctgtttaaacttttaaaatataaataacctatttgtgatcaagtag

gtatttttcagttcttttttttggcatccctattgttcacattatttaaacaaatgagta

taagtacttttgctacagatacaaatgtggcattaggagtcgattggcaataatataaaa

taagacttagaaaaaaatcattttggctgatgcatcttccaatagcttcttgcag

CAAATGTATTTCATCGCGATCTCAAACCAAAGAATATCTTGGCTAATGCTGATTGTAAGC

TCAAAATATGTGACTTTGGTCTTGCAAGGGTAGCTATAAGTGATACTCCAACTGCCATAT

TTTGGACG

gtattacaattccccctaccatgctgcccatttcttttagttactcaatatcgacaacag

cagctaatttcttatcctgtttttag

GATTATATCGCAACAAGGTGGTACCGAGCACCTGAACTATGTGGATCTTTTTTCTCCAAG

gtctgtatatgttaaatttcatatgaatcgtggtatcaacagcatttgtcattattttcc

ccgtcattattccaataatctgatttactttgtttactgtttaaggctttgtcctatgtg

accacaaagcaaagtaatctccccctggcatttccttagtagttcttttcctactccctc

tgtcccaaaattactgtctaaactttgtcctatcttaagtactgtacaaatttgtactaa

gcttaagacacttattttgtgacgaagggagtatttcctaacaggttatgttcaaatata

tattagctatgcaaaacaatgccaatcaggcagcatggtttaatattaaatgtttaagaa

cacattccaacatcgggcacagttcatggtttttttttgtcttccaccctctaacggatc

aaataatttttaatgtgcatattgttcttgtgttcatggtaatcatagtgcttcaatgtc

attgatgaataatcttgttttctgtatttcttttcgtaattaagctgtcaaagaaacaag

ggcatctctagttgtaactcaagtactcttatgtctaggtttgattaatccagctaaaca

tgttctggaatatcttgagcgctgtataacattatgttcttcaataccttgcag

TACACACCAGCAATAGATATATGGAGTATTGGATGTATATTTGCGGAACTTCTAACTGGC

AAACCTCTTTTTCCTGGGAAAAATGTGGTGCATCAACTTGACATAATCACAGACCTCCTG

GGAACGCCTTCTCCAGAAACAATTGCTAGG

gtcagtatggtactgtggttggcataaaacaacgtgttgtttccaatgtaatcctctgat

gtttgtagctatcctacag

ATTCGAAATGAGAAGGCCAGGCGCTACTTGAGCAGCATGAGGCGGAAAAAGCCTGTACCG

TTTACGCAGAAGTTTCCGAATGCAGATCCACTTGCATTAAATTTGTTGGAGAGAATGCTA

GCATTTGATCCAAAAGACCGGCCAAGTGCTGAAGAG

gtagtttgttttagtaccatgcctgccgacaattttccctgtgtttcttctttacttata

accttgaataccactagctaatgtattaggcatgttgacag

GCTCTTGCTGATCTTTATTTCAAGAACATAGCTAGTGTGGATAGGGAGCCTTCTGCACAG

CCCATCACTAAGCTTGAATTCGAGTTTGAGAGGCGAAGAATTACGAAGGACGACATAAGG

GAACTCATATACAGAGAAATTCTGGAATATCATCCAAACATGCTGAGGGAATTCCTTGAG

GGGGCTGAGCCAACTGGTTTCATGTACCCAAG

gtgagaacttctctttcatcattactttaacattcttcactagtttaattcctgagacaa

tgttgtgacattatttttgccagccagtctagttttgtctgaatttctaaaaaaaaatgt

accattaatgtctgtgcaggttcatgaataaatattaagtgtttatacagacactattat

ggttattttcctactcttttggtgacggtgatattgataaataaagcttgcaaaatttgt

tttatgtttattgttgattaggtagtgcatacctattctgtgtgttgcttggagtcagtc

aggcactgcataagatactgaccaattcatcgaactaacatcgtttgctctatttggatt

tcag

TGCAGTAGATCATTTCAAAAAACAATTCACATTCCTTGAAGAGCATTATGCAAAGGGATC

AACAGCAGCACCGCCTGAGAGGCAACATAATTCATTACCAAG

gtaagatattgactttgctttgacctggtatggatggactgatgtcacttctagccacaa

attggacgtctggttcttgactatctattttttttctgttaacag

GCCTAGTGTTATCTTTTCGGATAACCGACCACAGGGTTCAGATAGCCGACCACAGGGTGC

AGCCAACATTACCGATGACCTTTCCAGGTGTATAATCAGAGATAATACACAAAAGCCACG

CAGAGATACTGCTTCAGTTGGTGCAAACAGAGTTCCTCAAG

gtaagcacctgggccatccaatacttacttttgaatctgcaaacaagcctgcatgactgt

atcactaactgtatctatcgggcctctaactcaagactaacttgatacatcacttcatca

tacag

GTGCTGCTGTCGCAAGACCTGGTAAAGTGGTTGGTTCGGCACTTCGTTATGGTAACTGTT

CAACATCTGGTACTGAGCAATATGAACAGCGAAGGGTTATCACAGGCCAAGGAATTGTTC

CAAACGGCGTTCCTTCAGGCAGCTCATACCCTAGAAGAAATAACACCTGCAAGAGCGAAA

CAGGTGAAGCTGAAAGGATCGACGTGAGCCAAGCTGGGCCACCAAAGCCATATACAGGAA

ATAAACTACCTGCAACCGTGGATGGCCGCAACGGGCACTGGTAGACTTCTCTTCCAATGT

CAGAAAATCCTTGTCACTGGATCAGGAAGGGTTACTCATTCCAAGGGCACACCTTCAAAC

ATGGCATTCTGTACAGAGTTATATTATCTGTCACGCTTACATCTCCCGGAGACGATAGAA

CCGCATGCTAAGTCGTTGCGGCAGGCTAGTCTCCTGTTAAGCTCAAACTCTGCTCTGGTT

ACCTGCAATTGGGTAGATCCAGTTCAGCTGTTCAACAAGTATAACCAAAAACTGAAGCTT

CGAGCGGCATTCGCGACAAGGGACTGTTACTGACCTTACTGATGGTGTTCCTCAATTTGT

TTTTTCAAGCTGGTGGTCCTGAGGGAGGTTCTGAGAGCCCACTGTACACTATCTAATGTT

GTAACACGGAGTTGATTAATTGACTTTAAAGTATGTTCTTGTTGCTTGTAGTCTGTCATC

CAGCTGTAGTATTGAACTATTGAACACTGCT

**>HvMPK17 : MLOC_31474.2**

GCATCCAATCCGTCCATCCATCCTCACCGCCGACGACACCGCCGGAGACAGCGGCCGAGC

ACTCCATCTCGAGCCAGTGAGTGGCGGCGGAAGGGGGAGGCGAGAAGAGCTGTGGGCTGT

GGCTGGAGGCGGCGGCCAGCCAATGCCGCGGCGGGCGCAGGGGTAGGGAGGCGGCGCATG

GGGGGAGGGAACGGCATCGTCGACGGCTTCCGCCGCTTGTTCCACCGCCGCACGCCCTCC

GGCTCCGTGCTCGGCAGCTCCAACCAGTCCTCCGCCGGCGAGGACTCCTCGGAGCTCGAG

GCCGTCGAGGACCTGGATCTCGTGGGCCTCCGCCCCATCCGCGTCCCCAAGCGCAAGATG

CCGCTCCCCGTCGAGAGCCACAAGAAG

gtgagagcagtattccgatgctctctgctgcttattctcgatgcttccattttagttggg

gggcaggagcttttggcagatgctatctccatttctcgtcaccctcgattcgtcgtcgcg

agcagttcttagccaagatcggatctttgcggcacctgctcagctcagatacggcactac

agtacaatactcctactagcttgccttttctttccttttttttttcgccggacaccgcag

ctcagcaagtgagcttgattctccgattcaatttcccatctctggatcgatacggcgtag

gggccactgcttgcgatcgagctaccgatcggcctgccgctcgactgatctgggaaaata

ctatgcaaaaccgaatgccgtttcttcagagatgagcatctgctgcgtaaagccggcgcc

ctttggggcttctcttcacactttttggttgattttcaactactaaggttgctttcagca

tccgtatatcacttatccgaaggggcctcctcatgtccaatttgtccacttggttctaca

gcgactgaatcggccgctgatcagttaggttgacatggtccggacctcgtgctcaactgc

tcatgtccacctcactcttgcaccatggtggctgcttccttagtttctcagtaaacctga

ccatttccctgattgctcattgctgagaatagtccctcacctaccggcctagactttttt

tccgttttgggggttcgggctgtgtgttgtggtggcggtactgtaagatatcctatagga

gcccaacgacctgggaactggaactggtctaatggcattgaggatcgacattgttaaaaa

aaatgatgtttggtcataatagataatgtgttttataaccaactgtatttataagcttgt

gcgtttacttatgatttttgctttctgttgaatcgcttttactgatcaaatgttcagtta

cttaaatatatttttgaattcattcttcaatttcttcgatccaaaatgttccgtacatcc

gtaactggctttaacttgctcctgtgaattctggtcttgtag

AACATAATGGAGAAAGAATTCTTCACTGAGTACGGAGAGGCAAGCCAGTACCAAATCCAA

GAAGTTGTTGGCAAAGGGAGTTATGGAGTGGTTGCTGCTGCAATAGATACCCGCACCGGC

GAGCGGGTTGCGATTAAGAAGATCAATGATGTGTTTGAGCATGTCTCGGATGCCACACGC

ATCCTCCGTGAGGTCAAGCTCCTTCGGCTGCTCCGTCATCCGGACGTGGTAGAGATCAAG

CACATAATGCTCCCCCCTTCTCGGAGGGAGTTCCAAGATATATATGTTGTTTTCGAGCTC

ATGGAGTCGGATCTCCATCAGGTCATCAGAGCTAATGATGACCTCACGGCGGAGCATTAC

CAGTTTTTCCTTTACCAGCTTCTCCGCGCTCTCAAGTACATCCATGGGG

gtaagtcagtgcgctttgagttgatttaccaaattgtgactggacttctgtttatattct

gacatattttgctgtttatcttggcgcag

CTAATGTATTTCATCGCGATCTGAAGCCCAAGAATATACTGGCCAACGCAGACTGCAAAC

TGAAAATTTGTGACTTTGGACTTGCGCGTGTATCATTTAATGATGCTCCTTCAGCTATAT

TTTGGACG

gtaaagagcttgtcattgtatgtcacagttcacattttactatattttcttacttcttaa

tctgtatttctttgtgaag

GATTATGTAGCAACAAGGTGGTACAGAGCTCCTGAATTATGTGGCTCCTTTTTCTCGAAA

gtgagttcttcacttcctaaactcgtaaagtctcatacaagcaagattaatgctttcgga

gcactatatatgcagatgatgtctttatatttgtgatacatttatagatccctattgctg

tttttatttgtcttaaaaaactatgtaatctacagcttgtacaagtaaagtacacaaagt

taaggattaaacagtgcaacaagtttttgttgttatttctcattgaaattctgcacgcac

ccatttcttctgtcaaaacaagccgtaaactaactaatcagttgaatatttagagtagta

aaatactaaaatccatgtagagattttctattctttttttgcaagtgtcagatttgtcac

atcattctgataagtgcccatttgcatgcag

TACACTCCTGCTATTGATATTTGGAGTATTGGATGTATATTTGCCGAGCTTCTCACTGGA

CGACCACTTTTTCCTGGGAAGAATGTTGTACACCAGTTAGATATAATAACAGATCTTCTT

GGAACTCCATCATCAGAAACCTTATCTCGG

gtatgtttgttccccgcctcttatttttggttcaaccagccatatggtaatattcaaaga

gatgcagttcactaacaagctgaaacaatggatgtacagaatttcattcctgttgcaccc

ttttgtaaaacgcattgcaagccactagagcaatttccccagattctgtccagttatatc

tttcgtggtctatgcttgtctttggtctgcaatttttcaatggtccattatgttacagga

gtaataactcacagctgtctatgacctttgcag

ATTCGAAACGAGAAGGCCAGGAGGTACTTAAGTTGCATGCGAAAAAAACATCCTGTGCCC

TTGACTCAGAAATTTCCTAATGCTGATCCGTTGGCGGTTCGCCTACTGGGGCGTTTACTT

GCATTTGATCCTAAAGACCGGCCTTCAGCTGAAGAG

gtaaacttgtacgagtattctctttcacatttgtttttaacaatttcacaaacacgtata

tttgtcttgatatgctataattttgttgccttcttaattcttcag

GCTTTGGCAGACCCATATTTCGCATCTCTTGCTAATGTGGAACGTGAGCCTTCAAGGCAT

CCAATTTCGAAACTTGAGTTTGAGTTTGAGAGACGAAAGGTGACAAAAGATGATGTTAGA

GAATTGATCTATCGAGAGGTGAAGAGGCATAGCTTGGGTTTCCTATTTTGTGTTAATAAT

ACATTTTATGTCTCTCGATAATTGATTATTTGTTATGAGAACAAACAGATTTTGGAGTAC

CATCCACAAATGCTGGAGGAGTACATGAAAGGGGGAGATCAGATTAGCTTCCTCTATCCA

AG

gtgcaattctgacttgctttaattttcatgcaagttatcagactgaataaattacataat

taacatatctttatgattctacag

TGGGGTTGACCGCTTTAAGCGGCAGTTTGCGCACCTGGAGGAGCATTACAGCAAAGGAGA

ACGAGGTTCTCCACTGCAAAGAAAGCATGCCTCTTTACCGAG

gtacatataacaccgcagccatcagaaatccgaagtgccgagcttcttaccctgatctcg

gttaatcagttatttgtgctgagttctgcattgacttgtgtgtgacag

GCAGAGAGTAGGTGCATCGAACGACAGTAATAATGAACAGCATATTAGTGATCAGGAGAT

GAGTGCAGAGCCTGATGCCCATGGTGCAGTGAGCCCTCAAAAGCCACAGGATGCACCCGG

TGTTGGTCAGAATGGTCTGAGCCCCACCAGCTTGAGCTCGCGGACCTACCTCAAGAGCGC

GAGCATTAGTGCTTCCAAGTGTGTCGTTGTCAACCCGAATAAACAGCCAGAG

gtactagtgctgtattacctgaattcttcattagttattacctagttatcacaactaggc

atggcatagctttgcacaatcaacaaatgagaaaagatggtggtgcaagttgaacttcgt

ttggttcgctttagagctcaagcactgagtccgtttccattctttgtttctattattgtt

cag

TATGACGATGCGATCTCCGAGGAAACGGAAGGGGCCGTCGACGGACTATCCGAGAAGGTC

TCCAAGATGCATGCCTAGTGCAGCGCCGATGACGGAAGTCGGCACCGCATTTCTCATTTC

CGTCAGGTACACCGGATGCTGATAGCAAAGCAGTGCGGTCCTAGGGAAGCACTGCATACA

AGTTGGAGATTGTTTAGCGAGTGTGTGATCCTGCCAAGCATCGCTGCTGCACGCAGGAAC

TGTTGCCCTGCATGGGTTGATGTATTTTTACACTCAAGATTACAGATGTAACACCGGGAT

TAGTTCCTACACATATCATCTATGCATGATTGTAACGAACAGAGCAAGACATCTGAACGC

TGTCCTGTTTCGCTGTTGAACATTCTTAATATATCAACAAGCGTCACTGCTGTGC

**>HvMPK20-2 : MLOC_64743.1**

GCAGCAAAGCAGACAAGAGAAGGAAATCTACCTCCCTCCTCCCCTCCCCTCCCCTCCGGC

ATCCACCCGCTCTCCGGCGCGCCGCCAGATCGGGCCAGAAGAGGAGGGCGGGCACCTACC

TTACCCCTCTCCTCCCCGGCGGCAGCCTGCGCTCGGTCGCCGCGGCGTGTGCGTTTGGCG

GTTCGGGAGTCGGGCGGTCCGGCGCCGCGCGCGGGAGGGCAGGGGTCCGTCCGGTCTCCG

GAACCGTCTTGGAGGGCAGATCGGCCCGTCCGGCAACCGGTAATGCAGCGGAGCAAG

gtgggtttgtctgggttccgagccgcccctcctcctcctcctgcgtctgtccggtaggca

cgatctgcgcctgttcttgcgtgcggtgccgatgccttgcctgctgctcacctcggtgcg

ccagcacgatgccggcgtctttttgaatcgtcgccgccgtgttttcaaattcaaatcaaa

cccctttgacggattctttgctgctgcttttttgaccggatttcggcgctgccgatccct

ccttcctgcggccgatctgctgtttctgtccccagtgaaaattagtcaaatgccgtcggt

tccttccggtacggcgacaccgcgtgatgagttgcaggcagctcatgcctcacatgggaa

agttgaccgtctccttgccgcttcctgaatccagatgccctctgcgccaaccaaacatta

aacaaaatccgcaagatgccgcctttcccccgtcggtggtgcgcaaaattgtttgttgga

gtccttgcagatgcccaccagcaacctgccttcagtggcagaaaagttttagaactgttt

gattgctgctggtgctttggcttttcttttaatggttggttctgttgcaatgtggccatt

ggaaatctctgtcggtctgcactgattatctaacccccaccacaccaatcattatgacat

ttgagtaccccagatcaaccgtttaaagtcatctgtagccggtggctgtcttatctgttc

taacagttgggttttgagggtttgctgattcagtatgtgaaattcatgttttccag

AGTTCGGCGGAGGTGGACTTCTTCACGGAGTATGGCGACGCCAACCGGTACAAGATCCAG

GAGGTCATTGGCAAGGGGAGCTATGGGGTCGTCTGCTCTGCCCTTGATCTGCAGACCAGG

CAGAAAGTGGCGATCAAGAAGATACACAATATTTTCGAGCATACCTCCGACGCCGCACGG

ATTCTCCGTGAGATCAAGCTTCTGAGGCTCTTACGGCATCCCGACGTCGTGGAGATCAAG

CACATTATGTTGCCTCCCTCGAGGAAGGACTTCAAAGACATCTATGTTGTTTTTGAGCTC

ATGGAGTCTGATCTCCACCAAGTTATCAAGGCCAATGATGACTTGACAAAAGAGCATTAC

CAGTTCTTTCTCTACCAACTACTCCGAGCCCTCAAATACATTCATACTG

gtaagaagccgttaccttcaaggctactgttgttgcttggaaaacaatgtttcaccctca

aggtggtgtgtgttgtagtttacactcatgtgtagtaggtcagatcatcttttcatctcc

ttttcacttgcctctcaaacactcccccctagaggcttgctcttcaatattaagtccagt

gatgggtcagttatggtttgttcagtgtggagagctagatgatatacactgcggtttagt

caacagtcctcagtaacagtggatactttaagtatcaaaatgtgacttgctacgagcgca

gtcaaccaagttaaacaacctacagttgcatctttttcaacctacagttgcatctttcat

atttaaaagccagtgatggttcagttatggtttatcatttcatattaaatcctgtgtatc

atcatctctcaacctacagttgcatctttcttcgtttgctttgtgctatgcattacttgt

tcactattgtcacaatatgtatcatcccttatcagcaatttttaaaatatgccattacat

ccattcatatattaggctgcattctctgaatttctttgattgttttttttctaactttgg

ggccatagatcatcctgtagccatgatggatgcgaattatgcttatattctatcagtcaa

tataattagcacactattatttgatgcaacctttgttcaactcttgaaacgaaatatttt

ggtgtttttttcaatccttatatatggtaggcactctagtcaagattctgcaatttggtt

gctcattggaaatctctcccacttacttgccatcacatagcttgccatggtattgaattt

acactgccagaaattgtatcccaatgtttttcttgaatgacacactgtagaaaatgttat

ttgattgcgtgttctatttttatcacacatttgtttttgctgataaggacctcgcataac

tgcag

CTAGTGTTTACCATCGTGACCTGAAGCCCAAGAATATTTTAGCAAATTCTAACTGCAAAC

TGAAGATATGTGATTTTGGACTAGCACGAGTTGCATTCAATGATACCCCAACAACTGTCT

TCTGGACG

gtatgttagtacaaaactcaatttatgttttttattgcagcattttattttagtgtcatc

atttacagatatcatgtgcaatctctgcag

GATTACGTCGCGACAAGATGGTATAGAGCTCCAGAACTCTGTGGTTCCTTTTTCACAAAG

gtaaggtttaaagttcatggctttgtgcttattaaaaatgaagaaaagaactaggttctt

aatcctttgacaggcttatactgtaacaaagaacacacttacaatttttctagcatgaat

ttattgaatcgtgaggcaaatggcttatggttgatggttccgtatcatgggttatctaac

cttttgttgtataaacattgttttttgctttcatcttctctgatgataagttttctggtg

aaaatttaaatgcactgtgtctgctctttttatcgtggttccttttttttgccaaagtgt

aaattgtctatgctttattgttatggtttccccatctgtggtatagattagatctaatca

cagtcatttctatcattttgtatttggtgacctaatttcattcatgaattgatcttagca

tttgttcatcctatgttctacatgaatactcgatcactttcctcttaaataactttcaag

aacagtaaacatctcttactctcaagaacagtaaaagcatctttattaattcccaagatt

ttgatccgatgtatgttcttaatctattttgtatttttgtaaaatgcag

TATACACCTGCCATTGATATTTGGAGCATTGGGTGCATTTTTGCTGAGGTGCTGACAGGA

AAGCCTTTGTTTCCTGGTAAAAATGTTGTCCATCAGTTAGACTTGATGACTGATCTTCTA

GGCACACCTTCTATGGATACAATTTCTCGG

gtatggtactaaattcactcgaagagtatatatttagtttgtcgtggaaatgtattcagt

aagcccgaaatttgacttgttgtttttgttccttcccag

GTCCGAAATGAGAAAGCAAGAAGGTACTTGACCAGTATGAGAAAGAAAGACCCGGTCCCA

TTTTCTCAGAAGTTTCCTAACGCAGATCCATTGGGAGTAAAACTCTTGGAGAAGCTATTA

GCCTTTGATCCAAAGGACCGTCCAACTGCGGAAGAG

gtagtcttatggtgctattttgtctttctgacgccttggtttcttctctctgtttgcagc

attagtttgttatatttgtatggagtaagttgtaatgtttccttcattgtttgtattttg

tcag

GCATTGACTGATCCATACTTTAGAGGCCTTTCCAAGCCAGAGAGAGAACCATCCTGTCAG

CCAATTAGAAAAATGGAGTTTGACTTTGAGCACAGCAGGGTGTCAAAGGATGATATAAGG

GAGCTGATATTCCAGGAGATATTGGAATATCATCCGCAATTGCTGAAGAGCTACATAGAT

GGCACAGAGAGGACCACCTTCCTCTACCCAAG

gtttaagtgtttctatgtttatattccctcatttctgtttgttgtgtcaactcttactga

cagcaatatttcttggttatgtttgtcag

TGCTGTTGATCATTTTAAGAAGCAGTTCTCTCATCTCGAAGAAAGTGATGGCAGCGGCCC

TGTAGTCCCAGCAGATAGAAAACATGCATCTCTTCCTAG

gtaatgttgcatcgtgctcttgtcttgtgttcattttcttttccttccacatcattggca

cgtaatcaaaccacttactaatggtgcactttgaagtgcactgctcctttgcttcatatt

aagatggttcggatttcaaataatgggataattatcatgttatgaatttggtaagagttg

attatctggatggtgaaccggctatataaacttgagtgtgaagtgagtgatcacaggagt

tcaaagatctactactgtaatctattttaaacttgagtgaagtgactgggaacttaataa

ccggttttctcaacggagttggggttacaattgatatatataggttatcagatcagatgt

taggtcccgtgcgatactttatcatcactttgttagtcgaggttctgttccgtaatctaa

gtgagggcttaatcagtatgcgttcagaatgttgatacttgagacccacttcataatttg

gttgcttttttgccactcagttagcacacctgtatgcctttctcattatatgttttaata

ttagtaaggcaagcctgcgttattgctgataagcatcttcgcttttgcag

GTCAACTATTGTTCACTCGACTCCAATTCCTGCGAAGGACACTCGGCCACTCTTTGGCAA

GCCTTGCAGCAAAACTTCCTCAGAGACAGGGAGGTATGCTGGGAACGGTCACGGTGCTTC

TCAGGCTTCACACGCGGCACAAGCAG

gtgcctctgcgttcattctgctatacatacatattttgtcagcatttacttgaaacttaa

tgtcagagttgcgtatttcgcag

TGGTTTCACGGAGAGCTGCTGGTTCAGCGTTACCTTACGAAGGTGGAAGCGGTAAACACC

CTTACGACGTGGCAAGCAGGCCGGCGATGAGCACAGGATGCCCTCCTCAGCAGCAGATCC

CACAAATGTATGGGCAATATCAGCATCAAGCGCCTGCTGGCGCTGGTGCTGGTGCTGGTG

CTGGGATACCACAGGCCATGGGGGGCTATGCTTGTGGGGGTTACGCCAAAGGCACGGCGC

CTGCTCCGGCCATGAGAGCACCCACCTACCGACATGTTCCGGCGGGGCAGAAGAACGGTC

GTCTGGACAGACTGGCGGTGGAGACCACCGACATATACACACGGTCGCTCAACGGCATCG

TCGCCGCTGCCGCGGCATCGGCAGGCACCGCCAGCGCCCACAGGAAGGTCGGCACCGTCC

CGTTTGGCATGCCGACGACTTACTAGCGGCTATGAAGACGGCAGCGCCGCGTCTCCAGAC

TCGTTGATCGGAACGGTAGCAACCGGAGGCATTGGCCTGAAATAAGTTGGCGGATGAGGA

TCGTCTGGACGACGACGAAGGCATACATAGATGAATATGAAAACCTCTCTAGGCTACACA

AAAAAGACGGAGGGTTTGGCCAGATGAGCTGTTTATTTAGGTGGGAATGAAAATTAATTA

GCTGGTTAGCGGCTGGAGGCAGGTCAACTCAACACAGAAATGAAGTCTGTAATGTGCTAC

TGTACTATCCAACAGGCTAATTAATCAATTAATTAAAGGGATGATGGTCCATTGATCATC

ATCATATGATTCATTCACTCGTAGATCTACCATGCATTCATGCTGGATGGAAATGTTAGC

CTAGTATTTTGATTGTAGTTTTGTAGTACTACTGTCATATGAAGCACATTGTAGTCTTGA

CCAATCATATCACTGGATATC

**>HvMPK20-4 : MLOC_4609.1**

GGCACGAACCCGATCGCTCGCCCGAGACGAACCAGACGAGCCGGGACGAGGAGCTCCTTG

TGTCTTGCCCTTTGCGTCGCGCCCCCACCCACCGGGCCACAGGACGAAACCAACGCCACA

ACAACCAACTCACACCCCTTCCCCACCCCCCCGCGACTTGCTCAGTCGCAGCGAGCCAGG

CCACAAGCGTTCCGGTTCAGGTTCCGCTTGGGGTTGCGCTCGTTTCATCGCCGATCCGCC

CACCCCGCCATCCCGTACTCCCCCTCCCGGGGACAGAGATCCCCACCTCAAGCGCGCGCG

CGCCGGCGGTGACAAATGCGGGTTGATTCGCCGGCGGAACAGCGGCGGTGAGCGATGCAG

CCGGACCAGCAACAGCACCAGCAGCGGAGGAAG

gtgagaccgtgcgccaggggttgctgtttgttgcggggtggttgcctttcggtgcccgtg

cggggttgttggcgtttcgagatcggttcgtccggcggggtgggtcgacgaacctacctc

gcctcgcctcgggggagggtttctttttttctttttcatttgaggggtcgcactggactc

gtgggatccggcgcgcacgcccgtcaggatttgctcttgccattggcgccctgaagtaaa

ggtttgatttcagctgcggagtcgctgtgaattctcttgttccatttctttattaggaaa

aaaggggtccttttcactcctgtttctggatcacaattcatcttctcaattataatatct

gaaatcctcttctgcggttgggcttgggcagcgtcttgttttccgcctctaaaggaaatg

atattcttttccaacttttccattccatgctagtagttgaatactcattgctccttaaag

gatcgttgaggccttttcaaccctctaatcaacaccaggagaatgaaaagaaaaaggctg

attcagatgcaacctaagaggtgccgcaaacgcaaatgcggtattagattcgctgagtag

tctagttatgtttatctcaactcaacacaattctgtggtcttcttaccgtcctttttctt

gcaaaaaattatagccaatcatcttaattgtctcagtggattaggttaggttgtcaatat

attctatgcccagttgggaggcatattgatattgatatagatggttttatgcccaagaac

acatccttaaatagtcattcttgggccctttctgctcgataaacactgttttcctcattg

ctccagaccaaggaggtttgcatcggacagagagttgtagctaccgcctgcgtgtgatcc

agaagctaacggaatccaactttttgtgctagaatctctctgagtcagttggatgcagat

tacaagtctgctgtttcagttgtctgttccttaggaactaggaagtgacaaattttgact

gctgtcttgaggattttggtaatttgcttgaaaaaactgggtttaactttgtgccccctt

cctcacccccctcaaagtttgtttcatttaagcctatatatgtgggtagtatgtattatc

acctggggtttatccaaaggatgatctgaactttttctgggcttttactctaaattgggc

cttgtttctgtatgtatgttttttgtttcagttgtggaaaggctgtccattgtgcagtat

taactcccattttttcctcttctctcag

GGTTCATCGGATATGGACTTCTTCAGTGAATACGGCGATGCTAATAGATACAAAATTCAG

GAAGTCATCGGTAAAGGGAGTTACGGCGTCGTCTGTTCAGCTATTGACCAACATAATGGC

GACAAGGTGGCAATCAAGAAAATACACAATATCTTTGAGCATTTATCTGATGCTGCTCGG

ATCCTCCGTGAGATCAAATTACTCCGCCTATTGAGACATCCTGATATTGTTGAGATCAGA

CATATAATGTTGCCTCCATCAAGGAGGGATTTCAAGGATATTTATGTTGTCTTTGAGCTG

ATGGATACAGACCTCCACCAGGTCATCAAGGCCAATGATGACTTAACCAAAGAGCACCAC

CAGTTCTTTCTCTATCAGATGCTTCGTGCACTGAAATATATTCATACCG

gtatgctgctttttgaatttgggatccgagttaacatacagttttctattgtcgtgttat

aatctttcttgaatatctaggagtagttatcttatccatactctatccgtactttactgc

tgatgctttgctccgctccatttggagcacacaatttggagaaatgcactcatacataaa

atcactctagtgccaaacttataaaggcacatacattaattgtgccctatgccaccgtct

tgcgctaagtgtacgcttaagcccaattatacgcacattaagcacgattaagtgctatgc

gttttgctatcacctagagactaggtgtgctttttctctagaatagggcgccttttttca

ctaagtgttaacccaataatcaaggccgacatattctagagataattctcctttgtccca

cattatttatgtttttgttgaccatttgtcttctgatactttttatatttgacctatgat

tgacctgaaaaatatctcccactttgtttcacactttttgttgagaaatgtacacttcct

tgtatttacctttaaggttatcatgattattttccactatccattgttgagctactaaca

ttgttcatttactatgtctgtgatctgacaaatatttatgtgattgcag

CTAATGTTTATCATCGTGATTTGAAGCCAAAGAATATATTGGCAAATGCTAACTGTAAAC

TCAAAATATGTGATTTCGGGTTAGCACGAGTGGCATTCAATGACACTCCCACGACTGTAT

TTTGGACG

gtatgtaaattttggtagactagaatcacatattgtgtggccaaattagatttgtattca

aaggtagtcttttggcacttaatttttatcttctattcatgattgtttgcag

GATTATGTTGCTACTAGATGGTATAGGGCTCCTGAGCTTTGTGGGTCTTTCTTTACTAAG

gtaattagtctgcacacctctgttctgtttgctaggataccgtgtgcactactttccaca

gaaacatgcctcatgcaattgctgggctgggtatgctgtttaatactattcctactcaca

tagtaataaaagaagataatcattgtctacagctgttctgtacagtgcatatatacatgg

catgagaccgaattcaaggttcatatgtctattagatcctacgtagtcaactggtcttgc

taataaaatggcaatgccaacccttctagagctactgtttccacaactatatgctagtta

tctttaatacctgatctttccactctttaaaaggctgtgatgttgaattcaggatgctaa

atcggttgttctgtgcag

TATTCACCAGCTATTGACATATGGAGTATTGGTTGCATTTTTGCGGAGATTTTAACTGGG

AAACCTTTGTTTCCTGGTAAAAATGTAGTTCACCAGTTGGATTTAATGACTGATCTCTTG

GGTACGCCGTCACTGGATACAGTTTCCAGG

gtatgcttttccattcatgactcatgatccaaagtaaggtatccataaatgtcaagcatc

ctaatccctgtgcaaccgcag

ATCCGGAATGAGAAGGCAAGGAGGTACTTGAGTAGTATGAGGAAAAAACAAACGGTATGT

TTTTCTGAGAGGTTCCCTAAAGCAGATCCTGCTGCACTCAAACTTATGCAGCGGCTTTTA

GCATTTGACCCGAAGGATAGACCAACGGCTGAAGAG

gtaagtgtttccagataaaacttcagacttgaaaacaggtgttaaaaactgtcattctaa

caatgtttatttgatcataatcag

GCGTTAGCTGATCCATATTTTAAAGGCCTCGGGAAGGTAGAGAGAGAACCATCCTGCCAG

CCAATATCAAAATTCGAGTTTGAGTTTGAACGAAAAAAGGTGACCAAAGAGGACGTAAAG

GAACTTATATTCCGCGAGATATTGGAGTATCATCCTCAACTTCTCAAGGATTACATGAAT

GGAACCGAGAAAACGAACTTCCTATATCCTAG

gtttgtaccataagatctcttccatttgttttcattacataccaattttcataactgatc

cattcttggttcctacgttattatgaag

TGCTGTAGACAATTTCCGGAGGCAATTTGCCAACTTGGAGGAAAATGGAGGGAAGGGAGG

GGCAGTCGTTCCATCGGACAGGAAGCATGTTTCACTCCCCAG

gtacccactgcaagtattttacccatattatatgctgctatgttctgctttctgttttta

aaaaagatgccattaaattaaatgaatagctcatatgtgcatggttaaaatatagttctt

tggtgatctcatggtactgtggaatcgctttgcatgacaaaagatggtcaggtagtcagg

aatttcagttcaagtttttgatgatttggcatgataatttcagagactgtcaataaactt

tgcttgctgacatttagtttgcattttgcag

GACTACTACAGTTCATTCTACACCAATTCCTCCAAAAGATCAAAAGCCTTCCCAAGTTCC

CCAAAGGATTCCAACAG

gtagtagtttctcctaagtagatgtctagtgttctattttcatcagatggtaccaatatg

atatatatcctttgctccaatattcacag

GTAGACCAGGAAGAGTGGTTGGCCCGGTAATACCATTTGAGAATTCATGTGCTATGGATC

CTTACAGTCAACGAAGGGTGGCAAGGAACCCAGTACTTCCTGCAGCTGCTACCAACCTAT

CAGCATATGCATACCACCGAAAGTCAGACAATACAGAGAGAGAGTTACAGCAGGAGCTTG

AAAAAGACCGCATGCAGTACCAGCCGATGCAGCGTTTCATGGATGCCAAGATGGTCTCCC

CTGACTTGAGGTCTAGCTCCTATTACATGCCAAAGGGTGTCCCAAAGGCTGATGTAGCAG

AAAGGACCGCTTTGCAGCCAAACATGATGCAGGGAATCGCCCCGTTTAATGGCATTGCTG

CAGTTGGAGGTAGCTACAATAAGGCCAGTGCTGTTCAGTATGGAGTTTCAAGGATGTACT

AAGTGGTCCAGTGCAATGGCCACATCCAAATTACTTGGCTTAGCTGAAGTAATAACATGG

AAGAATCCAAGTACTTCCTGAGTTGCCTTATGGGCCAAAGATTCAGACAGTGGATCGATG

ATCTTTGATCGAGGAGGAAGTTGATGTCTCGTAAAGAACAAAAATAATAATAAGATCATG

CAAAGACAACATCCTGCCTGTTGATCTAGCTGCCAAGGAAACAATGACATTTGGTAATTT

GCTGCCCAGGGGAGAAGCCGAAGTTGTGGGTACAAGATGATTGAGGTCTATGTACTTTCA

AAGAACGGAAACATATCGGAATAATGAAAATGGTGAACTTCTTGTACATAAATAACTTGA

TTTTTCTTTTGTATTGCTGCCGGCGAGAGCCGTCAGTTTTGCTCCATATTCATGTGGTAA

TGTGATTTATTTTCTCGGTTTGAAGCACTTATGCAGTTATGCTTGTGTAGAAATTCAGCG

TTGTAATACTTTGGAGTATGTTAGATCTGTAATAGAATAACAAGACCAAACTTCAGTAAG

ATAGAATGGTAATGAACTGCTAGTTTTGGGGTTGGTTGGCTCAAC

**>HvMPK20-5 : MLOC_36752.2**

CGAGGCGCGGCCTTCTCCCCCCGACCCGGCGGCCGGCGGCCCGTGCTCCCCAGGATGCCG

GAGGCAAATGCGGGTGCCCGCGGCGGCGGCGAGCAGCGCAGCAAG

gtggggggaacggacggacagagtcgctttccgggccgaaagacaccttctttcttcctc

ctcttcttttttatcttctgccttgtcggcgccggccatgggttcgttcgtggtgagcta

cggtggtgctctgcttctgcggcgtggctttcggcgccggccatgggctccttctcggat

ttgggccgggtaaagatgtggatttcggggaggattcggacggattcggttcccccccaa

acaagaggaggccgcgatttcttgccttgctagctgagcggaggccccgcgtccaaaatc

caaatcctcgggacaggcggcaggatatctgcggcatatccgctcgctctctttcctatt

caattccttcctggaaatctcccggttctgcgccggggagacccctcttttggcagatgt

tgatgaggtgtcggtcggtcggtcgccgtggttggtgaatcgggcatcggattcggtgac

cgggcaagtttatccaaatctgcagttactttccttttagccagagatggatagcgcata

tattcagtccagcccgtggaatccgtgcctacccaggagttacctataagtttccccgta

tttttttcagccaaagggatatatcctttttcagtagcggtcgttcatcagactcctgac

attcttgttcttcgaattggttgctgtcagttttctgattttgggtgggtgagtgcaggc

caaggaagttctgactatggcgcatcagcagcagttacacctattgttaggattaattct

agattgtgaaaatatgtttttgtttatctgaactactgtaaccatggttgagtgtgtggc

tgtttttccaagccaacgtctgatctggatgtctttttcttttgcaaaagagaattttgc

catgtgcacggtgctaactttttccaccaattttcaactttcag

AGCTCAGACGTGATGAGTTTCTTCAGCGAATATGGAGATGCCAGCAGATACAAGATCGAA

GAGATCATTGGCAAAGGGAGCTACGGAGTCGTGTGTTCAGCCATCGACCGGCAAACCGGA

GACAAGGTGGCGATAAAGAAGATATCCAACATCTTCGAGCATATCACCGACGCCGCCCGG

ATCCTCCGCGAAATCAAGCTTCTCCGGCTTCTCAGGCACCCCGACATCGTCCAGATCAAG

CACATAATGCTGCCTCCGTCCAGGAGGGACTTCAAGGACATATTCGTTGTCTTTGAGCTC

ATGGACACCGACCTCCACCAGGTTATCAAGGCCAATGATGACCTCACAAAGGAGCACTTC

CAGTTCTTTCTATACCAGATGCTCCGTGCCATGAAATATATCCATACCG

gtaattaatttattaaggatcctcttagttggagattccctacccccctttttttcctga

aagagaggtcttcgagttctatttatagtatcatcacagtggtatgtctagagttaggag

tactattaacagttctgactagtgagtttgcatgccaatttcacttcaaccaatgatcga

tgatagtcctattttcatctgcattgcttgaaatgtttatcatctcttgttaagtatgat

agtcctatgttctgatgattaatcttcgtggctgcag

CTAACGTTTATCACCGTGATTTGAAGCCAAAAAATATATTGGCAAACGCTAACTGCAAAC

TCAAGATATGTGATTTTGGACTAGCAAGAGTTGCATTCAATGACACCCCTACAACTGTTT

TCTGGACG

gtatgttacttctacagtttacaactagattttatccacaatactctacttttgtgaatt

ctggttgacgaattggatagcatgctacgttctgtgccaaagaaccctacttgaaactta

aatctcaccgtcttctccaacatgatatgcag

GACTATGTTGCGACTAGGTGGTACAGAGCTCCGGAGCTGTGTGGTTCTTTCTTCACCAAG

gtaattaataatttcctcttcctgtggttttgttcattttagtactgtttgtggcatttc

gctacttcctcgttctagagggttgtatagcctaaaattctaggcgattagtagattttg

atccttattctctaatctttggatacatcactgtgctttagaggagtatggcatcgtagt

tataacaaggtgtttgttcaagcactaaagttgtgtacaaattgatatacattacttagt

gttacatttgccatcattcatttataccttttcagtttctatgttaacttgatgatacta

agttgactctttcaatgcag

TATTCACCGGCTATTGATACATGGAGCATTGGTTGCATTTTTGCGGAGATCTTGACAGGA

AAGCCTTTGTTCCCTGGTAAAAATGTGGTTCACCAGTTGGATTTGATGACTGATTTCTTA

GGCTCACCATCGCCTGACATTATTTCTCGG

gtatgatttaccgtttgcacaaatgttgtcaatcgagtacttctagcataaggtccagtt

ctctaatattttttatcaccgcag

ATTCGAAATGAGAAGGCAAGGAGGTATCTGAGCACCATGAGGAAGAAGCTGCCAGTACCT

TTTTCAGAAAAGTTCCCCAACGCAGATCCTGCAGCAGTCAAGCTCTTGCAAAAGCTTCTA

GCATTTGATCCAAAGGACCGACCGACTGCTGAAGAG

gtaggcatgcatgtcttagtgaaacatgaatttcatgttagcattttgtgctctcatgtg

ttctactacaaaatcgtactgtacattctaggtccatgcgtaattctaacaagttttctg

tgcatgatcag

GCGTTGGCTGACCCCTATTTCAAAGGTCTTGCGAAAGTGGAGAGAGAGCCATCATGCCAA

CCGATTTCGAAAATGGAGTTTGAGTTTGAACGGAGAAAGTTTACCAAAGAGGAGGTCAAG

GAACTTATATTCAGGGAGATATTGGAGTACCACCCTCAGCTTCTCAAGGATTACACCAAC

GGCTCAGAGAAAACCAACTTTCTATATCCTAG

gtttctaccttgacatagtatctttattaagctcttttctcctttttattctgtccatct

atgtagcaatgctcattatgtctcttgaattttccgctatgcag

TGCCGTCGACAACTTCCGGAGGCAATTTGCTAACTTAGAGGAAGATGGAGGAAAAGGCGG

GGCACCCGAGAGGAAGCATGTTTCTCTGCCGAG

gtaacttacactaatacaattcttctgaatgtgatcaactctactatctggttctaaatt

atttgtgccgccgcatacataacttgtgttaaaactaccaaaatgaattagttaatcaat

aagcttctgaaagatgtatccttgaataaattggcacaccgtggtactttacagatcttg

aaaaatctagaagtgtggagccctagagtacagaagttatgttggagtttcactattaac

ccttgatgatgggctgcttgcag

GACTACAACAGTTCACTCTACCCCAATTCCTACAACAAGTGGTCCGGCATCCCAAGCTCC

TCAAAGGATCCCAACAG

gtaacttgttctgtttaatttggtctggtacgtacctcctcatttgagcttcagagagag

caccagtcttgacacatttcatctctgacactattgcag

CTAGACCAGGCAGAGTGGTTGCCTCGGCGACACCGATCGAGAACGCGGCCTTCGCCGATC

GACAAACGGGTCGAAGGATGTCGAGGGACCCTGCGGCGCCTCCAGCAGCAGCTGCCGCGG

GCTACACCCTGAGGCCGGATTGCCCCGACAGGCAACAGCAGCAGCAGCAGCAGGAACTGG

AAAAGGACCGCACGCGCTACAGGCCGGCGCTCCATTTCAGGGACGCCAGGGTCGCGCCCG

AGGCCGAGGCGCGGCCCTCGGCCTACTACATTCCCCCGTTCAACGGCATAGCCGCGGTCG

CCGGTGGGTACAGCAAGGTCGGCGCGGCCGCAAGGATGTACTAGGGCGGGCAATGGGCAC

CGGAGGACGGTGGCCGGGTCGCCGCTCCAGCGAAGGCAGGGGCTGGGAGGGTGAACTCGT

CTCAAACTTAGGAGGTACTAGGAAACAGTGGAACAGGTGGTGCTCTTGTCTTGTGTACAT

ATTACCGGCATTCAAGCAATTGGTTGGTTGGTTCCGTGGTCTGCTGCTGCTGCTGTTTCC

TGGGTTGATAGATAGATAGGTTGAGAGTTGAAACAGTATGCTCTGCTCTGAGAGGTGAAA

TGGCCTCTGTTGAGGAGGGAGGAGGGGGTTGGGGGCTGTAGAGTTTTTCGACTTTTTTGT

TTTGTTTTGTTTCCGGATGGTGTTAATAGGATGGATGAATAAATGGATGATGGGAGCTCT

GATGGGCTTCTCCGTTGGTCAATTCTTGTGTCTGCTGCTGAATCTGTTGTACAGTATGAT

TTTCTCCTTTAGGCTGGTCATGGTGGGAGTAACTTAGATACAGTAGTAATATAACGCATT

CTAA

**>HvMPK21 : MLOC_11730.2.X (based on MLOC_11730.2)**

CCCCCTGCTCCCCGCGCCCGCGGCGACCGGGCAGCCCACCCACGCCCGCCGCCCAATGCG

CCGGCCCACCCGCGGAATAGGCAGCGGTGTATGGGCGGCCGCGCCCGCTCCCTCATCCGC

TGGCTGCGCCACCACCGCTCCCGCCGCGTCTCCTCCGCCTCATCCTCGTCCTCCTCCCAC

CTGACCCATACCAATACATCCTCCGCGACCACCGCCACCAGCGACCTGCGCGCCCGCTCG

CTCCCGCTGCAGCAGGACGAGGACGACGACGTCGAGGCCGGCTGGGAGGAGGAGCAGGAC

GAGTTCGCCGAGGGCCCCGAGTCCGACCCCGAGGGATACATTGTGCTCGAGCGGGAGGGG

GACGCCGGGAGCCTGCGCGTCGTCGTACCCCGCGCGCCTGCGCGCACCAAGCCGCCGCCA

CGCATGGATCCCGGCAAGAAG

gtgcgttcttgcttgggtcgccgtgcccccctcttctccgtaggccccttccgcgccgat

aactttccgccgttccggttcaatgccggctgccatcgatctctctcggttgaaatatgt

tgccgtactgccgggattgctccgttcgcttcatggattgaggaaaatccatccgtggga

ttgctctgttcttgctttcttttggttcttgcggatctgttcgtctggaatcgccgattt

tgacaaggtgacggtcaatgcggcatccagaagccatttcttggatatttgttatactca

ttaatgtcataagtataataaactcgtaatagtattagcttgtgcaaactgcgccacaca

tgaacgccgtccgtgacaacctaataaagcgcggcatttagggtgaaacagtgtcacggg

agcactgctttcgctgttcatgcctcatctgagacaaacatgatgcatggtggaagaggt

tacgcttttgcttttcaattattgttcctttgtggacgattccacgcttgccacctttgg

tagcagatagatatgtgagaaagatactgtttgaagtaccaaatgttcaaccacttcgtg

attcattcagtccaatcccagcaaaagcaagaagctttcttgagtgactttccagttgtg

tgcatgtcttctgatccctttttgcgtttgtcaatcaagagggcatccgtatcatcaatt

gagatgtcctatctttctttttctcgacgcgttttaaccaccaccggtgctggtggtaaa

tcagtatgttaatcgcctaaaaaagatcagtatgttagcacgctctgaactaatggtgcc

aatatgcgtttgctccttccttttcag

ACCTCGGAATCCGAGTTCTTCACGGAGTATGGTGAATTAAACCGGTATCAGGTCAGCGAG

GTCATTGGCAAAGGGAGTTATGGTGTTGTGGCTGCTGCTATCGACACCCAGACCGGCGAG

CGTGTGGCCATCAAAAAGATCAACGACGTCTTTGATCACGTCTCCGATGCCACCCGCATC

CTTAGGGAGATCAAGTTGCTCCGGTTGCTGCGTCACCCGGACATAGTTCAGATAAAGCAC

ATTATGCTCCCCCCTTCAAGGAGGGAATTCAGGGACATATATGTGGTCTTTGAGCTGATG

GAGTCCGATCTCCACCAGGTAATAAAAGCGAACGATGATCTCACACCAGAGCATCACCAG

TTCTTCTTGTATCAGCTGCTCCGGGGAATGAAGTACATCCATGCAG

gtgactggactccacaaaggaaatagataatggttcattcgacctttttcatctcatgtt

gctgtactgattgtttgtttacatgatggttttgcag

CGAGTGTTTTCCATCGGGATCTTAAGCCCAAGAATATTCTAGCGAATGCTGACTGCAAGC

TGAAGATTTGTGATTTTGGGCTTGCCCGTGTATCATTTAATGACGGGGCTCCATCAGCCA

TATTCTGGACG

gtagccattttagtaatattgagcagatagtgtttatcctgagcagccaaatcttggttt

ctgatgattttgcatgccttgtctag

GACTATGTTGCAACTAGATGGTATCGTGCTCCAGAATTGTGTGGCTCTTTTTTCTCAAAG

gtgatgtaattttcatcctaacttcatattaccaacaacaatacccaggctattaaaaaa

gcacaatgaccatactacagattttcttccaaaattgctgttttaatatgatgtgtttcg

atgcaacatagcaatactatttgttgatttcactagtaacacatgttgattattttccat

gtttgcaactaaaattcccttcccttttagagttgctactacagttctcaaatgtcggtt

caacctgtcatgatctgtatttcatattctgcactaagttctctcccagtaaaaatgcta

aaaaattgttacttatggtgttttgccctcttctttcatataatcatagctgaatgtagc

gacatggcttgggcgctaattcgaacttgatgatgtacttggttcttaattaatctatgt

gcatgtttagcaaatgctaaaagcagctgaactttgaattttgcag

TATACTCCTGCAATTGATATTTGGAGCGTAGGATGTATCTTTGCAGAAATGCTCACAGGG

AAGCCACTCTTTCCAGGGAAGAATGTTGTCCATCAATTGGATCTCATGACTGATGTACTT

GGCACTCCTTCAGCAGAATCTCTCGCAAAG

gtatgcttactccaccagcaagttagaacagcagttggacttatatattttgccacttcc

tatttttcataatttcataatatgcgaatttcctgccaaattgaaccttgcattgataac

ttgtgtacatggttattaatttattattggcttattccatttctctcggtatattttttg

catccacgcccaatattttcaagttctgattttccttaatatggttgcgtgcttagttat

ggtgcaacatggactacggatgtgggcacgaattggcatcaatatcagcacaatctcttg

ccatgttatatatttttcatatagaatgaaatgtaaactgtgggatgatgtttaaaccat

atgtcttttacacccttttactgaatggttgcag

ATACGGAATGAGAAAGCTCGGCGATACTTGAGCAATATGAGGAAGAAACCTAAAGTTCCC

CTTACCAAAAAATTTCCAGGCATCGATCCTATGGCTCTCCATTTGCTTGAGCGTCTTCTT

GCTTTTGATCCTAAGGATAGGCCAACTGCTGACGAG

gtaggaatgactgagaccaacttctctttactataaagatatattacatctttgaaaatg

ttggattaatcactgtcctcaatactgatatgtaaatgttcttcaag

GCCCTGACAGACCCATACTTTACTGGATTAGCAAATTCAGAACGCGAACCCATAGCACAG

CCCATCTCAAAACTTGAGTTTGAGTTCGAGAAGAGAAAGTTGGGCAAAGATGATGTCCGA

GAATTAATTTACAGAGAG

gtacaattgctcagctattttgtggagatacagtagagctgctgcttgactctgtagtta

actaacaacttctacttctttcag

ATTTTAGAGTACCATCCTCATATGTTGCAAGAATACCTACGTGGAGGAGACCAAATGAGC

TTCATGTACCCTAG

gtgagaccattatttccccttagaaacaagatttacttgtgttgagatgttatgatgact

atgctttactctgttctgcatacactgaattatgctagttatatgtatgattggttgatg

ctatattgcaaactctgatgtttgtgcatgtatgttgacag

TGGGGTGGATCGCTTTAAGCAGCAATTTGCTCATTTGGAAAAGGGTGGTGCAAAGGGTGA

TAAATCGAGTCCACAGTTGCGACAAAATGCTTCCTTACCAAG

gtaatatgttgtgaccgagacttaaatgtatacttccaaggcaatacatatctttatgta

gaaggggcatgtggagacttatggatgaaattatttgttttctaaccaaaatttctttcc

tttgacaaccttgtacaattttcctaaagatgatctgtttggacttggcaagaatattca

acaaaataaagacttcatcataagtatcatatttgttttgcttttttgcaagttgccaga

gatttaaaatcaaatatttttaatgttaaatttgatagagaataatgtttcacatgatta

gatgtttatatatataaaaaatgagtcatcctataaatagacaaccttttaagtttttgt

atctaacaattctcgcatcttgtctcag

GGAAAGAGCAATTGGCAATAAGCACGGAGATAGTGAGTACCAAGTAAAGCTGAATACAGG

TGAGAAGCCAGTACATGCATCAGTGACAGATGGAATAAGCAAACCCCTCATGAGCGCTCG

GAGCTTACTGAAGAGTGAAACCATGAGTGCTTCCAAGTGTATAGGTGAAATAAAAAATAA

AGAT

gtgagttcagctcatcctttgttacaacagtcaatatatttgcccctcttttcttatgaa

aattgctggtatatttgaattccatacttgcaggctgtttacccacttttttcccccctt

tcgagtgtttaattgtctatatctaagatatctctatctaatttgagcaatttcttagtt

aatgctgcaatgtccattcaacattttccag

GATGAATTGGAGAGCGTGGATGCAGCTGACGGTGTCTCTCAGAAGATTGCTCAACTGAAA

ACCTGATTTTCGAAGGTTACGAAGCGATCGGTAGTATATACTATGTACAACAGCATGTCT

ATAGGCGCTCAAGATCAGAATGATCTTTAACCGAAGCCAAGAATTCTATACACCAAATAA

GACCACAAAATCATCTGTTATGGTAACCTTGTTGTGCTGTAATGTAAAATTTTCTACTTG

TTTTTTCTACTTTGTCAAAGAACCATGTCTAACCGACAAGCTAGTTCCCCGTACTTTCAT

CGGAGGAGCTGCCATGACCCTGTACAATTTTGTCAATATGAAGTACCACGCCCCTATATT

TCTCTTCACATGGT

**>HvMPK24_n-term: ASM32608v1:6:62507485:62509104:1**

ATGGAGTTCTTCACAGAATATGGGGAGGCAAGCCAGTATCAGATCAAAGAGATCATTGGC

AAGGGAAGCTTCGGAGTAGTTGCTGCTGCAGTAGATACCCAAACTGGGGAGTGGGTTGCG

ATCAAGAAGATACATGATATGTTTGAGCATGCCTCAGATGGCACCCGCATTCTGCGGGAA

ATCAAGCTTCTTCGGCTTCTCCGCCACCCAAACATAGTTGAGATCAAACACATCCTGCTT

CCCCCTGCCCGAAGGGAATTTAGAGATATTTATGTTGTTTTTGAGCTCATGGAGTCAGAC

CTACAAAAAGTGATCCAAGTAAATGACAACCTCACTAAAGGGCATCACCGGTTTTTCTTG

TATCAACTTCTTCATGCCCTCAAGTACATCCACACAGGTTAGTTCAGAAACCGTCGGCAT

ATCATTCACCTTATCATCAACATTCACGTCTTATCAAGTGTGAATCCTCCGTACAAGTCT

TATTTTGAAACCTCATGAAACCCTGTCTTACTTTTCTACGTACAATTTATCTTCTCGCAG

CTAATGTATTTCATCGTGACTTAAAACCGAGCAACATACTTGCCAATTCGAACTGCAAGC

TAAAGATCTGTGACTTCGGGCTTGCACGCGCATCGCGTGATGATGTTCCCTCAGCTATAT

TTTGGACTGTAAGGCATTTGTCACTGTATTAAATAATTATTTTCTATTTCTCTCCTCAAG

TTACTTACCTAGTGCACTTTGCAATTTGAAGGACTATGTGGCTACAAGATGGTACCGTGC

TCCGGAATTATGTGGCTCATTTTTCTCCAAA

**>HvMPK24_c-term : ASM32608v1:6:47704546:47706434:-1**

TACACCCCTGCGATTGATATTTGGAGCATAGGGTGCATATTTGCTGAAGTTCTCACTCGA

AACCCATTATTTCCTGGGACGAATGTCACACACCAACTAGATCTGATAACAGATGTCCTT

GGAACTCCATCACATGAAACCCTATCCCAGGTATGTGTACATATTACCCATTCACCAATG

GATAAATATTTCACTGTTTTCCTGTTTTTTGTTAGTACCATACTTGGTTGCTTTTTACTG

GTGGTTTCTTTAAAATGAAGCATATATAGACTTGGCCACAGATAGAAATCTGACTTTCTG

TAAAATGCAATACAATGTGTCAGAACTACTTCCCCGACCTGGCTCACAAACACATGACTT

CTGTGTGGTTGGTTCTCTAAGTAAATCGTTTCCTAGATACTAACCTGTTTGAGAATTTTT

GCTACTTCTAGCACATGCCATTGCACAGTAGGGCTCAGGCAATGAATTTCTTAACATGGC

TATGACAACTGCAGATTCGCAATGAGAAGGCCAGGAGATATTTGACTGGCATGAAGAGGA

AACATCCTATCCCCTTTCCTCGTATGTTTTGTAGTGCTGATCCTCAGGCTGTCCGTCTCC

TAGAACGCTTACTCGCATTTGATCCTAAAGATCGACCTACTGCTGAAGAGGTATATTTTT

GACAATAAAGAGTCCTTGCTTTTATGATAACTGATTCCGACATTGATGAGTACATGAGTG

TAATATTTTGTATTTGTATTCTAGGCTTTAGCCGATCCATATTTTGAAGGACTTCCTAAG

TTGGAACATGAGCCTTCACCATACCCCTTTTCAAAACTTGATTTTGAGTTTGAGAGATGG

AAGCTATCAAAGGATGGTATAAGAGATCTGATATATCAGGAGGTAGAAAACCATCACTTC

AGTGTATGTATTAGGCTGACTATATTTGACTAACTTAAAATTTGATTTTTCCTTCCCTTG

AAAGCAAACAGATTTTGGAGTACCATCCGCAGATGCTTCAGGATTACATCAGAGGTGGAG

GACAGACTAGTTTCGTCTATCCAAGGTTAGGTCGTGTTGCCATCTGCTGCCAGCACACCA

GCACTAGCGTAGATGCTTAGCATACATATGAAATTGCATGATTTGTATGCCCATGTCACA

TTTTGGTTTCACACTGAAAACCTAGTTTAATACCCCTTATCAGCAGTATAATATTGTTCA

TAATCTAATACTATCACGTTGCAGTGGGGTTGATGGTATGAGACTGCAGTTTGCACATGC

TGAGGATAAGTACCTCAGAGGAGAAAGAGGTACTCCACTGCGGAGGCGACATGCATCTTT

GCCAAGGTACATGGGTCGTTATTAGCCGCTAGCAGCTTGAACTTATTGTAACTGCACAAA

TTCTTGTACAGTTCTGATTGCGCTTAGCTTGCTTCTGCACTCTTCTTCATGTGACAGGGA

AAGATTCCGTGCGCCAATGGGTAGTGATAATCAAGACTGTAACAATGAGAATAGGAGGAC

AGCATCTTCTGCTGGACAAATTATCATAAGACCACAACAAGTGGGGATGACGCATGCATA

TGTTTATCAAAATGGCACAGGCATTCCGGACTTCTGCTCAGGGTATTACTTGCAGAATGG

TGGTACCAGTGATTCCAGTTGTGCTATCGGGGAGAATGAAGGCCCCAAGGTACCTCCCGG

CACTAATACAACGACACTGCCTAAATTCTACCCAGGATCTTATAATTATCAGTGCACAGT

CACTCTTCTCGTGAGGACTCGTGTTTATGCGAGAGACTAACCAATAGCCAATCTGTTTTC

TGTTGTTGTTTTCCACTCTTCAGGAGAATGGCGTCTCCGAGGAGGAGAAGGTGGCCTATG

AACCGTCAGAACTGCTTGCCAAGATTTAG

>HvMPK25 : ASM32608v1:5:449484666:449486231:-1

ATGGCGAAGATGGTAGACCCCCCGAAGGGCAAGGGAAGCCACGGGAAGCATTACTACACC

ATGTGCCAGACGATGTTTGAGATCGACACCAAGTACGTGCCGATCGAGCCCATCGGAAGA

GGAGCCTACGGGGTGGTCTGCTCGTCGATGAACACGGATATATACGAACGAGAAGGTCGC

GATAAAAAAGATAAACAACGTCTTTGACAACCGCGAGGATGCGTTGAGGACGCTGCGCAA

GCTGAAGCTCCTTCGCCACTTGCGTCATGAGAATGTCATTGCTTTGAAGGATATATAATG

ATGCCGATGAAGAGGAGGAGCTACAAGGATGTCTACTTGGTGTCGGAACTCATGGACACG

GATCTGCATCAGATCATCAAGTCGCCTCAAGCCCTTTCCAATGACCACTGCCAGTATTTC

CTTTTTCAGGTATGCGGCAGTCACCTCTGTTTTTCATTCTTTCTTTCTTTTTGCTTGACA

CTTGTCCAGTGATATTTCATTTCTTCTCCGTTCTGTCATAAGTTGGTTCATTCACACGCA

CCTCATGCTCCATCCGGAAGTAATGCCTCTCGGCTTGCTTCGCTAGACACAGCCAATCCC

ATGAGTTATTTGGATGTAATGCCTATATCTTTAGTTTTTTACGCCGGCCATCTTACGATT

TCTCTAGTATTTTTCAATCGATCAATGGTCTAAGATGCCGCCTTTTATTGACTTTAGCTG

CTCCGAGGGCTCAAGTATCTTCATTCAGCGGGGATACTGCATAGAGACCTGAAACCAGGA

AACCTTCTGGTGAGTAATGCTACACCTACAAAGGCTTACTTAAAGATTTTACGTACAAAC

TGATGTGTAAGATTGTGATTGGTAATTAAGGGATGAGGGGCCCCACCCCCATTGAAAATC

AGGGGGGAGAGAAGAGTTAGTTTGGAAGGTTAAGTAAACTTTTGTAAGTTTTTGTAGGTC

TAGCATTATTGCCTTCTGGTTAACGGGAACTGCGACCTGAAGATCTGTGACTTTGGTCTT

GCTCGCACAAATAACACCAACGGTCTGTCTATGACTGAATATGTTGTCACCCGCTGGTAT

AGAGCTCCTGAGCTGCTGCTCAGCTTCGACAAATACGGCACCTCCGTAGATGTCTGGTCT

GTTGGCTGCATCTTTGCTGAGCTACTTGGCCGCAAGTCCATCTTTCCTGGAACCGATTGC

CTAAATCAGCTTAAGCTTATAGTCGATGTGCTTGGCACCATGAGCGACGCGAACCTTGAG

TTCATTGGCAACACGAAAGCTCGCAAGTACGTCAAATCCCTTTCGTACACCGTCGGGGTT

CCCCTCACCAGAATGTACCCACAAGCACACCCTCTTGCCATTGATCTATTGGAGAAGATG

TTGGTCTTCGATCCTTCCAAAAGGATCAGTGTCACCGAGGCTCTGGAGCACCCATATATG

TCTACGCTGTATGATCCCAGCGCAAACCATCCTGCCCAGGCGCCCATCGATCTCGATATA

GATGAGAAGCTCGGCGTGGATATGATCCGGGAAATGTTGTGGCAGGAGATGCTCCAGTAC

CCCTGA

**>AetMPK3 : EMT08915.X : F775_52499**

atggacggcgctccggtggccgagttccggccgacg

ATGACGCACGGCGGCCGCTTCCTCCTCTACAACATATTCGGCAACCAGTTCGAGATCACG

GCCAAGCCCCAGCCGCCGATCATGCCCATCGGCCGCGGCGCCTACGGGATCGTCTG

gtacgttccgcccgtcttaatttgctcctcaatcaccatcgatttcttgccaagattgtt

catgtcgttgcggctccgatcggagaacagaggggggattggctcgggccatggggaaag

ataggattaggccaattcactcctttctgtgaacgacactagtttagtactagcactata

actttatcagttgagcaaatatgtgaatctgtcagtgctctgtggttggtggtgtttaat

cttgtcctgaaattccacttgtggttgcgtgggcgcag

CTCGGTGATGAACTTCGAGACGAGGGAGATGGTGGCAATCAAGAAGATCGCAAACGCTTT

CGACAACAACATGGACGCCAAGCGCACGCTCCGGGAGATCAAGCTCCTGAGGCACCTCGA

CCACGAGAAC

gtaatcaacaactatcttttccgcttcagatgtgcgtatctcattgaattcggcaattca

ggcgctaagctgttccgctacatggatgcag

ATAGTAGGCCTCCGAGATGTGATCCCGCCGGCGATCCCGCAGTCCTTCAACGACGTCTAC

ATCGCCACCGAGCTCATGGACACGGACCTCCACCACATCATCCGCTCCAACCAAGAACTC

TCGGAAGAACACTGCCAG

gtactagcaaggaggggaaataagccattcattttgttcatgtttgtgacaatttgatcc

gtatgtatattcatgcgacgtggtgttcttgggtggctttcag

TACTTCCTGTACCAGCTGCTGCGCGGCCTCAAGTACATCCACTCGGCGAACGTGATCCAC

CGCGACCTCAAGCCGAGCAACCTGCTGCTGAACGCCAACTGCGACCTCAAGATCTGCGAC

TTCGGCCTGGCGCGGCCGTCGTCCGAGAGCGACATGATGACGGA

gtacgtggtcacgcggtggtaccgggccccggag

ctgctgctcaactccaccgactactc

cgcggccatcgacgtctggtccgtcggctgcatcttcatggagctcatcaaccgcgcgcc

gctctccccggggcgggaccacctgcaccag

ACGCTTGCTGCTCAACTCCACCGACTACTCCGCGGCCATCGACGTCTGGTCCGTCGGCTG

CATCTTCATGGAGCTCATCAACCGCGCGCCGCTCTTCCCGGGGAGGGACCACATGCACCA

GATGCGGCTCATCACGGAGGTGATCGGCACCCCCACCGACGACGACCTGGGCTTCATCCG

GAACGAGGACGCCAGGAGGTACATGAGGCACCTGCCGCAGTTCCCTCGCCGGTCCTTCCC

GGGACAGTTCCCCAAGGTGCAGCCCGCCGCGCTGGACCTCATCGAGAGGATGCTCACCTT

CAACCCGCTGCAGAGGATCACAG

gtgcgttgcgctccacgtccgcgtcactctgctttgatcttcacgtgtgaatttcgtagc

gccgcactgcactgcacgcaatgctgctgctgttagagcgccgtgctttgttgactggct

tcgtacggccagtgttggggtcagtgtcagtgctaaaaacatctttgtgctggttataag

taggaatagatgcaatctgcagccacttcggcccctatttttaagtttttactgcatgtg

gacatgtgctccctttttagacaaaaaccatgtgtgctgcccacttgattaagaaaagat

ctttgcctatctttggagctagtagtatgtgctgtagacgacaaggactgtgcgtgtagt

gcagaatcacactttttttagaagaacgtatctgtttttggcaacagatatgaatggaag

agatctgctgcagctactctcaaaggctagattcctgcatctgtttcacaacagcaataa

tagaagtagctttttgagacgtcggccagagaatagtagggaaggctaggaagtacagta

gtttctattttgttgttatacgtacttgatgtctgagtgacgcgcattctcactcaaatt

gctcttgtcacgtagaaagttactccctctgtaaagaagatccattcctaaagaaaaatt

atttaaggaggtttagattagacttttgttagcttttggccccccaaacactgaggattt

ttgtttggccccccaatgatcttcggctagctctgccactgcttgtactccagttaatta

gtgtctgactgatgtggattcttaatgaatttctttatggaggaagtatgtttcatcagt

taattagtgtctgactgatgtggattcttactgaaattgctcttgtgatgcag

TTGAAGAGGCGCTGGAGCACCCATACCTAGAGCGGCTTCACGACGTCGCCGACGAGCCCA

TCTGCACGGACCCCTTCTCCTTCGACTTCGAACAGCACCCACTGACGGAAGACCAGATGA
[truncated: 166,992 more chars]
