# Supplementary material for: Analysis of MAPK and MAPKK gene families in wheat and related Triticeae species
Source: BMC Genomics. 2018 Mar 5;19:178. doi: 10.1186/s12864-018-4545-9 (PMC5838963; doi:10.1186/s12864-018-4545-9)
Supplement: Supplementary file 5 — Genomic sequence of Triticeae MKKs. Genomic DNA sequences were obtained from Ensembl. Font colour of predicted genes differentiate UTRs (orange), exons (blue), introns (grey), sequences upstream/downstream of the predicted gene (green). Where Ensembl predictions for exon-intron structure did not match available transcript data, alternative exons are proposed and highlighted in yellow; corresponding accessions have an ‘X’ suffix. Black nucleotides are used for genomic sequence for which Ensembl did not predict a gene sequence or for predicted ncRNA sequences, where proposed exon sequences are highlighted in yellow. The underlined nucleotides highlighted in various colours indicate nucleotide variants as defined by Ensembl. Purple font is used for GenBank sequence that were used to fill in the gaps where complete gene sequence was not identified from Ensembl. (DOCX 274 kb) [file 12864_2018_4545_MOESM5_ESM.docx]

**Additional File 5. Genomic sequence of Triticeae** **MKKs.** Genomic DNA sequences were obtained from Ensembl. Font colour of predicted genes differentiate UTRs (orange), exons (blue), introns (grey), sequences upstream/downstream of the predicted gene (green). Where Ensembl predictions for intron/exon structure did not match available transcript data, alternative exons are proposed and highlighted in yellow; corresponding accessions have an ‘X’ suffix. Black nucleotides are used for genomic sequence for which Ensembl did not predict a gene sequence or for predicted ncRNA sequences, where proposed exon sequences are highlighted in yellow. The underlined nucleotides highlighted in various colours indicate nucleotide variants as defined by Ensembl. Purple font is used for GenBank sequence that were used to fill in the gaps where complete gene sequence was not identified from Ensembl.

**>TaMKK1-1(4AL) TRIAE_CS42_4AL_TGACv1_290148_AA0982230**

CTCGTCGCTCGTCACCAGCCCAGCCAAGCGCAGCACAGCAATTCAGCAAAGCAGAGCAAC

CCCCACCCGCCGTTTTTCTCCCCCCACCTTCCGCGTCCCCCCTCTCCACCTAGCAAGCTC

CGGCCGTCCGCCCGCGCCCGTCCCGTCCGTCCACGAGCGAGCCGGCCGGGCGCCGCCGCC

GACGCCGATGAAGAAGCCGGGCAAGCTCGCGCTGCCCTCCCAGGACTCCACCATCGGCAA

GTTCCT

gtaagcaatcaagctccggccgcccccacccctcttctgcccgtttctttctttcattca

ttcattcattcttcgggcgcgtcgccgtgctgatcggttcgattccggttgtggtcaccg

gggcag

GACGCAGAGCGGGACCTTCAAGGACGGCGACCTGCTCGTCAACAAGGACGGCCTCCGCAT

CGTCCCGCAGAGCGAGGAAGGCGAG

gtaagccggcccactcaacccggtcaggcacccccccccccccccccccccccccccccc

cccccccccccctaggtccaaaattaaaattccccaacccatcttgtcctggacacgcag

ggctgggcctggctggcagattggtacacagcctgtactgttctttttgtttttttgtaa

tttcgttgatgtcatcaacagatttaaaatttctttccggatgcgtgattggattggcag

caggtgccaagcagcgggttcctgtcctccggtggttaccttttcagaacaacgatttag

gaaaaataatgatacgcactatcgttaattttgattttattttattttcggtcaaaaatt

attatgtccctttttcagaccagccattcaggcaaaaatctaatcatttcttctcaagaa

gagatggcggccttgtatagatgtagtaaaactctactccctccattcctaaatataagt

ctcttttagagattccaatatagactaacatacggaacaaaatgagtgaatctacactct

aaaatacgtctatatacatccatacgtagtccatacagaaatctctaaaaaagacttatc

tttaggaacggagggaatatttttgatagtggccttgcatacatgagcagaaaaaagtgg

gacgtctgattggatcagagggcagggagcaggggtttcctatcccctaatttcagaata

actttttaggaaaaatgatgggagtactactatttactatttaggatttattttatttcc

cttaggtggtggaatacatgcggtcatctttcatgtggggatatcctcacatgcagttta

ttactgatgccatttatcatgcatccagtggttggtcatatttatccggaaatgctcatg

ctcacttgcttatactggaagagtgttctctgcacccatttttgttttgagaattgtcta

attgcattcctgttctgaacttattatactcttcatttatctagtctttgtctcaccttc

tttctgttgacatacacgacttctctag

GCTCCTCGTATCAAGCCTTTGGATAATAATCATCAGTTGAGCATAGACGATCTAGATTCA

ATCAAAGTGATCGGGAAAGGTAATAGCGGGACCGTGCAATTGGTGCGCCACAAATGGACT

GGCCAGTTTTTTGCTCTCAAG

gtattgtctcaaacatacattatttgatgcttcttctttaaatctttgctatgcattttt

ggttccttcaacttctttctgtaactatcctctctgctattttacatatctgttacaaag

ggtcttcacctccattggtgtatgttgtcatgcctgcag

GTTATACAGCTCAATATTCAGGAGAGCATACGCAAGCAGATGGCCCAGGAGTTGAAAATA

AGCTTGTTTACACAGTGCCAGTATGTTGTCACGTGCTATCAGTGTTTCTATGTCAATGGT

GTTATTTCTATTGCTTTGGAGTACATGGATGGTGGCTCTCTCGCTGATTTCCTCAAGGCT

GTTAGAACCGTTCCAGAGGCCTACCTGGCTGCAATTTGTAAGCAG

gccagtgaaatgtcacacaatcaaccacatagttttccctacttttttgtttgctatcag

aattgtcaccttaaaatgacatggaccttttcaattgacaattgcaacttccttgtgcag

GTGTTGAAAGGACTGATGTACTTGCATCATGAGAAGCGCGTTATACACCGAGATCTGAAA

CCATCGAATATATTGATAAATCATAGGGGTGAAGTAAAGATATCAGATTTTGGTGTTAGT

GCCATCATTTCTAGTTCTTCTGCACAGCGAGATACATTTACTGGCACATTTAACTACATG

GCG

gtgagtgaaagtttgtttaaattatgtagacatgccttccagtgtggcatgaaaagatgt

tcaccaatttttttgtttcagctaaccagtgtaaaatgtatactcttgacag

CCTGAAAGAATCAGTGGGCAGAAACATGGTCATCTGAGTGATATCTGGAGCTTGGGCCTA

GTTATGCTGGAATGTGCCACTGGCAATTTCCCATATCCTCCTCGTGAAAGCTTTTATGAA

CTTCTTGAAGCTGTTGTCGACCAACCATCACCTTCTGCACCATCAGACCAGTTTTCACCA

GAGTTCTGTTCATTCATTTCTGCTTG

gtatttgtgcttctccattgaaccccctttgttaatcaatgttactcgggcaatatttgc

aatttttagatctagagctctaaagtatgagcttaggtacaataccattatatgcttgca

gacacgtattatctgaacttccagatattttttgtttttctctcatctcatctgtgggca

gagtaattgatcttttatttatttatttatggaacatcagtgtccgaaagaaggctaata

tctgttttgttatttccttttggatttcag

TATCCAAAAAAATGCTGCAGATAGGTCATCTGCCCAAACCCTATCA

gtaaggaaatttctgtttgcttcgcaattttgtatgttcttctgaaagcatcattagctg

gctgactgatcttgatccatttcttgtatctgttgcctccgatgtccag

GCTCATCCATTCCTGAGCATGTACGACGACCTGAATATCGATCTGTCTGACTACTTCACG

ACTGCAGGATCACCGCTTGCCACCTTCAA

gtaatccaataacccactgaagagtacatacatggttactgcaattcctttacaattcca

atgatggtctattcttgttctttttactag

GCAAATCGCGTTGTGAGACGACAAATGCTCAACCATGTGTCATGAGCTGGAGCTAACTGA

AGCAAGTGAATGGAGTTATTTTTCTTTTCCTGTTATTCATGAACTGGGCTTTGATGATAT

CTTTGAACCAAATATGATACAATTGGCTTCATAATTATACAAATACAAGTCTGTAAAGCA

TCAGCGAGCATCGAAGTTTTGCCTGTCTGCATAACGGAAGCATCCGTTTGTTGTCCGACT

TTGACCTTTGTTATGTATGGGCTTATTGCTTTTGCAATTTTACTGTAATGAAACCATATT

TGCTGG

**>TaMKK1-1(7AS) TRIAE_CS42_7AS_TGACv1_570427_AA1835590.1.X (based on AA1835590.1)**

GCTCGTCACCAGCGCAGCAAAGCAGAGCAACCCCAACCGAACCGTCGTTTTTCTCCCCCC

ACCTTCCCTCCGCGTCCCCGCCCTCCCCTCTGAGGCCACCTAGCAAGCTCCGACCGTCCG

CCCGCGCCCGGCCCGTCCACGAGCGAGCTGCCCGGCCGCCGGCCGCCGATGCCGATGAAG

AAACCGGGCAAGCTCGCGCTGCCCTCCCAGGACTCCACCATCGGCAAGTTCCT

gtaagcaatccacctccgactgtccttctccctcctccttcagtttttcccctccttcgg

atgctttgctggtgcagtcgtgctgactggttttcttctccggcgcgggggcag

GACGCAGAGCGGCACGTTCAAGGACGGGGACCTGCTCGTCAACAAGGACGGGCTCCGCAT

CGTCCCCCAGAGCGAGGAAGGCGAG

gtaagccggcccactcaacccggcaccccctagattgaaaattaaaattccccagctcat

cttgtcctagacacgtagggctggacctgcctggcagattggtgcagcccgtactgtttt

ttgtactagtaatctcgttgatatcgtcagcagattttaaatttcttttgggatgcgtga

ttggattagcagcagggggttcctgtcctccggtgcttggttaccttttcagaagaacga

tttaggaaaaataatgatatgtactatcgttaattttaattttattttatttccctcaaa

gaacattatgtcccccttttcagaccagccattcagccaaaaatcgaatcaactcttctc

gacaagagatggtggccttgcatagatgtaaattgtacttattttttatagtgaccttgt

atacatgagattggatcagagggtagggatcaggggtttcctatcccctaatttcagaat

aacgtcttaggaaaaatgatgggagtactactatttaggatttattttatttcctttaga

tggtggaatacatgcggtcatctttcatgtggggatatcctcctatgcagtttattactg

ataccatttatcacgcatccagtagttggtcatatttatacggaaatgctgatgctcact

tgcttataccggaagagtgctgtctaccgccatttttgttttcggaattgtctaattgca

ttcctgttctgaacatattatactcttcatttatctagtctgtgtcttaccttctttctg

ttgacatactcggcttctctag

GCTCCCCCTATCAAGCCGTTGGATAATAATCATCAGCTGAGCATAGACGATCTAGATTCA

ATCAAAGTGATCGGGAAAGGTAATAGCGGAACCGTGCAATTAGTGCGCCACAAATGGACC

GGCCAGTTTTTTGCTCTCAAG

gtattgtcccaaacatacattatttgatgctttttttctttaaatgtttgctatgtattt

ttggttccttcgacttttttcgttacaaaattatggtggtgactgtaactatcctctctg

ctattttacctatatatatgttacaaagggtcttcacctccattgctgcactgttgtcat

gtctgcag

GTTATACAGCTCAATATTCAGGAAAGCATACGCAAGCAGATGGCCCAGGAGCTGAAAATA

AGCTTGTTTACACAGTGCCAGTATGTTGTCACATGCTATCAGTGTTTCTATGTCAATGGT

GTTATTTCTATTGCTTTGGAGTATATGGATGGTGGCTCTCTCGCTGATTTCCTCAAGGCT

GTTAGAACCGTTCCAGAGGCCTACCTTGCTGCAATTTGTAAGCAG

gccagtgaaatgtcacataatcaaccacatagtttccccttcttctttttttggctatca

gaatgtcgcctcaagatgaaatagaccttttcaatcgacaattgtaacttcctcatgcag

GTGTTGAAAGGGCTGATGTACTTGCACCACGAGAAGCGCGTTATACACCGAGATCTGAAA

CCATCGAATATATTGATAAATCATAGGGGTGAAGTAAAGATATCAGATTTTGGTGTTAGT

GCCATCATCTCGAGTTCTTCCGCACAGCGAGATACATTTACTGGCACATTTAACTACATG

GCG

gtgagtgaaagtttgtttaaattatgtagacatgtcttccagtgtggcatgaaaagatgt

ccaccaattttttttgttccagctaaccaatgtaaaatgtatactcttgacag

CCTGAAAGAATCAGTGGGCAGAAACATGGTTATATGAGTGATATCTGGAGCTTGGGCCTA

GTTATGCTGGAATGTGCCACTGGCAATTTCCCATATCCTCCTCGTGAAAGCTTTTATGAA

CTTCTTGAAGCTGTTGTCGACCAACCATCACCTTCTGCACCATCAGATCAGTTTTCACCA

GAGTTTTGTTCATTCATTTCTGCTTG

gtatttgtgcttcttcattgacccccctttgttaatcaatattacttgggcaatatttgt

aatttgtttagttctaaagtttaaaactatgagcttagctacaataccatcatagtgttt

tttctaccatttgcttgtgagttgtgaagtagtgattctctggaactggaatatgcttgc

ggacacgtattgtctgaacctacaatttgtttttctctcatctcatctgttggcagatta

attgatcttttatttatttgtttatttattatgcaacatcagtgtccaacagatggctaa

tatctgttttcttatttctttttggatttcag

TATCCAGAAAAATGCTGCAGATAGGTCATCTGCCCAAACCCTATCA

gtaaggaaatttctgtttgcttcccaattttgtatgttctttcgaaagcatcattagttg

gctgactgatcttgatccaattcttctatctgttgcctctgacgtccag

GCTCATCCATTCCTGAGCATGTACGATGACCTGAACATCGATCTTTCTGACTACTTCACG

ACCGCAGGATCACCACTTGCCACCTTCAA

gtaatccaataacccactgaagagtacatacatggttattgcaattcctttacaattcca

gtgatggtctattctcgttttttttactag

GCAAATCGCATTGTGAGACGACAAATGCTCAACCATGCGTCATGAGATGGAGCTAACTGA

AGCAAGTGAATGGAGTTTTTTCCCCCTTCCTTTTGTTCATGAACTGGGCTTTGGTGATAT

CTTTGAACCGAGTATGACACAGTTGGCTGCATAATTATACGAATACAAGTCTGTAAACCA

TCAGTGAGCATCGAAGTTTTGCCTGTCTGCATACCGGAGCCATCCGTTTGTGGTCTGACT

TAGACCTTTGTTATGTATGGGCTTATCGCTTTTGCATTTCATTTTTACTGTAATGAAACT

ACATTTTGCTGGGTCGCTTCATGACCTGATTTGGACTAGTATTTCCAAGCTTTTAGTTCT

GATGGTGATGTGACACTTCAAGTGGAGGGTGGTGGTGGGCTTCTGAAATCAGGAATGGTT

GCTTTCGTGAAGGGCAACTTATTGTAATTTGTCATTTCACAGAACTGAAATCCAAAATTG

ATCCATGCAAAGAAGAAATAAAATTATGTTATGTTGTGAAATAGTGTAATTAATTTAGTA

TACACAT

**>TaMKK1-1(7DS) TRIAE_CS42_7DS_TGACv1_622118_AA2033130.2**

ACGTCTAGAATGCTGGTGTTTTTCTAACGGAGTGGCTATTTTGTTTGCAAAATCGCAGCC

TTAAGATGTTCTCTGGAAGTTTGACACTACTTGTTGATGGGCCTTTCTTGTCACCTACTT

TTCTTCTTCTTACTAGCAGCACGCTTGCTGTGTAAACAGGTCTCTTGCATGGTACCTTTC

CACCCATCTTTAAAAATAATAAGCATGAAAATGCAGTAGAAATTATGGTTGAGCTTTTCG

AAG

gtgagctgagcatctcttggcactgggcttgccatgtatacgcagacaagatttgcatgt

agccggtttagcctttaatgtttacatcttggttgatgatgaattgatgatgatag

GGATGGCTCAACTCGGCGGAGAGGACAGGAGAGGAGGAGCCGGGGCTCGCATTTTCCACG

GTATTTCCATGTGGCCGGGGTGGGTAGTGGGCATGTGTGCGCCTCCCAAATTGGGTGGTG

AGGCGGCGTGAGAGCTACCCTTCCCTCTTCCCTGCTTGCCATGGCACTCGCACCACCGGC

GACCTGTAGCGCGCCGAGTCGTATACGCACGCCTTGTTATAATTCCTCTGGTGTACCGTC

TACACTGTCATCGAGTATTGTCCCCGTTACGCCAGCCATTTAATCCATGGAATTACGTGG

GCACCGTAATGCGCAATTTGACAGCGAAGAAAGGTCAAAGTGCAGACAGACGAGGTGGAC

CAAGACTCGTCGCTCGTCAGTCGTCACCAGCCCAGCGCAGCGCGGCAATTCAGCAAAGCA

GAACAACCCCCACCCGCCGTTTTTCTCCCCCCCACCTTCCCTCCGCGTCCCCTCCTCCGA

GGCCACCTAGCAAGCTCCGGCCGTCCGCCCGTCCACGAGCGAGCCGGCCGCCGGCCGCCG

CCGAAGCCGATGAAGAAGCCGGGAAAGCTCGCGCTGCCCTCCCAGGACTCCACCATCGGC

AAGTTCCT

gtaagcaatccccctccctccttcgtttgttttgttcttcgtggacgcgttccgtcgtgc

tgattggtttggtttcgcggcggccgtggtccggggggcag

GACGCAGAGCGGGACGTTCAAGGACGGCGACCTGCTCGTCAACAAGGACGGCCTCCGCAT

CGTCCCGCAGAGCGAGGAAGGCGAG

gtaaaccaaccaaccacactcaacccgggcaggcaccccctagattgaaaattaaaattc

cccagcccgtcttgtcctagacgcgtagggctggacctgcctcgcagattggtgcagccc

gtactgttttttgtaattccgttgatgccatcaacagattttaaatttctttccggtcgc

gtgattggattagcggcaggtgcccagcagggggttcctgtcctcccctggttacccttt

cagaagaacgatttaggaaaaataatgatatgcactatcattaattttaattttatttta

tttccgtcaaaaattattatgtcccctctttcagaccagccattcaggcaaaaatctaat

catttcttctcaagaagagatggcggccttgtatagatgtaaactgtactccctccgttc

ctaaatataagtcttttttagagattccaatatagactacatacggaacaaaatgagtga

atctacactctaaaatacgtctatatacatccatatgtagtccatatagaaatctctaaa

aagacttatatttaggaatggagggaatattttttatagtggccttgtatacacgagcag

aaaaaattgggaggtctgattggatcagagggtagggaggaggggtttcctatccaccaa

tttcagaataacgtcttaggaaaaatgatgggagtactactatttaggatttattttatt

tcccttaggtggtggaatacatgcggtcatctttcatgtggggatatccgcctatgcagt

ttattactgatgccatttatcatgcatccagtggttgctcatatttatccggaaatgctg

tggaagagtgctccattttctgtgtgggaattgtctagttgcattcttgtccttaacttg

ttataactcttcattcatctagtctgggtctcaccttctttctgttgacatactcgactt

ctctag

GCTCCTCCTATCAAGCCGTTGGATAATAATCATCAGTTGAGCATAGACGATCTAGATTCA

ATCAAAGTGATCGGGAAAGGTAATAGCGGAACCGTGCAGTTGGTGCGCCACAAATGGACT

GGCCAGTTTTTTGCTCTCAAG

gtattgtcccaaacatacattatttgatgtttttttctttatatgtttgctatgtatttt

tggttccttcgacttttttcgttacaaaattatggtggtgactgtaactatcctctctgc

tattttacctatatatgttacaaagggtcttcacctccattgctgcactgttgtcatgtc

tgcag

GTTATACAGCTCAATATTCAGGAAAGCATACGCAAGCAGATGGCCCAGGAGTTGAAAATA

AGCTTGTTTACACAGTGCCAGTATGTTGTCACATGCTATCAGTGTTTCTATGTCAATGGT

GTTATTTCTATTGCTTTGGAGTATATGGATGGTGGCTCTCTCGCTGATTTCCTCAAGGCT

GTTAGAACCGTTCCTGAGGCCTACCTTGCTGCAATTTGTAAGCAG

gccagtgaaatgtcacataatcaaccacaaagtttccccttcttcctttttcggctatca

gaatgtcgcctcaagatgaaatagaccttttcaatcgacaattgcaacttcctcatgcag

GTGTTGAAAGGGCTGATGTACTTGCATCACGAGAAGCGCGTTATACACCGAGATCTGAAA

CCATCGAATATATTGATAAATCATAGGGGTGAAGTAAAGATATCAGATTTTGGTGTTAGT

GCCATCATTTCTAGTTCTTCTGCACAGCGAGATACATTTACTGGCACATTTAACTACATG

GCG

gtgagtgaaagtttgtttaaattatgtagacatgtcttccagtgtggcatgaaaagatgt

tcatcaatttttttgtttcagctaaccaatgtaaaatgtatactcttgacag

CCTGAAAGAATCAGTGGGCAGAAACATGGTCATCTGAGTGATATCTGGAGCTTGGGCCTA

GTTATGCTGGAATGTGCCACTGGCAATTTCCCATATCCTCCTCGTGAAAGCTTTTATGAA

CTTCTTGAAGCTGTTGTCGACCAACCATCACCTTCTGCACCATCAGACCAGTTTTCACCA

GAGTTCTGTTCATTCATTTCTGCTTG

gtatttgtgcttctccattgaaccccctttgttaatcaatgttactcgggcaatatttgc

aatttttagatctagagctctaaagtatgagcttaggtacagtaccattatatgtttgcg

gacacgtattatctgaacttccagatattttttgtttttgtctcatctcatctgtgggca

gattatttgatcttttatttatttatttatttgtggaacatcagtgtccaaaagaaggct

aatatctgtttttttattgtttttttggatttcag

TATCCAAAAAAATGCTGCAGATAGGTCATCTGCCCAAACCCTATCA

gtaaggaaaattctgtttgcttcccatttttgtatgtacttttgaaagcatcattagttg

gctgactgatcttgatccaattcttctatctgttgcctcgacgtccag

GTTCATCCATTCCTGAGCATGTACGATGACCTGAATATCGATCTTTCTGACTACTTCAGG

ACCGCAGGATCACCGCTTGCCACCTTCAA

gtaatccaatgacccactgaagagtacatacatggttactgcaattcctttacaattcca

gtgatggtctattctccttttttttcctag

GCAAATCGCGTTGTGAGACGACAAATGCTCAACCATGCGTCATGAGCTGGAGCTAACTGA

AGCAAGTGAATGGAGTTATTTTTCTTTTCCTTTTGTTCATGAACTGGGCTTTGGTGATAT

CTTTGAACCGAGTATGATACAATTGGCTGCATAATTATACAAATACAAGTTTGTAAAGCA

TCAGTGAGGATCGAAGTTTTGCCTGTCTGCATACCGGAACCATCCGTTTGTTGTCTGGCT

TTGACCTTTGTTATGTATGGGCTTATCGCTTTTGCAGTTTTACTAATGAAACCATATTTG

CTGGGTTG

**>TaMKK1-2(6AS) TRIAE_CS42_6AS_TGACv1_485693_AA1550190.1.X (based on AA1550190.1)**

AAGCTGAGAGATGTTTGAGGGTCCCAATAGTCATATCGCTTGGTGCCATGGGACCCCAGC

TTCTTTGGGAGCTCAACATGGCCTCATCTGATGCAGGCTGCCTATCGAAGAGGGATGGGG

GGGGGGGGGGGGGTGAGAAGACCGAGAAATTATAGTGGTAATTGAGTAGAGGGGCACCTG

GTAATTATAGTGGTGGAGATCATGGAAGAGAAGGAATCTCCTGAGGTGCACAAGAACGTC

GTCCTCTCAGATCTAACTCCTCCTAACAGTGGTGGCCCTTTCACCAA

gtaatcgcatgcaggactacttaacttgttttctttcgatgggcataagaatggataatt

gtcatgccatgtctgcattgtgaccacgagattgtcgtcttgttttgattgcttgttcct

tgttaaattctcgcgggtgcccgatttcaacattgtgcattttgctttgtcacacagttg

atggttgcctttctatagtatacagggcacatgtgtacgcaatatccattaattaatttt

acctttgtttatgtgcctgtgtttag

ATGTTTTGGGCGCCATGAGACCCCTTATTGGGAAGTTGGACATGCTTCTCCTTAGGGATG

CTCCTCAGAAATGCTGCTCCAAGAGGATCAAGGACAGGATGCGCCTCCTCAAGGATGACG

TTCAAAAGATAAGTTCCTACCTTGATGAACTATTAGAAGTGGAAGACCCTCCGCCAATGG

CCATGTGCTGGATGAATGAGGCACGCGACCTGTCTTATGACAT

gtaggattacgtcgacagcttattatttgtgccgcctgaagatccctccctttttgccaa

caacatcaaggccaccagatccctccgcaaatggttcagtcgtgtcaagacttcccagac

tcaggttattagtgcagaaacgttatcagaattcaggaggtatgtccaggaggccattca

acgccaccagaggtataatctccattcttgcag

AACCTTGAGGCGTAGCTTTGTGTCCCATGGCCCTATGGTTCTTCCAAGGCCATATGAAGA

AACTGCAGACATAGTAATCGATGGCCGGATGAATGAATTTATCAACTCACTGGCTACCGA

TGGGGACCAGCAGCTCAAGGTGCTTTCTGTTCTTGGATCTGCTTGTCTTGCTAAAACCAC

ACTTGCTAGAGTGTTGTACAACCGATTTAGGAAGCAATACAATTGCCAAGCTTTCGTTCG

AGTATCCAAGAAACCTGATATGAAGAAAATTTTCTGTGACATGCTCTCGCAACTACAGCG

GCAACATCCTCCGCAACATTGTAGGGAAATTGATCTCATTCACGATATCAAGCAATATCT

ACAAGATAAAAG

gtagtgccttttttcttctttcaagatatatcaaaattcccatgttccctaaactctaga

gttgattatctgtatgcag

GTATTTAATTATTATTGATGATGTATGGGCTGCATCAGTATGGCATACTATTAATCATGT

TTCTCCAAAGGGTAATCATGGCAGCAGAATAATAACAACTACACAGATCGAAGATGTTGC

ATTAACATGTTGCTGCTATCAGTCAGAGTATGTTTTTGAGATGAAACACCTAGATGATGG

TCATTCTAGAAAGCTTTTCTTTAACAGACTTTTTTGCTCTGAAAGAGATTGTCCTGAACA

GTTCAAAGATGTTCTAAATGAAATAGTTGAAACATGTGATGGTTTGCCGTTAGCCAGAGC

CAGCATAGCAAGTGTTTTAGCAAGCCAGCCTGTCATGTCAACTGATCTTTTGACATACAT

CCATCGGTCCTTAAGCTCTTGTTTCTCGGCATCAGAAAGAACTAGACAAGCACTGAATCT

GAGCTTTAACAGTCTTCCTCAATATTTGAAGACATGTTTGCTTTATCATAGTATGTATCC

AGAGGGCTACACAATCTTGAAGGATGATTTGGTGAAGCAATGGGTGGCTGAAGGTTTGAT

CTATACGACAGAAGGGCAAGACATCGGGAAAGTTGCAGAAAGCTATCTCGATCAACTTAT

TGGTAGAAGATTCATCCAACTGATATGTGCCAACTACAACAATGAGGTGTTATCCTGTGC

AGTGCATGGCATGGTACATGATCTTATTGCACACAAATCTGCAGAAGAGAATTTCATTAT

GGCAATAGACTACAGCCGAAAGAAGGTGTCACTCTCTCAAAAGGTCCGTCGACTATCTCT

GCTCTTTGGTAATGCAAAATATGCCAAGACACCAGCAAACATCACAAAGGCACAAGTTCG

GTCACTTGGATTTTTTGGATTACTCGAGTCTATGCCTTGTATTACAGAGTTCAAGCTTCT

TCGTGTCCTGAACCTTCAACTGTTCGGTCATGCTGGCGATGACGTTGGCCCTGTAGACCT

CACTGGAATTTCAGAAATGTTTCAACTGAGATATTTGAAGATTGCAGGTAAT

gtctgcataaaactgccaaaccatgtgctacaatgtttggaaatactggatattccggat

gcaagagttgcttgtgttccacgagatatccatttaccaaacttgttacaccttggtctt

cctgttgataaaaatctgctggattggattaacagcaggatgtctctcagtgtggagagt

atgcaagctgcaggatcttcacctcaccagatcttctgcactttcttctgaccatctgaa

tagaagcatgagttctattatatctttacttggaggacatggcaacctgaaaactttagt

agtggctcatggctcatcggctaaaaatgctcgtggcgtttcagacgtcaccctgtcatg

ggatctcctggcacccccacttctccagagatttgagttctcgccacatagccatatcat

gttctcacgaattcctctgtggattgagaaacatggcaacctaagaattttgaagattgc

agtgagggaactccagatgagttgtgttgatatcctcag

AGGATCGCCTGCCCTCACTGCTCTCTCTCTCTGTATGTGGAGAAGGCGCCCTACGACAAG

ATCATCTTTGACAAGGCTGGGTTCTCAATTCTCAAGTACTTCAAGTTGAGGTTCACGAGT

GACATAGCTTGGATAAAATTTGAGAAAGGTGCAATGCCTAATCTCTGGAAGCTCAAGCTA

GTTTTCAATGCCATCCCCTATTTTGAAAAAGATGGTACTGCACTCATCAGCATCGAGCAT

ATGCCAGGCCTTAAAGAGATCTCCACAAATTTTGGGGGTGAAGCTCCTGATCTAGAGTAT

GCCTTGAGGACATTGGTTAGTAATCATCCGACCAATCCTATAATTAACATGCCATCAAAT

AAGACTCTGGGGGGAGAACAACCACATAGGAATCTGGAGCAAGAATTAGATGAGATTCTG

AAGGAAGATCCATATGGGAATCTGGAGCAAGAATCGGATGAAATTCTGGAGGAACAAGAG

CCAGATGAATACGATGAGAGATTAGAGAGGCGACAGGCTGATAAAAG

gtactacannnnnnnnnnnnnnnnnnnnnnnnnnnnnnnnnnnnnnnnnnnnnnnnnnnn

nnnnnnnnnnnnnnnnnnnnnnnnnnnnnnnnnnnnnnnnnnnnnnnnnnnnnnnnnnnn

nnnnnnnnnnnnnnnnnnnnnnnnnnnnnnnnnnnnnnnnnnnnnnnnnnnnnnnnnnnn

nnnnnnnnnnnnnnnnnnnnnnnnnnnnnnnnnnnnnnnnnnnnnnnnnnnnnnnnnnnn

nnnnnnnnnnnnnnnnnnnnnnnnnnnnnnnnnnnnnnnnnnnnnnnnnnnnnnnnnnnn

nnnnnnnnnnnnnnnnnnnnnnnnnnnnnnnnnnnnnnnnnnnnnnnnnnnnnnnnnnnn

nnnnnnnnnnnnnnnnnnnnnnnnnnnnnnnnnnnnnnnnnnnnnnnnnnnnnnnnnnnn

nnnnnnnnnnnnnnnnnnnnnnnnnnnnnnnnnnnnnnnnnnnnnnnnnnnnnnnnnnnn

nnnnnnnnnnnnnnnnnnnnnnnnnnnnnnnnnnnnnnnnnnnnnnnnnnnnnnnnnnnn

nnnnnnnnnnnnnnnnnnnnnnnnnnnnnnnnnnnnnnnnnnnnnnnnnnnnnnnnnnnn

nnnnnnnnnnnnnnnnnnnnnnnnnnnnnnnnnnnnnnnnnnnnnnnnnnnnnnnnnnnn

nnnnnnnnnnnnnnnnnnnnnnnnnnnnnnnnnnnnnnnnnnnnnnnnnnnnnnnnnnnn

nnnnnnnnnnnnnnnnnnnnnnnnnnnnnnnnnnnnnnnnnnnnnnnnnnnnnnnnnnnn

nnnnnnnnnnnnnnnnnnnnnnnnnnnnnnnnnnnnnnnnnnnnnnnnnnnnnnnnnnnn

nnnnnnnnnnnnnnnnnnnnnnnnnnnnnnnnnnnnnnnnnnnnnnnnnnnnnnnnnnnn

nnnnnnnnnnnnnnnnnnnnnnnnnnnnnnnnnnnnnnnnnnnnnnnnnnnnnnnnnnnn

nnnnnnnnnnnnnnnnnnnnnnnnnnnnnnnnnnnnnnnnnnnnnnnnnnnnnnnnnnnn

nnnnnnnnnnnnnnnnnnnnnnnnnnnnnnnnnnnnnnnnnnnnnnnnnnnnnnnnnnnn

nnnnnnnnnnnnnnnnnnnnnnnnnnnnnnnnnnnnnnnnnnnnnnnnnnnnnnnnnnnn

nnnnnnnnnnnnnnnnnnnnnnnnnnnnnnnnnnnnnnnnnnnnnnnnnnnnnnnnnnnn

nnnnnnnnnnnnnnnnnnnnnnnnnnnnnnnnnnnnnnnnnnnnnnnnnnnnnnnnnnnn

nnnnnnnnnnnnnnnnnnnnnnnnnnnnnnnnnnnnnnnnnnnnnnnnnnnnnnnnnnnn

nnnnnnnnnnnnnnnnnnnnnnnnnnnnnnnnnnnnnnnnnnnnnnnnnnnnnnnnnnnn

nnnnnnnnnnnnnnnnnnnnnnnnnnnnnnnnnnnnnnnnnnnnnnnnnnnnnnnnnnnn

nnnnnnnnnnnnnnnngattctgaaggaagatccatatgggaatctggagcaagaatcgg

atgaaattctggaggaacaagagccagatgaatacgatgagagattagagaggcgacagg

ctgataaaaggtactacaaatgttccttgtaagcacaacgaaatatgccctcctttactt

caacttctatgaggctgggtgcacccacttggtgcggacgcctcaatcaataaaatcttt

cttttaaagaaaaacatgtatgtcgattcttcggttaacctgatataattcttatatgtt

ctaacgaagctaatggttattgtaatcaatcag

GATTTCAAGGTCATCAGATCCATCTTCGCGTCTACATGTTCCAG

gtatattttctgtcaactggtggtaatataaatgtttcgccatcaagttaataaagtcac

ttcaagagaaaactaagcagcttgttctatgtgataaaaaaatttgtcccggactaccaa

ttcagaaatgattaatttctccaaccgctgacttgttagagatagaggcttgagcggaaa

ccggtgaccgtagtcaacccatctagtttagttgcatccgtccgtccatgacgttggcct

ctagttacaaataagactttgatcctgtgccagccgaatcagaaaagaatccctgattgc

acgtagcctggatttcaacctcctattttgtcttgcactttcttgtttcattgcttgctc

aagcgagatgcatcttcccctattttcttgcccccaaccaatcacattcacggcttggac

tgcagcatgcacaccatgaaaacatgcctaatttccggtcctatctcttttcacttatgc

accttagttatgtgctaaaatagcatgctttatcagattaaaaaatagcatgctttattg

ttatacaaactaattcctctttttcttcgtaattgagttgcatatctcttcacacaacct

cagtaacgatagcaggggtatgcgtaagcgaagccaccgtatcactttcctgacaaaaag

accttgggacatctggggtggttctttgctgcttcttttcaatcgcgtcgtgtttcatcg

ctcagtcgaccatggagagcagtctcctttcttagttgctactttggttaattcttgggg

gcgtgttgggttatatatatactctctcttcatgcaggcacatgacattttttttattaa

gcag

TACCAGTTG

gtgcttctgagatgggaagggagcctgatgcggaatccctgcggcaccaactactaatga

taacatgatgttgtcggtcaatctcgaag

CATTTGCTAGGGAAAATTATCTGAGCACTAACTTGGAAAGTTCTGTAAAGGAGATGGAAA

GAGTGGCAGGTTTAGAGGATCATCAGATGGAAGATAGCACACAG

gtaatttacacattccaactgttttgaattcttctgatccttcttagggacgtagctact

tgggttggggtgaagttaggaagtaatattcttgaggtaaataaatatatttaagtgact

agtatggaaggtggtagatccgccttcgagccttcggttctgcagggtgtgtgcctagat

tgtttcttctaaattaaattgctacaggggtagtgattttatagctcacaaagagaagtc

taacaatgtgcctttgagtgggtaatcaaaacaattttttatttttaactctaaggctat

ccaggatctactcgcagatgagtatgaggttgatttagaaaatattagatattgtgttag

aagtttagcttcttcaagtagacctaaatggtaggacgtggagtatctgctcccgggctc

aaatgctccatggtgaacagtaaattagaaaaaaaaagagtaaacaaattcaacaaattc

tgaaaaacaatcaggtaaactttgacaaatattttgtgtgcttgcatatttatcatgaca

ggacattcgtggaagtcgtggcgaaaaaacaaaatcgatgctcccaaaatgctattttcg

aaagcattttggagtactaaattttttttgccatgacttccacgaatgttatttcgtgat

gaaatttgtcaagcataccaaacacttgccaaagtttgtcacaatttttatttttatttt

tcgattttactgttcatcccgagcttattcgagctcgggagcagaagaggacttctgtta

atggtaaaggtgcttttgttggaggtagtagaggtatagaagcgataattcaaaatggct

cccaagagcacgtgcttctggctgcaagaactaatttttcggatgtccaaaaaatgaaaa

aataaatggatgtctattttttcgtgtcctttgtgtacatgtaaattttcatgagaaaaa

aaaacttttgttgcgtgtgtataaaaaaacaattttgatgcttcaagaaatgcttcttag

agcactttcttgcttcaagaaatgcttgtgggaatagtattttaactaagtacaatatta

tcaaaaggggctggaaaggtaaaactgaatagatatacccatattaaaagcacatgttga

gtttcgatcatttttatctcagccatcaacggcattttccaggagtatctcagccatcaa

cggcattttccaggagtatctcagccacaattggcttttccttgtattactgagaattaa

atttggacggaaaaggagggagacaagtcccacgctctcgatacggcgttaccactatga

gtttgtggaaattaccnnnnnnnnnnnnnnnnnnnnnnnnnnnnnnnnnnnnnnnnnnnn

nnnnnnnnnnnnnnnnnnnnnnnnnnnnnnnnnnnnnnnnnnnnnnnnnnnnnnnnnnnn

nnnnnnnnnnnnnnnnnnnnnnnnnnnnnnnnnnnnnnnnnnnnnnnnnnnnnnnnnnnn

nnnnnnnnnnnnnnnnnnnnnnnnnnnnnnnnnnnnnnnnnnnnnnnnnnnnnnnnnnnn

nnnnnnnnnnnnnnnnnnnnnnnnnnnnnnnnnnnnnnnnnnnnnnnnnnnnnnnnnnnn

nnnnnnnnnnnnnnnnnnnnnnnnnnnnnnnnnnnnnnnnnnnnnnnnnnnnnnnnnnnn

nnnnnnnnnnnnnnnnnnnnnnnnnnnnnnnnnnnnnnnnnnnnnnnnnnnnnnnnnnnn

nnnnnnnnnnnnnnnnnnnnnnnnnnnnnnnnnnnnnnnnnnnnnnnnnnnnnnnnnnnn

nnnnnnnnnnnnnnnnnnnnnnnnnnnnnnnnnnnnnnnnnnnnnnnnnnnnnnnnnnnn

nnnnnnnnnnnnnnnnnnnnnnnnnnnnnnnnnnnnnnnnnnnnnnnnnnnnnnnnnnnn

nnnnnnnnnnnnnnnnnnnnnnnnnnnnnnnnnnnnnnnnnnnnnnnnnnnnnnnnnnnn

nnnnnnnnnnnnnnnnnnnnnnnnnnnnnnnnnnnnnnnnnnnnnnnnnnnnnnnnnnnn

nnnnnnnnnnnnnnnnnnnnnnnnnnnnnnnnnnnnnnnnnnnnnnnnnnnnnnnnnnnn

nnnnnnnnnnnnnnnnnnnnnnnnnnnnnnnnnnnnnnnnnnnnnnnnnnnnnnnnnnnn

nnnnnnnnnnnnnnnnnnnnnnnnnnnnnnnnnnnnnnnnnnnnnnnnnnnnnnnnnnnn

nnnnnnnnnnnnnnnnnnnnnnnnnnnnnnnnnnnnnnnnnnnnnnnnnnnnnnnnnnnn

nnnnnnnnnnnnnnnnnnnnnnnnnnnnnnnnnnnnnnnnnnnnnnnnnnnnnnnnnnnn

nnnnnnnnnnnnnnnnnnnnnnnnnnnnnnnnnnnnnnnnnnnnnnnnnnnnnnnnnnnn

nnnnnnnnnnnnnnnnnnnnnnnnnnnnnnnnnnnnnnnnnnnnnnnnnnnnnnnnnnnn

nnnnnnnnnnnnnnnnnnnnnnnnnnnnnnnnnnnnnnnnnnnnnnnnnnnnnnnnnnnn

nnnnnnnnnnnnnnnnnnnnnnnnnnnnnnnnnnnnnnnnnnnnnnnnnnnnnnnnnnnn

nnnnnnnnnnnnnnnnnnnnnnnnnnnnnnnnnnnnnnnnnnnnnnnnnnnnnnnnnnnn

nnnnnnnnnnnnnnnnnnnnnnnnnnnnnnnnnnnnnnnnnnnnnnnnnnnnnnnnnnnn

nnnnnnnnnnnnnnnnnnnnnnnnnnnnnnnnntatgttggaaatgatatgcataaaact

gttccaatcttttactaactccaggggcagtagtactagtacaatcaggctcatgtcctg

ggcctgcagctagtgattctgtagcgtggcatcttatagccggacctcgtacagccacca

gattccagagctgtcaacatggggacaggaaccgcaacacgccaggtttgctggactagc

cgttgatttttgctgtggaatctgggcccttggggatgaaagagaggttgacagtgagaa

ctgcctgtcctaagcgttcgtgtcactgtcaaggcggtttccgtcagagcttccccggag

acaaacagtgtggttcagtttgcagagaaatcacatcatgttggttagccatcgggtcac

gaatgtattattgatctttttcatctttatctccggctgaatttgcagctgtctctaaca

tcatcgttgtctccagcttatcccgtctaggatgctggagtttctaatgaggtgtttact

atgtgaaattgcaatcttttggtttctagaactttttttaggaagttcaaaacctcttta

cgagcttttcttctcaccttttctttttgcggggtgcttttattcttactagccttgcta

tggtaaacaaacaagcttttcacatagggcctagaataacgctttttttatccattgtta

aaaatgatcagagtatagaaatttgacttatccggacgagaaaataagacatcacaaata

ttgtggcttttcaaaagtgagcttctcttctcactacacttttcatatagacaagctttt

catgtagcacaccacgtctttttggtggctatagccggttttaacttttttaagatgata

gcttccttaacatagcagagaggagccgggacttgcattttctgcagtattttcatctag

ctgaatcagtagtggatatgtgggtggattcagcgccgtcttgcgcctcccaaaatggat

catgtcgttagtatcaaaagctttgctcttgggatagaataccacttttgcagtctcagt

agaatcctcttgaaaacaaaatctgtagttgcaacaacaacagacgatgccaaggtcttt

ttgattcaaataaaatttgaagtattgtagaggactgtaattcctaagaatcttactatg

aggtttgtttgattaagtaagattgcaattgtatgtttattttcgtctacatatcctatg

tatgtatgtagtatgtacatactgtcttattgtgcactgtgtatatttttccaagtcgtt

gaccaattatgccaaactgccaattttgccagccatttatactggtactgtaatgcttaa

ttccag

AAAAAGCAGAGCAAAGATGAGGCGGACGAAGACTCATCCATCCACTCACCCCGGCGAGGG

AGCCCTACACTGCCGCTGCTGTTGAAGAAGCCGGGCAAGATCGCTCTCCCCTCCCACGAG

TCCACCATGGGCAGGTTCCT

gtaacctcaaaccctcacccctcttctttcgcatttttgttttccttggacgtattgctc

ttgcagtcgtgctgattgatttgatttcacagtggttgtggtcagctgggctccagcgcg

ggacagggacag

GATGCAGATCGGGATGTTCAAGGACGGCGACGGCAACCTGCTCGTCAACAAGGACGGCCT

CCGCATCACCCCGCAGACCAAGGAAGGCGAG

gtaagccactaaaacatgaccgggcacccaagatgaaaaattcctccacgcgtctggtct

tagccgttcataggaaaagaacgtagggctgcgctcggcatattggtagttgtattggtt

tttgtaatttcattgatgacatgaacagatttagatattaggatgtgtggttggattagc

aggtacggagcagggtttcgtgttctttttcagaacaacaattgaggacaaatgatacta

tgtacttccttcgtccgggattagttgtcgctcaaatggatctatctagacgtatttcaa

tgctagatacatccgtttgagcatcaactaattccgtgcggagggaatacataggtgatt

ttcatgtgggatatccgcaaatgcagttgttacagaaataatttatgatgtatccagaat

gctgatgctcacttgcttatagtggaagattgctttctaacaccatttcttgtgggaatt

gtctaattacattgttgttcttaacttgttgtgttcttcattcatctaacttgtgtcttg

ccttctttccgttgccatactcggcttctctag

GCTCCTCCTATAGAGCCGTTAGATAACCATCAGTTGAGCATACATGATCTGGAAGCAATC

AAAGTGATTGGGAACGGTAGTGGAACCGTGCAATTGGTGCGCCAAAAATGGACTGGCCAG

TTTCTTGCTCTCAAG

gtattgcccaaaaaataaattatttgatgatttataattttggttccttctatttctttc

tgtagaaagtagaaactatagtggtagttataatctctgatattttgcctagggtcttta

cccccattcatgtactgtcatgtttgcag

GTTATACAACTCAATATTCAGGAAAGCATACGCAAGCAACTTGCCCAGGAGTTGAAATTA

AGTTTGTCAACACAGTGCCAATATGTTGTCACATGCTATCAGTGTTTCTATGTCAATGGT

GTTATTTCTATTGCTTTGGAGTATATGGATGGAGGCTCTCTGGCTGATTTCCTGATGACT

GCTAGAACCGTTCCAGAGGCCTACCTTGCTGCAATTTGTAAGCAG

gccagtaaaatgacacataagtaactacataggtttttcttgttatcagaatgttttttt

ttcaccttaagatcaaatagaccttttcaatcgataatttttacttcctcgagcag

GTGTTGAAAGGACTGAT

gtaacttgcatcatgagaagcgcgttatacaccgagatctgaaaccatcgaatatattaa

taaatcatag

GGGTGAAGTAAAGATATCAGATTTTGTTGTTAGTGCCATCATTTCTAGTTCTTCTGCAAA

GCGAGATACATTTACTGGCACATTTAACTACATGGCG

gtgggtgaaagtttgtttgacttatataagcatgtcttccagtgtggcatgaaaagacgg

ccaccattttttgtttcagctaccaaatctaaaatgcatatatactcttgacag

CCAGAAAGAATCAGCGGGCAGAAACATG

gttatatgagtgatatattatatatggagcttgggcctagtcatgctggaatgtgccact

ggcaatttcccatatcctcctcgtgatagcttctatgaacttcttgaag

CTGTTGTCGACCAACCATCACCTTCTGCACCATCAGACCAGTTTTCACCAGAGTTCTGTT

CATTCATTTCTGCGTG

gtatttgtgcttctccattgcctcccccctcctcccccctcccccccctggttaatcaat

aacacttgagcaatatttgcaatttgctgagatttaaactgtgagcttagttaccatggt

gggttttctaccttttgcctgtcaagtagtgattctcacgccgaaattcctgctctggaa

tatgcatgccaacatcatcatatgtttctttttttacactcacctagtcaccttcatctg

tggacagatcaattgattttcttattttatttactggaagtcagtgtccaaaaaaggcta

atatctttttcttaatttctttctgaatttttcag

TATGCAAAAAGAGGCTACAAATAGGTCATCTGCTCAAATCCTATCA

gtaaggaaatttctgtctgcttcacaatttgtatgttcgtccgaaagcgtcatttgacac

agcaagctcactgatctttacccatttcttctatctgttgcctctgacacccag

GCTCATCCATTCCTGAGCATGTATGACGACCTGAATATTGATCTTGCTGTCTACTTCAGG

ACCGCGGGATCTCCACTTGTCACCTTCAA

gtaatggtcctataaccccactgaagagtacttacatggttactgcaattcctttataat

tccaatgctgtcgtattctccttttcttcctagaggagtcgcgttgtgagtcagacaaat

gcacaaccatgttattccccagcgtggaattgatcatgcatgggtagttgatcatccatg

taccgtgatagactttttttcctacagtgatagcactgtcagattacaaacacaagttcc

tggcaaaaaaaaaggattacagacacaagttctcccggtactctactgataaagtgacaa

ccttaatctaaaaagagataatttgtgttctggccataaaaagaggcaattgatgatcat

cttataaccggcatcactcgtgtataagcatgtcagtgagccgcatcaacatagaaggtt

gtgccactacattttgttctcacgcttctattgcagaggcaactggtggcacggataatc

tcaaatttgaagaatccatttcctttacaaaacagatctagggccttttctggactagtg

aattagcatgttaagcaactgtcaatgtatcctcagtaaaagggtttggcttatatgact

cacaactgataatgggatgctaaaaatggcagtgataattttaccaattgataatcgcct

ctcaaagaaaagaggtactggtgataatcatttgtgttcaccaacatcgtcacctgtgga

catccaaagaataatagtaaaaccagagctctgctcagttttacttaatcacagtgtcta

ttagtaaaaccagagctctgctcagttttacttaatcacagtgtctatcaactgattatt

cctttctttcttgtccctaccactagcctaaccatttgtgcctgcactctcactggaaag

gtaaccggtgttaatccgtttatgtacgttatacaaacatgtttggcgaagtatcaggtg

aaaatgactattcggtgaaaatctggaaatttctatcgtgaactgttggatcaggagcgg

acgtcatggatcacaggtgggagtcctaacaatccacttaaacgcacattagcagataac

ccccctctatattaatcttgaggtgaatagggctcgacccttagtgagatcctgagatct

gtttcttcgatcgtcttgagcgccgccggcgcgtaaccactgccctcagccggccgcggc

tggccctccgaccgtcgggatgggccttccatccaccacaattctccctctgccaaccgc

gcctcgtgccatggctgcccttccatcgacagtgatggggctttcgtcggctgcatgtcg

ccctccattggacttggctcaccctcttgtagccgggactcatccttaggagtacttgtc

accgctgcccctcacaattttgaaccctgacgtgccgctgcctcttcatagtcatataca

ggtaatccagtacatgaaacggacaagcatcgtaattcactagatgcttacctagaatga

cgagtaggatacactgataagacagtgtttttgtacgcaaattggttgtcactatcttca

gcatcatgagaaagctttggctgctacaaaagacagacattattttcaccgtttttgtca

agcatagaaattcttttttagaaaaaagatcacaagctacgcaatagaaagtcataggtc

aaccagtgtacagagcatgtatatttactatacggaacatggatatttggcgattgttgt

tgttcctcgattgataagaactaatcatgcgatgttgcattatag

ATGGAGCATGAACCTGAGTATGTCGACAATGGAGGGACCCTCCTCCGATCAATTTGGAAA

CTTTGGTACATGAAAAAAGAGAGCTACAAGTGGCCTATTTTAT

gtgagtaccattatcaccataatttcactcaaatctctctaataatcattgacttatatg

actactgggtgtttgtag

GTATGGCATTCGAGACTATGGAAGATGTCGAGAAGTTTTACAAGGCATATGCACACAATG

TTGGGTTTTCAATCAAAATTGAACAAAAAAGGGTTGTAGATAATGTGGTGGTCTGAGGCG

ATTTCTTTGTGGCAAAGTTGGTTTCCGAACAAATGAGGAGGAACACAAGGACTTGAAGGG

AGAGAAGCGAAGAAAACATGCAAGAAAGTTAACTAGATGTG

**>TaMKK1-2(6BS) TRIAE_CS42_6BS_TGACv1_513382_AA1639520.1.X (based on AA1639520.1)**

GTGGCATTAGATTGTTTATCCTCATGGCAGCTCATTTTTCTCTGCATATTTTCGACCACA

TATTTTGCAACAGTGTTTTTTGCCATGGTGTCCCCTCAGCATCTGTCCGTTTAGACGCTA

CGATCAAAGGCAGAAGCTGAGAGATGTTTGAGGGTCCCAATAGTCATCCGCTTGGTGCCA

TGGGATCCCAGCTTCTTTGGGAGCTCAACATGGCCTCATCTGTTGCAGGCTGCCTATAGA

AGAGGGATTAAGGCCGGATGCAGGTACCCTGCTTCAAAACACTGTTTTCTTTGGTGTGTG

TGTGTGTGGGGGGGGTGGGGGGGGGGGATTAGAGTGGTAATTGAGTAGAGGGCTACCTAG

TTCTGATTAATTGATGGAGCACCTGCGAGTGCTCAAGGAGGAGGTGGAGATCATGGAAGA

GGAGGAATCTCCTGAGGCGCACAAGAACGTTGTCCTCTCAAATCCAACTCCTCCTACCAG

TG

gtgaggccctttcactaagtaatcgcatgcaggactacttaacttgttttctttcgatgg

gcacaagaacggataattgtcgtgccatgtctgcattgtgaccacgagattgtcgtcttg

ttttgattgcttgttccttgttaaattctcgcgggtgcctgatttcaacattgtgcattt

tgctttgtcacacagttgatggttgcctttctacagtatacagggcacatgtgtacgcaa

tattcattaaataattttacctttgtttatgtgcctgtgtttag

ATGTTTTGGGTGCCATGAGACCCCTTATTGGGAAGTTGGACATGCTTCTCCTTAGGGATG

CTCCTCAGAAATGCTGCTCCAAGAGGATCAAGGACAGGATGCGCCTCCTCAAAGATGATG

TTCAAAAGATAAGTTCCTACCTTGATGAACTATTAGAGTTGGAAGACCCTCCGCCAATGG

CCATGTGCTGGATGAATGAGGCACGTGACCTGTCTTACGACATGGAGGATTACGTCGACA

GCTTATTATTTGTGCCGCCTGAAGATCCCTCCCTTTTTGCTAACAACATCAAGGCCACCA

GATCCCTCCGCAAATGGTCCAGTCGTGTCAAGACTTCCCAGACTCAGGTTATTAGTGCAG

AAACATTATCGGAATTCAGGAAGTATGTCCAGGAGGCCATTCAACGGCACCAGAGGTACA

ATCTCCATTCTTGCAGAACCTTGAGGCGTAGATTTGTGTTGCATGGCCCTATGGTTCTTC

CAAGGCCATATGAAGAAACTACAGACATAGTAATCGATGGCCGGATGAATGAATTTATCA

ACTCGCTGGCTACCGATGGGGACCAGCAGCTCAAGGTGCTTTCTGTTCTTGGATCTGCTT

GTATTGGTAAAACCACACTTGCTAGAGTGTTGTACAACAGACTTAGGAAGCAATACAATT

GCCGAGCTTTCATTCGAGTATCCAAGAAACCTGATACAAAGAAAATTTTCTGTGACATGC

TCTCGCAACTCAAGCGGCAACATCCTCCGCAACATTGTAGGGAAATTGATCTCGTTCACG

ATATCAAGCAATATCTACAAGATAAAAG

gtagtgcctctttatcaaaattctcatggtccctaaacccttagagttgattatctgtat

gcag

GTATTTAATTATTATTGATGATGTATGGGCTGCATCAGTATGGGATACTATTAATCATGT

TTTTCCAAAGGGTAATCATGGCAGCAGAATAATAACAACTACACAGATCGAAGATGTTGC

ATTAACATGTTGCTGCTATCAGTCAGAGTATGTTTTTGAGATGAAATACCTAGATGATGA

TCATTCTAGAAAGCTTTTCTTTAACAGACTATTTTGCTCTGAAAGAGATTGTCCTGAACA

GTTCAAAGATGTTCTAAATGAAATTGTTGAAACATGTGATGGTTTGCCGTTAGCCACAGT

TAGCATAGCAAGTGTTTTAGCAAGCCAGCCTGTCATGTCAATTGATCTTTTGACATACAT

CCATCGGTCGTTAAGCTCTTGCTTCTCGGCATTAGAAAGAACTAGACAAGCACTGAATCT

GAGCTTCAACAGTCTTCCTCAATATTTGAAGACATGTTTGCTTTATCTTAGTATGTATCC

AGAGGGCTACACAATCTTGAAGGATGATTTGGTGAAGCAATGGGTGGCTGAAGGTTTGAT

CTATACAACAGAAGGGCAAGACATCGGGAAAGTTGCAGAAAGCTATCTCGATCAACTTCT

TGGTAGAAGATTCATCCAACCGATATGTGTCAACTACAACAATGAGGTGTTATCCTGTGC

AGTGCATGGCATGGTACATGATCTTATTGCACACAAATCTGCAGAAGAGAATTTCATTGT

GGCAATAGACTACAGCCAAAAGAATGTGCCACTCTCTCAAAAGGTCCGTCGACTATCTCT

CCTCTTTGGTAATGCAAAATATGCCAAGACACCAGCAAACATCACAAAGGCACAAGTTCG

GTCACTTGGATTTTTTGGATTACTCGAGTCTATGCCTTGTATTACAGAGTTCAAGCTTCT

TCGTGTCCTGAACCTTCAACTGTTCGGTCATGCTGGCGATGACGACGGCCCTGTAGACCT

CACTGGAATTTCAGAAATGTTTCAACTGAGATATATGAAGATTGCAGGTAATGTCTGCAT

AAAACTGCCAAACCATGTGCTACGATGTTTGGAAATACTGGATATTGCGTATGCAAGAGT

TGCTTGTGTTCCACGAGATATCCATTTACCAAACTTGTTACACCTTGGTCTTCCTCTTGA

TAAAAATCTGCTGGATTGGATTAACAGCAGGATGTCTCTCAGTGTGGAGAGAGTACGCAA

GCTGCAGGATCTTCACCTGACCAGATCTTCTGCACTTTCTTCTGACCATCTGAATAGAAG

CATGAGTTCTATTTTATCTTTACTTGGAGGACATGGCAACCTGAAAACTTTAGTAGTGGC

TCATGGCTCATCGGCTAAAAATTCTCTTGGCGTTTCAGACGTCACCCTGTCATGTGATCT

GCTGGCACCCCCACTTCTCCAGAGATTTGAATTCTCGCCACATAGCCGTATCTTGTTCTC

AAGAATTCCTCTGTGGATTGAGAAACATGGCAACCTAAGAATTTTGAAGATTGCAGTGAG

GGAACTGCAGATGAGTTGTGTTGATATCCTCAGAGGATCGCCTGCCCTCACTGCTCTCTC

TCTGTATGTGGAGAAGGCGCCCTACGAAAAGATCATCTTTGACAAGGCTGGGTTCTCGAT

TCTCAAGTACTTCAAGTTGAGGTTCACGAGTGACATAGCTTGGATAAAATTTGAGAAAGG

TGCAATGCCTAATCTCTGGAAGCTCAAGCTAGTTTTCAATGCCATCCCCTCTTTTGAAAA

AGATGGTACTGCACTCATCAGCATCGAGCATATGCCAGGCCTTAAAGAGATCTCCACAAA

ATTTGGGGGTGAAGCTCCTGATCTAGAGTATGCCTTGAGGACCTTGGTTAGTAATCATCC

GACCAATCCTATAATTAACATGCCATCAAATAAGACTCTGGGGGGAGAACAACGACATAG

GAATCTGGAGCAAGAATCAGATGAGATTCTGAAGGAAGATCCATATGGGAATCTGGAGCA

AGAATCGGATGAAATTCTGGAGGAACAAGAGCCAGATGAATACGATGAGAGATTAGAGAG

GCGACAG

gctgataaaaggngattctgaaggaagatccatatgggaatctggagcaagaatcggatg

aaattctggaggaacaagagccagatgaatacgatgagagattagagaggcgacag

GCTGATAAAAG

gtactacaaatgttccttgtaagcacaacgaaatatgccctcctttacttcaacttctat

gaggctgggtgcacccacttggtgcagacgcctcaatcgataaaatctttctttttaaga

aaaacatcaatgtcgattcttcagttaacctgatataattcttatatgttctaacgaagc

taatggttattgtaatcaatcag

GATTTCAAGGTCATCAGATCCATCTTCGCGTCTACATGTTCTAG

gtacattttctgtcaactggtggtaatataaatgtttcgccatcaagttaataaagtcac

ttcaagagaaaactaagcagcttgttctatgtgataaaaattttatcccggactaccaat

tcggaaacgattaatttctccaaccgctgacttgttagagatagaggcttgagcggaaac

cggtgaccatagccaacccatctagtttagttgcgtccgtcattccctgacatcggccta

tagttacaaatgagactttgatcctgtgccagccgaatcagaaaagaatccctgattgca

cgtagcctggatttcaacctcctattttgtcttgcactttcttgtttcattgcttgctca

agcgagatgcatcttcccctattttcatggccccaaccaatcacattcacggcttggact

gcagcatgcacaccatgaaaacatgcctaatttcaggtcctatctctcttcacttatgca

ccttagttatgtgctaaaatagcatgctttatcagattaaaaaatagcatgctttattgt

tatacaaactaattcctctttttcttcgtaattgagttgcatatctcttcacacaacatc

agtaacaatagcaggggtatgcataagcgaagccaccatatcactttcctgacaaaaata

ccttgggacatctggggtggttctttgctactccctccgtccggaaatacttgtcatcaa

aatgaataaaagaggatgtacctagatgtattttagtctagatacattcctttttgtcca

ttttgatgacaagtattttcggacggagggagtacttctttaccgaaaaagggttgcccc

ccgctttatattataaagcaacggtcacacgatacaaatgctggggcctcagcacaagca

agcccaaaagaaacaaagaaaggaactaaagagaaaataatgccaacaacggcggatcaa

cgaaaacgatgaggacccgcaaccgccgcgcccaccggataataccaccacgctcctagc

actccggaccgccgcataccaaacaacaccctcaagaaggatcgcgacgatgacgacgct

gttgcccggacatgtcctagggtttcccccgatatgcgaggggtgaggtggaaggggtat

gcccgacgcccttcaggaaggcacggcggaccccacgagcgtcaccgcgtcggtgccggc

cctgccgacaaggatttctcccgaccaccaaccaaccacgaccccagacgatccatcccg

cgccaccaaacacaccgcccaccaacttgcgccaccacgatcgagcagtctccatcaccg

tctcaccatggcaaacgaaacgggaccgacggaaacaagacgacgcaaccggggaccagg

agcggcaacatcagcagcctcgcgggaggggacatcctccatcgccctcggcgggatccg

gccggacgcagcacaggggctcgcgaagcccccgtcgccgcgctgcagaacgcgagccac

cgtcgccaccaccccccgcacgagtcacgcccccgacccgccggagacgccgcaagcaga

ccaggccaggcccgctcagacccagatcgagccgaaaagggcccagaccaggccaccgcg

tcgccccgccgccaagcggccgcgccaccgccggatccatagtcgccgaggagcaccccc

gtcggtcgccgtccgcccgagccgaggtgccgtgcgggggggaaaggccagggctgccgc

cgccggcaccacgcgggcaaggcccggtggcccaggccgacggcggcagaaggggaggag

ggggaggggagggctgggaggggtgacgagagcccccggtcgccccgctcggtgaggcga

cgcgggaggtggagaggggagatggaaagatcgtcttgctacttcttttcaatcgggtcg

tgtttcatctcccagtcgaccatggagagcagtctcctttcttagttgctattttggtta

attcttgggggcgtgttgggttatatatatactctctcttcatgcaggcacatgacattt

tttattatttttattaagcag

TACCAGTTGGTGCTTCTGAGATGGGAAGTGAGCCTGATGTGGAAATCCCTGTGGCACCAA

CTACTAATGATAACATGATGTTGTCGGTCAATCTCGAAGCATTTGCTAGGGAAAATTATC

CGAGCACTAACTTGGAAAGTTCTGTAAAGGAGATGGAAAGAGTGGCAGGTTTAGAGGATC

ATCAGATGGAAGATAGCACACAG

gtaatttacacattccaactgttttggatttttctgatccttcttagggacatagctact

aggattggggtgaagttaggaagtaatattcttgaggtaaataaatatatttaagtgact

agtatggaagctggtagatccgcctttgagccttgggttctacagggtgtgtgcctagat

tgtttcttctaaattaaaattctacaagggtagtaattttatagctcacaagagaagtct

agcaatatgccttgagtgggttgggttgcccggtggtgcagcttcccatcacttatctgg

gcatcccgttgtcgacgcgacggccttccgccgcccagttgcagcccctcgttgatacgg

tggctgcccggcttcctacctggaaggcgtggctcatgaacaaagccgggcgnnnnnnnn

nnnnnnnnnnnnnnnnnnnnnnnnnnnnnnnnggcgtcttgcccttgtcaaatcggtcct

caacgcaataccagtgcaccagctgctcgcgttcgctcccccgaagaagacgctcaaaca

attggcgaagatccagcgaggcttcttgtgggctgggcgggccgtggccaacggtggcca

ttgccatgtcaactggagccgtgtgtgccgtcctatcgcgtatggtgggctaggagtccg

agacctggagcgcgctggcctcgcgctacgcttgcgatggctctggttcgcccgcgtcga

tccagaccgtgcttggcaaggtctcgatctgcagttctcacacgaggagcgcgcgctctt

ctttgcctccactaccatgatcattggaaatggctcgatggcgcttttttgggaagatcg

ctggattggcgggcagtctgtcggtgagatagctcccctactctaccaatgcattcccaa

acatcgacgcaaggccaggacggtggctgaagggctggcgggtaactcttgggcacatga

tattcaaggggtcctgggtctgcacgagatcggacaatacttactgctatggcaggctgt

gtgccacaccatcctgacgatttcgagccggatcacctgctctggaggtggaccaccaat

ggcacctactcggcgcgctcatgttatgcggccagtttccagggatccacgcggtgccat

tcttggaagctggtatggaagagttgggcgcctccacgggtaaaaattttccattggctc

gccgaccaggatcgctgctggactgctgaaaggctcgcgcgacgtggcttgcagcatcac

cctcgatgcctcctttgcgaccaggccgtggaaactatccagcacctgctgctgacctgc

ccctttgcacgtcagacctggcacattgtccttgattggttgcatattccggtccaaatc

cccgatcaggagcacacggtcacggagtggtggctgagcgcgaaagagctcgcgccgccg

gcacatcggaaggccctcaaatccatcgcgcttcttgtgccctggatgctatggaagcac

aggaacgcctgtgtcttcgacaacgccacgccatccatcgaatcactagtggttaggatc

aaggacgaggcccggtgttgggctaaagcaggagcccagggacttcgggttgttctgccc

acaacctgggatgtccactgattttgtgcttttctttgtactgctcacctcctaggagga

ttgttctaccttttccatgaaatgaaatgcaaaggtcctttgcgttttctcgaaaaaaaa

aatatgccttgagtggggaaccaaaacaattttttattttaactctaaggctatccagga

tttactcgcagatgagtatggggttgatttagaaaatgttagatagctgtgttagaagtt

tagcttcttcaagtagacctaaatggtaggacgtggagtatctactcccgagctcaaatg

atccctgatgaacagtaaattcgaaaaaaaatagtaaacaaattctgaatattttttctg

gtaaacttagaaaaatattttgtgtgcttgcaaatttataataacaggacattcatggaa

gtcgtggcaaaaaaacaaaatcgatgctcctgaaatgctatttttgaaagcattttggag

tattgtttattttattttatttttgccatgacttccacgaatgttatttcgtgatgaaaa

ttttcaagcataccaaacatttgtcaaagtttgtcacattttttttattttacagttcat

cccgagctcaatgagctcgggagcagaagaggacttctgtaaatggtaaaggtgcttttg

ttggcggtaggagaggtacagaagtgataattcaaaatggctcccaagagcacatgcttc

tggctgcaaaaactaaattttcgggtgtccaaaaaatgaaaaaaaaatggatgtctactt

ttttgtgtcctttgtgtacatgtaaattttcatgagaaaaaaaaacttttgtgccccgtg

tatggaatttgttttttgatgcttcaagaaatgcttcttagagcactcttgcttcaagaa

atgcttgtgggaatagtattttaggtaagtactccctccgttcctaaatataagtctttg

tagatattgcactacaaactacatacggatgtatatagacatatttgagagtgtagattc

actcattttgctccgtatgtagtccatagtggaatctctaaaaagacttacatttaggaa

cggaggaagtacaatattatcaaaaggggaaggaaaggtaatactgaatgtagctatacc

catattaaaagcacatgttatgtttcgatcatttttatctcagccatcaacggcattctc

caggagtatctcagccacaactggcttttccttgtattaccgagaattaaatttggatgg

aaaaagagggagacaagtcccacactctcgatacggcgataccgctatgagtttgtggaa

attacccacaacctgggctttggttagcgggcggtaaattcggttgccgcctgccagtaa

cccccccccccccccccacacacacacactgggaatatgttggaaatgatatgcataaac

tgttccaatttttttactaactccaggggcagtagtactagtaaaatcaggctcattcct

gggcctgcagctagtgattctgtagagtggcttcttatagcaggatcctagtacagccac

cagattccagagctgtcaacgtggggacaggaaccacaacacgccaggtttgctggacta

gccgttgatttttgctgtggaatctgggtgcttggggatgaaagagaggttgatagtgag

aactgcctgtcctaagccttcgtgtcactgttacggcggcttccgtgtgagcttccccag

agacagatgacgtcgttcagtttgcagagaaatcgcacatcatgttgcctaggatgctgg

agtttccaatgaggtgtttactatgcaaaatcgcaatcttttggtttcttttaggaagtt

caaaacctctttatgaacttttcttctcagtttttctttttgcggggtgctttattctta

ctagccttgctatggtaaacaaacaagcttttcacatagggcctagaataacggtttttt

atccatctttaaaaatgatcagagtatagaaattttacttagccggacgagaaaataaga

catcacaaatattgcggctttcgaaagtgagcttctcttctcactagacttgtcatatag

acaagcttttcatgtagcacaccacttctttttggtggctatagccggttttaacttttt

taagatgattgcttgcttaccgtagcagagaggagccgggacttgcattttccgcagtat

tttcatctagctgaatcagtagtggatgtgtgggtggattcggcgccgtcttgcgcctcc

caaactggatcatgccgttagcatgaaaagctttggtcttgggatagaataccacttttg

cagtctcagtagaatcctcttaaaattcaaaggattgtagagggctttaattcttaagaa

tcttactacaggtttgtttgattaagatagattgcaattgtacgtttatttttgtctaca

catcctatgtatgtatgtacatgccgccttattgtgcactatgtatattttttcaagccg

ttgaccaattatgccaaactgccaattttgccagccatttatactggtaccgtaatgctt

aattccag

AAAAAGCAGAGCAAAGATGAGGCGGACGAAGACTCATCCATCCACTCACCCCGGCGAGGG

AGCCCTACACCGCCGCTGCTGTTGAAGAAGCCGGGAAAGATCGCTCTCCCCTCCCACGAG

TCCACCATGGGCAGATTCTT

gtaacctcaaaccctcacccctcttctttcgccatttttgttttcattggacgtattgct

cttgcagtcgtgctgattgatttgatttcacggtggttgtggtcag

CTGGGCTCCAGCGCGGGACGGGGACAGGATGCAGATCGGGATGTTCAAGGACGGCGACGG

CAACCTGCTCGTCAACAAGGACGGCCTCCGCATCACCCCGCAGACCGAGGAAGGCGAG

gtaagccactaaaacatgaccgggcactcagatgaaaaattcctccacgcgtctggtctt

agccgttcataggaaaagaacgtagggctgcgctcggcatattggtagttgtattggttt

tcgtaatttcattgatgacgtgaacatatttagatattaggatgtgtggttggattagca

ggtacggagcaggggtttcgtattctttttcagaacaacaatttaggacagatgatacta

tgtatttccttcgtccgggattagtgctcaaacggatctatctagacgtatttcaatgcg

agatacatccgtttgtgcgtcaactaattccatgcggagggaatacataggtgattttca

tgtgggatatccgcaaatgcagttattacagaagtaatttatgatgtatccaatggttaa

atttatcctgaatgccgatgctcacttgcttatagtggaagattgctgtttaacaccatt

tcttgtgggaattgtctaattacattgttgttcttaacttgttatgctcttcattcatct

aacttgtgtctcgccttctttccgttgccatactcgacttctctag

GCTCCTCCTATAGAGCCGTTAGATAACCATCAGTTGAGCATACATGATCTGGAAGTAATC

AAAGTGATTGGGAAAAGTAGTAGTGGAACCGTGCAATTGGTGCGCCAGAAATGGACTGGC

CAGTTTTTTGCTCTCAAG

gtattgcccaaaaaataaagtatttgatgatttataattttggttccttctatttctttc

tgtaggaagtagaaactatagtggtagttataatctctgatattttgcctagggtcttta

ccccaattcatgtgactttcatgtttgcag

GTTATACAACTCAATATTCAGGAAAGCATACGCAAGCAACTTGCCCAGGAGTTGAAAATA

AGTTTGTCAACACAGTGTCAATATGTTGTCACATGCTATCAGTGTTTCTATGTCAATGGT

GTCATTTCTATTGCTTTGGAGTATATGGATGGAGGCTCTCTGGCTGATTTCCTGATGACT

GTTAGAACCGTTCCAGAGGCCTACCTTGCTGCAATTTGTAAGCAG

gccagtaaaatgacacataagtaaccacatagttttttgttattatcagaatgttttttt

tttcaccttaagatcaaatagaccttttcaatcgataatttttacttcctcgtgcag

GTGTTGAAAGGACTGATGTACTTGCATCATGAGAAGCGCATTATACACCGAGATCTGAAA

CCATCAAATATATTAATAAATCATAGGGGTGAAGTAAAGATATCAGATTTTGGTGTTAGT

GCCATCATTTCTAGTTCTTCTACACAGCGAGATATATTTACTGGCATATTTAACTACATG

GCG

gtgcgtgaaagtttgtttgaattatgtaagcatgtcttccagtgtggcatgaaaagacgt

ccaccattttttgtttcagctaacgaatctaaaatgtatactcttgacag

CCAGAAAGAATCAGCGGGCAGAAACATGGTTATATGAGTGACATATGGAGCTTGGGCCTA

GTTATGCTGGAATGTGCCACTGGCAATTTCCCATATCCTCCTCGTGATAGCTTTTATGAA

CTTCTTGAAGCTGTTGTCGACCAACCATCACCTTCTGCACCATCAGACCAGTTTTCACCA

GAGTTCTGTTCATTCATTTCTGCGTG

gtatttgtgcttctccattgcccccctcctccctgttaatcaacaatacttgagcaatat

ttgcaatttgttaagatttaaactgtgagcttagttatcatagtggtttttctacctttt

gcctgtcaagtagtgattctcacgccaaaattcctgcgctggaatatgcatgccaacatc

atcacatgtttctttttgttgcactcacctagtcaccttcatctgtggacagatcaactg

atttccttattttatttactggaaatcagtgtccaaaagaaggctaatatctattctctt

aatttctttctgaatttttcag

CATGCAAAAAGAGGCTACAAATAGGTCATCTGCACAAATCCTATCA

gtaaggaaatttctgtcggcttcacagtttgtatgttcgtccgaaagcgtcatttgacac

agcaagccactgatctttacccatttcttctatctgtttcctctgacacccag

GCTCATCCATTCCTGAGCATGTACAACGACCTGAATATTGATCTGGCTGACTACTTCAGG

ACCGCGGGATCTCCACTTGTCACCTTCAAGTAATGGTCCTGTGTAACCCCACTGAAGAGT

ACTTACATGGTTACTGCAATTCCTTTATAATTCCAATGCTGTTGTATTCTCTCTTTTTCT

TCCTAGGGGAATTGTGTTGTGAGTCAGACAAATGCACAACCATGTTATTCCCCAACACGG

AATTGATCATGCATGTAGCGTGATAGATTTTTTAATGTTTTCTACAGTGATATCACTGTC

AGATTACAAACACAAGTTCCTAAAAAAAAGATTACAAACACAAGTTCTCCCAGTAAATAA

TACGGTGCTCTACTGATCAAGCTACTGATAAAGTGATAACCTTAAT

**>TaMKK1-2(U) TRIAE_CS42_U_TGACv1_644387_AA2139180.1.X (based on AA2139180.1)**

GATGTTTTGATCCGAAGCAGAGGCAGAAGCAAAGTCAGGGAGACGGGGGCCACGATCAGT

GCTTCGTTGGGCGCCATGAGACCCCTTATTGGGAAGTTGGACATGCTTCTCCTTAGGGAT

GCTCCTCAGAAATGCTGCTCCAAGAGGATCAAGGACAGGATGCGCCTCCTCAAAGATGAC

GTTCAAAAGATAAGTTCCTACGTTGATGAACTATCAGAGGTGGAAGACCCTCCGCCAATG

GCCATGTGCTGGATGAATGAGGCACGCGACCTGTCTTACGACATGGAGGATTACGTCGAC

AGCTTATTATTTGTGCCGCCTGAAGATCCCTCCCTTTTTGCCAACAACATCAAGGCCACC

AGATCCCTCCGCAAATGGTTCAGTCGTGTCAAGACTTCCCAGACTCAGGTTATTAGTGCG

GGAACATTATCAGAATTCAGGAGGTATGTCCAGGAGGCCATTCAACGGCACCAGAGGTAT

AATCTCCATTCTTGCAGAACCTTGAGGCGTAGGTTTGTGTCCCATGGCCCTATGATTCTT

CCAAGGCCATATGAAGAAACTGCAGACATAGTAATCGATGGCCGGATGAATGAATTTATC

AACTCACTGGCTACCGATGGGGACCAGCAGCTCAAGGTGCTTTCTGTTCTTGGATCTGCT

TGTCTTGGTAAAACCACACTTGCTAGAGTGTTGTACAACAGATTTAGGAAGCAATACAAT

TGCCGAGCTTTCATTCGAGTATCCAAGAAGCCTGATACGAAGAAAATATTCTGTGACATG

CTCTCACAACTCAAGCGGCAACATCCTCTGCAACATTGTAGGGAAATTGATCTCATTCAC

GATATCAAGCAATATCTACAGGATAAAAG

gtagtgccttttttcttcattcaagataatatcaaaattcccatggtccctaaaccctta

gagttgattatctgtatgcag

GTATTTAATTATTATTGATGATGTATGGGCTGCATCAGTATGGGATACTATTAATCATGT

TTTTCCAAAGGGTAATCATGGCAGTAGAATAATAACAACTACACAGATCGAAGATGTTGC

ATTAACATGTTGCTGCTATCAGTCAGAGTATGTTTTTGAGATGAAACACCTAGATGATGA

TCATTCTAGAAAGCTTTTCTTTAACAGACTATTTTGCTCTGAAAGAGATTGTCCTGAACA

GTTCAAAGATGTTCTAGATGAAATTGTTGAAACATGTGATGGTTTGCCGTTAGCCACAGT

TAGCATAGCAAGTGTTTTAGCAAGCCAGCCTGTCATGTCAATTGATCTTTTGACATACAT

CCATCGGTCGCTAAGCTCTTGTTTCTCGGCATCAGAAAGAACTAGACAAGCACTGAATCT

GAGCTTTAACAGTCTTCCTCAATATTTGAAGACATGTTTGCTTTATCTTAGTATGTATCC

AGAGGGCTACACAATCTTGAAGGATGATTTGGTGAAGCAATGGGTGGCTGAAGGTTTGAT

CTATACAACAGAAGGGCAAGACATCTTGAAAGATGCAGAGAGCTATCTCGATCAACTTAT

TGGTAGAAGATTCATCCAACCGATATGTGTCAACTACAACAATGAGGTGTTATCCTGTGC

AGTGCATGGCATGGTACATGATCTTATTGCACACAAATCCGCAGAAGAGAATTTCATTGT

GGCAATAGACTACAGCCAAAAGAATGTGCCACTCTCTCAAAAGGTCCGTCGACTATCTCT

CCTCTTTGGTAATGCAAAATATGCCAAGATACCAGCAAACATCACAAAGGCACAAGTTCG

GTCACTTGGATTTTTTGGATTACTCGAGTCTATGCCTTGTATTACAGAGTTCAAGCTTCT

TCGTGTCCTGAACCTTCAACTGTTCCGTCATGCTGGCGATGACGATGGCCCTGTAGACCT

CACTGGAATTTCAGAAATGTTTCAACTGAGATATTTGAAGATTGCAGGTAATGTCTGCAT

AAAACTGCCAAACCATGTGCTACAATGTTTGGAAATACTGGATATTGCGGATGCAAGAGT

TGCTTGTGTTCCACGAGATATCCATTTACCAAACTTGTTACACCTTGGTCTTCCTGTTGA

TAAAAATCTGCTGGATTGGATTAACAGCAGGATGTCTCTCAGTGTGGAGAGAGTACGGAA

GCTGCAGGATCTTCACCTGACCAGATCTTCTGCACTTTCTTCTGACCATCTGAATAGAAG

CATGAGTTCTATTTTATCTTTACTTGGAGGACATGGCAACCTGAAAACTTTAGTAGTGGC

TCATGGCTCATCGGCTAAAAATGCTCGTGGCGCTTCAGATGTCACCCTGTCATTGGATCT

CCTGGCACCCCCACTTCTCCAGAGATTTGAATTCTCGCCACATAGCCATATCATGTTCTC

GCGAATTCCTCTGTGGATTGAGAAACATGGCAACCTAAGAATTTTGAAGATTGCAGTGAG

GGAACTGCAGATGAGTTGTGTTGATATCCTTAGAGGATCGCCTGCCCTCACTGCTCTCTC

TCTGTATGTGGAGAAGGCGCCCTACGACAAGATCATCTTTGACAAGGCTGGGTTCTCAAT

TCTCAAGTACTTCAAGTTGAGGTTCACGAGTGACATAGCTTGGATAAAATTTGAGAAAGG

TGCAATGCCTAATCTCTGGAAGCTCAAGATAGTTTTCAATGCCATCCCCTCTTTTGAAAA

AGATGGTACTGCACTCATCAGCATCGAGCATATGCCAGGCCTTAAAGAGATCTCCACAAA

ATTTGGGGGTGAAGCTCCTGATCTAGAGTATGCCTTGAGGACCTTGGTTAGTAATCATCC

GACCAATCCTATAATTAACATGCCATCAAATAAGACTCTGGGGGGAGAACAACCACATAG

GAATCTGGAGGAAGAATCAGATGAGATTCTGAAGGAAGATCCATATGGGAATCTGGAGCA

AGAATCGGATGAAATTCTGGAGGAACAAGAGCCAGATGAATACGATGAGAGATTAGAGAG

GCGACAGGCTGATAAAAGGNAGATTCTGAAGGAAGATCCATATGGGAATCTGGAGCAAGA

ATCGGATGAAATTCTGGAGGAACAAGAGCCAGATGAATACGATGAGAGATTAGAGAGGCG

ACAGGCTGATAAAAG

gtactacaaatgttccttgtaagcacaacgaaatatgccctcctttacttcaacttctat

gaggctgggtgcacccacttggtgtggacgcctcaatcaataaaatatttcttttaaaga

aaaacatcaacgtcgattcttcagttaacctgatataattcttatatgttctaacgaagc

taatggttattgtaatcaatcag

GATTTCAAGGTCATCAGATCCATCTTCGCGTCTACATGTTCCAG

gtacattttctgtcaactggtggtaatataaatgtttcgccatcaagttaataaagtcac

ttcaagagaaaactaagcagcttgttctataaaggaaaaggacagacccagtgcatagaa

gctcccacacaaggtggggtctggggagggattataggaacctagtcttacccctgcaaa

gtgcaatgcagagaggctggttcgaacccaggacctcttggcacaagtggggaggacttc

accactgcgccaggcctgccctccaagcagcttgttctatgtgataaaaaatttgtcccg

gactaccaattcggaaacgattaatctctccaaccgctgacttgttagagatagaggctt

gagtggaaaccggtgaccgtagccaacccatctagtgtaattgcatccgtccatccatgg

catcagcctctagttacaaatgagactttgatcctgtgccagccgaatcagaaaagaatc

cctgattgcacgtagcctggatttcaatctcctattttgtcttgcactttcttgtttcat

tgcttgctcaagcgagatacatcttcccctattttcttgcccccaaccaatcgcattcac

ggcttggactgcagcatgcacaccttgaaaacatgcctaatttccggtcctatctctctt

cacttatgcaccttagttatgtgctaaaatagcatgctttatcagattaaaaaatagcat

gctttattgttatacaaactaattcctctttttcttcgtaattgagttgcatatttcttc

acacaacatcagtaacgatagcaggggtatgcgtaagcgaagccaccggatcactttcct

gacaaaaagaccttgggacatctggggtggttctttgctgcttcttttcaatcgtgtcct

gtttcatcgctcagtcgaccatggagagcagtctcctttcttagttgctactttggttac

ttcttgggggcgtgttgggttatatatatactctctcttcatgcaggcacatgacatttt

ttttaatttttattaagcag

TACCAGTTGGTGCTTCTGAGATGGGAAGTGAGCCTGATGTGGAAATCCCTGCGGCACCAA

CTACTAATGATAATACGATGTTGTCAGTCAATCTCGAAGCATTTGCTAAGGAAAATTATC

CGAGCACTAACTTGGAAAGTTCTGTAAAGGAGATGGAAAGAGTGGCAGGTTTAGAGGATC

ATCAGATGGAAGATAGCACACAG

gtaatttacacattccaactgttttgaattcttctgatccttcttagggacgtagctact

aggattggggtgaagttaggaagtaatattcttgaggtaaataaatatatttaagtgact

agtatggaagctggtagatccgccttcgagccttgggttctacagggtgtgtgcctagat

tgtttcttctaaattaaaattctacaagggtagtgattttatagctcacaagagaagtct

aacaatgtgcctttgagtggggaaccaaaacaaattttttttaactctaaggctatccag

gatttactcgcagatgagtatggggttgatttagaaaatgttagatagctgtgttagaag

tttagcttcttcaattagacctaaatggtaggacatggagtatctactcccgagctcaaa

tgctccctgatgaacagtaaattcgaaaaaaaatagtaaacaaattcaacaaattctgtt

ttttctggtaaactttgacaaatattttgtgtgcttgcaaatttataataacaggacatt

cgtggaagtcgtggcaaaaaaacagaatcgatgctcccaaaatgctatttttgaaagcat

tttggaatattgtttattttattttatttttgccatgacttccatgaatgttatttcgtg

atgaaaattttcaagcataccaaatatttgtcaaagttcgtcacatttttttagaatgtt

tcaatttttttactgttcatcccgagctcaatgagctcgggagcagaagaggacttccgt

aaatggtaaaggtgcttttgttggcggtaggagaggtacagaagtgataattcaaaatgg

ctcccaagagcacatgcttctggctgcaaaaactaattttccggatgtccaaaaaatgga

aaaaaatggatgtctacttttttgtgtcctttgtgtacatgtaaattttcatgagaaaaa

aaaacttttgtgccctgtgtgtgaaaaaactattttgatacttcaagaaatgcttcttag

agcactttcttgcttcaagaaatgcttgtgggaatagtattttaactaagtacaatatta

tcaaaaggggctggaaaggtaaaactgaatgtagctatacccatagtaaaagcacatgtt

acgtttcgatcatttttatctcagccatcaacggcattttccaggagtatctcagccaca

actggcttttccttgtattaccgagaattaaatttggatggaaaaagagggagacaagtc

ccacactctcgatatggcgataccgctatgagtttgtggaaattacccacaacctgggtt

ttggttagcgggcggtaaattcggttaccgcctggccggtaaccgcgtttcttgcccccc

ccccctcccccccacccacactgggtaaacggactttccgggcaaagaatcctagatatt

ttgaatgaattttctgcggactcaaatttcatntgaatatgttggaaatgatatgcataa

actgttccaatctttttactaactccaggggcagtagtactagtaaaatcaggctcattc

ctgggcctgcagctagtgattctgtagagtggcttcttatagcaggacctagtacagcca

ccagattccagagctgtcaacgtggggacaggaaccacaacatgccaggtttgctggact

agccgttgatttttgctgtggaatctgggtgcttggggatgaaagagaggttgatactga

gaactgcctgtcctaagccttcatgtcactgtcatggcggcttccgtgtgagcttcccca

gagacagatggcgtcgttcagtttgcagagaaatcgcacatcatgttgtctaggatgctg

gagtttccattgaggtgtttactatgcaaaatcgcaatcttttggtttctagaacttctt

ttaggaagttcaaaacctctttatgagcttttcttctcactctttctttttgtggggtgc

ttttattcttactagccttgctatggtaaacaaacaagcttttcacatagggcctagaat

aacacttttttatccatctttaaaaatgatcagagtatagaaattttacttagccggacg

agaaaataagacatcacaaatattgcggctttcgaaagtgagcttctcttctcactagac

ttgtcatatagacaagcctttcatgtagcacaccacttctttttggtggctatagccggt

tttaactttttaaagatgattgcttgcttaccatagcagagaggagccgggacttgcatt

ttccgcagtattttcatctagctgaatcagtagtggatgtgtgggtggattcggcgccgt

cttgcgcctcccaaactggatcttgccgttagcgtgaaaagctttggtcttgggatagaa

taccacttttgcagtctcagtagaatcctcttaaaatttaaaggattgtagaggacttta

attcttaagaatcttactacgaggtttgtttgattaagtaagattgcaattgtatgttta

tttttgtctacacatcctatttatgtatgtacatagtgccttattgtgcactatgtatat

tttttcaagccgttgaccaattatgccaaactgccaattttgccagccatttatactggt

actgtaatgcttaattccag

AAAAAGCAGAGCAAAGATGAGGCGGACGAAGACTCATCCATCCACTCACCCCGGCGAGGG

AGCCCTACACCGCCGTTACTGTTGAAGAAGCCGGGCAAGATCGCTCTCCCCTCCCACGAG

TCCACCATGGGCAGATTCCT

gtaacctcaaaccctcacccctcttctttcgcatttttgttttccttggacgtattgctc

ttgcagtcgtgctgattgatttgatttcacggcggttgtggtcag

CTGGGCTCCAGCGTGGGACAGGGACAGGATGCAGATCGGGATGTTCAAGGACGGCGACGG

CAACCTGCTCGTCAACAAGGACGGCCTCCGCATCACCCCGCAGACCGAGGAAGGCGAG

gtaagccactaaaacatgaccgggcacccaagatgaaaaattcctccacgcgtctggtct

tagccgttcataggaaaagaacgcagggctgtgctcggcatattggtagttgtattggtt

ttcgtaatttcattgatgacatgaacagatttagatattaggatgtgtggttggattagc

aggtacggagcaggggtttcgtattctttttcagaacaacaatttaggacagatgatact

atgtacttccttcatctgggattagtgctcaaacggatctatctagacgtatttcaatgc

gagatacatccgtttgagcgtcaactaattccgcgcggaggaaatacataggtaattttc

atgtgagatatccgcaaatgcagttgttacagaaataatttatgatgtatccaatggtta

aatttatcctgaatgctgatgctcacttgcttatagtggaagattgctgtctaacaccat

ttcttgtgggaattgtctaattacattgttgttcttaacttgttatgctcttcattcatc

taacttgtgtcttgccttctttctgttgccatactcgacttcgctag

GCTCCTCCTATAGAGCCGTTAGATAACCATCAGTTGAGCATACATGATCTGGAAGCAATC

AAAGTGATTGGGAAAGGTAGTAGTGGAACCGTGCAATTGGTGCGCCAGAAATGGACTGGC

CAGTTTTTTGCTCTCAAG

gtattgcccaaaaaataaattatttgatgatttataattttggttccttctatttctttc

tgtagaaagtagaaactatagtggtagttataatctctgatattttgcctagggtcttta

cccccattcatgtactgtcatgtttgcag

GTTATACAACTCAATATTCAGGAAAGCATACGCAAGCAACTTGCCCAGGAGTTGAAATTA

AGTTTGTCAACACAGTGCCAATATGTTGTCACATGCTATCAGTGTTTCTATGTCAATGGT

GTTATTTCTATTGCTTTGGAGTATATGGATGGAGGCTCTCTGGCTGATTTCCTGATGACT

GCTAGAACCGTTCCAGAGGCCTACCTTGCTGCAATTTGTAAGCAG

gccagtaaaatgacacataagtaaccacatagttttttgttgttatcagaatgttttttt

caccttaaggtcaaatagaccttttcaaccgataatttttacttcctcgtgcag

GTGTTGAAAGGACTGATGTACTTGCATCATGAGAAGCGCGTTATACACCGAGATCTGAAA

CCATCGAATATACTAATAAATCATAGGGGTGAAGTAAAGATATCAGATTTTGGTGTTAGT

GCCATCATTTCTAGTTCTTCTGCACAGCGAGATACATTTACTGGCACATTTAACTACATG

GCG

gtgcgtgaaattttgtttgaattatgcaagcatgtcttccagtgtggcatgaaaagacgt

ccaccattttttgtttcaactaacgaatctaaaatgtatactcttgacag

CCGGAAAGAATCAGTGGGCAGAAACATGGTTACATGAGTGATATATGGAGCTTGGGCCTA

GTTATGCTGGAATGTGCCACTGGCAATTTCCCATATCCTCCTCGTGGTAGCTTTTATGAA

CTTCTTGAAGATGTTGTCGACCAACCATCACCTTCTGCACCATCAGACCAGTTTTCACCA

GAGTTCTGTTCATTCATTTCTGCGTG

gtatttacgcttctccattacccccctcccccctgttaatcaataatacttaagcaatgt

ttgcaatttgttgagatttaaactgtgaacttagttatcatagtgttttttctacctttt

gcctgtcaagtagtgattctcacgccaaaattcctgctctggaatatgcatgccaacatc

atcatatgtttctttttgttacactcacctagtcaccttcatctgtggacagatcaactg

atttccttattttatttactggaaatcagtgtccaaaagaaggctaatatctattttctt

aatttctttctgaatttttcag

TATGCAAAAAGAGGCCACAAATAGATCATCTGCACAAATCCTATCA

gtaaggaaatttctgtctgcttcacaatttgtatgttcgtccgaaagcgtcatttgacac

agcaagctctctgatctttacccatttcttctatctgttgcctctgacacccag

GCTCATCCATTCCTCAGCATGTACGACGACCTGAATGTTGATCTTGCTGTCTACTTCAGG

ACTGCGGGATCTCCACTTGTCACCTTCAA

gtaatggtcctataaccccactgaagagtacttacatggttactgcaaattcctttacaa

ttccaatgctgtcgtattctctctttttcttcctaggggaaccatgttgagtcagacaaa

tgcacaaccatgttattccccagcgcggaattgatcaggcatgtgtagttgatcatgcat

gtactgtgtttcctacagtgatagcactgtcagattacagacacaagttctcgcagtaaa

taatactgtgctgataaagtgataaccttaatttaaaaagggataatttgtgatcttgac

ataaaaaagaggcaattgatgattatcttataaccggcatcacacgtgtataaccatgtc

agtgagccgcatcaacatagaaggttgtgccactacattttgttctcacgcttctattgc

agaggcaactggtgtcacggcttatgttgacataaccgcagtataatctcaaatttgaag

aatccatttcctttacgaaacagatctagggccttttctggactagtgaattagcatgtt

aagcaactgtcaatgtatcctcagtaaaagggtttggcttatatgactcacaactgataa

tgggatgctaaaaatggcagtgataattttaccaattaataatcccctcccaaagaaaag

aggtactggtgataatcatttgtgttcaccaacatcgtcacctgtggacatccaaagaat

aatagtaaaaccagagctctgctcagttttacttaatcacagtgtctgccaactgattat

tcctttctttcttgtccctaccactagcctaaccatttgtgcctgcactctcactggaaa

ggtaaccggtgttaatccgtttacatatgttacaaacatgttttgcttaatcacattact

ggagtaaacgtttgttagtcacaataactagattattcatcttttgccag

GAATAAGTGAGACCTTTTTTCTTCTTCGCAAATGTGGAACTCATCAACTCACAGAATGCA

CGGCCATCAAGCAAGCAAGCGCGTAATTGGACTGAAGCAAAGTCACTCTATCTTATCATG

CCCAGAGATAAGATAACAGAGGGAAGTTGGCATGAAAAACATTTCAGTTTGTTAGGCCAC

TACTCAATTTTGAAGAAAGATAGACATGTGAGTTTTATCTAAACTTTGGGAAGTGTGAAG

TATGCTCAATCACACCACTCAAGCAAAAAGTTTGTGAATCTCAAAGCAAAGGTTATTGAA

TCCCCAACACCAGGTAGGCAGTTTCGTTTCAGCATACAGGAAGGAGCATCTTCCAACTCT

CAGGTCACTTCAACCTCCATTAGGCAGAAGCAAGCCTCTAAATTCTGAGTTCGGAACCAG

ATGGAAATTCAAGAAAAACTCGTGCAAAATGGGTCACCACAGAACATGTTTCGCTTAATC

CAGCACTGACGCAAAAGGTTTGGTAATCTCAACATGCAGCGGCAGATCCAAGCTAACATT

GCCCTCTCATTGGTTCACCTATCCCTGTTACTGATCGCAAGAACCAAAACTATTCATCTT

CTTTTTTTGCCTGGAATAGTATGTGAGACCTTTTAA

**>TaMKK1-3(7DS) TRIAE_CS42_7DS_TGACv1_623511_AA2053970.1.X (based on AA2053970.1)**

CGGTCTCGTCGGCGGCGGAGATGGGCTACGACCGCTGCGGAGGACGCGGCCGGCGTGTGC

GGGCTGTACCTCAGCATGTTGAAGGGCGTGTTCGCGCACGAGGTCAGGGGCGCGTGCTCC

CGGCGGACGAGGGACGTCGCCTCGCTGGAAGCCAGGGGCGCATGCTCGAGCGGGAGGTGG

GAGAACGCCTCGCCGGGGCGCATGCTCGCGCCGGAGAAGGGCGGCCGCCTCACCGGAGCC

GTCAGCAGGCTTCGCCGAGGCGCGTGCTCACGGGAGGAGGGAAGCCGTGCCACCGGGACC

GGCGGTGCTCGCCCTGATGCAGTCTGGGTGCAACTCCACTATCTACTACAACCTCCAGGC

GGCCGCCCTCGTCGCGGACCCGCCAGCTCCACTGCCGATCTCCGCGGCCTCTGCGCCATC

GCCGTCACCGCGGCGACCGCCAACGCCTCCGCCATCGCGTTCGTGCTCGCCAACACCACC

TGGGGCACACCAGAGCCCGGTAGCGACGGGCGCACACAACAGGTGCCGACGCTCCTGATG

AGGACCTGCGCCGGCAA

gtacggcgaggcggaaggaggtagtcgtccacacgtgggggagggggtgattgctttttc

ttttttgatacaagggtgtgtgtgcaatttctttgccaaattagctccttacaatctggg

cctacctgccagaaagtgatgaaacgcaacaaatcgagcgatcagtgctttctgcaaaaa

gtttaccagatatgcggtggcttttgcaaataattagggaccatgtagtttttcacaaac

gaagcctaaaatatggtggttttctgcaattaactcatcatggtgaacaaggtactcgtt

atgacgtccctccgcctaggccccacctcctcaccctagccgccgggcaagcccggggcg

ccagggatgacggcggcggggcctcggttgccctgcctctgcgtggggaaccccggatct

agagcggcgactccactggcaagacacagccggctagcagccgtgcaggcggtggatctg

gcgtcccggccgcgcgggggcgggatccgatggtcgttggcggccggcggcggcttctgc

ggcagtccgtgtacgcgatctggcgcctcccctcctcccggttcggcgtgcgggccagcc

tggtcgggccgcgcttcgtcttggtcgtcggttctgtacaggaggcactgctggtggcgg

agatctagttcgggtgaaatccctggccgaccatgaccggccgcgcagacgacgaagcct

gagggcgacgttcccctccttggagcgtcggtatggattgatctcctcacttccccttcc

cctaggtctcccgggcgccctaactttgttgggcggcggcggcgctcacagcgtcgtttc

attcatgaaggcgccgctttgggaaccttggggttgggtggcgcttgtgagtggtgggcg

acggcggtggtgcggccctatcctagcatggatttacgtctgttgcttggagatagactc

acgtaggtggaggtcgtcgtctggcgtcgtggtggcgtcaatggcggaggacctgtcaag

gctcacgtaggtggaggttgtcgtttggcgccgtggtggcgtcaatggcggaggacctgc

caaggctcgcgtaggtggaagtcgtcgtggtggcgtcgatggcggaggacctggcaaggt

tgtcacctcaatctcctcagaagatggaccagtggaagatggcggccgcgacacatgtga

gttcgtcggaccggtttgagccccggacccggtcggggattccggctttagatgttaggc

ttgggtgagaggtctggatatgtggcccagcttgcacctcttcatcatttgcataggagt

agcgtcagatgttgccaagatacggattcaggcatattgttgttctactttgtaagatcc

tcgagaataatcaataaaatggccgcatgcatctcccagatgcagaggccgggagtcatc

cttcttttctaaaaaaaagtaataataatagatggcaaaagtatgagctgcacacctgaa

acttgtcgccctgagaactgagaagaatagacttgtttaccatgatggatcttatcggca

gcctccaggttggcgctccacaaagtttttgtcttcgtccttgttaaatacgctcgtgtt

gacctgtgtgttgttccacggtaaggcaagagatgttgaggcagtggcaaccgatactac

tctgttggtgatgggcaagcctggttcatgactcaagctgcactctgagtctgtcacaca

gttgagatgacagggtgcgtgtctatgtaaaaacatccaattcctcccaagcagactcga

tttgctttccacttgtaattcgagctggcctcaagtaccactcggcaatctttggaaaac

cattggttcttgaaaatgcctccatctgcaagggtccaccttgcagaccatcacccaaaa

atttaggatgcttaaataaatttcagcaaaggcgctaagcgcataaaacttgaacacgga

gattacatggtataatatggcattccaggttgcaaatgcgcaatgttgcaattaactgta

gtttctaatttaagaatattccaaataaatgaagaatcttaaggttttagcactataaga

tttcatacttcagctgccgtatgtgatgccagataagggcagtttgcacgcgccataagc

accgaccggactgtttcacagttaattcactcatccacctgatataaaatgaagaaacaa

caagtcacttgaaaagagctttttttacggtaccttatactgctattggaaaaaagcagt

ataagcctcatctaaccgttcatttctagagtattatggacctgttcaaagggtaaaaaa

aaaaatttaatgttcaccgggggcggagagtttcccccacctgaatatatttgatgatca

ggggagaaccccagggaagtgttttacagatgaccgtgtccagaacttctctcgtaacca

taaacgaagatcgattctccgcctctgcaccgttcagccgttagccgccaccgggtttgc

ctggacattggcgacctccgcaacttgaaacagcaattacgccagatgttcgagcgctcg

cctcatcggggaatggctcctcggacattgctttgcaagcatgtggtgaccggctgattg

tcattgggcatccaagaaatgctactggtggcacagtggttgagcttcatgcatggatcc

cggatgagcaaccggccatgtggaatttggttgccacggagccattaagccgtaagttcg

cagtaaattggagatttactttgccgtaatgggtttctgagtctgtccattgaataaaga

ttggaagcatgcttaactagcggcagcatttcgatcaacagcaattttagtgctttgcgg

aagcttttgaccaatacttgatttatctttgggctttcgctccaacaaaggcatgttttt

ttctattatgaacatactccataaggtacctaccttatattcaataccatcctaattttt

ctttgcccaatttgatccatgatatgcttttgttgaactgcgttatcaagttcttcgtat

taatgatatggtgttctgcagtgtatgttgtgtagtgttggagaacctgaggagcacttg

tgccttgtgtgctctgttcctaaagcgtggttaagtgcacactgatgttctatttatgta

cgtagttgagttgtgaagcttagctgccgcagaccccttgatgatggatgccgtcaattc

gttctgtcgcaacctctgcttttgctctgttttctcaactctgctttacctctagttagc

ctcctctactcctctgctttgctctgctttctaaactctgaatatttgcatgtcccttcc

tctgcttttgctttgttttatgagctctgaatatttggttgtagcttcagaccggtttca

ggcgtcaggttgactccactcggggcctcacagacaatcttcctccttccgaccacggga

cagacggacggagatgctgccgccctcctcacgctaatggcggttgctacctatcctctc

tttctttttaacaaggataatacacactagactgcgtgatactcgatctacagcgtcaaa

tctacttcaccaaagacacaccatccagaatggttgaaatgttcagttattttcttttcc

aaagaatgagcaatcgaatcagcgaaatcggaacggtcctgctctggacggaactttcat

gcgtaaattattgtgatataaaaatggatcggtggattgcatgtaccccgtctcgagcct

ggtgttttcactttcgtcagacagagaagtcgacagggtcggttaatcttgctcgagaca

gagctgggttgtgtttcaagtatcaggttggcattttaagtagctgcagcttctgttgcc

tcgacagctgggttgtccctttaagcctttgggcattgtgctatcaatttctgatgcggt

aatggtggattcttaggtgctggatgtaccgctatggttatggggactcttctcctaaag

acgataaagcgcttctcataagtttgtcctgaatgcacatgagtttgtttctcatgaatt

cagatttggtgacttcctgctatcctgtgacagtccatcatttatgtttatgttgttgga

caatggttccatgaatgtaatcaggacatcacttgccgattatgtgtgtcctaaattatg

cgtgctaattgtgcatactaatcttagaatgaaaataatataatagaatgttgcacttta

cgccaaacactacatttggtacagaaataataatttgggggattgcaaatgggaattcgg

gatggaggtttgttcccctgcttggtaatggcaactcacggtctttttttttttgaaaag

gaggatatcccccggcctctgcatcaaaacgatgcatgcagccatcttattacagatgaa

aacactaatactagaaaacaagtttccccattctcctcgcattatttgatttgctgcttg

ggactcaagagacgaacaactcttctctgctatgcaactgaagctcctgaacaacatgtt

aatggcggacggagacgctgcctgcccctctcctcgcgtcaatagcggatactacctttt

ttcccttggagcaccgaccggactatttcagagttaattccctcgtccacctgatacaaa

atgaggaaacaacgggtcagtcgaaaaggacatggaattttactcacatacaggttgttt

gaatggatcgtttgaagaacttggcgacatccagctccaaacagacccactaggctgggc

aactctgttgatctccagcgacataagctgaacttcctctgtttttctgaactctgaata

tttgcatgtactagttagcttcatctgcttttgctcagtattctgaaaataagaagagtt

ggcaacaacaaatgtactgtatgatcgaagagaatcagcaacctgttctggaatcaaaca

caccaattgttgaagaaaccacacgaacggaaccaccgacttcatcccttgtcgccccat

gcctttgtatgagctccatgtgttggggatgtacacggaacggaagggtatttctcaaca

gcccaatggtgaagagcgtggttgcaaggcatggtggcgtgctgtggcggcttggatgtg

gaggcgatgcggcggtcatgaagccgtggtgtggtgcggcagccccaccgatggcgattt

ttgttgttgttgttctccatgttacaaagtcttctcttttgccgagcatatgaacttggc

aaatcatatgtgccaagtggcaacaacgctgttgtccgctctgttccgtctggtcttttt

ttttatgtcgtgtactggcttttgacactcagcaaatgcgcgacagaactatgagtaggc

agagccaatgggcaagcaatacttgccaaagccaaaagttgacaatgagaactagtgtca

atgtcaggcacaaaatgcctgcatattttgtcatattaaaactagcttatctttctggtt

tgatatgaatcatatcactacctgcatatttcgtcatgctaaaactaccttgtataaata

ggagaaaactaattgagatgaagaagaagttgattggaatggtatgtttagaacatttta

tgaaaatgtgaggattaagattgcttgcag

GGACCCCAGGAAAATTCCTTTTGAGAGACTAATTGAGATGAAGAAGAAGTTATTCTTACT

TGGCTTCACTGTGGAAGGCTTTGAACGGGTGGGAGGCAAAAACTCTGTGGTAGAAATTGA

AGATGAGAATGGAGATGATGATCTTGAGGAAGATAATGGTAACCAAGAACCAAATGATTT

ATCCAAGAACAATGATGATGATGAACTTATTGATGATGACACTGCCATTGATGATCTTGA

TAAGGCAAATTTTAAAGCCCAAGGTGGTACTAGCTCTTCTGCTGGGAAAAAAG

gtgcaacacaatatttgactgccagggggatgatctggaaaatttcactatggagtgcct

ttcaaacaaggagaatgtggttgagaatgaggtgaatgagaagatgtgctctttgactga

gggtgccctgattaacaaaagtgaggggatttgtactcctgaaaaaacttggcctaaaaa

agatgatgatggtaatcaaagtgacaatgtgagctactgtttgaacctcctaaactctgt

agacatgtctgtgtctgatgatgatgaatacgaggaagaggtggatgtgggagttctctc

ccctgggactgtttagaaaattcaagaatctggcataaaacgttcattattggagatttt

ggaccaagccagatctggtggaaacaaggaagacaggaacttaaagtggggccctgttaa

agcccagaagtatgacactaggctgcatgggaatgtggatatgaagaagaatctggagat

tcctccaactttcaaaggtaaatatttccctaccaatgattctttcctccttgctagtca

agttgttcaagtaggtcttgccattggtaagaataatgataatatgtttgacattataga

agatcttaaagttcaagaacaaaaaaatgcattgcctttgctgataatgaacctgaagta

gctttacctgataatattgatctgacacaagaagatttagttgggactcctgctagtgat

ggctctgtgacatcagttggcactgctgatggtacagatcaatcccctgctgtccttact

aaggttaagccttgtggcttacctcacccttttagaataaactgatgatagatcttatct

ggaatatcagaggtctaagtaagcctggtaaagttcagtgcctttgtgatgtgattacta

aagctaatcctgatttcattggttttcaagagactaagaaagaaactatttctgagggtt

tcctcagagctttggatggcaaaaagctttttagctggcattttctcccagctataggga

ctgctgggggtattttagttggccttaaaaatgaggtgattgggtttaccaataatagat

tttctgttagggctactgttaagaataaatgggatggttttttgtggcatttagtcatag

tttatggttcttcctaccctgaattcaaagtggattttgtttctgagttgcatgacatct

ttgaatccagctcttatcctattttgatttgtggtgacttcaacctagtgagagagagta

aagagaagagcaatggccttgttgatcaacaaagttctttgttgttcaatgactggatta

acagatggggtcttatggaaatttctatttccaacaggagatttacttggtctaataatc

aggagaacccagtttttgctgtcctggatagggttttttcttccatagattgggatggtc

atttccccatgtcttccttggttgccctacccagggctggcagtgaccatgcccccctgg

tgttagatactagggggagaagaaatcatggatctaagatgttcagatttgagaagtggt

ggctctcccaacctgacttcaatcaggttgtggaaaatgcttggaaatctgcttcctcta

atatttcctcggtggataattggatggttaagtctagggttctcaggaagaaaatcaaag

gatgctcttaaaaaaagggaaaaaacatcctggtggagctagacaagctggatgtctctt

ctgagacacaaaacctttccccaagtgattttgctagattaggaaatctcaaaaaggaat

tggaagatatttggagaaaagaagaaactgctttgtggtagaggtctagagatagaaaga

tcttggaaggagataggaacaatgcctatttccaagcattagcaaatcaaagacacggga

aaaaccagctttcagaattagatggccccaatggggtagtgacctctacttctgacatgc

taaaagtggctactgacttttataagaagctttttgcttttgagactaaacccgatattc

atcttgaggctgatttttgggaagaggatgagaaagtttctgagcttgataaaattaact

tagagaaacctttttatgaagaggaaattaagcaagctatcaagaactcttatgcctgtg

gggctcctagccctgatggtctctctttttattttaccaaactttttgggacatcataaa

agaggacttcatgtggatggtcagggattttgaagctggttctctagatatttgtagact

taattttgctttgattactcttattccaaaaaatccttttgctagagaaatgaaaaattt

taggcctattagcctaagtaactgtgctgttaagattttctctaaagccatgactactag

agttgctcccttgtgtgataaacttatttcatctaaccagactgccttcattaaggggag

gtttattctggaaagtgtcgtgatggctcatgaagtgattcatgaggttcacaggtctgg

gagcagtggacttattcttaagttggattatgagaaggcttatgatagggttaattggga

ttttattaaagaaatgctcctttctaggggttttggaactaaatgggttaattgggtctt

tagtaccctgcatcaagggacctttcaggtcaggatcaatgagaccaatggccacatttt

gtggctgggaaggggctaaaacagggtgatcctcactctcctttacttttcaacttggtt

gcagatgtgtttactaaaatgctatccaaagctgctagagctgaccttattgttggcctc

ttgccccaggtgattcctggaggtgtcatcagcttgcagtatgctgatgacactatccta

tttttgcaagattcaatggcttatgccaagaaactgaaatggatcctgacttgttttgaa

agcctttcgggtatgaaaattaactttaataaaagtgacctgcataccatcaatgttcct

ttggagatgtccaacaattttgctcaaattttctgctgtcagtttggagattttccattt

aaatatctgggagtgcctcttcattataaaaaactcaggagggaggacatccagcatatt

attgataggattatcaaaaatattgctggttggctgggtaaacatctttcctacagaggc

aaattaattctgctaactacttgtttagttagcattcctgcttatctgatgtctgtcatg

aaattccctaagtgggccattgatgccattacctctcagatgtctcatttcttttggggc

aatatgggtgataatcataaataccatctggctagttgggggctgattgcttagaaaaaa

gaatttgggggtctaggggttccaaatcttagagaatataacatggccctccttgcctca

tggggaaggagatcctatgattgtagggatagtgattggaagaaagtcattaacttcaaa

tatgctactgataaacccaatctctactgggggaaacctgggttaggctctcctttttgg

aaaagcctaacctgggctttggaaggtgccaaaacttttactagttggattcctggaaat

ggtgtaaacattgccttctggcatgattcctggattgggggatactcccttaaaaccata

ttctgggagttgtttgacatttgccaacaacaagatgccactgtggcccaggtttgggat

ggtaatactctacagctgactttcaggaggtgtgtagatgatagcattatggaaaaatgg

tatgagctatgtgagatagttagtgcttttatccctgggtgttctgggatatatacagtt

aaatccttttacaaaatgattaactttggtggaatatcttctcatattaaagatgccatt

tggaaaattaaagttccacctaatatccatgtttttctctggctggcttataacaacaag

tgcttgactagagataacctagccaagaggagacacgtggatgatcctacctgtgttttc

tgtgcggaaaatgaatcgattaatcatctattttttgaatgtgtggtagccagtcatatt

tggaatacaattgctgaggggatggacatccctattccatcttcctttttgtctctgtct

gctttgtggaaagtgaaaaagaagaatgaagctatcaatactgttacctctgctaccttg

tggggcttatggcttctgcgcaatgaatttgtgtttcaggggcgaaaatggcgaagcatt

cgctgcatcatggacctggtgggagggctgatgagacaatggaagattttgtgctccgac

gttgacgtccagggtgcgcttcttctccggcggaggcggcggcagcggcttccctggcgg

aggcgggcctgcctccggtggtgaccgcggtgaccgcggcaaagcacccactgacgctgg

cagccacacccgcaccggcaacaacactcgcgggcacggcgctgggcgcggccgtgggcg

cggcggccccaacggcgggcgcggccagcagcatctgatcccggggggcagcactccctg

gatggggtacttcgccccatggggcacgccgttccctccccaagggcgcgcgccctgggt

gcctccaaacgccgctagggtgctcggtccgcggcccggcaaccctgctcacgcctaccc

cgtcgtctaccccggcgcgccctcctcttccggccaccctccaccgccgccatcatggga

tcaggccggcctgatcgcggccatgcagaacatgtccatgcagcagcagcagcccgacga

gtggtacctcgactcgggcgcctcctcccacgtgacagagaacccaggtaacctcgacac

gttctgttctccctccaagtattcttctcgcagtatcattgtaggggatgggtctcacct

cgcgttgtcacaaacctcatctccgttcggcgtttttgcattgataattctgtcaccatt

gaattttacccatttgtctttgttgtgaaggatctagccacttagaccccgctcatgagc

tgcagtagcttcggtcagctttactccttcgccggcggcaacaccaccaccacggccgct

ctctccgtcaacatcggcaacctttggcatcggcgccttggtcacccaagcgctgcctca

ctgtctcgcttagccaaatagtttttaccagattgtaataacacacttcacccccgaggt

gcttgtgatgcttgtcaattaggccatcagacacgtttacctttcccagattctatgtca

tttaccacttttccttttcaactagtgcattgtgatctctggacgtctctggtcgctagc

ttctctggctataaatactacttgcttgttatggatgacttcactcattttacgtggacc

tttcctctcagtcacaaatccgacgctgctgccactcttgaacacttttacatgtatgtt

ctcgctcagtttcacgttcttctccaatgcgtccaatgcgacaatgggggcgagttcctc

aacgttcgcctccgcacctttctctacgatcgcagcgtcacccttcgtctctcctgccca

cacacttccccgcaaaacggcaaggccgagcgcgccattcgctccacgaacgacatcctt

cgcactctccttctccaagcgcatatgcctcctcccttttgggttgaagccctccacacc

gccacctccctcctcaatatcagaccatcccgcgccatctctcactacactccttacttc

ctgctccatggcacttccccctcatacgcctcccttcgcacctttgggtgcttgtgctac

ccaaacctgtacgccaccatgccacataaactcgcccctcgctcggttcgttgcattctc

ctcggcatgcctctcgagcataaaggctatcgttgcctcgacctggaatcccgacgcgtt

atcacctcccggcacgttatttttgatgaggataccttcccatacgctgctgcctcggac

ggccacaccatcgcacgagcgcatgcaccccgcgccacagaaaatccccccgcgatccgc

cttggatcggccgcccacccacccgaaccaacccgcacgcatgccgcaccggccccactc

tcccgcccgcccgcaccagctcagccgcatgcagcgccgcgccaaatcccccgcatgcag

ctccctccccccacttcctcggatcgcccgccagattcgcctagccccagcgcatcttta

ccgaaatccgtgcctccctcccccaccactcccggcccgcacccgcgcgtcgcgccttcc

tctcccactagctcaccgctgttggggaacgtagcaaaaattcaaaattttctacgcatc

accaagatcaatctatggagtaatctagcaacgaggggaaggggagtgaatctacatacc

cttgtagatcgcgatgcggaagcgttgcaagaacgcggatgagggagtcgtactcgtagc

gattcagatcgcggttgattccgatctaagcaccgaaannnnnnnnnnnnnnnnnnnnnn

nnnnnnnnnnnnnnnnnnnnnnnnnnnnnnnnnnnnnnnnnnnnnnnnnnnnnnnnnnnn

nnnnnnnnnnnnnnnnnnnnnnnnnnnnnnnnnnnnnnnnnnnnnnnnnnnnnnnnnnnn

nnnnnnnnnnnnnnnnnnnnnnnnnnnnnnnnnnnnnnnnnnnnnnnnnnnnnnnnnnnn

nnnnnnnnnnnnnnnnnnnnnnnnnnnnnnnnnnnnnnnnnnnnnnnnnnnnnnnnnnnn

nnnnnnnnnnnnnnnnnnnnnnnnnnnnnnnnnnnnnnnnnnnnnnnnnnnnnnnnnnnn

nnnnnnnnnnnnnnnnnnnnnnnnnnnnnnnnnnnnnnnnnnnnnnnnnnnnnnnnnnnn

nnnnnnnnnnnnnnnnnnnnnnnnnnnnnnnnnnnnnnnnnnnnnnnnnnnnnnnnnnnn

nnnnnnnnnnnnnnnnnnnnnnnnnnnnnnnnnnnnnnnnnnnnnnnnnnnnnnnnnnnn

nnnnnnnnnnnnnnnnnnnnnnnnnnnnnnnnnnnnnnnnnnnnnnnnnnnnnnnnnnnn

nnnnnnnnnnnnnnnnnnnnnnnnnnnnnnnnnnnnnnnnnnnnnnnnnnnnnnnnnnnn

nnnnnnnnnnnnnnnnnnnnnnnnnnnnnnnnnnnnnnnnnnnnnnnnnnnnnnnnnnnn

nnnnnnnnnnnnnnnnnnnnnnnnnnnnnnnnnnnnnnnnnnnnnnnnnnnnnnnnnnnn

nnnnnnnnnnnnnnnnnnnnnnnnnnnnnnnnnnnnnnnnnnnnnnnnnnnnnnnnnnnn

nnnnnnnnnnnnnnnnnnnnnnnnnnnnnnnnnnnnnnnnnnnnnnnnnnnnnnnnnnnn

nnnnnnnnnnnnnnnnnnnnnnnnnnnnnnnnnnnnnnnnnnnnnnnnnnnnnnnnnnnn

nnnnnnnnnnnnnnnnnnnnnnnnnnnnnnnnnnnnnnnnnnnnnnnnnnnnnnnnnnnn

nnnnnnnnnnnnnnnnnnnnnnnnnnnnnnnnnnnnnnnnnnnnnnnnnnnnnnnnnnnn

nnnnnnnnnnnnnnnnnnnnnnnnnnnnnnnnnnnnnnnnnnnnnnnnnnnnnnnnnnnn

nnnnnnnnnnnnnnnnnnnnnnnnnnnnnnnnnnnnnnnnnnnnnnnnnnnnnnnnnnnn

nnnnnnnnnnnnnnnnnnnnnnnnnnnnnnnnnnnnnnnnnnnnnnnnnnnnnnnnnnnn

nnnnnnnnnnnnnnnnnnnnnnnnnnnnnnnnnnnnnnnnnnnnnnnnnnnnnnnnnnnn

nnnnnnnnnnnnnnnnnnnnnnnnnnnnnnnnnnnnnnnnnnnnnnnnnnnnnnnnnnnn

nnnnnnnnnnnnnnnnnnnnnnnnnnnnnnnnnnnnnnnnnnnnnnnnnnnnnnnnnnnn

nnnnnnnnnnnnnnnnnnnnnnnnnnnnnnnnnnnnnnnnnnnnnnnnnnnnnnnnnnnn

nnnnnnnnnnnnnnnnnnnnnnnnnnnnnnnnnnnnnnnnnnnnnnnnnnnnnnnnnnnn

nnnnnnnnnnnnnnnnnnnnnnnnnnnnnnnnnnnnnnnnnnnnnnnnnnnnnnnnnnnn

nnnnnnnnnnnnnnnnnnnnnnnnnnnnnnnnnnnnnnnnnnnnnnnnnnnnnnnnnnnn

nnnnnnnnnnnnnnnnnnnnnnnnnnnnnnnnnnnnnnnnnnnnnnnnnnnnnnnnnnnn

nnnnnnnnnnnnnnnnnnnnnnnnnnnnnnnnnnnnnnnnnnnnnnnnnnnnnnnnnnnn

nnnnnnnnnnnnnnnnnnnnnnnnnnnnnnnnnnnnnnnnnnnnnnnnnnnnnnnnnnnn

nnnnnnnnnnnnnnnnnnnnnnnnnnnnnnnnnnnnnnnnnnnnnnnnnnnnnnnnnnnn

nnnnnnnnnnnnnnnnnnnnnnnnnnnnnnnnnnnnnnnnnnnnnnnnnnnnnnnnnnnn

nnnnnnnnnnnnnnnnnnnnnnnnnnnnnnnnnnnnnnnnnnnnnnnnnnnnnnnnnnnn

nnnnnnnnnnnnnnnnnnnnnnnnnnnnnnnnnnnnnnnnnnnnnnnnnnnnnnnnnnnn

nnnnnnnnnnnnnnnnnnnnnnnnnnnnnnnnnnnnnnnnnnnnnnnnnnnnnnnnnnnn

nnnnnnnnnnnnnnnnnnnnnnnnnnnnnnnnnnnnnnnnnnnnnnnnnnnnnnnnnnnn

nnnnnnnnnnnnnnnnnnnnnnnnnnnnnnnnnnnnnnnnnnnnnnnnnnnnnnnnnnnn

nnnnnnnnnnnnnnnnnnnnnnnnnnnnnnnnnnnnnnnnnnnnnnnnnnnnnnnnnnnn

nnnnnnnnnnnnnnnnnnnnnnnnnnnnnnnnnnnnnnnnnnnnnnnnnnnnnnnnnnnn

nnnnnnnnnnnnnnnnnnnnnnnnnnnnnnnnnnnnnnnnnnnnnnnnnnnnnnnnnnnn

nnnnnnnnnnnnnnnnnnnnnnnnnnnnnnnnnnnnnnnnnnnnnnnnnnnnnnnnnnnn

nnnnnnnnnnnnnnnnnnnnnnnnnnnnnnnnnnnnnnnnnnnnnnnnnnnnnnnnnnnn

nnnnnnnnnnnnnnnnnnnnnnnnnnnnnnnnnnnnnnnnnnnnnnnnnnnnnnnnnnnn

nnnnnnnnnnnnnnnnnnnnnnnnnnnnnnnnnnnnnnnnnnnnnnnnnnnnnnnnnnnn

nnnnnnnnnnnnnnnnnnnnnnnnnnnnnnnnnnnnnnnnnnnnnnnnnnnnnnnnnnnn

nnnnnnnnnnnnnnnnnnnnnnnnnnnnnnnnnnnnnnnnnnnnnnnnnnnnnnnnnnnn

nnnnnnnnnnnnnnnnnnnnnnnnnnnnnnnnnnnnnnnnnnnnnnnnnnnnnnnnnnnn

nnnnnnnnnnnnnnnnnnnnnnnnnnnnnnnnnnnnnnnnnnnnnnnnnnnnnnnnnnnn

nnnnnnnnnnnnnnnnnnnnnnnnnnnnnnnnnnnnnnnnnnnnnnnnnnnnnnnnnnnn

nnnnnnnnnnnnnnnnnnnnnnnnnnnnnnnnnnnnnnnnnnnnnnnnnnnnnnnnnnnn

nnnnnnnnnnnnnnnnnnnnnnnnnnnnnnnnnnnnnnnnnnnnnnnnnnnnnnnnnnnn

nnnnnnnnnnnnnnnnnnnnnnnnnnnnnnnnnnnnnnnnnnnnnnnnnnnnnnnnnnnn

nnnnnnnnnnnnnnnnnnnnnnnnnnnnnnnnnnnnnnnnnnnnnnnnnnnnnnnnnnnn

nnnnnnnnnnnnnnnnnnnnnnnnnnnnnnnnnnnnnnnnnnnnnnnnnnnnnnnnnnnn

nnnnnnnnnnnnnnnnnnnnnnnnnnnnnnnnnnnnnnnnnnnnnnnnnnnnnnnnnnnn

nnnnnnnnnnnnnnnnnnnnnnnnnnnnnnnnnnnnnnnnnnnnnnnnnnnnnnnnnnnn

nnnnnnnnnnnnnnnnnnnnnnnnnnnnnnnnnnnnnnnnnnnnnnnnnnnnnnnnnnnn

nnnnnnnnnnnnnnnnnnnnnnnnnnnnnnnnnnnnnnnnnnnnnnnnnnnnnnnnnnnn

nnnnnnnnnnnnnnnnnnnnnnnnnnnnnnnnnnnnnnnnnnnnnnnnnnnnnnnnnnnn

nnnnnnnnnnnnnnnnnnnnnnnnnnnnnnnnnnnnnnnnnnnnnnnnnnnnnnnnnnnn

nnnnnnnnnnnnnnnnnnnnnnnnnnnnnnnnnnnnnnnnnnnnnnnnnnnnnnnnnnnn

nnnnnnnnnnnnnnnnnnnnnnnnnnnnnnnnnnnnnnnnnnnnnnnnnnnnnnnnnnnn

nnnnnnnnnnnnnnnnnnnnnnnnnnnnnnnnnnnnnnnnnnnnnnnnnnnnnnnnnnnn

nnnnnnnnnnnnnnnnnnnnnnnnnnnnnnnnnnnnnnnnnnnnnnnnnnnnnnnnnnnn

nnnnnnnnnnnnnnnnnnnnnnnnnnnnnnnnnnnnnnnnnnnnnnnnnnnnnnnnnnnn

nnnnnnnnnnnnnnnnnnnnnnnnnnnnnnnnnnnnnnnnnnnnnnnnnnnnnnnnnnnn

nnnnnnnnnnnnnnnnnnnnnnnnnnnnnnnnnnnnnnnnnnnnnnnnnnnnnnnnnnnn

nnnnnnnnnnnnnnnnnnnnnnnnnnnnnnnnnnnnnnnnnnnnnnnnnnnnnnnnnnnn

nnnnnnnnnnnnnnnnnnnnnnnnnnnnnnnnnnnnnnnnnnnnnnnnnnnnnnnnnnnn

nnnnnnnnnnnnnnnnnnnnnnnnnnnnnnnnnnnnnnnnnnnnnnnnnnnnnnnnnnnn

nnnnnnnnnnnnnnnnnnnnnnnnnnnnnnnnnnnnnnnnnnnnnnnnnnnnnnnnnnnn

nnnnnnnnnnnnnnnnnnnnnnnnnnnnnnnnnnnnnnnnnnnnnnnnnnnnnnnnnnnn

nnnnnnnnnnnnnnnnnnnnnnnnnnnnnnnnnnnnnnnnnnnnnnnnnnnnnnnnnnnn

nnnnnnnnnnnnnnnnnnnnnnnnnnnnnnnnnnnnnnnnnnnnnnnnnnnnnnnnnnnn

nnnnnnnnnnnnnnnnnnnnnnnnnnnnnnnnnnnnnnnnnnnnnnnnnnnnnnnnnnnn

nnnnnnnnnnnnnnnnnnnnnnnnnnnnnnnnnnnnnnnnnnnnnnnnnnnnnnnnnnnn

nnnnnnnnnnnnnnnnnnnnnnnnnnnnnnnnnnnnnnnnnnnnnnnnnnnnnnnnnnnn

nnnnnnnnnnnnnnnnnnnnnnnnnnnnnnnnnnnnnnnnnnnnnnnnnnnnnnnnnnnn

nnnnnnnnnnnnnnnnnnnnnnnnnnnnnnnnnnnnnnnnnnnnnnnnnnnnnnnnnnnn

nnnnnnnnnnnnnnnnnnnnnnnnnnnnnnnnnnnnnnnnnnnnnnnnnnnnnnnnnnnn

nnnnnnnnnnnnnnnnnnnnnnnnnnnnnnnnnnnnnnnnnnnnnnnnnnnnnnnnnnnn

nnnnnnnnnnnnnnnnnnnnnnnnnnnnnnnnnnnnnnnnnnnnnnnnnnnnnnnnnnnn

nnnnnnnnnnnnnnnnnnnnnnnnnnnnnnnnnnnnnnnnnnnnnnnnnnnnnnnnnnnn

nnnnnnnnnnnnnnnnnnnnnnnnnnnnnnnnnnnnnnnnnnnnnnnnnnnnnnnnnnnn

nnnnnnnnnnnnnnnnnnnnnnnnnnnnnnnnnnnnnnnnnnnnnnnnnnnnnnnnnnnn

nnnnnnnnnnnnnnnnnnnnnnnnnnnnnnnnnnnnnnnnnnnnnnnnnnnnnnnnnnnn

nnnnnnnnnnnnnnnnnnnnnnnnnnnnnnnnnnnnnnnnnnnnnnnnnnnnnnnnnnnn

nnnnnnnnnnnnnnnnnnnnnnnnnnnnnnnnnnnnnnnnnnnnnnnnnnnnnnnnnnnn

nnnnnnnnnnnnnnnnnnnnnnnnnnnnnnnnnnnnnnnnnnnnnnnnnnnnnnnnnnnn

nnnnnnnnnnnnnnnnnnnnnnnnnnnnnnnnnnnnnnnnnnnnnnnnnnnnnnnnnnnn

nnnnnnnnnnnnnnnnnnnnnnnnnnnnnnnnnnnnnnnnnnnnnnnnnnnnnnnnnnnn

nnnnnnnnnnnnnnnnnnnnnnnnnnnnnnnnnnnnnnnnnnnnnnnnnnnnnnnnnnnn

nnnnnnnnnnnnnnnnnnnnnnnnnnnnnnnnnnnnnnnnnnnnnnnnnnnnnnnnnnnn

nnnnnnnnnnnnnnnnnnnnnnnnnnnnnnnnnnnnnnnnnnnnnnnnnnnnnnnnnnnn

nnnnnnnnnnnnnnnnnnnnnnnnnnnnnnnnnnnnnnnnnnnnnnnnnnnnnnnnnnnn

nnnnnnnnnnnnnnnnnnnnnnnnnnnnnnnnnnnnnnnnnnnnnnnnnnnnnnnnnnnn

nnnnnnnnnnnnnnnnnnnnnnnnnnnnnnnnnnnnnnnnnnnnnnnnnnnnnnnnnnnn

nnnnnnnnnnnnnnnnnnnnnnnnnnnnnnnnnnnnnnnnnnnnnnnnnnnnnnnnnnnn

nnnnnnnnnnnnnnnnnnnnnnnnnnnnnnnnnnnnnnnnnnnnnnnnnnnnnnnnnnnn

nnnnnnnnnnnnnnnnnnnnnnnnnnnnnnnnnnnnnnnnnnnnnnnnnnnnnnnnnnnn

nnnnnnnnnnnnnnnnnnnnnnnnnnnnnnnnnnnnnnnnnnnnnnnnnnnnnnnnnnnn

nnnnnnnnnnnnnnnnnnnnnnnnnnnnnnnnnnnnnnnnnnnnnnnnnnnnnnnnnnnn

nnnnnnnnnnnnnnnnnnnnnnnnnnnnnnnnnnnnnnnnnnnnnnnnnnnnnnnnnnnn

nnnnnnnnnnnnnnnnnnnnnnnnnnnnnnnnnnnnnnnnnnnnnnnnnnnnnnnnnnnn

nnnnnnnnnnnnnnnnnnnnnnnnnnnnnnnnnnnnnnnnnnnnnnnnnnnnnnnnnnnn

nnnnnnnnnnnnnnnnnnnnnnnnnnnnnnnnnnnnnnnnnnnnnnnnnnnnnnnnnnnn

nnnnnnnnnnnnnnnnnnnnnnnnnnnnnnnnnnnnnnnnnnnnnnnnnnnnnnnnnnnn

nnnnnnnnnnnnnnnnnnnnnnnnnnnnnnnnnnnnnnnnnnnnnnnnnnnnnnnnnnnn

nnnnnnnnnnnnnnnnnnnnnnnnnnnnnnnnnnnnnnnnnnnnnnnnnnnnnnnnnnnn

nnnnnnnnnnnnnnnnnnnnnnnnnnnnnnnnnnnnnnnnnnnnnnnnnnnnnnnnnnnn

nnnnnnnnnnnnnnnnnnnnnnnnnnnnnnnnnnnnnnnnnnnnnnnnnnnnnnnnnnnn

nnnnnnnnnnnnnnnnnnnnnnnnnnnnnnnnnnnnnnnnnnnnnnnnnnnnnnnnnnnn

nnnnnnnnnnnnnnnnnnnnnnnnnnnnnnnnnnnnnnnnnnnnnnnnnnnnnnnnnnnn

nnnnnnnnnnnnnnnnnnnnnnnnnnnnnnnnnnnnnnnnnnnnnnnnnnnnnnnnnnnn

nnnnnnnnnnnnnnnnnnnnnnnnnnnnnnnnnnnnnnnnnnnnnnnnnnnnnnnnnnnn

nnnnnnnnnnnnnnnnnnnnnnnnnnnnnnnnnnnnnnnnnnnnnnnnnnnnnnnnnnnn

nnnnnnnnnnnnnnnnnnnnnnnnnnnnnnnnnnnnnnnnnnnnnnnnnnnnnnnnnnnn

nnnnnnnnnnnnnnnnnnnnnnnnnnnnnnnnnnnnnnnnnnnnnnnnnnnnnnnnnnnn

nnnnnnnnnnnnnnnnnnnnnnnnnnnnnnnnnnnnnnnnnnnnnnnnnnnnnnnnnnnn

nnnnnnnnnnnnnnnnnnnnnnnnnnnnnnnnnnnnnnnnnnnnnnnnnnnnnnnnnnnn

nnnnnnnnnnnnnnnnnnnnnnnnnnnnnnnnnnnnnnnnnnnnnnnnnnnnnnnnnnnn

nnnnnnnnnnnnnnnnnnnnnnnnnnnnnnnnnnnnnnnnnnnnnnnnnnnnnnnnnnnn

nnnnnnnnnnnnnnnnnnncttccttcaacctaagaaactcatgactatctctaccacta

ccggccccatttctcccatcccacctacttacaaacatgctctcaaagacccaaactggt

acaatgcaatgcttgaagagtttaatgctttgttggacaacaaaacgtggtctttggttg

cttgccctgcaggtgtgaaacttggtgaccggcaagtgggtgttccgccacaagctacat

ccggacggttcgttggctcggtacaaggcgcggtgggttcttcgcgggttcacgcagcag

cccggcgtcaactacggggagactttcagccccatggtgaagccggccaccatccgcgtc

atcctcgccctcgccgtcagcaacaactgggccataaaccaactcgacgtcaagaacgcc

ttcctccatggcaccctcgacgaggtcgtctacagtcagcaacctgctggcttcgtcgac

accgcacgcccctccgccgtgtgcaagcttcacaagtccttgtatgggcttaaacaggct

ccgcgggcgtgatttcagcggttcaccacgttccttgcgtcgctcggcttcgtggcgtcc

aagtgtgactcgtctctcttcatcctccgccgcggggccgcggtggcgtatcttcttctc

tacgtggacgacataattctcacagctagcactgccgcgctgctcacctccatcaccact

gctctcacatcggagttctccatgacaaacctcggcgcgcttcaccacttcctcggcatc

aatgtacagcgcagtgccgccggtttgtttctctcgcagcagcagtacacgcttgagatc

ctcgaccgcgcctccatgctaaactgcaaacccgtcgctactccggtcgacaccaaagca

aagctctccgccaccgagggtcctccggctcccgatccctcgctctttcgcagcctcgct

ggtgccctacaataccccacactcactcgccctgacatagcctacgctgtgcagcaagta

tgtctcgtcatgcactcgccgcgcgccccgcatgtcgccctgcttaagaggatcttgcgc

tatcttcgcggcacctcccatctcggccttcacctccgcccgtctgcctcatcgcaactc

gtcacctacactgatgctgactgggccgggtgcccctacacacgccggtcgacgtccggc

tactgcatgtacattggggacaaccttgtgtcctggtcctcgaagcgacaaaccacggtg

tcgcggtccagttcggaggcggagtatcgagccgtcgccaacgccgtcgccgaatcgtgt

tggctccggcaactcctacatgaggtcgggcgtccgccggcacgcgccaccgtcgtctac

tgcgacaatgtgtccgcggtgtatctctcctccaaccctgtacaacaccagcgtaccaag

catgtggagatcgatcttcacttcatccggatcgcgtcgccttcggcgatgtccgcgtcc

tgcacgttccaacaacctcgcagttcgccgacatcttcaccaaggggctgcccacgagca

tcttcaccgagttccggtccagcctcaacgtcgtcgacgaccccgtttcgactgcggggg

gctgttggaagacgcccgttgtgcccacctgaccgagtcctgcggtggaccgaacacgcc

gcgcacgtcgcacacagggcgtgcgtccgttgcgcacgatctccctctcgggcggcctcg

tctgttgtagctcatgtgtatatatatgtgtaactgtgtgtatgataaatatacggtgaa

ttacacctttcagctttcgttactccatgtcaagcacctgtacttgcacctaatttccct

aagcgctgccgtgtcggtcgccgctctaaacctacccatgatttgtcggtttaagttgag

attctttttgtccctagattcgtagatggtgttgaaatcaccattgatgagccatggctc

gccgctgggggggggggggcggcggtctgctctatctaattcaccgaggaaaccatcttt

tgtggcgtggtccgtgggtctgtagacgatacttgaccaaaaactagtcgaaggaactaa

tgttacttttgccgtgatggagtagggccggatgcagtgggagactacagaactaaatta

tcccagaagatcgacgccccacagcagatggccgcggccggcagcaccacacacatagat

agtcaaggtccgccaacctcgcgcacaagctgctgcgaccaagactcatgctttgtttct

tgaatgcagaggatgtccagccggtgtgcgtcttctacgtcacataccgcggataatggt

gctcatatggatggcactcgatacccaattatgagctggaacttccatgggcttgtggac

ctttttgaagaaccatcagaggtgttttcagtccagaggtagttgttccacagaggctca

tctgtggcagtgttcttgcggacccgccagagatgggggtgtacctcactcggtatgccc

cgttcgctgtgcttccggtgatgggttgttctgtactactccctccgttctaaaataatt

gaagttcatggattgtcttaagtcaagcttctctaagtttgacgaagtctatataaaaat

atagcaacatctacaaaaccaaatttacctcattagattcattatgacatatattttcgt

agatctttgtagatctgtaaacttggtccacacctttcacggtgttacaactgacacacc

cttcatattgttaattaagcgacagtattcattgactaattgtacctttgttgtgtgtgt

ttagatgttccgatctgagatggag

GCAATGAGAATGTCAAGGAGACAGGGGCCCCTATCAGTGCTTCGCTGGGTGCCATGAGAG

CTCTTATTGGGAAGTTGGACATGTTGCTAGATCCTTCATCCCCTCAGGGATGCTCCTCCA

AGAGAGTCAAGGATGAGACGCACCTCCTCAAAGATGACGTTGAAACGATGAGCTCCTACC

TTGATAACCTGTCAGAGGGGGAGGATCCTCCTCCGACTGCCAAGTGCTGGATGAATGAGG

CGCGTGACTTGTCTTACGACATGGAGGATTACATCGACAATTTATTATTTGTGCCGCCTG

AAGACCCCTCCCTTGTTACCAACAACACCAAGACCACCATATCCCTCCGCAAATGGTTCC

GTCGTGTCAAGACTCCCAAGACTCAG

gtcattggtgctgaaactctattagaattcaggatgtatgtccaggaggcgattgaacgg

catcggaggtacgatctccattcttgcaacaacataacgcgtaggtttgtgtcccttggc

cctatgcctattccaaggcggtatgaagaagcttccgacatagtaatcgatggccggatg

aacaaatttatcaactcactggctaacgatggggacaagcagctcaaggtgctatctatt

cttggatctgcttgtcttggtaaaactacagttgctagagtgttgtacaacagatttagg

aagcaatatagttgtcgagctttcattcgagtgtccaagaaacctgatacgaagagaatt

ttctttgacatgctatcgcaacttcagcggcagcatcccccacaatattataaggaaact

gaactcattcaaaatatcatgcaatatctgcaagataaaaggtagtgcttttttatttct

tcattcaagatatatcgaaaatcccatgttctctaatgtgagttaattattagcatgcag

gtatttaattatcattgatgatgtatgggctgcatcagtgtgggatattattaatcatgc

ttttccaaagggtagtcatggtagcagaataataacaaccacacagatcgaagacattgc

attaacctgttgcagttatcagtcagagtatgtttttgagatgaaacacctagatgatga

tcactctagaaagcttttctttaacagactttttggctccgaatgtgattgtcctgaaca

gttcaaagaggttctaaatgaaattgttgaaacatgtgatggtttgccactagctacagt

gagcatatctagtcttttatcaagccagcctgccatgacaattgatctattaatgtgcat

tcagcagtcgtttagctcttgtttttcggcatcagaaagaacaagacaagcactgaattt

gagtttcaacaatcttcctcaatatttgaagacatgtttgctttatcttagtatgtatcc

agagggctatgtgttctaaaaggatgatctggtgaagcaatgggtagctgaaggtttgat

ctacacaacagaagggcaagacattgatgaagttgctgaaagctatctggatcaacttgt

tgggagaagattcatccagccgatatgtgtgaactacaacaatgaggtgttgtcctatgc

agtgcatgacatggtacatgatcttattgcacacaagtctgcagaagagaatttcattgt

ggcaatagactacagccggaagaatgtttcactctctcaaaaggtccgtcgactgtctct

cctctttggcgatgcgagatatgccaagacaccagcaaacatcacaaagtcacaagttcg

gtcacttaggttttttggattagtcaactctatgccttgtattacagagttcaagcttct

tcgtgtcctgaacctgcaactactcagtcatactggcgatgacgacagccctgtagacct

cactgggatttcaaaaatgtttcaactgagatatttgaagattgcaggtaatgtctgcat

aaaactgccaaaccatgtgctacaatgtttggaaatcttggatattatggatgtaatagt

tgcttgtgtcccacgagatatccattttccaaacttgttacaccttggtcttcctgttga

taaaaatctgccggattggattaacagaaggatgtctcccggtgtggagagagtacgaaa

gctgcatgccctgcaggatcttcatctaaccagattttctacacattcttctgaccatct

gaatagaagcatgggttctattttatccttaattggagggcatggtaacctgaaaactgt

agtagtggctcatggctcaatggctaaaaatgctcgtggtgcttcagatgtcaccctttc

gtgggatctcctggcacccccacttctccagagatttgagttttcaccacatagccgtat

catattcccacgaattcctctgtggattgataaacatggcaagctaagaattttgaagat

taccgtacaggaactattgtccaatagtgttggtattctcagaacattgcctgccctcac

tgctctgtccctgtatgtggagaaggcgccctacgacaaaatcatattcgacaaggccgg

gttctcagttctcaagtatttttgaggttcacaagtggcatagcttggataaaatttgtg

gagggtgcaatgcgtaatctctggaagcttaagttagtttttgattctattccctcattg

gagaaagatggctttgcagttaccagcatcgagcatatttttttttcccgaaaaggggga

tctccccggcctctgcatcagagtgatgcatacggccatcttattaacgaaataaaaggt

tccaacaaggttccaaggtctccgactgagaagtaataaaaagacagctcacacagagca

aaagaggctggacacacagactagccaagataagatgccacaaccggctgactaaagata

gataggtaaactaattgcctatcctattacatgaccgccatccaaaccggttgaagatat

cccgagttaccatctcccagcggatagatccagtaaccaaatgctccctggccgccatcg

gagtgagtagcgaccatgaacggatcatggccgtggcacggaaaataacctgcaaaaaat

gaatacgtgatgttctgttaaaaaccaaatcatttctgcaagtccagatagtccacaaca

tagcgcaaactccaacacgaatatgtttcgttaattcaggctcaatcccagtgagccatg

ccccaaataacgtagtgacagaattcggtggagtaatattaaaagcaatgtgaactgtcc

gccaaagtactctggcaagcgggcaatcaaaaaagagatgtttgatcgtctcgtcccgat

cacagaagctacacctcgtaggtcctctccaattacgcttaatcaagttatccttggtta

aaataacctgtttatggacaaaccacataaacacttttattttcagaggaactttgacat

cccaaacatgcttggaagtaggaatggagctcgaattaattacatcaatatacattgatt

taaccgtgaattctccagacctagtcagtttccagcgcaattcatcgggttgttgagaaa

gatggacatccattagtctccgaaccagcatcgagcatatgccaggccttaaagagatct

ggacaaaatttggggggtcagctgctgatttagagtatgccttaatgaccttcattagta

accatccgagcaatcctataatcaacatgccatcagacaatactctgggggaacaaaaac

catataggaatctggagcacgactcagatgaggttcttaaggaagatgagccatatagga

atctggagcaggaatcagatgaaattctggaggaacaagagccagataaatacggtgaga

gattaaagaggcgatcggctgataaaaggtactaccaatgttccttggaaagacaaggaa

atatgccatcctttacttcaacttttattgggctgagtgcacccacgtggcgcggaggcc

tcaataaagaaaatatttttataagagaaacatcgacttcttcttcagttaacctgatat

aattcttatatgttctaacgaagctaatggttgctgtaataatcaatcaggattccatgg

tcaccggatccctcttcgcgcctgcatgttccaggtacattttctgtcaactgggtgtaa

tataaatgtttcgccatcaagttattcatccacttggtggtatttgggaggctcttcaag

agaaaactaagcagcttggtctatgtgatataaaatttgtcacgaactaccaattcggaa

acgattaattctctccaattggtgacttgctagagattgaggcttgagcggaagcaggta

actgtagcaaatccatctagttaccctcagcaatagctctgtgctcaaaatatcgtgtat

tggggtgaacctttccccaccctccctcccgctcgccccccacgaggcgacgggaggaaa

ccctaccgcgccgccgccaactctttccccgccccctcctcctccctcgccgccgccaga

gggcgcggccgggcgaagcctggccggcggcggcggcggcgaggcctcctcgttccccct

cccagtggccttggcgcgggctgacgcggctggatcggggtgcctcgctatcccggggcg

aggcggcgcggaggcgtggtgatggcgctgcgtgtggcggtggcgtgacgggctgggcgt

cggagggcgcgccggtgcgtggagcagcgggggcggcgagcggctggtgcgggcggtcct

ggcggcgggtggatctggcgtgacggcggttgtgtcgaggcagggcgtcggtgacgggtt

ccagtggcctgggccgactcctggctgggcgctgcggtggtgtgggtcgcgggcgacaag

gatctacgggcggccgcccctaccctggcttcgggctggtgggggtggcggcgcagtggt

ggtggctggcggggcgtgttggtcgagtccgcgtcggattcttcctggtctggcgctcgg

acgtcggcggacggcgggaggaggcatctccggtgagctttcatggctgcagatgtgggt

tgatttggtctccctcgtcggcgggcacgcggttgccgatggcctcgcggagatgtgtgt

cagctaccatggtggggcgacatgggggtgctcgcgggggagcttggtaggagggatagg

tggcctcaatgcggtcgcgccacctgtcgccggcacggtttgccggtccggatgcgtccg

ctcctccttcttcccctttccccgtgtttggcacggagcttggttggagggatgggtggc

ctcgatgcggtcgcgtcacccgtcgccgacacgatttgccggtccggattcgtccgctcc

cctttctccccctttcctcggccgtaggcgggttggctcaggtttaccagcggagcgtct

gcgggtggttccgtcggtcgagggccatcggttgatccaaagctggggcctttgtgtagg

ttgtctggttgcagggcggcggctctggcggtgggaggcgcgacgtttgtgggcggagcg

tgcgtctcagatgcgggctggcaaacatggccgcgtgggtggcatggttgcgggtggttg

gcggagttcaggttcaacggtggagtgtgagtgccggggtacaccacttgtccagtcctt

tgactggccggcggtggcggtggccgttgtcgccgtacccttcctggaggctccgtcgtg

gcgtttccactcttgtcgtcttgctccgggtgaaaacatgatcttcgctgatcgggcggt

ggcggctttccatggtcgcatccttcttggaggcaccgctttggaggcctggcttcgttt

tggtccggttcacctctcgcctggcaccattgtatcttgccttgggtgtgtgcagtgtgt

gcgttgttcggtatctcgattgtactcggtgatgtttgctttatataatataaagcgggg

aaccctttttcggcaatccatctagtttagttgcatacgtcggtccatgacgtcagcttc

tagttacaaatcagactctttgatcttgtgccagccgaatcagaaaagaatccctgattg

cacgtagcctggatttcaacctcctattttctcttactctttcttccttcaatgcttgct

caagcgacatgcatcttcccctattttcttgcctccaatcaatcacatacacggcttggg

catgcaacatgcacaccatgaaaaacatgcctaatttctggtcctgtctctcttcttttc

ttatgcacctaagttaggtgctaaaatagcatgctttattgttataccccctctgtaaac

taatataagaatctttagatcattactttcgtttacagagggagtacaaactaattcctc

cttttcttcgtaattgaggcacatatatcttcgtacaacatcaggggtatgcgtaagcga

agccaccgtattactttcctgacaaacaagacattgggacatcatgcgctgattctttgc

tgtttcttttcgcttgtgtcgtgtttcgccgctcagtggaccagggaaagcggtttcctt

tgcgtaattgctatgttgtttaattcttgggggtgtgttgagttatatatatactctctc

tgcaagcaggcacatgatattttttttttgctttttcattaagcattaccagttgctact

tttgagatgggaaatgagcctaatgtgggaatccctacggcaccgactactaatgataat

atgatgttgccggtcaatctcgaagcatttgcttgggaaaattatgcgagcactaacttg

gaagttctataaagatggaaagagtggcaggtttagaggatcagcagatggaagatgaca

cacgggtaatttacacattccaactgttttgaattattcttatgctctcttatggacgta

gctagtaggatagaggtgaagttagaaagtaatattcttgaggtaaataaatatatatta

gtgactagtatggaaacggataaatctgcgttcaagccttaggctctggagggtgtgtgt

gcctggttatttctttgccgatggccactctcggcaaactgacgacacgtggcaaccgcc

gtccccatctgccgtccgtgaagtcactttattttgtcgagaggtgttgtttgacactcg

gcaaagggtttgatgaatgtccatgttttggctctcggcaaagtcctgcttgcctgagag

gtgtatgccgggtggcctttgccgagtgcaactctcggcaaaggctttgccggcggttat

tggctctttgccgagtgtccgtggcacccggcgtataggtcgattccagtagtgcaaggg

gtagtccattttgatcttttaatttggaagctgtagatatattgattttatagctcgcga

agagaaatttaataatgtgccgttagagttattcgggatcacgaagatatagtgactgag

gaatcaaaattagaattcttttaattctaaggctattcggaatctgccaactatgctaga

catttgaactggtactgtaatgtgtaatttgacag

CAAGGAAATGTCAATTTCGAGAAAAAGCAGAGCAAAGACGAGGCGGACGAAGAATCATCC

ATCCACTCACCCAGGCGAGGGAGCGATACACCGCCGCCACCGCTGAAGAAGCCGGGCAAG

ATCACACTCCCCTCCCACGAGGCCACCATGGGCAAATTCTG

gtagcctcaaaccgtcatccctcttctttcgaatgtttgttttccttggacgtgttgctc

ttgcagtcgtgctgattgattttatttcactccggttgtggtcagctggtctacagcgcg

ggccggggacag

GACCCGGAGTGGGACGTCCAAGTACAGCGACGGCGACCTGCTCGTCAACAAGGATGGCTT

CCGCATCATACCGCAGACCAAGGAAGGCAAG

gtaagccactaaagcgcgacccagcacccaagatgaaaaattcctccgctcgggagaaaa

gaacgtagggctgcacttggcagattggtagttgtattggttttcataatttcattgatg

gcatgaacagactaagaaattgggatgtgtggttggattagtaggtatggaacaggggtt

tcgtattcttattcagaacaacaatttaggacagatgatactaggtaggccatgtagcat

tttattttatttcttttgggtggtgaatacataggtgattttcatgtgggatatccgcaa

atgcagtatgttactgaagtaaataatgatgtatctagtggaagagtgctgtctagcacc

atttcttgtgggagttgtctaattacattattgttcttaacctggcatgctcttcattca

tctaatttgtgtctcgccttctttctgttgacatactcgacttctctag

GCTCCTCCTGTAGAGCCGCCCGAGAACCATGAGTTAAGCATAGATGATCTGGATGCGATC

AAAGAGATTGGGAAAGGTAGTAGCGGGACCGTGCAATTGGTGCATCATAAATGGACTGGA

CAGTTTTTTGCTCTGAAG

gtattgtccgaaacatacattatctggtgcttcatgtgtgtggttcactggttccttcct

tccaacattttgcccatatgttgcaaagggtcttcacctccattggtgtattgtcatgtc

ttcag

GTTATACAGCTCAATGTTCAGGAAAGCATCCGCAAGCAGATTGCTCAGGAGTTGAAAATA

AACCTGTCAACGCAGTGTCAATATGTTGTCACATGCTATCAGTGTTTCTATATCAATGGT

GTTATTTCTATTGCTTTGGAGTATATGGATGGTGGATCTCTGGCTGATTTCCTGAAGGGT

GTTGGAACCATTCCAGAGTCCTACCTTGCTGCAGTTTGTAAGAAG

gccagtgagatgtcacataagcaaccacgtgattttttttgctaccggaatgccacctta

agattaagtagaccttttcaattgatagttacaacttccttgtgcag

GTGTTGAAAGGACTGATGTACTTGCATCACGAGAGGCGCATTATACACCGAGATCTCAAA

CCATCGAATACATTGATAAATCATATGGGTGAAGTAAAGATATCAGATTTTGGTGTTAGT

GCCATCATTTCTAATTCTTCTGCACAGCGAGATATGTTTACTGGCACATTTAACTACATG

GCA

gtgggtgaaagtttgttcaatttatgtaaatatgccgtccagtgtggcatgaaaagatgt

ccagcattttaatttcagctaaccaatctaaaatgtatactcttgacag

CCAGAAAGAATCAGTGGGCAGAAACATGGTTATATGAGTGATATCTGGAGCTTGGGCCTA

GTTATGCTGGAATGTGCCACCGGCAAGTTCCCATATCCTCCTTGTGATAGCTTCTATGAA

CTTCTTGAAGCTGTTGTCGACCAACCACCACCCTCTGCACCATCAGACAAGTTTTCACCA

CAGTTTTGTTCATTCATTTCTGCGTGGTATTTGTACTTCTCCATTGCCCCCTTCTGCCCC

TGTTAATTAATGTTACTTGAGCAGTATTTGCAAGTTGTTGAGATTTAAACTGTGAGCTTA

GATACCAAAATAATGGTAGTCGGTTTTG

taccttttgctttgtcgagtagtgattcgcacggcaacaatcagtgattctctggtagcg

gacacattatctgaacttccagatatacatatttttattctcttatcttcatggacagct

aaattgatttccttcccttacgggatatcagtgtcgaaaagaaggctaatatctgttttc

ttaatttcttctttctggattttcagtatgcaaaaaaatgcgccagataggtcatatgct

caactcctatcagtaaggacatttctgtctgcttcacaatgtgtatgttcttccaaaagc

tcactgatcttgacccatttcttctatccgtttgctcctacacccaggctcatccgtttc

tgagcatgtacgacaaccagaatatcgatctttctgactacttcacgaccgcaggatcac

cacttgccaccttcaagtaatggtcctataaccccactgaagagtacttacatggttact

**>TaMKK3-1?partial TRIAE_CS42_3B_TGACv1_228257_AA0826360.1.X (based on AA0826360.1)**

ccccatagagggttgagccagttcccccaaaatcatacgtcttacaagcctgatgcaagg

cctacatgtgagcaacttctgtcacacccattcatcaagcggtacgaggaagccggcgtc

GACTTGGCGGCATACGTCGGGGGTGTTGTGGATCCAACAGAAAGCTTGAAACAAATAGCT

GAGATGCTTGCTGTTCATTATTACCTCCTGTTCAATGGCTCTGATGGGTCCTGGAATCAT

ATGAAGACATTGTACATGGAGGAATCATCTTTCAGCTTCTTAGGGAATGTGTGTGTCGAG

CAAAGTGTCATATTTGATACTCTGTCAACTATAAGAAACAAGTTGAAAGGTGATCAGCCC

GGTGGGGGAATTGTTCATGTTGTTGAGAAGATACATTATGGTGCAAATGGGGAAAGAGAG

ATCACTGTTCGTGTGTTCGGACCATTCATTGTGGGCAACCAATTTCTAGTACTTGGTGAA

GGGCTGCAAGCCGAAGGGATGCCCAGCCTGGACGAGCTCACGGTTGACATTCCAAGAATT

CGG

gtaggtcagttccgggagcagttcatcatgcagccagggatttccatggaatgctattac

atatcaagacaagatctctacatcatccagtcctagtcctcacaacatttgtaatctcca

agaacttttgggcctagttcagccttcatatcacgattgccatatttgcattactttttt

tttgaaatccctattccacaatatttgcatttagcagccttcaccgcatcattttcatcc

ttaatcttgatgaaatggttccacatctctacatcagcaagttttgacaagatttgtaat

ttgtaagatttcttgggcctagctagtttagcttaaacttagctgctcacaaaaacaaca

cagagaattgtcttcaagaaattacaatcaacttttttttcgcttctattgcctgagctt

attatttactctctctgttgaaaggtctgtaatgtgcatggactaaaatgtatcgtggct

aatggacattggcctattttgtaaaaaaaaaaatttgcaaaaaatatgagcacaagaacg

tttttcaaacaccattcaaatgttaaatgttacgacaaaaatatgggccattgttacttg

agaacaaatttcaaataaaagaacggatgtgctgcacatacgatgattttctgagatatt

tgtcgattgaatttcatcgagttagttgcgcagaaccaccacaagtagatactatactac

cgtgctcggaaataaaatcatcggtattactctaagtagagcaggctcgaaattgttcat

gttgttgagaagatacattgtggtgcaaatgggaaaacagaaatcactattcgtgtgtcc

ggatcattaattgtgggcaattaatttctaatacttggtgaagggttgcaagctgaaggg

atgcccggccctgacgagctcacgattgacattccaagaattcgggtaggccagttccag

gagcagttcatcatgcagctaggggtttccatgaaatgctacaacatatcaagacaagat

ctctacatcagccagtctcgacaagatttgtaatttgtaagatttcttgagatagtttag

cttaaacttagctgctcacaaaaacaacactgaaaattagtatccgagaagttacaatca

attttttttggcttcggtcgcctgatcttattttttactctgtctgctgaaaagtatata

atgcccatggctaaaatgcatcctaactaactgacgctgacccattttgaatgtttttgc

tgaaaataccattaaaatgggtcattgtcacaaatttcaaatcaaaggaacgtttttcaa

acaccattgaaatgttaaatgttacgaaaaacatatgggtcattgttacaaatttcaaat

taaaagaacggatgtaatgcacatgcgatgattttatgagacattcgtcaactgaattta

atcgagttaattgcacagaaccatcacaattgtggcatcatttgtagaaaagaaaagaca

gttttccattttttttgatttgcaatttgcagcaagtgcattttgtgcggtattggtgtg

tgccgtggtggcagcagtctactcctcaaaacgtatgcacattggcacaacgtccatagc

tcgttcaaaaatgttttaaaaaatcaaataaaaatgtagcacattggcacaacgtcggtc

tatgttgtcgcaaaaattcaaatcaaaattcaaaacattgcatgagatataaaaacaaca

aaatttggcatcaatgtgttaacgggccaaatctaaagcccaacttatgttgtgtgctat

tcaatgccaattnnnnnnnnnnnnnnnnnnnnnnnnnnnnnnnnnnnnaatctaaagccc

aacttatgttgtgtgctattcaatgccaattttttgatttttgtatctcgagcaatgttt

caaattttgagttaaaattttacgacaacatgaaccaacgttgtgtcaatgtgctatttt

tttcagatttttaaaacattttttaacgggctatgggcgtctggtgcaccggtaacacca

caagcactggtgtgtattggtgtgtgccgtggtggcagcagtctactcctcaaaacgtat

gctcttcgactttgtatgcatttccatggatctccttggcacggccaccttcactaaaat

tacaaaagcaacaacacccaaagatgcacaaaataaataattaaaaacaactaacagaaa

aaacctaagcgggaaatgactggagagagcgcacctatgattcgggaaagtatctggtgc

accggtgcttatggtgctaccggtgcaccggacgcccatagctcgttcaaaaatgtttta

aaaaatcaaataaaaatgtagcacattggcacaacgtcggtctatgttgtcgcaaaaatt

caaatcaaaattcaaaacattgcatgagatataaaaacaacaaannnnnnnnnnnnnnnn

nnnnnnnnnnnnnnnnnnnnnnnnnnnnnnnnnnnnnnnnnnnnnnnnnnnnnnnnnnnn

nnnnnnnnnnnnnnnnnnnnnnnnnnnnnnnnnnnnnnnnnnnnnnnnnnnnnnnnnnnn

nnnnnnnnnnnnnnnnnnnnnnnnnnnnnnnnnnnnnnnnnttcagattttttaaacatt

tttaacgcgctatggacgtctggtgcaccggtaacaccacaagcactggtgcacgagata

ctttccccctatgattctatctatctgatggtgaaacgagaagtggaggtccagtccaga

agtggccttgtacaatcaatcaagaatggcaacagagcagggacctacgtgtaacaaact

aagactagtcggccacagtgcacgcacaaggcaaagagatcatcgcccaaagaaaggacc

tatttgtaagctccaaaataaacgagggcacgaatgaaaggccccggggaacttatctgt

aaggaactcagggaccggcgcgcgaaatctcaaaaaaatttggaccatttctcgatttgt

gcgtgtcattcttgcgcaggggccatgctaatcttctctgtatcgttccaattttatcgg

atgtccccgaagggacaagtctgctgccgcgctcaggcttataaaccggtgtgtctcccc

tacgctggccgaggtgggactaaacatcagcgagtttcgttcctccgagcgcccggtgcc

cccacccagcctcgtgtcctcgcgtgggtgggctgcatcccctcgcgcggcccagctcgg

ccgacggcccagcccagccggccaggctggcggaggacgagaccatcattatcag

CGGCAGCGGCGGCCGCTTTCCTCCACACACACACACACACCATTATTTTCCCCTCCCTCC

TCCTGACCGCCCACCCACCTACCAGCCACCGAGGCGGCCGATCCCCCCTCCCCAGCCGCG

CCGCGCCACCGCCACCGCCGCCGCCGGCAGCCGTTCGTCCCGAG

gtacggacgagctttcccttcccttcccttcccttcctttcccccagcgatcctcccagc

caaatccctcccgctaggggacctcgcacgccgctctagatctagatccgccccgtcgcg

tccgcaggtgttcgatccctccccctcgctcgctcgcccgcagcgaatccggcgaggcga

ggcccaagtagatcgatccctccgccccgccatgaagggcccttccggttcctcgggcgg

ccgcgatccgtgggcgcctcctcccggttctggtggtggcggcggcggcggatccagcga

gccggccaagaagcagcagcggaccgccgcgagctcgagccaggccgacgaggcgtccac

ctcctcctcctcctcctcacagcctccgccacagcagccgccgcctgcgccggatggggg

agatgtgccgtatctgggggaggacctgatgttcgaggtgctgcggcgggcggaggcgcg

gacgctggcctcggcggcgtgcgtgagccggggctggcgggccctcgcgcaggacgagcg

gctctgggaggccgcctgcgtgcgcgagtgggccgacctcggcttctccgagcagcagct

ccgcgccgtcgtgctctcgctgggcggcttccgacgcttgcacgccgtctccatccggcc

catccagcggcgccgtgctggtgttcctgctgcgcctgcacagggaaggggaaggcagca

gcctcctcccaggctgggccgggaccaggtccagctgtcgctgtcgctgttctcgatcgg

gttctttcagagcatgcctaatcagccccctcctaagaagaaagatgagggtgatggcag

tgataagggtggaggtgggcggtgcgggtgatccagctggtttgcctaacagctagtctg

gacaagagagagcataggaagtcgggctgattgataagaggcatgacggagagggagtgg

gactgcaaggtggtcggattatgctgttctaatatggacaggtgtatgggttctgatagg

tcataaattcacctggaaatgtggataggtgtatgggtactgatgggttggtgggagttt

agttgaagtgtcacagggcagatggggtgccatacctatggcacatggaatcactttggg

gagaaatggccccaaatctatgagctctggattctggttgcttgattttgatttgacagt

acatagtttgttgcttaagttggtttgaattattgtgtaaaattctcgccttaaaggttt

gagcaatgtgaaatcgtgtgattgaagtgtcaagctaatttacttcagtaacaatggcta

gatttaagcttccatgtggttgttgtaataatgaaatggtatctgacatttagctaactg

aaatttctcgatactccag

ATTGACCTTTGGTGACTGTTAGAA

**>TaMKK3-2(5BL) TRIAE_CS42_5BL_TGACv1_405566_AA1330480.6.X (based on AA1330480.6)**

GCGAAGAATTTTATTTCTTGAAAAGATGAATAGTTACTATGAAACATATCTTTTTAACAA

ATGGTGAGGCCTTTTTTTCTACTAGAGTTGTGATGTCTTTGAATTTGAAAAGAAAAGAGG

AAGCAATGGTCGTTTTATGATACTCCTACTATAGTAAAAATGAAAGGTGAAAAAGAAAAA

CTAAGGTATGCGAGAAGCGGCATATAGACAGAGGAGGGAGCAGAGTGGCATCCTCCTTCC

TCTCTTCCGCCCCCGTCGCCGGGCCGCAGGCAGCTCACCGCCGCCGCCTCCTCCCCCCGC

CCACCGCCTCCTCACCGTCCGTCCCAACCCCTTCGCGCCCCTTTGTATCCTCCGCTATCC

GCCCGTACAGATCCGTCCGGCGAGCATCCTCAGCCGCCCCCGACGGGCTCGCCGCCGCCA

GGACCTCACCGCTGCCCCCCTCACCGCGAAGAGAACCGAG

gtgatcccgctcccccaaatcccgattcccatttcgtcagtttccagttcgatttggaat

cggtgcgggtcagagaattccagtgtatgttccattgatctgttggggactgccggatgt

aatcag

GTCCGCCCAAGCTCCAATTTTTCGCCTGGGGAACAGACCGCCCGCCGCCTTCTCTGCTCC

ACTGAAGATACGGTGATTCCTTCCTGCTTG

gtaagccccaattttgttttattttattttgctgggctcattatcattcactggcttgtg

ctacatgcagccagattttgtctgctgttgttgatgcaaactgttagcccattcattatt

agacttctctgacagcctcctgtgaaccacgcag

GACGACTGGGATCATCAGCTTGCGCCGGCTGACTACAAGCGAGCCCCCATGGCGGGGCTA

GAGGAGTTAAAGAAGAAGCTGCAGCCCTTGCTGTTCGACGACTCGGACAAGGGCGGCGTC

AGCACCCGGGTTCCCTTCCCGGAGGATACATGCGATTCCTATGTG

gtaaattctttcttctctgcatgccagccaaattaggttgttggtgtcaatcactagact

ggggttgttgtctgttaatagaattggtacaagctgtttgagttctgttttccagtcatg

aaaaccatcctgctgtcagcaagtgaatgtacatgctcaagggttctgccgcttcagtgt

agcgatttttctgtatgggcctagtcatctgcccaacttgaccaatcgatcctcacacat

gaagtgatatttataaagaatattttttggtcatgagattaccctttggaagtttttttt

ttcatttagaattccctgagcgctgtttttaatctttgtgactttaaacttgttagatgc

ggaagtgtttgttttttatgttctttctttcgtgaag

GTATCTGATGGTGGAACGATAAATTTACTGAGTAGATCGTTTGGTGAGTATAACATCAAT

GAGCATGGCTTTCACAAGCGAAGTACTGGGCCAGAAGAGCCCGATACCGGTGAGAAGGCA

TACCGATGTGCATCTGAAGACATGCATATATTTGGTCCCATTGGAAGCGGAGCAAGCAGC

GTTGTTCAGAGAGCTATTTTCATACCAGTTCATCGTATTCTGGCCTTGAAGAAGATAAAC

ATATTTGAGAAG

gtacattcttatggcccgaaatgcattttactaccacattgttgtgcacttctgcttttt

tagttataagggtgttttcgttttttgttgataactagtctaaacctcattcgtgttttc

aaacaaaatagtattaaagggagcaaaacaaacgcttattcatctgtatgtggtttattg

catgctggaacatccttccctttcttttggagtcatgtgcatcacaatgggaccattaaa

tgcagcacattcccctgaagaattttggttcctagcgataatctgtcaatttctccattg

cctattcacaatgtttcctatttagaagggaacaacatctcaatggtagggtgttggata

tgtaaaagcgaagcagtgtgagactgtcactatatacattgtttcgttgggattacaaat

aatattcgggcagtaacttctcatcacactcagcctaggcagttgcacttaacttgaaga

aatgtactgtcaggacatgctcgactaatggaaagatcacactaaatcctcatccttagc

tacatgcattgtttcctgacgcatctgcaatagtgatttcagaatggggccaggacctgc

acaaggaagggtggcaaaaaacacactggacaggggatttcggttgctaggcatttgttg

gctatccccccctcccgcacacacacatttgtagtttcaagctgcctatttactgatttc

ccatccacagtgcttctagttttatatttccatccactagagatggtatgcctcatttca

tgatatttgattgagcaaagaaagagaacaacaagttcatctcatctctagtctcctctg

acaccaaagcaagctctctccaccaaaccatgccttgacacattggccccataatttggt

attccctccgtaaaggaattagcatgcatcacaatatgacatgttcattttccttttaac

actcccaatctcaaggcgggcaaatatcccactacgcaattttctgtatgtaagctggag

tgaaacaagttggagttgagtatattaaaccaaaacatgtatttgcattgcattgaaggc

agcacaaagggattctggtttatttgcagtggcagtcaagtagcagagtctgttctgact

gcagcatgaaagcagttacaccaaagaatataaatgctcactcttctatgcttcagttca

tatactattgtggcgcaatatgtttggtattgcacgaagtgattttggtactttatgttt

gtctatatattttgctattgcagttgataatctgatttacaaaattttattttgatattt

ttcacgtgtcttcataactttacag

GAGAAAAGGCAACAAATTCTTAATGAGATGAGAACGTTATGTGAAGCAAGTTGCTATCCT

GGTTTAGTTGAATTCCAGGGTGCATTTTACATGCCCGATTCTGGACAAATAAGCATTGCC

CTTGAATACATGGATGGTGGCTCTTTAGCAGACGTTATAAGAGTCAAGAAATCAATACCA

GAGCCAGTTCTTGCGCATATGCTACTGAAAGTATTACTT

gtatggatcttccacacatgctcttcctttcatgtccgctttaatcaacattaagctgtg

tcctgatgtatttttgaagttataatttattctctgtacaattctgaag

GGCCTGAAGTACTTGCATGAAGTAAGACATCTAGTGCATAGAGATCTAAAGCCAGCGAAT

ATACTGGTAAACCTCAAGGGAGAGGCAAAAATTACAGACTTTGGTGTAAGTGCTGGTTTG

GACAATACAATGGCTATG

gtatgctacttcttggtgacttttgatattggttaatctgagctaattattgtataatag

tatagcaatgccatcaaatcatttagcatgaatgcgctacatattttctgctaaataaat

atgtttcag

TGTGCTACCTTTGTAGGAACCGTGACATATATGTCACCTGAGAGAATTCGTAATGAGAAC

TACTCTTATGCTGCTGATATTTGGAGTCTTGGACTAACGATATTGGAGTGTGCTACTGGT

AAATTTCCATATAATGTCAATGAAGGCCCAGCCAATCTCATGCTGCAG

gtgattggctactcagattttttggttcttgtctgttttcattcataatgtatgttggga

tgcaatatcatcccttttagtctgatgcaaaatgcgtggactatttgggctgttcacatg

gtaggttgtatttggacttgttgagatggcaaaaaatgttacatttctgcaagtctgtcc

tctggctatgttagtttccacaatctttttccttttcctggctgctttaacttttgtaag

ttgtatttgtatttgattatataaattatctgacaatgtaataaatatgtaccgttacat

atattggtcttatgaatgttctcatcatcatttgtctgttggaatgttgttttctgtcac

ttcataggttgccctatcttaatcttaaaatctcatacttcagatactgttttctttcac

tcaattgctgttctctcttaatcataaactctcttatttcag

ATACTCGATGATCCATCACCAGCACCACCAGAAGATGCCTATACACCAGAATTTTGTTCC

TTCATAAATGATTGCTTGCGGAAAGATGCTGATGCAAGGCCTACATGCGAGCAG

gtaaaggaaacatgtatatgctaactttcatgtaatctgagtccagtaggttagcaatct

gcatcaatgtattgcggtctatttgaatgttattacatattactgataataatatttctt

gataaagtattggtggcagtaactgtcagagtactgtgtagctgtgtggttaagtacagt

tgcttttttaactgatttgccttctcttgtaacgatgtcccactggcctttggttgatga

ggagttgaggacatcggtgcccctatcattggtccattggaatgaatggcgaccttggtt

tctcttctcttactcgcaccatcatgtccatacttccataccctagcagcccagagttgc

gggcgctgctacagtcaggccaccaccgccaccaatgttcgctgtccaccataggcctct

tatactcgggcccagaatgactctgcaccctctcctctgctccagatgtgcctctgctgt

agtacctgccgtgccactgtccttgctgctgctgcttctggtggccatcaatgacctcaa

gctcagatctnnnnnnnnnnnnnnnnnnnnnnnnnnnnnnnnnnnnnnnnnnnnnnnnnn

nnnnnnnnnnnnnnnnnnnnnnnnnnnnnnnnnnnnnnnnnnnnnnnnnnnnnnnnnnnn

nnnnnnnnnnnnnnnnnnnnnnnnnnnnnnnnnnnnnnnnnnnnnnnnnnnnnnnnnnnn

nnnnnnnnnnnnnnnnnnnnnnnnnnnnnnnnnnnnnnnnnnnnnnnnnnnnnnnnnnnn

nnnnnnnnnnnnnnnnnnnnnnnnnnnnnnnnnnnnnnnnnnnnnnnnnnnnnnnnnnnn

nnnnnnnnnnnnnnnnnnnnnnnnnnnnnnnnnnnnnnnnnnnnnnnnnnnnnnnnnnnn

nnnngggggggggggggtatgtgcagctagggatggaaaccttaggcacagtgagaagga

aatgaggtgatggaccaagtagaagatgggttactccctgataattgatataacgtaacg

tgtgcctatctcattataatgggggtctgccttacatgtgctggactctacaacatgaac

tggacttggcaacatgtctgttataataggagcactacttgaattctgatcaccagtttc

tctgtgcctactgtggtttccttgcctccttaatgatcacatgtccgttgtggtaggatt

gcagtttccctttcaattgcttaggctgagttgtgagtcatgtcatccttgatccagtga

agcacattaggcaatgcatcggctgttgtagatccagtgaagcatgatctctccaagcac

attggagttgatgcttcctgcatgaaattgcagatgcaagagcaggttgttgctctccag

tatgtgccatcagaactctagttagctgatttcttcgccaaggcacattctagagctttg

ttgttgatctgcctttaattttgtgtagggtgttattggaggtccatttcacaaacttgt

ataagcaaaatgtacgtactgattagttatggcttggtctagtaacagaatgttaattat

ctcgacgatgcattttacatcttctatccttttccattgtttggttttcttatagttgat

taacatgtttaatgtaatgcag

CTTTTGTCACACCCATTCATCAAGAGGTATGAGCAAACTGGTGTGGACTTGGCAGCATAT

GTCAGGGGTGTTGTTAACCCAACAGAAAGATTAAAGCAAATAGCAGAG

gtgaattctgttgagcatatttcagcttactgttctttctacttcagttaactataaggg

cgcacacgtgatacgaaacatgccccctatatcaagaactgatgctagaccttagaagtg

gggctgggagggagagctggggtagaggctagatggaggaggtatagttaagcactcgct

tgatggagttactgccagcttgccttctttggcctctgccgcctaaccgctggtggtcac

cactcactaggggcgccattcctatttgttctgtccgcctttccttgggtacaaaacaga

gacagtttatttttttgtctttcagttttagtttagttcattgtttcttttcccgatata

taatatagctagtatacaataggtcgtcgacctggagtctggggctgcgttgttgtgcac

tatggttccaaagaacactaccccatcccctatcaaccctacttgttgggccaccttaac

cgtgtctggctgatcggttttgctgatagctatgattgtacattagaaaataggaagaac

aaagtggcacaagtgtgatccatttgaacgaatgcactgcagtattcctatacttccgtt

gaacgagtaaaatgttacaccagtgctagtcatgtatagaatactggcctgataggtcag

ttcagattattctaaaacttgataatttctttgtttgctgcccagagaaaccctttggtt

ttctcttggagcggagtgcgtcaaaaaataaatactaatatataagtcgagagtgttcga

gcccctctatccccatactctctttaactttcctatttatcctctctttccatttccact

cgtctgttgattcttcaacattagctggccttttcccacaattcagatctcaattcaaac

tcaagaacgaatcatatgtatcttcctagattgatagaactcgcctattagacccaaacg

catccaacggatacttactatttgatttattccttttcttaacactcctcacgatctcta

acataacttatccaaggccttcacggatatgctcgtctcctgttgtcttgtggacccaca

cacatgataataatatccaagcttttgaagtgaaaattaatcaattagctcccttttacg

ttttagaaaacctcattaaaatgtgatgaaaaaactgatgatcctgtgtgtgaagtactg

agctgatttcccccccccccccactcaatcccag

ATGCTTGCTGTTCATTATTACCTCCTGTTTAATGGCTCTGAAGGACCTTGGAATCATATG

AAGACATTCTACAGGGAAGAATCATCTTTCAG

gtacctgaggcagtgttacatgttttaatattaccttaaaacttagttgtaccacagcag

atagcatcttattgctttttaggcgtttaacctttacgtaaattgtggccccagtaactg

gttacggtattcttagaaacagtttggtactaataatatggaaagtgtgttgtgtgatca

tcaatatgaaaacttgctgaaactagcatggagttgccaacaggttgatgtatctagctg

aattaatagttgcactttatcctgtaggcagtgttgtgcttctcttgttctacttctacc

aggtagtaaccggctggttactgccatctttccaatctgtttctgagcatatgcatgata

tgtggttcctgtttttgcag

TTTTTCAGGGAATGTGTATGTCGGACAAAGTGCCATATTTGATACTTTATCAAATATAAG

AAAGAAGTTAAAAGGTGATCGGCCTAGAGAAAAAATTGTTCATGTTGTTGAGAAGCTACA

CTGCCGTGCGAATGGGGAAACAGAAATCGCTATTCGTGTGTCTGGGTCATTCATCACGGG

CAACCAATTCCTAATATTTGGTGAAGGGTTGCAAGCTGAAGGGATGCCCAGCTTGGACGG

AATTGATATCGACATTCCAAGCAAGCGCGTAGGCCAGTTCCGGGAGCAGTTCACCGTGCA

TCCAGGGACTTCCATGGGGTGCTACTACATAGCAAAGCAAGACCTCTACATCGTTCAGTC

CTGAAAACAATGTATAAACTGTAAGGTGAGCCTTCTAAATCTCATGTGTACTCTTTGATG

GATCGCTCTTAATGCCGTGTGGATCATCTGTTTGCTTAT

**>TaMKK3-2(5DL) TRIAE_CS42_5DL_TGACv1_432926_AA1394740.1**

GCCACAAGCGGCATATAGACAGAGGACGGAGCAGAGTGGCATCCTCCTTCCTCTCTTCCT

CCCCCGTCGCCGGGCTCACCACCGCCGCCGCCTCCTCCCCCCGCCCGTCGCCTCCTCACC

GTCGGCCCCAGCCACGCCGCGCCCCTCCGCATCCTCTGCTCTCCGCCCGTACAGATCCGT

CCGGCGAGCATCCTCGGCCCGCTCCCGACGGGCTCGCCGCCGCCAGGACCTCACCGCCCG

CCCTCCTCACTACGAAGAGAACCGAG

gtgatcccgctcccccaaagcctgatccccatttcatcagttgcctagttcgatttagaa

tcggtgcgggttagagaattccagtgtatgcatgctctattgatctgttggcgcttgttg

gatgtaatcag

GTCCGCCGCCCAAGCTCCAATTTTTCGTCCGGGGAACCGAACCCAACCGTCCGCCGCCTT

CTCTGCTCAACTGAAGAGACCGTGATTCCTCGCTGCTTG

gtaagcccaattttatttttttatttatttttgggcggccagattttgtctgctgttgtt

gatgctaactgttaacccatccaccattag

GGTTCTCTGACAGCCTCCTGTGAACCAACGCAGGACGACGTACATTTGCGCCCGTCTGGT

CACCAGCGAGCCCCCATGTCGGGGCTAGAGGAGTTAAAGAAGAAGCTGCAGCCCTTGCTG

TTCGACGACTCGGACAAGGGCGGCGTCAGCACCCGAGTTCCCTTCCCGGAGGACACATGC

GATTCCTATGTG

gtaaaacacaccctgtcattctttcttctctgcatgcaaaccaaatttggttgtctgtgt

caaacaccagcggcattacacccgggttgttgtctgtaaatggaattggtaccagttgtt

tcacttctgctttccagtcatgaaaaccatcctgccagcaactgaatatacatgctcaat

ggttctgtacctttggggtagcactttcgctgtatgggcctagacatctgcacaacttga

ccaatcaatcctggcatatggaagtgataggacaaagattattgtttgtgatcatgagat

tactctttgaaagtttccaattagaatttatctgagctttgtttttaatctttgtgactt

aaacttgtttagctgtggaagtgtttgttttttatgttctttctttcatgaag

GTGTCTGATGGTGGAACAATAAATTTACTGAGTAGATCGTTTGGTGAGTATAACATCAAT

GAGCATGGCTTTCATAAGCGAAGTACTGGGCCAGAAGAGCCAGATACCGGTGAGAAGGCA

TACCGATGTGCATCTGAAGACATGCATATATTTGGTCCCATTGGAAACGGAGCAAGCAGC

GTTGTTCAGAGAGCTATTTTCATACCAGTTCATCGAATTCTGGCCTTGAAGAAGATAAAC

ATATTTGAGAAG

gtacattctttacggcccgaaatgcagtttacaaccacattgttgtgcattgctagtttc

catgaacatctcgacaagggtgttttcgcttttaatttcctaataattgctcattctgct

tcattggactgaacttataaagaagatcctgaaaacttgacttaatatttgtacccacat

tcaagtggaacttgtgttgatatctagtcgaattctcattcgtgttttgaaacaaaatag

tattaaagggagcaaaacaaacccttgttcttctatgcgcggttcacttgtatgctggag

catccttccctttcttttggggtcatgtgcatcacaatgggcccatgatatgcagcacat

gggtgggtaacatcaatcacattcccctgaaggatttggttactagtgataagctgtcaa

tatctctattgcctattcacaatgtttcttatttagaagggaagaacatgtgttggatat

gtaaagtgaagcaatgtgaaactgttactatgtatacattgtttcgttgggattacaaat

agtactattcggacagtaacttcttatcagacccagtctaggtagttgcaccgaacttgc

agaaacgtactcttactcatctaaacttgcctagctagttaggacatgcccgactaatgg

aaagacgacattaactcctcatccttagctacctgcactgtttcctgacacatctgcaat

agtgatttccgaatgggcccatgacctgcacaaggaagggtggcaaaaaatacccaggac

ggtggatttcggtttttagaatttcaagccacctatttaccgatttctcatccacagcgc

tgtctagttttatatttccatctactagagatggtatggctcatttcatgatatttgatt

aagcaaagaatgagaacaacaagtttatctcatctctagtctcctctgacaccaaagcaa

gctctctccaccatgccatgctctcgacacattggccccataatttagtagcatgcatca

caatatgacatgttcatttccttttaaccactctcaaggcgaccaaatatcccactacgc

aattttctgtatgtaagctggagtgaaacaagttctagttgagtatattaaaccaaaaca

tgtatttgcactgcattgaaggcagcacaaagggattctggtttatttgcagtggcagtc

aagtagcagagtcagttctgactgcagaatgaaagtagttacaccaaagaatagaaatgc

tcactcttctatgcttcaattcatatactattgcggcacaacatgcttggcattgcgcga

agtgattttggtactttatgtttgtctatatattttgctatacttaatccattctgtttc

agttgataaactgatttacaaaattttcttttcatattttcacgtgtcttcataccttta

cag

GAGAAAAGGCAACAAATTCTTAATGAGATGAGAACGTTATGTGAAGCAAGTTGCTATCCT

GGTTTAGTTGAATTCCAGGGTGCATTTTACATGCCCGATTCTGGACAAATAAGCATTGCC

CTTGAATACATGGATGGTGGCTCTTTAGCAGACGTTATAAGAGTCAAGAAATCAATACCA

GAGCCAGTTCTTGCGCATATGCTACTGAAAGTATTACTT

gtatggatcttccacacacgctcttcctttcatgtccgcgttaatcaacattaagctgtg

tcctgatgtatttttgaagttataatttattctttgtacaattctgaag

GGCCTGAAGTACTTGCATGAAGTAAGACATCTAGTGCATAGAGATCTAAAGCCAGCGAAT

ATACTGGTAAACCTCAAGGGAGAGGCAAAAATTACAGACTTTGGTGTAAGTGCTGGTTTG

GACAATACAATGGCTATG

gtatgctacttggtgacttttgatattggttaatctgagctaattattgtataatagtat

tgcaatgccttcaaatcatttagcgtgaatgtgctacatattttctgctaaataaatatg

tttcag

TGTGCTACCTTTGTAGGAACCGTGACATATATGTCACCTGAGAGAATTCGTAATGAGAAC

TACTCTTATGCTGCTGATATTTGGAGTCTTGGACTAACGATATTGGAGTGTGCTACTGGT

AAATTTCCATATAATGTCAATGAAGGCCCAGCCAATCTCATGCTGCAG

gtgattggctactcagatttttcagttcttgtctgttttcagtcattatgtatggtggga

tgcaatatcatcccttttagtctgatgcaaaatgcgtgaactatttgagcgattcacatg

gtagtttgtatttggacttgttgagatggcaaaaaatgttatatttctgcaagtctgtcc

gctggttatgctagtttctgcaacctttttctttttcctggctgctttaacttttgtcag

ttgtatttttatttgaatatataaattagctcaatgaaataaatatgtaccgttacatat

attggtcttatgaatgttctcatcatcatttgtctgttggaatgttgttttctgtcactt

catatgttgccctatcttaatcttaaaatctcatacttcagatactgttttctttcactc

aattgctgttctctcttaatcataaactctcttatttcag

ATACTCGATGATCCATCACCAGCACCACCAGAAGATGCCTATACACCAGAATTTTGTTCC

TTCATAAATGATTGCTTGCGGAAATATGCTGATGCAAGGCCTACATGCGAGCAG

gtaaagaaaacatgtatatgctaactttcatgtaatctgagtccagtaggttagcaattt

gcatcaatctattgtggtctatttgaatgttattacatattactggtaataacttttctt

gataaagcattggtgtcagtaattgtcaagtactgtgtacttgtctggttaagtatatgt

tttttttaactgatttgccttctcttgtaattatgtcccactggcctttggttgatgagg

agttgaggacatcggtgcccctatcattggtccattggaatgaatggagaccttggtttc

tcttctcttactcgcaccatcatgtccatacttccataccctagcagcccagagtcgcag

gcgctgctacagccaggccaccaccgccaccaatgttcactgtccaccatagacctctta

tactcggcaacgtgcccagaatgactctgcgccctctcctctgctccagatgtgcctctg

ctgtagtgcctgccatgccactgtccttgcagccgtcatgccactgtccttgctgctgct

gcttctggtggccatcaatgacctcaagctcagatctggtcacttttggtgcttgggcac

cgtggagatatgttcagctagggatgggaaaccttaggcacagggagaaggaaatgaggt

gatggagcaagtagaagatgggttactccctgataattgatttaacgtgtggctatctca

ttataatgagggccttacatgtgctggactctacaacatgaactggacttggcaacatgt

ctgttataataggaacactacttgaattctgatcaccagtttctctgtgcctactgttgt

ttccttgcttccttaatgatcacatgtccgttgtagtaggattgcagtttcccttttgta

tgcttaggctgagttgtgagtcctgtcatccttgatccagtgaagcacattaggcaatgc

atcggctgttgtagatccagtgaagcatgatctttccaagcacattggagttgatgcttc

ctgcatgaaattgcagatgcaagagcaggttgttgctctccagtatgtgccatcagaact

ctagttagctgatttcttcaccaaggcacattctagagctttgtttgttgatctgccttt

aattttgtgtagggtgttattggaggtccatttcacaaacttttataagcaaaatgtacg

tgctgattagttatggcttggtctagtaacagaatgttatttatcttgacgatgcatttt

acatcttctatccttttccattgtttggttttcttatagttgattaacatgtttaatgta

atgcag

CTTTTGTCACACCCATTCATCAAGAGGTATGAGCAAACTGGTGTGGACTTGGCAGCATAT

GTCAGGGGTGTTGTTAACCCAACAGAAAGATTGAAGCAAATAGCGGAG

gtgaattctgtagagcatatttcagcttactgttctttctacttcagttaactataaggg

cgcacacatgatatgaaacatgcgccctatatcaagaactgatgctagaccttagaagtg

gggctgggagggagagctggggtagaggctagatggaggaggtatagttaagcactcgct

tgatggagttactgccagcttgccttctttggcctctgccgcctcaccgctggtggtcac

cactcaccaggggtgccattcctatttgttctgtccgcctttgcttgggtacaaaacaga

ggtagggagagagacatatttttttgtctttcaattttagtttagttcattgtttctttt

cccaatctataatatagctagtatacagtaggtcatcgacctggagtctggggctgcgtt

gttgtgcgctatggttccaaagaacactaccccatcctctatgaaccctacttgttgagc

caccttaaccgtgtctggctgatcgattttgttgataactatgattgtacattagaaaat

aggaagaacaaagtggcacaagtgtgatccatttgaacgaatgcactgcaggattcctat

acttccattgaacgagtaaaatgttacactagtgctagtcctgtatagaatactggcctg

ataggtcagttcagattattccaaaacttgataatttctttgtttgctgctcagagaaac

cctttggttttctttttgagcggaatccgtcaaaaaataaatactaatatataagtcgag

agtgttcaagcccctctatccccatactctctttgactttactatttatcctctcctttt

ccgtttccactcgtctgttgattcttcaacattagctggcccggttccacaattcagatc

tcaattcaaactcaagaacgaatcatatgtatcttcctagattgatagaactcctgctat

tagacccaaacgcatccaacggatacttactatttgatttattccttttcttaagactcc

tcaccatctctaacataacttatccaaggccttcacggatatgcttgtctcctgttgtct

tgtggacccacacacatgataataatatccaaacttttgaagtgaaaactaatcaattag

ctccttttacgttttagaaaatgtcattaaaatgtgaagaaaaaactgacgatcctgtgt

gtgtagtactaagcaggttctttttcccactcatcccag

ATGCTTGCTGTTCATTATTACCTCCTGTTTAATGGCTCTGAAGGACCTTGGGATCATATG

AAGACATTCTACAGGGAAGAATCATCTTTCAG

gtacctgaggcaacgttacatgttttaacatgatcataaaactttagtcgtaccacagca

catagcatcctactgctttttaggcatttaacctttatgtaaattatgactccagtaact

ggttacagtattcttaaaattagtttggtactaataatatcaagaatgtgttgtgtgatc

ctcaatatgaaaatttgctaaaactagcatggagttgccaacaagttgatgtatctagtt

gaattgacagttgcactttatcctataggcagtgttgtgcttctcttgttctacttctac

caggtagtaactagctggttactgccatttttccaaatttatcctgtcaagcattgttgc

ttctgttgtgcctctatactatagttgtgtgtggaagaaaccgagctgtttctgaacaca

tgcatgatatgtggttcctgtttttgcag

TTTTTCAGGGAATGTGTATGTCGGACAAAGTGCCATATTTGATACTTTATCAAATATAAG

GAAGAAGTTAAAAGGTGATCGGCCTAGAGAAAAAATTGTTCATGTTGTTGAGAAGCTGCA

TTGTCGTGCGAATGGGGAAACAGAAATCGCTATCCGTGTGTCTGGATCATTCATCACGGG

CAACCAATTCCTAATATCTGGTGAAGGGTTGCAAGCTGAAGGGATGCCCAGCCTGGACGA

AATCAATATCGACATTCCAAGCAAGCGGGTAGGCCAGTTCCGGGAGCAGTTTACCGTGCT

TCCAGGGACTTCCATGGGATGCTACTACATAGCAAAGCAAGACCTCTACATCGTCCAGTC

CTGAAAACAATGTATAAAGTGTAAG

gtaagcctcacaaatctcatgtatactgtttgacaacaaaatcttacgtgtgtttctggt

cgagctcaacgtctgatactctttatcttccatttgaaacagatctaatttttgttgtca

accaagctttcagtttgatggattgctcttattgccatcgatcatttgtttgcttatgag

attgcttggtttgcag

TTATGTTCCGTTTGTGCCGTCAATGTCCATGATGAAAGCTAAGTCATGGTTTCCACGCCT

CGCGACCCCATAACTTCGACATGCTTCAGATCACAATGCAGTCCTGCTACCATTGACATT

GATTCCATTTCTTCTCGATGCGATCAATCAAACTTGTAGCTGAAACATAACTTGATTGTT

GTTTGTAAGGTGGTAAGGAAAATGAAGCGATTCTAATGATTCATGTGTGTAGATAGATAC

TCTTCACACATGGTGCAGGTTTAGTCGAGGCTGCTCAATTTCTGTTGTAGATGAGAAGAT

TGCACGTGTGTGCGCTAATAAGTGACGCCTCTCAATGTAGGTTTGTATTACTAATGCTTA

TGTATAAAAGACTATCTATAATTAACTCCGCTGGTACTGTATCATTATGTTTGACATTTT

TCTTTTTTCTTGAACTGGTACTAAGATGTCTGAGAAGTTCAAATTTGACGTACTTTATTT

CGAGGGAGTAATCTTGTCTATAGCAAGAGCTGGCAACCGATTTTTGCTTTCTGTGTTGTT

TATTTATTTAAAATTAAGTAAATTCCAATAGTGAAG

**>TaMKK3-3(5AL) TRIAE_CS42_5AL_TGACv1_373997_AA1187200.3.X (based on AA1187200.3)**

ACGACCCAAATCCCTATCAAACAGACAGAATCCGATTACGCGGTGGAGTTGGCCTTACCG

ACCTACTGCGCCGCCTCCGGCCATCTCTCAAATATGGGACTATCGTACTGGCGGCGGAGG

AAAGCATGTGCGTCCCTTCCCGTCGTGGCAAACGGAAGCGAAATCAAAGCCTCACTCTGT

TTTCTCTGCGCCCTCTCCCTGACCCTTCTCCTCTTAGGCTTAGCCTTGTTTGGCTGTAAT

GGCTGGACTGGAGGACCTGAAGCGGAGGCTGCAGCCCATCTTCTTCGACGCCGATGGCAA

TGTTGTGCCGCCCCCCGCCGCCGACGGGACATCCGACGACTCCAGCTCTGACGACTGCGA

G

gtaaccactctcttcgtttcagccgtttggccagttcgctgagatctatgtttatcttca

ttctgttcaaccaaccaaattccttgcttatgttgctcatagtatgttttttcttaagcc

tgctataaaaatatatatgatgctcatatttaatcttacttttgtaaaggttttggatag

tggaactgtcaatttattgagtaggtcctctgatgaatataacatcagtaagcttggctt

ccattaacgaacaaccagaccagatggggactatgctacagataaggcatatcgatgctc

ttgtcatgatatgcatatttttgattctgttggtattggtgcaagcagcgttgtccatag

agctatttatgtaccagtccatcgagttttggcactcaagaaaattagcatttttgacaa

g

gtgagtgcaaaactttttatagtccatatgccataagctgacatctctaggttcccctt

acatgaaacaaaatctatggatacccctttttttctttttcttttaattaactttcacat

ggaactacaagtgtagaattcatggcttattctccatttaatgcctattcatagacattg

tttggaccaagcttattctctttatatttattataacgtattctctgatatatattacag

GAAAAGAGACAACAGATTCTTAATGAGATCACAACGTTATCAGCAGCATCTTGTTATCCA

GGTTTAGTTGAATTCCAAGGAATTTTTTACACTCCGGACTCTGGAGAAATATACTTTGCT

CTTGAGTATATGGATGGTGGTTCATTAGCAGATATTATCAGGGTCAAGAAATTCATAACA

GAACCAGTTCTTTCACATATGCTACAGAAAGTGTTGCTA

gtacgtgtctcttgcctccatatgttctgatgttagctctattcaaatcaggcctgtgaa

atttaagtgactagttagtctctgttaatttattag

GCTCTGCGCTACTTGCATGAAGTGAGGCGTTTAGTTCACAGAGATATAAAGCCAGCAAAT

TTGCTTCTAAATCTAAAGCGTGATACGAAAATTACAGACTTTGGTGTAACTTCTGGACTG

CATGATTCAGTTACCATG

gtatgctacttcttggtgatcgaaacttctcttaggaacagcaagctgttctaaaatagt

attaagtggaaatctgactatctagattgaataagcggtatatatgtgtagtaattgtct

tccag

TGTGCTACCTTCCTGGGCAGTGTCACATATATGTCTCCCGAGAGAATTCGGAACGAAAAT

TACTCATATGCTGCTGATATCTGGAGCCTTGGACTAACAGCATTGGAGTGTGCAACTGGA

AGATACCCATATGATGTAAATGGGGGCGAGGCCGACCTCATGCTGCAG

gtgaggtggttggcggtcaagcagtgttcatttgtataattttctactgcctttcgtgat

catcttatttatggaaaaaaaatcatgatctcatttatgtgcatctgttggtatattgtt

aattaaagctgttttcgaaatttggcttgtttttcgtaggcgttctacatctgtttcgga

atttctactggatttagaacttctatttcatttctgctttccaacatgaatggtctgaca

ataatgcaatactagttcggtagttcctcttgtgagtattatattattgatgtatagtgc

aatcctcctttcccaccttactgtgttgcagtcttgcag

ATATTGGAAGATCCATCGCCAACACCACCACAACATATGCATTCAGAAGAGTTTTGCTCG

TTCATTGATGCTTGCTTGCAGAAAGATGCTGATGCAAGACCAACATGTAATGAG

gtaaagtgacgtctagtacaattacttttttcaagagaaacgatacgagggtttccaact

gcgtaaatcttgtgataatctttatttattatttattttttgtggggagtgataaacttt

attatgcagtaagtataggattttgcgacactatctgagagtaatgtctgatggatgttt

cagctctttagtcaacagattagcggtctaaggtgtgtctgaggtgataattgcaaaaaa

gcggtctcatatgttgaattgttttacaaattattcattgtcaactaactacataaatag

gttatttgcaacaaagacgaaatggggtttattttcctctctgaaagaaaaatcttattt

gaattacatactgaatatcttgtag

CTTTTGTCTCATTCCTTCATCAAGAAATACGAGGGACCTGGTGTGGACTTGGCAGAGTAC

AACAAAAGTGTTCATGATCCAGCGGAAAGATTATCGCAGATAGCACAT

gtgagttcctaatagctttcgttatcttctgtttttcctgttctgctgcctttccatatg

gaggaaaatgaacctgatgttccacaagttcacatacaatgatgttggctatgcttagac

aatagaacattccgacaattcaacaaataaactgactcttactgcagttgtttcaatctt

tttaatctggtttcttgctatccgggattcaaaatattcatgttaaaatttttgacctca

ttgaggatttcggttgccacaagcatagtttaaaaaaccggaccgggccagtggtcggac

cgggaaaaactgaaccggcggcctcggccgttttttaagctaataagaccgttctgcaat

tggaccggagaaaaccagtcaagccggccagttttctgaaaaaaaaacgctaaataaacc

aggcctttgaactggaaaaaaccaggaaaatggttaatgtagaatttgggagaagcggga

gtagtaggcattgcgtgctcatggcccaataaccaactcggatgtgctactttgttgtct

attatagggaagtaattttattcgatcattgtttaacactgttttatttgtccattgtct

actaaatgtatattattttatggtaaaaaaccgacgatgaaccgatgaaccggcggtcca

actgatgaaaacctgaaccgacaggctcacaggttcgatcttcggttcagttttttaaac

tatggccacaagtatatctcagaaaaagaaattggagcagggaaaaacatgatttgtggt

tttgctatccatggagtgggacgaatttacaggaactttgccaccgccaagtcacttacc

atttcgcttgaatatactcggccctaaacgtttctcccctcaaatatgagtgcacccgtc

atgctcacgcggctgctcacctcagctacagccgcttacgccgctgctctcttcacctcc

aaccactcttgaaatagtcttttgccctgctttatagataaagcagccaccatttccaca

caagtagttaaagtgcaggaaagtaaatagaaggtctgctggggcacaaataagcccaaa

agcaaaaaagaaaaggcgacacaagactccgcagagttaaaagatcaactgacagggggc

gaagcgagttggcggactgccaccagaagatcatcaatcatgccgtctagccggtccctg

tcccgctgcctacaaagcgggtaccactgccgcaagaaggccaaaattttaaacacagag

tcagaagcacactgcaagaggactcacttgataaccatcttgtcacgtacagtccattgg

gtccaggtaatcactgcgaacactaacctgaagaggcgcctcctcctaccggtatgtttg

gcctgagcctccaggaactcgctcaggtccggcgcctgccactcaagcgctagagcctca

cggacaaagttccataaggaggcagcgggacgagagaagaaaatgtgggtcctggtctcc

tggatttggaaaccacacaaagaaggctaaggccatcacccagcccatgccgcttcgcca

cctccaaacccgatgggaggcgattacagagcagctgccaaacaaagattttgatcttca

ggcagtggagcttttcacaagtgggaggtccacacaagggtcgacacacaatagagggca

cggtatgccaacccggcagagaagagtgccgaggaggtgactgtgccaagacacgacgcc

cggcgacctaaacagcgatgatggaagagccgtccgcaactcggtctaggctaaggtttc

ctttggccgaatgttcgttggaacggtatgtcccaattcccattgtgggccgccgaggct

accaagagcaccggatccgagcaaatcatgaaatggtccgggaatttggcacgcaacgtg

gtcctaccgagccacgggtcgagccaaaagagggtaccctctccatcgcctatgaagaat

tgatacccaagtgaatctcatgcttgatcgactggagggatttcaagaactgagatcctt

cccaacggtcacaagccaggagagggtggccctgcaggtgtttggcttttataagctgca

gccataggccaccctcgtccccatggatccgccacacccacctcagcatgagtaccacat

ttgaactgctcccaggggcagcagaagtgtgctcgctgttgatgtataaatgtattgcct

agtctcttccatcagattggtcttttggttgcattgactagagcatgcacttctacatgg

tattagagtcaagaggtcttgagttcaagaccggctggcgcaattaaattgcaggccact

ttcggtccacgtttaggcctgagggagccacacgtgagggggagtgttgacatatgaatg

tattgcctagtctcttccatcagatcggtgttttggttgcattgggtagagcatgcactt

ctacactccccacggtggagcacatccaaaccggaaaagagagaagaaaaaaaggaggtg

tgctcctgcacgaaccacaggtctgctgctcccgcgctggagcacatcggaacccgaaag

gaaaaatgaaggtgtgcttccatcgcgggtggagcacatgaagttgtgaaccttaggttc

atttcagatatggctggtaaatttggttacacttgaagttgtgaaccttaggttcattga

tctatgtgtagtaaaagatcaagtactttctgaaaaaagaatgtaggcctcctcatctaa

gagtattaccttttgataccgaaatgctgtttcctgactacaggcaaaccgcgtactcat

ttgatattgaatatgtgttaggttcattgatctatgtgtagtaaaagatcaagtactctc

tgaaaaaagaatgtaggcctcctcatctaagagtataaccttttgataccgaaatgctgt

ttcctgactacaggcaaaccacgtactcatttgatattgaatatgtgatctgcctttgat

accctttgttttgaaggaagggtatttcttttacgtatattggtacacaaaggtaagttc

cttgcttgttgcttgaatcataggcagatgtctgagtgcatgtttgctctaatacattag

ccagagatatatagtttggtagcgtttgttaagtcaactactcatctacgtgacttacgg

ccattgttatgtttattgcag

ATGCTTGCTGTACATTACTACCTGATCTTTGGTGTCACATGAAGTCATTCTATGGACAAG

ATTCTACTTTCAG

gtagaggaatatcttgggctacgtttctctcgtagtaaccaatcccttactgtattttcc

agttgtccaaaataccttagcttccactgttgcttggatcacatgtctttattatgcttg

gctgcggaactataattgaactttctttcctgttattttacatgaactgtgcagacttat

tgacatgatatttttattctag

TTTCTCAGGGGAAACACATGTCGGTAAGAGCGACATATTCGATACTTTGTCAAGAATAAG

GGAAATGCTAAAAGGTAACAGCCGTTGCGAGAAGATTGGCCGTGTGATGGAGAAGGTTTA

CTGTCGTGCGCATTGGGAAGAAGGGATGAGTGTCCGAGTTTCTGGATCATTCATTATGGG

GAACGAGTTCCTTGTGTGCGCAGATGGGTTTTGCGCTGAAGGGATGCTAAGCATGGTCGA

ACTCTCTCCCGACATTCTCAGCAAGCAGGCAGGCCATTTCCAGGAAGATTTTTTCATGGA

GCCAGGGACTGCCATGGGATGCTATGTGATATCAAAGCAAGAATTGCACATCGGCGTATC

ATGAAGTCATTCGCCTTCTTGCATGCAATCATTACATATGATGATTTTTGTGCTTTTTAC

TGCCGTGCATTTGCTGAACTGATCTATCATGCATGTTATCTTCCCATTTGACGCTGAAGA

AAATAATCTTCTAGCATATCGACATATATCGACATTGAGATATTGGAATCTATTATTTTT

CTCGAATACGCACGAGTGTGCGTATTATATATTAATAGAAGGAAAAGGAGGAAGAGCCCC

ATCCGTGAATATTTACATGGCGTTTACACAACGCGATCCACCGTGGTCTCTAGGTGGATT

ACTCACCGTGTACCCACCCATCTACTCCTACACCTACAGGGAAAATGTTAAGATCAGCGT

GCTCCAGATCACACCCTCTGATTCTATCCTCCATAGCACATGTCGCACATCCGGGCTTTT

TCCATCAAATACAATTGAATTCCGATGCTTTCACAACTCCCACGTAACCAATACCTG

**>TaMKK3-3 TRIAE_CS42_5BL_TGACv1_405070_AA1319320.2.X (based on AA1319320.2)**

CGGTCATCTCTCAAATTTGGGACTATCGCACTGGCGACGGAGGAAAGCATGTGCGTCCCG

TCTCGTCGTGGCAAACGGAAGCGAAATCAAAGCCTCACTCTGTTTCTCTGCGCCCTCTCC

CTCACCCTTCTCTTAGGCTTAGCCTTGTTTGGCTGTAATGGCTGGACTGGAGGACCTGAA

GCGGAGGCTGCAGCCCATCTTCTTCGACGCCGATGGCAATGTTGTGCCGCCCCCCGCCGC

CGACGGGACGTCCGACGACTCCAGCTCTGACGACTGCGAG

gtaaccactctcttcgtttcagccgtttggtcagttcgctgagatctatgtttatcttca

ttctgttcaaccaacaaaattccttgcttatgttgctcatactatgttttttcttaagcc

tgctataaaaatatatatgatgctcatattttaatcttacttttgtaaag

GTTTTGGATGGTGGAACTGTCAATTTATTGAGTAGGTCCTCTGATGAATATAACATCAGT

AAGCTTGGCTTCCATAAACGAACAACCAGACCAGATGGGGACTATGCTACAGATAAGGCA

TATCGATGTTCTTGTCATGATATGCATATTTTTGATTCTGTTGGTAATGGTGCAAGCAGC

GTTGTCTATAGAGCTATTTATGTACCAGTCCATCGAGTTTTGGCACTCAAGAAAATCAAC

ATTTTTGACAAG

gtgagtgcaaaaaaatttatagtccatatgccataagctgacatctctatgttcccctta

catgaaacaaaatctatggatacccttcttttttaaaattaactttcacatggaactaca

agtgtcgaattcatagcttattctccaattaatgcctatagtactccagtattagttgga

aattgtttgaaccaagctgcagcaagtagaaccagaaatgcagcaagtactatagacatt

ttttggaccaagcttattctctttatatttattataacgtattctctgatatatatcaca

g

GAAAGGAGACAACAGATTCTTAATGAGATTAGAACGTTATCAGCAGCATCTTGTTATCCA

GGTTTAGTTGAATTCCAAGGAGTTTTTTACACCCCGGACTCTGGAGAAATATACTTTGCT

CTTGAGTATATGGATGGTGGTTCATTAGCAGATATTATCAGGGTCAAGAAATTCATAACA

GAACCAGTTCTTTCACATATGCTACAGAAAGTGTTGCTA

gtacgtgcctcttgccaccgtatgttctgatgttagctctattcaaatcaggcctgtgaa

atttaagtgactagttggtctgtgttaatttattag

GCTCTGCGCTACTTGCATGAAGTGAGGCGTTTAGTTCACAGAGATATAAAGCCAGCAAAT

TTGCTTCTAAATCTAAAGGGTGATACGAAAATTACAGACTTCGGTGTAACTTCTGGACTG

CATGATTCAGTTACCATG

gtatgctacttcttggtgaccgaaacttctcttagcaacatcatgctgttctaaatagta

ttaagtggacatctgagtatctagattgaataagcagtatatatgtgtagtaattgtctt

ccag

TGTGCTACCTTCCTGGGCAGTGTCACATATATGTCTCCCGAGAGAATTCGGAACGAAAAT

TACTCATATGCTGCTGATATCTGGAGCCTTGGACTAACAGCATTGGAGTGTGCAACTGGA

AGATACCCATATGATGTAAATGGGGGCGAGGCCGACCTCATGCTGCAG

gtgaggtggttggcggtcaagcagtgttcatttgtataattttctactgcctttcatgat

catcttatttatggaaagaataatcatgatctcatttatgtgtatctgttggtatattgt

tgattaaagctgttttcggaatttggtttgtttttcgtaggcgttctacatctgttttcc

gaatttctactggatttagaacttctatttcatttctgctttccaacatgaatggtctga

caataatgcaatactagttcggtagttcctcttgtgagtattatattattgatgtataat

gcaatcctcctttcccaccttactgtgttgcagttttgcag

ATATTGGAAGATCCATCGCCAACACCGCCACAACATATGCATTCAGAAGAGTTTTGCTTG

TTCATCGATGCTTGCTTGCAGAAAGATGCTGATGCAAGACCAACATGTGATGAG

gtaaagtgacgtctagtacaattaaggtttttcaagagaaacgatacgagggtttccaac

tgcgtaaatcttgtgataatctttatttattatttaatttttgcggggagtgataaactt

tattatgcagtaagtataggattttgcaacactatctgagagtaaagtctgatggatgtt

tcagctctttagtcaacagattagcggtctgaggtgataatttcaaaaaaccggtctcat

atgttgaattgttttacaaattattcattgtcaactaactacataaataggttatttgca

acaaagacgaaatggggtttattttgctctctgaaataaataaaaaatcttatttgaatc

acatactgaacatcttgtag

CTTTTGTCTCATTCCTTCATCAAGAAATACGAGGGACCTGGTGTGGACTTGGCAGAGTAC

AACAGAAGTGTTCATGATCCAGCAGAAAGATTATCACAAATAGCACAT

gtgagttcctaatagctttcgttagcttatatttttcctgttctgctgcctttccatatg

gaggaaaatgaacctgatgttcggcaagttcacatacaatgatgttagctatgcttagac

aatagaacattccgacaattcaacaaataactgactcttactgcagttgtttcaatcttt

tgaatctggtttcttgctatccgggattcaaaattcatgttaaaaattttgacctcattg

aggatttcgtttgccacaagcatagtttaaaaaaccggaccgggccagcggtcggagcgg

aaaaaaactgaaccggcggacttggcggtttttgaagctagaagaccattctgcaattgg

accggagaaaaccagtcaagccggccggttttctggaaaaaaacgctgaataaaccaggc

ctttgaaccggaaaaaccaggagaatgtttaatgtagaatttgggagaagcgggattcaa

tcctaggatgcgtgagtagtaggcattgcgagctcatggtccaataaccaactcggacgt

gctactttgttgtctattatagggaagtaattttatttgaccattgtttaacactgtttc

atttggccattgtctactaaacgtatattattttatggtaaaaaaccgacgttgaaccgg

tgaaccggcggtccaactgatgaaaacctgaaccgacaggctcacaggttcgatcttcgg

tgcagtttttaaaactatggccacaagtatatctcagaaaaagaaattggagctgggaaa

acgtgatttgtggttttgctatccatggagtgggacgaatttacaggaactttgccgctg

ccaagtcacttaccgtttcgcttgaatatactcggccctaaacgtttctcccctcgaata

tgagtgcgcccgtcgtgctcacgcggccgctcacctcagttacagccgcttcgccgctgc

tctcttcacctccaaccactcttgaatagtctttcaccccgctttatagataaagcaacc

accatttccacacaagtagttaaagtgcaggaaagtaaatagaagatctgctggggcaca

aataagcccaaaagcaaaaaaaaaaggcggcacaagactccgcagagttaaaagatcaac

ggatagggtgcgaagcgagttggcggactgccaccagaagatcatcaatcatgccgtcta

gccggtccctgtcccgctgcctacaaagcgggtaccactgccgcaagaaggccaaaattt

taaacacaaagtcagaagcacactgcaagaagactcgcttgataaccatcttgtcacgta

cagtccattgggttcaggtaatcactgcgaacactaacctgaagaggcgccgcctcctac

cggtatgtttggcctgagcctccaggaactcgctcaggtccggcgcctgccactcaagcc

ctagagcctcacggacaaagtcccacaaggaggcagcgggacgcgagaagaaaatgtggg

tcccggatttgggaaccacacaaagaaggctaaggccatcacccggcccatgccgcttca

ccacctccaaacccgatgggaggcgattatagagcagctgccaaacaaagattttgatct

tcagaggcagcggagcttttcacaaatgggaggtacacacaagggtcgacgcacaataga

gggcacggtatgccaacccggcagagaagagcgccgagaaggtgactgtgccaagacacg

acgcccggggacctcaacagcgatgacggaagagccgtccgcaactcggtctaggctaag

gtttcctttggccgaatgttcgttggaacgggatgtcccaattcccattgtgggccgccg

aggctaccaagagcaccggatccgagcaaatcatgaaatggcccaggaattcggcacgca

acgtggtcctaccgagccacgggttgagccaaaagaaggtaccctttccatcgcctatgg

agaattgatacccaagtgaatctcatgcttgatcgactggagggatttccagaactgaga

tccttcccaacggtcaaaagccaggagaggatggccctgcaggtgtttggcttttataag

ctgcagccataggccaccctcgtccctaaggatccgccacacccacctcagcatgagtac

cacatttgaactgctcccagggcagcagaagtgtggtccctgttgacgtataaatgtatt

gcctagtctcttccattagattggtcttttggttgcattgactagagcatgcacttctac

atggtatcagagccaagcggtcttgagttcaagacccttgcagcccacttccggtccacg

tttaggcccgagggagccacacgtgagtgggagtgttgacgtataaatgtattgcctagt

cttttccggttttttggttgcattggctagagcatgcacttctacactccccacggtgga

gcacatccaaaccggaaaagagagaagaaaaaaggaggtgggctcctgcacgaaccacag

gttgctgctcccgcgctggagcacattggaacccgaaaggaaaaatgaaggtgtgcttcc

atcgtggtggagcacatgaagttgtgaaccttaggttcatttcagatatggctggtaaat

ttggttacacttgaagttgtgaaccttaggttcattgatctatgtgtagtaaaagatcaa

gtactttctgaaaaaagaatgtaggcctcctcgtctaagtgtataaccttttgaccgaaa

tgccgtttcctgattacaggcaaaccagtactcactttgatattgaatatgtgatctgcc

tttgatacccttagttttgaaggaagggtatttcttttccgtatattggtactcaatggt

aagttccttgcttgttgcttgaatcataggcagatgtctgagtgcatgtttactttaata

cattagccagagatataaagtttggtagcgttagttaggtcaactactcatctacatgac

ttacggccattgttatgtttattgcag

ATGCTTGCTGTACATTACTACCTGATCTTTGACGGTGGTGATGACCAATGGCATCACATG

AAGTCATTCTATGGACAAGATTCTACTTTCAG

gtagaggaatatcttgggctatgtttctctcgtagtaaccaatccgttgctgtattttcc

tgttgtgcaaaataccttagcttccactgttgcttggatcgcatgtctttattatgcttg

gctccggaactataattgaactttctttcctgttattttacatgaattgtgcagacttat

tgacatgatatttttattctag

TTTCTCAGGGGAAACACATGTCGGTAAGAGCGACATATTCGATACTTTGTCAAGAATAAG

GGAAATGCTAAAAGGTAACAGCCGTTGCGAGAAGATTGGCCGTGTGATGGAGAAGGTTTA

CTCGTGCACATGGGGAAGAAGGGACGAGTGTTCGAGTTTCTGGATCATTCATTATGGGGA

ACGAGTTCCTTGTGTGCGCAGATGGGTTTTGTGCTGAAGGGATGCTAAGCATCGTCGAAC

TCTCTCCCGACATTCTCAGCAAGCAGGCAGGCCATTTCCAGGAAGATTTTTTCATGGAGC

CAGGGACTGCCATGGGATGCTATGTGATATCAAGGCAAGAATTGCACATTGGCGTATCAT

GAAGTCATTCGCCTTCTTGCATGCAATCATTACATATGATGATTTTTGTGCTTTTTACTG

CCGGGCATTTGCTGAACCGATCTATCATGCATGTTATCTTTCCATTTTATGCTGAAGAAA

ATAATCTTCTAGCATATCGACATTGAGGTATTGGAATCTATTCAGTTGAGCCTCAACACA

GTCTACAATGAACTGATAACATCGGTTATTGCCTCTCTGCATTCAGCATTTACGCTTGTA

AGTTGAATGACGGGGAGACATTTAGATGACGGGGAAAAGCTTTCCCATGCTATGAACTGC

ATATGTTAGATATACGACAGTGATTTTTTCTATT

**>TaMKK3-3(5DL) TRIAE_CS42_5DL_TGACv1_435057_AA1445420.4**

ATAGCGGGAGTGGCCTCCATCTCCATCCCTTGTTATCGGGAAGACAGCAAATGAGACAAG

ACAAGAGGAGGCGCCGGCACTTCCTTGTCGCTGGATTCACGACTTGCCGACCTACCTGCG

CCGCCTCAGGCCATCTCTCAAATCTGGGACTATTGCACTGGCGACGGAGGAAAGCATGTG

CGTCCCTTCCCGTCGTGGCAAACGGAAGCGAAATCAAAGCCTCACTCTGTTTTCTCTGCG

CCCTCTCCCTCACCCTTCTCCTCTTAGGCTTAGCCTTGTTTGGCTGTAATGGCTGGACTG

GAGGAGCTGAAGCGGAGGCTGCAGCCCATCTTCTTCGACGCCGATGGCAATGTTGTGCCG

CCCCCCGCCGCCGACGGGACGTCCGACGACTCCAGCTCTGACGACTGCGAG

gtaaccactctcttcgtttcagccgtttggtcagttcgctgagatctatgtttatcttca

ttctgttcaaccaacaaaattccttgcttatgttgctcatagtatgtttttttcttaagc

ctgctataaaaatacatatgatgctcatattttaatcttacttttgtaaag

GTTTTGGATGGTGGAACTGTCAATTTATTGAGTAGGTCCTCTGATGAATATAACATCAGT

AAGCTTGGCTTCCATAAACGAACAACCAGACCAGATGGGGACTATGCTACAGATAAGGCA

TATCGATGTTGTTGTCATGATATGCATATTTTTGATTCTGTTGGTAACGGTGCAAGCAGC

GTTGTCCATAGAGCTATTTATGTACCAGTCCATCGAGTTTTGGCACTCAAGAAAATCAAC

ATTTTTGACAAG

gtgagtgcaaaactttttatagtccatataccataagctgacatctctatgttcccctta

catgaaacaaaatctatggatacccccccttttttttcctttttcttttaattaactttc

atatggaactaactacaagtgtcaaattcatcgcttgttctccatttaatgcctatagta

ctccagtattagttttttttttttttttttttgcgcatagtactccagtattagttggaa

attgtttgaaccaagctgcagcaagtagaaccggaaatgcagcaagtactatagactttg

tttggaccaagcttcttctctttatatttattataacgtattctctggtatatattacag

GAAAAGAGACAACAAATTCTTAATGAGATTAGAACGTTATCAGCAGCATCTTGTTATCCA

GGTTTAGTTGAATTCCAAGGAGTTTTTTATACCCCGGACTCTGGAGAAATATACTTTGCT

CTTGAGTATATGGATGGTGGTTCATTAGCAGATATTATCAGGGTCAAGAAATTCATAACA

GAACCAGTTCTTTCACATATGCTACAGAAAGTGTTGCTA

gtacgtgtctcttgcctccataagttctgatgttagctctattcaaatcaggcctgtgaa

ctttaaatgactagttggtctgtgttaatttattag

GCTCTGCGCTACTTGCATGAAGTGAGGCGTTTAGTTCACAGAGATATAAAGCCAGCAAAT

TTGCTGCTAAATCTAAAGGGTGATACGAAAATTACAGACTTTGGTGTATCTTCTGGATTG

CATGATTCAGTTACCATG

gtatgctatttcttggtgaccgaaacttctcttagcaacagcaagctgttctaaaatagt

attaagtggacatctgagtatctagattgaataagcagtatatatgtgtagtaattgtct

tccag

TGTGCTACCTTCCTGGGCAGTGTCACATATATGTCTCCTGAGAGAATTCGGAACGAAAAT

TACTCATATGCTGCTGATATCTGGAGCCTTGGACTAACAGCATTGGAGTGTGCAACTGGA

AGATACCCATATGATGTAAATGGGGGCGAGGCCGACCTCATGCTGCAG

gtgaggtggttggtggtcaagcagtgttcatttgtataattttctactgcctttcgtgat

catcttatttatggaaaatatcatgatctcatttatgtgtatctgttggtatattgttga

gtaaagctgttttcggaatttggcttgtttttcataggcgttctacatctgtttccgaat

ttctactggatttagaacttctatttcatttctgctttccaacatgaatggtctgacaat

aatgcaatactagttcggtagttcctcttgtgagtattatattattgatgtataatgcaa

tcctcctttcccaccttactgtgttgcagtcttgcag

ATATTGGAAGATCCATCGCCAACACCACCGCAACATATGCATTCAGAAGAGTTTTGCTCG

TTCATCGATGCTTGCTTGCAGAAAGATGCTGATGCAAGACCAACATGTGATCAG

gtaaagtgacgtctagtacaattaatgtttttcaaaagaaacggtaggagggtttccgac

tgcgtaaatcttgtgataatctttatttatttatttatttattgcggggagtgataaact

ttatgatgcagtatagggttttgcgacactatctgagagtaatgtctgatggatacagat

tagcggtctaaggtgtattaatcacacacacacacacacacacacacacacacacacaca

attctgaggtgataattgcaaaaaagtggtctcatatgttgaattgttttgcaaattatt

cattgtcaactaactacttaaataggttatttgcaacaaagacgaaatggggtttatttt

gctctctgaaattaaaaaaatcttatttgaatcacatactaaacatcttgtag

CTTTTGTCTCATTCCTTCATCAAGAAATACGAGGGACCTGGTGTGGACTTGGCAGAGTAC

AACAAAAGTGTTCATGATCCAGCGGAAAGATTATCACAGATAGCACAT

gtgagttcctaatagccttcgttagcttatatttttcctgttctgctgcctttccatatg

gcaggaaaatgaacctgatgttctccaagttcacatacaatgatgttagctatgcttaga

caatagaacattccaacaattcagcaaataaactgactcttactgcagttgtttcaatct

ttttaatctggtttcttgctatccgggattcaaaatattcatgttaaaattttagacctc

gttgaggatttcggttgccacaagcatagtttaaaaaaccagaccgggccagcggtcgga

ccgaaaaaaactggaaccggcggcgtcggcggttttttaagctaataagacggttctgca

attggaccagagaaaaccagtcaagccggccggttttctggaaaaaaaacgacgaataaa

ccaggcctttgaaccggaaaaaaccaggaaaatgtttaatgtagaatttgggagaagcgg

gattcaatcctaggatgtgtgagtagtaggcattgcgtgctcatggcccagtaaccaact

cggtcgtgctactttgttgtctattatagggaagtaattttatttgaccattgtttaaca

ctgttttatttggccattgtctactaaacgtatattattttatggtaaaaacccgacgaa

tgaaccggtgaaccggcggtccaactgacgaaaacctgaaccaacaggctcaccggttcg

atcttcggttcagttttttaaactatggccacaagtatatctcagaaaaataaattggag

cagggaaaaacgtgctttgtggttttgctattcatggagtgggacgaatttacaggaact

ttgccactgccaggtcacttactgtttcgcttgaatatactcggccctaaatgtttctcc

ccttgaatatgaatgcgcccgccatgctcacgtggccgctcacctcagctatagccgctt

acgccgctgctctcttcacctccaaccactcttgaaatagtctttcaccccgctttatag

ataaagcaaccaccatttccacacaagtagttaaagtgcaggaaagtaaatagaaggtct

gctggggctcaaataagcccaaaagcaaaaaagagaaggcgacacaagactccggagagt

taaaagatcaacggatagggcgcgaagcgagttggcgggctgccaccagaagatcatcaa

tcatgccattctagccggtccctgtccgctgcctacaaagcaggtaccactgctgcaaga

aggccaaaattttaaacagagtcagaagcacactgcaagaagactcgcttgataaccatc

ttgtcacgtacagtccattgggtccaggtaatcactgcaaacactaaccagaaggggcgc

ctcctcctaccggtatgtttggtctgagcctccaggaactcgctcaggtccggcgcctgc

cactcaagccctagagcctcacggacaaagttccataaggaggcagcgggacgagagaag

aaaatgtgggtcccggatttgggaaccacgcaaagaaggctaaggccatcacccggccca

tgccgctttgccacctccacacccgatgggaggcgattacatagcagctgccaaacaaag

attttgatcttcatagacagcggaggttttcacaagtgggaggtccacacaagagtctgg

cgcacaatagagggcacggtatgccaacccggcagagaagagcgccgaggaggtgatgtg

ccaagacacgacgccaggggacctcaacagcgatgacagaagagccgtccacaacttggt

ctaggctaaggtttcctttggccgaatgttcgttggaacgggatatcccaattcccatta

tgggctgccgaggctaccaagagcaccggatccgagcaaatcacgaaatggcccgggaat

tcggcacgcaacgtggtcctaccgagccaggggtcgagccaaaagagggcaccctctcca

tcgcctatggagaaactgattttttttttgaaaaggaggatacacccccggcctatgcat

ctgggagatgcatgcagccactatattaattattcacaaagaccttacaaaataatacat

cagtaagcctgaagccaccatcttggcaacacctgtcgctactcctatgcccttgatgaa

ccgaatatccgagccgaataccaaacagacatcgcaacaaagcctaacatctaaagccgg

atgccccagcccagccacatattgggactgtgtcacacaccggtccggcgcactctcaga

agccgccgccaccgtcttccactggtcaatctccagagcaggtactgacacaccgacctt

gtcaggcctgccatcgacgccaccacggcgccagacagcgccaccatcctgcacgtatcc

atccggccgcgcccgtcgccgaaactccgcagcgccatgccgctgggatccatcgtcagc

catgcctatggagaaactgacacccaagtgaatctcatgcttgatcgactggaaggattt

tcagaactgagatccctcccaacggtcacaagccaggagaggatggccctgcaggtgttt

ggctctttataagctgcagccataggccaccctcacctcagcgtgagtaccacatttaaa

ctgctcccacgggcagcagaagtgtgctctccacgatggagcacatccaaaccggaaaag

agagaagaaaaaaaaaaggaggtgtgctcctgcacgagccacaggtatacctggccatac

ctcgggccgggctgggcctagccaagcccgacgcaaaaaacccaggcccgggcctggccc

ggcccggccatcgggcctgttttttgggcctgagcccggcccaaacacgtaaaagcccgt

cgggctccgggccggcccggcccgaccttcagaaaagtgcaaaaacgacgggcccgggcc

cggcccggcccggccatcgggctcaaaatctaggcccgagcctggcccgggagcagcgtc

gggctgggccgggtcgggccgggccgggcttccatggccaggtatagccacaggtctgct

cccgcgctggagcacatcggaacccgaaaggagccttggcgcagtggcaaagctgctgcc

ttgtgaccatgaggtcacgggttcaagtcctggaaacagcctcttgcagaaatgtaggga

aaggctgcgtacaatagacccaaagtggtcggacccttccccagaccctgcgcaagcaag

agctacatgcaccgggctgcctttttttgaagttgtgaaccttacattcattgatctata

tgtagtaaaagataaagtactttctgaaaaaagaatgtaggcctcttcatctaagagtat

aaccttttgataccgaaatgctgtttcctgactacaggcaaaccatgtactcactttgat

attgggaccctgcgcaagcaggagctacatgcactgggctgcccttttttgaagttgtga

accttatgttcattgatgtatgtgtagtaaaagatcaagtactttctgaaaaaagaatgt

aggcctcttcatctaagagtataaccttttgataccgaaatgctgtttcctgactacagg

caaaccatgtactcacttgatattgaatatgtgatctgcctttgatacccttagttttga

aggaagggtatttcttttccgtatattagtactcaaaggtaagttccttgcttgttgctt

gaatcataggcagatgtctgagtgcatgtttactctatacattagccagagatatatagt

ttggtagcattagttaagtcaactactctactcatctacacgacttacggccattgttat

gtttatcgcag

ATGCTTGCTGTACATTACTACCTGATCTTTGACGGTGGTGATGACCAATGGCGTCACATG

AAGTCATTCTATGGACAAGATTCTACTTTCAG

gtagaggaatatcttgggctacgtttctctcgtagtaacaaatcccttactgtattttcc

agttgtgcaaaataccttagtttccactgcttggatcgcatgtctttattatgcttggct

ccggaactataattgaactttctttcctgttaattgtacatgaactgtggagacttattg

acatgatatttttattctag

TTTCTCAGGGGAAACACATGTCGGTAAGAGCGACATATTCGATACTTTGTCAAGAATAAG

GGAAATGCTAAAAGGTAACAGCCGTTGCGAAAAGATTGGCCGTGTGATGGAGAAGGTTTA

CTGCCGTGCACATGGGGAAGAAGGGATGAGTGTTCGAGTTTCTGGATCATTCATTATGGG

GAACGAGTTCCTCGTGTGCGCAGATGGGTTTTGTGCTGAAGGGATGCTAAGCATCGTCGA

ACTCTCTCCCGACATTCTCAGCAAGCAGGCAGGCCATTTCCAGGAAGATTTTTTCATGGA

GCCAGGGACTGCCATGGGATGCTATGTGATATCAAGGCAAGAATTGCACATTGGCGTATC

ATGAAGTCATTCGCCTTCTTGCATGCGGTCATTACATATGATGATTTTTGTGCTTTTTAC

TGCCGTGCATTTGCTGAACTGATCTATCATGCATGTTGTCTTTCCATTTGATGCTGAAGA

AAATAATCTTCTAGCATATCGACATTGAGGTATTGGAATTTATTCAATTGAGGTTCAACA

GAGTCTACAATGAACTGAAAACATCGGTTATTGCCTCTCTGCATTCAGCATTTATGCTTG

TAAGTTTAATGACGGGGAGACATTTACGCTTGTAAAATGACGGGGAAAAGCATTCCCATG

CTATGAAGCAAATTCCATGTTATGCTACAGTGTTTTTTCTTTCT

**>TaMKK4(6Al) TRIAE_CS42_6AL_TGACv1_472053_AA1517260.1.X (reverse strand)**

AACAAGAAATTGCATTTTTAACAGCCCATACTGTATCATCATCATCATCATCATCAGAAT

AATCATCATCATCATCATCAGAATAATCATCATCATTTGGAGGGGAGAACATTTAATTGA

CATTAACAGGAACTAAAGAAAAAGCTTGAGCTTAATTTCAGGAGCGACGGCAACAACAGA

GGGATTGGATGGGTAAATTTGTGGTGGTGGCTTCACTACCCAAAATGGCTAAGAGGAGGA

GGAAAGATCGATCGCCCCTCCTCCTAGATCCCACGTCCCGCCACCGGCCACCGTCCACCC

ATGATCCCTCCGAATTTAACTTGGTTACGGCGGGGGAGCCGTCAGCACGGCGGGGCGGCG

AGGGCCTGCGGCTGCTGCGGCGGCGGCGAGGCGATGAACCGGTGCTGCAGCAGCTGCGCC

GCGGACGGCCGCTTCGCCGGGTTTTTCTGGAGGCAGCAGCTGATGAAGCTCCGCAGCTCC

GGGGACGCGGTGGGCGGCGCCGCCGGCGACTCGGAGTAGCAGATGGCGCACATGAGGGCC

GCCCAGTCGCCCTGCTTCCCCAGGTTCTCCCCGAGCGGGAAGCGGCCCAGGTAGAACTCG

AGGATGCTGAGGCCGAAGCTCCAGATGTCGCCGGCGTAGCCGTTGTAGTTGCCGTCGTTG

AGGTCGGTGTTGATGCGCTCGGGGCTCATGTACGCGATGGTGCCCACGGAGGAGTTGCAG

GGGTCCATGGTCTGGTTCAGGATGCGCCCCACCCCGAAGTCGGCGATCTTCACGCGCCGC

CCGCAGTCGATGAGCAGGTTGGACGGCTTGATGTCGCGGTGCACGATGTGGCACCGGTGG

AGGTAGGCGATCCCCGACAGCACCTGCCGCGCCACGTCCGCGAGGAAGGACTCGGACGCG

ATGCGCCGGCCGTCCAGCGACCCGCCGTCCATGTACTCGAGCAGGATCTGCAGCTCGCCG

GCCTGCTCGTACATGCCGTGGCAGCGCACGATGGACGGGTGCTCCGCCGTGCGCAGGATG

GCGATCTCCCGCGTGATCTGCCGCCGGACCGCCTCGTCGTGGTGCCCGTAGAGCACCTTG

AGCGCGTAGGCGCGGCCCGTGGGCGCGTGCCGCACCAGCCACACCGTCCCGCCGGCGCCG

CTCCCGACGCGGCGGACCCGCTCCAGCTCGCCCAGCGGCGGGGGCGCGGGCGGCGCGGAG

CCGGCCGAGTTGGGCGGGGTCATGGACATGGGCATGGGCATGGACGACGCGCCGCCCGAC

CCCGACCCCGACCCGGACGAGGACGTGGACGACGGCGCGGAGGACGGGGGCGGGGGCAGC

GGCAGCGGCACGGCCAGCGACGTCAGGTCGCGCTGCGGGAGCGGGAGCGTGAGATCCGGC

CGGCGCCGCGCCCGGCCCGGCGTGCCGGGCTGCTGCTGCTGCAGGCCCGGCCGCGCGTTC

GGCGGCCCGCCCGGACGCATCGCGCGGTCTGGCCGGTCCGAAAGGCGAGGCTGGCTCCTC

CCCTGCTCCGCGCGGCGGCCGGATCGGTCGGCGTCGGCGATTGCCGTCGTATGGGGCGGG

GTGGGGTGGGGTGGGGTGGGATCGATCGGGTCGGGGAGGAGGGATTGGAGGGAGGCGAGT

ATTTTTTATAGCCGCGAGGGAGGCCAAGGGACGGGGAGAGAGACGGAGGGAGGGAGCTCT

GCTCGCTCAGCCGATGGAGATGCAGCCGTGACGCCGGGGGAACGGCAGGCAGGCAGGCCC

GGTAATAAAAGAACCAACTTTCC

**>TaMKK4(6BL) TRIAE_CS42_6BL_TGACv1_502626_AA1625820.1**

GTCACGGCTGCATCTCGCTCGGCTGAGCGAGCGAGCGAGCGAGCAGAGCTCCCTCCCTCC

GTCTCTCCTCCCCGTCCCTTGGCCTCCCTCGCGGCTATAAAAAACACTCGCCTCCCTCCA

ATCCCTCTTCCCCGACCCGATCGATCCCACCCCCACCCCACCCCGCCCCATACGGCGACA

ATCGCCGACGCCGACCGATCCGGCCGCCGCGCGGAGCAGGGGAGGAGCCAGCCTCGCGTT

TCGGGCCGGCCAGAGCGCGCGATGCGTCCGGGCGGGCCGCCGAACGCGCGGCCGGGCCTG

CAGCAGCAGCAGCCCGGCACGCCGGGCCGGGCGCGGCGCCGGCCGGATCTCACGCTGCCG

CTGCCCCAGCGCGACCTGACGTCGCTGGCCGTGCCGCTGCCGCTGCCCCCGCCCCCGTCC

TCGGCGCCGTCGTCCACGTCGTCCTCGGGGTCGGGGTCGGGGTCGGGCGGCGCGTCGTCC

ATGCCCATGCCCATGTCCATGACCCCGCCCAACTCGGCCGGCTCCGCGCCGCCCGCCCCG

CCGCCGCTGGGCGAGCTGGAGCGCGTGCGGCGCGTCGGGAGCGGCGCCGGCGGGACGGTG

TGGCTGGTGCGGCACGCGCCCACGGGCCGGGCCTACGCGCTCAAGGTGCTCTACGGGCAC

CACGACGAGGCGGTCCGGCGGCAGATCACGCGGGAGATCGCCATCCTGCGCACCGCGGAG

CACCCGTCCATCGTGCGCTGCCACGGCATGTACGAGCAGGCCGGCGAGCTGCAGATCCTG

CTCGAGTACATGGACGGCGGGTCCCTGGACGGCCGGCGCATCGCGTCCGAGGCCTTCCTC

GCCGACGTAGCGCGGCAGGTGCTGTCGGGGATCGCGTACCTCCACCGGCGCCACATCGTG

CACCGCGACATCAAGCCGTCCAACCTGCTCATCGACTCGGGGCGGCGCGTGAAGATCGCC

GACTTCGGGGTGGGGCGCATCCTGAACCAGACCATGGACCCCTGCAACTCCTCCGTGGGC

ACCATCGCGTACATGAGCCCCGAGCGCATCAACACGGACCTCAACGACGGCAACTACAAC

GGCTACGCCGGCGACATCTGGAGCTTCGGCCTCAGCATCCTCGAGTTCTACCTGGGCCGC

TTCCCGCTGGGGGAGAACCTGGGGAAGCAGGGCGACTGGGCGGCGCTCATGTGCGCCATC

TGCTACTCCGAGTCGCCGGCCGCCCCGCCCACGGCCTCCCCGGAGCTGCGGAGCTTCATC

AGCTGCTGCCTCCAGAAGAACCCGGCGAAGCGGCCGTCGGCGGCGCAGCTGCTGCAGCAC

CGGTTCATCGCCTCGCCGCCGCAGCAGCAGCCGCAGGTCCTCGCCGCCCCGCCGTGCTGA

CGGCCCGCCCGCCGTAACCAAGTTAAATTCGGACGGATCATGGACGGACGGACGGACGGA

CGGACGGACGGTGGCCGGCGGCGAGACGCGGGATCTAGGGGGAGGAGGGGCGATCTGTCT

TCTTCTTAGCCATTTTGGGAACTGAAGCCACCACCACAAATTTACCCATCCATCCCTCGG

AGTCCTGTTGTTGCTGTCGCCCCCGAAATTAAGCTCAAGCTTTTTCTTTAGTTTCCGTTA

ATGTCAATTAAATGTTCTCCCCTCCAAATGATGATGATTCTGATTCTGATTCTGATGATG

ATGATATAATATGGGCCGTTAAGAAATCAATTTTCTTGTT

**>TaMKK4(6DL) TRIAE_CS42_6DL_TGACv1_528809_AA1716310.1.X (based on AA1716310.1)**

GCCCACCTCTCTCATACGACGGCAATCACCGACGCCGACCGATCCGGCCGCCGCGCGGAG

CAGGGGAGGAGCCAGCCTCGCGTTTCGGGCCGGCCAGAGCGCGCGATGCGTCCGGGCGGG

CCGCCGAACGCGCGGCCGGGGCTGCAGCAGCAGCAGCCGGGCACGCCGGGCCGCGCGCGG

CGCCGGCCGGATCTCACGCTCCCGCTCCCGCAGCGCGACCTGACGTCGCTGGCCGTGCCG

CTTCCGCTGCCCCCGCCCCCGTCCTCGGCTCCGTCGTCCACGTCCTCGTCCGGGTCGGGC

GGCGCGTCGTCCATGCCCATGCCCATGTCCATGACCCCGCCCAACTCGGCCGGCTCCACG

CCGCCCGCGCCCCCGCCGCTCGGCGAGCTGGAGCGCGTGCGGCGCGTCGGGAGCGGCGCC

GGCGGGACGGTGTGGCTGGTGCGGCACGCGCCCACGGGCCGCGCCTACGCGCTCAAGGTG

CTCTACGGGCACCACGACGAGGCGGTCCGGCGGCAGATCACGCGCGAGATCGCCATCCTG

CGCACGGCCGAGCACCCGTCCATCGTGCGCTGCCACGGCATGTACGAGCAGGCCGGCGAG

CTGCAGATCCTGCTCGAGTACATGGACGGCGGGTCCCTGGACGGCCGCCGCATCGCGTCC

GAGTCGTTCCTCGCCGACGTGGCGCGGCAGGTGCTGTCGGGGATCGCCTACCTCCACCGG

CGCCACATCGTGCACCGCGACATCAAGCCGTCCAACCTGCTCATCGACTGCGCGCGGCGC

GTGAAGATCGCCGACTTCGGGGTGGGGCGCATCCTGAACCAGACCATGGACCCCTGCAAC

TCCTCCGTGGGCACCATCGCGTACATGAGCCCCGAGCGCATCAACACCGACCTCAACGAC

GGCAACTACAACGGCTACGCCGGCGACATCTGGAGCTTCGGCCTCAGCATCCTCGAGTTC

TACCTGGGCCGCTTCCCGCTCGGGGAGAACCTGGGGAAGCAGGGCGACTGGGCGGCGCTC

ATGTGCGCCATCTGCTACTCCGAGTCGCCGGCGGCGCCGCCCACCGCGTCCCCGGAGCTG

CGGAGCTTCATCAGCTGCTGCCTCCAGAAGAACCCGGCGAAGCGGCCGTCGGCGGCGCAG

CTGCTGCAGCACCGGTTCATCGCCTC

gccgccgcagcagcagccgcag

GCCCTCGCCGCCCCGCCGTGCTGACGGCTCGCCCGCCGTAACCAAGTTAAATTCGGAGGG

ATCATGGACGGACGGACGGACGGTGGCCGGCGGCGAGACGTGGGATCTAGGAGGAGGAGG

GGCGATCTTTCTTCTTCTTAGCCATTTTGGGTAGTGAAGCCACCACCACAAATTTACCCA

TCCATCCCTCTGTTGTTGCTGTCGCTCCTGAAATTAAGCTCAAGCTTTTCTTTAGTTTCT

CTGTTAAATGTTAATTAAATGTTGTCTCCTTCAAATGATGATTCTGAGGAGGATGATGAA

GATATAATATGGTCCGTTAAGAAACCAATTTCTTGTTC

**>TaMKK5(7AS) TRIAE_CS42_7AS_TGACv1_572257_AA1851770.1**

CCAAGAAGCAGCGGCGGCCCTTCCGTCCGACGCGCGCAGCACGCTTCCGGCTTGCTGTCC

TCTCCCACTCCCACCCTCGGTACAGGACAGGTCGCCTCCCCCAACAAACCCGACCCCCCA

TCAAACAGACGGCGCGCGCAACCAGACAAATCCCCGCTACCCGGCACCAGCAGCAGTCCC

CACTCGGGAGCCATGCGTCCGGCCGGCGGCAGCCTCCCGGCCCAGCCGGGCACGCCGGGC

CGCCCGCGCCGCCGCCCGGATCTCACCCTCCCGATGCCGCAGCGCCCGGACGTCTCCTCC

TCGCTCGCCGTCCCGCTCCCGCTCCCGCCCCCGACCGCCGCTCCCCCCACCGGCGCCGGC

CCCGCCCAGCCGCTCGTGGCGCCGCCCGCGGCCGCCCCGCCCCCGCCCCCGCTCCACGAG

CTGGAGCGCGTGCGCCGGGTCGGCAGCGGCGCGGGCGGCACCGTGTGGATGGTGCGGCAC

CGCGCCACGGGCCGCTGCTACGCGCTCAAGGTGCTCTACGGGAACCACGACGACGCGGTG

CGGCGCCAGATCGCGCGGGAGATCGCCATCCTGCGCACCGCCGAGCACCCGGCCGTCGTG

CGCTGCCACGGCATGTACGAGCGCGGCGGCGAGCTGCAGATCCTGCTCGAGTACATGGAC

GGCGGCTCCCTCGACGGCCGCCGCATCGCCGACGAGCCCTTCCTGGCGCACGTGGCGCGC

CAGGTGCTCTCCGGCATCGCGTACCTCCACCGCCGCCACATCGTGCACCGCGACATCAAG

CCCTCCAACCTGCTCATCGACTCGGCGCGCCGCGTCAAGATCGCCGACTTCGGGGTGGGC

CGCATCCTGAACCAGACCATGGACCCCTGCAACTCCTCCGTCGGCACCATCGCCTACATG

AGCCCCGAGCGCATCAACACCGACATCAACGACGGCGCCTACGACGGCTACGCCGGCGAC

ATCTGGAGCTTCGGCCTCAGCATCCTCGAGTTCTACCTCGGCAGGTTCCCCTTCGGCGAG

AACCTCGGCCGCCAGGGCGACTGGGCCGCGCTCATGGTCGCCATCTGCTACTCCGACCCG

CCCGAGCCGTCCCCCGCCACTGCCTCGCCCGAGTTCCGGGGCTTCATCGCCTGCTGCCTG

CAGAAGAACCCCGCCAACCGCCTCTCCGCCGCGCAGCTGCTGCAGCACCCCTTCGTCGCC

TTGCCGCAGCCGCAGCCGCTCGCCGCCCCGCCGTCATCGTGACCGCCGCGACCTCCCAAC

ACGGATCTCGATCCACTTCTTGCACCACCGCGCTTCCAAATCCAGCACTGGTCAGGATCG

ATGGAACTGGGCTGCCGGGGGAATCTTGGAAGGTTCTCCCTGACCATTTTGGGTATTCTT

CTCGTCCACCATCGTCCTCCCTCATCAAATCATCCATTGTTGCTGCTGCTGCTGCTCCAG

TTAAGGTTCCCTTTCCCTCTCCTGTGAATCTGTGATGTTCGTTGCTACTTCCTGTTACGG

CCTTGTAATAGTAGCAAAAGCTGTGTGCACAAAAAGGATTTTGTACCGCAAGATTGGTCG

AATATAGACACGATATCCATTCTGCCATTGCCTCTCCAACTGCTGCAAACGAAATTCCGT

TGGAAATTTCATCTGTACATGTAGCTCATCACATGATGATATGGCCGTCCCCCATCTTGC

CAGGGGCATCTTCCATTTATTTTATCTGTGTTTGCTTATTATACTAGTTGCAAGTCGCCG

TGGCTCGATGGAATTATTGGATTGGATGATTCTATATTTGCTGGTTGGATAGAGGGGAAT

CTCGGAATAGGAATCGGACCAGCTTTGTTTTGTTTCTTTCTTTCAGTGTTGTAAAGTGAT

ATGAGGGAAAGGTGTTCCTGAACCTCCTCTGCCTTTCTTGTTTTCAATTCTGGTTTGCCT

TTTCTTCGTCCGGCCAAAGATGATTTTTGTTTTGGCTATTGGCATGCACATCATGGGTTT

CAATTCATGTTCACTTTTGTTCCACTTGGTGAAAATGGACGTCAAGAATGAATTTCCTTT

GGAGTCAATGATCCTGGCCAATTGACCTTTGTGATTAGTTTAGTTAGAGTGAGTATAAAC

CTTTCTGATTAGT

**>TaMKK5(7BS) TRIAE_CS42_7BS_TGACv1_592906_AA1946090.1**

CAGGACAGGTCGTCCACCCCAACAAACCCGACCCCCCATCAAACAGACGGCGCGCAACCA

GACAAATCCCCGCTAGGCGGCAGCGCCAGCGCCACTGCCAACCCCCACCCCCACGCGAGC

CATGCGTCCGGCCGGCATCAGCCTCCCGGCCCAGCCGGGCACGCCGGGCCGCCCGCGCCG

CCGCCCGGATCTCACCCTCCCGATGCCGCAGCGCCCGGACGTCTCCTCCTCCCTCGCCGT

CCCGCTCCCGCTCCCGCCCCCGACCGCCGCTCCCCCCGGCGCCGCCCAGCCGCTCGTGGC

CCCGCCCGCGGCGACCCCGCCCCCGCCCCCGCTCCACGAGCTCGAGCGCGTGCGCCGCGT

CGGCAGCGGCGCGGGCGGGACGGTGTGGATGGTGCGGCACCGCGCCACGGGCCGCTGCTA

CGCGCTCAAGGTGCTCTACGGGAACCACGACGACGCGGTGCGGCGGCAGATCGCGCGGGA

GATCGCCATCCTGCGCACCGCCGAGCACCCGGCGGTGGTGCGCTGCCACGGCATGTACGA

GCGCGGCGGCGAGCTGCAGATCCTGCTCGAGTACATGGACGGCGGCTCCCTCGACGGCCG

CCGCATCGCCGACGAGCCCTTCCTCGCCCACGTCGCCCGCCAGGTGCTCTCCGGGATCGC

ATACCTCCACCGGCGCCACATCGTGCACCGCGACATCAAGCCCTCCAACCTGCTCATCGA

CTCGGCGCGCCGCGTCAAGATCGCCGACTTCGGGGTGGGGCGGATCCTGAACCAGACCAT

GGACCCCTGCAACTCCTCCGTCGGCACCATCGCCTACATGAGCCCCGAGCGCATCAACAC

CGACATCAACGACGGCGCCTACGACGGCTACGCCGGCGACATCTGGAGCTTCGGCCTCAG

CATCCTCGAGTTCTACCTCGGCAGGTTCCCCTTCGGCGAGAACCTCGGCCGGCAGGGCGA

CTGGGCCGCGCTCATGGTCGCCATCTGCTACTCGGACCCGCCCGAGCCGTCGCCCGCCAC

CGCCTCGCCAGAGTTCCGGGGCTTCATCGCCTGCTGCCTGCAGAAGAACCCGGCCAACCG

CCTCTCCGCCGCGCAGCTGCTGCAGCACCCCTTCGTCGCGTTGCCGCAGCCGCAGCCGCT

CGCCGCCCCGCCGTCATGACCGCCGCGACCTCCCAATACGGATCTCGATCCACTTCTTGC

ACCACCGCACTCTCAAATCTAGCATATCTGGTGGAGGGAGGATCGATGGAAACTGAGCTG

CCGGGGGAATCTTGGAAGGTTCTCCCGGACCATTTCGGGTATTCTTCTCGTCCACCATCG

TCTCATCAAATCATCCATCCATTGTTGTCGCTGCTGCTGCTGTTTTAGTTAAGGTTTCCT

TTTCCTCCCCTGTTGCTGTGATGTTCGTTGCTACTTCCTGTTACGGCCTTGTAATAGTAG

CAAAAGCTGTGTGCACAAAAAGGATTTTGTACCGCAAGATTGGTCGAATATAGACACGAT

ATCCATTCCGCCATTGCCTCCCACCGCTGCAAAGGAAATTTCATCTGTACATGTAGCAAA

CAAGCAGTGCCATCACATGATGATATGGCTCCCATCTTGCCAGGGACATCTTCCATTTAT

TTTATCTGTGTTTGCTTATTATACTAGTTGCAAGTCGCCGTGGCTCGATGGAATTATTGG

ATTGGATTCTAAAAATGCTGGTTGGATAGAGGGGAATCTCGAAATAGGAATCGAAGCAAC

TTTGTTTTGTTTCTTTCAGCGTTGTAAAGTAATGAGGGAAAGGTGTTTCCTGAACCTCCT

CCTCCTTTTCTGTATCCTGTTCTTGTTTGCCTTTTCTTCACTCAGCTAAAGATGGTTTTT

GTTTTGGCAACTGGCATGCGCATCAGGATTTCAATTCATGTTTACTTTTGTTCCACTTGA

TGAAAATGGACGTCTGCAATGAATTTCCTTTGTGATT

**>TaMKK5(7DS) TRIAE_CS42_7DS_TGACv1_623332_AA2052160.1.X (based on AA2052160.1)**

CACCCTCGGAGTCAGTACAGGTCGCCTCCCCCAACAAACCCGATCCCTCATCAAACAGAC

GGCGCGCAACCAGACAAATCCCCGTTAGCCGGCACCAGCAGCCGCGCCACTGCCCACCCC

CACCCCCACCCCCACGCGAGCCATGCGTCCGGCCGGCGGCGGCAGCAGCCTCCCGGCCCA

GCCTGGGCCGGGCACGCCGGGCCGCCCGCGCCGCCGCCCGGATCTCACCCTCCCGATGCC

GCAGCGCCCGGACGTCTCCTCCTCCCTCGCCGTCCCGCTCCCGCTCCCTCCCCCGACCGC

CGCTCCCCCCGGCGCCGGCCCCGCCCAGCCGCTCGTGGCCCCGCCCGCGCCCCCGCCCCC

GCTCCACGAGCTGGAGCGCGTGCGCCGCGTGGGCAGCGGCGCGGGCGGCACCGTGTGGAT

GGTGCGGCACCGCGCCACGGGCCGGTGCTACGCGCTCAAGGTGCTCTACGGGAACCACGA

CGACACGGTGCGGCGGCAGATCGCGCGGGAGATCGCCATCCTGCGCACCGCCGAGCACCC

TGCCGTCGTCCGCTGCCACGGCATGTATGAGCGCGGCGGCGAGCTGCAGATCCTGCTCGA

GTACATGGACGGCGGCTCCCTCGACGGCCGCCGCATCGCCGACGAGCCCTTCCTCGCCCA

CGTGGCCCGGCAGGTGCTCTCGGGCATCGCGTACCTCCACCGGCGCCACATCGTGCACCG

CGACATCAAGCCCTCCAACCTGCTCATCGACTCGTCGCGCCGCGTCAAGATCGCCGACTT

CGGGGTGGGGCGGATCCTGAACCAGACCATGGACCCCTGCAACTCCTCCGTCGGCACCAT

CGCCTACATGAGCCCCGAGCGC

atcaacaccgacatcaacgac

GGCGCCTACGACGGCTACGCCGGCGACATCTGGAGCTTCGGCCTCAGCATCCTCGAGTTC

TACCTCGGCAGGTTCCCCTTCGGCGAGAACCTCGGCCGGCAGGGCGACTGGGCCGCGCTC

ATGGTCGCCATCTGCTACTCGGACCCGCCCGAGCCGTCGCCCGCCACCGCCTCGCCCGAG

TTCCGGGGATTCATCGCCTGCTGCCTGCAGAAGAACCCGGCCAACCGCCTCTCCGCCGCG

CAGCTGCTGCAGCACCCCTTCGTCGCCTTGCCGCAGCCGCAGCCGCTCGCCGCCCCGCCG

TCATCATGA

CCGCCGCGACCTCCCAACACGGATCTCGATCCACTTCTTGCACCACCGCGC

TTCCAAATCCAGCATCAGGTGGAGGAACTGAGCTGCCGGGGGAATCCTGGAAGGTTCTCC

CTGACCATTTCGGGTATTCTTCTCGTCCACCATCGTCTCATCAAATCATCCATCCATTGT

TGTCGCTGCTGCTGCTGCTGCTCCAGTTAAGGTTCCCTTTTACTCTCCCGTTCCTGTGAT

GTTCGTTGCTACTTCCTGTTACGGCCTTGTAATAGCAAAAGCTGTGTGCACAAAAAGGAT

TTTGTACCGCAAGATTGGTCGAATATAGACACGATATCCATTCTGCCATTGCCTCCCGCT

GCTGCAAACGAAATCCCGTTAAGAAATTTCATCTGTACATGTAGCACAGAAGCAGTGCCA

TCATATGATGATATGCTCCCCATCTTGCCAGGGACATGTTCCATTTATTTTATCTGTGTT

TGCTTATTATACTACTTGCAAGTCGCCGTGGCTCGATGGAATTATTGGATTGGATTCTAT

AATCTACATATGCTGGTTGGATAGAGGGGAATCTTGAAATAGGAATCGGACCAACTTTGT

TTTGTT

**>TaMKK6(4AL) TRIAE_CS42_4AL_TGACv1_290485_AA0985860.1**

ACACACAGCTGAGTCCATCACAGCCCAGTCGGCTTCACTGAGCGCGACCACTGTCTGACT

CTGTACTGAGTCTACTGACCAGGGCAGTCCCCCCGCCCCTCCCCTCCCCTCCCCTCCCTC

CGCTAGCCGCCACGTCCCCGGCCGACCCGACCACCCCTCGCCGTCGGCCGGACCGCATCT

CCCCCCCTCACCCCCACCTCCAGATCCGCGCGCTCCCCTAGCCTCCCCCCGCCCGCCGCC

GCCGGGAACTTTCTGCCGAGCTCGATCCATCCAGTGGCCGGCTACAGCGACCGCCCGCCG

CGCGGATCGATCCAGCGACCAACCTGGAGCACGGCTGCTAGCCGTCCCCTCACCAGTCAA

CCCAAGTCGCACCACGTGCCACCATTTCGACCAGCTCCGCCGCTGCTATTTTTCTCCCTA

TATTCCTCCCCTCACACCGACGGCGCCCCGGCCGCTCCTCCCCGTCCGCCGCGCCCCCG

gtgagcgagcatatccggcctgatcgaaagtcagtatctttatgccgactcgtcggatct

atgtctaacctgcttccggctttgtccag

CTCCGCGTCCATCTCCCGCCCGAGAAACCCTAGCCAGAGGAAGGAGGACACCATGAGGGG

GAAGAAGCCGCTCAAGGAGCTCACACTCTCCGTGCCGGCGCAGGAGACGCCCGTCGATAA

GTTCCT

gtaggtctcccccattcctcaccctaaatttcatgttttttttccctctcccctttcccc

cactgaagccgtcaaaagtttgagctttatttctacgtaataatgagagatttttgtggg

gcctgggtagacttgttttctgtgtcatctttgatttgatctgttggttttgttgcgggt

tgtgatggagctgggtagccagtagtagtctgaagcatagatttactttgatggcattca

ttaagtgtagtgcttgatttggaattggtacggtcggagctgctgagctgactctgcatt

ttgtatgtcacgaaagtattcaagtccagtgattaatttgaactcaatatgggttgagtt

gctgagctgcttgtggtctttcag

GACGGCGAGCGGTACGTTCAAGGACGGTGAACTGCGACTTAATCAAAGAGGTTTGCAGCT

TATCTCCGAGGAAAACGGAGATGAACAT

gtgagttcttggctcaatattttgtaatgactaactctgttgtttgttaatctgcccagt

tgtgtgttcctgttgtattgtactcgtattcattaacaagggtggtttatgtgttgcgtg

cgtcactgattcagtgcactcagagggtctactgcattcaataaaccagggggaaccaac

aaaacatttgataagatttgtgattgaatagtatatgtactactatataaacgtacacgt

cgccacacaaagtacaatataacctgaatacagtaagacataccgaaaaaatagtcatta

ctgaacctggaactcacgtagtcatcgctcttttcctcggagataagccacgagcctctt

tgatcaagtctaaaagaccaccagaaaccaaagacaaccatttaattcacacattacttc

ttgtttcttcaagtatctcaaaaacatttctagacacaaaaaagtagtgcagtaaaatat

caagtcctcagtagcttcaacaggaaaaaggaaaacaaaacatgctatataaataaaacg

ggcagtttaagccaacgagtcaagaaaacctacatggtttactgacaggtgtcgctccgg

cagaatgagctggagctccttgagcggttccttcccccccatggtctcttcctatggtta

ggatttcttgaatgggagatgaatatggaactggctgccagagcctgaaaacaagtcgag

gagtaggtaggagaccaggtgaactgctatgaagatatggagttcggatctgcccagcgg

aggtgcgcaagagggagagggagagagagagtgctcgcatttgttggtgacttaggcagt

cagctgtcagcctaagtcgcagcatgtgttactttcatcgaccaaagttacaaatttatg

gaccgcagggtcagaggaacccttctagttcaaactttgtagtcgttttatccaaaatca

taacatatttgcagatttctcaataattgtttcatgattattttttaatgcctaggattg

aaattatgtactctgctgctcgctgctgcttattagatgccaccaacagaagttttatag

ttagtgggaatagggtatgttgtctaaaggttactgtagaatcccaactaggatatattg

ttactcagtttgtgcttgttatcatttggataaggatcaagttgtttctgaattctgagt

acatgttgatttgtttattttcctcag

CAATCAACAAAAATGAAGGTGGAAGATGTGCAGTTATCAATGGATGACCTCGAGATGATT

CAGGTCATTGGTAAGGGAAGTGGTGGTGTTGTCCAACTAGTGCGGCACAAGTGGGTGGGC

ACATTTTATGCCTTGAAG

gtaacagaacactaaatttatgtatgcctaagttggatttttattgttactgttagagca

ttccggtaaactgctactccctccgtcccataatataagagcgtttttgacactagtgta

gtgtcaaaagcgctcttatattatgggacggagggagtagttttttctgttatactgttt

atttttttattcaagtttgttaacatgaaaaataatctattcatatctgtttgacgtgtc

acgacaattggattattctacctctgaagattagttttgtttagtcagcttgtgatggca

tgacagtatattcagttagatgcacgcttacgcacactattaaaatgagttatagcaaaa

ggactgatatttggcacacgtgtgccatataagcaaaactgccacacctctacttctttc

attttaaagtaccacacgatcccccgtcctttgacctcgcctcctctgaaatgaccccgg

aggctcgtccgagaaacctgtttctcctgcacatctcgcgcgcccacccccgagaaaact

gtcacttcttcacgtctaccacatgatttctcctcaggacaaagttaccatgacattcat

atgcaagttatcaggcctgtgttgcgtaacttaccatactatttacatagaacgtaccag

gtactatgcttcaacaactacaccccttcaaagtcaccactgtattttatacacaagtta

gaaggttttcgacgtgtaaaataccgtggtacttacacataagttatcagggtatatgta

catcaacctccccctggtcaaagttaccatgggggattgtacatgagttaccgggtctat

agtttgtatattatcatgttacttgcacctaaaaacctgggtgtgtttcggtagcatttc

ccataggtcaaaaattaccacgatgtttttgcgtaacttaccacgcctatagcacgtgtg

cccctcatgacactaaacattatgtcactttctcgtggcacgtcatgtgttgtgttcatc

caagaggcccacgcgcgaggcgagtgtatgtctttaacaacttatttttctttggtaaaa

aaagttaccatcatgtttatattgcaagttatcggttatgcggtgcttatattatcatgt

tatttacatgaaaattattggggatgttttgaacaactttttcctgggagaaaaagttat

catggtgattctaggtaatttatcaagcccgtagtgcttaaaatatcatgctatttacac

aaaatttatcaggtgtatgtttcagcaaattctcccccacgggtcagaaacttatcatgg

tgtttagttatcgcagtgcatatattttcatgctatttacacataaaaatgccagaggag

ggaggtatactaccgtgctatttacacagaagacatcggagtatcttttcaaaaaaaaat

ccctacgatcaaagttaccatggtgtttctacctcagttatcagatgtgcgctgcgtata

ttaccatctatttacacataaattatcgggtatcttttcacatttatcttccccaatggt

taaagttaccacgatgtttgtatctaaattatcaggtctatagcgtgaatgttatcgtgc

tatttacagaaattaccggggggcgggttcaacaaatttattcccaatgatcaaagttac

cacgatgcttgaaacaaaaaaaattcagtgctaacatattaaaggcaaaaacatgtcaaa

aacagtatttcccttagcttgttcaacaatcagtgtcataggtgaggtggttagttccct

ttgttgattacctgtagaccagagatcaaatcctagtccaagcattatttttgcccgaaa

attgggaagtcgcgtggggccataattgacgattacgaaaattaggaagtcacgagcggg

tgtgcccgcgggaaaggaaagagatcgggagacgtgcaggaaaggaaagaaatcgggagg

gaggtcggggaggacgtgcgggaaaggaagaagatcgggaggcgtgtggcagtttcgcaa

tctggcacacggttgccatatacatatttcgatagcaaaaacatctagtactggcctgca

aaatctgtatggaacactgatttgagaactgacgctaatgcatgagaatacatcaggtaa

caatatatgggcgattaaatgttttcaagcgttgttcctgatctttgtactgccgtggca

tttctccatatgatctatcagttaattgactattgcaatttcttcatag

GGCATTCAAATGAACATTCAGGAGTCAGTTCGCAAACAAATAGTACAGGAGCTCAAAATA

AATCAAGCAACACAGAGCCCACATATAGTCCTTTGCCATCAATCTTTTTACCACAATGGT

GTAATATATCTTGTTCTGGAATATATGGATCGTGGATCTCTTGCAGACATAATTAAACAA

GTTAAAACCATTCTGGAGCCATACCTCGCAGTACTTTGCAAGCAG

gtatgtatatgcttggcctattgtttctgctttgacttttgtattcagaatattcttaaa

attctgtcacatatgttggaagttggagctacaatctggcacatctaacatcagtagtgt

ctgaataaatcag

GTTTTGGAGGGATTGTTATATCTTCATCATGAAAGGCATGTGATTCACAGAGACATAAAG

CCTTCTAACTTGTTAGTCAACCATAAAGGTGAAGTAAAGATTACTGATTTTGGGGTGAGT

GCAGTGCTAGCAAGTTCGATTGGTCAGCGTGATACATTTGTTGGAACCTACAACTATATG

GCG

gtatgtaaaaagaccagttttgtcttctgactgcggcttatgctcataacggttatctgg

ggtgcataaattttccttcatagtttcttcctttcttccttgtgcag

CCTGAGCGGATTAGTGGCAGCTCCTATGACTACAAGAGTGATGTATGGAGTTTGGGCTTG

GTAATACTTGAATGCGCCATTGGTCGCTTCCCTTATACTCCTCCAGAGGGAGAAGGTTGG

TTAAGCTTCTATGAACTATTGGAAGCAATTGTTGACCAGCCACCACCTTCTGCACCTGCA

GATCAGTTCTCTCCAGAATTCTGCTCATTTATCTCTTCCTG

gtatgctaacattattctcaggaattacacatgaaattagttcagtcactatatttctat

tttgtcctcttgcttagtatgcttgaatgaacttcttgcccttttttctaaagttctgat

ttccttcgcgtgtccttcttgttgttttaatagttatctgctgtctcgtctcacagctca

tggactttgtccactcaattattatccccccctttgataaaatgtgttgtctattcaagt

attactttcccctttgaatgaacttcttgccctgttttctaaagttctgatttcctccac

gtgtccttcccgtaaatattttaaattgttggttaggccaacttgttttaatagttatct

gctttctcatctcacagctcatggactttgtccactcaattattattcccccctttaaaa

aaaatgtgtttgatctatttaagtattactttcccctttgaacaagcgtggtcttaatag

aactgttttagtgtgataatttgggcttcattaattctggagaataactgtttaaactgc

ggggtgtttctcagataagaattagtgaaatcctcagatgcccaaattttataatggtgc

tttttcaatagcaaacggatgaatcaatgtttcttaatgaatattacatttcagtacagc

ccaagaaatcccagccctcccaaacaaatcaacttgcgcccccggacaacaactactcag

ggcaacttgccaatgaactgtttatcaaaccctgtttaagtagcttgttggcccttgaaa

cttcagtttagctaatctggagtttgagttatcaatcatgtaacaaccctgattgtcagt

aatgtcaaaatgtacacattgatttaagttagctatgtcaatatctgatctaattgttgc

cttgactaacaaaaccttgacagtggggttgataaaccagttaacaatccggcatcttat

cttaccaatggattaaggcatgccaaaaaaatatggaaaataaccctcctactatacagg

gtgtatgaagttgaccaaatgatgttatactatgaatcctttttatgttgtgtgattcaa

aaggaagaggaaggaacaccagagaacaataggcatggaaggcatcacaaaaactcaaac

caaagcctcctctagtgttagacgaggaacaagggctttagccttccatcatgttcccat

gcccactgctcttaggtcatcttttcttcttcttcatgtctcgcaatggacatctcatac

catcttaatatatccacatatacgttcagtttttgtgctaaaagattcagcacacaaaaa

ggaagaggaaggagcgcttaagaacgataggaatgagaggcattgggaaataccaaacgg

gatttccacctcaacacaagaggtgaagaaccgataacttaggctttcctctatgctctc

atccccattgctcttaggccctccctttcttcctcttcctagctcgcttttgagatttca

catcgaatatgtgcagacatatagttggtttctaaactaaagcaaccaagcaagccaaaa

aaggaagaggaaggagtgctttagaacaataggaatgggaggcattgggaaacaccaaac

tagattttcacctctaacactagagatgaaaaccgagcaatttaggctttcctccatgtt

cccattcccactactctaaggccctctattcttcctcttccgggctcgcattcgaggtct

catgttatctaaacatgtggacacatgtctatggtttctccatcagagctatcacgtaag

ccaaaaggaacaggaagaagtgcacaagaacggtagaaatgggaggcatcaggaaaggat

ccatgccaaatatttgcacctccgatactacaggtgcaaacaaacaagcattgctttcct

ccatgttcccattcccattgctcttgagctctctcttcctcttcccggcttactttcagt

gcgtacaaatctagagacaaggctctcgaaggacaagcttgaagtgttggacaaagtgtt

tcagctttgaggtataaataattggtggtgcaccatatgcagaaccttagaggagaaagc

aatgaagacacccagtgcatggaggcaccattctacagtcaatgataatggggtcacata

atcaccagccctgtcctattgtcctctactcttaattaagggcatgtgcgtaaagatctc

ttttgctcacctttattacccatatccgaaactggtacataatatctgtaccagccttct

tttagaacaatatctgtatcatcctgtgcatttatagtgtcaaaatacgcagcaataaga

agttataagctaaaaataatatgaaattagacatattaaaccctcgaaaaattacaaaac

agtgggcaaagaaccaccaacgtgacaattaattggaacacaaggtactttggacatgag

ctgcgttattgcagatgacagaatgtgattacaacatgattagagggatctcatatctgc

ataaccaaaatgtctatagaccattttatcaagaaaaaaaagtttagtcctattattttt

taagtaatttagtatccaaattctgcaggctgtacacaactacatgtcatgggaaaatat

aggaaaaacatatctccaaaggaactgtcacccttgatccctaggataaaacacatgaag

gtatagtgatcacatattcagtctaaactcattatacacatagtattttaagaacaaggt

ttggacacaaaggaagaacaaggtcccgaagagagtatggtacttcttccacccatcgaa

gtacttcccagttcaatcttgtagctctgccaccatctctccaaggattaaatcgcccag

gttgagatgcaaacagcctgcaaggttccagagtggggaaataagccttatcccgcacat

ctaaacaatatataaaaaacaacaccaatttatgatgaagtttacaaaactgcaaattcc

attggagttcacaattttcgtagctgcagataaatccattagtttgctatatcccatgtt

tgaactactgaggaaataataaaaataatctgaatgaacatagtgatattgtctcatgtt

ttgtgtgccaattacgatctttcgtactccgtttctgctcgtagtcttagctagagtggt

cagagtggtgtgtcattgatctgattaactgatagagtaatgcacttacatgcatttacc

atgcgactctgtctaaactcctcagtcctcacgtcatgcttgacctgcatcaactggctg

atatgtaccattcaaatactattacttcagaacttcctttgatttagtgtgataatttgg

gcttcattaattctggagaataactgtttaaactgtgcagtgttcttcagataagaatta

gtgaaagcctcatatgtccagattttataatggtgtgttttgcaacagcaaacggacgaa

tcaatatttttttttaaaccataatattacatttcagtacagcccaagaaattccagccc

tcccaaacagatcgacttgcgcttccagacaacagctactcagggccacttgccactgaa

actacctgattatcaaatcctgtttaagtagcttgtgtgcccttgaaacttaaagcagtt

tagctaatctggcgtttgagttattaatcatgtaacaaccctaattgtcagtttaatgtc

aaaatgtacttactgatttaagtttgctatgtcaatatctgatctaattgttgccttgat

tgacaaaaccttgactttgaggttgtagtataaaccagctaacaatccggcatcttatct

aaccgatggattaagtcatgccagaaaaagatggaaaataaccctcctactatgcagggt

gtatgaacttgaccaaatcatgttatactatgaatcctttttatgttgtgtatccaaaag

gaagaggaaggggcaccaaagaacaataggcatggaaggcatcagaaaaactcaaaccaa

agcctcctctagtgttagacggggaacaagggctttagccttccatcatgttcccatgcc

cactgctcttaagtcaccttttcttcctcttcatgtctcgcaatagacatctcatactat

ctcaatatatccacatatacgttcagtttttctgctaaaagattcagcaaacaaaaagga

agaggaaggagcgcttaagaacgataggaatgagaggcatcgggaaaataccaaacggga

tttccacctcaacacaagaggtgaagaaccgataacttaggctttcctctatgctctcat

ccccattgctcttaggccctccctttcttcctcttcctagctcgcttttgagatttcaca

tcgaatatgtgcaaacatatagttggtttctaaactaaagcaaccaagcaagccaaaaaa

ggaagaggaaggagtgctttagaacaataggaatgggaggcattgggaaacaccaaacta

gatttttcacctccaacactagaggtgaaaaccgagcaatttaggctttcctccatgttc

ccattcccactactctaaggccctctattcttcctcttccgggctcgcattcgaggtctc

atgttatctaaacatgtggacacatgtctatggtttctccatcagagctatcatgtaagc

caaaaggaacaggaagaagtgcacaagaacggtaggaacgggaggcatcaggaaaggatc

catgccaaatatttgcacctccgatactacaggtgcaaacaaacaagcattgctttcctc

catgttcccattcccattgctcttgagctctctcttcctcttctgggcttactttcagtg

cgtacaaatctagcgagacaaggctctcgaaggacaagcttgaattgttggacaaagtgt

ttcagctttgaggtataaataattggtggtgcaccatatgcagaaccttagaggagaaag

caatgaagacacccggtgcatggaggcaccattctacagtcaatgataatggggtcatat

aatcaccggtcctgtcctattgtcctctactcttaattaagggcatgtgcgtaaagatct

cttttgctcacctttattacccatatccgaaactggtacataatatctgtaccagccttc

ttttagaacatctgtatcagcctgtgcatttatagtgtcaatatatgtagcaataagaag

ttataagctaaaaataatatgaaattagacaaattaaacccttgaaaaattacataacag

tgggcaaagaaccaccaatgtgacaattaattggaacacaaggtactttggacatgagct

gcattatttgcagatgacagaatgtgattacaacatgattagagggatctcatatctgca

aaaccaaaatgtctgtagactattttataaagaaaaaaagtttagtcctattatttttta

agtaatttagtatccaaattctgcaggctgcacacaactacatggcatgagaaaatatag

gaaaaacatatctccaaagaaaccatcacccttgatccctaggaggaaaacacatgaagg

tatagtgatcacatattcagtctaaactcatttatacacagagtattttaagaataagga

tcggacacaaaggaagaacaaggtcttgaagagagcatggtacttcttccacccatcaaa

gtacttcccagttcaaccttgtagctcggccaccatccctccaaggattaaatcgcccag

gttgagatgcaaacagactgcaaggttccagagtggggaaataagccttatcctgcacat

taaacaatatataaaaaacaacaccaatttatgatgaattgtacaaaactgcaaattcca

tcggagttcacaattttcgaagctgcagatatatcctgttgcggtaatggttgtttcttg

ctagtctgtgtattttatccattagtttgctatatcccatgtgtgaactatttaggaaat

gataaaaataatctgaatgaacatattgatattgcttcatgttttgtgtgccaattacca

tcttttgcactccgatttgtttcatgttttgtgtgccaattaccatcttttgcactccga

tttctgctcgtagtcttagctagagtggtcagagtggtgtgtcattgatgtgattaactg

ataagagtaatacgcttgcatgcatttaccatgcgactctgtctaaactcctcagtcctc

acataatgcttgacctgcatcaattggctgatatctaccattcaaatactattacttcag

aacttcctttgatttagtgtgataatttgggcttcattaattctggagaataactgttta

aactgtgcaatgttcttcagataagaattagtgaaatcctcagatgtccagattttataa

tggtgttttttgcaacagcaaacggacgaatcaatgtttttttcatgatattacatttca

gtgcatcccaagaaattccagccctcacaaatggatcgactttcggcaacttgccactga

aactacctgtttaccaaaccctgtttaagtagcttgtgggcccttgaaacttcaagcagt

ttagctaatctggtgtttgagttattaatcatgtaacaaccctaattttcagtttaatgt

caaaatctacttcctgatttaagttagctatgtcaatatctgatctaattgttgccttga

ttgacaaacccttgactttgaggctgtagtataaaccagctaacaatccggcatcttatc

taaccgatggattaaggcatgccaaaaaaaacatggaaaataaccctcctactatgcagg

gtgtatgaacttgaccaaatcagttatactatgaatcctttttaggttgtgtatcccatg

aagaggaaggggcaccaaagaacaataggcatggaaggcatcacaaaaactcaaaccaaa

gcctcctctagtgttagacgaggaacaagggctttagccttccatcatgttcccatgccc

actgctcttaggtcatcttttcttcttcttcatgtctcgcaatggacatctcataccatc

ttaatatatccacatatacgttcagtttttctgctaaaagattcagcacacaaaaaggaa

gaggaaggagcgcttaagaacgataggaatgagaggcgtcgggaaaataccaaacgggat

ttccacctcagcacaagaggtgaagaaccgataacttaggctttcctctatgttctcatc

cccattgctcttaggccctctctttcttcctcttcctagctcgcttttgagatttcacat

cgaatatatgcaaacgaatagttggtttctaagctaaagcaatcaagcaagccaaaaagg

aagaggaaggagtgctttagaacaataggaatgggaggcattgggaaacaccaaactaaa

ttctcacctccaacactggaggtgaaaacggagcaagttaggctttcctccatgttccca

ttcccactactctaaggccctctattcttcctcttctgggcttgccattgaggtctcatg

ttatctaaacatgtggacacatgtctatggtttctccatcagagctattgcgtaagccaa

aaggaagaggaagaagtgcacaagaacggtaggaatgggaggcatcaggaaaggatccat

gcccaatatttgcacctccaacactgcaggtgcaaacaaacaagcagggctttcctccat

gttcccattcccagtgctcttgagctcactcttcctcttcccggcttactttcagtgcct

acaaatatagcgatacaaaatctctcgaagtacaagcttgaattgctggacaaagtgctt

gagctttgaggtataaataatgggtggtgcaccgtatgcagagaaagtaattaagacacc

cggtgcatggcggcaacattctacagtcaatgataatgggaccacataatcacctgccct

gtccttttgtcctctactcttaatttaaggcatgtgcgtaaagatttcctccgcttacct

ttattacgcatatccgaaactggtacgtaatatctgtaccgatctgtatcagcctgtgta

tttgtagtgtcaaaatatgtagcaataagaagttgtgagctaaaagtaatatgagattga

acaaattaaaccctccaagtaaacttggaaaaaatacaatacagtgggggcaaagaacca

ccaaagtgacaattaattggaaaaaaaaaggtacttcggacatcggctgcagtattgcag

atgacagaatgtgattaaaacatgataagagagatctcatatctccaaggattaaatcac

ccaggcagagatgcaaacagccagcaaggttccatagtggggaaataaaccttatcctgc

acatctaaacaatatatagaagacaacaccattttatgatcaagtgtacaaattcaatca

gagttcgcaattttcgaagctgcagatatatcttgttgcagtcatgcttgtttcttgcta

gcctgcgtatttatccattagttttctatcccatgtgtgaactctttaggaattgatgat

aaaaataatctgaatgcacatatcgttacatgttttgtgtgccaattaccatcttttgca

ctccgatttctgctcgtactcagagtggtgtgtcattgatgtgattaactaataccgtaa

tacacttacatgtatttaccatgcgactctgtctaaactccccagtcctcacatcatgct

tgacctgcagcaattggctcatatctaccactcaaatactattcttttttcaagcaacca

gcaagagctctgcttctcatgtagaagagggttgaccagtttataaggaaacccggctga

aaaccgaccattcaaatactattactttcgaacttcctttgatttacagtcaactctgtt

tattggctatatgttttattgctctatattttagttagtgagtaaattcataatgagatg

tgcgcatcctgaaggcaaccgatgatttctgctagcttagcttttgaaatttgtgccatc

tgtgtagtatatatactgtagtgttatttcatattgctatcttgctttgcag

CATACAAAAGGACCCCGCCGAGCGGATGTCTGCTTCAGAACTCTTG

gtgagcattcccttccacttgacaacttcttgtagcatgttcaactaaccgtggcaccat

tgattctgccgactagtgtgtgtgtgtgtgtgtgctgaatcgtcgttattaaactaacat

gttcaactaacatgttcgatgttattgtcgcag

AACCACGCCTTCATCAAGAAGTTTGAGGACAAGGACCTAGACCTCCGGATCCTTGTCGAG

AGCCTGGAGCAGCCCATGAACGTGCCCGAGTGAGGCCACCGGTCGGAGAGGTGAGGCTTT

GCAGCGTGTTTCTTCCGGGGCTGCCCTCCCGAACGGCAATCGTGTATTGCTGTGAACCTG

ATGAATGAGTGACCCCCCACCCCTGTATGTTGATGTTTATGTTGGAAAGATTCCCAGAGG

ATTCTCATGGACTATTAATGTATGGTTAATGGATGAAGGCCCTTTTGTTGCGTGTCTTGC

TATGAATGAATGGATCTGGTGTGGC

**>TaMKK6(4BL) TRIAE_CS42_4BL_TGACv1_320624_AA1044950.2**

ATTTAGGGCTTTGAAAGCTACGCTGGGCCAGGCCACACACAGCTGAGTCCATCATAATAG

CCCAGTGCCCCAGTCGGCTTCACTGAGCGCGACCACTGTCTGACTCTGTACTGAGTCTAC

TGACCAGGGCAGCCCCTCCTCCCTCCCCTCCCCTCCGTCCGCTCGCCGCCACGTCCCCGG

CCGACCCCTCGCCGCCGGACCGCATCTCCTCCCCTCACCTCCCACCTCCAGATCCGCGCG

CTCCCCGCCCGCCGCCGCCGGGAACTTTCCTCCGAGCTTGATCCATCCAGTGGCCGGCGA

CAGCGACCGACCGCCGCGCGGATCGATCCAGCGACCAACCTGGAGCACGGCTGCTAGCCG

TCCCCTCCCAGTCAACCCAAGTCGCACCACGTGCCACCATTTTGACCAGCTCCGCCGCTG

CTATTTTTCTCCCTAAATTCCTCCCCCGGCCTTCCCTCACACCGACGGCGCCCCGGCCGC

TCCTCTCCGTCCGTCGTGCCTCCG

gtgagcgtccgtatccggcctggtccaaagtccgtatttttatgccgactcgtccgatct

ccatctaacctgcttccggctttgtccag

CTCCGCGTCCATCTCCCGCCCCAGAAACCCTAGCCAGAGGGAGGAGGACACCATGAGGGG

GAAGAAGCCGCTCAAGGAGCTCACCCTCTCCGTGCCTGCGCAGGAGACGCCCGTCGATAA

GTTTCT

gtatgtctcccccattccttggcctaaatttcatgtttttccctctcttttccccctact

aaagctatcacaggtttgggctttatgtctaataaatgagagattttttgggtgcctggg

tagatttttgtggtttatttgtggattctgatggggctgggtagccggtactgggttgct

aaagcatagatttactttgaaggcattaagtgtagtgcttgatttgaaattggtacggcc

tgacctgctgagctgactcagcaatctgtatgtcaacgaaagtatttaagtccaatgatt

agtttgaacttaatatgtgttgagttgctgagctgcatgtggtatttcag

GACTGCGAGCGGTACATTCAAGGATGGTGAACTGCGGCTTAATCAAAGAGGTTTGCAGCT

TATCTCCGAGGAAAATGGAGATGAACAT

gtgagttctcggttcaatactttttaatgactacttttgttgtctctcaatttgtccaat

tgtgtttgtgttgtattgtacttcgatcgttcatgtttgatgcgtgggcgcgcatgcgtg

ttgcactgttgcatgcgtcagagtcagtgcactcagattgaaccgtatatacgccactat

aaaaaaagcgtacatgtcgccacatgaagtgcaatacaacacaaacactaaattaccgaa

ccaggaacttgcatattcatccctgtcaaagacaaccatttaatttgcacattactttgt

gttctgaagaaacaagaagtgtctcaaaaacatttctagacacaaaaaagtagtgcagta

aaagatcaagccttcaatagcttcaacaggaaaaaggagaaggaaaaggaaaacatatta

tataaaataggcagtttaggccaaggaatcaaggaaacctacatgagtttattgagaggc

gtctcctacgctggcagaatgagctggagttccttgagcggcctcttccccctcatggtt

gtaacaccccggccacaaccatcggttcctggccgttatgcctgacgggatctagactag

ccccatagaccaacactagtctttcctgtgcactttgtcctcactcgtgcgcacctcaga

aggacttctcggtcagtcacccatcctcaaattaccccatgcgaagcaacctggaggttc

tttggagatgagcttctgaaaaagaaggcgcaccttgttgaaatgagtagtctatcattc

ctattaagccatgatgtcacaatggtaggtatcttcctatggttagtgtttcttcgctag

gagatgaatatggaactggccaaagccttaaactagttggggagtaggtaggagaccagg

tgaactgctatggagatatggagtttggatctgcccagctgaggcacgctgagagagcgg

agcaaccttgaggaaggcctgggccacgatagggtagatggaagtgaagatggggcatgt

tgtacttccatcgaccaaagttgctaatttatggaccgcgggggccagaggaaccctttc

agtttaaacttggtagccatttcatccataatcataacatatttgcagaaatttccatta

tgtttcgtgattatcttgagtagatgttagtttttaatgcctaggattgaaattatgtac

tctgctgctcgctattggttattagatgccaccaacagaaaccacaacaagaatatattg

ttcttcagtttgtgtttattagtatttggataatgatcaagttgtttccgaattctgttt

gcatcttgatttgtttcttttcctcag

CAATCAACAAAAATGAAGGTGGAAGATGTGCAGTTATCAATGGATGACCTCGAAATGATT

CAGGTCATTGGTAAGGGAAGTGGTGGCGTCGTCCAACTAGTGCGGCACAAGTGGGTGGGC

ACATTTTATGCCTTGAAG

gtaacagtacactaaattcacctagtgggccgcacaaatgcctatgatggatttttttgt

tgatgttagagcattggggtagactgcttgtttttttatcgtacctgtttatatattttc

atgtttgttaacatggaagttaatatgtttatctgtttgaacagatttgcattctatgca

ttgttatgacaatctgattattctacatctgaagacttatttgttttgttttgtcacttt

gtgatggcatggcaatatattcaattagatccacatttatgcacaccattaaaatgagtt

gtagcaaaaacatctagtactggctgatctttgcctgcaaaatctccacagaacaccaat

tttaacggaaactgacactaatgtatgagaatacatcaggtaacgatatacagccgatta

aacattttcaagcattattcctggtctttgtactgctgtggcatttcgttgtccatatga

tctatcagtcaattgattattttcaatttcttcacag

GGAATTCAAATGAACATTCAGGAGTCAGTACGCAAACAAATAGTACAGGAGCTCAAAATA

AATCAAGCAACACAGAGCCCCCATATCGTCCTTTGCCATCAATCTTTTTACCACAATGGT

GTAATATATCTTGTTCTGGAATATATGGATCGTGGATCTCTTGCAGACATAATTAAACAA

GTTAAAACCATTCTGGAGCCATACCTTGCAGTACTTTGCAAGCAG

gtatgtatccgtttggcctatggtttgactgcatccggaatattcaaatttggtcacatc

cgatggaagttgtagctagagtctggcacatctaacattaataatgtcttaacaaatcag

GTTTTGGAGGGTCTGTTATATCTTCATCATGAAAGACATGTGATTCACAGGGACATAAAG

CCATCTAACTTGTTAGTTAACCATAAAGGTGAAGTAAAGATTACTGATTTTGGGGTGAGT

GCAGTGCTAGCAAGTTCAATCGGTCAGCGTGATACATTTGTTGGAACCTACAACTATATG

GCG

gtatgtaaaaagatctgctttgtcttctgactgcggcttatgctcataatggttatctgg

ggtgctcaaaatttgctgtaatagttttctccctttcctttttgtgcag

CCTGAGCGGATTAGTGGCAGCTCCTATGACTACAAGAGTGATATATGGAGTTTGGGCTTG

GTAATACTTGAATGCGCCATTGGCCGCTTCCCCTATACTCCTCCGGAAGGAGAAGGTTGG

CTAAGCTTCTATGAACTATTAGAAGCAATTGTTGACCAGCCACCACCTTCTGCACCAGCA

GATCAGTTCTCTCCAGAATTCTGCTCATTTATCTCTTCCTG

gtacgctaaacattattccgaggaactacatatgaaattagctcagtcactgcatttctg

ttttgtcttcttgcttagtatgtttgaatgaacttcttgccctgttttctaaagttctga

ttttcttttgcgtgtctttcacgtaaatattttaaattgtttgtaaagttaacttgtttt

aatagttatctgctttctcgtccacagctcatggactttgtccactcaagtattatttcc

ccctttgagaaaatgtgtttttgtctattcaagtatactttcccctttgaacaagtgtgg

tcttaatagaattgttttagtgtgataatttggacttcattaattctggagaataaatgt

ttaaactgcgcggtgttcttcagattagaattagtgaaatcttcagatgtccagagttta

taatggtgacagcaaacggccgaatcaatgttcttattataaaaattacatttcagtaca

gcccaagaaattgcagccatcccaaaaggatcgacttgcgtctccagacaacagctactc

agggcaacttgccatgtaactatctgtttatcaaaccctgtttaagtagcttattggcct

ttgaaacttcaagcagtttagctaatctggagtttgagttatcaatcatgtaacaaccct

gattttcagtttaatgtcaaaacatacttattgatttaagttagctatgttaatatctga

tctaattgttgtgttgattgacaaaaccatgactgtgaggttatagtataaaccagttaa

caatctggcatcttatcttactgatggattaaggcatgccaaaaaaacacggaaaataac

cctcctactatgcagggtgtatgaacctgaccaaatcatgttgtgtgacccaaaaggaag

aggaaggagtaccaaagaacaataggcatatgaggcatcacaaaaactcaaaccaaagac

tccgatgaggaacgagggctttagccttccatcacgttcccatgcccactgctcttaggt

cttcttcctcttcatgtctcacaatggacatcttataccagctgaatatatccacataaa

tgttcagtttttctaaagatttggcaaacaaaaaggaagaggaaggagcgcttaagaacg

ataggaatgagaggcactgggaaataccaaatggcatttccacctcaacacaagatgtga

agaaccgataacttaggctttcctctatgttctcatacccattgctcttaggccctctgt

ttcttcctcttcctagctcgtttttgagatttcacatcgaatatgtgcaaaaatatagtt

ggtttgtaagctaaagcaaccaagcaagccaaaaaaggaagaggaaggagtgctttagaa

caatgggaatgggaggcattgggaaacaccaaactaaattctcacctccaacactagagg

tgaaaaccgagcaggttaggctttcctccatgttcccattcccactactctaaggccctc

tattcttcctcttccgggctcgcctttgaggtctcatgttatctaaacatgtgggcacat

gtctatggtttctccatcagagctatcacgtaagcgaaaaggaagaggaaggagtgcgca

agaatggtaggaatgggaggcatcaggaaaggatccatgccaaatatttgcacctccaac

acttgaggtgcaaacaaacatgcagggctttcctccatgttcccattcccaatgctcttg

agttctctctttcttcctcttcccggcttactttcagtgcctacaaatctaatgaaacaa

aaactctcaaagaacaagcttgaattgttggacaaagcgattcagctttgaggtataagt

aatgggtggtgcaacatatgcagaaccttagaggagaaactaatgaagacacctgatgca

tgtgggcaccattctacagtcaatgataatggggccacataatttccggccctgtcctat

tgtcctctactcttaatttagggcacatgtgtaaagatctcttctccttacctttattac

ccatatcagaaactggtacataatatccataccagccttttttagaacaatatccgtatc

agcctgtgcatttatggtgtcaaaatatgtagcaataaggagttagagctaaaagtaata

tgaaattggacaaattaaaccctgtaagtaaacttggaaaaattacacaacagtggggca

aagattggaacataatgtacttcggaccgctgcagtattagagatgacataatgtgatca

aaacatgataagagagatctcatatctgcaaaaccaaaatatctataggctattttatca

agaaaaaatgtgtatagtcgtattgctcttgaagtgtttagtatccaaattctgcaggct

gtacacaactacatggcatgtgaaaaatataggaaaaaacatctctccaaagaaaccatc

accctcgatccctagcagtggcggagccagcctgtgaatgcagctcgggcaatacttaac

tgtgatagtgtgagctggcacctgccgaaagaaataatccttctaaccatagattatcac

tagtgcttaggagtaattttctggactagcccaggcagccgcccggggttgccgggaggt

ggccccgtcactgatccctaggaggaaaaaccttgaagaataaggtcacatattccgtct

aaactcactatacacagtattttaagaacaaggttgggacacaaaggaagaataagatcc

cgaagagagtatggtacttcttccatccatcaaagtacttcccagttcaaccttgtagct

cagccaccattccccaaggattaaaacagctaggttgagatgcaaacagccagcaaggtt

caagagtggggaaataagcctaatcccgcacatctaaacaatgtataaaaagacaaccat

ttatgaattatgatcaagtgcacaattccattggagtttacaattttttcgacgctgcag

atatatcttgttgcagtcattgctgtttcttgctagcctgcatatctatccattagtttg

ctatcccatgtgtgaactctaggaattgatggtaaaaataatctgcaatgcacatatctt

tacatgttctgtgtgcaaattgccatatttcactctgatggtaaaaataatctgcaatgc

acataaggactaaatcacatcaatgacacaccacctattgttcccatgcccactgctctt

gggccctctttccttcctcttcctgtcttactatgggcatctcatatcatctgaatatat

gcatatgtatgtttttttttctaaagcgatttagcaaacaaaaaggaagaggaaggagcg

cctaacaacgataggaatgagaggcatcggaaaatgccaaactgaattgccacctcaaga

caagaggtgaagaatcatcaattttaggctttcctctatgttctcattcccactgctctt

acgccctctctttcttcctcttcctagctcgcttttgagatcccacatcgaatatgtgca

cacatgtatttggtttctaagctaaaggaaccaagcaagccaaaaaaggaagaggaagga

gtgctttagaacaataggaatgggaggcatcgggaaacaccaaagcaagttctcacctcc

aacactagaggtgaaaacaaagcaacttaggctttcctccatgttcccattcccactgct

cttatgccctcttttcttccacttcccggcttgccttcaagctctcaagttatctaaata

tgtggacacatgtctatcgtttctccatcaaagctactgtgcaatccaaaaggtagagga

aggagtgcacaacaacggtaggaatgggaggcattgggaaaggatccatgccaaatatga

aatgtttgcacctccaacactagaggtgcaaacagaaaagcagggctttcctccatgttc

ccattcccagtgctcttgagctctctctttcttcctcttcctggcttacattcagtgcct

agaaatttagcgaaacaaaggtctcgaaggacaagtttgaattgttggaccaagtgtttc

agctttgaggtataaataatgggtggtgcgccatatgcagaaccttagaggagaaagcaa

tgaagacacccggtgcatgggggcaccattctacagtcaatgataatggggccacataat

cactggccctgtcctattgtcctctactcttaatttatgacatgtgcgtaaagatctctt

ctgcttacctttattaaccatatccgaaactggtacataatatccgtaccagcctttttt

tagaacaatatctgtatcagtctgtgcatttagtgtcaaaacatgtagcagtaagaagtt

atgacctaaaaataatatgaatttggataaattaaaccctctaagtaaacttcgaaaaat

tacaaaactgtgggcaaagaaccaccaaagtgacagttaattggtacacatggtactttg

gacatgagctgcattatttcagataacagaatgtgattacaacatgatgagagggatctc

atatctgcgaaaccaaaatgtctataggcaattttatcaagaaaaaatgtgtatagtctt

aagtaatttagtatccaaattctgcaggctgtacacaactacatgacacgagagaaatat

aggaatatatctccaaagaaaccatcaccctcaattcctagagaacacatgaaggtatag

tgatcacatattcagtctaaactcattatacacatagtattttaagaacaaggttcgcac

agaaaggaagaacaagatcccgaagagagtatggtagctcgtccacccatccctccaaga

atttaatcacccaggttgagatgcaaacagccagaaggttccggagtggggaaataagcc

ttatcccgcacatctaaacaacatataaaaaacaacaccgttttatgatcaagggtacaa

ataccattggagttcacaattttcgaagctgcagatatatctttgttgcagtcatggttg

tttcttgccatcctgcttatttatccattagtttgctatcccatgtgtgaactctttagg

aactgatgataaaaataatctggatgcacagattgctacatgttttgtgtgctaattacc

attttttgcgctctgatttctgctcgtactcttaactacagtggacagagtggtgtgtca

tcgatgtgattaactaaaacagtaagacactaaaacgcatttaccatgcgactctgtcta

aactccacagtcctcacatcattcttgacctgcagcaattggctcatatctaccattcaa

atactttcttttttcgagcaaccggcaagaactcctcagtcctcacatcattcttgacct

gcagcaatttagcaattggctcatatctcatatcatctgaacatatgcatatgcatgttt

tttttcctaagctgatttagcaaacaataagaggaatgagcgcctaagaacgataggaat

gagaggcatcgggaaatgcccaactgaattgccacctcaagacaagaggtggagaaccag

caatttaggctttcctctatgttctcattcccactgctcttatgccctctctttcttcct

cttcctagctcgcttttcagatcccacatcgaatatgtgcacacatatatttggtttcta

agctaaagtaaccaagcaagccataaaaggaagaggaaggagtgctcgagaacaatagga

atgggaggcatcgggaaacaccaaagcaagttctcaccttcaacactagaggtgaaaaca

aagcaagttggctttcctccatgttcccactcccactgctcttatgccctcttttcttcc

acttcccggcttgccttcaaactctcaagttacatgtctactgtttctccatcaaagcta

ctatgcaatccaaaaggaagaggaaggagtgcacaacaacggtaggaatgggaggcattg

ggaaaggatccatgccaaatctttgcacctccaacgctagaggtgcaaacgaaaaagcag

ggctttcctccatgttcccattcccagtgttcttgagctctctctttcttcctcttcccg

gcttactttcagtgcctagaaatctagcgaaacaaaagctctcgaaggacaagcttgaat

tgttggaccaagtgtatcagctttgaggtataaataatgggtggtgcaccatgcagaacc

ttagaggagaaagcaatgaagacatctggtgcatgggggcaccattctacagtcaatgac

agtggggccacataatcactggccctgtcctattgtcctctactcttaatttatggcatg

cacgttaagatctcttctccttaccttcattatccatatccgaaactggtacataatatc

cacaccagccttttttagaacaatatccgtatcagcctgtgcatttatggtgtcaaaata

tctagcaataagaagttatgagctaaaagtaatatgaaattgggcaaattaagccctgta

agtattaagccctgtaagtaaacttggaaaaattaaaaaacagtggggcaaagaaccacc

gaagtgacaactaattggaacacaaggtacttaggacatccgctgcagtattagagatga

cagaatgtgattaaaacatgataagagagatctcatatatgcaaaaccaaaatgtctaga

ggctattttatcaagaaaaaaaagtgtatagtgctattgttttttaagtatttagtatac

aaattctgcaggctgtacacaactacatggcatgtgagaaatatagaaaaaaatatatct

ccaaagtaaccatcaccctcgatccctagcagtggcggagccagcctgtgaatgtagccc

gggcaagacttaactgtgagagtgtgagctaccacctgcccaaagaaataatccttctaa

ccaaaccatagattatcactagtgcttgggagtaactttctggactagcccaggcagcca

cctggggttgccgggaggtgagcccgtcactgatccctagaggaaaacacttgaaggtat

agtgatcacatattcactattcaatctaaactcaataaacacatagtattttaagaacaa

ggttgggacacaaaggaagaacacgatcccgaagagtgtatggtacttcctccacccatt

gaagcacttcccagttcaaccttgtagctcggccaccatcctccaaggattaaaacagag

gttgagatgcaaacatccagcaaggttcaagagtggggaaataagccttatcccacacat

ctaaacaatatataaaaagacaaccatttaagatcaagtgtacaatttccattggagttt

acaacttctttgatgctacagatatatcttgttgcagccatggttgttttttgctagcct

gcatatctatccattagtttgctatcccatgtgtgaactctaggaattgatgataaaaat

aacctgcaatgcacatatcgttacatgttctgtgtgcaaattaccatatttcacactctg

atttctgcttgtactcttagctacagtggtgtcattgatgtgatttagtctctagcatga

gttcaacatagggaactactgaaaatttgtaccattctatgtgttaaagttgtgtgtctc

atcttgttaagtctcacggtgtgcttgtttatatttctaagaaggccccttttcagatat

agatatgtatacttcatatatataatacctgaaaggtaaattaaatgttttggcatcagg

attcgtgtgcagaataatctttaacacagaaatttgcagtccaactatgtagtgtgaatg

aacttgaataaaatcgtattatactactatgaatcctcttcatgttgtgcaacacaaaaa

gacgagcaaggggctccaaagaataataggcatgggaggcatcaggaaaactcaaccata

gcctctttcagcgttgcacgaggaactgagagagctttagccttccatcatgttcccatg

cccactgctcttgggccatctttccttcctcatcctgtctcacaatgggcatctcatatc

atctgaacatatgcatatgtatgtttttttcctaaagtgatttagcaaacaataaggaag

aggaaggagcgcctaagaacgataggaatgagaggcatcgggaaatgccaaactgaattg

ccacctcaagacaagaggtgaagaaccattaagttaggctttcctctatgttctcattcc

cactgctcttacgccctctctttcttcctcttccttgctccttttgagatcccacatgga

atatgtgcacacatattttgtttctaagctaaaacaacccaaaaaaggaagaggaaggag

tgcttgagaactgtaggaatgggaggcaccgggaaacaccaaagcaagctctcacctcca

acactagaggtgaaaacaaagcaagttaggctttcctccgtgttcccactcccactgctc

ttatgccctcttctcttccacttcccggcttgccttcaaactctcaagttatctaaatat

gtggacacatgtctaccgtttctccatcaaagctactatgcaatccaaaaggaagaggaa

ggagtgcacaacaacggtaggaatgggaggcatcaggaaaggacccatgcctaatatttg

cacctccaacactagaggcgcaaacaaacaagcagggctttcctccatgttcccattccc

agtgctcttgagctctctcattcttcctcttcccggcttactttcagtgcctacaaatct

agagaaacaaaagctctctgaaggacaagcttgaattgttggacaaagtgtttcagcttt

gaggtataaataatgggtggtgcgccatatgcagaaccttagaggagaaagcaatgaaga

cacccggtgcatgagggcaccattttaagtcaatgataatggggccacataaggaccggc

ggtgtcctattgtcctctactcttaatttagggtatgtgcataaagatctcttgtgatta

cctttattacccatatctgaaactggtacataatatccataccagccttttttctagaac

attatctgtgtctgcctgtgcatttatggtgtcaaaatatgtagcgataaaaagttatga

gctaaaagaatatgaaattggataaatcaaaccctgtaagtaaacttggaaaaaagtaca

aaatagtggggcaaaaaaccaccaaagtgacaactaattggaacacaaggtactttgggc

atgagctgcgcattattatagatgacagaatgtgattacaacatgattagagagatctca

tatctgcaaaacgaaaatgtctataggccattttattaataaaagtgtgtatagtcctat

tgttttttttaagtaatttagtatccaaattctgcaggctgtacacaactacatggcatg

agaaattataggaaacatatctccaaagaaaacatcaaacacatgaaggtatcacatatt

cagtctaaactcattatacacacagtattttaagaacatggttcggacacaacggaagaa

caagatcccgaagagagtatggtagttcttctacccatcaaagtactttccagttcaacc

gtaggtcggccaccatccctccaagattaaatatcccaggttgggatgcaaatagccagc

agggttccagagtggggaaataagccttatcccacacatctaaacaatatgtaaaagaca

actccaatttatgatcaagtgtacaaattccattgtagttcacaattctcgaagttgcag

atatatcttgttgcagtcatggttgtttcttgctagcctgcatatttatccattagtttg

ctatcccatgtgtgaactctttaggaactgatggtaaaaacaatctgaatgcacatatta

ttacatgttttgtatgccaattaccatttttcgcactccgatttctgctggtagtcttag

ctacggtggtcagagtggtgtgtcattgatgtgattaactaatacagtaatgcacttaca

tgcatttaccatgtgacattgtctaaactcctcagtcctcacatcatgcttgacctgcag

cgattggctcatatctaccattcaaattctactttttttcgagcaactggcaaaagccct

gcctttccatatagaagagaattgaccaatttattaggaaacctggccgaaaacagacca

tttgaatactttagaacttacagtcaactccatttattggatatatgttttattggtcta

tattttagttagcgattattcataatgaaatttgcacatcttgaaggcaaccgatggttc

ctgctagcttttgctttgtagatttgtgcagtctgtgtagtatatacatgcaggagcata

tcatattgcagtgttatttcatattactatcttgctttgcag

CATACAGAAGGACCCCGCCGAACGGATGTCTGCTTCAGAACTCTTG

gtgagcattcccttccactttacaagttgttgtagcgtgtccaactaactgtggtaccgt

tgattctgtcgactagtgtgtgtgctgaatcgtcgttcgttcgatgttgttgtcgcag

AACCATGCCTTCATCAAGAAGTTCGAGGACAAGGATCTAGACCTGAGGATCCTCGTCGAG

AGCCTCGAGCAGCCCATGAATGTGCCCGAGTGAGGCCGTCGCTCGGGGAGGTGAGGCTTT

GCAGCGAGTTTCTTCCAGGGCCGCCCTCCTGACGGCAATTGTGTATTGCTGTGAACCTGA

TGAATGAGTGACCAGCCCCCCACCCCTGTATGTGATGATGTTATGTTGGAAAATTCCCAG

AGGATTCGAATGGATTATTAATGTATGGTAATGAATCAAGGCCCTTTTGATGTGTGTCTT

GTTATGAATGAATGGATCTGCTGTGGCTA

**>TaMKK6(4DL) TRIAE_CS42_4DL_TGACv1_342786_AA1122060.5**

ACTGTCTGACTCTCTACTGAGTCTACTGACCAGGGCAGCCCCTCCCACCTCCCCTCCCCT

CCGTCCGCTAGCCGCCACGTCCCCGGCCGACCCGACCACCCCTCGCCGCCGGACCGCATC

TCCTCCCCTCACCTCCCACCTCCAGATCCGCGCGCCCCCTGACCCCTCCCCGCCCGCCGC

CGCCGGGAACTTTCTTCCGAGCTCGATCCATCCAGTGGCCGGCGACAGCGACCGCCCGCC

GCGCGGATCGATCCAGCGACCAACGTGGAGCACGGCTGCTAGCCGTGCCCTCTCAGTCAA

CCCAAGTCGCACCACGTGCCACCATTTTGACCAGCTCCGCCGCTGCTATTTTTCTCCCTA

AATTCCTCCCCCGGCCCCGGCCTCCCCTCGCACCGACGGCGCCCCGGCCGCTCCTCCCCG

TCCGCCGCGCCTCCGGTGAGCTTCCGTATCCGGCCTGGTCCAAAGTCCGTATCTTTATGC

CGACTCGTCCGATCTCCATCTAACCTGCTTCCGGCTTTGTCCAGCTCCGCGTCCATCTCC

CGCCCGAGAAACCCTAGCCAGAGGAAGGAGGACACCATGAGGGGCAAGAAGCCGCTCAAG

GAGCTCACGCTCTCCGTGCCGGCGCAGGAGACGCCCGTCGATAAGTTCCT

gtatgtctcccccattccttggcctaaatttcatgtttcccccctctctttttcccccac

taaagctatcacaggtttgcgctttatgtctaataaatgagagattttttgggtgcctgg

gtagatttttgtggtttatttgtggattttgatggggctgggtagccattactgggttgc

tgaagcatatatttactttgaaggcattaagtgtagtgcttgatttgaaattattacggt

ctgagctgctggctgactcagcagtctgtatgtcaacgaaagtatttaagtccaatgatt

agtttgaactcaatacgtgttgagttgctgagctgcctgtggtatttcag

GACTGCGAGCGGTACATTCAAGGATGGTGAACTGCGGCTTAATCAAAGAGGTTTGCAGCT

TATCTCCGAGGAAAATGGAGATGAACAT

gtgagttcccggttcaatagttttaaatgactacttttgttctctctcaatctgtccaat

tgtgtttgtgttgtattgtacttcgatcgttcatgtttgatgcgtgggcgcgcatgcgtg

ttgcactgttgcgtgcttcagagtcagtgcactcagagggtctactgcatttgatcaacc

aggtggaaccaacaaaacatccgataagatttgagattgaatcgtatatacgccactata

aaaaaaagcatacatgtcgccacatgaagtgcaatacaacacaaacactaaattaccgaa

cgaggaacttacatattcatctctgttttcctcagagatgccacaaaccgctttgattaa

gtctaaagacctccagcaatcaaagacaaccatttaatttgcacattacttcgtgttctg

aagaaacaagaagtgtctcaaaaacatttctagacacaaaaaagtagtgcagtaaaagat

caagccttcaatagcttcaacaggaaaagggagaaggaaaaggaaaacatattatataaa

atgggcagtttaggccaatgaatcaaggaaacctacatgagtttattgagaggcgtctcc

tacgccggcagaacgagctggagttccttgagcggcctcttccccctcatggttgtaacg

ccccggccacaaccatcggttcctggccgttatgcctgacgggatctagactagccccat

agaccaacactagtctttcctgcgcactttgtcctcactcgtgcgcacctcagaaggact

tctcggtcagtcacccatcctcaaattaccccatgcgaagcaacctggaggttctttgga

gatgagcttctgaaaaagaaggcgcaccttgttgaaatgagtagtctatcattcctatta

agccaggatgtcacaatggtatcttcctatggttagtgtttcttcgctaggagatgaata

tggaactggccaaagccttaaactagttggggagtaggtaggagaccaggtgaactgcta

tggagatatggagtttggatctgcccagcggaggcatgctgagagagcggagcaaccttg

aggaaggcctgggccacgatagggtagatggaagtgaagatggggcatgttgtactttca

tcgaccaaagttgctaatttatggaccgcgggggtcagaggaacccttacagtttaaact

tggtagccatttcatccataatcataacatatttgcaaaattttccattatgtttcatga

ttatcttgagtagatgttagtttttaatgcctaggattgaaattatgtactctgctgctc

gctattggttattagatgccaccaacagaaaccacaacaagaatatattgttcttcagtt

tgtgtttattggtatttggataatgatcaagttgtttccgaattctgtttgcatcttgat

ttgtttcttttcctcag

CAATCAACAAAAATGAAGGTGGAAGATGTGCAGTTATCAATGGATGACCTCGAAATGATT

CAGGTCATTGGTAAGGGAAGTGGTGGCGTCGTCCAATTAGTGCGGCACAAGTGGGTGGGC

ACATTTTATGCCTTGAAG

gtaacagtacactaaattcacctagtgggccgcacaaatgcctgtgatggatttttttgt

tgatgttagagcattggggtagactgcttgtttttttatcgtacctgtttatattttttc

atgtttgttaacatggaaattaatatgtttatctgtttgaacagatttgcattctatgca

ttgttagacgatctgattattctacatctgaagacttatttgttttgtttagtcactttg

tgatggcatggcaatatattcaatttgatccgcatttatgcacaccattaaaatgagttg

tagcaaaaacatctagtactggctgatctttgcctgcaaaatctccatagaacaccaatt

ttaatggaaactgacactaatgtatgagaatacatcaggtaatgatatacagccgattaa

acattttcaagcattattcctggtctttgtactgctgtggcatttcgttgtccatatgat

ctatcaatcaattgattattttcaatttcttcatag

GGCATTCAAATGAACATTCAGGAGTCAGTACGCAAACAAATAGTACAGGAGCTCAAAATA

AATCAAGCAACACAGAGCCCCCATATCGTCCTTTGCCATCAATCTTTTTACCACAATGGT

GTAATATATCTTGTTCTGGAATATATGGATCGTGGATCTCTTGCAGACATAATTAAACAA

GTTAAAACCATTCTGGAGCCATACCTTGCAGTACTTTGCAAGCAG

gtatgtatacgtttggcctatggtttgactgcatccggaatattcaaattttgtcacatc

tgatggaagttgaagctagagtctggcacatctaacattaataatggcttaacaaatcag

GTTTTGGAGGGTCTGTTATATCTTCATCATGAAAGGCATGTGATTCACAGGGACATAAAG

CCATCTAACTTGTTAGTTAACCATAAAGGTGAAGTAAAGATTACTGATTTTGGGGTGAGT

GCAGTGCTAGCAAGTTCAATCGGTCAGCGTGATACATTTGTTGGAACCTACAACTATATG

GCG

gtatgtaaaaagatctgctttgtcttctgactgcggcttatgctcataatggttatctgg

ggtgcacagaatttgctgtgatagttttcttcctttcctttttgtgcag

CCTGAGCGGATTAGTGGCAGCTCCTATGACTACAAGAGTGATGTATGGAGTTTGGGCTTG

GTAATACTTGAATGCGCCATTGGCCGCTTCCCCTATACTCCTCCGGAAGGAGAAGGCTGG

CTAAGCTTCTATGAACTATTAGAAGCAATTGTTGACCAGCCACCACCTTCTGCACCAGCA

GATCAGTTCTCTCCAGAATTCTGCTCATTTATCTCTTCCTG

gtacgctaaacattattccgaggaactacacatgaaattagctcagtcactgcatttctg

ttttgtcttcttgcttagtatgtttgaatgaacttcttgccctgttttctaaagttctga

ttttcttttccgtgtctttcacgtaaaaatattttaaattgtttgtaacgctaacttctt

ttaatatttctcaaaaaaaaaacttcttttaatagttatctgctttctcgtccaaagctc

atggactttgtccactcaagtattattcccccatttatgaaaatgtgttttgtctattca

agtattactttcccctttgaacaagtgtggtcttaatagaactgttttagtgtgataact

tggacttcattaattctggaaataaatgtttaaactgcgcggtgttcttcagattagaat

tagtgaattcctcagaagtccagattttataatggtgttttttgtaacagcaaacggccg

aatcaatgttcttattataaaaattacatttcagtacagcccaagaaattccagccatcc

caaaaggatcgacttgcgtctccagacaatagctactcagggcaacttgccatgaaacta

tctgtttatcaaaccctgttcaagtagcttgttggcccttgaaacttcaagcagtttagc

taatttgaagtttgagttatgaatcatgtaacaaccctgattttcagtttaatgttaaaa

tgtacttgttgatttaagttagctatgttaatatctgatctaattgttgtgttgattgac

aaaaccttgactgaggttgtagtataaaccagttaacaatctggcatcttatcttactga

tggattaaggcatgccaaaaaaacacggaaaataacgcgcctactatgcagggtgtatga

acctgaccaaatcatgtcgtgtgacccaaaaggaagaggaaggagtaccaaagaacaaat

aggcacaggaggcatcacaaaaactcaaaccaaagactccgacgaggaacgagggcttta

gccttccatcacgtccccatgcccactgctcttaggtcttcttcctcttcatgtctcaca

atggacatcttataccagctgaatatatccacataaatgttcagtttttccactaaaaga

tttggcaaacaaaaaggaagaggaaggagcgcttaggaacgataggaatgagaggcactg

ggaaataccaaatggcatttccacctcaacacaagatgtgaagaaccgataacttaggct

ttcctctatgttctcatacccattgctcttaggccctctgtttcttcctcttcctagctc

gcttttgagatttcacatcgaatatgtgcaaacatatagttggtttgtaagctaaagcaa

ccaagcaagccaaaaaaggaagaggaaggagtgctttagaacaataggaatgggtggcat

tgggaaacaccaaactaaattctcacctccaacactagaggtgaaaccgagcaggttagg

ctttcctccatgttcccattcccactactctaaggccctctattcttcctcttccgggct

cgcctttgacgtctcatgttatctaaacatgtggacacatgtctatggtttctccatcag

agctatcacataagcgaaaaggaagaggaaggagtgcacaagaacggtaggaatgggagg

catcaggaaaggatccatgccaaatatttgcacctccaacacttgaggtgcaaacaaaca

tgcagggctttcctccatgttcccattcccagtgctcttgagttctctctttcttcctct

tcccggcttactttcagtgcctacaaatctaatgaaacaaaaactctcaaagaacaagct

tgaattgttggacaaagtgattcagctttgaggtataaataatgggtggtgcaacatatg

cagaaccttagagaagaaactaatgaagacacctgatgcatgtgggcaccattctacagt

caatgataatggggccacataatcaccggccctgtcctattgtcctctactcttaattta

gggcacatgtgtaaagatctcttctccttacctttattacccatatcagaaactggtaca

tgatatccataccagccttttttagaacaatatccgcatcagcctgtgcatttatggtgt

caaaatatgtagcaataaggagttagagctgaaagtaatatgaaattggacaaattaaac

cctgtaagtaaacttggaaaaattacacaacagtggggcaaagaaccactgaagtgacaa

ctaattggaacataatgtacttcggaccgctgcagtattagagatgacataatgtgatca

aaacatgataagagagatctcatatctgcaaaaccaaaatatctataggctattttatca

agaaaaaatgtgtatagtcgtattgctcttgaagtgtttagtatccaaattctgcaggct

gtacacaactacatggcatgtgagaaatataggaaaaaacatatctccaaagaaaccatc

accctcgatccctagcagtggcggagccagcctatgaatgcagctcgggcaatacttaac

tgtgatagtgtgagctggcacctgccgaaagaaataatccttctaaccatagattatcac

tagtgcttaggagtaattttctggactagcccaggcagccgcccggggttgccgggagtt

ggctctgtcactgatccctaggaggaaaaaacttgaagaataaggtcacatattccgtct

aaactaaggtttacgattcgatgagtcgacgagtcgtcctgaggttgagactcaatagac

taagcgtgagtagtcgacactaaagttgcgactcatagactagtcgacactgaagtgtag

tcggccctggagttgagactcatggactagtcttgactagtccttggactcataatccat

ctaaactcctatacatagtattttaagaacaaggttgggacacaaaggaagaataagatc

ccgaagagagtatggtacttcttccatccatcaaagtacttcccagttcaaccttgtagc

tcagccaccatcccccaaggattaaaacagttaggttgagatgcaaacagccagcaaggt

tcaagagtggggaaataagccttatcccacacatctaaacaatgtataaaaagacaacca

tttatgatcaagtgtacaattccattggagtttacaatttttttgacgctgcagatatat

cttgttgcagtcatggctgtttcttgctagcctgcatatctatccattagtttgctatcc

catgtgtgaactctaggaattgatggtaaaaataatctgcaatgcacatatctttacatg

ttctgtgtgcaaattgccatatttcacactctgatttctgcttgtactcttagctacaac

ataaggactaaatcacatcaatgacacaccacctattgttcccatgcccactgctcttgg

gccctctttcctttctcttcctgtctcaccatgggcatctcatatcatctgaatatatgc

atatgtatgttttttttctaaagcgatttagcaaacaaaaaggaagaggaaggagcgcct

aagaacgataggaatgagaggcatcgggaaatgccaaactgaattgccacctcaaggcaa

gaggtgaagaaccattaagttaggctttcctctatgttctcattcccactgctcttacgc

cctctctttcttcctcttccttgctcgcttttgagatcccacatcgaatatgtgcacaca

tatatttggtttctaagctaaagtaaccaagcaagccaaaaaaggaagaggaaggagtgc

ttgagaacaataggaatgggaggcatcgggaaacaccaaagcaagttatcacctccacac

tagaggtgaaaacaaagcaagttaggctttcctccatgttcccattcccactgctcttat

gccctcttttcttccacttccctgctcgccttcaagctctcaagttatctaaatacgtgg

acacatgcctatcgtttctccatcaaagctactgtgcaatccaaaaggaagaggaaggag

tgcacaacaacggtaggaatgggaggcattgggaaaggatccatgccaaatatttgcacc

tccaacactagaggtgcaaacagaaaagcagggctttcctccatgttcccattcccagtg

ctcttgagctctctctttcttcctcttcctggcttactttcagtgcctagaaatttagcg

aaacaaaggtctcgaaggacaagtttgaattgttggaccaagtgtttcagcttggaggta

taaataatgggtggtgcaccatatgcagaaccttagaggagaaagcaatgaagacacccg

gtgcatgggggcaccattctacagtcaatgagaatggggccacataatcactggctctgt

cctattgtcctctactcttaatttatgacatgtgcgtaaagatctcttctgcttaccttt

attaaccatatccgaaactggtacataatatccgtaccagcctttttttagaacgatatc

tgtatcagcctgtgcatttagtgtcaaaacacgtagcaataagaagttatgagctaaaaa

taatatgaatttggataaattaaaccctctaagtaaacttcgaaaaattacaaaactgtg

ggcaaagaaccaccaaagtgacaattaattggaacacatggtactttggacatgagctgc

attatttcagatgacagaatgtgattacaacatgatgagagggatctcatatctgcggaa

ccaaaatgtctataggccattttatccagaaaaaatgtgtatagtcttaagtaatttagt

atccaaattctgcagctgtacacaactacatggcacgagagaaatataggaatatatctc

caaagtaaccatcaccctcaattcctagagaacacatgaaggtatagtgatcagtgatca

catagtcagtctaaactcattatacacatagtattttaagaacaaggttcacatacaaag

gaagaacaagatcccgaagagagtatggtagctcgtccacccatcgaagtactttccagt

tcaaccttgtagctcggccaccatccatccaataatttaatcacccaggttgagatgcaa

acagccagaaggttccagagtggggaaataagccttatcccgcacatctaaacaacatat

aaaaaacaacaccattttatgatcaagggtacaaataccattggagttcacaattttcaa

agctgcagatatatctttgttgcagtcatggttgtttcttgccatcctgcgtatttatcc

attagttttgctatcccatatgtgaactctttaggaactgatgataaaaataatctgaat

gcacagattgttacatgttttgtgtgccaattaccatttttcgcgctctgatttctgctc

gtactcttagctacagtggacagagtggtgtgtcatcgatgtgattaactaaaacagtaa

gacactaaaacgcatttaccatgcgactgtctaaactccacagttctcacatcattcttg

acctgcagcaattggctcatatctaccattcaaatactattttttttcgagcaaccggca

agaactcctcagtcctcacatcattcttgacctgcagcaatttagcaattggctcatatc

tcatatcatctgaacatatgcatatgtatgttttttcttcctaaagtgatttagcaaaca

ataaggaagaggaaggagcgcctaagaacgataggaatgagaggcatcgggaaatgccca

actgaattgccacctcaagacaagaggtgaagaaccagcaatttaggctttcctctatgt

tctcattcccactgctcttatgccctctctttcttcctcttcctagctcgcttttgagat

cccacatcgaatatgtgcacacatatatttggtttctaagctgaagtaaccaagcaagcc

ataaaaggaagaggaaggagtgctcgagaacaataggaatgggaggcatcgggaaacacc

aaagcaagttctcaccttcaacactagaggtgaaaacaaagcaagttggctttcctccat

gttcccactcccactgctcttatgccctcttttcttccacttcccggcttgccttcaaac

tctcaagttatctaaatatgtggacacatgtctactgtttctccatcaaagctactatgc

aatccaaaaggaagaggaaggagtgcacaacaacggtaggaatgggaggcatcaggaaag

gacccatgcctaatatttgcacctccaacactcgaggtgcaaacaaacaagcagggcttt

cctccatgttcccattcccagtgctcttgagctctctcattcttcctcttcctggcttac

tttcagtgcctacaaatctagagaagcaaaagctctcggaaggacaagcttgaattgttg

gacaaagtgtttcagctttgaggtataaataatgggtggtgcgccatatgcagaacctta

gagggcaaagcaatgaagacacccggtgcatgagggcaccattttacagtgagtgataat

ggggccacataatcaccggcggtgtcctgttgtcctatactcttaatttagggcatgtgc

gtaaagatctcttctggttacctttattacctatatccgaaactggtacataatatctgt

accagccttttttctagaacaatatctgtgtcggcctgtgcatttatggtgtcaaaatat

gtagcaataagaagttatgagctaaaagaatatgaaattggataaatcaaaccctgtaag

taaacttggaaaaaatacaaaacagtgggggcaaagaaccaccaaagtgacaagtaattg

gaacacaaggtactttgggcataagctgcattattatagatgacaaaatgggattacaac

atgataagagagatctcatatctgcaaaaccaaaatgtctatagtccattttattaagaa

aagtgtgtatagtcctattgttttttaagtaattgtagtatccaaattctgcaggctgta

cacaactacatggcatgagaaattatagggaaaaacatatctccaaagaaaacatcaaac

acatgatggtatcacatattcagtctaaactcattaatacgcacagtattttaagaacat

ggttcggacacaacggaagaacatgatcccgaagagagtatggtagttcttccacccatc

aaagtacttcccagttcaaccgtagctcggccaccatccctccaagattaaatcacccag

gttgggatgcaaatagccagcagggttccagagtggggaaataagccttatcccgcacat

ctaaacaaatatgtgaaaaacaacaccaatttatgatcaagtgtacaaattccattgaag

ttgacaattctcgaagttgcagatatatctcgttgcagtcatggttgtttcttgctagcc

tgcgtatttatccattagtttgctatcccatgtgtgaactctttaggaactgatgataaa

aacaatctgaacgcacatattattacatgttttgtgtgtcaattaccatttttcgcactc

cgatttctgctggtagtcttagctatatggtggtcagagtggtgtgtcattgatgtgatt

aactaatacagtaatacacttacatgcatttaccatgtgactctgtctaaactcctcagt

cctcacatcatgcttgacctgcagcgattggctcaatctaccattcaaattctacttttt

ttttagagcaactggcaagagctctgccttttcacatagaagagaattgaccaatttata

aggaaacccggccgaaaacagaccattcaaatactatttagtttagaacttacagtcaac

tccgtttattggatatatgtgttattggtctatattttagttagtgattattcttaatga

aatttgcacatcttgaaggcaacggatggttcctgctagctttgctttatagatttgtgc

cgtctgtgtagtatatacaagcaggagcatatcatattgcagtgttatctcatattacta

tattgctttacag

CATACAGAAGGACCCCGCCGAACGGATGTCTGCTTCAGAACTCTTG

gtgagcattcccttccactttacaagttgttgtagcgtgttcaactaaccgtggtaccgt

tgattctgtcgactagtgtgtgtgctgaatcgtcgttcgatgttgttgtcgcag

AACCATGCCTTCATCAAGAAGTTCGAGGACAAGGACCTAGACCTGCGGATCCTCGTCGAG

AGCCTCGAGCAGCCCATGAACGTGCCCGAGTGAGGCCGTCGCTCGGAGAGGTGAGGCCTT

GCGACGAGTTTCTTCCAGGGCTGCCCTCATGACGGCAATTGTGTATTGCTGTGAACCTGA

TGAATGAGTGACCGACCCCCCACCCCTGTATGTGATGATGTTTATGTTGGAAAAATTCCT

AGAGGATTGGAATGGATTATTAATGTATGGTAATGACTTGAGGCCCTTTTGTTGCGCGTC

TTGTTATGAATGAATCTGGTGTGGTTAGTAATTTGTGGTGCATGAGAGTCGAACGAACCG

TGATGTTG

**>TaMKK10-2(4AS) TRIAE_CS42_4AS_TGACv1_307146_AA1017600.1**

CACTCACCCGCCCCCATATATACCAGCAGGGAGCACTCCGCCTCCATCACTAGTCAGCTG

CCTCCGGAGTTCACCTCACCCACCCACCCACCTCGCCTCACCTCTCCCGCCCAACTCCCT

CCATTCCTTCCCATGGCTCTCGTCCGGCAGCGCCGCCAGCTACCCCACCTCACCCTCCCG

CTCGACCACTTCGCCCTGCGCGCCCCGCCGCCGCCGCTGCCCCCCGCCGTCGCGCCGTGC

GCCCCCGCCGAGGGCCTCGCCCGCCTCTCCGACTACGAGCGGATCTCCCAGCTCGGCCAG

GGCAACGGCGGCACCGTCTTCAAGGCGCGCCACCGCCGCACCGCGCAGCACTTCGCGCTC

AAGCTCTTCGCCGCCGGGGACGGGGACCCCTCCGCGGCCCGCGAGGCCGAGATCCTCATG

CTCGCCTCGGGCGCGCCGCACGTGGTGCGCCTCCACGCCGTCATCCCGTCGCCCGCCGCG

GAGCAGCCGGCCGCGCTGGCGCTGGAGCTCGTGTCGGGGGGCTCCCTCGCGGGCCTCCTC

CGCGCGCTGGGCCGGCCCATGGGGGAGCGCCCCATCGCCGCCGTGGCGCGGCAGGCGCTG

CTGGGGCTGGCGGCCCTGCACGCGCTCCGCGTCGTGCACCGCGACCTCAAGCCGGCCAAC

CTGCTGGTCGGCGCGGGCGGCGAGGTGAAGATCGCCGACTTCGGCGCCGGCAAGGTACTG

CGGCGGCGGCTCGACCCCTGCGCGTCCTACGTCGGCACGGCCGCCTACATGTCCCCGGAG

CGGTTCGACCCGGAGGCCTACTCCGGGGACTACGACCCGTACGCGGCGGACGTGTGGAGC

CTCGGGATGGCGATCCTGGAGCTGTACCTGGGCCACTTCCCGCTCCTCCCGGCGGGGCAG

CGCCCGGACTGGGCCGCGCTCATGTGCGCCATCTGCTTCGGCGAGGCGCCCGAGGCCCCC

GCCGCGGCGTCGGACGAGTTCCGTGACTTCGTGGCCCGCTGCCTGGAGAAGAAGGCCGGG

CGGCGCGCGTCCGTGGCGGAGCTGCTCCAGCACCCGTTCATCGCGGAGCGCGACGCGGAG

GAGGCGCAGCGCTGCCTCGCCGCGCTCGTGGCGGAGGCGGCGGAGCTGGGCGACCAGTAG

CCTAGCCAAACCGCCCGCCGGTTAATTAGTAGTCGGAGAGTTAGGAGTCCATCCATCCAA

GCTGATGTGTACATAATCCGCGCCACGGGGAGGAATAAACCTGTCCCATGTGGCGTTTTT

CTTTTCACCTTTTTGCCGTACCCAATGATTATTCCTTCGTTGTTTCACGTGAATTTGCAA

GGGACAGTTTTGATAATTGATGGCACCTTCTTCATCAGAATCAGAAGCATGGCATATGGC

GTCAGTGGAATGTTCTGATCGATCTGTCATGTTTTAAGGAAATTTCTCGAGCTATAAACC

TGTGAAAGGCTC

**>TaMKK10-2(4BL) TRIAE_CS42_4BL_TGACv1_323005_AA1073590.1**

TACGCGAATTCCGCTCGCTTCCGCTCACCTGCCACCCGTCGCCGTCCCTTGCGTCCCACA

CTCCATATATACCAGCAGCGAGCACTCCGCCCCTTTCACTAGTCAGTTGCCTCCCTCCGG

AGCTCACCTCACCCACCCACCTCGCCTCCCCCCCCAACGCCACCCATCCCATCTCATGGC

TCTCGTCCGGCAGCGCCGCCAGCTACCCCACCTCACCCTCCCGCTCGACCACTTCGCCCT

GCGCGCCCCGCCGCCGCCGCCCCCCGCCGTCGCGCCGTGCGAGGGCCTCGCCCGCCTCTC

CGACTACGAGAGGATCTCCCAGCTCGGCCAGGGCAACGGCGGCACCGTCTTCAAGGCGCG

CCACCGCCGCACCGCGCAGCAGGTCGCGCTCAAGCTCTTCGCCGCGGGGGACGGGGATCC

CTCCGCGGCCCGCGAGGCCGAGATACTCATGCTCGCCTCGGGCGCGCCGCACGTCGTGCG

CCTCCACGCCGTCATCCCGTCGCCCGCGGCGGAGCAGCCGGCGGCGCTGGCGCTCGAGCT

CGTGTCGGGGGGCTCCCTCGCGGGCCTCCTCCGCGCGCTGGGCCGGCCCATGGGGGAGCG

CCCCATCGCCGCCGTGGCGCGGCAGGCGCTGCTGGGGCTCGCGGCCCTGCACGCGCTCCG

CGTCGTGCACCGCGACCTCAAGCCCGCGAACCTGCTGGTCGGCGCGGGCGGCGAGGTGAA

GATCGCCGACTTCGGCGCCGGCAAGGTCCTGCGGCGGCGGCTGGACCCCTGCGCGTCCTA

CGTCGGCACGGCCGCCTACATGTCCCCGGAGCGGTTCGACCCGGAGACCTACTCCGGCGA

CTACGACCCGTACGCGGCGGACGTGTGGAGCCTCGGTATGGCGATCCTGGAGTTGTACCT

GGGCCACTTCCCGCTCCTCCCCGCGGGGCAGCGCCCGGACTGGGCCGCGCTCATGTGCGC

CATCTGCTTCGGCGAGGCGCCCGAGGCCCCCGCCGCGGCGTCGGACGAGTTCCGGGACTT

CGTGGCCCGGTGCCTGGAGAAGAAGGCCGGGCGGCGCGCGTCCGTGGCGGAGCTGCTCGG

GCACCCCTTCATCGCGGAGCGCGACGCGGAGGAGGCGCAGCGCTGCCTCGCCGCGCTGGT

GGCGGAGGCGGCGGAGCTGGGCGACCAGTAGCCGCCGGTTAATTAGTAGGAGGAGAGTTA

GGAGTCCATCAAAGCTGATGTGTACATAATCTTCGCCACGGGGAGGAATAAACCTGTCCC

ATGTGGCGTTTCCCTTTTCACCTTTTTGCCGTATCCAAGGATTCCTTCGTTTTTTCACGT

GAATTGCAAGCGACAGTTTTGAGAATTGATACCTTCTTCATCAGAATCAGAAGCATAAGC

ATAACCCGTAAAAGGCTCAGTTCCTATCGGGAACAGCAGAGCGAACAGCAGACACAAGAA

ACATGCATTGCACAA

**>TaMKK10-2(4DL) TRIAE_CS42_4DL_TGACv1_344093_AA1143600.1**

GTCGCCGTCCCCCCTCCATATATACCAGCAGGGAGCACTCCGCCTCTATCACTAGTCAGC

TGCCTCCGGAGTTCACCTCACCCACCCACCTCACCTCTCCTCTCCTGCCCAACTCCCATC

TCATGGCCCTCGTCCGGCAGCGCCGCCAGCTACCCCACCTCACCCTCCCGCTCGACCACT

TCGCCCTGCGCGCCCCGCCGCCGCCGCCGCCCCCCGCCGTCGCGCCGTGCGAGGGCCTCG

CCCGCCTCTCCGACTACGAGAGGATCTCCCAGCTCGGCCAGGGCAACGGCGGCACCGTCT

TCAAGGCGCGCCACCGCCGCACCGCGCAGCAGGTCGCGCTCAAGCTCTTCGCGGCCGGGG

ACGGGGACCCCTCCGCGGCCCGCGAAGCCGAGATACTCATGCTGGCCTCGGGCGCGCCGC

ACGTCGTGCGCCTCCACGCCGTCATCCCGTCGCCCGCGGCGGAGCAGCCGGCCGCGCTGG

CGCTGGAGCTCGTGTCGGGGGGCTCCCTCGCGGGCCTCCTCCGCGCGCTCGGCCGGCCCA

TGGGGGAGCGCCCCATCGCCGCCGTGGCGCGGCAGGCGCTGCTGGGGCTCGCGGCCCTGC

ACGCGCTCCGCGTCGTGCACCGCGACCTCAAGCCGGCCAACCTGCTCGTCGGCGCGGGCG

GCGAGGTGAAGATCGCCGACTTCGGCGCCGGCAAGGTCCTGCGGCGGCGGCTGGACCCCT

GCGCGTCCTACGTCGGCACGGCCGCCTACATGTCCCCGGAGCGGTTCGACCCGGAGGCCT

ACTCCGGCGACTACGACCCGTACGCGGCGGACGTGTGGAGCCTCGGGATGGCGATCCTGG

AGCTGTACCTGGGCCACTTCCCGCTCCTCCCCGCGGGGCAGCGCCCGGACTGGGCCGCGC

TCATGTGCGCCATCTGCTTCGGCGAGGCGCCCGAGGCCCCCGCCGCGGCGTCGGACGAGT

TCCGGGACTTCGTGGCCCGCTGCCTGGAGAAGAAGGCCGGGCGGCGCGCGTCCGTGGCGG

AGCTGCTCCAGCACCCGTTCATCGCGGAGCGCGACGCCGAGGAGGCGCAGCGCTGCCTCG

CCGCGCTGGTGGCGGAGGCGGCGGAGCTGGGCGACCAGTAGCGTAGCCAAACTGCCCGCC

GGTTAATTAGTAGGACAGTTTTAGGAGTCCATCGAAGCTGATGTGTACATAATCTGCGCC

ACGGGGAGGAATAAACCTGTCCCATGTGGCGTTTTCCTTTTCGACTTTTTGCCGTATCCA

AGGATTCCTTCGTTTTTTCACGTGAATTGCAAGAGACAGTTTTGAGAATTGATGGTACAT

TCTTCATCAGAATCAGAAGCATAACATACGGTGTTTGTGGAATGTTCTGATCGATCTGTC

ATGTTTAAGGAAATTTCTCGAGCTATAATAAACCCGTAAAAGGCTCAGTTC

**>TaMKK10-1/3(4AL) TRIAE_CS42_4AL_TGACv1_288275_AA0943400.1.1**

AAGCAAAGCAGCAGCAGCTCAGATCGCTCAGCGTTACGGTGGTGATCATCAGCCTACGGT

GGTGACAGACACATGGCTCTGATCAGGGAGAAGAGGCTTCCGCAGCTGCACCTCGCGCTG

CCCGTCCCGTCCCGCGCCGCCGCGCAGGAGCTCGGCGTCCTCGCCCGGCGCCCCAACCCG

ACGGCCACCAAGGCCTCCACCCCGTCGGCGCTGTCCAGCCAGTTACGCCTCGCCGACTTC

GACAAGCTCGCCGTCCTGGGCCGCGGGAACGGCGGCACCGTCTACAAGGTGCGCCACCGG

GAGACGTGCGCGCTCTACGCGCTCAAGGTCCAGCACTACGGCGACCCCGCCGCGGCCGCC

GAGGCCGACGTCCTCGGCCGCACGGCCTCGCCATTCGTCGTCCGGTGCCATTCCGTGCTC

CCCGCCGCGGCATCCGGTGACGTCGCTCTACTCCTCGAGCTGGTGGACGGCGGGTCGCTC

GACTCCATCCGGAGCCGCCGCGGCGCGTTCACGGAGGCCGCGCTCGCGGAGGTGGCGGCG

CAGGCGCTGTCGGGGCTGGCGTACCTCCACGCCCGCCGCATCGTGCACCTCGACATCAAG

CCGGCGAACCTCCTCGCCAGCACGGCCGGGGAGGTGAAGGTCGCGGACTTCGGCATCGCC

AGGGTGCTCGCACGCGCCGGCGACCACTGCACGTCGTACGCCGGCACCTCCGCGTACATG

AGCCCGGAGCGCTTCGACCCGGAGGCGCACGGGGGGCACTACGACCCGTACGCCGCCGAC

GTGTGGAGCCTGGGGGTCACGCTCCTTGAGCTCTTCATGGGCAGGTACCCGCTCCTCCCC

GCCGGGCAGCAGCCGACCTGGGCCGCGCTCATGTGCGCCATCTGCTTCGGCGAGCCGCCC

GTGCTGCCCGACGGCGCGGCCTCGCCGGAGCTCCGGGGGTTCGTCGCCGCGTGCCTGCAA

AAGGACTACCGCAACAGGGCGTCCGTGGCGGAGCTGCTTGCTCACCCGTTCGTGGCCGGG

AGGGACGTGGCAGCGTCGAAACGCGCGCTCCGGAAGCTGGTCGCCGACGCCTCGTCGTCG

TTGTAGTGCCGGCATGCATACGTGATCCCGCTGGGCGGCAGAGCCATGCACATACATGTA

GGATCAGTTTTGCTAGCCATGTAGAGTGCTACTACATAGGATGCAGGGCATACCACGTCA

TTAGTTAGCTTAGTGATGACCTAGGAGTACTTAGTCACTTGTTCAGACATTGGTGATGTT

TTGATTCCACACCACTGTAAATTACCTTTTGTCCCTCAACTATCGACTAATATTTCATAA

ACTTCAATATGGGAC

tgttaaaccctcgatcaagtcagaccggtagagctgtgtcagtcttttag..........

**>TaMKK10-1/3(4BSc1) TRIAE_CS42_4BS_TGACv1_327917_AA1078630.1.1.X (based on AA1078630.1.1)**

ctggtcatggcctcctcccgcaatcaaggtcggactaaaccgagcttgcctcaccggcct

ACAGCAGCCCTAACATCGGCGTCCTTGGCCGGCGCTCCAACCCCACGGCGGCCAAGGCCT

CCACCCCGTCGACGTTGTCCAGCCAGTTCCGCCTCGCTGACTTCGACAAGCTCGCCGTCC

TGGGCCGCGGGAACGGCGGCACCGTCTACAAGGTCCGCCATCGGAAGACGAGCACGCGCA

CTCTACGCGCTCAAGGTCCAGCACTACGGCGACCCCGCCGCCGCCGCCAAGGCGGACGTT

CTCAGCCGCACTGCCTCGCCATTCGTCGTCCGGTGGCACTCCGTGCTCCTTGCCACAGCA

TCTGGCGACGCCCCCCTGCTCCTCGAGCTGGTCGACGGGGGGTCCATCAACTCCGTCAGG

AGCCGCCGCGGCGCGTTCACGGAGGCCGCCCTCACGGAGGTGACAACGCAGGTGCTGTCG

GGGCTGGCGTACCTCCACGCCCGCCGCATCATGCACCTCGACATCAAGCCGGCCAACCTC

CTCGTGAGCACCGCCGGGGAGGTCAAGGTCGCCGACTTCGGCATCGCCGAGCTGCTCGCC

CGCACCGGCGAGCACTACGCGTCGTACATCGGCACCTCCGCGTACATGAGCCTGGAGCAC

TTCGACCAGGAGGCGCACCCAGGGCCCTATGATCCGTACGCCGCCGACGTGTGGAGCCTG

GGTGTCACACTCCTTGAGCTCTTCATGGGCAGGTACCCGCTCCTGCCCGCCGGGCAGCAG

CCGACTTGGGTCGCGCTCATGTGCGCTATCTTCTTCGGCAACCGCCAGTGCTGCCCGACG

GCGCAGCCTCGCCGGAGCTCCGGGGATTCGTTGCCGCGTGCCTGCAAAAGGACTACCGCA

AGAGGGCGTCCATCGTGGAGCTGCTTGCTCACCCATTCGTCGCCGTGAGGGACGTGGCAG

CGTCGAAACACACACTATG

**>TaMKK10-1/3(4BSc2)TRIAE_CS42_4BS_TGACv1_328797_AA1093760.1.1**

CCAAAGCAGAGCAGCAGCAGCTCAGATCACTCGGCGTTACGGTGGTGATCATCAGCCTAC

GGTGGTGACTGACGCATGGCTCTGATCAGGGAGAAGAGGCTTCCGCAGCTGCACCTCGCG

CTGCCCGTCCCGTCCCGCGCCGCCGCGCAGGAGCTCGGCGTCCTCGGCCGGCGGCCCAAC

CCCACGGCCACCAAGGCCTCCACCCCGTCGGCGCTGTCCAGCCAGTTCCGCCTCGCCGAC

TTCGACAAGCTCGCCGTCCTGGGCCGCGGGAACGGCGGCACCGTGTACAAGGTCCGCCAC

CGGGAGACGTGCGCGCTCTACGCGCTCAAGGTGCAGCACTACGGCGATCCCGCCGCGGCC

GCCGAGGCCGACGTCCTCAGCCGCACGGCCTCGCCATTCGTCGTCCGGTGCCACTCCGTG

CTCCCCGCCGCGGCATCCGGCGACGTCGCCCTGCTCCTCGAGCTGGTCGACGGGGGCTCG

CTCGACTGTATCAGGAGCCGCCGCGGCGCGTTCACGGAGGCCGCGCTCGCGGAGGTGGCG

GCGCAGGCGCTGTCGGGGCTGGCGTACCTCCACGCCCGCCGCATCGTGCACCTCGACGTC

AAGCCGGCGAACCTTCTCGCCAGCACGGCCGGGGAGGTCAAGGTCGCCGACTTCGGCATC

GCCAAGGTGCTCGCCCGCGCCGGCGACCACTGCACGTCGTACGCCGGCACCTCCGCGTAC

ATGAGCCCCGAGCGCTTCGACCCGGAGGCGCACGGTGGGCACTACGACCCGTACGCCGCC

GACGTGTGGAGCCTCGGGGTCACGCTCCTTGAGCTCTTCATGGGCAGGTACCCGCTCCTG

CCCGCCGGGCAGCAGCCCACCTGGGCCGCGCTCATGTGCGCCGTCTGCTTCGGCGAGCCG

CCCGTGCTGCCCGACGGCGCGGCCTCGCCGGAGCTCCGGGGATTCGTCGCCTCGTGCCTG

CAGAAGGACTACCGCAACAGGGCGTCCGTGGCGGAGCTGCTTGCTCACCCATTCGTGGCC

GGGAGGGACGTTGCAGCGTCGAAACGCGCGCTCCGGAAGCTGGTCGCCGATGCCTCGTCG

TCATTGTAGTGCCGGGATACGGATCCCGCTGGGCGGCACAGCCATGCACATATACTGATA

TACATAGAATCAATTTTGCTAGATATG

**>TaMKK10-1/3(4BSc3) TRIAE_CS42_4BS_TGACv1_328797_AA1093770.1.1.X (based on AA1093770.1.1)**

AAGCAAAGCAGCAGTAACTCGATCACTCAGCTCAGGCAGTCAGTTTGATCAGCCTACGGT

GGTGACAGACACATGGCTCTCAGGGAGAAGAGGCTTCCGCAGCTGCACCTCGCGCTGCCC

GTCCCGTCCCGCGCCGCCGCCCAGGAGCTCGGCGTCCTCGGCCGGCGCCCAAACCCCACG

GCCACCAAGGCCTCCACCCCGTCGGCGCTGTCCAGCCAGTTCCGCCTCGCCGACTTCGAC

AAGCTCGCCGTCCTGGGCCGCGGGAACGGTGCCACCGTCTACAAGGTCCGCCACCGGGAG

ACGAGCGCGCTCTACGCGCTCAAGGTCCAGCACTACGGCGACCCCGCCGCGGCCGCCGAG

GCGAACATCCTCAGCCGCACGGCCTCGCCATTTTGTCGTGCGGTGCCCTCCGTGCTCCCC

GCCGCAGCCTCCGGGGACGTCGCCCTGCTCCTCGAGCTGGTCGACGGGGGGTCGCTCGAC

TCCGTCAGGAGCCGCCGCGGCGCGTTCACGGAGACCGCGCTCGCGGAGGTGGCTGCGCAG

GCGCTGTCGGGGCTGGCGTACCTCCACGCCCGCCGCATCGTGCACCTCGACGTCAAGCCG

GCGAACCTCCTCGTCACCACCGCCGGGGAGGTCAAGGTCGCGGACTTCGGCATCGCCAAG

GTGCTCGCCCGCGCCGGCGACCACTACACGTCGTACGCCGGCACCTCCGCGTACATGAGC

CCGGAGTGCTTCGACCCGGAGGCGCACGGGGGGCACTACGACCC

gtacgccgccgacgtgtggagcctgggggtcacactccttgagctcttcatgggcacttg

ggcag

GTACACGCTCCTGCCCGCCGGGCAGCAGCCGACCTGGGCCCCGCTCATGTGCGCCGTCTG

CTTCGGCGAGCCGCCCGTGCTGCCCGACGCCGCAGCCTCGCCGGAGCTCCGGGGATTCGT

CGCCTCGTGTCTGCACAAGGACTACCGCAACAGGGCGTCCGTGGCGGAGCTGCTTGTTCA

TCCATTCGTCGCCAGGAGGGACGTGGTAGTGTCGAAACGCGCGCTCCGGAAGCTGGTCGC

CGACGCCTCGTCGTCGTTGTAGTGCCGGCATGTGTGATCGGTGATCCCGCTGGGCGGTAG

AGCCATGCACATATACTGATATACTCCATAGGATCAATTTTGCTATAGAGATGTACAGTG

CCATATATGGAGAGCTTAGTGATAACACACCGGTTAGCTTAGTGATCACCTAGGCTAGGA

TTAGGAATGTACTCCCTTGTTCAGACATTGGTGATGTACT

**>TaMKK10-1/3(4BSc4) TRIAE_CS42_4BS_TGACv1_329516_AA1102150.1.1**

GGCGGTGATCATCGGCCGACAGCGGTGACAGACACATGGCTCTGATACGTGAGAAGAGGC

TTCCGCAGCTGCACCTTTCGTTGCCTGTCCCGCCCCGCGCGGCCGCGCAGGAGCTCGGCG

TCCTTGGCCGGCGCCCCAACCCCGCGGTCGCGCCGGCGCCGACGGCCACCAAGGCGTCGA

CCCCGTCGGGGCTGTCCAGCCAGTTCCGCCTGGCCGACTTCGAGAAGCTTGCCGTCCTGG

GCCGCGGGAACGGCGGCACGGTCTACAAGGTCCGCCACCGGGAGACGTGCGCGCTCTACG

CGCTCAAGGTGCAGCACTACGGCGACCCCGCTGCGGCCAACGAGGCCGACGTCCTCGGCC

GCACCGGCTCGCCCTTCGTCGTCCGCTGCCACTCCGTGCTCCCCGCCGCGACCTCCGGCG

ACGTCGCGCTGCTCCTCGAGCTGGTGGACGGCGGGTCGCTCGACTCCGTCAGGAGCCGCC

GCGGCGCGTTCACGGAGGCCGCGCTTGCGGAGGTGGCGGCGCAAGCGCTGTCCGGGCTGG

CGTACCTCCACGCCCGCCGCATCGTGCACCTCGACGTCAAGCCGGCCAACCTCCTCGTCA

GCACCGCCGGGGAGGTCAAGGTCGCCGACTTCGGCATCGCCAAGGTGGTCGCCCGCGCCG

GCGACCACTGCACGTCGTACGCCGGCACCTCCGCGTACATGAGCCCCGAGCGCTTCGACC

CGGAGGCGCACGGCGGGCACTACGACCCGTACGCCGCCGACGTGTGGAGCCTGGGGGTCA

CGCTCCTTGAGCTCTTCATGGGCAGGTACCCGCTCCTCCCCGCCGGGCAGCGGCCGAGCT

GGGCGGCGCTCATGTGCGCCGTCTGCTTCGGCGAGCCGCCGGTGCTGCCCGCCGGTGCCT

CCTCGCCGGAGCTCCGGGGATTCGTCGCCTCGTGCCTGCAAAAGGACTACCGAAACAGGG

CGTCCGTCGCGGAGCTGCTTGCTCACCCATTCGTCGCCGGCAGGGACATGCCCGTGTCGA

AATGCGCGCTCCGGAAGCTGGTCGCCGACGCCTCGTCTTCGTTGTAGTGCAGGCATACGT

GATCGTGTTAGGCGGCAGGGCCATGCGCATAGGAGTACATAGGATCAATTTTGCTAGCCA

TGTACAATGCCATGTATGCAGAGCAGAACACATGAGTAGTTAGTTTAGGGATGACCTAGG

TCAGACACTGGTGATGTTTTAGCATTTGGGACTAGATGCATTTTGCATCACGGTGGAAAC

GGATAAACACATGTTTTTATTCCACACCACTGTAAAATAAGAAAATATTACCTTTTGT

**>TaMKK10-1/3(4BSc5) TRIAE_CS42_4BS_TGACv1_329775_AA1103890.1.1**

GCAGTTAAGCAGCGGCAGCGGCAGCTCAGATCACTCGGCTTACGGTGGTGATCATCAGTC

GACAGCGGTGACAGACAGACACATGGCTCTCATACGGGAGAAGAGGCTTCCGCAGCTGCA

TCTCTCGCTGCCCGTCCCGCCCCGCGCCGTTGCCCAGGAGCTCGGCGTCCTCGGCCGGCG

CCCAAACCCCGCGGTCGCGCCGGCGCCGACGGCCACCAAGGCCTCGACCCCGTCGGCTCT

GTCCAGCCAGTTCCGCCTGGCCGACTTCGAGAAGCTCGCCGTCCTGGGCCGCGGGAACGG

CGGCACGGTCTACAAGGTCCGCCACCGGGAGACAAGCGCGCTCTACGCGCTCAAGGTGCA

GCACTACGGCGACCCCGCCGCGGCCGCCGAGGCCGACGTCCTCAGCCGCACCGCCTCGCC

CTTCGTCGTCCGCTGCCACTCCGTCCTCCCCGCCGCGGCGTCCGGCGACGTCGCGCTGCT

CCTCGAGCTGGTCGACGGCGGGTCGCTCGACTCCGTCAGGACCCGCCGCGGCGCGTTCGC

GGAGGCCGCGCTCGCGGAGGTGGCCGCTCAGGCGCTGTCGGGGCTGGCATACCTCCACGC

CCGCCGCATCGTGCACCTCGACGTCAAGCCGGCCAACCTCCTCATCAGCACCGCCGGGGA

GGTCAAGGTCGCCGACTTCGGCATCGCCAAGGTGCTCGCCCGCGCGGGCGACTACTGCAC

GTCGTACGCCGGCACCTCCGCGTACATGAGCCCGGAGCGCTTCGACCCGGAGGCGCACGG

CGGGCACTACGACCCGTACGCCGCCGACGTGTGGAGCCTGGGGGTCACGCTCCTTGAGCT

CTTCATGGGCAGGTACCCGCTCCTCCCCGCCGGGCAGCGGCCGAGCTGGGCGGCGCTCAT

GTGCGCCGTCTGCTTCGGCGAGCCGCCCGCTGCCAGACGGTGACTCCTCGCCAGAGCTCC

GGGGGTTCGTCGCCGCGTGCCTGCAAAAGGACTACCGAAACAGGGCGTCCGTCGCGGAGC

TGCTTGCTCACCCATTCGTCGCCGGGAGGGACGTGGCACCGTCGAGATGCGCGCTCCGGA

AGCTGGTCGCCGACGCCTCGTCGCCGTCGTTGGAGTGCCGGCATACGTCATCGTATTGG

**>TaMKK10-1/3(4DSc1) TRIAE_CS42_4DS_TGACv1_362002_AA1175570.1.1**

CCCCAAAGCAGCAGCACTACGGTGGTGATCATCAGCCTACGGTGGTGACTGACACATGGC

TCTGATCAGGGAGAAGAGGCTTCCGCAGCTGCACCTCGCGCTGCCCGTCCCGTCCCGCGC

CGCCGCCCAGGAGCTCGGCGTCCTCGGCCGGCGCCCCAACCCCACGGCCACCAAGGCCTC

CACCCCGTCGGCGCTGTCCAGCCAGTTCCGCCTCGCCGACTTCGACAAGCTCGCCGTCCT

GGGCCGCGGGAACGGCGGCACCGTCTACAAGGTGCGCCACCGGGAGACGTGCGCGCTCTA

CGCGCTCAAGGTGCAGCACTACGGCGACCCCGCCGCTGCCGCCGAGGCGGACGTCCTCAG

CCGCACCGCCTCGCCATTCGTCGTCCGGTGCCACTCCGTGCTCCCCGCCGCGGCCTCCGG

CGACGTCGCGCTGCTCCTCGAGCTGGTTGACGGCGGGTCGCTCGACTCCATCAGGAGCCG

CCGCGGCGCGTTCACGGAGGCCGCGCTCGCGGAGGTGGCCGCGCAGGCGCTGTCGGGGCT

GGCGTACCTCCACGCCCGCCGCATCGTGCACCTCGACATCAAGCCGGCGAACCTCCTCGC

CAGCACGGCCGGGGAGGTGAAGGTCGCCGACTTCGGCATCGCCAGGGTGCTCGCCCGCGC

CGGTGACCACTGCGCGTCGTACGCCGGCACCTCCGCGTACATGAGCCCGGAGCGGTTCGA

CCCGGAGGCGCACGGGGGGCAGTACGACCCGTACGCCGCCGACGTGTGGAGCCTGGGGGT

CACACTCCTTGAGCTCTTCATGGGCAGGTACCCGCTCCTCCCCGCCGGGCAGAGGCCGAG

CTGGGCCGCGCTCATGTGCGCCGTCTGCTTCGGCGAGCCGCCCGTGCTGCCCGACGGCGC

GGCCTCGCCGGAGCTCCGGGGGTTCGTCGCCGCATGCCTGAGCAAGGACTACCGCAACAG

GTCGTCCGTCGCGGAGCTGCTTGCTCACCCGTTCGTCGCCGGGAGGGACGTGGCAGCGTC

GAAATGCGCGCTTCGGAAGCTGGTCGCCGACGCCTCGTCGCCGTCGTTGGAGTGCCGGCA

TACGTGATCGTGTTAGGCGGCAGGGCCATGCACATACACATAGGATCAGTTTTGCTAGCC

ATGTATAGTGCCATGTATGCAGGGCATAACCCATCTAATTAGTTTAGCGATGACCTAGGA

ATACTTAGTCACTAATTCAGACATAGGTGATGTTTTAGCTTTTGCGACTAGATGCGTTTT

GCATCACGTATAAACAGATGTTTTGATT

**>TaMKK10-1/3(4DSc2) TRIAE_CS42_4DS_TGACv1_362154_AA1177260.1.1**

GTGATCATCAGCCTACAGCGGTGAAAGACACATGGCTCTGATACGGGAGAAGAGGCTTCC

ACAGCTGCACCTCTCGCTGCCCGTCCCGCCCCGCGCCGCCCCGCAGGAGCTCGGCGTCCT

CGGCCGGCGCCCAAACCCCGCGGTCCCGCCGGCGCCGACGGCCACCAAGGCGTCGACCCC

GTCGGCGCTGTCCAGCCAGTTCCGCCTGGCCGACTTCGAGAAGCTCGCCGTCCTGGGCCG

CGGGAACGGCGGCACCGTATACAAGGTCCGCCACCGGGAGACGCACGCGCTATACGCGCT

CAAGGTGCAGCACTACGGCGACCCCGCCGCGGCCGCCGAGGCGGACGTGCTCAGCCGCAC

CGCCTCGCCCTTCGTCGTCCGGTGCCACTCCGTGCTCCCCGCCGCGGCGTCCGGCGACGT

CGCGCTGCTCCTCGAGCTGGTCGACGGCGGGTCGCTCGACTCCGTCAGGACCAGCCGCGG

CGCGTTCGCGGAGGCCGCGCTCGCGGAGGTGGCCGCGCAGGCGCTGTCGGGGCTGGCGTA

CCTCCACGCCCGCCGCATCGTGCACCTCGACGTCAAGCCGGGCAACCTCCTCGTGAGCAC

CGCCGGGGAGGTCAAGGTCGCCGACTTCGGCATCGCCAAGGTGCTCGCCCGCGCCGGCGA

CCACTGCGCGTCGTACGCCGGCACCTCCGCTTACATGAGCCCCGAGCGCTTCGACCCGGA

GGCGCACGGCGGGCACTACGACCCGTGCGCCGCCGACGTGTGGAGCCTGGGGGTCACGCT

CCTTGAGCTCTTCATGGGCAGGTACCCGCTCCTCCCCGCCGGGCAGCGGCCGAGCTGGGC

GGCGCTCATGTGCGCCGTCTGCTTCGGCGACCCGCCGGTGCTGCCCGAGGGCGCCTCCTC

GCCGGAGCTCCGGGGGTTCGTCGCCGCGTGCCTGCGCAAGGACTACCGCAACAGGGCGTC

CGTCGCGGAGCTGCTTGCCCACCCATTCGTCGCCGGGAGGGACGTGGCAGCGTCGAGATG

CGCGCTCCGGAAGCTGGTCGCTGACGCCTCGTCGCCGTCGTCGGACTGCCGGCATACGTA

ACGTGATCGTGTTAAGCGGCAGGGCCATCCACATACATAGGATCAATTTTGCTAGCCATG

TACAGTGCCACGTATGCAGAGCATAACACATCAGTTAGATTAGTGTTAACCTAGAAGCTA

CTATTAGGAATGTACGTACTCACTTGTGCAGACTAAATAAGAATTTCCGTA

**>TaMKK10-4(3B) TRIAE_CS42_3B_TGACv1_220919_AA0723740.1.1**

AAACATCATCCATTGCTCAATCATCAATTTCACATCATACTATTGCAAACACAGTTCTTG

CAAAACACAACGCACGTACACACGGCGATCAAGATCATGGCGGCTGCCAGAGAAAGACGA

CTACCGCAGCTTCACCTCACGCTCGACGCTCCCACGTGGGCCTTCCGGTGCCCCGCCCCG

GCGCCGGTCACCGCGGCGACGCCGTCCACCTCGGCGGCTCGGCCGGACGGCGAGTTCCGC

CTGAGCGACTTCGACAGGCTCTCCGTCCTTGGGCGCGGGAACGGCGGCACCGTCCACAAG

GTCACGCACCGCCGCACGTCGGCGCTGTATGCGCTCAAGATCATTCACCGCGGCCACCCC

GGCGCTGACGAGGAGGTAGAAGTTGTACGCCGCGTCGACTCGCCTCACATCGTCCGGTGT

CACTCGGTCCTCCTGACGGGTTCCGGCGACTCAGCGTTACTCCTGGAGCTGATGGACGGC

GGTTCCCTCGACTCGCTCGTCCGCGCCGGCCAGGGAGGCTTCCCGGAGTCGGCTCTGGCG

GAGGTGGCGGCGCAGGCGCTGTCCGGCCTGGCGTACCTCCGCGCACGCCGCGTCGTCCAC

CGGGACATCAAGCCTGCGAACCTTCTGGTCAACAGTGCCGGGCAGGTGAAGATCGCCGAC

TTCGGCATAGCCGAGGTCGTCTCCCGCGCCGGCAAGTACCGCGCGGCCTACGAGGGCACC

GCCGCGTACATGAGCCCTGAGCGCTTCGACACGGAGCGGTCGTTGCACGTCGCCGGCGAC

GAGGAGGGCCCCGTCGACCCCTACGCCGCCGACGTGTGGGGGCTGGGGGTTACCGTCCTG

GAGCTCCTCATGGGGCGGTACCCGCTGCTCCCCGCCGGGCAGGAGCTGAGCTGGGCGGCG

CTCATGTGTGCCGTCTGCTTCGGCGAGCTGCCGGCACTTCCCGACGGTGCGGCATCGCCG

GAGCTCCGGGGTTTCGTGGCCGCTTGCCTGCAGAAGGACCATCGGAAGCGAGCGTCCGTC

GCAGAGCTGCTCGTGCACCCGTTCGTCGCCGGGAGAGACGTTGCGGCGTCGAGACGGGCG

CTACGCGAAGTGATCGACCAGCGGTGTCGATAAGCACGCGCGTGTAAATGACTAAATGTT

GAACTGTCAC

**>TaMKK10-4(4ALc1) TRIAE_CS42_4AL_TGACv1_289532_AA0972640.1.1**

AAACATCATCCGTTACTCAATCATCAATTTCATCATTCTATTGCCAATACTGTTCTTGCG

AAACACACGGCGACTCAAGATCATGGCGGCTGCCAGAGAAAGGAGGCTACCGCAGCTTCA

CCTCACGCTCGACGCGTCCACGTGGGCCTTCCGGTGCCCCGCCTCGGCTACGGTCGCCGC

GGCGACGCCGTCCACCTCGGCGGCTCGGCCGGACGGTGAGTTCCGCCTGATCGACTTCGA

CAGGCTCTCCGTCCTTGGTCGCGGGAACGGCGGCACCGTCCACAAGGTCTCGCACCGCCG

CACGTCGGCGCTGTACGCGCTCAAGATCATTCACCGCGGCCACCCCGGCGCCGACGAGGA

GGTAGAAGTTGTACGGCGCGTCGACTCCCCGCACATCGTCCGGTGCCACTCGGTGCTCCC

GACGGCGTCCGGCGACTCCGCCTTACTCCTCGAGCTGATGGACGGCGGTTCGCTCGATTC

GCTCGTCCGCGCCGGCCAGGGAGGCTTCCCGGAGGAGGCCCTTGCGGAGGTGGCCGCGCA

GGCGTTGTCTGGCCTAGCATACCTCCGCGCCCGCCGCGTCGTCCACCGCGACATCAAGCC

GGCCAACCTCCTTATCAACAAAGCCGGGCAAGTCAAGATCGCCGACTTCGGCATAGCCGA

GGTCGTCTCCCGCGCCGGCAAATACCGCGCGGCCTACGAGGGCACCGCCGCGTACATGAG

CCCCGAGCGCTTCGACACGGAGCGGTCGTTGCAGGGCGACGGCGACGAGGAGGGCCCCGT

CGACCCCTACGCCGCCGACGTGTGGGGGCTGGGGGTGACCGTCCTGGAGCTCCTCATGGG

GCGGTACCCGCTGCTCCCCGCCGGGCAGGAGCTGAGCTGGGCGGCGCTCATGTGCGCCGT

GTGCTTCGGCGAGCTGCCGGCACTTAGCGACGGTGCAGCGTCGCCGGAGCTCCGGAGTTT

CGTGGCCGCTTGCCTACAGAAGGACCACCGGAAGCGAGCTTCCGTCTCAGAGCTGCTCGT

GCACCCATTCGTCGCCGGGAGAGACGTAGCGGCGTCGAGACGGGCGCTAGGCGAAGTGAT

CAAGCAGCGGTGCTGATAGGCGTGTAAAACTGTAAATGTTGAACTGTCACATTCTCTAAT

CGTAAATACCTAAGCTAGGCATTTGCACATACAAATAGTGTACCATACTTTGAACTTGCG

AGGGTCTTACTCTCTAAGGGTGTATTTGATTGGGGAACAAAGTGGAATGGAATGT

**>TaMKK10-4(4ALc2) TRIAE_CS42_4AL_TGACv1_291020_AA0991400.1**

AACATCATCCGTTACTCGATCATCAATTTCATCATACTATTGCCGATACAGTTCTTGCGA

AACACACACACATACACACGGCGACTCAAGATCATGGCGGCTGCCAGAGAAAGACGACTG

CCGCAGCTTCACCTCAAGCTCGACGCTCCCACGTGGGCCTTCCGGTGCCCCGCCCCGGCG

CCGGTCACCGCGGCGACGCCGTCCACGTTGGCGGCTCGGCCGGACGGCGAGTTCCGCCTG

ATCGACTTCTACCGGCTCTCCGTCCTTGGTCGCGGGAACGGCGGCACCGTCCACAAGGTC

TCGCACCGCCGCACGTCGGCGCTGTACGCGCTCAAGATCATTCACCGCGGCCACCCCGGC

GCCGACGAGGAGGTAGAAGTTGTACGGCGCGTCGACTCGCCGCACATCGTCCGGTGCCAC

TCGGTGCTCCCGACGGCGTCCGGCGACTCCGCCTTACTCCTCGAGCTGATGGACGGCGGT

TCGCTCGACTCGCTCGTCCGCGCCGGCCAGGGAGGCTTCCCGGAGGAGGCGCTGGCGGAG

GTGGCCGCGCAGGCGCTGTCCGGCCTGGCGTACCTCCGCGCCCGCCGCGTCGTCCATCGC

GACATCAAGCCTGCGAACCTCCTGGTCAACAGTGGCGGGCAGGTCAAGATCGCCGACTTC

GGCATAGCCGAGGTCGTCTCCCGCGCCGGCAAATATCGCGCGGCCTACGAGGGCACCGCC

GCGTACATGAGCCCCGAGCGCTTCGACACGGAGCGGTCGTTGCAGGGCGACGGCGACGAG

GAGGGCCCCGTCGACCCCTACGCCGCCGACGTGTGGGGGCTGGGGGTGACCGTCCTGGAG

CTCCTCGTGGGGCGCTACCCGCTGCTCCCCGCCGGGCAGGAGCTGACCTGGGCGGCGCTC

ATGTGCGCCGTCTGCTTCGGCGAGCTGCCGGCACTTCCCGACGGGGCGGCGTCGCCGGAG

CTCCGGAGTTTCGTGGCCGCTTGCCTGCAGAAGGACCACCGGAAGCGGGCGTCCGTCGGG

GAGCTGCTCGTGCACCCGTTCGTCGCCGGGAGAGACGTAGCGGCGTCGAGACGGGCGCTA

CGCAAATTGATCGAGCAGCGGTGTTGATAGACACGCGCGTGTAAATGTTGAACTGTCACA

TTCTCGAATCGTGAATACCTAAGCTAGGCATTTGCACATACAGATAGTGGACCATACTTT

GAGCTTCGAAGGGTCTCGCTCCCTGAGAGCATCTCCAGCCCATACCTTATACACTACCGG

AATAACCTTATATGCCTACG

**>TaMKK10-4(4BS) TRIAE_CS42_4BS_TGACv1_329491_AA1101870.1.1**

ATCATCCATTACTCGATCATCAATTTCATCATACTTTTGAGAATACAGTTCTTGCAAAAC

ACACACACACATACACACGGCGATCAAGATCATGGCGGCTGCCAGAGAAAGACGTCTACC

GCAGCTTCACCTCACGCTCGACGCTCCCACGTGGGCCTTCCGGTGCCCGGCCCCGGCGCC

AGTCACCGCGGCGACGCCGTCCACCTCGGCGGCTCGGCCGGACGGCGAGTTCCGCCTGAG

CGACTTCGACAGGCTCTCCGTCCTTGGGCGCGGGAACGGCGGGACCGTCCACAAGGTCTC

GCACCGCCGCACGTCGGCGCTGTACGCGCTCAAGATCATTCACCGCGGCCATCCCGGCGC

CGACGAGGAGGTAGATGTTGTGCGGCGCGTCGACTCCCCATACATCGTCCGGTGCCACTC

GGTACTCCCGACGGCTTCCGGCGACTCCGCCTTACTCCTCGAGCTGATGGACGGCGGTTC

GCTCGACTCGCTCGTCCGCGGCGGCCAGGGAGGCTTCCCGGAGGAGGCGCTGGCGGAGGT

GGCGGCGCAGGCGCTGTCCGGCCTGGCCTACCTCCGCGCCCGCCGCGTCGTCCACCGCGA

CATCAAGCCTGCGAACCTTCTGGTCAACAGGGCCGGGCAGGTCAAGATCGCCGACTTCGG

CATAGCCGAGGTCGTCTCCCGCGCCGGCAAGTACCGCGCGGCCTACGAGGGCACCGCCGC

GTACATGAGCCCCGAGCGCTTCGACACGGAGCGGTCGTTGCACGGCGACGGCGACGAGGA

GGGCCCCGTCGACCCCTACGCCGCCGACGTGTGGGGGCTGGGGGTGACCGTCCTGGAGCT

TCTGATGGGGCGCTACCCGCTGCTCCCCGCCGGGCAGGAGCTGACCTGGGCGGCGCTCAT

GTGCGCCGTCTGCTTCCGCGAGCTGCCGGCACTTGCCGACGGTGCAGCGTCGCCGGGGCT

CCGGGGTTTCGTGGCCGCTTGCCTGCAGAAGGACCACCGGAAGCGGGCGTCCGTCGGGGA

GCTGCTCGTGCACCCGTTCGTCGCCGGGAGAGACGTAGCGGCGTCAAGACGGGCGCTACG

CGAGGCGATCGAGCAGCGGTGTCGATAAGCAATTAAGCACGCGCGTGTAAATGTTGAACC

GTCACATTCTCGAATCGTAAATACCTAAGCTAGGCATTTGCACATAATACAGATAGTGGA

CCATACTTTGAACTTGTAAGGGTATCACTATGTGAGAGCATCTTTAGCAGATTTCTTATA

TCACGGTACCGTAAAAGCATTATAAGAAAAATTCTTAAACCTGTTTTACGGTACCATCCG

GACATCAGCGGAATAGATCGCGTAAATGTTT

**>TaMKK10-4(4DS) TRIAE_CS42_4DS_TGACv1_361832_AA1173360.1**

AAAAAAACATCATCCATTACTCGATCATCAATTTCATCATACTTTTGCGAATACAGTTCT

TGCAAAACACACACACACACACATACACACGGCGATCAAGATCATGGCGGCTGCCAGAGA

AAGACGACTACCGCAGCTTCACCTCACGCTCGACGCCCCCACGTGGGCCTTCCGGTGCCC

GGCCCCTGCTCCGGTCACCGCGGCGACGCCGTCCACGTCGGCGGCTCGGCCGGACGGCGA

GTTCCGCCAGAGCGACTTCGACAGGCTCTCCGTCCTTGGGCGCGGGAACGGCGGCACCGT

CCACAAGGTCTCGCACCGCCGCACGTCGGCGCTGTACGCGCTCAAGATCATTCACCGCGG

CCACCCCGGCGTCGACGAGGAGGTAGAAGTTGTACGGCGCGTCGACTCGCCGCACATCGT

TCGATGTCACTCGGTCCTCCCGACGGCGTCCGGCGACTCCGCCTTACTCCTCGAGCTGAT

GGACTGTGGTTCCCTCGACTCGCTCTTCCGCGCCGGCCAGGGAGGCTTCCCGGAGGAGGC

GCTGGCGGAGGTGGCGGCGCAGGCGCTGTCCGGCCTGGCGTACCTCCGCGCCCGCCGCGT

CGTCCACCGCGACATCAAGCCTGCCAACCTTCTGGTCAACAGTGCCGGGCAGGTCAAGAT

CGCCGACTTCGGCATCGCCGAGGTCGTCTCCCGCGCCGGCAAATACCGCGCGGCCTACGA

GGGCACAGCCGCGTACATGAGCCCCGAGCGCTTCGACACGGAGCGGTCGTTGCAGGGCGA

CGGCGACGAGGAGGGCCCCGTCGACCCCTACGCCGCCGACGTGTGGGGGCTGGGGGTGAC

CGTCCTGGAGCTCCTCATGGGGCGGTACCCGCTGCTCCCCGCCGGGCAGAAGCCGAGCTG

GGCGGCGCTCATGTGCGCCGTCTGCTTCGGCGAGCTGCCGGCACTTCCCGACGGCGCGGC

GTCGCCGGAGCTCCGTAGTTTCGTGGCCGCTTGCCTGCTGAAGGACCACCGGAAGCGAGC

GTCCGTCGGGGACCTGCTCGTGCACCCGTTCGTCGCCGGGAGAGACGTAGCGGCGTCGAG

ACGGGCGCTACGCGAAGTGATCGAGCAGCGGTGTGGATAAGCACGCGCGTGTAAATGTTG

AATTGTCACGTTCTCGAATCGTAAATACCTAAGCTAGGCATTTGCACATACAGATAGTGG

ACCATACTTTGAGCTTGGAAGGGTCTCAATCTCTAAGAGCATCTCTAGCCAATTCTTTTT

TTGACGGTACCGTAAAAGTATTATAAGAAAAATCCTTAAACCGG

**>TaMKK10-5(4AL) TRIAE_CS42_4AL_TGACv1_292438_AA0998860.1**

GCCACAATTTTCAGTATAAAGCAAACCTGAATTCATCCATCCATCATTCCCCCGAAAAGG

AAACCTCTGAATTCGAAGACTCGATCCAAAATAATCAATCAGCTTGCAAGTTCGATCTCA

AGAGTCCAGACGCACAAGGCGATCAATTTAATGGCGGCCGCCAGGGAAAGACGGCTGCCG

CAGCTGCACCTCACGCTCGACGCCCCCACGTGGGCCTTCCGGTGCCCGGCCCCGGCGCCG

GTCACCGCGGCTACGCCGTCCACGTCGGCGGCTCGGCCGGACGGCGAGTTCCGCCAGAGC

GACTTTGACATGCTCAAAGTCCTTGGGCGCGGGAACGGCGGGACCGTCCACAAGGTCTCG

CACCGCCGCACGTCGGCGCTGTACGCGCTCAAGGTCATTCACCGCGGCCACCCCGGCGCC

GGCGATGAGGTGGATGTTGTGCGGCGCGTCGACTCCCCGCACATCGTTCGGTGTCACTCG

GTGCTCCCGACGGCGTCCGGCGACTCTGCTTTGCTCCTCGAGCTGATGGACGGCGGTTCG

CTCGACTCGCTCGTCCGCGGCGGCCAGGGAGGCTTCCCGGAGGCGGCTCTGGCGGAGGTG

GCGGCGCAGGCGCTGTCCGGCCTGGCGTACCTCCGCGCGCGCCGCGTGGTCCACCGGGAC

ATGAAACCGGCGAACCTCCTGGTCAACAGGGCCGGGCAGGTCAAAATCGGCGACTTCGGC

ATCGCCGAGGTCGTCTCCCGCGCCGGCAAGTACCGCGCTGCCTACGAGGGCACCGCGGCA

TATATGAGCCCCGAGAGATTCGACACGGAGCGGTTGCAGGACGGCGAGGGGGGCCGCGTC

GACCCGTACGCCGCCGACGTGTGGGGGCTGGGCGTGACCATCCTAGAGCTCCTCATGGGG

CGCTACCCGCTGCTCCCCGCCGGGCAGGAGCTGACCTGGGCGGCGCTCATGTGCGCCATC

TGCTTCGGTGAGCTGCCAGCACTTCCCGACGGCGCGGCGTCGCCGGAGCTCCGGAGTTTC

GTGTCCGCCTGCCTGCAGAAGGACCACCGGAAGCGGGCATCAGTGGCGGAGCTCCTCGCG

CACCCGTTCGTCGCCGGAAGGGACGTGGCATCGGCGAGACATGCGCTCCGGGAAGTGATC

GCGCAGCGCGTTTAGATATTCAACTGTCCGTTCACAAACTGTAGCTAGACATTTGCACAG

CTGCAGATACAGATAGTGGAAGAACTCTGGAATTTTAAATCGACGAGATAGTTTAATTTA

GTTTTGGTGTCTATCTGTTGCATTATTTCTGTCGGCCAGATTACAGATCATCAATTTGAC

GTCGCTAACCAGGGGACGACAGTTTTTAAAGCAATCCCAGTGGACGCTCTTAGGACCGTC

AAATCCCAATCGAACGTATGGCAATTACTCTGCGCAGCATGGGCAATTTTCTGCCATTTA

TTTTGGTATCAGATGGCAATTTTTGCTTTAGAGCAGGGCAAATCCCTTGCAGTAGCACAT

GCCAATTTTATCTGTTTTAGCATGGTAATTCTGGGCGTTCGCTGGAAATCTTAAAAATCT

G

**>TaMKK10-5(4BS) TRIAE_CS42_4BS_TGACv1_331242_AA1109740.1**

CCTCCTCCATCATTCCCCCCTGAATTCGAAGGCTCGATCCAAAATCATCAATCAGCTTCG

AAATTCGATCTTAAGAGTTCAGACGCACAAGGCGATCAATCTAATGGCGGCCGCCCGAGA

AAGACGTCTGCCGCAGCTGCACCTCACGCTCGACGCCCCAACGTGGGCCTTCCGGTGCCC

GGCCCCGGCGCCGGTCACCGCGGCGACGCCGTCCACCTCGGCGGCTCGGCCGGACGGCGA

GTTCCGCCTGAGCGACTTTGACATGCTCAAGGTCCTTGGACGCGGGAACGGCGGGACCGT

CCATAAGGTCTCGCACCGCCGCACGTCGGCGCTGTACGCGCTCAAGATCATTCACCGCGG

CCATCCCGGCGCCGGCGAGGAGGTGGACGTCGTCCGCCGCGTCGACTCGCCTCACATCGT

CCGGTGTCACTCGGTACTCCCGACGGCGTCCGGCGACTCTGCTTTGCTCCTCGAGCTGAT

GGACGGCGGTTCGCTCGATTCGCTCGTCCGCGGCGGCCAGGGAGGCTTCCCGGAGGCGGC

GCTGCCGGAGGTGGCGGCGCAGGCGCTGTCCGGCCTGGCGTACCTCCGCTCACGCCGCGT

CGTCCACCGGGACATGAAGCCGGCGAACCTCCTGGTCAACAGGGCCGGGCAGGTCAAAAT

CGGCGACTTCGGCATCGCCGAGGTCGTCTCCCGGGCCGGCAAGTACCGCGCGGCATACGA

GGGCACCGCCGCCTACATGAGCCCCGAGCGATTCGACACGGAGCGGTTGCACGACGGCGA

GGGGAACCGCGTCGACCCCTACGCCGCCGACGTGTGGGGGCTGGGCGTGACCGTCCTGGA

GCTTCTGATTGGGCGCTACCCGCTGCTCCCCGCCGGGCAGGAGCTGACCTGGGCGGCGCT

CATGTGCGCCATCTGCTTCGGTGATCTGCCGGCACTTCCCGATGGCGCGGCGTCGCCGGA

GCTCCGGAGTTTCGTGTCCGCCTGCCTGCAGAAGGACCACCGGAGGCGGCCATCTGCCGC

GGAGCTCCTCGCGCACCCGTTCGTCGCCGGGAGGGACGTGGCAGCAGCGAGACATGCGCT

CCGGGAAGTGATCGAGCAGCGCGTGTAGATATTGGACTGTCCGTTCTCAAACTGTAGCCA

AACATTTGCACAGCTGCAGATACAGATAGTGGAAGAACACTAAAATTTTAAATCGACCAG

ATAGTTTAATATGGTACAACAGTTTTGGTGTCTATCCGTTGCATTATTTCTGTCGACAAG

ATTACAGATCATCAATTTGACATCGCTAACCAGGGAACGTCATTTTTGTAAGCAATCCCA

GTGAACACTCTTACAGCAAGTCTAGTAGAGCCCTCGAACCCTCAAACCCCTAAAAATAAA

TGCCTTTTTACAGT

**>TaMKK10-5(4DS) TRIAE_CS42_4DS_TGACv1_362002_AA1175550.1.1**

CGATCCATCCATCATTCCCCCGAAAAGAAAACCTCAATTCGAAGGCTCGATCCAAAATCA

TCAATCAGCTTCCAAATTCGATCTTAAGAGCTCAGACGCACAAGGCGATCAATCTAATGG

CGGCCGCCCGAGAAAGACGGCTGCCGCAGCTGCACCTCACGCTCGACGCCCCAACGTGGG

CCTTCCGGTGCCCGGCCCCGGCGCCGGTCACCGCGGCGACGCCGTCCACCTCGGCGGCTC

GGCCGGACGGCGAGTTCCGCCTGAGCGACTTTGACATGCTCAAAGTCCTTGGGCGCGGGA

ACGGCGGGACCGTCCATAAGGCCTCGCACCGCCGCACGTCGGCGCTGTACGCGCTCAAGA

TTATTCACCGCGGCCATCCCGGCGCCGACGAGGAGGTGGATGTTGTGCGCCGCGTCGACT

CGCCGCACATCGTCCGGTGTCACTCGGTGCTCCCGACGCCGTCCGGCGACTCTGCCTTGC

TCCTCGAGCTGATGGACGGCGGTTCGCTCGACTCGCTCGTCCGCGGCGGCCAGGGAGGCT

TCCCGGAGGCGGCGCTGGCCGAGGTGGCGGCCCAGGCGCTGGCCGGCCTGGCGTACCTCC

GCGCGCGCCGCGTGGTCCACCGGGACATGAAGCCGGCGAACCTCCTGGTCAACAGGGCCG

GTCAGGTCAAAATCGGCGACTTCGGGATCGCCGAGGTCGTCTCCCGCGCCGGCAAGTACC

GCGCGGCCTACGAGGGCACCGCCGCGTACATGAGCCCCGAGAGATTCGACACGGAGCGGC

TGCACGAGGGCGAGGAGGACCGCGTCGACCCCTACGCCGCCGACGTGTGGGGGCTGGGCG

TGACCGTCCTGGAGCTCCTCATGGGGCGCTACCCGCTGCTCCCCGCCGGGCAGGAGCTGA

CCTGGGCGGCGCTCATGTGCGCCATCTGCTTCGGTGAGCTCCCAGCACTTCCCGACGGCT

CGGCGTCGCCGGAACTCCGGAGCTTCGTGTCCGCGTGCCTGCAGAAAGACCACCGGAAGC

GTGCATCAGTTGCGGAGCTCGTCGCGCACCCGTTCGTCGCCGGGAGGGACGTGGCAGCGG

CGAGACATGCGCTCCGGGAAGTGATCGCCCAGCGCGTTTAGATATTCAACTGTCCGTTCA

CAAACTGTAGCTAGACATTTGCACAGCTGCAGATACAGATAGTGGAAGAACTCAGGAATT

TTAAATCGACCAGATAACAGTTTTGGTGTCTATCCGTTGCATTATTTCTGTCGACCAGAT

TACAGATCATCAATTTGACGTCGCTAACCAGGGAACGTCAGTTTTTAAAGCAATC

**>HvMKK1-1 MLOC_5024.1**

CGGGCATATGCGTGTGCGCCTGCTACCAAAGACTAGTGGTTTTCTTTTCAGCCTTCTCAT

TGTACAGCGAGCGTGCGTAGGATAGGACGTGTGCTTTGCTCGTATGCGTTTTTTCCAAGG

GTAACGCGTAATTGGACAGCGAGGAAAGGTCAAATCTCCAGGCGACCAAAGCAAAGACCG

GTGAGCAGGACCCGTCGTCACCAGCAACCCCTCCCCCCAACCGCCGTTTTCCTCCCCCCA

CCCTTCCCTCCGCGTCTCCACCCTCCCCTCCCCTCCCCTCTCCTCCGAGGCCGTCCAGCC

CACGAGCGAGCCGATGAAGAAGCCGGGCAAGCTCGCGCTGCCCTCCCACGACTCCACCAT

CGGCAAATTCCT

gtaagcagctaacctcctcctcctcctcctgctgctgctgctgtccatctgccccctcct

tcttctgacgcgctgatttggttttggtttccgcccggggggagcag

CACGCAGAGCGGGACGTTCAAGGACGGCGACCTGCTCGTCAACAAGGACGGCCTCCGCAT

CGTCCCCCAGAGCGAGGAAGGCGAG

gtaagcccaaaccagccagccaatcaaatcccccgcctcatctcatcttcgcctagaaac

gtagggctggacccggcagatcggtacaaccacaaccaacctctactgttttttttttcg

taataatttcgttcgtgtcatcaatcaatacatttacaaagcacacataaaaaaaaaaga

gtgggacgtgtgactagcgttggcatgtgccggccaggggcttcctaccgtccagtggtg

gttacccttttcggaacgacgatttagggggaagttgtctgattgcattctgtccacaaa

ttcgattgtgcccttgtatgcgaaaacggagggagtaatacgtactagtaccatttatac

tggtactatctgtactgatttcgtcgacggcatgagcagcgaaaattggcacgcgcggtt

accaggtttcaagtggttgctcatatttttccggaaatggtgatgctcgcttgcttatgg

tggaagagctgtctatcgccattttttgttttgggaactgtctaattgcaacctgttctt

aactttttgtactcttcatttatcttgtctctgtctcaccttcttcccgttcacgtactc

gacttctctag

GCACCTCCTATCAAGCCGTTGGATAATCACCAGTTGAGCATTGATGATCTGGACTCGATC

AAAGTGATCGGGAAAGGTAGCAGCGGAACCGTGCAGCTGGTGCGCCACAAATGGACTGGC

CAGTTTTTCGCTCTCAAG

gtattgcctttgtctcaaacatacattatctctgctgcttcatgtttgtggttcctttga

ctttgtttctgtacaaaacttacggtggtcgttgtaactagcctctgtctctgtggtatt

ttgcctacatgtatctgcag

GTTATACAACTCAATATTCAGGAAAGCATACGCAAACAGATTGCCCAGGAGTTGAAAATA

AGCTTGTCAACACAGTGCCAGTATGTTGTCACATGCTATCAGTGTTTCTATGTCAATGGT

GTCATTTCTATTGCTTTGGAGTATATGGATGGCGGCTCTCTCGCTGATTTCCTCAAGGCT

GTTAGAACAGTTCCAGAGGCATACCTCGCTGCAATTTGTAAGCAG

gccagtgaaatgtcacatctccctaatatcttttggctatcagaaattcagaatgtcacc

ttaagatcaaatagaccttttcaattgacaattgcaacttcctcgtgcag

GTGTTGAAAGGGCTGATGTACTTGCATAATGAGAAGCACGTTTTACACCGAGATCTGAAA

CCATCGAATATATTGATAAATCACAGGGGTGAAGTTAAGATATCGGATTTTGGTGTTAGT

GCCATCATTGCTAGTTCTTCCGCACAGCGAGATACTTTTACTGGCACGTTTAACTACATG

GCG

gtgggtgaaagttttctcaagtttatgtaaacatgtcttccagtgtggcatgaatatatg

cccaccatttttttttgtttcagctaaccaatgtagaatgtatactctcgacag

CCTGAAAGAATCAGTGGGCAGAAACATGGTTATATGAGTGATATCTGGAGCTTGGGTCTA

GTTATGTTGGAATGTGCCACTGGCAACTTCCCATATCCTCCTCGTGATAGCTTTTATGAA

CTTCTTGAAGCTGTTGTCGACCAACCATCACCTTCTGCACCACCAGACCAGTTCTCAGCA

GAGTTCTGTTCATTCGTTTCCGCATG

gtatttgtgcttctccattgaccccccccccttcttaagcactgttcatttgaaaattgt

gtagatttaaatgaggagcttagttacaaatattatcatttttttttaccttttgcttgt

gaagtagtgattctcaatccaaaattcttgctttggaatatccatcatattatctgaact

tccaaatatgtttttgttattctctcatctcatctgtagacagattaaatggtttctgta

ttttacttacggagcattagtgtctgaaagaaggctaatatatctgttctcttaatttct

ttctggattttttcag

TATCCAAAAAAATGCTACAGATAGGTCATCTGCCCAAACCCTATCG

gtaaggaaatttctgtccgcttcacaattttgtatgttcattcgaaagcatcattagcta

gcttattactgatcttgatccatgtcttctatccgttgcctccgacgcccag

GCTCATCCGTTCCTGAGCATGTACGACGACCTGAACATCGATCTCTCTGACTACTTCACG

ACCGCGGGATCGCCACTTGCCACCTTCAA

gtaatggatcctataaaccgctgaagtgcacatacatggttactgcaattcctttacaat

tccagtgatggcctatcttcgttttcacag

GCAAATCGCATTGTGAGGCGACAAATGCTCGACCACGTGCCGTGAGCTGGAGGACAATCT

GTAGAGCGACGCTAACTAAAGCAAGTGATTGGAGTTTTTCTTTACCTTCCTTTTGTTCAG

AACTTGGGGTATGATGATATGTTTGAACTGAGTAAGACATTATTGGCTGCACTGCATAAT

CGTACAAAAAATACAGCATTCTAGCGTGAGTGAGGACCCAAGTTTTGCCTGGCTGCACGC

CGGAACTCAACTGCCCGTTATGTATCGGCGTCCCGGTTTTGAATTTTACTGTAAACGAAA

CCATGTTTACTGGATCGCCTTTTACAATCCGATTTGGAGCAGCATTTG

**>HvMKK1-2(N-term) AC264970.1 Hordeum vulgare subsp. vulgare cultivar Morex clone BAC 0404L15, *** SEQUENCING IN PROGRESS ***, 48 unordered pieces (reverse comp; 5’ end not shown here is 100% match for ASM32608v1:6:24835876:24838005:-1)**

…ATGGGAAAATTCCTGTAACCTCA

AACCATCATCCCTCTTCTTTCGAATTTTTGTTTTCCTTGGACGTATTGCTCTTGCAGTCGTGCTGATTGA

TTTAATTTCACGGCGGTTGTGGGGACAGGACGCAGATCGGGGCGTTCAAGGACGGCGACGACCACCTCCT

CGTCAACAAGGACGGCCTCCGCATCATCCCGCAGATCGAGGAAGGCGAGGTAAGCACTAAAGCACGACTG

GGCACCCAAAATAAAAAATTCCTCCACGCGTCTGGTCTTAGCCGTTCCTGGGAAAAGAATGTAGGGCTGC

GCTATGCATATTGGTAGTTGTATTGGTTTTCGTAATTTCATTGATGATATGAACAGATTTATATTGGGAT

GCGTGGTTGGATTAGCAGGTACGGAGCAGGGGTTTCGTATTTTTTTCTTCAGAACAACAATTTAGGACAG

ATGATATTATGTATCTCCTTCGTCCGGGATTAGTTTCACTCAAACGGACCTATCTAGACGTATTTTAATG

CTCAATACATCTGTTTGAGCGTCAACTAATTCCGCGTGGAGGTAATACATAGATTATTTTCATGCGGGAT

ATCCGCAAATGCAGTTGTTACAGAAATAATTTATAATGTATCCAGTGGTTAAATTTATAGAGAGAATTCC

TTATTTAAACATTATGCTTAAATTTTATGCCCTATTTGACATCGAAAAATTATTTGTTCCCTGTATAACA

ACAAGTCTAATTTTTATGCCTTTTATAACATTTTTGTCCACTTTAAGTCTAAATGACATCTGAAAAGACC

CTTTTGTTCCTCGTGTGCTATGCTTGTGTACGTGCTGCTGCGTGTGTGCGCGCGCATACAGATATGCTAT

TGATGTTGCGTGTGTGCTGTTGCATGTTTGTGTGTGCCGTTGCATGCGCTGCTATGTGTGTGTGTGCACG

CGCGTGCTGCATGTGCTGCTACTTGTGTGCATGCATATGTGCGTGTGCTGCTGCTGTTGTGTATGTGGTG

TTGCGTGTGTTTGCGCGTGCTGCTGCTATGTGTGTGTGCGCCACGTGTGTGTTGTGTGCGTGTGTGTGCG

GTTACGTGTGTGTGTGTTCTTACATGTGTGTTGTGTGCGGTTACGTGTGTGTTGTGTGCGTGCGTGTGTG

TGCTATTACGTGTGTGTTGTGTGCGTGCATGTGTGCTATTACGTGTGTGTTGTGTGTGTGTGCGTGTGTG

TGTGTGTTGTGTGTGTTGTTGCATGTGTATTTATTATTGCGTGTGTGTGCTACTGTTGATGTGTATGCAC

GCTACTGCCCCGCATTATTCTGTGTGCTACTATCCCGCACATACATACCACATGAGGGGCAAAATAGTCT

TTTTAGATGTCATTTAGGCTTAAAATGAACAAAAGTGTCATAAAGGGCATAAAATTTAGACTTGTTATTA

TATAGTAAAGAAAAAGTTGTTCAATGTTAAATAAGGAAACGAAATTCAAAATAATGTTAAATAAGAAATT

GTTTCAAATTTATACTGAATGTTGATGCTCACTTGCTTGTAGTGTCTAACACCATTTCTTGTGGGAATTG

TCTAATTACATTGTTATTCTTGTTACAGTATATGTGGATTGCGTTAGCCATTTCCATCAGTTCGGACTTT

TGGTTGCGTTGGCTAGT

…

**>HvMKK1-2(C-term) ASM32608v1:6: 24835876: 24837993:-1**

NNNNNNNNNNNNNNNNNNNNNNNNNNNN

NNNNNNNNNNNNNNNNNNNNNNNNNNNNNNNNNNNNNNNNNNNNNNNNNNNNNNNNNNNN

GCATGAAGCTTAACATGGTATCAGAGCTAAGGTCTTGAGTTCAAGTCCTGACTTCCGCAA

TTTATTAAAAATTGTTGGTAGCCCCCTTTGTGTCCACGTAGAGGCCTCTTGAGTCATATG

TGAGTTTCACGTGCCGTCGCTCTCTTCCGATTGCACGTGTTGACTTGTCTTCCCCATCAC

ACGTGAGAGGGGGTGTTACAGTATATGTGGATTGCGTTAGCCCTTTTCATCAGTTCGGAC

TTTTGGTTGCGTTGGCTAGTGCATGAAGGTTAACAGCTCTTAACTTGTTATGCTCTTCAT

TCATCTAACTTGTGTCTTGTCTTCTTTCCGTTGGCACACTCGACTTCTCTAGGCTCCTCC

TATAAAGTCGTTAGATAACCATCAGTTGAGCATAAATGATTTGGAAGCAATCAAGGTGAT

CGGGAAAGGTAGTAGTGGAACCGTGCAATTGGTGCGCCAGAAATGGACTGGCCAGTTTTT

TGCTCTCAAGGTATTGCCCGAAACATAAATTATTTGATGATTTGTAATTTCGGTTCCTTC

CATTTCTTTCTGTAGAAACTATAGTGGCAGTTATAATTGTCTTTACCCCCATTCATGTAC

TGTCATGTTTGCAGGTTATACAACTCAATATTCAGGAAAGCATACGCAAGCAGATTGCCC

GGGAGTTGAAAATAAGTCTGTCAACACAGTGCCAATATGTTGTCACATGCTACCAGTGTT

TCTATGTCAATGGTGTTATTTGTATTGCTTTGGAGTATATGGATGGAGGCTCTCTGGCTG

ATTTCCTGATGACTGTTAGAACTGTTCCAGAGGCCTACCTTGCTGTGATTTGTAAGCAGG

CCAGTAAAATGATGCATAAGTAACCACATAATTATTTGTTGTTATCAGAATGTTACCTTA

AGATCAAATAGACCTTTTCAATCGATAATTGCAACTTCCTTATGCAGGTGTTGAAAGGAC

TGATGTACTTGCATCATGAGAAGCAAATTATACACCGAGATCTGAAACCATCGAATATAT

TAATAAATCATAGGGGTGAAGTAAAGATATCAGATTTCGGTGTTAGTGCCATCATTTCTA

GTTCTTCTGCACAGCGAGATACATTTACCGACACATTTAACTACATGGCGGTGGGTGAAA

GTTTGTTTGAATTATGTAAGTATGTCTTCAGTGTGGAATGAAAAGACGTCCACCATTTTT

TGTTTCAGCTAACGAATCTAAAATGTATACTCTTGACAGCCAGAAAGAATCAATGGGCAG

AAACATGGTTATAGGAGTGATATATGGAGCTTGGGCCTAGTTATGCTGGAATGTGCCACT

GGCAATTTCCCATATCCTCCTTGTGATAGCTTTTATGAACTTTTTGAAACTGTTGTTGAC

CAACCATCACCTTCTGCACCAACAGACCAGTTTTCAGCAGAGTTCTGTTCATTCATTTCT

GCATGGTATTTGTGCTTCTCCATTGCCCCCCTCCCCCCCTGTTAATCAATAATACTTGAG

CAATATTTGCAATTTGTTGAGATTTAAACTGTGAGCTTAGTTATCATAGTGTTTTTTTTA

CCTTTTGCCTGTCAAGTAGGAGTAGTAGTGATTCTCACGCCAAAATTTCTGCTCTAGAAT

ATGCATGCCAACATCATCATATGTTTCTTTTTGTTACACTCACCTAGTCACCTTTAACTG

TGGACAGATCAACTAATTTCCTTATTTTATTTACTGGAAATCAGTGTCCAAAATAAGGCC

AATATCTATTTTCTTAATTTCTTTCTGAATTTGTCAGTATGCACAAAGAGGCTACAAATA

GGTCATCTGCTCAAACCCTATCAGTAAGGAATTTTCTGTCTGCTTCACAATCTGTATGTT

CATCCGAAAGTGTCATTTGACAGAGCAAGATCACTGATCTTTACCCATTTTTTCTATCTG

TTGCCTCTGATACCCAGGCTCACCCATTCCTGAGCATGTACGATGACCTGAATATTGATC

TTGCTGTGTACTTCAGGACCGCGGGATCTCCACTTGTCACCTTCAAGTAA

**>HvMKK3-2(N-term) MLOC_51567.4.X (based on MLOC_51567.4)**

CGGCATATAGACAAGAGACAGAGGACGGAGCAGAGGGGCATCCTCCTTCCCTCCTTCCTC

CCCCGTCGCCGGGCTCGCCGCCGCCGCCCGCCGCCCGCCGCCTCCTCCTAACCGTCGCGC

CGCCCCTTCGCGTCCTCCGCTCTCCGCCCGTACAGATCCGTCCGGCGAGCATCCCCGGCC

GCTCCTCCCGACGGGCTCGCCGCCACCTACAGGACCTCACCGCCCGCCCTCCTCACCGCG

AGGCGAACCGAG

gtgaccccgctcccccaaatcccgattcccatttcgtcagttgccaagttcgattcggaa

tcggcgccggttagagaattccagtgtggtatgttcactattgtttgatctgttcgcgat

gtaaccag

GTCCGCCGCCCAAGCTCCAATTTTTCGCCTGGGATTCTCTACCCAACCGAAGAGACCGTG

ATTCCTCGCTGCTTG

gtaatcccaattttgtttatttttttggctgggctcattattactcactggcttgtgcta

taggcagccagattttgtgtgctgttgttgatgcaaactgctacccatccatccatccat

ccaccattag

GGTTATTTTGATTGACAGTCCCCATGGCGGGGCTAGAGGAGTTGAAGAAGAAGCTGCAGC

CGTTGCTGTTCGACGACTCGGACAAGGGCGGCGTCAGCACCAGGGTTCCCTTCCCCGAGG

ACACATGCGATTCCTATGTG

gtaaactcacagtgtcagtcttttcttctctgcatgcaaaccaaattaggttgtctctgt

ctgtgtctgtgtcaaacaccggcagccagcattaggcgtggttgttgtctgttaagggaa

ttggtacaagttgtttcagtccagttttccagcagctgaatatacatgctccaacttagt

catctgcacagcctgaccaatcgctcctcacacagacagctttcttttggaattcctcca

acttagtcaatggtgtatctgtgaattcaaacatgtttacatgtgcaaatgttttttttt

tatgttctttctttcatgaag

GTGTCTGATGGTGGAACGATAAATTTACTGAGTAGATCGTTTGGTGAGTATAACATCAAT

GAGCATGGCTTTCATAAGCGAAGTACTGGCCCAGAAGAGCCAGATACTGGTGAGAAGGCA

TACCGATGTGCATCTGAAGACATGCATATATTTGGTCCCATTGGAAATGGAGCAAGCAGT

GTCGTGCAGAGAGCTATTTTCATACCAGTTCATCGAATTCTGGCCTTGAAGAAGATAAAC

ATATTTGAGAAG

gtacattctttatggcccgaaatgcactttactaccacattgttgtacacttctgctttt

tgtttatatatgggtgttttgttttttgttgattactagtctaaacctcatttgtgtttt

caaacaaaataatattaaagggagcaaaacaaacctttattcatctatacgtggtttact

tgcatgctggagcatccttccctttcttttggagtcatgtgcatcacaatgggcccatga

agtgcagcacatgaatgggtagcatcaatcacattcccctgaagaattttggtcactagt

gataagctgtcaatttctctattgcctgttcacagtgtctcctatttactccctccgttc

caaaatataagtctttttagagatttcactagaggatcacatacggaacaaaatgagtga

atnnnnnnnnnnnnnnnnnnntatatacatccgtatgtagccccctagtggagcctctta

aaagacttatatttaggaacggagggagtagaagggaagaacatctcaattacagggtgt

tggatatttagaagcgaagcaatgttagactctcactatgtacattgtttttttggaatt

acaaatagtatccggacactaacttcttatcagactcagactggcacttaacttgcagac

acgtactcttactcatgtaaactcgcctagccagttaggacttgccgactagtggaaaga

tgacactaaatcctcatccttagctacctgcattatttcctggctcatctgcaatagtga

tttcagaatgggcccatgacctgcacaaggaagggtgccaaaaatacccacgatagagga

tttcagttactagggacccctcccacacatctttagaatttcaagctgcctatttaccaa

tttctcattcacagtgctctctagttttatatttcgagatgacatggttcatatcatgat

atttaattaagcaaagaaagagaacaagttcatctcatctctagtctcctctgaccccaa

agcaaactctcgccaccatgccatgctctcgacacatttgccctataatttagtagcatg

aatgacaatgtgacgtgttcattttccttttaatcactcccaaggcaaccaaatatccca

ctacacaattttcttgatgtaagcctgagtgaaacaagttggagttgagtatattaaacc

aaaacatgtatttccattgcattgaaggcagcacaaagggatactggtttatttgcagtg

gtagtcaagtaccagagtcagttctgacctgcagcatggaagcagatacaccaaagaata

gaaatgctctctcttctatgcttcaattcatataccattgcagcgcaccgtgtttggtat

tgcacggagtaattttggtactttgtgtttgtccatatattttgctataattaatccttt

ctgtttcagttgataaactgatttacaaaattttcttttgatattttatacgtgtcttca

tacctttacag

GAGAAAAGGCAACAAATTCTTAATGAGATGAGAACATTATGTGAAGCAAGTTGCTATCCT

GGTTTAGTTGAATTCCAGGGTGCATTTTACATGCCTGATTCTGGACAAATAAGCATTGCC

CTTGAATACATGGATGGTGGCTCCTTAGCAGATGTTATAAAAGTCAAGAAATCAATACCA

GAGCCGGTTCTTGCACATATGCTACTGAAAGTATTACTT

gtatggatcttccacacatactattccttccatgtccgtgttaatcaacattaagctgtg

acctgatgtatttttgaagttataatttgttctttgtacaattctgaag

GGCCTGAAGTACTTGCATGAGGCAAGACACCTAGTGCATAGAGATCTAAAGCCAGCGAAT

ATATTGGTAAACCTCAAGGGAGAGGCAAAAATTACAGACTTTGGCGTAAGTGCTGGTTTG

GACAATACAATGGCTATG

gtatgctacttcttggtgactttttatattgcttaatctgagctaattattttataatag

tgtagcaatgccatcaaatcattttgcgtgaatgcgcaacatattttctgctaaataaat

atgtttcag

TGTGCTACCTTTGTAGGCACCGTGACATATATGTCACCTGAGAGAATTCGTAATGAGAAC

TACTCTTATGCTGCTGATATTTGGAGTCTTGGACTAACGATATTGGAGTGTGCTACTGGT

AAATTTCCATATAATGTCAATGAAGGCCCAGCCAATCTCATGCTGCAG

gtgattgcctactcagattttccggttcttgtctcccttttagtctgatgtaaaatgcgt

ggactatttgagctgttcacatagtaggttgtatttggattcgttgagatggcaagaaat

cttacatttctgcaagtctgttcgttggttatgttagtttccacaaacttctttttcctt

gctggtttaacttttgtaagttctatttttatttgaatatataaatcatctgacaatgta

ttccctctgtttagatcactaaagtagtgatcnnnnnnnncttatattagtttacagagg

gagttttaaatatgtactgttacatatatattggtcttatgaatgttttcaccatcattt

gtctgtgggaatgttgtttttagtcacttcatttgttgccctatctttatcttaaaatct

catacttcagatactgttttctttcactcagttgctatcctttcttaatccgacaaaatt

tcttatttcag

ATACTCGATGATCCATCGCCAGCACCACCAGAGGACGCCTATACACCAGAGTTTTGTTCC

TTCATAAATGATTGCTTGCGGAAAGATGCTGATGCAAGGCCTACATGCGAGCAG

gtaaagaaaactttcatgtaatctgcgtccagtaggttagtaatttgcgttaatgttttg

cggtttatttgaatggtactacatattactggtaatattttttcttgataaagcattgct

ggcagtaactgtctgagtgtgtagttgtgtggttaagtatagagttttattaactgattt

gccttctcttgtaactatgtcacactggcctttggttgatgaggacattgctgcccctat

cattggtccattggaatgaacgtaggaggcccattccgcacagagaccttggtttctctt

ctttctttcttgcaccatcatgtccatacttccataccctagcccggattagctgtcacc

gccacgttcaggcgaccaccgccaccaatgttggctgtccgccataggcctcttgtactc

ggcaacgtgcccagaatgacgctgcgccctcactctgctccagatgtgcctctgctgtag

agcctgccgtgacctgctcttgcctgtcgtgctactgtccttgctgctgctgcttctggt

ggccacccatgatctctagctatctggtcgcttttgtgttagggcctggatctgggtgtg

ccaccaatcgtccatgctctgcgttagcacttgaacttttggagggtcaggaacttggtg

cttgggcaccgtggaggtatgtgcagctaggatggaaaccttaggcgcagggagaatgaa

atgaggtgatggagcaagtagaagatgggtaactccctgattaattgatttaacatgtag

ctatctcgttataatgagggcctgccttacatgtgctggactctggtcctaaatataata

tgagctggacatggcaacatgtccgttataataggaacaccacttgaattctgatcacca

gtctctctgtgcctactgtggtttccttccctcagtgattccctggaagatcaagcagat

tgcagtttctcgttcgaatgcttaggctgagttgcaagtcatgtcatccttgatccagtg

aagcaccttaagcaatgcatcggctagtctatatcactgatgcattttctctccaagcac

attggagttgatgcttcctgtttggaattgcagatgcaagagcaggttgttgctctccag

tatgttccatcagaactctagtcagctgatttcttcaccaaggcgcattctagagctttg

ttgttgatctgcctttaattttgtatagggtgttattggaggtccatttcacacacttat

aggcaaaatgtacgtaatgattagttttttcttttgaaaaggaggatgacccccggcctc

tgcatctgggagatgcatgcagctactttattaattatggcttggtctaataacagaatg

ttaattatcttgacgatgcattttaaatctttggtccctttccattttttggttttctta

taattgattaacatgcttaacattccacag

CTTTTGTCGCACCCATTCATCAAGAGGTATGAGGAAACTGGTGTGGACTTGGCAGCATAT

GTCAGGGGTGTTGTTAATCCAACAGAGAGATTAAAGCAAATAGCAGAG

gtgagttctttagagcatgtttcagcttattgttttttctacttcagttcaatatcagga

cacatacctgatacatgaaacatcccccctatgtcaag

AACTGGTGCTAGAACTTTGAAGGGGGCAGATGCTAGATGGAGGAGATATTGACAAACACT

GTTGCTTGATGATGTTACCGCGGCTCACCTTCTTTTGCCTCTGCCAACTCACCGCTGGTG

GTCACCATTCCTATTTGTTCCACTTCCATCTACCTCTGTTTTGGCATGAGATGGAGAAGG

GGAGGGGGAGAGGGAGAAGGAGAGCATTCACCGAAGAGCACTAACCCCACCCCCGTCAAT

CCTACTTGTTGAGCCACCTTAACCATTTCCCATGGCACTGTTCC

**>MKK3-2(C-term) : ASM32608v1:5:556255205:556260000:-1**

ATGCTTGCTGTTCATTATT

ACCTCCTGTTTGATGGCTCTGAAGGACCCTGGAACCATATGAAGACATTCTACAGGGAAG

AATCATCTTTCAGGTACCTGAGGCGATGTTATATTTTGTAACATGATCATAAAACTTGTC

TGTACCATACCACATAGCATCTTATCGCTGTTTAGGCATTTAACCTGTATGTATTTTGTG

GCTCCAATAACTGGTTACATTATTCTTAAAATCAGCTTGGTGATAGTAATATCAAGAATA

TGTTCTGTGAAGGATAATTTGAAAACTAGCTGAAACGATAGCATTGAGTTGCCAACAAAG

TTGATGGAGCTAGCTGAATTGACCGTTGCACTTTACATAATTGACCCTTCCTTGTTAATC

TGGGCAAAGCATGAGCCGCTTACTTCCATTTGTTCCCTACTTTATCCTGTAGGCAGTGTT

GTTTCTCTTGTTTTACCAGGTAGTAACTGGCTGGTTACTGCCAATTTTCCAAATTTATCC

TGTTGCTTCTTTTGTGTCACCGTTATAGGTTAGATGATACTGTGTGTCGAAGAATCCAAG

CTGTTTCTGAGCATACACATGATATGTTGTGTTGCCTATCCTGTTTTTGCAGTTTTTCAG

GGAATGTGTATGTGGGACAAAGTGCCATATTTGATACTTTATCAAATATAAGAAAGAAGT

TAAAAGGTGATCGGCCTAGAGAAAAAATTGTTCATGTTGTTGAGAAGCTACATTGTCGTG

CGAATGGGGAAACAGAAATCGCTATTCGTGTGTCTGGATCATTTATCACGGGCAACCAAT

TCCTAATATTTGGTGAAGGGTTGCAAGCTGAAGGGATGCCCAGTCTGGACGAAATTGATA

TCGACATTCCAAGCAAGCGGGTAGGCCAGTTCCGGGAGCAGTTCACCGTGCATCCAGGGA

CTTCCATGGGGTGCTACTACATAGCAAAGCAAGACCTCTACATCATCCAGTCATGA

**>HvMKK3-3(N-term): ASM32608v1:5:282831404:282831930:-1**

ATGGCTGGACTGGAGGAGCTGAAGCGGAGGCTGCAGCCCATCTTCTTCGAC

GCCGATGGCAATGTTGTGCCGCCCCCCGCCGCCGACGGAACGCACGACGACTCCAGCTCT

GACGACTGCGAGGTAACCACTCTCTTCGTCTCAGGCGTTTGCTCAGTTCGAGATCTATGC

TTATCTGCATTCTCTTCAACCAACAAAATTCCTTGCTTATGTCGCTGTAAAAATATATGA

TGCTCATATTTTAATATATTAAATCTTACTGTACTTTTGTAAAGGTTTTGGATGGTGGAA

CTGTCAATTTATTGAGTAGGTCTTCTGATGAATATAACATCAGTAAGCTTGGCTTCCATA

AACGAACAACTAGACCAGACGGTGACTATGCTACAGATAAGGCATATCGATGTTCTTGTC

ATGATATGCATATTTTTGATTCTGTTGGTAATGGTGCAAGCAGCGTTGTCCACAGAGCTA

TTTATGTGCCAGTTCATCGAGTTTTGGCACTCAAGAAAATCAACATTTTTGAGAAG

**>HvMKK3-3(internal) : ASM32608v1:5:85174242:85175936:1**

GAAAAGAGACAACAGATTCTTAACGAGATTAGAACGTTATCAGCAGCATCTTGTTATC

CAGGTTTAGTTGAATTCCAAGGAGTTTTTTACACTCCGGACTCTGGAGAAATATACTTTG

CACTTGAGTATATGGATGGTGGTTCATTAGCAGATATTATCAGGGTCAAGAAATTCATAA

CAGAACCAGTTCTTTCACATATGCTACAGAAAGTGTTGCTAGTACGTGTCTCTTGCCTCC

ATACCTTCTGATGTTAGCTCTATTCAAATCAGGCCTGTGAAATTTAAGTGACTAGTTGGT

CTGTGTTACTTTATTAGGCTCTGCGCTACTTGCATGAAGTGAGGCGTTTAGTTCACAGAG

ATATAAAGCCAGCAAATTTGCTGCTAAATCTAAAGGGTGATACAAAAATTACAGACTTTG

GTGTAACTTCTGGACTTCATGATTCAATTACCATGGTATGCTACTTCTTGGTGACCGAAA

CTTCTCGTAGCAACAGCAAGCTGTTCTAAAATAGTATTAAGTGGACATCTGAGTATCTAG

ATTGAATTAGCAGTATATATATGTAGTAATTGTCTTCCAGTGTGCTACCTTCCTGGGCAG

TGTAACATATATGTCTCCCGAGAGAATTCGGAACGAAAATTACTCATATGCGGCTGATAT

CTGGAGCCTTGGACTAACAGCGTTGGAGTGTGCAACTGGAAGATACCCATATGATGTAAG

CGGGGGCGAGGCCGACCTCATGCTGCAGGTGAGGTGGTGGTCAAGCAGTGTTCATTTGTG

TAATTTTCTACTACCTTTCGTGAGCATCTTATTTACGGAACAAATCATGATCTCATTCAT

GTGTGTCTGTTGGTATATTGTTGATTAAAACTGTTTTCGGAATTTGGCTTGTTTTCCGTA

GGTGTTCTACATCTGTTTCCGAGTTTCTACTAGGTTTGGAACTTCTATTTCATTTCTGCT

TTCCAACATGAATGGTCTGACAATAATACAATACTAGTTCCTCTTATGAGTATTATATTA

TTGATGTATAATGCAATCCTCCTTTCCCACCTTACTGTGTTGCAGTCTTGCAGATACTAG

AAGATCCATCGCCAACACCACCGCAACATATGCATTCAGAGGAGTTTTGCTCGTTCATCG

ATGCTTGCTTGCAGAAAGATGCTGATGCAAGACCAACATGTGATCAGGTAAACTGACATC

TAGTACAATTAACGTTTTTCAAAAGAAACAGTAGGAGGGTTTCCAACTGCCTAAATCCTG

TGAAAAACTTTATTTATTGCGGGGAGTGATAAAATTTATTTATACAGTATAGGATTTTGC

GACACTATCTGAGAGTAATGTCTGATGGATAATTTAACTCTAGTCAACAGATAAGCAGTC

TATAAGGTGTGTTAATCACACACACACACACACACACATCATTCTGAGATTATAATCGCA

AAAAGCGGTCTAATATGTTGAATTGTTTTACTAATTATTCAACTAACTACATAAATAGGT

TATTTGCAACAAAGACGAAATGGAGTTTATTTTGCTCTCTGAAATAAATAAATAAATCTT

ATTTGAATCACATACTGAACATCTTGTAGCTTTTGTCTCATGCCTTCATCAAGAAATACG

AGGGACCTGGTGTGGACTTGGCAGAGTACAATAAAAGTGTTCATGATCCATCGGAAAGAT

TATCACAGATAGCACAT

**>HvMKK3-3(C-term) : ASM32608v1:5:111354822:111355452:-1**

ATGCTTGCCGTA

CATTACTACCTGATCTTTGACGGTGGTGATGACCAATGGTGTCACATGAAGTCATTCTAT

GAACAAGATTCTACTTTCAGGTAAGCGGAATCTTGGGCTACGTTTCTCTTGTAGTAAGTA

GTAACCAACCCCTTACTGCATTTTCCGGTTGTGCAAAGTAGTACTCCCTTCGATCCATAT

TAATTGTCTTGAGAGCAAAATCCAGTTGTTTTACATGAACCGTGGAGACTTATTGACATG

ATATTTGTATTCTAGTTTCTCAGAGGAAACACATTTTGGTAAGAGCGACATATTCGATAC

TTTGTCCAGAATAAGGGAAATGCTAAAAGATAACAGCCGTTGCAAGAAGATTGGCCGTGT

GATGGAGAAGGTTTACTGTCGCGCACATGGGGAGGAAGGGATGAGTGTTCGAGTTTCGGG

ATCATTCATTATGGGGAACGAGTTCCTCGTGTGCGCAGATGGGTTTTGCGTTGAAGGGAT

GCTGAGCATTGTCGAAATCTCTCCCGACATTCTCAGTAAGCAGGCATGCCATTTCCAGGA

AGATTTTTTCATGGAGCCAGGGACTGCCATGGGATGCTATGTGATATCAAGGCAAGAATT

GCACATCCCCGTATCATGA

**>HvMKK4(N-term) : AK374401(n-term CDS sequence)**

ATGCGTCCGGGC

GGGCCGCCGAACGCGCGGCCGGGGCTGCAGCCGCAGCAGCCGGGCACGCCGGGGCGTGCGCGGCGCCGGC

CGGATCTCACGCTGCCTCTGCCGCAGCGCGACCTGACGTCGCTCGCCGTGCCGCTGCCGCTTCCCCCGCC

CCCGTCCTCCGCGCCGTCGTCCTCGGGCTCGGGGTCGGGGTCCGGCGGGGCGTCGTCCATGCCCATGCCC

ATGTCCATGACCCCGCCCAACTCGGCCGGCTCCGCGCCGCCCGCGCCCCCGCCGCTGGACGAGCTGGAGC

GCGTGCGCCGCGTCGGGAG

**>HvMKK4 : ASM32608v1:6:510407211:510408187:1**

…NNNNNNN

CGGCGCCGGCGGGACGGTGTGGCTGGTTCGGCACGCGCCCACGGGCCGCGCCTACGCGCT

CAAGGTGCTCTACGGGCACCACGACGAGGCCGTCCGGCGGCAGATCACGCGGGAGATCGC

CATCCTGCGCACCGCGGAGCACCCGTCCATCGTGCGGTGCCACGGCATGTACGAGCAGGC

CGGCGAGCTGCAGATCCTGCTCGAGTACATGGACGGCGGGTCCCTGGACGGGCGGCGCAT

CGCGTCCGAGGTGTTCCTGGCGGACGTGGCGCGGCAGGTGCTGTCCGGCATCGCCTACCT

CCACCGGCGCCACATCGTGCACCGCGACATCAAGCCCTCCAACCTCCTCATCGACTCGGG

GCGGCGCGTGAAGATCGCCGACTTCGGGGTGGGGCGCATCCTGAACCAGACCATGGACCC

CTGCAACTCCTCCGTGGGCACCATCGCCTACATGAGCCCCGAGCGCATCAACACCGACCT

CAACGACGGC

AACTACAACGGCTACGCCGGCGACATCTGGAGCTTCGGCCTCAGCATTCT

CGAGTTCTACCTGGGCCGCTTCCCCCTCGGGGAGAACCTGGGGAAGCAGGGCGACTGGGC

GGCCCTCATGTGCGCCATCTGCTACTCCGAGTCGCCGGC

GGCCCCGCCCACCGCGTCCCC

GGAGCTGAGGAGCTTCATCAGCTGCTGCCTCCAGAAGAACCCGGCGAAGCGGCCGTCGGC

GGCGCAGCTGCTGCAGCACCGGTTCATCGCCTCGCCGCCGCAGCAGCAGCCGCAGGTCCT

CGCCGCCCCGCCGTGCCAACCGCCCGCCACAATAAATCAAGTTGGGATGGA

GCGT

NN

**>HvMKK5(N-term) : ASM32608v1:7:71862432:71862578:1 (homologies with AK370268)**

ATGCGTCCGGCCGGCACCAGCAGCAGCCTCCCGGCCCAGCCGGGCACGCCGGGCCGCCCG

CGCCGCCGCCCGGATCTCACTCTCCCGATGCCGCAGCGCCCGGACGTCTCCTCCTCCCTC

GCCGTCCCGCTCCCCCTCCCTCCCCCT

**>AK370268(internal) (base pair position: 255-359)**

accaccgctccccccgccggagccaccggccccgcccagccgctggcg

gccccgcccccgcccccgctgcacgagctcgagcgcgtgcgccgcgtgggcagcggc

**>HvMKK5(C-term) MLOC_21954.1.X (based on MLOC MLOC_21954.1)**

....................ctaccgtctgctcacaacacacagtttgttagagtgactc

gtttgtgttattatctttcttcccgcacgactctgattgttgacctatttgccgcgtatc

acacacactcctagttaagctaaatggtgtgtgctctgctgtatcatcacacacattttc

tcatagcgaactgtgtgctctctatcgcacacatctggatctttataaccatttgtgtta

ccttggctaatcgcaaacaattcataagaacaaattgtgtgtcgtagaccacacatgcaa

tgtttttttaaattgtatttgatggatccgtcatcgcacactgtttttgttattggcaag

ggatcatgaactatattatttgcgattcatgcatcgcgcacagttttgttgaagggtctg

tgattgtagtatcgtgttagcagcatcccctgatgacgagttgatgaatatacttctcgc

cctatgttggttaccccaagtggaaggnnnnnnnnnnnnnnnnnnnnnnnnnnnnnnnnn

nnnnnnnnnnnnnnnnnnnnnnnnnnnnnnnnnnnnnnnnnnnnnnnnnnnnnnnnnnnn

nnnnnnngcgggcgggacggtgtggatggtgcgccacaggcccacggcccgctgctacgc

gctcaaggtgctctacgggaaccacgacgacgcggtgcggcgccagatcgcgcgggagat

cgccatcctgcgcacggccgagcacccggcggtggtccgctgccacggcatgtacgagcg

cggcggggagctgcagatcctgctcgagtacatggacggcgggtccctcgacggccgccg

catcgccgacgagcccttcctcgcccacgtggcccggcaggtgctctccgggatcgccta

cctccaccgccgccacatcgtgcaccgcgacatcaagccctccaacctgctcatcgactc

ggcgcggcgcgtcaagatcgccgacttcggggtgggccggatcctgaaccagaccatgga

CCCCTGCAACTCCTCCGTCGGCACCATCGCTTACATGAGCCCCGAGCGCATCAACACCGA

CATCAACGACGGCGCCTACGACGGCTACGCCGGCGACATCTGGAGCTTCGGCCTCAGCAT

CCTCGAGTTCTACCTCGGCAGGTTCCCCTTCGGCGAGAACCTCGGCCGGCAGGGCGACTG

GGCCGCGCTCATGGTCGCCATCTGCTACTCGGACCCGCCCGAGCCGTCCCCCGCCACCGC

CTCGCCCGAGTTCCGCGGCTTCATCGCCTGCTGCCTGCAGAAGAACCCCGCCAAGCGCCT

CTCCGCCGCGCAGCTGCTGCAGCACCCTTTCGTCGCCTTGCCACAGCCGCAGCCCCTCGC

CGCCCCGCCCTCATGATCTCGATCCACTCCCGCACCGCACTTCCAAATCCAGCATCAGGT

CTGGTGGAGGGAGGATCGATGGAACCGAGCTGCCGGGGGAATCTTGCAAGGTTCTCTACT

CCCTGATCATTTTGGGTATTCTTCTCGTCCACCATCGTACCATGAAATCATCCATCCATT

GCTGCTGCCGCTGCTGCTGCTCCAATTAAGGTTTCCCTTTCCTCTCCCGTTCCTGTGGTG

TTCGTTGTTACTACTCTTGTTACGACCTTGTAATAGCAAAAGCTGTGTGCACAAAAAGGA

TTTTGTACTGCAAGATTCGTCGAATATAGACACGATATCCGACCCGATCCG

**>HvMKK6(N-term) MLOC_4417.1.X (based on MLOC_4417.1)**

TCCCCCAGCATCCCTCCGCCCGCCGGGAACTTTCCTCCGAGCTCGATCCATCCAGTGGCC

GGCGACAGCGACCGCCCGCCGCGCCGATCGATCCAGCCAGCAACCTGGAGTCCCAGTCAA

CCCAAGTCGCACCACGTGCCACCATTTTGACGAGCTCCGCCGCTGCTACCCCGTAGTATT

TTTCTCCCTAAATTCCTGCCCCCGGCCTCCCCTCACACCGACGGCGCCCCGGCCGCCCCT

CCCCGCCCGCCGCACCTCCGGTGAGCGCCTGACCCGAAGTCCGCGTCTTCACGCCGACCC

TTCCGATCTCCGTCTAACCTGCTTCCGGCTCTGTCCAGCTCCGCGTCCACCTCCCGCCCG

AGAAACCCTAGCCACAGCAAGGAGGAGGCCATGAGGGGGAAGAAGCCGCTCAAGGAGCTC

ACGCTCTCCGTGCCGGCGCAGGAGACGCCCGTCGATAAGTTCCT

gtaggcctcccccattcctcggcctgaatttcatgttttttcttttctttccccattgaa

gccatcaaaggtttgcgctttattatgtctaatgtaatgagagtttttttggggcctgga

tggagttgtttcctatgagtatattatctttgatctgttggtttatttgtgggttgttat

ggagctgggtagccagtagtgggttgctcaagcatagatttgctttgaaggcattaagtg

tagcgcttgatttgaaattggtagcggtctgagctgctgagctgaccctgcaatctctat

gtcaaccaaagtattcaagtccagtgattggtttgaactcgatatgggttgagttgctga

gctgcctgtggtatttcag

GACGGCGAGCGGTACATTCAAGGATGGTGAACTGCGGCTTAATCAAAGAGGTTTGCAGCT

TATCTCCGAGGAAAATGGGGATGAACAC

gtgagttctcggttcaatattttttaatgaccatttttgttgtctgtcaatctgtccaat

tgtgtttgtgttgtatcgcacttcgatcactcatggttgatgcgcaggggcgcccccgtg

ttgcactgttgcgtgcgtcactgagtcagtgcactcagagggtttactgcattcgatcaa

ccagagggagccaacaaaacatccgataagatttgagattgaatcgtatatacaccacta

taaagaaaacgcacatgttgccacacgaagtacaatacaacgcaaactctaaattactga

accaggaacttacatattcaccctgttttcctcagagatgccacaaacctccttgattaa

gtctaaagacctccagcaatcaaagataaccatttaacttgcacatcacttcatgttctg

aagaaacaagaagtgtctcaaaaacattctagacacaaaaaagtagcacggtaaaagatc

aagccttcaatagcttcgacaggaaaaaggacaaggaaaaggaaaacacattatataaaa

tgggcaatttaggccaaggaatcaaggaaacctacatgagtttattgacaggcatctcct

gcgccggcaggacgagcgggagttccttgaacggtctcttccccctcatggttgtaactc

cccggccacaaccaccggctcccggcagttatgcctgacgggatctatactagccccata

ggtcaacaccagtctttcctgcgcacgttgtcctcactcgtgcgcacctcagaagaactt

cctggtcagtcacccatcctcaaagtaccccatgccaagcaacctggaggttctttggag

acgagcttccgaaaaagaaggtgcacctgttgaaatgagtagtctatcattcctattaag

ccaggatgtcacaatggtatcttcctatggttagtgtttcttcggtagaagatgaatatg

gaactggccaaagcctgaaagctagttggggagtaggtatgagaccaggtgaactgccat

ggagatatggagttcggatctgcccagcggaggcatgctgtgagatcggacaaccttcag

ggaggcctgggccacgatagggtagatggaagtaaagattactgtatacccatcaaccaa

agttgctaatttatgaaccgtggggggtcagaggaacccttgcagtttaaatttggtagc

cgattcatccataatcataacatagttgcaaaatttccattattgtttcatgattatctt

gagtagatgttagtttttaatgcatacgattgaaattatgtactctgctgctcgctatta

gttattagatgccactgacagaagtttaatagcttgtgggaataggctatcttgtgccaa

aggttactgtataaccacaacaagaatatattgttcttcagtttgtgtttgttgctattt

ggataatgatcaagttgtttccgaattcttattgcatcttgatttgtttcttttccccag

CAATCAACAAAAATGAAGGTGGAAGATGTGCAGTTATCAATGGATGACCTCGAAATGATT

CAGGTCATTGGTAAGGGAAGTGGTGGCGTCGTCCAACTAGTGCGGCACAAGTGGGTGGGC

ACATTTTATGCCTTGAAG

gtaacagtacactaaattcacctagtgggccgcacaaatgcccatgatggatttttttgt

tgatttagagcactgtgcagactgcttgttttttagtgtttatatttttttcttgtttgt

taacatggaaattgatatctttatctgtttgaacagatttgcattctatgcatttttatg

acaatctgattattctacatccgaagacttggttgttttgtttagtcactttgtgatggc

atgcagtatattcaattagattcacacttatgcacaccattaaagtgagttgtagcaaaa

acatctagtactggctgatctttgcctgcaaaatctccatagaacaccaattttaatgga

aattgacgctaacatatgagaatacatcaggtaacgatatacagccgagtaaacattttc

aagcattgttcctggtctttgcactgctgtggcatttcgtcgtccatatgatctatcagt

caattgactattttcaattttttcatag

GGCATTCAAATGAACATTCAGGAGTCAGTACGCAAACAGATAGTACAGGAGCTCAAAATA

AATCAAGCAACACAGAGCCCCCATATTGTCCTTTGCCATCAATCTTTTTACCACAATGGT

GTAATATATCTTGTTCTGGAATATATGGATCGTGGATCTCTTGCAGACATAATTAAACAA

GTCAAAACCATTCTGGAGCCATACCTTGCAGTACTTTGCAAGCAG

gtacgcatacgtttggcctatggtttgacttctgcatccggaatattcaaattttgacac

atctgatggaagttgaagctacagtctggcacatctaacaccaataatgtcttaataaat

cag

GTTTTGGAGGGTCTGTTATATCTTCATCATGAAAGGCATGTGATTCACAGGGACATAAAG

CCATCTAACTTGTTAGTTAACCATAAAGGTGAAGTAAAGATTACTGATTTTGGGGTGAGT

GCAGTGCTAGCAAGTTCAATCGGTCAGCGTGATACATTTGTTGGAACCTACAACTATATG

GCG

gtatgtaaaaagatctgctttgtcttctgactgcggcttatgctaataatgcttatctgg

ggtgcacaaaatttgctgtcatagttttcttcctttctttcttgtgcag

CCTGAGCGGATTAGTGGCAGCTCCTATGACTACAAGAGTGATGTATGGAGTTTGGGCTTG

GTAATACTTGAATGCGCCATTGGCCGCTTCCCCTATACTCCTCCGGAAGGAGAAGGTTGG

TTAAGCTTCTATGAACTATTAGAAGCAATTGTTGACCAGCCACCACCTTCTGCACCTGCA

GATCAGTTCTCTCCAGAATTTTGCTCATTTATCTCTTCCTGGTATGTCAAACATTATTCT

GAGGAACTACACATGAAATTAGCTCAGT

**>HvMKK6(C-term) : ASM32608v1:4:519072245:519072491:-1**

CATACAAAAGGATCCCGCCGAACGGAAGTCGGCTTCAGAACTCTTGGTGAGCATTTCCTT

CCACTTTACAAGTTGTTGTAGTGTGGTACCACCGGTTCTGTTTGCTAGTTAGTGTTGTGC

TGAACCGTCGTTCGTTCGTTCGATGTTGTCGCAGAACCATGCCTTCATCAAGAAGTTCGA

GGACAAGGACCTAGACCTGCGGATCCTCGTCGAGAGCCTCGAGCAGCCCATGAACGTTCC

CGAGTGA

**>HvMKK10(partial) MLOC_58682.1.X (based on MLOC_58682.1)**

....................ctctttgtcgtccatgccatnnnnnnnnnnnnnnnnnnnn

nnnnnnnnnnnnnnnnnnnnnnnnnnnnnnnnnnnnnnnnnnnnnnnnnnnnnnnnnnnn

nnnnnnnnnnnnnnnnnnnngcccgcggtcgccgaggtggccgcgcggtgcgccctcggg

CTGGCCCACCTCCACGCGCGCGGCGTCGCGCACCTCGACCTCAAGCCCGACAACCTGCTC

GCCGGCGCCCGCGGGGACGTCAAGATCGCCGACTTCGGCGTCTCGCGGATCTTCTGCCGC

GACGGCCAGCGGCGCCCTCCCCGGGTCTCCATCGCCGTCGGCACCACCGCGTACATGAGC

CCCGAGCGGTTCGCGCCCAACGCCCAGGCCGGCTCGCGCGGGGCGTGCGCCGCCGACGTG

TGGAGCCTCGGCGTCACCGTCCTGGAGCTCTTCTTGGGCCACCGCCCTGTCCTGCCCGCC

GAACGGACGCCGTCATGGAAGATGCTCAAGGAGGCCATCTGCTACGGGGAACCACCGTCG

GTGCCGGGGAGCGCGGCGGCGTCGGCGGAGCTACGCGGGTTCGTGGCCGCGTGTGTGCAG

AAGGATCCCCGGAGGCGCGCCACGGTGCCGCAGCTCCTCGCTCACCCGTTCGTGGCGCGC

CGGGACGTCGAGGCGTCGAGCCGCGCGCTACGGCACGTCATCGTGGAAACCATGTAAGAT

ATCGGGACAGAGACAAGATCAACTGTATTCTTGAGAACGTAGTGCTCTTTCAATTATTTT

GTAGCTAAATTATGGTGTAGATCTCTGAACTTATAGACAAGGCATTTCTGTTTCTATTTT

TAAATATTTGGTCTCTCAAGATGGATTCGACTATCCAAAAGTCGTAGCATTTCTTTATGG

AATCCAAAAGTAGTTTTGGTCTTCATGATGCCTCCATCCGTTTGGCATATAAGCGGCTTT

ATTCATATTTATAC

**>HvMKK10-2 MLOC_77796.1.X (based on MLOC_77796.1)**

caaagtactcaacatgacttgataggaccggtttgcataggtcacgtatacaggnnnnnn

nnnnnnnnnnnnnnnnnnnnnnnnnnnnnnnnnnnnnnnnnnnnnnnnnnnnnnnnnnnn

nnnnnnnnnnnnnnnnnnnnnnnnnnnnnnnnnntgacgcaggcgaggcccgcggacttc

GGCGCCGGCAAGGGGCTGCGGCGGCGGCTCGACCCCTGCGCGTCCTACGTCGGCACGGCC

GCCTACATGTCCCCGGAGCGGTTCGACCCGGAGACCTACGCCGGCGACTACGACCCGTAC

GCGGCGGACGTGTGGAGCCTCGGGATGGCGATCCTGGAGCTGTACCTGGGACACTTCCCG

CTCCTCCCCGCGGGGCAGCGCCCGGACTGGGCCGCGCTCATGTGCGCCATATGCTTCGGC

GAGGCGCCCGAGGCCCCCGCCGCGGCGTCGGACGAGTTCCGGGACTTCGTGGCGCGGTGC

CTCGAGAAGAAGGCCGGCCGGCGCGCGTCCGTGGCGGAGCTGCTCGAGCACCCGTTCATT

GCCGAGCGCGACGCCGAGGAGGCGCAGCGCTGCCTCGCCGCGCTCGTGGCGGAGGCCGCG

GAGCTGGGCGACCAGTAGCCAAACTGGTTAATTATTAGGAGGAGAGTTAGGAGTCCATCC

ATCCGTCCAAGCTGATGACGTGTACATAAATCTAGGCCACGGGGAGAAATAAGCTGTCCC

ATGTGGCGTTTCCCTTTTTTTCTTCCCATCCTTTTGCCGTATCCAAGAGGATTCCTTCGT

TCTTTCACGTAAATTGCGACAGACAGTTTTGAGAATTGATACTACCTTCTTCATCACAAT

CAGAAGCTACTCTACTCTACATGTTAGTGCGAAAATCTGATCGATCTGTGATGGTTAAGG

AAATTTCACGTGCTATAAACCTATAAGGCTCAGTTCCTATAACACAGCGAGCAGACACAC

GAGAACAGATGCATTACACAACAGGATATTGACACAGCTATTGTGTGAGTCTATAGGGAT

GAGTGCACGATCAACTATTGTGTGAGTCTATAGGGATGAGTGCACGATCGTATAATCGCA

TACGTGAGTGATTTTGATTGTACTCGTTTAAAAAAAAAAAGACAGAGCACTTTTACTAGG

**>HvMKK10-1/3(N-term) FJWB02050966.1**

ATGGCTATGATACGTGAGAAGAGGCTGCCGCAGCTGCACCTC

GCGCTGCCCGTCCCGTCCCGCGCCGCCGCCCAGGACCTCGCTGTCATCGCCCGGCGCACC

AACCCGGCGGCCACCAAGGCCTCGACTCCGTCGGCGCTGTCCAGCCAGTTCCGCCTCGCC

GACTTCGACAAGCTCGCCGTCCTGGGCCGCGGGAACGGCGGCACCGTCTACAAGGCCCGC

CACCGGGAGACGTGCGCGCTCTACGCGCTCAAGGTGCAGCACTACGGCGACCCCGCCGCG

GCCGCCGAGGCCGACGTCCTCAGCCGCACCGCCTCGCCC

**>HvMKK10-1/3(C-term) ASM32608v1:morex_contig_1636696:1:1144:1 (upstream sequence not available)**

TTCGTCGTCCGCTGCCACTCCGTCCTCCCCGCGGACGCCTCCGGCGACGTCGCGCTGCTC

CTCGAGCTGGTCGACGGCGGGTCGCTCGACTCGATCAGGAGCCGCCGCGGCGCGTTCGCG

GAAGCCGCCCTCGCGGAGGTGGCCGCGCAGGCGCTGTCCGGGCTGGCGTACCTCCACGCA

CGCCGCATCGTGCACCTCGACATCAAGCCGGCCAACCTCCTCGTGAGCACGGCCGGGGAT

GTCAAGGTCGCCGACTTCGGCATCGCCAAGGTGCTCGCCCGCGCCGGCGACCAGTGCACG

TCCTACGCCGGCACCTCCGCGTACATGAGCCCCGAGCGCTTCGACCCGGAGGCGCACGGC

GGGCACTACGACCCGTACGCCGCCGACGTGTGGAGCCTGGGCGTCACGCTCCTTGAGCTC

TTCATGGGCAGGTACCCGCTCCTGCCCGCCGGGCAGCAGCCGACCTGGCCCGCTCTCATG

TGCGCCGTCTGCTTCGGTGAGCCGCCAGTGCTGCCCGACGGCGCGGCCTCGCCGGAGCTC

CGGGGATTCATCGCCGCGTGCCTGCAAAAGGACTACCGCAGCAGGGCGTCCGTCGCGGAG

CTGCTTGCTCACCCATTCGTCGCCGGGAGGGAGGTGGCAGCTTCGAAACGCGCGCTCCGG

AAGCTGGTCGCCGACGCCTCGTCTTCGTTGTAG

**>HvMKK10-4 : ASM32608v1:4:23822609:23823643:-1**

ATGGCGGCTGCAAGAGAAAGACGACTACCGCATCTTCACCTCACGCTCGACGCCCCCACG

TGGGCCTTCCGGTGCCCCGCCCCGGCGTCGGTCACCGTGGCCACGCTGTCCACGTCGGCG

CCTCGGCCGGACGGCGAGTTCCGCCTGATCGACTTCGACAGGCTCTCCGTCCTTGGGCGC

GGGAACGGTGGAATCGTCCACAAGGTCTCGCACCGCCGCACGTCGGCGCTATACGCGCTC

AAGATCATTCACCGTGGCCACCCCGGCGCCGATGAGGAGGTGGACGTCGTTCGCCGCGTC

GACTCGCCGCACATCGTCCGGTGCCACTCCGTCCTCCCGACGGCGTCCGGCGACTCCGCC

TTACTCCTCGAGCTAATGGATGGCGGCTCGCTCGACTCGCTCGTCCGCGGCGGCCATGGA

GGCTTCCCGGAGGAGGCGCTGGCGGAGGTGGCTGCCCAGGCGTTGTCTGGCCTAGCATAC

CTCCGTGCACGCCGCGTCGTGCACCGGGACATCAAGCCGGCGAACCTTCTGGTCAACAGT

GCCGGACATGTCAAGATCGCCGACTTCGGCATAGCCGAGGTCGTCTCCCGCGCCGGAAAA

TACCGCGCGGCCTACGAGGGCACCGCCGCGTACATGAGCCCCGAGCGCTTTGACACGGAG

CGGTCGTTGCATGGCGACGGCGACGAGCAGGACCGCGTCGACCCCTACGCCGCGGACGTG

TGGGGGCTGGGGGTGACCGTCTTGGAGCTCCTTGTCGGTCGGTACCCGCTGCTCCCGGCC

GGCCAGAAGCCAAGCTGGGCGGCGCTCATGTGCGCCATCTGCTTCGGCGAGCTGCCGACA

CTTCTTGACAGCACGGCGTCGCCGGAGCTCCGGGGTTTCGTGGCCGCTTGCCTGCAGAAG

GACCACCGGAAGCGTGCGTCCGTGGCGGAGCTGCTGGTGCACCCGTTCGTCACCGGGAGG

GACGTAGCGGCGTCCAGACGGGCGCTATGCCAGGTGATCGAGCAGCGATCGATGCCGATA

AGCACGCGCGTGTAA

**>HvMKK10-5 ASM32608v1:4:23882277:23883281:-1**

ATGGCGGCGGCCAGAGAAAGACGGCTGCCGCAGCTGCACCTCACGCTCGACGCCCCCGCG

TGGGCCTTCCGGTGCCCGGCCCCGGCGCCGGTCACCGCGGCGACGCCGTCCACGTCGGCG

GCTCGGCCGGACGGCGAGTTCCGCCAGAGCGACTTTGAGAGGCTCTCTGTCCTTGGCCGC

GGGAACGGTGGAATCGTCCACAAGGTCTCGCACCGCCGCACGTCGGCGCTGTACGCGCTC

AAGATCATCCACCGCGGCCATCCCGGCGCCGATGAGGAGGTGGATGTTGTGCGGCGCGTC

GACTCGCCGCACATCGTCCGGTGCCACTCCGTCCTCCCGACGGCGTCCGGCGACTCTGCC

TTACTCCTCGAGCTGATGGACGGCGGCTCGCTCGACTCGCTCGTGCGCGGCGGCCAGGGA

GGCTTCCCGGAGGCGGCGCTGGCGGAGGTGGCGGCGCAGGCGCTCTCCGGCCTGGCATAC

CTCCGCGCACGCCGCGTCGTCCACCGCGACATCAAGCCGGCAAACCTCCTGGTCAACAGG

GCCGGCCAGGTCAAAATCGGCGACTTCGGCATCGCCGAGGTCGTCTCCCGGGCCGGCAAG

TACCGCGCGGCCTACGAGGGCACCGCCGCGTACATGAGCCCCGAGCGGTTCGACACGGAG

CGGCTGCACGACGGCGAGGAGGGCCGCGTCGACCCCTACGCCGCCGACGTGTGGGGGCTG

GGCGTGACCGTCCTGGAGCTTCTCATGGGGCGCTACCCGCTGCTCCCGGCCGGGCAGATG

CCGACCTGGGCGGCGCTCATGTGCGCCATCTGCTTCGGCGAGCTGCCGGCGATTGCCGAC

GGCGCGGCGTCGCCGGAGCTCCGGAGCTTCGTGTCCGCCTGCCTGCAGAAGGACCACCGG

AAGCGGGCCTCTGTCGCCGAGCTCGTCGCGCACCCGTTCGTCGCCGGGAGGGACACGGCA

GCTTCGAGACACGCGCTCCGGGAAGTCATCGAGCAGCGCCAATAA

**>AetMKK1-1 : ASM34733v1:Scaffold59969:73755:76762:-1**

ATGAAGAAGCCGGGAAAGCTCGCGCTGCCCTCCCAGGACTCCACCATCGGCAAGTTCCTG

TAAGCAATCCCCCTCCCTCCTTCGTTTGTTTTGTTCTTCGTGGACGCGTTCCGTCGTGCT

GATTGGTTTGGTTTCGCGGCGGCCGTGGTCCGGGGGGCAGGACGCAGAGCGGGACGTTCA

AGGATGGCGACCTGCTCGTCAACAAGGACGGCCTCCGCATCGTCCCGCAGAGCGAGGAAG

GCGAGGTAAACCAACCAACCACACTCAACCCGGGCAGGCACCCCCTAGATTGAAAATTAA

AATTCCCCAGCCCGTCTTGTCCTAGACGCGTAGGGCTGGACCTGCCTCGCAGATTGGTGC

AGCCCGTACTGTTTTTTGTAATTCCGTTGATGCCATCAACAGATTTTAAATTTCTTTCCG

GTCGCGTGATTGGATTAGCGGCAGGTGCCCAGCAGGGGGTTCCTGTCCTCCCCTGGTTAC

CCTTTCAGAAGAACGATTTAGGAAAAATAATGATATGCACTATCATTAATTTTAATTTTA

TTTTATTTCCGTCAAAAATTATTATGTCCCCTCTTTCAGACCAGCCATTCAGGCAAAAAT

CTAATCATTTCTTCTCAAGAAGAGATGGCGGCCTTGTATAGATGTAAACTGTACTCCCTC

CGTTCCTAAATATAAGTCTTTTTTAGAGATTCCAATATAGACTACATACGGAACAAAATG

AGTGAATCTACACTCTAAAATACGTCTATATACATCCATATGTAGTCCATATAGAAATCT

CTAAAAAGACTTATATTTAGGAATGGAGGGAATATTTTTTATAGTGGCCTTGTATACACG

AGCAGAAAAAATTGGGAGGTCTGATTGGATCAGAGGGTAGGGAGGAGGGGTTTCCTATCC

ACCAATTTCAGAATAACGTCTTAGGAAAAATGATGGGAGTACTACTATTTAGGATTTATT

TTATTTCCCTTAGGTGGTGGAATACATGCGGTCATCTTTCATGTGGGGATATCCGCCTAT

GCAGTTTATTACTGATGCCATTTATCATGCATCCAGTGGTTGCTCATATTTATCCGGAAA

TGCTGTGGAAGAGTGCTCCATTTTCTGTGTGGGAATTGTCTAGTTGCATTCTTGTCCTTA

ACTTGTTATAACTCTTCATTCATCTAGTCTGGGTCTCACCTTCTTTCTGTTGACATACTC

GACTTCTCTAGGCTCCTCCTATCAAGCCGTTGGATAATAATCATCAGTTGAGCATAGACG

ATCTAGATTCAATCAAAGTGATCGGGAAAGGTAATAGCGGAACCGTGCAGTTGGTGCGCC

ACAAATGGACTGGCCAGTTTTTTGCTCTCAAGGTATTGTCCCAAACATACATTATTTGAT

GTTTTTTTCTTTATATGTTTGCTATGTATTTTTGGTTCCTTCGACTTTTTTCGTTACAAA

ATTATGGTGGTGACTGTAACTATCCTCTCTGCTATTTTACCTATATATGTTACAAAGGGT

CTTCACCTCCATTGCTGCACTGTTGTCATGTCTGCAGGTTATACAGCTCAATATTCAGGA

AAGCATACGCAAGCAGATGGCCCAGGAGTTGAAAATAAGCTTGTTTACACAGTGCCAGTA

TGTTGTCACATGCTATCAGTGTTTCTATGTCAATGGTGTTATTTCTATTGCTTTGGAGTA

TATGGATGGTGGCTCTCTCGCTGATTTCCTCAAGGCTGTTAGAACCGTTCCTGAGGCCTA

CCTTGCTGCAATTTGTAAGCAGGCCAGTGAAATGTCACATAATCAACCACAAAGTTTCCC

CTTCTTCCTTTTTCGGCTATCAGAATGTCGCCTCAAGATGAAATAGACCTTTTCAATCGA

CAATTGCAACTTCCTCATGCAGGTGTTGAAAGGGCTGATGTACTTGCATCACGAGAAGCG

CGTTATACACCGAGATCTGAAACCATCGAATATATTGATAAATCATAGGGGTGAAGTAAA

GATATCAGATTTTGGTGTTAGTGCCATCATTTCTAGTTCTTCTGCACAGCGAGATACATT

TACTGGCACATTTAACTACATGGCGGTGAGTGAAAGTTTGTTTAAATTATGTAGACATGT

CTTCCAGTGTGGCATGAAAAGATGTTCATCAATTTTTTTGTTTCAGCTAACCAATGTAAA

ATGTATACTCTTGACAGCCTGAAAGAATCAGTGGGCAGAAACATGGTCATCTGAGTGATA

TCTGGAGCTTGGGCCTAGTTATGCTGGAATGTGCCACTGGCAATTTCCCATATCCTCCTC

GTGAAAGCTTTTATGAACTTCTTGAAGCTGTTGTCGACCAACCATCACCTTCTGCACCAT

CAGACCAGTTTTCACCAGAGTTCTGTTCATTCATTTCTGCTTGGTATTTGTGCTTCTCCA

TTGAACCCCCTTTGTTAATCAATGTTACTCGGGCAATATTTGCAATTTTTAGATCTAGAG

CTCTAAAGTATGAGCTTAGGTACAGTACCATTATATGTTTGCGGACACGTATTATCTGAA

CTTCCAGATATTTTTTGTTTTTGTCTCATCTCATCTGTGGGCAGATTATTTGATCTTTTA

TTTATTTATTTATTTGTGGAACATCAGTGTCCAAAAGAAGGCTAATATCTGTTTTTTTAT

TGTTTTTTTGGATTTCAGTATCCAAAAAAATGCTGCAGATAGGTCATCTGCCCAAACCCT

ATCAGTAAGGAAAATTCTGTTTGCTTCCCATTTTTGTATGTACTTTTGAAAGCATCATTA

GTTGGCTGACTGATCTTGATCCAATTCTTCTATCTGTTGCCTCGACGTCCAGGTTCATCC

ATTCCTGAGCATGTACGATGACCTGAATATCGATCTTTCTGACTACTTCAGGACCGCAGG

ATCACCGCTTGCCACCTTCAAGTAATCCAATGACCCACTGAAGAGTACATACATGGTTAC

TGCAATTCCTTTACAATTCCAGTGATGGTCTATTCTCCTTTTTTTTTCCTAGGCAAATCG

CGTTGTGA

**>AetMKK1-2 : EMT27166.X**

..........agaaattatagtggtaattgagtagaggggtagctggttctgattaattg

ATGGAGCACCTGCGAGAGCTCAAGGAGGAGGTGGAGATCATGGAAGAGGAGGAATCTCCT

GAGGTGCACAAGAACGTCTTCCTCTCAGATCCAACTCCTCCTACCAGTGGTGAGGCCCTT

TCACCAA

gtaatcgcatgcaggactacttaacttgttttctttcgatgggcataagaatggataatt

gtcgtgccatgtctgcattgtgaccacaagattgtcgtcttgttttgattgcttgttcct

tgatgcgggtccccgatttcaacattgtgcattttgctttgtttatgtgcgtgtgtacac

aatattcattaattaattttacctttgtttatgtgcgtgtgtttagatgttttgatctga

agcagag

GCAGAAGCAAAGTCAGGGAGACGGGGGCCACGATCAGTGCTTCGTTGGGCGCCATGAGAC

CCCTTATTGGGAAGTTGGACATGCTTCTCCTTAGGGATGCTCCTCAGAAATGCTGCTCCA

AGAGGATCAAGGACAGGATGCGCCTCCTCAAAGATGACGTTCAAAAGATAAGTTCCTACC

TTGATGAACTATCAGAGGTGGAAGACCCTCCACCAATGGCCATGTGCTGGATGAATGAGG

CACGCGACCTGTCTTACGACATGGAGGATTACGTCGACAGCTTATTATTTGTGCCGCCTG

AAGATCCCTCCCTTTTTGCCAACAACATCAAGGCCACCAGATCCCTCCGCAAATGGTTCA

GTCGTGTCAAGACTTCCCAGACTCAGGTTATTAGTGCGGGAACGTTATCAGAATTCAGGA

GGTATGTCCAGGAGGCCATTCAACGGCACCAGAGGTATAATCTCCATTCTTGCAGAACCT

TGAGGCGTAGGTTTGTGTCCCATGGCCCTATGGTTCTTCCAAGGCCATATGAAGAAACTG

CAGACATAGTAATCGATGGCCGGATGAATGAATTTATCAACTCACTGGCTACAGATGGGG

ACCAGCAGCTCAAG

gtgctttctgttcttggatctgcttgtcttggtaaaaccacacttgctagagtgttgtac

aacagatttaggaagcaatacaattgccgagctttcattcgagtatccaagaagcctgat

acgaagaaaatattctgtgacatgctctcacaactcaagcggcaacatcctctgcaacat

tgtagggaannnnnnnnnnnnnnnnnnnnnnnnnnnnnnnnnnnnnnnnnnnnnnnnnnn

nnnnnnnnnnnnnnnnnnnnnnnnnnnnnnnnnnnnnnnnnnnnnnnnnnnnnnnnnnnn

nnnnnnnnnnnnnnnnnnnnnnnnnnnnnnnnnnnnnnnnnnnnnnnnnnnnnnnnnnnn

nnnnnnnnnnnnnnnnnnnnnnnnnnnnnnnnnnnnnnnnnnnnnnnnnnnnnnnnnnnn

nnnnnnnnnnnnnnnnnnnnnnnnnnnnnnnnnnnnnnnnnnnnnnnnnnnnnnnnnnnn

nnnnnnnnnnnnnnnnnnnnnnnnnnnnnnnnnnnnnnnnnnnnnnnnnnnnnnnnnnnn

nnnnnnnnnnnnnnnnnnnnnnnnnnnnnnnnnnnnnnnnnnnnnnnnnnnnnnnnnnnn

nnnnnnnnnnnnnnnnnnnnnnnnnnnnnnnnnnnnnnnnnnnnnnnnnnnnnnnnnnnn

nnnnnnnnnnnnnnnnnnnnnnnnnnnnnnnnnnnnnnnnnnnnnnnnnnnnnnnnnnnn

nnnnnnnnnnnnnnnnnnnnnnnnnnnntagggaaattgatctcattcacgatatcaagc

aatatctacag

GATAAAAG

gtaatgccttttttcttcattcaagataatatcaaaattcccatggtccctaaaccctta

gagttgattatctgtatgcag

GTATTTAATTATTATTGATGATGTATGGGCTGCATCAGTATGGGATACTATTAATCATGT

TTTTCCAAAGGGTAATCATGGCAGTAGAATAATAACAACTACACAGATTGAAGATGTTGC

ATTAACATGTTGCTGCTATCAGTCAGAGTATGTTTTTGAGATGAAACACCTAGATGATGA

TCATTCTAGAAAGCTTTTCTTTAACAGACTATTTTGCTCTGAAAGAGATTGTCCTGAACA

GTTCAAAGATGTTCTAAATGAAATTGTTGAAACATGTGATGGTTTGCCGTTAGCCACAGT

TAGCATAGCAAGTGTTTTAGCAAGCCAGCCTGTCATGTCAATTGATCTTTTGACATACAT

CCATCGGTCGTTAAGCTCTTGTTTCTCGGCATCAGAAAGAACTAGACAAGCACTGAATCT

GAGCTTTAACAGTCTTCCTCAATATTTGAAGACATGTTTGCTTTATCTTAGTATGTATCC

AGAGGGCTACACAATCTTGAAGGATGATTTGGTGAAGCAATGGGTGGCTGAAGGTTTGAT

CTATACAACAGAAGGGCAAGACATCCGGAAAGATGCAGAAAGCTATCTCGATCAACTTAT

TGGTAGAAGATTCATCCAACCGATATGTGTCAACTACAACAATGAGGTGTTATCCTGTGC

AGTGCATGGCATGGTACATGATCTTATTGCACACAAATCCGCAGAAGAGAATTTCATTGT

GGCAATAGACTACAGCCAAAAGAATGTGCCACTCTCTCAAAAGGTCCGTCGACTATCTCT

CCTCTTTGGTAATGCAAAATATGCCAAGATACCAGCAAACATCACAAAGGCACAAGTTCG

GTCAATTGGATTTTTTGGATTACTCGAGTCTATGCCTTGTATTACAGAGTTCAAGCTTCT

TCGTGTCCTGAACCTTCAACTGTTCCGTCATGCTGGCGATGACGACGGCCCTGTAGACCT

CACTGGAATTTCAGAAATGTTTCAACTCAGATATTTGAAGATTGCAGGTAATGTCTGCAT

AAAACTGCCAAACCATGTGCTACAATGTTTGGAAATACTGGATATTGCGGATGCAAGAGT

TGCTTGTGTTCCACGAGATATCCATTTACCAAACTTGTTACACCTTGGTCTTCCTGTTGA

TAAAAATCTGCTGGATTGGATTAACAGCAGGATGTCTCTCAGTGTGGAGAGAGTACGCAA

GCTGCAGGATCTTCACCTGACCAGATCTTCTGCACTTTCTTCTGACCATCTGAATAGAAG

CATGAGTTCTATTTTATCTTTACTTGGAGGACATGGCAACCTGAAAACTTTAGTAGTGGC

TCATGGCTCATCGGCTAAAAATGCTCGTGGCGCTTCAGATGTCACCCTGTCATTGGATCT

CCTGGCACCCCCACTTCTCCAGAGATTTGAATTCTTGCCACATAGCCATATCATGTTCTC

GCGAATTCCTCTGTGGATTGAGAAACATGGCAACCTAAGAATTTTGAAGATTGCAGTGAG

GGAACTGCAGATGAGTTGTGTTGATATCCTTAGAGGATCGCCTGCCCTCACTGCTCTCTC

TCTGTATGTGGAGAAGGCGCCCTACGACAAGATCATCTTTGACAAGGCTGGGTTCTCAAT

TCTCAAGTACTTCAAGTTGAGGTTCACGAGTGACATAGCTTGGATAAAATTTGAGAAAGG

TGCAATGCCTAATCTCTGGAAGCTCAAGATAGTTTTCAATGCCATCCCCTCTTTTGAAAA

AGATGGTACTGCACTCGTCAGCATCGAACATATGCCAGGCCTTAAAGAGATCTCCACAAA

ATTTGGGGGTGAAGCTCCTGATCTAGAGTATGCCTTGAGGACCTTGGTTAGTAATCATCC

GACAAATCCTATAATTAACATGCCATCAAATAAGACTCTGGGGGGAGAACAACCACATAG

GAATCTGGAGCAAGAATCAGATGAGATTCTGAAGGAAGATCCATATGGGAATCTGGAGCA

AGAATCGGATGAAATTCTGGAGGAACAAGAGCCAGATGAATACGATGAGAGATTAGAGAG

GCGACAGGCTGATAAAAG

gtactacaaatgttccttgtaagcacaacgaaatatgccctcctttacttcaacttctat

gagtctgggtgcacccacttggtgtggacgcctcaatcaataaaatttttctttgaaaga

aaaacatcaacgtcgattcttcagttaacctgatataattcttatatgttctaacgaagc

taatggttattgtaatcaatcag

GATTTCAAGGTCATCAGATCCATCTTCGCGTCTACATGTTCCAG

gtacattttctgtcaactggtggtaatataaatgtttcgccatcaagttaataaagtcac

ttcaagagaaaactaagcagcttgttctataaaggaaaaggacagacccagtgcatagaa

gctcccacacaaggtggggtctggggagggattataggaacctagtcttacccctgcaaa

gtgcaatgcagagaggctggttcgaacccaggacctcttggcacaagtggggaggacttc

accactgcgccaggcctgccctctaagcagcttgttctatgtgataaaaaatttgtcccg

gactaccaattcggaaacgattaatctctccaaccgctgacttgttagagatagaggctt

gagtggaaaccggtgaccgtagccaacccatctagtgtaattgcatccgtccatccatga

cgtcggcctctagttacaaatgagactttgatcctgggccagccgaatcagaaaagaatc

cctgattgcacgtagcctggatttcaatctcctattttgtcttgcactttcttgtttcat

tgcttgctcaagcgagatgcatcttcccctattttcttgcccccaaccaatcgcattcac

ggcttggactgcagcatgcacaccttgaaaacatgcctaatttccggtcctatctctctt

cacttatgcaccttagttatgtgctaaaatagcatgctttatcagattaaaaaatagcat

gctttattgttatacaaactaattcctctttttcttcgtaattgagttgcatatttcttc

acacaacatcagtaacgatagcaggggtatgcgtaagcgaagccaccggatcactttcct

gacaaaaagaccttgggacatctggggtggttctttgctgcttcttttcaatcgtgtcct

gtttcatcgctcagtcgaccatggagagcagtctcctttcttagttgctactttggttac

ttcttgggggcgtgttgggttatatatatagtctctcttcatgcaggcacatgacatttt

ttttaatttttattaagcagtaccagttggtgcttctgagatgggaagtgagcctgatgt

ggaaatccctgcggcctactaatgataacacgatgttgttggtcaatctcgaag

CATTTGCTAAGGAAAATTATCCGAGCACTAACTTGGAAAGTTCTGTAAAGGAGATGGAAA

CAGTGGCAGGTTTAGAGGATCATCAGATGGAAGATAGCACACAG

gtaatttacacattccaactgttttgaattcttctgatccttcttagggacgtagctact

aggattggggtgaagttaggaagtaatattcttgaggtaaataaatatatttaagtgact

agtatggaagctggtagatccgccttctagccttggcttctacagggtgtgtgcctagat

tgtttcttctaaattaaaattctacaagggtagtgattttatagctcacaagagaagtct

aacaatgtgcctttgagtggggaaccaaaacaatttttttttaactcttaaggctatcca

ggatttactcgcagatgagtatggggttgatttagaaaatgttagatagctgtgttagaa

gtttagcttcttcaattagacctaaatggtaggacatggagtatctactcccgagctcaa

atgctccctgatgaacagtaaattcgaaaaaaaatagtaaacaaattctgttttttctgg

taaactttgacaaatattttgtgtgcttgcaaatttataataacaggacattcgtggaag

tcgtggcaaaaaaacaaaatcgatgctcccaaaatgctatttttgaaagcattttggagt

attgtttattttattttatttttgccatgaatgttatttcgtgatgaaaattttcaagca

taccaaacatttgtcaaagttcgtcacatttttttagaatgtttcaaattttttttactg

ttcatcccgagctcaatgagctcgggagcagaagaggacttctgtaaatggtaaaggtgc

ttttgttggtggtaggagaggtacagaagtgataattcaaaatggctcccaagagcacat

gcttctggctgcaaaaactaatttttcggatgtccaaaaaatggaaaaaaatggatgtct

acttttttgtgtcctttgtgtacatgtaaattttcatgagaaaaaaaaacttttgtgccc

tgtgtgtgaaaaaactattttgatacttcaagaaatgcttcttagagcactttcttgctt

caagaaatgcttgtgggaatagtattttaactaagtacaatattatcaaaaggggctgga

aaggtaaaactgaatgtagctataccatagtaaaagcacatgttacgtttcgatcatttt

tatctcag

CCATCAACGGCATTTTCCAGGAGTATCTCAGCCACAACTGGCTTTTCCTTGTATTACCGA

GAATTAAATTTGGATGGAAAAAGAGGGAGACAAGTCCCACACTCTCGATATGGCGATACC

GCTATGAGTTTGTGGAAATTACCCACAACCTGGGTTTTGGTTAGCGGGCG

gtaaattcggttaccgcctggccgggcgtttcttgnnnnnnnnnnnnnnnnnnnnnnnnn

nnnnnnnnnnnnnnnnnnnnnnnnnnnnnnnnnnnnnnnnnnnnnnnnnnnnnnnnnnnn

nnnnnnnnnnnnnnnnnnnnnnnnnnnnnnnnnnnnnnnnnnnnnnnnnnnnnnnnnnnn

nnnnnnnnnnnnnnnnnnnncccccccccccccctccctccctggtcttcgcaagtgaat

atgttggaaatgatatgcataaactgttccaatctttttactaactccag

GGGCAGTAGTACTAGTAAAATCAGGCTCATTCCTGGGCCTGCAGCTAGTGATTCTGTAGA

GTGGCTTCTTATAGCAGGACCTAGTACAGCCACCAGATTCCAGAGCTGTCAACGTGGGGA

CAGGAACCACAACATGCCAG

gtttgctggactagccgttgatttttgctgtggaatctgggtgcttggggatgaaagaga

ggttgatactgagaactgcctgtcctaagccttcatgtcactgtcacggcggcttccgtg

tgagcttccccagagacagatggcgtcgttcagtttgcagagaaatcgcacatcatgttg

tctaggatgctggagtttccaatgaggtgtttactatgcaaaatcgcaatcttttggttt

ctagaacttcttttaggaagttcaaaacctctttatgagcttttcttctcactctttctt

tttgcggggtgcttttattcttactagccttgctatggtaaacaaacaagcttttcacat

agggcctagaataacacttttttatccatctttaaaaatgatcagagtatagaaatttta

cttagccggacgagaaaataagacatcacaaatattgcggctttcgaaagtgagcttctc

ttctcactagacttgtcatatagacaagcctttcatgtagcacaccacttctttttggtg

gctatagccggttttaactttttaaagatgattgcttgcttaccatagcagagaggagcc

gggacttgcattttctgcagtattttcatctagctgaatcagtagtggatgtgtgggtgg

attcggcgccgtcttgcgcctcccaaactggatcttgccgttagcgtgaaaagctttggt

cttgggatagaataccacttttgcactctcagtagaatcctcttaaaatttaaaggattg

tagaggactttaattcttaagaatcttactacgaggtttgtttgattaagtaagattgca

attgtacgtttatttttgtctacacatcctatttatgtatgtacatggtgccttattgtg

cactatgtatattttttcaagccgttgaccaattatgccaaactgccaattttgccagcc

atttatactggtactgtaatgcttaattccagaaaaagcagagcaaagatgaggcggacg

aagactcatccatccactcaccccggcgagggagccctacaccgccgttgctgttgaaga

agccgggcaagatcgctctcccctcccacgagtccaccatgggcagattcctgtaacctc

aaaccctcacccctcttctttcgcatttttgttttccttggatgtattgctcttgcagtc

gtgccgattgatttgatttcacggcggttgtggtcagctgggctccag

CGTGGGACGGGGACAGGATGCAGATCGGGATGTTCAAGGACGGCGACGGCAACCTGTTCG

TCAACAAGGACGGCCTCCGCATCACCCCGCAGACCGAGGAAGGCGAG

gtaagccactaaaacatgaccgggcacccaagatgaaaaattcctccacgcgtctggtct

tagccgttcatagggaaagggaaagaatgcaggactgtgctcggcatattggtagttgta

ttggttttcgtaatttcattgatgacatgaacagatttagatattaggatgtgtggttgg

attagcaggtacggagcaggggttttgtattctttttcagaacaacaatttaggacagat

gatactatgtacttccttcatccgggattagtgctcaaacggatctatctagacgtattt

caatgcgagatacatccgtttgagcgtcaactgattccgcgcggaggaaatacataggta

attttcatgtgagatatccgcaaatgcagttgttacagaaataatttatgatgtatccaa

tggttaaatttatcctgaatgctgatgctcatttgcttatagtggaagattgctgtctaa

caccatttcttgtgggaattgtctaattacattgttgttcttaacttgttatgctcttca

ttcatctaacttgtgtctcgccttctttctgttgccatactcgacttctctag

GCTCCTCCTATAGAGCCGTTAGATAACCATCACTTGAGCATACATGATCTGGAAGCAATC

AAAGTGATTGGGAAAGGTAGTAGTGGAACCGTGCAATTGGTGCGCCAGAAATGGACTGGC

CAGTTTTTTGCTCTCAAG

gtattgcccaaaaaataaattatttgatgatttataattttggttccttctatttctttc

tgtagaaagtagaaactatagtggtagttataatctctgatattgtgcctagggtcttta

cccccattcatgtactgtcatgtttgcaggttatacaactcaatattcaggaaagcatac

ggaagcaacttgcccaggagttgaaattaagtttgtcaacacagtgccaatatgttgtca

catgctatcagtgtttctatgtcaatggtgttatttctattgctttggagtatatggatg

gaggctctctggctgatttcctgatgactgctagaaccgttccagaggcctaccttgctg

caatttgtaagcag

gccagtaaaatgacacataagtaaccacatagttttttgttgttat

cagaatgtttttttcacctcaaggtcaaatagaccttttcaaccgataatttttacttcc

tcgtgcag

GTGTTGAAAGGACTGATGTACTTGCACCATGAGAAGCGCGTTATACACCGAGATCTGAAA

CCATCGAATATACTAATAAATCATAGGGGTGAAGTAAAGATATCAGATTTTGGTGTTAGT

GCCATCATTTCTAGTTCTTCTGCACAGCGAGATACATTTACTGGCACATTTAACTACATG

GCG

gtgcgtgaaattttgtttgaattatgtaagcatgtcttccagtgtggcatgaaaagacgt

ccaccattttttgtttcagctaacgaatctaaaatgtatactcttgacag

CCGGAAAGAATCAGTGGGCAGAAACATGGTTACATGAGTGATATATGGAGCTTGGGCCTA

GTTATGCTGGAATGTGCCACTGGCAATTTCCCACATCCTCCTCGTGGTAGCTTTTATGAA

CTTCTTGAAGATGTTGTCGACCAACCATCACCTTCTGCACCATCAGACCAGTTTTCACCA

GAGTTCTGTTCATTCATTTCTGCGTG

gtatttacgcttctccattacccccctccccctgttaatcaataatacttaagcaatgtt

tgcaatttgttgagatttaaactgtgaacttagttatcatagtggtttttctaccttttg

cctgtcaagcagtgattctcacgccaaaattcctgctctggaatatgcatgccaacatca

tcatatgtttctttttgttacactcacctagtcaccttcatctgtggacagatcaactga

tttccttattttatttactggaaatcag

TGTCCAAAAGAAGGCCACAAATAGATCATCTGCACAAATCCTATCA

gtaaggaaatttctgtctgcttcacaatttgtatgtttgtccgaaagcgtcatttgacac

agcaagctctctgatctttacccatttcttctatctgttgcctctgacacccag

GCTCATCCATTCCTCAGCATGTACGACGACCTGAATGTTGATCTTGCTGTCTACTTCAGG

ACCGCGGGATCTCCACTTGTCACCTTCAAGTAA

**>AetMKK3 :** EMT30938 + EMT30937

..........cgccctctccctcacccttctcctcttaggcttagccttgtttggctgta

ATGGCTGGACTGGAGGAGCTGAAGCGGAGGCTGCAGCCCATCTTCTTCGACGCCGATGGC

AATGTTGTGCCGCCCCCCGCCGCCGACGGGACGTCCGACGACTCCAGCTCTGACGACTGC

GAG

gtaaccactctcttcgtttcagccgtttggtcagttcgctgagatctatgtttatcttca

ttctgttcaaccaacaaaattccttgcttatgttgctcatagtatgtttttttcttaagc

ctgctataaaaatacatatgatgctcatattttaatcttacttttgtaaag

GTTTTGGATGGTGGAACTGTCAATTTATTGAGTAGGTCCTCTGATGAATATAACATCAGT

AAGCTTGGCTTCCATAAACGAACAACCAGACCAGATGGGGACTATGCTACAGATAAGGCA

TATCGATGTTCTTGTCATGATATGCATATTTTTGATTCTGTTGGTAACGGTGCAAGCAGC

GTTGTCCATAGAGCTATTTATGTACCAGTCCATCGAGTTTTGGCACTCAAGAAAATCAAC

ATTTTTGACAAG

gtgagtgcaaaactttttatagtccatatgccataagctgacatctctatgttcccctta

catgaaacaaaatctatggatacccccccttttttttcctttttcttttaattaactttc

atatggaactaactacaagtgtcaaattcatcgcttgttctccatttaatgcctatagta

ctccagtattagtttttttttttttttttgcgcatagtactccagtattagttggaaatt

gtttgaaccaagctgcagcaagtagaaccggaaatgcagcaagtactatagactttgttt

ggaccaagcttcttctctttatatttattataacgtattctctggtatatattacag

GAAAAGAGACAACAAATTCTTAATGAGATTAGAACGTTATCAGCAGCATCTTGTTATCCA

GGTTTAGTTGAATTCCAAGGAGTTTTTTATACCCCGGACTCTGGAGAAATATACTTTGCT

CTTGAGTATATGGATGGTGGTTCATTAGCAGATATTATCAGGGTCAAGAAATTCATAACA

GAACCAGTTCTTTCACATATGCTACAGAAAGTGTTGCTA

gtacgtgtctcttgcctccataagttctgatgttagctctattcaaatcaggcctgtgaa

ctttaaatgactagttggtctgtgttaatttattag

GCTCTGCGCTACTTGCATGAAGTGAGGCGTTTAGTTCACAGAGATATAAAGCCAGCAAAT

TTGCTGCTAAATCTAAAGGGTGATACGAAAATTACAGACTTTGGTGTATCTTCTGGATTG

CATGATTCAGTTACCATG

gtatgctatttcttggtgaccgaaacttctcttagcaacagcaagctgttctaaaatagt

attaagtggacatctgagtatctagattgaataagcagtatatatgtgtagtaattgtct

tccag

TGTGCTACCTTCCTGGGCAGTGTCACATATATGTCTCCTGAGAGAATTCGGAACGAAAAT

TACTCATATGCTGCTGATATCTGGAGCCTTGGACTAACAGCATTGGAGTGTGCAACTGGA

AGATACCCATATGATGTAAATGGGGGCGAGGCCGACCTCATGCTGCAG

gtgaggtggttggtggtcaagcagtgttcatttgtataattttctactgcctttcgtgat

catcttatttatggaaaatatcatgatctcatttatgtgtatctgttggtatattgttga

gtaaagctgttttcggaatttggcttgtttttcataggcgttctacatctgtttccgaat

ttctactggatttagaacttctatttcatttctgctttccaacatgaatggtctgacaat

aatgcaatactagttcggtagttcctcttgtgagtattatattattgatgtataatgcaa

tcctcctttcccaccttactgtgttgcagtcttgcagatattggaagatccatcgccaac

accaccgcaacatatgcattcagaagagttttgctcgttcatcgatgcttgcttgcag

AAAGATGCTGATGCAAGACCAACATGTGATCAG

gtaaagtgacgtctagtacaattaatgtttttcaaaagaaacggtaggagggtttccgac

tgcgtaaatcttgtgataatctttatttatttatttatttattgcggggagtgataaact

ttatgatgcagtatagggttttgcgacactatctgagagtaatgtctgatggatacagat

tagcggtctaaggtgtattaatcacacacacacacacacacacacacacacaattctgag

gtgataattgcaaaaaagtggtctcatatgttgaattgttttgcaaattatccattgtca

actaactacttaaataggttatttgcaacaaagacgaaatggggtttattttgctctctg

aaattaaaaaaatcttatttgaatcacatactaaacatcttgtagcttttgtctcattcc

ttcatcaag

AAATACGAGGGACCTGGTGTGGACTTGGCAGAGTACAACAAAAGTGTTCATGATCCAGCG

GAAAGATTATCACAGATAGCACAT

gtgagttcctaatagccttcgttagcttatatttttcctgttctgctgcctttccatatg

gcag

GAAAATGAACCTGATGTTCTCCAAGTTCACATACAATGA

tgttagctatgcttagacaatagaacattccaacaattcagcaaataaac

tgactcttactgcagttgtttcaatctttttaatctggtttcttgctatcc

gggattcaaaatattcatgttaaaattttagacctcgttgaggatttcggttgccacaag

catagtttaaaaaaccagaccgggccagcggtcggaccgaaaaaaactggaaccggcggc

gtcggcggttttttaagctaataagacggttctgcaattggaccagagaaaaccagtcaa

gccggccggttttctggaaaaaaaacgacgaataaaccaggcctttgaaccggaaaaaac

caggaaaatgtttaatgtagaatttgggagaagcgggattcaatcctaggatgtgtgagt

agtaggcattgcgtgctcatggcccagtaaccaactcggtcgtgctactttgttgtctat

tatagggaagtaattttatttgaccattgtttaacactgttttatttggccattgtctac

taaacgtatattattttatggtaaaaacccgacgaatgaaccggtgaaccggcggtccaa

ctgacgaaaacctgaaccaacaggctcaccggttcgatcttcggttcagttttttaaact

atggccacaagtatatctcagaaaaataaattggagcagggaaaaacgtgctttgtggtt

ttgctattcatggagtgggacgaatttacaggaactttgccactgccaggtcacttactg

tttcgcttgaatatactcggccctaaatgtttctccccttgaatatgaatgcgcccgcca

tgctcacgtggccgctcacctcagctatagccgcttacgccgctgctctcttcacctcca

accactcttgaaatagtctttcaccccgctttatagataaagcaaccaccatttccacac

aagtagttaaagtgcaggaaagtaaatagaaggtctgctggggctcaaataagcccaaaa

gcaaaaaagagaaggcgacacaagactccggagagttaaaagatcaacggatagggcgcg

aagcgagttggcgggctgccaccagaagatcatcaatcatgccattctagccggtccctg

tccgctgcctacaaagcaggtaccactgctgcaagaaggccaaaattttaaacagagtca

gaagcacactgcaagaagactcgcttgataaccatcttgtcacgtacagtccattgggtc

caggtaatcactgcaaacactaaccagaaggggcgcctcctcctaccggtatgtttggtc

tgagcctccaggaactcgctcaggtccggcgcctgccactcaagccctagagcctcacgg

acaaagttccataaggaggcagcgggacgagagaagaaaatgtgggtcccggatttggga

accacgcaaagaaggctaaggccatcacccggcccatgccgctttgccacctccacaccc

gatgggaggcgattacatagcagctgccaaacaaagattttgatcttcatagacagcgga

ggttttcacaagtgggaggtccacacaagagtctggcgcacaatagagggcacggtatgc

caacccggcagagaagagcgccgaggaggtgatgtgcgaagacacgacgccaggggacct

caacagcgatgacagaagagccgtccacaacttggtctaggctaaggtttcctttggccg

aatgttcgttggaacgggatatcccaattcccattatgggctgccgaggctaccaagagc

accggatccgagcaaatcacgaaatggcccgggaattcggcacgcaacgtggtcctaccg

agccaggggtcgagccaaaagagggcaccctctccatcgcctatggagaaactgattttt

tttttgaaaaggaggatacacccccggcctatgcatctgggagatgcatgcagccactat

attaattattcacaaagaccttacaaaataatacatcagtaagcctgaagccaccatctt

ggcaacacctgtctctactcctatgcccttgatgaaccgaatatccgagccgaataccaa

acagacatcgcaacaaagcctaacatctaaagccggatgccccagcccagccacatattg

ggactgtgtcacacaccggtccggcgcactctcagaagccgccgccaccgtcttccactg

gtcaatctccagagcaggtactgacacaccgaccttgtcaggcctgccatcgacgccaga

cagcgccaccatcctgcacgtatccatccggccgcgcccgtcgccgaaactccgcagcgc

catgccgctgggatccatcgtcagccatgcctatggagaaactgacacccaagtgaatct

catgcttgatcgactggagggattttcagaactgagatccctcccaacggtcacaagcca

ggagaggatggccctgcaggtgtttggctctttataagctgcagccataggccaccctca

cctcagcgtgagtaccacatttaaactgctcccacgggcagcagaagtgtgctctccacg

ATGGAGCACATCCAAACCGGAAAAGAGAGAAGAAAAAAAAAGGAG

gtgtgctcctgcacgagccacaggtatacctggccatacctcgggccgggctgggcctag

ccaagcccgacgcaaaaaacccaggcccgggcctggcccggcccggccatcgggcctgtt

ttttgggcctgagcccggcccaaacacgtaaaagcccgtcgggctccgggccggcccggc

ccgaccttcagagaaatcaaaaataagtggcatggttctaaacccacgacacttattttg

gaaccgagggagtacatatttacaaattactcccagcttcgtctggccggtaccatttcc

caacaggaacgggacacataattgccatttgagacatttaaatgctataaaattaagcat

agcatccagtgtcgaatagtggggaaaggctgcgtacaatagacccaaagtggtcggacc

cttccccagaccctgcgcaagcaagagctacatgcaccgggctgcctttttttgaagttg

tgaaccttacattcattgatctatatgtagtaaaagataaagtactttctgaaaaaagaa

tgtaggcctcttcatctaagagtataaccttttgataccgaaatgctgtttcctgactac

aggcaaaccatgtacttactttgatgttgggaccctgcgcaagcaggagctacatgcact

gggctgcccttttttgaagttatgaaccttatgttcattgatgtatgtgtagtaaaagat

caagtactttctgaaaaaagaatgtaggcctcttcatctaagagtataaccttttgatac

gaaatgctgtttcctgactacaggcaaaccatgtactcacttgatattgaatatgtgatc

tgcctttgatacccttagttttgaaggaagggtatttcttttccgtatattagtactcaa

aggtaagttccttgcttgttgcttgaatcataggcagatgtctgagtgcatgtttactct

atacattagccagagatatatagtttggtagcattagttaagtcaactactctactcatc

tacacgacttacggccattgttatgtttatcgcag

ATGCTTGCTGTACATTACTACCTGATCTTTGACGGTGGTGATGACCAATGGCGTCACATG

AAGTCATTCTATGGACAAGATTCTACTTTCAG

gtagaggaatatcttgggctacgtttctctcgtagtaacaaatcccttactgtattttcc

agttgtgcaaaataccttagtttccactgcttggatcgcatgtctttattatgcttggct

ccggaactataattgaactttctttcctgttaattgtacatgaactgtggagacttattg

acatgatatttttattctag

TTTCTCAGGGGAAACACATGTCGGTAAGAGCGACATATTCGATACTTTGTCAAGAATAAG

GGAAATGCTAAAAGGTAACAGCCGTTGCGAAAAGATTGGCCGTGTGATGGAGAAGGTTTA

CTGCCGTGCACATGGGGAAGAAGGGATGAGTGTTCGAGTTTCTGGATCATTCATTATGGG

GAACGAGTTCCTCGTGTGCGCAGATGGGTTTTGTGCTGAAGGGATGCTAAGCATCGTCGA

ACTCTCTCCCGACATTCTCAGCAAGCAGGCAGGCCATTTCCAGGAAGATTTTTTCATGGA

GCCAGGGACTGCCATGGGATGCTATGTGATATCAAGGCAAGAATTGCACATTGGCGTATC

ATGA

agtcattcgccttcttgcatgcggtcattacatatgatgatttttgtgct..........

**>AetMKK3-2 : EMT25805.X**

ATGGCGGGGCTAGAGGAGTTGAAGAAGAAGCTGCAGCCCTTGCTGTTCGACGACTCGGAC

AAGGGTGGCGTCAGCACCCGGGTTCCCTTCCCGGAGGACACATGCGATTCCTATGTG

gtaaaacaaaacacaccctgtcattctttcttctctgcatgcaaatcagattaggttgtc

tgtgtcaaacaccagcggcattagacccgggttgttgtctgcaaatggaattggtaccag

ttgtttcacttctgctttccagtcatgaaaaccatcctgccagcaactgaatatacatgc

tcaatggttctgtacctttggggtagcactttcgctgtatgggcctagacatctgcacaa

cttgaccaatcaatcctggcacatggaagtgataggacaaagattattgtttgtgatcat

gagattactctttgaaagttttcaattagaatttatctgagctttgtttttaatctttgt

gacttaaacttgtttagctgtggaagtgtttgttttttatgttctttctttcatgaag

GTGTCTGATGGTGGAACAATAAATTTACTGAGTAGATCGTTTGGTGAGTATAACATCAAT

GAGCATGGCTTTCATAAGCGAAGTACTGGGCCAGAAGAGCCAGATACCGGTGAGAAGGCA

TACCGATGTGCATCTGAAGACATGCATATATTTGGTCCCATTGGAAGCGGAGCAAGCAGC

GTTGTTCAGAGAGCTATTTTCATACCAGTTCATCGAATTCTGGCCTTGAAGAAGATAAAC

ATATTTGAGAAG

gtacattctttacggcccgaaatgcagtttacaaccacattgttgtgcattgctagtttc

catgaacatctcgacaagggtgttttcgcttttaatttcctaataattgttcattctgct

tcattggactgaacttataaagaagatcctgaaaacttgacttaatatttgtacccacat

tcaagtggaacttgtgttgatatctagtcgaattctcattcgtgttttgaaacaaaatag

tattaaagggagcaaaacaaacccttgttcttctatgcgcggttcacttgtatgctggag

catccttccctttcttttggagtcatgtgcatcacaatgggcccatgatatgcagcacat

gggtgggtaacatcaatcacattcccctgaaggatttggttactagtgataagctgtcaa

tatctctattgcctattcacaatgtttcttatttagaagggaagaacgtgtgttggatat

gtaaagtgaagcaatgtgaaactgttactatgtatacattgtttcgttgggattacaaat

agtactattcagacagtaacttcttatcagacccagtctaggtagttgcaccgaacttgc

agaaacgtactcttactcatctaaacttgcctagctagttaggacatgcccgactaatgg

aaagacgacattaactcctcatccttagctacctgcattgtttcctgacacatctgcaat

agtgatttccgaatgggcccatgacctgcacaaggaagggtggcaaaaaatacccaggac

ggtggatttcggtttttagaatttcaagccgcctatttaccgatttctcatccacagtgc

tgtctagttttacatttccatctactagagatggtatggctcatttcatgatatttgatt

aagcaaagaaagagaacaacaagtttatctcatctctagtctcctctgacaccaaagcaa

gctctctccaccatgccatgctctcgacacattggccccataatttagtagcatgcatca

caatatgacatgttcatttccttttaaccactctcaaggcgaccaaatatcccactacgc

aattttctgtatgtaagctggagtgaaacaagttctagttgagtatattaaaccaaaaca

tgtatttgcactgcattgaaggcagcacaaagggattctggtttatttgcagtggcagtc

aagtagcagagtcagttctgactgcagaatgaaagtagttacaccaaagagtagaaatgc

tcactcttctatgcttcaattcatatactattgcggcacaacatgcttggcattgcgcga

agtgattttggtactttatgtttgtctatatattttgctatacttaatccattctgtttc

agttgataaactgatttacaaaattttcttttcatattttcacgtgtcttcataccatta

cag

GAGAAAAGGCAACAAATTCTTAATGAGATGAGAACGTTATGTGAAGCAAGTTGCTATCCT

GGTTTAGTTGAATTCCAGGGTGCATTTTACATGCCCGATTCTGGACAAATAAGCATTGCC

CTTGAATACATGGATGGTGGCTCTTTAGCAGACGTTATAAGAGTCAAGAAATCAATATCA

GAGCCAGTTCTTGCGCATATGCTACTGAAA

gtattactt

gtatggatcttccacacacgctcttcctttcatgtccgcgttaatcaacat

taagctgtgtcctgatgtatttttgaagttataatttattctttgtacaattctgaag

GGCCTGAAGTACTTGCATGAAGTAAGACATCTAGTGCATAGAGATCTAAAGCCAGCGAAT

ATACTGGTAAACCTCAAGGGAGAGGCAAAAATTACAGACTTTGGTGTAAGTGCTGGTTTG

GACAATACAATGGCTATG

gtatgctgcttggtgacttttgatattggttaatctgagctaattattgtataatagtat

tgcaatgccttcaaatcatttagcgtgaatgcgttacatattttctgctaaataaatatg

tttcag

TGTGCTACCTTTGTAGGAACCGTGACATATATGTCACCTGAGAGAATTCGTAATGAGAAC

TACTCTTATGCTGCTGATATTTGGAGTCTTGGACTAACGATATTGGAGTGTGCTACTGGT

AAATTTCCATATAATGTCAATGAAGGCCCAGCCAATCTCATGCTGCAG

gtgattggctactcagatttttcagttcttgtctgttttcagtcattatgtatggtggga

tgcaatatcatcccttttagtctgatgcaaaatgcgtgaactatttgagcgattcacatg

gtagtttgtatttggacttgttgagatggcaaaaaatgttatatttctgcaagtctgtcc

gctggttatgctagtttctgcaacctttttctttttcctggctgctttaacttttgtcag

ttgtatttttatttgaatatataaattagctgacaatgaaataaatatgtaccgttacat

atattggtcttatgaatgttctcatcatcatttgtctgttggaatgttgttttctgtcac

ttcatatgttgccctatcttaatcttaaaatctcatacttcagatactgttttctttcac

tcaattgctgttctctcttaatcataaactctcttatttcag

ATACTCGATGATCCATCACCAGCACCACCAGAAGATGCCTATACACCAGAATTTTGTTCC

TTCATAAAAGATTGCTTGTGGAAAGATGCTGATGCAAGGCCTACATGCGAGCAG

gtaaagaaaacatgtatatgctaacttccatgtaatctgagtccagtaggttagcaattt

gcatcaatctattgtggtctatttgaatgttattacatattactggtaataacttttctt

gatttgccttctcttgtaattatgtcccactggcctttggttgatgaggagctgaggaca

tcggtgcccctatcattggtccattggaatgaatggagaccttggtttctcttctcttac

tcacaccatcatgtccatacttccataccctagcagcccagagtcgcgggcgctgctaca

gccaggccaccaccgccaccaatgttcactgtccaccatagacctcttatactcggcaac

gtgcccagaatgactctgcgccctctcctctgctccag

ATGTGCCTCTGCTGTAGTGCCTGCCGTGCCACTGTCCTTGCAGCCGTCATGCCACTGTCC

TTGCTGCTGCTGCTTCTGGTGGCCATCAATGACCTCAAGCTCAGATCTGGTCACTTTTGG

TGCTTGGGCACCGTGGAG

gtatgttcagctagggatgggaaaccttaggcacagggagaaggaaatgaggtgatggag

caagtagaagatgggttactccctgataattgatttaacgtgtggctatctcattataat

gagggccttacatgtgctggactctacaacatgaactggacttggcaacatgtctgttat

aataggaacactacttgaattctgatcaccagtttctctgtgcctactgtggtttccttg

cttccttaatgatcacatgtccgttgtagtaggattgcagtttcccttttgtatgcttag

gctgagttgtgagtcctgtcatccttgatccagtgaagcacattaggcaatgcatcggct

gttgtagatccagtgaagcatgatctttccaagcacattggagttgatgcttcctgcatg

aaattgcag

ATGCAAGAGCAGGTTGTTGCTCTCCA

gtatgtgccatcagaactctagttagctgatttcttcaccaaggcacattctagagcttt

gtttgttgatctgcctttaatttttgtgtag

GGTGTTATTGGAGGTCCATTTCACAAACTTTTATAAGCAAAAT

gtacgtgctgattagttatggcttggtctagtaacagaatgttaattatcttgacgatgc

attttacatcttctatccttttccattgtttggttttcttatagttgattaacatgttta

atgtaatgcag

CTTTTGTCACACCCATTCATCAAGAGGTATGAGCAAACTGGTGTGGACTTGGCAGCATAT

GTCAGGGGTGTTGTTAACCCAACAGAAAGATTGAAGCAAATAGCGGAG

gtgaattctgtagagcatatttcagcttactgttctttctacttcagttaactataaggg

cgcacacatgatatgaaacatgcgccctatatcaagaactgatgctagaccttagaagtg

gggctgggagggagagctggggtagaggctagatggaggaggtatagttaagcactcgct

tgatggagttactgccagcttgccctctttggcctctgccgcctcaccgctggtggtcac

cactcaccaggggtgccattcctatttgttctgtccgcctttgcttgggtacaaaacaga

ggtagggagagagacatatttttttgtctttcaattttagtttagttcattgtttctttt

cccaatctataatatagctagtatacaataggtcatcgacctggagtctggggctgcgtt

gttgtgcgctatggttccaaagaacactaccccatcctctatgaaccctacttgttgagc

caccttaaccgtgtctggctgatcgattttgttgataactatgattgtacattagaaaat

aggaagaacaaagtggcacaagtgtgatccatttgaacgaatgcactgcaggattcctat

acttccattgaacgagtaaaatgttacactagtgctagtcatgtatagaatactggcctg

ataggtcagttcagattattccaaaacttgataatttctttgtttgctgctcagagaaac

cctttggttttctttttgagcggaatccgtcaaaaaataaatactaatatataagtcgag

agtgttcaagcccctctatccccatactctctttaactttactatttatcctctcctttt

ccgtttccactcgtctgttgattcttcaacattagctggcccggttccacaattcagatc

tcaattcaaactcaagaacgaatcatatgtatcttcctagattgatagaactcctgctat

tagacccaaacgcacccaacggatacttactatttgatttattccttttcttaagactcc

tcaccatctctaacataacttatccaaggccttcacggatatgcttgtctcctgttgtct

tgtggacccacacacatgataataatatccaaacttttgaagtgaaaactaatcaattag

ctccttttacgttttagaaaatctcattaaaatgtgaagaaaaaactgacgatcctgtgt

gtgtagtactaagcagattctttttcccactcatcccag

ATGCTTGCTGTTCATTATTACCTCCTGTTTAATGGCTCTGAAGGACCTTGGAATCATATG

AAGACATTCTACAGGGAAGAATCATCTTTCAG

gtacctgaggcaacgttacatgttttaacatgatcataaaactttagtcgtaccacagca

catagcatcctactgctttttaggcatttaacctttatgtaaattatggctccagtattc

ttaaaattagtttggtactaataatatcaagaatgtgttgtgtgatcctcaatatgaaaa

tttgctaaaactagcatggagttgccaacaagttgatgtatctagttgaattgacagttg

cactttatcctataggcagtgttgtgcttctcttgttctacttctaccaggtagtaacta

gctggttactgccatttttccaaatttatcctgtcaagcattgttgcttctgttgtgcct

ctatactatagttgtgtgtggaagaaaccgagctgtttctgaacacatgcttgacatgtg

gttcctgtttttgcag

TTTTTCAGGGAATGTGTATGTCGGACAAAGTGCCATATTTGATACTTTATCAAATATAAG

GAAGAAGTTAAAAGGTGATCGGCCTAGAGAAAAAATTGTTCATGTTGTTGAGAAGCTGCA

TTGTCGTGCGAATGGGGAAACAGAAATCGCTATCCGTGTGTCTGGATCATTCATCACGGG

CAACCAATTCCTAATATTTGGTGAAGGGTTGCAAGCTGAAGGGATGCCCAGCCTGGACGA

AATCAATATCGACATTCCAAGCAAGCGGGTAGGCCAGTTCCGGGAGCAGTTTACCGTGCT

TCCAGGGACTTCCATGGGATGCTACTACATAGCAAAGCAAGACCTCTACATCGTCCAGTC

CTGA

**>AetMKK4 : EMT05580.X**

gatcgatcccacnnnnnnnnnnnnnnnnnnnnnnnnnnnnnnnnnnnnnnnnnnnnnnnn

nnnnnnnnnnnnnnnnnnnnnnnnnnnnnnnnnnnnnnnnnnnnnnnnnnnnnnnnnnnn

nnnnnnnnnnnnnnnnnnnnnnnnnnnnnnnnnnnnnnnnnnnnnnnnnnnnnnnnnnnn

nnnnnnnnnnnnnnnnnnnnnnnnnnnnnnnnnnnnnnnnnnnnnnnnnnnnnnnnnnnn

nnnnnnnnnnnnnnnnnnnnnnnnnnnnnnnnnnnnnnnnnnnnnnnnnnnnnnnnnnnn

nnnnnnnnnnnnnnnnnnnnnnnnnnnnnnnnnnnnnnnnnnnnnnnnnnnnnnnnnnnn

nnnnnnnnnnnnnnnnnnnnnnnnnnnnnnnnnnnnnnnnnnnnnnnnnnnnnnnnnnnn

nnnnnnnnnnnnnnnnnnnnnnnnnnnnnnnnnnnnnnnnnnnnnnnnnnnnnnnnnnnn

nnnnnnnnnnnnnnnnnnnnnnnnnnnnnnnnnnnnnnnnnnnnnnnnnnnnnnnnnnnn

nnnnnnnnnnnnnnnnnnnnnnnnnnnnnnnnnnnnnnnnnnnnnnnnnnnnnnnnnnnn

nnnnnnnnnnnnnnnnnnnnnnnnnnnnnnnnnnnnnnnnnnnnnnnnnnnnnnnnnnnn

nnnnnnnnnnnnnnnnnntcgttcccccccgcgggggcgcggcgggttctgtcgcggatc

gcctacctgcaccggcgccacatcgtgcaccgcgacatcaagccgtccaacctgctcatc

gactgcgcgcggcgcgtgaagatcgccgacttcggggtggggcgcatcctgaaccagacc

ATGGACCCCTGCAACTCCTCCGTGGGCACCATCGCGTACATGAGCCCCGAGCGCATCAAC

ACCGACCTCAACGACGGCAACTACAACGGCTACGCCGGCGACATCTGGAGCTTCGGCCTC

AGCATCCTCGAGTTCTACCTGGGCCGCTTCCCGCTCGGGGAGAACCTGGGGAAGCAGGGC

GACTGGGCGGCGCTCAT

gtgcgccatctgctactccgagtcgcnnnnnnnnnnnnnnnnnnnnnnnnnnnnnnnnnn

nnnnnnnnnnnnnnnnnnnnnnnnnnnnnnnnnnnnnnnnnnnnnnnnnnnnnnnnnnnn

nnnnnnnnnnnnnnnnnnnnnnnnnnnnnnnnnnnnnnnnnnnnnnnnnnnnnnnnnnnn

nnnnnnnnnnnnnnnnnnnnnnnnnnnnnnnnnnnnnnnnnnnnnnnnnnnnnnnnnnnn

nnnnnnnnnnnnnnnnnnnnnnnnnnnnnnnnnnnnnnnnnnnnnnnnnnnnnnnnnnnn

nnnnnnnnnnnnnnnnnnnnnnnnnnnnnnnnnnnnnnnnnnnnnnnnnnnnnnnnnnnn

nnnnnnnnnnnnnnnnnnnnnnnnnnnnnnnnnnnnnnnnnnnnnnnnnnnnnnnnnnnn

nnnnnnnnnnnnnnnnnnnnnnnnnnnnnnnnnnnnnnnnnnnnnnnnnnnnnnnnnnnn

nnnnnnnnnnnnnnnnnnnnnnnnnnnnnnnnnnnnnnnnnnnnnnnnnnnnnnnnnnnn

nnnnnnnnnnnnnnnnnnnnnnnnnnnnnnnnnnnnnnnnnnnnnnnnnnnnnnnnnnnn

nnnnnnnnnnnnnnnnnnnnnnnnnnnnnnnnnnnnnnnnnnnnnnnnnnnnnnnnnnnn

nncggagcttcatcagctgctgcctccagaagaacccggcgaagcggccgtcggcggcgc

agctgctgcagcaccggttcatcgcctcgccgccgccgccgcagcagccgcaggccctcg

ccgccccgccgtgctgacggcccgcccgccgtaaccaagttaaattcggagggatcatgg

acggacggacggacggtggccggcggcgagacgtgggatctaggaggaggggacatcgat

ctttcttccttctagccattttgggtagtgaagccagcaccacaaatttacccatccatc

cctctgttgttgctgtcgctcctgaaattaagctcaagcttttctttagtttctctgtta

aatgttaattaaatgttgtctccttcaaatgatgattctgatgaggataatgaagatata

atatggtccgttaagaaaccaatttcttgttccaaattcgcaacatcgtcacagcaggcg

gtggtgatgtgctctgctctgctcctctcggtagagaagaagaggatgatgggatgacga

catgagaagcttcttggatgccgaggcggattcggccggatcaccaccatggatataaaa

atggaacgaattttgaagcggggggaccagaacaactaactttaatcggagccctctctt

gcttgttcctctggtgttcttggtgaatccgaatcgcatcgggcatccgctctctttatt

cccgtgaaaaagatgggtgctcctacactagtagcaatggccatccatgtagagagagta

gccgcaatcatgcaaggcacatcagagcagcagcaataatgggcgcatccatccacaaat

attccggcaccgttgcttccttttcctccctcgcctgtcagttgcgttgcgccctctcct

ctcttcccttttgtttcgggccctacctacacggtggttttggatgcttggattctggtg

gtcatttttgggggttatgctcggcatcccccgtgctaattcgccatgggagtaaaggga

agcgtacgagagtgagcacgagagagggacccgtggttggaatggaatggacatggatag

atttgtaggccttggaaaggggcggcacgcacaaaaggagcggcgaggcgagcgctccat

taatggaggcagcccatgtggcgtggcgtggagctttctctcgtgcttgttcgtacgtac

tcgttgtacgtacgtgtcccattaactctgatgatggctggcaggtaggcccggccggga

atatgcaagtaaatagtgtgcttcatcctgcagtaagtggagacacgcttcttctcgtgc

catgccatgcattgtgcag

GCGGCACGAAGATTCCTCGCTCCTGCAATTTAATGGCGTGTTCCACCATTGGCAGCTTGG

TGGATTCTTCTGA

**>AetMKK5 : EMT03871.X**

ATGGCGCTCGCTGTAGTTATTAGAAACGTGAGCTTTTTTCCCTTCTGGACGGAATGGAAC

gtgagctaatctggtgcgagagacagtgacgcgcacacattttccccgctttttctccga

gcccaagaagcagcggcggcccttccgttttcccggccgacgcgacgcgcgcagcacgct

tccggcttctcgtcctctccccctcccatcctcggagtcagtacaggtcgcctcccccaa

caaacccgatcccccatcaaacagacggcgcgcaaccagacaaatccccgttagccggca

ccagcagccgcgccactgcccacccccacccccacccccacccnnnnnnnnnnnnnnnnn

nnnnnnnnnnnnnnnnnnnnnnnnnnnnnnnnnnnnnnnnnnnnnnnnnnnnnnnnnnnn

nnnnnnnnnnnnnnnnnnnnnnnnnnnnnnnnnnnnnnnnnnnnnnnnnnnnnnnnnnnn

nnnnnnnnnnnnnnnnnnnnnnnnnnnnnnnnnnnnnnnnnnnnnnnnnnnnnnnnnnnn

nnnnnnnnnnnnnnnnnnnnnnnnnnnnnnnnnnnnnnnnnnnnnnnnnnnnnnnnnnnn

nnnnnnnnnnnnnnnnnnnnnnnnnnnnnnnnnnnnnnnnnnnnnnnnnnnnnnnnnnnn

nnnnnnnnnnnnnnnnnnnnnnnnnnnnnnnnnnnnnnnncctgctgcgctacacgcacc

gcgcccccccagccccccgccgcatcgccgacgagcccttcctcgcccacgtggcccggc

ag

GTGCTCTCGGGCATCGCGTACCTCCACCGGCGCCACATCGTGCACCGCGACATCAAGCCC

TCCAACCTGCTCATCGACTCGTCGCGCCGCGTCAAGATCGCCGACTTCGGGGTGGGGCGG

ATCCTGAACCAGACCATGGACCCCTGCAACTCCTCCGTCGGCACCATCGCCTACATGAGC

CCCGAGCGCATCAACACCGACATCAACGACGGCGCCTACGACGGCTACGCCGGCGACATC

TGGAGCTTCGGCCTCAGCATCCTCGAGTTCTACCTCGGCAGGTTCCCCTTCGGCGAGAAC

CTCGGCCGGCAGGGCGACTGGGCCGCGCTCATGGTCGCCATCTGCTACTCGGACCCGCCC

GAGCCGTCGCCCGCCACCGCCTCGCCCGAGTTCCGGGGATTCATCGCCTGCTGCCTGCAG

AAGAACCCGGCCAACCGCCTCTCCGCCGCGCAGCTGCTGCAGCACCCCTTCGTCGCCTTG

CCGCAGCCGCAGCCGCTCGCCGCCCCGCCGTCATCATGA

**>AetMKK6 : EMT19002.X**

ATGAGGGGCAAGAAGCCGCTCAAGGAGCTCACGCTCTCCGTGCCGGCGCAGGAGACGCCC

GTCGATAAGTTCCT

gtatgtctcccccattccttggcctaaatttcatgtttcccccctctctttttcccccac

taaagctatcacaggtttgcgctttatgtctaataaatgagagattttttgggtgcctgg

gtagatttttgtggtttatttgtggattttgatggggctgggtagccattactgggttgc

tgaagcatagatttactttgaaggcattaagtgtagtgcttgatttgaaattattacggt

ctgagctgctggctgactcagcagtctgtatgtcaacgaaagtatttaagtccaatgatt

agtttgaactcaatacgtgttgagttgctgagctgcctgtggtatttcag

GACTGCGAGCGGTACATTCAAGGATGGTGAACTGCGGCTTAATCAAAGAGGTTTGCAGCT

TATCTCCGAGGAAAATGGAGATGAACAT

gtgagttcccggttcaatagttttaaatgactacttttgttttctctcaatctgtccaat

tgtgtttgtgttgtattgtacttcgatcgttcatgtttgatgcgtgggcgcgcatgcgtg

ttgcactgttgcgtgcttcagagtcagtgcactcagagggtctactgcatttgatcaacc

aggtggaaccaacaaaacatccgataagatttgagattgaatcgtatatacgccactata

aaaaaaagcatacatgtcgccacatgaagtgcaatacaacacaaacactaaattaccgaa

cgaggaacttacatattcatctctgttttcctcagagatgccacaaacctctttgattaa

gtctaaagacctccagcaatcaaagacaaccatttaatttgcacattacttcgtgttctg

aagaaacaagaagtgtctcaaaaacatttctagacacaaaaaagtagtgcagtaaaagat

caagccttcaatagcttcaacaggaaaagggagaaggaaaaggaaaacatattatataaa

atgggcagtttaggccaatgaatcaaggaaacctacatgagtttattgagaggcgtctcc

tacgccggcagaacgagctggagttccttgagcggcctcttccccctcatggttgtaacg

ccccggccacaaccatcggttcctggccgttatgcctgacgggatctagactagccccat

agaccaacactagtctttcctgcgcactttgtcctcactcgtgcgcacctcagaaggact

tctcggtcagtcacccatcctcaaattaccccatgcgaagcaacctggaggttctttgga

gatgagcttctgaaaaagaaggcgcaccttgttgaaatgagtagtctatcattcctatta

agccaggatgtcacaatggtatcttcctatggttagtgtttcttcgctaggagatgaata

tggaactggccaaagccttaaactagttggggagtaggtaggagaccaggtgaactgcta

tggagatatggagtttggatctgcccagcggaggcatgctgagagagcggagcaaccttg

aggaaggcctgggccacgatagggtagatggaagtgaagatggggcatgttgtactttca

tcgaccaaagttgctaatttatggaccgcgggggtcagaggaacccttacagtttaaact

tggtagccatttcatccataatcataacatatttgcaaaattttccattatgtttcatga

ttatcttgagtagatgttagtttttaatgcctaggattgaaattatgtactctgctgctc

gctattggttattagatgccaccaacagaaaccacaacaagaatatattgttcttcagtt

tgtgtttactggtatttggataatgatcaagttgtttccgaattctgtttgcatcttgat

ttgtttcttttcctcag

CAATCAACAAAAATGAAGGTGGAAGATGTGCAGTTATCAATGGATGACCTCGAAATGATT

CAGGTCATTGGTAAGGGAAGTGGTGGCGTCGTCCAATTAGTGCGGCACAAGTGGGTGGGC

ACATTTTATGCCTTGAAG

gtaacagtacactaaattcacctagtgggccgcacaaatgcctgtgatggatttttttgt

tgatgttagagcattggggtagactgcttgtttttttatcgtacctgtttatattttttc

atgtttgttaacatggaaattaatatgtttatctgtttgaacagatttgcattctatgca

ttgttagacgatctgattattctacatctgaagacttatttgttttgtttagtcactttg

tgatggcatggcaatatattcaatttgatccgcatttatgcacaccattaaaatgagttg

tagcaaaaacatctagtactggctgatctttgcctgcaaaatctccatagaacaccaatt

ttaatggaaactgacactaatgtatgagaatacatcaggtaatgatatacagctgattaa

acattttcaagcattattcctggtctttgtactgctgtggcatttcgttgtccatatgat

ctatcaatcaattgattattttcaatttcttcatag

GGCATTCAAATGAACATTCAGGAGTCAGTACGCAAACAAATAGTACAGGAGCTCAAAATA

AATCAAGCAACACAGAGCCCCCATATCGTCCTTTGCCATCAATCTTTTTACCACAATGGT

GTAATATATCTTGTTCTGGAATATATGGATCGTGGATCTCTTGCAGACATAATTAAACAA

GTTAAAACCATTCTGGAGCCATACCTTGCAGTACTTTGCAAGCAG

gtatgtatacgtttggcctatggtttgactgcatccggaatattcaaattttgtcacatc

tgatggaagttgaagctagagtctggcacatctaacattaataatggcttaacaaatcag

GTTTTGGAGGGTCTGTTATATCTTCATCATGAAAGGCATGTGATTCACAGGGACATAAAG

CCATCTAACTTGTTAGTTAACCATAAAGGTGAAGTAAAGATTACTGATTTTGGGGTGAGT

GCAGTGCTAGCAAGTTCAATCGGTCAGCGTGATACATTTGTTGGAACCTACAACTATATG

GCG

gtatgtaaaaagatctgctttgtcttctgactgcggcttatgctcataatggttatctgg

ggtgcacaaaatttgctgtgatagttttcttcctttccttcttgtgcag

CCTGAGCGGATTAGTGGCAGCTCCTATGACTACAAGAGTGATGTATGGAGTTTGGGCTTG

GTAATACTTGAATGCGCCATTGGCCGCTTCCCCTATACTCCTCCGGAAGGAGAAGGCTGG

CTAAGCTTCTATGAACTATTAGAAGCAATTGTTGACCAGCCACCACCTTCTGCACCAGCA

GATCAGTTCTCTCCAGAATTCTGCTCATTTATCTCTTCCTG

gtacgctaaacattattccgaggaactacacatgaaattagctcagtcactgcatttctg

ttttgtcttcttgcttag

TATGTTTGAATGA

acttcttgccctgttttctaaagttctgattttcttttccgtgtctttcacgtaaaaata

ttttaaattgtttgtaacgctaacttcttttaatatttctcaaaaaaaaaacttctttta

atagttatctgctttctcgtccaaagctcatggactttgtccactcaagtattattcccc

catttatgaaaatgtgttttgtctattcaagtattactttcccctttgaacaagtgtggt

cttaatagaactgttttagtgtgataacttggacttcattaattctggaaataaatgttt

aaactgcgcggtgttcttcagattagaattagtgaattcctcagaagtccagattttata

atggtgttttttgtaacagcaaacggccgaatcaatgttcttattataaaaattacattt

cagtacagcccaagaaattccagccatcccaaaaggatcgacttgcgtctccagacaata

gctactcagggcaacttgccatgaaactatctgtttatcaaaccctgttcaagtagcttg

ttggcccttgaaacttcaagcagtttagctaatttgaagtttgagttatgaatcatgtaa

caaccctgattttcagtttaatgttaaaatgtacttgttgatttaagttagctatgttaa

tatctgatctaattgttgtgttgattgacaaaaccttgactgaggttgtagtataaacca

gttaacaatctggcatcttatcttactgatggattaaggcatgccaaaaaaacacggaaa

ataacgcgcctactatgcagggtgtatgaacctgaccaaatcatgtcgtgtgacccaaaa

ggaagaggaaggagtaccaaagaacaaataggcacaggaggcatcacaaaaactcaaacc

aaagactccgacgaggaacgagggctttagccttccatcacgtccccatgcccactgctc

ttaggtcttcttcctcttcatgtctcacaatggacatcttataccagctgaatatatcca

cataaatgttcagtttttccactaaaagatttggcaaacaaaaaggaagaggaaggagcg

cttaggaacgataggaatgagaggcactgggaaataccaaatggcatttccacctcaaca

caagatgtgaagaaccgataacttaggctttcctctatgttctcatacccattgctctta

ggccctctgtttcttcctcttcctagctcgcttttgagatttcacatcgaatatgtgcaa

acatatagttggtttgtaagctaaagcaaccaagcaagccaaaaaaggaagaggaaggag

tgctttagaacaataggaatgggtggcattgggaaacaccaaactaaattctcacctcca

acactagaggtgaaaccgagcaggttaggctttcctccatgttcccattcccactactct

aaggccctctattcttcctcttccgggctcgcctttgacgtctcatgttatctaaacatg

tggacacatgtctatggtttctccatcagagctatcacataagcgaaaaggaagaggaag

gagtgcacaagaacggtaggaatgggaggcatcaggaaaggatccatgccaaatatttgc

acctccaacacttgaggtgcaaacaaacatgcagggctttcctccatgttcccattccca

gtgctcttgagttctctctttcttcctcttcccggcttactttcagtgcctacaaatcta

atgaaacaaaaactctcaaagaacaagcttgaattgttggacaaagtgattcagctttga

ggtataaataatgggtggtgcaacatatgcagaaccttagagaagaaactaatgaagaca

cctgatgcatgtgggcaccattctacagtcaatgataatggggccacataatcaccggcc

ctgtcctattgtcctctactcttaatttagggcacatgtgtaaagatctcttctccttac

ctttattacccatatcagaaactggtacatgatatccataccagccttttttagaacaat

atccgcatcagcctgtgcatttatggtgtcaaaatatgtagcaataaggagttagagctg

aaagtaatatgaaattggacaaattaaaccctgtaagtaaacttggaaaaattacacaac

agtggggcaaagaaccactgaagtgacaactaattggaacataatgtacttcggaccgct

gcagtattagagatgacataatgtgatcaaaacatgataagagagatctcatatctgcaa

aaccaaaatatctataggctattttatcaagaaaaaatgtgtatagtcgtattgctcttg

aagtgtttagtatccaaattctgcaggctgtacacaactacatggcatgtgagaaatata

ggaaaaaacatatctccaaagaaaccatcaccctcgatccctagcagtggcggagccagc

ctatgaatgcagctcgggcaatacttaactgtgatagtgtgagctggcacctgccgaaag

aaataatccttctaaccatagattatcactagtgcttaggagtaattttctggactagcc

caggcagccgcccggggttgccgggagttggctctgtcactgatccctaggaggaaaaaa

cttgaagaataaggtcacatattccgtctaaactaaggtttacgattcgatgagtcgacg

agtcgtcctgaggttgagactcaatagactaagcgtgagtagtcgacactaaagttgcga

ctcatagactagtcgacactgaagtgtagtcggccctggagttgagactcatagactagt

cttgactagtccttggactcataatccatctaaactcactatacatagtattttaagaac

aaggttgggacacaaaggaagaataagatcccgaagagagtatggtacttcttccatcca

tcaaagtacttcccagttcaaccttgtagctcagccaccatcccccaaggattaaaacag

ctaggttgagatgcaaacagccagcaaggttcaagagtggggaaataagccttatcccac

acatctaaacaatgtataaaaagacaaccatttatgatcaagtgtacaattccattggag

tttacaatttttttgacgctgcagatatatcttgttgcagtcatggctgtttcttgctag

cctgcatatctatccattagtttgctatcccatgtgtgaactctaggaattgatggtaaa

aataatctgcaatgcacatatctttacatgttctgtgtgcaaattgccatatttcacact

ctgatttctgcttgtactcttagctacaacataaggactaaatcacatcaatgacacacc

acctattgttcccatgcccactgctcttgggccctctttcctttctcttcctgtctcacc

atgggcatctcatatcatctgaatatatgcatatgtatgttttttttctaaagcgattta

gcaaacaaaaaggaagaggaaggagcgcctaagaacgataggaatgagaggcatcgggaa

atgccaaactgaattgccacctcaaggcaagaggtgaagaaccattaagttaggctttcc

tctatgttctcattcccactgctcttacgccctctctttcttcctcttccttgctcgctt

ttgagatcccacatcgaatatatgcacacatatatttggtttctaagctaaagtaaccaa

gcaagccaaaaaaggaagaggaaggagtgcttgagaacaataggaatgggaggcatcggg

aaacaccaaagcaagttatcacctccacactagaggtgaaaacaaagcaagttaggcttt

cctccatgttcccattcccactgctcttatgccctcttttcttccacttccctgctcgcc

ttcaagctctcaagttatctaaatacgtggacacatgcctatcgtttctccatcaaagct

actgtgcaatccaaaaggaagaggaaggagtgcacaacaacggtaggaatgggaggcatt

gggaaaggatccatgccaaatatttgcacctccaacactagaggtgcaaacagaaaagca

gggctttcctccatgttcccattcccagtgctcttgagctctctctttcttcctcttcct

ggcttactttcagtgcctagaaatttagcgaaacaaaggtctcgaaggacaagtttgaat

tgttggaccaagtgtttcagcttggaggtataaataatgggtggtgcaccatatgcagaa

ccttagaggagaaagcaatgaagacacccggtgcatgggggcaccattctacagtcaatg

agaatggggccacataatcactggctctgtcctattgtcctctactcttaatttatgaca

tgtgcgtaaagatctcttctgcttacctttattaaccatatccgaaactggtacataata

tccgtaccagcctttttttagaacgatatctgtatcagcctgtgcatttagtgtcaaaac

acgtagcaataagaagttatgagctaaaaataatatgaatttggataaattaaaccctct

aagtaaacttcgaaaaattacaaaactgtgggcaaagaaccaccaaagtgacaattaatt

ggaacacatggtactttggacatgagctgcattatttcagatgacagaatgtgattacaa

catgatgagagggatctcatatctgcggaaccaaaatgtctataggccattttatccaga

aaaaatgtgtatagtcttaagtaatttagtatccaaattctgcagctgtacacaactaca

tggcacgagagaaatataggaatatatctccaaagtaaccatcaccctcaattcctagag

aacacatgaaggtatagtgatcagtgatcacatagtcagtctaaactcattatacacata

gtatttgaagaacaaggttcacatacaaaggaagaacaagatcccgaagagagtatggta

gctcgtccacccatcgaagtactttccagttcaaccttgtagctcggccaccatccatcc

aataatttaatcacccaggttgagatgcaaacaaccagaaggttccagagtggggaaata

agccttatcccgcacatctaaacaacatataaaaaacaacaccattttatgatcaagggt

acaaataccattggagttcacaattttcaaagctgcagatatatctttgttgcagtcatg

gttgtttcttgccatcctgcgtatttatccattagttttgctatcccatatgtgaactct

ttaggaactgatgataaaaataatctgaatgcacagattgttacatgttttgtgtgccaa

ttaccatttttcgcgctctgatttctgttcgtactcttagctacagtggacagagtggtg

tgtcatcgatgtgattaactaaaacagtaagcactaaaacgcatttaccatgcgactgtc

taaactccacagttctcacatcattcttgacctgcagcaattggctcatatctaccattc

aaatactatttttttcgagcaaccggcaagaactcctcagtcctcacatcattcttgacc

tgcagcaatttagcaattggctcatatctcatatcatctgaacatatgcatatgtatgtt

ttttcttcctaaagtgatttagcaaacaataaggaagaggaaggagcgcctaagaacgat

aggaatgagaggcatcgggaaatgcccaactgaattgccacctcaagacaagaggtgaag

aaccagcaatttaggctttcctctatgttctcattcccactgctcttatgccctctcttt

cttcctcttcctagctcgcttttgagatcccacatcgaatatgtgcacacatatatttgg

tttctaagctgaagtaaccaagcaagccaaaaaaggaagaggaaggagtgcttgagaaca

ataggaatgggaggcatcgggaaacaccaaagcaagttatcacctccacactagaggtga

aaacaaagcaagttaggctttcctccatgttcccattcccactgctcttatgccctcttt

tcttccacttccctgctcgccttcaagctctcaagttatctaaatacgtggacacatgcc

tatcgtttctccatcaaagctactgtgcaatccaaaaggaagaggaaggagtgcacaaca

acggtaggaatgggaggcatcaggaaaggacccatgcctaatatttgcacctccaacact

cgaggtgcaaacaaacaagcagggctttcctccatgttcccattcccagtgctcttgagc

tctctcattcttcctcttcctggcttactttcagtgcctacaaatctagagaagcaaaag

ctctcggaaggacaagcttgaattgttggacaaagtgtttcagctttgaggtataaataa

tgggtggtgcgccatatgcagaaccttagagggcaaagcaatgaagacacccggtgcatg

agggcaccattttacagtgagtgataatggggccacataatcaccggcggtgtcctgttg

tcctatactcttaatttagggcatgtgcgtaaagatctcttctggttacctttattacct

atatccgaaactggtacataatatctgtaccagccttttttctagaacaatatctgtgtc

ggcctgtgcatttatggtgtcaaaatatgtagcaataagaagttatgagctaaaagaata

tgaaattggataaatcaaaccctgtaagtaaacttggaaaaaatacaaaacagtgggggc

aaagaaccaccaaagtgacaagtaattggaacacaaggtactttgggcataagctgcatt

attatagatgacaaaatgggattacaacatgataagagagatctcatatctgcaaaccaa

aatgtctatagtccattttattaagaaaagtgtgtatagtcctattgttttttaagtaat

tgtagtatccaaattctgcaggctgtacacaactacatggcatgagaaattatagggaaa

aacatatctccaaagaaaacatcaaacacatgatggtatcacatattcagtctaaactca

ttaatacgcacagtattttaagaacatggttcggacacaacggaagaacatgatcccgaa

gagagtatggtagttcttccacccatcaaagtacttcccagttcaaccgtagctcggcca

ccatccctccaagattaaatcacccaggttgggatgcaaatagccagcagggttccagag

tggggaaataagccttatcccgcacatctaaacaaatatgtgaaaaacaacaccaattta

tgatcaagtgtacaaattccattgaagttgacaattctcgaagttgcagatatatctcgt

tgcagtcatggttgtttcttgctagcctgcgtatttatccattagtttgctatcccatgt

gtgaactctttaggaactgatgataaaaacaatctgaacgcacatattattacatgtttt

gtttgtcaattaccatttttcgcactccgatttctgctggtagtcttagctatatggtgg

tcagagtggtgtgtcattgatgtgattaactaatacagtaatacacttacatgcatttac

catgtgactctgtctaaactcctcagtcctcacatcatgcttgacctgcagcgattggct

caatctaccattcaaattctactttttttttcgagcaactggcaagagctctgccttttc

acatagaagagaattgaccaatttataaggaaacccggccgaaaacagaccattcaaata

ctatttagtttagaacttacagtcaactccgtttattggatatatgtgttattggtctat

attttagttagtgattattcttaatgaaatttgcacatcttgaaggcaacggatggttcc

tgctagctttgctttatagatttgtgccgtctgtgtagtatatacaagcaggagcatatc

atattgcagtgttatctcatattactatattgctttacagcatacagaaggaccccgccg

aacggatgtctgcttcagaactcttggtgagcattcccttccactttacaagttgttgta

gcgtgttcaactaaccgtggtaccgttgattctgtcgactagtgtgtgtgctgaatcgtc

gttcgatgttgttgtcgcagaaccatgccttcatcaagaagttcgaggacaaggacctag

acctgcggatcctcgtcgagagcctcgagcagcccatgaacgtgcccgagtgaggccgtc

gctcggagaggtgaggccttgcgacgagtttcttccagggctgccctcatgacggcaatt

gtgtattgctgtgaacctgatgaatgagtgaccgaccccccacccctgtatgtgatgatg

tttatgttggaaaaattcctagaggattggaatggattattaatgtatggtaatgacttg

aggcccttttgttgcgcgtcttgttatgaatgaatctggtgtggttagtaatttgtggtg

catgagagtggaacgaaccgtgatgttgcattgtcatgcgtctgtgtcgcggtccggttt

atgtggttcgtactgggagtgcaaccttggttgtgccctgggaatgaccctatgttttgc

accctaactgttgccatagttgctaggcagtaataaaaaaaaatcaagctgcctgggaag

tctatgatcaggcagttcagtttttgttttgcactggagcaacgaagggtccgcacaaca

aaggaatcactagaagcaacagtccagtagtgtatgttgtgctgttgggatagcaagtgc

aattagtcatatcgtgatgtcaagaactagaatccttgcttcttattatgacagtccttg

actgaatatttttcttatagttatgttttgccgttgtttcatatcatgatgtcgaaggaa

ggacccgaatatgtaccgaaggtcagttttttagacatggcgcatcaaatcacgtttttt

cacggcaaactatgtccgttgaaggatgcaagttttcttgcaaatactagaaaaaaaaag

gttggaagtttagtgggcgagcatggcaaatccggttagctcgtgtatttttgccaggga

attgctgtgttctgtaaactaaataaatttgccatcattgcggtaatataaatagacatg

ggaaacgttggtatcgtttcctttcctttttttttggtggagaagcaccaccgacggcgt

agcgagcagtgagtgagtagtactcgtccgctggcgttgaggagggcgtgtagtgcggtg

cggtcgctggctggctggcaggcaggacggcggcagccgggatccgggacaggcgcacgc

tcgcccggccggtgannnnnnnnnnnnnnnnnnnnnnnnnnnnnnnnnnnnnnnnnnnnn

nnnnnnnnnnnnnnnnnnnnnnnnnnnnnnnnnnnnnnnnnnnnnnnnnnnnnnnnnnnn

nnnnnnnnnnnnnnnnnnnnnnnnnnnnnnnnnnnnnnnnnnnnnnnnnnnnnnnnnnnn

nnnnnnnnnnnnnnnnnnnnnnnnnnnnnnnnnnnnnnnnnnnnnnnnnnnnnnnnnnnn

nnnnnnnnnnnnnnnngccgtccccccactcctcggccgcgacaaactcgcatctccggc

ccgatacctcgtctcgatccatcccgtccgcctgcattccgccccactcccagccagttg

gttcggccttttcctaccgtttgcgttcgtttcctttccccacaccgcacgcgccggaag

gaacccggccgcgaaagtcgcttttcgagactttcccactcaccaggccaggccaggcta

ggggttttctttctttcttttcgcttttccggctggccatgtgaatctcgccgcccccgc

ttgtcctcgtgctggccccccccccctgactgacccactcgctgactcactcactcactc

gccgccctctcttccttccttccttccggagcttccactc....................

# >AetMKK10-5 : EMT23048.X

acgcacaaggcgatcaatctaatggcggccgcccgagaaagacggctgccgcagctgcac

ctcacgctcgacgccccaacgtgggccttccggtgcccggccccggcgccggtcaccgcg

gcgacgccgtccacctnnnnnnnnnnnnnnnnnnnnnnnnnnnnnnnnnnnnnnnnnnnn

nnnnnnnnnnnnnnnnnnnnnnnnnnnnnnnnnnnnnnnnnnnnnnnnnnnnnnnnnnnn

nnnnnnnnnnnnnnnnnnnnnnnnnnnnnnnnnnnnnnnnnnnnnnnnnnnnnnnnnnnn

nnnnnnnnnnnnnnnnnnnnnnnnnnnnnnnnnnnnnnnnnnnnnnnnnnnnnnnnnnnn

nnnnnnnnnnnnnnnnnnnnnnnnnnnnnnnnnnnnnnnnnnnnnnnnnnnnnnnnnnnn

nnnnnnnnngcgctggccggcctggcgtacctccgcgcgcgccgcgtggtccaccgggac

ATGAAGCCGGCGAACCTCCTGGTCAACAGGGCCGGTCAGGTCAAAATCGGCGACTTCGGG

ATCGCCGAGGTCGTCTCCCGCGCCGGCAAGTACCGCGCGGCCTACGAGGGCACCGCCGCG

TACATGAGCCCCGAGAGATTCGACACGGAGCGGCTGCACGACGGCGAGGAGGACCGCGTC

GACCCCTACGCCGCCGACGTGTGGGGGCTGGGCGTGACCGTCCTGGAGCTCCTCATGGGG

CGCTACCCGCTGCTCCCCGCCGGGCAGGAGCTGACCTGGGCGGCGCTCATGTGCGCCATC

TGCTTCGGTGAGCTCCCAGCACTTCCCGACGGCTCGGCGTCGCCGGAACTCCGGAGCTTC

GTGTCCGCGTGCCTGCAGAAAGACCACCGGAAGCGTGCATCAGTTGCGGAGCTCGTCGCG

CACCCGTTCGTCGCCGGGAGGGACGTGGCAGCGGCGAGACATGCGCTCCGGGAAGTGATC

GCGCAGCGCGTTTAG

**>AetMKK10 : EMT19304.X**

ATGGCCTCCAGCTCGAGTTCATCTTCTGCTGCCCAAG

gtaggtgtaccggaatatctagcctacacgatccaagtgggtctatcaaatacaaccaga

tgtcgacatacgctcacccttatgaatgcacatatgctcatctaccccgatgagcacctc

tgagatactgagccgaaaaagggttcatcagttattggcgatcgggccgacaagctcacc

atccccacaatagaaagtcaccacacgatctccccccacaaggagcctcgatgctgtgca

gcactacgccgccgaccaccagacgacctgctcggcgagcagagcagcctgccctgtcaa

caatttgaaggtaacattgcaacaaatgtttcaaagttttaggaagccttgtatcagaaa

tgtcttccctgcaagagtgcaacagcaattggctgcaaatctgttgcaagaacaatgcat

gaatgctgatgtacatacacgtgcaattaacgaacacgcaggaacaaataatatactaaa

gcaatgaagatgagaaccatgacctgactccaaaaccaagctgatctcgggccaacatgg

tgtgctaagcggtcgtgcgtgatggcctacgctggcatgcaccgcatcctttcttcaagg

ttgtgctgaaccagaaccagatatgatccgctgactccatgcatggtccaacaacacccg

cacccgtcagcatccatctcacaatctagttttatctatcatgcatcttttttatatttg

tgttgtgttcattctctcgcttagccagtctgcatcttaggactagtaaatatctcctcc

cgctgccctggtagaatatcttctgaattatggctatgttttcccactaagcttgccagt

tgagaggtctggacccacgtcacctggaattaaagatgacagtgttcgttagcgacggcg

gtccttaagcccccaaatgcgaggcttattgagtggataactatctagccccacttgaca

cttgtttataaattagtcgggctcattttaaagttggaacgcatgatagaattataaagg

tttctagcttacatgaactatagtttgggttgtcctcccaccaaccagtcctcttatgta

ctttcatgcacttgaactactcgtgtggacgtggtaaagcggaccgagaacctaattttt

gagagtacaacacacacatataacaaagtgaaacgaatataaacatacaaaaccaatgtc

cactaaatagattatacttgtggtagctaccatgctacgtacacactagcaaatttaact

ttcatgttaccagaagagggcatcatgttctgaatactagcttaatttactatttacata

agaaattatgagatactaattgtgaaacttgactttcctaaatcaatttatggacttgaa

agaaattacgtagttaaagcatttttggaatttcttgacattatttgtagatatatgtat

attacattatttgtagatatatgtatatcttttagaaattttgtgcttcctccaatccaa

cttacttgtcacagctctagtaacatggatcaaagggagtaactcgatttatggacttaa

agtattctattctaatctatcatgcatcttttggtttgtgttgtgttcattcacgcgctc

aaccagtctgcatcttaggactagtaaatatctcctccgctaccagaagaatatctcctg

aattaggcatatgttttcacactaagcttaccaattaagaggtacatatccacatcaccc

gaaattaaagataacaatgttcgttagcgacggcggtcctttcaacccccaagtgtttgg

gattaatatcaagatcaggcttagcatagtggagaattatataaccccacttgacacttc

ttcaaaaattagtcagactagtttaacagttttaacacatgacaaattagtcataatact

tgaattatagtagaacacacacatataataaagggaaaacaaacataaacatacaaagca

aaatgtgtgtgtactagttcaactgaataacatcatacaaaccctaaaaactaacataat

acaagactcgcggtagctaccatgctacaaacacactagcaaatttgatctaagtgatat

tggaaaggacatcatgttctgaatagtagcttacttactattcacataggaaattttgag

atacttatgatggtgaaatttgactttcttaactcgattagtggacttaaattatgtttg

gaactttgtgacactgtagatagatgtacattatgttgatgtagtgtatatattttagca

ctttttttaataacactttgctagtctaacaatgtattgatagcaggagaaggtggaggc

ggtggcaggagctggagaatgacgaggcggcagtagcggcaattgcagttagggtatagc

gtagtgaagtgaagattgatctcaagacatgtgaatggagatctaacggctcggggggaa

acaaatcaagttccgctgcctaggccaaatacatacatcaaactcctaactcctaaagca

ttggtgtattattatagagcggtattaagcttacaagtgtatcttaccagccaccaacga

ttctcgtccggaaacgtaatagaggaaaaccggtgtaaacattacatgcccaaaacggcg

acccaacccaagcatccggaacaaaccaactgtctattttttgcctaccaatgttttagg

gggtatttcgaaggaaacgtttggcataactctccttaaatttcagactcaataacatca

tgaatatccattcttgcacacaaagataattaagcatattttcacatcgttttcaaaaga

aaggtttaaaaagattcggaagttacacatcaaaatctcagcacagtcagccaaatttaa

ggcaggggaacttgcgccgccacatcgcagtataaagcaagctaacctcacctccacaaa

aaaacatcatccattactcgatcatcaatttcatcatacttttgcgaatacagttcttgc

aaaacacacacacacacacatacacacggcgatcaagatcatggcggctgccagagaaag

acgactaccgcagcttcacctcacgctcgacgcccccacgtgggccttccggtgcccggc

ccctgctccggtcaccgcggcgacgccgtccacgtcggcggctccgccgcggcgcggggt

gcggcgcaaggacatacacannnnnnnnnnnnnnnnnnnnnnnnnnnnnnnnnnnnnnnn

nnnnnnnnnnnnnnnnnnnnnnnnnnnnnnnnnnnnnnnnnnnnnnnnnnnnnnnnnnnn

nnnnnnnnnnnnnnnnnnnnnnnnnnnnnnnnnnnnnnnnnnnnnnnnnnnnnnnnnnnn

nnnnnnnnnnnnnnnnnnnnnnnnnnnnnnnnnnnnnnnnnnnnnnnnnnnnnnnnnnnn

nnnnnnnnnnnnnnnnnnnnnnnnnnnnnnnnnnnnnnnnnnnnnnnnnnnnnnnnnnnn

nnnnnnnnnnnnnnnnnnnnnnnnnnnnnnnnnnnnnnnnnnnnnnnnnnnnnnnnnnnn

nnnnnnnnnnnnnnnnnnnnnnnnnnnnnnnnnnnnnnnnnnnnnaagatcgccgccttc

ggcatag

TCGAGGTCGTCTCCCGCGCCGGCAAATACCATGCGGCCTACGAGGGCACCGCCGCGTACA

TGAGCCCCGAGCGCTTCGACACGGAGCGGTCATTGCAGGGCGACGGTGAAGAGAAGGGCC

CCGTCGACCCCTACGTCACCGACGTGTGGGGCCTGGGGTGA

**>TuMKK1-1 TRIUR3_03040.T1.X**

ATGAAGAAACCGGGCAAGCTCGCGCTGCCCTCCCAGGACTCCACCATCGGCAAG

TTCCT

gtaagcaatccacctccgactgtccttctccctcctccttcagtttttcccctccttcgg

atgctttgctggtgcagtcgtgctgactggttttcttctccggcgcgggggcag

GACGCAGAGCGGCACGTTCAAGGACGGGGACCTGCTCGTCAACAAGGACGGGCTCCGCAT

CGTCCCCCAGAGCGAGGAAGGCGAG

gtaagccggcccactcaacccggcaccccctagattgaaaattaaaattccccagctcat

cttgtcctagacacgtagggctggacctgcctggcagattggtgcagcccgtactgtttt

ttgtactagtaatctcgttgatatcgtcagcagattttaaatttcttttgggatgcgtga

ttggattagcagcaggggttcctgtcctccggtgcttggttaccttttcagaagaacgat

ttaggaaaaataatgatatgtactatcgttaattttaattttattttatttccctcaaag

aacattatgtcccccttttcagaccagccattcagccaaaaatcgaatcaactcttctcg

acaagagatggtggccttgcatagatgtaaattgtacttattttttatagtgaccttgta

tacatgagattggatcagagggtagggatcaggggtttcctatcccctaatttcagaata

acgtcttaggaaaaatgatgggagtactactatttaggatttattttatttcctttagat

ggtggaatacatgcggtcatctttcatgtggggatatcctcctatgcagtttattactga

taccatttatcacgcatccagtagttggtcatatttatacggaaatgctgatgctcactt

gcttataccggaagagtgctgtctaccgccatttttgtttttggaattgtctaattgcat

tcctgttctgaacatattatactcttcatttatctagtctgtgtcttaccttctttctgt

tgacatactcggcttctctag

GCTCCCCCTATCAAGCCGTTGGATAATAATCATCAGCTGAGCATAGACGATCTAGATTCA

ATCAAAGTGATCGGGAAAG

gtaatagcggaaccgtgcaattggtgcgccacaaatggaccggccagttttttgctctca

aggtattgtcccaaacatacattatttgatgctttttttctttaaatgtttgctatgtat

ttttggttccttcgacttttttcgttacaaaattatggtggtgactgtaactatcctctc

tgctattttacctatatatatgttacaaagggtcttcacctccattgctgcactgttgtc

atgtctgcag

GTTATACAGCTCAATATTCAGGAAAGCATACGCAAGCAGATGGCCCAGGAGCTGAAAATA

AGCTTGTTTACACAGTGCCA

gtatgttgtcacatgctatcagtgtttctatgtcaatggtgttatttctattgctttgga

gtatatggatggtggctctctcgctgatttcctcaaggctgttagaaccgttccagaggc

ctaccttgctgcaatttgtaagcaggccagtgaaatgtcacataatcaaccacatagttt

ccccttctttttttggctatcagaatgtcgcctcaagatgaaatagaccttttcaatcga

caattgtaacttcctcatgcag

GTGTTGAAAGGGCTGATGTACTTGCACCACGAGAAGCGCGTTATACACCGAGATCTGAAA

CCATCGAATATATTGATAAATCATAGGGGTGAAGTAAAGATATCAGATTTTGGTGTTAGT

GCCATCATCTCGAGTTCTTCCGCACAGCGAGATACATTTACTGGCACATTTAACTACATG

GCG

gtgagtgaaagtttgtttaaattatgtagacatgtcttccagtgtggcatgaaaagatgt

ccaccaattttttttgttccagctaaccaatgtaaaatgtatactcttgacag

CCTGAAAGAATCAGTGGGCAGAAACATGGTTATATGAGTGATATCTGGAGCTTGGGCCTA

GTTATGCTGGAATGTGCCACTGGCAATTTCCCATATCCTCCTCGTGAAAGCTTTTATGAA

CTTCTTGAAGCTGTTGTCGACCAACCATCACCTTCTGCACCATCAGATCAGTTTTCACCA

GAGTTTTGTTCATTCATTTCTGCTTG

gtatttgtgcttcttcattgacccccctttgttaatcaatattacttgggcaatatttgt

aatttgtttagttctaaagtttaaaactatgagcttagctacaataccatcatagtgttt

tttctaccatttgcttgtgagttgtgaagtagttattctctggaactggaatatgcttgc

ggacacgtattgtctgaacctacaatttgtttttctctcatctcatctgttggcagatta

attgatcttttatttatttgtttatttattatgcaacatcagtgtccaacagaaggctaa

tatctgttttcttatttctttttggatttcag

TATCCAGAAAAATGCTGCAGATAGGTCATCTGCCCAAACCCTATCA

gtaaggaaatttctgtttgcttcccaattttgtatgttctttcgaaagcatcattagttg

gctgactgatcttgatccaattcttctatctgttgcctctgacgtccag

GCTCATCCATTCCTGAGCATGTACGATGACCTGAACATCGATCTTTCTGACTACTTCACG

ACCGCAGGATCACCACTTGCCACCTTCAAGTAA

tccaataacccactgaagagtacatacatggttattgcaattcctttacaattccagtga

tggtctattctcgttttttttactaggcaaatcgcattgtga

**>TuMKK1-2 : TRIUR3_10169.T1.X**

atg

cagatcgggatgttcaaggacggcgacggcaacctgctcgtcaacaaggacggcctccgc

atcaccccgcagaccgaggaaggcgaggtaagccactaaaacatgaccgggcacccaaga

tgaaaaattcctccacgcgtctggtcttagccgttcataggaaaagaacgtagggctgcg

ctcggcatattggtagttgtattggttttcgtaatttcattgatgacatgaacagattta

gatattaggatgtgtggttggattagcaggtacggagcagggtttcgtgttctttttcag

aacaacaattgaggacaaatgatactatgtacttccttcgtccgggattagttgtcgctc

aaatggatctatctagacgtatttcaatgctagatacatccgtttgagcatcaactaatt

ccgtgcggagggaatacataggtgattttcatgtgggatatccgcaaatgcagttgttac

agaaataatttatgatgtatccagaatgctgatgctcacttgcttatagtggaagattgc

tttctaacaccatttcttgtgggaattgtctaattacattgttgttcttaacttgttgtg

ttcttcattcatctaacttgtgtctcgccttctttccgttgccatactcgacttctctag

GCTCCTCCTATAGAGCCGTTAGATAACCATCAGTTGAGCATACATGATCTGGAAGCAATC

AAAGTGATTGGGAAAGGTAGTAGTGGAACCGTGCAATTGGTGCGCCAGAAATGGACTGGC

CAGTTTCTTGCTCTCAAG

gtattgcccaaaaaataaattatttgatgatttataattttggttccttctatttctttc

tgtagaaagtagaaactatagtggtagttataatctctgatattttgcctagggtcttta

cccccattcatgtactgtcatgtttgcag

GTTATACAACTCAATATTCAGGAAAGCATACGCAAGCAACTTGCCTAGGAGTTGAAATTA

AGTTTGTCAACACAGTGCCAATATGTTGTCACATGCTATCAGTGTTTCTATGTCAATGGT

GTTATTTCTATTGCTTTGGAGTATATGGATGGAGGCTCTCTGGCTGATTTCCTGATGACT

GCTAGAACCGTTCCAGAGGCCTACCTTGCTGCAATTTGTAAGCAG

GCCA

gtaaaatgacacataagtaactacataggtttttcttgttatcagaatgttttttttcac

cttaagatcaaatagaccttttcaatcgataatttttacttcctcgagcaggtgttgaaa

g

GACTGATGTACTTGCATCATGAGAAGCGCGTTATACACCGAGATCTGAAACCATCGAATA

TATTAATAAATCATAGGGGTGAAGTAAAGATATCAAATTTTGGTGTTAGTGCCATCATTT

CTAGTTCTTCTGCAAAGCGAGATACATTTACTGGCACATTTAACTACATGGCGGTGGGTG

AAAGTTTGTTTGACTTATATAAGCATGTCTTCCA

gtgtggcatgaaaagacggccaccattttttgtttcagctaccaaatctaaaatgcatat

atactcttgacag

CCAGAAAGAATCAGCGGGCAGAAA

C

ATGCTGGAATGTGCCACTGGCAATTTCCCATATCC

TCCTCGTGATAGCTTCTATGAACTTCTTGAAGCTGTTGTCGACCAACCATCACCTTCTGC

ACCATCAGACCAGTTTTCACCAGAGTTCTGTTCATTCATTTCTGCGTG

gtatttgtgcttctccattgcctcccccctcctcnnnnnnnnnnnnnnnnnnnnnnnnnn

nnnnnnnnnnnnnnnnnnnnnnnnnnnnnnnnnnnnnnnnnnnnnnnnnnnnnnnnnnnn

nnnnnnnnnnnnnnnnnnnnnnnnnnnnnnnnnnnnnnnnnnnnnnnnnnnnnnnnnnnn

nnnnnnnnnnnnnnnnnnnnnnnnnnnnnnnnnnnnnnnnnnnnnnnnnnnnnnnnnnnn

nnnnnnnnnnnnnnnnnnnnnnnnnnnnnnnnnnnnnnnnnnnnnnnnnnnnnnnnnnnn

nnnnnnnnnnnnnnnnnnnnnnnnnnnnnnnnnnnnnnnnnnnnnnnnnnnnnnnnnnnn

nnnnnnnnnnnnnnnnnnnnnnnnnnnnnnnnnnnnnnnnnnnnnnnnnnnnnnnnnnnn

nnnnnnnnnnnnnnnnnnnnnnnnnnnnnnnnnnnnnnnnnnnnnnnnnnnnnnnnnnnn

nnnnnnnnnnnnnnnnnnnnnnnnnnnnnnnnnnnnnnnnnnnnnnnnnnnnnnnnnnnn

nnnnnnnnnnnnnnnnnnnnnnnnnnnnnnnnnnnnnnnnnnnnnnnnnnnnnnnnnnnn

nnnnnnnnnnnnnnnnnnnnnnnnnnnnnnnnnnnnnnnnnnnnnnnnnnnnnnnnnnnn

nnnnnnnnnnnnnnnnnnnnnnnnnnnnnnnnnnnnnnnnnnnnnnnnnnnnnnnnnnnn

nnnnnnnnnnnnnnnnnnnnnnnnnnnnnnnnnnnnnnnnnnnnnnnnnnnnnnnnnnta

gaagctgccccccccctggttaatcaataacacttgagcaatatttgcaatttttgctga

gatttaaactgtgagcttagttaccatagtgggttttctaccttttgcctgtcaagtagt

gattctcacgccgaaattcctgctctggaatatgcatgccaacatcatcatatgtttctt

ttttttacactcacctagtcaccttcatttgtggacagatcaattgatttccttatttta

tttactggaagtcagtgtccaaaaaaggctaatatctttttcttaatttctttctgaatt

tttcag

TATGCAAAAAGAGGCTACAAATAGGTCATCTGCTCACATCCTATCA

gtaaggaaatttctgtctgcttcacaatttgtatgttcgtccgaaagcgtcatttgacac

agcaagctcactgatctttacccatttcttctatctgttgcctctgacacccag

GCTCATCCATTCCTGAGCATGTATGACGACCTGAATATTGATCTTGCTGTCTACTTCAGG

ACCGCGGGATCTCCACTTGTCACCTTCAAG

taa

**>TaMKK3partial TRIUR3_00549.X**

ctaatcaagggtgaataaaaaattgcagactttggtgtgagcgctggtttggacggcacg

ATGGCCATGCGTGCCACCTTTGTAGGCACCGTGACATACATGTTGCCCGAGAGAATTCGC

GGCGACAACTACTTGTACGCCGCTGATATCTGGAGTCTCGGGCTGACGGTACTGGAGTGT

GCTACCGGTAAATTTCCCTATGATTTCAGTGAAGGCCCAACCAATCTCATGCTGCAGATA

CTCGATGATCCATCGCCGACACCACCAGAAGATGCATATTCACCGGAGTTCCGTTCATTT

ATAGATGACTGCTTGCAGAAAGAGCCTGATGCAAGGTCTACATGTGAGCAGCTTTTGTCA

CACCCATTCATCAAGAGGTATGAGGAAGCCGGCGTCGACTTGGCGGCATACGTCGGGGGT

GTTGTGAATCCAACAGAAAGGTTGAAGCAAATAGCAGAGATGCTTGTTGTTCATTATTAC

CTTCTGTTCAATGGCTCTGATGGATCCTGGCATCATATGGAGACACCGTACATGGAAGAA

TCATCTTTCCGCTTCTCAAGGAATGTGTATGTCGGGCAAAGTGCCATATTTGATACTTTG

TCAACCATAAGAAACAAGTTTAAAGGTGATCAGCCTAGTGGGGGGATTTTTCATGTTGTT

GAGAAGATACATTGTGGTGCAGATGGGGAAAGAGAGATCACCGTTCGTGTGTCTGGATCA

TTCATTGTGGGCAACCAATTTCTAATACTTGGTGAAGGGCTGCAAGCTGAAGGGATGCCC

GGCCCAGACGAGCTCGCAATTGACATTCCAAGCAATCGGGCAGGCCAGTTCCGGGAGCAG

TTCATCATGCAGCCAGGGATTTCCATGGAATGCTACTACATACCAAGACAAGATCTCTAC

ATCAGCTAG

**>TuMKK3-2 : TRIUR3_07377.T1**

ATGTCGGGGCTAGAGGAGTTGAAGAAGAAGCTGCAGCCCTTGCTGTTCGACGACACGGAC

AAGGGCGGCGTCAGTACCCGGGTTCCCTTCCCGGAGGATACATGCGATTCCTATGTG

gtaaaacacagcctctcattctttcttctctgcatgcaaaccaaattaggttgtacaccg

gcagcattagcctggggttgttgtctgttaatggaattggtaccagttgtttcacttttg

ttttccagtcatgaaaaccatcctgccagcaactgaatgtacatgctcaatgcttctgta

cctttggggtagcaagttcgctgcatggacctacttgaccaatcgatcctgacacatgaa

gtgataggatgtacaatattatacgtagtcataagattggatattagtcataattactta

actgaagagaatcacttaccctttggaaactatcatttagaattccctcagctttgtttt

tatctttgtgaatttaaacttgtttagatgtggaagtgtttgttttttaagttcttcctt

tcgtgaag

GTGTCTGATGGTGGAACGATAAATTTACTGAGTAGATCGTTTGGTGAGTATAACATCAAT

GAGCATGGCTTTCATAAGCGAAGTACCGGACCAGAAGAGCCAGATACCGGTGAGAAGGCA

TACCGATGTGCCTCTGAAGACATGCACATATTTGGTCCCATTGGGAATGGAGCAAGCAGT

GTCGTGCAGAGAGCTATTTTCATACCAGTTCATCGAATTCTGGCCTTGAAGAAGATAAAC

ATATTTGAGAAG

gtacattctttctcgtccgaaatgcagcttacaaccacattgttgtgcacttctggttta

catgaacaccccaacaaggctgttttcgcttttaatttcctagtcattgttcattctact

tcattggactgaacatataaagaagattgtgagaacttggcttaatatttgtacccacat

ttaagtggaacttgtgttgataactagtctgaacctcattcgtgttttgaaacaaaatag

tattaaagggagcaaacaaaccgtgtggtttacttgcatgctggaccattctttcctttc

tttaggagtcatgtgcatgagtgcatcacaatgggcccatgatatccagcacatggatgg

gtagcatcaatcacattgccctgaaggattttggttactagtgataagctgccaatttct

ctattgcatattcacaatgtttcctatttagaagggaaaaacatctcaatggcagggtgt

tggatatgtggaagcgaaacagtgtgagactgtcactatgtacattgtttcgttgggatt

acaaatagtattcggacagtaacttcatatcagactcagtctaggcagttgcacttagca

gaaacgtactcttactcatctaaactcgcctaaccagttaggacatgcctgactaataga

aatatgacacatcatccttagctacctgcattgtttcctgactcatctgcaaaatgggcc

catgacctgcagaaggaagggtggcaaaaatgcccaggataggggatttcagttgctagg

gtagggtccnnnnnnnnnnnnnnnnnnnnnnnnnnnnnnnnnnnnnnnnnnnnnnnnnnn

nnnnnnnnnnnnnnnnnnnnnnnnnnnnnnnnnnnnnnnnnnnnnnnnnnnnnnnnnnnn

nnnnnnnnnnnnnnnnnnnnnnnnnnnnnnnnnnnnnnnnnnnnnnnnnnnnnnnnnnnn

nnnnnnnnnnnnnnnnnnnnnnnnnnnnnnnnnnnnnnnnnnnnnnnnnnnnnnnnnnnn

nnnnnnnnnnnnnnnnnnnnnnnnnnnnnnnnnnnnnnnnnnnnnnnnnnnnnnnnnnnn

nnnnnnnnnnnnnnnnnnnnncccccccccccccccgcgcgcgcgctacacacacactct

agaatttcaagctgcctatttattgatttctcattcacagtgctctctagttttatattt

ccaactactacagatgccatggctcatatcatgatatttgattaagcaaagaaagagaac

agaagttcgtctcctctctagtcttctctgaccccaaaggaagctctcttcaccatgcca

tgctctcgacacatttgcaccataatttagtagcaggcgaccaaatatcccactacacaa

ttttcttggtgtaagctggagtgaaacaagttggagttgagtatattaaaccaaaacatg

tatttgcattgcattgaaggcagcacgaagggattctggtttattgcagtggcagtcaag

tagcagagtcagttctgactgcatcattaaagcagatacaccaaagaatagaaatgctct

ctctctctctcttctatgcttcaattcatatactgttgcatcagaacatttttggtattg

catggagtaattttagtattttatgtttgtctatatatttgattgatataattaattcat

tctatttgagttgataaactgatttgcgaagttttcttttaatatttttcacgtgtcttc

atacctttacag

GAGAAAAGGCAACAAATTCTTAATGAGATGAGAACATTATGTGAAGCAAGTTGCTATCCT

GGTTTAGTTGAATTCCAGGGTGCATTTTACATGCCTGATTCTGGACAAATAAGCATTGCC

CTTGAATACATGGATGGTGGCTCTTTAGCAGACGTTATAAAAGTCAAGAAATCAATACCA

GAGCCAGTTCTTGCACATATGCTACAGAAAGTGTTACTT

gtatggatcttccacatcctcttcctttcatgtctgtgttgatcaaaatcaggctgtgac

ctgatgtgtttttgaagtaataaattattctttgtacaattctgaag

GGCCTGAAGTACTTGCATGAAGTAAGACATCTAGTGCATAGAGATCTAAAGCCAGCAAAT

TTACTGGTAAACCTTAAGGGCGAAGCAAAAATTACAGATTTTGGTGTAAGTGCTGGTTTG

GACAATACAATGGCTATG

gtatgctacttctttgtgacaattatcacgctgttcttttgatattccttaaatatgagc

taattatttaataatagtacatcaatttcatctaatcatttaaagtgaatgaacaatatg

ttttctgctaaataaatatgttccag

TGTGCTACCTTTGTAGGCACTGTGACATATATGTCACCTGAGAGAATCCGCAATGAGAAC

TACTCCTATGCTGCTGATATTTGGAGTCTTGGACTAACGATATTGGAGTGTGCTACTGGT

AAATTTCCATATAATGTCAACGAAGGCCCAGCCAATCTCATGCTACAG

gtgattgactactcagatttttttttattcttgtccatcattcattacgtatgttgggat

ggaatatcatcccttttacagactgatgcaaagtgtggggactatttgagctgttcacat

ggtagccttggtgggttgtatttgaattcgttgatatggcaaagaatgttccatttctgc

atgtctgttcgatgattatattagtttccacaatattttcttttttccttgctggtttat

gttttgtaagttgtttttttcattttctttgaatatataaattatctgtcacttcacttg

ttgccctatcttaatcttaaaatcacatacttcaggtactgtttttttcccactcgattg

ttgtcctctcttacaatcctaaaatctcttatgtcag

ATACTCGATGATCCATCACCAACACCACCAGAAGATGCCTATACACCAGAATTTTGTTCC

TTCATAAATGATTGCTTGAGGAAAGATGCTGATGCAAGGCCTACATGCGAGCAG

gtagaaaacatgtatattctaactttcatgcaatctgagtccagtaggttagcaatttgc

atcaatgtcttgtggtctgtttgaatgttattacatattactggtaataactttttttga

taaagcattagtggcaataactgtcagagtattgtatagttgtgtggttaagtatattta

ggtatttattaattgatttaccttctcttgtaagtatgtcacactggcctttggttgatg

aagacatcaccgcccctatcattggtccattggaataaacgaggaggcccattccacata

gagaccttggtttctcttctctccctctcgtaccatcatgtacagacttccataccctag

cccacagcagccaccattgctgcagccaggacaccatcaccaccaatgttcgccgtccac

cgtatgtgcagctggggctggaaaccttaggcgcagggagaaggaaagtacgtgatggat

caagtagaagatgggtttcttctaactccctggttaactgatttaacgtgtggttacctc

attatattgaggggctgccttacatgtgctagactctagtcctaatacaacatgatctgg

acttggcaacatgtccattataataggaccactacctaaactaagaccagtgatcagaat

tatcactatcccactacactcaccttcttcgtgttatgtgatgccttcgagtgaccatca

gtcctgccttttctaatcttggttcagctctctgcacattaggcgtacacagatactact

tgggctagtgataattctgatcactggtctctctgtgcctactgcctcaatgatttcctg

gaagatcaagcacattgtagttttccttccgaatgcttaggccgagttcgttaggttgct

agtcaagcagatccagtgaagcatcttaattcttaagcaatgcatcggctactctacgtc

agtgaagcatgatctctccaagcacattggagttgatgcttcctgcatgaaattgcagat

gcaggagcgggttgttgctctccagtatgtgccatcagaactctagttagctgatttctt

caccgaagcgcattctatagctttgttgttgatctgcctttaattttgtgtatggtatta

ttggaggtccatttcacaaacttatatgcaaaatgtaggtactgattagttatggcttgg

tctaataacagaatgttaattatcttgacaatgcattttacatcttttatccttttccat

tgtttggttttcttatagtggactaacatgtttaacattctacag

CTTTTGTCACACGCATTTATCAAGAGGTATGAGCAAACTGGTGTGGACTTGGCAGCATAT

GTCAGGGGTGTTGTTAATCCAACAGAGAGATTAAAGCAAATAGCAGAG

gtgaattctttagagcatatttcagcttattgttttcttctacttcagttaaatatcagg

acacacacttgatatgaaacatgccccctatgtcaagaattgaagctagaactttgaagg

gggtcagggagggggaggtggggtagaggctatatgttggagatagacaaacactgttgc

ttgatgatgttaccgtggctcactttcttttgcctctgccacctcactgctggtggtcac

cattcctatttgttccacttccatctacctccatttgggaacgagaggaagaaggggaga

gcaagagggtcaggtttataatacagccagtatataatcagtcacctccaccaaggaaca

cttccgcacccccatcgatcctacttgttgagccaccttaaccatgttccatggccctgt

tccacaactcggttgatccattttgcttgtaactataggttgtacattagaaaaatggaa

gaacaatgtggcgcaagtgtgaaccatttgagcggatgcactacagtattcttatacttc

cattggttgagtaaaatattgctctagcgccttcattttgttgaactgcagtgctagtcc

ttcgtgtgtagaacaactggtctgatagtttgtttgtgccctaatttgtttgtttgtgtc

ctgataatttgtttgtgccctaatttgtttgttattgcccagagaaacccttttgttttc

cttttaatgaacaaagtccattgaaaaagaaattatttataagccgtgagggtttgagtg

cccctatccccatacctgccttaactatgttatttatccattcttttccttttcaactcg

cctgctgattctttgacgttagctcgtttggttccacatttcggatctcaattcaaacgt

gtagattaaaaggacttttgttaatagacccacccgtatcaaacaagggtactcactact

tgatttgttccttttttcaagactcagtattctcgaacataacttatccaatgctttcac

ggacacacttgtccaaatatcactcagaaattcagactgaaatgatggtatgcccaaata

tccaagctcaaagtgaaaagtaatccattagctcccttttagttctggcaattcattaaa

gatgcacaggttaccactatatgtacttaagcagatttcccccctgaatcccag

ATGCTTGCTGTTCATTATTACCTCCTTTTTAATGGCTCTGAAGGACCTTGGAATTATATG

AAGACATTCTACAAGGAAGAATCATCTTTCAG

gtacccgaggcaatgttacaatttttttagcatgatcctaaaacatagatgtactcttac

tgctgtttaggcctttaacctttatgtagattgtacctccaatagttggttacagtatcg

ttgaaattattttggtactaataacatcaagtatgtgttctgtgatggttaataacttaa

tatgaaaacttgctgagacgatatcattgagttgccagctagttgatggagctagctgaa

ttgaccgttgcacttttgcacaattcatcatttcctgttaatctggcccatttgtttcct

actttatcctataggcagtgttgctttccaaatttatcctgtcaggcattgttgcttctg

ttgtgcctctatattactgtgtgtcgaagaacccaagctgtttctgagcacatgcatgat

atgtggtgttatctcctgtttgtgcag

TTTTTCAGGGAATGTGTATGTCGGACAAAGTGCCATATTTGATACTTTATCAAATATAAG

AAAGAAGTTGAAAGGTGACCGGCCTAGAGAAAAAATTGTTCATGTTGTTGAGAAGCTACA

TTGCCGCGCGAATGGGGAAACAGAAATTGCTATCCGTGTGTCTGGATCATTCATCACGGG

CAACCAATTCCTGATATTTGGTGAAGGGTTGCAAGCTGAAGGGATGCCCAGCTTGGAGGA

AATTGATATTGACATTCCAAGCAAGCGGGTTGGCCAGTTCCGGGAGCAGTTTACCGTGCA

TCCAGGGACTTCCATGGGATGCTACTACATAGCAAAGCAAGATCTCTACATCGTCCAGTC

ATGA

**>TuMKK3-3 : TRIUR3_10006/ TRIUR3_10007**

ATGGCCGACGCCCCGTCGAGTTCCGGCGGCCTGGCCGTCGACCCGCCCGCAAAGGTGAAG

AAGAAGGCGTCAAGGAAGCCACG

gtcagagtgcacgctggaggagatcgccaagttggacgcggaatcgacgaagaggaggaa

ggagagcggtcgtcaaggtcaatgccgccgcgaccaagttcgccgcccaacgcgatgagc

tggaggccgcgcggcgcaatgccgcggccgacaaggaggaccttgtcaacaaagcgcacg

ccatcctcatgcttggcatgggccgtccggccgggttccctgcag

CGCCGCCGTCGACACCGCGCCCCGCGACCGTCATCGACCTCAACGTCACGCCTGGGTCAA

GACTAACTCCAAGGTTGATGACATACGTGATGCCTCATCCATGACATTGCATGACACTTT

GCATGGCATGATGTCCCTAAATGATGTGAGGGACAAGAAGAAGCGGCAAAGCAAGGAGGA

GCAAATGAAGCAATACCTAGACCTTCAAAGAAAGAAACTTGAGATAGAGGAGGCGGCCAA

GAGGAGGAAGATCGACATGGAGGAGGTGGCCCGGCAAAGGCATCCCGACATGGAGGAGGC

GGCCCGGCAAAGGCAGCTCGACATCGAGGCCGACAAT

gtcaaggctaggcagaggcagctcgacatcgaggccaccaatgccgccacccaagcgaag

gagggggccnnnnnnnnnnnnnnnnnnnnnnnnnnnnnnnnnnnnnnnnnnnnnnnnnnn

nnnnnnnnnnnnnnnccgttctttttggaggttgacatgattgtcgcccgcccgctgggc

cgctggctgtgtgccggcgagaaaacattcattttgatggccggctgtgttgccggccgc

tgactgtgttgccggcgagaacaactattcattttggagggtggttgtgttgccggccgc

tggctgatgccgatgatgaacgtgtaggccgctggctctattgccggcgtgatgaaccag

ggccgctgacctgaactaaggcttgatatctgaaacgttatttttgattttttaaataaa

tatgaacatgatggggccgctgactgtgttgccggcgagaacaactattcattttggagg

gtggttgtgttgccggccgctggctgatgccgatgatgaacgtgtaggccgctggctcta

ttgccggcgtgatgaaccagggccgctgacctgaactaaggcttgatatctgaaacgtta

tttttgattttttaaataaatatgaacatgatggggcaaatggatgcggccgcgcgctgg

gtgcacggccaccgcatcccaggacacgcccgaatacgacccaaatccctatcaaacaga

cagaatccgattacgcggtggagttggccttaccgacctactgcgccgcctccggccatc

tctcaaatatgggactatcgtactggcggcggaggaaagcatgtgcgtcccttcccgtcg

tggcaaacggaagcgaaatcaaagcctcactctgttttctctgcgccctctccctgaccc

ttctcctcttaggcttagccttgtttggctgtaatggctggactggag

GACCTGAAGCGGAGGCTGCAGCCCATCTTCTTCGACGCCGATGGCAATGCTGTGCCGCCC

CCCGCCGCCGACGGGACATCCGACGACTCCAGCTCTGACGACTGCGAG

gtaaccactctcttcgtttcagccgtttggccagttcgctgagatctatgtttatcttca

ttctgttcaaccaaccaaattccttgcttatgttgctcatagtatgttttttcttaagcc

tgctataaaaatatatatgatgctcatatttaatcttacttttgtaaag

GTTTTGGATAGTGGAACTGTCAATTTATTGAGTAGGTCCTCTGATGAATATAACATCAGT

AAGCTTGGCTTCCATAAACGAACAACCAGACCAGATGGGGACTATGCTACAGATAAGGCA

TATCGATGCTCTTGTCATGATATGCATATTTTTGATTCTGTTGGTATTGGTGCAAGCAGC

GTTGTCCATAGAGCTATTTATGTACCAGTCCATCGAGTTTTGGCACTCAAGAAAATTAGC

ATTTTTGACAAG

gtgagtgcaaaactttttatagtccatatgccataagctgacatctctaggttcccctta

catgaaacaaaatctatggataccccttttttttctttttcttttaattaactttcacat

ggaactacaagtgtagaattcatagcttattctccatttaatgcctattcatagacattg

tttggaccaagcttattctctttatatttattataacgtattctctgatatatattacag

GAAAAGAGACAACAGATTCTTAATGAGATCACAACGTTATCAGCAGCATCTTGTTATCCA

GGTTTAGTTGAATTCCAAGGAATTTTTTACACTCCGGACTCTGGAGAAATATACTTTGCT

CTTGAGTATATGGATGGTGGTTCATTAGCAGATATTATCAGGGTCAAGAAATTCATAACA

GAACCAGTTCTTTCACATATGCTACAGAAAGTGTTGCTA

gtacgtgtctcttgcctccatatgttctgatgttagctctattcaaatcaggcctgtgaa

atttaagtgactagttagtctctgttaatttattag

GCTCTGCGCTACTTGCATGAAGTGAGGCGTTTAGTTCACAGAGATATAAAGCCAGCAAAT

TTGCTTCTAAATCTAAAGGGTGATACGAAAATTACAGACTTTGGTGTAACTTCTGGACTG

CATGATTCAGTTACCATG

gtatgctacttcttggtgatcgaaacttctcttagcaacagcaagctgttctaaaatagt

attaagtggaaatctgactatctagattgaataagcggtatatatgtgtagtaattgtct

tccag

TGTGCTACCTTCCTGGGCAGTGTCACATATATGTCTCCCGAGAGAATTCGGAACGAAAAT

TACTCATATGCTGCTGATATCTGGAGCCTTGGACTAACAGCATTGGAGTGTGCAACTGGA

AGATACCCATATGATGTAAATGGGGGCGAGGCCGACCTCATGCTGCAG

gtgaggtggttggcggtcaagcagtgttcatttgtataattttctactgcctttcgtgat

catcttatttatggaaaaaaaatcatgatctcatttatgtgtatctgttggtatattgtt

aattaaagctgttttcggaatttggcttgtttttcgtaggcgttctacatctgtttcgga

atttctactggatttagaacttctatttcatttctgctttccaacatgaatggtctgaca

ataatgcaatactagtttggtagttcctcttgtgagtattatattattgatgtatagtgc

aatcctcctttcccaccttactgtgttgcagtcttgcagatattggaagatccatcgcca

acaccaccacaacatatgcattcagaagagttttgctcgttcattgatgcttgcttgcag

AAAGATGCTGATGCAAGACCAACATGTAATGAG

gtaaagtgacgtctagtacaattaatttttttcaagagaaacgatacgagggtttccaac

tgcgtaaatcttgtgataatctttatttattatttaatttttgtggggagtgataaactt

tattatgcagtaagtataggattttgcgacactatctgagagtaatgtctgatggatgtt

tcagctctttagtcaacagattagcggtctaaggtgtgtctgaggtgataattgcaaaaa

agcggtctcatatgttgaattgttttacaaattattcattgtcaactaactacataaata

ggttatttgcaacaaagacgaaatggggtttattttcctctctgaaagaaaaatcttatt

tgaattacatactgaatatcttgtag

cttttgtctcattccttcatcaag

AAATACGAGGGACCTGGTGTGGACTTGGCAGAGTACAACAAAAGTGTTCATGATCCAGCG

GAAAGATTATCGCAGATAGCACAT

gtgagttcctaatagctttcgttatcttctgtttttcctgttctgctgcctttccatatg

gag

GAAAATGAACCTGATGTTCCACAAGTTCACATACAATGA

tgttagctatgcttagacaatagaacat

tccgacaattcaacaaataaactgactcttactgcagttgtttcaatctttttaatctgg

tttcttgctatccgggattcaaaatattcatgttaaaatttttgacctcgttgaggattt

cggttgccacaagcatagttcaaaaaaccggaccgggccagcggtcggaccgggaaaaac

cgaaccggcggcctcggccgttttttaagctaataagaccgttctgcaattggaccggag

aaaaccagtcaagccggccggttttctgaaaaaaaacgctaaataaaccaggcctttgaa

ctggaaaaaaccaggaaaatggttaatgtagaatttgggagaagcgggagtagtaggcat

tgcgtgctcatggcccaataaccaactcggatgtgctactttgttgtctattatagggaa

gtaattttattcgaccattgtttaacactgttttatttggccattgtctactaaacgtat

attattttatggtaaaaaaccgacgatgaaccgatgaaccggcggtccaactgatgaaaa

cctgaaccgacaggctcacaggttcgatcttcggttcagttttttaaactatggccacaa

gtatatctcagaaaaagaaattggagcagggaaaaacatgatttgtggttttgctatcca

tggagtgggacgaatttacaggaactttgccaccgccaagtcacttaccatttcgcttga

atatactcggccctaaacgtttctcccctcaaatatgagtgcacccgtcatgctcacgcg

gccgctcacctcagctacagccgcttacgccgctgctctcttcacctccaaccactcttg

aaatagtctttcgccctgctttatagataaagcagccaccatttccacacaagtagttaa

agtgcaggaaagtaaatagaaggtctgctggggcacaaataagcccaaaagcaaaaaaga

aaaggcgacacaagactccgcagagttaaaagatcaactgacagggggcgaagcgagttg

gcggactgccaccagaagatcatcaatcatgccgtctagccggtccctgtcccgctgcct

acaaagcgggtaccactgccgcaagaaggccaaaattttaaacacagagtcagaagcaca

ctgcaagaggactcacttgataaccatcttgtcacgtacagtccattgggtccaggtaat

cactgcgaacactaacctgaagaggcgcttcctcctaccggtatgtttggcctgagcctc

caggaactcgctcaggtccggcgcctgccactcaagcgctagagcctcacggacaaagtt

ccataaggaggcagcgggacgagagaagaaaatgtgggtcctggtctcctggatttggaa

accacacaaagaaggctaaggccatcacccagcccatgccgcttcgccacctccaaaccc

gatgggaggcgattacagagcagctgccaaacaaagattttgatcttcaggcagtggagc

ttttcacaagtgggaggtccacacaagggtcgacacacaatagagggcacggtatgccaa

cccggcagagaagagtgctgaggaggtgactgtgccaagacacgacgcccggcgacctaa

acagcgatgatggaagagccgtccgcaactcggtctaggctaaggtttcctttggccgaa

tgttcgttggaacggtatgtcccaattcccattgtgggccgccgaggctaccaagagcac

cagatccgagcaaatcatgaaatggtccgggaatttggcacgcaacatggtcctaccgag

ccacaggtcgagccaaaagagggtaccctctccatcgcctatgaagaattgatacccaag

tgaatctcatgcttgatcgactggagggatttcaagaactgagatccttcccaacggtca

caagccaggagagggtggccctgcaggtgtttggcttttataagctgcagccataggcca

ccctcgtccccatggatccgccacacccacctcagcatgagtaccacatttgaactgctc

ccaggggcagcagaagtgtgctcgctgttgatgtataaatgtattgcctagtctcttcca

tcagattggtcttttggttgcattgactagagcatgcacttctacatggtattagagcca

agaggtcttgagttcaagaccggctggcgcaattaaattgcaggccactttcggtccacg

tttaggcctgagggagccacacgtgagggggagtgttgacatatgaatgtattgcctagt

ctcttccatcagatcggtgttttggttgcattgggtagagcatgcacttctacactcccc

acggtggagcacatccaaaccggaaaagagagaagaaaaaaaaggaggtgtgctcctgca

cgaaccacaggtctgctgctcccgcgctggagcacattggaacccgaaaggaaaaaatga

aggtgtgcttccatcgcgggtggagcacatgaagttgtgaaccttaggttcatttcagat

ATGGCTG

gtaaatttggttacacttgaagttgtgaaccttaggttcattgatctatgtgtagtaaaa

gatcaagtactttctgaaaaaagaatgtaggcctcctcatctaagagtattaccttttga

taccgaaatgctgtttcctgactacaggcaaaccgcgtactcatttgatattgaatatgt

gttaggttcattgatctatgtgtagtaaaagatcaagtactctctgaaaaaagaatgtag

gcctcctcatctaagagtataaccttttgataccgaaatgctgtttcctgactacaggca

aaccacgtactcatttgatattgaatatgtgatctgcctttgataccctttgttttgaag

GAAGGGTATTTCTTTTACGTATATTGGTACACAAAG

gtaagttccttgcttgttgcttgaatcataggcagatgtctgagtgcatgtttgctctaa

tacattagccagagatatatagtttggtagcgtttgttaagtcaactactcatctacgtg

acttacggccattgttatgtttattgcag

ATGCTTGCTGTACATTACTACCTGATCTTTGGCGTCACATGAAGTCATTCTATGGACAAG

ATTCTACTTTCAG

gtagaggaatatcttgggctacgtttctctcgtagtaaccaatcccttactgtattttcc

agttgtccaaaataccttagcttccactgttgcttggatcacatgtctttattatgcttg

gctgcggaactataattgaactttctttcctgttattttacatgaactgtgcagacttat

tgacatgatatttttattctag

TTTCTCAGGGGAAACACATGTCGGTAAGAGCGACATATTCGATACTTTGTCAAGAATAAG

GGAAATGCTAAAAGGTAACAGCCGTTGCGAGAAGATTGGCCGTGTGATGGAGAAGGTTTA

CTGTCGTGCGCATGGGGAAGAAGGGATGAGTGTCCGAGTTTCTGGATCATTCATTATGGG

GAACGAGTTCCTTGTGTGCGCAGATGGGTTTTGCGCTGAAGGGATGCTAAGCATGGTCGA

ACTCTCTCCCGACATTCTCAGCAAGCAGGCAGGCCATTTCCAGGAAGATTTTTTCATGGA

GCCAGGGACTGCCATGGGATGCTATGTGATATCAAAGCAAGAATTGCACATCGGCGTATC

ATGA

**>TuMKK4 TRIUR3_14235.T1.X**

ATGGCCACCAAAGGG

gtaataaacacccacttaggtttagagctaggctttactaagatgatacagtttacatac

tatgagaacgctatcagatcaaggattagcgcaaattgaatgtgagttgcgatgggagtg

tgaggccaaagaaattgagggcggagaagggcattgaaatagaagaagaactatatttag

ttttaacccaacaaattaaccaagagatgcataatggttttgtaaagattttaggacctc

aagtttatacagaaatatttatatcaaagagttgaatccatcctctatttcctaaaagta

cttttcactctaaaatataattctttctcttaaaaaacaatatatgagttcgtgaggagt

tggtttaccatgttatctctttatctacaactacttttaaccacacataatttgtaagtt

ggcactagttaagttgtatctaagcacatatgtaaaaaagctagcccacagttcatagtt

cgctttcaaagtcattttttttcatcaaaccactaattccttgtagctagttattccttt

cagcttgttattcagcactaaaaaaccaactcctccccatgaccgtcttgtccatccatg

aaagtttctctggctattggattatttgggtacgcccgttgtctcgaagggaggtggctc

cccgcgagcccgagtataaatttttaaatcaacgtcttcaatggtaaacgatctctatcc

ccacatgtactcacacacttgagttaatgatgctcgacaaaacaaattcccagaaatggt

ctatacaaggacacgtagctatgtgacgaaacctattccaagaacggtcgatgcaagcac

acatatctgagtgattacgaaaatgatttttatagtggaaatcgtaaaagtcaacatgca

gatgggaattttgtggatctcgaaacggccgatcttcctgttctcaacgctgccagcaag

cttgtgggctacatcccagcaacggttgatacaagcgcacatatcggagtaattagaaaa

ccagtcttgagataaaaaattgtaaagggagacatgtataccgatgttcctattcaccac

accaccatcaagctcaccgacgcaaaaccccagttgcctgtcggcggtggaaaccctaac

tccttcccgtgaccgtcttgtctatctatgacaatttcttgcgctagtggactattttgg

gaggattgttgttgggtaggaaggtggccgattgcgagctcgggtagaatttcctaaaag

cagcggctcccgtcgtaaaacgacatggcgcttcacgtgtacttcatgcactcgagttaa

tgatgcgcgacaaaacaaattaacggttcagacgatcacacgtaagagagttaatgtcac

gcgacgaaacaaattcccggtaacgatcgatacaagcgcacgtacgggaataatgacaca

aacgcttcacaatgaaaatcgtaaatgcgggcatgcacatggcaattccgttgatcctaa

aacggaaatttaaaccccgtcgatgggatcttaaaaagtcgatgttctcgcacggcaatt

ttacaggataaccgtaggctcatttcgtttcacagaaatacaatacagacgcagacactc

actagtttggaatatatataggagtataaatttccccgttgttacgtgtcacgtggtaga

gatgtggcgtgatgagtgctgcaatgtttggactgtgccccaagtgaatgctgttccgga

attgcagagtaacggaggcgacggccagccgggaggcaggatgcaacggacacggcaaac

cagcggaacggaatggaacggaacatcaccggtgagcgagaaaggggggagaaaagcgct

gtgggaaagttggttcttttattaccgggcctgcctgcctgccgttcccccggcgtcacg

gctgcatctccatcggctgagcgagcagagctccctccctccgtctctctcccggtccct

tggcctccctcgcggctataaaaaatactcgcctccctccaatccctcttccccgacccg

atcnnnnnnnnnnnnnnnnnnnnnnnnnnnnnnnnnnnnnnnnnnnnnnnnnnnnnnnnn

nnnnnnnnnnnnnnnnnnnnnnnnnnnnnnnnnnnnnnnnnnnnnnnnnnnnnnnnnnnn

nnnnnnnnnnnnnnnnnnnnnnnnnnnnnnnnnnnnnnnnnnnnnnnnnnnnnnnnnnnn

nnnnnnnnnnnnnnnnnnnnnnnnnnnnnnnnnnnnnnnnnnnnnnnnnnnnnnnnnnnn

nnnnnnnnnnnnnnnnnnnnnnnnnnnnnnnnnnnnnnnnnnnnnnnnnnnnnnnnnnnn

nnnnnnnnnnnnnnnnnnnnnnnnnnnnnnnnnnnnnnnnnnnnnnnnnnnnnnnnnnnn

nnnnnnnnnnnnnnnnnnnnnnnnnnnnnnnnnnnnnnnnnnnnnnnnnncggcggcaga

tcacgcgggagatcgccatcctgcgcacggcggagcacccgtccatcgtgcgctgccacg

gcatgtacgagcaggccggcgagctgcagatcctgctcgagtacatggacggcggggcgc

tggaccgccggcnnnnnnnnnnnnnnnnnnnnnnnnnnnnnnnnnnnnnnnnnnnnnnnn

nnnnnnnnnnnnnnnnnnnnnnnnnnnnnnnnnnnnnnnnnnnnnnnnnnnnnnnnnnnn

nnnnnnnnggacgtggcgcggcag

ATGCTGTCGGGGATCGCCTACCTCCACCGGCGCCACATCGTGCACCGCGACATCAAGCCG

TCCAACCTGCTCATCGACTGCGGGCGGCGCGTGAAGATCGCCGACTTCGGGGTGGGGCGC

ATCCTGAACCAGACCATGGACCCCTGCAACTCCTCCGTGGGCACCATCGCGTACATGAGC

CCCGAGCGCATCAACACCGACCTCAACGACGGCAACTACAACGGCTACGCCGGCGACATC

TGGAGCTTCGGCCTCAGCATCCTCGAGTTCTACCTGGGCCGCTTCCCGCTCGGGGAGAAC

CTGGGGAAGCAGGGCGACTGGGCGGCCCTCATGTGCGCCATCTGCTACTCCGAGTCGCCG

GCGGCGCCGCCCCCCGCGTCCCCGGAGCTGCGGAGCTTCATCAGCTGCTGCCTCCAGAAA

CACCCGGCGAAGCGGCCGTCCGCGGCGCAGCTGCTGCAGCACCGGTTCATCGCCTCGCCG

CCGCCGCAGCAGCCGCAGGCCCTCGCCGCCCCGCCGTGCTGA

**>TuMKK5 : TRIUR3_05471.T1.X**

ATGGCGCTCGCTGCAGGTAGAAAC

gtgagcttttttcccttctggacggaaaggaacgtgatctaatctggcgcgagagacagt

gacgcgcaacgcatacattttccccgctttttctccgagcccaagaagcagcggcggccc

ttccgtgttccgtccgacgcgcgcagcacgcttccggcttgctgtcctctcccactccca

ccctcggtacaggacaggtcgcctcccccaacaaacccgatcccccatcaaacagagaga

cggcgcgcaaccagacaaatccccgctnnnnnnnnnnnnnnnnnnnnn

nnnnnnnnnnnn

nnnnnnnnnnnnnnnnnnnnnnnnnnnnnnnnnnnnnnnnnnnnnnnnnnnnnnnnnnnn

nnnnnnnnnnnnnnnnnnnnnnnnnnnnnnnnnnnnnnnnnnnnnnnnnnnnnnnnnnnn

nnnnnnnnnnnnnnnnnnnnnnnnnnnnnnnnnnnnnnnnnnnnnnnnnnnnnnnnnnnn

nnnnnnnnnnnnnnnnnnnnnnnnnnnnnnnnnnnnnnnnnnnnnnnnnnnnnnnnnnnn

nnnnnnnnnnnnnnnnnnnnnnnnnnnnnnnnnnnnnnnnnnnnnnnnnnnnnnnnnnnn

nnnnnnnnnnnnnnnnnnnnnnnnnnnnnnnnnnnnnnnnnnnnnnnnnnnnnnnnnnnn

nnnnnnnnnnnnnnnnnnnnnnnnnnnnnnnnnnnnnnnnnnnnnnnnnnnnnnnnnnnn

nnnnnnnnnnnnnnnnnnnnnnnnnnnnnnnnnnnnnnnnnnnnnnnnnnnnnnnnnnnn

nnnn

tacccgacgag

CCCTTCCTGGCGCACGTGGCGCGCCAGGTGCTCTCCGGCATCGCTTACCTCCACCGCCGC

CACATCGTGCACCGCGACATCAAGCCCTCCAACCTGCTCATCGACTCGGCGCGCCGCGTC

AAGATCGCCGACTTCGGGGTGGGCCGCATCCTGAACCAGACCATGGACCCCTGCAACTCC

TCCGTCGGCACCATCGCCTACATGAGCCCCGAGCGCATCAACACCGACATCAACGACGGC

GCCTACGACGGCTACGCCGGCGACATCTGGAGCTTCGGCCTCAGCATCCTCGAGTTCTAC

CTCGGAAG

gttccccttcggcgagaacctcggnnnnnnnnnnnnnnnnnnnnnnnnnnnnnnnnnnnn

nnnnnnnnnnnnnnnnnnnnnnnnnnnnnnnnnn

tcatcatga

ccgccgcgacctcccaa

cacggatctcgatccacttcttgcaccaccgcgcttccaaatccagcatcaggtggtcag

gatcgatggaactgggctgccgggggaatcttggaaggttctcccagaccatttcgggta

ttcttctcgtccaccatcgtcctccctcatcaaatcatccattgttgctgctgctgctcc

ggttaaggtttcctttccctctcctgtgaatctgtgatgttcgttgctacttcctgttac

ggccttgtaatagtagcaaaagctgtgtgcacaaaaaggattttgtaccgcaagattggt

cgaatatagacacgatatccattctgccattgcctcccgccgctgcaaacgaaattccgt

tggaaatttcatctgtaaatgtagctcatcacatgatgaatatggcccgcccccatcttg

ccagggacatcttccatttattttatctatgtttgcttattatactggttgcaagtcgcc

gtggctcgatggaattattggattggattctataatctacatatgctgggtggatagagg

ggaatcttgaaatggaaatcggaccagctttgttttgtttctttctttcag

TGTTGTAAAGTGA

**>TuMKK6 : TRIUR3_04435.T1 (add stop codon from downstream sequence)**

ATGAGGGGGAAGAAGCCGCTCAAGGAGCTCTCCGTGCCGGCGCAGGAGACGCCCGTCGAT

AAGTTCCT

gtaggt

ctcccccattcctcaccctaaatttcatgttttttttccctctcccctttcccc

cactgaagccgtcaaaagtttgagctttatttctacgtaataatgagagatttttgtggg

gcctgggtagacttgttttctgtgtcatctctgatttgatctgttggttttgttgcgggt

tgtgatggagctgggtagccagtagtagtttgctgaagcatagatttactttgatggcat

tcattaagtgtagtgcttgatttggaattggtacggtcggagctgctgagctgactctgc

attttgtatgtcacgaaagtattcaagtccagtgattaatttgaactcaatatgggttga

gttgctgagctgcttgtggtctttcag

GACGGCGAGCGGTACGTTCAAGGACGGTGAACTGCGACTTAATCAAAGAGGTTTGCAGCT

TATCTCCGAGGAAAACGGAGATGAACAT

gtgagttcttggctcaatattttgtaatgactaactctgttgtttgttaatctgcccagt

tgtgtgttcctgttgtattgtactcgtattcattaacaagggtggtttatgtgttgcgtg

cgtcactgattcagtgcactcagagggtctactgcattcaatcaacctgggggaaccaac

aaaacatttgataagatttgtgattgaatagtatatgtactactatataaacgtacacgt

tgccacacaaagtacaatataacctgaatacagtaagacataccgaaaaaatagtcatca

ctgaacctggaactcacgtagtcatcgctcttttcctcggagataagccacgagcctctt

tgatcaagtctaaaagaccaccagaaaccaaagacaaccatttgattcacacattacttc

ttgtttcttcaagtatctcaaaaacatttctagacacaaaaaagtagtgcagtaaaatat

caagccctcagtagcttcaacaggaaaaaggaaaacaaaacatgctatataaataaaatg

ggcagtttaagccaaggagtcaagaaaacctacatggtttactgacaggtgtcgctccgg

cagaatgagctggagctccttgagcggttccttccccctcatggtctcttcctatggtta

ggatttcttgaatgggagatgaatatggaactggctgccagagcctgaaaacaagtcgag

gagtaggtaggagaccaggtgaactgctatgaagatatggagttcggatctgcccagcgg

aggtgcgcaagagggagagggagagagagagtgctcgcatttgttggtgacttaggcagt

cagctgtcagcctaagtcgcagcatgtgttactttcatcgaccaaagttacaaatttatg

gaccgcagggtcagaggaacccttctagttcaaactttgtagtcgttttatccaaaatca

taacatatttgcagatttctcaataattgtttcatgattattttttaatgcctaggattg

aaattatgtactctgctgctcgctgctgcttattagatgccatcaacagaagttttatag

ttagtgggaatagggtatgttgtctaaaggttactgtagaatcccaactaggatatattg

ttactcagtttgtgcttgttatcatttggataaggatcaagttgtttctgaattctgagt

acatgttgatttgtttattttcctcag

CAATCAACAAAAATGAAGGTGGAAGATGTGCAGTTATCAATGGATGACCTCGAGATGATT

CAGGTCATTGGTAAGGGAAGTGGTGGTGTTGTCCAACTAGTGCGGCACAAGTGGGTGGGC

ACATTTTATGCCTTGAAG

gtaacagaacactaaatttatgtatgcctaagttggatttttattgttactgttagagca

ttccggttaactgctactccctccgtcccataatataagagcgtttttgacactagtgta

gtgtcaaaagcgctcttatattatgggacggagggagtagttttttctgttatactgttt

atttttttattcaagtttgttaacatgaaaaataatctattcatatctgtctgaacatat

ttaaattctacgtgtcacgacaattggattattctacctctgaagattagttttgtttag

tcagcttgtgatggcatgacagtatattcagttagatgcacgcttacgcacactattaaa

atgagttatagcaaaaggactgatatttggcacacgtgtgccatataagcaaaactgcca

cacctctacttctttcattttaaagtaccacacgatcccccgtcctttgacctcgcctcc

tctgaaatgaccccggaggctcgtccgagaaacctgtttctcccgcacatctcgcgcgcc

cacccccgagaaaactgtcacttcttcacgtctaccacatgatttctcctcaggacaaag

ttaccatgacattcatatgcaagttatcaggcctgtgttgcgtaacttaccatactattt

acatagaacgtaccaggtactatgcttcaacaactacaccccttcaaagtcaccactgta

ttttatacacaagttagaaggttttcgacgtgtaaaataccgtggtacttacacataagt

tatcagggtatatgtacatcaacctccccctggtcaaagttaccatgggggattgtacat

gagttaccgggtctatagtttgtatattatcatgttacttgcacctaaaaacctgggtgt

gtttcggtagcatttcccataggtcaaaaattaccatgatgtttttgcgtaacttaccac

gcctatagcacgtgtgcccctcatgacactaaacattatgtcactttctcgtggcacgtc

atgtgttgtgttcatccaagaggcccacgcgcgaggcgagtgtatgtctttaacaactta

tttttctttggtaaaaaaagttaccatcatgtttatattgcaagttatcggttatgcggt

gcttatattatcatgttatttacatgaaaattattggggatgttttgaacaactttttcc

tgggagaaaaagttatcatggtgattctaggtaatttatcaagcccgtagtgcttaaaat

atcatgctatttacacaaaatttatcaggtgtatgtttcagcaaattctcccccacgggt

cagaaacttatcatggtgtttagttatcgcagtgcatatattttcagcatatgttttcat

actatttacacataaaaatgccagaggagggaggtatactaccgtgctatttacaagaag

ataccggagtagattttcaaaaaaaaaatccctacggtcaaagttaccatggtgtttcta

cctcagttatcagatgtgcgctgcgtatattaccatctatttacacataaattatcgggt

atcttttcacatttatcttccccaatggttaaagttaccacgatgtttgtatctaaatta

tcaggtctatagcgtgaatgttatcgtgctatttacagaaattaccagggggcgggttca

acaaatttattcccaatgatcaaagttactattaaaggcaaaaatatgtcaaaaaacagt

atttcccttagaatgttcaacaatgagtgtgatagctgaggtggttagccccttctgtta

attacctgcagaccagagatcaaatcctagtctaagcattatttttgctgaaaatcgaga

aggcatgtggggccataaatggcgattacgaaaattaggaaggcacgagcgggtgtgtcc

gataaaatcggggaggacgtgcgggaaaggaaagaaatcggaggaggacgtgagagaaag

gaaagaaattgggaggtcatgcgaaaaagaaaggaaatcgggagagaattgatggcattg

gatgcgtgtggcagtttcgcaatctgtcacacggttgtcatatacatatttcgtataaaa

aatcttaacaaattgaccctgttgataaaaaaattttggccaaaaaccatacaagtaaca

gaaaaaggaaaagaaaatgggcgcccatggcaagcacgacaactagctgacaccataaga

gcagttcttttgagcagattatgaagaatctcggtcccgaaaatcccgagcgaaaagaac

ttgattgaacttccaaaatctcaaccggattctgcagaatcctagtgcaatcacaaaatc

ccaaaatacctggtttcttccacaatctcaggatctggctagtcccacaaaagtaaacgt

ttcgaagtatggctgtacctctattgtatcatgtatttatggtcaaaatgccaccccgca

gtccagccgaggaccccggaaacgagcggacgcacccgattgctcgcccgactcctggtt

cccgcaccgcatcggggccgcagtagaccagagatcaaatcctagtccaagcattatttt

tgcccgaaaattgggaagtcgcgtggggccataattgacgattacgaaaattaggaagtc

acgagcgggtgtgcccgcgggaaaggaaagagatcgggagacgtgcaggaaaggaaagaa

atcgggagggaggtcggggaggacgtgcgggaaaggaagaagatcgggaggcgtgtggca

gtttcgcaatctggcacacggttgccatatacatatttcaatagcaaaaacatctagtac

tggcctgcaaaatctctatggaacactgatttgagaactgacgctaatgcatgagaatac

atcaggtaacaatatatgggcgattaaatgttttcaagcgttgttcctgatctttgtact

gccgtggcatttctccatatgatctatcagttaattgactattgcaatttcttcatag

GGCATTCAAATGAACATTCAGGAGTCAGTTCGCAAACAAATAGTACAGGAGCTCAAAATA

AATCAAGCAACACAGAGCCCACATATAGTCCTTTGCCATCAATCTTTTTACCACAATGGT

GTAATATATCTTGTTCTGGAATATATGGATCGTGGATCTCTTGCAGACATAATTAAACAA

GTTAAAACTATTCTGGAGCCATACCTCGCAGTACTTTGCAAGCAG

gtatgtatatgcttggcctattgtttctgctttgacttttgtattcagaatattcttaaa

attctgtcacatatgttggaagttggagctacaatctggcacatctaacatcagtagtgt

ctgaataaatcag

GTTTTGGAGGGACTGTTATATCTTCATCATGAAAGGCATGTGATTCACAGAGACATAAAG

CCTTCTAACTTGTTAGTCAACCATAAAGGTGAAGTAAAGATTACTGATTTTGGGGTGAGT

GCAGTGCTAGCAAGTTCGATTGGTCAGCGTGATACATTTGTTGGAACCTACAACTATATG

GCG

gtatgtaaaaagaccagttttgtcttctgactgcggcttatgctcataacggttatctgg

ggtgcataaattttccttcatagtttcttcctttcttccttgtgcag

CCTGAGCGGATTAGTGGCAGCTCCTATGACTACAAGAGTGATGTATGGAGTTTGGGCTTG

GTAATACTTGAATGCGCCATTGGTCGCTTCCCTTATACTCCTCCAGAGGGAGAAGGTTGG

TTAAGCTTCTATGAACTATTGGAAGCAATTGTTGACCAGCCACCACCTTCTGCACCTGCA

GATCAGTTCTCTCCAGAATTCTGCTCATTTATCTCTTCCTG

gtatgctaacattattctcaggaattacacatgaaattagttcagtcactgtatttctat

tttgtcctcttgcttagtatgcttgaatgaacttcttgcccttttttctaaagttctgat

ttccttcgcgtgtccttcttgttgtttagtagttatctgctttctcgtctcacagctcat

ggactttgtccactcaattattatccccccctttgataaaatgtgttgtctattcaagta

ttactttcccctttgaacgaacttcttgccctgttttctaaagttctgatttcctccacg

tgtccttcccgtaaatattttaaattgttggttaggccaacatgttttaatagttatctg

ctttctcatctcacagctcatggactttgtccactcaattattattcccccctttaaaaa

aatgtgtttgatctatttaagtattacttccccctttgaacaagcgtggtcttaatagaa

ctgttttagtgtgataatttgggcttcattaattctggagaataactgtttaaactgcgc

ggtgtttctcagataagaattagtgaaatcctcagatgcccaaattttataatggtgctt

tttcaatagcaaacggatgaatcaatgtttcttaatgaatattacatttcagtacagccc

aagaaatcacagccctcccaaacaaatcaacttgcgcccccggacaacaactactcaggg

caacttgccaatgaactgtttatcaaaccctgtttaagtagcttgttggcccttgaaact

tcagtttagctaatctggagtttgagttatcaatcacgtaacaaccctgattgtcagtaa

tgtcaaaatgtacacattgatttaagttagctatgtcaatatctgatctaattgttgcct

tgactaacaaaaccttgacagtggggttgataaaccagttaacaatccggcatcttatct

taccaatggattaaggcatgccaaaaaaatatggaaaataaccctcctactatacagggt

gtatgaagttgaccaaatgatgttatactatgaatcctttttatgttgtgtgattcaaaa

ggaagaggaaggaacaccagagaacaataggcatggaaggcatcacaaaaactcaaacca

aagcctcctctagtgttagacgaggaacaagggctttagccttccatcatgttcccatgc

ccactgctcttaggtcatctttgcttcctcttcatgtttcacaatggacatctcatacca

tctgaacatatccacatatatgttcagtttttgtgctaaagatttagtgaacaaaaagga

agaggaaggagcgcttaagaacgataggaatgagaggcatcgggaaataccaaacgggat

ttccacctcaacacaagaggtgaagaaccgataacttaggctttcctctatgctctcatc

cccattgctcttaggccctccctttcttcctcttcctagctcgcttttgagatttcacat

cgaatatgtgcagacatatagttggtttctaaactaaagcaaccaagcaagccaaaaaag

gaagaggaaggagtgctttagaacaataggaatgggaggcattgggaaacaccaaactag

attttcacctctaacactagagatgaaaaccgagcaatttaggctttcctccatgttccc

attcccactactctaaggccctctattcttgctcttccgggctcgcattcgaggtctcat

gttatctaaacatgtggacaatgtctatggtttctccatcagagctatcacgtaagccaa

aaggaacaggaagaagtgcacaagaacggtagaaatgggaggcatcaggaaaggatccat

gccaaatatttgcacctccgatactacaggtgcaaacaaacaagcattgctttcctccat

gttcccattcccattgctcttgagctctctcttcctcttcccggcttactttcagtgcgt

acaaatgtagagacaaggctctcgaaggacaagcttgaagtgttggacaaagtgtttcag

ctttgaggtataaataattggtggtgcaccatatgcagaaccttagaggagaaagcaatg

aagacacccagtgcatggaggcaccattctacagtcaatgataatggggtcacataatca

ccagccctgtcctattgtcctctactcttaattaagggcatgtgcgtaaagatctctttt

gctcacctttattacccatatccgaaactggtacataatatctgtaccagccttttttta

gaacaatatctgtatcatcctgtgcatttatagtgtcaaaatacgcagcaataagaagtt

ataagctaaaaataatatgaaattagacatattaaaccctcgaaaaattacaaaacagtg

ggcaaagaaccaccaacgtgacaattaattggaacacaaggtacttcggacatgagctgc

gttattgcagatgacagaatgtgattacaacatgattagagggatctcatatctgcataa

ccaaaatgtctatagaccattttatcaagaaaaaaagtttagtcctattattttttaagt

aatttagtatccaaattctgcaggctgtacacaactacatgtcatgggaaaatataggaa

aaacatatctccaaaggaactgtcacccttgatccctaggataaaacacatgaaggtata

gtgatcacatattcagtctaaactcattatacacatagtattttaagaacaaggtttgga

cacaaaggaagaacaaggtcccgaagagagtatggtacttcttccacccatcgaagtact

tcccagttcaatcttgtagctctgccaccatctctccaaggattaaatcgcccaggttga

gatgcaaacagcctgcaaggttccagagtggggaaataagccttatcccgcacatctaaa

caatatataaaaaacaacaccaatttatgatgaagtgtacaaaactgcaaattccattgg

agttcacaattttcgtagctgcagataaatccattaggttgctatatcccatgtttgaac

tactgaggaaataataaaaataatctgaatgaacatagtgatattgtctcatgttttgtg

tgccaattaccatctttcgtactccgtttctgctcgtagtcttagctagagtggtcagag

tggtgtgtcattgatctgattaactgatagagtaatgcacttacatgcatttaccatgcg

actctgtctaaactcctcagtcctcacgtcatgcttgacctgcatcaactggctgatatg

taccattcaaatactattacttcagaacttcctttgatttagtgtgataatttgggcttc

attaattctggagaataactgtttaaactgtgcagtgttcttcagataagaattagtgaa

agcctcatatgtccagattttataatggtgtgttttgcaacagcaaacggacgaatcaat

attttttttaaaccataatattacatttcagtacagcccaagaaattccagccctcccaa

acagatcgacttgcgcttccagacaacagctactcagggccacttgccactgaaactacc

tgattatcaaatcctgtttaagtagcttgtgtgcccttgaaacttaaagcagtttagcta

atctggcgtttgagttattaatcatgtaacaaccctaattgtcagtttaatgtcaaaatg

tacttactgatttaagttagctatgtcaatatctgatctaattgttgccttgattgacaa

aaccttgactttgaggttgtagtataaaccagctaacaatccggcatcttatctaaccga

tggattaagtcatgccagaaaaagatggaaaataaccctcctactatgcagggtgtatga

acttgaccaaatcatgttatactatgaatcctttttatgttgtgtatccaaaaggaagag

gaaggggcaccaaagaacaataggcatggaaggcatcagaaaaactcaaaccaaagcctc

ctctagtgttagacggggaacaagggctttagccttccatcatgttcccatgcccactgc

tcttaagtcaccttttcttcctcttcatgtctcgcaatagacatctcatactatctcaat

atatccacatatacgttcagtttttctgctaaaagattcagcaaacaaaaaggaagagga

aggagcgcttaagaacgataggaatgagaggcatcgggaaaataccaaacgggatttcca

cctcaacacaagaggtgaagaaccgataacttaggctttcctctatgctctcatccccat

tgctcttaggccctccctttcttcctcttcctagctcgcttttgagatttcacatcgaat

atgtgcaaacatatagttggtttctaaactaaagcaaccaagcaagccaaaaaaggaaga

ggaaggagtgctttagaacaataggaatgggaggcattgggaaacaccaaactagatttt

tcacctccaacactagaggtgaaaaccgagcaatttaggctttcctccatgttcccattc

ccactactctaaggccctctattcttcctcttccgggctcgcattcgaggtctcatgtta

tctaaacatgtggacacatgtctatggtttctccatcagagctatcatgtaagccaaaag

gaacaggaagaagtgcacaagaacggtaggaatgggaggcatcaggaaaggatccatgcc

aaatatttgcacctccgatactacaggtgcaaacaaacaagcattgctttcctccatgtt

cccattcccattgctcttgagctctctcttcctcttccgggcttactttcagtgcgtaca

aatctagcgagacaaggctctcgaaggacaagcttgaattgttggacaaagtgtttcagc

tttgaggtataaataattggtggtgcaccatatgcagaaccttagaggagaaagcaatga

agacacccagtgcatggaggcaccattctacagtcaatgataatggggtcacataatcac

cagccctgtcctattgtcctctactcttaattaagggcatgtgcgtaaagatctcttttg

ctcacctttattacccatatccgaaactggtacataatatctgtaccagcctttttttag

aacaatatctgtatcatcctgtgcatttatagtgtcaaaatacgcagcaataagaagtta

taagctaaaaataatatgaaattagacatattaaaccctcgaaaaattacaaaacagtgg

gcaaagaaccaccaacgtgacaattaattggaacacaaggtacttcggacatgagctgcg

ttattgcagatgacagaatgtgattacaacatgattagagggatctcatatctgcataac

caaaatgtctatagaccattttatcaagaaaaaaagtttagtcctattattttttaagta

atttagtatccaaattctgcaggctgtacacaactacatgtcatgggaaaatataggaaa

aacatatctccaaaggaactgtcacccttgatccctacgaggaaaacacatgaaggtata

gtgatcacatattcagtctaaactcatttatacacagagtattttaagaataaggttcgg

acacaaaggaagaacaaggtcctgaagagagcatggtacttcttccacccatcaaagtac

ttcccagttcaaccttgtagctcggccaccatccctccaaggattaaatcgcccaggttg

agatgcaaacagactgcaaggttccagagtggggaaataagccttatcctgcacattaaa

caatatataaaaaacaacaccaatttatgatgaattgtacaaaactgcaaattccatcgg

agttcacaattttcgaagctgcagatatatcctgttgcggtaatggttgtttcttgctag

tctgtgtattttatccattagtttgctatatcccatgtgtgaactatttaggaaatgata

aaaataatctgaatgaacatattgatattgcttcatgttttgtgtgccaattaccatctt

ttgcactccgatttgtttcatgttttgtgtgccaattaccatcttttgcactccgatttc

tgctcgtaggcttagctagagtggtcagagtggtgtgtcattgatgtgattaactgataa

gagtaatacgcttgcatgcatttaccatgcgactctgtctaaactcctcagtcctcacat

aatgcttgacctgcatcaattggctgatatctaccattcaaatactattacttcagaact

tcctttgatttagtgtgataatttgggcttcattaattctggagaataactgtttaaact

gtgcaatgttcttcagataagaattagtgaaatcctcagatgtccagcttttataatggt

gtgttttgcaacagcaaacggacgaatcagtgtttttttcatgatattacatttcagtgc

atcccaagaaattccagccctcacaaatggatcgactttcggcaacttgccactgaaact

acctgtttaccaaaccctgtttaagtagcttgtgggcccttgaaacttcaagcagtttag

ctaatctggtgtttgagttattaatcatgtaacaaccctaattttcagtttaatgtcaaa

atctacttcctgatttaagttagctatgtcaatatctgatctaattgttgccttgattga

caaacccttgactttgaggctgtagtataaaccagctaacaatccggcatcttatctaac

cgatggattaaggcatgccaaaaaaacatggaaaataaccctcctactatgcagggtgta

tgaacttgaccaaatcagttatactatgaatcctttttaggttgtgtatcccatgaagag

gaaggggcaccaaagaacaataggcatggaaggcatcacaaaaactcaaaccaaagcctc

ctctagtgttagacgaggaacaagggctttagccttccatcatgttcccatgcccactgc

tcttgggtcatcttttcttcttcttcatgtctcgcaatggacatctcataccatcttaat

atatccacatatacgttcagtttttctgctaaaagattcagcacacaaaaaggaagagga

aggagcgcttaagaacgataggaatgagaggcgtcgggaaaataccaaacgggatttcca

cctcagcacaagaggtgaagaaccgataacttaggctttcctctatattctcatccccat

tgctcttaggccctctctttcttcctcttcctagctcgcttttgagatttcacatcgaat

atatgcaaacgaatagttggtttctaagctaaagcaatcaagcaagccaaaaaggaagag

gaaggagtgctttagaacaataggaatgggaggcattgggaaacaccaaactaaattctc

acctccaacactggaggtgaaaaccgagcaagttaggctttcctccatgttcccattccc

actactctaaggccctctattcttcctcttctgggcttgccattgaggtctcatgttatc

taaacatgtggacacatgtctatggtttctccatcagagctattgcgaaagccaaaagga

agaggaagaagtgcacaagaacggtaggaatgggaggcatcaggaaaggatccatgccca

atatttgcacctccaacactgcaggtgcaaacaaacaagcagggctttcctccatgttcc

cattcccagtgctcttgagctcactcttcctcttcccggcttactttcagtgtctacaaa

tatagcgatacaaaagctctcgaagtacaagcttgaattgttggacaaagtgcttgagct

ttgaggtataaataatgggtggtgcaccgtatgcagagaaagtaattaagacacccggtg

catggcggcaacattctacagtcaatgataatggggccacataatcacctgccctgtcct

tttgtcctctactcttaatttaaggcatgtgcgtaaagatttcctccgcttacctttatt

acgcatatccgaaactggtacgtaatatctgtaccgatctgtatcagcctgtgtatttgt

agtgtcaaaatatgtagcaataagaagttgtgagctaaaagtaatatgagattgaacaaa

ttaaaccctctaagtaaacttggaaaaaatacaatacagtgggcgaagaaccaccaaagt

gacaattaattggaaaaaaaaggtacttcggacatcggctgcagtattgcagatgacaga

atgtgattaaaacatgataagagagatctcatatctccaaggattaaatcacccaggcag

agatgcaaacagccagcaaggttccatagtggggaaataaaccttttcctgcacatctaa

acaatatatagaagacaacaccattttatgatcaagtgtacaaattcaattagagttcac

aattttcgaagctgcagatatatatcttgttgcagtcatgcttgtgtcttgctagcctgc

gtatttatccattagttttctatcccatgtgtgaactctttaggaattgatgataaaaat

aatctgaatgcacatatcgttacatgttttgtgtgccaattaccatcttttgcactccgg

tttctgctcgtactcttagctacagtggtcagagtggtgtgtcattgatgtgattcacta

atactgtaatacactttcatgcatttaccatgcaactctgtctaaactcctcagtccgca

catcatgcttgacctgcagcaattggctcatatctaccatttgaatactctcttttttca

agcaaccggcaagagctcttccttttcatatagaggagaattgaccggtttataaggaaa

accgtccgagaaccaaccattcgaatactattactttagaacttcctttgatgtacagtc

aactccgtttattggctatatgttttattggtctttattttagttagcgagcattcaaat

gaaatctgcacatgttgaaggcaactgttgatttctgctagcttagctttatagatttgt

gccgtctgtgtagtgttatttcataccgctatcttgctttgcag

CATACAAAAGGATCCTGCCGAGCGGATGTCTGCTTCAGAACTCTTG

gtgagcattcccttccacttgacaacttgttgtagcatgttcaactaaccgtggcaccat

ttgattctgccgactagtgtgtgtgtgctgaatcgtcgttatgttctgtgttgttgtcgc

ag

AACCACGCCTTCATCAAGAAGTTTGAGGACAAGGACCTAGACCTCCGGATCCTTGTCGAG

AGCCTGGAGCAGCCCATGAACGTGCCCGAG

tgaggccgtcgctcgtagaggtgaggctttgtggcgactttcttccaggg..........

**>TuMKK10-1/3 TRIUR3_19643-T1.X**

ATGAAGATGAGAACCATGACCAAGCTAATCTCGGGCCAAAATG

gtgtgctaagcggtcgtgcgtgatggccgacgttggcacgggccgcatcatttcttcaag

cttgcgctaaacctgaaccagatatgatctgctgactccatgcatggtccaacaacaccc

gcacccgtcagcatccatctcactctctagtcttatctatcatgcatcttttttattttt

gtgttgtgttcattctctcgttcagccggtctacatcttaggactagtaaatatctcctc

cgctgccctgggagaatatcttctgaattagggctatgttttcccactaaccttgccaat

taagaggtatgtacccacgtcacctcgaattaaagatgacagtgttcgttagcgacggcg

gtccttaagcccccaaatgcgaggcttattggcagagtggataactatctagccccactt

gacacttgtttacaaattagtcgggcccattttaaatttggcacgcatgatagaattata

aaggtatctagcttacatgaattattgtttgtgttgtcctcccactaaccagtcctcttg

tgtactttcctgcaattgaactacttgtgtggatgtggtaaagcggaccgaaaacctaat

attgagagtacaacacacatataacaaagtgaaacgaacataaacatacaaaaccaaatg

gtgtgcacgtccactaaatagattatacttgtggtagctaccatgctacgtacacactag

caaatttaactttcatgttaccagaagagggcatcatgttctgaatactagcttagttta

ctatttacataagaaattatgagaaactaattgtgaaacatgactttcgtaactcaattt

atggacttgaaagaccttttttggaatttttttgacattatttgtagacatatgtatatt

acattgatgtagtatatatcttttagaaatgttgtgttccctccgatccaaattacttgt

cacagctctagtaatatggatcagagacagtaactcgatttatggacttaaggtattcta

ttctaatctatcatgcatctttttgtttgtgttgtgttcattcccgcgctcaaccagtct

gcatcttaggactagtaaatatctcctctgctaccagaagaatatctcctgaattaggca

tatgttttcacactaagcttaccaattaagaggtacatatccacatcacctgaaattaaa

gatgacaatgttcgttagcgacggcggtcctttcaacccccaagtgtttgggattaatat

caagatcaggcttagcatagtggataattatataaccccacttgacacttcttcaaaaat

tagtcagactagtttaacagttttaacacatgaaaaaattagtcataatacttgaagtat

agtagaacagacacatataataaaggaaaaaacataaacatacaaagcaaaatctaaaga

ataacataatacacgactcgtggtagctaccatgctacaaacacactagcaaatctgatc

taagtgatattggaaagcacatcatgttctgaatagtaccttacttactattcacataag

aaaattttgagatacttatgttggtgaaatttgcctttcttaactcggttagtggacttg

aattatgtttggaactttgtgacagttgacactgtagatagacgtacattatgttgatgt

agtgcatatattttagcactttttttttgtataacacttcgctagtctaacaatgtattg

ataacacggtcatgaaaataaaaggagaaggtggaggtgtggtggcggaagctggagaat

gacgaggcgacagtagcggcaattgcagttagggtatagcatagcgaagtgaagattgat

ctcaagacatgtgaatgaagatctaacaactaggggaagacaaaccaagttccactgcct

aggccaaatacatacatcaaaatcataattccaaaagcattggtgtattattatacaacg

gtattaagcttacaagtgtatcttaccagccatcagcgattctcggccggaaacggaatg

gaggaaaccggtgtaaacattacatgcccaaaacggcgacccaacccaagcatctggaac

aaacccaaccgtctattttttaccaagcaatgttttacgggggtatttcgaaggaaagct

ttggcataaatctccttaagtttcagactcaataacatcatgaatatccattcttgcaca

caagataaataagcagattttcacatcgttttcaaaagaaaggtttaaaaagattcggaa

gttagacatcaaaatctcagcacagtcagccaaattaacttgcgccgccaggtcgtagta

taaagctaacctcacctccacacaaaaacatcatccgttactcgatcatcaatttcatca

ttctattgccaatacagttcttgcgaaacacacacacatacacacggcgactcaagatca

tggcggctgccagagaaagacgactgccgcagnnnnnnnnnnnnnnnnnnnnnnnnnnnn

nnnnnnnnnnnnnnnnnnnnnnnnnnnnnnnnnnnnnnnnnnnnnnnnnnnnnnnnnnnn

nnnnnnnnnnnnnnnnnnnnnnnnnnnnnnnnnnnnnnnnnnnnnnnnnnnnnnnnnnnn

nnnnnnnnnnnnnnnnnnnnnnnnnnnnnnnnnnnnnnnnnnnnnnnnnnnnnnnnnnnn

nnnnnnnnnnnnnnnnnnnnnnnnnnnnnnnnnnnnnnnnnnnnnnnnnnnnnnnnnnnn

nnnnnnnnnnnnnnnnnnnnnnnnnnnnnnnnnnnnnnnnnnnnnnnnnnnnnnnnnnnn

nnnnnnnnnnnnnnnnnnnnnnnnnnnnnnnnnnnnnnnnnnnnnnnnnnnnnnnnnnnn

nnnnnnnnnnnnnnnnnnnnnnnnnnnnnnnnnnnnnnnnnnnnnnnnnnnnnnnnnnnn

nnnnngggaaggccacgagcgcggactgcgctatag

CCGAGGTCGTCTCCCGCGCCGGCAAATACCGCGCGGCCTACGAGGGCACCGCCGCGTAC

ATGAGCCCCGAGCGCTTCGACACGGAGCGGTCGTTGCAGGGCGACAGCGACGAGGAGGGCC

CCGTCGACCCCTACGCCGCCGACGTGTGGGGGCTGGGGGTGACCGTCCTGGAGCTCCTCG

TGGGGCGCTACCCGCTGCTCCCCGCCGGGCAGGAGCTGACCTGGGCGGCGCTCATGTGCG

CCGTCTGCTTCGGCGAGCTGCCGGCACTTCCCGACGGCGCGGCGTCGCCGGAGCTCCGGA

GTTTCGTGGCCGCTTGCCTGCAGAAGGAACACCGGAAGCGGGCGTCCGTCGGGGAGCTGC

TCGTGCACCCGTTCGTCGCCGGGAGAGAC

gtagcggcgtcgagacgggcgctacgcaaattgatcgagcagcggtgttgatagacacgc

gcgtgtaaatgttgaactgtcacattctcgaatcgtgaatacctaagctaggcatttgca catacagacagtggaccatactttgagcttcgaagggtctcgctccctgagagcatctcc

agcccataccttatacactactggaataaccatatatgcctacggccttatctatgccga

cagctgccgtcggcatagatggagctatgccgacggttcggggaataccgtcggcatagg

ttcatccannnnnnnnnnnnnnnnnnnnnnnnnnnnnnnnnnnnnnnnnnnnnnnnnnnn

nnnnnnnnnnnnnnnnnnnnnnnnnnnnnnnnnnnnnnnnnnnnnnnnnnnnnnnnnnnn

nnnnnnnnnnnnnnnnnnnnnnnnnnnnnnnnnnnnnnnnnnnnnnnnnnnnnnnnnnnn

nnnnnnnnnnnnnnnnnnnnnnnnnnnnnnnnnnnnnnnnnnnnncccgccgcgtcccga

aggcatagcctcgccacatggcccctcctggtagcccctggccgagaacaatgcctacgg

ccgtaggcatagatatcatctttttttattttaaattatttttatgtttttaaaagttaa

aatttgaataatttaaatctaacttatctaaacttaaacatacacaaattatatatcgac

ttggggtaaaattttccatagattatgaatatacagtttgtttttgtttttgagtatgtt

agaaatgtgacctcgtgtacttttgcatataggtccttatagtttgttaaaatcataagt

aattgatagttggatcaattttgacaaattttatatggtttttcatcagaatcttgaaag

tagggatggcagttttgcccatgggtatgggtacccgtgggcaccctacccgaaaacaat

gggtatgggcaagacttgaagagattgtctacccacgggtaatgggtacccatacccgca

aaatatatgggtagggcatgggtaagaaattgtgcccacgggtaacccaatggataccca

acaaaaattaataaatgaaaataactcctataacatgtgggttaacatccaactcatatg

gtccaaccctaaatctcgtattccttaaacaagtcatagctcatagactcataatcacct

gctcgccactcctcaaacgtcctatgtgactccttaaaagatgaaatatcgactcttagc

tactagactcttgtcgaacactcgatccagtgatccccaattcccaccaccgccttccac

tacgtcggccttccaccaaccgccgctcatactcccctaatgcctccaagaggccaccca

ctggaggaggcaacatacgtgttatactactctaccatgatcaattgaattttgaactca

tttctctacctcataataatttgactgctatatattgtacccaatggatacccattgggt

ataggatacctgatgggtatgggcatgggtgtcagtttatacccatgggtattgaagtgg

gtgggtataaaaagttcctatgggtatgggtttgggtagaaggaggttgcacccgcccat

accctacccattgccatccctacttgaaaggattctgttgctatactatttttaaaattt

gaacaaacttaaatcatgtttgcttcaaatttatagaaaacacccttttgttttgaattt

gttgtatatttttcattccttggccaattgagacaaattttatatctacatccatcagat

tgtttcaaggattgtggtggccggcgcaaacacccggcaaccggctccggagtgtgactc

cttcatggacggcaatgtcatcgtcacttggagggggaaacggccacgttgggtcgacat

ggagggaatcttatgatgggtttgaaccaaaccttgttctcaagtgttcctatgggctga

cctataaccaggtgaaaacgtccattggtcaaatcccgtctgacggaagtcaaaggcgta

gacctccaggtcaacaagtctagggttaggccggttgggggccttcggggggtagcaatg

ccaccagagcattacgtcgggtcctcatacatgtcttaaggtgtggtggcatgtctgaag

aggcaatacgcggctccggtgtgaggtccccccccctcacggacggcaatgaaatcgaag

taatccctctacggttttggcgttgcatgaatatttctgaacactcagagcgtgatcaat

tcatgaaatttttacacagtgcagacacatgtgatataataggcgtggaaatttcttata

ttttttaaagatgcaagagtatgtgaaaaaatcccccactatggattgttcttcgcgtgt

ctacgagtgtggccaggggccttcgggggctagctatgccaccaaatcttgtgttgggtc

ctcttacatgtctgtcagtgtggtgtcaggtgcaaaacacccgtcatgcggctccggagt

gtgacccccttcacggaaggcgccaccgccggcacttggagggaggaaacggccgcgtcg

ggttgacacgagggggatccggtggtgggttgggcccagtcgggcgccttcgggggtagc

aatgccaccaaaacttgcattgtgtcttaatatatgtatacaagtgtgattaagtgttga

aatagccaatggagcaacgggcagcacacttccttcacaaaaccggacacgttctctctc

gataaccatatactaccttggagatggtgtagtttacaagtggcctcttgtggggcttct

gcgaaacatgctcaaacttttaccacgccctaaaaggaccatatgactacaccatgccaa

gtcccgaggattttttggcatcgtatgatttcccgtggattttaacggcaagatctggca

tgccccgaggtacattcaccctgcggtggtggggcctcccccttctttggttgcactagg

cctacacataccgcaaagacatcatatgtgatttaacaaaccacttctggacatcatttc

ag

GTGCTCGCCAAGTAG

**>TuMKK10-4 TRIUR3_30060-T1.X**

atggcggct

GCCAGAGAAAGGAGGCTACCGCAGCTTCACCTCACGCTCGACGCGCCCACGTGGGCCTTC

CGGTGCCCCGCCTCGGCGCCGGTCACCGCGGCGACGCCGTCCACCTCGGCGGCTCGGCCG

GACGGTGAGTTCCGCCTGATCGACTTCGACAGGCTCTCCGTCCTTGGTCGCGGGAACGGC

GGCACCGTCCACAAGGTCTCGCACCGCCGCACGTCGGCGCTGTACGCGCTCAAGATCATT

CACCGCGGCCACCCCGGCGCCGACGAGGAGGTAGAAGTTGTACGGCGCGTCGACTCGCCG

CACATCGTCCGGTGCCACTCGGTGCTCCCGACGGCGTCCGGCGACTCCGCCTTACTCCTC

GAGCTGATGGACGGCGGTTCGCTCGACTCGCTCGTCCGCGCCGGCCAGGGAGGCTTCCCG

GAGGAGGCCCTTGCGGAGGTGGCCGCGCAGGCGTTGTCTGGCCTAGCATACCTCCGCGCC

CGCCGCGTTGTCCACCGCGACATCAAGCCGGCCAACCTCCTTATCAACAAAGCCGGGCAA

GTCAAGATCGCCGACTTCGGCATAGCCGAGGTCGTCTCCCGCGCCGGCAAATACCGCGCG

GCCTACGAGGGCACCGCCGCGTACATGAGCCCCGAGCGCTTCGACACGGAGCGGTCGTTG

CAGGGCGACGGCGACGAGGAGGGCCCCGTCGACCCCTACGCCGCCGACGTGTGGGGGCTG

GGGGTGACCGTCCTGGAGCTCCTCATGGGGCGGTACCCGCTGCTCCCCGCCGGGCAGGAG

CTGAGCTGGGCGGCGCTCATGTGCGCCGTGTGCTTCGGCGAGCTGCCGGCACTTAGCGAC

GGTGCAGCGTCGCCGGAGCTCCGGAGTTTCGTGGCCGCTTGCCTACAGAAGGACCACCGG

AAGCGAGCTTCCGTCTCAGAGCTGCTCGTGCACCCATTCGTCGCCGGGAGAGACGTAGCG

GCGTCGAGACGGGCGCTAGGCGAAGTGATC

aagcagcggtgctga

**>TuMKK10 TRIUR3_19642-T1.X**

atggcggccgccagggnnnnnnnnnn

nnnnnnnnnnnnnnnnnnnnnnnnnnnnnnnnnnnnnnnnnnnnnnnnnnnnnnnnnnnn

nnnnnnnnnnnnnnnnnnnnnnnnnnnnnnnnnnnnnnnnnnnnnnnnnnnnnnnnnnnn

nnnnnnnnnnnnnnnnnnnnnnnnnnnnnnnnnnnnnnnnnnnnnnnnnnnnnnnnnnnn

nnnnnnnnnnnnnnnnnnnnnnnnnnnnnnnnnnnnnnnnnnnnnnnnnnnnnnnnnnnn

nnnnnnnnnnnnnnnnnnnnnnnnnnnnnnnnnnnnnnnnnnnnnnnnnnnnnnnnnnnn

nnnnnnnnnnnnnnnnnnnnnnnnnnnnnnnnnnnnnnnnnnnnnnnnnnnnnnnnnnnn

nnnnnnnnnnnnnnnnnnnnnnnnnnnnnnnnnnnnnnnnnnnnnnnnnnnnnnnnnnnn

nnnnnnnnnnnnnnnnnnnnnnnnnnnnnnnnnnnnnnnnnnnnnnnnnnnnnnnnnnnn

nnnnnnnnnnnnnnnnnnnnnnnnnnnnnnnnnnnnnncgtgaccatcctagagctcctc

ATGGGGCGCTACCCGCTGCTCCCCGCCGGGCAGGAGCTGACCTGGGCGGCGCTCATGTGC

GCCATCTGCTTCGGTGAGCTGCCAGCACTTCCCGACGGCGCGGCGTCGCCGGAGCTCCGG

AGTTTCGTGTCCGCCTGCCTGCAGAAGGACCACCGGAAGCGGGCATCAGTGGCGGAGCTC

CTCGCGCACCCGTTCGTCGCCGGAAGGGACGTGGCATCGGCGAGACATGCGCTCCGGGAA

GTGATCGCGCAGCGCGTTTAG
