# Supplementary material for: Analysis of MAPK and MAPKK gene families in wheat and related Triticeae species
Source: BMC Genomics. 2018 Mar 5;19:178. doi: 10.1186/s12864-018-4545-9 (PMC5838963; doi:10.1186/s12864-018-4545-9)
Supplement: Supplementary file 6 — Triticale MPK and MKK transcript sequences. Transcripts were identified from an in-house transcript assembly library which has been submitted to GenBank by Laroche et al. TsMPKs and TsMKKs sequences are provided. Blue and red fonts are used to mark putative start and stop codons, respectively. (PDF 152 kb) [file 12864_2018_4545_MOESM6_ESM.pdf]

Additional File 6. Triticale MPK and MKK transcript sequences. Transcripts were identified from an in-house transcript assembly library which has been submitted to GenBank by Laroche et al. *TsMPKs* and *TsMKKs* sequences are provided. Blue and red fonts are used to mark putative start and stop codons, respectively.

>TsMPK3 : contig10429
CTGATTATAAAATCTATTTCTTCGTAGACTAGAGAATGATTTGCTCTCAAATCATACTCCAAATTTTTTATTCGCAT
AGCCAAGGGACAAAGGTCTGAAGAAGCAGCAACAACTTACACGGAACAAACAAACAAATGCAGTGCGCAACCTACCA
TGTTCTTTGATCTTACTCCTATTTACAGACAGTTTTACACCGAAATCATACTATTGGGGGTTGGGCAACTAGGAATG
AATCGT**CTA**GTATCGGAAGTTGGGGTTCAACTCGAGGGCTTCGTTGAATATGAGCTGCTTCATCTGGTCTTCCGTCA
GAGGGTGCTGCTCGAAGTCGAAGGAGAAGGGGTCCGTGCAGATGGGCTCATCGGCGACGTCGTGAAGCCGCTCTAGG
TACGGGTGCTCGAGCGCCTCTTCAACTGTGATCCTCTGCAGCGGGTTGAAGGTGAGCATCCTCTCGATGAGGTCCAG
CGCGGCGGGCTGCACCTTGGGGAACTGTCCCGGGAAGGACCGGCGAGGGAACTGCGGCAGGTGCCTCATGTACCTCC
TGGCGTCCTCGTTCCGGATGAAGCCCAGGTCGTCGTCGGTGGGGGTGCCGATCACCTCCGTGATGAGCCGCATCTGG
TGCATGTGGTCCCTCCCCGGGAAGAGCGGCGCGCGGTTGATGAGCTCCATGAAGATGCAGCCGACGGACCAGACGTC
GATGGCGGCCGAGTAGTCGGTGGAGTTGAGCAGCAGCTCCGGGGCCCGGTACCACCGCGTGACCACGTACTCCGTCA
TCATGTCGCTCTCCGACGACGGCCGCGCGAGGCCGAAGTCGCAGATCTTGAGGTCGCAGTTGGCGTTCAGTAGCAGG
TTGCTCGGTTTCAGGTCGCGGTGGATCACGTTCGCCGAGTGGATGTACTTGAGGCCGCGCAGCAGCTGGTACAGGAA
GTACTGGCAGTGTTCTTCTGAGAGTTCTTGGTTGGAGCGGATGATGTGGTGGAGGTCCGTGTCCATGAGCTCGGTGG
CGATGTAGACGTCGTTGAAGGACTGCGGGATCGCCGGCGGGATCACATCTCGGAGGCCTACTATGTTCTCGTGGTCG
AGGTGCCTGAGGAGCTTGATCTCCCGGAGCGTGCGCTTGGCGTCCATGTTGTTGTCGAAGGCGTTTGCGATCTTCTT
GATTGCCACCATCTCCCTCGTCTCGAAGTTCATCACCGAGCAGACGATCCCGTAGGCGCCGCGGCCGATGGGCATGA
TCGGCGGCTGGTACTTGGCCGTGATCTCGAACTGGTTGCCGAATATGTTGTAGAGGAGGAAGCGGCCGCCGTGCGTC
ATCGTCGGCCGGAACTCGGCCACCGGAGCGCCGTC**CAT**TCTCAAACAGCCAAGAAACTCTCAAAACCCAATCACAGG
AGGCAACACGCACGCACGCACGCGCAACGGGATGAGGCTGATGAGCTGATGATTGACTTTATCCTGGCTCTCGATTC
TTGTAGGGGGGAAGAGGAGAGAATTATAAATAGAAAAGGGCGGCTCCGCTAATGCTCTTGGCCCA

>TsMPK4 : contig67966
TTTTGAGATAATAAAAGGCAATATGTCAACTCAGATATACATGGGATGTTTAAATAAGTGTACACAAAATTACAAGC
CTTAGATACACAAGAAGAATTTATTCAATCAACCGGTATAATACAAGCCCAGGCGCAAGCCATCTCATACAAATGTC
GTTTCTTCAGAAAGGCCCCAAAAGAAATAGTTACAGCAACAAATAATCCATGCCCATTCGCCCAATAAGCGAGCATA
AAATAAATAGATGCACAAGACATGGAAATATCGGCTTCACTAGAGCTAGAGGTAGATCTTCAATGCCCTCAGCTGGT
AATTTGGTCTTGGCTCTTAGTAGGGAGGATCAGGGTTAAATGCTAAAGTTTCCCTCCAGATGAGCTCTTTCATATGT
TCTTCTGTAAAAGATGGTTGCTCAAAATCAAAGCTGAAAGGTGCCGGGCAAGTGGGTTCTTCATTGATGTCATGAAG
AGAAGCCAAGTATGGGTGATGCAGAGCCTCATCAACAGTGATGCGTCTGCTTGGGTCAAACACCAGCATTCTCTCTA
ACAGATCAACTGCACCATCAGACATGTTGCGGAAGCGCAGGCGGAAGTCCTGCCTTGGGTACTGTGGTAATTGCT**TC**
ATGTATCTTCGTGCATTATCACTCCGAAGAAACCCAGGCTCGAATCATCTGGTGATCCTATCAGCTCAGTGATCAAT
TTTAACTGTTGGATGTAATCTCTTCCAGGAAATAGGGGTTGACGAGTAATAATTTCACCAAGTATACACCCAACTGA
CCAGACATCAATAGCAGCAGTATATTGTGAACAGTTCAACAGCAACTCTGGTGCTCGGTACCAACGAGTGACCACAT
ACTCTGTCATGAGATCGGTTTCTGAAGTGGTCCTTGCAAGCCCAAAGTCTGCAATCTTGAGATCGCAATTTGCGTTT
AGGAACAAATTGCTTGGCTTCAGATCACGGTGCAAGACATTTGCTGAGTGCACATATTTTAGCCCTCGAAGCAACTG
ATACAAGAAGTACTGGCAATGGTCATCAGTCAATGATTGATTTGAGCGAATGATCTGGTGGAGATCTGTGTCCATCA
ACTCAGTAACAATGTACACATCCTTAAAATCATCTCTTCTTGGGGGGCGTATTAAATCCTTTATGGCAAGAATATTC
TCATGGTCCATGTGGCGAAGAAGCTTTATTTCTCTCAGCGTCCGCTTGGCGTCGATGTGGTTGTCAAACGCATTTCC
AATCTTCTTGATCGCAACCTCCTCTCCTGTATCCGAACTAACCGCCGCGCAGACAATGCCGTAGGCGCCTCGGCCGA
TGGGGCGGATGGGAGGGGCGTACTTGGAGGCGACCTCGAAGAGGTTGCCGTAGACGTTGTAGAGCACGTAGCGGCCG
CCGTGCGTCGCCATCCCCTGGATCTGCGCGGCCCCGCCCGCGGCGCCGCCGCCGCCGCCGGAGGTATC**CAT**GGCCTC
TTCGTCGCCGCTCGCGAGGGGGAGGAGCCGAGGAGGGGTGGAGAGTAGGGTTGGGTTCTAGGGTTGGTGCTCCGGAG
CGACTAGTCGTTCATGGGCTGGGGACTCTGGTGGAGAAGCTTCCCCCGGTGATGTTTTCTACTTGTA

>TsMPK6 : contig251617
GCGGCGGCCGCGGCGGGGTCGGCGCCGGGCGGCGCG**ATG**GAGAACATCCAGGCCACGCTCACCCACGGCGGGAGGTT
CATCCAGTACAACATCTTCGGCAACGTCTTCGAGGTCACCGCCAAGTACAAGCCCCCCATCCTCCCCATCGGCAAGG
GCGCCTACGGCATCGTCTGCTCCGCGCTCAACTCCGAGACGGGGGAGCAGGTGGCCATCAAGAAGATCGCCAACGCC
TTCGACAACAAGATCGACGCCAAGCGCACGCTGCGGGAGATCAAGCTGCTCCGCCACATGGACCACGAGAATATTGT
TGCAATAAGGGATATTATACCTCCTGCACAAAGGACTGCATTTAATGATGTCTATATTGCATATGAATTGATGGACA
CCGACCTGCATCAAATTATTCGCTCAAATCAAGCTTTATCGGAGGAGCACTGCCAGTATTTCCTTTATCAGATCCTT
CGTGGCTTGAAGTATATACATTCAGCAAATGTTCTCCACCGAGACTTGAAGCCTAGCAATCTTCTTTTGAATGCAAA
CTGTGACCTAAAAATTTGTGATTTTGGACTTGCTCGTACCACCTCAGAAACTGATTTTATGACCGAGTATGTTGTGA
CAAGATGGTACAGGGCACCAGAGCTTCTGTTGAACTCCTCCGAATATACTGCAGCAATTGATGTGTGGTCTGTGGGC
TGTATATTTATGGAACTGATGGATCGGAAACCTTTGTTTCCGGGAAGAGACCATGTCCATCAGCTACGCCTACTAAT
GGAGCTCATTGGAACACCAAATGAGGCCGATTTGGATTTTGTAAATGAAAATGCAAGAAGATATATCCGCCAACTTC
CCCGTCATGCAAGGCAATCATTATCTGAGAAGTTTCCACATGTTCACCCTTCAGCAATTGACTTGGTTGAAAAGATG
CTGACTTTCGATCCTAGACAGAGAATAACAGTTGAAGGCGCACTTGCGCATCCTTACTTGGCATCGCTGCATGACAT
AAGTGATGAGCCAGTCTGCACGATGCCCTTCAGCTTTGACTTCGAGCAGCATGCGTTGTCGGAAGAACAAATGAAGG
ATCTAATCCACCAAGAGGGCATCGCGTTCAACCCTGATTACCAG**TAG**CTGGAGTTCCTTTGATCGGCTTCATTCATA
TGGAAAGTTTTCGGTCCTCCTGCCGCCATAAAATGTCGCTAGCTGTAAATAATTGCCTCACCCAGAGAATCAAAAGG
AGATGGCGTGTTAAGGGTAGATGACAAGAGCTGTGGTGGTCAAATTTCCGTGTAGCCTATGGATTCTTGTGCTTGTG
TATGTTGTTTTATGTGGATTTTTTCCCCTTCTGCTTGAAGATGTTTCAGCATTTTCCGTAAGATGCGATAGTCCGTG
AACGATGGCTGCCTAATTTCTGTGGCCGTCATGAGATTTTTACATTGTGGTGGATTATGGATTATGTAATGTTGTCC
TGGTTAATGAACTCCGACTGACTGATTGGAATATTGTGTTTGAATGCGGACTGACCGTGATTTAGTGGTTGCTGTTT
GAAGTTGTAGA

>TsMPK7 : contig199810
ACTTGAAAGTTTAGACCTCCTTACATAGAGCAGCTTCTAAAATTACAACTTACAATCATGGGGAAAAGGGAATATTA
CAACTTACACAAGCTTCTTCATAACCCAATTCTTAATAAACCGAATTCCGCTTCTACAACACAGTAGTCCATATACA
TAGAGCAGTGTCTAAGATTACAAACTTACAATCAGGGGAAAAAAGAGAGAAGATTACAACTTACACAAGCTTCTTCA
TAACCAGATTCTTGATAACCGAATTCCGTTTCTACAACACAGTAGTCTATATATACTAGCAAGTACTGATCCACAGC
TAGAGAAATCCATACGCCGTTTATTGTGCACATATTTACATAAACAGGTCACTCGACGGCATTGCGATAATTGCATT
AAAGCTTTATATGAAGAACGAACATGTGAAGACCCTGGTGTGGCACACTGCTCTCACATGTTCATTCCTGCTTG**TCA**
CATATTCACCATCCTGGCGGCCTCAGGGTGATACTGGAGCATCTCCTGCCACAACATTTCTCGGATCATATCTGTGC
CAATGTTTTCATCTATGTCGAGATCGATGGGCACCTGAGCAGGAGGGTTTGCGCTGGGATCATACAGCGGGGACATG
TAGGGGTGCTCCAGAGCCTGGGTGACACTGATCCTCTTGGAAGGGTCAAAGACAAGCATCTTCTGCAACAGATCAAT
GGCAAGAGGATGGGCGTGCGGGTACATGATACTGAGGGGCATCCCCGGGGTGTATGGAAGGGATTTAATGTAGTTGC
GAGCTTTTGGGTTGTCAATGAACGCAAGGTCAGTTTCGCTCATGGTGCCAAGAACATTGACTATAAGCTTAAGCTGA
TTAAGGCACTCGGTTCCTGGAAAGATCGGCTTGCGGCCAAGTAGCTCAGCAAAGATGCAGCCAACAGACCAGACATC
TATGGAGGTGCCATAGTTGTCGCAGCAGAGCAGCAGCTCGGGAGCTCTGTACCAGCGGGTGACAACGTATTCAGTCA
TAAACTGACCTTTAGTGTTATTTGTGCGAGCCAGACCAAAGTCACAGATCTTCAGGTCACAGTTCGCATTGACCAGA
AGGTTCCCAGGCTTCAGGTCTCTATGGAGTATCCCCGCATGAAGGTACTTCAGTCCTCGGAGCAGCTGAAAAAGGAA
ATACTGGCAGTGGTCATTGGACAGCGGCTGAGACGACTTGACTATCTGATGCAGATCCGTGTCCATGAGCTCGGAGA
CCAAGTAGACATCCTTGAAGCTCCTCCTATGAATCGGCATCATTATATCCTTCAAAGCAATGACATTCTCATGACGC
AGGTGCCTGAGGAGCTTCAGCTCCCGCAGCGTCCTCAACGCATCCACGCGGTTGTCAAAGACATTGTTTATCTTCTT
GATGGCGACCTTCTCGTTGGTCTCCTGGTTGATGGATGAGCAAACTATCCCGTAAGCACCTCTGCCAATGGGCTTGA
TCGGCACGTACTTGGTGTCGATCTCGAACATGGTCTGCCACATGGTGTAGTAGTGCTTCCCGTGGTTTCCCATGCCG
TTCGGGGGATCCACCATCATCGC**CAT**TTTCTCAGGACGCGGCGGCAGATCTGGAGAAGCAGAGCACGTCCGGGCTCG
TCCGGGAGCGGGCGAGGGAGGAAGGGGATGGGGAGCGCACGCGGAGACGAAGGCGGAGGCCAAAGGCAGAGAGCGAC
CGAGCAGGAGGGCGCTCTGTCTATTTTTTATTTCGGGGAAGGAAGGAAGGAAGGAACGAACGTACGCACGCACGTAG
CTCCACTGGACTTGTGGGAGTGGTGACGAGGAGATGGCGATGGATGGGTGCCGCTCAGGTACAGCGAAGGGGCGGGC
AGCTAGC

>TsMPK11 : contig120398
GCGCGGCGTGCGTTTCTTTCTTTCTTGGCTAGCTCCTCGATCAAGGTGTTCGCGCGCAGGCGTGCGGGTCG**ATG**CGC
ATGGAGGGTGGAGGCGCCGGGGCAGGAGGAGGAGGAGGTCACGGCGGCGGCCATGGCCTCGGCGGCGAGGCGCAGAT
CAAGGGCACGCTCACCCACGGCGGCAGGTACGTGCAGTACAACGTCTACGGCAACCTCTTTGAGGTCTCTGCTAAGT
ACGTCCCACCCATCCGACCTGTCGGCCGCGGCGCCTGCGGCATCATCTGTGCTGCTGTAAATGCACAGACTCGTGAG
GAGGTCGCTATCAAGAAGATTGGTAATGCGTTTGACAACCAGATCGATGCCAAACGCACTTTGCGAGAAGTAAAGCT
GCTTCGCCACATGAATCATGAGAATGTGATTTCAATAAAGGACATCATACGCCCACCAAGGCGGGAGAACTTCAACG

ATGTTTACATCGTCTATGAACTGATGGACACTGATCTTCACCACCTTCTAAGATCAAACCAGCCACTCACGGATGAT
CACTGTCAGTATTTTCTCTACCAAGTGCTCCGAGGATTGAAGTATGTGCATTCAGCAAAGGTCTTGCACCGGGACCT
CAGGCCGAGCAACCTGCTGCTCAATGCCAAGTGTGAACTCAAGATTGGAGATTTTGGCTTGGCTAGGACCACCACTG
AGACTGACTTCATGATGGAGTATGTTGTTACTCGGTGGTACAGGGCGCCTGAGCTCCTGCTCAACTGCTCGGAGTAC
ACTGCAGCAATTGATATATGGTCAGTGGGTTGCATCCTCGGTGAGATTGCTATGAGGGAGCCACTGTTCCCTGGAAA
AGATTATGTTCATCAGCTGAGGCTAATTACTGAGCTGATAGGCTCACCAGATGACACGAGCCTTGGGTTTCTTCGAA
GTGATAATGCCCGCAGATACGTGAGGTCTCTTCCTCAATACCCGAAACAGCATTTTGGTTCACGGTTCCCCAGTATG
TCCACCGGCGCCGTGGATTTGCTTGAGAGGATGCTCGTGTTTGATCCGAGCAAGAGGATTACTGTTGATGAGGCTCT
ATGCCATCCGTACTTAGCATCCCTTCATGAGATAAACGATGACCCTGTCTGCCCAGCGCCTTTCAGCTTCGACTTTG
AGCAGCCATCATTTACTGAGGAAGATATCAAAGAACTTATTTGGAGGGAGGCTCTCAAGTTCAACCCTGAACCAATT
CAC**TGA**AAAGTTGCCAATCTTTATCTTTACCTAATAATAAAGGACTGAGGCTTTCATAGTTCTTCATACGTCGTCTG
TTTTTATCCGTTGATTTTATGTAAACCTCAAATAGTTGGCGGCTGAGATTTAAAACTAGATGTACACACCTAAGGAA
AAAAACGCTCGTC

>TsMPK11 : contig88156
GCGCGGCGTGCGTTTCTTTCTTTCTTGGCTAGCTCCTCGATCAAGGTGTTCGCGCGCAGGCGTGCGGGTCG**ATG**CGC
ATGGAGGGTGGAGGCGCCGGGGCAGGAGGAGGAGGAGGTCACGGCGGCGGCCATGGCCTCGGCGGCGAGGCGCAGAT
CAAGGGCACGCTCACCCACGGCGGCAGGTACGTGCAGTACAACGTCTACGGCAACCTCTTTGAGGTCTCTGCTAAGT
ACGTCCCACCCATCCGACCTGTCGGCCGCGGCGCCTGCGGCATCATCTGTGCTGCTGTAAATGCACAGACTCGTGAG
GAGGTCGCTATCAAGAAGATTGGTAATGCGTTTGACAACCAGATCGATGCCAAACGCACTTTGCGAGAAGTAAAGCT
GCTTCGCCACATGAATCATGAGAATGTGATTTCAATAAAGGACATCATACGCCCACCAAGGCGGGAGAACTTCAACG
ATGTTTACATCGTCTATGAACTGATGGACACTGATCTTCACCACCTTCTAAGATCAAACCAGCCACTCACGGATGAT
CACTGTCAGTATTTTCTCTACCAAGTGCTCCGAGGATTGAAGTATGTGCATTCAGCAAAGGTCTTGCACCGGGACCT
CAGGCCGAGCAACCTGCTGCTCAATGCCAAGTGTGAACTCAAGATTGGAGATTTTGGCTTGGCTAGGACCACCACTG
AGACTGACTTCATGATGGAGTATGTTGTTACTCGGTGGTACAGGGCGCCTGAGCTCCTGCTCAACTGCTCGGAGTAC
ACTGCAGCAATTGATATATGGTCAGTGGGTTGCATCCTCGGTGAGATTGCTATGAGGGAGCCACTGTTCCCTGGAAA
AGATTATGTTCATCAGCTGAGGCTAATTACTGAGCTGATAGGCTCACCAGATGACACGAGCCTTGGGTTTCTTCGAA
GTGATAATGCCCGCAGATACGTGAGGTCTCTTCCTCAATACCCGAAACAGCATTTTGGTTCACGGTTCCCCAGTATG
TCCACTGGCGCCATGGATTTGCTTGAGAGGATGCTCGTATTTGATCCGAGCAAGAGGATTACTGTTGATGAGGCTCT
ATGCCATCCTTATTTAGCATCCCTTCATGAGATAAATGATGAACCTGTCTGCCCAGCGCCTTTCAGCTTCGACTTCG
AGCAGCCATCATTTACTGAGGAAGATATCAAAGAACTCATTTGGAGGGAGGCTCTCAAGTTCAACCCGGAACCAATT
CAC**TGA**AAAGTTCCCAATGTAGAAAAAGAAGCAATTTCAAATGGCAAATCTATCAGCTCCGTGGGAAAATACATGAG
CTGACAGCACGATGTACAGAGTAATGAGTGATCACGTCTCAGCATCTTGTCATACGGCTTTGTTGATGACACCATGA
CTTCGAAAACCTGGAAATTTAGGAAACATGAACGCTTGATCAATAATTTGTTAGCCAAAAAAAGTGGGGCCATTATC
TGTATCATGTCAGGTTGTATCTACCCCCTTTTTGTTGTATGCTTAACAAGACCTGAGTTATGGACTATGGTATTACA
GCTTCCGGCCACTCTCCGGTCGTCAGTGTTGTTCACCATCCAACAGATGTTACTTATTATTA

>TsMPK14 : contig220413
CCCCACCCGCATCCTCCCTCCCTCCCCCAGCTGCAATTCACCTCCGAATTCGGTCGCCCATGGCGCCGCGGCTGCTC
TGATCCTACCCACCTACCCCTCCCCCCCCCTCTCCTCTCCAGCTGCTCTCCCTTCCCCTCTCGTTTGAAA**ATG**GCAA
TGCTGGTGGATCCTCCGAATGGCATGGGAAACCAAGGGAAGCACTACTACTCAATGTGGCAAACCTTGTTTGAGATT
GACACCAAGTATGTGCCTATCAAGCCCGGCCGAGGAGCTTATGGAATAGTTTGCTCATCCATAAACCGTGAGACAAA
CGAGAAAGTAGCGATAAAGAAGATACATAATGTATTCGACAACCGTGTGGATGCACTAAGGACCTTGCGGGAGCTGA
AACTCCTCCGGCATCTCCGCCATGAGAATGTTATTTCTTTGAAGGATATAATGATGCCTGTACAAAGGAGGAGCTTT
AAGGATGTGTACTTGGTTTATGAGCTCATGGATACTGACCTGCATCAGATAATCAAATCGCCTCAGGGGCTTTCCAA
TGACCACTGCCAATATTTTCTTTTTCAGTTGCTTCGAGGACTGAAATACCTCCATTCAGCAGAGATACTCCACAGAG
ACCTAAAACCTGGGAACCTACTGGTGAATGCAAACTGTGATCTGAAGATATGTGATTTTGGTCTTGCACGTACAAAC
AGTAGTAAAGGCCAGTTTATGACTGAATACGTCGTCACCCGCTGGTATAGGGCTCCTGAGTTGCTGCTTTGCTGTGA
CAACTACGGCACTTCCATCGATGTTTGGTCTGTTGGCTGCATCTTTGCTGAGCTACTTGGCCGCAAGCCTATTTTTC
CTGGGACAGAGTGCCTCAATCAGCTAAAACTGATAGTCAATGTTCTTGGCACCATGAGCGAGTCTGACCTGGAGTTC
ATCGACAACCCAAAAGCTCGCAGGTATATCAAGACCCTCCCCTACACTCCCGGTGTTCCTCTCGCAAGTATGTACCC
ACATGCACACCCTCTGGCCATCGATCTATTACAGAAGATGCTCATCTTCGACCCTACCAAAAGGATCAGTGTTACCC
AGGCCCTTGAGCACCCTTACATGTCTCCTCTGTATGACCCAAGCGCAAACCCTCCCGCGCAAGTGCCCATCGATCTC
GACATAGATGAGAACATCAGCTCAGAGATGATCAGGGAAATGATGTGGCAGGAGATGCTTCACTACCACCCTGAAGC
CGCCACTGCAGTAAACATG**TGA**CGGCGTTCTTGCAGTGCCCCGGGAAGAACTCGGCAGGCTCACCTCTTTTTTCCTC
GAAAAGACTACTGCGATTATCGCACCTATTAAGTAACCACGACGTGCAGTGTGGAGAGTTATCTCCGTGTAAATATG
CAGTGCGATAAGAACCGCATATGGATAGTTCTTGTTATGGACCACTATTTGGTGTATGTATACTGTTGTGTTGTTGT

ATGAGCTCATGAAAGAACTGTTGAAGCGGATTCAGTAAGTTGTTGATTTGTGATGGCCCTCCTGTGTTTGTTGTTGC
AGTTGTTGGTATTTGAAGCAGACCTGCTGTTATTCGCTTGTGTGTTTTATTTAGTACTATTAATCGG

>TsMPK16 : contig209684
CTACTAACTTACTGCATATCACATTACAAATTCCGCACAAAAAACCTGGGGTTTCAATAGCGAGCAGGGTCCAAATA
ATGTGTTATCAGAAACAGTGTTCAATAGTTCAATACTACAGCTGGATGACAGACTACCAAGCAACAAGAACATACTT
TAAAGTCAATTTAACTCCGTGTTACAACATTAGATGTACAGTGGGCTCTCTGAACCTCCCTCAGGGCCACCAGCTTG
GAGAAACGAATTGCGGAACACCATCAGTCAGGTCAGTAACAGTCCTTTGTCGCGAATGCCACTCAAGCTTCCAGTTT
TTGGTTATACTTGTTGAACAGTTGAACTGGATCTACCCAATTGCAGGTAACCAGACCAGAGTTGAGCTTAACAGGAG
ACTAGCCTGCCACAACGACCTTGCATACAGATCTATCGTCTCCGGGAGATGTAACCGTGACAGATAATATAACTCTG
TACAGAATGGCATGTTTGAAGGTGCCTCCTTGGAGTGAGTAACCCTTCCTGATCCAGTGACAAGGATTTTCTGGCAT
TGCCACAGCAGTCTACCAGTGCCCGTTGCGGCCATCCACAGTTGCAGGTAGTTTATTTCCAGTATATGGCTTTGGTG
GCCCAGCTTGGC**TCA**CGTCGATCCTTTCAGCTTCACCTGTTTCGCTCTTGCAGGTGTTATTTCTTCTAGGGTATGAG
CTGCCCGAAGGAACACCGTTTGGAACAATTCCTTGGCTTGTGATGACCCTTCGCTGTTCATATTGCTCGGTACCAGA
TGTTGAACAGTTACCATAACGAAGTGCCGAACCAACCACTTTACCAGGCCTTGCGACAGCAGCTCCTTAGGAACTCT
GTTTGCACCAACTGAAGCAGGATCTCTGCGTGGCTTTTGTGCATTATCTCTGATTATACACCTGGAAAGATCATCCG
TAATGTTGGCAGCACCCTGTGGTCGGCTATCTGAACCCTGTGGCCGATTATCCGAATAGATAACACTCGGCCTTGGT
AATGAATTATGTTGCCTCTCAGGCGGTGCTGCTGTTGATCCCTTTGCATAATGCTCTTCAAGGAATGTGAATTGCTT
TTTGAAATGATCTACTGCACTTGGGTACATGAAATTAGTTGGCTCCGCCCCCTCAAGGAATTCCCTCAGCATGTTTG
GATGATATTCCAGAATTTCTCTGTATATGAGTTCCCTTATGTCGTCCTTCGTAATTCTTCGTCTCTCAAACTCAAAC
TCGAGCTTAGTAATGGGCTGTGCAGAAGGCTCCCTATCCACACTAGCTATGTTCTTGAAATAAAGATCAGCAAGAGC
CTCTTCAGCACTTGGCCGGTATTTTGGATCAAATGCTAGCATTCTCTCTAACAAATTTAATGCAAGTGGATCTGCAT
TCGGAAACTTCTGTGTAAACGGTACAGGCTTTTTCCGCCTCATACTGCTCAAGTAGCGTCTGGCCTTCTCATTTCGA
ATCCTAGCAATTGTTTCTGGAGCAGGCGTTCCCAGTAGATCTGTGATTATATCAAGTTGATGCACCACATTTTTCCC
AGGAAAAAGAGGTTTGCCAGTTAGAAGTTCCGCAAATATACATCCAATACTCCATATATCTATTGCTGGTGTGTACT
TGGAGAAAAAAGATCCACATAGCTCAGGTGCTCGGTACCACCTTGTTGCGATATAATCCGTCCAAAATATGGCGGTT
GGAGTATCACTTATAGCTACCCTTGCAAGACCGAAGTCACATATTTTGAGCTTACAATCAGCATTAGCCAAGATATT
CTTTGGTTTGAGATCTCGATGAAATACATTTGCTGTATGTATGTATTTCAATCCTCGGAGCAACTGATACAAGAAAA
ACTGATAATGTTCTGGAGTCAAGTCATCATTCGCCTTTATAACTTGGTGCAAATCAGACTCCATGAGTTCGAAAACA
ACGTATATATCCTTGAACTCTCTCCTCGACGGAGGAAGTAGAATATGTTTTATTTCCACAATATCAGGGTGTCTTAG
GAGCCTAAGCAACTTGATCTCGCGGAGTATCCGTGTCGCATCAGACACATGTTCAAAGATGTCGTTTATCTTCTTGA
TAGCAACTTTCTCACCCGTGTGAGTATCCAAAGCAGAGCAGACCACGCCATAACTCCCTTTTCCTATAACCTCTTCT
ATCTTGTATCTGTTTCCCTCGCCATACTCGGTAAAAAAGTC**CAT**TTGAGATGTTCGCTCACGCGCGCACACGACGAC
GAGGGGGAGGAGTCCAGCTGCGCTGCGCTGCGATCCAAGGCGAAGCTCGAGGACAGAGGGGAGGGGGCGGTGGTAGC
TGGGAG

>TsMPK16 : contig244429
AAACATACGTCGCAACTCCATTTTTACTCGGGCTACTTGATTACTGCATACATCATTATAAATTCCGCACTCAATAG
CGAGCAGGGTCCAAATAATGTGTTATCAGAAATAGCCGTGTTTAATAGTTCAATACTACAGCTTGATGACAGACTAC
AAGCAACAAGAACATTAAAGTCAATTAATTAACTCCGTGTTACAACATTAGATAGTGTACAGTGGGCTCTCTGAACC
TCCCTCAAGGCCACCAGCTTGGAGAAACAAATCGAGGAACACCATCAGTCAGGTCAGTAACAGTCCTTTGTCGTGAA
TGCCGCTCAAAGTTTCCAGTTTTTTTGTTATACTTGTTGAACAGTTGAACTGGATCTACCCAATTGCAGGTAACCAG
ACCAGAGTTGAGCTTAACAGGAGACTAGCCTGTCGCAAAGGCCTCTCATACAGTTCTATCGTCTCCGGGAGATGTAA
GCTTGACAGATAATATAACTCTGTACAGAATGGCATGTTTGAAGGTGTGTCCTTGGAATGAGGTGACAAGGATTTTC
TGGCATTGTCACAGCAGT**CTA**CCAGTGCCCATTGCGGCCATCCACAGTTGCAGGTAGTTTATTTCCGGTATATGGCT
TTGGTGGCACGGCTTGGCTCACGTCGATCCTTTCAGCTTCACCTGTTTCACTCTTGCAGGTGTTATTTCTTCTAGGG
TATGAGCTGCCTGAAGGAACGCCGTTTGGAACAATTCCTTGGCTTGTGATGACCCTTCGCTGTTCATATTGCTCAGT
ACCAGATGTTGAACAGTTACCATAACGAAGTGCCGAACCAACCACTTTACCAGGCCTTGCGACAGCAGCACCCTTAG
GAACTCTGTTTGCACCAACTGAAGCAGGATCTCTGCGTGGCTTTTGTGCATTATCTCTGATTATACACCTGGAAAGA
TCATCCGTAATGTTGGCAGCACCCTGTGGTCGGCTATCTGAACCCTGTGGCCGATTATCCGAATAGATAACACTCGG
CCTTGGTAATGAATTATGTTGCCTCTCAGGCGGCGCTGCTGTTGATCCCTTTGCATAATGCTCTTCAAGGAATGTGA
ATTGCTTTTTGAAATGATCTACTGCACTTGGGTACATGAAATTAGTTGGCTCTGCCCCCTCAAGGAATTCCCTCAGC
ATGTTTGGATGATATTCCAGAATTTCTCTGTATATGAGTTCCCTTATGTCGTCCTTCGTAATTCTTCGTCTCTCAAA
CTCGAACTCAAGTTTCGAAATTGGATGCCTTGAAGGCTCACGCTCCACATTGGCAAGACTTGCAAAATATGGGTCTG
CCAAAGCCTCTTCAGCTGAAGGCCGGTCTTTAGGATCAAATGCAAGTAAACGCCCTAGTAAGCGAACCGCCAATGGA
TCAGCATTAGGAAATTTCTGAGTCAAGGGTACAGGATGCTTTTTTCGCATGCAACTTAAGTAGCGCCTGGCCTTCTC
ATTTCGAATCCTAGCAATTGTTTCTGGAGCAGGTGTTCCCAGGAGATCTGTGATTATATCAAGTTGGTGCACCACAT

TTTTCCCAGGAAAAAGAGGTTTGCCAGTTAGAAGTTCCGCAAATATACATCCAATACTCCATATATCTATTGCTGGT
GTGTACTTGGAGAAAAAAGATCCACATAGTTCAGGTGCTCGGTACCACCTTGTTGCGATATAATCCGTCCAAAATAT
GGCGGTTGGAGTATCACTTATAGCTACCCTTGCAAGACCGAAGTCACATATTTTGAGCTTACAATCAGCATTAGCCA
AGATATTCTTTGGTTTGAGATCTCGATGAAATACATTTGCTGTATGTATGTATTTCAATCCTCGGAGCAACTGATAC
AAGAAAAAACTGATAATGTTCCGGAGTCAAGTCATCATTGGCCTTTATAACTTGGTGCAAATCAGACTCCATGAGTTC
GAAAACAACGTATATATCCTTGAACTCTCTCCTCGACGGAGGAAGTAGAATATGTTTTATTTCCACGATATCGGGAT
GTCTTAGGAGCCTAAGCAACTTGATCTCGCGGAGTATCCGCGTCGCATCAGACACATGTTCAAAGATGTCGTTTATC
TTCTTGATAGCAACTTTCTCACCCGTGTGAGTATCCAAAGCAGAGCAGACCACGCCATAACTCCCTTTTCCTATAAC
CTCTTCAATCTTGTATCTGTTTCCCTCGCCATACTCGGTAAAAAAGTC**CAT**TTGAGATGTTCGCTCACGCGCGCGCA
CGACGACGGGCGGGGGGAGGAGCCCAGCTGCGCTGCGATCCAAGGCGAAGCTCGAGGACAGAGAAGAGGGGGGCGGT
GGTAGCTGGGAAGTCAGGAGAGAGCCCCACACACGTGGCCTCCGCTTTCGGAATTTATACCGGAGATTTATTTCTCT
CTTTGCTTAACTAGAT

>TsMPK17 : contig26009
TCACCGGCGGCAGGCAGAGAACTCACCGACGTACAAATGCCAAGTAACTGATCCAAGAGTCGAGACAAATTGGGGAG
AATGAATGATCACAGCTCTTGAAATGGAGGGGGAATCCTTTGGACTTGTCTTCTGTATTTTGTAGACCGTGGAGCAA
TTGCGTTGCCATATCGCACACGTCCGGTAGGAACTGAGACCGGTCTGGTCGTCCCCCGTCATTATCAGTCTCGGAAG
AATCACGCACCAAGAGGCGGCACAACTTCCTTGGCAGATGCCGTCGACCTCGATGCTGAAACTAGGCTACAAATGAC
GGGGAAACATCGTTCAAATTGAGAAAAATACTACTAGATGTTTCTAAATCTGTACTCAGGGTCCAGCTTAATTGCCA
CATTTATCCAAAACGCGAAAAGAAAGGAAATGAAACTCAGCACTTGTTGATATAGATGCAGAGCAGCAACGCTTCTT
GATATACTATTAAGGATGTTCGACAAGGAAACATGACAAGTGTTCATCAGATGTCTTGGTCCGTTCGTTACAATCAT
GTATACTTAGATGCTAGAGAGCAAATGTCGGTGTTACATTGCGAGCTTGAGTGTAAAAATACATCAATCCATGCAGG
GTACACCAACGATGCTGGGCACTTGGCAGGAACACGCACTCGCTAAACCATCCCCAGCTTGCAGAGCGGCGCAGTGC
TCTGCTTCCCTAGGACAGCGCTGCTCTGCTATCATCATCCGGTGTACATGACAGAAATGCGGTGCAGAGCTCGACTT
TCGTTTGTCGTCCCCTGCA**CTA**GGCGTGCATCTTGGAGACCTTCTCGGATAGTCCGTCGACGGCCCCTTCCGCTTCC
TCGGAGATTGCATCGTCATACTCTGGCTGTTTATTCGGGTTGACAACGACACACTTGGAAGCGCTAATGCTCGCGCT
CTTGAGGTAGGTCCGCGAGCTTAAGCTGGTGGGGCTCAGACCATTCTGGCCAACGCCGGGTGCATCCTGTGCCTTTT
GAGGGCTCTCTGTACCATGGGCATCAGGCTCTGCACTCATCTCCTGATCACTAATATGCTGTTCATTATTACCGTCG
TTCGACGCACCTACTCTCTGCCTTGGTAAAGAGGCATGCTTTCTTTGCAGCGGGGAACCTCGTTCTCCTTTGCTGTA
ATGCTCCTCCAGGTGTGCAAACTGCCGTTTAAAGCGGTCAACACCACTTGGATAGAGGAAGCTAATCTGGTCACCAC
CTTTCATGTATTCCTCCAGCATCTGTGGATGGTACTCCAGAATCTCTCGATAGATCAACTCCCTAACATCATCTTTT
GTAACCTTCCGTCTCTCAAACTCAAACTCAAGTTTCGAAATTGGATGCCTTGAAGGCTCACGCTCCACATTGGCAAG
ACTTGCAAAATATGGGTCTGCCAAAGCCTCTTCAGCTGAAGGCCGGTCTTTAGGATCAAATGCAAGTAAACGCCCTA
GTAAGCGAACCGCCAATGGATCAGCATTAGGAAATTTCTGAGTCAAGGGGACAGGATGCTTTTTTCGCATGCAACTT
AAGTATCTCCTGGCCTTTTCATTTCTAATCCGAGATAAGGTTTCTGATGATGGAGTTCCAAGAAGATCTGTTATTAT
ATCTAACTGGTGTACAACATTCTTCCCAGGAAAAAGTGGTCGTCCAGTGAGAAGCTCGGCAAATATGCATCCAATAC
TCCAAATATCAATAGCAGGAGTGTATTTCGAGAAAAAGGAGCCACATAATTCAGGGGCTCTGTACCACCTTGTTGCT
ACATAATCCGTCCAAAATATAGCTGAAGGAGCATCATTAAATGATACACGCGCAAGTCCAAAGTCACAAATTTTCAG
TTTGCAGTCTGCGTTGGCCAGTATATTCTTGGGCTTCAGATCGCGATGAAATACATTAGCCCCATGGATGTACTTGA
GAGCGCGGAGAAGCTGGTACAGGAAAAACTGGTAATGCTCCGCCGTGAGGTCATCATTAGCTCTGATGACCTGATGG
AGATCCGACTCCATCAGCTCAAAAACAACGTATATATCTTGGAACTCCCTCCGAGAAGGAGGGAGCATTATGTGCTT
GATCTCTACCACGTCTGGATGACGTAGCAGCCGAAGGAGCTTGACCTCACGGAGGATGCGCGTGGCATCCGAGACGT
GCTCAAACACATCGTTGATCTTCTTAATCGCAACCCGCTCGCCGGTGCGGGTATCTATTGCAGCAGCAACTACTCCA
TAACTCCCCTTGCCAACAACTTCTTGGATTTGGTACTGGCTTGCCTCTCCATACTCTGTGAAGAATTCTTTCTCCAT
TATGTTCTTCTTGTGGCTCTCGACGGGGAGCGGCATCTTGCGCTTGGGGACGCGGATGGGGCGGAGGCCCACGAGAT
CCAGGTCCTCGACGGCCTCGACGTCGGAGGGGTCCTCGCCGGCGGAGGACTGGTTGGAGCTGGCGAGCACGGAGCCG
GAGGGCGTGCGGCGGTGGAACAAGCGGCGGAAGCCGTCGACGATGCCGTTCCCTCCTCC**CAT**GTATCCCCTCCCTAC
CCCTGCGGCATTGGCTAGCTGCCCGCCTCCTCCTCCAGCCACAGCCCACAGCTCCCCCTACTCTTCCTCCTCCCGCC
GCCACTCACCGCCACTCAAGGAGACCCCGGCAGCTCACTCACTCTCTCCGCCGCGCTGTCCCCGGCGGTGTCGTCGG
CGCAGAGGACGGACGGATTGGATGCGGGAGGAAACGGCGGGGTCTGCTCTTATACTGCCC

>TsMPK17 : contig75139
TTTTCGCAGCAGTAACGCTTCTTGATATATTAAGAATTTTCGACAGCGAAACATGACAGCGTCAAGATGTCTTGCTC
TGTTCGTTGTTACAATCATGCATAGATGATAGGTGTAGGAACTAATCCCAGTGTTACATTGAGTGTAAAAATACATC
AATCCATGCAGGGCCAGCTCCTGCGTGCAGCAACGACGCTTGACAGGAACATGCACTCCGCTAAACCATCTCCAACT
TGCAGTGCTTTGCTATCAGCTATCAGCATCCGGTGTACCTGACAGAAATGAGAAATGCGGTGCCGACTTCCGTCGTC
GTTGCTGCA**CTA**GGCATGCATCTTTGAGACCTTCTCGGATAGTCCGTCAACGGCTCCTTCTGTTTCCTCAGAGATCG

CGTCGTCATACTCTGGCTGTTTATTCGGGTTGACAACGACGCACTTGGAAGCGCTAATGCTCGCGCTCTTGAGGTAG
GTCCGCGAGCTCAAGCTGGTGGGGCTCAGACCATTCTGGCCAACACCGGGTGCATCCTGTGACTTTTGAGGGCTCAC
TGCACCATGGGCATCAGGCTCTGCACTCATCTCCTGATCACTAATATGCTGCTCATTATTACCGTCGTTCGATGCAC
CTACCCTCTGCCTTGGTAAAGAGGCATGCTTTCTTTGCAGCGGAGAACCTCGTTCTCCTTTGCTGTAATGCTCCTCC
AGGTGTGCAAACTGCCGTTTAAAGCGGTCAACACCACTTGGATAGAGGAAGCTAATCTGGTCTCCACCTTTCATGTA
TTCCTCCAGCATCTGTGGATGGTACTCCAAAATCTCTCGATAGATCAATTCCCTAACATCATCTTTTGTAACCTTTC
GTCTCTCAAACTCAAACTCGAGTTTCGAAATTGGATGCCTTGATGGCTCACGCTCCACATTGGCAAGAGATGCAAAA
TATGGGTCTGCTAAAGCCTCTTCAGCTGAAGGCCGGTCTTTAGGATCAAATGCAAGTAAACGACCCAGTAGGCGAAC
CGCCAATGGATCGGCATTAGGAAATTTCTGAGTCAAGGGTACAGGGTGTTTTTTCCGCATGCAACTTAAGTACCTCC
TGGCCTTCTCATTTCGAATCCGAGATAAGGTTTCTGATGATGGAGTTCCAAGAAGATCTGTTATTATATCTAACTGG
TGTACAACATTCTTCCCAGGAAAGAGTGGCCGTCCAGTGAGAAGCTCGGCAAATATGCATCCAATACTCCAAATATC
AATAGCAGGAGTGTATTTCGAGAAAAAGGAGCCACATAATTCAGGGGCTCTGTACCACCTTGTTGCTACATAATCCG
TCCAAAATATAGCTGAAGGAGCATCATTAAATGATACACGCGCAAGTCCAAAGTCACAAATTTTCAGTTTGCAGTCT
GCGTTGGCCAGTATATTCTTGGGCTTTAGATCACGATGAAATACATTAGCGCCATGGATGTACTTGAGAGCACGGAG
AAGCTGGTAAAGGAAAAACTGGTAATGCTCCGCCGTGAGGTCATCATTAGCTCTGATGACCTGATGGAGATCCGACT
CCATGAGCTCGAAAACAACATATATATCTTGGAACTCCCTCCGAGAAGGGGGGAGCATTATGTGCTTGATCTCCACC
ACGTCTGGATGACGTAGCAGCCGAAGGAGCTTGACCTCGCGGAGGATGCGTGTGGCATCCGAGACGTGCTCAAACAC
ATCGTTGATCTTCTTAATCGCAACCCGCTCGCCGGTGCGGGTATCTATTGCAGCAGCAACTACTCCATAACTCCCCT
TGCCAACAACTTCCTGGATTTGGTACTGGCTTGCCTCTCCATACTCTGTGAAGAATTCTTTCTCCATTATGTTCTTC
TTGTGGCTCTCGACGGGGAGCGGCATCTTGCGCTTGGGGACGCGGATGGGGCGGAGGCCCACGAGATCCAGGTCCTC
GGCGGCCTCGGCGTCGGAGGAGTCCTCGCCGGCGGAGGACTGGTTGGAGCTGCCGAGCACGGAGCCGGAGGGCGTGC
GGCGGTGGAACAAGCGGCGGAAGCCGTCGACGATGCCGTTCCCTCCCCC**CAT**GTATCCCCTCCCTACCCCTGCGGCA
TTGGCTAGCTGCTAGCCGCCTCCTCCAGCCACAGCCCACAGCTCCTCCTACTCCTCCTCCACCAACTCTTCTCGCCT
CCCCCTCCCGCCGCCACTCACTGGCTCGAGGTGGAGTGCTCGGCCGCTATCTCCGGCGGTGTCGTCGGCGCTGAGGA
CGGACGGATTGGATGCGGGGCGCAAACGGCGGGGTGTGCTCTTATACTGCCCGGCGCGATTTGGGGGCGGGGCGGAA
GGAATTTTCTCTCCTTTTTCGGCTCTGCGTTTTTCTGCCGTGTGGGTGACAGATACAAGCTGTCACAGCACACGCTC
GCTCGCTCACACACGGTGTGTGTTGACGAAGGCAAACG

>TsMPK17 : contig38651
TTTTTTAGACGCGGAGCAGTAACGCTTCTTGATATATTAAGATTAAGAATGTTCGACACAAACAGGACAGTGTTTAG
ATGCATTGCTCTGTTCGTTACAATCCGGCATAGATGACAGAGAGCAAATCGCAGTGTTACGTTGTGAGCTTGAATGT
AAAAATACATCAAAGACAGACAGGGCCAGCTCCTGGGTACAGCAACGATGCTTGGCAGGAACATGCACTCGGCTAAA
CCAATCTCCAACTTGCAGCGCTCTGCTTCCTTAGGAGTGCACTGCTATGCTATCAGCATCCGGCGTACCTGACAGAA
ATGCGGCGCCGAGCTCGACTTTCATCGGCGTCCCTGCA**CTA**GGCGTGCATCTTGGAGACCTTCTCGGATAGTCCATC
GACGGCCCCTTCCGTTTCCTCAGAGATTGCATCGTCATACTCTGGCTGTTTATTCGGGTTGACAACGACACACTTGG
AAGCACTAATGCTCGCGCTCTTGAGGTAGGTCCGCGAGCTTAAGCTAGTTGGGCTCAGACCATTCGGGCCAACACTG
GGTGCATCCTGTGACTTTTGAGGGCTCACTGCGCCATGGGCATCAGGCTCTGCACTCATCTCCTGATCACTAATATG
CTGTTCATTATTACCGTCGTTCGACGCACCTACTCTCTGCCTTGGTAAAGAGGCATGCTTTCTTTGCAGCGGGGAAC
CTCGTTCTCCTTTGCTGTAATGCTCCTCCAGGTGTGCAAACTGCCGTTTAAAGCGGTCAACACCACTTGGATAGAGG
AAGCTAATCTGGTCTCCACCTTTCATGTATTCCTCCAGCATCTGTGGATGGTACTCCAAAATCTCTCGATAAATCAA
TTCCCTAACATCATCTTTTGTCACCTTTCGTCTCTCAAACTCAAACTCAAGTTTCGAAATTGGATGCCTTGAAGGCT
CGCGCTCCACATTGGCAAGAGATGCAAAATATGGGTCTGCTAAAGCCTCTTCAGCTGAAGGACGGTCTTTAGGATCA
AATGCAAGTAAACGACCCAGTAGGCGAACCGCCAATGGATCAGCATTAGGAAATTTCTGAGTCAAGGGTACAGGGTG
TTTTTTTCGCATGCAACTTAAGTACCTCCTGGCCTTCTCATTTCGAATCCGAGATAAGGTTTCTGATGATGGAGTTC
CAAGAAGATCTGTTATTATATCTAACTGGTGTACGACATTCTTCCCAGGAAAGAGTGGCCGTCCAGTGAGAAGCTCG
GCAAATATGCATCCAATACTCCAAATATCAATAGCAGGAGTGTATTTCGAGAAAAAGGAGCCACATAATTCAGGGGC
CCTGTACCACCTTGTTGCTACATAATCCGTCCAAAATATAGCTGAAGGAGCATCATTAAATGATACACGCGCAAGTC
CAAAGTCACAAATTTTCAGTTTGCAGTCTGCGTTGGCCAGTATATTCTTGGGCTTCAGATCGCGATGAAATACATTA
GCCCCATGGATGTACTTGAGAGCGCGGAGAAGCTGGTAAAGGAAAAACTGGTAATGCTCAGCTGTGAGGTCATCATT
CGCTCTGATGACCTGATGGAGATCTGACTCCATCAGCTCGAAAACAACATATATATCTTGGAACTCCCTCCGAGAAG
GGGGGAGCATTATGTGCTTGATCTCTACCACGTCTGGATGACGTAGCAGCCGAAGGAGCTTGACCTCGCGGAGGATG
CGTGTGGCATCCGAGACGTGCTCAAACACGTCATTGATCTTCTTAATCGCAACCCGCTCGCCGGTGCGGGTATCTAT
TGCAGCAGCAACTACTCCATAACTCCCCTTGCCAACAACTTCTTGGATTTGGTACTGGCTCGCCTCTCCATACTCTG
TGAAGAATTCTTTCTCCATTATGTTCTTCTTGTGGCTCTCGACGGGGAGCGGCATCTTGCGCTTGGGGACGCGGATG
GGGCGGAGGCCCACGAGATCCAGGTCCTCGACGGCCTCGACGTCGGAGGAGTCCTCGCCGGCGGAAGACTGGTTGGA
GCTGCCGAGCACGGAGCCGGAGGGCGTGCGGCGGTGGAACAAGCGGCGGAAGCCGTCGACGATGCCGTTCCCTCCTC
C**CAT**GTATCCCCTCCCTACCCCTGCGGCATTGGCTAGCTGCCCGCCTCCTCCAGCCACAGCCCACAGCTCCTCCTCC

TACTACTACTACTACTCTTCTCGCCTCCCCCTTCCGCCGCCACTCACTGGCTCGAGATGGAGTGCTCGGCCGCTATC
TCCGGCGGTGTCGTCGGCGGTGAGGATGGA

>TsMPK20-1 : contig53750
GGGCTGCCCTGCGCTGTAAACCTTGTGGCATCCCCACCCAAGCTTGATCCGCCACTTCGTTTCTCGCTGTTTCGTTA
AGCCACCAGCCGCACTCCAGGAAGGCATGTCGGATGCGCCTGACTGGCCACTAATACATCTGCGACATACTGTAGGG
AACAACGCCGACCTTCCTGTGAGCACTTGCCGCCACTCCTGTTGTAGTTGGAGCTATGCCATTAAGGGATCTCGAGT
ATAGGTTTGATTCCGCTGATAGCCTA**TCA**GAAGAACCCCTTTTCGGCCCTGCTGACTGATGGAAGGGAGGTGCTCTC
ATGTCCAACATCAGGCACAGTTGAACTGTTTGCGCAAGCATAGGCCTGCTGGTGCAGCGTATACATTTCGGCCTGCG
ATGGCTCGGAGCAAGCTGACTTTGCCGGTGTCTGATAATAACCGTATATTTGCGGGATTTGTTGTTGGGGAGGATAA
CCGGAGCTCATTGCTGCCACTCTTCTTGGGTCATATGTATCCTTCATGCCACCATTTTCGTAAGGCAGAACTGGACC
AACAACCCTTCCTGGTCTTGCTGTTGGCACTCTTTGTGGAGCCTGTGAGGTTCTCTGGGCATTGCCAGAATATTTTT
CTGAATCTCGATGATTTTTGTAAGACTCGTCAGGCGAAGGCTTATCCCTTGATGGGCCAATACGGGGTTGTTCCTTG
CCATGAATTGGAGCTGAGTGAACAATAGTAGACCTAGGAAGAGAGGTGTGCTTTCTATCCATTGGAACTACAGGACC
ATTCTCACTGTTTTCTTCAAGATGGGCAAACTGCTTCCTAAATTGATCAACAGCACTTGGGTATAGAAAGGTTGTCC
TTTCAGTGCCATTGGTGTAGTCTTTGAGCAATTGTGGATGGTATTCCAATATCTCACGAAATATAAGCTCCCTTATG
TCTTCTTTTGTCACTCTTCTACGCTCAAACTCAAACTCCATTTTTGTGATTGGTTGGCAGGATGGTTCTCTCTCAAC
CTTGGCAAGCCCTTTAAAGTACGGATGAGACAATGCCTCTTCAGCAGTTGGGCGGTCCTTTGGATCGAACGCTAAAA
GCCTTTGCAACAGGTCTAAGGCCAAAGGATCTGCATTGAGAAATTTATGCGAAAATGAAATTGGCTCTTTCTTTCTC
ATGCTGCTCAGATACCTCCTTGCTTTCTCATTCCGGACCCGAGAGATTGTATCCATAGATGGTGTGCCCAGAAGGTC
AGTCATCAAATCTAACTGATGTACAACATTTTTACCAGGAAACAAAGGTTTTCCTGTCAAGACCTCAGCAAAGATGC
ATCCGATGCTCCAGACATCAATGGCGGGTGTATACTTTGAAAAGAAAGAACCACAGAGCTCTGGAGCTCTATACCAT
CTTGTCGCAACGTAATCCGTCCAAAAGATTGTTGTGGGGGTATCGTTGAATGCAACTCGTGCCAATCCAAAATCACA
AATCTTCAGTTTGCAGTTGGAATTTGCCAATATATTCTTTGGCTTTAGGTCCCGGTGATAAACATTAGCAGTGTGAA
TGTACTTCAGAGCCCGGAGTAATTGATAAAGAAAGAACTGGTAATGCTCCTTTGTCAAGTCATCATTAGCCTTTATA
ACCTGGTGGAGATCAGACTCCATGAGCTCAAAAACGACATAAATATCTTTGAAGTCTCTTCTTGATGGAGGTAACAT
GATATGCTTGATCTCAACAATGTCAGGATGTCTTAAGAGCCTCAGAAGCTTGATCTCACGGAGAATCCGTGCAGCAT
CAGATATGTGCTCAAAGATATCATGTATCTTTTTTATTGCCACTTTCTCTCCCGTGTGGACGTCTATTGCGGAGCAC
ACGACACCATAGCTGCCCTTTCCAACGATTTCCTGAATCTTGTATCGACTTGCGTCGCCGTACTCCGTAAAAAACTC
TGCCTCTGCGGAACTCTTCTTGCGCTGATCCTGCTG**CAT**CTCGCCGCTCACCGGATCTCGCCCCGAATCTCCTCGGC
TCCCCCGTACCGTAAGGGCGGCAACAACAACAGCACCAAAGCCCGCGCCTCCGAATCGACACGGTCAGACACACGGC
ATTTGCTGGCGCCGGGCCGAAAAGCGCACACGCCGCAGGGCGCCGGCGGGCGGATCGTGAGACGAGACGAGGCGACG
AAGGGAGGGCGGGAAACTGGGGGGAGACCCTAACCCTAGCGACGGGGCGCTGGGGCAGGGCTAGGGCTCCCGCGCGC
TCTCTCTCCTGTTCCTCCTCCTCTCCTGGTCATGTACCGCGCTCCGGGGAGGGGAATAA

>TsMPk20-1 : contig64364
TACATGGGGGCCCGCGCAAACGCATTGTGGCCGTTGCTTTTCCTTGTCGGTGGGCAAAGCTGTTGGCTTTTCTTGTG
GGGTTCTCTTGTACATGGGGGTGCACGACCACTTGACTCACAAGTGGCGCGTGTCACCATTTCAATTTCGCAACCAA
CACTTCAAGATCTTGCTTCTCAAGAAAATTTCTTTGATCTACCTGCACAGCAGTTGAATTTGGCATCAATTCCTTAA
TACTGGTTGTAGCTTCCCAATGATTACTTATTTTACATGTCGTCCATTAAAAGTATCTGGAGAGTTCCGCAGAGGCA
GAGTTTTTTACGGAGTACGGCGACGCAAGTCGATACAAGATTCAGGAAATCGTTGGAAAGGGCAGCTATGGTGTCGT
GTGCTCCGCAATAGACGTCCACACCGGAGAGAAAGTGGCGATAAAAAAGATACATGATATCTTTGAGCACATATCTG
ATGCTGCACGGATTCTCCGTGAGATCAAGCTTCTGAGGCTCTTAAGACATCCTGACATTGTTGAGATCAAGCATATT
ATGTTACCTCCATCGAGAAGAGACTTCAAAGATATTTATGTCGTTTTTGAGCTCATGGAGTCTGATCTCCACCAGGT
TATAAAGGCTAATGATGACTTGACAAAGGAGCATTACCAGTTCTTTCTTTACCAATTACTCCGGGCTCTGAAGTACA
TTCACACTGCTAATGTTTATCACCGGGACCTAAAGCCAAAGAATATATTGGCAAATTCCAACTGCAAACTGAAGATT
TGTGATTTTGGATTGGCACGAGTTGCATTCAACGATACCCCCACAACAATCTTTTGGACGGATTATGTTGCGACAAG
ATGGTATAGAGCTCCAGAGCTCTGTGGTTCCTTCTTTTCAAAGTATACACCCGCCATTGATGTCTGGAGCATCGGAT
GCATCTTTGCTGAGGTCTTGACAGGAAAACCTTTGTTTCCTGGTAAAAATGTTGTACATCAGTTAGATTTGATGACT
GATCTTCTGGGCACACCATCTATGGATACAATCTCTCGGGTCCGGAATGAGAAAGCAAGGAGGTATCTGAGCAGCAT
GAGAAAGAAAGAGCTGATTTCATTTTCGCATAAATTTCCCAATGCAGATCCTTTGGCCTTAGACCTGTTGCAAAGGC
TTTTAGCGTTTGATCCAAAGGACCGCCCAACTGCTGAAGAGGCATTGTCTCATCCGTACTTTAAAGGGCTTGCCAAG
GTTGAGAGAGAACCATCCTGCCAACCAATCACAAAAATGGAGTTTGAGTTTGAGCGTAGAAGAGTGACAAAAGAAGA
CATAAGGGAGCTTATATTTCGTGAGATATTGGAATACCATCCACAATTGCTCAAAGACTACACCAATGGCACTGAAA
GGACAACCTTTCTATACCCAAGTGCTGTTGATCAATTTAGGAAGCAGTTTGCTCATCTTGAAGAAAATAGTGAGAAT
GGCCCTGTAGTTCCAATGGATAGAAAGCACACCTCTCTTCCTAGGTCTACTATTGTTCACTCAGCTCCAATTCATGG
CAAGGAACAACCCCGTATTGGCCCATCAAGGGATAAGCCTTCGCCTGACGAGTCTTATAAAAATCATCGAGATTCAG

AAAAATATTCTGGCAATGTCCAGAGAACCTCACAGGCTCCACAAAGAGTGCCAACAGCAAGACCAGGAAGGGTTGTT
GGTCCAGTTCTGCCTTACGAAAATGGTGGCATGAAGGATACATATGACCCACGAAGAGTGGACAAGACAAATAGAAA
ACTACCGGTTAATACCTACCCCAAACAACAAATCCCGCAAATATACGGTTATTATCAGACACCGGCAAAGTCAGCTT
GCTCCGAGCCATCGCAGGCCGAAATGTATACGCTGCACCAGCAGGCCTATGCTTGCGCAAACAGTTCAACTGTGCCT
GATGTTGCTCTGGACATGAGAGCACCTCCGTTCCATCAATCAGCAGGGCCGAAAAGGGGTTCTTCTGATAGGCTATC
AGCGGAATCAAACCTATACTCGAGATCCCTTAATGGCATGGCTCCAACTACAACAGGAGTGGCGGCAAGTGCTCACA
GGAAGGTCGGCGTTGTTCCCTACGGTATGTCGCAGATGTAT**TAG**TGGCCAGTCAGGCGCATCCGACATGCCATCCTG
GAGTGCGGCTGGTGGCTAACGAAACAGCGAGGAACGAAGTGGCGGATTAAGCTTGGGTGGGGATGCCGCAAGGTTTA
CAGCGCAGGGCAGCCCATGGTTTGATGCTTATGTGTGCTGTCTGATCTGGAAGCGTGTGAAGCTCTTCGTTCTGTTC
CGGGCCATCAGCTTGTTGTATCTGCCTGTGTGCACACTGGATGGGGGTGGGTAGTCAGTCCAGTGGCTTGTAATCTG
AGCTATTTAGGCGCCGGTTACTTTTTCTAGGGCGAGGAGACAGAAGGGGAAGGAAGGAATGCTAACCCGTTTTCTGT
TGTAGAGATGTTTGTCATCAATGTGCAACTTCAGAAAGATATATATAAACTTATAAAGGACACTTTTGCTGCAGGTG
ATAATTGCCACCACGTAGTCG

>TsMPK20-1 : contig185846
TTTTGCCATATATAGAACCGGTGGCCACTGATTCGACTACGTGGTGGCAATTATCACCTGGAGCAACAGTGTGCTTT
ATAAGTTTATATATATCTTTCTGAAGTTGCACATTGATGACAAACATCTCTATACAACAGAAAACGGGTTAGCAATT
CCTTCCTTTCCCTTCTGTCTCCTCGCCCAAGAAAATGTAACCGGCGCCTAAATAGCTCAGATTACAAGCCACTGGAC
TGACTACCCACCCCCATCCAGTGTGCACACAGGCAGATACAACAAGCTGATGGCCCGAAACAGAACGAAGAGCTTCA
CACGCTTCCAGATCAGACAGCAAAGATGAGCATCAAACCCTGGGCTGCCCTGCGCTGTAAACCTTGCGGCATCCCCA
CCCAAGCTTGATCCGCCACTTCGTTTCTCGCTGTTTCGTTCGCCACCAGCCGCCTTCCAGGAAGGCATGTCGGATGT
GCCTGATTGGCCA**CTA**ATACATCTGCGACATGCCGTAGGGAACGACGCCGACCTTTCTGTGACCGCTTGCCGCCACT
CCTGTTGTAGTTGGAGCCATGCCATTAAGGGATCTCGTGTATAGGTTTGATTCTGCTGATAGCCTATCAGAAGAACC
CCTTTTCGGCCCTGCTGACTGATGGAAGGGAGGTGCTCTCATGTCCAGAGCAACATCAGGCACAGTTGAACTGTTTG
CGCAAGCATAAGCCTGCTGGTGCAGCGTATACATTTCGGCCTGCGATGGCTCTGAGCAAGCTGACTTTGCCGGTGTC
TGATAATAACCGTATATTTGCGGGATTTGTTGTTGGGGAGGATAACCTGAGCTCATTGCTGCCACTCTTCTTGGGTC
ATATGTATCCTTCATGCCACCATTTTCGTAAGGCAGAACTGGACCAACAACCCTTCCTGGTCTTGCTGTTGGCACTC
TTTGTGGAGCCTGTGAGGTTCTCTGGGCATTGCCAGAATATTTTTCTGAATCTCGATGATTTTTGTAAGACTCGTCA
GGCGAAGGCTTATCCCTTGATGGGCCAATACGGGGTTGTTCCTTGCCATGAATTGGAGCTGAGTGAACAATAGTAGA
CCTAGGAAGAGAGGTGTGCTTTCTATCCATTGGAACTACAGGACCATTCTCACTGTTTTCTTCAAGATGGGCAAACT
GCTTCCTAAATTGATCAACAGCACTTGGGTATAGAAAGGTTGTCCTTTCAGTGCCATTGGTGTAGTCTTTGAGCAAT
TGTGGATGGTATTCCAATATCTCACGAAATATAAGCTCCCTTATGTCTTCTTTTGTCACTCTTCTACGCTCAAACTC
AAACTCCATTTTTGTGATTGGTTGGCAGGATGGTTCTCTCTCAACCTTGGCAAGCCCTTTAAAGTACGGATGAGACA
ATGCCTCTTCAGCAGTTGGGCGGTCCTTTGGATCGAACGCTAAAAGCCTTTGCAACAGGTCTAAGGCCAAAGGATCT
GCATTGAGAAATTTATGCGAAAATGAAATTGGCTCTTTCTTTCTCATGCTGCTCAGATACCTCCTTGCTTTCTCATT
CCGGACCCGAGAGATTGTATCCATAGATGGTGTGCCCAGAAGGTCAGTCATCAAATCTAACTGATGTACAACATTTT
TACCAGGAAACAAAGGTTTTCCTGTCAAGACCTCAGCAAAGATGCATCCGATGCTCCAGACATCAATGGCGGGTGTA
TACTTTGAAAAGAAAGAACCACAGAGCTCTGGAGCTCTATACCATCTTGTCGCAACGTAATCCGTCCAAAAGATTGT
TGTGGGGGTATCGTTGAATGCAACTCGTGCCAATCCAAAATCACAAATCTTCAGTTTGCAGTTGGAATTTGCCAATA
TATTCTTTGGCTTTAGGTCCCGGTGATAAACATTAGCAGTGTGAATGTACTTCAGAGCCCGGAGTAATTGATAAAGA
AAGAACTGGTAATGCTCCTTTGTCAAGTCATCATTAGCCTTTATAACCTGGTGGAGATCAGACTCCATGAGCTCAAA
AACGACATAAATATCTTTGAAGTCTCTTCTTGATGGAGGTAACATGATATGCTTGATCTCAACAATGTCAGGATGTC
TTAAGAGCCTCAGAAGCTTGATCTCACGGAGAATCCGTGCAGCATCAGATATGTGCTCAAAGATATCATGTATCTTT
TTTATTGCCACTTTCTCTCCCGTGTGGACGTCTATTGCGGAGCACACGACACCGTAGCTGCCCTTTCCAACGATTTC
CTGAATCTTGTATCGACTTGCGTCGCCGTACTCCGTAAAAAACTCTGCCTCTGCGGAACTCTTCTTGCGCTGATCCT
GCTG**CAT**CTCGCCGCTCACCGGATCTCGCCCGGAATCTCCTCGGTTCCCCTGTGCCGTACGGGCGGCAACGACAACA
aCAACACCAAGCCCACCTCCGAATCACCACGGTCAGACACACGGCATTTGCT

>TsMPK20-2 : contig111052
AAATGAATCATATGATGATGATCAATGGACCATCATCCCTTTAATCAATTAATCAGTCTGTTGGATAGTAAGCAGTA
GCACAGTACAGACTTCATTTCTGTGTTGAGTTGACCTGTCTCCAGCCGGCCGCTAACCGGCTAATTAATTTTCATTT
CCACCTAAATAAACAGCTCGTCTGGCCACACCCGTCCACGGTCCACCCTCCGTCTTTTTGTGTGTAGCCTAGAGACG
ACATGGAACAGGATTTCAGACCTACTATCTCTGTATGCCTTCGTCGTCGTCGTCGTCGTCAGACGATCCTCATCCGC
CAACTTATTTCAGGCCAATGCCTCCCTCCGGTTGCTACGGTTCCGGCCGAAGTGTCTAGAGACGCGCCGCTGCCATC
TTCTTAGACG**CTA**GTAAGTCGTCGGCATGCCAAACGGGACGGCGCCAACCTTCCTGTGGGCGCTGGCAGTGCCAGCC
GATGCTGCGGCGGCGGCGACGATGCCGTTGAGCGACCGCGTGTATATGTCGGTGGTCTCCACCGCCAGTCTGTCCAG
CGGACCGTTCTTCTGCCCCGCCGGAAGGTGGCGGTAGGGGGGTGCTCTCATGGCTGGAGCAGCAGCAGCAGCATTCG

GCGCTGTGCCTTTGGTGTATCCACCACAGGCATAGCCCCCCATGGTTTGTGGTATCCCAGCGCCAGCACCAGGAGCT
GCTTGATGCTGATATTGCCCATACAATTGTGGGATCTGCTGCTGAGGAGGGCATCCTGAGCTCATCGCCGGCCTGCT
TGCTGCGTCATAAGGGTATCTGCCGACTCCAGCTTCACTGGGTAATGCTGAGCCAGCAGCTCTCCGTGAAACCACTG
CTTGTGCCGCGTGTGAAGCCTGAGAAGCACCGTGACCATTCCCAGCATACCTCCCTGTCTCCGAGGAAGTTTTGCTG
CAAGGCTTGCCAAAGAGCGGCCGAGTGTCCTTCGCGGGAATTGGAGTCGAGTGAACAATAGTGGACCTAGGAAGAGA
TGCATGTTTTCTATCTGCTGGGACTACAGGGCCGCTACCATCGCTTTCTTCGAGATGAGAGAACTGCTTCTTAAAAT
GATCGACAGCACTTGGGTAGAGAAAGGTTGTCCTCTCTGTGCCATCAGTGTAGCTCTTCAGCAATTGCGGATGATAT
TCCAATATCTCCTGGAATATCAGCTCCCTTATATCATCCTTCGACACTCTGCTGTGCTCAAAGTCAAACTCCATTTT
CTGATTGGCTGACAGGATGGTTCTCTCTCTGGCTTGGAAAGGCCTCTAAAGTATGGATCAGTCAATGCCTCTTCCGC
AGTTGGACGGTCTTTTGGATCAAAGGCTAATAGCTTCTCCAAGAGTTTTACTCCCAAAGGATCTGCATTAGGAAACT
TCTGAGAAAATGGGACCGGGTCTTTCTTTCTCATACTGCTCAAGTACCTTCTTGCTTTCTCGTTTCGGACCCGAGAA
ATTGTATCCATTGAAGGTGTGCCTAGAAGATCAGTCATCAAGTCTAACTGATGGACAACATTTTTACCAGGAAACAA
AGGCTTTCCTGTCAGCACCTCAGCAAAAATACACCCAATGCTCCAGATGTCAATGGCAGGTGTATACTTTGTGAAGA
AGGATCCGCAGAGCTCTGGAGCTCTATACCATCTTGTCGCGACATAATCCGTCCAGAAGACAGTTGTTGGGGTATCG
TTGAATGCAACTCGTGCTAGTCCAAAATCACATATCTTCAATTTGCAGTTAGAATTTGCCAAAATATTCTTGGGCTT
CAGGTCACGATGGTAAACACTAGCAGTATGAATGTATTTGAGGGCTCGGAGTAGCTGGTAGAGAAAGAACTGGTAAT
GCTCTTTTGTCAAGTCATCGTTGGCCTTAATAACTTGGTGGAGATCAGACTCCATGAGCTCAAAAACAACATAAATG
TCTTTGAAGTCCTTTCTGGAGGGAGGCAACATAATGTGCTTGATCTCGACGACGTCGGGATGCCGCAAGAGCCTCAG
AAGCTTGATCTCACGGAGAATCCGCGCGGCGTCGGAGGTATGCTCGAAAATATTGTGTATCTTCTTGATGGCCACTT
TCTGCCTGGTCTGCAGATCAAGGGCAGAGCACACAACCCCATAGCTCCCCTTGCCAACCTCCTGGATCTTGTACCGG
TTGGCGTCGCCGTACTCCGAGAAGTCCACCTCCGCCGAACTCTTGGTCCGCTGCGCCTGCTG**CAT**TGCCGCTTGCCG
GGCGGGCCGATCTGCGCTCCAAGGCCGCTCCGGAAGCCGGACCCCTGCCCTCCCGCGGCGCCGGACCCCCCGCCTCC
CGGTCGGTCGGCCGAGCCGCCCGCCGAACTCACACGTATGCCGCGGGCCGATGCCGGGCCCAGGGTGCCGGACGGAC
TCTTCTGGCCCGATCTGGCGGCGCGCCGGAGAGAAGGGAGGGGAGCGGGATG

Comment [FN1]: Deletion? Requires an extra T in order to translate into full length MAPK sequence.

>TsMPK20-2 : contig113411
AAATGAATCATATGATGATGATCAATGGACCATCATCCCTTTAATCAATTAATCAGTCTGTTGGATAGTAAGCAGTA
GCACAGTACAGACTTCATTTCTGTGTTGAGTTGACCTGTCTCCAGCCGGCCGCTAACCGGCTAATTAATTTTCATTT
CCACCTAAATAAACAGCTCGTCTGGCCACACCCGTCCACGGTCCACCCTCCGTCTTTTTGTGTGTAGCCTAGAGACG
ACATGGAACAGGATTTCAGACCTACTATCTCTGTATGCCTTCGTCGTCGTCGTCGTCGTCAGACGATCCTCATCCGC
CAACTTATTTCAGGCCAATGCCTCCCTCCGGTTGCTACGGTTCCGGCCGAAGTGTCTAGAGACGCGCCGCTGCCATC
TTCTTAGACG**CTA**GTAAGTCGTCGGCATGCCAAACGGGACGGCGCCAACCTTCCTGTGGGCGCTGGCAGTGCCAGCC
GATGCTGCGGCGGCGGCGACGATGCCGTTGAGCGACCGCGTGTATATGTCGGTGGTCTCCACCGCCAGTCTGTCCAG
CGGACCGTTCTTCTGCCCCGCCGGAAGGTGGCGGTAGGGGGGTGCTCTCATGGCTGGAGCAGCAGCAGCAGCATTCG
GCGCTGTGCCTTTGGTGTACCCACCACAGGCATAGCCCCCCATGGCCTGTGGTATCCCAGCACCAGCAGGTGCTTGA
TGCTGATATTGCCCATACATTTGTGGGATCTGCTGCTGAGGAGGGCATCCTGGGCTAATCGTCGGCCTGCTTGCCGC
GTCATAAGGTAACGCTGAACCAGCAGCTCTCCGTGAAACCACTGCTTGTGCCGCATGTGAAGCCTGAGAAGCACCGC
GACCGTTCCCAGCATACCTCCCTGTCTCCGAGGAAGTCTTGCTGCAAGGCTTGCCAAAGAGCGGCCGAGTGTCCTTC
GCGGAAATTGGAGCCGAGTGAACAATAGTGGACCTAGGAAGAGATGCGTGTTTTCTATCTGCTGGGACTACAGGGCC
ACTACCATCGCTTTCTTCGAGATGAGAGAACTGCTTCTTAAAATGATCGACAGCACTTGGGTAGAGAAAGGTTGTCC
TCTCTGTGCCATCTACGTAGCTCTTCAGTAATTGCGGATGATATTCCAATATCTCCTGGAATATCAGCTCCCTTATA
TCATCCTTTGACACTCTGCTGTGCTCAAAGTCAAACTCCATTTTTCTGATTGGCTGACAGGATGGTTCTCTCTCTGG
CTTGGAGAGGCCTCTAAAGTATGGATCAGTCAATGCCTCTTCCGCAGTTGGACGGTCCTTCGGATCAAAAGCTAATA
GCTTCTCCAAGAGTTTTACTCCCAAAGGATCTGCATTAGGAAACTTCTGAGAAAATGGGACTGGGTCTTTCTTTCTC
ATACTGCTCAAGTACCTTCTTGCTTTCTCGTTTCGGACCCGAGAAATTGTATCCATTGAGGGTGTGCCTAGAAGATC
AGTCATCAAGTCTAACTGATGGACAACATTTTTACCAGGAAACAAAGGTTTTCCTGTCAAGACCTCAGCAAAGATGC
ATCCGATGCTCCAGACATCAATGGCGGGTGTATACTTTGAAAAGAAAGAACCACAGAGCTCTGGAGCTCTATACCAT
CTTGTCGCAACGTAATCCGTCCAAAAGATTGTTGTGGGGGTATCGTTGAATGCAACTCGTGCCAATCCAAAATCACA
AATCTTCAGTTTGCAGTTGGAATTTGCCAATATATTCTTTGGCTTTAGGTCCCGGTGATAAACATTAGCAGTGTGAA
TGTACTTCAGAGCCCGGAGTAATTGATAAAGAAAGAACTGGTAATGCTCCTTTGTCAAGTCATCATTAGCCTTTATA
ACCTGGTGGAGATCAGACTCCATGAGCTCAAAAACGACATAAATATCTTTGAAGTCTCTTCTTGATGGAGGTAACAT
GATATGCTTGATCTCAACAATGTCAGGATGTCTTAAGAGCCTCAGAAGCTTGATCTCACGGAGAATCCGTGCAGCAT
CAGATATGTGCTCAAAGATATCATGTATCTTTTTTATCGCCACTTTCTCTCCGGTGTGGACGTCTATTGCGGAGCAC
ACGACACCATAGCTGCCCTTTCCAACGATTTCCTGAATCTTGTATCGACTTGCGTCGCCGTACTCCGTAAAAAACTC
GGCCTCTGCGGAACTCTTCTTGCGCTGATCCTGCTG**CAT**CTCGCCGCTCACCGGATCTCGCCCAGAATCTCCTCAGC
TCCCCCGTACCGTAAGGTAAGGGCGGCAACGACAACACCAAGCCCGCCTCCGAATGGACACGGTCAGACACACGGCA

TTTGCCGGCGCCGGGCCGGGCCGGCGAGGACGAAAAGCGCACACGCCGCAGGGCGCCGGCGGACGGATCGTGAGACG
AGACGAGACGAGGCGACGAAGGGAGGGCGGGAAAACTGGGGGGGGAGACCCTAACCCTAGCGACGGG

>TsMPK20-3 : contig68811
AGAGGAGGAAAGAGAAGAGAAGAGAAAATATCCCTCCCACCTCGTCACCCCAGCTTCCTCCGCTGCCTCACCACAGC
ACGCCGACGAGCTCTCTCCCACGCACGACGCCCCCTCCCCCTCCCCCCAGATCCGAAGCGACTGCCGCCGGCTCGCG
ATCTTCCCGAACCGTACAAAAACCCGGCCGTGGAGGCGGCCACGGCAGCCACGTACGGTGGCGGCGAGGGGTGTATG
AGGAGCAGGAACGACTGCGGGAACGCGATTCGGAGCAGGAGCCGGTGGGGCAAGTGCGGCTGACCCGGCCTGAGCAA
GGACATCCACGGCCGGTCTGTCTGTCTGGCGGGATCGTGGCAGGAGGGAAAAGAAAGAAAGAAAGGTCGATCTTGGG
AAGCTTCCACTACGGGCTCCGAG**ATG**CAGAACAGCGATTTGCGCAAGAAGAGTGCAGCGGAAATTGATTTCTTTACT
GAATATGGCGATACTAATCGATACAAAGTTCTGGAGGTCATAGGCAAAGGTAGTTATGGACTTGTATGTTCTGCAAA
TGATACACAAACAGGAGAGAAGGTTGCAATAAAGAAGATACACAACATTTTTGAGCATATATCGGATGCTGCACGCA
TACTCCGTGAAATCAAGCTTCTCAGGCTTCTTAGGCACCCTGATGTAGTGGAAATAAAGCATATCTTGCTCCCCCCA
TCCAAAAAGGATTTCAAAGATATATATGTTGTCTTTGAACTTATGGAGTCAGATCTTCATCAAGTAATAAAGGCTAA
TGATGATTTGACGAGGGAGCATTATCAGTTTTTCTTGTATCAGATGCTTCGAGCTTTGAAATATATGCACACAGCAA
ATGTCTATCACCGAGATTTGAAGCCCAAGAATGTGCTTGCCAATGCAAATTGCAAACTCAAAATTTGTGACTTTGGC
TTGGCGAGAGTTGCATTCAATGATGCACCTACGACAGTCTTCTGGACAGATTATGTGGCAACGAGATGGTATAGAGC
ACCTGAACTTTGTGGGTCCTTCTATTCTAAGTATACACCAGCTATTGATATATGGAGTATAGGGTGCATTTTTGCCG
AGGTGTTGATTGGAAAGCCTCTATTTCCTGGTAAAAATGTTGTTCACCAGCTGGATTTGATAACTGATGTTCTGGGG
ACACCTTCATTAGATGCTATTTCTCAGGTGCGGAATGACAAGGCAAGAAAATATCTGACATGCATGCGGAAGAAACA
GCCTGCTTCGTTTTCGCAGAAATTTCCAAAGGCTGACCCATTAGCATTACGATTGCTTAGGAGGCTTCTAGCTTTTG
ATCCAAAGGATCGTCCCTCTGCTGAAGAGGCATTGGCAGATCCATACTTTAATGGACTAGCAAAGGTAGAGCGAGAA
CCATCTTGTCAACCGATACCAAAAATTGAATTTGAATTTGAGGGTCGTAGAGTAACAAAGGAGGACATCAAGGAACT
GATCTTTGAAGAAATTTTGGAGTATCATCCTCAATTACTGAAGGAGCATATCAGTGGAACAGACAGACGAAACTTTG
TTCATTTAAGTGCTGTTGACCAATTTAAGAAACGCTTCGCTGAACTCGAGGAAAATGGTGGCGAAAATGGATCAGCT
GTTTCATCACAGAGGAAACATTCTTCTTTGCCAAGGCAATCCTTCAACCCCCAT**TAG**ACGTGTAATGACCTGTGACA
GTGAAAACTCACAGGATATGAAGATCCAAGAAGCTTACCATCTGTACTCCCTCGGCAACCAAAATATGAAGCACCGC
ACTCTCAGAACCAAGTATCTAGTGGGTCAGTGCTTGATGCGACAGGTCCAACTCCCAACACAATATGTACATGGAGG
AGTATATGTAGACTCCAGAACAGGGAACTTACTTCTATCTGCATCATGGTATAAGGGTTGATATACCAGGTGAGGAG
TATATGTAGACTCCAGATCAGGGAACTTACTTCTATCTGCATCATGGTATAAGGGTTGATATACCAGGTGACAGTAT
CGCACAGGCAGTCTCTGTTCCTCATGGCTCAGTTCTGGCTGTATGTTGCAGCATGGCGTTTCCAGGGATGTTTCGAG
CCTAGGGTTATTTGGCATTGAATCATGGAGGTCTGGCTTTTTTATTTGTTAGTCCCCCAAATCATGGAGGTCGAACC
TAGGATTCCAGCTTCATCTTGTACATGGGATGTTTCACAGCTTCTTTGCAACCAAGCCCCCCAAATGTACCACACAG
TTGGATCATAATGTGCCTCCTAGGGATTGAGTTGCAAAGCTCGTTTGTTCTGAATGCTTAAACTTTTTCCATGGGAA
GCTGTGAGGTGGCTAGGTCAAAGAATAGGATTGTCGTGATTTACTTCAACCGCTAATATCAGTTGTTTAGGAAATAC
TCCTGCATTTTAACGCGAGGGACCTTGCTGTGTTTAGCAGCGTGTTAACCAAAGGAACTTGAGCATATAATCTGAAT
CATTTCCAATAGTTACTGTAAAGGAATGTGCGAAGGTCGGATGCCATAGACCAAATTAACTGTTCCGGTGAGCTGGG
TAAATAAACTGTTCTTTTCCATCGAAAA

>TsMPK20-3 : contig19795
AGAGGAGGAAAGAGAAGAGAAGAGAAAATATCCCTCCCACCTCGTCACCCCAGCTTCCTCCGCTGCCTCACCACAGC
ACGCCGACGAGCTCTCTCCCACGCACGACGCCCCCTCCCCCTCCCCCCAGATCCGAAGCGACTGCCGCCGGCTCGCG
ATCTTCCCGAACCGTACAAAAACCCGGCCGTGGAGGCGGCCACGGCAGCCACGTACGGTGGCGGCGAGGGGTGTATG
AGGAGCAGGAACGACTGCGGGAACGCGATTCGGAGCAGGAGCCGGTGGGGCAAGTGCGGCTGACCCGGCCTGAGCAA
GGACATCCACGGCCGGTCTGTCTGTCTGGCGGGATCGTGGCAGGAGGGAAAAGAAAGAAAGAAAGGTCGATCTTGGG
AAGCTTCCACTACGGGCTCCGAG**ATG**CAGAACAGCGATTTGCGCAAGAAGAGTGCAGCGGAAATTGATTTCTTTACT
GAATATGGCGATACTAATCGATACAAAGTTCTGGAGGTCATAGGCAAAGGTAGTTATGGACTTGTATGTTCTGCAAA
TGATACACAAACAGGAGAGAAGGTTGCAATAAAGAAGATACACAACATTTTTGAGCATATATCGGATGCTGCACGCA
TACTCCGTGAAATCAAGCTTCTCAGGCTTCTTAGGCACCCTGATGTAGTGGAAATAAAGCATATCTTGCTCCCCCCA
TCCAAAAAGGATTTCAAAGATATATATGTTGTCTTTGAACTTATGGAGTCAGATCTTCATCAAGTAATAAAGGCTAA
TGATGATTTGACGAGGGAGCATTATCAGTTTTTCTTGTATCAGATGCTTCGAGCTTTGAAATATATGCACACAGCAA
ATGTCTATCACCGAGATTTGAAGCCCAAGAATGTGCTTGCCAATGCAAATTGCAAACTCAAAATTTGTGACTTTGGC
TTGGCGAGAGTTGCATTCAATGATGCACCTACGACAGTCTTCTGGACAGATTATGTGGCAACGAGATGGTATAGAGC
ACCTGAACTTTGTGGGTCCTTCTATTCTAAGTATACACCAGCTATTGATATATGGAGTATAGGGTGCATTTTTGCCG
AGGTGTTGATTGGAAAGCCTCTATTTCCTGGTAAAAATGTTGTTCACCAGCTGGATTTGATAACTGATGTTCTGGGG
ACACCTTCATTAGATGCTATTTCTCAGGTGCGGAATGACAAGGCAAGAAAATATCTGACATGCATGCGGAAGAAACA
GCCTGCTTCGTTTTCGCAGAAATTTCCAAAGGCTGACCCATTAGCATTACGATTGCTTAGGAGGCTTCTAGCTTTTG

ATCCAAAGGATCGTCCCTCTGCTGAAGAGGCATTGGCAGATCCATACTTTAATGGACTAGCAAAGGTAGAGCGAGAA
CCATCTTGTCAACCGATACCAAAAATTGAATTTGAATTTGAGGGTCGTAGAGTAACAAAGGAGGACATCAAGGAACT
GATCTTTGAAGAAATTTTGGAGTATCATCCTCAATTACTGAAGGAGCATATCAGTGGAACAGACAGACGAAACTTTG
TTCATTTAAGTGCTGTTGACCAATTTAAGAAACGCTTCGCTGAACTCGAGGAAAATGGTGGCGAAAATGGATCAGCT
GTTTCGTCACAGAGGAAACATTCTTCTTTGCCAAGGCAATCCTTCAACCCTCGT**TAG**ACGTGTAATGTCCTGTACAG
TCAAAACTCACAGGAAAGATCCAAGAAGCTTACCATCTGTACTCCCTTGGCAACCAAATATCAAGCACCGCACTCTC
AGAACCCAGTATCTAGTGGGCCAGTGCTTGATGCAACAGGTCCAACTCCCAACACAATATGTGCCTGGAGGAATGTG
TGTAGACTCCAGATCAGGGAACTTGTACTTCTATCTGCATCATGGTATAAGGGTTGATATACCAGGTCACAGTATCG
CACAGGCAGTCTGTTCCTAATGGCTCAGTTCCGGCTGTACGTTGCAGCATGGCGTTGCCAGGGATGTTTCGAGCTGG
GTTATCGGCATTGAATCATGGAGGACTGACTTTCTTATTTGTCAGTCCCCCAAATCATGGAGGTCGAACCTAGGATT
CCAGCTTCATCTTGTACATGGGATGTTTCACAGCTTCTTTGCAACCGAGCCCCCCAAATGTACCAGACAGTTGGATC
ATAATGTGCCTCCTAGGGATTGCGTTGCAAAGCTCGTTTGTTCTGAATGATGAAACTTTTTCCATGGGAAGCTGTGA
GGTGGCTAGGTCAAAGAACATGATCGTTGCGATTTACATCAACCAGTTGTTTAAGAAATACTCCTACATTTTAACGC
GAGGGACCTTGCTGTGTTTAGCAGTGTGTTAACCAAAGAAACTTGAGCATATAGTCTGAATCATTTCCAATAGTTGC
TGTAAAGAAATGTGCGAAAGGTCGAATGCCAGAGACCAAATTAACTGTTCTGGTGAGCTGGGTAAATAAATTGTTTG
TCTCCGTCGATATTGATGTTGTTGAGATTCTGTTCATACATCTTTTCTCAGATGTATGCCAATTGCTT

>TsMPK20-4 : contig251629
CTTACAAGTCGATCGCTCGCACCAGACGAACGAGACGAGACGGAGCTCCTTGTGTTTGCCCTTTGCGTTGCCCCCAC
CCTCACCGGGCCACAGGACGAAACCAACGCCACAACAACTCACACCCCCTTCGCCCGCGAGTTGCTCAGTCGCAGCG
AGCCGGGCCACAAGACACGAGCGTTCCGGTTCAGGCTGGGGTTGGGGTTGCGCTCGTTTCATCGTCGATCCGCCCAC
CCCGCCATACCGGCCCGGGGACAGAGATCTCTCCCTCGCGCGCGCGTGCCGGCGGCGACAAATGCGGCTTGATTCGC
CGGCGGACCAGCGGCGGTAAGCG**ATG**CAGCCGGACCAGCAACAGCAGCAGCAGCGGAGGAAGGGTTCACCGGAGATG
GACTTCTTCAGTGAATATGGCGATGCTAATAGATACAAGATTCAGGAAGTCATCGGTAAAGGGAGTTACGGTGTCGT
TTGTTCAGCTATTGACCAACATACTGGCGACAAGGTGGCAATCAAGAAAATACACAATATCTTTGAGCATTTATCTG
ATGCTGCTCGGATTCTCCGTGAGATCAAATTACTCCGCCTATTGAGACATCCTGATATAGTTGAGATCAGGCATATA
ATGTTGCCTCCGTCAAGGAGGGATTTCAAGGATATTTATGTCGTCTTTGAGCTGATGGATACAGACCTCCACCAAGT
CATCAAGGCCAACGATGACTTGACCAAAGAGCACCACCAGTTCTTTCTCTATCAGATGCTTCGTGCACTGAAATATA
TTCATACCGCTAATGTTTATCATCGTGATTTGAAGCCAAAGAATATATTGGCAAATGCTAACTGTAAACTCAAAATA
TGTGATTTTGGGCTAGCACGAGTGGCATTCAATGACACTCCCACGACTGTATTTTGGACGGATTATGTTGCTACTAG
ATGGTATAGGGCTCCTGAGCTTTGTGGATCTTTCTTTACTAAGTATTCACCAGCTATTGACATATGGAGTATTGGTT
GCATTTTTGCGGAGATTTTAACTGGGAAACCTTTGTTTCCTGGTAAAAATGTAGTTCACCAGTTGGATTTAATGACT
GATCTCTTGGGTACGCCGTCACTGGATACTGTTTCCAGGATCCGAAATGAGAAGGCAAGGAGGTACTTGAGTAGTAT
GAGGAAAAAACAACCGGTATGTTTTTCTGAGAGGTTCCCCAAAGCAGATGCTGCTGCACTCAAACTTATGCAGCGGC
TTTTAGCATTTGACCCCAAGGATAGACCAACGGCAGAAGAGGCGTTAGCTGATCCATATTTTAAAGGCCTTGGGAAG
GTAGAGAGAGAACCATCCTGCCAGCCAATATCGAAATTTGAGTTTGAGTTTGAACGGAAAAAGGTGACAAAAGAGGA
CGTAAAGGAACTTATATTCCGCGAGATATTGGAGTATCATCCTCAACTTCTCAAGGATTACATGAATGGAACTGAAA
AAACGAACTTCCTATATCCTAGTGCTGTAGACAATTTCCGGAGGCAATTTGCTAACTTGGAGGAAAATGGAGGGAAG
GGAGGGGCAATCGTTCCATCGGACAGGAAGCATGTTTCGCTCCCCGTTCTTTGGTCTCATGGTACTATGGAATCGCT
TTACATGACAAAAGATGGTCAGGTAGTCAGGAATTTCAGTTCAAGTTTGATGATTTGGCATGATAATTTCAGAGACT
GCCACTCAACTTTGCTTGCTGACATTTTTGCATTTGACAGGACTACTACAGTTCATTCTACACCAATTCCTCCAAAA
GATCAGAAGTCTTCCCAAGTTCCCCAAAGGATTCCAACAGGTAGACCAGGAAGAGTGGTTGGCCCGGTAATACCATT
TGAGAATTCATGTGCTATGGATCCTTACAGTCAACGAAGGGTGGCGAGGAATCCAGTACTTCCTGCAGCTGCTACCA
ATGTATCAGCATACGCATACCACCGAAAGTCAGACAGTTCAGAGAGAGAGTTACAGCAGGAGCTTGAAAAAGACCGC
ATGCAGTACCAGCCGATGCAGCGTTTCATGGATGCCAAGATGGTCTCCCCTGACTTGAGGTCTACCTCCTATTACAT
GCCAAAGGGTGTCCCAAAAGCCGATGTAGCAGAAAGGACTGGTTTGCAGCCAAACATGATGCAGGGAATTGCCCCGT
TTAATGGCATTGCTGCAGTTGGAGGTAGCTACAATAAGGCCAGTGCTGTTCAGTATGGAGTTTCAAGGATGTAC**TAA**
GTGGTCTAGTGCGATGGCCACTTCCAAAATGCTTGGCTTAGCTGAAGTAATAACATCGAAGAATCGAAGTACTTCCT
GAGTTGCCTTATGGGCGGAAGGTTCAGGCAGTGGATCGATGATCTTTGATCGAGGAGGAAGTTAATGTCTCGTAAAG
AACAAAAAAATAAGATCATGCAAAGACAACATCCTGCCTGCTGATCTAGCTGCCAAGGAAAGAATGGCATCTGGTAA
TTTGCTGCTAAGGGGAAAAGCCGAAGTTGGGGGTACAAGAAGATTGAGGTCTATGTAGTTTGAAAGAACGGAAACAT
ATCGGAATAATGGAAATGGTGAACTTCTGTACATAAATAACTTTGATTTTTTTTTTGTATTACTGTCGGCGAGAGCT
GCCAGTTTTGCTCCATGTTCATGTGGCAATGTGATTTATTTTTCTGGGTTTGAAGCACTTATGCAGTTATGCTTCTG
TAGGAGTTCAGCGCTGTAATACTTTGGAGTATGTTTAGATCTGTAATAGAATAACAAGACCAAACTTCAGTAAGATA
GAATGGTAATGAACTGCTAGTTTTGGGGGTTGTTGGCTCAACCGTGGTTAAAA

>TsMPK20-5 : contig67306
GCCTGCTTGGACTTGTTTAACACCCACCACCGCTCCCTCCTCCCTCCTCCGGGTGACGTCGCCGCCGACCGGGCCGG
ACCGGCGGCGAGGCGAGGCGCGGCCTTCTCTCTCCCCTGGGCGGCCGGCCTCCCGTGCTCCCCAGG**ATG**CCGGAGGC
AAATGCGGGTGGCCGCGGCGGCGGCGAGCAGCGCAGCAAGGTTCAGAGTTCAGACGTGATGAGTTTCTTCAGTGAAT
ATGGAGACGCCAGCAGATACAAGATCGAAGAAATAATTGGCAAAGGGAGTTATGGAGTGGTGTGTTCAGCAATCGAC
CGGCAGACCGGCGACAAGGTGGCCATAAAGAAGGTGTCCAACATCTTCGAGCATATAACCGACGCCGCTCGGATGCT
CCGCGAGATCAAGCTTCTCCGGCTTCTCAGGCACCCCGACATCGTCCAGATCAAGCACATTATGCTGCCTCCCTCCA
GGAGGGACTACAAGGACATATATGTTGTCTTTGAGCTCATGGACACCGACCTCCACCAGGTTATCAAGGCCAATGAT
GACCTCACAAAGGAGCACTACCAGTTCTTTCTCTACCAGATGCTCCGGGCGCTCAAATACATCCATACAGCGAACGT
CTATCATCGTGATTTGAAGCCAAAAAACATATTGGCAAATGCAAACTGCAAGCTCAAGATATGCGATTTTGGACTAG
CAAGAGTTGCATTCAATGACACCCCTACAACTGTTTTCTGGACGGACTATGTTGCCACTAGGTGGTACAGAGCTCCG
GAGCTCTGTGGTTCTTTCTTTACCAAGTATTCACCGGCTATCGATACATGGAGCATTGGTTGCATTTTTGCAGAGAT
TTTGACAGGAAAGCCTTTGTTCCCCGGTAAAAATGTGGTTCACCAGTTGGATATGATGACTGATTTCTTAGGCTCAC
CGTCGCCTGACATTATTTCTCGGATTCGAAATGAGAAGGCAAGGAGGTATCTGAGCACCATGAAGAAGAAACTGCCA
GTATCTTTTTCAGAAAAGTTCCCCAATGCAGATCCTGCAGCAGTCAAGCTCTTGCAAAAGCTTCTAGCATTTGATCC
AAAGGACCGACCCACTGCTGAAGAGGCGTTGGCTGATCCCTATTTCAATGGCCTCGCGAAAGTGGAGAGAGAACCAT
CATGCCAGCCGATTTCGAAAATGGAGTTTGAGTTTGAACGTAGAAAGTTTACCAGAGAGGACGTCAAGGAACTTATA
TTCAGGGAGATATTGGAGTACCACCCTCAGCTTCTCAAGGATTACAGCAACGGCTCAGAGAAAACGAACTTTCTATA
TCCTAGTGCTGTCGACAACTTCCGGAGGCAATTTGCTAACTTAGAGGAAGATGGAGGAAAGGGCGGGGCAGCCGAGA
GGAAGCATGTTTCTCTGCCCAGGACTACAACAGTTCACTCTACCCCAGTTCCTACAACAAATTGTCCGGCCTCCCAA
GCTCCTCAAAGGATTCCAGCAGCTAGACCGGGCAGAGTGATTGCCTCAGCGACACCAACCGAGAACGCGGCCTTCAC
CGATCGACAAATGGGCCGAAGGATGGCGAGGGACCCCGCGGCTAGCAGCAGCGGCGCCGGCTACCACCAGAGGCCGG
ACTGCTCCGACAGGCAACAGGAGGTGGAGAAGGACCGCGCGCACTACAGGCCGGCGCACCATTTCAGGGACGCCAGG
GTGGCGCCCGAGGCCGAGGCGCGGCCCTCGGCGTACCACATTCCCCCGTTCAACGGCATCGCCGCGGTCGCCGGCGG
GTACAGCAAGGTCGGCGTGGCAGGAAGGATGTAC**TAG**CGCAATTGCTGGGCGAGTTTGCCGGGAGCTGGAGGACGGC
CGCGATCGATGGCCGTTGTGGGGAATGCAGGGGCTGGGAGGGTCTCAAATTTAGGACGTAGGAGACAGCCGGACAAG
GTGGTGCTCTTGTGTACATAGATATTACCGGCAGCCAAATTGGTTGGTCTGTTTTTCTGGGTTGATAGATATGTTGA
AAGTTGGAACAGTGA

>TsMPK20-5 : contig67307
GCCTGCTTGGACTTGTTTAACACCCACCACCGCTCCCTCCTCCCTCCTCCGGGTGACGTCGCCGCCGACCGCACCGG
CGGCGGGGCGAGGCGCGGCGCGGCGCGGCCTTCTATCTCCCGTGCTCCCCAGG**ATG**CCGGAGGCAAATGCGGGTGCC
CGCGGCGGCGGCGAGCAGCGCAGCAAGGTTCAGAGTTCGGACGTGATGAGTTTCTTCAGTGAATATGGAGATGCAAG
CAGATACAAGATTGAGGAAATCATCGGCAAAGGGAGTTATGGGGTGGTATGTTCAGCCATCGACCGGCAGACCGGCG
ACAGGGTGGCCATAAAGAAGGTGTCCAACATCTTCGAGCACATAACCGACGCCGCTCGGATGCTCCGCGAGATCAAG
CTTCTCCGGCTTCTCAGGCACCCCGACATCGTCCAGATCAAGCACATAATGCTGCCTCCCTCCAGGAGGGACTACAA
GGACATATTTGTTGTCTTTGAGCTCATGGACACGGACCTCCACCAGGTTATCAAGGCCAACGATGACTTGACAAAGG
AGCACTACCAGTTCTTTCTCTACCAGATGCTCCGCGCGCTCAAATACATCCATACTGCTAACGTTTATCATCGTGAT
TTGAAGCCAAAAAACATATTGGCAAATGCAAACTGCAAGCTCAAGATATGTGATTTTGGACTAGCAAGAGTTGCATT
CAATGACACCCCTACAACTGTTTTCTGGACGGACTATGTTGCCACTAGGTGGTACAGAGCTCCGGAGCTCTGTGGTT
CTTTCTTTACCAAGTATTCACCGGCTATTGATACCTGGAGCATTGGTTGCATTTTTGCGGAGATCTTGACAGGAAAG
CCTTTGTTCCCTGGTAAAAATGTGGTTCAGCAGTTGGATATGATGACTGATTTCTTAGGCTCGCCGTCGCCTGAGAT
TATTTCTCGGATTCGAAATGAAAAGGCAAGGAGGTACCTGAGCAGCATGAGGAAGAAACTGCCAGTACCTTTTGCAG
AGAAGTTCCCCAAGGCAGATCCTGCAGCAGTCAAGCTCTTGCAAAAGCTTCTAGCATTTGATCCAAAGGACCGACCG
ACTGCTGAAGAGGCGTTGGCTGATCCCTATTTCAACGGCCTCGCGAAAGTGGAGAGAGAACCATCATGCCAACCGAT
TTCGAAAATGGAGTTTGAGTTTGAACGCAGAAAGTTTACCAGAGAGGACATCAAGGAACTTATATTCAGGGAGATAT
TGGAGTACCACCCTCAGCTTCTCAAGGACTACACCAACGGCTCTGAAAAAACAAACTTTCTATATCCTAGTGCCGTG
GACAACTTCCGGAGGCAATTTGCTAACTTGGAGGAAGATGGAGGAAAAGGCGGGGCACCCGAGAGGAAGCATGTTTC
TCTCCCGAGGACTACAACAGTTCACTCTACCCCAGTTCCTACAACAAATGGCCCGGCCTCCCAAGCTCCTCAAAGGA
TCCCAACAGCCCGACCAGGCAGAGTGATTGCCTCAGCGATACCGACCGAGAACACGGCCTTTGCCGATCGACAAACA
GGTCGAAGGATGGCGAGGGACCCCGCAGCGCCTCCAGCAGCAGCGGTTGTGGGCTACCACCTGAAGCCGGACAGCTC
CGACAGGCAACCAGAGCAGCAGCAGCAGGAGGTCGAAAAGGAGCGCGCGCGCTACAGGCCGGCGCACCATTTCAGGG
ACGCCAGGGTGGCGCCTGAGGCCGAGGCGCGGCCCTCGGCGTACCACATTCCCCCGTTCAACGGCATCGCGGCGGCC
GCAGGCGGGTACAGCAAGGTCGGCGCGGCAGAGAGGATGTAC**TAG**CGCAATTGCTCGGCGAATTTGCCGGGAGCTGG
AGGACGGCCGCGACCGTCGTGGGGAATGCAGGAATTTAGGAGGTAGGAGACAGCCGAAGAGGTGGTGCTCTTGTGTA
CATATATATTACCGGCAGCCAATTGGTTGGTTCTGTGGTCTGGGTTGATAGATAGGTTGATGAAAGTTGAAACAGTA

TGCTCTGCTTATCTGAGAGGTGAAGCGCCTCTGTTGAGGAGGGAGGGGAGGGGGTTGAGGGGCTGTTTTTCTTTTTC
TTTTTGTTTCTGGATTGTGTTAATAGGATGGATGAATAAATCGAGGCTTCTCTGTTGGTAAAC

>TsMPK21-1 : contig53625 (partial)
GGAGTCCCGGCTGTAG**ATG**CCCTCCCCTCCCCTCGTTTTAAAACCGCAGCCGCTCCTCCTCCCCACTTCCCCTCCTG
CACCCACCCACTCCGCCGAGAAACAGCAACAGCCACAGCCACGCGCACACACCAGATCGTCGATAGCGATGGCAGCG
GCAGCAGCAGCGGCAGCGGGAACGAGGGAGGAGATGGTCTACATGGCGAAGCTGGCGGAGCAGGCCGAGCGGTACGA
GGAGATGGTCGAGTTCATGGGGGAGGCCGGGAGGCTGCGCGTCGCCCTACCCCGCGCGCCCCGCGCGCACCAACCCGC
CGCCACGCATGGATCCCGGCAAGAAGACCTCGGAATCCGAGTTCTTCACGGAGTATGGTGAGTTAAACCGGTATCAG
GTCAGCGAGGTCATTGGCAAAGGGAGTTATGGAGTTGTGGCTGCTGCTATCGACACCCAGACCGGCGAGCGTGTGGC
CATCAAGAAGATCAATGACGTCTTTGATCATGTCTCCGATGCCACCCGCATCCTTAGGGAGATCAAGTTGCTCCGGT
TGCTGCGCCACCCGGACATAGTTCAGATCAAGCACATTATGCTCCCCCCTTCAAGGAGGGAATTCAGGGACATATAT
GTGGTCTTTGAGCTGATGGAGTCCGATCTCCATCAGGTAATAAAAGCGAACGATGATCTCACACCAGAGCATCACCA
GTTCTTCTTGTATCAGCTGCTCCGGGGAATGAAGTACATCCATGCAGCGAGTGTTTTCCATCGGGATCTTAAGCCCA
AGAATATTCTAGCGAATGCTGACTGCAAGCTGAAGATTTGTGATTTTGGGCTTGCCCGTGTATCATTTAATGACGGG
GCTCCATCAGCCATATTCTGGACGGACTATGTTGCAACTAGATGGTATCGTGCTCCAGAATTGTGTGGCTCTTTTTT
CTCAAAGTACACTCCTGCAATTGATATTTGGAGCGTAGGGTGTATCTTTGCAGAAATGCTCACAGGGAAGCCACTCT
TTCCAGGGAAGAATGTTGTCCATCAATTGGATCTCATGACTGATGTACTTGGCACTCCTTCAGCAGAATCTCTCGCT
AAGATACGGAATGAGAAAGCTCGGCGATACTTGAGCAATATGAGGAAGAAGCCTAAAGTTCCCCTTACCAAAAAATT
TCCAGGCATCGATCCTATGGCTCTCCATTTGCTTGAGCGTCTTCTTGCTTTTGATCCTAAGGATAGGCCAACTGCTG
ACGAGGCCCTGACAGACCCATACTTTAACGGATTAGCAAATTCAGAACGTGAACCCATAGCACAGCCCATCTCAAAA
CTTGAGTTTGAGTTCGAGAAGAGAAAGTTGGGCAAAGATGATGTCCGAGAATTAATTTACAGAG

>TsMPK21-2 : contig68757
TCCGGACCGGAGCACGGGGCGACTGGCGCGGCGGGCGCC**ATG**GCTAAAAAGGGCTCGGATAAGGCGGGCTTCTTCAC
GGAGTACGGCGAAGCGACCCGGTACGAGGTCTGCGAAGTGGTCGGCAAAGGCAGCTACGGCGTCGTGGCGTCTGCCG
TCGACACCCACACCGGCGAGAGCGTCGCCATCAAAAAGATCGACGACGTCTTCGAGCATGTCGCCGACGCCACCCGC
ATTCTCCGCGAGATCAAGCTGCTCCGCCTTCTGCGCCATCCAGACATCGTCCAGATCAAGCACATCATGCTCCCGCC
GTCGCGCCGCGAGTTCCGTGACATCTACATAATCTTCGAGCTCATGGAGTCCGACCTCCACCAGGTCATCAAGGCCA
ACGACGACCTCAGCCCGGAGCACCATCAGTTCTTTTTCTACCAGCTGCTTCGTGGGATGAAGTACATACACGCTGCC
AATGTCTTCCACCGGGACCTCAAGCCCAAGAACATTCTAGCCAATGCCGATTGCAAGCTCAAGATTTGCGACTTCGG
CCTTGCTCGGGTTTCCTTTGACGACACCCCGTCCGCGATATTCTGGACGGATTATGTAGCAACAAGGTGGTATCGTG
CTCCAGAATTATGTGGCTCCTTCTTTTCAAAGTATACTCCTGCGATTGATATTTGGAGCATAGGATGTATATTTGCA
GAAATGCTTACAGGGAGGCCGCTCTTTCCTGGCAAAAATGTGGTACATCAATTGGATCTCATGACTGATCTACTCGG
CACTCCTTCGGCAGAATCTATTTCTAGGATTCGAAACGAAAAAGCTCGGCGATATTTGGGAAACATGAAAAAGAAAC
ATCCAATACCTTTTTCTCAGAAGTTTCCTGGTGTAGATCCCATGGCACTCCATTTGCTTGAGCGTCTTCTTGCTTTT
GATCCCGCAGATCGGCCAACCGCTGCAGAGGCCTTGGCAGACCCATACTTTACTGGATTGGCAAATTCTGAACTCGA
ACCCACAACGCAACCCATCTCGAAACTTGAGTTTGAGTTTGAGAGAAGGAAGCTGGCCAGAGAGGATTTACGTGAAT
TAATCTACAGAGAGATTTTAGAGTACCATCCTCAGATGTTGCAAGAATACCTACGTGGAGGAGACCAGATGAGCTTC
ATGTACCCTAGTGGGGTGGATCGCTTTAAGCAGCAATTCGCTCATTTGGAAAAAGGTGGTGCAAAGGGTGAAAAATC
CAGTCCACAGTTGCGACAAAATGCTTCCTTACCAAGGGAAAGAGCAATTGGCAATAAGCACGGAGATAGTGAGTACC
AAGTAAAGCTGAATGCAGGTGAGAAGCCAGTACATGCATCAGTGACAGATGGAATAAGCAAACCCCTCATGAGCGCT
CGGAGCTTACTGAAGAGTGAAACCATGAGTGCTTCCAAGTGTATAGGTGAAATAAAAAATAAAGATGATGAATACGA
GAGCGTGGATGCAGCTGACGGCGTCTCTCAGAAGATCGCTCAACTGAAAACC**TGA**TTTTCGAAGGTTACAGAAGCGA
TTGGTAGTATATACTATGTACAACAGCATGTCTATAGGCGCTCAAGATCAGAATGATCTTTAACCAAAGCCAAGAAT
TCTATACAACAAATAAGACCACAAAATCATCTGTTATGGTAACCTTGTTGTGCTGTAATGTAAAATTTTCTACTCGT
TTTTTGTACTTTGTCAAAGAACCATGTCTAACCGACAAGCTAGTTCCTCGTCCTTTCCTCGGAGGAGCTGCCATGAT
CCTGTACAATTTTGTCAATATGAAGTATCACGCCCCTATATTTCTGTTCATATGGTGCTATTCTGATCTTGTAAACC
TTCTGCAATCAGAGATGCTCTTTGCCTCTGTTAATTTATGCAAATGTTTGTCTAGAGACGCTCCTTGTGCCCGCTAT
ATATGCAAATGCTTGCCATATTTTCCA

>TsMPK21-2 : contig201182
TCCGGACCGGAGCACGGGGCGACTGGCGCGGCGGGCGCC**ATG**GCTAAAAAGGGCTCGGATAAGGCGGGCTTCTTCAC
GGAGTACGGCGAAGCGACCCGGTACGAGGTCTGCGAAGTGGTCGGCAAAGGCAGCTACGGCGTCGTGGCGTCTGCCG
TCGACACCCACACCGGCGAGAGCGTCGCCATCAAAAAGATCGACGACGTCTTCGAGCATGTCGCCGACGCCACCCGC
ATTCTCCGCGAGATCAAGCTGCTCCGCCTTCTGCGCCATCCAGACATCGTCCAGATCAAGCACATCATGCTCCCGCC
GTCGCGCCGCGAGTTCCGTGACATCTACATAATCTTCGAGCTCATGGAGTCCGACCTCCACCAGGTCATCAAGGCCA

ACGACGACCTCAGCCCGGAGCACCATCAGTTCTTTTTCTACCAGCTGCTTCGTGGGATGAAGTACATACACGCTGCC
AATGTCTTCCACCGGGACCTCAAGCCCAAGAACATTCTAGCCAATGCCGATTGCAAGCTCAAGATTTGCGACTTCGG
CCTTGCTCGGGTTTCCTTTGACGACACCCCGTCCGCGATATTCTGGACGGATTATGTAGCAACAAGGTGGTATCGTG
CTCCAGAATTATGTGGCTCCTTCTTTTCAAAGTATACTCCTGCGATTGATATTTGGAGCATAGGATGTATATTTGCA
GAAATGCTTACAGGGAGGCCGCTCTTTCCTGGCAAAAATGTGGTACATCAATTGGATCTCATGACTGATCTACTCGG
CACTCCTTCGGCAGAATCTATTTCTAGGATTCGAAACGAAAAAGCTCGGCGATATTTGGGAAACATGAAAAAGAAAC
ATCCAATACCTTTTTCTCAGAAGTTTCCTGGTGTAGATCCCATGGCACTCCATTTGCTTGAGCGTCTTCTTGCTTTT
GATCCCGCAGATCGGCCAACCGCTGCAGAGGCCTTGGCAGACCCATACTTTACTGGATTGGCAAATTCTGAACTCGA
ACCCACAACGCAACCCATCTCGAAACTTGAGTTTGAGTTTGAGAGAAGGAAGCTGGCCAGAGAGGATTTACGTGAAT
TAATCTACAGAGAGATTTTAGAGTACCATCCTCAGATGTTGCATGACTATCTTCGTGGTGGAGATCAGGCAAACTTT
CTTTACCCAAGTGGGGTGGATCGTTTCAAGAGGCAATTTGTTCATCTCGAAGAAATCGGTGCTAAGGGTGAAAAGAC
TAGCCCGCAGCTGCGGCAGCATGCTTCCTTACCAAGGGAAAGAGTTATCGGCAGTGATGATCCTGAAAAGCCAAATG
CAGACTACTGTATAAAATTGCATGTAGGTGAGCTACCAGGTCACACATCGGTGACGGATGGCCTTAACAAGCCACTG
TTGAATACTCGAAACTTCTTGAAGAGCGAAAGCATTGGTGCCTCCAAGTGCATCGTCGTCAAAGAAAAGCGAGAAAA
AGATGAGGAATCTATGTCTGAGTATATGCATGAAGCATCTGAT**TAG**GAAGCGAAAAGGATTGCTCAACTCAAAAGTT
TGTGATGGTACGGGGAAAACAGAAGCACGTCCGATCCATGGGGGCTTCACTTCAGAATCCCGAGCGCACGAATCAGA
TCGATGAACCACACTGGTGCCGGTGCCTGTGCCGGTGCCGGATAGTCATCTCATTGGTTAGCAGAGCCTAGGATTTA
TTTCAATTTTTTGGGCACAACTTTGATCGATGCTCCCATTCATGTAACTAGTAGAGTTTAGAATGTAACCAACAGAG
TGTACAATTGTCCTTTTCTAAAACAGAGTGTACAATTGTAACCAACATATACTGGAGTACATGGCTCGACTTCTTAT
CACTCGTGCAGTGCTTTCTT

>TsMPK21-2 : contig221182
TCCGGACCGGAGCACGGGGCGACTGGCGCGGCGGGCGCC**ATG**GCTAAAAAGGGCTCGGATAAGGCGGGCTTCTTCAC
GGAGTACGGCGAAGCGACCCGGTACGAGGTCTGCGAAGTGGTCGGCAAAGGCAGCTACGGCGTCGTGGCGTCTGCCG
TCGACACCCACACCGGCGAGAGCGTCGCCATCAAAAAGATCGACGACGTCTTCGAGCATGTCGCCGACGCCACCCGC
ATTCTCCGCGAGATCAAGCTGCTCCGCCTTCTGCGCCATCCAGACATCGTCCAGATCAAGCACATCATGCTCCCGCC
GTCGCGCCGCGAGTTCCGTGACATCTACATAATCTTCGAGCTCATGGAGTCCGACCTCCACCAGGTCATCAAGGCCA
ACGACGACCTCAGCCCGGAGCACCATCAGTTCTTTTTCTACCAGCTGCTTCGTGGGATGAAGTACATACACGCTGCC
AATGTCTTCCACCGGGACCTCAAGCCCAAGAACATTCTAGCCAATGCCGATTGCAAGCTCAAGATTTGCGACTTCGG
CCTTGCTCGGGTTTCCTTTGACGACACCCCGTCCGCGATATTCTGGACGGATTATGTAGCAACAAGGTGGTATCGTG
CTCCAGAATTATGTGGCTCCTTCTTTTCAAAGTATACTCCTGCGATTGATATTTGGAGCATAGGATGTATATTTGCA
GAAATGCTTACAGGGAGGCCGCTCTTTCCTGGCAAAAATGTGGTACATCAATTGGATCTCATGACTGATCTACTCGG
CACTCCTTCGGCAGAATCTATTTCTAGGATTCGAAACGAAAAAGCTCGGCGATATTTGGGAAACATGAAAAAGAAAC
ATCCAATACCTTTTTCTCAGAAGTTTCCTGGTGTAGATCCCATGGCACTCCATTTGCTTGAGCGTCTTCTTGCTTTT
GATCCCGCAGATCGGCCAACCGCTGCAGAGGCCTTGGCAGACCCATACTTTACTGGATTGGCAAATTCTGAACTCGA
ACCCACAACGCAACCCATCTCGAAACTTGAGTTTGAGTTTGAGAGAAGGAAGCTGGCCAGAGAGGATTTACGTGAAT
TAATCTACAGAGAGATTTTAGAGTACCATCCTCAGATGTTGCATGACTATCTTCGTGGTGGAGATCAGGCAAACTTT
CTTTACCCAAGTGGGGTGGATCGTTTCAAGAGGCAATTTGTTCATCTCGAAGAAATCGGTGCTAAGGGTGAAAAGAC
TAGCCCGCAGCTGCGGCAGCATGCTTCCTTACCAAGGGAAAGAGTTATCGGCAGTGATGATCCTGAAAAGCCAAATG
CAGACTACTGTATAAAATTGCATGTAGGTGAGCTACCAGGTCACACATCGGTGACGGATGGCCTTAACAAGCCACTG
TTGAATACTCGAAACTTCTTGAAGAGCGAAAGCATTGGTGCCTCCAAGTGCATCGTCGTCAAAGAAAAGCGAGAAAA
AGATGAGGAATCTATGTCTGAGTATATGCATGAAGCATCTGAT**TAG**GAAGCGAAAAGGATTGCTCAACTCAAAAGTT
TGTGATGGTACGGGGAAAACAGAAGCACGTCCGATCCATGGGGGCTTCACTTCAGAATCCCGAGCGCACGAATCAGA
TCGATGAACCACACTGGTGTGCCGGATGGTCATCTCGCTGGTTGGCATATTTCAATTTTTTGGCCGCAAGATCGATA
CTCCCATTCATGTAACTAGTAGAGTTTAGAATGTAACCGACAGTGTAGAATGTACTACTAACCAACATATACTGGAA
TACATGACTCGACTTCTTATTACTCGTGGAATGCTTTCTTA

>TsMPK24 : contig116794
ATCTTGCTCCGTCTTGTACCCAATCAGCACAAACGCCCGATTGCTTCCTATCTGTTTAATTTGCTTCTTGTGGCTGC
AGGCACCACTTTTTGGCCTTGGCGTGCAGACTTTGGATATAATCCCGATTAGCTTATCGCATCCTCGCTAATTGGTA
ACATTGGGC**ATG**GAGTTCTTCACAGAATATGGGGAGGCAAGCCAGTATCAGATCGAAGAGATCATTGGCAGGGGAAG
CTTCGGAGTAGTTGCTGCTGCAGTAGATACCCAAACTGGGGAGCGGGTTGCGATCAAGAAGATACATGATATGTTTG
AGCATGCCTCAGATGGCACCCGCATTCTTTGGGAAATCAAGCTTCTTCGGCTTCTCCGCCACCCAAACATAGTTGAG
ATCAAACACATCCTGCTCCCCCCTGCCCGAAGGGAGTTTAGAGATATTTATGTTGTTTTTGAGCTCATGGAGTCAGA
CCTACAAAAAGTGATCCAAGTAAATGAGAACCTCACCGCAGGGCATCACCGGTATTTCTTGTATCAACTTCTTCATG
CCCTCAAGTACATCCATGCAGCTAATGTATTTCATCGTGACTTAAAACCGAGCAACATACTTGTCAATTCGAACTGC
AAACTAAAGATCTGTGACTTTGGGCTTGCACGCCCATTGGATGATGCTCCCTTGGCTATATTTTGGACTGACTATGT

GGCTACAAGGTGGTACCGTGCTCCTGAATTATGTGGCTCATTTTTCTCCAAATACACCCCTGCAATTGATATTTGGA
GCATAGGGTGCATATTTGCTGAAGTTCTCACTGGAAAACCATTATTTCCTGGGAGGAATGGCACACACCAACTAGAT
CTGATAACAGATGTCCTTGGAAATCCATCATGTGAAACCCTATCCCAGATTCGTAATGAGAAGGCGAGGAGATATTT
GACTGGCATGAAGAGGAAACATCCTATCCCCTTTGCTAGTGTGTTTTGTAATGCCGATCCTAAGGCTGTCCGTCTCC
TAGAACGCCTACTCGCATTTGATCCTAAAGATCGACCTACTGCTGAAGAGGCTTTAGCTGATCCATATTTTGAAGGA
CTTCGTAAGTTGGAACATGAGCCTTTACCACACCCCTTTTCAAAACTTGAGTTTGAATTTGAGAGATTGAAGCTAAC
AAAGGGTGGTGTAAGAGATCTGATATATCGAGAGATTTTGGAGTACCATCCACAGATGCTTCAGGATTATATCAGAG
CTGGAGGACAGACTAGTTTCGTCTATCCAAGTGGGGTTGATGGTATGAGGCTGCAGTTTGTACATCTTGAGGAGAAC
CACCTGAGAGGAGAAAGAGGCACTCCACTGCGGAGGCGACATGCATCTTTGCCAAGGGAAAGAGTCTGTGCACCAAA
AGGTAGCGATAATCAAGACTGTAACAATGAGAGAAGGAGGACAGCATCTTCTGCTGCCCAAACTACCATAAGATCAC
AACAAGAGGGGCTGACACATGCATATGTTTATCAAAATGGCACAAGCATTCCGAACTTCTGCTCTGGGTATTACTTG
CAGAATGGTAGTACCAGTGCTTCCAGTTGTGTCATCGAAGAGAATGAAGGCCTGGAGGAGAACGGCGTCTCCGAGGA
GGAGAAGGTGGCCTATGAACTGTCGGAAAGGCTTGCCAGGATC**TAG**C

>TsMPK25 : contig146788
GTACATGGGGAAGCCGACGTGAGTTCTTCCTCACAATTGCCACTCCTCTGTCAGCTCAAGCGCAGTGCTGGTACTGC
TGTGTCATTGACGGTTCACATCTGTTGCTTCGTCCCGAGAAAAAGAAA**ATG**GCGAAGATGGTACGTCCTCCGAATGG
CAAGGGAAAGCATGGCAAGCATTACTACACTATGTGCCAGACAATGTTTGAGATCGACACAAAGTACGTGCCAATCA
AGCCCATCGGAAGAGGATCTTACGGGATCGTTTGCTCATCGGTGAACAAGGATACGAACGAGCAAGTCGCGATAAAA
AAGATAAACAATGTCTTTGACAACCGTGTGGATGCGTTGAGGATGCTGCGAGAGATGAAGCTCCTTCGGCACTTGCG
TCATGAGAATGTCATTGCTTTGAAGGATATAATGATGCCAATACAGAAGAGGAGCTTCAAAGATGTCTACTTGGTCT
CTGAACTGATGGACACAGATCTGGATCGGATCATCAACTCGTCTCAAACGCTTTCGAATGACCACTGTCAATATTTC
CTTTTTCAGCTGCTCCGAGGCCTGAAGTATCTTCATTCAGCAGGGATACTCCATAGAGACCTGAAACCACTCAACCT
TCTAGTTGATGGAAACTGTGACCTGAAGATCTGTGACTTTGGTCTTGCCCGAACAAATAATACTGAAGATCAGTGTA
TGACTGAATATGTTGTCACCCGCTGGTATAGAGCTCCAGAGCTGCTGCTCTCCAACGACAACTATGGCACCTCCATA
GATGTCTGGTCTGTTGGCTGCATCTTTGCCGAGCTTCTTGGCCGCAAGCCGATCTTCCCTGGAACCGACAGCCTAAA
TCAGCTTCAGCTTATAGTTAATGTTCTTGGCACCATGAACAATGCTGACCTTGAGTTTATTGATAATCCGAGACCCC
ATAGTTACATCAAATCCCTTTCATACACCGCTGGGATTCCCCTCACCGGAATGTACCCACAAGCGCACCCGCTTGCC
ATTGACCTGTTGCAGAAGATGCTGGTCTTTAATCCTTCCAAAAGGATTAGTGTCACTGAGGCTCTGGAGCACCCCTA
TATGTCTAGGTTGTATGATCCGAGCGTAAGTCGTCCTGCTCAGGCACCCATCAATCTTGATATAGATGAAAACCTCA
GCGTGGAGATGCTCCGAGAAATGATGTGGCAGGAGATGCTCCAGTACCACGAGGCTGCCATGATGGTGAATATG**TGA**
CAAGCATGAATGGACATGTGAAGAGCTATTTGCCACACACCCACAGTAGGGTCCTCGCATGTCGTTTTTATATAAAG
CTTACAGTGATTATTGCAGCTATAAAGTACTCAAGTTA

>TsMKK3-2 : contig143511
GCTCACCGCCGCCGCCTCCTCCCCCCGCTCACCGCCTCCTCACCGTCCGTCCCAACCCCTTCGCGCCCCTTTGTATC
CTCCGCTATCCGCCCGTACAGATCCGTCCGGCGAGCATCCTCAGCCGCCCCCGACGGGCTCGCCGCCGCCAGGACCT
CACCGCTGCCCCCCTCACCGCGAAGAGAACCGAGGTCCGCCCAAGCTCCAATTTTTCGCCTGGGGAACAGACCGCCC
GCCGCCTTCTCTGCTCCACTGAAGATACGGTGATTCCTTCCTGCTTGGACGACTGGGATCATCAGCTTGCGCCGGCT
GACTACAAGCGAGCCCCC**ATG**GCGGGGCTAGAGGAGTTAAAGAAGAAGCTGCAGCCCTTGCTGTTCGACGACTCGGA
CAAGGGCGGCGTCAGCACCCGGGTTCCCTTCCCGGAGGATACATGCGATTCCTATGTGGTATCTGATGGTGGAACGA
TAAATTTACTGAGTAGATCGTTTGGTGAGTATAACATCAATGAGCATGGCTTTCACAAGCGAAGTACTGGGCCAGAA
GAGCCCGATACCGGTGAGAAGGCATACCGATGTGCATCTGAAGACATGCATATATTTGGTCCCATTGGAAGCGGAGC
AAGCAGCGTTGTTCAGAGAGCTATTTTCATACCAGTTCATCGTATTCTGGCCTTGAAGAAGATAAACATATTTGAGA
AGGAGAAAAGGCAACAAATTCTTAATGAGATGAGAACGTTATGTGAAGCAAGTTGCTATCCTGGTTTAGTTGAATTC
CAGGGTGCATTTTACATGCCCGATTCTGGACAAATAAGCATTGCCCTTGAATACATGGATGGTGGCTCTTTAGCAGA
CGTTATAAGAGTCAAGAAATCAATACCAGAGCCAGTTCTTGCACATATGCTACTGAAAGTATTACTTGGCCTGAAGT
ACTTGCATGAAGTAAGACATCTAGTGCATAGAGATCTAAAGCCAGCGAATATACTGGTAAACCTCAAGGGAGAGGCA
AAAATTACAGACTTTGGTGTAAGTGCTGGTTTGGACAATACAATGGCTATGTGTGCTACCTTTGTAGGCACTGTGAC
ATATATGTCACCTGAGAGAATTCGTAATGAGAACTACTCTTATGCTGCTGATATTTGGAGTCTTGGACTAACGATAT
TGGAGTGTGCTACTGGTAAATTTCCATATAATGTCAATGAAGGCCCAGCCAATCTCATGCTGCAGATACTCGATGAT
CCATCACCAGCACCACCAGAAGATGCCTATACACCAGAATTTTGTTCCTTCATAAATGATTGCTTGCGGAAAGATGC
TGATGCAAGGCCTACATGCGAGCAGCTTTTGTCACACCCATTCATCAAGAGGTATGAGCAAACTGGTGTGGACTTGG
CAGCATATGTCAGGGGTGTTGTTAACCCAACAGAAAGATTAAAGCAAATAGCAGAGATGCTTGCTGTTCATTATTAC
CTCCTGTTTAATGGCTCTGAAGGACCTTGGAATCATATGAAGACATTCTACAGGGAAGAATCATCTTTCAGTTTTTC
AGGGAATGTGTATGTCGGACAAAGTGCCATATTTGATACTTTATCAAATATAAGAAAGAAGTTAAAAGGTGATCGGC
CTAGAGAAAAAATTGTTCATGTTGTTGAGAAGCTACACTGCCGTGCGAATGGGGAAACAGAAATCGCTATTCGTGTG

TCTGGGTCATTCATCACGGGCAACCAATTCCTAATATTTGGTGAAGGGTTGCAAGCTGAAGGGATGCCCAGCTTGGA
CGGAATTGATATCGACATTCCAAGCAAGCGCGTAGGCCAGTTCCGGGAGCAGTTCACCGTGCATCCAGGGACTTCCA
TGGGGTGCTACTACATAGCAAAGCAAGACCTCTACATCGTTCAGTCC**TGA**AAACAATGTATAAACTGTAAGTTATGT
TCCGTTTGTGCCGTCAATGATTCCAGTTCCCGATGAAAGCTAGCTATGTCATGGTTTCCACTTTCTCGCGGCCCCTT
GACTTCGACATCGACAGGCTTGAGACCACAACGCAGTCCTGCTACCATTGACATTGATTCCATTTCTTCTCGATGCG
AGCAATCAAACCTGTAGCTGAAACATAACTTGATCGTTGACCACTTGTTTGTAAGGTGGCAAGGAGGATGAAGCGAT
TCTAATGATTGTTGTGCATAGATAGATACTCTTGACACATGGTGCGGGTTTATTCGAGGCTGCTCGATTTCTGTTGT
AGATGAGAAGATTGCGCGTGTGCGCTAATAAGTGACGCAGCCCCAATGTAGGTCTGTACTACTCATGCTTATGTGGG
AAAAAAAGACTATCTATAA

>TsMKK3-2 : contig224242
GCTCACCGCCGCCGCCTCCTCCCCCCGCTCACCGCCTCCTCACCGTCCGTCCCAACCCCTTCGCGCCCCTTTGTATC
CTCCGCTATCCGCCCGTACAGATCCGTCCGGCGAGCATCCTCAGCCGCCCCCGACGGGCTCGCCGCCGCCAGGACCT
CACCGCTGCCCCCCTCACCGCGAAGAGAACCGAGGTCCGCCCAAGCTCCAATTTTTCGCCTGGGGAACAGACCGCCC
GCCGCCTTCTCTGCTCCACTGAAGATACGGTGATTCCTTCCTGCTTGGACGACTGGGATCATCAGCTTGCGCCGGCT
GACTACAAGCGAGCCCCC**ATG**GCGGGGCTAGAGGAGTTAAAGAAGAAGCTGCAGCCCTTGCTGTTCGACGACTCGGA
CAAGGGCGGCGTCAGCACCCGGGTTCCCTTCCCGGAGGATACATGCGATTCCTATGTGGTATCTGATGGTGGAACGA
TAAATTTACTGAGTAGATCGTTTGGTGAGTATAACATCAATGAGCATGGCTTTCACAAGCGAAGTACTGGGCCAGAA
GAGCCCGATACCGGTGAGAAGGCATACCGATGTGCATCTGAAGACATGCATATATTTGGTCCCATTGGAAGCGGAGC
AAGCAGCGTTGTTCAGAGAGCTATTTTCATACCAGTTCATCGTATTCTGGCCTTGAAGAAGATAAACATATTTGAGA
AGGAGAAAAGGCAACAAATTCTTAATGAGATGAGAACGTTATGTGAAGCAAGTTGCTATCCTGGTTTAGTTGAATTC
CAGGGTGCATTTTACATGCCCGATTCTGGACAAATAAGCATTGCCCTTGAATACATGGATGGTGGCTCTTTAGCAGA
CGTTATAAGAGTCAAGAAATCAATACCAGAGCCAGTTCTTGCACATATGCTACTGAAAGTATTACTTGGCCTGAAGT
ACTTGCATGAAGTAAGACATCTAGTGCATAGAGATCTAAAGCCAGCGAATATACTGGTAAACCTCAAGGGAGAGGCA
AAAATTACAGACTTTGGTGTAAGTGCTGGTTTGGACAATACAATGGCTATGTGTGCTACCTTTGTAGGCACTGTGAC
ATATATGTCACCTGAGAGAATTCGTAATGAGAACTACTCTTATGCTGCTGATATTTGGAGTCTTGGACTAACGATAT
TGGAGTGTGCTACTGGTAAATTTCCATATAATGTCAATGAAGGCCCAGCCAATCTCATGCTGCAGATACTCGATGAT
CCATCACCAGCACCACCAGAAGATGCCTATACACCAGAATTTTGTTCCTTCATAAATGATTGCTTGCGGAAAGATGC
TGATGCAAGGCCTACATGCGAGCAGCTTTTGTCACACCCATTCATCAAGAAGTATGAGCCAACTGGTGTGGACTTGG
CAGCATATGTCAGGGGTGTTGTTAATCCAACAGAGAGATTAAAGCAAATAGCAGAGATGCTTGCTGTTCATTATTAC
CTCCTGTTTGATGGCTCTGAAGGACCTTGGAATCATATGAAGACATTCTACAAGGAAGAATCGTCTTTCAGTTTTTC
AGGGAATGTGTATGTCGGACAAAGTGCCATATTTGATACTTTGTCGAATATAAGAAAGAAGTTAAAAGGTGATCGGC
CTAGAGAAAAAATTGTTCATGTTGTTGAGAAGCTACATTGTCGTGCCAACGGGGAATCAGAAATCGCTATTCGTGTG
TCTGGATCATTCATCACGGGCAACCAGTTCCTAATATTTGGTGAAGGATTGCAAGCTGAAGGGATGCCCAGCTTGGA
CGAAATTGGTGTTGACATTCCAAGCAAGCGGGTAGGTCAGTTCCGGGAACAGTTCACCGTGCTTCCAGGGACTTCCA
TGGGGTGCTACTACATAGCAAAGCAAGACCTCTACATCGTCCAGTCC**TGA**AAATAATGAACAAAGTTTATGTTCCGT
TTGTGACGTCAATGATTCCAGTTGATGATGAAAGCTATGTCATGGCTTCCACGCCTCGCGGCCCCTTAACTCCGACA
GGCTTCAGATCACAAGACAGTCCTGCTACCATTGACATTGATTCCATTTCTTCTCGATGCGAGCAAGCAAACTTGTA
GCTCAAACATAACTTAATTGTTGTTTGTAAGGTGGTAAGGTCAATGAAGCGATTCTAATGATTGTTGTGTGTAGATA
GATACTCTTGACACATGGCGCGGGTTTAGTTGAGGCTGCTCAATTTCTGTTGTAGATGAGAAGATTGCGCGTGTGTG
TGCGCACGCTAATAAGTGGCACGGCTCAATGTAGGTTTGTACTACTAGTACTAGTATGTGAAAAAGACTTATCAATA
ATTAACTCCGCTGGTACTATATGGTTCTGTTTGACAT

>TsMKK3-2 : contig229009
CCGGCGAGCATCCCGACGGGCTCGCCGCCGCCAGCCGCCCTCCTCAGCCGAGAGCGAATCGAGGTCCGCCCAAGCTC
CAATTTTTCGTCCGGGGGAACCGAACCGTCCGCCGCCTTCTTCTCTGCTCAACTGAAGAGACGGTGGTTCCTCGCTG
CTTGGTACGTAGGGTTCTCTGACAGCCTCCCTGTGAACCACCCAGAGCGTACCCTTGCGCCGGGTGGTCACCAGCGA
GTCCCC**ATG**TCGGGGCTAGAGGAGTTGAAGAAGAAGCTGCAGCCCTTGCTGTTCGACGACACGGACAAGGGCGGCGT
CAGTACCCGGGTTCCCTTCCCGGAGGACACATGCGATTCCTATGTGGTGTCTGATGGTGGAACGATAAATTTACTGA
GTAGATCGTTTGGTGAGTATAACATCAATGAGCATGGCTTTCATAAGCGAAGCACCGGACCAGAAGAGCCAGATAGC
GGTGAGAAGGCATACCGATGTGCCTCTGAAGACATGCACATATTTGGTCCCATTGGGAATGGAGCAAGCAGTGTCGT
GCAGAGAGCTATTTTCATACCAGTTCATCGAATTCTGGCCTTGAAGAAGATAAACATATTTGAGAAGGAGAAAAGGC
AACAAATTCTTAATGAGATGAGAACATTATGTGAAGCAAGTTGCTATCCTGGTTTAGTTGAATTCCAGGGTGCATTT
TACATGCCTGATTCTGGACAAATAAGCATTGCCCTTGAATACATGGATGGTGGCTCTTTAGCAGACGTTATAAGAGT
CAAGAAATCAATACCAGAGCCAGTTCTTGCACACATGCTACTGAAAGTATTACTTGGCCTGAAGTACTTGCATGAAG
TAAGACATCTAGTGCATAGAGATCTAAAGCCAGCGAATATACTGGTAAACCTCAAGGGAGAGGCAAAAATTACAGAC
TTTGGTGTAAGTGCTGGTTTGGACAATACAATGGCTATGTGTGCTACCTTTGTAGGCACTGTGACATATATGTCACC

TGAGAGAATTCGTAATGAGAACTACTCTTATGCTGCTGATATCTGGAGTCTTGGACTAACGATATTGGAGTGTGCTA
CTGGTAAATTTCCATATAATGTCAATGAAGGCCCAGCCAATCTCATGCTGCAGATACTCGATGATCCATCACCAGCA
CCACCAGCAGATGGCTATACACCAGAATTTTGTTCCTTCATAAATGATTGCTTGAGGAAAGATGCTGATGCAAGGCC
TACATGCGAGCAGCTTTTGTCACACGCATTTATCAAGAGGTATGAGCAAACTGGTGTGGACTTGGCAGCATATGTCA
GGGATGTTGTTAATCCAACAGAGAGATTAAAGCAAATAGCAGAGATGCTTGCTGTTCATTATTACCTCCTGTTTAAT
GGCTCTGAAGGACCTTGGAATTATATGAAGACATTCTACAAGGAGGAATCATCTTTCAGTTTTTCAGGGAATGTGTA
TGTCGGGCAAAGTGCCATATTTGATACTTTGTCGAATATAAGAAAGAAGTTAAAAGGTGATCGGCCTAgagaaaaaa
ttgttcatgttgttgagaagctacattgccgcgcgaaaTGGGGAAACAGAAATTGCTATCCGTGTGtcTGGATCATT
CATCACGGGCAACCAATTCCTGATATTTGG**TGA**AGGGTTGCAAGCTGAAGGGATGCCCAGCTTGGAGGAAATTGATA
TTGACATTCCAAGCAAGCGGGTTGGCCAGTTCCGGGAGCAGTTTACCGTGCATCCAGGGACTTCCATGGGATGCTAC
TACATAGCAAAGCAAGATCTCTACATCGTCCAGTCATGAAAACAATGTATAAAGTGTAAGTGATGTTCCGGTTTGTG
CCGTCAATGATTCCAGTTCATGATGAAAGCTATGTCATGGTTTCCACTTCTCGCGGCCCCATAACTTCGACAGGCTT
CAGATCACAATG

>TsMKK3-3 : contig90697
CTCTGCTCCTTGTTTGGCTGTA**ATG**GCTGGACTGGAGGACCTGAAGCGGAGGCTGCAGCCCATCTTCTTCGACGCCG
ATGGCAATGTTGTGCCGCCCCCCGCCGCCGACGGGACATCCGACGACTCCAGCTCTGACGACTGCGAGGTTTTGGAT
AGTGGAACTGTCAATTTATTGAGTAGGTCCTCTGATGAATATAACATCAGTAAGCTTGGCTTCCATAAACGAACAAC
CAGACCAGATGGGGACTATGCTACAGATAAGGCATATCGATGTTCTTGTCATGATATGCATATTTTTGATTCTGTTG
GTAATGGTGCAAGCAGCGTTGTCCATAGAGCTATTTATGTACCAGTTCATCGAGTTTTGGCACTCAAGAAAATCAAC
ATTTTTGACAAGGAAAGGAGACAACAGATTCTTAATGAGATTAGAACGTTATCAGCAGCATCTTGTTATCCAGGTTT
AGTTGAATTCCAAGGAGTTTTTTACACCCCGGACTCTGGAGAAATATACTTTGCTCTTGAGTATATGGATGGTGGTT
CATTAGCAGATATTATCAGGGTCAAGAAATTCATAACAGAACCAGTTCTTTCACATATGCTACAGAAAGTGTTGCTA
GCTCTGCGCTACTTGCATGAAGTCAGGCGTTTAGTTCACAGAGATATAAAGCCAGCAAATTTGCTTCTAAATCTAAA
GGGTGATACGAAAATTACAGACTTCGGTGTAACTTCTGGACTGCATGATTCAGTTACCATGTGTGCTACCTTCCTGG
GCAGTGTCACATATATGTCTCCCGAGAGAATTCGGAACGAAAATTACTCATATGCTGCTGATATCTGGAGCCTTGGA
CTAACAGCATTGGAGTGTGCAACTGGAAGATACCCATATGATGTAAGTGGGGGTGAGGCCGACCTCATGCTGCAGAT
ATTGGAAGATCCATCGCCAACACCACCACAACATATGCATTCAGAGGAGTTTTGCTCGTTCATCGATGCTTGCTTGC
AGAAAGATGCTGATGCAAGACCAACATGTGATGAGCTTTTGTCTCATTCCTTCATCAAGAAATACGAGGGACCTGGT
GTGGACTTGGCAGAGTACAACAGAAGTGTTCATGATCCAGCAGAAAGATTATCACAAATAGCACATATGCTTGCTGT
ACATTACTACCTGATCTTTGACGGTGGTGATGACCAATGGCATCACATGAAGTCATTCTATGGACAAGATTCTACTT
TCAGTTTCTCAGGGGAAACACATGTCGGTAAGAGCGACATATTCGATACTTTGTCAAGAATAAGGGAAATGCTAAAA
GGTAACAGCCGTTGCGAGAAGATTGGCCGTGTGATGGAGAAGGTTTACCGTGCACATGGGGAAGAAGGGATGAGTGT
TCGAGTTTCTGGATCATTCATTATGGGGAACGAGTTCCTTGTGTGCGCAGATGGGTTTTGTGCTGAAGGGATGCTAA
GCATGGAACTCTCTCCCGACATTCTCAGCAAGCAGGCAGGCCATTTCCAGGAAGATTTTTTCATGGAGCCAGGGACT
GCCATGGGATGCTATGTGATATCAAGGCAAGAATTGCACATCGGCGTATCA**TGA**AGTCATTCGCCTTCTTGCATGCA
ATCATTACATATGATGATTTTTGTGCTTTTTACTGCCGTGCATTTGCTGAACTGATCTATCATGCATGTTATCTTCC
ATTTGATGCTGAAGAAAACAATCTTCTAGCATATCGACATCGAGGTATTGGAATCTATTCAGTTGAGGCTCAACACA
GTCTACAATGAACTGAAAACATCGATTTATTGTCTCTCTGCATTCAGCATTTACGCTTGTAAGTTGAATGACTGGG

>TsMKK4 : contig104479
TCTATCCACGACCACAAATACGGCGACAATCGCCGACGCCGACCGATCCGGCCGCCGCGCGGAGCAGGGGAGGAGCC
AGCCTCGCCTTTCGGGCCGGCCAGAGCGCGCG**ATG**CGTCCGGGCGGGCCGCCGAACGCGCGGCCGGGCCTGCAGCAG
CAGCAGCCCGGCACGCCGGGCCGGGCGCGGCGCCGGCCGGATCTCACGCTCCCGCTCCCGCAGCGCGACCTGACGTC
GCTGGCCGTGCCGCTGCCGCTGCCCCCGCCCCCGTCCTCCGCGCCGTCGTCCACGTCCTCGTCCGGGTCGGGGTCGG
GCGGCGCGTCGTCCATGCCCATGCCCATGTCCATGACCCCGCCCAACTCGGCCGGCTCCGCGCCGCCCGCCCCGCCG
CCGCTGGGCGAGCTGGAGCGCGTGCGGCGCGTGGGAGGCGCCGGCGGGACGGTGTGGCTGGTGCGGCACGCGCCCAC
GGGCCGCGCCTACGCGCTGAAGGTGCTGTACGGGCACCACGACGAGGCGGTGCGGCGGCAGATCACGCGCGAGATCG
CCATCCTGCGCACCGCGGAGCACCCGTCCATCGTGCGCTGCCACGGCATGTACGAGCAGGCCGGCGAGCTGCAGATC
CTGCTCGAGTACATGGACGGCGGGTCCCTGGACGGCCGGCGCATCGCGTCCGAGGCCTTCCTCGCCGACGTAGCGCG
GCAGGTGCTGTCGGGGATCGCGTACCTCCACCGGCGCCACATCGTGCACCGCGACATCAAGCCGTCCAACCTGCTCA
TCGACTCGGGGCGCGTGAAGATCGCCGACTTCGGGGTGGGGCGGATCCTGAACCAGACCATGGACCCCTGCAACTCC
TCCGTGGGCACCATCGCGTACATGAGCCCCGAGCGCATCAACACGGACCTCAACGACGGCAACTACAACGGCTACGC
CGGCGACATCTGGAGCTTCGGCCTCAGCATCCTCGAGTTCTACCTGGGCCGCTTCCCGCTGGGGGAGAACCTGGGGA
AGCAGGGCGACTGGGCGGCGCTCATGTGCGCCATCTGCTACTCCGAGTCGCCGGCCGCCCCGCCCACGGCCTCCCCG
GAGCTGCGGAGCTTCATCAGCTGCTGCCTCCAGAAGAACCCGGCGAAGCGGCCGTCGGCGGCGCAGCTGCTGCAGCA
CCGGTTCATCGCCTCGCCGCCGCAGCAGCAGCAGCCGCAGGTCCTCGCCGCCCCGCCGTGC**TGA**CGGCCCGCCCGCC

GTAACCAAGTTAAATTCGGACGGATCATGGACGGACGGACGGACGGACGGACGGACGGTGGCCGGCGGCGAGACGCG
GGATCTAGGGGGAGGAGGGGCGATCTGTCTTCTTAGCCATTTTGGGAACTGAAGCCACCACCACAAATTTACCCATC
CATCCCTCGGAGTCCTGTTGTTGCTGTCGCCCCCGAAATTAAGCTCAAGCTTTTTCTTTAGTTTCCGTTAATGTCAA
TTAAATGTTCTCCCCTCCAAATGATGATGATTCTGATTCTGATTCTGATGAT

>TsMKK5 : contig141775
TCTATCCACGACCACAAATACGGCGACAATCGCCGACGCCGACCGATCCGGCCGCCGCGCGGAGCAGGGGAGGAGCC
AGCCTCGCCTTTCGGGCCGGCCAGAGCGCGCG**ATG**CGTCCGGGCGGGCCGCCGAACGCGCGGCCGGGCCTGCAGCAG
CAGCAGCCCGGCACGCCGGGCCGGGCGCGGCGCCGGCCGGATCTCACGCTCCCGCTCCCGCAGCGCGACCTGACGTC
GCTGGCCGTGCCGCTGCCGCTGCCCCCGCCCCCGTCCTCCGCGCCGTCGTCCACGTCCTCGTCCGGGTCGGGGTCGG
GCGGCGCGTCGTCCATGCCCATGCCCATGTCCATGACCCCGCCCAACTCGGCCGGCTCCGCGCCGCCCGCCCCGCCG
CCGCTGGGCGAGCTGGAGCGCGTGCGGCGCGTGGGAGGCGCCGGCGGGACGGTGTGGCTGGTGCGGCACGCGCCCAC
GGGCCGCGCCTACGCGCTGAAGGTGCTGTACGGGCACCACGACGAGGCGGTGCGGCGGCAGATCACGCGCGAGATCG
CCATCCTGCGCACCGCGGAGCACCCGTCCATCGTGCGCTGCCACGGCATGTACGAGCAGGCCGGCGAGCTGCAGATC
CTGCTCGAGTACATGGACGGCGGGTCCCTGGACGGCCGGCGCATCGCGTCCGAGGCCTTCCTCGCCGACGTAGCGCG
GCAGGTGCTGTCGGGGATCGCGTACCTCCACCGGCGCCACATCGTGCACCGCGACATCAAGCCGTCCAACCTGCTCA
TCGACTCGGGGCGCGTGAAGATCGCCGACTTCGGGGTGGGGCGGATCCTGAACCAGACCATGGACCCCTGCAACTCC
TCCGTCGGCACCATCGCCTACATGAGCCCCGAGCGCATCAACACCGACATCAACGACGGCGCCTACGACGGCTACGC
CGGCGACATCTGGAGCTTCGGCCTCAGCATCCTCGAGTTCTACCTCGGCAGGTTCCCCTTCGGCGAGAACCTCGGCC
GGCAGGGCGACTGGGCCGCGCTCATGGTCGCCATCTGCTACTCGGACCCGCCCGAGCCGTCGCCCGCCACCGCCTCG
CCAGAGTTCCGGGGCTTCATCGCCTGCTGCCTGCAGAAGAACCCGGCCAACCGCCTCTCCGCCGCGCAGCTGCTGCA
GCACCCCTTCGTCGCGTTGCCGCAGCCGCAGCCGCTCGCCGCCCCGCCGTCA**TGA**CCGCCGCGACCTCCCAATACGG
ATCTCGATCCACTTCTTGCACCACCGCACTCTCAAATCT

>TsMKK5 : contig154845
CCACCCCCCACGGAAGCC**ATG**CGTCCGGCCGGCGGCAGCCTCCCGGCCCAGCCGGGCACGCCGGGCCGCCCGCGCCG
CCGCCCGGATCTCACCCTCCCGATGCCGCAGCGCCCGGACGTCTCCTCCTCCCTCGCCGTCCCGCTCCCCCTCCCGC
CCCCGACCGCCGCCCCCCTCCCGGGGCCGGCCCCGCCAGCCGCTGCGGCCCCGCCCCCGCCCCCGCTCCACGAGCTG
GAGCGCGTGCGGCGCGTGGGAGGCGCCGGCGGGACGGTGTGGCTGGTGCGGCACGCGCCCACGGGCCGCGCCTACGC
GCTGAAGGTGCTGTACGGGCACCACGACGAGGCGGTGCGGCGGCAGATCACGCGCGAGATCGCCATCCTGCGCACCG
CGGAGCACCCGTCCATCGTGCGCTGCCACGGCATGTACGAGCAGGCCGGCGAGCTGCAGATCCTGCTCGAGTACATG
GACGGCGGGTCCCTGGACGGCCGGCGCATCGCGTCCGAGGCCTTCCTCGCCGACGTAGCGCGGCAGGTGCTGTCGGG
GATCGCGTACCTCCACCGGCGCCACATCGTGCACCGCGACATCAAGCCGTCCAACCTGCTCATCGACTCGGGGCGCG
TGAAGATCGCCGACTTCGGGGTGGGGCGGATCCTGAACCAGACCATGGACCCCTGCAACTCCTCCGTCGGCACCATC
GCCTACATGAGCCCCGAGCGCATCAACACCGACATCAACGACGGCGCCTACGACGGCTACGCCGGCGACATCTGGAG
CTTCGGCCTCAGCATCCTCGAGTTCTACCTCGGCAGGTTCCCCTTCGGCGAGAACCTCGGCCGGCAGGGCGACTGGG
CCGCGCTCATGGTCGCCATCTGCTACTCGGACCCGCCCGAGCCGTCGCCCGCCACCGCCTCGCCAGAGTTCCGGGGC
TTCATCGCCTGCTGCCTGCAGAAGAACCCGGCAAAGCGCCTCTCCGCCGCGCAGCTGCTGCAGCACCCTGTCGCCTT
GCCGCAGCCGCAGCCGCTCGCCGCCCCGCCGTCATCG**TGA**CCGCCGCGACCTCCCAACACGGATCTCGATCCACTTC
TTGCACCACCGCGCTTCCAAATCCAGCATATCTGGTGGAGGGAGGATCGATGGAAACTGAGCTGCCGGGGGAATCTT
GGAAGGTTCTCCCGGACCATTTCGGGTATTCTTCTCGTCCACCATCGTCTCATCAAATCATCCATCCATTGTTGTCG
CTGCTGCTGCTGCTCCAGTTAAGGTTCCTTTTCCTCTCCTGTGAATCTGTGATGTTCGTTGCTACTTCCTGTTACGG
CCTTGTAATAGTAGCAAAAGCTGTGTGCACAAAAAGGATTTTGTACCGCAAGATTGGTCGAATATAGACACGATATC
CATTCTGCCATTGCCTCCCACTGCTGCAAAGGAAATTTCATCTGTACATGTAGCTCATCACATGATGATATGGCGTC
CCCATCTTGCCAGGGGCATCTTCCATTTATTTTATCTATGTTTGCTTATTATACTAGTTGCAAGTCGCCGTGGCTCG
ATGGAATTATTGGATTGGATGATTCTATATTTGCTGGTTGGATAGAGGGGAATCTCGGAATAGGAATCGGACCAGCT
TTGTTTTGTTTCTTTCTTTCAGTGTTGTAAAGTGATATGAGGGAAAGGTGTTCCTGAACCTCCTCTGCCTTTCTTGT
TTTCAATTCTGGTTTGCCTTTTCTTCGTCCGGCCAAAGATGATTTTTGTTTTGGCTATTGGCATGCACATCATGGGT
TTCAATTCATGTTTACTTTTGTTC

>TsMKK6 : contig212597
CCTCCCTCCGCTAGCCGCCACGTCCCCGGCCAACCCGACCACCCCTCGCCGTCGGCCGGACCGCATCTCCTCCGCTA
ACCCCGCACCTCCAGATCCGCGCGCTACCCTAGCCTCCCTCCGCCCGCCGCCGCCGGGAACTTTCTGCCGAGCTCGA
TCCATCCAGTGGCCGGCTACAGCGACCGCCCGCCGCGCGGATCGATCCAGCGAGCAACCGTCTGCTAGCCGTCACCT
CTCCAGTCAACCCAAGTCGCACCACGTGCCACCATTTTGACCAGCTCCGCCGCTGCTATTTTTCTCCCTAAATTCCT
CCCCTCACGCCGACGGCGCCCCAGCCGCTCCTCCCCGTCCGCCGCGCCTCCGCTCCGCGTCCATCTCCCGCCCGAGA
AACCCTAGCCAGAGGAAGGAGGACACC**ATG**AGGGGGAAGAAGCCGCTCAAGGAGCTCACACTCTCCGTGCCGGCGCA

```
GGAGACGCCCGTCGATAAGTTCCTGACGGCGAGCGGTACGTTCAAGGACGGTGAACTGCGACTTAATCAAAGAGGTT
TGCAGCTTATCTCCGAGGAAAACGGAGATGAACATCAATCAACAAAAATGAAGGTGGAAGATGTGCAGTTATCAATG
GATGACCTCGAGATGATTCAGGTCATTGGTAAGGGAAGTGGTGGTGTTGTCCAACTAGTGCGGCACAAGTGGGTGGG
CACATTTTATGCCTTGAAGGGCATTCAAATGAACATTCAGGAGTCAGTTCGCAAACAAATAGTACAGGAGCTCAAAA
TAAATCAAGCAACACAGAGCCCACATATAGTCCTTTGCCATCAATCTTTTTACCACAATGGTGTAATATATCTTGTT
CTGGAATATATGGATCGTGGATCTCTTGCAGACATAATTAAACAAGTTAAAACCATTCTGGAGCCATACCTCGCAGT
ACTTTGCAAGCAGGTTTTGGAGGGACTGTTATATCTTCATCATGAAAGGCATGTGATTCACAGAGACATAAAGCCTT
CTAACTTGTTAGTCAACCATAAAGGTGAAGTAAAGATTACTGATTTTGGGGTGAGTGCAGTGCTAGCAAGTTCGATT
GGTCAGCGTGATACATTTGTTGGAACCTACAACTATATGGCGCCTGAGCGGATTAGTGGCAGCTCCTATGACTACAA
GAGTGATGTATGGAGTTTGGGCTTGGTAATACTTGAATGCGCCATTGGTCGCTTCCCTTATACTCCTCCAGAGGGAG
AAGGTTGGTTAAGCTTCTATGAACTATTAGAAGCAATTGTTGACCAGCCACCACCTTCTGCACCTGCAGATCAGTTC
TCTCCAGAATTCTGCTCATTTATCTCTTCCTGCATACAAAAGGACCCTGCCGAGCGGAAGTCTGCTTCAGAACTCTT
GAACCACGCCTTCATCAAGAAGTTTGAGGACAAGGACCTAGACCTCCGGATCCTGGTGGAGAGCCTCGAGCAGCCCA
TGAACGTGCCCGAGTGAGTCCGTCGCTCGCTTGTAGAGGCAAGGCTTTGTGGCAGAGTTTCTTTCAGGGCTGGGCTG
CCCTCCTGACGGCAATTGTGTATTTGCTGTGAACCTGATGATTGAGTGACCAGCCCCACCCCTGTATGTGATGATGT
TATGTTGGAAAATTCCCAGAGGATTCGAATGGATTTATTAATGTATGGTAATGGATAAGAAGGCCCTTTTGTTGCGT
GTCTTGTTATGAATGAATGGATCTGCTGTGGCTAGTTGCTTGTAGTGCATGAGAATGCAAGGAACCGTGATGCATCG
TGCAGCGTCGTGTGGCGGTCCGGTTTATGTGGTTTGTACTTGGAGTGCAACCTTGGTTGTCCCCTGGGAATGACCCT
ATGTTTTTGCTAGTGATATACCCTGACTGCTG
```

>TsMKK10 : contig129481 (partial)

```
CGGAGTTCACCTCACCCACCCACCTCACCCCACCTCTCCCGCCCAACTCCCATCTCATGGCTCTCGTCCGGCAGCGC
CGCCAGCTACCCCACCTCACCCTCCCGCTCGACcACTtCGCCcTCGCGCGCCCGCcGCCGCcGCCCcccGCCGTCGC
GCCGTGCGAGGGCctcGCCcgccTCTCCGACTACGAGaGGATCTCCCAGCTCGGCCAGGGCAACGGCGGCACCGtCT
tCAAGGCGCGCCACCGCCGCACCGCGCAGCAggTCGCGCTCAAGCTCTTCGCCgCGGGGgACGGGGAtCCcTCCGCG
GCCCGCGAGGCCGAGATACTCATGCTCGCCTCGGGCGCGCCGCACGTCGTGCGCCTCCACGCCGTCATCCCGTCGCC
CGCGGCGGAGCAGCCGGcGgCGCTGGCGCTcgAGCTCGTgTCGGGGGGCTCCCTCGCGGGCCTCCTCCGCGCGaTGG
gCCGGCCCATGGGGGAGCGCCCCATCGCCGCcGTGGCGCGGCAGGCGCTGCTGGGgCTCGCGgCCCTGCACGCGCTC
CGCGTCGTGCACCGCGACCTCAAGCCcGCgAACCTGCTGgTCGGcgCGGgcGGcGAGGTGAAGATCgCCGATTtCGG
CGCCGGCAAGGTCcTGCGG
```
