# Supplementary material for: Analysis of MAPK and MAPKK gene families in wheat and related Triticeae species
Source: BMC Genomics. 2018 Mar 5;19:178. doi: 10.1186/s12864-018-4545-9 (PMC5838963; doi:10.1186/s12864-018-4545-9)
Supplement: Supplementary file 7 — Alignment and domain analysis of Triticeae MPKs. The amino acid sequences from multiple genomic copies, along with the accessions reported in Additional file 3, of all Triticeae MPKs identified were aligned in Clustal Omega (www.ebi.ac.uk/Tools/msa/clustalo/) under default settings. A lower case ‘p’ next to the gene name indicates that only a partial sequence was identified for the given gene. The PK domain is in black font, and the ‘MAP kinase’ signature (specific to MPKs) is bolded where ‘MAP kinase’ flanks (F and C) are in red font; N- and C-terminal extensions in teal font. Missing sequence information, namely ‘X’ residues, are shown in pink font. Highlighted sequences are the ATP binding signature (blue), the catalytic C-loop (red), the activation T-loop (black), CD domain (teal) and EF-hand CBP (pink). Sequence deviations from the T(E/D)Y activation loop of plant MPKs are marked in yellow font. Previously reported conserved MPK domains [18] are written in blue font above the alignment, including the conserved clade-specific docking domain found at the C-terminal end of the PK signature (highlighted grey; deviations from proposed sequence in a darker shade). Deviations from these sequences are highlighted in yellow; some of these deviations were observed in the ATP binding signature and are highlighted in a bright blue. Additional conserved sequences described as putative phosphorylation sites distinct from the T-loop [18] are marked with a box. An asterix indicates a stop codon. (PDF 230 kb) [file 12864_2018_4545_MOESM7_ESM.pdf]

Additional File 7. Alignment and domain analysis of Triticeae MPKs. The amino acid sequences from multiple genomic copies, along with the accessions reported in Additional File 3, of all Triticeae MPKs identified were aligned in Clustal Omega (www.ebi.ac.uk/Tools/msa/clustalo/) under default settings. A lower case 'p' next to the gene name indicates that only a partial sequence was identified for the given gene. The PK domain is in black font, and the 'MAP kinase' signature (specific to MPKs) is bolded where 'MAP kinase' flanks (F and C) are in red font; N- and C-terminal extensions in teal font. Missing sequence information, namely 'X' residues, are shown in pink font. Highlighted sequences are the ATP binding signature (blue), the catalytic C-loop (red), the activation T-loop (black), CD domain (teal) and EF-hand CBP (pink). Sequence deviations from the T(E/D)Y activation loop of plant MPKs are marked in yellow font. Previously reported [1] conserved MPK domains are written in blue font above the alignment, including the conserved clade-specific docking domain found at the C-terminal end of the PK signature (highlighted grey; deviations from proposed sequence in a darker shade). Deviations from these sequences are highlighted in yellow; some of these deviations were observed in the ATP binding signature and are highlighted in a bright blue. Additional conserved sequences described as putative phosphorylation sites distinct from the T-loop [1] are marked with a box. An asterix indicates a stop codon.

Reference:

1. Mohanta TK, Arora PK, Mohanta N, Parida P, Bae H. Identification of new members of the MAPK gene family in plants shows diverse conserved domains and novel activation loop variants. BMC Genomics. 2015;16:1-

MPK3

```
                                                             IGxGxYGxV             IKKIxxxF    DAxRxLRE                          FxDIYxxxELM
TuMPK3              MDGAPVAEFRPTMTHGGRFLLYNIFGNQFEITAKYQPPIMPIGRGAYGIVCSVMNFETREMVAIKKIANAFDNNMDAKRTLREIKLLRHLDHENIVGLRDVIPPAIPQSFNDVYIATELM
HvMPK3(4H)          MDGAPVAEFRPTMTHGGRFLLYNIFGNQFEITAKYQPPIMPIGRGAYGIVCSVMNFETREMVAIKKIANAFDNNMDAKRTLREIKLLKHLDHENIVGLRDVIPPAIPQSFNDVYIATELM
Locus_5782          MDGAPVAEFRPTMTHGGRFLLYNIFGNQFEITAKYQPPIMPIGRGAYGIVCSVMNFETREMVAIKKIANAFDNNMDAKRTLREIKLLKHLDHENIVGLRDVIPPAIPQSFNDVYIATELM
AetMPK3             MDGAPVAEFRPTMTHGGRFLLYNIFGNQFEITAKPQPPIMPIGRGAYGIVCSVMNFETREMVAIKKIANAFDNNMDAKRTLREIKLLRHLDHENIVGLRDVIPPAIPQSFNDVYIATELM
AAC28850            MDGAPVAEFRPTMTHGGRFLLYNIFGNQFEITAKYQPPIMPIGRGAYGIVCSVMNFETREMVASKKIANAFDNNMDAKRTLREIKLLRHLDHENIVGLRDVIPPAIPQSFNDVYIATELM
TaMPK3(4AS)         MDGAPVAEFRPTMTHGGRFLLYNIFGNQFEITAKYQPPIMPIGRGAYGIVCSVMNFETREMVAIKKIANAFDNNMDAKRTLREIKLLRHLDHENIVGLRDVIPPAIPQSFNDVYIATELM
TaMPK3(4BL)         MDGAPVAEFRPTMTHGGRFLLYNIFGNQFEITAKYQPPIMPIGRGAYGIVCSVMNFETREMVAIKKIANAFDNNMDAKRTLREIKLLRHLDHENIVGLRDVIPPAIPQSFNDVYIATELM
ScMPK3              MDGAPVAEFRPTMTHGGRFLLYNIFGNQFEITAKYQPPIMPIGRGAYGIVCSVMNFETREMVAIKKIANAFDNNMDAKRTLREIKLLRHLDHENIVGLRDVIPPAIPQSFNDVYIATELM
TsMPK3(R/A/B)       MDGAPVAEFRPTMTHGGRFLLYNIFGNQFEITAKYQPPIMPIGRGAYGIVCSVMNFETREMVAIKKIANAFDNNMDAKRTLREIKLLRHLDHENIVGLRDVIPPAIPQSFNDVYIATELM
TaMPK3(4DL)         MDGAPVAEFRPTMTHGGRFLLYNIFGNQFEITAKYQPPIMPIGRGAYGIVCSVMNFETREMVAIKKIANAFDNNMDAKRTLREIKLLRHLDHENIVGLRDVIPPATPQSFNDVYIATELM
                    ********************************** **************************** ***********************:***************** **************

                      DLxxVI                QxLRxLKYxH     HRDLKPxN LxNxNCxLKIxDFGLAR             TRWYRAPEL          IDxWS(V/I)GC
TuMPK3              DTDLHHIIRSNQELSEEHCQYFLYQLLRGLKYIHSANVIHRDLKPSNLLLNANCDLKICDFGLARPSSESDMMTEYVVTRWYRAPELLLNSTDYSAAIDVWSVGCIFMEL-------PGR
HvMPK3(4H)          DTDLHHIIRSNQELSEEHCQYFLYQLLRGLKYIHSANVIHRDLKPSNLLLNANCDLKICDFGLARPSSESDMMTEYVVTRWYRAPELLLNSTDYSAAIDVWSVGCIFMELINRAPLFPGR
Locus_5782          DTDLHHIIRSNQELSEEHCQYFLYQLLRGLKYIHSANVIHRDLKPSNLLLNANCDLKICDFGLARPSSESDMMTEYVVTRWYRAPELLLNSTDYSAAIDVWSVGCIFMELINRAPLFPGR
AetMPK3             DTDLHHIIRSNQELSEEHCQYFLYQLLRGLKYIHSANVIHRDLKPSNLLLNANCDLKICDFGLARPSSESDMMTEYVVTRWYRAPELLLNSTDYSAAIDVWSVGCIFMELINRAPLFPGR
AAC28850            DTDLHHIIRSNQELSEEHCQYFLYQLLRGLKYIHSANVIHRDLKPSNLLLNANCDLKICDFGLARPSSESDMMTEYVVTRWYRAPELLLNSTDYSAAIDVWSVGCIFMELINRAPLFPGR
TaMPK3(4AS)         DTDLHHIIRSNQELSEEHCQYFLYQLLRGLKYIHSANVIHRDLKPSNLLLNANCDLKICDFGLARPSSESDMMTEYVVTRWYRAPELLLNSTDYSAAIDVWSVGCIFMELINRAPLFPGR
TaMPK3(4BL)         DTDLHHIIRSNQELSEEHCQYFLYQLLRGLKYIHSANVIHRDLKPSNLLLNANCDLKICDFGLARPSSESDMMTEYVVTRWYRAPELLLNSTDYSAAIDVWSVGCIFMELINRAPLFPGR
ScMPK3              DTDLHHIIRSNQELSEEHCQYFLYQLLRGLKYIHSANVIHRDLKPSNLLLNANCDLKICDFGLARPSSESDMMTEYVVTRWYRAPELLLNSTDYSAAIDVWSVGCIFMELINRAPLFPGR
TsMPK3(R/A/B)       DTDLHHIIRSNQELSEEHCQYFLYQLLRGLKYIHSANVIHRDLKPSNLLLNANCDLKICDFGLARPSSESDMMTEYVVTRWYRAPELLLNSTDYSAAIDVWSVGCIFMELINRAPLFPGR
TaMPK3(4DL)         DTDLHHIIRSNQELSEEHCQYFLYQLLRGLKYIHSANVIHRDLKPSNLLLNANCDLKICDFGLARPSSESDMMTEYVVTRWYRAPELLLNSTDYSAAIDVWSVGCIFMELINRAPLFPGR
                    **************************************************************************************************************       ***

                                                                                KMLTFDP(K/R)(Q/K)RITVE(D/E)AL
TuMPK3              DHMHQMRLITEVIGTPTDDDLGFIRNEDARRYMRHLPQFPRRSFPGQFPKVQPAALDLIERMLTFNPLQRITVEEALEHPYLERLHDVADEPICTDPFSFDFEQHPLTEDQMKQLIFNEALELNPNFRY*
HvMPK3(4H)          DHMHQMRLITEVIGTPTDDDLGFIRNEDARRYMRHLPQFPRRPFPGQFPKVQPAALDLIERMLTFNPLQRITVEEALEHPYLERLHDVADEPICTDPFSFDFEQHPLTEDQMKQLIFNEALELNPNFRY*
Locus_5782          DHMHQMRLITEVIGTPTDDDLGFIRNEDARRYMRHLPQFPRRPFPGQFPKVQPAALDLIERMLTFNPLQRITVEEALEHPYLERLHDVADEPICTDPFSFDFEQHPLTEDQMKQLIFNEALELNPNFRY*
AetMPK3             DHMHQMRLITEVIGTPTDDDLGFIRNEDARRYMRHLPQFPRRSFPGQFPKVQPAALDLIERMLTFNPLQRITVEEALEHPYLERLHDVADEPICTDPFSFDFEQHPLTEDQMKQLIFNEALELNPNFRY*
AAC28850            DHMHQMRLITEVIGTPTDDDLGFIRNEDARRYMRHLPQFPRRSFPGQFPKVQPAALDLIERMLTFNPLQRITVEEALEHPYLERLHDVADEPICTDPFSFDFEQHPLTEDQMKQLIFNEALELNPNFRY*
TaMPK3(4AS)         DHMHQMRLITEVIGTPTDDDLGFIRNEDARRYMRHLPQFPRRSFPGQFPKVQPAALDLIERMLTFNPLQRITVEEALEHPYLERLHDVADEPICTDPFSFDFEQHPLTEDQMKQLIFNEALELNPNFRY*
TaMPK3(4BL)         DHMHQMRLITEVIGTPTDDDLGFIRNEDARRYMRHLPQFPRRSFPGQFPKVQPAALDLIERMLTFNPLQRITVEEALEHPYLERLHDVADEPICTDPFSFDFEQHPLTEDQMKQLIFNEALELNPNFRY*
ScMPK3              DHMHQMRLITEVIGTPTDDDLGFIRNEDARRYMRHLPQFPRRSFPGQFPKVQPAALDLIERMLTFNPLQRITVEEALEHPYLERLHDVADEPICTDPFSFDFEQHPLTEDQMKQLIFNEALELNPNFRY*
TsMPK3(R/A/B)       DHMHQMRLITEVIGTPTDDDLGFIRNEDARRYMRHLPQFPRRSFPGQFPKVQPAALDLIERMLTFNPLQRITVEEALEHPYLERLHDVADEPICTDPFSFDFEQHPLTEDQMKQLIFNEALELNPNFRY*
TaMPK3(4DL)         DHMHQMRLITEVIGTPTDDDLGFIRNEDARRYMRHLPQFPRRSFPGQFPKVQPAALDLIERMLTFNPLQRITVEEALEHPYLERLHDVADEPICTDPFSFDFEQHPLTEDQMKQLIFNEALELNPNFRY*
                    ****************************************** ***************************************************************************************
```

MPK4

```
                                                                   IGxGxYGxV             IKKIxxxF    DAxRxLRE                          FxD
ScMPK4            MDTSGGGGGAAGGAAQIQGMATHGGRYVLYNVYGNLFEVASKYAPPIRPIGRGAYGIVCAAVSSDTGEEVAIKKIGNAFDNHIDAKRTLREIKLLRHMDHENILAIKDLIRPPRRDDFKD
HvMPK4(1H)        MDTSGGGGGAAGGAAQIQGMATHGGRYVLYNVYGNLFEVASKYAPPIRPIGRGAYGIVCAAVSSDTGEEVAIKKIGNAFDNHIDAKRTLREIKLLRHMDHENILAMKDLIRPPRRDDFKD
BAJ97968.1        MDTSGGGGGAAGGAAQIQGMATHGGRYVLYNVYGNLFEVASKYAPPIRPIGRGAYGIVCAAVSSDTGEEVAIKKIGNAFDNHIDAKRTLREIKLLRHMDHENILAMKDLIRPPRRDDFKD
TaMPK4(1AL)       MDTSGGGGGAAGGAAQIQGMATHGGRYVLYNVYGNLFEVASKYAPPIRPIGRGAYGIVCAAVSSDTGEEVAIKKIGNAFDNHIDAKRTLREIKLLRHMDHENILAIKDLIRPPRRDDFKD
TsMPK4(R/A)       MDTSGGGGGAAGGAAQIQGMATHGGRYVLYNVYGNLFEVASKYAPPIRPIGRGAYGIVCAAVSSDTGEEVAIKKIGNAFDNHIDAKRTLREIKLLRHMDHENILAIKDLIRPPRRDDFKD
AetMPK4           MDN-SGGAATAGGAAQIQGMATHGGRYVLYNVYGNLXEVASKYAPPIRPIGRGAYGIVCAAVSSDTGEEVAIKKIGNAFDNHIDAKRTLREIKLLRHMDHENILAIKDLIRPPRRDDFKD
TaMPK4(1BL)       MDT-SGGGTAAGGAAQIQGMATHGGRYVLYNVYGNLFEVASKYAPPIRPIGRGAYGIVCAAVSSDTGEEVAIKKIGNAFDNHIDAKRTLREIKLLRHMDHENILAIKDLIRPPRRDDFKD
TaMPK4(1DL)       MDT-SGGGAAAGGAAQIQGMATHGGRYVLYNVYGNLFEVASKYAPPIRPIGRGAYGIVCAAVSSDTGEEVAIKKIGNAFDNHIDAKRTLREIKLLRHMDHENILAIKDLIRPPRRDDFKD
                  **. .**. :************************** ********************************************************************:**************

                  IYxxxELM  DLxxVI                QxLRxLKYxH     HRDLKPxN LxNxNCxLKIxDFGLAR             TRWYRAPEL          IDxWS^V_IGC
ScMPK4            VYIVTELMDTDLHQIIRSNQSLTDDHCQYFLYQLLRGLKYVHSANVLHRDLKPSNLFLNANCDLKIADFGLARTTSETDLMTEYVVTRWYRAPELLLNCSQYTAAIDVWSVGCILGEIIT
HvMPK4(1H)        VYIVTELMDTDLHQIIRSNQSLTDDHCQYFLYQLLRGLKYVHSANVLHRDLKPSNLFLNANCDLKIADFGLARTTSETDLMTEYVVTRWYRAPELLLNCSQYTAAIDVWSVGCILGEIIT
BAJ97968.1        VYIVTELMDTDLHQIIRSNQSLTDDHCQYFLYQLLRGLKYVHSANVLHRDLKPSNLFLNANCDLKIADFGLARTTSETDLMTEYVVTRWYRAPELLLNCSQYTAAIDVWSVGCILGEIIT
TaMPK4(1AL)       VYIVTELMDTDLHQIIRSNQSLTDDHCQYFLYQLLRGLKYVHSANVLHRDLKPSNLFLNANCDLKIADFGLARTTSETDLMTEYVVTRWYRAPELLLNCSQYTAAIDVWSVGCILGEIIT
TsMPK4(R/A)       VYIVTELMDTDLHQIIRSNQSLTDDHCQYFLYQLLRGLKYVHSANVLHRDLKPSNLFLNANCDLKIADFGLARTTSETDLMTEYVVTRWYRAPELLLNCSQYTAAIDVWSVGCILGEIIT
AetMPK4           VYIVTELMDTDLHQIIRSNQSLTDDHCQYFLYQLLRGLKYVHSANVLHRDLKPSNLFLNANCDLKIADFGLARTTSETDLMTEYVVTRWYRAPELLLNCSQYTAAIDVWSVGCILGEIIT
TaMPK4(1BL)       VYIVTELMDTDLHQIIRSNQSLTDDHCQYFLYQLLRGLKYVHSANVLHRDLKPSNLFLNANCDLKIADFGLARTTSETDLMTEYVVTRWYRAPELLLNCSQYTAAIDVWSVGCILGEIIT
TaMPK4(1DL)       VYIVTELMDTDLHQIIRSNQSLTDDHCQYFLYQLLRGLKYVHSANVLHRDLKPSNLFLNANCDLKIADFGLARTTSETDLMTEYVVTRWYRAPELLLNCSQYTAAIDVWSVGCILGEIIT
                  ************************************************************************************************************************

                                                                                    KML^V_IFDPxKRIIVDEAL
ScMPK4            RQPLFPGRDYIQQLKLITELIGSPDDSSLGF-RSDNARRYMKQLPQYPRQDFRLRFNMSDGAVDLLERMLVFDPSRRITVDEALHHPYLASLHDINEEPTCPAPFSFDFEQPSFTEEHMKELIWRETLAFNPDPPY*
HvMPK4(1H)        RQPLFPGRDYIQQLKLITELIGSPDDSSLGFLRSDNARRYMKQLPQYPRQDFRLRFNMSDGAVDLLERMLVFDPSRRITVDEALHHPYLASLHDINEEPTCPAPFSFDFEQPSFTEEHMKELIWRETLAFNPDPPY*
BAJ97968.1        RQPLFPGRDYIQQLKLITELIGSPDDSSLGFLRSDNARRYMKQLPQYPRQDFRLRFNMSDGAVDLLERMLVFDPSRRITVDEALHHPYLASLHDINEEPTCPAPFSFDFEQPSFTEEHMKELIWRETLAFNPDPPY*
TaMPK4(1AL)       RQPLFPGRDYIQQLKLITELIGSPDDSSLGFLRSDNARRYMKQLPQYPRQDFRLRFNMSDGAVDLLERMLVFDPSRRITVDEALHHPYLASLHDINEEPTCPAPFSFDFEQPSFTEEHMKELIWRETLAFNPDPPY*
TsMPK4(R/A)       RQPLFPGRDYIQQLKLITELIGSPDDSSLGFLRSDNARRYMKQLPQYPRQDFRLRFNMSDGAVDLLERMLVFDPSRRITVDEALHHPYLASLHDINEEPTCPAPFSFDFEQPSFTEEHMKELIWRETLAFNPDPPY*
AetMPK4           RQPLFPGRDYIQQLKLITELIGSPDDSSLGFLRSDNARRYMKQLPQYPRQDFRLRFNMSDGAVDLLERMLVFDPSRRITVDEALHHPYLASLHDINEEPTCPAPFSFDFEQPSFTEEHMKELIWRETLAFNPDPPY*
TaMPK4(1BL)       RQPLFPGRDYIQQLKLITELIGSPDDSSLGFLRSDNARRYMKQLPQYPRQDFRLRFNMSDGAVDLLERMLVFDPSRRITVDEALHHPYLASLHDINEEPTCPAPFSFDFEQPSFTEEHMKELIWRETLAFNPDPPY*
TaMPK4(1DL)       RQPLFPGRDYIQQLKLITELIGSPDDSSLGFLRSDNARRYMKQLPQYPRQDFRLRFNMSDGAVDLLERMLVFDPSRRITVDEALHHPYLASLHDINEEPTCPAPFSFDFEQPSFTEEHMKELIWRETLAFNPDPPY*
                  ******************************* *********************************************************************************************************
```

MPK6

```
                                                                                       IGxGxYGxV             IKKIxxxF    DAxRxLRE
AAO16560.1       MDAGGAQPPDSEMAEAGAGAA---AAAGTAPGGAMDNIQATLTHGGRFIQYNIFGNVFEVTAKYKPPILPIGKGAYGIVCSALNSETGEQVAIKKIANAFDNKIDAKRTLREIKLLRHMD
TaMPK6(7DS)      MDAGGAQPPDSEMAEAGAGA---AAAAGTAPGGAMDNIQATLTHGGRFIQYNIFGNVFEVTAKYKPPILPIGKGAYGIVCSALNSETGEQVAIKKIANAFDNKIDAKRTLREIKLLRHMD
AK333212.1       MDAGGAQPPDSEMAEAGAGA---AAAAGTAPGGAMDNIQATLTHGGRFIQYNIFGNVFEVTAKYKPPILPIGKGAYGIVCSALNSETGEQVAIKKIANAFDNKIDAKRTLREIKLLRHMD
RFL3769          MDAGGAQPPDSEMAEAGAGA---AAAAGTAPGGAMDNIQATLTHGGRFIQYNIFGNVFEVTAKYKPPILPIGKGAYGIVCSALNSETGEQVAIKKIANAFDNKIDAKRTLREIKLLRHMD
AetMPK6p         --------------------------------------------------------------------------------SALNSETGEQVAIKKIANAFDNKIDAKRTLREIKLLRHMD
ScMPK6           ----------------------------------MENIQATLTHGGRFIQYNIFGNVFEVTAKYKPPILPIGKGAYGIVCSALNSETGEQVAIKKIANAFDNKIDAKRTLREIKLLRHMD
TsMPK6(R)        ----------------------------------MENIQATLTHGGRFIQYNIFGNVFEVTAKYKPPILPIGKGAYGIVCSALNSETGEQVAIKKIANAFDNKIDAKRTLREIKLLRHMD
BAJ90351.1       MDAGGAQPPDAEMAEAGGAAAA--AAAAGAPGGAMENIQATLSHGGRFIQYNIFGNVFEVTAKYKPPILPIGKGAYGIVCSALNSETGEQVAIKKIANAFDNKIDAKRTLREIKLLRHMD
BAJ86277.1       MDAGGAQPPDAEMAEAGGAAAA--AAAAGAPGGAMENIQATLSHGGRFIQYNIFGNVFEVTAKYKPPILPIGKGAYGIVCSALNSETGEQVAIKKIANAFDNKIDAKRTLREIKLLRHMD
BAK07440.1       MDAGGAQPPDAEMAEAGGAAAA--AAAAGAPGGAMENIQATLSHGGRFIQYNIFGNVFEVTAKYKPPILPIGKGAYGIVCSALNSETGEQVAIKKIANAFDNKIDAKRTLREIKLLRHMD
TaMPK6(7BS)      MDAGGAQPPDAEMAEAGAAA---AAAAGSAPGGAMDNIQATLTHGGRFIQYNIFGNIFEVTAKYKPPILPIGKGAYGIVCSALNSETGEQVAIKKIANAFDNKIDAKRTLREIKLLRHMD
TaMPK6(7AS)      MDAGGAQPPDSEMAEAGAAAAAAAAAAGAAPGGAMDNIQATLTHGGRFIQYNIFGNVFEVTAKYKPPILPIGKGAYGIVCSALNSETGEQVAIKKIANAFDNKIDAKRTLREIKLLRHMD
HvMPK6(7H)p      ------------------------------------------------------------------------------------------------------------------------

                                   FxDIYxxxELM   DLxxVI                 QxLRxLKYxH      HRDLKPxN  LxNxNCxLKIxDFGLAR             TRWYRAPEL
AAO16560.1       HENIVAIRDIIPPAQRTAFNDVYIAYELMDTDLHQIIRSNQALSEEHCQYFLYQILRGLKYIHSANVLHRDLKPSNLLLNANCDLKICDFGLARTTSETDFMTEYVVTRWYRAPELLLNS
TaMPK6(7DS)      HENIVAIRDIIPPAQRTAFNDVYIAYELMDTDLHQIIRSNQALSEEHCQYFLYQILRGLKYIHSANVLHRDLKPSNLLLNANCDLKICDFGLARTTSETDFMTEYVVTRWYRAPELLLNS
AK333212.1       HENIVAIRDIIPPAQRTAFNDVYIAYELMDTDLHQIIRSNQALSEEHCQYFLYQILRGLKYIHSANVLHRDLKPSNLLLNANCDLKICDFGLARTTSETDFMTEYVVTRWYRAPELLLNS
RFL3769          HENIVAIRDIIPPAQRTAFNDVYIAYELMDTDLHQIIRSNQALSEEHCQYFLYQILRGLKYIHSANVLHRDLKPSNLLLNANCDLKICDFGLARTTSETDFMTEYVVTRWYRAPELLLNS
AetMPK6p         HENIVAIRDIIPPAQRTAFNDVYIAYELMDTDLHQIIRSNQALSEEHCQYFLYQILRGLKYIHSANVLHRDLKPSNLLLNANCDLKICDFGLARTTSETDFMTEYVVTRWYRAPELLLNS
ScMPK6           HENIVAIRDIIPPAQRTAFNDVYIAYELMDTDLHQIIRSNQALSEEHCQYFLYQILRGLKYIHSANVLHRDLKPSNLLLNANCDLKICDFGLARTTSETDFMTEYVVTRWYRAPELLLNS
TsMPK6(R)        HENIVAIRDIIPPAQRTAFNDVYIAYELMDTDLHQIIRSNQALSEEHCQYFLYQILRGLKYIHSANVLHRDLKPSNLLLNANCDLKICDFGLARTTSETDFMTEYVVTRWYRAPELLLNS
BAJ90351.1       HENIVAIRDIIPPAQRTAFNDVYIAYELMDTDLHQIIRSNQALSEEHCQYFLYQILRGLKYIHSANVLHRDLKPSNLLLNANCDLKICDFGLARTTSETDFMTEYVVTRWYRAPELLLNS
BAJ86277.1       HENIVAIRDIIPPAQRTAFNDVYIAYELMDTDLHQIIRSNQALSEEHCQYFLYQILRGLKYIHSANVLHRDLKPSNLLLNANCDLKICDFGLARTTSETDFMTEYVVTRWYRAPELLLNS
BAK07440.1       HENIVAIRDIIPPAQRTAFNDVYIAYELMDTDLHQIIRSNQALSEEHCQYFLYQILRGLKYIHSANVLHRDLKPSNLLLNANCDLKICDFGLARTTSETDFMTEYVVTRWYRAPELLLNS
TaMPK6(7BS)      HENIVAIRDIIPPAQRTAFNDVYIAYELMDTDLHQIIRSNQALSEEHCQYFLYQILRGLKYIHSANVLHRDLKPSNLLLNANCDLKICDFGLARTTSETDFMTEYVVTRWYRAPELLLNS
TaMPK6(7AS)      HENIVAIRDIIPPAQRTAFNDVYIAYELMDTDLHQIIRSNQALSEEHCQYFLYQILRGLKYIHSANVLHRDLKPSNLLLNANCDLKICDFGLARTTSETDFMTEYVVTRWYRAPELLLNS
HvMPK6(7H)p      ------------------------------------------------QYFLYQILRGLKYIHSANVLHRDLKPSNLLLNANCDLKICDFGLARTTSETDFMTEYVVTRWYRAPELLLNS
                                                                 ************************************************************************
```

MPK6 continued

Motifs: IDxWS$^{V}_{I}$GC … KMLTFDP$^{K}_{R}$$^{Q}_{K}$RITVE$^{D}_{E}$AL

```
AAO16560.1      SEYTAAIDVWSVGCIFMELMDRKPLFPGRDHVHQLRLLMEVFPCGISSLQLIGTPNEADLDFVNENARRYIRQLPRHARQSLSEKFPHVHPSAIDLVEKMLTFDPRQRITVEGALAHPYL
TaMPK6(7DS)     SEYTAAIDVWSVGCIFMELMDRKPLFPGRDHVHQLRLLME----------LIGTPNEADLDFVNENARRYIRQLPRHARQSLSEKFPHVHPSAIDLVEKMLTFDPRQRITVEGALAHPYL
AK333212.1      SEYTAAIDVWSVGCIFMELMDRKPLFPGRDHVHQLRLLME----------LIGTPNEADLDFVNENARRYIRQLPRHARQSLSEKFPHVHPSAIDLVEKMLTFDPRQRITVEGALAHPYL
RFL3769         SEYTAAIDVWSVGCIFMELMDRKPLFPGRDHVHQLRLLME----------LIGTPNEADLDFVNENARRYIRQLPRHARQSLSEKFPHVHPSAIDLVEKMLTFDPRQRITVEGALAHPYL
AetMPK6p        SEYTAAIDVWSVGCIFMELMDRKPLFPGRDHVHQLRLLME----------LIGTPNEADLDFVNENARRYIRQLPRHARQSLSEKFPHVHPSAIDLVEKMLTFDPRQRITVEGALAHPYL
ScMPK6          SEYTAAIDVWSVGCIFMELMDRKPLFPGRDHVHQLRLLME----------LIGTPNEADLDFVNENARRYIRQLPRHARQSLSEKFPHVHPSAIDLVEKMLTFDPRQRITVEGALAHPYL
TsMPK6(R)       SEYTAAIDVWSVGCIFMELMDRKPLFPGRDHVHQLRLLME----------LIGTPNEADLDFVNENARRYIRQLPRHARQSLSEKFPHVHPSAIDLVEKMLTFDPRQRITVEGALAHPYL
BAJ90351.1      SEYTAAIDVWSVGCIFMELMDRKPLFPGRDHVHQLRLLME----------LIGTPNEADLDFVNENARRYIRQLPRHARQSLSEKFPHVHPSAIDLVEKMLTFDPRQRITVEGALAHPYL
BAJ86277.1      SEYTAAIDVWSVGCIFMELMDRKPLFPGRDHVHQLRLLME----------LIGTPNEADLDFVNENARRYIRQLPRHARQSLSEKFPHVHPSAIDLVEKMLTFDPRQRITVEGALAHPYL
BAK07440.1      SEYTAAIDVWSVGCIFMELMDRKPLFPGRDHVHQLRLLME----------LIGTPNEADLDFVNENARRYIRQLPRHARQSLSEKFPHVHPSAIDLVEKMLTFDPRQRITVEGALAHPYL
TaMPK6(7BS)     SEYTAAIDVWSVGCIFMELMDRKPLFPGRDHVHQLRLLME----------LIGTPNEADLDFVNENARRYIRQLPRHARQSLSEKFPHVHPSAIDLVEKMLTFDPRQRITVEGALAHPYL
TaMPK6(7AS)     SEYTAAIDVWSVGCIFMELMDRKPLFPGRDHVHQLRLLME----------LIGTPNEADLDFVNENARRYIRQLPRHARQSLSEKFPHVHPSAIDLVEKMLTFDPRQRITVEGALAHPYL
HvMPK6(7H)p     SEYTAAIDVWSVGCIFMELMDRKPLFPGRDHVHQLRLLME----------LIGTPNEADLDFVNENARRYIRQLPRHARQSLSEKFPHVHPSAIDLVEKMLTFDPRQRITVEGALAHPYL
                ****************************************          **********************************************************************

AAO16560.1      ASLHDISDEPVCTMPFSFDFEQHALSEEQMKDLIHQEGITFNPDYQ*
TaMPK6(7DS)     ASLHDISDEPVCTMPFSFDFEQHALSEEQMKDLIHQEGITFNPDYQ*
AK333212.1      ASLHDISDEPVCTMPFSFDFEQHALSEEQMKDLIHQEGITFNPDYQ*
RFL3769         ASLHDISDEPVCTMPFSFDFEQHALSEEQMKDLIHQEGITFNPDYQ*
AetMPK6p        ASLHDISDEPVCTMPFSFDFEQHALSEEQMKDLIHQEGITFNPDYQ*
ScMPK6          ASLHDISDEPVCTMPFSFDFEQHALSEEQMKDLIHQEGIAFNPDYQ*
TsMPK6(R)       ASLHDISDEPVCTMPFSFDFEQHALSEEQMKDLIHQEGIAFNPDYQ*
BAJ90351.1      ASLHDISDEPVCTMPFSFDFEQHALSEEQMKDLIHQEGIAFNPDYQ*
BAJ86277.1      ASLHDISDEPVCTMPFSFDFEQHALSEEQMKDLIHQEGIAFNPDYQ*
BAK07440.1      ASLHDISDEPVCTMPFSFDFEQHALSEEQMKDLIHQEGIAFNPDYQ*
TaMPK6(7BS)     ASLHDISDEPVCTMPFSFDFEQHALSEEQMKDLIHQEGIAFNPDYQ*
TaMPK6(7AS)     ASLHDISDEPVCTMPFSFDFEQHALSEEQMKDLIHQEGIAFNPDYQ*
HvMPK6(7H)p     ASLHDISDEPVCTMPFSFDFEQHALSEEQMKDLIHQEGIAFNPDYQ*
                ***************************************:*******
```

MPK7

```
                                                         IGxGxYGxV             IKKIxxxF    DAxRxLRE                          FxDIYxxxELM  DL
TaMPK7(7DLc2)      MAMMVDPPNGMGNHGKHYYTMWQTMFEIDTKYVPIKPIGRGAYGIVCSSINQETNEKVAIKKINNVFDNRLDALRTLRELKLLRHLRHENVIALKDIMMPIHRRSFKDVYLVSELMDMDL
TuMPK7             MAMMVDPPNGMGNHGKHYYTMWQTMFEIDTKYVPIKPIARGAYGIVCSSINQETNEKVAIKKINNVFDNRVDALRTLRELKLLRHLRHENVIALKDIMMPIHRRSFKDVYLVSELMDTDL
TsMPK7(B)          MAMMVDPPNGMGNHGKHYYTMWQTMFEIDTKYVPIKPIGRGAYGIVCSSINQETNEKVAIKKINNVFDNRVDALRTLRELKLLRHLRHENVIALKDIMMPIHRRSFKDVYLVSELMDTDL
TaMPK7(7BL)        MAMMVDPPNGMGNHGKHYYTMWQTMFEIDTKYVPIKPIGRGAYGIVCSSINQETNEKVAIKKINNVFDNRVDALRTLRELKLLRHLRHENVIALKDIMMPIHRRSFKDVYLVSELMDTDL
AET10457.1         MAMMVDPPNGMGNHGKHYYTMWQTMFEIDTKYVPIKPIGRGAYGIVCSSINQETNEKVAIKKINNVFDNRVDALRTLRELKLLRHLRHENVIALKDIMMPIHRRSFKDVYLVSELMDTDL
tplb0018e09        MAMMVDPPNGMGNHGKHYYTMWQTMFEIDTKYVPIKPIGRGAYGIVCSSINQETNEKVAIKKINNVFDNRVDALRTLRELKLLRHLRHENVIALKDIMMPIHRRSFKDVYLVSELMDTDL
HvMPK7(7H)         MAMMVDPPNGIGNHGKHYYTMWQTMFEIDTKYVPIKPIGRGAYGIVCSSINQETNEKVAIKKINNVFDNRVDALRTLRELKLLRHLRHENVIALKDIMMPIHRRSFKDVYLVSELMDTDL
AK252980.1         MAMMVDPPNGIGNHGKHYYTMWQTMFEIDTKYVPIKPIGRGAYGIVCSSINQETNEKVAIKKINNVFDNRVDALRTLRELKLLRHLRHENVIALKDIMMPIHRRSFKDVYLVSELMDTDL
AetMPK7            MAMMVDPPNGMGNHGKHYYTMWQTMFEIDTKYVPIKPIGRGAYGIVCSSINQETNEKVAIKKINNVFDNRVDALRTLRELKLLRHLRHENVIALKDIMMPIHRRSFKDVYLVSELMDTDL
TaMPK7(7DLc1)      MAMMVDPPNGMGNHGKHYYTMWQTMFEIDTKYVPIKPIGRGAYGIVCSSINQETNEKVAIKKINNVFDNRVDALRTLRELKLLRHLRHENVIALKDIMMPIHRRSFKDVYLVSELMDTDL
TaMPK7(7AL)        MAMMVDPPNGMGNHGKHYYTMWQTMFEIDTKYVPIKPIGRGAYGIVCSSINQETNEKVAIKKINNVFDNRVDALRTLRELKLLRHLRHENVIALKDIMMPIHRRSFKDVYLVSELMDTDL
AET10456.1         MAMMVDPPNGMGNHGKHYYTMWQTMFEIDTKYVPIKPIGRGAYGIVCSSINQETNEKVAIKKINNVFDNRVDALRTLRELKLLRHLRHENVIALKDIMMPIHRRSFKDVYLVSELMDTDL
ScMPK7             MAMMVDPPNGMGNHGKHYYTMWQTMFEIDTKYVPIKPIGRGAYGIVCSSINQETNEKVAIKKINNVFDNRVDALRTLRELKLLRHLRHENVIALKDIMMPIHRRSFKDVYLVSELMDTDL
                   **********:***************************.*****************************:********************************************** **

                   xxVI                QxLRxLKYxH     HRDLKPxN LxNxNCxLKIxDFGLAR              TRWYRAPEL          IDxWS^V_IGC
TaMPK7(7DLc2)      HQIVKSSQPLSNDHCQYFLFQVLRGLKYLHSAGILHRDLKPGNLLVNANCDLKICDFGLARTNNTKGQFMTEYVVTRWYRAPELLLCCDNYGTSIDVWSVGCIFAELLGRKPIFPGTECL
TuMPK7             HQIVKSSQPLSNDHCQYFLFQLLRGLKYLHSAGILHRDLKPGNLLVNANCDLKICDFGLARTNNTKGQFMTEYVVTRWYRAPELLLCCDNYGTSIDVWSVGCIFAELLGRKPIFPGTECL
TsMPK7(B)          HQIVKSSQPLSNDHCQYFLFQLLRGLKYLH-AGILHRDLKPGNLLVNANCDLKICDFGLARTNNTKGQFMTEYVVTRWYRAPELLLCCDNYGTSIDVWSVGCIFAELLGRKPIFPGTECL
TaMPK7(7BL)        HQIVKSSQPLSNDHCQYFLFQLLRGLKYLHSAGILHRDLKPGNLLVNANCDLKICDFGLARTNNTKGQFMTEYVVTRWYRAPELLLCCDNYGTSIDVWSVGCIFAELLGRKPIFPGTECL
AET10457.1         HQIVKSSQPLSNDHCQYFLFQLLRGLKYLHSAGILHRDLKPGNLLVNANCDLKICDFGLARTNNTKGQFMTEYVVTRWYRAPELLLCCDNYGTSIDVWSVGCIFAELLGRKPIFPGTECL
tplb0018e09        HQIVKSSQPLSNDHCQYFLFQLLRGLKYLHSAGILHRDLKPGNLLVNANCDLKICDFGLARTNNTKGQFMTEYVVTRWYRAPELLLCCDNYGTSIDVWSVGCIFAELLGRKPIFPGTECL
HvMPK7(7H)         HQIVKSSQPLSNDHCQYFLFQLLRGLKYLHSAGILHRDLKPGNLLVNANCDLKICDFGLARTNNTKGQFMTEYVVTRWYRAPELLLCCDNYGTSIDVWSVGCIFAELLGRKPIFPGTECL
AK252980.1         HQIVKSSQPLSNDHCQYFLFQLLRGLKYLHSAGILHRDLKPGNLLVNANCDLKICDFGLARTNNTKGQFMTEYVVTRWYRAPELLLCCDNYGTSIDVWSVGCIFAELLGRKPIFPGTECL
AetMPK7            HQIVKSSQPLSNDHCQYFLFQLLRGLKYLHSAGILHRDLKPGNLLVNANCDLKICDFGLARTNNTKGQFMTEYVVTRWYRAPELLLCCDNYGTSIDVWSVGCIFAELLGRKPIFPGTECL
TaMPK7(7DLc1)      HQIVKSSQPLSNDHCQYFLFQLLRGLKYLHSAGILHRDLKPGNLLVNANCDLKICDFGLARTNNTKGQFMTEYVVTRWYRAPELLLCCDNYGTSIDVWSVGCIFAELLGRKPIFPGTECL
TaMPK7(7AL)        HQIVKSSQPLSNDHCQYFLFQLLRGLKYLHSAGILHRDLKPGNLLVNANCDLKICDFGLARTNNTKGQFMTEYVVTRWYRAPELLLCCDNYGTSIDVWSVGCIFAELLGRKPIFPGTECL
AET10456.1         HQIVKSSQPLSNDHCQYFLFQLLRGLKYLHSAGILHRDLKPGNLLVNANCDLKICDFGLARTNNTKGQFMTEYVVTRWYRAPELLLCCDNYGTSIDVWSVGCIFAELLGRKPIFPGTECL
ScMPK7             HQIVKSSQPLSNDHCQYFLFQLLRGLKYLHSAGILHRDLKPGNLLVNANCDLKICDFGLARTNNTKGQFMTEYVVTRWYRAPELLLCCDNYGTSIDVWSVGCIFAELLGRKPIFPGTECL
                   *********************:********* ****************************************************************************************
```

MPK7 continued

KMLIFDP$^{S}_{T}$KRISVTEAL

```
TaMPK7(7DLc2)     NQLKLIVNVLGTMSKADLAFIDNPKAGNYIKSLPYTPGMPLSIMYPHAHPLAIDLLQKMLVFDPSKRISVTQALEHPYMSPLYDPSANPPAQVPIDLDIDENIGTDMIREMLWQEMLQYHPGAARMVNM*-
TuMPK7            NQLKLIVNVLGTMSEADLAFIDNPKARNYIKSLPYTPGIPLSSMYPQAHPLAIDLLQKMLVFDPSKRISVTQALEHPYMSPLYDPSANPPAQVPIDLDIDENIGTDMIREMLWQGMLQYHPQAARGVMNM*
TsMPK7(B)         NQLKLIVNVLGTMSETDLAFIDNPKARNYIKSLPYTPGMPLSIMYPHAHPLAIDLLQKMLVFDPSKRISVTQALEHPYMSPLYDPSANPPAQVPIDLDIDENIGTDMIREMLWQEMLQYHPEAARMVNM*-
TaMPK7(7BL)       NQLKLIVNVLGTMSETDLAFIDNPKARNYIKSLPYTPGMPLSIMYPHAHPLAIDLLQKMLVFDPSKRISVTQALEHPYMSPLYDPSANPPAQVPIDLDIDENIGTDMIREMLWQEMLQYHPEAARMVNM*-
AET10457.1        NQLKLIVNVLGTMSETDLAFIDNPKARNYIKSLPYTPGMPLSIMYPHAHPLAIDLLQKMLVFDPSKRISVTQALEHPYMSPLYDPSANPPAQVPIDLDIDENIGTDMIREMLWQEMLQYHPEAARMVNM*-
tplb0018e09       NQLKLIVNVLGTMSETDLAFIDNPKARNYIKSLPYTPGMPLSIMYPHAHPLAIDLLQKMLVFDPSKRISVTQALEHPYMSPLYDPSANPPAQVPIDLDIDENIGTDMIREMLWQEMLQYHPEAARMVNM*-
HvMPK7(7H)        NQLKLIVNVLGTMSEADLAFIDNSKARKYIKSLPYTPGIPLSSMYPQAHPLAIDLLQKMLVFDPSKRISVTQALEHPYMSPLYDPSANPPAQVPIDLDIDENIGTDMIREMLWQEMLQYHPEAARMVNM*-
AK252980.1        NQLKLIVNVLGTMSEADLAFIDNSKARKYIKSLPYTPGIPLSSMYPQAHPLAIDLLQKMLVFDPSKRISVTQALEHPYMSPLYDPSANPPAQVPIDLDIDENIGTDMIREMLWQEMLQYHPEAARMVNM*-
AetMPK7           NQLKLIVNVLGTMSEADLAFIDNPKARNYIKSLPYTPGMPLSSMYPRAHPLAIDLLQKMLVFDPSKRISVTQALEHPYMSPLYDPSANPPAQVPIDLDIDENIGTDMIREMLWQEMLQYHPEAARMVNM*-
TaMPK7(7DLc1)     NQLKLIVNVLGTMSEADLAFIDNPKARNYIKSLPYTPGMPLSSMYPRAHPLAIDLLQKMLVFDPSKRISVTQALEHPYMSPLYDPSANPPAQVPIDLDIDENIGTDMIREMLWQEMLQYHPEAARMVNM*-
TaMPK7(7AL)       NQLKLIVNVLGTMSEADLAFIDNPKARNYIKSLPYTPGIPLSSMYPQAHPLAIDLLQKMLVFDPSKRISVTQALEHPYMSPLYDPSANPPAQVPIDLDIDENIGTDMIREMLWQEMLQYHPEAARMVNM*-
AET10456.1        NQLKLIVNVLGTMSEADLAFIDNPKARNYIKSLPYTPGIPLSSMYPQAHPLAIDLLQKMLVFDPSKRISVTQALEHPYMSPLYDPSANPPAQVPIDLDIDENIGTDMIREMLWQEMLQYHPEAARMVNM*-
ScMPK7            NQLKLIVNVLGTMSEADLAFIDNPKARNYIKSLPYTPGMPLSTMYPQAHPLAIDLLQKMLVFDPSKRISVTQALEHPYMSPLYDPSANPPAQVPIDLDIDENIGTDMIREMLWQEMLQYHPEAARMVNM*-
                  **************::******* ** :**********:*** ***:*********************************************************************** ****** *** *
```

MPK11

```
                                                                                IGxGxYGxV             IKKIxxxF    DAxRxLRE
HvMPK11(7H)       MRMEGGGVGAGGGGGGGHGGGHGLGGEAQIKGTLTHGGRYVQYNVYGNLFEVSAKYVPPIRPVGRGACGIICAAVNAHTREEVAIKKIGNAFDNQIDAKRTLREVKLLRHMNHENVISIK
BAJ95789.1        MRMEGGGVGAGGGGGGGHGGGHGLGGEAQIKGTLTHGGRYVQYNVYGNLFEVSAKYVPPIRPVGRGACGIICAAVNAQTREEVAIKKIGNAFDNQIDAKRTLREVKLLRHMNHENVISIK
ScMPK11           MRMEGGGAWA--GGGGGHGGGHGLGGEAQIKGTLTHGGRYVQYNVYGNLFEVSAKYVPPIRPVGRGACGIICAAVNAQTREEVAIKKIGNAFDNQIDAKRTLREVKLLRHMNHENVISIK
TsMPK11(U)        MRMEGGGAGA--GGGGGHGGGHGLGGEAQIKGTLTHGGRYVQYNVYGNLFEVSAKYVPPIRPVGRGACGIICAAVNAQTREEVAIKKIGNAFDNQIDAKRTLREVKLLRHMNHENVISIK
TsMPK11(A)        MRMEGGGAGA--GGGGGHGGGHGLGGEAQIKGTLTHGGRYVQYNVYGNLFEVSAKYVPPIRPVGRGACGIICAAVNAQTREEVAIKKIGNAFDNQIDAKRTLREVKLLRHMNHENVISIK
TaMPK11(7BL)      MRMEGGGAGA---GGG------GLGGEAQIKGTLTHGGRYVQYNVYGNLFEVSAKYVPPIRPVGRGACGIICAAVNAQTREEVAIKKIGNAFDNQIDAKRTLREVKLLRHMNHENVISIK
JV870088.1        MRMEGGGAGA---GGG------GLGGEAQIKGTLTHGGRYVQYNVYGNLFEVSAKYVPPIRPVGRGACGIICAAVNAQTREEVAIKKIGNAFDNQIDAKRTLREVKLLRHMNHENVISIK
TaMPK11(7DL)      MRMEGGGAGA---GGGGHGGGHGLGGEAQIKGTLTHGGRYVQYNVYGNLFEVSAKYVPPIRPVGRGACGIICAAVNAQTREEVAIKKIGNAFDNQIDAKRTLREVKLLRHMNHENVISIK
TaMPK11(7AL)      MRMEGGGAGA---GGGGHGGGHGLGGEAQIKGTLTHGGRYVQYNVYGNLFEVSAKYVPPIRPVGRGACGIICAAVNAQTREEVAIKKIGNAFDNQIDAKRTLREVKLLRHMNHENVISIK
HAAB01041577.1    MRMEGGGAGA---GGGGHGGGHGLGGEAQIKGTLTHGGRYVQYNVYGNLFEVSAKYVPPIRPVGRGACGIICAAVNAQTREEVAIKKIGNAFDNQIDAKRTLREVKLLRHMNHENVISIK
TuMPK11           MRMEGGGAGA---GGGGHGGGHGLGGEAQIKGTLTHGGRYVQYNVYGNLFEVSAKYVPPIRPVGRGACGIICAAVNAQTREEVAIKKIGNAFDNQIDAKRTLREVKLLRHMNHENVISIK
AetMPK11          MRMEGGGAGA---GGGGHGGGHGLGGEAQIKGTLTHGGRYVQYNVYGNLFEVSAKYVPPIRPVGRGACGIICAAVNAQTREEVAIKKIGNAFDNQIDAKRTLREVKLLRHMNHENVISIK
                  *******. *   ***      *******************************************************:******************************************

                            FxDIYxxxELM  DLxxVI                QxLRxLKYxH     HRDLKPxN LxNxNCxLKIxDFGLAR             TRWYRAPEL          ID
HvMPK11(7H)       DIIRPPRRENFNDVYIVYELMDTDLHHLLRSNQPLTDDHCQYFLYQVLRGLKYVHSAKVLHRDLRPSNLLLNAKCELKIGDFGLARTTTETDFMMEYVVTRWYRAPELLLNCSEYTAAID
BAJ95789.1        DIIRPPRRENFNDVYIVYELMDTDLHHLLRSNQPLTDDHCQYFLYQVLRGLKYVHSAKVLHRDLRPSNLLLNAKCELKIGDFGLARTTTETDFMMEYVVTRWYRAPELLLNCSEYTAAFD
ScMPK11           DIIRPPRRENFNDVYIVYELMDTDLHHLLRSNQTLTDDHCQYFLYQVLRGLKYVHSAKVLHRDLRPSNLLLNAKCELKIGDFGLARTTTETDFMMEYVVTRWYRAPELLLNCSEYTAAID
TsMPK11(U)        DIIRPPRRENFNDVYIVYELMDTDLHHLLRSNQPLTDDHCQYFLYQVLRGLKYVHSAKVLHRDLRPSNLLLNAKCELKIGDFGLARTTTETDFMMEYVVTRWYRAPELLLNCSEYTAAID
TsMPK11(A)        DIIRPPRRENFNDVYIVYELMDTDLHHLLRSNQPLTDDHCQYFLYQVLRGLKYVHSAKVLHRDLRPSNLLLNAKCELKIGDFGLARTTTETDFMMEYVVTRWYRAPELLLNCSEYTAAID
TaMPK11(7BL)      DIIRPTRRENFNDVYIVYELMDTDLHHLLRSNQPLTDDHCQYFLYQVLRGLKYVHSAKVLHRDLRPSNLLLNAKCELKIGDFGLARTTTETDFMMEYVVTRWYRAPELLLNCSEYTAAID
JV870088.1        DIIRPTRRENFNDVYIVYELMDTDLHHLLRSNQPLTDDHCQYFLYQVLRGLKYVHSAKVLHRDLRPSNLLLNAKCELKIGDFGLARTTTETDFMMEYVVTRWYRAPELLLNCSEYTAAID
TaMPK11(7DL)      DIIRPPRRENFNDVYIVYELMDTDLHHLLRSNQPLTDDHCQYFLYQVLRGLKYVHSAKVLHRDLRPSNLLLNAKCELKIGDFGLARTTTETDFMMEYVVTRWYRAPELLLNCSEYTAAID
TaMPK11(7AL)      DIIRPPRRENFNDVYIVYELMDTDLHHLLRSNQPLTDDHCQYFLYQVLRGLKYVHSAKVLHRDLRPSNLLLNAKCELKIGDFGLARTTTETDFMMEYVVTRWYRAPELLLNCSEYTAAID
HAAB01041577.1    DIIRPPRRENFNDVYIVYELMDTDLHHLLRSNQPLTDDHCQYFLYQVLRGLKYVHSAKVLHRDLRPSNLLLNAKCELKIGDFGLARTTTETDFMMEYVVTRWYRAPELLLNCSEYTAAID
TuMPK11           DIIRPPRRENFNDVYIVYELMDTDLHHLLRSNQPLTDDHCQYFLYQVLRGLKYVHSAKVLHRDLRPSNLLLNAKCELKIGDFGLARTTTETDFMMEYVVTRWYRAPELLLNCSEYTAAID
AetMPK11          DIIRPPRRENFNDVYIVYELMDTDLHHLLRSNQPLTDDHCQYFLYQVLRGLKYVHSAKVLHRDLRPSNLLLNAKCELKIGDFGLARTTTETDFMMEYVVTRWYRAPELLLNCSEYTAAID
                  ***** *************************** ************************************************************************************:*

                                                                                                    KML(V/I)FDPxKRIIVDEAL
HvMPK11(7H)       IWSVGCILGEIAMREPLFPGKDYVHQLRLITEL-IGSPDDTSLGFLRSDNARRYVRSLPQYPKQHFGSRFPNMSTGAMDLLERMLVFDPSKRITVDEALCHPYLASLHEINDEPVCPAPF
BAJ95789.1        IWSVGCILGEIAMREPLFPGKDYVHQLRLITEL-IGSPDDTSLGFLRSDNARRYVRSLPQYPKQHFGSRFPNMSTGAMDLLERMLVFDPSKRITVNEALCHPYLASLHEINDEPVCPAPF
ScMPK11           IWSVGCILGEIAMREPLFPGKDYVHQLRLITEL-IGSPDDTSLGFLRSDNARRYVRSLPQYPKQHFGSRFPSMSTGAVDLLERMLVFDPSKRITVDEALCHPYLASLHEINDDPVCPAPF
TsMPK11(U)        IWSVGCILGEIAMREPLFPGKDYVHQLRLITEL-IGSPDDTSLGFLRSDNARRYVRSLPQYPKQHFGSRFPSMSTGAVDLLERMLVFDPSKRITVDEALCHPYLASLHEINDDPVCPAPF
TsMPK11(A)        IWSVGCILGEIAMREPLFPGKDYVHQLRLITEL-IGSPDDTSLGFLRSDNARRYVRSLPQYPKQHFGSRFPSMSTGAMDLLERMLVFDPSKRITVDEALCHPYLASLHEINDEPVCPAPF
TaMPK11(7BL)      IWSVGCILGEIAMREPLFPGKDYVHQLRLITEL-IGSPDDTSLGFLRSDNARRYVRSLPQYPKQHFGSRFPSMSTGAMDLLERMLVFDPSKRITVDEALCHPYLASLHEINDEPVCPAPF
JV870088.1        IWSVGCILGEIAMREPLFPGKDYVHQLRLITEL-IGSPDDTSLGFLRSDNARRYVRSLPQYPKQHFGSRFPSMSTGAMDLLERMLVFDPSKRITVDEALCHPYLASLHEINDEPVCPAPF
TaMPK11(7DL)      IWSVGCILGEIATREPLFPGKDYVHQLRLITELXIGSPDDTSLGFLRSDNARRYVRSLPQYPKQHFGSRFPSMSTGAMDLLERMLVFDPSKRITVDEALCHPYLASLHEINDEPVCPAPF
TaMPK11(7AL)      IWSVGCILGEIAMREPLFPGKDYVHQLRLITEL-IGSPDDTSLGFLRSDNARRYVRSLPQYPKQHFGSRFPSMSTGAMDLLERMLVFDPSKRITVDEALCHPYLASLHEINDEPVCPAPF
HAAB01041577.1    IWSVGCILGEIAMREPLFPGKDYVHQLRLITEL-IGSPDDTSLGFLRSDNARRYVRSLPQYPKQHFGSRFPSMSTGAMDLLERMLVFDPSKRITVDEALCHPYLASLHEINDEPVCPAPF
TuMPK11           IWSVGCILGEIAMREPLFPGKDYVHQLRLITEL-IGSPDDTSLGFLRSDNARRYVRSLPQYPKQHFGSRFPSMSTGAMDLLERMLVFDPSKRITVDEALCHPYLASLHEINDEPVCPAPF
AetMPK11          IWSVGCILGEIATREPLFPGKDYVHQLRLITEL-IGSPDDTSLGFLRSDNARRYVRSLPQYPKQHFGSRFPSMSTGAMDLLERMLVFDPSKRITVDEALCHPYLASLHEINDEPVCPAPF
                  ************ ******************** *************************************.*****:*****************:****************:*******
```

MPK11 continued

```
HvMPK11(7H)        SFDFEQPSFTEEDIKELIWREALKFNPEPIH*
BAJ95789.1         SFDFEQPSFTEEDIKELIWREALKFNPEPIH*
ScMPK11            SFDFEQPSFTEEDIKELIWREALKFNPEPIH*
TsMPK11(U)         SFDFEQPSFTEEDIKELIWREALKFNPEPIH*
TsMPK11(A)         SFDFEQPSFTEEDIKELIWREALKFNPEPIH*
TaMPK11(7BL)       SFDFEQPSFTEEDIKELIWREALKFNPEPIH*
JV870088.1         SFDFEQPSFTEEDIKELIWREALKFNPEPIH*
TaMPK11(7DL)       SFDFEQPSFTEEDIKELIWREALKFNPESIH*
TaMPK11(7AL)       SFDFEQPSFTEEDIKELIWREALKFNPEPIH*
HAAB01041577.1     SFDFEQPSFTEEDIKELIWREALKFNPEPIH*
TuMPK11            SFDFEQPSFTEEDIKELIWREALKFNPEPIH*
AetMPK11           SFDFEQPSFTEEDIKELIWREALKFNPESIH*
                   **************************** ***
```

MPK14

```
                                                   IGxGxYGxV                   IKKIxxxF    DAxRxLRE                                      FxDIYxxxELM
AetMPK14          MAMLVDPPNGMGNQGKHYYSMWQTLFEIDTKYVPIKPIGRGAYGIVCSSINRETNEKVAIKKIHNVFDNRVDALRTLREL--KLLRHLRHENVISLKDIMMPVQRRSFKDVYLVYELMDT
TsMPK14(R/B)      MAMLVDPPNGMGNQGKHYYSMWQTLFEIDTKYVPIKP-GRGAYGIVCSSINRETNEKVAIKKIHNVFDNRVDALRTLREL--KLLRHLRHENVISLKDIMMPVQRRSFKDVYLVYELMDT
TaMPK14(6DS)      MAMLVDPPNGMGNQGKHYYSMWQTLFEIDTKYVPIKPIGRGAYGIVCSSINRETNEKVAIKKIHNVFDNRVDALRTLRELXLKLLRHLRHENVISLKDIMMPVQRRSFKDVYLVYELMDT
AK332648.1        MAMLVDPPNGMGNQGKHYYSMWQTLFEIDTKYVPIKPIGRGAYGIVCSSINRETNEKVAIKKIHNVFDNRVDALRTLREL--KLLRHLRHENVISLKDIMMPVQRRSFKDVYLVYELMDT
HvMPK14(6H)       MAMLVDPPNGMGNQGKHYYSMWQTLFEIDTKYVPIKPIGRGAYGIVCSSINRETNEKVAIKKIHNVFDNRVDALRTLREL--KLLRHLRHENVISLKDIMMPVQRRSFKDVYLVYELMDT
BAJ85436.1        MAMLVDPPNGMGNQGKHYYSMWQTLFEIDTKYVPIKPIGRGAYGIVCSSINRETNEKVAIKKIHNVFDNRVDALRTLREL--KLLRHLRHENVISLKDIMMPVQRRSFKDVYLVYELMDT
BAJ84961.1        MAMLVDPPNGMGNQGKHYYSMWQTLFEIDTKYVPIKPIGRGAYGIVCSSINRETNEKVAIKKIHNVFDNRVDALRTLREL--KLLRHLRHENVISLKDIMMPVQRRSFKDVYLVYELMDT
TaMPK14(6AS)      MAMLVDPPNGMGNQGKHYYSMWQTLFEIDTKYVPIKPIGRGAYGIVCSSINRETNEKVAIKKIHNVFDNRVDALRTLREL--KLLRHLRHENVISLKDIMMPVQRRSFKDVYLVYELMDT
TaMPK14(6BS)      MAMLVDPPNGMGNQGKHYYSMWQTLFEIDTKYVPIKPIGRGAYGIVCSSINRETNEKVAIKKIHNVFDNRVDALRTLREL--KLLRHLRHENVISLKDIMMPVQRRSFKDVYLVYELMDT
ScMPK14           MAMLVDPPNGMGNQGKHYYSMWQTLFEIDTKYVPIKPIGRGAYGIVCSSINRETNEKVAIKKIHNVFDNRVDALRTLREL--KLLRHLRHENVISLKDIMMPVQRRSFKDVYLVYELMDT
TuMPK14           MAMLVDPPNGMGNQGKHYYSMWQTLFEIDTKYVPIKPIGRGAYGIVCSSINRETNEKVAIKKIHNVFDNRVDALRTLREL--KLLRHLRHENVISLKDIMMPVQRRSFKDVYLVYELMDT
                  ************************************* ******************************************  **************************************

                  DLxxVI                QxLRxLKYxH     HRDLKPxN  LxNxNCxLKIxDFGLAR          TRWYRAPEL         IDxWSYIGC
AetMPK14          DLHQIIKSPQGLSNDHCQYFLFQLLRGLKYLHSAEILHRDLKPGNLLVNANCDLKICDFGLARTNSSKGQFMTEYVVTRWYRAPELLLCCDNYGTSIDVWSVGCIFAELLGRKPIFPGTE
TsMPK14(R/B)      DLHQIIKSPQGLSNDHCQYFLFQLLRGLKYLHSAEILHRDLKPGNLLVNANCDLKICDFGLARTNSSKGQFMTEYVVTRWYRAPELLLCCDNYGTSIDVWSVGCIFAELLGRKPIFPGTE
TaMPK14(6DS)      DLHQIIKSPQGLSNDHCQYFLFQLLRGLKYLHSAEILHRDLKPGNLLVNANCDLKICDFGLARTNSSKGQFMTEYVVTRWYRAPELLLCCDNYGTSIDVWSVGCIFAELLGRKPIFPGTE
AK332648.1        DLHQIIKSPQGLSNDHCQYFLFQLLRGLKYLHSAEILHRDLKPGNLLVNANCDLKICDFGLARTNSSKGQFMTEYVVTRWYRAPELLLCCDNYGTSIDVWSVGCIFAELLGRKPIFPGTE
HvMPK14(6H)       DLHQIIKSPQGLSNDHCQYFLFQLLRGLKYLHSAEILHRDLKPGNLLVNANCDLKICDFGLARTNSSKGQFMTEYVVTRWYRAPELLLCCDNYGTSIDVWSVGCIFAELLGRKPIFPGTE
BAJ85436.1        DLHQIIKSPQGLSNDHCQYFLFQLLRGLKYLHSAEILHRDLKPGNLLVNANCDLKICDFGLARTNSSKGQFMTEYVVTRWYRAPELLLCCDNYGTSIDVWSVGCIFAELLGRKPIFPGTE
BAJ84961.1        DLHQIIKSPQGLSNDHCQYFLFQLLRGLKYLHSAEILHRDLKPGNLLVNANCDLKICDFGLARTNSSKGQFMTEYVVTRWYRAPELLLCCDNYGTSIDVWSVGCIFAELLGRKPIFPGTE
TaMPK14(6AS)      DLHQIIKSPQGLSNDHCQYFLFQLLRGLKYLHSAEILHRDLKPGNLLVNANCDLKICDFGLARTNSSKGQFMTEYVVTRWYRAPELLLCCDNYGTSIDVWSVGCIFAELLGRKPIFPGTE
TaMPK14(6BS)      DLHQIIKSPQGLSNDHCQYFLFQLLRGLKYLHSAEILHRDLKPGNLLVNANCDLKICDFGLARTNSSKGQFMTEYVVTRWYRAPELLLCCDNYGTSIDVWSVGCIFAELLGRKPIFPGTE
ScMPK14           DLHQIIKSPQGLSNDHCQYFLFQLLRGLKYLHSAEILHRDLKPGNLLVNANCDLKICDFGLARTNSSKGQFMTEYVVTRWYRAPELLLCCDNYGTSIDVWSVGCIFAELLGRKPIFPGTE
TuMPK14           DLHQIIKSPQGLSNDHCQYFLFQLLRGLKYLHSAEILHRDLKPGNLLVNANCDLKICDFGLARTNSSKGQFMTEYVVTRWYRAPELLLCCDNYGTSIDVWSVGCIFAELLGRKPIFPGTE
                  ************************************************************************************************************************

                                                                   KMLIFDPSTKRISVTEAL
AetMPK14          CLNQLKLIVNVLGTMSESDLEFIDNPKARRYIIP---TLPVPLASMYPHAHPLAIDLLQKMLIFDPTKRISVTQALEHPYMSPLYDPSANPPAQVPIDLDIDENISSEMIREMMWQEMLHYHPEAAAAVNM*
TsMPK14(R/B)      CLNQLKLIVNVLGTMSESDLEFIDNPKARRYIKTLPYTPGVPLASMYPHAHPLAIDLLQKMLIFDPTKRISVTQALEHPYMSPLYDPSANPPAQVPIDLDIDENISSEMIREMMWQEMLHYHPEAATAVNM*
TaMPK14(6DS)      CLNQLKLIVNVLGTMSESDLEFIDNPKARRYIKTLPYTPGVPLASMYPHAHPLAIDLLQKMLIFDPTKRISVTQALEHPYMSPLYDPSANPPAQVPIDLDIDENISSEMIREMMWQEMLHYHPEAAAAVNM*
AK332648.1        CLNQLKLIVNVLGTMSESDLEFIDNPKARRYIKTLPYTPGVPLASMYPHAHPLAIDLLQKMLIFDPTKRISVTQALEHPYMSPLYDPSANPPAQVPIDLDIDENISSEMIREMMWQEMLHYHPEAAAAVNM*
HvMPK14(6H)       CLNQLKLIVNVLGTMSESDLEFIDNPKARRYIKTLPYTPGVPLASMYPHAHPLAIDLLQKMLIFDPTKRISVTQALEHPYMSPLYDPSANPPAQVPIDLDIDENISSEMIREMMWQEMLHYHPEAAAAVNM*
BAJ85436.1        CLNQLKLIVNVLGTMSESDLEFIDNPKARRYIKTLPYTPGVPLASMYPHAHPLAIDLLQKMLIFDPTKRISVTQALEHPYMSPLYDPSANPPAQVPIDLDIDENISSEMIREMMWQEMLHYHPEAAAAVNM*
BAJ84961.1        CLNQLKLIVNVLGTMSESDLEFIDNPKARRYIKTLPYTPGVPLASMYPHAHPLAIDLLQKMLIFDPTKRISVTQALEHPYMSPLYDPSANPPAQVPIDLDIDENISSEMIREMMWQEMLHYHPEAAAAVNM*
TaMPK14(6AS)      CLNQLKLIVNVLGTMSESDLEFIDNPKARRYIKTLPYTPGVPLASMYPHAHPLAIDLLQKMLIFDPTKRVSVTQALEHPYMSPLYDPSANPPAQVPIDLDIDENISSEMIREMMWQEMLHYHPEAATAVNM*
TaMPK14(6BS)      CLNQLKLIVNVLGTMSESDLEFIDNPKARRYIKTLPYTPGVPLASMYPHAHPLAIDLLQKMLIFDPTKRISVTQALEHPYMSPLYDPSANPPAQVPIDLDIDENISSEMIREMMWQEMLHYHPEAATAVNM*
ScMPK14           CLNQLKLIVNVLGTMSESDLEFIDNPKARRYIKTLPYTPGVPLASMYPHAHPLAIDLLQKMLIFDPTKRISVTQALEHPYMSPLYDPSANPPAQVPIDLDIDENISSEMIREMMWQEMLHYHPEAATAVNM*
TuMPK14           CLNQLKLIVNVLGTMSESDLEFIDNPKARRYIKTLPYTPGVPLASMYPHAHPLAIDLLQKMLIFDPTKRISVTQALEHPYMSPLYDPSANPPAQVPIDLDIDENISSEMIREMMWQEMLHYHPEAATAVNM*
                  ********************************     *  *****************************:********************************************************:*****
```

MPK16

```
                                      IGxGxYGxV             IKKIxxxF    DAxRxLRE                          FxDIYxxxELM  DLxxVI
TsMPK16(U1)         MDFFTEYGEGNRYKIEEVIGKGSYGVVCSALDTHTGEKVAIKKINDIFEHVSDATRILREIKLLRLLRHPDIVEIKHILLPPSRREFKDIYVVFELMESDLHQVIKANDDLTPEHYQFFL
TsMPK16(U2)         MDFFTEYGEGNRYKIEEVIGKGSYGVVCSALDTHTGEKVAIKKINDIFEHVSDATRILREIKLLRLLRHPDIVEIKHILLPPSRREFKDIYVVFELMESDLHQVIKANDDLTPEHYQFFL
AK354221.1          MDFFTEYGEGNRYKIEEVIGKGSYGVVCSALDTHTGEKVAIKKINDIFEHVSDATRILREIKLLRLLRHPDIVEIKHILLPPSRREFKDIYVVFELMESDLHQVIKANDDLTPEHYQFFL
HvMPK16(1H)         MDFFTEYGEGNRYKIEEVIGKGSYGVVCSALDTHTGEKVAIKKINDIFEHVSDATRILREIKLLRLLRHPDIVEIKHILLPPSRREFKDIYVVFELMESDLHQVIKANDDLTPEHYQFFL
AK251500.1          MDFFTEYGEGNRYKIEEVIGKGSYGVVCSALDTHTGEKVAIKKINDIFEHVSDATRILREIKLLRLLRHPDIVEIKHILLPPSRREFKDIYVVFELMESDLHQVIKANDDLTPEHYQFFL
BAJ88123.1          MDFFTEYGEGNRYKIEEVIGKGSYGVVCSALDTHTGEKVAIKKINDIFEHVSDATRILREIKLLRLLRHPDIVEIKHILLPPSRREFKDIYVVFELMESDLHQVIKANDDLTPEHYQFFL
TaMPK16(1BS)        MDFFTEYGEGNRYKIEEVIGKGSYGVVCSALDTHTGEKVAIKKINDIFEHVSDATRILREIKLLRLLRHPDIVEIKHILLPPSRREFKDIYVVFELMESDLHQVIKANDDLTPEHYQFFL
ABC54584.1          MDFFTEYGEGNRYKIEEVIGKGSYGVVCSALDTHTGEKVAIKKINDIFEHVSDATRILREIKLLRLLRHPDIVEIKHILLPPSRREFKDIYVVFELMESDLHQVIKANDDLTPEHYQFFL
AetMPK16            MDFFTEYGEGNRYKIEEVIGKGSYGVVCSALDTHTGEKVAIKKINDIFEHVSDATRILREIKLLRLLRHPDIVEIKHILLPPSRREFKDIYVVFELMESDLHQVIKANDDLTPEHYQFFL
TaMPK16(U)          MDFFTEYGEGNRYKIEEVIGKGSYGVVCSALDTHTGEKVAIKKINDIFEHVSDATRILREIKLLRLLRHPDIVEIKHILLPPSRREFKDIYVVFELMESDLHQVIKANDDLTPEHYQFFL
TuMPK16             MDFFTEYGEGNRYKIEEVIGKGSYGVVCSALDTHTGEKVAIKKINDIFEHVSDATRILREIKLLRLLRHPDIVEIKHILLPPSRREFKDIYVVFELMESDLHQVIKANDDLTPEHYQFFL
TaMPK16(1AS)        MDFFTEYGEGNRYKIEEVIGKGSYGVVCSALDTHTGEKVAIKKINDIFEHVSDATRILREIKLLRLLRHPDIVEIKHILLPPSRREFKDIYVVFELMESDLHQVIKANDDLTPEHYQFFL
                    ************************************************************************************************************************

                     QxLRxLKYxH     HRDLKPxN LxNxNCxLKIxDFGLAR                 TRWYRAPEL           IDxWS^V_IGC
TsMPK16(U1)         YQLLRGLKYIHTANVFHRDLKPKNILANADCKLKICDFGLARVAISDTPTAIFWTDYIATRWYRAPELCGSFFSKYTPAIDIWSIGCIFAELLTGKPLFPGKNVVHQLDIITDLLGTPAP
TsMPK16(U2)         YQLLRGLKYIHTANVFHRDLKPKNILANADCKLKICDFGLARVAISDTPTAIFWTDYIATRWYRAPELCGSFFSKYTPAIDIWSIGCIFAELLTGKPLFPGKNVVHQLDIITDLLGTPAP
AK354221.1          YQLLRGLKYIHTANVFHRDLKPKNILANADCKLKICDFGLARVAISDTPTAIFWTDYIATRWYRAPELCGSFFSKYTPAIDIWSIGCIFAELLTGKPLFPGKNVVHQLDIITDLLGTPSP
HvMPK16(1H)         YQLLRGLKYIHTANVFHRDLKPKNILANADCKLKICDFGLARVAISDTPTAIFWTDYIATRWYRAPELCGSFFSKYTPAIDIWSIGCIFAELLTGKPLFPGKNVVHQLDIITDLLGTPSP
AK251500.1          YQLLRGLKYIHTANVFHRDLKPKNILANADCKLKICDFGLARVAISDTPTAIFWTDYIATRWYRAPELCGSFFSKYTPAIDIWSIGCIFAELLTGKPLFPGKNVVHQLDIITDLLGTPSP
BAJ88123.1          YQLLRGLKYIHTANVFHRDLKPKNILANANCKLKICDFGLARVAISDTPTAIFWTDYIATRWYRAPELCGSFFSKYTPAIDIWSIGCIFAELLTGKPLFPGKNVVHQLDIITDLLGTPSP
TaMPK16(1BS)        YQLLRGLKYIHTANVFHRDLKPKNILANADCKLKICDFGLARVAISDTPTAIFWTDYIATRWYRAPELCGSFFSKYTPAIDIWSIGCIFAELLTGKPLFPGKNVVHQLDIITDLLGTPAP
ABC54584.1          YQLLRGLKYIHTANVFHRDLKPKNILANADCKLKICDFGLARVAISDTPTAIFWTDYIATRWYRAPELCGSFFSKYTPAIDIWSIGCIFAELLTGKPLFPGKNVVHQLDIITDLLGTPAP
AetMPK16            YQLLRGLKYIHTANVFHRDLKPKNILANADCKLKICDFGLARVAISDTPTAIFWTDYIATRWYRAPELCGSFFSKYTPAIDIWSIGCIFAELLTGKPLFPGKNVVHQLDIITDLLGTPAP
TaMPK16(U)          YQLLRGLKYIHTANVFHRDLKPKNILANADCKLKICDFGLARVAISDTPTAIFWTDYIATRWYRAPELCGSFFSKYTPAIDIWSIGCIFAELLTGKPLFPGKNVVHQLDIITDLLGTPAP
TuMPK16             YQLLRGLKYIHTANVFHRDLKPKNILANADCKLKICDFGLARVAISDTPTAIFWTDYIATRWYRAPELCGSFFSKYTPAIDIWSIGCIFAELLTGKPLFPGKNVVHQLDIITDLLGTPAP
TaMPK16(1AS)        YQLLRGLKYIHTANVFHRDLKPKNILANADCKLKICDFGLARVAISDTPTAIFWTDYIATRWYRAPELCGSFFSKYTPAIDIWSIGCIFAELLTGKPLFPGKNVVHQLDIITDLLGTPAP
                    *****************************:****************************************************************************************:*

                                                           LLE^R_KLLAFDPKDRPTAEEAL
TsMPK16(U1)         ETIARIRNEKARRYLSSMRRKKPVPFTQKFPNADPLALNLLERMLAFDPKYRPSAEEALADLYFKNIASVDREPSAQPITKLEFEFERRRITKDDIRELIYREILEYHPNMLREFLEGAE
TsMPK16(U2)         ETIARIRNEKARRYLSCMRKKHPVPLTQKFPNADPLAVRLLGRLLAFDPKDRPSAEEALADPYFASLANVEREPSRHPISKLEFEFERRRITKDDIRELIYREILEYHPNMLREFLEGAE
AK354221.1          ETIARIRNEKARRYLSSMRRKKPVPFTQKFPNADPLALNLLERMLAFDPKDRPSAEEALADLYFKNIASVDREPSAQPITKLEFEFERRRITKDDIRELIYREILEYHPNMLREFLEGAE
HvMPK16(1H)         ETIARIRNEKARRYLSSMRRKKPVPFTQKFPNADPLALNLLERMLAFDPKDRPSAEEALADLYFKNIASVDREPSAQPITKLEFEFERRRITKDDIRELIYREILEYHPNMLREFLEGAE
AK251500.1          ETIARIRNEKARRYLSSMRRKKPVPFTQKFPNADPLALNLLERMLAFDPKDRPSAEEALADLYFKNIASVDREPSAQPITKLEFEFERRRITKDDIRELIYREILEYHPNMLREFLEGAE
BAJ88123.1          ETIARIRNEKARRYLSSMRRKKPVPFTQKFPNADPLALNLLERMLAFDPKDRPSAEEALADLYFKNIASVDREPSAQPITKLEFEFERRRITKDDIRELIYREILEYHPNMLREFLEGAE
TaMPK16(1BS)        ETIARIRNEKARRYLSSMRRKKPVPFTQKFPNADPLALNLLERMLAFDPKYRPSAEEALADLYFKNIASVDREPSAQPITKLEFEFERRRITKDDIRELIYREILEYHPNMLREFLEGAE
ABC54584.1          ETIARIRNEKARRYLSSMRRKKPVPFTQKFPNADPLALNLLERMLAFDPKYRPSAEEALADLYFKNIASVDREPSAQPITKLEFEFERRRITKDDIRELIYREILEYHPNMLREFLEGAE
AetMPK16            ETIARIRNEKARRYLSSMRRKKPVPFTQKFPNADPLALNLLERMLAFDPKDRPSAEEALADLYFKNIASVDREPSAQPITKLEFEFERRRITKDDIRELIYREILEYHPNMLREFLEGAE
TaMPK16(U)          ETIARIRNEKARRYLSSMRRKKPVPFTQKFPNADPLALNLLERMLAFDPKDRPSAEEALADLYFKNIASVDREPSAQPITKLEFEFERRRITKDDIRELIYREILEYHPNMLREFLEGAE
TuMPK16             ETIARIRNEKARRYLSSMRRKKPVPFTHKFPNADPLALNLLERMLAFDPKDRPSAEEALADLYFKNIASVDREPSAQPITKLEFEFERRRITKDDIRELIYREILEYHPNMLREFLEGAE
TaMPK16(1AS)        ETIARIRNEKARRYLSSMRRKKPVPFTHKFPNADPLALNLLERMLAFDPKDRPSAEEALADLYFKNIASVDREPSAQPITKLEFEFERRRITKDDIRELIYREILEYHPNMLREFLEGAE
                    ****************.**:*:***:*:*********:.** *:****** ********** ** .:*.*:**** :**:****************************************
```

MPK16 continued

```
TsMPK16(U1)       PTNFMYPSAVDHFKKQFTFLEEHYAKGSTAAPPERQHNSLPRPSVIYSDNRPQGSDSRPQGAANITDDLSRCIIRDNAQKPRRDPASVGANRVPKELLSQGLVKWLVRHF----------
TsMPK16(U2)       PTNFMYPSAVDHFKKQFTFLEEHYAKGSTAAPPERQHNSLPRPSVIYSDNRPQGSDSRPQGAANITDDLSRCIIRDNAQKPRRDPASVGANRVPKGAAVARPGKVVGSALRYGNCSTSGT
AK354221.1        PTGFMYPSAVDHFKKQFTFLEEHYAKGSTAAPPERQHNSLPRPSVIFSDNRPQGSDSRPQGAANITDDLSRCIIRDNTQKPRRDTASVGANRVPQGAAVARPGKVVGSALRYGNCSTSGT
HvMPK16(1H)       PTGFMYPSAVDHFKKQFTFLEEHYAKGSTAAPPERQHNSLPRPSVIFSDNRPQGSDSRPQGAANITDDLSRCIIRDNTQKPRRDTASVGANRVPQGAAVARPGKVVGSALRYGNCSTSGT
AK251500.1        PTGFMYPSAVDHFKKQFTFLEEHYAKGSTAAPPERQHNSLPRPSVIFSDNRPQGSDSRPQGAANITDDLSRCIIRDNTQKPRRDTASVGANRVPQGAAVARPGKVVGSALRYGNCSTSGT
BAJ88123.1        PTGFMYPSAVDHFKKQFTFLEEHYAKGSTAAPPERQHNSLPRPSVIFSDNRPQGSDSRPQGAANITDDLSRCIIRDNTQKPRRDTASVGANRVPQGAAVARPGKVVGSALRYGNCSTSGT
TaMPK16(1BS)      PTNFMYPSAVDHFKKQFTFLEEHYAKGSTAAPPERQHNSLPRPSVIYSDNRPQGSDSRPQGAANITDDLSRCIIRDNAQKPRRDPASVGANRVPKGAAVARPGKVVGSALRYGNCSTSGT
ABC54584.1        PTNFMYPSAVDHFKKQFTFLEEHYAKGSTAAPPERQHNSLPRPSVIYSDNRPQGSDSRPQGAANITDDLSRCIIRDNAQKPRRDPASVGANRVPKGAAVARPGKVVGSALRYGNCSTSGT
AetMPK16          PTNFMYPSAVDHFKKQFTFLEEHYAKGSTAAPPERQHNSLPRPSVIYSDNRPQGSDSRPQGAANITDDLSRCIIRDNTQKPRRDPASVGANRVPQGAAVARPGKVVGSALRYGNCSTPGT
TaMPK16(U)        PTNFMYPSAVDHFKKQFTFLEEHYAKGSTAAPPERQHNSLPRPSVIYSDNRPQGSDSRPQGAANITDDLSRCIIRDNTQKPRRDPASVGANRVPQGAAVARPGKVVGSALRYGNCSTPGT
TuMPK16           PTNFMYPSAVDHFKKQFTFLEEHYAKGSTAAPPERQHNSLPRPSVIYSDNRPQCSDSRPQGAANITDDLSRCIIRDNAQKPRRDPASVGANRVPQGAAVARPGKVVGSALRYGNCSTSGT
TaMPK16(1AS)      PTNFMYPSAVDHFKKQFTFLEEHYAKGSTAAPPERQHNSLPRPSVIYSDNRPQCSDSRPQGAANITDDLSRCIIRDNAQKPRRDPASVGANRVPQGAAVARPGKVVGSALRYGNCSTSGT
                  **.*******************************************:****** ***********************:****** *********:        * :   :

TsMPK16(U1)       ------VMVTVQHLVPSNMNSEGSSQAKELFQTVFLRAAHT---LEEITPARAKQVKL----KGST----
TsMPK16(U2)       EQYEQRRVITSQGIVPNGVPSGSSYPRRNNTCKSETGEAERIDVSQAVPPKPYTGNKLPATVDGRNGHW*
AK354221.1        EQYEQRRVITGQGIFPNGVPSGSSYPRRNNTCKSETGEAERIDVSQAGPPKPYTGNKLPATVDGRNGHW*
HvMPK16(1H)       EQYEQRRVITGQGIVPNGVPSGSSYPRRNNTCKSETGEAERIDVSQAGPPKPYTGNKLPATVDGRNGHW*
AK251500.1        EQYEQRRVITGQGIVPNGVPSGSSYPRRNNTCKSETGEAERIDVSQAGPPKPYTGNKLPATVDGRNGHW*
BAJ88123.1        EQYEQRRVITGQGIVPNGVPSGSSYPRRNNTCKSETGEAERIDVSQAGPPKPYTGNKLPATVDGRNGHW*
TaMPK16(1BS)      EQYEQRRVITSQGIVPNGVPSGSSYPRRNNTCKSETGEAERIDVSQAVPPKPYTGNKLPATVDGRNGHW*
ABC54584.1        EQYEQRRVITSQGIVPNGVPSGSSYPRRNNTCKSETGEAERIDVSQAVPPKPYTGNKLPATVDGRNGHW*
AetMPK16          EQYEQRRVITSQGVVPNGVPSGSSYPRRNNTCKSETGEAERIDVSQAGPPKPYTGNKLPATVDGRNGHW*
TaMPK16(U)        EQYEQRRVITSQGVVPNGVPSGSSYPRRNNTCKSETGEAERIDVSQAGPPKPYTGNKLPATVDGRNGHW*
TuMPK16           EQYEQRRVVTSQGIVPNGVPSGSSYPRRNNTCKSETGEAERIDVSQAGPPKPYTGNKLPATVDGRNGHW*
TaMPK16(1AS)      EQYEQRRVVTSQGIVPNGVPSGSSYPRRNNTCKSETGEAERIDVSQAGPPKPYTGNKLAATVDGRNGHW*
                         ::* * :.*..: * .*   ::   .     *.     :   *   .  **    .* .
```

MPK17

```
                                                                                                    IGxGxYGxV             IKKIxx
TaMPK17(U)      MGGGNGIVDGFRRLFHRRTPSGSVLGSSNQSSAGEDSSDVEAVEDLDLVGLRPIRVPKRKMPLPVESHKKNIMEKEFFTEYGEASQYQIQEVVGKGSYGVVAAAIDTRTGERVAIKKIND
TsMPK17(Ub)     MGGGNGIVDGFRRLFHRRTPSGSVLGSSNQSSAGEDSSDVEAVEDLDLVGLRPIRVPKRKMPLPVESHKKNIMEKEFFTEYGEASQYQIQEVVGKGSYGVVAAAIDTRTGERVAIKKIND
ABD97883.1      MGGGNGIVDGFRRLFHRRTPSGSVLGSSNQSSAGEDSSDVEAVEDLDLVGLRPIRVPKRKMPLPVESHKKNIMEKEFFTEYGEASQYQIQEVVGKGSYGVVAAAIDTRTGERVAIKKIND
AetMPK17        MGGGNGIVDGFRRLFHRRTPSGSVLGSSNQSSAGEDSSDVEAAEGLDLVGLRPIRVPKRKMPLPVESHKKNIMEKEFFTEYGEASQYQIQEVVGKGSYGVVAAAIDTRTGERVAIKKIND
TaMPK17(7DL)    MGGGNGIVDGFRRLFHRRTPSGSVLGSSNQSSAGEDSSDVEAAEGLDLVGLRSIRVPKRKMPLPVESHKKNIMEKEFFTEYGEASQYQIQEVVGKGSYGVVAAAIDTRTGERVAIKKIND
AAX20165.1      MGGGNGIVDGFRRLFHRRTPSGSVLGSSNQSSAGEDSSDVEAAEGLDLVGLRSIRVPKRKMPLPVESHKKNIMEKEFFTEYGEASQYQIQEVVGKGSYGVVAAAIDTRTGERVAIKKIND
AAX20166.1      MGGGNGIVDGFRRLFHRRTPSGSVLGSSNQSSAGEDSSDVEAAEGLDLVGLRSIRVPKRKMPLPVESHKKNIMEKEFFTEYGEASQYQIQEVVGKGSYGVVAAAIDTRTGERVAIKKIND
TsMPK17(R)      MGGGNGIVDGFRRLFHRRTPSGSVLASSNQSSAGEDPSDVEAVEDLDLVGLRPIRVPKRKMPLPVESHKKNIMEKEFFTEYGEASQYQIQEVVGKGSYGVVAAAIDTRTGERVAIKKIND
ScMPK17         MGGGNGIVDGFRRLFHRRTPSGSVLASSNQSSAGEDPSDVEAVEDLDLVGLRPIRVPKRKMPLPVESHKKNIMEKEFFTEYGEASQYQIQEVVGKGSYGVVAAAIDTRTGERVAIKKIND
HvMPK17(7H)     MGGGNGIVDGFRRLFHRRTPSGSVLGSSNQSSAGEDSSELEAVEDLDLVGLRPIRVPKRKMPLPVESHKKNIMEKEFFTEYGEASQYQIQEVVGKGSYGVVAAAIDTRTGERVAIKKIND
BAK07695.1      MGGGNGIVDGFRRLFHRRTPSGSVLGSSNQSSAGEDSSELEAVEDLDLVGLRPIRVPKRKMPLPVESHKKNIMEKEFFTEYGEASQYQIQEVVGKGSYGVVAAAIDTRTGERVAIKKIND
AK252439.1      MGGGNGIVDGFRRLFHRRTPSGSVLGSSNQSSAGEDSSELEAVEDLDLVGLRPIRVPKRKMPLPVESHKKNIMEKEFFTEYGEASQYQIQEVVGKGSYGVVAAAIDTRTGERVAIKKIND
ABC65848.1*     MGGGNGIVDGFRRLFHRRTPSGSVLGSSNQSSAGEDSSDAEAAEDLDLVGLRPIRVPKRKMPLPVESHKKNIMEKEFFTEYGEASQYQIQEVVGKGSYGVVAAAIDTRTGERVAIKKIND
TuMPK17p        MGGGNGIVDGFRRLFHRRTPSGSVFGSSNQSSAGEDSSDAEAAEDLDLVGLRPIRVPKRKMPLPVESHKKNIMEKEFFTEYGEASQYQIQEVVGKGSYGVVAAAIDTRTGERVAIKKIND
TaMPK17(7AL)    MGGGNGIVDGFRRLFHRRTPSGSVLGSSNQSSAGEDSSDAEAAEDLDLVGLRPIRVPKRKMPLPVESHKKNIMEKEFFTEYGEASQYQIQEVVGKGSYGVVAAAIDTRTGERVAIKKIND
TsMPK17(A)      MGGGNGIVDGFRRLFHRRTPSGSVLGSSNQSSAGEDSSDAEAAEDLDLVGLRPIRVPKRKMPLPVESHKKNIMEKEFFTEYGEASQYQIQEVVGKGSYGVVAAAIDTRTGERVAIKKIND
ABC54587.1      MGGGNGIVDGFRRLFHRRTPSGSVLGSSNQSSAGEDSSDAEAAEDLDLVGLRPIRVPKRKMPLPVESHKKNIMEKEFFTEYGEASQYQIQEVVGKGSYGVVAAAIDTRTGERVAIKKIND
AK333198.1*     MGGGNGIVDGFRRLFHRRTPSGSVLGSSNQSSAGEDSSDAEAAEDLDLVGLRPIRVPKRKMPLPVESHKKNIMEKEFFTEYGEASQYQIQEVVGKGSYGVVAAAIDTRTGERVAIKKIND
                ************************:.********** *: **.*.******* *******************************************************************

                xF    DAxRxLRE                          FxDIYxxxELM  DLxxVI                QxLRxLKYxH     HRDLKPxN LxNxNCxLKIxDFGLAR
TaMPK17(U)      VFEHVSDATRILREVKLLRLLRHPDVVEIKHIMLPPSRREFQDIYVVFELMESDLHQVIRANDDLTAEHYQFFLYQLLRALKYIHGANVFHRDLKPKNILANADCKLKICDFGLARVSFN
TsMPK17(Ub)     VFEHVSDATRILREVKLLRLLRHPDVVEIKHIMLPPSRREFQDIYVVFELMESDLHQVIRANDDLTAEHYQFFLYQLLRALKYIHGANVFHRDLKPKNILANADCKLKICDFGLARVSFN
ABD97883.1      VFEHVSDATRILREVKLLRLLRHPDVVEIKHIMLPPSRKEFQDIYVVFELMESDLHHVIRANDDLTAEHYQFFLYQLLRALKYIHGANVFHRDLKPKNILANADCKLKICDFGLARVSFN
AetMPK17        VFEHVSDATRILREVKLLRLLRHPDVVEIKHIMLPPSRREFQDIYVVFELMESDLHQVIRANDDLTAEHYQFFLYQLLRALKYIHGANVFHRDLKPKNILANADCKLKICDFGLARVSFN
TaMPK17(7DL)    VFEHVSDATRILREVKLLRLLRHPDVVEIKHIMLPPSRREFQDIYVVFELMESDLHQVIRANDDLTAEHYQFFLYQLLRALKYIHGANVFHRDLKPKNILANADCKLKICDFGLARVSFN
AAX20165.1      VFEHVSDATRILREVKLLRLLRHPDVVEIKHIMLPPSRREFQDIYVVFELMESDLHQVIRANDDLTAEHYQFFLYQLLRALKYIHGANVFHRDLKPKNILANADCKLKICDFGLARVSFN
AAX20166.1      VFEHVSDATRILREVKLLRLLRHPDVVEIKHIMLPPSRREFQDIYVVFELMESDLHQVIRANDDLTAEHYQFFLYQLLRALKYIHGANVFHRDLKPKNILANADCKLKICDFGLARVSFN
TsMPK17(R)      VFEHVSDATRILREVKLLRLLRHPDVVEIKHIMLPPSRREFQDIYVVFELMESDLHQVIRANDDLTAEHYQFFLYQLLRALKYIHGANVFHRDLKPKNILANADCKLKICDFGLARVSFN
ScMPK17         VFEHVSDATRILREVKLLRLLRHPDVVEIKHIMLPPSRREFQDIYVVFELMESDLHQVIRANDDLTAEHYQFFLYQLLRALKYIHGANVFHRDLKPKNILANADCKLKICDFGLARVSFN
HvMPK17(7H)     VFEHVSDATRILREVKLLRLLRHPDVVEIKHIMLPPSRREFQDIYVVFELMESDLHQVIRANDDLTAEHYQFFLYQLLRALKYIHGANVFHRDLKPKNILANADCKLKICDFGLARVSFN
BAK07695.1      VFEHVSDATRILREVKLLRLLRHPDVVEIKHIMLPPSRREFQDIYVVFELMESDLHQVIRANDDLTAEHYQFFLYQLLRALKYIHGANVFHRDLKPKNILANADCKLKICDFGLARVSFN
AK252439.1      VFEHVSDATRILREVKLLRLLRHPDVVEIKHIMLPPSRREFQDIYVVFELMESDLHQVIRANDDLTAEHYQFFLYQLLRALKYIHGANVFHRDLKPKNILANADCKLKICDFGLARVSFN
ABC65848.1*     VFEHVSDATRILREVKLLRLLRHPDVVEIKHIMLPPSRREFQDIYVVFELMESDLHQVIRANDDLTAEHYQFFLYQLLRALKYIHGANVFHRDLKPKNILANADCKLKICDFGLARVSFN
TuMPK17p        VFEHVSDATRILREVKLLRLLRHPDVVEIKHIMLPPSRREFQDIYVVFELMESDLHQVIRANDDLTAEHYQFFLYQLLRALKYIHGANVFHRDLKPKNILANADCKLKICDFGLARVSFN
TaMPK17(7AL)    VFEHVSDATRILREVKLLRLLRHPDVVEIKHIMLPPSRREFQDIYVVFELMESDLHQVIRANDDLTAEHYQFFLYQLLRALKYIHGANVFHRDLKPKNILANADCKLKICDFGLARVSFN
TsMPK17(A)      VFEHVSDATRILREVKLLRLLRHPDVVEIKHIMLPPSRREFQDIYVVFELMESDLHQVIRANDDLTAEHYQFFLYQLLRALKYIHGANVFHRDLKPKNILANADCKLKICDFGLARVSFN
ABC54587.1      VFEHVSDATRILREVKLLRLLRHPDVVEIKHIMLPPSRREFQDIYVVFELMESDLHQVIRANDDLTAEHYQFFLYQLLRALKYIHGANVFHRDLKPKNILANADCKLKICDFGLARVSFN
AK333198.1*     VFEHVSDATRILREVKLLRLLRHPDVVEIKHIMLPPSRREFQDIYVVFELMESDLHQVIRANDDLTAEHYQFFLYQLLRALKYIHGANVFHRDLKPKNILANADCKLKICDFGLARVSFN
                **************************************:****************:****************************************************************
```

MPK17 continued

TRWYRAPEL **IDxWS$^{V}_{I}$GC** **LLE$^{R}_{K}$LLA**

```
TaMPK17(U)      DAPSAIFWTDYVATRWYRAPELCGSFFSKYTPAIDIWSIGCIFAELLTGRPLFPGKNVVHQLDIITDLLGTPSSETLSRIRNEKARRYLSCMRKKHPVPLTQKFPNADPLAVRLLGRLLA
TsMPK17(Ub)     DAPSAIFWTDYVATRWYRAPELCGSFFSKYTPAIDIWSIGCIFAELLTGRPLFPGKNVVHQLDIITDLLGTPSSETLSRIRNEKARRYLSCMRKKHPVPLTQKFPNADPLAVRLLGRLLA
ABD97883.1      DAPSAIFWTDYVATRWYRAPELCGSFFSKYTPAIDIWSIGCIFAELLTGRPLFPGKNVVHQLDIITDLLGTPSSETLSRIRNEKARRYLSCMRKKHPVPLTQKFPNADPLAVRLLGRLLA
AetMPK17        DAPSAIFWTDYVATRWYRAPELCGSFFSKYTPAIDIWSIGCIFAELLTGRPLFPGKNVVHQLDIITDLLGTPSSE----IRNEKARRYLSCMRKKHPVPLTQKFPNADPLAVRLLGRLLA
TaMPK17(7DL)    DAPSAIFWTDYVATRWYRAPELCGSFFSKYTPAIDIWSIGCIFAELLTGRPLFPGKNVVHQLDIITDLLGTPSSETLSRIRNEKARRYLSCMRKKHPVPLTQKFPNADPLAVRLLGRLLA
AAX20165.1      DAPSAIFWTDYVATRWYRAPELCGSFFSKYTPAIDIWSIGCIFAELLTGRPLFPGKNVVHQLDIITDLLGTPSSETLSRIRNEKARRYLSCMRKKHPVPLTQKFPNADPLAVRLLGRLLA
AAX20166.1      DAPSAIFWTDYVATRWYRAPELCGSFFSKYTPAIDIWSIGCIFAELLTGRPLFPGKNVVHQLDIITDLLGTPSSETLSRIRNEKARRYLSCMRKKHPVPLTQKFPNADPLAVRLLGRLLA
TsMPK17(R)      DAPSAIFWTDYVATRWYRAPELCGSFFSKYTPAIDIWSIGCIFAELLTGRPLFPGKNVVHQLDIITDLLGTPSSETLSRIRNEKARRYLSCMRKKHPVPLTQKFPNADPLAVRLLGRLLA
ScMPK17         DAPSAIFWTDYVATRWYRAPELCGSFFSKYTPAIDIWSIGCIFAELLTGRPLFPGKNVVHQLDIITDLLGTPSSETLSRIRNEKARRYLSCMRKKHPVPLTQKFPNADPLAVRLLGRLLA
HvMPK17(7H)     DAPSAIFWTDYVATRWYRAPELCGSFFSKYTPAIDIWSIGCIFAELLTGRPLFPGKNVVHQLDIITDLLGTPSSETLSRIRNEKARRYLSCMRKKHPVPLTQKFPNADPLAVRLLGRLLA
BAK07695.1      DAPSAIFWTDYVATRWYRAPELCGSFFSKYTPAIDIWSIGCIFAELLTGRPLFPGKNVVHQLDIITDLLGTPSSETLSRIRNEKARRYLSCMRKKHPVPLTQKFPNADPLAVRLLGRLLA
AK252439.1      DAPSAIFWTDYVATRWYRAPELCGSFFSKYTPAIDIWSIGCIFAELLTGRPLFPGKNVVHQLDIITDLLGTPSSETLSRIRNEKARRYLSCMRKKHPVPLTQKFPNADPLAVRLLGRLLA
ABC65848.1*     DAPSAIFWTDYVATRWYRAPELCGSFFSKYTPAIDIWSIGCIFAELLTGRPLFPGKNVVHQLDIITDLLGTPSSETLSRIRNEKARRYLSCMRKKHPVPLTQKFPNADPLAVRLLGRLLA
TuMPK17p        DAPSAIFWTDYVATRWYRAPELCGSFFSKYTPAIDIWSIGCIFAELLTGRPLF--------------------------------------------------------------------
TaMPK17(7AL)    DAPSAIFWTDYVATRWYRAPELCGSFFSKYTPAIDIWSIGCIFAELLTGRPLFPGKNVVHQLDIITDLLGTPSSETLSRIRNEKARRYLSCMRKKHPVPLTQKFPNADPLAVRLLGRLLA
TsMPK17(A)      DAPSAIFWTDYVATRWYRAPELCGSFFSKYTPAIDIWSIGCIFAELLTGRPLFPGKNVVHQLDIITDLLGTPSSETLSRIRNEKARRYLSCMRKKHPVPLTQKFPNADPLAVRLLGRLLA
ABC54587.1      DAPSAIFWTDYVATRWYRAPELCGSFFSKYTPAIDIWSIGCIFAELLTGRPLFPGKNVVHQLDIITDLLGTPSSETLSRIRNEKARRYLSCMRKKHPVPLTQKFPNADPLAVRLLGRLLA
AK333198.1*     DAPSAIFWTD*VATRWYRAPELCGSFFSKYTPAIDIWSIGCIFAELLTGRPLFPGKNVVHQLDIITDLLGTPSSETLSRIRNEKARRYLSCMRKKHPVPLTQKFPNADPLAVRLLGRLLA
                ********** ******************************************
```

FDPKDRPTAEEAL

```
TaMPK17(U)      FDPKDRPSAEEALADPYFASLANVEREPSRHPISKLEFEFERRKVTKDDVRELIYREILEYHPQMLEEYMKGGDQISFLYPSGVDRFKRQFAHLEEHYSKGERGSPLQRKHASLPRQRVG
TsMPK17(Ub)     FDPKDRPSAEEALADPYFASLANVEREPSRHPISKLEFEFERRKVTKDDVRELIYREILEYHPQMLEEYMKGGDQISFLYPSGVDRFKRQFAHLEEHYSKGERGSPLQRKHASLPRQRVG
ABD97883.1      FDPKDRPSAEEALADPYFASLANVEREPSRHPISKLEFEFERRKVTKDDVRELIYREILEYHPQMLEEYMKGGDQISFLYPSGVDRFKRQFAHLEEHYSKGERGSPLQRKHASLPRQRVG
AetMPK17        FDPKDRPSAEEALADPYFASLANVEREPSRHPISKLEFEFERRKVTKDDVRELIYREILEYHPQMLEEYMKGGDQISFLYPSGVDRFKRQFAHLEEHYSKGERGSPLQRKHASLPRQRVG
TaMPK17(7DL)    FDPKDRPSAEEALADPYFASLANVEREPSRHPISKLEFEFERRKVTKDDVRELIYREILEYHPQMLEEYMKGGDQISFLYPSGVDRFKRQFAHLEEHYSKGERGSPLQRKHASLPRQRVG
AAX20165.1      FDPKDRPSAEEALADPYFASLANVEREPSRHPISKLEFEFERRKVTKDDVRELIYREILEYHPQMLEEYMKGGDQISFLYPSGVDRFKRQFAHLEEHYSKGERGSPLQRKHASLPRQRVG
AAX20166.1      FDPKDRPSAEEALADPYFASLANVEREPSRHPISKLEFEFERRKVTKDDVRELIYREILEYHPQMLEEYMKGGDQISFLYPSGVDRFKRQFAHLEEHYSKGERGSPLQRKHASLPRQRVG
TsMPK17(R)      FDPKDRPSAEEALADPYFASLANVEREPSRHPISKLEFEFERRKVTKDDVRELIYREILEYHPQMLEEYMKGGDQISFLYPSGVDRFKRQFAHLEEHYSKGERGSPLQRKHASLPRQRVG
ScMPK17         FDPKDRPSAEEALADPYFASLANVEREPSRHPISKLEFEFERRKVTKDDVRELIYREILEYHPQMLEEYMKGGDQISFLYPSGVDRFKRQFAHLEEHYSKGERGSPLQRKHASLPRQRVG
HvMPK17(7H)     FDPKDRPSAEEALADPYFASLANVEREPSRHPISKLEFEFERRKVTKDDVRELIYREVKRHSLGFL-------------FCVNNTFYVSR*-----------------------------
BAK07695.1      FDPKDRPSAEEALADPYFASLANVEREPSRHPISKLEFEFERRKVTKDDVRELIYREVKRHSLGFL-------------FCVNNTFYVSR*-----------------------------
AK252439.1      FDPKDRPSAEEALADPYFASLANVEREPSRHPISKLEFEFERRKVTKDDVRELIYREILEYHPQMLEEYMKGGDQISFLYPSGVDRFKRQFAHLEEHYSKGERGSPLQRKHASLPRQRVG
ABC65848.1*     FDPKDRPSAEEVNSSTFV------------------------------------------------------------------------------------------------------
TuMPK17p        ------------------------------------------------------------------------------------------------------------------------
TaMPK17(7AL)    FDPKDRPSAEEALADPYFASLANVEREPSRHPISKLEFEFERRKVTKDDVRELIYREILEYHPQMLEEYMKGGDQISFLYPSGVDRFKRQFAHLEEHYSKGERGSPLQRKHASLPRQRVG
TsMPK17(A)      FDPKDRPSAEEALADPYFASLANVEREPSRHPISKLEFEFERRKVTKDDVRELIYREILEYHPQMLEEYMKGGDQISFLYPSGVDRFKRQFAHLEEHYSKGERGSPLQRKHASLPRQRVG
ABC54587.1      FDPKDRPSAEEALADPYFASLANVEREPSRHPISKLEFEFERRKVTKDDVRELIYREILEYHPQMLEEYMKGGDQISFLYPSGVDRFKRQFAHLEEHYSKGERGSPLQRKHASLPRQRVG
AK333198.1*     FDPKDRPSAEEALADPYFASLANVEREPSRHPISKLEFEFERRKVTKDDVRELIYREILEYHPQMLEEYMKGGDQISFLYPSGVDRFKRQFAHLEEHYSKGERGSPLQRKHASLPRQRVG
```

MPK17 continued

```
TaMPK17(U)         ASNDGNNEQHISDQEMSAEPDAHGAVSPQKSQDAPSVGPNGLSPTSLSSRTYLKSASISASKCVVVNPNKQPEYDDAISEETEGAVDGLSEKVSKMHA*
TsMPK17(Ub)        ASNDGNNEQHISDQEMSAEPDAHGAVSPQKSQDAPSVGPNGLSPTSLSSRTYLKSASISASKCVVVNPNKQPEYDDAISEETEGAVDGLSEKVSKMHA*
ABD97883.1         ASNDGNNEQHISDQEMSAEPDAHGTVSPQKSQDAPSVGPNGLSPTSLSSRTYLKSASISASKCVVVNPNKQPEYDDAISEETEGAVDGLSEKVSKMHA*
AetMPK17           ASNDGNNEQHISDQEMSAEPDAHGTVSPQKSQDAAGVGQNGLSPTSLSSRTYLKSASISASKCVVVNPNKQPEYDDAISEETEGAVDGLSEKVSKMHA*
TaMPK17(7DL)       ASNDGNNEQHISDQEMSAEPDAHGTVSPQKSQDAAGVGQNGLSPTSLSSRTYLKSASISASKCVVVNPNKQPEYDDAISEETEGAVDGLSEKVSKMHA*
AAX20165.1         ASNDGNNEQHISDQEMSAEPDAHGTVSPQKSQDAAGVGQNGLSPTSLSSRTYLKSASISASKCVVVNPNKQPEYDDAISEETEGAVDGLSEKVSKMHA*
AAX20166.1         ASNDGNNEQHISDQEMSAEPDAHGAVSPQKSQDAPGVGQNGLSPTSLSSRTYLKSASISASKCVVVNPNKQPEYDDAISEETEGAVDGLSEKVSKMHA*
TsMPK17(R)         ASNDGNNEQHISDQEMSAEPDAHGTESPQKAQDAPGVGQNGLSPTSLSSRTYLKSASISASKCVVVNPNKQPEYDDAISEEAEGAVDGLSEKVSKMHA*
ScMPK17            ASNDGNNEQHISDQEMSAEPDAHGTESPQKAQDAPGVGQNGLSPTSLSSRTYLKSASISASKCVVVNPNKQPEYDDAISEEAEGAVDGLSEKVSKMHA*
HvMPK17(7H)        ----------------------------------------------------------------------------------------------------
BAK07695.1         ----------------------------------------------------------------------------------------------------
AK252439.1         ASNDGNNEQHISDQEMSAEPDARGAVSPQKPQDAPGVGQNGLSPTSLSSRTYLKSASISASKCVVVNPNKQPEYDDAISEETEGAVDGLSEKVSKMHA*
ABC65848.1*        ----------------------------------------------------------------------------------------------------
TuMPK17p           ----------------------------------------------------------------------------------------------------
TaMPK17(7AL)       ASNDGNNEQHISDQEMSAEPDAHGAVSPQKSQDAPGVGQNGLSPTSLSSRTYLKSASISASKCVVVNPNKQPEYDDAISEETEGAVDGLSEKVSKMHA*
TsMPK17(A)         ASNDGNNEQHISDQEMSAEPDAHGAVSPQKSQDAPGVGQNGLSPTSLSSRTYLKSASISASKCVVVNPNKQPEYDDAISEETEGAVDGLSEKVSKMHA*
ABC54587.1         ASNDGNNEQHISDQEMSAEPDAHGAVSPQKSQDAPGVGQNGLSPTSLSSRTYLKSASISASKCVVVNPNKQPEYDDAISEETEGAVDGLSEKVSKMHA*
AK333198.1*        ASNDGNNEQHISDQEMSAEPDAHGAVSPQKSQDAPGVGQNGLSPTSLSSRTYLKSASISASKCVVVNPNKQPEYDDAISEETEGAVDGLSEKVSKMHA*
```

MPK20-1

```
AK252802.1p       ------------------------------------------------------------------------------------------------------------------------
TaMPK20-1(3B)     ------------------------------------------------------------------------------------------MQQDQRKKSSAEAEFFTEYGDASRYKIQEI
TsMPK20-1(U2)p*   VHGGPRKRIVAVAFPCRWAKLLAFLVGFSCTWGCTTT*LTSGACHHFNFATNTSRSCFSRKFL*STCTAVEFGINSLILVVASQ*LLILHVVH*KYLESSAEAEFFTEYGDASRYKIQEI
TsMPK20-1(U1)     ------------------------------------------------------------------------------------------MQQDQRKKSSAEAEFFTEYGDASRYKIQEI
ScMPK20-1         ------------------------------------------------------------------------------------------MISVILFQSSAEAEFFTEYGDASRYKIQEI
TsMPK20-1(U3)     ------------------------------------------------------------------------------------------MQQDQRKKSSAEAEFFTEYGDASRYKIQEI
AetMPK20-1        ------------------------------------------------------------------------------------------MQQDQRKKSSAEAEFFTEYGDASRYKIQEI
TaMPK20-1(3AL)    ------------------------------------------------------------------------------------------MQQDQRKKSSAEAEFFTEYGDASRYKIQEI
AKS50324.1        ------------------------------------------------------------------------------------------MQQDQRKKSSAEAEFFTEYGDASRYKIQEI
TuMPK20-1         ------------------------------------------------------------------------------------------MQQDQRKKSSAEAEFFTEYGDASRYKIQEI
TaMPK20-1(3DL)    ------------------------------------------------------------------------------------------MQQDQRKKSSAEAEFFTEYGDASRYKIQEI

                  IGxGxYGxV            IKKIxxxF     DAxRxLRE                             FxDIYxxxELM  DLxxVI                 QxLRxLKYxH     HR
AK252802.1p       ------------------------------------------------------------------------------------------------------------------------
TaMPK20-1(3B)     VGKGSYGVVCSAIDVHTGEKVAIKKIHDIFEHISDAARILREIKLLRLLRHPDIVEIKHIMLPPSRRDFKDIYVVFELMESDLHQVIKANDDLTKEHYQFFLYQLLRALKYIHTANVYHR
TsMPK20-1(U2)p*   VGKGSYGVVCSAIDVHTGEKVAIKKIHDIFEHISDAARILREIKLLRLLRHPDIVEIKHIMLPPSRRDFKDIYVVFELMESDLHQVIKANDDLTKEHYQFFLYQLLRALKYIHTANVYHR
TsMPK20-1(U1)     VGKGSYGVVCSAIDVHTGEKVAIKKIHDIFEHISDAARILREIKLLRLLRHPDIVEIKHIMLPPSRRDFKDIYVVFELMESDLHQVIKANDDLTKEHYQFFLYQLLRALKYIHTANVYHR
ScMPK20-1         VGKGSYGVVCSAIDVHTGEKVAIKKIHDIFEHISDAARILREIKLLRLLRHPDIVEIKHIMLPPSRRDFKDIYVVFELMESDLHQVIKANDDLTKEHYQFFLYQLLRALKYIHTANVYHR
TsMPK20-1(U3)     VGKGSYGVVCSAIDVHTGEKVAIKKIHDIFEHISDAARILREIKLLRLLRHPDIVEIKHIMLPPSRRDFKDIYVVFELMESDLHQVIKANDDLTKEHYQFFLYQLLRALKYIHTANVYHR
AetMPK20-1        VGKGSYGVVCSAIDVHTGEKVAIKKIHDIFEHISDAARILREIKLLRLLRHPDIVEIKHIMLPPSRRDFKDIYVVFELMESDLHQVIKANDDLTKEHYQFFLYQLLRALKYIHTANVYHR
TaMPK20-1(3AL)    VGKGSYGVVCSAIDVHTGEKVAIKKIHDIFEHISDAARILREIKLLRLLRHPDIVEIKHIMLPPSRRDFKDIYVVFELMESDLHQVIKANDDLTKEHYQFFLYQLLRALKYIHTANVYHR
AKS50324.1        VGKGSYGVVCSAIDVHTGEKVAIKKIHDIFEHISDAARILREIKLLRLLRHPDIVEIKHIMLPPSRRDFKDIYVVFELMESDLHQVIKANDDLTKEHYQFFLYQLLRALKYIHTANVYHR
TuMPK20-1         VGKGSYGVVCSAIDVHTGEKVAIKKIHDIFEHISDAARILREIKLLRLLRHPDIVEIKHIMLPPSRRDFKDIYVVFELMESDLHQVIKANDDLTKEHYQFFLYQLLRALKYIHTANVYHR
TaMPK20-1(3DL)    VGKGSYGVVCSAIDVHTGEKVAIKKIHDIFEHISDAARILREIKLLRLLRHPDIVEIKHIMLPPSRRDFKDIYVVFELMESDLHQVIKANDDLTKEHYQFFLYQLLRALKYIHTANVYHR

                  DLKPxN LxNxNCxLKIxDFGLAR                TRWYRAPEL          IDxWS(V/I)GC
AK252802.1p       ------------------------------------------------------------------------------------------------------------------------
TaMPK20-1(3B)     DLKPKNILANSNCKLKICDFGLARVAFNDTPTTIFWTDYVATRWYRAPELCGSFFSKYTPAIDVWSIGCIFAEVLTGKPLFPGKNVVHQLDLMTDLLGTPSMDTISRVRNEKARRYLSSM
TsMPK20-1(U2)p*   DLKPKNILANSNCKLKICDFGLARVAFNDTPTTIFWTDYVATRWYRAPELCGSFFSKYTPAIDVWSIGCIFAEVLTGKPLFPGKNVVHQLDLMTDLLGTPSMDTISRVRNEKARRYLSSM
TsMPK20-1(U1)     DLKPKNILANSNCKLKICDFGLARVAFNDTPTTIFWTDYVATRWYRAPELCGSFFSKYTPAIDVWSIGCIFAEVLTGKPLFPGKNVVHQLDLMTDLLGTPSMDTISRVRNEKARRYLSSM
ScMPK20-1         DLKPKNILANSNCKLKICDFGLARVAFNDTPTTIFWTDYVATRWYRAPELCGSFFSKYTPAIDVWSIGCIFAEVLTGKPLFPGKNVVHQLDLMTDLLGTPSMDTISRVRNEKARRYLSSM
TsMPK20-1(U3)     DLKPKNILANSNCKLKICDFGLARVAFNDTPTTIFWTDYVATRWYRAPELCGSFFSKYTPAIDVWSIGCIFAEVLTGKPLFPGKNVVHQLDLMTDLLGTPSMDTISRVRNEKARRYLSSM
AetMPK20-1        DLKPKNILANSNCKLKICDFGLARVAFNDTPTTIFWTDYVATRWYRAPELCGSFFSKYTPAIDVWSIGCIFAEVLTGKPLFPGKNVVHQLDLMTDLLGTPSMDTISRVRNEKARRYLSSM
TaMPK20-1(3AL)    DLKPKNILANSNCKLKICDFGLARVAFNDTPTTIFWTDYVATRWYRAPELCGSFFSKYTPAIDVWSIGCIFAEVLTGKPLFPGKNVVHQLDLMTDLLGTPSMDTISRVRNEKARRYLSSM
AKS50324.1        DLKPKNILANSNCKLKICDFGLARVAFNDTPTTIFWTDYVATRWYRAPELCGSFFSKYTPAIDVWSIGCIFAEVLTGKPLFPGKNVVHQLDLMTDLLGTPSMDTISRVRNEKARRYLSSM
TuMPK20-1         DLKPKNILANSNCKLKICDFGLARVAFNDTPTTIFWTDYVATRWYRAPELCGSFFSKYTPAIDVWSIGCIFAEVLTGKPLFPGKNVVHQLDLMTDLLGTPSMDTISRVRNEKARRYLSSM
TaMPK20-1(3DL)    DLKPKNILANSNCKLKICDFGLARVAFNDTPTTIFWTDYVATRWYRAPELCGSFFSKYTPAIDVWSIGCIFAEVLTGKPLFPGKNVVHQLDLMTDLLGTPSMDTISRVRNEKARRYLSSM
```

MPK20-1 continued

```
                          LERKLLAFDPKDRPTAEEAL
AK252802.1p         ------------------------------------------------------------------------------------------------------------------------
TaMPK20-1(3B)       RKKELISFSHKFPNADPLALDLLQRLLAFDPKDRPTAEEALSHPYFKGLAKVEREPSCQPITKMEFEFERRRVTKEDIRELIXXXXXXXXXXXXX-XXXXXXXXXXXXXSAVDQFRKQFA
TsMPK20-1(U2)p*     RKKELISFSHKFPNADPLALDLLQRLLAFDPKDRPTAEEALSHPYFKGLAKVEREPSCQPITKMEFEFERRRVTKEDIRELIFREILEYHPQLLKDYTNGTERTTFLYPSAVDQFRKQFA
TsMPK20-1(U1)       RKKEPISFSHKFLNADPLALDLLQRLLAFDPKDRPTAEEALSHPYFKGLAKVEREPSCQPITKMEFEFERRRVTKEDIRELIFREILEYHPQLLKDYTNGTERTTFLYPSAVDQFRKQFA
ScMPK20-1           RKKEPISFSHKFLNADPLALDLLQRLLAFDPKDRPTAEEALSHPYFKGLAKVEREPSCQPITKMEFEFERRRVTKEDIRELIFREILEYHPQLLKDYTNGTERTTFLYPSAVDQFRKQFA
TsMPK20-1(U3)       RKKEPISFSHKFLNADPLALDLLQRLLAFDPKDRPTAEEALSHPYFKGLAKVEREPSCQPITKMEFEFERRRVTKEDIRELIFREILEYHPQLLKDYTNGTERTTFLYPSAVDQFRKQFA
AetMPK20-1          RKKELISFSHKFPNADPLALDLLQRLLAFDPKDRPTAEEALSHPYFKGLAKVEREPSCQPITKMEFEFERRRVTKEDIRELIFREILEYHPQLLKDYTNGTERTTFLYPSAVDQFRKQFA
TaMPK20-1(3AL)      RKKELISFSHKFPNADPLALDLLQRLLAFDPKDRPTAEEALSHPYFKGLAKVEREPSCQPITKMEFEFERRRVTKEDIRELIFREILEYHPQLLKDYTNGTERTTFLYPSAVDQFRKQFA
AKS50324.1          RKKELISFSHKFPNADPLALDLLQRLLAFDPKDRPTAEEALSHPYFKGLAKVEREPSCQPITKMEFEFERRRVTKEDIRELIFREILEYHPQLLKDYTNGTERTTFLYPSAVDQFRKQFA
TuMPK20-1           RKKELISFSHKFPNADPLALDLLQRLLAFDPKDRPTAEEALSHPYFKGLAKVEREPSCQPITKMEFEFERRRVTKEDIRELIFREILEYHPQLLKDYTNGTERTTFLYPSAVDQFRKQFA
TaMPK20-1(3DL)      RKKELISFSHKFPNADPLALDLLQRLLAFDPKDRPTAEEALSHPYFKGLAKVEREPSCQPITKMEFEFERRRVTKEDIRELIFREILEYHPQLLKDYTNGTERTTFLYPSAVDQFRKQFA

AK252802.1p         HLEENTENGPVVPMDRKHTSLPRSTIVHSAPIHAKEQPRIGPSRDKPSPDESYKNPRDSEKYSGNVQRTSLAPQRVPTARPGRVVGPVLPYENGGMKDTYDPRRVAAAM---NSGYAPQQ
TaMPK20-1(3B)       HLEENSENGPVVPMDRKHTSLPRSTIVHSAPIHGKEQPRIGPSRDKPSPDESYKNHRDSEKYSGNVQRTSQAPQRVPTARPGRVVGPVLPYENGGMKDTYDPRRVAAMNSGYP----PQQ
TsMPK20-1(U2)p*     HLEENSENGPVVPMDRKHTSLPRSTIVHSAPIHGKEQPRIGPSRDKPSPDESYKNHRDSEKYSGNVQRTSQAPQRVPTARPGRVVGPVLPYENGGMKDTYDPRRVDKTNRKLPVNTYPKQ
TsMPK20-1(U1)       HLEENSENGPVVPMDRKHTSLPRSTIVHSAPIHGKEQPRIGPSRDKPSPDESYKNHRDSEKYSGNAQRTSQAPQRVPTARPGRVVGPVLPYENGGMKDTYDPRRVAAMSSG----YPPQQ
ScMPK20-1           HLEENSENGPVVPMDRKHTSLPRSTIVHSAPIHGKEQPRIGPSRDKPSPDESYKNHRDSEKYSGNAQRTSQAPQRVPTARPGRVVGPVLPYENGGMKDTYDPRRVAAMSSG----YPPQQ
TsMPK20-1(U3)       HLEENSENGPVVPMDRKHTSLPRSTIVHSAPIHGKEQPRIGPSRDKPSPDESYKNHRDSEKYSGNAQRTSQAPQRVPTARPGRVVGPVLPYENGGMKDTYDPRRVAAMSSG----YPPQQ
AetMPK20-1          HLEENSENGPVVPMDRKHTSLPRSTIVHSAPIHGKEQPRIGPSRDKPSPDESYKNHRDSEKYSGNVQRTSQAPQRVPTARPGRVVGPVLPYENVGMKDTYDPRRVAAMSSG----YPPQQ
TaMPK20-1(3AL)      HLEENSENGPVVPMDRKHTSLPRSTIVHSAPIHGKEQPRIGPSRDKPSPDESYKNHRDSEKYSGNVQRTSQAPQRVPTARPGRVVGPVLPYENGGMKDTYDPRRVAAMSSG----YPPQQ
AKS50324.1          HLEENSENGPVVPMDRKHTSLPRSTIVHSAPIHGKEQPRIGPSRDKPSPDESYKNHRDSEKYSGNVQRTSQAPQRVPTARPGRVVGPVLPYENGGMKDTYDPRRVAAMSSG----YPPQQ
TuMPK20-1           HLEENSENGPVVPMDRKHTSLPRSTIVHSAPIHGKEQPRIGPSRDKPSPDESYKNHRDSEKYSGNVQRTSQAPQRVPTARPGRVVGPVLPYENGGMKDTYDPRRVAAMSSG----YPPQQ
TaMPK20-1(3DL)      HLEENSENGPVVPMDRKHTSLPRSTIVHSAPIHGKEQPRIGPSRDKPSPDESYKNHRDSEKYSGNVQRTSQAPQRVPTARPGRVVGPVLPYENVGMKDTYDPRRVAAMSSG----YPPQQ
                    *****:***************************.********************* *********.**** ********************** ***********            *:*

AK252802.1p         QIPQIYGYYQTP-KSACSEPSQAEMYTLHQQAYACANSSTVPDVALDMRAPPFHQSAVPKRGSSDRLSAESNLYSRSLNGMAPTTSGVAASGHRKVGVVPYGVSQMY*
TaMPK20-1(3B)       QIPQIYGYYQTPAKSACSEPSQAEMYTLHQQAYACANSSTVPDVALDMRAPPFHQSAGPKRGSSDRLSAESNLYSRSLNGIAPTTTGVAASAHRKVGVVPYGMSQMY*
TsMPK20-1(U2)p*     QIPQIYGYYQTPAKSACSEPSQAEMYTLHQQAYACANSSTVPDVALDMRAPPFHQSAGPKRGSSDRLSAESNLYSRSLNGMAPTTTGVAASAHRKVGVVPYGMSQMY*
TsMPK20-1(U1)       QIPQIYGYYQTPAKSACSEPSQAEMYTLHQQAYACANSSTVPDVGHESTSLPSVSRAE------------KGFF----------------------------------
ScMPK20-1           QIPQIYGYYQTPAKSACSEPSQAEMYTLHQQAYACANSSTVPDVALDMRAPPFHQSAGPKRGSSDRLSAESNLYTRSLNGMAPTTTGVAASGHRKVGVVPYGMSQMY*
TsMPK20-1(U3)       QIPQIYGYYQTPAKSACSEPSQAEMYTLHQQAYACANSSTVPDVALDMRAPPFHQSAGPKRGSSDRLSAESNLYTRSLNGMAPTTTGVAASGHRKVGVVPYGMSQMY*
AetMPK20-1          QIPQIYGYYQTPAKSACSEPSQAEMYTLHQQVYACANSSTVPDVALDMRAPPFHQSAGPKRGSSDRLSAESNLYSRSLNGMAPTTTGVAASAHRKVGVVPYGMSQMY*
TaMPK20-1(3AL)      QIPQIYGYYQTPAKSACSEPSQAEMYTLHQQAYACANSSTVPDVALDMRAPPFHQSAGPKRGSSDRLSAESNLYSRSLNGMAPTTTGVAASAHRKVGVVPYGMSQMY*
AKS50324.1          QIPQIYGYYQTPAKSACSEPSQAEMYTLHQQAYACANSSTVPDVALDMRAPPFHQSAGPKRGSSDRLSAESNLYSRSLNGMAPTTTGVAASAHRKVGVVPYGMSQMY*
TuMPK20-1           QIPQIYGYYQTPAKSACSEPSQAEMYTLHQQAYACANSSTVPDVALDMRAPPFHQSAGPKRGSSDRLSAESNLYSRSLNGMAPTTTGVAASAHRKVGVVPYGMSQMY*
TaMPK20-1(3DL)      QIPQIYGYYQTPAKSACSEPSQAEMYTLHQQAYACANSSTVPDVALDMRAPPFHQSAGPKRGSSDRLSAESNLYSRSLNGMAPTTTGVAASAHRKVGVVPYGMSQMY*
                    ************ ******************.************. :  : *  . *              ..::
```

MPK20-2

```
                                                  IGxGxYGxV             IKKIxxxF    DAxRxLRE                          FxDIYxxxELM  DLxxVI
BAJ92365.1          ---MQRSKSSAEVDFFTEYGDANRYKIQEVIGKGSYGVVCSALDLQTRQKVAIKKIHNIFEHTSDAARILREIKLLRLLRHPDVVEIKHIMLPPSRKDFKDIYVVFELMESDLHQVIKAN
AK361419.1          ---MQRSKSSAEVDFFTEYGDANRYKIQEVIGKGSYGVVCSALDLQTRQKVAIKKIHNIFEHTSDAARILREIKLLRLLRHPDVVEIKHIMLPPSRKDFKDIYVVFELMESDLHQVIKAN
HvMPK20-2(1H)       ---MQRSKSSAEVDFFTEYGDANRYKIQEVIGKGSYGVVCSALDLQTRQKVAIKKIHNIFEHTSDAARILREIKLLRLLRHPDVVEIKHIMLPPSRKDFKDIYVVFELMESDLHQVIKAN
ScMPK20-2           MQQAQRSKTSAEVDFFTEYGDANRYKIQEVIGKGSYGVVCSALDLQTRQKVAIKKIHNIFEHTSDAARILREIKLLRLLRHPDVVEIKHIMLPPSRKDFKDIYVVFELMESDLHQVIKAN
TsMPK20-2(U1)       MQQAQRTKSSAEVDF-SEYGDANRYKIQE-VGKGSYGVVCSALDLQTRQKVAIKKIHNIFEHTSDAARILREIKLLRLLRHPDVVEIKHIMLPPSRKDFKDIYVVFELMESDLHQVIKAN
TsMPK20-2(U2)       MQQDQRKKSSAEAEFFTEYGDASRYKIQEIVGKGSYGVVCSAIDVHTGEKVAIKKIHDIFEHISDAARILREIKLLRLLRHPDIVEIKHIMLPPSRRDFKDIYVVFELMESDLHQVIKAN
AetMPK20-2p         --------SSAEVDFFTEYGDANRYKIQEVIGKGSYGVVCSALDLQTRQKVAIKKIHNIFEHTSDAARILREIKLLRLLRHPDVVEIKHIMLPPSRKDFKDIYVVFELMESDLHQVIKAN
TaMPK20-2(1DL)      MQQAQRSKSSAEVDFFTEYGDANRYKIQEVIGKGSYGVVCSALDLQTRQKVAIKKIHNIFEHTSDAARILREIKLLRLLRHPDVVEIKHIMLPPSRKDFKDIYVVFELMESDLHQVIKAN
tplb0059e09         MQQAQRSKSSAEVDFFTEYGDANRYKIQEVIGKGSYGVVCSALDLQTRQKVAIKKIHNIFEHTSDAARILREIKLLRLLRHPDVVEIKHIMLPPSRKDFKDIYVVFELMESDLHQVLKAN
TaMPK20-2(1BL)      MQQAQRSKGSAEADFFTEYGDANRYKIQEVIGKGSYGVVCSALDLQTRQKVAIKKIHNIFEHTSDAARILREIKLLRLLRHPDVVEIKHIMLPPSRKDFKDIYVVFELMESDLHQVIKAN
TuMPK20-2p          MQQAQRTKSSAEVDFFTEYGDANRYKIQEVIGKGSYGVVCSALDLQTRQKVAIKKIHNIFEHTSDAARILREIKLLRLLRHPDVVEIKHIMLPPSRKDFKDIYVVFELMESDLHQVIKAN
TaMPK20-2(1AL)      MQQAQRTKSSAEVDFFTEYGDANRYKIQEVIGKGSYGVVCSALDLQTRQKVAIKKIHNIFEHTSDAARILREIKLLRLLRHPDVVEIKHIMLPPSRKDFKDIYVVFELMESDLHQVIKAN
AK331818.1          MQQAQRTKSSAEVDFFTEYGDANRYKIQEVIGKGSYGVVCSALDLQTRQKVAIKKIHNIFEHTSDAARILREIKLLRLLRHPDVVEIKHIMLPPSRKDFKDIYVVFELMESDLQQVIKAN
                             ***.:* :*****.****** :***********:*::* :********:**** ********************:************:****************:**:***

                                 QxLRxLKYxH     HRDLKPxN LxNxNCxLKIxDFGLAR                 TRWYRAPEL           IDxWSVIGC
BAJ92365.1          DDLTKEHYQFFLYQLLRALKYIHTASVYHRDLKPKNILANSNCKLKICDFGLARVAFNDTPTTVFWTDYVATRWYRAPELCGSFFTKYTPAIDIWSIGCIFAEVLTGKPLFPGKNVVHQL
AK361419.1          DDLTKEHYQFFLYQLLRALKYIHTASVYHRDLKPKNILANSNCKLKICDFGLARVAFNDTPTTVFWTDYVATRWYRAPELCGSFFTKYTPAIDIWSIGCIFAEVLTGKPLFPGKNVVHQL
HvMPK20-2(1H)       DDLTKEHYQFFLYQLLRALKYIHTASVYHRDLKPKNILANSNCKLKICDFGLARVAFNDTPTTVFWTDYVATRWYRAPELCGSFFTKYTPAIDIWSIGCIFAEVLTGKPLFPGKNVVHQL
ScMPK20-2           DDLTKEHYQFFLYQLLRALKYIHTASVYHRDLKPKNILANSNCKLKICDFGLARVAFNDTPTTVFWTDYVATRWYRAPELCGSFFTKYTPAIDIWSIGCIFAEVLTGKPLFPGKNVVHQL
TsMPK20-2(U1)       DDLTKEHYQFFLYQLLRALKYIHTASVYHRDLKPKNILANSNCKLKICDFGLARVAFNDTPTTVFWTDYVATRWYRAPELCGSFFTKYTPAIDIWSIGCIFAEVLTGKPLFPGKNVVHQL
TsMPK20-2(U2)       DDLTKEHYQFFLYQLLRALKYIHTANVYHRDLKPKNILANSNCKLKICDFGLARVAFNDTPTTIFWTDYVATRWYRAPELCGSFFSKYTPAIDVWSIGCIFAEVLTGKPLFPGKNVVHQL
AetMPK20-2p         DDLTKEHYQFFLYQLLRALKYIHTASVYHRDLKPKNILANSNCKLKICDFGLARVAFNDTPTTVFWTDYVATRWYRAPELCGSFFTKYTPAIDIWSIGCIFAEVLTGKPLFPGKNVVHQL
TaMPK20-2(1DL)      DDLTKEHYQFFLYQLLRALKYIHTASVYHRDLKPKNILANSNCKLKICDFGLARVAFNDTPTTVFWTDYVATRWYRAPELCGSFFTKYTPAIDIWSIGCIFAEVLTGKPLFPGKNVVHQL
tplb0059e09         DDLTKEHYQFFLYQLLRALKYIHTASVYHRDLKPKNILANSNCKLKICDFGLARVAFNDTPTTVFWTDYVATRWYRAPELCGSFFTKYTPAIDIWSIGCIFAEVLTGKPLFPGKNVVHQL
TaMPK20-2(1BL)      DDLTKEHYQFFLYQLLRALKYIHTASVYHRDLKPKNILANSNCKLKICDFGLARVAFNDTPTTVFWTDYVATRWYRAPELCGSFFTKYTPAIDIWSIGCIFAEVLTGKPLFPGKNVVHQL
TuMPK20-2p          DDLTKEHYQFFLYQLLRALKYIHTASVYHRDLKPKNILANSNCKLKICDFGLARVAFNDTPTTVFWTDYVATRWYRAPELCGSFFTKYTPAIDIWSIGCIFAEVLTGKPLFPGKNVVHQL
TaMPK20-2(1AL)      DDLTKEHYQFFLYQLLRALKYIHTASVYHRDLKPKNILANSNCKLKICDFGLARVAFNDTPTTVFWTDYVATRWYRAPELCGSFFTKYTPAIDIWSIGCIFAEVLTGKPLFPGKNVVHQL
AK331818.1          DDLTKEHYQFFLYQLLRALKYIHTASVYHRDLKPKNILANSNCKLKICDFGLARVAFNDTPTTVFWTDYVATRWYRAPELCGSFFTKYTPAIDIWSIGCIFAEVLTGKPLFPGKNVVHQL
                    *************************.*************************************:*********************:*******:**************************
```

MPK20-2 continued

LE$^{R}_{K}$LLAFDPKDRPTAEEAL

```
BAJ92365.1          DLMTDLLGTPSMDTISRVRNEKARRYLTSMRKKDPVPFSQKFPNADPLGVKLLEKLLAFDPKDRPTAEEALTDPYFRGLSKPEREPSCQPIRKMEFDFEHSRVSKDDIRELIFQEILEYH
AK361419.1          DLMTDLLGTPSMDTISRVRNEKARRYLTSMRKKDPVPFSQKFPNADPLGVKLLEKLLAFDPKDRPTAEEALTDPYFRGLSKPEREPSCQPIRKMEFDFEHSRVSKDDIRELIFQEILEYH
HvMPK20-2(1H)       DLMTDLLGTPSMDTISRVRNEKARRYLTSMRKKDPVPFSQKFPNADPLGVKLLEKLLAFDPKDRPTAEEALTDPYFRGLSKPEREPSCQPIRKMEFDFEHSRVSKDDIRELIFQEILEYH
ScMPK20-2           DLMTDLLGTPSMDTISRVRNEKARRYLSSMRKKDPVPFSQKFPNADPLGVKLLEKLLAFDPKDRPTAEEALTDPYFRGLSKPEREPSCQPIRKLEFDFEHSRVSKDDIRELIFQEILEYH
TsMPK20-2(U1)       DLMTDLLGTPSMDTISRVRNEKARRYLSSMRKKDPVPFSQKFPNADPLGVKLLEKLLAFDPKDRPTAEEALTDPYFRGLSKPEREPSCQPIRKMEFDFEHSRVSKDDIRELIFQEILEYH
TsMPK20-2(U2)       DLMTDLLGTPSMDTISRVRNEKARRYLSSMRKKDPVPFSQKFPNADPLGVKLLEKLLAFDPKDRPTAEEALTDPYFRGLSKPEREPSCQPIRKMEFDFEHSRVSKDDIRELIFQEILEYH
AetMPK20-2p         DLMTDLLGTPSMDTISRVRNEKARRYLSSMRKKDPVPFSQKFPNADPLGVKLLEKLLAFDPKDRPTAEEALTDPYFRGLSKPEREPSCQPIRKMEFDFEHSRVSKDDIRELIFQEILEYH
TaMPK20-2(1DL)      DLMTDLLGTPSMDTISRVRNEKARRYLSSMRKKDPVPFSQKFPNADPLGVKLLEKLLAFDPKDRPTAEEALTDPYFRGLSKPEREPSCQPIRKMEFDFEHSRVSKDDIRELIFQEILEYH
tplb0059e09         DLMTDLLGTPSMDTISRVRNEKARRYLSSMRKKDPVPFSQKFPNADPLGVKLLEKLLAFDPKDRPTAEEALTDPYFRGLSKPEREPSCQPIRKMEFDFEHSRVSKDDIRELIFQEILEYH
TaMPK20-2(1BL)      DLMTDLLGTPSMDTISRVRNEKARRYLSSMRKKDPVPFSQKFPNADPLGVKLLEKLLAFDPKDRPTAEEALTDPYFRGLSKPEREPSCQPIRKMEFDFEHSRVSKDDIRELIFQEILEYH
TuMPK20-2p          DLMTDLLGTPSMDTISRVRNEKARRYLSSMRKKDPVPFSQKFPNADPLGVKLLEKLLAFDPKDRPTAEEALTDPYFRGLSKPEREPSCQPIRKMEFDFEHSRVSKDDIRELIFQEILEYH
TaMPK20-2(1AL)      DLMTDLLGTPSMDTISRVRNEKARRYLSSMRKKDPVPFSQKFPNADPLGVKLLEKLLAFDPKDRPTAEEALTDPYFRGLSKPEREPSCQPIRKMEFDFEHSRVSKDDIRELIFQEILEYH
AK331818.1          DLMTDLLGTPSMDTISRVRNEKARRYLSSMRKKDPVPFSQKFPNADPLGVKLLEKLLAFDPKDRPTAEEALTDPYFRGLSKPEREPSCQPIRKMEFDFEHSRVSKDDIRELIFQEILEYH
                    ***************************:*****************************************************************:**************************

BAJ92365.1          PQLLKSYIDGTERTTFLYPSAVDHFKKQFSHLEESDGSGPVVPADRKHASLPRSTIVHSTPIPAKDTRPLFGKPCSKTSSETGRYAGNGHGASQASHAAQAVVSRRAAGSALPYEGGSGK
AK361419.1          PQLLKSYIDGTERTTFLYPSAVDHFKKQFSHLEESDGSGPVVPADRKHASLPRSTIVHSTPIPAKDTRPLFGKPCSKTSSETGRYAGNGHGASQASHAAQAVVSRRAAGSALPYEGGSGK
HvMPK20-2(1H)       PQLLKSYIDGTERTTFLYPSAVDHFKKQFSHLEESDGSGPVVPADRKHASLPRSTIVHSTPIPAKDTRPLFGKPCSKTSSETGRYAGNGHGASQASHAAQAVVSRRAAGSALPYEGGSGK
ScMPK20-2           PQLLKSYVDGTERTTFLYPSAVDHFKKQFSHLEESDGSGPVVPADRKHASLPRSTIVHSTPIPAKDTRPLFGKPCSKTSSETGRYAGNGHGASQASHAAQAVVSRRAAGSALPSEGGVGR
TsMPK20-2(U1)       PQLLKSYTDGTERTTFLYPSAVDHFKKQFSHLEESDGSGPVVPADRKHASLPRSTIVHSTPIPAKDTRPLFGKPCSKTSSETGRYAGNGHGASQASHAAQAVVSRRAAGSALPSEAGVGR
TsMPK20-2(U2)       PQLLKSYVDGTERTTFLYPSAVDHFKKQFSHLEESDGSGPVVPADRKHASLPRSTIVHSAPISAKDTRPLFGKPCSKTSSETGRYAGNGRGASQASHAAQAVVSRRAAGSAL--------
AetMPK20-2p         PQLLKSYIDGTERTTFLYPSAVDHFKKQFSHLEESDGSGSVVPADRKHASLPRSTIVHSTPIPAKDTRPLFGKPCNKTSSETGRYAGNGHGASQASHAAQAVVSRRAAGSAL--------
TaMPK20-2(1DL)      PQLLKSYIDGTERTTFLYPSAVDHFKKQFSHLEESDGSGPVVPADRKHASLPRSTIVHSTPIPAKDTRPLFGKPCNKTSSETGRYAGNGHSASQASHAAQAVVSRRAAGSAL--------
tplb0059e09         PQLLKSYIDGTERTTFLYPSAVDHFKKQFSHLEESDGSGPVVPADRKHASLPRSTIVHSTPIPAKDTRPLFGKPCNKTSSETGRYAGNGHSASQASHAAQAVVSRRAAGSAL--------
TaMPK20-2(1BL)      PQLLKSYTDGTERTTFLYPSAVDHFKKQFSHLEESDGSGPVVPADRKHASLPRSTIVHSTPIPAKDTRPLFGKPCNKTSSETGRYAGNGHGASQASHAAQAVISRRAAGSAL--------
TuMPK20-2p          PQLLKSYVDGTERTTFLYPSAVDHFKKQFSHLEESDGSGPVVPADRKHASLPRSTIVHSAPISAKDTRPLFGKPCSKTSSETGRYAGNGHGASQASHAAQAVVSRRAAGSAL--------
TaMPK20-2(1AL)      PQLLKSYVDGTERTTFLYPSAVDHFKKQFSHLEESDGSGPVVPADRKHASLPRSTIVHSAPISAKDTRPLFGKPCSKTSSETGRYAGNGRGASQASHAAQAVVSRRAAGSAL--------
AK331818.1          PQLLKCYVDGTERTTFLYPSAVDHFKKQFSHLEESDGSGPVVPADRKHASLPRSTIVHSAPISAKDTRPLFGKPCSKTSSETGRYAGNGRGASQASHAAQAVVSRRAAGSAL--------
                    *****.* ******************************* ******************:** ************.*************:.***********:*********
```

MPK20-2 continued

```
BAJ92365.1          HPYDVASRPAMSTGCPPQQQIPQMYGQYQHQAPAGAGAGAGAGIPQAMGGYACGGYAKGTAP-----APAMRAPTYRHVPAGQKNGRLDRLAVETTDIYTRSLNGIVAAAAASAGTASAHRKVGTVPFGMPTTY*
AK361419.1          HPYDVASRPAMSTGCPPQQQIPQMYGQYQHQAPAGAGAGAGAGIPQAMGGYACGGYAKGTAP-----APAMRAPTYRHVPAGQKNGRLDRLAVETTDIYTRSLNGIVAAAAASAGTASAHRKVGTVPFGMPTTY*
HvMPK20-2(1H)       HPYDVASRPAMSTGCPPQQQIPQMYGQYQHQAPAGAGAGAGAGIPQAMGGYACGGYAKGTAP-----APAMRAPTYRHVPAGQKNGRLDRLAVETTDIYTRSLNGIVAAAAASAGTASAHRKVGTVPFGMPTTY*
ScMPK20-2           YPYDAASRPAMSSGCPPQQQIPQLYGQYQHQAAPGA----GAGIPQTMGGYSCGGYTKGTAPNAAAAAPAMRAPPYRHLPVGQKNGPLDRLAVETTDIYTRSLNGIVAAAAASAGTAGAHRKVGAVPFGMPTTY*
TsMPK20-2(U1)       YPYDAASRPAMSSGCPPQQQIPQLYGQYQHQAAPGA----GAGIPQTMGGYACGGYTKGTAPNAAAAAPAMRAPPYRHLPAGQKNGPLDRLAVETTDIYTRSLNGIVAAAAASAGTASAHRKVGAVPFGMPTTY*
TsMPK20-2(U2)       -PYDAASRPTISPGCPPQQQIPQMYGQYQHQAPA------GAGIPQAMGGYACGGYTKGTAPNAAAAAPAMRAPPYRHLPAGQKNGPLDRLAVETTDIYTRSLNGIVAAAAASAGTASAHRKVGAVPFGMPTTY*
AetMPK20-2p         -PYDAASRPAMSPGCP-QQQIPQMYGQYQHQAAPGA----GAGIPQAMGGYACGAYTKGTAPNAAAAAPAMRAPPYRHLPAGQKNGPLDRLAVETTDIYTRSLNGIVAAAAASAGATSAHRKVGAVPFGMPTTY*
TaMPK20-2(1DL)      -PYDAASRPTISPGCP-QQQIPQMYGQYQHQAAPGA----GAGIPQTMGGYACGGYTKGTAPNAA-AAPAMRAPPYRHLPAGQKNGPLDRLAVETTDIYTRSLNGIVAAAAASAGATSAHRKVGAVPFGMPTTY*
tplb0059e09         -PYDAASRPTISPGCP-QQQIPQMYGQYQHQAAPGA----GAGIPQTMGGYACGGYTKGTAPNAA-AAPAMRAPPYRHLPAGQKNGPLDRLAVETTDIYTRSLNGIVAAAAASAGATSAHRKVGAVPFGMPTTY*
TaMPK20-2(1BL)      -PYDAASRPTISPGCPPQQQIPQMYGQYQHQAPPGA--GAGAGIPQAMGGYACGGYTKGTAPNAAAAAPAMRAPPYRHLPTGQKNGPLDRLAVETTDIYTRSLNGIVAAAAASAGATGAHRKVGAVPFGMPTTY*
TuMPK20-2p          -PYDAASRPTISPGCPPQQQIPQMYGQYQHQAPA------GAGIPQAMGGYACGGYTKGTAPDAA-RG---------------------------------------------------------------------
TaMPK20-2(1AL)      -PYDAASRPTISPGCPPQQQIPQMYGQYQHQAPA------GAGIPQAMGGYACGGYTKGTAPNAA-AAPAMRAPPYRHLPAGQKNGPLDRLAVETTDIYTRSLNGIVAAAAASAGTASAHRKVGAVPFGMPTTY*
AK331818.1          -PYDAASRPTISPGCPPQQQIPQMYGQYQHQAPA------GAGIPQAMGGYACGGYTKGTAPNAA-AAPAMRAPPYRHLPAGQKNGPLDRLAVETTDIYTRSLNGIVAAAAASAGTASAHRKVGAVPFGMPTTY*
                     ***.****::* *** ******:********        ******:****:**.*:*****     .
```

MPK20-3

IGxGxYGxV IKKIxxxF DAxRxLRE FxDIYxxxELM DLxxVI

```
Locus4929        MQNIDLRKKSAAEIDFFTEYGDTNRYKVLEVIGKGSYGLVCSANDTQTGEKVAIKKIHNIFEHISDAARILREIKLLRLLRHPDVVEIKHILLPPSKKDFKDIYVVFELMESDLHQVIKA
AetMPK20-3       MQNNDLRKKSAAEIDFFTEYGDTNRYKVLEVIGKGSYGLVCSANDTQTGEKVAIKKIHNIFEHISDAARILREIKLLRLLRHPDVVEIKHILLPPSKKDFKDIYVVFELMESDLHQVIKA
TaMPK20-3(7DL)   MQNNDLRKKSAAEIDFFTEYGDTNRYKVLEVIGKGSYGLVCSANDTQTGEKVAIKKIHNIFEHISDAARILREIKLLRLLRHPDVVEIKHILLPPSKKDFKDIYVVFELMESDLHQVIKA
AKL80627.1       MQNNDLRKKSAAEIDFFTEYGDTNRYKVLEVIGKGSYGLVCSANDTQTGEKVAIKKIHNIFEHISDAARILREIKLLRLLRHPDVVEIKHILLPPSKKDFKDIYVVFELMESDLHQVIKA
ScMPK20-3        MQNSDLRKKSAAEIDFFTEYGDTNRYKVLEVIGKGSYGLVCSANDTQTGEKVAIKKIHNIFEHISDAARILREIKLLRLLRHPDVVEIKHILLPPSKKDFKDIYVVFELMESDLHQVIKA
TaMPK20-3(7AL)   MQNGDLRKKSAAEIDFFTEYGDTNRYKVLEVIGKGSYGLVCSANDTQTGEKVAIKKIHNIFEHISDAARILREIKLLRLLRHPDVVEIKHILLPPSKKDFKDIYVVFELMESDLHQVIKA
TuMPK20-3        MQNGDLRKKSAAEIDFFTEYGDTNRYKVLEVIGKGSYGLVCSANDTQTGEKVAIKKIHNIFEHISDAARILREIKLLRLLRHPDVVEIKHILLPPSKKDFKDIYVVFELMESDLHQVIKA
TaMPK20-3(7BL)   MQNSDLRKKSAAEIDFFTEYGDTNRYKVLEVIGKGSYGLVCSANDTQTGEKVAIKKIHNIFEHISDAARILREIKLLRLLRHPDVVEIKHILLPPSKKDFKDIYVVFELMESDLHQVIKA
                 *** ********************************************************************************************************************
```

QxLRxLKYxH HRDLKPxN LxNxNCxLKIxDFGLAR TRWYRAPEL IDxWS$^{Y}_{I}$GC

```
Locus4929        NDDLTREHYQFFLYQMLRALKYMHTANVYHRDLKPKNVLANANCKLKICDFGLARVAFNDAPTTVFWTDYVATRWYRAPELCGSFYSKYTPAIDIWSIGCIFAEVLIGKPLFPGKNVVHQ
AetMPK20-3       NDDLTREHYQFFLYQMLRALKYMHTANVYHRDLKPKNVLANANCKLKICDFGLARVAFNDAPTTVFWTDYVATRWYRAPELCGSFYSKYTPAIDIWSIGCIFAEVLIGKPLFPGKNVVHQ
TaMPK20-3(7DL)   NDDLTREHYQFFLYQMLRALKYMHTANVYHRDLKPKNVLANANCKLKICDFGLARVAFNDAPTTVFWTDYVATRWYRAPELCGSFYSKYTPAIDIWSIGCIFAEVLIGKPLFPGKNVVHQ
AKL80627.1       NDDLTREHYQFFLYQMLRALKYMHTANVYHRDLKPKNVLANANCKLKICDFGLARVAFNDAPTTVFWTDYVATRWYRAPELCGSFYSKYTPAIDIWSIGCIFAEVLIGKPLFPGKNVVHQ
ScMPK20-3        NDDLTREHYQFFLYQMLRALKYMHTANVYHRDLKPKNVLANANCKLKICDFGLARVAFNDAPTTVFWTDYVATRWYRAPELCGSFYSKYTPAIDIWSIGCIFAEVLIGKPLFPGKNVVHQ
TaMPK20-3(7AL)   NDDLTREHYQFFLYQMLRALKYMHTANVYHRDLKPKNVLANANCKLKICDFGLARVAFNDAPTTVFWTDYVATRWYRAPELCGSFYSKYTPAIDIWSIGCIFAEVLIGKPLFPGKNVVHQ
TuMPK20-3        NDDLTREHYQFFLYQMLRALKYMHTANVYHRDLKPKNVLANANCKLKICDFGLARVAFNDAPTTVFWTDYVATRWYRAPELCGSFYSKYTPAIDIWSIGCIFAEVLIGKPLFPGKNVVHQ
TaMPK20-3(7BL)   NDDLTREHYQFFLYQMLRALKYMHTANVYHRDLKPKNVLANANCKLKICDFGLARVAFNDAPTTVFWTDYVATRWYRAPELCGSFYSKYTPAIDIWSIGCIFAEVLIGKPLFPGKNVVHQ
                 ************************************************************************************************************************
```

LE$^{R}_{K}$LLAFDPKDRPTAEEAL

```
Locus4929        LDLITDVLGTPSLDAISQVRNDKARKYLTYMRKKQPASFSQKFPKADPLALRLLRRLLAFDPKDRPSAEEALADPYFNGLAKVEREPSCQPIPKIEFEFEGRRVTKEDIKELIFEEILEY
AetMPK20-3       LDLITDVLGTPSLDAISQVRNDKARKYLTCMRKKQPASFSQKFPKADPLALRLLRRLLAFDPKDRPSAEEALADPYFNGLAKVEREPSCQPIPKLEFEFEGRRVTKEDIKELIFEEILEY
TaMPK20-3(7DL)   LDLITDVLGTPSLDAISQVRNDKARKYLTCMRKKQPASFSQKFPKADPLALRLLRRLLAFDPKDRPSAEEALADPYFNGLAKVEREPSCQPIPKLEFEFEGRRVTKEDIKELIFEEILEY
AKL80627.1       LDLITDVLGTPSLDAISQVRNDKARKYLTCMRKKQPASFSQKFPKADPLALRLLRRLLAFDPKDRPSAEEALADPYFNGLAKVEREPSCQPIPKLEFEFEGRRVTKEDIKELIFEEILEY
ScMPK20-3        LDLITDVLGTPSLDAISQVRNDKARKYLTCMRKKHPASFSQKFPKADPLALRLLRRLLAFDPKDRPSAEEALADPYFNGLAKVEREPSCQPIPKIEFEFEGRRVTKEDIKELIFEEILEY
TaMPK20-3(7AL)   LDLITDVLGTPSLDAISQVRNDKARKYLTCMRKKQPASFSQKFPKADPLALRLLRRLLAFDPKDRPSAEEALADPYFNGLAKVEREPSCQPIPKIEFEFEGRRVTKEDIKELIFEEILEY
TuMPK20-3        LDLITDVLGTPSLDAISQVRNDKARKYLTCMRKKQPASFSQKFPKADPLALRLLRRLLAFDPKDRPSAEEALADPYFNGLAKVEREPSCQPIPKIEFEFEGRRVTKEDIKELIFEEILEY
TaMPK20-3(7BL)   LDLITDVLGTPSLDAISQVRNDKARKYLTCMRKKQPASFSQKFPKADPLALRLLRRLLAFDPKDRPSAEEALADPYFNGLAKVEREPSCQPIPKIEFEFEGRRVTKEDIKELIFEEILEY
                 ***************************** ****:***********************************************************:*************************
```

```
Locus4929        HPQLLKEHISGTDRPNFVHLSAVDQFKKRFAELEENGSENGSAVSSQRKHSSLPRQSFNPR-
AetMPK20-3       HPQLLKEHISGTDRRNFVHLSAVDQFKKRFAELEENGGENGSAVSTQRKHSSLPR*------
TaMPK20-3(7DL)   HPQLLKEHISGTDRRNFVHLSAVDQFKKRFAELEENGGENGSAVSTQRKHSSLPRQSFNPR*
AKL80627.1       HPQLLKEHISGTDRRNFVHLSAVDQFKKRFAELEENGGENGSAVSSQRKHSSLPRQSFNPR*
ScMPK20-3        HPQLLKEHISGTDRPNFVHLSAVDQFKKRFAELEENGGENGSAASSQRKHSSLPRQSFNPR*
TaMPK20-3(7AL)   HPQLLKEHISGTDRRNFVHLSAVDQFKKRFAELEENGGENGSAVSSQRKHSSLPRQSFNPH*
TuMPK20-3        HPQLLKEHISGTDRRNFVHLSAVDQFKKRFAELEENGGENGSAVSSQRKHSSLPRQSFNPH*
TaMPK20-3(7BL)   HPQLLKEHISGTDRRNFVHLSAVDQFKKRFAELEENGGENGSAVSSQRKHSSLPRQSFNPR*
                 ************** **********************.*****.*:*********
```

MPK20-4

```
                                                          IGxGxYGxV             IKKIxxxF    DAxRxLRE                          FxDIYxxxELM  DL
HvMPK20-4(3H)       MQPDQQQ--HQQRRKGSSDMDFFSEYGDANRYKIQEVIGKGSYGVVCSAIDQHNGDKVAIKKIHNIFEHLSDAARILREIKLLRLLRHPDIVEIRHIMLPPSRRDFKDIYVVFELMDTDL
BAJ97013.1          MQPDQQQ--HQQRRKGSSDMDFFSEYGDANRYKIQEVIGKGSYGVVCSAIDQHNGDKVAIKKIHNIFEHLSDAARILREIKLLRLLRHPDIVEIRHIMLPPSRRDFKDIYVVFELMDTDL
BAJ96852.1          MQPDQQQ--HQQRRKGSSDMDFFSEYGDANRYKIQEVIGKGSYGVVCSAIDQHNGDKVAIKKIHNIFEHLSDAARILREIKLLRLLRHPDIVEIRHIMLPPSRRDFKDIYVVFELMDTDL
ScMPK20-4           MQPDQQ--QQQQRRKGSPEMDFFSEYGDANRYKIQEVIGKGSYGVVCSAIDQHTGDKVAIKKIHNIFEHLSDAARILREIKLLRLLRHPDIVEIRHIMLPPSRRDFKDIYVVFELMDTDL
TsMPK20-4(U)        MQPDQQ--QQQQRRKGSPEMDFFSEYGDANRYKIQEVIGKGSYGVVCSAIDQHTGDKVAIKKIHNIFEHLSDAARILREIKLLRLLRHPDIVEIRHIMLPPSRRDFKDIYVVFELMDTDL
TuMPK20-4           ----MQPDQHQQRRKGSSEMDFFSEYGDANRYKIQEVIGKGSYGVVCSAIDQHTGDKVAIKKIHNIFEHLSDAARILREIKLLRLLRHPDIVEIRHIMLPPSRRDFKDIYVVFELMDTDL
TaMPK20-4(3AL)      ----MQPDQNQQRRKGSSEMDFFSEYGDANRYKIQEVIGKGSYGVVCSAIDQHTGDKVAIKKIHNIFEHLSDAARILREIKLLCLLRHPDIVEIRHIMLPPSRRDFKDIYVVFELMDTDL
AetMPK20-4          MQPD--QHQQQQRRKGSSEMDFFSEYGDANRYKIQEVIGKGSYGVVCSAIDQHTGDKVAIKKIHNIFEHLSDAARILREIKLLRLLRHPDIVEIRHIMLPPSRRDFKDIYVVFELMDTDL
TaMPK20-4(3DL)      MQPD--QHQQQQRRKGSSEMDFFSEYGDANRYKIQEVIGKGSYGVVCSAIDQHTGDKVAIKKIHNIFEHLSDAARILREIKLLRLLRHPDIVEIRHIMLPPSRRDFKDIYVVFELMDTDL
TaMPK20-4(3B)       MQPDQQQHQHQQRRKGSSEMDFFSEYGDANRYKIQEVIGKGSYGVVCSAIDQHTGDKVAIKKIHNIFEHLSDAARILREIKLLRLLRHPDIVEIRHIMLPPSRRDFKDIYVVFELMDTDL
AK333309.1p         MQPDQQQHQHQQRRKGSSEMDFFSEYGDANRYKIQEVIGKGSYGVVCSAIDQHTGDKVAIKKIHNIFEHLSDAARILREIKLLRLLRHPDIVEIRHIMLPPSRRDFKDIYVVFELMDTDL
                             :******** :**********************************.***************************** ************************************

                    xxVI                QxLRxLKYxH     HRDLKPxN LxNxNCxLKIxDFGLAR                 TRWYRAPEL           IDxWS^Y_IGC
HvMPK20-4(3H)       HQVIKANDDLTKEHHQFFLYQMLRALKYIHTANVYHRDLKPKNILANANCKLKICDFGLARVAFNDTPTTVFWTDYVATRWYRAPELCGSFFTKYSPAIDIWSIGCIFAEILTGKPLFPG
BAJ97013.1          HQVIKANDDLTKEHHQFFLYQMLRALKYIHTANVYHRDLKPKNILANANCKLKICDFGLARVAFNDTPTTVFWTDYVATRWYRAPELCGSFFTKYSPAIDIWSIGCIFAEILTGKPLFPG
BAJ96852.1          HQVIKANDDLTKEHHQFFLYQMLRALKYIHTANVYHRDLKPKNILANANCKLKICDFGLARVAFNDTPTTVFWTDYVATRWYRAPELCGSFFTKYSPAIDIWSIGCIFAEILTGKPLFPG
ScMPK20-4           HQVIKANDDLTKEHHQFFLYQMLRALKYIHTANVYHRDLKPKNILANANCKLKICDFGLARVAFNDTPTTVFWTDYVATRWYRAPELCGSFFTKYSPAIDIWSIGCIFAEILTGKPLFPG
TsMPK20-4(U)        HQVIKANDDLTKEHHQFFLYQMLRALKYIHTANVYHRDLKPKNILANANCKLKICDFGLARVAFNDTPTTVFWTDYVATRWYRAPELCGSFFTKYSPAIDIWSIGCIFAEILTGKPLFPG
TuMPK20-4           HQVIKANDDLTKEHHQFFLYQMLRALKYIHTANVYHRDLKPKNILANANCKLKICDFGLARVAFNDTPTTVFWTDYVATRWYRAPELCGSFFTKYSPAIDIWSIGCIFAEILTGKPLFPG
TaMPK20-4(3AL)      HQVIKANDDLTKEHHQFFLYQMLRALKYIHTANVYHRDLKPKNILANANCKLKICDFGLARVAFNDTPTTVFWTDYVATRWYRAPELCGSFFTKYSPAIDIWSIGCIFAEILTGKPLFPG
AetMPK20-4          HQVIKANDDLTKEHHQFFLYQMLRALKYIHTANVYHRDLKPKNILANANCKLKICDFGLARVAFNDTPTTVFWTDYVATRWYRAPELCGSFFTKYSPAIDIWSIGCIFAEILTGKPLFPG
TaMPK20-4(3DL)      HQVIKANDDLTKEHHQFFLYQMLRALKYIHTANVYHRDLKPKNILANANCKLKICDFGLARVAFNDTPTTVFWTDYVATRWYRAPELCGSFFTKYSPAIDIWSIGCIFAEILTGKPLFPG
TaMPK20-4(3B)       HQVIKANDDLTKEHHQFFLYQMLRALKYIHTANVYHRDLKPKNILANANCKLKICDFGLARVAFNDTPTTVFWTDYVATRWYRAPELCGSFFTKYSPAIDIWSIGCIFAEILTGKPLFPG
AK333309.1p         HQVIKANDDLTKEHHQFFLYQMLRALKYIHTANVYHRDLKPKNILANANCKLKICDFGLARVAFNDTPTTVFWTDYVATRWYRAPELCGSFFTKYSPAIDIWSIGCIFAEILTGKPLFPG
                    *************************************************************************************************************************

                                                                               LE^R_KLLAFDPKDRPTAEEAL
HvMPK20-4(3H)       KNVVHQLDLMTDLLGTPSLDTVSRIRNEKARRYLSSMRKKQTVCFSERFPKADPAALKLMQRLLAFDPKDRPTAEEALADPYFKGLGKVEREPSCQPISKFEFEFERKKVTKEDVKELIF
BAJ97013.1          KNVVHQLDLMTDLLGTPSLDTVSRIRNEKARRYLSSMRKKQTVCFSERFPKADPAALKLMQRLLAFDPKDRPTAEEALADPYFKGLGKVEREPSCQPISKFEFEFERKKVTKEDVKELIF
BAJ96852.1          KNVVHQLDLMTDLLGTPSLDTVSRIRNEKARRYLSSMRKKQTVCFSERFPKADPAALKLMQRLLAFDPKDRPTAEEALADPYFKGLGKVEREPSCQPISKFEFEFERKKVTKEDVKELIF
ScMPK20-4           KNVVHQLDLMTDLLGTPSLDTVSRIRNEKARRYLSSMRKKQPVCFSERFPKADAAALKLMQRLLAFDPKDRPTAEEALADPYFKGLGKVEREPSCQPISKFEFEFERKKVTKEDVKELIF
TsMPK20-4(U)        KNVVHQLDLMTDLLGTPSLDTVSRIRNEKARRYLSSMRKKQPVCFSERFPKADAAALKLMQRLLAFDPKDRPTAEEALADPYFKGLGKVEREPSCQPISKFEFEFERKKVTKEDVKELIF
TuMPK20-4           KNVVHQLDLMTDLLGTPSLDTVSRIRNEKARRYLSSMRKKQPVCFSERFPKADPAALKLMQRLLAFDPKDRPTAEEALADPYFKGLGKVEREPSCQPISKFEFEFERKKVTKEDVKELIF
TaMPK20-4(3AL)      KNVVHQLDLMTDLLGTPSLDTVSRIRNEKARRYLSSMRKKQPVCFSERFPKADPAALKLMQRLLAFDPKDRPTAEEALADPYFKGLGKVEREPSCQPISKFEFEFERKKVTKEDVKELIF
AetMPK20-4          KNVVHQLDLMTDLLGTPSLDTVSRIRNEKARRYLSSMRKKQPVCFSERFPKADPAALKLMQRLLAFDPKDRPTAEEALADPYFKGLGKVEREPSCQPISKFEFEFERKKVTKEDVKELIF
TaMPK20-4(3DL)      KNVVHQLDLMTDLLGTPSLDTVSRIRNEKARRYLSSMRKKQPVCFSERFPKADPAALKLMQRLLAFDPKDRPTAEEALADPYFKGLGKVEREPSCQPISKFEFEFERKKVTKEDVKELIF
TaMPK20-4(3B)       KNVVHQLDLMTDLLGTPSLDTVSRIRNEKARRYLSSMRKKQPVCFSERFPKADPAAFKLMQRLLAFDPKDRPTAEEALADPYFKGLGKVEREPSCQPISKFEFEFERKKVTKEDVKELIF
AK333309.1p         KNVVHQLDLMTDLLGTPSLDTVS--------------------------------------------------------------------------------------------------
                    ***********************
```

MPK20-4 continued

```
HvMPK20-4(3H)     REILEYHPQLLKDYMNGTEKTNFLYPS----------------------------AVDNFRRQFANLEENGGKGGAVVPSDRKHVSLP--------------------------------
BAJ97013.1        REILEYHPQLLKDYMNGTEKTNFLYPS----------------------------AVDNFRRQFANLEENGGKGGAVVPSDRKHVSLP--------------------------------
BAJ96852.1        REILEYHPQLLKDYMNGTEKTNFLYPS----------------------------AVDNFRRQFANLEENGGKGGAVVPSDRKHVSLP--------------------------------
ScMPK20-4         REILEYHPQLLKDYMNGTEKTNFLYPGFYHKISSIFFHYMPIFITDPFLVPMLLSAVDNFRRQFANLEENGGKGGAIVPSDRKHVSLPFFGDL---------------------MVLWNR
TsMPK20-4(U)      REILEYHPQLLKDYMNGTEKTNFLYPS----------------------------AVDNFRRQFANLEENGGKGGAIVPSDRKHVSLPVLWSHGTMESLYMTKDGQVVRNFSSSLMIWHD
TuMPK20-4         REILEYHPQLLKDYMNGTEKSNFLYPS----------------------------AVDNFRRQFANLEENGGKGGAIVPSDRKHVSLP--------------------------------
TaMPK20-4(3AL)    REILEYHPQLLKDYMNGTEKTNFLYPS----------------------------AVDNFRRQFANLEENGGKGGAIVPSDRKHVSLP--------------------------------
AetMPK20-4        REILEYHPQLLKDYMNGTEKTNFLYPS----------------------------AVDNFRRQFANLEENGGKGGAIVPSDRKHVSLP--------------------------------
TaMPK20-4(3DL)    REILEYHPQLLKDYMNGTEKTNFLYPS----------------------------AVDNFRRQFANLEENGGKGGAIVPSDRKHVSLP--------------------------------
TaMPK20-4(3B)     REILEYHPQLLKDYMNGTEKTNFLYPS----------------------------AVDNFRRQFANLEENGGKGGAIVPSDRKHVSLP--------------------------------
AK333309.1p       ------------------------------------------------------------------------------------------------------------------------

HvMPK20-4(3H)     -----------------RTTTVHSTPIPPKDQKPSQVPQRIPTGRPGRVVGPVIPFENSCAMDPYSQRRVARNPVLPAAATNLSAYAYHRKSDNTERELQQELEKDRMQYQPMQRFMDAK
BAJ97013.1        -----------------RTTTVHSTPIPPKDQKPSQVPQRIPTGRPGRVVGPVIPFENSCAMDPYSQRRVARNPVLPAAATNLSAYAYHRKSDNTERELQQELEKDRMQYQPMQRFMDAK
BAJ96852.1        -----------------RTTTVHSTPIPPKDQKPSQVPQRIPTGRPGRVVGPVIPFENSCAMDPYSQRRVARNPVLPAAATNLSAYAYHRKSDNTERELQQELEKDRMQYQPMQRFMDAK
ScMPK20-4         FT-----------QKMVRTTTVHSTSIPPKEQKSSQVPQRIPTGRPGRVVGPVIPFENSCAMDPYSQRRVARNPVLPAAATNVSAYAYHRKSDSSERELQQELEKDRMQYQPMQRFMDAK
TsMPK20-4(U)      NFRDCHSTLLADIFAFDRTTTVHSTPIPPKDQKSSQVPQRIPTGRPGRVVGPVIPFENSCAMDPYSQRRVARNPVLPAAATNVSAYAYHRKSDSSERELQQELEKDRMQYQPMQRFMDAK
TuMPK20-4         -----------------RTTTVHSTPIPPKDQKSSQVPQRIPTGRPGRVVGPVIPFENSCAMDPYSQRRVARNPVLPAAATNVSAYAYHRKSDSSERELQQELEKDRMQYQPMQRFMDAK
TaMPK20-4(3AL)    -----------------RTTTVHSTPIPPKDQKSSQVPQRIPTGRPGRVVGPVIPFENSCAMDPYSQRRVARNPVLPAAATNVSAYAYHRKSDSSERELQQELEKDRMQYQPMQRFMDAK
AetMPK20-4        -----------------RTTTVHSTPIPPKDQKSSQVPQRIPTGRPGRVVGPVIPFENSCAMDPYSQRRVARNPVLPAAATNVSAYAYHRKSDSSERELQQELEKDRMQYQPMQRFMDAK
TaMPK20-4(3DL)    -----------------RTTTVHSTPIPPKDQKSSQVPQRIPTGRPGRVVGPVIPFENSCAMDPYSQRRVARNPVLPAAATNVSAYAYHRKSDSSERELQQELEKDRMQYQPMQRFMDAK
TaMPK20-4(3B)     -----------------RTTTVHSTPIPPKDQKSSQVPQRIPTGRPGRVVGPVIPFENSCAMDPYSQRRVARNPVLPAAATNVSAYAYHRKSDSSERELQQELEKDRMQYQPMQRFMDAK
AK333309.1p       ------------------------------------------------------------------------------------------------------------------------

HvMPK20-4(3H)     MVSPDLRSSSYYMPKGVPKADVAERTALQPNMMQGIAPFNGIAAVGGSYNKASAVQYGVSRMY*
BAJ97013.1        MVSPDLRSSSYYMPKGVPKADVAERTALQPNMMQGIAPFNGIAAVGGSYNKASAVQYGVSRMY*
BAJ96852.1        MVSPDLRSSSYYMPKGVPKADVAERTALQPNMMQGIAPFNGIAAVGGSYNKASAVQYGVSRMY*
ScMPK20-4         MVSPDLRSTSYYMPKGVPKADVAERTGLQPNMMQGIAPFNGIAAVGGSYNKASAVQYGVSRMY*
TsMPK20-4(U)      MVSPDLRSTSYYMPKGVPKADVAERTGLQPNMMQGIAPFNGIAAVGGSYNKASAVQYGVSRMY*
TuMPK20-4         MVSPDLRSTSYYMPKGAPKADVAERTGLQPNMMQGIAPFNGIAAVGGSYNKASAVQYGVSRMY*
TaMPK20-4(3AL)    MVSPDLRSTSYYMPKGAPKADVAERTGLQPNMMQGIAPFNGIAAVGGSYNKASAVQYGVSRMY*
AetMPK20-4        MVSPDLRSTSYYMPKGVPKADVAERTGLQPNMMQGIAPFNGIAAVGGSYNKASAVQYGVSRMY*
TaMPK20-4(3DL)    MVSPDLRSTSYYMPKGVPKADVAERTGLQPNMMQGIAPFNGIAAVGGSYNKASAVQYGVSRMY*
TaMPK20-4(3B)     MVSPDLRSTSYYMPKGVPKADVAERTGLQPNMMQGIAPFNGIAAVGGSYNKASAVQYGVSRMY*
AK333309.1p       ----------------------------------------------------------------
```

MPK20-5

```
                                                               IGxGxYGxV             IKKIxxxF    DAxRxLRE                          FxDIYxxxELM
HvMPK20-5(1H)         MPEANAGARGGGEQRSK--SSDVMSFFSEYGDASRYKIEEIIGKGSYGVVCSAIDRQTGDKVAIKKISNIFEHITDAARILREIKLLRLLRHPDIVQIKHIMLPPSRRDFKDIFVVFELM
BAJ88795.1            MPEANAGARGGGEQRSK--SSDVMSFFSEYGDASRYKIEEIIGKGSYGVVCSAIDRQTGDKVAIKKISNIFEHITDAARILREIKLLRLLRHPDIVQIKHIMLPPSRRDFKDIFVVFELM
TaMPK20-5(1AL)        MLEENAGAHIGGEQRDKVQSSDVMSFFSEYGDASRYKIEEIIGKGSYGVVCSAIDRQTGDKVAIKKVSNIFEHITDAARILREIKLLRLLRHPDIVQIKHIMLPPSRRDYKDIFVVFELM
AKL80628.1            MPEANAGARGGGEQRSKVQSSDVMSFFSEYGDASRYKIEEIIGKGSYGVVCSAIDRQTGDRVAIKKVSNIFEHITDAARMLREIKLLRLLRHPDIVQIKHIMLPPSRRDYKDIFVVFELM
TsMPK20-5(U1)         MPEANAGGRGGGEQRSKVQSSDVMSFFSEYGDASRYKIEEIIGKGSYGVVCSAIDRQTGDKVAIKKVSNIFEHITDAARMLREIKLLRLLRHPDIVQIKHIMLPPSRRDYKDIYVVFELM
ScMPK20-5             MPEANAGGRGGGEQRSKVQSSDVMSFFSEYGDASRYKIEEIIGKGSYGVVCSAIDRQTGDKVAIKKVSNIFEHITDAARMLREIKLLRLLRHPDIVQIKHIMLPPSRRDYKDIYVVFELM
TsMPK20-5(U2)         MPEANAGARGGGEQRSKVQSSDVMSFFSEYGDASRYKIEEIIGKGSYGVVCSAIDRQTGDRVAIKKVSNIFEHITDAARMLREIKLLRLLRHPDIVQIKHIMLPPSRRDYKDIFVVFELM
AetMPK20-5            MPEANAGARGGGEQRSKVQSSDVMSFFSEYGDASRYKIEEIIGKGSYGVVCSAIDRQTGDRVAIKKVSNFFEHITDAARMLREIKLLRLLRHPDIVQIKHIMLPPSRRDYKDIFVVFELM
TaMPK20-5(1BL)        MPEANAGARGGGEQRSKVQSSDVMSFFSEYGDASRYKIEEIIGKGSYGVVCSAIDRQTGDRVAIKKVSNIFEHITDAARMLREIKLLRLLRHPDIVQIKHIMLPPSRRDYKDIFVVFELM
TaMPK20-5(U)          MPEANAGARGGGEQRSKVQSSDVMSFFSEYGDASRYKIEEIIGKGSYGVVCSAIDRQTGDRVAIKKVSNIFEHITDAARMLREIKLLRLLRHPDIVQIKHIMLPPSRRDYKDIFVVFELM
                      * * *** .: ***** .*  ***************************************:*****:**:**********:*****************************:***:******

                        DLxxVI                QxLRxLKYxH     HRDLKPxN LxNxNCxLKIxDFGLAR                 TRWYRAPEL           IDxWS^V_IGC
HvMPK20-5(1H)         DTDLHQVIKANDDLTKEHFQFFLYQMLRAMKYIHTANVYHRDLKPKNILA ANCKLKICDFGLARVAFNDTPTTVFWTDYVATRWYRAPELCGSFFTKYSPAIDTWSIGCIFAEILTGKP
BAJ88795.1            DTDLHQVIKANDDLTKEHFQFFLYQMLRAMKYIHTANVYHRDLKPKNILANANCKLKICDFGLARVAFNDTPTTVFWTDYVATRWYRAPELCGSFFTKYSPAIDTWSIGCIFAEILTGKP
TaMPK20-5(1AL)        DTDLHQVIKANDDLTKEHYQFFLYQMLRALKYIHTANVYHRDLKPKNILANANCKLKICDFGLARVAFNDTPTTVFWTDYVATRWYRAPELCGSFFTKYSPAIDTWSIGCIFAEILTGKP
AKL80628.1            DTDLHQVIKANDDLTKEHYQFFLYQMLRALKYIHTANVYHRDLKPKNILANANCKLKICDFGLARVAFNDTPTTVFWTDYVATRWYRAPELCGSFFTKYSPAIDTWSIGCIFAEILTGKP
TsMPK20-5(U1)         DTDLHQVIKANDDLTKEHYQFFLYQMLRALKYIHTANVYHRDLKPKNILANANCKLKICDFGLARVAFNDTPTTVFWTDYVATRWYRAPELCGSFFTKYSPAIDTWSIGCIFAEILTGKP
ScMPK20-5             DTDLHQVIKANDDLTKEHYQFFLYQMLRALKYIHTANVYHRDLKPKNILANANCKLKICDFGLARVAFNDTPTTVFWTDYVATRWYRAPELCGSFFTKYSPAIDTWSIGCIFAEILTGKP
TsMPK20-5(U2)         DTDLHQVIKANDDLTKEHYQFFLYQMLRALKYIHTANVYHRDLKPKNILANANCKLKICDFGLARVAFNDTPTTVFWTDYVATRWYRAPELCGSFFTKYSPAIDTWSIGCIFAEILTGKP
AetMPK20-5            DTDLHQVIKANDDLTKEHYQFFLYQMLRALKYIHTANVYHRDLKPKNILANANCKLKICDFGLARVAFNDTPTTVFWTDYVATRWYRAPELCGSFFTKYSPAIDTWSIGCIFAEILTGKP
TaMPK20-5(1BL)        DTDLHQVIKANDDLTKEHYQFFLYQMLRALKYIHTANVYHRDLKPKNILANANCKLKICDFGLARVAFNDTPTTVFWTDYVATRWYRAPELCGSFFTKYSPAIDTWSIGCIFAEILTGKP
TaMPK20-5(U)          DTDLHQVIKANDDLTKEHYQFFLYQMLRALKYIHTANVYHRDLKPKNILANANCKLKICDFGLARVAFNDTPTTVFWTDYVATRWYRAPELCGSFFTKYSPAIDTWSIGCIFAEILTGKP
                      ******************:**********:********************************************************************************************

                                                                                     LE^R_KLLAFDPKDRPTAEEAL
HvMPK20-5(1H)         LFPGKNVVHQLDLMTDFLGSPSPDIISRIRNEKARRYLSTMRKKLPVPFSEKFPNADPAAVKLLQKLLAFDPKDRPTAEEALADPYFKGLAKVEREPSCQPISKMEFEFERRKFTKEEVK
BAJ88795.1            LFPGKNVVHQLDLMTDFLGSPSPDIISRIRNEKARRYLSTMRKKLPVPFSEKFPNADPAAVKLLQKLLAFDPKDRPTAEEALADPYFKGLAKVEREPSCQPISKMEFEFERRKFTKEEVK
TaMPK20-5(1AL)        LFPGKNVVQQLDMMTDFLGSPSPEIISRIRNEKARRYLSSMRKKLPVPFSEKFPKADPAAVKLLQKLLAFDPKDRPTAEEALADPYFNGLAKVEREPSCQPISKMEFEFERRKFTREDVK
AKL80628.1            LFPGKNVVQQLDMMTDFLGSPSPEIISRIRNEKARRYLSSMRKKLPVPFSEKFPKADPAAVKLLQKLLAFDPKDRPTAEEALADPYFNGLAKVEREPSCQPISKMEFEFERRKFTREDVK
TsMPK20-5(U1)         LFPGKNVVHQLDMMTDFLGSPSPDIISRIRNEKARRYLSTMKKKLPVSFSEKFPNADPAAVKLLQKLLAFDPKDRPTAEEALADPYFNGLAKVEREPSCQPISKMEFEFERRKFTREDVK
ScMPK20-5             LFPGKNVVHQLDMMTDFLGSPSPDIISRIRNEKARRYLSTMKKKLPVSFSEKFPNADPAAVKLLQKLLAFDPKDRPTAEEALADPYFNGLAKVEREPSCQPISKMEFEFERRKFTREEVK
TsMPK20-5(U2)         LFPGKNVVQQLDMMTDFLGSPSPEIISRIRNEKARRYLSSMRKKLPVPFAEKFPKADPAAVKLLQKLLAFDPKDRPTAEEALADPYFNGLAKVEREPSCQPISKMEFEFERRKFTREDIK
AetMPK20-5            LFPGKNVVQQLDMMTDFLGSPSPEIISRIRNEKARRYLSSMRKKLPVPFSEKFPKADPAAVKLLQKLLAFDPKDRPTAEEALADPYFNGLAKVEREPSCQPISKMEFEFERRKFTREDIK
TaMPK20-5(1BL)        LFPGKNVVQQLDMMTDFLGSPSPEIISRIRNEKARRYLSSMRKKLPVPFAEKFPKADPAAVKLLQKLLAFDPKDRPTAEEALADPYFNGLAKVEREPSCQPISKMEFEFERRKFTREDIK
TaMPK20-5(U)          LFPGKNVVQQLDMMTDFLGSPSPEIISRIRNEKARRYLSSMRKKLPVPFSEKFPKADPAAVKLLQKLLAFDPKDRPTAEEALADPYFNGLAKVEREPSCQPISKMEFEFERRKFTREDIK
                      ********:***:**********:***************:*:***** *:****:********************************:***************************:*::*
```

MPK20-5 continued

```
HvMPK20-5(1H)           ELIFREILEYHPQLLKDYTNGSEKTNFLYPSAVDNFRRQFANLEEDGGKGGAPERKHVSLPRTTTVHSTPIPTTSGPASQAPQRIPTARPGRVVASATPIENAAFADRQTGRRMSRDPAA
BAJ88795.1              ELIFREILEYHPQLLKDYTNGSEKTNFLYPSAVDNFRRQFANLEEDGGKGGAPERKHVSLPRTTTVHSTPIPTTSGPASQAPQRIPTARPGRVVASATPIENAAFADRQTGRRMSRDPAA
TaMPK20-5(1AL)          ELIFREILEYHPQLLKDYSNGSEKTNFLYPSAVDNFRRQFANLEEDGGKGGAAERKHVSLPRTTTVHSTPVPTTNCPASQAPQRIPAARPGRVIASATPTENAAFTDRQMGRRMARDPAG
AKL80628.1              ELIFREILEYHPQLLKDYSNGSEKTNFLYPSAVDNFRRQFANLEEDGGKGGAAERKHVSLPRTTTVHSTPVPTTNCPASQAPQRIPAARPGRVIASATPTENAAFTDRQMGRRMARDPAG
TsMPK20-5(U1)           ELIFREILEYHPQLLKDYSNGSEKTNFLYPSAVDNFRRQFANLEEDGGKGGAAERKHVSLPRTTTVHSTPVPTTNCPASQAPQRIPAARPGRVIASATPTENAAFTDRQMGRRMARDPAA
ScMPK20-5               ELIFREILEYHPQLLKDYNNGSEKTNFLYPSAVDNFRRQFANLEEDGGKGGAPERKHVSLPRTTTVHSTPVPTTNGPASQAPQRIPTARPGRVIASAIPTENTAFADRQTGRRMARDPAA
TsMPK20-5(U2)           ELIFREILEYHPQLLKDYTNGSEKTNFLYPSAVDNFRRQFANLEEDGGKGGAPERKHVSLPRTTTVHSTPVPTTNGPASQAPQRIPTARPGRVIASAIPTENTAFADRQTGRRMARDPAA
AetMPK20-5              ELIFREILEYHPQLLKDYTNGSEKTNFLYPSAVDNFRRQFANLEEDGGKGGAPERKHVSLPRTTTVHSTPVPTTNGPTSQAPQRIPTARPGRVIASATPTENAAFADRQMGRRMVRDPAA
TaMPK20-5(1BL)          ELIFREILEYHPQLLKDYTNGSEKTNFLYPSAVDNFRRQFANLEEDGGKGGAPERKHVSLPRTTTVHSTPVPTTNGPAYQAPQRIPTARPGRVIASATPTKNAAFADRQTGRRMARDPAA
TaMPK20-5(U)            ELIFREILEYHPQLLKDYTNGSEKTNFLYPSAVDNFRRQFANLEEDGGKGGAPERKHVSLPRTTTVHSTPVPTTNGPASQAPQRIPTARPGRVIASATPTENAAFTDRQMGRRMARDPAA
                        ******************.********************************* *****************:***. *: *******:******:*** * :*:**:*** **** ****.

HvMPK20-5(1H)           PPAAAAAGYTLRPDCPDRQQQQQQQELEKDRTRYRPALHFRDARVAPEAEARPSAYYIPPFNGIAAVAGGYSKVGAAARMY*
BAJ88795.1              PPAAAAAGYTLRPDCPDRQQQQQQQELEKDRTRYRPALHFRDARVAPEAEARPSAYYIPPFNGIAAVAGGYSKVGAAARMY*
TaMPK20-5(1AL)          APA-AAAGHHLRPDCSDRQ-----HEVEKDRAHYRPSHHFRDARVAPEAEARPSAYYIPPFNGIAAV-------GAAGRMY*
AKL80628.1              APA-AAAGHHLRPDCSDRQ-----HEVEKDRAHYRPSHHFRDARVAPEAEARPSAYYIPPFNGIAAV-------GAAGRMY*
TsMPK20-5(U1)           SS--SGAGYHQRPDCSDRQ-----QEVEKDRAHYRPAHHFRDARVAPEAEARPSAYHIPPFNGIAAVAGGYSKVGVAGRM*-
ScMPK20-5               PPAAAAVGYHLKPDSSDRQQQEQQQEVEKDRARYRPAHHFRDARVAPEAEARPSAYHIPPFNGIAAVAGGYSKVGAAERMY*
TsMPK20-5(U2)           PPAAAVVGYHLKPDSSDRQPEQQQQEVEKERARYRPAHHFRDARVAPEAEARPSAYHIPPFNGIAAAAGGYSKVGAAERMY*
AetMPK20-5              PPAA-AAGYHLKPDCSDRQQEQQQQELEKDRARYRPTHHFRDARVAPEAEARPSAYHIPPFNGIAAVAGGYSKVGAAGRMY*
TaMPK20-5(1BL)          PPAAAAAGYHQRPDCSDRQQ-----EVEKDRAHYRPAHHFRDARVAPEAEARPSAYHIPPFNGIAAVAGGYSKVGVAGRMY*
TaMPK20-5(U)            PPAA-AAGYHQRSGCSDRQQ-----ELEKDRTHYRPAHHFRDARVAPEAEARPSAYYIPPFNGIAAAAGGYSKVGAAGRMY*
                              .*:  : .. ***      *:**:*::***: ******************:*********.       *.* **
```

MPK21-1

```
BAK08351.1          ----------------------------------------------------------------------------------------------------MDPGKKTSESEFFTEYGELN
TsMPK21-1(U)p       MPSPPLVLKPQPLLLPTSPPAPTHSAEKQQQPQPRAHTRSSIAMAAAAAAAAGTREEMVYMAKLAEQAERYEEMVEFMGEAGRLRVALPRAPARTNPPPRMDPGKKTSESEFFTEYGELN
TaMPK21-1(1DL)      ----------------------------------------------------------------------------------------------------MHTHSHTSESEFFTEYGELN
AetMPK21-1          ----------------------------------------------------------------------------------------------------MHTHSHTSESEFFTEYGELN
ScMPK21             ----------------------------------------------------------------------------------------------------MDPGKKTSESEFFTEYGELN
HvMPK21(1H)         ----------------------------------------------------------------------------------------------------MDPGKKTSESEFFTEYGELN
TaMPK21-1(1BL)      ----------------------------------------------------------------------------------------------------MDPGKKTLESEFFTEYGELN
TaMPK21-1(1AL)      ----------------------------------------------------------------------------------------------------MDPGKKTSESEFFTEYGELN
TuMPK21-1           ----------------------------------------------------------------------------------------------------MDPGKKTSESEFFTEYGELN
AKL80629.1          ----------------------------------------------------------------------------------------------------MDPGKKTSESEFFTEYGELN
tplb0009k03         ----------------------------------------------------------------------------------------------------MDPGKKTSESEFFTEYGELN
                                                                                                                        *.  .:* ************

                           IGxGxYGxV             IKKIxxxF    DAxRxLRE                          FxDIYxxxELM  DLxxVI                QxLRxLKYxH
BAK08351.1          RYQVSEVIGKGSYGVVAAAIDTQTGERVAIKKINDVFDHVSDATRILREIKLLRLLRHPDIVQIKHIMLPPSRREFRDIYVVFELMESDLHQVIKANDDLTPEHHQFFLYQLLRGMKYIH
TsMPK21-1(U)p       RYQVSEVIGKGSYGVVAAAIDTQTGERVAIKKINDVFDHVSDATRILREIKLLRLLRHPDIVQIKHIMLPPSRREFRDIYVVFELMESDLHQVIKANDDLTPEHHQFFLYQLLRGMKYIH
TaMPK21-1(1DL)      RYQVSEVIGKGSYGVVAAAIDTQTGERVAIKKINDVFDHVSDATRILREIKLLRLLRHPDIVQIKHIMLPPSRREFRDIYVVFELMESDLHQVIKANDDLTPEHHQFFLYQLLRGMKYIH
AetMPK21-1          RYQVSEVIGKGSYGVVAAAIDTQTGERVAIKKINDVFDHVSDATRILREIKLLRLLRHPDIVQIKHIMLPPSRREFRDIYVVFELMESDLHQVIKANDDLTPEHHQFFLYQLLRGMKYIH
ScMPK21             RYQVSEVIGKGSYGVVAAAIDTQTGERVAIKKINDVFDHVSDATRILREIKLLRLLRHPDIVQIKHIMLPPSRREFRDIYVVFELMESDLHQVIKANDDLTPEHHQFFLYQLLRGMKYIH
HvMPK21(1H)         RYQVSEVIGKGSYGVVAAAIDTQTGERVAIKKINDVFDHVSDATRILREIKLLRLLRHPDIVQIKHIMLPPSRREFRDIYVVFELMESDLHQVIKANDDLTPEHHQFFLYQLLRGMKYIH
TaMPK21-1(1BL)      RYQVSEVIGKGSYGVVAAAIDTQTGERVAIKKINDVFDHVSDATRILREIKLLRLLRHPDIVQIKHIMLPPSRREFRDIYVVFELMESDLHQVIKANDDLTPEHHQFFLYQLLRGMKYIH
TaMPK21-1(1AL)      RYQVSEVIGKGSYGVVAAAIDTQTGERVAIKKINDVFDHVSDATRILREIKLLRLLRHPDIVQIKHIMLPPSRREFRDIYVVFELMESDLHQVIKANDDLTPEHHQFFLYQLLRGMKYIH
TuMPK21-1           RYQVSEVIGKGSYGVVAAAIDTQTGERVAIKKINDVFDHVSDATRILREIKLLRLLRHPDIVQIKHIMLPPSRREFRDIYVVFELMESDLHQVIKANDDLTPEHHQFFLYQLLRGMKYIH
AKL80629.1          RYQVSEVIGKGSYGVVAAAIDTQTGERVAIKKINDVFDHVSDATRILREIKLLRLLRHPDIVQIKHIMLPPSRREFRDIYVVFELMESDLHQVIKANDDLTPEHHQFFLYQLLRGMKYIH
tplb0009k03         RYQVSEVIGKGSYGVVAAAIDTQTGERVAIKKINDVFDHVSDATRILREIKLLRLLRHPDIVQIKHIMLPPSRREFRDIYVVFELMESDLHQGNKANDDLTPEHHQFFLYQLLRGMKYIH
                    ********************************************************************************************  **************************

                         HRDLKPxN LxNxNCxLKIxDFGLAR                 TRWYRAPEL           IDxWS^Y_IGC
BAK08351.1          AASVFHRDLKPKNILANADCKLKICDFGLARVSFNDGAPSAIFWTDYVATRWYRAPELCGSFFSKYTPAIDIWSVGCIFAEMLTGKPLFPGKNVVHQLDLMTDVLGTPSAESLAKIRNEK
TsMPK21-1(U)p       AASVFHRDLKPKNILANADCKLKICDFGLARVSFNDGAPSAIFWTDYVATRWYRAPELCGSFFSKYTPAIDIWSVGCIFAEMLTGKPLFPGKNVVHQLDLMTDVLGTPSAESLAKIRNEK
TaMPK21-1(1DL)      AASVFHRDLKPKNILANADCKLKICDFGLARVSFNDGAPSAIFWTDYVATRWYRAPELCGSFFSKYTPAIDIWSVGCIFAEMLIGKPLFPGKNVVHQLDLMTDVLGTPSAESLAKIRNEK
AetMPK21-1          AASVFHRDLKPKNILANADCKLKICDFGLARVSFNDGAPSAIFWTDYVATRWYRAPELCGSFFSKYTPAIDIWSVGCIFAEMLIGKPLFPGKNVVHQLDLMTDVLGTPSAESLAKIRNEK
ScMPK21             AASVFHRDLKPKNILANADCKLKICDFGLARVSFNDGAPSAIFWTDYVATRWYRAPELCGSFFSKYTPAIDIWSVGCIFAEMLTGKPLFPGKNVVHQLDLMTDVLGTPSAESLAKIRNEK
HvMPK21(1H)         AASVFHRDLKPKNILANADCKLKICDFGLARVSFNDGAPSAIFWTDYVATRWYRAPELCGSFFSKYTPAIDIWSVGCIFAEMLTGKPLFPGKNVVHQLDLMTDVLGTPSAESLAKIRNEK
TaMPK21-1(1BL)      AASVFHRDLKPKNILANADCKLKICDFGLARVSFNDGAPSAIFWTDYVATRWYRAPELCGSFFSKYTPAIDIWSVGCIFAEMLTGKPLFPGKNVVHQLDLMTDVLGTPSAESLAKIRNEK
TaMPK21-1(1AL)      AASVFHRDLKPKNILANADCKLKICDFGLARVSFNDGAPSAIFWTDYVATRWYRAPELCGSFFSKYTPAIDIWSVGCIFAEMLTGRPLFPGKNVVHQLDLMTDVLGTPSAESLAKIRNEK
TuMPK21-1           AASVFHRDLKPKNILANADCKLKICDFGLARVSFNDGAPSAIFWTDYVATRWYRAPELCGSFFSKYTPAIDIWSVGCIFAEMLTGRPLFPGKNVVHQLDLMTDVLGTPSAESLAKIRNEK
AKL80629.1          AASVFHRDLKPKNILANADCKLKICDFGLARVSFNDGAPSAIFWTDYVATRWYRAPELCGSFFSKYTPAIDIWSVGCIFAEMLIGKPLFPGKNVVHQLDLMTDVLGTPSAESLAKIRNEK
tplb0009k03         AASVFHRDLKPKNILANADCKLKICDFGLARVSFNDGAPSAIFWTDYVATRWYRAPELCGSFFSKYTPAIDIWSVGCIFAEMLIGKPLFPGKNVVHQLDLMTDVLGTPSAESLAKIRNEK
                    *********************************************************************************** *:**********************************
```

MPK21-1 continued

$\mathbf{LE^{R}_{K}LLAFDPKDRPTAEEAL}$

```
BAK08351.1          ARRYLSNMRKKPKVPLTKKFPGIDPMALHLLERLLAFDPKDRPTADEALTDPYFTGLANSEREPIAQPISKLEFEFEKRKLGKDDGERELEGQIGHHGRSPGTIIGTREEPVARRGGAGI
TsMPK21-1(U)p       ARRYLSNMRKKPKVPLTKKFPGIDPMALHLLERLLAFDPKDRPTADEALTDPYFNGLANSEREPIAQPISKLEFEFEKRKLGKDDVRELIYR----------------------------
TaMPK21-1(1DL)      ARRYLSNMRKKPKVPLTKKFPGIDPMALHLLERLLAFDPKDRPTADEALTDPYFTGLANSEREPIAQPISKLEFEFEKRKLGKDDVRELIYREILEYH--PQML------QEYLRGGDQM
AetMPK21-1          ARRYLSNMRKKPKVPLTKKFPGIDPMALHLLERLLAFDPKDRPTADEALTDPYFTGLANSEREPIAQPISKLEFEFEKRKLGKDDVRELIYREILEYH--PQML------QEYLRGGDQM
ScMPK21             ARRYLSNMRKKPKVPLTKKFPGIDPMALHLLERLLAFDPKDRPTADEALTDPYFNGLANSEREPVAQPISKFEFDFEKRKLGKDDVRELIYREILEYH--PQML------QEYLRGGDQM
HvMPK21(1H)         ARRYLSNMRKKPKVPLTKKFPGIDPMALHLLERLLAFDPKDRPTADEALTDPYFTGLANSEREPIAQPISKLEFEFEKRKLGKDDVRELIYREILEYH--PHML------QEYLRGGDQM
TaMPK21-1(1BL)      ARRYLSNMRKKPKVPLTKKFPGIDPMALHLLERLLAFDPKDRPTADEALTDPYFTGLANSEREPIAQPISKLEFEFEKRKLGKDDVRELIYREILEYH--PQML------QEYLRGGDQM
TaMPK21-1(1AL)      ARRYLSNMRKKPKVPLTKKFPGIDPMALHLLERLLAFDPKDRPTADEALTDPYFNGLANSEREPIAQPISKLEFEFEKRKLGKDDVRELIYREILEYH--PQML------QEYLRGGDQM
TuMPK21-1           ARRYLSNMRKKPKVPLTKKFPGIDPMALHLLERLLAFDPKDRPTADEALTDPYFNGLANSEREPIAQPISKLEFEFEKRKLGKDDVRELIYREILEYH--PQML------QEYLRGGDQM
AKL80629.1          ARRYLSNMRKKPKVPLTKKFPGIDPMALHLLERLLAFDPKDRPTADEALTDPYFTGLANSEREPIAQPISKLEFEFEKRKLGKDDVRELIYREILEYH--PQML------QEYLRGGDQM
tplb0009k03         ARRYLSNMRKKPKVPLTKKFPGIDPMALHLLERLLAFDPKDRPTADEALTDPYFTGLANSEREPIAQPISKLEFEFEKRKLGKDDVRELIYREILEYH--PQML------QEYLRGGDQM
                    ******************************************************.*********:******:**:********** .. :

BAK08351.1          ANRWAASRGRRWRYPAFRRRSHRHRRWRRPQEQALRTQGKSRSPPRTADRRPAPHRRWARKARSTGASLAARQRHRRRRSRSSRSSSSSSTRSPAGRSVSRTAWARRGRSSPSRASLPSP
TsMPK21-1(U)p       ------------------------------------------------------------------------------------------------------------------------
TaMPK21-1(1DL)      SF----------MYPSGVDRFKQ--QFAHLEK--GGAKGEKSSP-QLRQNASLPRERA------IGNKHGDSD----------YQVKLNTGEKPVHASV------TDGISKPLMSA----
AetMPK21-1          SF----------MYPSGVDRFKQ--QFAHLEK--GGAKGEKSSP-QLRQNASLPR--A------IGNKHGDSD----------YQVKLNTGEKPVHASV------TDGISKPLMSA----
ScMPK21             SF----------MYPSGVDRFKQ--QFAHLEK--GGAKGEKSSP-QLRQNASLPRERA------IGNKHGDSE----------YQVKLNTGEKPVHASV------TDGISKPLMSA----
HvMPK21(1H)         SF----------MYPSGVDRFKQ--QFAHLEK--GGAKGDKSSP-QLRQNASLPRERA------IGNKHGDSE----------YQVKLNTGEKPVHASV------TDGISKPLMSA----
TaMPK21-1(1BL)      SF----------MYPSGVDRFKQ--QFAHLEK--GGAKGEKASP-QLRQNASLPRERA------IGNKHGDSE----------YQVKLNAGEKPVHASV------TDGISKPLMSA----
TaMPK21-1(1AL)      SF----------MYPSGVDRFKQ--QFAHLEK--GGAKGEKSSP-QLRQNASLPRERA------IGNKHGDSD----------YQVKLNAGEKPVHASV------TDGISKPLMSA----
TuMPK21-1           SF----------MYPSGVDRFKQ--QFAHLEK--GGAKGEKSSP-QLRQNASLPRERA------IGNKHGDSD----------YQVKLNAGEKPVHASV------TDGISKPLMSA----
AKL80629.1          SF----------MYPSGVDRFKQ--QFAHLEK--GGAKGEKSSP-QLRQNASLPRERA------IGNKHGDSD----------YQVKLNTGEKPVHASV------TDGISKPLMSA----
tplb0009k03         SF----------MYPSGVDRFKQ--QFAHLEK--GGAKGEKSSP-QLRQNASLPRERA------IGNKHGDSD----------YQVKLNTGEKPVHASV------TDGISKPLMSA----

BAK08351.1          SSGASRRPSSRRSWAPCSRRTAATSCGWPRRSAPTGGSSRAG*----
TsMPK21-1(U)p       -----------------------------------------------
TaMPK21-1(1DL)      ---RSLLKSETMSASKCIGEIKNKDDEYESVDAADGVSQKVAQLKT*
AetMPK21-1          ---RSLLKSETMSASKCIGEIKNKDDEYESVDAADGVSQKVAQLKT*
ScMPK21             ---RSLLKSETMSASKCIGEIKNKDDEYESVDAADGVSQKIAQLKT*
HvMPK21(1H)         ---RSLLKSETMSASKCIGEIKNKDDELESVDAADGVSQKIAQLKT*
TaMPK21-1(1BL)      ---RSLLKSETMSASKCIGEVKNKDDEYESVDAADGVSQKVAQLKT*
TaMPK21-1(1AL)      ---RSLLKSETMSASKCIGEIKNKDDEYESVDAADGVSQKIAQLKT*
TuMPK21-1           ---RSLLKSETMSASKCIGEIKNKDDEYESVDAADGVSQKIAQLKT*
AKL80629.1          ---RSLLKSETMSASKCIGEIKNKDDEYESVDAADGVSQKVAQLKT*
tplb0009k03         ---RSLLKSETMSASKCIGEVKNKDDEYESVDAADGVSQKVAQLKT*
```

MPK21-2

```
                                                  IGxGxYGxV             IKKIxxxF    DAxRxLRE                          FxDIYxxxELM  DLxxVI
TsMPK21-2(U1)       MAKKGSDKAGFFTEYGEATRYEVCEVVGKGSYGVVASAVDTHTGESVAIKKIDDVFEHVADATRILREIKLLRLLRHPDIVQIKHIMLPPSRREFRDIYIIFELMESDLHQVIKANDDLS
TaMPK21-2(3AL)      MAKKGSDKAGFFTEYGEATRYEIGEVVGKGSYGVVAAAVDTHTGERVAIKKIDDVFEHVADATRILREIKLLRLLRHPDIVEIKHIMLPPSRREFRDIYIIFELMESDLHQVIKANDDLS
HvMPK21-2(U)        MSKKGSDKAGFFTEYGEATRYEVGEVVGKGSYGVVAAAVDTHTGERVAIKKIDDVFEHVSDATRILREVKLLRLLRHPDIVQIKHIMLPPSRREFRDIYIVFELMESDLHQVIKANDDLS
TsMPK21-2(U2)       MAKKGSDKAGFFTEYGEATRYEVCEVVGKGSYGVVASAVDTHTGESVAIKKIDDVFEHVADATRILREIKLLRLLRHPDIVQIKHIMLPPSRREFRDIYIIFELMESDLHQVIKANDDLS
TsMPK21-2(U3)       MAKKGSDKAGFFTEYGEATRYEVCEVVGKGSYGVVASAVDTHTGESVAIKKIDDVFEHVADATRILREIKLLRLLRHPDIVQIKHIMLPPSRREFRDIYIIFELMESDLHQVIKANDDLS
TaMPK21-2(3B)       MAKKGSDKGGFFTEYGEATRYQVGEVVGKGSYGVVASAVDTHTGESVAIKKIDDVFEHVADATRILREIKLLRLLRHPDIVQIKHIMLPPSRREFRDIYIIFELMESDLHQVIKANDDLS
AK330685.1          MAKKGSDKGGFFTEYGEATRYQVGEVVGKGSYGVVASAVDTHTGESVAIKKIDDVFEHVADATRILREIKLLRLLRHPDIVQIKHIMLPPSRREFRDIYIIFELMESDLHQVIKANDDLS
AetMPK21-2          MAKKGTDKGAFFTEYGEATRYQVGEVVGKGSYGVVASAVDTHTGEPVAIKKIDDVFEHVADATRILREIKLLRLLRHPDIVQIKHIMLPPSRREFRDIYIIFELMESDLHQVIKANDDLS
TaMPK21-2(3DL)      MAKKGTDKGAFFTEYGEATRYQVGEVVGKGSYGVVASAVDTHTGEPVAIKKIDDVFEHVADATRILREIKLLRLLRHPDIVQIKHIMLPPSRREFRDIYIIFELMESDLHQVIKANDDLS
                    *:***:**..***********:: ************:******** *************:********:************:******************:*******************

                             QxLRxLKYxH     HRDLKPxN LxNxNCxLKIxDFGLAR                 TRWYRAPEL           IDxWS^V_IGC
TsMPK21-2(U1)       PEHHQFFFYQLLRGMKYIHAANVFHRDLKPKNILANADCKLKICDFGLARVSFDDTPSAIFWTDYVATRWYRAPELCGSFFSKYTPAIDIWSIGCIFAEMLTGRPLFPGKNVVHQLDLMT
TaMPK21-2(3AL)      PEHFQFFFYQLLRGMKYMHAANVFHRDLKPKNILANADCKLKICDFGLSRVSFNDTPSAIFWTDYVATRWYRAPELCGSFFSKYTPAIDIWSIGCIFAEMLTGRPLFPGKNVVHQLDLMT
HvMPK21-2(U)        PEHHQFFFYQLLRGMKYIHAANVFHRDLKPKNILANADCKLKICDFGLARVSFNDTPSAIFWTDYVATRWYRAPELCGSFFSKYTPAIDIWSIGCIFAEMLSGRPLFPGKNVVHQLDLMT
TsMPK21-2(U2)       PEHHQFFFYQLLRGMKYIHAANVFHRDLKPKNILANADCKLKICDFGLARVSFDDTPSAIFWTDYVATRWYRAPELCGSFFSKYTPAIDIWSIGCIFAEMLTGRPLFPGKNVVHQLDLMT
TsMPK21-2(U3)       PEHHQFFFYQLLRGMKYIHAANVFHRDLKPKNILANADCKLKICDFGLARVSFDDTPSAIFWTDYVATRWYRAPELCGSFFSKYTPAIDIWSIGCIFAEMLTGRPLFPGKNVVHQLDLMT
TaMPK21-2(3B)       PEHHQFFFYQLLRGMKYIHAANVFHRDLKPKNILANADCKLKICDFGLARVSFNDTPSAIFWTDYVATRWYRAPELCGSFFSKYTPAIDIWSIGCIFAEMLTGRPLFPGKNVVHQLDLMT
AK330685.1          PEHHQFFFYQLLRGMKYIHAANVFHRDLKPKNILANADCKLKICDFGLARVSFNDTPSAIFWTDYVATRWYRAPELCGSFFSKYTPAIDIWSIGCIFAEMLTGRPLFPGKNVVHQLDLMT
AetMPK21-2          PEHHQFFFYQLLRGMKYIHAANVFHRDLKPKNILANADCKLKICDFGLARVSFNDTPSAIFWTDYVATRWYRAPELCGSFFSKYTPAIDIWSIGCIFAEMLTGRPLFPGKNVVHQLDLMT
TaMPK21-2(3DL)      PENHQFFFYQLLRGMKYIHAANVFHRDLKPKNILANADCKLKICDFGLARVSFNDTPSAIFWTDYVATRWYRAPELCGSFFSKYTPAIDIWSIGCIFAEMLTGRPLFPGKNVVHQLDLMT
                    **:.*************:******************************:****:***********************************************:******************

                                                                    LE^R_KLLAFDPKDRPTAEEAL
TsMPK21-2(U1)       DLLGTPSAESISRIRNEKARRYLGNMKKKHPIPFSQKFPGVDPMALHLLERLLAFDPADRPTAAEALADPYFTGLANSELEPTTQPISKLEFEFERRKLAREDLRELIYREILEYHPQML
TaMPK21-2(3AL)      DLLGTPSEESISRVRNEKARRYLGNMKKKHPIPFSQKFPGVDPMALHLLERLLAFDPADRPTAAEALADPYFTGLANSELEPTAQPISKLEFEFERRKLAREDLRELIYREILEYHPQML
HvMPK21-2(U)        DLLGTPSAESISRIRNEKARRYLGNMKKKHPIPFSQKFPGVDPMALHLLERLLAFDPADRPTAAEALADPYFTGLANSELEPTTQPISKLEFEFERRKLAREDVRELIYREILEYHPQML
TsMPK21-2(U2)       DLLGTPSAESISRIRNEKARRYLGNMKKKHPIPFSQKFPGVDPMALHLLERLLAFDPADRPTAAEALADPYFTGLANSELEPTTQPISKLEFEFERRKLAREDLRELIYREILEYHPQML
TsMPK21-2(U3)       DLLGTPSAESISRIRNEKARRYLGNMKKKHPIPFSQKFPGVDPMALHLLERLLAFDPADRPTAAEALADPYFTGLANSELEPTTQPISKLEFEFERRKLAREDLRELIYREILEYHPQML
TaMPK21-2(3B)       DLLGTPSAESISRIRNEKARRYLGNMKKKHPIPFSQKFPGVDPMALHLLERLLAFDPADRPTAAEALADPYFTGLANSELEPTTQPISRLEFEFERRKLGREDVRELIYREILEYHPQML
AK330685.1          DLLGTPSAESISRIRNEKARRYLGNMKKKHPIPFSQKFPGVDPMALHLLERLLAFDPADRPTAAEALADPYFTGLANSELEPTTQPISRLEFEFERRKLGREDVRELIYREILEYHPQML
AetMPK21-2          DLLGTPSAESISRIRNEKARRYLGNMKKKHPIPFSQKFPGVDPMALHLLERLLAFDPTDRPTAAEALADPYFTGLANSELEPTTQPISRLEFEFERRKLAREDVRELIYREILEYHPQML
TaMPK21-2(3DL)      DLLGTPSAESISRIRNEKARRYLGNMKKKHPIPFSQKFPGVDPMALHLLERLLAFDPTDRPTAAEALADPYFTGLANSELEPTTQPISRLEFEFERRKLAREDVRELIYREILEYHPQML
                    ******* *****:*******************************************:*************************:****:**********.***:****************
```

MPK21-2 continued

```
TsMPK21-2(U1)       QEYLRGGDQMSFMYPSGVDRFKQQFAHLEKGGAKGEKSSPQLRQNASLPRERAIGNKH----GDSEYQVKLNAGEKPVHASVTDGISKPLMSARSLLKSETMSASKCIGEIKNKDDEYES
TaMPK21-2(3AL)      HDYLRGGDQANFLYPSGVDRFKSQFVHLEEIGAKGEKTSPQLRQHASLPRERVIGSSDEPEKPNADYCIKLHVDELPGHTSVTDGLNKPLLNTRNFLKSESIGASKCIVVKEKQEKDEES
HvMPK21-2(U)        HEYLCGGDQANFLYPSGVDRFKRQFVHLEEIGAKGEKTSPQLRQHASLPRERVISSSDEPEKPNADYCIKLHVGELPGHTSVTDGLNKPLLNTRNFLKSESIGASKCIVIKEKREKDEES
TsMPK21-2(U2)       HDYLRGGDQANFLYPSGVDRFKRQFVHLEEIGAKGEKTSPQLRQHASLPRERVIGS-DDPEKPNADYCIKLHVGELPGHTSVTDGLNKPLLNTRNFLKSESIGASKCIVVKEKREKDEES
TsMPK21-2(U3)       HDYLRGGDQANFLYPSGVDRFKRQFVHLEEIGAKGEKTSPQLRQHASLPRERVIGS-DDPEKPNADYCIKLHVGELPGHTSVTDGLNKPLLNTRNFLKSESIGASKCIVVKEKREKDEES
TaMPK21-2(3B)       HEYLRGGDQANFLYPSGVDRFKRQFVHLEEIGAKGEKTSPQLRQHASLPRERVIGSSDEPEKPNADYCIKLHVGEVPGYTSVTDGLNKPLLNTRNFLKSESIGASKCIVVKEKREKDEES
AK330685.1          HEYLRGGDQANFLYPSGVDRFKRQFVHLEEIGAKGEKTSPQLRQHASLPRERVIGSSDEPEKPNADYCIKLHVGEVPGYTSVTDGLNKPLLNTRNFLKSESIGASKCIVVKEKREKDEES
AetMPK21-2          HEYLRGGDQANFLYPSGVDRFKRQFVHLEEIGAKGEKTSPQLRQHASLPRERVIGSSDEPEKPNADYCIKLHVGEVPGHTSVTDGLNKPLLNTRNFLKSESIGASKCIVVKEKREKDEES
TaMPK21-2(3DL)      HEYLRGGDQANFLYPSGVDRFKRQFVHLEEIGAKGEKTSPQLRQHASLPRERVIGSSDEPEKPNADYCIKLHVGEVPGHTSVTDGLNKPLLNTRNFLKSESIGASKCIVVKEKREKDEES
                    ::** **** .*:********* **.***: ******:******:*******.*.. .     :::* :**:..* * ::*****:.***:.:*.:****::.*****   ::::.: **

TsMPK21-2(U1)       VDA-----ADGVSQKIAQLKT*-
TaMPK21-2(3AL)      MSEYMHEASD*------------
HvMPK21-2(U)        MSEYMHEVSDEEPKMIAQPKSL*
TsMPK21-2(U2)       MSEYMHEASD*------------
TsMPK21-2(U3)       MSEYMHEASD*------------
TaMPK21-2(3B)       MSEYMHEASD*------------
AK330685.1          MSEYMHEASD*------------
AetMPK21-2          MSDYMHEA*--------------
TaMPK21-2(3DL)      MSDYMHEA*--------------
```

MPK24

```
                                  IGxGxYGxV             IKKIxxxF    DAxRxLRE                          FxDIYxxxELM  DLxxVI
HvMPK24(6H)     MEFFTEYGEASQYQIKEIIGKGSFGVVAAAVDTQTGEWVAIKKIHDMFEHASDGTRILREIKLLRLLRHPNIVEIKHILLPPARREFRDIYVVFELMESDLQKVIQVNDNLTKGHHRFFL
TaMPK24(6BS)    MEFFTEYGEASQYQIEEIIGQGSFGVVAAAVDTQTGERVAIKKIHDMFEHAPDGTRILREIKLLRLLRHPNIVGVKHILLPPARREFRDIYVVFELMESDLQKVIQVNENLTAGHHRYFL
AK334728.1      MEFFTEYGEASQYQIEEIIGQGSFGVVAAAVDTQTGERVAIKKIHDMFEHAPDGTRILREIKLLRLLRHPNIVGVKHILLPPARREFRDIYVVFELMESDLQKVIQVNENLTAGHHRYFL
ScMPK24         MEFFTEYGEASQYQIKEIIGRGSFGVVAAAVDTQTGERVAIKKIHDMFEHASDGTRILREIKLLRLLRHPNIVEIKHILLPPARREFRDIYVVFEFMESDLQKVMQVNENLSADHHRFFL
TaMPK24(U)      MEFFTEYGEASQYQIEEIIGKGSFGVVAAAVDTQTGERVAIKKIHDMFEHASNGTRILREIKLLRLLRHPNIVETKHILLPPARREFRDIYVVFELMESDLQKVIQANENLTAGHHRFFL
TsMPK24(U)      MEFFTEYGEASQYQIEEIIGRGSFGVVAAAVDTQTGERVAIKKIHDMFEHASDGTRILWEIKLLRLLRHPNIVEIKHILLPPARREFRDIYVVFELMESDLQKVIQVNENLTAGHHRYFL
TuMPK24         MEFFTEYGEASQYQIEEIIGKGSFGVVAAAVDTQTGERVAIKKIHDMFEHASNGTRILREIKLLRLLRHPNIVETKHILLPPARREFRDIYVVFELMESDLQKVIQANENLTAGHHRFFL
TaMPK24(6DS)    MEFFTEYGEASQYQIEEIIGRGSFGVVAAAVDTQTGERVAIKKIHDMFEHASDGTRILWEIKLLRLLRHPNIVEIKHILLPPARREFRDIYVVFELMESDLQKVMQVNENLTAGHHRYFL
AetMPK24        MEFFTEYGEASQYQIEEIIGRGSFGVVAAAVDTQTGERVAIKKIHDMFEHASDGTRILWEIKLLRLLRHPNIVEIKHILLPPARREFRDIYVVFELMESDLQKVMQVNENLTAGHHRYFL
                ***************:****:**************** ************* :***** **************  ********************:********:*.*:**: .***:**

                 QxLRxLKYxH     HRDLKPxN LxNxNCxLKIxDFGLAR                 TRWYRAPEL           IDxWS(V/I)GC
HvMPK24(6H)     YQLLHALKYIHAANVFHRDLKPSNILANSNCKLKICDFGLARASRDDVPSAIFWTDYVATRWYRAPELCGSFFSKYTPAIDIWSIGCIFAEVLTRNPLFPGTNVTHQLDLITDVLGTPSH
TaMPK24(6BS)    YQLLHALKYIHAANVFHRDLKPSNILANSNCKLKICDFGLARALRDDAPLAIFWTDYVATRWYRAPELCGSFFSKYTPAIDIWSIGCIFAEVLTGKPLFPGMNVTHQLDLITNVLGTPSY
AK334728.1      YQLLHALKYIHAANVFHRDLKPSNILANSNCKLKICDFGLARALRDDAPLAIFWTDYVATRWYRAPELCGSFFSKYTPAIDIWSIGCIFAEVLTGKPLFPGMNVTHQLDLITNVLGTPSY
ScMPK24         YQLLAALKYIHAANVFHRDLKPSNILVNSNCKLKICDFGLARPLCDDAPLAIFWTDYVATRWYRAPELCGSFFSKYTPAIDIWSIGCIFAEVLTRKPLFPGRNGTHQLDLI-TILGNPSC
TaMPK24(U)      YQLLHALKYIHAANVFHRDLKPSNILVNSNCKLKICDFGLARASRDDPPLAIFWTDYVATRWYRAPELCGSFFSKXXXXXXXXXXXXXXXXXXXXXXXXXXXXVKHQLDLITDVLGTPSY
TsMPK24(U)      YQLLHALKYIHAANVFHRDLKPSNILVNSNCKLKICDFGLARPL-DDAPLAIFWTDYVATRWYRAPELCGSFFSKYTPAIDIWSIGCIFAEVLTGKPLFPGRNGTHQLDLITDVLGNPSC
TuMPK24         YQLLHALKYIHAANVFHRDLKPSNILVNSNCKLKICDFGLARASRDDPPLAIFWTDYVATRWYRAPELCGSFFSKYTPAIDIWSIGCIFAEVLTRKPLFPGTNVKHQLDLITDVLGTPSY
TaMPK24(6DS)    YQLLHALKYIHAANVFHRDLKPSNILVNSNCKLKICDFGLARASRDDPPLAIFWTDYVATRWYRAPELCGSFFSKYTPAIDIWSIGCIFAEVLTRKPLFPGTNVKHQLDLITDVLGTPSH
AetMPK24        YQLLHALKYIHAANVFHRDLKPSNILVNSNCKLKICDFGLARASRDDPPLAIFWTDYVATRWYRAPELCGSFFFKYTPAIDIWSIGCIFAEVLTRKPLFPGTNVKHQLDLITDVLGTPSH
                **** *********************.***************   ** * *********************** *                             .******  :**.** 

                                                        LE(R/K)LLAFDPKDRPTAEEAL
HvMPK24(6H)     ETLSQIRNEKARRYLTGMKRKHPIPFPRMFCSADPQAVRLLERLLAFDPKDRPTAEEALADPYFEGLPKLEHEPSPYPFSKLDFEFERWKLSKDGIRDLIYQEILEYHPQMLQDYIRGGG
TaMPK24(6BS)    ETLSQIRNEKARRYLTGMKRKHPIPFPSIFCNADPQAVRLLERLLAFDPKDRPTAEEALADPYFEGFPNLEHEPSPHPFSKLEFEFERWKLTKDGVRDLIYREILEYHPQMLQDYIRAGG
AK334728.1      ETLSQIRNEKARRYLTGMKRKHPIPFPSIFCNADPQAVRLLERLLAFDPKDRPTAEEALADPYFEGFPNLEHEPSPHPFSKLEFEFERWKLTKDGVRDLIYREILEYHPQMLQDYIRAGG
ScMPK24         ETLSQIRNEKARRYLTGMKRKHPIPFASMFCNADPKAVRLLERLLAFDPKDRPTAEEALADPYFEGLRKLEHEPLPHPFSKLEFEFERLKLTKGGVRDLIYREILEYHPQMLQDYIRAGG
TaMPK24(U)      ETLSQIRNEKARIYLTGMKRKHPIPFPSMFCNADPQAVRLLERLLAFDPKDRPTAEEALADPYFEGFPNLEHEPSPHPFSKLEFEFERWKLTKDGIRDLIYREILEYHPQMLQDYIGAGG
TsMPK24(U)      ETLSQIRNEKARRYLTGMKRKHPIPFASVFCNADPKAVRLLERLLAFDPKDRPTAEEALADPYFEGLRKLEHEPLPHPFSKLEFEFERLKLTKGGVRDLIYREILEYHPQMLQDYIRAGG
TuMPK24         ETLSQIRNEKARIYLTGMKRKHPIPFPSMFCNADPQAVRLLERLLAFDPKDRPTAEEALADPYFEGFPNLEHEPSPHPFSKLEFEFERWKLTKDGIRDLIYREILEYHPQMLQDYIGAGG
TaMPK24(6DS)    ETLSQIRNEKARRYLTGMKRKHPIPFPSMFCNADPQAVRLLERLLAFDPKDRPTAEEALADPYFEGFPNLEHEPSPHPFSKLEFEFERWKLTKDGIRDLIYREILEYHPQMLQDYIRAGG
AetMPK24        ETLSQIRNEKARRYLTGMKRKHPIPFPSMFCNADPQAVRLLERLLAFDPKDRPTAEEALADPYFEGFPNLEHEPSPHPFSKLEFEFERWKLTKDGIRDLIYREILEYHPQMLQDYIRAGG
                ************ *************  :**.***:******************************: :***** *:*****:***** **:*.*:*****:************** .**
```

MPK24 continued

```
HvMPK24(6H)         QTSFVYPSGVDGMRLQFAHAEDKYLRGERGT-PLRRRHASLPRERFRAPMGSDNQDCNNENRRTASSAGQIIIRPQQVGMTHAYVYQNGTGIPDFCSGYYLQNGGTSDSSCAIGENEGPK
TaMPK24(6BS)        QTSFVYPSGVDGMRLQFVHLEENHLRGERGT-PLRRRHASLPRERVCAPMGIDNQDCNNERR-TASSAAQITIRSQQEGLTHAYVYQNGTGIPDFCSGYYLQNGSTSASSCVIEENEGPK
AK334728.1          QTSFVYPSGVDGMRLQFVHLEENHLRGERGT-PLRRRHASLPRERVCAPMGIDNQDCNNERR-TASSAAQITIRSQQEGLTHAYVYQNGTGIPDFCSGYYLQNGSTSASSCVIEENEGPK
ScMPK24             QTSFVYPSGVDGMRLQFVHLEENHLRGERGTTPPQRRHASLPRERVCTPMGSDKHDCNNENRRTASCAAQTIIRSQQGGLTHAYVNQNGTSIPNFCSGYYLQNGSASASSCVIEENEGPK
TaMPK24(U)          QTSFVYPSGVDGMRLQFVHLEENHLRGERGT-PLRRRHASLPRERVCAPKGSDNQDCNNERRRTASSAAQTTIRSQQEGLTHAYVYQNGTSIPNFCSGYYLQNGSTSASSCVIDEHEGLE
TsMPK24(U)          QTSFVYPSGVDGMRLQFVHLEENHLRGERGT-PLRRRHASLPRERVCAPKGSDNQDCNNERRRTASSAAQTTIRSQQEGLTHAYVYQNGTSIPNFCSGYYLQNGSTSASSCVIEENEGLE
TuMPK24             QTSFVYPSGVDDMRLQFVHLEENHLRGERGT-PLRRRHASLPRERVCAPKGSDNQDCNNERRRTASSAAQTTIRSQQEGLTHAYVYQNGTSIPNFCSGYYLQNGSTSASSCVIDEHEGL-
TaMPK24(6DS)        QTSFVYPSGVDDMRLQFVHLEENHLRGERGT-PLRRRHASLPRERVCAPKGSDNQDCNNERRRTASSAAQTTIRSQQEGLTHAYVYQNGTSIPNFCSGYYLQNGSTSASSCVIDDHEGLK
AetMPK24            QTSFVYPSGVDDMRLQFVHLEENHLRGERGI----------DRERVCAPKGSDNQDCNNERRRTASSAAQTTIRSQQEGLTHAYVYQNGTSIPNFCSGYYLQNGSTSASSCVIDDHEGLK
                    ***********.*****.* *:::******            ***. :* * *::*****.* ***.*.*  ** ** *:***** ****.**:**********.:* ***.* ::**

HvMPK24(6H)         ENGVSEEEKVAYEPSELLAKI*
TaMPK24(6BS)        ENGVSA-EKVAYELSERLARI*
AK334728.1          ENGVSA-EKVAYELSERLARI*
ScMPK24             ENGVPEEEKVAYELSERLARI*
TaMPK24(U)          ENGVSEEEKVAYELSQCFARI*
TsMPK24(U)          ENGVSEEEKVAYELSERLARI*
TuMPK24             ENGVSEEEKVAYELSQCFARI*
TaMPK24(6DS)        ENGVSEEEKVAYELSQCFARI*
AetMPK24            ENGVSEEEKVAYELSQCFARI*
                    ****   ****** *: :*:**
```

MPK25

```
                                                       IGxGxYGxV             IKKIxxxF    DAxRxLRE                          FxDIYxxxELM  DL
TsMPK25(U)        MAKMVRPPNGKGKHGKHYYTMCQTMFEIDTKYVPIKPIGRGSYGIVCSSVNKDTNEQVAIKKINNVFDNRVDALRMLREMKLLRHLRHENVIALKDIMMPIQKRSFKDVYLVSELMDTDL
TaMPK25(5AL)      MAKMVAPLNGKQNHGKHYYTMDQTMFEIDTKYVPIKPIGSGSYGVVFSFMNKDTNEKVAIKKINNVFDSREDALRTLREMKLLRHLTHENVIALKDVMMPIRRRSFKDVYLVSELMDTDL
HvMPK25(5H)       MAKMVDPPKGKGSHGKHYYTMCQTMFEIDTKYVPIEPIGRGAYGVVCSSMNTDTNEKVAIKKINNVFDNREDALRTLRKLKLLRHLRHENVIALKDIMMPMKRRSYKDVYLVSELMDTDL
TaMPK25(5BL)      MAKMIDPPKGKGNSGKHYYIMCGTMFEIDTKYVPIKPIGRGSYGVVCSSMNKDTNEKVAIKKINNIFDNREDALRTLRELKLLRHLSHENVIALKDVMMPIQRRNFKDVYLVSELMDTDL
ScMPK25           MKKMVGPPNGRRNHGKHYYTMGQTMFEIDTKYVPIKPIGKGAYGMVCSSMNKDTDEKVAIKKINNVFDNREDALRTLRELKLLRHLCHENVIALKDVMMPIQKRSFKDVYFVSELMDTDL
AetMPK25          -----------------------------------------------------------------------------------------------------------------ELMDTDV
TaMPK25(5DL)      MAKMVDPPKGKGNHGKHYYIMCETMFEIDTKYVPIKPIGKRSYGVVCSSMNKDTNEKVVIKKINNVFDT-TDALRTLRELKLLRHLSHENVIALKDVMMPIQRRNFKDVYLVAGLMDTDL
                                                                                                                                    *****:

                  xxVI                QxLRxLKYxH     HRDLKPxN LxNxNCxLKIxDFGLAR              TRWYRAPEL          IDxWS$^{V}_{I}$GC
TsMPK25(U)        DRIINSSQTLSNDHCQYFLFQLLRGLKYLHSAGILHRDLKPLNLLVDGNCDLKICDFGLARTNNTEDQCMTEYVVTRWYRAPELLLSNDNYGTSIDVWSVGCIFAELLGRKPIFPGTDSL
TaMPK25(5AL)      QHIINSSQTLSNDHCQYFLFQLLRGLKYLHSARILHRDLKPGNLLVNGNCDLKICDFGLARTNNTKGQLMTEYVGTRWYRAPELLLSCNNYGNSIDVWSVGCIFAELLGRKSIFPGTDCL
HvMPK25(5H)       HQIIKSPQALSNDHCQYFLFQLLRGLKYLHSAGILHRDLKPGNLLVNGNCDLKICDFGLARTNNTNGLSMTEYVVTRWYRAPELLLSFDKYGTSVDVWSVGCIFAELLGRKSIFPGTDCL
TaMPK25(5BL)      QKIISSCITLSNDHCQYFLFQLLRGLKYLHSAGILHRDLKPENLLVNANCDLKICDFGLARTINAKVQSMTGYVVTRWYRAPELLLGCDNYGTSIDDWSVGCIFAELLGRKSIFPGTDCL
ScMPK25           QQIINSSQTLSNDHCQYFLFQLLRGLKYLHSAGVLHRDLKPGNLLVNGNCDLKICDFGLARTNNTKGQSMTEYVVTRWYRAPELLLSCDNYGASIDVWSVGCIFAELLGRKSIFPGTDCL
AetMPK25          QKIINSSIMLSNDQCQYFLFQLLRGLKYLHSAGILHRDLKPGNLLVNGKCDLKICDFGLARTNNAKGQFMTEYVVTRWYRAPELLLSCDNYGTSIDVWSVGCIFAELLGRKSIFPGTDCL
TaMPK25(5DL)      QKIINSSITLSNDHCQYLLFQLLRGLKYLHSAGILHRDLKPGNLLVNGNC--------LARTNNAKGQFMTEYVVTRWYRAPELLLSCDNYGTSIDVWSVGCILAELLGRKSIFPGTDCL
                  .:**.*   ****:***:************** :******* ****:.:*        **** *::   ** ** ***********. ::** *:* ******:******* ******.*

                                                                           KMLIFDP$^{S}_{T}$KRISVTEAL
TsMPK25(U)        NQLQLIVNVLGTMNNADLEFIDNPRPHSYIKSLSYTAGIPLTGMYPQAHPLAIDLLQKMLVFNPSKRISVTEALEHPYMSRLYDPSVSRPAQAPINLDIDENLSVEMLREMMWQEMLQYHEAAMMVNM*
TaMPK25(5AL)      NQLKLIVNVLGTMSGADLKFIENKKTRKYIKSLPYTIGIPLTGMYPQAHPLA-DLLQKMLVFDPSKRISVTEALEHPYMSTLYDHNANHPAQVPIDIDIDEKLSVEMIREMLWREMLQYP*--------
HvMPK25(5H)       NQLKLIVDVLGTMSDANLEFIGNTKARKYVKSLSYTVGVPLTRMYPQAHPLAIDLLEKMLVFDPSKRISVTEALEHPYMSTLYDPSANHPAQAPIDLDIDEKLGVDMIREMLWQEMLQYP*--------
TaMPK25(5BL)      NQLKLIVNVLGTMSDDDLEFIDNMKARKYIKSLSYADGIPLTSMYPQADPLAIDLLQKMLVFDPSNRISVTEALEHPYMSTLYDPDANPPAQAPIDLDIDEKLGVDMIREMLWQEMLQYP*--------
ScMPK25           NQLKLIVNVLGTMSGADLEFIDSMKARKYIKSLSHAVGTPLTRMYPQAHPLAIDLLQKMLVFDPSKRISVTEALEHPYMSMLYDPSANPPAQAPIDLDIDEKLGVEMIREMLWQEMLQYP*--------
AetMPK25          NQLKLILNVLGTMSDDDLEFIGNMKARKYIKSLSYTAGIPLTRMYPQAHPLAIDLLQKMLVFDPSKRISVTEALEHPYMSVLYDPGANPPAQAPIDLDIDEKLGVEMIREMLWQEMLQYP*--------
TaMPK25(5DL)      NQLKLIVNVLGTMSDDDLEFIGNMKARKYIKSLSYTAGIPLTRMYPQAHPLAIDLLQKMLVFDPSKRISVTEALEHPYMSVLYDPSANPPAQAPIDLDIDEKLGVEMIREMLWQEMLQYP*--------
                  ***:**::*****.. :*:** . : :.*:*** :: * *** *****.*** ***:*****:**:************** *** ... ***.**::****:*.*:*:***:*:*****
```
