# Supplementary material for: Analysis of MAPK and MAPKK gene families in wheat and related Triticeae species
Source: BMC Genomics. 2018 Mar 5;19:178. doi: 10.1186/s12864-018-4545-9 (PMC5838963; doi:10.1186/s12864-018-4545-9)
Supplement: Supplementary file 8 — Alignment and domain analysis of Triticeae MKKs. The amino acid sequences from multiple genomic copies, along with the accessions reported in Additional file 3, of all Triticeae MKKs identified were aligned in Clustal Omega (www.ebi.ac.uk/Tools/msa/clustalo/) under default settings. A lower case ‘p’ next to the gene name indicates that only a partial sequence was identified for the given gene. The protein kinase domain is in black font, and the N- and C-terminal extensions in teal font. Missing sequence information, namely ‘X’ residues, are shown in pink font. Highlighted sequences are the ATP binding signature (blue) which carries the P-loop consensus sequence (GxGxxG), the catalytic C-loop (red) with the DΨK consensus (where Ψ refers to the aliphatic amino acids L/I/V, but not M), the activation T-loop (black), NTF2 domain (pink). Sequence deviations from the S/TxxxxxS/T activation loop of plant MKKs are marked in yellow font. Other deviations from consensus are highlighted in yellow. An asterix indicates a stop codon. (PDF 173 kb) [file 12864_2018_4545_MOESM8_ESM.pdf]

Additional File 8. Alignment and domain analysis of Triticeae MKKs. The amino acid sequences from multiple genomic copies, along with the accessions reported in Additional File 3, of all Triticeae MKKs identified were aligned in Clustal Omega (www.ebi.ac.uk/Tools/msa/clustalo/) under default settings. A lower case ‘p’ next to the gene name indicates that only a partial sequence was identified for the given gene. The protein kinase domain is in black font, and the N- and C-terminal extensions in teal font. Missing sequence information, namely ‘X’ residues, are shown in pink font. Highlighted sequences are the ATP binding signature (blue) which carries the P-loop consensus sequence (GxGxxG), the catalytic C-loop (red) with the DΨK consensus (where Ψ refers to the aliphatic amino acids L/I/V, but not M), the activation T-loop (black), NTF2 domain (pink). Sequence deviations from the S/TxxxxxS/T activation loop of plant MKKs are marked in yellow font. Other deviations from consensus are highlighted in yellow. An asterix indicates a stop codon.

MKK1

```
                                                                          GxGxxG
HvMKK1-2(6H)     --------------------XXXXXXXXXXXXXXXXXXXXXXXXXXXXXXAPPIKSLDN-HQLSINDLEAIKVIGKGSSGTVQLVRQKWTGQFFALKVIQLNIQESIRKQIARELKISLS
TaMKK1-3(7DS)    ---------------MGKFWTRSGTSKYSDGDLLVNKDGFRIIPQTKEGKAPPVEPPEN-HELSIDDLDAIKEIGKGSSGTVQLVHHKWTGQFFALKVIQLNVQESIRKQIAQELKINLS
HvMKK1-1(7H)     MKKPGKLALPSHDSTIGKFLTQSGTFK--DGDLLVNKDGLRIVPQSEEGEAPPIKPLDN-HQLSIDDLDSIKVIGKGSSGTVQLVRHKWTGQFFALKVIQLNIQESIRKQIAQELKISLS
HP624471.1       MKKPGKLALPSQDSTIGKFLTQSGTFK--DGDLLVNKDGLRIVPQSEEGEAPPIKPLDNNHQLSIDDLDSIKVIGKGNSGTVQLVRHKWTGQFFALKVIQLNIQESIRKQMAQELKISLF
TaMKK1-1(7DS)    MKKPGKLALPSQDSTIGKFLTQSGTFK--DGDLLVNKDGLRIVPQSEEGEAPPIKPLDNNHQLSIDDLDSIKVIGKGNSGTVQLVRHKWTGQFFALKVIQLNIQESIRKQMAQELKISLF
AetMKK1-1        MKKPGKLALPSQDSTIGKFLTQSGTFK--DGDLLVNKDGLRIVPQSEEGEAPPIKPLDNNHQLSIDDLDSIKVIGKGNSGTVQLVRHKWTGQFFALKVIQLNIQESIRKQMAQELKISLF
TaMKK1-1(4AL)    MKKPGKLALPSQDSTIGKFLTQSGTFK--DGDLLVNKDGLRIVPQSEEGEAPRIKPLDNNHQLSIDDLDSIKVIGKGNSGTVQLVRHKWTGQFFALKVIQLNIQESIRKQMAQELKISLF
TuMKK1-1         MKKPGKLALPSQDSTIGKFLTQSGTFK--DGDLLVNKDGLRIVPQSEEGEAPPIKPLDNNHQLSIDDLDSIKVIGKGNSGTVQLVRHKWTGQFFALKV-QLNIQESIRKQMAQELKISLF
ScMKK1           MKKPGKLALPSQDSTIGKFLTQSGTFK--DGDLLVNKDGLRIVPQSEEGEAPPIKPLDNNHQLSIDDLDSIKVIGKGNSGTVQLVRHKWTGQFFALKVIQLNIQESIRKQMAQELKISLF
TaMKK1-1(7AS)    MKKPGKLALPSQDSTIGKFLTQSGTFK--DGDLLVNKDGLRIVPQSEEGEAPPIKPLDNNHQLSIDDLDSIKVIGKGNSGTVQLVRHKWTGQFFALKVIQLNIQESIRKQMAQELKISLF
HP621269.1       --------------------MQIGMFKDGDGNLLVNKDGLRITPQTEEGEAPPIEPLDN-HQLSIHDLEAIKVIGKGSSGTVQLVRQKWTGQFFALKVIQLNIQESIRKQLAQELKLSLS
TaMKK1-2(6BS)    --------------------MQIGMFKDGDGNLLVNKDGLRITPQTEEGEAPPIEPLDN-HQLSIHDLEVIKVIGKSSSGTVQLVRQKWTGQFFALKVIQLNIQESIRKQLAQELKISLS
TaMKK1-2(U)      --------------------MQIGMFKDGDGNLLVNKDGLRITPQTEEGEAPPIEPLDN-HQLSIHDLEAIKVIGKGSSGTVQLVRQKWTGQFFALKVIQLNIQESIRKQLAQELKLSLS
AetMKK1-2        --------------------MQIGMFKDGDGNLFVNKDGLRITPQTEEGEAPPIEPLDN-HHLSIHDLEAIKVIGKGSSGTVQLVRQKWTGQFFALK-IQLNIQESIRKQLAQELKLSLS
TaMKK1-2(6AS)    --------------------MQIGMFKDGDGNLLVNKDGLRITPQTKEGEAPPIEPLDN-HQLSIHDLEAIKVIGNG-SGTVQLVRQKWTGQFLALKVIQLNIQESIRKQLAQELKLSLS
TuMKK1-2         --------------------MQIGMFKDGDGNLLVNKDGLRITPQTEEGEAPPIEPLDN-HQLSIHDLEAIKVIGKGSSGTVQLVRQKWTGQFLALKVIQLNIQESIRKQLA*ELKLSLS
                                                                   ** ::  :* *.***.**: ** **:. *******::******:***  ***:*******:* ***:.*

                                                                                     DΨK
HvMKK1-2(6H)     TQCQYVVTCYQCFYVNGVICIALEYMDGGSLADFLMTVRTVPEAYLAVICKQQVLKGLMYLHHEKQIIHRDLKPSNILINHRGEVKISDFGVSAIISSSSAQRDTFTDTFNYMAPERING
TaMKK1-3(7DS)    TQCQYVVTCYQCFYINGVISIALEYMDGGSLADFLKGVGTIPESYLAAVCKK-VLKGLMYLHHERRIIHRDLKPSNTLINHMGEVKISDFGVSAIISNSSAQRDMFTGTFNYMAPERISG
HvMKK1-1(7H)     TQCQYVVTCYQCFYVNGVISIALEYMDGGSLADFLKAVRTVPEAYLAAICKQ-VLKGLMYLHNEKHVLHRDLKPSNILINHRGEVKISDFGVSAIIASSSAQRDTFTGTFNYMAPERISG
HP624471.1       TQCQYVVTCYQCFYVNGVISIALEYMDGGSLADFLKAVRTVPEAYLAAICKQ-VLKGLMYLHHEKRVIHRDLKPSNILINHRGEVKISDFGVSAIISSSSAQRDTFTGTFNYMAPERISG
TaMKK1-1(7DS)    TQCQYVVTCYQCFYVNGVISIALEYMDGGSLADFLKAVRTVPEAYLAAICKQ-VLKGLMYLHHEKRVIHRDLKPSNILINHRGEVKISDFGVSAIISSSSAQRDTFTGTFNYMAPERISG
AetMKK1-1        TQCQYVVTCYQCFYVNGVISIALEYMDGGSLADFLKAVRTVPEAYLAAICKQ-VLKGLMYLHHEKRVIHRDLKPSNILINHRGEVKISDFGVSAIISSSSAQRDTFTGTFNYMAPERISG
TaMKK1-1(4AL)    TQCQYVVTCYQCFYVNGVISIALEYMDGGSLADFLKAVRTVPEAYLAAICKQ-VLKGLMYLHHEKRVIHRDLKPSNILINHRGEVKISDFGVSAIISSSSAQRDTFTGTFNYMAPERISG
TuMKK1-1         TQCQYVVTCYQCFYVNGVISIALEYMDGGSLADFLKAVRTVPEAYLAAICKQ-VLKGLMYLHHEKRVIHRDLKPSNILINHRGEVKISDFGVSAIISSSSAQRDTFTGTFNYMAPERISG
ScMKK1           TQCQYVVTCYQCFYVNGVISIALEYMDGGSLADFLKAVRTVPEAYLAAICKQ-VLKGLMYLHHEKRVIHRDLKPSNILINHRGEVKISDFGVSAIISSSSAQRDTFTGTFNYMAPERISG
TaMKK1-1(7AS)    TQCQYVVTCYQCFYVNGVISIALEYMDGGSLADFLKAVRTVPEAYLAAICKQ-VLKGLMYLHHEKRVIHRDLKPSNILINHRGEVKISDFGVSAIISSSSAQRDTFTGTFNYMAPERISG
HP621269.1       TQCQYVVTCYQCFYVNGVISIALEYMDGGSLADFLMTARTVPEAYLAAICKQ-VLKGLMYLHHEKRIIHRDLKPSNILINHRGEVKISDFGVSAIISSSSTQRDIFTGIFNYMAPERISG
TaMKK1-2(6BS)    TQCQYVVTCYQCFYVNGVISIALEYMDGGSLADFLMTVRTVPEAYLAAICKQ-VLKGLMYLHHEKRIIHRDLKPSNILINHRGEVKISDFGVSAIISSSSTQRDIFTGIFNYMAPERISG
TaMKK1-2(U)      TQCQYVVTCYQCFYVNGVISIALEYMDGGSLADFLMTARTVPEAYLAAICKQ-VLKGLMYLHHEKRVIHRDLKPSNILINHRGEVKISDFGVSAIISSSSAQRDTFTGTFNYMAPERISG
AetMKK1-2        TQCQYVVTCYQCFYVNGVISIALEYMDGGSLADFLMTARTVPEAYLAAICKQ-VLKGLMYLHHEKRVIHRDLKPSNILINHRGEVKISDFGVSAIISSSSAQRDTFTGTFNYMAPERISG
TaMKK1-2(6AS)    TQCQYVVTCYQCFYVNGVISIALEYMDGGSLADFLMTARTVPEAYLAAICKQ-VLKGLMYLHHEKRVIHRDLKPSNILINHRGEVKISDFVVSAIISSSSAKRDTFTGTFNYMAPERISG
TuMKK1-2         TQCQYVVTCYQCFYVNGVISIALEYMDGGSLADFLMTARTVPEAYLAAICKQ-VLKGLMYLHHEKRVIHRDLKPSNILINHRGEVKISNFGVSAIISSSSAKRDTFTGTFNYMAPERISG
                 **************:****.***************  . *:**:***.:**: *********:*::::******** **** ******:* *****:.**::** **. *********.*
```

MKK1 continued

```
HvMKK1-2(6H)       QKHGYRSDIWSLGLVMLECATGNFPYPPCDSFYELFETVVDQPSPSAPTDQFSAEFCSFISACMHKEATNRSSAQTLSAHPFLSMYDDLNIDLAVYFRTAGSPLVTFK*----
TaMKK1-3(7DS)      QKHGYMSDIWSLGLVMLECATGKFPYPPCDSFYELLEAVVDQPPPSAPSDKFSPQFCSFISACMQKNAPDRSYAQLLSAHPFLSMYDNQNIDLSDYFTTAGSPLATFK*----
HvMKK1-1(7H)       QKHGYMSDIWSLGLVMLECATGNFPYPPRDSFYELLEAVVDQPSPSAPPDQFSAEFCSFVSACIQKNATDRSSAQTLSAHPFLSMYDDLNIDLSDYFTTAGSPLATFKQIAL*
HP624471.1         QKHGHLSDIWSLGLVMLECATGNFPYPPRESFYELLEAVVDQPSPSAPSDQFSPEFCSFISACIQKNAADRSSAQTLSVHPFLSMYDDLNIDLSDYFRTAGSPLATFKQIAL*
TaMKK1-1(7DS)      QKHGHLSDIWSLGLVMLECATGNFPYPPRESFYELLEAVVDQPSPSAPSDQFSPEFCSFISACIQKNAADRSSAQTLSVHPFLSMYDDLNIDLSDYFRTAGSPLATFKQIAL*
AetMKK1-1          QKHGHLSDIWSLGLVMLECATGNFPYPPRESFYELLEAVVDQPSPSAPSDQFSPEFCSFISACIQKNAADRSSAQTLSVHPFLSMYDDLNIDLSDYFRTAGSPLATFKQIAL*
TaMKK1-1(4AL)      QKHGHLSDIWSLGLVMLECATGNFPYPPRESFYELLEAVVDQPSPSAPSDQFSPEFCSFISACIQKNAADRSSAQTLSAHPFLSMYDDLNIDLSDYFTTAGSPLATFKQIAL*
TuMKK1-1           QKHGYMSDIWSLGLVMLECATGNFPYPPRESFYELLEAVVDQPSPSAPSDQFSPEFCSFISACIQKNAADRSSAQTLSAHPFLSMYDDLNIDLSDYFTTAGSPLATFKQIAL*
ScMKK1             QKHGYMSDIWSLGLVMLECATGNFPYPPRESFYELLEAVVDQPSPSAPSDQFSPEFCSFISACIQKDAADRSSAQTLSAHPFLSMYDDLNINLSDYFTTAGSPLATFKQIAL*
TaMKK1-1(7AS)      QKHGYMSDIWSLGLVMLECATGNFPYPPRESFYELLEAVVDQPSPSAPSDQFSPEFCSFISACIQKNAADRSSAQTLSAHPFLSMYDDLNIDLSDYFTTAGSPLATFKQIAL*
HP621269.1         QKHGYMSDIWSLGLVMLECATGNFPYPPRDSFYELLEAVVDQPSPSAPSDQFSPEFCSFISACMQKEATNRSSAQILSAHPFLSMYNDLNIDLADYFRTAGSPLVTFK*----
TaMKK1-2(6BS)      QKHGYMSDIWSLGLVMLECATGNFPYPPRDSFYELLEAVVDQPSPSAPSDQFSPEFCSFISACMQKEATNRSSAQILSAHPFLSMYNDLNIDLADYFRTAGSPLVTFK*----
TaMKK1-2(U)        QKHGYMSDIWSLGLVMLECATGNFPYPPRGSFYELLEDVVDQPSPSAPSDQFSPEFCSFISACMQKEATNRSSAQILSAHPFLSMYDDLNVDLAVYFRTAGSPLVTFK*----
AetMKK1-2          QKHGYMSDIWSLGLVMLECATGNFPHPPRGSFYELLEDVVDQPSPSAPSDQFSPEFCSFISACVQKKATNRSSAQILSAHPFLSMYDDLNVDLAVYFRTAGSPLVTFK*----
TaMKK1-2(6AS)      QKHGYMSDIWSLGLVMLECATGNFPYPPRDSFYELLEAVVDQPSPSAPSDQFSPEFCSFISACMQKEATNRSSAQILS-VPFLSMYDDLNIDLAVYFRTAGSPLVTFK*----
TuMKK1-2           QK-------------MLECATGNFPYPPRDSFYELLEAVVDQPSPSAPSDQFSPEFCSFISACMQKEATNRSSAHILS-VPFLSMYDDLNIDLAVYFRTAGSPLVTFK*----
                   **             *******:**:**  *****:* ***** **** *:** :****:***::*.* :** *: **  ******:: *::*: ** ******.***
```

MKK3

```
                                                                                                        GxGxxG
TaMKK3(3B)p       ------------------------------------------------------------------------------------------------------------------------
TuMKK3p           ------------------------------------------------------------------------------------------------------------------------
HvMKK3-3(6H)      MAGLEELKRRLQPIFFDADGNVVPPPAADGTHDDSSSDDCEVTLDGGTVNLLSRSSDEYNISKLGFHKRTTRPDGDYATDKAYRCSCHDMHIFDSVGNGASSVVHRAIYVPVHRVLALKK
TaMKK3-3(5AL)     MAGLEDLKRRLQPIFFDADGNVVPPPAADGTSDDSSSDDCEV-LDSGTVNLLSRSSDEYNISKLGFHKRTTRPDGDYATDKAYRCSCHDMHIFDSVGIGASSVVHRAIYVPVHRVLALKK
TuMKK3-3          MAGLEDLKRRLQPIFFDADGNAVPPPAADGTSDDSSSDDCEV-LDSGTVNLLSRSSDEYNISKLGFHKRTTRPDGDYATDKAYRCSCHDMHIFDSVGIGASSVVHRAIYVPVHRVLALKK
TaMKK3-3(5BL)     MAGLEDLKRRLQPIFFDADGNVVPPPAADGTSDDSSSDDCEV-LDGGTVNLLSRSSDEYNISKLGFHKRTTRPDGDYATDKAYRCSCHDMHIFDSVGNGASSVVYRAIYVPVHRVLALKK
TsMKK3-3(U)       MAGLEDLKRRLQPIFFDADGNVVPPPAADGTSDDSSSDDCEV-LDSGTVNLLSRSSDEYNISKLGFHKRTTRPDGDYATDKAYRCSCHDMHIFDSVGNGASSVVHRAIYVPVHRVLALKK
ScMKK3-3          MAGLEELKRRLQPIFFDADGNVVPPPAADGTSDDSSSDDCEV-LDGGTVNLLSRSSDEYNISKLGFHKRTTRPDGDYATDKAYRCSCHDMHIFDSVGNGASSVVHRAIYVPVHRVLALKK
TaMKK3-3(5DL)     MAGLEELKRRLQPIFFDADGNVVPPPAADGTSDDSSSDDCEV-LDGGTVNLLSRSSDEYNISKLGFHKRTTRPDGDYATDKAYRCCCHDMHIFDSVGNGASSVVHRAIYVPVHRVLALKK
TsMKK3-2(U2)p     MSGLEELKKKLQPLLFDDTDKGGVSTRVP---FPEDTCDSYVVSDGGTINLLSRSFGEYNINEHGFHKRSTGPEEPDSGEKAYRCASEDMHIFGPIGNGASSVVQRAIFIPVHRILALKK
BAJ96085.1        ----------------------------------------MKVSDGGTINLLSRSFGEYNINEHGFHKRSTGPEEPDTGEKAYRCASEDMHIFGPIGNGASSVVQRAIFIPVHRILALKK
AKS50325.1        MAGLEELKKKLQPLLFDDSDKGGVSTRVP---FPEDTCDSYVVSDGGTINLLSRSFGEYNINEHGFHKRSTGPEEPDTGEKAYRCASEDMHIFGPIGNGASSVVQRAIFIPVHRILALKK
TuMKK3-2          MSGLEELKKKLQPLLFDDTDKGGVSTRVP---FPEDTCDSYVVSDGGTINLLSRSFGEYNINEHGFHKRSTGPEEPDTGEKAYRCASEDMHIFGPIGNGASSVVQRAIFIPVHRILALKK
ScMKK3-1          MSGLEELKKKLQPLLFDDSDKGGVSTRVP---FPEDTCDSYVVSDGGTINLLSRSFGEYNINEHGFHKRSTGPEEPDTGEKAYRCASEDMHIFGPIGNGASSVVQRAIFIPVHRILALKK
TsMKK3-2(U1)      MAGLEELKKKLQPLLFDDSDKGGVSTRVP---FPEDTCDSYVVSDGGTINLLSRSFGEYNINEHGFHKRSTGPEEPDTGEKAYRCASEDMHIFGPIGSGASSVVQRAIFIPVHRILALKK
HvMKK3-2(5H)      MAGLEELKKKLQPLLFDDSDKGGVSTRVP---FPEDTCDSYVVSDGGTINLLSRSFGEYNINEHGFHKRSTGPEEPDTGEKAYRCASEDMHIFGPIGNGASSVVQRAIFIPVHRILALKK
BAU62004.1        MAGLEELKKKLQPLLFDDSDKGGVSTRVP---FPEDTCDSYVVSDGGTINLLSRSFGEYNINEHGFHKRSTGPEEPDTGEKAYRCASEDMHIFGPIGNGASSVVQRAIFIPVHRILALKK
TaMKK3-2(5BL)     MAGLEELKKKLQPLLFDDSDKGGVSTRVP---FPEDTCDSYVVSDGGTINLLSRSFGEYNINEHGFHKRSTGPEEPDTGEKAYRCASEDMHIFGPIGSGASSVVQRAIFIPVHRILALKK
TsMKK3-2(B)       MAGLEELKKKLQPLLFDDSDKGGVSTRVP---FPEDTCDSYVVSDGGTINLLSRSFGEYNINEHGFHKRSTGPEEPDTGEKAYRCASEDMHIFGPIGSGASSVVQRAIFIPVHRILALKK
AetMKK3-2         MAGLEELKKKLQPLLFDDSDKGGVSTRVP---FPEDTCDSYVVSDGGTINLLSRSFGEYNINEHGFHKRSTGPEEPDTGEKAYRCASEDMHIFGPIGSGASSVVQRAIFIPVHRILALKK
TaMKK3-2(5DL)     MSGLEELKKKLQPLLFDDSDKGGVSTRVP---FPEDTCDSYVVSDGGTINLLSRSFGEYNINEHGFHKRSTGPEEPDTGEKAYRCASEDMHIFGPIGNGASSVVQRAIFIPVHRILALKK
```

MKK3 continued

```
                                                                                                                           DΨK
TaMKK3(3B)p      ------------------------------------------------------------------------------------------------------------------------
TuMKK3p          ------------------------------------------------------------------------------------------------------------KIADFGVSAGLD
HvMKK3-3(5H)     INIFEKEKRQQILNEIRTLSAASCYPGLVEFQGVFYTPDSGEIYFALEYMDGGSLADIIRVKKFITEPVLSHMLQKVLLALRYLHEVRRLVHRDIKPANLLLNLKGDTKITDFGVTSGLH
TaMKK3-3(5AL)    ISIFDKEKRQQILNEITTLSAASCYPGLVEFQGIFYTPDSGEIYFALEYMDGGSLADIIRVKKFITEPVLSHMLQKVLLALRYLHEVRRLVHRDIKPANLLLNLKRDTKITDFGVTSGLH
TuMKK3-3         ISIFDKEKRQQILNEITTLSAASCYPGLVEFQGIFYTPDSGEIYFALEYMDGGSLADIIRVKKFITEPVLSHMLQKVLLALRYLHEVRRLVHRDIKPANLLLNLKGDTKITDFGVTSGLH
TaMKK3-3(5BL)    INIFDKERRQQILNEIRTLSAASCYPGLVEFQGVFYTPDSGEIYFALEYMDGGSLADIIRVKKFITEPVLSHMLQKVLLALRYLHEVRRLVHRDIKPANLLLNLKGDTKITDFGVTSGLH
TsMKK3-3(U)      INIFDKERRQQILNEIRTLSAASCYPGLVEFQGVFYTPDSGEIYFALEYMDGGSLADIIRVKKFITEPVLSHMLQKVLLALRYLHEVRRLVHRDIKPANLLLNLKGDTKITDFGVTSGLH
ScMKK3-3         IN-FEKEKRQQILNEIRTLSAASCYPGLVEFQGVFYTPDSGEIYFALEYMDGGSLADIIRVKKFITEPVLSHMLQKVLLALRYLHEVRRLVHRDIKPANLLLNLKGDTKITDFGVTSGLH
TaMKK3-3(5DL)    INIFDKEKRQQILNEIRTLSAASCYPGLVEFQGVFYTPDSGEIYFALEYMDGGSLADIIRVKKFITEPVLSHMLQKVLLALRYLHEVRRLVHRDIKPANLLLNLKGDTKITDFGVSSGLH
TsMKK3-2(U2)p    INIFEKEKRQQILNEMRTLCEASCYPGLVEFQGAFYMPDSGQISIALEYMDGGSLADVIRVKKSIPEPVLAHMLLKVLLGLKYLHEVRHLVHRDLKPANILVNLKGEAKITDFGVSAGLD
BAJ96085.1       INIFEKEKRQQILNEMRTLCEASCYPGLVEFQGAFYMPDSGQISIALEYMDGGSLADVIKVKKSIPEPVLAHMLLKVLLGLKYLHEARHLVHRDLKPANILVNLKGEAKITDFGVSAGLD
AKS50325.1       INIFEKEKRQQILNEMRTLCEASCYPGLVEFQGAFYMPDSGQISIALEYMDGGSLADVIKVKKSIPEPVLAHMLQKVLLGLKYLHEVRHLVHRDLKPANLLVKLKGEAKITDFGVSAGLD
TuMKK3-2         INIFEKEKRQQILNEMRTLCEASCYPGLVEFQGAFYMPDSGQISIALEYMDGGSLADVIKVKKSIPEPVLAHMLQKVLLGLKYLHEVRHLVHRDLKPANLLVNLKGEAKITDFGVSAGLD
ScMKK3-1         INIFEKEKRQQILNEMRTLCEASCYPGLVEFQGAFYMPDSGQISIALEYMDGGSLADVIRVKKSIPEPVLAHMLLKVLLGLKYLHEVRHLVHRDLKPANILVNLKGEAKITDFGVSAGLD
TsMKK3-2(U1)     INIFEKEKRQQILNEMRTLCEASCYPGLVEFQGAFYMPDSGQISIALEYMDGGSLADVIRVKKSIPEPVLAHMLLKVLLGLKYLHEVRHLVHRDLKPANILVNLKGEAKITDFGVSAGLD
HvMKK3-2(5H)     INIFEKEKRQQILNEMRTLCEASCYPGLVEFQGAFYMPDSGQISIALEYMDGGSLADVIKVKKSIPEPVLAHMLLKVLLGLKYLHEARHLVHRDLKPANILVNLKGEAKITDFGVSAGLD
BAU62004.1       INIFEKEKRQQILNEMRTLCEASCYPGLVEFQGAFYMPDSGQISIALEYMDGGSLADVIKVKKSIPEPVLAHMLLKVLLGLKYLHEARHLVHRDLKPANILVNLKGEAKITDFGLSAGLD
TaMKK3-2(5BL)    INIFEKEKRQQILNEMRTLCEASCYPGLVEFQGAFYMPDSGQISIALEYMDGGSLADVIRVKKSIPEPVLAHMLLKVLLGLKYLHEVRHLVHRDLKPANILVNLKGEAKITDFGVSAGLD
TsMKK3-2(B)      INIFEKEKRQQILNEMRTLCEASCYPGLVEFQGAFYMPDSGQISIALEYMDGGSLADVIRVKKSIPEPVLAHMLLKVLLGLKYLHEVRHLVHRDLKPANILVNLKGEAKITDFGVSAGLD
AetMKK3-2        INIFEKEKRQQILNEMRTLCEASCYPGLVEFQGAFYMPDSGQISIALEYMDGGSLADVIRVKKSISEPVLAHMLLKVLLGLKYLHEVRHLVHRDLKPANILVNLKGEAKITDFGVSAGLD
TaMKK3-2(5DL)    INIFEKEKRQQILNEMRTLCEASCYPGLVEFQGAFYMPDSGQISIALEYMDGGSLADVIRVKKSIPEPVLAHMLLKVLLGLKYLHEVRHLVHRDLKPANILVNLKGEAKITDFGVSAGLD
```

MKK3 continued

```
TaMKK3(3B)p        ------------------------------------------------------------------------------------------PDARPTCEQLLSHPFIKRYEEAGVDLAAYV
TuMKK3p            GTMAMRATFVGTVTYMLPERIRGDNYLYAADIWSLGLTVLECATGKFPYDFSEGPTNLMLQILDDPSPTPPEDAYSPEFRSFIDDCLQKEPDARSTCEQLLSHPFIKRYEEAGVDLAAYV
HvMKK3-3(5H)       DSITMCATFLGSVTYMSPERIRNENYSYAADIWSLGLTALECATGRYPYDVSGGEADLMLQILEDPSPTPPQHMHSEEFCSFIDACLQKDADARPTCDQLLSHAFIKKYEGPGVDLAEYN
TaMKK3-3(5AL)      DSVTMCATFLGSVTYMSPERIRNENYSYAADIWSLGLTALECATGRYPYDVNGGEADLMLQILEDPSPTPPQHMHSEEFCSFIDACLQKDADARPTCNELLSHSFIKKYEGPGVDLAEYN
TuMKK3-3           DSVTMCATFLGSVTYMSPERIRNENYSYAADIWSLGLTALECATGRYPYDVNGGEADLMLQILEDPSPTPPQHMHSEEFCSFIDACLQKDADARPTCNELLSHSFIKKYEGPGVDLAEYN
TaMKK3-3(5BL)      DSVTMCATFLGSVTYMSPERIRNENYSYAADIWSLGLTALECATGRYPYDVNGGEADLMLQILEDPSPTPPQHMHSEEFCLFIDACLQKDADARPTCDELLSHSFIKKYEGPGVDLAEYN
TsMKK3-3(U)        DSVTMCATFLGSVTYMSPERIRNENYSYAADIWSLGLTALECATGRYPYDVSGGEADLMLQILEDPSPTPPQHMHSEEFCSFIDACLQKDADARPTCDELLSHSFIKKYEGPGVDLAEYN
ScMKK3-3           DSVTMCTTFLGSVTYMSPERIRNENYSYAADIWSLGLTALECATGRYPYDVSGGEADLMLQILEDPSPTPPQHMHSEEFCSFIDACLQKDADARPTCDQLLSHAFIKKYEGPGVDLAEYN
TaMKK3-3(5DL)      DSVTMCATFLGSVTYMSPERIRNENYSYAADIWSLGLTALECATGRYPYDVNGGEADLMLQILEDPSPTPPQHMHSEEFCSFIDACLQKDADARPTCDQLLSHSFIKKYEGPGVDLAEYN
TsMKK3-2(U2)p      NTMAMCATFVGTVTYMSPERIRNENYSYAADIWSLGLTILECATGKFPYNVNEGPANLMLQILDDPSPAPPADGYTPEFCSFINDCLRKDADARPTCEQLLSHAFIKRYEQTGVDLAAYV
BAJ96085.1         NTMAMCATFVGTVTYMSPERIRNENYSYAADIWSLGLTILECATGKFPYNVNEGPANLMLQILDDPSPAPPEDAYTPEFCSFINDCLRKDADARPTCEQLLSHPFIKRYEETRVDLAAYV
AKS50325.1         NTMAMCATFVGTVTYMSPERIRNENYSYAADIWSLGLTILECATGKFPYNVNEGPANLMLQILDDPSPTPPEDAYTPEFCSFINDCLRKDADARPTCEQLLSHPFIKRYEQTGVDLAAYV
TuMKK3-2           NTMAMCATFVGTVTYMSPERIRNENYSYAADIWSLGLTILECATGKFPYNVNEGPANLMLQILDDPSPTPPEDAYTPEFCSFINDCLRKDADARPTCEQLLSHAFIKRYEQTGVDLAAYV
ScMKK3-1           NTMAMCATFVGTVTYMSPERIRNENYSYAADIWSLGLTILECATGKFPYNVNEGPANLMLQILDDPSPAPPADGYTPEFCSFINDCLRKDADTRPTCEQLLSHPFIKKYEPTGVDLAAYV
TsMKK3-2(U1)       NTMAMCATFVGTVTYMSPERIRNENYSYAADIWSLGLTILECATGKFPYNVNEGPANLMLQILDDPSPAPPEDAYTPEFCSFINDCLRKDADARPTCEQLLSHPFIKKYEPTGVDLAAYV
HvMKK3-2(5H)       NTMAMCATFVGTVTYMSPERIRNENYSYAADIWSLGLTILECATGKFPYNVNEGPANLMLQILDDPSPAPPEDAYTPEFCSFINDCLRKDADARPTCEQLLSHPFIKRYEETGVDLAAYV
BAU62004.1         NTMAMCATFVGTVTYMSPERIRNENYSYAADIWSLGLTILECATGKFPYNVNEGPANLMLQILDDPSPAPPEDAYTPEFCSFINDCLRKDADARPTCEQLLSHPFIKRYEETRVDLAAYV
TaMKK3-2(5BL)      NTMAMCATFVGTVTYMSPERIRNENYSYAADIWSLGLTILECATGKFPYNVNEGPANLMLQILDDPSPAPPEDAYTPEFCSFINDCLRKDADARPTCEQLLSHPFIKRYEQTGVDLAAYV
TsMKK3-2(B)        NTMAMCATFVGTVTYMSPERIRNENYSYAADIWSLGLTILECATGKFPYNVNEGPANLMLQILDDPSPAPPEDAYTPEFCSFINDCLRKDADARPTCEQLLSHPFIKRYEQTGVDLAAYV
AetMKK3-2          NTMAMCATFVGTVTYMSPERIRNENYSYAADIWSLGLTILECATGKFPYNVNEGPANLMLQILDDPSPAPPEDAYTPEFCSFIKDCLWKDADARPTCEQLLSHPFIKRYEQTGVDLAAYV
TaMKK3-2(5DL)      NTMAMCATFVGTVTYMSPERIRNENYSYAADIWSLGLTILECATGKFPYNVNEGPANLMLQILDDPSPAPPEDAYTPEFCSFINDCLRKYADARPTCEQLLSHPFIKRYEQTGVDLAAYV
                                                                                                                          *:* **::**** ***:**   **** *
```

MKK3 continued

```
TaMKK3(3B)p        GGVVDPTESLKQIAEMLAVHYYLLFNGSDGSWNHMKTLYMEESSFSFLGNVCVEQSVIFDTLSTIRNKLKGDQPGGGIVHVVEKIHYGANGEREITVRVFGPFIVGNQFLVLGEGLQAEG
TuMKK3p            GGVVNPTERLKQIAEMLVVHYYLLFNGSDGSWHHMETPYMEESSFRFSRNVYVGQSAIFDTLSTIRNKFKGDQPSGGIFHVVEKIHCGADGEREITVRVSGSFIVGNQFLILGEGLQAEG
HvMKK3-3(5H)       KSVHDPSERLSQIAHMLAVHYYLIFDGGDDQWCHMKSFYEQDSTFSFSEETHFGKSDIFDTLSRIREMLKDNSRCKKIGRVMEKVYCRAHGEEGMSVRVSGSFIMGNEFLVCADGFCVEG
TaMKK3-3(5AL)      KSVHDPAERLSQIAHMLAVHYYLIF--------HMKSFYGQDSTFSFSGETHVGKSDIFDTLSRIREMLKGNSRCEKIGRVMEKVYCRAHWEEGMSVRVSGSFIMGNEFLVCADGFCAEG
TuMKK3-3           KSVHDPAERLSQIAHMLAVHYYLIF--------HMKSFYGQDSTFSFSGETHVGKSDIFDTLSRIREMLKGNSRCEKIGRVMEKVYCRAHGEEGMSVRVSGSFIMGNEFLVCADGFCAEG
TaMKK3-3(5BL)      RSVHDPAERLSQIAHMLAVHYYLIFDGGDDQWHHMKSFYGQDSTFSFSGETHVGKSDIFDTLSRIREMLKGNSRCEKIGRVMEKVY-RAHGEEGTSVRVSGSFIMGNEFLVCADGFCAEG
TsMKK3-3(U)        RSVHDPAERLSQIAHMLAVHYYLIFDGGDDQWHHMKSFYGQDSTFSFSGETHVGKSDIFDTLSRIREMLKGNSRCEKIGRVMEKVY-RAHGEEGMSVRVSGSFIMGNEFLVCADGFCAEG
ScMKK3-3           KSVHDPSERLSQIAHMLAVHYYLIFDGGDDQWRHMKSFYGQDSTFSFSGETHVGKSDIFDTLSRIREMLKGNSRCEKIGRVMEKVYCRAHGEEGMSVRVSGSFIMGNEFLVCADGFCAEG
TaMKK3-3(5DL)      KSVHDPAERLSQIAHMLAVHYYLIFDGGDDQWRHMKSFYGQDSTFSFSGETHVGKSDIFDTLSRIREMLKGNSRCEKIGRVMEKVYCRAHGEEGMSVRVSGSFIMGNEFLVCADGFCAEG
TsMKK3-2(U2)p      RDVVNPTERLKQIAEMLAVHYYLLFNGSEGPWNYMKTFYKEESSFSFSGNVYVGQSAIFDTLSNIRKKLKGDRPREKIVHVVEKLHCRAKWGNRNCYPCVWIIHHGQP------------
BAJ96085.1         RGVVNPTERLKQIAEMLAVHYYLLFNGSEGPWNHMKTFYREESSFRYLRRCYIL*-----------------------------------------------------------------
AKS50325.1         RGVVNPTERLKQIAEMLAVHYYLLFNGSEGPWNYMKTFYKEESSFSFSGNVYVGQSAIFDTLSNIRKKLKGDRPREKIVHVVEKLHCRANGETEIAIRVSGSFITGNQFLIFGEGLQAEG
TuMKK3-2           RGVVNPTERLKQIAEMLAVHYYLLFNGSEGPWNYMKTFYKEESSFSFSGNVYVGQSAIFDTLSNIRKKLKGDRPREKIVHVVEKLHCRANGETEIAIRVSGSFITGNQFLIFGEGLQAEG
ScMKK3-1           RGVVNPTERLKQIAEMLAVHYYLLFDGSEGPWNHMKTFYKEESSFSFSGNVYVGQSAIFDTLSNIRKKLKGDRPREKIVHVVEKLHCRANGESEIAIRVSGSFITGNQFLIFGEGLQAEG
TsMKK3-2(U1)       RGVVNPTERLKQIAEMLAVHYYLLFDGSEGPWNHMKTFYKEESSFSFSGNVYVGQSAIFDTLSNIRKKLKGDRPREKIVHVVEKLHCRANGESEIAIRVSGSFITGNQFLIFGEGLQAEG
HvMKK3-2(5H)       RGVVNPTERLKQIAEMLAVHYYLLFDGSEGPWNHMKTFYREESSFSFSGNVYVGQSAIFDTLSNIRKKLKGDRPREKIVHVVEKLHCRANGETEIAIRVSGSFITGNQFLIFGEGLQAEG
BAU62004.1         RGVVNPTERLKQIAEMLAVHYYLLFNGSEGPWNHMKTFYREESSFSFSGNVYVGQSAIFDTLSNIRKKLKGDRPREKIVHVVEKLHCRANGETEIAIRVSGSFITGNQFLIFGEGLQAEG
TaMKK3-2(5BL)      RGVVNPTERLKQIAEMLAVHYYLLFNGSEGPWNHMKTFYREESSFSFSGNVYVGQSAIFDTLSNIRKKLKGDRPREKIVHVVEKLHCRANGETEIAIRVSGSFITGNQFLIFGEGLQAEG
TsMKK3-2(B)        RGVVNPTERLKQIAEMLAVHYYLLFNGSEGPWNHMKTFYREESSFSFSGNVYVGQSAIFDTLSNIRKKLKGDRPREKIVHVVEKLHCRANGETEIAIRVSGSFITGNQFLIFGEGLQAEG
AetMKK3-2          RGVVNPTERLKQIAEMLAVHYYLLFNGSEGPWNHMKTFYREESSFSFSGNVYVGQSAIFDTLSNIRKKLKGDRPREKIVHVVEKLHCRANGETEIAIRVSGSFITGNQFLIFGEGLQAEG
TaMKK3-2(5DL)      RGVVNPTERLKQIAEMLAVHYYLLFNGSEGPWDHMKTFYREESSFSFSGNVYVGQSAIFDTLSNIRKKLKGDRPREKIVHVVEKLHCRANGETEIAIRVSGSFITGNQFLISGEGLQAEG
                    .* :*:* *.***.**.*****:*        :*:: * ::*:* :  .  .
```

MKK3 continued

```
TaMKK3(3B)p        MPSLDELTVDIPRIRVGQFREQFIMQPGISMECYYISRQDLYIIQS*
TuMKK3p            MPGPDELAIDIPSNRAGQFREQFIMQPGISMECYYIPRQDLYIS*--
HvMKK3-3(5H)       MLSIVEISPDILSKQACHFQEDFFMEPGTAMGCYVISRQELHIPVS*
TaMKK3-3(5AL)      MLSMVELSPDILSKQAGHFQEDFFMEPGTAMGCYVISKQELHIGVS*
TuMKK3-3           MLSMVELSPDILSKQAGHFQEDFFMEPGTAMGCYVISKQELHIGVS*
TaMKK3-3(5BL)      MLSIVELSPDILSKQAGHFQEDFFMEPGTAMGCYVISRQELHIGVS*
TsMKK3-3(U)        MLSM-ELSPDILSKQAGHFQEDFFMEPGTAMGCYVISRQELHIGVS*
ScMKK3-3           MLSIVELSPDILSKQAGHFQEDFFMEPGTAMGCYVISRQELHIAVS*
TaMKK3-3(5DL)      MLSIVELSPDILSKQAGHFQEDFFMEPGTAMGCYVISRQELHIGVS*
TsMKK3-2(U2)p      IPDIW-----------------------------------------
BAJ96085.1         ----------------------------------------------
AKS50325.1         MPSLEEIDIDIPSKRVGQFREQFTVHPGTSMGCYYIARQDLYIIQA*
TuMKK3-2           MPSLEEIDIDIPSKRVGQFREQFTVHPGTSMGCYYIAKQDLYIVQS*
ScMKK3-1           MPSLDEIGVDIPSKRVGQFREQFTVLPGTSMGCYYIAKQDLYIVQS*
TsMKK3-2(U1)       MPSLDEIGVDIPSKRVGQFREQFTVLPGTSMGCYYIAKQDLYIVQS*
HvMKK3-2(5H)       MPSLDEIDIDIPSKRVGQFREQFTVHPGTSMGCYYIAKQDLYIIQS*
BAU62004.1         MPSLDEIDIDIPSKRVGQFREQFTVHPGTSMGCYYIAKQDLYIIQS*
TaMKK3-2(5BL)      MPSLDGIDIDIPSKRVGQFREQFTVHPGTSMGCYYIAKQDLYIVQS*
TsMKK3-2(B)        MPSLDGIDIDIPSKRVGQFREQFTVHPGTSMGCYYIAKQDLYIVQS*
AetMKK3-2          MPSLDEINIDIPSKRVGQFREQFTVLPGTSMGCYYIAKQDLYIVQS*
TaMKK3-2(5DL)      MPSLDEINIDIPSKRVGQFREQFTVLPGTSMGCYYIAKQDLYIVQS*
```

MKK4

```
                                                                                                            GxGxxG
AK374401.1      MRPGGPPNARPGLQPQQPGTPGRARRRPDLTLPLPQRDLTSLAVPLPLPPPPSSAPS---SSGSGSGSGGASSMPMPMSMTPPNSAGSAPPAPPPLDELERVRRVGSGAGGTVWLVRHAP
TsMKK4(U)       MRPGGPPNARPGLQQQQPGTPGRARRRPDLTLPLPQRDLTSLAVPLPLPPPPSSAPSSTSSSG--SGSGGASSMPMPMSMTPPNSAGSAPPAPPPLGELERVRRVG-GAGGTVWLVRHAP
HvMKK4(6H)      MRPGGPPNARPGLQPQQPGTPGRARRRPDLTLPLPQRDLTSLAVPLPLPPPPSSAPS---SSGSGSGSGGASSMPMPMSMTPPNSAGSAPPAPPPLDELERVRRVGSGAGGTVWLVRHAP
TaMKK4(6AL)     MRPGGPPNARPGLQQQQPGTPGRARRRPDLTLPLPQRDLTSLAVPLPLPPPPSSAPSSTSSSGSGSGSGGASSMPMPMSMTPPNSAGSAPPAPPPLGELERVRRVGSGAGGTVWLVRHAP
TaMKK4(6DL)     MRPGGPPNARPGLQQQQPGTPGRARRRPDLTLPLPQRDLTSLAVPLPLPPPPSSAPSSTSSS----GSGGASSMPMPMSMTPPNSAGSTPPAPPPLGELERVRRVGSGAGGTVWLVRHAP
ScMKK4          -------------------------------------------------------------------------MPMPMSMTPPNSAGSAPPAPPPLGELERVRRVGSGAGGTVWLVRHAP
TaMKK4(6BL)     MRPGGPPNARPGLQQQQPGTPGRARRRPDLTLPLPQRDLTSLAVPLPLPPPPSSAPSSTSSSGSGSGSGGASSMPMPMSMTPPNSAGSAPPAPPPLGELERVRRVGSGAGGTVWLVRHAP
TuMKK4          -------------------------------------XXXX-----------XXXXXXXXXXXXGGRSRGRSPSCARRS-TRPSCAATAC----------TSRPASCRSCSSTWTAGRWT
AetMKK4         -------------------------------------XXX------------------------------------------XXXXXX------------XXXXXXXXXXXXXXXXXXXX

                                                                                                                   DΨK
AK374401.1      TGRAYALKVLYGHHDEAVRRQITREIAILRTAEHPSIVRCHGMYEQAGELQILLEYMDGGSLDGRRIASEVFLADVARQVLSGIAYLHRRHIVHRDIKPSNLLIDSGRRVKIADFGVGRI
TsMKK4(U)       TGRAYALKVLYGHHDEAVRRQITREIAILRTAEHPSIVRCHGMYEQAGELQILLEYMDGGSLDGRRIASEAFLADVARQVLSGIAYLHRRHIVHRDIKPSNLLIDSG-RVKIADFGVGRI
HvMKK4(6H)      TGRAYALKVLYGHHDEAVRRQITREIAILRTAEHPSIVRCHGMYEQAGELQILLEYMDGGSLDGRRIASEVFLADVARQVLSGIAYLHRRHIVHRDIKPSNLLIDSGRRVKIADFGVGRI
TaMKK4(6AL)     TGRAYALKVLYGHHDEAVRRQITREIAILRTAEHPSIVRCHGMYEQAGELQILLEYMDGGSLDGRRIASESFLADVARQVLSGIAYLHRCHIVHRDIKPSNLLIDCGRRVKIADFGVGRI
TaMKK4(6DL)     TGRAYALKVLYGHHDEAVRRQITREIAILRTAEHPSIVRCHGMYEQAGELQILLEYMDGGSLDGRRIASESFLADVARQVLSGIAYLHRRHIVHRDIKPSNLLIDCARRVKIADFGVGRI
ScMKK4          TGRAYALKVLYGHHDEAVRRQITREIAILRTAEHPSIVRCHGMYEQAGELQILLEYMDGGSLDGRRIASEAFLADVARQVLSGIAYLHRRHIVHRDIKPSNLLIDAARRVKIADFGVGRI
TaMKK4(6BL)     TGRAYALKVLYGHHDEAVRRQITREIAILRTAEHPSIVRCHGMYEQAGELQILLEYMDGGSLDGRRIASEAFLADVARQVLSGIAYLHRRHIVHRDIKPSNLLIDSGRRVKIADFGVGRI
TuMKK4          AGXXXXXXXXX---------------------------------XXXXXXXXXXXXXXXXXXXXXXXXXXXXXXDVARQMLSGIAYLHRRHIVHRDIKPSNLLIDCGRRVKIADFGVGRI
AetMKK4         XXXXXXXXXXX---------------------------------XXXXXXXXXXXXXXXXXXXXXXXXXXSFPPAGARRVLSRIAYLHRRHIVHRDIKPSNLLIDCARRVKIADFGVGRI
                                                                             **::** ****** ***************.. ************

AK374401.1      LNQTMDPCNSSVGTIAYMSPERINTDLNDGGPAHRVPGAEEL-----HQLLPPEEPGEAAVGGAAA-AAPVHRLAA-----AAAAAGPRRPAVLTARHNK-----SSWDGSLDGRWPAAR
TsMKK4(U)       LNQTMDPCNSSVGTIAYMSPERINTDLNDGNYNGYAGDIWSFGLSILEFYLGRFPLGE-NLGKQGDWAALMCAICYSESPAAPPTASPELRSFISCCLQKNPAKRPSAAQLLQHRFIASP
HvMKK4(6H)      LNQTMDPCNSSVGTIAYMSPERINTDLNDGNYNGYAGDIWSFGLSILEFYLGRFPLGE-NLGKQGDWAALMCAICYSESPAAPPTASPELRSFISCCLQKNPAKRPSAAQLLQHRFIASP
TaMKK4(6AL)     LNQTMDPCNSSVGTIAYMSPERINTDLNDGNYNGYAGDIWSFGLSILEFYLGRFPLGE-NLGKQGDWAALMCAICYSESPAAPPTASPELRSFISCCLQKNPAKRPSAAQLLQHRFIASP
TaMKK4(6DL)     LNQTMDPCNSSVGTIAYMSPERINTDLNDGNYNGYAGDIWSFGLSILEFYLGRFPLGE-NLGKQGDWAALMCAICYSESPAAPPTASPELRSFISCCLQKNPAKRPSAAQLLQHRFIASP
ScMKK4          LNQTMDPCNSSVGTIAYMSPERINTDLNDGNYNGYAGDIWSFGLSILEFYLGRFPLGE-NLGKQGDWAALMCAICYSESPAAPPTASPELRSFISCCLQKNPAKRPSAAQLLQHRFIASP
TaMKK4(6BL)     LNQTMDPCNSSVGTIAYMSPERINTDLNDGNYNGYAGDIWSFGLSILEFYLGRFPLGE-NLGKQGDWAALMCAICYSESPAAPPTASPELRSFISCCLQKNPAKRPSAAQLLQHRFIASP
TuMKK4          LNQTMDPCNSSVGTIAYMSPERINTDLNDGNYNGYAGDIWSFGLSILEFYLGRFPLGE-NLGKQGDWAALMCAICYSESPAAPPPASPELRSFISCCLQKHPAKRPSAAQLLQHRFIASP
AetMKK4         LNQTMDPCNSSVGTIAYMSPERINTDLNDGNYNGYAGDIWSFGLSILEFYLGRFPLGE-NLGKQGDWAALMCAICYSESXXXXXXXXXXXRSFISCCLQKNPAKRPSAAQLLQHRFIASP
                ******************************.     . .  .:     .  *     **   :*  .  ** :  :.                :.::.  :*      *     *: *: *:
```

MKK4 continued

```
AK374401.1        RGIYE--------------------ER*
TsMKK4(U)         PQQQQPQVLAAPPC*-------------
HvMKK4(6H)        PQ-QQPQVLAAPPCQPPATINQVGMERX
TaMKK4(6AL)       PP-QQPQALAAPPC*-------------
TaMKK4(6DL)       PQ-QQPQALAAPPC*-------------
ScMKK4            PQ-QQPQVLAAPPC*-------------
TaMKK4(6BL)       PQ-QQPQVLAAPPC*-------------
TuMKK4            P-PQQPQALAAPPC*-------------
AetMKK4           PPPQQPQALAAPPC*-------------
                      :
```

MKK5

```
                                                                                                                                  GxGxxG
TuMKK5             ------------XXXXX---------XXXXXXXXXXXXXXXXXXXXXXXXXXXXXXXXXXXXXXXXXXXXXXXXXXXXXXXXXXXXXXXXXXXXXXXXXXXXXXXXXXXXXXXXXXXXXX
AetMKK5            --------------------------------------------------------------------------------XXXXXXXXXXX--XXXXXXXXXXXXXXXXXXXXXXXXXXX
TsMKK5(U1)         MRPGGPPNARPGLQQQQPGTPGRARRRPDLTLPLPQRDLT--SLAVPLPLPPPPSSAPSSTSSSGSGSGGASSMPMPMSMTPPNSAGSAPPAPPPLGELERVRRVG-GAGGTVWLVRHAP
TsMKK5(U2)         MRPAGGS--LP--A--QPGTPGRPRRRPDLTLPMPQRPDVSSSLAVPLPLPPPTAAPLPGP----------------------APPAAAAPPPPPLHELERVRRVG-GAGGTVWLVRHAP
TaMKK5(7AS)        MRPAGGS--LP--A--QPGTPGRPRRRPDLTLPMPQRPDVSSSLAVPLPLPPPTAAP-TG-------AGPAQ---------PLVAPPAAAPPPPPLHELERVRRVGSGAGGTVWMVRHRA
TaMKK5(7BS)        MRPAGIS--LP--A--QPGTPGRPRRRPDLTLPMPQRPDVSSSLAVPLPLPPPTAAP-----------GAAQ---------PLVAPPAATPPPPPLHELERVRRVGSGAGGTVWMVRHRA
TaMKK5(7DS)        MRPAGGGSSLP--AQPGPGTPGRPRRRPDLTLPMPQRPDVSSSLAVPLPLPPPTAAP--G-------AGPAQ---------PLVAPPA---PPPPLHELERVRRVGSGAGGTVWMVRHRA
ScMKK5p            -------------------------------------------------PPAPPAAAPLPG------AGPAQ---------PLAAP-----PPPPLHELERVRRVGSGAGGTVWMVQHRA
HvMKK5(7H)         MRPAGTSSSLP--AQP--GTPGRPRRRPDLTLPMPQRPDVSSSLAVPLPLPPP-------------------------------------------------------AGGTVWMVRHRP
AK370268           MRPAGTSSSLP--AQP--GTPGRPRRRPDLTLPMPQRPDVSSSLAVPLPLPPPTTAPPAGA------TGPAQ---------PLAAP-----PPPPLHELERVRRVGSGAGGTVWMVRHRP

                                                                                                                   DΨK
TuMKK5             XXXXXXXXXXXXXXXXXXXXXXXXXXXXXXXXXXXXXXXXXXXXXXXXXXXXXXXXXXXXXXXXXXXPDEPFLAHVARQVLSGIAYLHRRHIVHRDIKPSNLLIDSARRVKIADFGVGRI
AetMKK5            XXXXXXXXXXXXXXXXXXXXXXXXXXXXXXXXXXXXXXXXXXXXXXXXXXXXXXXXXXXXXXXXRRIADEPFLAHVARQVLSGIAYLHRRHIVHRDIKPSNLLIDSSRRVKIADFGVGRI
TsMKK5(U1)         TGRAYALKVLYGHHDEAVRRQITREIAILRTAEHPSIVRCHGMYEQAGELQILLEYMDGGSLDGRRIASEAFLADVARQVLSGIAYLHRRHIVHRDIKPSNLLIDSG-RVKIADFGVGRI
TsMKK5(U2)         TGRAYALKVLYGHHDEAVRRQITREIAILRTAEHPSIVRCHGMYEQAGELQILLEYMDGGSLDGRRIASEAFLADVARQVLSGIAYLHRRHIVHRDIKPSNLLIDSG-RVKIADFGVGRI
TaMKK5(7AS)        TGRCYALKVLYGNHDDAVRRQIAREIAILRTAEHPAVVRCHGMYERGGELQILLEYMDGGSLDGRRIADEPFLAHVARQVLSGIAYLHRRHIVHRDIKPSNLLIDSARRVKIADFGVGRI
TaMKK5(7BS)        TGRCYALKVLYGNHDDAVRRQIAREIAILRTAEHPAVVRCHGMYERGGELQILLEYMDGGSLDGRRIADEPFLAHVARQVLSGIAYLHRRHIVHRDIKPSNLLIDSARRVKIADFGVGRI
TaMKK5(7DS)        TGRCYALKVLYGNHDDTVRRQIAREIAILRTAEHPAVVRCHGMYERGGELQILLEYMDGGSLDGRRIADEPFLAHVARQVLSGIAYLHRRHIVHRDIKPSNLLIDSSRRVKIADFGVGRI
ScMKK5p            TARRYALKVLYGNHDDAVRRQIAREIAILRTAEHPAVVRCHGMYERGGELQILLEYMDGGSLDGRRIADEPFLAHVARQVLSGIAYLHRRHIVHRDIKPSNLLIDSARRVKIADFGVGRI
HvMKK5(7H)         TARCYALKVLYGNHDDAVRRQIAREIAILRTAEHPAVVRCHGMYERGGELQILLEYMDGGSLDGRRIADEPFLAHVARQVLSGIAYLHRRHIVHRDIKPSNLLIDSARRVKIADFGVGRI
AK370268           TARCYALKVLYGNHDDAVRRQIAREIAILRTAEHPAVVRCHGMYERGGELQILLEYMDGGSLDGRRIADEPFLAHVARQVLSGIAYLHRRHIVHRDIKPSNLLIDSARRVKIADFGVGRI
                                                                                   .* ***.*******************************. ************

TuMKK5             LNQTMDPCNSSVGTIAYMSPERINTDINDGAYDGYAGDIWSFGLSILEFYLGRFPFGENLGXXXXXXXXXXXXXXXXX---------------------------------------------XXXXXXSS*
AetMKK5            LNQTMDPCNSSVGTIAYMSPERINTDINDGAYDGYAGDIWSFGLSILEFYLGRFPFGENLGRQGDWAALMVAICYSDPPEPSPATASPEFRGFIACCLQKNPANRLSAAQLLQHPFVALPQPQPLAAPPSS*
TsMKK5(U1)         LNQTMDPCNSSVGTIAYMSPERINTDINDGAYDGYAGDIWSFGLSILEFYLGRFPFGENLGRQGDWAALMVAICYSDPPEPSPATASPEFRGFIACCLQKNPANRLSAAQLLQHPFVALPQPQPLAAPPS*-
TsMKK5(U2)         LNQTMDPCNSSVGTIAYMSPERINTDINDGAYDGYAGDIWSFGLSILEFYLGRFPFGENLGRQGDWAALMVAICYSDPPEPSPATASPEFRGFIACCLQKNPAKRLSAAQLLQHPV-ALPQPQPLAAPPSS*
TaMKK5(7AS)        LNQTMDPCNSSVGTIAYMSPERINTDINDGAYDGYAGDIWSFGLSILEFYLGRFPFGENLGRQGDWAALMVAICYSDPPEPSPATASPEFRGFIACCLQKNPANRLSAAQLLQHPFVALPQPQPLAAPPSS*
TaMKK5(7BS)        LNQTMDPCNSSVGTIAYMSPERINTDINDGAYDGYAGDIWSFGLSILEFYLGRFPFGENLGRQGDWAALMVAICYSDPPEPSPATASPEFRGFIACCLQKNPANRLSAAQLLQHPFVALPQPQPLAAPPS*-
TaMKK5(7DS)        LNQTMDPCNSSVGTIAYMSPERINTDINDGAYDGYAGDIWSFGLSILEFYLGRFPFGENLGRQGDWAALMVAICYSDPPEPSPATASPEFRGFIACCLQKNPANRLSAAQLLQHPFVALPQPQPLAAPPSS*
ScMKK5p            LNQTMDPCNSSVGTIAYMSPERINTDINDGAYDGYAGDIWSFGLSILEFYLGRFPFGENLGRQGDWAALMVAICYSDPPEPSPATASPEFRGFIACCLQKNPAKRLSAAQLLQHPFVALPQPQPLAAPPS*-
HvMKK5(7H)         LNQTMDPCNSSVGTIAYMSPERINTDINDGAYDGYAGDIWSFGLSILEFYLGRFPFGENLGRQGDWAALMVAICYSDPPEPSPATASPEFRGFIACCLQKNPAKRLSAAQLLQHPFVALPQPQPLAAPPS*-
AK370268           LNQTMDPCNSSVGTIAYMSPERINTDINDGAYDGYAGDIWSFGLSILEFYLGRFPFGENLGRQGDWAALMVAICYSDPPEPSPATASPEFRGFIACCLQKNPAKRLSAAQLLQHPFVALPQPQPLAAPPS*-
                   *************************************************************                       ::
```

MKK6

```
                                                                                              GxGxxG
TuMKK6          MRGKKPLKEL--SVPAQETPVDKFLTASGTFKDGELRLNQRGLQLISEENGDEHQSTKMKVEDVQLSMDDLEMIQVIGKGSGGVVQLVRHKWVGTFYALKGIQMNIQESVRKQIVQELKI
AK333543.1      MRGKKPLKELTLSVPAQETPVDKFLTASGTFKDGELRLNQRGLQLISEENGDEHQSTKMKVEDVQLSMDDLEMIQVIGKGSGGVVQLVRHKWVGTFYALKGIQMNIQESVRKQIVQELKI
TaMKK6(4BL)     MRGKKPLKELTLSVPAQETPVDKFLTASGTFKDGELRLNQRGLQLISEENGDEHQSTKMKVEDVQLSMDDLEMIQVIGKGSGGVVQLVRHKWVGTFYALKGIQMNIQESVRKQIVQELKI
ScMKK6          MRGKKPLKELTLSVPAQETPVDKFLTASGTFKDGELRLNQRGLQLISEENGDEHQSTKMKVEDVQLSMDDLEMIQVIGKGSGGVVQLVRHKWVGTFYALKGIQMNIQESVRKQIVQELKI
AK250233.1      MRGKKPLKELTLSVPAQETPVDKFLTASGTFKDGELRLNQRGLQLISEENGDEHQSTKMKVEDVQLSMDDLEMIQVIGKGSGGVVQLVRHKWVGTFYALKGIQMNIQESVRKQIVQELKI
HvMKK6(4H)      MRGKKPLKELTLSVPAQETPVDKFLTASGTFKDGELRLNQRGLQLISEENGDEHQSTKMKVEDVQLSMDDLEMIQVIGKGSGGVVQLVRHKWVGTFYALKGIQMNIQESVRKQIVQELKI
TsMKK6(R)       MRGKKPLKELTLSVPAQETPVDKFLTASGTFKDGELRLNQRGLQLISEENGDEHQSTKMKVEDVQLSMDDLEMIQVIGKGSGGVVQLVRHKWVGTFYALKGIQMNIQESVRKQIVQELKI
TaMKK6(4AL)     MRGKKPLKELTLSVPAQETPVDKFLTASGTFKDGELRLNQRGLQLISEENGDEHQSTKMKVEDVQLSMDDLEMIQVIGKGSGGVVQLVRHKWVGTFYALKGIQMNIQESVRKQIVQELKI
TaMKK6(4DL)     MRGKKPLKELTLSVPAQETPVDKFLTASGTFKDGELRLNQRGLQLISEENGDEHQSTKMKVEDVQLSMDDLEMIQVIGKGSGGVVQLVRHKWVGTFYALKGIQMNIQESVRKQIVQELKI
AetMKK6         MRGKKPLKELTLSVPAQETPVDKFLTASGTFKDGELRLNQRGLQLISEENGDEHQSTKMKVEDVQLSMDDLEMIQVIGKGSGGVVQLVRHKWVGTFYALKGIQMNIQESVRKQIVQELKI
                **********  ************************************************************************************************************

                                                                                      DΨK
TuMKK6          NQATQSPHIVLCHQSFYHNGVIYLVLEYMDRGSLADIIKQVKTILEPYLAVLCKQVLEGLLYLHHERHVIHRDIKPSNLLVNHKGEVKITDFGVSAVLASSIGQRDTFVGTYNYMAPERI
AK333543.1      NQATQSPHIVLCHQSFYHNGVIYLVLEYMDRGSLADIIKQVKTILEPYLAVLCKQVLEGLLYLHHERHVIHRDIKPSNLLVNHKGEVKITDFGVSAVLASSIGQRDTFVGTYNYMAPERI
TaMKK6(4BL)     NQATQSPHIVLCHQSFYHNGVIYLVLEYMDRGSLADIIKQVKTILEPYLAVLCKQVLEGLLYLHHERHVIHRDIKPSNLLVNHKGEVKITDFGVSAVLASSIGQRDTFVGTYNYMAPERI
ScMKK6          NQATQSPHIVLCHQSFYHNGVIYLVLEYMDRGSLADIIKQVKTILEPYLAVLCKQVLEGLLYLHHERHVIHRDIKPSNLLVNHKGEVKITDFGVSAVLASSIGQRDTFVGTYNYMAPERI
AK250233.1      NQATQSPHIVLCHQSFYHNGVIYLVLEYMDRGSLADIIKQVKTILEPYLAVLCKQVLEGLLYLHHERHVIHRDIKPSNLLVNHKGEVKITDFGVSAVLASSIGQRDTFVGTYNYMAPERI
HvMKK6(4H)      NQATQSPHIVLCHQSFYHNGVIYLVLEYMDRGSLADIIKQVKTILEPYLAVLCKQVLEGLLYLHHERHVIHRDIKPSNLLVNHKGEVKITDFGVSAVLASSIGQRDTFVGTYNYMAPERI
TsMKK6(R)       NQATQSPHIVLCHQSFYHNGVIYLVLEYMDRGSLADIIKQVKTILEPYLAVLCKQVLEGLLYLHHERHVIHRDIKPSNLLVNHKGEVKITDFGVSAVLASSIGQRDTFVGTYNYMAPERI
TaMKK6(4AL)     NQATQSPHIVLCHQSFYHNGVIYLVLEYMDRGSLADIIKQVKTILEPYLAVLCKQVLEGLLYLHHERHVIHRDIKPSNLLVNHKGEVKITDFGVSAVLASSIGQRDTFVGTYNYMAPERI
TaMKK6(4DL)     NQATQSPHIVLCHQSFYHNGVIYLVLEYMDRGSLADIIKQVKTILEPYLAVLCKQVLEGLLYLHHERHVIHRDIKPSNLLVNHKGEVKITDFGVSAVLASSIGQRDTFVGTYNYMAPERI
AetMKK6         NQATQSPHIVLCHQSFYHNGVIYLVLEYMDRGSLADIIKQVKTILEPYLAVLCKQVLEGLLYLHHERHVIHRDIKPSNLLVNHKGEVKITDFGVSAVLASSIGQRDTFVGTYNYMAPERI
                ************************************************************************************************************************

TuMKK6          SGSSYDYKSDVWSLGLVILECAIGRFPYTPPEGEGWLSFYELLEAIVDQPPPSAPADQFSPEFCSFISSCIQKDPAERMSASELLNHAFIKKFEDKDLDLRILVESLEQPMNVPE*
AK333543.1      SGSSYDYKSDIWSLGLVILECAIGRFPYTPPEGEGWLSFYELLEAIVDQPPPSAPADQFSPEFCSFISSCIQKDPAERMSASELLNHAFIKKFEDKDLDLRILVESLEQPMNVPE*
TaMKK6(4BL)     SGSSYDYKSDIWSLGLVILECAIGRFPYTPPEGEGWLSFYELLEAIVDQPPPSAPADQFSPEFCSFISSCIQKDPAERMSASELLNHAFIKKFEDKDLDLRILVESLEQPMNVPE*
ScMKK6          SGSSYDYKSDVWSLGLVILECAIGRFPYTPPEGEGWLSFYELLEAIVDQPPPSAPADQFSPEFCSFISSCIQKDPAERKSASELLNHAFIKKFEDKDLDLRILVESLEQPMNVPE*
AK250233.1      SGSSYDYKSDVWSLGLVILECAIGRFPYTPPEGEGWLSFYELLEAIVDQPPPSAPADQFSPEFCSFISSCIQKDPAERKSASELLNHAFIKKFEDKDLDLRILVESLEQPMNVPE*
HvMKK6(4H)      SGSSYDYKSDVWSLGLVILECAIGRFPYTPPEGEGWLSFYELLEAIVDQPPPSAPADQFSPEFCSFISSCIQKDPAERKSASELLNHAFIKKFEDKDLDLRILVESLEQPMNVPE*
TsMKK6(R)       SGSSYDYKSDVWSLGLVILECAIGRFPYTPPEGEGWLSFYELLEAIVDQPPPSAPADQFSPEFCSFISSCIQKDPAERKSASELLNHAFIKKFEDKDLDLRILVESLEQPMNVPE*
TaMKK6(4AL)     SGSSYDYKSDVWSLGLVILECAIGRFPYTPPEGEGWLSFYELLEAIVDQPPPSAPADQFSPEFCSFISSCIQKDPAERMSASELLNHAFIKKFEDKDLDLRILVESLEQPMNVPE*
TaMKK6(4DL)     SGSSYDYKSDVWSLGLVILECAIGRFPYTPPEGEGWLSFYELLEAIVDQPPPSAPADQFSPEFCSFISSCIQKDPAERMSASELLNHAFIKKFEDKDLDLRILVESLEQPMNVPE*
AetMKK6         SGSSYDYKSDVWSLGLVILECAIGRFPYTPPEGEGWLSFYELLEAIVDQPPPSAPADQFSPEFCSFISSCIQKDPAERMSASELLNHAFIKKFEDKDLDLRILVESLEQPMNVPE*
                **********:******************************************************************* *************************************
```

MKK10

```
                                                                                                GxGxxG
TuMKK10p                MAAARXXXX---------------------------------------------------------------------XXXXXXXX----------XXXXXX--XXXXXXXXXXX-X---
AetMKK10p               MAAARERRLPQLHLTLDAPTWAFRC--------------PAPAPVTAATPSTSAAPPRRGVRRKDIHXXXXXXXXXXXXXXXXXXXXXXXXXXXXXXXXXX--XXX--XXXXXX-X---
HvMKK10(1H)p            ------------------------------------------------------------------------------------------------------------------------
ScMKK10p                ------------------------------------------------------------------------GEGASGVVTKVRLRGGDRRPRVALKVAHYPDDTDAGREQDEVLRRACG
BAJ94499.1              MALVRQRR-QLPHLTLPLDHFALRA------------P-PPPPPA--A------APCEGLPRLSDYERISQLGQGNGGTVFKARHR--RTSQHFALKLFAAGDGDPSAAREAEILML---
HvMKK10-2(4H)           MALVRQRR-QLPHLTLPLDHFALRA------------P-PPPPPA--A------APCEGLPRLSDYERISQLGQGNGGTVFKARHR--RTSQHFALKLFAAGDGDPSAAREAEILML---
TaMKK10-2(4AS)          MALVRQRR-QLPHLTLPLDHFALRA------------P-PPPLPP-AV---APCAPAEGLARLSDYERISQLGQGNGGTVFKARHR--RTAQHFALKLFAAGDGDPSAAREAEILML---
TsMKK10p                MALVRQRR-QLPHLTLPLDHFALAR------------P-P-PPPP-AV---APC---EGLARLSDYERISQLGQGNGGTVFKARHR--RTAQQVALKLFAAGDGDPSAAREAEILML---
TaMKK10-2(4BL)          MALVRQRR-QLPHLTLPLDHFALRA------------P-PP-PPP-AV---APC---EGLARLSDYERISQLGQGNGGTVFKARHR--RTAQQVALKLFAAGDGDPSAAREAEILML---
TaMKK10-2(4DL)          MALVRQRR-QLPHLTLPLDHFALRA------------P-PPPPPP-AV---APC---EGLARLSDYERISQLGQGNGGTVFKARHR--RTAQQVALKLFAAGDGDPSAAREAEILML---
TuMKK10-1/3             MAAARERRLPQXXXXXXXXXXXXXXXXXXXXXXXXXXXXX--------X--XXXXXXXXXXXX--------XXXXXXXXXXXXXXX--XX-XXXXXXXXXXX--X----XXXXXX-X---
TaMKK10-1/3(4BSc1)      MASSRNQGR--TKPSLPHRPTAALTSASLAGAPTPRRPRPPPRRRCPASSASLTSTSSPSW----------AAGTAAPSTRSAIGR--RARALYALKVQHYG--DPAAAAKADVL-S---
TaMKK10-1/3(4BSc4)      MALIREKRLPQLHLSLPVPPRAAAQELGV----LGRRPNPAVAPAPTATKASTPSGLSSQFRLADFEKLAVLGRGNGGTVYKVRHR--ETCALYALKVQHYG--DPAAANEADVL-G---
TaMKK10-1/3(4BSc5)      MALIREKRLPQLHLSLPVPPRAVAQELGV----LGRRPNPAVAPAPTATKASTPSALSSQFRLADFEKLAVLGRGNGGTVYKVRHR--ETSALYALKVQHYG--DPAAAAEADVL-S---
TaMKK10-1/3(4DSc2)      MALIREKRLPQLHLSLPVPPRAAPQELGV----LGRRPNPAVPPAPTATKASTPSALSSQFRLADFEKLAVLGRGNGGTVYKVRHR--ETHALYALKVQHYG--DPAAAAEADVL-S---
TaMKK10-1/3(4BSc3)      -MALREKRLPQLHLALPVPSRAAAQELGV----LGRRPNP------TATKASTPSALSSQFRLADFDKLAVLGRGNGATVYKVRHR--ETSALYALKVQHYG--DPAAAAEANIL-S---
HvMKK10-1/3(U)          MAMIREKRLPQLHLALPVPSRAAAQDLAV----IARRTNP------AATKASTPSALSSQFRLADFDKLAVLGRGNGGTVYKARHR--ETCALYALKVQHYG--DPAAAAEADVL-S---
TaMKK10-1/3(4AL)        MALIREKRLPQLHLALPVPSRAAAQELGV----LARRPNP------TATKASTPSALSSQLRLADFDKLAVLGRGNGGTVYKVRHR--ETCALYALKVQHYG--DPAAAAEADVL-G---
TaMKK10-1/3(4BSc2)      MALIREKRLPQLHLALPVPSRAAAQELGV----LGRRPNP------TATKASTPSALSSQFRLADFDKLAVLGRGNGGTVYKVRHR--ETCALYALKVQHYG--DPAAAAEADVL-S---
TaMKK10-1/3(4DSc1)      MALIREKRLPQLHLALPVPSRAAAQELGV----LGRRPNP------TATKASTPSALSSQFRLADFDKLAVLGRGNGGTVYKVRHR--ETCALYALKVQHYG--DPAAAAEADVL-S---
AetMKK10-5              MAAARERRLPQLHLTLDAPTWAFRC--------------PAPAPVTAATPSTXXXXXXXXXXXXXXXXXXXXXXXXXXXXXXXXXX--XXXXX---XXXX-X--X----XXXXXX-----
HvMKK10-5(4H)           MAAARERRLPQLHLTLDAPAWAFRC--------------PAPAPVTAATPSTSAARPDGEFRQSDFERLSVLGRGNGGIVHKVSHR--RTSALYALKIIHRG--HPGADEEVDVV-R---
TaMKK10-5(4DS)          MAAARERRLPQLHLTLDAPTWAFRC--------------PAPAPVTAATPSTSAARPDGEFRLSDFDMLKVLGRGNGGTVHKASHR--RTSALYALKIIHRG--HPGADEEVDVV-R---
TaMKK10-5(4AL)          MAAARERRLPQLHLTLDAPTWAFRC--------------PAPAPVTAATPSTSAARPDGEFRQSDFDMLKVLGRGNGGTVHKVSHR--RTSALYALKVIHRG--HPGAGDEVDVV-R---
TaMKK10-5(4BS)          MAAARERRLPQLHLTLDAPTWAFRC--------------PAPAPVTAATPSTSAARPDGEFRLSDFDMLKVLGRGNGGTVHKVSHR--RTSALYALKIIHRG--HPGAGEEVDVV-R---
HvMKK10-4(4H)           MAAARERRLPHLHLTLDAPTWAFRC--------------PAPASVTVATLSTSAPRPDGEFRLIDFDRLSVLGRGNGGIVHKVSHR--RTSALYALKIIHRG--HPGADEEVDVV-R---
TaMKK10-4(3B)           MAAARERRLPQLHLTLDAPTWAFRC--------------PAPAPVTAATPSTSAARPDGEFRLSDFDRLSVLGRGNGGTVHKVTHR--RTSALYALKIIHRG--HPGADEEVEVV-R---
TaMKK10-4(4BS)          MAAARERRLPQLHLTLDAPTWAFRC--------------PAPAPVTAATPSTSAARPDGEFRLSDFDRLSVLGRGNGGTVHKVSHR--RTSALYALKIIHRG--HPGADEEVDVV-R---
TaMKK10-4(4DS)          MAAARERRLPQLHLTLDAPTWAFRC--------------PAPAPVTAATPSTSAARPDGEFRQSDFDRLSVLGRGNGGTVHKVSHR--RTSALYALKIIHRG--HPGVDEEVEVV-R---
TaMKK10-4(4ALc2)        MAAARERRLPQLHLKLDAPTWAFRC--------------PAPAPVTAATPSTLAARPDGEFRLIDFYRLSVLGRGNGGTVHKVSHR--RTSALYALKIIHRG--HPGADEEVEVV-R---
TaMKK10-4(4ALc1)        MAAARERRLPQLHLTLDASTWAFRC--------------PASATVAAATPSTSAARPDGEFRLIDFDRLSVLGRGNGGTVHKVSHR--RTSALYALKIIHRG--HPGADEEVEVV-R---
TuMKK10-4               MAAARERRLPQLHLTLDAPTWAFRC--------------PASAPVTAATPSTSAARPDGEFRLIDFDRLSVLGRGNGGTVHKVSHR--RTSALYALKIIHRG--HPGADEEVEVV-R---
```

MKK10 continued

```
                                                                                                      DΨK
TuMKK10p               -XXXXXXXXXXXXXXXXXXX-XXXXXXXXXXXXXXXXXX-XXX-XXXXXXXXXXXXXXXXXXXXXXXXXXXXXXXXXXXXXXXXXXXXXXXXXXXXXXXXXXXXXXXX----XXXXXXX
AetMKK10p              -XXXXXXXXXXXXXXXX-XX-XXXXXXXXXXXXXXXXXX-X-X--XXXXXXXXXXXXXXXXXXXXXXXXXXXXXXXXXXXXXXXXXXXXXKIAAFGIVEVVSRAGKYHA----AYEGTAA
HvMKK10(1H)p           ---------------XXXX--XXXXXXXXXXXXXXXXXXXXXXXXXXXXXPAVAEVAARCALGLAHLHARGVAHLDLKPDNLLAGARGDVKIADFGVSRIFCRDGQRRPPRVSIAVGTTA
ScMKK10p               AGPPSPYVVRCHAVLRGAG--GEPACLLELMDAGSLHDVLRRRAGRGLPEPALAEVAARCALALAHLHARGVAHLDLKPDNLLASARGDVKIADFGVSRIFSRDGQRRPPRVSVAVGTTA
BAJ94499.1             -ASGAPHVVRLHAVIPSPAAAQPAALALELVSGGSLAGLLRAL-GRPMGERPIAAVARQALLGLEALHALRVVHRDLKPANLLVGPGGEVKIADFGAGKVLRRRLDPCA----SYVGTAA
HvMKK10-2(4H)          -ASGAPHVVRLHAVIPSPAAAQPAALALELVSGGSLAGLLRAL-GRPMGERPIAAVARQALLGLEALHALRVVHRDLKPANLLVGPGGEVKIADFGAGKVLRRRLDPCA----SYVGTAA
TaMKK10-2(4AS)         -ASGAPHVVRLHAVIPSPAAEQPAALALELVSGGSLAGLLRAL-GRPMGERPIAAVARQALLGLAALHALRVVHRDLKPANLLVGAGGEVKIADFGAGKVLRRRLDPCA----SYVGTAA
TsMKK10p               -ASGAPHVVRLHAVIPSPAAEQPAALALELVSGGSLAGLLRAM-GRPMGERPIAAVARQALLGLAALHALRVVHRDLKPANLLVGAGGEVKIADFGAGKVLR------------------
TaMKK10-2(4BL)         -ASGAPHVVRLHAVIPSPAAEQPAALALELVSGGSLAGLLRAL-GRPMGERPIAAVARQALLGLAALHALRVVHRDLKPANLLVGAGGEVKIADFGAGKVLRRRLDPCA----SYVGTAA
TaMKK10-2(4DL)         -ASGAPHVVRLHAVIPSPAAEQPAALALELVSGGSLAGLLRAL-GRPMGERPIAAVARQALLGLAALHALRVVHRDLKPANLLVGAGGEVKIADFGAGKVLRRRLDPCA----SYVGTAA
TuMKK10-1/3            --X--XXXXXXXXXXXX-XX--X-XXXXXXXXXXXXXXX------XXXXXXXXXXXXXXXXXXXXXXXXXXXXXXXXXXXXXXXXXXGKATSADCAIAEVVSRAGKYRA----AYEGTAA
TaMKK10-1/3(4BSc1)     -RTASPFVVRWHSVLLATAS-GDAPLLLELVDGGSINSV-RSR-RGAFTEAALTEVTTQVLSGLAYLHARRIMHLDIKPANLLVSTAGEVKVADFGIAELLARTGEHYA----SYIGTSA
TaMKK10-1/3(4BSc4)     -RTGSPFVVRCHSVLPAATS-GDVALLLELVDGGSLDSV-RSR-RGAFTEAALAEVAAQALSGLAYLHARRIVHLDVKPANLLVSTAGEVKVADFGIAKVVARAGDHCT----SYAGTSA
TaMKK10-1/3(4BSc5)     -RTASPFVVRCHSVLPAAAS-GDVALLLELVDGGSLDSV-RTR-RGAFAEAALAEVAAQALSGLAYLHARRIVHLDVKPANLLISTAGEVKVADFGIAKVLARAGDYCT----SYAGTSA
TaMKK10-1/3(4DSc2)     -RTASPFVVRCHSVLPAAAS-GDVALLLELVDGGSLDSV-RTS-RGAFAEAALAEVAAQALSGLAYLHARRIVHLDVKPGNLLVSTAGEVKVADFGIAKVLARAGDHCA----SYAGTSA
TaMKK10-1/3(4BSc3)     -RTASPFCRAVPSVLPAAAS-GDVALLLELVDGGSLDSV-RSR-RGAFTETALAEVAAQALSGLAYLHARRIVHLDVKPANLLVTTAGEVKVADFGIAKVLARAGDHYT----SYAGTSA
HvMKK10-1/3(U)         -RTASPFVVRCHSVLPADAS-GDVALLLELVDGGSLDSI-RSR-RGAFAEAALAEVAAQALSGLAYLHARRIVHLDIKPANLLVSTAGDVKVADFGIAKVLARAGDQCT----SYAGTSA
TaMKK10-1/3(4AL)       -RTASPFVVRCHSVLPAAAS-GDVALLLELVDGGSLDSI-RSR-RGAFTEAALAEVAAQALSGLAYLHARRIVHLDIKPANLLASTAGEVKVADFGIARVLARAGDHCT----SYAGTSA
TaMKK10-1/3(4BSc2)     -RTASPFVVRCHSVLPAAAS-GDVALLLELVDGGSLDCI-RSR-RGAFTEAALAEVAAQALSGLAYLHARRIVHLDVKPANLLASTAGEVKVADFGIAKVLARAGDHCT----SYAGTSA
TaMKK10-1/3(4DSc1)     -RTASPFVVRCHSVLPAAAS-GDVALLLELVDGGSLDSI-RSR-RGAFTEAALAEVAAQALSGLAYLHARRIVHLDIKPANLLASTAGEVKVADFGIARVLARAGDHCA----SYAGTSA
AetMKK10-5p            --XXX-XXX-XXXX-------XXXXXXXXXXXXXXXXXXXXXX-XXXXXXXXXXXXXXXALAGLAYLRARRVVHRDMKPANLLVNRAGQVKIGDFGIAEVVSRAGKYRA----AYEGTAA
HvMKK10-5(4H)          -RVDSPHIVRCHSVLPT-AS-GDSALLLELMDGGSLDSLVRGG-QGGFPEAALAEVAAQALSGLAYLRARRVVHRDIKPANLLVNRAGQVKIGDFGIAEVVSRAGKYRA----AYEGTAA
TaMKK10-5(4DS)         -RVDSPHIVRCHSVLPT-PS-GDSALLLELMDGGSLDSLVRGG-QGGFPEAALAEVAAQALAGLAYLRARRVVHRDMKPANLLVNRAGQVKIGDFGIAEVVSRAGKYRA----AYEGTAA
TaMKK10-5(4AL)         -RVDSPHIVRCHSVLPT-AS-GDSALLLELMDGGSLDSLVRGG-QGGFPEAALAEVAAQALSGLAYLRARRVVHRDMKPANLLVNRAGQVKIGDFGIAEVVSRAGKYRA----AYEGTAA
TaMKK10-5(4BS)         -RVDSPHIVRCHSVLPT-AS-GDSALLLELMDGGSLDSLVRGG-QGGFPEAALPEVAAQALSGLAYLRSRRVVHRDMKPANLLVNRAGQVKIGDFGIAEVVSRAGKYRA----AYEGTAA
HvMKK10-4(4H)          -RVDSPHIVRCHSVLPT-AS-GDSALLLELMDGGSLDSLVRGG-HGGFPEEALAEVAAQALSGLAYLRARRVVHRDIKPANLLVNSAGHVKIADFGIAEVVSRAGKYRA----AYEGTAA
TaMKK10-4(3B)          -RVDSPHIVRCHSVLLT-GS-GDSALLLELMDGGSLDSLVRAG-QGGFPESALAEVAAQALSGLAYLRARRVVHRDIKPANLLVNSAGQVKIADFGIAEVVSRAGKYRA----AYEGTAA
TaMKK10-4(4BS)         -RVDSPYIVRCHSVLPT-AS-GDSALLLELMDGGSLDSLVRGG-QGGFPEEALAEVAAQALSGLAYLRARRVVHRDIKPANLLVNRAGQVKIADFGIAEVVSRAGKYRA----AYEGTAA
TaMKK10-4(4DS)         -RVDSPHIVRCHSVLPT-AS-GDSALLLELMDCGSLDSLFRAG-QGGFPEEALAEVAAQALSGLAYLRARRVVHRDIKPANLLVNSAGQVKIADFGIAEVVSRAGKYRA----AYEGTAA
TaMKK10-4(4ALc2)       -RVDSPHIVRCHSVLPT-AS-GDSALLLELMDGGSLDSLVRAG-QGGFPEEALAEVAAQALSGLAYLRARRVVHRDIKPANLLVNSGGQVKIADFGIAEVVSRAGKYRA----AYEGTAA
TaMKK10-4(4ALc1)       -RVDSPHIVRCHSVLPT-AS-GDSALLLELMDGGSLDSLVRAG-QGGFPEEALAEVAAQALSGLAYLRARRVVHRDIKPANLLINKAGQVKIADFGIAEVVSRAGKYRA----AYEGTAA
TuMKK10-4              -RVDSPHIVRCHSVLPT-AS-GDSALLLELMDGGSLDSLVRAG-QGGFPEEALAEVAAQALSGLAYLRARRVVHRDIKPANLLINKAGQVKIADFGIAEVVSRAGKYRA----AYEGTAA
```

MKK10 continued

```
TuMKK10p             XXXXXXXXXXXXXXXXXXXXXXXXXXXXXXXXXXVTILELLMGRYPLLPAGQELTWAALMCAICFGELPALPDGAASPE-LRSFVSACLQKDHRKRASVAELLAHPFVAGRDVASARHAL
AetMKK10p            YMSPERFDTERSLQGDGEEKGPVDPYVTDVWGLG--------------------------------------------------------------------------------------
HvMKK10(1H)p         YMSPERFAPNA-------QAGSRGACAADVWSLGVTVLELFLGHRPVLPAERTPSWKMLKEAICYGEPPSVPGSAAASAELRGFVAACVQKDPRRRATVPQLLAHPFVARRDVEASSRAL
ScMKK10p             YMSPERFAPNA-------QAGPRGACAADVWSLGVTVLELFLGHRPVLPAERTPSWKMFKEAICYGEPLGVPDSAAASDELRGFVAACVQKDPRRRATVPQLLAHPFVARRDVEASRRAL
BAJ94499.1           YMSPERFDPET-------YAGDYDPYAADVWSLGMAILELYLGHFPLLPAGQRPDWAALMCAICFGEAPEAPAAAS--DEFRDFVARCLEKKAGRRASVAELLEHPFIAERDAEEAQRCL
HvMKK10-2(4H)        YMSPERFDPET-------YAGDYDPYAADVWSLGMAILELYLGHFPLLPAGQRPDWAALMCAICFGEAPEAPAAAS--DEFRDFVARCLEKKAGRRASVAELLEHPFIAERDAEEAQRCL
TaMKK10-2(4AS)       YMSPERFDPEA-------YSGDYDPYAADVWSLGMAILELYLGHFPLLPAGQRPDWAALMCAICFGEAPEAPAAAS--DEFRDFVARCLEKKAGRRASVAELLQHPFIAERDAEEAQRCL
TsMKK10p             ------------------------------------------------------------------------------------------------------------------------
TaMKK10-2(4BL)       YMSPERFDPET-------YSGDYDPYAADVWSLGMAILELYLGHFPLLPAGQRPDWAALMCAICFGEAPEAPAAAS--DEFRDFVARCLEKKAGRRASVAELLGHPFIAERDAEEAQRCL
TaMKK10-2(4DL)       YMSPERFDPEA-------YSGDYDPYAADVWSLGMAILELYLGHFPLLPAGQRPDWAALMCAICFGEAPEAPAAAS--DEFRDFVARCLEKKAGRRASVAELLQHPFIAERDAEEAQRCL
TuMKK10-1/3          YMSPERFDTERSLQGDSDEEGPVDPYAADVWGLGVTVLELLVGRYPLLPAGQELTWAALMCAVCFGELPALPDGAASPE-LRSFVAACLQKEHRKRASVGELLVHPFVAGRDVAASRRAL
TaMKK10-1/3(4BSc1)   YMSLEHFDQEA-------HPGPYDPYAADVWSLGVTLLELFMGRYPLLPAGQQPTWVALMCAIFFGNRQCCPTAQPRRSSGDSLPRACKRTTARGRPSWSCLLTHSSP*-----------
TaMKK10-1/3(4BSc4)   YMSPERFDPEA-------HGGHYDPYAADVWSLGVTLLELFMGRYPLLPAGQRPSWAALMCAVCFGEPPVLPAGASSPE-LRGFVASCLQKDYRNRASVAELLAHPFVAGRDMPVSKCAL
TaMKK10-1/3(4BSc5)   YMSPERFDPEA-------HGGHYDPYAADVWSLGVTLLELFMGRYPLLPAGQRPSWAALMCAVCFGEPPAARR*----------------------------------------------
TaMKK10-1/3(4DSc2)   YMSPERFDPEA-------HGGHYDPCAADVWSLGVTLLELFMGRYPLLPAGQRPSWAALMCAVCFGDPPVLPEGASSPE-LRGFVAACLRKDYRNRASVAELLAHPFVAGRDVAASRCAL
TaMKK10-1/3(4BSc3)   YMSPECFDPEA-------HGGHYDPYAADVWSLGVTLLELFMG-YTLLPAGQQPTWAPLMCAVCFGEPPVLPDAAASPE-LRGFVASCLHKDYRNRASVAELLVHPFVARRDVVVSKRAL
HvMKK10-1/3(U)       YMSPERFDPEA-------HGGHYDPYAADVWSLGVTLLELFMGRYPLLPAGQQPTWPALMCAVCFGEPPVLPDGAASPE-LRGFIAACLQKDYRSRASVAELLAHPFVAGREVAASKRAL
TaMKK10-1/3(4AL)     YMSPERFDPEA-------HGGHYDPYAADVWSLGVTLLELFMGRYPLLPAGQQPTWAALMCAICFGEPPVLPDGAASPE-LRGFVAACLQKDYRNRASVAELLAHPFVAGRDVAASKRAL
TaMKK10-1/3(4BSc2)   YMSPERFDPEA-------HGGHYDPYAADVWSLGVTLLELFMGRYPLLPAGQQPTWAALMCAVCFGEPPVLPDGAASPE-LRGFVASCLQKDYRNRASVAELLAHPFVAGRDVAASKRAL
TaMKK10-1/3(4DSc1)   YMSPERFDPEA-------HGGQYDPYAADVWSLGVTLLELFMGRYPLLPAGQRPSWAALMCAVCFGEPPVLPDGAASPE-LRGFVAACLSKDYRNRSSVAELLAHPFVAGRDVAASKCAL
AetMKK10-5p          YMSPERFDTERLHDG---EEDRVDPYAADVWGLGVTVLELLMGRYPLLPAGQELTWAALMCAICFGELPALPDGSASPE-LRSFVSACLQKDHRKRASVAELVAHPFVAGRDVAAARHAL
HvMKK10-5(4H)        YMSPERFDTERLHD---GEEGRVDPYAADVWGLGVTVLELLMGRYPLLPAGQMPTWAALMCAICFGELPAIADGAASPE-LRSFVSACLQKDHRKRASVAELVAHPFVAGRDTAASRHAL
TaMKK10-5(4DS)       YMSPERFDTERLHE---GEEDRVDPYAADVWGLGVTVLELLMGRYPLLPAGQELTWAALMCAICFGELPALPDGSASPE-LRSFVSACLQKDHRKRASVAELVAHPFVAGRDVAAARHAL
TaMKK10-5(4AL)       YMSPERFDTERLQD---GEGGRVDPYAADVWGLGVTILELLMGRYPLLPAGQELTWAALMCAICFGELPALPDGAASPE-LRSFVSACLQKDHRKRASVAELLAHPFVAGRDVASARHAL
TaMKK10-5(4BS)       YMSPERFDTERLHD---GEGNRVDPYAADVWGLGVTVLELLIGRYPLLPAGQELTWAALMCAICFGDLPALPDGAASPE-LRSFVSACLQKDHRRRPSAAELLAHPFVAGRDVAAARHAL
HvMKK10-4(4H)        YMSPERFDTERSLHGDGDEQDRVDPYAADVWGLGVTVLELLVGRYPLLPAGQKPSWAALMCAICFGELPTLLDSTASPE-LRGFVAACLQKDHRKRASVAELLVHPFVTGRDVAASRRAL
TaMKK10-4(3B)        YMSPERFDTERSLHVAGDEEGPVDPYAADVWGLGVTVLELLMGRYPLLPAGQELSWAALMCAVCFGELPALPDGAASPE-LRGFVAACLQKDHRKRASVAELLVHPFVAGRDVAASRRAL
TaMKK10-4(4BS)       YMSPERFDTERSLHGDGDEEGPVDPYAADVWGLGVTVLELLMGRYPLLPAGQELTWAALMCAVCFRELPALADGAASPG-LRGFVAACLQKDHRKRASVGELLVHPFVAGRDVAASRRAL
TaMKK10-4(4DS)       YMSPERFDTERSLQGDGDEEGPVDPYAADVWGLGVTVLELLMGRYPLLPAGQKPSWAALMCAVCFGELPALPDGAASPE-LRSFVAACLLKDHRKRASVGDLLVHPFVAGRDVAASRRAL
TaMKK10-4(4ALc2)     YMSPERFDTERSLQGDGDEEGPVDPYAADVWGLGVTVLELLVGRYPLLPAGQELTWAALMCAVCFGELPALPDGAASPE-LRSFVAACLQKDHRKRASVGELLVHPFVAGRDVAASRRAL
TaMKK10-4(4ALc1)     YMSPERFDTERSLQGDGDEEGPVDPYAADVWGLGVTVLELLMGRYPLLPAGQELSWAALMCAVCFGELPALSDGAASPE-LRSFVAACLQKDHRKRASVSELLVHPFVAGRDVAASRRAL
TuMKK10-4            YMSPERFDTERSLQGDGDEEGPVDPYAADVWGLGVTVLELLMGRYPLLPAGQELSWAALMCAVCFGELPALSDGAASPE-LRSFVAACLQKDHRKRASVSELLVHPFVAGRDVAASRRAL
```

MKK10 continued

```
TuMKK10p                REVIAQRV*---------
AetMKK10p               ------------------
HvMKK10(1H)p            RHVIVETM*---------
ScMKK10p                REIIVETM*---------
BAJ94499.1              AALVAEAAELGDQ*----
HvMKK10-2(4H)           AALVAEAAELGDQ*----
TaMKK10-2(4AS)          AALVAEAAELGDQ*----
TsMKK10p                ------------------
TaMKK10-2(4BL)          AALVAEAAELGDQ*----
TaMKK10-2(4DL)          AALVAEAAELGDQ*----
TuMKK10-1/3             RKLIEQRC*---------
TaMKK10-1/3(4BSc1)      ------------------
TaMKK10-1/3(4BSc4)      RKLVADASSSL*------
TaMKK10-1/3(4BSc5)      ------------------
TaMKK10-1/3(4DSc2)      RKLVADASSPSSDCRHT*
TaMKK10-1/3(4BSc3)      RKLVADASSSL*------
HvMKK10-1/3(U)          RKLVADASSSL*------
TaMKK10-1/3(4AL)        RKLVADASSSL*------
TaMKK10-1/3(4BSc2)      RKLVADASSSL*------
TaMKK10-1/3(4DSc1)      RKLVADASSPSLECRHT*
AetMKK10-5p             REVIAQR-----------
HvMKK10-5(4H)           REVIEQRQ*---------
TaMKK10-5(4DS)          REVIAQRV*---------
TaMKK10-5(4AL)          REVIAQRV*---------
TaMKK10-5(4BS)          REVIEQRV*---------
HvMKK10-4(4H)           CQVIEQRSMPISTRV*--
TaMKK10-4(3B)           REVIDQRCR*--------
TaMKK10-4(4BS)          REAIEQRCR*--------
TaMKK10-4(4DS)          REVIEQRCG*--------
TaMKK10-4(4ALc2)        RKLIEQRC*---------
TaMKK10-4(4ALc1)        GEVIKQRC*---------
TuMKK10-4               GEVIKQRC*---------
```
