# Supplementary material for: Analysis of MAPK and MAPKK gene families in wheat and related Triticeae species
Source: BMC Genomics. 2018 Mar 5;19:178. doi: 10.1186/s12864-018-4545-9 (PMC5838963; doi:10.1186/s12864-018-4545-9)
Supplement: Supplementary file 9 — Yeast-two-hybrid table and relevant MAPK sequences. Results from Y2H gave weak (+) and strong (+++) positive or negative (−) MPK-MKK and MKK-MKKK interactions are presented in the table below (p.2). Positive Y2H interactions for orthologous genes observed in Arabidopsis [49], rice [4, 65], Brachypodium [11], maize [50] are also shown. The MPK and MKK sequences used that do not match a nucleotide identifier from Additional file 3 are marked as clones or synthetic (syn), and the alignment for these sequences are provided at the end of this file (p.6–18). TaMKKK sequences are provided (p.4–5) along with phylogenetic analysis (p.3) of MPKKK sequences used here together with MPKKK sequences identified in Wang et al. [9]. (PDF 231 kb) [file 12864_2018_4545_MOESM9_ESM.pdf]

Additional File 9. Yeast-two-hybrid table and MAPK sequences. Weak (+) and strong (+++) positive or negative (-) MPK-MKK and MKK-MKKK interactions are presented in the table (p. 2). Positive Y2H interactions for orthologous genes observed in Arabidopsis [1], rice [2, 3], brachypodium [4], maize [5] are also shown. MPK and MKK sequences used that do not match a nucleotide identifier from Additional File 3 are marked as clones or synthetic (syn), and the alignment for these sequences are provided at the end of this file (p. 6-18). TaMKKK sequences are provided (p. 4-5) along with phylogenetic analysis (p. 3) of MKKK sequences used here together with MKKK sequences identified in Wang et al. [6].

Reference:

1. Lee JS, Huh KW, Bhargava A, Ellis BE. Comprehensive analysis of protein-protein interactions between Arabidopsis MAPKs and MAPK kinases helps define potential MAPK signalling modules. Plant Signal. Behav. 2008;3:1037-41.

2. Singh R, Lee M-O, Lee J-E, Choi J, Park JH, Kim EH, et al. Rice mitogen-activated protein kinase interactome analysis using the yeast two-hybrid system. Plant Physiol. 2012;160:477-87.

3. Wankhede DP, Misra M, Singh P, Sinha AK. Rice mitogen activated protein kinase kinase and mitogen activated protein kinase interaction network revealed by *in-silico* docking and yeast two-hybrid approaches. PLoS ONE. 2013;8:e65011.

4. Chen L, Hu W, Tan S, Wang M, Ma Z, Zhou S, et al. Genome-wide identification and analysis of MAPK and MAPKK gene families in *Brachypodium distachyon*. PLoS ONE. 2012;7:e46744.

5. Kong X, Pan J, Zhang D, Jiang S, Cai G, Wang L, et al. Identification of mitogen-activated protein kinase kinase gene family and MKK–MAPK interaction network in maize. Biochem. Biophys. Res. Commun. 2013;441:964-9.

6. Wang M, Yue H, Feng K, Deng P, Song W, Nie X. Genome-wide identification, phylogeny and expressional profiles of mitogen activated protein kinase kinase kinase (MAPKKK) gene family in bread wheat (*Triticum aestivum* L.). BMC Genomics. 2016;17:668.

Table. Weak (+) and strong (+++) positive or negative (-) MPK-MKK and MKK-MKKK Y2H interactions. n.d. = no data. Positive interactions for orthologous genes were observed in [a]Arabidopsis [1], [b]rice [2, 3], [c]brachypodium [4], [d]maize [5]. It should be noted that Arabidopsis and rice have only one paralogue of MKK3, and Arabidopsis has only one MKK10. The reported MKK6-MPK16 in rice was observed for OsMPK16-1, whereas the OsMKK6-OsMPK16-2 was negative. Other notable interactions from previous reports include: OsMKK10-2 with OsMPK7 [3]; BdMKK3-1, but not BdMKK3-2, with BdMPK7-1/11 [4], and ZmMKK3-1 with ZmMPK6-2/7 [5].

	**TaMKK1-1** (syn)	**TaMKK3-2a** (KT187393)	**TaMKK3-2b** (clone)	**TaMKK3-3** (syn)	**TaMKK4** (syn)	**TaMKK5a** (syn)	**TaMKK5-2b** (syn)	**TaMKK6** (RFL_4066)	**TaMKK10-2** (syn)	**TaMKK10-1/3** (4AL)	**TaMKK10-4** (4DS)
**TaNPK-1** (syn)	-	+	+	-	+	-	-	-	-	-	-
**TaZIK7** (Traes_2BS_18264AA5C)	-	+++	-	-	-	-	-	-	-	-	-
**TaMKKKA2** (syn)	-	-	-	-	-	n.d.	-	+	-	-	-
**TaMKKK2b** (syn)	-	-	-	-	-	-	-	-	-	-	-
**TaMKKK2a** (syn)	-	-	-	-	-	n.d.	-	+	-	-	-
**TaFLR** (AY173961)	-	-	-	-	-	-	-	-	-	-	-
**EDR-1** (AY743662)	-	-	-	-	-	-	-	-	-	-	-
**TaCTR1** (DQ211934)	-	-	-	-	-	-	-	-	-	-	-
**AK336073**	-	-	-	-	-	-	-	-	-	-	-
**AK335039**	-	-	-	-	-	-	-	+	-	-	-
**TaMEKK1** (AK334817)	-	-	-	-	-	-	-	-	-	-	-
**TaMEKK22** (AK333042)	-	-	-	-	-	-	-	-	-	-	-
**AK330734**	-	-	-	-	-	-	-	-	-	-	-
**TaMPK3** (4DL)	-	-	-	-	-[a,b]	-	-	-	-	-	-
**TaMPK4** (clone)	+++[a,b,d]	-	-	-	-	-	-	+++[a,b]	-	+	-
**TaMPK6** (7DS)	-[b]	-[b]	-[b]	-[b]	-[a,b]	+[a,b]	-[a,b]	+[a,b,c]	-[b,d]	+	-
**TaMPK7** (7BL)	-	-[a,b]	-[a,b]	-[a,b]	-	n.d.	-	-	-[b]	-	-
**TaMPK11** (7BL)	-[a]	-	-	-	-	n.d.	-	-[a]	-	-	-
**TaMPK14** (6DS)	-	-[a,b]	-[a,b]	-[a,b]	-	-	-	+++	-	-	-
**TaMPK16** (1BS)	n.d.	n.d.	n.d.	n.d.	n.d.	-	n.d.	-[b]	-	-	-
**TaMPK17** (clone)	-	-	-	-	-	-	-	-	-[a]	-[a]	-[a]
**TaMPK20-1** (3AL)	-	-	-	-	-	-	-	-[b]	-	-	-
**TaMPK20-2** (clone)	-	-	-	-	-	-	-	-	-	-	-
**TaMPK20-3** (KR422425)	-	-	-	-	-	-	-	-[b]	-	-	-
**TaMPK20-4** (clone)	-	-	-	-	-	-	-	-[b]	-	-	-
**TaMPK20-5** (KR422426)	-	-	-	-	-	-	-	-	-	-	-
**TaMPK21-1** (KR422427)	-	-	-	-	-	-	-	-	-	-	-
**TaMPK21-2** (clone)	-	-	-	-	-	-	-	-	-	-	-
**TaMPK24** (clone)	-	-	-	-	-	-	-	-	-	-	-

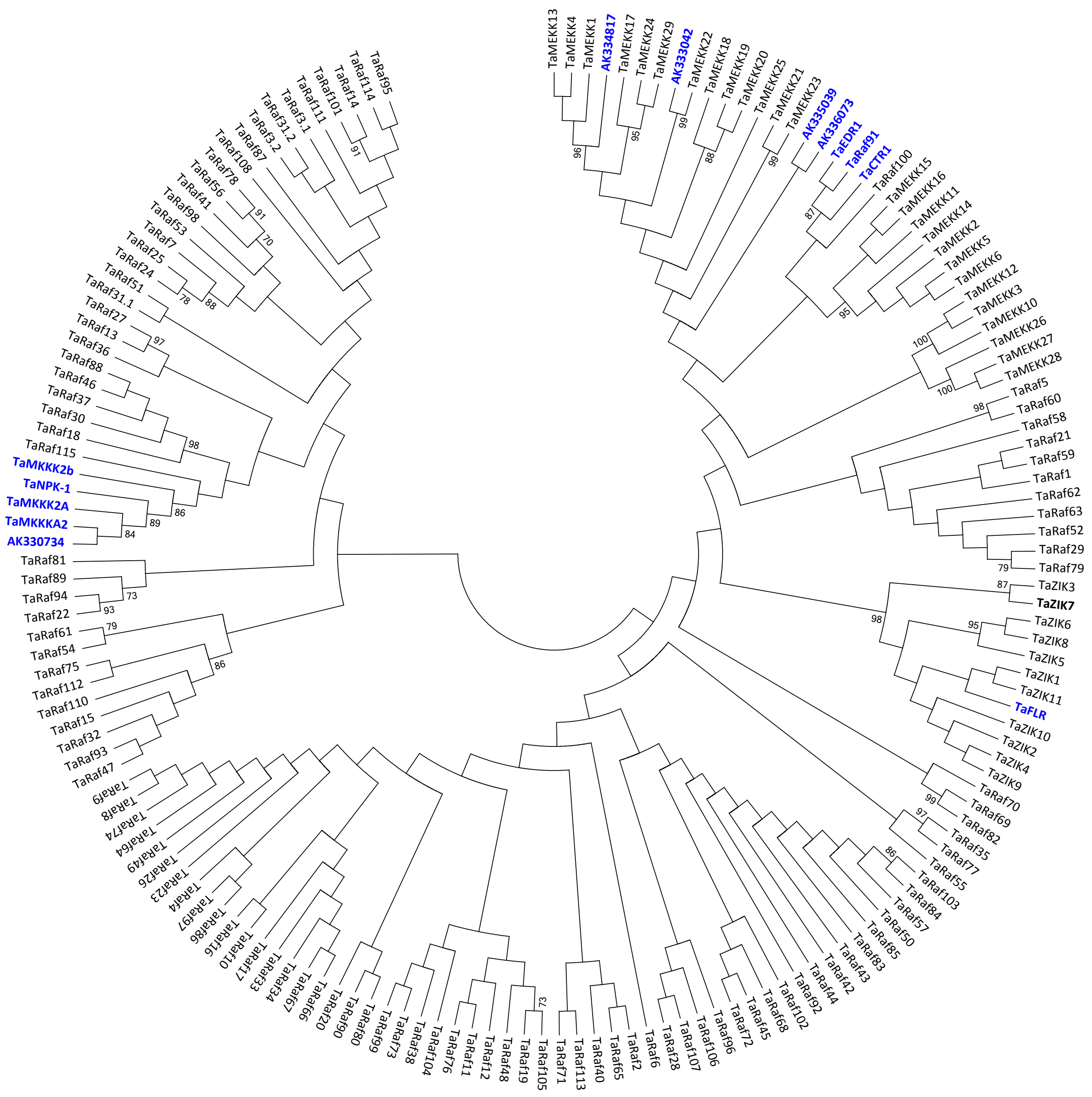

Phylogenetic Tree. Previously reported TaMAPKKK protein sequences [6] were aligned with TaMAPKKK (blue font) sequences used for Y2H experiment presented in the Table above. TaMAPKKK sequences used for Y2H are presented below.

>TaFLR (AY173961)
MSSPRRPEMSPAARNSDNGYVETDPTGRYGRFDELLGKGAMKSVYRGFDEVRGVEVAWNQANLADVLRTPDALQRMYSEVHLLSTLRHDAIIAFHASWVSVSSPSPRGGCTGGTPRRTFNFITELFSSGTLRAYRLRYPRVSLRAVRGWARQILRGLAY
LHAHDPPVIHRDLKCDNVFVNGHQGTVKIGDLGLAAVLRGAQAAHSVIGTPEFMAPEMYDEDYDELVDVYSFGMCMLEMLTVEYPYAECSNPAQIYKKVTSGKLPDAFYRVDDADARRFIGRCLVPASHRPSAQELLLDRFLSTQDTTMTLSPPPLLPA
LPTSGDRKDNPEEAEPVAARTDMTITGKLNTDDDTIFLKVQIVDEAGHSRNIYFPFDIAGDTATEVAREMVKELDITDRDPSEIAAMIEQEITRLVPDWVGGGCDDQQEYYTYADNDDNEEQPPFYYLSSSPTSSNGSHCGTGPTTSGGGYAGWFQDYA
VSSDDDETSSTRSALHYSSEEAQPEEKPGVSKTGQVKATRFGPGDSGTAGHDVSSSRAGRPRHHRGSPDAGGDEGRPRRQQGRMTRNRSMVDVRSQLLHRTLVEELNKRMFFNTVGAVENIGFRRIPGYGGGPSSSSATVTSSRGGDQRGRRSGKDKHQ
FFMF

>TaNPK-1
MAIAVSGQWTRLRTLGCGASGAVVTLAADAASGELFAVKSVRAVDAAQLSREQGILSGLSSPHVVGCIGGGGDRDGSYHLFLEFAPGGSLADEAARNGGRLEERAIRAYAADVLRGLAYIHGKSLVHGDVKSRNVVIGADGRAMIADFGCARALGSAGP
IGGTPAFMAPEVARGEEQGPAADVWALGCTVVEMATGRAPWGEMDNVLAAVHRIGYTDAVSEVPTWLSAEAKSFLAMCFARNARDRCTAAQLLEHPFVALQAGEAKARWVSPKSTLDAAFWESETDEEEEEVSESPCERIKSLSCSVLPDWDSDEGWID
VLGEQCEEACDSPAATKGPADVSSRAPSKVLGSPAAPAEDAAVVGTLSSDELDAEDEPFGDDIILAADPLVDRQIQVCWSSR

>TaMKKKA2
MDVAVAKQLRRIRTLGRGASGAVVWLASDEASGELLAVKSAGAGGAAQLEREGSVLTGLCSPHIIPCLGSRAAECGEYQLFLEFAPGGSLADEAARSGGCLPEPAIRAYAGDVARGLEYLHARSLVHGDVKARNVVIGGDGRARLTDFGCARAVDSLLP
MGGTPAFMAPEVARVEEQGPASDVWALGCTVVEMATGRAPWSDMNDLLAAVHRIGYTAAVPEVPGWLSAEAKDFLDGCFRRQPSDRSTAAQLLDHPFVASAAAAGDYKAAPAKQQYTSPKSTLQDALWDSDTDDEADEMSATPAERIGALACGNSALPD
WDSDDGWIDVCDEVHRVPDSPPADAGYDLVWPEESDAEREPFAVAADDSNDIPRNAVVTDSSIWQDSYVRPVHLGSCRNQFHPFQSDGDENLRFDRRCNKDRVTGDDPVAAAEVVPAPAKHHVPLEVLDLHPEHAPLVHRQPNHAREPLDDQPGVHHLH
ELARDDEHGEVLVVLDLLVEAQRLPGPLLVALPRRSSP

>TaMKKK2A
MSVSKQWTRVRTLGRGASGAEVFLAADDASGELFAVKSASAACAAALRREQRVMAGLSSPRVVSCIGGRGAPDGSYQLFLEFAPGGSLADQMATGLDELAVRGYAADVAAGLAYLHSAGMVHGDVKARNVVIGADGRAKLADFGCSRKAGADVPIIGGT
PAFMAPEVARGEEQSPAADVWALGCMVVEMATGRTPWSGMDGDALAALHRIGYTEAMPEVPEWLSADAKDFLARCLVRQASDRCTAAQLLEHPFLASAVVEAKAQAVESKWVSPKSTLDAAFWESDSDTEEAEHDSSAETRIRALACPASELPDWDSDE
GWIDVLSSPTGATGAVPVVVEEMTDLDEDATTDEEPSGAESRVLAITLDVEYNSVLNAVEACDDSFRRHQSLECLASHELSCTELLLCNRKTNAIDLVVAQALCFRTAALCFTAPHCDTFE

>TaMKKK2b
MGVGEWTRGPAIGRGSSATVSIAVDRRTGGVIAVKSVGADRAAELRREQGILRGLTSPYVVRCLEAEVSASVDGGLDMLMEYAPSGSLADEIRRCGGRCAEALVRSRARDILLGLAHVHAAGVAHCDVKGRNVLIASDGRALIADFGCARRTGGGIAGE
ERQRPTGGTPMFMAPEAARGEEQGPAADIWAVGCTVIEMATGAAPWQRFASPVATLHHVAFSGEAPEFPACLSDQGKDFLARCLRQDPRERWTAEQLLEHEFVAAAGTASSSNSAPGITEKATFVSPKSVLDQALWEDDDDDTAADTADPTDRVRALAA
GAPAVPDWTWDASWITVHAGPSGXADEEPAMPSEPEASTDTDSDDSPVGDGGSAGRAAAAEVGASSSHQASHANSDRYDGTGSCNVERSDDGNHVVSDDCSTVPITSNGFFSDTTSCFACPSRSQAGRTGPFTVLLYHISSPRCCSSCHLFLASPLLAG
VSGTRSPT

>TaEDR1 (AY743662)
MKIPFVTKWSHRSSEPAGPSNSAAAQQQQQAPSSPSAPSPPPPVASTEAVGDEFILQEEEYQMQLALALSASASGAEEAGDPDGEQIRKAKLMSLGKGDPVTNTDRGGGDTPESLSRRYRDYNFLDYNEKVIDGFYDIFGLSAESSGQGKIPSLAELQM
SIGDLGYEVIVVDYKFDNALQEMKEVAECCLLGCPDITVLVRRIAEVVADHMGGPVIDANEMITRWLSKSIEQRTSHQTSLLHIGSIEIGLSRHRALLFKILADIVGIPCKLVKGSHYTGVEDDAINIIKMDDKREFLVDVMAAPGTLIPADVFNSKGT
PFKFSQTLGQNQVVESASNIEDDPVALQSEHKRNQGHMFANNNRISDNLSSYENTVTAGSSASEPGTLDPRMQLGKTSTLASAPSKQKKNLQLIPDSHETEESRKLFAEFDPFNATKSGKSSLVFKGLNNRNNEFQRRRENVVPPSVRSQQPLVMKNWS
ACNDISNNKQYNVADGSVPRRNATDNASSSQLALSTAKHYNSNVRELNDRVYAAPARNYDNKIVGTSAMAKALTGECPDRSQVPPGLYYDKMLGTSSMNAASTSGIGKVAEKDPHNDPGKGPIYSRFDGELSKNAQGFTPERDEHKENCGSHDHKMLYP
DPRKSPLDRFMDRPRQSIECVFPSQVGSNKADMVLDEVSECEILWEDLVIDERIGIGSYGEVYHADWNGTEVAVKKFLDQEFYGDALEEFRCEVRIMRRLRHPNIVLFMGAVTRPPHLSIVSEYLPRGSLYKIIHRPNCQIDEKRRIKMALDVARGMNC
LHTSVPTIVHRDLKSPNLLVDDNWTVKVCDFGLSRLKHSTFLSSKSTAGTPEWMAPEVLRNEQSNEKCDIYSFGVILWELATLRKPWHGMNQMQVVGAVGFQDRRLDIPKEVDPIVASIIRDCWQKDPNLRPSFIQLTSYLKTLQRLVIPSHQETASNH
VPYEISLYR

>TaCTR1 (DQ211934)
MPHRRRLHNLVPPLPPPPAAPHPAFDLSVAESRLPLLADYGRLKPVDDLPAPAAPPSAHWSAGSAFTGTEPATASTSTAATGSSLXPASSVARDDTWVRRSRESYYLQLSLAIRITSEAFLAGVPPELLVRRLGPGDAAAEQHADVPADAAAVSYRLWV
NGCLSWGDKIAHGFYNXMGIDPHXWAMCNADEGEGRRLPTLAALREVDASDQSSLEVVLVDKCGDSVLVDLERRALDLYRALGATLDLVRRLALLVSDHMGGALRSEDGDLYMRWKAGSKRLRKQQKSVVVPIGRLSIGFCRHRAILFKALADFIGLPC
RIAQGCKYCSAPHRSSCLVKIENDRRYSREYVVDLVVAPGSICSPDSSINGQLLXSVSSPFKTSCTASLENYAAPVAAWNRAIADDRCNSVFSDSQXSVAGDKNPVQADTKCGQVMQNDNCNNMSVFQVXRQFKAMEVGTEGVNKENIPGLTLPKHLLA
ESSFAMDWLEISWDELELKERIGAGSFGTVYRADWHGSDVAVKVLTDQGDGEAQLKEFLREISIMKRVRHPNVVLFMGAVTKCPHLSIVTEYLPRGSLFRLISXASSGEILDLRRRLRMALDVAKGINYLHCLNPPIVHWDLKTPNMLVDKNWSVKVGD
FGLSRFXATTFISSKSVAGTPEWMAPEFLRGEPSNEKCDVYSFGVILWELLTMQQPWGGLGPAQVVGAVAFQNRRLPIPKDTIPELAALVESCWSDDPRQRPSFSSIVDTLKKLLKSMQGSGS

>AK336073
MGTPQRPRPRQLARTNAMRNSSYSADAGAAGDDDLAAYGRIQLSVDRAARASPGARAGYASQTSFRIHGGRGGGEEVAELFRQLGLSGPEDFAIPPAVYAAANAARRAASLEEPSPAALPSGVPEISGRDVVVASRLQPAGDGEEAGLATELVQSETIQ
VSAKSYQRPWAESKAILVESERVETSTREVTAVSEPENAGEGKGNDKLAKVEISREERTREVVVEATREKATGALALVVAESNSCDIEHLVSPSPNRRFRRTITSWIKGGHIGSGSFGSVYEAISDDGFFFAVKEVSLIDQGINAKQRIVQLEHEVSLL
SRLEHDNIVQYYGTDKEDGKLYIFLELVTQGSLAALYQKYCLQDSQVSAYTRQILNGLNYLHQRNVLHRDIKCANILVDANGLVKLADFGLAKEMSILSQARSSKGTVFWMAPEVAKAKPHGPPADIWSLGCTVLEMLTGKVPYPDMEWTHALLKIGRG
IPPKIPNTLSEDARDFIAKCVQANQKDRPSAAQLLEHPFVKRPLQH

>AK335039
MGRPSQDTATSTSPSGSCRSSKRGPRLDRRNASKNIGYEYDPAKLFCSYPPSPSRASSASASAAPSVASSVDLSSFRIGGSGDGGGDVQLLCRNLGLSGPEDFAISLTDWEAHKAFRSSAFSSASSSPSARSQPDRPVRESPLRHEAVEEPTLPADADF
ELPAKETARDPPVEAPVRPAWLDPPEPARPDVKKPGCEGGIKGVRPPPVMLKPPPSMALPPASQAGSTWDILLSFAPEEQGQPQAIRSVPDFGDPDAEDEDAAEVLTLEDLRLGESSEEFTGTSSISTTNDDETTESMFYISPNGRFKRKIRSWSRGVL
LGSGSFGTVYEGISDEGVFFAVKEVSLHDQGSNAQQCIFQLEQEIALLSQFEHENIVHYFGTDKEDSKLYIFLELVTQGSLVSLYQKYRLRDTHVSAYTRQILNGLNYLHERNIVHRDIKCANILVHANGSVKLADFGLAKQTSKLNVLKSCKGTVYWM
APEVVNPKKTYGPAADIWSLGCTVLEMLTRQLPYPDLEWTQALYRIGKGEPPAIPSGLSKEARDFISQCVKPNPEDRPSASKLLDHPFVNRSMRSIRSMRTYTRPNSSTRGTSG

>AK334817
MPAWWKGKGRSKSKAAAPAGDAGTIPAGRDGEKDRKNKKASSFDEALIGREGRGKQLQQPAPAVGHPLPRPASMPSASAPASASASASSGGSSSLGSSAASDEPLDLGIYRISDANRTPAIDSRKQSLVLEEGRFVVNNLASENNRSCEPSVSPRKEFQ
PNILDLPSDRTTYCHGRKSTEIVFATRMPNSPPSSRGKHCPTSPVHSRAFGQCPGSPTAWQDDARNSSSPHPLPLPPGSPCTSSRSLHSQWKKGKLLGSGTFGQVYLGFNSEGGQMCAIKEVKVIADDSNSKECLRQLNQEMLLLNQLSHPNIVQYYGS
ELSSETLSVYLEFVSGGSIHKLLQEYGPFGETILRNYTAQILSGLAYLHGRNTVHRDIKGANILVDPNGDIKLADFGMAKHISAYTSIKSFKGSPYWMAPEVIMNTNGYSLSVDIWSLGCTILEMATARPPWSQYEGVAAIFKIGNSKDIPDIPDHLSS
EAKSFLKLCLQRDPAARPTAAQLIEHPWVKDQASVRSSRSGSGITRDMFSTSTDGSKATVKTSIELSSYRSLSPLRDTNLRMRNLVVPASSIPSISTHRASAISASNVRMNMSLPVSPCTSPLRQYRQSNRSCLPSPPHPAYSAGAANYSPINNVLYPT
RPSNYLTDPWLETPRQKTQTFDSPRRL

>AK333042
MAFSPRSPWSRSKKPDIYSTVVVHDDEDDARGGGAARAEDDDDDDPSALPPLLQRLPKDFGGASFDDDDDPYSSDLDDASLSATVVIKRGAPASTSSSSRSPFLDLRRSSPRAAEADPFSTFVVHGTARSGGASSPRESVSGTFIRHSGGPPSPRESVS
GTFIRHTRGSSSPHESFSGTFIHHTSSASSPRDSASGAGAGFGSSFITPSSGQAEEDRQPSLLMQQQQSRRKASMSSLPDSVTREDPSTKYELLHELGKGSYGAVYKARDLRTQELVAVKIISLTEGEEGYEDIRGEIEMLQQCSHPNVVRYFGSYQGE
EYLWIVMEYCGGGSVADLIGITEEPLDEPQIAYICRETLKGLAYLHTIFKVHRDIKGGNILLTEQGEVKLGDFGVAAQLTRTMSKRNTFIGTPHWMAPEVIQESRYDGKVDVWALGVSAIEMAEGMPPRSTVHPMRVIFMISSEPAPMLEDKEKWSLLF
HDFIAKCLTKDARLRPPAIEMLKHKFIEKCNTGASKMLAKIKEAKIIRETAVQNQLPDSDDAMDATVRINEDYGETVPTNSQSTHETKNDGSGGDFGTMIVHPEDGDEAAESSIFPRAEFIPGLGSINSFTHDPKRAELISKFWAESTAESDASKERDL
YGLPDIQEPKTMPRSTGTVKHHKGVEGTVLRHDITASPGVASTMNKLSSSPSRKAFSVQDKLWSIYAAGNTVPIPFLKAIDISPLALVSDSVAGNGPAGSSTTDALEAVRELFSGDGQAKKGRKGQNEAPLPPGVHDRLTTSPTLMNLAQALAYHKTCY
EDMPLQDSQATEEQQTIQNLCDTLRTILRL

>AK330734
MDATTAKQLRRLRTLGRGASGAVVWLASDDASDQLLAVKSAGAGAADTLRREGSVMAGLCSPHIVPCLGSRAAPGGEYQLFLEFAPRGSLADEAARSGGSLAERAIQGYAADVARGLAYLHGNSLVHGDVKARNVMVGADGRAKLADFGCARAADSDRT
IGGTPAFMAPEVARGEEQGLAADVWALGCTVIEMATGRAPWGDMDDVFAAVHRIGYTDAVPELPASLSPQAKDFLCKCLARNPRHRSTAAQLLEHPFLASAFRDVDAEPAKQDWMSPKSTLNAEFWESDEESETEDMLTSAAERIASLASPCSALPDWE
SDDGWIDVHGERPEASETTSATAIAGTDFGPWSREEGLEAELGVRFADADAGRWYDLVRNVDGAHYFGWYERDFSVSVATDRPLCLIVSHGRKIVKFGCHCDRERTMHFDFAQIFRSLELPSHAVCILSSTEWGYCRFLAFSFYN

```
TaMKK1-1(7AS)      MKKPGKLALPSQDSTIGKFLTQSGTFKDGDLLVNKDGLRIVPQSEEGEAPPIKPLDNNHQ LSIDDLDSIKVIGKGNSGTVQLVRHKWTGQFFALKVIQLNIQESIRKQMAQELKISLFTQ
TaMKK1-1(4AL)      MKKPGKLALPSQDSTIGKFLTQSGTFKDGDLLVNKDGLRIVPQSEEGEAPRIKPLDNNHQ LSIDDLDSIKVIGKGNSGTVQLVRHKWTGQFFALKVIQLNIQESIRKQMAQELKISLFTQ
TaMKK1-1(7DS)      MKKPGKLALPSQDSTIGKFLTQSGTFKDGDLLVNKDGLRIVPQSEEGEAPPIKPLDNNHQ LSIDDLDSIKVIGKGNSGTVQLVRHKWTGQFFALKVIQLNIQESIRKQMAQELKISLFTQ
TaMKK1-1(syn)      MKKPGKLALPSQDSTIGKFLTQSGTFKDGDLLVNKDGLRIVPQSEEGEAPPIKPLDNNHQ LSIDDLDSIKVIGKGNSGTVQLVRHKWTGQFFALKVIQLNIQESIRKQMAQELKISLFTQ
                   ************************************************** ********* ************************************************************

TaMKK1-1(7AS)      CQYVVTCYQCFYVNGVISIALEYMDGGSLADFLKAVRTVPEAYLAAICKQVLKGLMYLHH EKRVIHRDLKPSNILINHRGEVKISDFGVSAIISSSSAQRDTFTGTFNYMAPERISGQKH
TaMKK1-1(4AL)      CQYVVTCYQCFYVNGVISIALEYMDGGSLADFLKAVRTVPEAYLAAICKQVLKGLMYLHH EKRVIHRDLKPSNILINHRGEVKISDFGVSAIISSSSAQRDTFTGTFNYMAPERISGQKH
TaMKK1-1(7DS)      CQYVVTCYQCFYVNGVISIALEYMDGGSLADFLKAVRTVPEAYLAAICKQVLKGLMYLHH EKRVIHRDLKPSNILINHRGEVKISDFGVSAIISSSSAQRDTFTGTFNYMAPERISGQKH
TaMKK1-1(syn)      CQYVVTCYQCFYVNGVISIALEYMDGGSLADFLKAVRTVPEAYLAAICKQVLKGLMYLHH EKRVIHRDLKPSNILINHRGEVKISDFGVSAIISSSSAQRDTFTGTFNYMAPERISGQKH
                   ************************************************************ ************************************************************

TaMKK1-1(7AS)      GYMSDIWSLGLVMLECATGNFPYPPRESFYELLEAVVDQPSPSAPSDQFSPEFCSFISAC IQKNAADRSSAQTLSAHPFLSMYDDLNIDLSDYFTTAGSPLATFKQIAL
TaMKK1-1(4AL)      GHLSDIWSLGLVMLECATGNFPYPPRESFYELLEAVVDQPSPSAPSDQFSPEFCSFISAC IQKNAADRSSAQTLSAHPFLSMYDDLNIDLSDYFTTAGSPLATFKQIAL
TaMKK1-1(7DS)      GHLSDIWSLGLVMLECATGNFPYPPRESFYELLEAVVDQPSPSAPSDQFSPEFCSFISAC IQKNAADRSSAQTLSVHPFLSMYDDLNIDLSDYFRTAGSPLATFKQIAL
TaMKK1-1(syn)      GHLSDIWSLGLVMLECATGNFPYPPRESFYELLEAVVDQPSPSAPSDQFSPEFCSFISAC IQKNAADRSSAQTLSVHPFLSMYDDLNIDLSDYFTTAGSPLATFKQIAL
                   *::********************************************************* **************.***************** ****************

1: TaMKK1-1_7AS_  100.00   99.14   98.85   99.14
2: TaMKK1-1_4AL_   99.14  100.00   99.14   99.43
3: TaMKK1-1_7DS_   98.85   99.14  100.00   99.71
4: TaMKK1-1_syn_   99.14   99.43   99.71  100.00
```

TaMKK1-1(syn) protein sequences used for Y2H interactions (blue font) did not match a nucleotide identifier from Additional File 3. Sequence was aligned with Ensembl sequences encoding TaMKK1-1 using Clustal Omega (http://www.ebi.ac.uk/Tools/msa/clustalo/). Percent identity of aligned sequences are also provided.

```
TaMKK3-2(clone)     MAGLEELKKKLQPLLFDDSDKGGVSTRVPFPEDTCDSYVVSDGGTINLLSRSFGEYNINEHGFHKRSTGPEEPDTGEKAYRCASEDMHIFGPIGSGASSVVQRAIFIPVHRILALKKINI
TaMKK3-2(5BL)       MAGLEELKKKLQPLLFDDSDKGGVSTRVPFPEDTCDSYVVSDGGTINLLSRSFGEYNINEHGFHKRSTGPEEPDTGEKAYRCASEDMHIFGPIGSGASSVVQRAIFIPVHRILALKKINI
TaMKK3-2(5DL)       MSGLEELKKKLQPLLFDDSDKGGVSTRVPFPEDTCDSYVVSDGGTINLLSRSFGEYNINEHGFHKRSTGPEEPDTGEKAYRCASEDMHIFGPIGNGASSVVQRAIFIPVHRILALKKINI
                    *:**********************************************************************************************.*************************

TaMKK3-2(clone)     FEKEKRQQILNEMRTLCEASCYPGLVEFQGAFYMPDSGQISIALEYMDGGSLADVIRVKKSIPEPVLAHMLLKVLLGLKYLHEVRHLVHRDLKPANLLVKLKGEAKITDFGVSAGLDNTM
TaMKK3-2(5BL)       FEKEKRQQILNEMRTLCEASCYPGLVEFQGAFYMPDSGQISIALEYMDGGSLADVIRVKKSIPEPVLAHMLLKVLLGLKYLHEVRHLVHRDLKPANILVNLKGEAKITDFGVSAGLDNTM
TaMKK3-2(5DL)       FEKEKRQQILNEMRTLCEASCYPGLVEFQGAFYMPDSGQISIALEYMDGGSLADVIRVKKSIPEPVLAHMLLKVLLGLKYLHEVRHLVHRDLKPANILVNLKGEAKITDFGVSAGLDNTM
                    ************************************************************************************************:**:********************

TaMKK3-2(clone)     AMCATFVGTVTYMSPERIRNENYSYAADIWSLGLTILECATGKFPYNVNEGPANLMLQILDDPSPTPPEDAYTPEFCSFINDCLRKDADARPTCEQLLSHAFIKRYEQTGVDLATYVRGV
TaMKK3-2(5BL)       AMCATFVGTVTYMSPERIRNENYSYAADIWSLGLTILECATGKFPYNVNEGPANLMLQILDDPSPAPPEDAYTPEFCSFINDCLRKDADARPTCEQLLSHPFIKRYEQTGVDLAAYVRGV
TaMKK3-2(5DL)       AMCATFVGTVTYMSPERIRNENYSYAADIWSLGLTILECATGKFPYNVNEGPANLMLQILDDPSPAPPEDAYTPEFCSFINDCLRKYADARPTCEQLLSHPFIKRYEQTGVDLAAYVRGV
                    *****************************************************************:******************** ************* *************:*****

TaMKK3-2(clone)     VNPTERLKQIAEMLAVHYYLLFNGSEGPWNYMKTFYKEESSFSFSGNVYVGQSAIFDTLSNIRKKLKGDRPREKIVHVVEKLHCRANGETEIAIRVSGSFITGNQFLIFGEGLQAEGMPS
TaMKK3-2(5BL)       VNPTERLKQIAEMLAVHYYLLFNGSEGPWNHMKTFYREESSFSFSGNVYVGQSAIFDTLSNIRKKLKGDRPREKIVHVVEKLHCRANGETEIAIRVSGSFITGNQFLIFGEGLQAEGMPS
TaMKK3-2(5DL)       VNPTERLKQIAEMLAVHYYLLFNGSEGPWDHMKTFYREESSFSFSGNVYVGQSAIFDTLSNIRKKLKGDRPREKIVHVVEKLHCRANGETEIAIRVSGSFITGNQFLISGEGLQAEGMPS
                    *****************************::*****:*********************************************************************** ***********

TaMKK3-2(clone)     LEEIDIDIPSKRVGQFREQFTVHPGTSMGCYYIARQDLYIIQA
TaMKK3-2(5BL)       LDGIDIDIPSKRVGQFREQFTVHPGTSMGCYYIAKQDLYIVQS
TaMKK3-2(5DL)       LDEINIDIPSKRVGQFREQFTVLPGTSMGCYYIAKQDLYIVQS
                    *: *:***************** ***********:*****:*:
```

```
1: TaMKK3-2_clone_  100.00   97.71   96.56
2: TaMKK3-2_5BL_     97.71  100.00   98.47
3: TaMKK3-2_5DL_     96.56   98.47  100.00
```

TaMKK3-2(clone) protein sequences used for Y2H interactions (blue font) did not match a nucleotide identifier from Additional File 3. Sequence was aligned with Ensembl sequences encoding TaMKK3-2 using Clustal Omega (http://www.ebi.ac.uk/Tools/msa/clustalo/). Percent identity of aligned sequences are also provided.

```
TaMKK3-3(5AL)      MAGLEDLKRRLQPIFFDADGNVVPPPAADGTSDDSSSDDCEVLDSGTVNLLSRSSDEYNI SKLGFHKRTTRPDGDYATDKAYRCSCHDMHIFDSVGIGASSVVHRAIYVPVHRVLALKKI
TaMKK3-3(5BL)      MAGLEDLKRRLQPIFFDADGNVVPPPAADGTSDDSSSDDCEVLDGGTVNLLSRSSDEYNI SKLGFHKRTTRPDGDYATDKAYRCSCHDMHIFDSVGNGASSVVYRAIYVPVHRVLALKKI
TaMKK3-3(syn)      MAGLEDLKRRLQPIFFDADGNVVPPPAADGTSDDSSSDDCEVLDGGTVNLLSRSSDEYNI SKLGFHKRTTRPDGDYATDKAYRCCCHDMHIFDSVGNGASSVVHRAIYVPVHRVLALKKI
TaMKK3-3(5DL)      MAGLEELKRRLQPIFFDADGNVVPPPAADGTSDDSSSDDCEVLDGGTVNLLSRSSDEYNI SKLGFHKRTTRPDGDYATDKAYRCCCHDMHIFDSVGNGASSVVHRAIYVPVHRVLALKKI
                   *****:**************************************.*************** ************************.*********** ******:****************

TaMKK3-3(5AL)      SIFDKEKRQQILNEITTLSAASCYPGLVEFQGIFYTPDSGEIYFALEYMDGGSLADIIRV KKFITEPVLSHMLQKVLLALRYLHEVRRLVHRDIKPANLLLNLKRDTKITDFGVTSGLHD
TaMKK3-3(5BL)      NIFDKERRQQILNEIRTLSAASCYPGLVEFQGVFYTPDSGEIYFALEYMDGGSLADIIRV KKFITEPVLSHMLQKVLLALRYLHEVRRLVHRDIKPANLLLNLKGDTKITDFGVTSGLHD
TaMKK3-3(syn)      NIFDKERRQQILNEIRTLSAASCYPGLVEFQGVFYTPDSGEIYFALEYMDGGSLADIIRV KKFITEPVLSHMLQKVLLALRYLHEVRRLVHRDIKPANLLLNLKGDTKITDFGVSSGLHD
TaMKK3-3(5DL)      NIFDKEKRQQILNEIRTLSAASCYPGLVEFQGVFYTPDSGEIYFALEYMDGGSLADIIRV KKFITEPVLSHMLQKVLLALRYLHEVRRLVHRDIKPANLLLNLKGDTKITDFGVSSGLHD
                   .*****:******** ****************:*************************** ******************************************** *********:*****

TaMKK3-3(5AL)      SVTMCATFLGSVTYMSPERIRNENYSYAADIWSLGLTALECATGRYPYDVNGGEADLMLQ ILEDPSPTPPQHMHSEEFCSFIDACLQKDADARPTCNELLSHSFIKKYEGPGVDLAEYNK
TaMKK3-3(5BL)      SVTMCATFLGSVTYMSPERIRNENYSYAADIWSLGLTALECATGRYPYDVNGGEADLMLQ ILEDPSPTPPQHMHSEEFCLFIDACLQKDADARPTCDELLSHSFIKKYEGPGVDLAEYNR
TaMKK3-3(syn)      SVTMCATFLGSVTYMSPERIRNENYSYAADIWSLGLTALECATGRYPYDVNGGEADLMLQ ILEDPSPTPPQHMHSEEFCSFIDACLQKDADARPTCDQ--------KYEGPGVDLAEYNK
TaMKK3-3(5DL)      SVTMCATFLGSVTYMSPERIRNENYSYAADIWSLGLTALECATGRYPYDVNGGEADLMLQ ILEDPSPTPPQHMHSEEFCSFIDACLQKDADARPTCDQLLSHSFIKKYEGPGVDLAEYNK
                   ************************************************************ ******************* ****************::        *************:

TaMKK3-3(5AL)      SVHDPAERLSQIAHMLAVHYYLIF--------HMKSFYGQDSTFSFSGETHVGKSDIFDT LSRIREMLKGNSRCEKIGRVMEKVYCRAHWEEGMSVRVSGSFIMGNEFLVCADGFCAEGM
TaMKK3-3(5BL)      SVHDPAERLSQIAHMLAVHYYLIFDGGDDQWHHMKSFYGQDSTFSFSGETHVGKSDIFDT LSRIREMLKGNSRCEKIGRVMEKVY-RAHGEEGTSVRVSGSFIMGNEFLVCADGFCAEGM
TaMKK3-3(syn)      SVHDPAERLSQIAHMLAVHYYLIFDGGDDQWRHMKSFYGQDSTFSFSGETHVGKSDIFDT LSRIREMLKGNSRCEKIGRVMEKVYCRAHGEEGMSVRVSGSFIMGNEFLVCADGFCAEGM
TaMKK3-3(5DL)      SVHDPAERLSQIAHMLAVHYYLIFDGGDDQWRHMKSFYGQDSTFSFSGETHVGKSDIFDT LSRIREMLKGNSRCEKIGRVMEKVYCRAHGEEGMSVRVSGSFIMGNEFLVCADGFCAEGM
                   ************************        **************************** ************************* *** *** **************************

TaMKK3-3(5AL)      LSMVELSPDILSKQAGHFQEDFFMEPGTAMGCYVISKQELHIGVS
TaMKK3-3(5BL)      LSIVELSPDILSKQAGHFQEDFFMEPGTAMGCYVISRQELHIGVS
TaMKK3-3(syn)      LSIVELSPDILSKQAGHFQEDFFMEPGTAMGCYVISRQELHIGVS
TaMKK3-3(5DL)      LSIVELSPDILSKQAGHFQEDFFMEPGTAMGCYVISRQELHIGVS
                   **:*********************************:********

1: TaMKK3-3_5AL_  100.00   97.09   97.25   97.29
2: TaMKK3-3_5BL_   97.09  100.00   98.45   98.09
3: TaMKK3-3_syn_   97.25   98.45  100.00   99.61
4: TaMKK3-3_5DL_   97.29   98.09   99.61  100.00
```

TaMKK3-3(syn) protein sequences used for Y2H interactions (blue font) did not match a nucleotide identifier from Additional File 3. Sequence was aligned with Ensembl sequences encoding TaMKK3-3 using Clustal Omega (http://www.ebi.ac.uk/Tools/msa/clustalo/). Percent identity of aligned sequences are also provided.

```
TaMKK4(6DL)      MRPGGPPNARPGLQQQQPGTPGRARRRPDLTLPLPQRDLTSLAVPLPLPPPPSSAPSSTS SS----GSGGASSMPMPMSMTPPNSAGSTPPAPPPLGELERVRRVGSGAGGTVWLVRHAP
TaMKK4(syn)      MRPGGPPNARPGLQQQQPGTPGRARRRPDLTLPLPQRDLTSLAVPLPLPPPPSSAPSSTS SSGSGSGSGGASSMPMPMSMTPPNSAGSAPPAPPPLGELERVRRVGSGAGGTVWLVRHAP
TaMKK4(6AL)      MRPGGPPNARPGLQQQQPGTPGRARRRPDLTLPLPQRDLTSLAVPLPLPPPPSSAPSSTS SSGSGSGSGGASSMPMPMSMTPPNSAGSAPPAPPPLGELERVRRVGSGAGGTVWLVRHAP
TaMKK4(6BL)      MRPGGPPNARPGLQQQQPGTPGRARRRPDLTLPLPQRDLTSLAVPLPLPPPPSSAPSSTS SSGSGSGSGGASSMPMPMSMTPPNSAGSAPPAPPPLGELERVRRVGSGAGGTVWLVRHAP
                 ************************************************************ **    **********************:*******************************

TaMKK4(6DL)      TGRAYALKVLYGHHDEAVRRQITREIAILRTAEHPSIVRCHGMYEQAGELQILLEYMDGG SLDGRRIASESFLADVARQVLSGIAYLHRRHIVHRDIKPSNLLIDCARRVKIADFGVGRI
TaMKK4(syn)      TGRAYALKVLYGHHDEAVRRQITREIAILRTAEHPSIVRCHGMYEQAGELQILLEYMDGG SLDGRRIASESFLADVARQVLSGIAYLHRCHIVHRDIKPSNLLIDCGRRVKIADFGVGRI
TaMKK4(6AL)      TGRAYALKVLYGHHDEAVRRQITREIAILRTAEHPSIVRCHGMYEQAGELQILLEYMDGG SLDGRRIASESFLADVARQVLSGIAYLHRCHIVHRDIKPSNLLIDCGRRVKIADFGVGRI
TaMKK4(6BL)      TGRAYALKVLYGHHDEAVRRQITREIAILRTAEHPSIVRCHGMYEQAGELQILLEYMDGG SLDGRRIASEAFLADVARQVLSGIAYLHRRHIVHRDIKPSNLLIDSGRRVKIADFGVGRI
                 ************************************************************ **********:****************** ***************..*************

TaMKK4(6DL)      LNQTMDPCNSSVGTIAYMSPERINTDLNDGNYNGYAGDIWSFGLSILEFYLGRFPLGENL KQGDWAALMCAICYSESPAAPPTASPELRSFISCCLQKNPAKRPSAAQLLQHRFIASPP
TaMKK4(syn)      LNQTMDPCNSSVGTIAYMSPERINTDLNDGNYNGYAGDIWSFGLSILEFYLGRFPLGENL GKQGDWAALMCAICYSESPAAPPTASPELRSFISCCLQKEPAKAPSAAQLLQHRFIASPP
TaMKK4(6AL)      LNQTMDPCNSSVGTIAYMSPERINTDLNDGNYNGYAGDIWSFGLSILEFYLGRFPLGENL GKQGDWAALMCAICYSESPAAPPTASPELRSFISCCLQKNPAKRPSAAQLLQHRFIASPP
TaMKK4(6BL)      LNQTMDPCNSSVGTIAYMSPERINTDLNDGNYNGYAGDIWSFGLSILEFYLGRFPLGENL GKQGDWAALMCAICYSESPAAPPTASPELRSFISCCLQKNPAKRPSAAQLLQHRFIASPP
                 ************************************************************  **************************************:*** ****************

TaMKK4(6DL)      QQQPQALAAPPC
TaMKK4(syn)      QQQPQVLAAPPC
TaMKK4(6AL)      PQQPQALAAPPC
TaMKK4(6BL)      QQQPQVLAAPPC
                  ****.******
```

1: TaMKK4_6DL_	100.00	98.37	98.91	98.64
**2: TaMKK4_syn_**	**98.37**	**100.00**	**98.92**	**98.66**
3: TaMKK4_6AL_	98.91	98.92	100.00	98.66
4: TaMKK4_6BL_	98.64	98.66	98.66	100.00

TaMKK4(syn) protein sequences used for Y2H interactions (blue font) did not match a nucleotide identifier from Additional File 3. Sequence was aligned with Ensembl sequences encoding TaMKK4 using Clustal Omega (http://www.ebi.ac.uk/Tools/msa/clustalo/). Percent identity of aligned sequences are also provided.

```
TaMKK5b(syn)      MRPAGGS--LPA--QPGTPGRPRRRPDLTLPMPQRPDVSSSLAVPLPAPSPDRRSPRRR----PRPAARGPARAPPPLHELERVRRVGSGAGGTVWMVRHRATGRCYALKVLYGNHDDTV
TaMKK5(7DS)       MRPAGGGSSLPAQPGPGTPGRPRRRPDLTLPMPQRPDVSSSLAVPLPLPPPTAAPPGA-GPAQPLVAPP---APPPPLHELERVRRVGSGAGGTVWMVRHRATGRCYALKVLYGNHDDTV
TaMKK5a(syn)      MRPAGAG--LPA--QPGTPGRPRRRPDLTLPMPQRPDVSSSLAVPLPLPPPTAAPPGRRPA---ARGPARGDPAPPPLHELERVRRVGSGAGGTVWMVRHAPTGRCYALKVLYGNHDDAV
TaMKK5(7AS)       MRPAGGS--LPA--QPGTPGRPRRRPDLTLPMPQRPDVSSSLAVPLPLPPPTAAPPTGAGPAQPLVAPPAAAPPPPPLHELERVRRVGSGAGGTVWMVRHRATGRCYALKVLYGNHDDAV
TaMKK5(7BS)       MRPAGIS--LPA--QPGTPGRPRRRPDLTLPMPQRPDVSSSLAVPLPLPPPTAAPP---GAAQPLVAPPAATPPPPPLHELERVRRVGSGAGGTVWMVRHRATGRCYALKVLYGNHDDAV
                  ***** .  ***   ******************************** * *    *          .       **************************  ****************:*

TaMKK5b(syn)      RRQIAREIAILRTAEHPAVVRCHGMYERGGELQILLEYMDGGSLDGRRIADEPFLAHVARQVLSGIAYLHRRHIVHRDIKPSNLLIDSARRVKIADFGVGRILNQTMDPCNSSVGTIAYM
TaMKK5(7DS)       RRQIAREIAILRTAEHPAVVRCHGMYERGGELQILLEYMDGGSLDGRRIADEPFLAHVARQVLSGIAYLHRRHIVHRDIKPSNLLIDSSRRVKIADFGVGRILNQTMDPCNSSVGTIAYM
TaMKK5a(syn)      RRQIAREIAILRTAEHPAVVRCHGMYERGGELQILLEYMDGGSLDGRRIADEPFLAHVARQVLSGIAYLHRRHIVHRDIKPSNLLIDSSRRVKIADFGVGRILNQTMDPCNSSVGTIAYM
TaMKK5(7AS)       RRQIAREIAILRTAEHPAVVRCHGMYERGGELQILLEYMDGGSLDGRRIADEPFLAHVARQVLSGIAYLHRRHIVHRDIKPSNLLIDSARRVKIADFGVGRILNQTMDPCNSSVGTIAYM
TaMKK5(7BS)       RRQIAREIAILRTAEHPAVVRCHGMYERGGELQILLEYMDGGSLDGRRIADEPFLAHVARQVLSGIAYLHRRHIVHRDIKPSNLLIDSARRVKIADFGVGRILNQTMDPCNSSVGTIAYM
                  ****************************************************************************************:*******************************

TaMKK5b(syn)      SPERINTDINDGAYDGYAGDIWSFGLSILEFYLGRFPFGENLGRQGDWAALMVAICYSDPPEPSPATASPEFRGFIACCLQKNPANRLSAAQLLQHPFVALPQPQPLAAPPS-
TaMKK5(7DS)       SPERINTDINDGAYDGYAGDIWSFGLSILEFYLGRFPFGENLGRQGDWAALMVAICYSDPPEPSPATASPEFRGFIACCLQKNPANRLSAAQLLQHPFVALPQPQPLAAPPSS
TaMKK5a(syn)      SPERINTDINDGAYDGYAGDIWSFGLSILEFYLGRFPFGENLGRQGDWAALMVAICYSDPPEPSPATASPEFRGFIACCLQKNPANRLSAAQLLQHPFVALPQPQPLAAPPSS
TaMKK5(7AS)       SPERINTDINDGAYDGYAGDIWSFGLSILEFYLGRFPFGENLGRQGDWAALMVAICYSDPPEPSPATASPEFRGFIACCLQKNPANRLSAAQLLQHPFVALPQPQPLAAPPSS
TaMKK5(7BS)       SPERINTDINDGAYDGYAGDIWSFGLSILEFYLGRFPFGENLGRQGDWAALMVAICYSDPPEPSPATASPEFRGFIACCLQKNPANRLSAAQLLQHPFVALPQPQPLAAPPS-
                  ****************************************************************************************************************

1: TaMKK5b_syn_  100.00   95.00   93.59   94.77   95.01
2: TaMKK5_7DS_    95.00  100.00   95.91   97.97   97.95
3: TaMKK5a_syn_   93.59   95.91  100.00   94.80   95.91
4: TaMKK5_7AS_    94.77   97.97   94.80  100.00   99.13
5: TaMKK5_7BS_    95.01   97.95   95.91   99.13  100.00
```

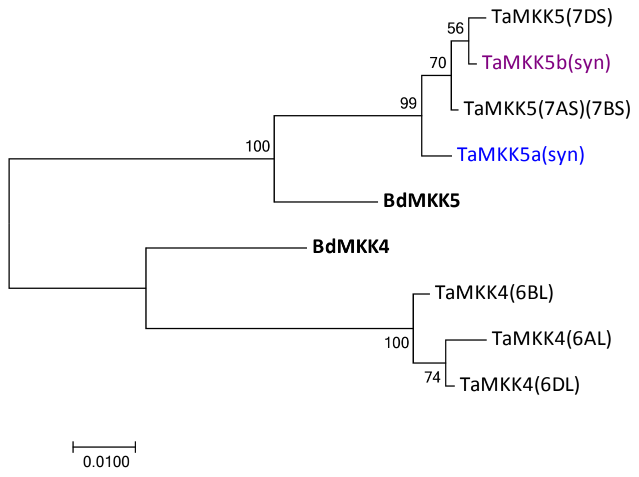

TaMKK5a(syn) and TaMKK5b(syn) protein sequences used for Y2H interactions (blue font) did not match a nucleotide identifier from Additional File 3. Sequence was aligned with Ensembl sequences encoding TaMKK5 using Clustal Omega (http://www.ebi.ac.uk/Tools/msa/clustalo/). Percent identity of aligned sequences are also provided. Since percent identity is low for synthetic TaMKK5s compared with variability with genomic copies, the phylogenetic analysis (Clade C MKK branch shown above) shows relationship between TaMKK5a and 5b with MKK5.

```
TaMKK10-2(syn)      MALVRQRRQLPHLTLPLDHFALRAPPPPP-PAVAP---CEGLARLSDYERISQLGQGNGG TVFKARHRRTAQQVALKLFA----DPSAAREAEILMLASGAPHVVRLHAVIPSPAAEQPA
TaMKK10-2(4AS)      MALVRQRRQLPHLTLPLDHFALRAPPPPLPPAVAPCAPAEGLARLSDYERISQLGQGNGG TVFKARHRRTAQHFALKLFAAGDGDPSAAREAEILMLASGAPHVVRLHAVIPSPAAEQPA
TaMKK10-2(4BL)      MALVRQRRQLPHLTLPLDHFALRAPPP-PPPAVAPC---EGLARLSDYERISQLGQGNGG TVFKARHRRTAQQVALKLFAAGDGDPSAAREAEILMLASGAPHVVRLHAVIPSPAAEQPA
TaMKK10-2(4DL)      MALVRQRRQLPHLTLPLDHFALRAPPPPPPPAVAPC---EGLARLSDYERISQLGQGNGG TVFKARHRRTAQQVALKLFAAGDGDPSAAREAEILMLASGAPHVVRLHAVIPSPAAEQPA
                    ***************************   *****    ********************* ************:.******    ************************************

TaMKK10-2(syn)      ALASSSVSGGSLAGLLRRLGRPMGERPIAAVARQALLGLAALHALRVVHRDLKPANLLVG AGGEVKIADFGAGKVLRRRLDPCASYVGTAAYMSPERFDPEAYSGDYDPYAADVWSLGMA
TaMKK10-2(4AS)      ALALELVSGGSLAGLLRALGRPMGERPIAAVARQALLGLAALHALRVVHRDLKPANLLVG AGGEVKIADFGAGKVLRRRLDPCASYVGTAAYMSPERFDPEAYSGDYDPYAADVWSLGMA
TaMKK10-2(4BL)      ALALELVSGGSLAGLLRALGRPMGERPIAAVARQALLGLAALHALRVVHRDLKPANLLVG AGGEVKIADFGAGKVLRRRLDPCASYVGTAAYMSPERFDPETYSGDYDPYAADVWSLGMA
TaMKK10-2(4DL)      ALALELVSGGSLAGLLRALGRPMGERPIAAVARQALLGLAALHALRVVHRDLKPANLLVG AGGEVKIADFGAGKVLRRRLDPCASYVGTAAYMSPERFDPEAYSGDYDPYAADVWSLGMA
                    *** . *********** ****************************************** *****************************************:******************

TaMKK10-2(syn)      ILELYLGHFPLLPAGQRPDWAALMCAICFGEAPEAPAAASDEFRDFVARCLEKKAGRRAS VAELLQHPFIAERDAEEAQRCLAALVAEAAELGDQ
TaMKK10-2(4AS)      ILELYLGHFPLLPAGQRPDWAALMCAICFGEAPEAPAAASDEFRDFVARCLEKKAGRRAS VAELLQHPFIAERDAEEAQRCLAALVAEAAELGDQ
TaMKK10-2(4BL)      ILELYLGHFPLLPAGQRPDWAALMCAICFGEAPEAPAAASDEFRDFVARCLEKKAGRRAS VAELLGHPFIAERDAEEAQRCLAALVAEAAELGDQ
TaMKK10-2(4DL)      ILELYLGHFPLLPAGQRPDWAALMCAICFGEAPEAPAAASDEFRDFVARCLEKKAGRRAS VAELLQHPFIAERDAEEAQRCLAALVAEAAELGDQ
                    ************************************************************ ***** *****************************
```

```
1: TaMKK10-2_syn_  100.00   97.55   98.15   98.77
2: TaMKK10-2_4AS_   97.55  100.00   98.49   99.10
3: TaMKK10-2_4BL_   98.15   98.49  100.00   99.40
4: TaMKK10-2_4DL_   98.77   99.10   99.40  100.00
```

TaMKK10-2(syn) protein sequences used for Y2H interactions (blue font) did not match a nucleotide identifier from Additional File 3. Sequence was aligned with Ensembl sequences encoding TaMKK10-2 using Clustal Omega (http://www.ebi.ac.uk/Tools/msa/clustalo/). Percent identity of aligned sequences are also provided.

```
TaMPK4(clone)     MDTS-GGGTAAGGAAQIQGMATHGGRYVLYNVYGNLFEVASKYAPPIRPIGRGAYGIVCA AVSSDTGEEVAIKKIGNAFDNHIDAKRTLREIKLLRHMDHENILAIKDLIRPPRRDDFKD
TaMPK4(1AL)       MDTSGGGGGAAGGAAQIQGMATHGGRYVLYNVYGNLFEVASKYAPPIRPIGRGAYGIVCA AVSSDTGEEVAIKKIGNAFDNHIDAKRTLREIKLLRHMDHENILAIKDLIRPPRRDDFKD
TaMPK4(1BL)       MDTS-GGGTAAGGAAQIQGMATHGGRYVLYNVYGNLFEVASKYAPPIRPIGRGAYGIVCA AVSSDTGEEVAIKKIGNAFDNHIDAKRTLREIKLLRHMDHENILAIKDLIRPPRRDDFKD
TaMPK4(1DL)       MDTS-GGGAAAGGAAQIQGMATHGGRYVLYNVYGNLFEVASKYAPPIRPIGRGAYGIVCA AVSSDTGEEVAIKKIGNAFDNHIDAKRTLREIKLLRHMDHENILAIKDLIRPPRRDDFKD
                  **** *** *************************************************** ************************************************************

TaMPK4(clone)     VYIVTELMDTDLHQIIRSNQSLTDDHCQYFLYQLLRGLKYVHSANVLHRDLKPSNLFLNA NCDLKIADFGLARTTSETDLMTEYVVTRWYRAPELLLNCSQYTAAIDVWSVGCILGEIIT
TaMPK4(1AL)       VYIVTELMDTDLHQIIRSNQSLTDDHCQYFLYQLLRGLKYVHSANVLHRDLKPSNLFLNA NCDLKIADFGLARTTSETDLMTEYVVTRWYRAPELLLNCSQYTAAIDVWSVGCILGEIIT
TaMPK4(1BL)       VYIVTELMDTDLHQIIRSNQSLTDDHCQYFLYQLLRGLKYVHSANVLHRDLKPSNLFLNA NCDLKIADFGLARTTSETDLMTEYVVTRWYRAPELLLNCSQYTAAIDVWSVGCILGEIIT
TaMPK4(1DL)       VYIVTELMDTDLHQIIRSNQSLTDDHCQYFLYQLLRGLKYVHSANVLHRDLKPSNLFLNA NCDLKIADFGLARTTSETDLMTEYVVTRWYRAPELLLNCSQYTAAIDVWSVGCILGEIIT
                  ************************************************************ ************************************************************

TaMPK4(clone)     RQPLFPGRDYIQQLKLITELIGSPDDSSLGLLRSDNARRYMKQLPQYPRQDFRLRFRNMS DGAVDLLERMLVFDPSRRITVDEALHHPYLASLHDINEEPTCPAPFSFDFEQPSFTEEHM
TaMPK4(1AL)       RQPLFPGRDYIQQLKLITELIGSPDDSSLGFLRSDNARRYMKQLPQYPRQDFRLRFRNMS DGAVDLLERMLVFDPSRRITVDEALHHPYLASLHDINEEPTCPAPFSFDFEQPSFTEEHM
TaMPK4(1BL)       RQPLFPGRDYIQQLKLITELIGSPDDSSLGFLRSDNARRYMKQLPQYPRQDFRLRFRNMS DGAVDLLERMLVFDPSRRITVDEALHHPYLASLHDINEEPTCPAPFSFDFEQPSFTEEHM
TaMPK4(1DL)       RQPLFPGRDYIQQLKLITELIGSPDDSSLGFLRSDNARRYMKQLPQYPRQDFRLRFRNMS DGAVDLLERMLVFDPSRRITVDEALHHPYLASLHDINEEPTCPAPFSFDFEQPSFTEEHM
                  ******************************:***************************** ************************************************************

TaMPK4(clone)     KELIWRETLAFNPEILPT
TaMPK4(1AL)       KELIWRETLAFNPDPPY-
TaMPK4(1BL)       KELIWRETLAFNPDPPY-
TaMPK4(1DL)       KELIWRETLAFNPDPPY-
                  *************:

1: TaMPK4_clone_  100.00   98.40   98.67   98.40
2: TaMPK4_1AL_     98.40  100.00   99.73   99.73
3: TaMPK4_1BL_     98.67   99.73  100.00   99.73
4: TaMPK4_1DL_     98.40   99.73   99.73  100.00
```

TaMPK4(clone) protein sequences used for Y2H interactions (blue font) did not match a nucleotide identifier from Additional File 3. Sequence was aligned with Ensembl sequences encoding TaMPK4 using Clustal Omega (http://www.ebi.ac.uk/Tools/msa/clustalo/). Percent identity of aligned sequences are also provided.

```
TaMPK17(clone)      MGGGNGIVDGFRRSFHRRTPSGSVLGSSNQSSAGEDSSDVEAAEGLDLVGLRSIRVPKRK MPLPVESHKKNIMEKEFFTEYGEASQYQIQEVVGKGSYGVVAAAIDTRTGERVAIKKIND
TaMPK17(7DL)        MGGGNGIVDGFRRLFHRRTPSGSVLGSSNQSSAGEDSSDVEAAEGLDLVGLRSIRVPKRK MPLPVESHKKNIMEKEFFTEYGEASQYQIQEVVGKGSYGVVAAAIDTRTGERVAIKKIND
TaMPK17(7AL)        MGGGNGIVDGFRRLFHRRTPSGSVLGSSNQSSAGEDSSDAEAAEDLDLVGLRPIRVPKRK MPLPVESHKKNIMEKEFFTEYGEASQYQIQEVVGKGSYGVVAAAIDTRTGERVAIKKIND
TaMPK17(U)          MGGGNGIVDGFRRLFHRRTPSGSVLGSSNQSSAGEDSSDVEAVEDLDLVGLRPIRVPKRK MPLPVESHKKNIMEKEFFTEYGEASQYQIQEVVGKGSYGVVAAAIDTRTGERVAIKKIND
                    ************* *************************.**.*.******* ******* ************************************************************

TaMPK17(clone)      VFEHVSDATRILREVKLLRLLRHPDVVEIKHIMLPPSRREFQDIYVVFELMESDLHQVIR ANDDLTAEHYQFFLYQLLRALKYIHGANVFHRDLKPKNILANADCKLKICDFGLARVSFN
TaMPK17(7DL)        VFEHVSDATRILREVKLLRLLRHPDVVEIKHIMLPPSRREFQDIYVVFELMESDLHQVIR ANDDLTAEHYQFFLYQLLRALKYIHGANVFHRDLKPKNILANADCKLKICDFGLARVSFN
TaMPK17(7AL)        VFEHVSDATRILREVKLLRLLRHPDVVEIKHIMLPPSRREFQDIYVVFELMESDLHQVIR ANDDLTAEHYQFFLYQLLRALKYIHGANVFHRDLKPKNILANADCKLKICDFGLARVSFN
TaMPK17(U)          VFEHVSDATRILREVKLLRLLRHPDVVEIKHIMLPPSRREFQDIYVVFELMESDLHQVIR ANDDLTAEHYQFFLYQLLRALKYIHGANVFHRDLKPKNILANADCKLKICDFGLARVSFN
                    ************************************************************ ************************************************************

TaMPK17(clone)      DAPSAIFWTDYVATRWYRAPELCGSFFSKYTPAIDIWSIGCIFAELLTGRPLFPGKNVVH QLDIITDLLGTPSSETLSRVRNEKARRYLSCMRKKHPVPLTQKFPNADPLAVRLLGRLLA
TaMPK17(7DL)        DAPSAIFWTDYVATRWYRAPELCGSFFSKYTPAIDIWSIGCIFAELLTGRPLFPGKNVVH QLDIITDLLGTPSSETLSRIRNEKARRYLSCMRKKHPVPLTQKFPNADPLAVRLLGRLLA
TaMPK17(7AL)        DAPSAIFWTDYVATRWYRAPELCGSFFSKYTPAIDIWSIGCIFAELLTGRPLFPGKNVVH QLDIITDLLGTPSSETLSRIRNEKARRYLSCMRKKHPVPLTQKFPNADPLAVRLLGRLLA
TaMPK17(U)          DAPSAIFWTDYVATRWYRAPELCGSFFSKYTPAIDIWSIGCIFAELLTGRPLFPGKNVVH QLDIITDLLGTPSSETLSRIRNEKARRYLSCMRKKHPVPLTQKFPNADPLAVRLLGRLLA
                    ************************************************************ *******************:****************************************

TaMPK17(clone)      FDPKDRPSAEEALADPYFASLANVEREPSRHPISKLEFEFERRKVTKDDVRELIYREILE YHPQMLEEYMKGGDQISFLYPSGVDRFKRQFAHLEEHYSKGERGSPLQRKHASLPRQRVG
TaMPK17(7DL)        FDPKDRPSAEEALADPYFASLANVEREPSRHPISKLEFEFERRKVTKDDVRELIYREILE YHPQMLEEYMKGGDQISFLYPSGVDRFKRQFAHLEEHYSKGERGSPLQRKHASLPRQRVG
TaMPK17(7AL)        FDPKDRPSAEEALADPYFASLANVEREPSRHPISKLEFEFERRKVTKDDVRELIYREILE YHPQMLEEYMKGGDQISFLYPSGVDRFKRQFAHLEEHYSKGERGSPLQRKHASLPRQRVG
TaMPK17(U)          FDPKDRPSAEEALADPYFASLANVEREPSRHPISKLEFEFERRKVTKDDVRELIYREILE YHPQMLEEYMKGGDQISFLYPSGVDRFKRQFAHLEEHYSKGERGSPLQRKHASLPRQRVG
                    ************************************************************ ************************************************************

TaMPK17(clone)      ASNDGNNEQHISDQEMSAEPDAHGAVSPQKSQDAPGVGQNGLSPTSLSSRTYLKSASISA SKCVVVNPNKQPEYDDAISEETEGAVDGLSEKVSKMHA
TaMPK17(7DL)        ASNDGNNEQHISDQEMSAEPDAHGTVSPQKSQDAAGVGQNGLSPTSLSSRTYLKSASISA SKCVVVNPNKQPEYDDAISEETEGAVDGLSEKVSKMHA
TaMPK17(7AL)        ASNDGNNEQHISDQEMSAEPDAHGAVSPQKSQDAPGVGQNGLSPTSLSSRTYLKSASISA SKCVVVNPNKQPEYDDAISEETEGAVDGLSEKVSKMHA
TaMPK17(U)          ASNDGNNEQHISDQEMSAEPDAHGAVSPQKSQDAPSVGPNGLSPTSLSSRTYLKSASISA SKCVVVNPNKQPEYDDAISEETEGAVDGLSEKVSKMHA
                    ************************:********* .** ********************* **************************************
```

**1:**	**TaMPK17_clone**	**100.00**	**99.31**	**99.13**	**98.79**
2:	TaMPK17_7DL	99.31	100.00	99.13	98.79
3:	TaMPK17_7AL	99.13	99.13	100.00	99.31
4:	TaMPK17_U	98.79	98.79	99.31	100.00

TaMPK7(clone) protein sequences used for Y2H interactions (blue font) did not match a nucleotide identifier from Additional File 3. Sequence was aligned with Ensembl sequences encoding TaMPK7 using Clustal Omega (http://www.ebi.ac.uk/Tools/msa/clustalo/). Percent identity of aligned sequences are also provided.

```
TaMPK20-2(clone)      MQQAQRSKGSAEADFFTEYGDANRYKIQEVIGKGSYGVVCSALDLQTRQKVAIKKIHNIF EHTSDAARILREIKLLRLLRHPDVVEIKHIMLPPSRKDFKDIYVVFELMESDLHQVIKAN
TaMPK20-2(1AL)        MQQAQRTKSSAEVDFFTEYGDANRYKIQEVIGKGSYGVVCSALDLQTRQKVAIKKIHNIF EHTSDAARILREIKLLRLLRHPDVVEIKHIMLPPSRKDFKDIYVVFELMESDLHQVIKAN
TaMPK20-2(1BL)        MQQAQRSKGSAEADFFTEYGDANRYKIQEVIGKGSYGVVCSALDLQTRQKVAIKKIHNIF EHTSDAARILREIKLLRLLRHPDVVEIKHIMLPPSRKDFKDIYVVFELMESDLHQVIKAN
TaMPK20-2(1DL)        MQQAQRSKSSAEVDFFTEYGDANRYKIQEVIGKGSYGVVCSALDLQTRQKVAIKKIHNIF EHTSDAARILREIKLLRLLRHPDVVEIKHIMLPPSRKDFKDIYVVFELMESDLHQVIKAN
                      ******:*.***.*********************************************** ************************************************************

TaMPK20-2(clone)      DDLTKEHYQFFLYQLLRALKYIHTASVYHRDLKPKNILANSNCKLKICDFGLARVAFNDT PTTVFWTDYVATRWYRAPELCGSFFTKYTPAIDIWSIGCIFAEVLTGKPLFPGKNAVHQL
TaMPK20-2(1AL)        DDLTKEHYQFFLYQLLRALKYIHTASVYHRDLKPKNILANSNCKLKICDFGLARVAFNDT PTTVFWTDYVATRWYRAPELCGSFFTKYTPAIDIWSIGCIFAEVLTGKPLFPGKNVVHQL
TaMPK20-2(1BL)        DDLTKEHYQFFLYQLLRALKYIHTASVYHRDLKPKNILANSNCKLKICDFGLARVAFNDT PTTVFWTDYVATRWYRAPELCGSFFTKYTPAIDIWSIGCIFAEVLTGKPLFPGKNVVHQL
TaMPK20-2(1DL)        DDLTKEHYQFFLYQLLRALKYIHTASVYHRDLKPKNILANSNCKLKICDFGLARVAFNDT PTTVFWTDYVATRWYRAPELCGSFFTKYTPAIDIWSIGCIFAEVLTGKPLFPGKNVVHQL
                      ************************************************************ *******************************************************.****

TaMPK20-2(clone)      DLMTDLLGTPSMDTISRVRNEKARRYLSSMRKKDPVPFSQKFPNADPLGVKLLEKLLAFD PKDRPTAEEALTDPYFRGLSKPEREPSCQPIRKMEFDFEHSRASKDDIRELIFQEILEYH
TaMPK20-2(1AL)        DLMTDLLGTPSMDTISRVRNEKARRYLSSMRKKDPVPFSQKFPNADPLGVKLLEKLLAFD PKDRPTAEEALTDPYFRGLSKPEREPSCQPIRKMEFDFEHSRVSKDDIRELIFQEILEYH
TaMPK20-2(1BL)        DLMTDLLGTPSMDTISRVRNEKARRYLSSMRKKDPVPFSQKFPNADPLGVKLLEKLLAFD PKDRPTAEEALTDPYFRGLSKPEREPSCQPIRKMEFDFEHSRVSKDDIRELIFQEILEYH
TaMPK20-2(1DL)        DLMTDLLGTPSMDTISRVRNEKARRYLSSMRKKDPVPFSQKFPNADPLGVKLLEKLLAFD PKDRPTAEEALTDPYFRGLSKPEREPSCQPIRKMEFDFEHSRVSKDDIRELIFQEILEYH
                      ************************************************************ ******************************************.*****************

TaMPK20-2(clone)      PQLLKSYVDGTERTTFLYPSAVDHFKKQFSHLEESDGSGPVVPADRKHASLPRSTIVHSA PISAKDTRPLFGKPCSKTSSETGRYAGNGRGASQASHAAQAVVSRRAAGSALPYDAASRP
TaMPK20-2(1AL)        PQLLKSYVDGTERTTFLYPSAVDHFKKQFSHLEESDGSGPVVPADRKHASLPRSTIVHSA PISAKDTRPLFGKPCSKTSSETGRYAGNGRGASQASHAAQAVVSRRAAGSALPYDAASRP
TaMPK20-2(1BL)        PQLLKSYTDGTERTTFLYPSAVDHFKKQFSHLEESDGSGPVVPADRKHASLPRSTIVHST PIPAKDTRPLFGKPCNKTSSETGRYAGNGHGASQASHAAQAVISRRAAGSALPYDAASRP
TaMPK20-2(1DL)        PQLLKSYIDGTERTTFLYPSAVDHFKKQFSHLEESDGSGPVVPADRKHASLPRSTIVHST PIPAKDTRPLFGKPCNKTSSETGRYAGNGHSASQASHAAQAVVSRRAAGSALPYDAASRP
                      ******* ***************************************************: ** ************.*************:.***********:*****************

TaMPK20-2(clone)      TISPGCPPQQQIPQMYGQYQHQAPA----GAGIPQAMGGYACGGYTKGTAPNAA-AAPAM RAPPYRHLPAGQKNGPLDRLAVETTDIYTRSLNGIVAAAAASAGTASAHRKVGAVPFGMPTTY
TaMPK20-2(1AL)        TISPGCPPQQQIPQMYGQYQHQAPA----GAGIPQAMGGYACGGYTKGTAPNAA-AAPAM RAPPYRHLPAGQKNGPLDRLAVETTDIYTRSLNGIVAAAAASAGTASAHRKVGAVPFGMPTTY
TaMPK20-2(1BL)        TISPGCPPQQQIPQMYGQYQHQAPPGAGAGAGIPQAMGGYACGGYTKGTAPNAAAAAPAM RAPPYRHLPTGQKNGPLDRLAVETTDIYTRSLNGIVAAAAASAGATGAHRKVGAVPFGMPTTY
TaMPK20-2(1DL)        TISPGCP-QQQIPQMYGQYQHQAAPGA--GAGIPQTMGGYACGGYTKGTAPNAA-AAPAM RAPPYRHLPAGQKNGPLDRLAVETTDIYTRSLNGIVAAAAASAGATSAHRKVGAVPFGMPTTY
                      ******* ***************      ******:****************** ***** *********:**********************************::.****************

1: TaMPK20-2_clone  100.00   99.16   97.83   97.49
2: TaMPK20-2_1AL     99.16  100.00   97.66   97.99
3: TaMPK20-2_1BL     97.83   97.66  100.00   98.50
4: TaMPK20-2_1DL     97.49   97.99   98.50  100.00
```

TaMPK20-2(clone) protein sequences used for Y2H interactions (blue font) did not match a nucleotide identifier from Additional File 3. Sequence was aligned with Ensembl sequences encoding TaMPK20-2 using Clustal Omega (http://www.ebi.ac.uk/Tools/msa/clustalo/). Percent identity of aligned sequences are also provided.

```
TaMPK20-3(7DL)        MQNNDLRKKSAAEIDFFTEYGDTNRYKVLEVIGKGSYGLVCSANDTQTGEKVAIKKIHNI FEHISDAARILREIKLLRLLRHPDVVEIKHILLPPSKKDFKDIYVVFELMESDLHQVIKA
TaMPK20-3(clone)      MQNNDLRKKSAAEIDFFTEYGDTNRYKVLEVIGKGSYGLVCSANDTQTGEKVAIKKIHNI FEHISDAARILREIKLLRLLRHPDVVEIKHILLPPSKKDFKDIYVVFELMESDLHQVIKA
TaMPK20-3(7AL)        MQNGDLRKKSAAEIDFFTEYGDTNRYKVLEVIGKGSYGLVCSANDTQTGEKVAIKKIHNI FEHISDAARILREIKLLRLLRHPDVVEIKHILLPPSKKDFKDIYVVFELMESDLHQVIKA
TaMPK20-3(7BL)        MQNSDLRKKSAAEIDFFTEYGDTNRYKVLEVIGKGSYGLVCSANDTQTGEKVAIKKIHNI FEHISDAARILREIKLLRLLRHPDVVEIKHILLPPSKKDFKDIYVVFELMESDLHQVIKA
                      ***.******************************************************** ************************************************************

TaMPK20-3(7DL)        NDDLTREHYQFFLYQMLRALKYMHTANVYHRDLKPKNVLANANCKLKICDFGLARVAFND APTTVFWTDYVATRWYRAPELCGSFYSKYTPAIDIWSIGCIFAEVLIGKPLFPGKNVVHQ
TaMPK20-3(clone)      NDDLTREHYQFFLYQMLRALKYMHTANVYHRDLKPKNVLANANCKLKICDFGLARVAFND APTTVFWTDYVATRWYRAPELCGSFYSKYTPAIDIWSIGCIFAEVLIGKPLFPGKNVVHQ
TaMPK20-3(7AL)        NDDLTREHYQFFLYQMLRALKYMHTANVYHRDLKPKNVLANANCKLKICDFGLARVAFND APTTVFWTDYVATRWYRAPELCGSFYSKYTPAIDIWSIGCIFAEVLIGKPLFPGKNVVHQ
TaMPK20-3(7BL)        NDDLTREHYQFFLYQMLRALKYMHTANVYHRDLKPKNVLANANCKLKICDFGLARVAFND APTTVFWTDYVATRWYRAPELCGSFYSKYTPAIDIWSIGCIFAEVLIGKPLFPGKNVVHQ
                      ************************************************************ ************************************************************

TaMPK20-3(7DL)        LDLITDVLGTPSLDAISQVRNDKARKYLTCMRKKQPASFSQKFPKADPLALRLLRRLLAF DPKDRPSAEEALADPYFNGLAKVEREPSCQPIPKLEFEFEGRRVTKEDIKELIFEEILEY
TaMPK20-3(clone)      LDLITDVLGTPSLDAISQVRNDKARKYLTCMRKKQPASFSQKFPKADPLALRLLRRLLAF DPKDRPSAEEALADPYFNGLAKVEREPSCQPIPKLEFEFEGRRVTKEDIKELIFEEILEY
TaMPK20-3(7AL)        LDLITDVLGTPSLDAISQVRNDKARKYLTCMRKKQPASFSQKFPKADPLALRLLRRLLAF DPKDRPSAEEALADPYFNGLAKVEREPSCQPIPKIEFEFEGRRVTKEDIKELIFEEILEY
TaMPK20-3(7BL)        LDLITDVLGTPSLDAISQVRNDKARKYLTCMRKKQPASFSQKFPKADPLALRLLRRLLAF DPKDRPSAEEALADPYFNGLAKVEREPSCQPIPKIEFEFEGRRVTKEDIKELIFEEILEY
                      ************************************************************ **********************************:*************************

TaMPK20-3(7DL)        HPQLLKEHISGTDRRNFVHLSAVDQFKKRFAELEENGGENGSAVSTQRKHSSLPRQSFNPR
TaMPK20-3(clone)      HPQLLKEHISGTDRRNFVHLSAVDQFKKRFAELEENGGENGSAVSSQRKHSSLPRQSFNPR
TaMPK20-3(7AL)        HPQLLKEHISGTDRRNFVHLSAVDQFKKRFAELEENGGENGSAVSSQRKHSSLPRQSFNPH
TaMPK20-3(7BL)        HPQLLKEHISGTDRRNFVHLSAVDQFKKRFAELEENGGENGSAVSSQRKHSSLPRQSFNPE
                      *********************************************:**************:
```

```
1: TaMPK20-3_7DL     100.00   99.76   99.05   99.29
2: TaMPK20-3_clone    99.76  100.00   99.29   99.52
3: TaMPK20-3_7AL      99.05   99.29  100.00   99.52
4: TaMPK20-3_7BL      99.29   99.52   99.52  100.00
```

TaMPK20-3(clone) protein sequences used for Y2H interactions (blue font) did not match a nucleotide identifier from Additional File 3. Sequence was aligned with Ensembl sequences encoding TaMPK20-3 using Clustal Omega (http://www.ebi.ac.uk/Tools/msa/clustalo/). Percent identity of aligned sequences are also provided.

```
TaMPK20-4(clone)     -MPEANAGARGGGEQRSKVQSSDVMSFFSEYGDASRYKIEEIIGKGSYGVVCSAIDRQTG DRVAIKKVSNIFEHITDAARMLREIKFLRLLRHPDIVQIKHIMLPPSRRDYKDIFVVFEL
TaMPK20-4(3AL)       ----M-----QPDQNQQRRKGSSEMDFFSEYGDANRYKIQEVIGKGSYGVVCSAIDQHTG DKVAIKKIHNIFEHLSDAARILREIKLLCLLRHPDIVEIRHIMLPPSRRDFKDIYVVFEL
TaMPK20-4(3DL)       MQPD-------QHQQQQRRKGSSEMDFFSEYGDANRYKIQEVIGKGSYGVVCSAIDQHTG DKVAIKKIHNIFEHLSDAARILREIKLLRLLRHPDIVEIRHIMLPPSRRDFKDIYVVFEL
AK333309.1           MQPDQ-----QQHQHQQRRKGSSEMDFFSEYGDANRYKIQEVIGKGSYGVVCSAIDQHTG DKVAIKKIHNIFEHLSDAARILREIKLLRLLRHPDIVEIRHIMLPPSRRDFKDIYVVFEL
TaMPK20-4(3B)        MQPDQ-----QQHQHQQRRKGSSEMDFFSEYGDANRYKIQEVIGKGSYGVVCSAIDQHTG DKVAIKKIHNIFEHLSDAARILREIKLLRLLRHPDIVEIRHIMLPPSRRDFKDIYVVFEL
                                  ::.: :.*. *.********.****:*:**************::** *:*****: *****::****:*****:* ********:*:**********:***:*****

TaMPK20-4(clone)     MDTDLHQVIKANDDLTKEHYQFFLYQMLRALKYIHTANVYHRDLKPKNILANANCKLKIC DFGLARVAFNDTPTTVFWTDYVATRWYRAPELCGSFFTKYSPAIDTWSIGCIFAEILTGK
TaMPK20-4(3AL)       MDTDLHQVIKANDDLTKEHHQFFLYQMLRALKYIHTANVYHRDLKPKNILANANCKLKIC DFGLARVAFNDTPTTVFWTDYVATRWYRAPELCGSFFTKYSPAIDIWSIGCIFAEILTGK
TaMPK20-4(3DL)       MDTDLHQVIKANDDLTKEHHQFFLYQMLRALKYIHTANVYHRDLKPKNILANANCKLKIC DFGLARVAFNDTPTTVFWTDYVATRWYRAPELCGSFFTKYSPAIDIWSIGCIFAEILTGK
AK333309.1           MDTDLHQVIKANDDLTKEHHQFFLYQMLRALKYIHTANVYHRDLKPKNILANANCKLKIC DFGLARVAFNDTPTTVFWTDYVATRWYRAPELCGSFFTKYSPAIDIWSIGCIFAEILTGK
TaMPK20-4(3B)        MDTDLHQVIKANDDLTKEHHQFFLYQMLRALKYIHTANVYHRDLKPKNILANANCKLKIC DFGLARVAFNDTPTTVFWTDYVATRWYRAPELCGSFFTKYSPAIDIWSIGCIFAEILTGK
                     *******************:**************************************** ********************************************* **************

TaMPK20-4(clone)     PLFPGKNVVQQLDMMTDFLGSPSPEIISRIRNEKARRYLSSMRKKLPVPFSEKFPKADPA AVKLLQKLLAFDPKDRPTAEEALADPYFNGLAKVEREPSCQPISKMEFEFERRKFTREDV
TaMPK20-4(3AL)       PLFPGKNVVHQLDLMTDLLGTPSLDTVSRIRNEKARRYLSSMRKKQPVCFSERFPKADPA ALKLMQRLLAFDPKDRPTAEEALADPYFKGLGKVEREPSCQPISKFEFEFERKKVTKEDV
TaMPK20-4(3DL)       PLFPGKNVVHQLDLMTDLLGTPSLDTVSRIRNEKARRYLSSMRKKQPVCFSERFPKADPA ALKLMQRLLAFDPKDRPTAEEALADPYFKGLGKVEREPSCQPISKFEFEFERKKVTKEDV
AK333309.1           PLFPGKNVVHQLDLMTDLLGTPSLDTVS-------------------------------- ------------------------------------------------------------
TaMPK20-4(3B)        PLFPGKNVVHQLDLMTDLLGTPSLDTVSRIRNEKARRYLSSMRKKQPVCFSERFPKADPA AFKLMQRLLAFDPKDRPTAEEALADPYFKGLGKVEREPSCQPISKFEFEFERKKVTKEDV
                     *********:***:***:**:**  : :*

TaMPK20-4(clone)     KELIFREILEYHPQLLKDYSNGSEKTNFLYPSAVDNFRRQFANLEEDGGKGGA---AERK HVSLPRTTTVHSTPVPTTNCPASQAPQRIPAARPGRVIASATPTENAAFTDRQMGRRMAR
TaMPK20-4(3AL)       KELIFREILEYHPQLLKDYMNGTEKTNFLYPSAVDNFRRQFANLEENGGKGGAIVPSDRK HVSLPRTTTVHSTPIPPKDQKSSQVPQRIPTGRPGRVVGPVIPFENSCAMDPYSQRRVAR
TaMPK20-4(3DL)       KELIFREILEYHPQLLKDYMNGTEKTNFLYPSAVDNFRRQFANLEENGGKGGAIVPSDRK HVSLPRTTTVHSTPIPPKDQKSSQVPQRIPTGRPGRVVGPVIPFENSCAMDPYSQRRVAR
AK333309.1           ------------------------------------------------------------ ------------------------------------------------------------
TaMPK20-4(3B)        KELIFREILEYHPQLLKDYMNGTEKTNFLYPSAVDNFRRQFANLEENGGKGGAIVPSDRK HVSLPRTTTVHSTPIPPKDQKSSQVPQRIPTGRPGRVVGPVIPFENSCAMDPYSQRRVAR

TaMPK20-4(clone)     DPAGAPAAA----AGHHLRPDC--SDRQHEVEKDRAHYRPSHHFRDARVAPEAEARPSAY Y-----------------------IPPFNGIAAVGAAGRM-----------Y
TaMPK20-4(3AL)       NPVLPAAATNVSAYAYHRKSDSSERELQQELEKDRMQYQPMQRFMDAKMVSPD-LRSTSY YMPKGAPKADVAERTGLQPNMMQGIAPFNGIAAVGGSYNKASAVQYGVSRMY
TaMPK20-4(3DL)       NPVLPAAATNVSAYAYHRKSDSSERELQQELEKDRMQYQPMQRFMDAKMVSPD-LRSTSY YMPKGVPKADVAERTGLQPNMMQGIAPFNGIAAVGGSYNKASAVQYGVSRMY
AK333309.1           ------------------------------------------------------------ ----------------------------------------------------
TaMPK20-4(3B)        NPVLPAAATNVSAYAYHRKSDSSERELQQELEKDRMQYQPMQRFMDAKMVSPD-LRSTSY YMPKGVPKADVAERTGLQPNMMQGIAPFNGIAAVGGSYNKASAVQYGVSRMY
```

**1: TaMPK20-4_clone**	**100.00**	**76.81**	**77.22**	**76.75**	**83.59**
2: TaMPK20-4_3AL	76.81	100.00	99.14	98.80	98.07
3: TaMPK20-4_3DL	77.22	99.14	100.00	99.66	99.62
4: TaMPK20-4_3B	76.75	98.80	99.66	100.00	100.00
5: AK333309.1	83.59	98.07	99.62	100.00	100.00

TaMPK20-4(clone) protein sequences used for Y2H interactions (blue font) did not match a nucleotide identifier from Additional File 3. Sequence was aligned with Ensembl sequences encoding TaMPK20-4 using Clustal Omega (http://www.ebi.ac.uk/Tools/msa/clustalo/). Percent identity of aligned sequences are also provided.

```
TaMPK21-2(3AL)      MAKKGSDKAGFFTEYGEATRYEIGEVVGKGSYGVVAAAVDTHTGERVAIKKIDDVFEHVA DATRILREIKLLRLLRHPDIVEIKHIMLPPSRREFRDIYIIFELMESDLHQVIKANDDLS
TaMPK21-2(3B)       MAKKGSDKGGFFTEYGEATRYQVGEVVGKGSYGVVASAVDTHTGESVAIKKIDDVFEHVA DATRILREIKLLRLLRHPDIVQIKHIMLPPSRREFRDIYIIFELMESDLHQVIKANDDLS
AK330685.1          MAKKGSDKGGFFTEYGEATRYQVGEVVGKGSYGVVASAVDTHTGESVAIKKIDDVFEHVA DATRILREIKLLRLLRHPDIVQIKHIMLPPSRREFRDIYIIFELMESDLHQVIKANDDLS
TaMPK21-2(clone)    MAKKGSDKGAFFTEYGEATRYQVGEVVGKGSYGVVASAVDTHTGEPVAIKKIDDVFEHVA HATRILREIKLLRLLRHPDIVQIKHIMLPPSRREFRDIYIIFELMESDLHQVIKANDDLS
TaMPK21-2(3DL)      MAKKGTDKGAFFTEYGEATRYQVGEVVGKGSYGVVASAVDTHTGEPVAIKKIDDVFEHVA DATRILREIKLLRLLRHPDIVQIKHIMLPPSRREFRDIYIIFELMESDLHQVIKANDDLS
                    *****:**..***********::*************:******** ************** .*******************:***************************************

TaMPK21-2(3AL)      PEHFQFFFYQLLRGMKYMHAANVFHRDLKPKNILANADCKLKICDFGLSRVSFNDTPSAI FWTDYVATRWYRAPELCGSFFSKYTPAIDIWSIGCIFAEMLTGRPLFPGKNVVHQLDLMT
TaMPK21-2(3B)       PEHHQFFFYQLLRGMKYIHAANVFHRDLKPKNILANADCKLKICDFGLARVSFNDTPSAI FWTDYVATRWYRAPELCGSFFSKYTPAIDIWSIGCIFAEMLTGRPLFPGKNVVHQLDLMT
AK330685.1          PEHHQFFFYQLLRGMKYIHAANVFHRDLKPKNILANADCKLKICDFGLARVSFNDTPSAI FWTDYVATRWYRAPELCGSFFSKYTPAIDIWSIGCIFAEMLTGRPLFPGKNVVHQLDLMT
TaMPK21-2(clone)    PENHQFFFYQLLRGMKYIHAANVFHRDLKPKNILANADCKLKICDFGLARVSFNDTPSAI FWTDYVATRWYRAPELCGSFFSKYTPAIDIWSIGCIFAEMLTGRPLFPGKNVVHQLDLMT
TaMPK21-2(3DL)      PENHQFFFYQLLRGMKYIHAANVFHRDLKPKNILANADCKLKICDFGLARVSFNDTPSAI FWTDYVATRWYRAPELCGSFFSKYTPAIDIWSIGCIFAEMLTGRPLFPGKNVVHQLDLMT
                    **:.*************:******************************:*********** ************************************************************

TaMPK21-2(3AL)      DLLGTPSEESISRVRNEKARRYLGNMKKKHPIPFSQKFPGVDPMALHLLERLLAFDPADR PTAAEALADPYFTGLANSELEPTAQPISKLEFEFERRKLAREDLRELIYREILEYHPQML
TaMPK21-2(3B)       DLLGTPSAESISRIRNEKARRYLGNMKKKHPIPFSQKFPGVDPMALHLLERLLAFDPADR PTAAEALADPYFTGLANSELEPTTQPISRLEFEFERRKLGREDVRELIYREILEYHPQML
AK330685.1          DLLGTPSAESISRIRNEKARRYLGNMKKKHPIPFSQKFPGVDPMALHLLERLLAFDPADR PTAAEALADPYFTGLANSELEPTTQPISRLEFEFERRKLGREDVRELIYREILEYHPQML
TaMPK21-2(clone)    DLLGTPSAESISRIRNEKARRYLGNMKKKHPIPFSQKFPGVDPMALHLLERLLAFDPTDR PTAAEALADPYFTGLANSELEPTTQPISRLEFEFERRKLAREDVRELIYREILEYHPQML
TaMPK21-2(3DL)      DLLGTPSAESISRIRNEKARRYLGNMKKKHPIPFSQKFPGVDPMALHLLERLLAFDPTDR PTAAEALADPYFTGLANSELEPTTQPISRLEFEFERRKLAREDVRELIYREILEYHPQML
                    ******* *****:*******************************************:** ***********************:****:**********.***:****************

TaMPK21-2(3AL)      HDYLRGGDQANFLYPSGVDRFKSQFVHLEEIGAKGEKTSPQLRQHASLPRERVIGSSDEP EKPNADYCIKLHVDELPGHTSVTDGLNKPLLNTRNFLKSESIGASKCIVVKEKQEKDEES
TaMPK21-2(3B)       HEYLRGGDQANFLYPSGVDRFKRQFVHLEEIGAKGEKTSPQLRQHASLPRERVIGSSDEP EKPNADYCIKLHVGEVPGYTSVTDGLNKPLLNTRNFLKSESIGASKCIVVKEKREKDEES
AK330685.1          HEYLRGGDQANFLYPSGVDRFKRQFVHLEEIGAKGEKTSPQLRQHASLPRERVIGSSDEP EKPNADYCIKLHVGEVPGYTSVTDGLNKPLLNTRNFLKSESIGASKCIVVKEKREKDEES
TaMPK21-2(clone)    HEYLRGGDQANFLYPSGVDRFKRQFVHLEEIGAKGEKTSPQLRQHASLPRERVIGSSDEP EKPNADYCIKLHVGEVPGYTSVTDGLNKPLLNTRNFLKSESIGASKCIVVKEKREKDEES
TaMPK21-2(3DL)      HEYLRGGDQANFLYPSGVDRFKRQFVHLEEIGAKGEKTSPQLRQHASLPRERVIGSSDEP EKPNADYCIKLHVGEVPGHTSVTDGLNKPLLNTRNFLKSESIGASKCIVVKEKREKDEES
                    *:******************** ************************************* *************.*:**:**********************************:******

TaMPK21-2(3AL)      MSEYMHEASD
TaMPK21-2(3B)       MSEYMHEASD
AK330685.1          MSEYMHEASD
TaMPK21-2(clone)    MSEYMHEASD
TaMPK21-2(3DL)      MSDYMHEA--
                    **:*****
```

1: TaMPK21-2_3AL	100.00	95.71	95.71	95.10	95.08
2: TaMPK21-2_3B	95.71	100.00	100.00	98.78	98.36
3: AK330685.1	95.71	100.00	100.00	98.78	98.36
4: TaMPK21-2_clone	95.10	98.78	98.78	100.00	99.18
5: TaMPK21-2_3DL	95.08	98.36	98.36	99.18	100.00

TaMPK21-2(clone) protein sequences used for Y2H interactions (blue font) did not match a nucleotide identifier from Additional File 3. Sequence was aligned with Ensembl sequences encoding TaMPK21-2 using Clustal Omega (http://www.ebi.ac.uk/Tools/msa/clustalo/). Percent identity of aligned sequences are also provided.

```
TaMPK24(6BS)        MEFFTEYGEASQYQIEEIIGQGSFGVVAAAVDTQTGERVAIKKIHDMFEHAPDGTRILRE IKLLRLLRHPNIVGVKHILLPPARREFRDIYVVFELMESDLQKVIQVNENLTAGHHRYFL
TaMPK24(U)          MEFFTEYGEASQYQIEEIIGKGSFGVVAAAVDTQTGERVAIKKIHDMFEHASNGTRILRE IKLLRLLRHPNIVETKHILLPPARREFRDIYVVFELMESDLQKVIQANENLTAGHHRFFL
TaMPK24(6DS)        MEFFTEYGEASQYQIEEIIGRGSFGVVAAAVDTQTGERVAIKKIHDMFEHASDGTRILWE IKLLRLLRHPNIVEIKHILLPPARREFRDIYVVFELMESDLQKVMQVNENLTAGHHRYFL
TaMPK24(clone)      MEFFTEYGEASQYQIEEIIGKGSFGVVAAAVDTQTGERVAIKKIHDMFEHASNGTRILRE IKLLRLLRHPNIVETKHILLPPARREFRDIYVVFELMESDLQKVIQANENLTAGHHRFFL
                    ********************:****************************** :***** * *************  *****************************:*.**********:**

TaMPK24(6BS)        YQLLHALKYIHAANVFHRDLKPSNILANSNCKLKICDFGLARALRDDAPLAIFWTDYVAT RWYRAPELCGSFFSKYTPAIDIWSIGCIFAEVLTGKPLFPGMNVTHQLDLITNVLGTPSY
TaMPK24(U)          YQLLHALKYIHAANVFHRDLKPSNILVNSNCKLKICDFGLARASRDDPPLAIFWTDYVAT RWYRAPELCGSFFSKXXXXXXXXXXXXXXXXXXXXXXXXXXXXVKHQLDLITDVLGTPSY
TaMPK24(6DS)        YQLLHALKYIHAANVFHRDLKPSNILVNSNCKLKICDFGLARASRDDPPLAIFWTDYVAT RWYRAPELCGSFFSKYTPAIDIWSIGCIFAEVLTRKPLFPGTNVKHQLDLITDVLGTPSH
TaMPK24(clone)      YQLLHALKYIHAANVFHRDLKPSNILVNSNCKLKICDFGLARASRDDPPLAIFWTDYVAT RWYRAPELCGSFFSKYTPAIDIWSIGCIFAEVLTRKPLFPGTNVKHQLDLITDVLGTPSY
                    **************************.**************** *** ************ ***************                            *.*******:******:

TaMPK24(6BS)        ETLSQIRNEKARRYLTGMKRKHPIPFPSIFCNADPQAVRLLERLLAFDPKDRPTAEEALA DPYFEGFPNLEHEPSPHPFSKLEFEFERWKLTKDGVRDLIYREILEYHPQMLQDYIRAGG
TaMPK24(U)          ETLSQIRNEKARIYLTGMKRKHPIPFPSMFCNADPQAVRLLERLLAFDPKDRPTAEEALA DPYFEGFPNLEHEPSPHPFSKLEFEFERWKLTKDGIRDLIYREILEYHPQMLQDYIGAGG
TaMPK24(6DS)        ETLSQIRNEKARRYLTGMKRKHPIPFPSMFCNADPQAVRLLERLLAFDPKDRPTAEEALA DPYFEGFPNLEHEPSPHPFSKLEFEFERWKLTKDGIRDLIYREILEYHPQMLQDYIRAGG
TaMPK24(clone)      ETLSQIRNEKARRYLTGMKRKHPTPFPSMFCNADPQAVRLLERLLAFDPKDRPTAEEALA DPYFEGFPNLEHEPSPHPFSKLEFEFERWKLTKDGIRDLIYREILEYHPQMLQDYIGAGG
                    ************ ********** ****:******************************* ***********************************:******************** ***

TaMPK24(6BS)        QTSFVYPSGVDGMRLQFVHLEENHLRGERGTPLRRRHASLPRERVCAPMGIDNQDCNNER R-TASSAAQITIRSQQEGLTHAYVYQNGTGIPDFCSGYYLQNGSTSASSCVIEENEGPKE
TaMPK24(U)          QTSFVYPSGVDGMRLQFVHLEENHLRGERGTPLRRRHASLPRERVCAPKGSDNQDCNNER RRTASSAAQTTIRSQQEGLTHAYVYQNGTSIPNFCSGYYLQNGSTSASSCVIDEHEGLEE
TaMPK24(6DS)        QTSFVYPSGVDDMRLQFVHLEENHLRGERGTPLRRRHASLPRERVCAPKGSDNQDCNNER RRTASSAAQTTIRSQQEGLTHAYVYQNGTSIPNFCSGYYLQNGSTSASSCVIDDHEGLKE
TaMPK24(clone)      QTSFVYPSGVDGMRLQFVHLEENHLRGERGTPLRRRHASLPRERVCAPKGSDNQDCNNER RRTASSAAQTTIRSQQEGLTHAYVYQNGTSIPNFCSGYYLQNGSTSASSCVIDEHEGLEE
                    ***********.************************************ * ********* * ******* *******************.**:*******************:::** :*


TaMPK24(6BS)        NGVSA-EKVAYELSERLARI
TaMPK24(U)          NGVSEEEKVAYELSQCFARI
TaMPK24(6DS)        NGVSEEEKVAYELSQCFARI
TaMPK24(clone)      NGLPGS--------------
                    **:
```

1:	TaMPK24_6BS	100.00	88.55	93.98	93.80
2:	TaMPK24_U	88.55	100.00	91.80	93.00
3:	TaMPK24_6DS	93.98	91.80	100.00	96.50
**4:**	**TaMPK24_clone**	**93.80**	**93.00**	**96.50**	**100.00**

TaMPK24(clone) protein sequences used for Y2H interactions (blue font) did not match a nucleotide identifier from Additional File 3. Sequence was aligned with Ensembl sequences encoding TaMPK24 using Clustal Omega (http://www.ebi.ac.uk/Tools/msa/clustalo/). Percent identity of aligned sequences are also provided.
