# Supplementary material for: Analysis of MAPK and MAPKK gene families in wheat and related Triticeae species
Source: BMC Genomics. 2018 Mar 5;19:178. doi: 10.1186/s12864-018-4545-9 (PMC5838963; doi:10.1186/s12864-018-4545-9)
Supplement: Supplementary file 11 — Phylogenetic analysis of MPK11s. Phylogenetic analysis was carried out on full-length protein sequences of Arabidopsis, Brachypodium, rice and Triticeae clade B MPKs along with MPK4 and MPK11 from six other species. The monocot species (maize (Zm), sorghum (Sb), foxtail (Si), switch grass (Pv)) and eudicots (tomato (Sl) and potato (St)) were each shown to carry one or more clade B MPK with MEY activation loops [18]. SlMPK4–1 (accession JF791807) was previously reported as SlMAPK6 [16]. MPKs with MEY activation loops are shown in blue font. All of the monocot species with the MEY motifs also carried Tyr kinase signatures instead of the classic S/T kinase domain. AtMPK12 carried a Tyr kinase signature, but with a TEY activation loop typical of clade B MPKs. Three clade D members were included in the analysis, namely AtMPK16 and two OsMPK16s. It should be noted that the accession originally described for OsMPK16–2 (LOC_Os08g06060) [18], appears to have inadvertently been permuted with that for OsMPK4–2 (LOC_Os05g05160) (Tapan K. Mohanta, personal communications). (PDF 99 kb) [file 12864_2018_4545_MOESM11_ESM.pdf]

Additional File 11. Phylogenetic analysis of MPK11s. Phylogenetic analysis was carried out on full-length protein sequences of Arabidopsis, Brachypodium, rice and Triticeae clade B MPKs along with MPK4 and MPK11 from six other species. The monocot species (maize (Zm), sorghum (Sb), foxtail (Si), switchgrass (Pv)) and eudicots (tomato (Sl) and potato (St)) were each shown to carry one or more clade B MPK with MEY activation loops [1]. SlMPK4-1 (accession JF791807) was previously reported as SlMAPK6 [2]. MPKs with MEY activation loops are shown in blue font. All of the monocot species with the MEY motifs also carried Tyr kinase signatures instead of the classic S/T kinase domain. AtMPK12 carried a Tyr kinase signature, but with a TEY activation loop typical of clade B MPKs. Three clade D members were included in the analysis, namely AtMPK16 and two OsMPK16s. It should be noted that the accession originally described for OsMPK16-2 (LOC_Os08g06060) [1], appears to have inadvertently been permuted with that for OsMPK4-2 (LOC_Os05g05160) (Tapan K. Mohanta, personal communications).

Reference:

1. Mohanta TK, Arora PK, Mohanta N, Parida P, Bae H. Identification of new members of the MAPK gene family in plants shows diverse conserved domains and novel activation loop variants. BMC Genomics. 2015;16:1-20.
2. Kong F, Wang J, Cheng L, Liu S, Wu J, Peng Z, Lu G. Genome-wide analysis of the mitogen-activated protein kinase gene family in *Solanum lycopersicum*. Gene. 2012;499:108-20.

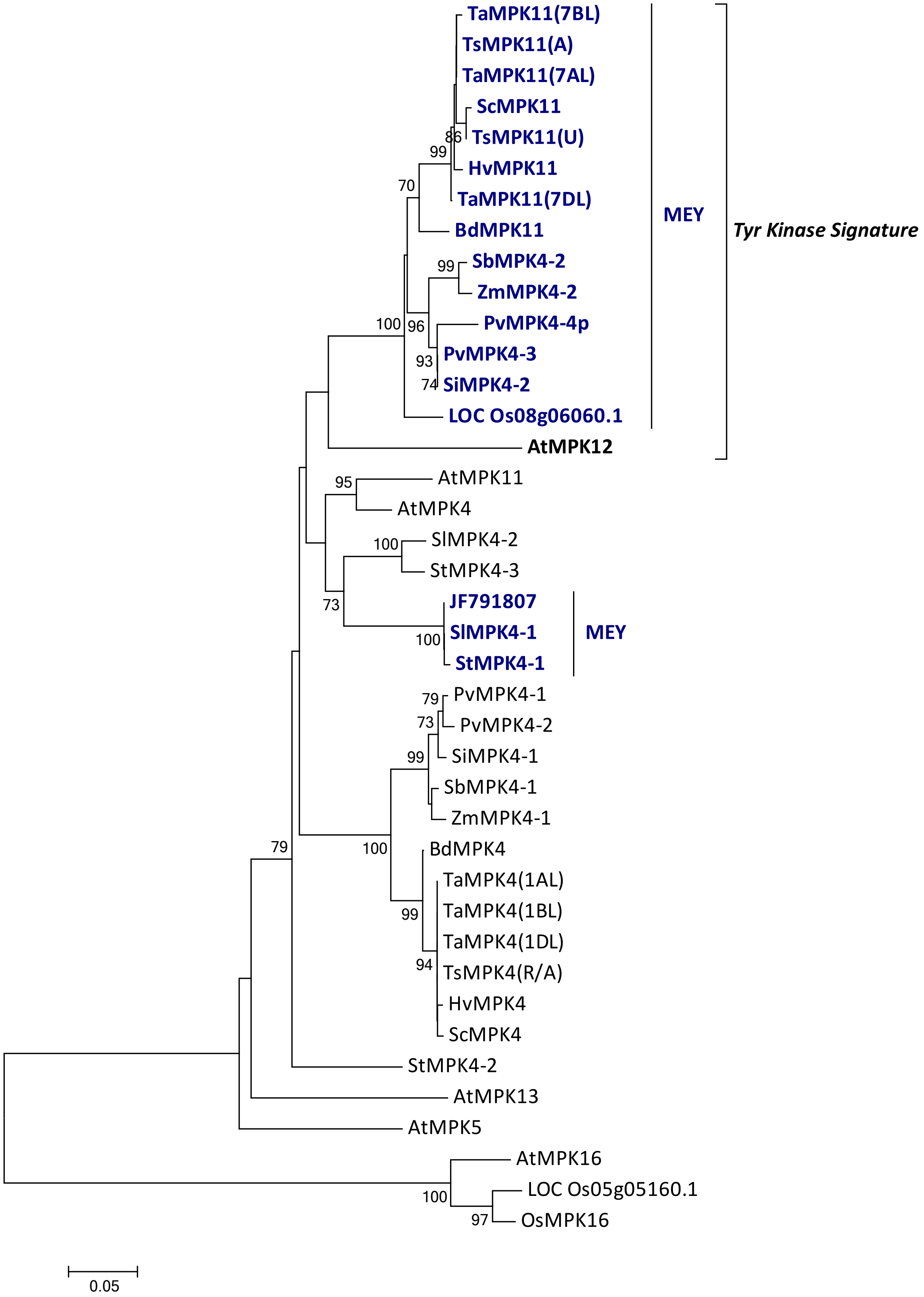
TaMPK11(7BL)
TsMPK11(A)
TaMPK11(7AL)
ScMPK11
TsMPK11(U)
HvMPK11
TaMPK11(7DL)
BdMPK11
SbMPK4-2
ZmMPK4-2
PvMPK4-4p
PvMPK4-3
SiMPK4-2
LOC Os08g06060.1
AtMPK12
MEY
Tyr Kinase Signature
AtMPK11
AtMPK4
SlMPK4-2
StMPK4-3
JF791807
SlMPK4-1
StMPK4-1
MEY
PvMPK4-1
PvMPK4-2
SiMPK4-1
SbMPK4-1
ZmMPK4-1
BdMPK4
TaMPK4(1AL)
TaMPK4(1BL)
TaMPK4(1DL)
TsMPK4(R/A)
HvMPK4
ScMPK4
StMPK4-2
AtMPK13
AtMPK5
AtMPK16
LOC Os05g05160.1
OsMPK16
0.05
