# Supplementary material for: Analysis of MAPK and MAPKK gene families in wheat and related Triticeae species
Source: BMC Genomics. 2018 Mar 5;19:178. doi: 10.1186/s12864-018-4545-9 (PMC5838963; doi:10.1186/s12864-018-4545-9)
Supplement: Supplementary file 12 — qRT-PCR primer sequences. Forward (F) and reverse (R) primers used to amplify wheat MPKs and MKKs. Housekeeping genes, wheat elongation factor 1-alpha (TEF1) accession M90077 and a hexose transporter (Contig5) accession CK155621 are shown in blue font. (PDF 85 kb) [file 12864_2018_4545_MOESM12_ESM.pdf]

Additional File 12. qRT-PCR primer sequences. Forward (F) and reverse (R) primers used to amplify wheat MPKs and MKKs. Housekeeping genes, wheat elongation factor 1-alpha (*TEF1*) accession M90077 and a hexose transporter (*Contig5*) accession CK155621 are shown in blue font.

**Primer Name**	**Primer Sequence 5'-3'**	**Primer Name**	**Primer Sequence 5'-3'**
TaMPK3-F	GGAGATCAAGCTCCTCAGGC	TaMKK1-F	GCGAGGCTCCTCCTATCAAG
TaMPK3-R	ACTGGCAGTGTTCTTCCGAG	TaMKK1-R	CCATCTGCTTGCGTATGCTT
TaMPK4-F	TCGAGCCTGGGATTTCTTCG	TaMKK3-2-F	GCAAGCAGCGTTGTTCAGAG
TaMPK4-R	GTCAACAGTGATGCGTCTGC	TaMKK3-2-R	TTGTCCAGAATCGGGCATGT
TaMPK6-F	CAGCTTATCTCCGAGGAAAACG	TaMKK3-3-F	AGCCGTTGCGAAAAGATTGG
TaMPK6-R	TTGTGCCGCACTAGTTGGA	TaMKK3-3-R	GCTTGCTGAGAATGTCGGGA
TaMPK7-F	GGAAACCACGGGAAGCACTA	TaMKK4-F	GACATCAAGCCGTCCAACCT
TaMPK7-R	CTTGATGGCGACCTTCTCGT	TaMKK4-R	CGTTGTAGTTGCCGTCGTTG
TaMPK11-F	AGTAAAGCTGCTTCGCCACA	TaMKK5-F	CGCATCAACACCGACATCAA
TaMPK11-R	TCCTCGGAGCACTTGGTAGA	TaMKK5-R	GGAGTAGCAGATGGCGACC
TaMPK14-F	GTCGTCACCCGCTGGTATAG	TaMKK6-F	CAGCTTATCTCCGAGGAAAACG
TaMPK14-R	CAGGTCAGACTCGCTCATGG	TaMKK6-R	TTGTGCCGCACTAGTTGGA
TaMPK16-F	TTCGGATAATCGGCCACAGG	TaMKK10-1/3a-F	CTCAAGGTCCAGCACTACGG
TaMPK16-R	ACAGCAGCACCCTTAGGAAC	TaMKK10-1/3a-R	CCACCAGCTCGAGGAGTAGA
TaMPK17-F	GCAGTGAGCCCTCAAAAGTC	TaMKK10-1/3b-F	CCAGGGCCCTATGATCCGTA
TaMPK17-R	CATCGACGGCTCCTTCTGTT	TaMKK10-1/3b-R	TTGCCGAAGAAGATAGCGCA
TaMPK20-1-F	ATAAGCCTTCGCCTGACGAG	TaMKK10-4-F	GAGAAAGGAGGCTACCGCAG
TaMPK20-1-R	TTCGTAAGGCAGAACTGGACC	TaMKK10-4-R	AAGTCGATCAGGCGGAACTC
TaMPK20-2-F	GCTATGGGGTTGTGTGCTCT	TaMKK10-5-F	GATTATTCACCGCGGCCATC
TaMPK20-2-R	CCTTTCTGGAGGGAGGCAAC	TaMKK10-5-R	TCCATCAGCTCGAGGAGCAA
TaMPK20-3-F	GCACCTGAACTTTGTGGGTC	TEF1-F	GGTGATGCTGGCATAGTGAA
TaMPK20-3-R	CCTTGTCATTCCGCACCTGA	TEF1-R	GATGACACCAACAGCCACAG
TaMPK20-4-F	AAGACCGCATGCAGTACCAA	Contig5-F	CTGCAGTGCGTCCATATTTT
TaMPK20-4-R	GTTTGGCTGCAAACCAGTCC	Contig5-R	AACAAGAACGATGCCGAGTT
TaMPK20-5-F	TCTATCAGATGCTCCGTGCG		
TaMPK20-5-R	GTGGCAACATAGTCCGTCCA		
TaMPK21-1-F	CGGCAAGAAGACCTCGGAAT		
TaMPK21-1-R	GGTGGCATCGGAGACATGAT		
TaMPK21-2-F	GCAACAAGGTGGTATCGTGC		
TaMPK21-2-R	AGATTCTGCCGAAGGAGTGC		
TaMPK24-F	CTCCGCCACCCAAACATAGT		
TaMPK24-R	CCTGCGGTGAGGTTCTCATT		
